# Impact of Induced Fitting and Secondary Noncovalent Interactions on Site-Selective and Enantioselective C–H Functionalization of Arylcyclohexanes

**DOI:** 10.1021/jacs.5c06398

**Published:** 2025-06-24

**Authors:** Duc Ly, Yannick T. Boni, Korkit Korvorapun, Volker Derdau, John Bacsa, Djamaladdin G. Musaev, Huw M. L. Davies

**Affiliations:** † Department of Chemistry, 1371 Emory University, 1515 Dickey Drive, Atlanta, Georgia 30322, United States; ‡ Sanofi-Aventis Deutschland GmbH, R&D, Integrated Drug Discovery, Industriespark Höchst, 65926 Frankfurt am Main, Germany; § Cherry L. Emerson Center for Scientific Computation, 1371 Emory University, 1521 Dickey Drive, Atlanta, Georgia 30322, United States

## Abstract

A major challenge in organic synthesis is the selective functionalization of C–H bonds. As most organic compounds contain multiple C–H bonds with similar properties, distinguishing between them requires precise control. In this study, we show how transition metal catalysts can adopt many of the characteristics associated with enzymes, leading to unprecedented site-selectivity in the C–H functionalization step. The catalysts are dirhodium complexes that adopt a bowl-shaped shape on formation. The flexible microenvironment within the bowl causes an induced fitting to occur as the reagent and substrate approach the catalyst. The key factors controlling the selectivity are noncovalent interactions between the approaching substrate and the catalyst wall, which cause a specific C–H bond in the substrate to be placed close to the metal-bound reagent.

## Graphical abstract

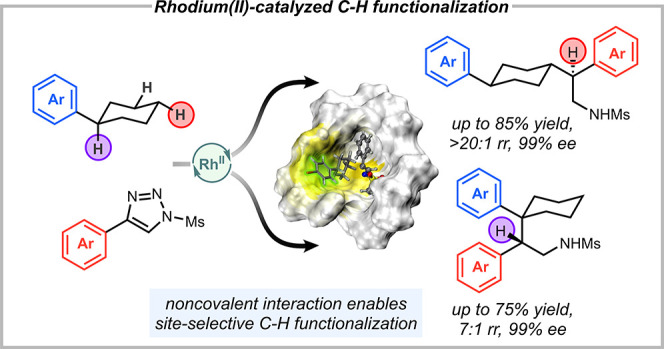

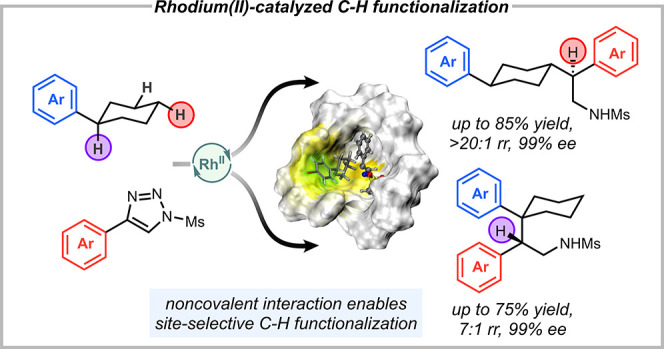

## Introduction

Carbon–hydrogen (C–H) functionalization represents an exciting new strategy for synthesis. Instead of focusing on reactions occurring at functional groups, the transformations are conducted at the C–H bonds, which previously were considered as generally unreactive.^1,2^ To maximize the synthetic potential of this strategy, it is necessary to distinguish between the multiple C–H bonds present in most organic molecules. The most widely used methods rely on functionality in the substrates to control site-selectivity, such as directing groups,^3–5^ or activating groups,^6–8^ or alternatively, the reactions are conducted intramolecularly.^7,9,10^ An ideal solution would be to use catalysts to control the site-selectivity so that different C–H bonds in the substrate can be functionalized by simply selecting the right catalyst.^11–14^ Achieving such a selectivity is difficult because C–H bonds often have very similar properties. Many examples are known of enzymes achieving exceptional site-selectivity because they can orientate a specific C–H bond to be close to the functionalizing reagent through secondary interactions between the substrate and the protein scaffold.^15–20^ They suffer, however, from a lack of generality, often resulting in exceptional results for a narrow range of substrates. Altering the enzyme to favor other substrates or to achieve a different selectivity profile involves considerable re-engineering of the enzyme.^15–20^ Transition metal and small molecule catalysts typically have a much wider substrate scope but the controlling elements for site-selectivity are more limited, relying on influencing the steric or electronic environment at the reagent causing the C–H functionalization.^11,12^ More subtle controlling elements such as noncovalent interactions are of considerable current interest.^21–24^ Here we describe the design of C_4_-symmetric bowl-shaped dirhodium catalysts, which display many characteristics of enzymes. Noncovalent interactions between the reagent and the wall of the catalyst cause an induced fit when the reagent binds to the catalyst and a further catalyst shape alteration occurs when the substrate approaches the catalyst-bound reagent. The overall effect of these secondary interactions is to stabilize both the rhodium-carbene intermediate and the transition state for C–H functionalization. Furthermore, these interactions cause the placement of a specific C–H bond near the rhodium-bound carbene, leading to unprecedented site-selectivity.

We have been exploring the use of chiral dirhodium catalysts to control the selectivity of C–H functionalization using donor–acceptor carbenes as the reactive intermediates.^25^ The donor group in donor–acceptor carbenes attenuates their high reactivity thus making them amenable to subtle catalyst control.^26,27^ Our initial studies were primarily conducted at activated C–H bonds such as allylic^28^ and benzylic^29^ or a bond to heteroatoms such as oxygen^30^ or nitrogen.^8^ More recently, however, we have demonstrated that site-selectivity between unactivated C–H bonds can also be achieved.^31–33^ Depending on the steric influence of the ligands around the rhodium carbene, the electronically favored 3° sites in the substrates can be sterically blocked and by appropriate catalyst selection C–H functionalization can be tuned to occur at the most accessible 3°, 2° or 1° C–H bonds.^31–33^

One of the intriguing features of the dirhodium tetracarboxylate catalysts is the way that the ligands self-assemble in a defined way to generate catalysts with symmetry higher than that of the ligands themselves. Recently, we prepared a *bowl*-shaped C_4_-symmetric dirhodium lantern complex, Rh_2_(*S*-TPPTTL)_4_ (**1**), that is capable of unprecedented site-selectivity in the C–H functionalization of alkylcyclohexanes leading to a clean reaction at the C3-equatorial position (Figure 1A).^14,34^ *A priori*, one would not have expected much selectivity between the C3 and C4 equatorial C–H bonds in monosubstituted cyclohexanes beyond the statistical 2:1 ratio because both sites are in similar electronic and steric environments. Even sterically crowded catalysts would not have been expected to differentiate among these equatorial sites. A detailed computational study revealed that the site-selectivity was caused by how the alkylcyclohexane **3** could fit into the bowl, as illustrated in Model A, and not because of steric crowding at the carbene *per se*.^34^ When the carbene attacks the C3 equatorial position, the alkyl group points out of the bowl, which minimizes the steric clash, whereas when it attacks C4, the alkyl group points directly toward the wall of the catalyst, resulting in severe steric interference.^34^

**Figure 1:**
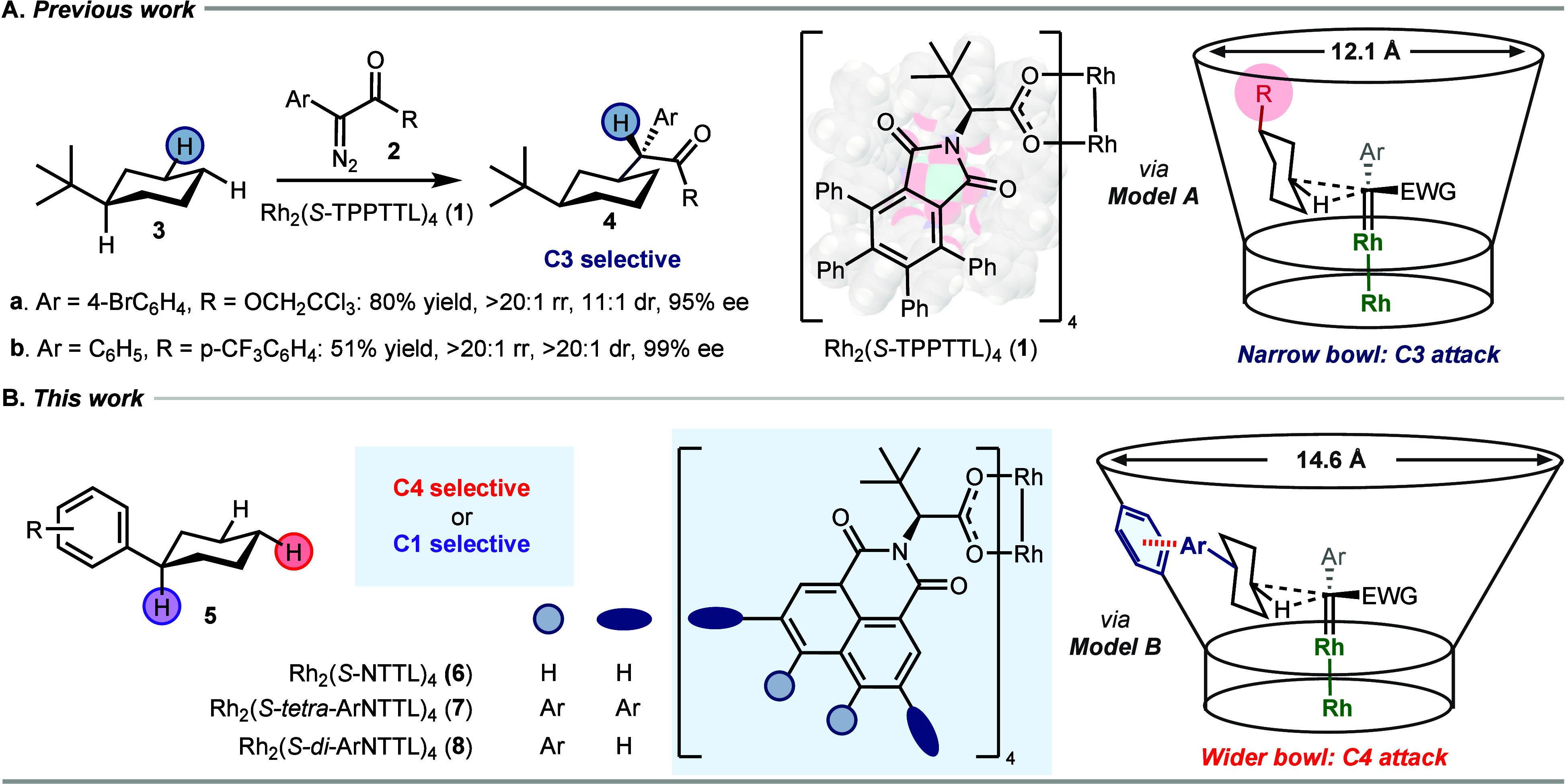
Background. (A) Previous work: site-selective C–H functionalization of cyclohexane derivatives at C3, as illustrated by the Rh_2_(*S*-TPPTTL)_4_ (**1**)-catalyzed reaction of the aryl diazo compounds **2a, b** with *tert*-butylcyclohexane (**3**) to generate the C–H functionalization products **4a**–**4b**. The regioselectivity is proposed to be due to steric factors in which the cyclohexane’s substituent points out of the bowl when attack occurs at C3, as illustrated in Model A. (B) Current work: site-selective C–H functionalization of arylcyclohexane **5** at either C4 or C1 using new catalysts derived from Rh_2_(*S*-NTTL)_4_ (**6**). The two new classes of catalysts **7** and **8** have been designed to have a wider bowl than Rh_2_(*S*-TPPTTL)_4_ to accommodate the possibility of favorable noncovalent interactions between the substrate and the catalyst wall, as illustrated in Model B, leading to a different site-selectivity profile.

Inspired by the concept of catalyst-controlled C–H functionalization and the potential role of noncovalent interactions, we wished to determine if catalysts could be designed to mitigate steric influence and maximize attractive interactions between the substrate and the wall of the catalyst (Figure 1B). We hypothesized that it would be necessary to have C_4_-symmetric dirhodium tetracarboxylate catalysts with a wider bowl than Rh_2_(TPPTTL)_4_ (**1**) such that when the reaction occurs at C4, the cyclohexane substituent fits into the bowl leading to a favorable interaction between the substituent and the bowl, as illustrated in Model B. We anticipated that derivatives of the naphthylimido catalyst Rh_2_(NTTL)_4_ (**6**) would have a wider bowl and would be a suitable framework for new catalyst design. We expected that π interactions between the catalyst, substrate, and reagent would likely be the most effective interactions, and hence, arylcyclohexanes **5** were used as substrates. Herein, we disclose two new sets of dirhodium catalysts, Rh_2_(*S*-*tetra-*ArNTTL)_4_ (**7**) and Rh_2_(*S*-*di-*ArNTTL)_4_ (**8**), capable of driving site-selective carbene C–H functionalization to the C4-position and benzylic C1-position in the cyclohexyl ring.

## Results and Discussion

### Catalyst Development

To challenge the hypothesis behind this study, we generated a library of catalysts with a wider bowl framework. The synthesis of the Rh_2_(*S*-*tetra-*ArNTTL)_4_ (**7**) was achieved via a four-step synthesis starting from naphthalic anhydride (see Scheme S2 for the synthetic details). The key step is an exhaustive 16-fold Suzuki–Miyaura cross-coupling on Rh_2_(*S*-*tetra*-BrNTTL)_4_ (**9**) to yield a series of Rh_2_(*S*-*tetra-*ArNTTL)_4_ (**7**) (Figure 2A). X-ray structures for **7a** and **7c** revealed that the ligands on complex formation had self-assembled into bowl-shaped structures similar to that of the phthalimido catalyst Rh_2_(*S*-TPPTTL)_4_ (**1**). In these types of catalysts, the carbene binds to the rhodium within the bowl, whereas the other rhodium is protected by the four flanking *tert*-butyl groups. Even though the desired bowl-shaped structure had been formed, we initially were concerned that the bowl is too regular and the *tetra*-arylnaphthylimido catalysts would not be capable of high asymmetric induction. Therefore, we designed a second series of naphthylimido catalysts consisting of 4,5-*di*-arylated derivatives (**8**), which were generated from Rh_2_(*S*-*di*-BrNTTL)_4_ catalyst (**10**) by an 8-fold Suzuki–Miyaura cross-coupling with aryl boronic acids to generate a range of Rh_2_(*S*-*di*-ArNTTL)_4_ (**8**) (see Scheme S1). The X-ray structure of **8a** and **8d** revealed that the ligands of this complex have self-assembled to bowl-shaped structures but now with clear gaps in the bowl at four quadrants. An interesting feature of these polyarylated bowl-shaped structures is that the complexes are formed with an induced helical chirality,^34^ and this can be readily seen in the X-ray structure of **8a** and **8d**. X-ray structures were also obtained for **7b**, **8b** and **8c** (see Figure S1).

**Figure 2:**
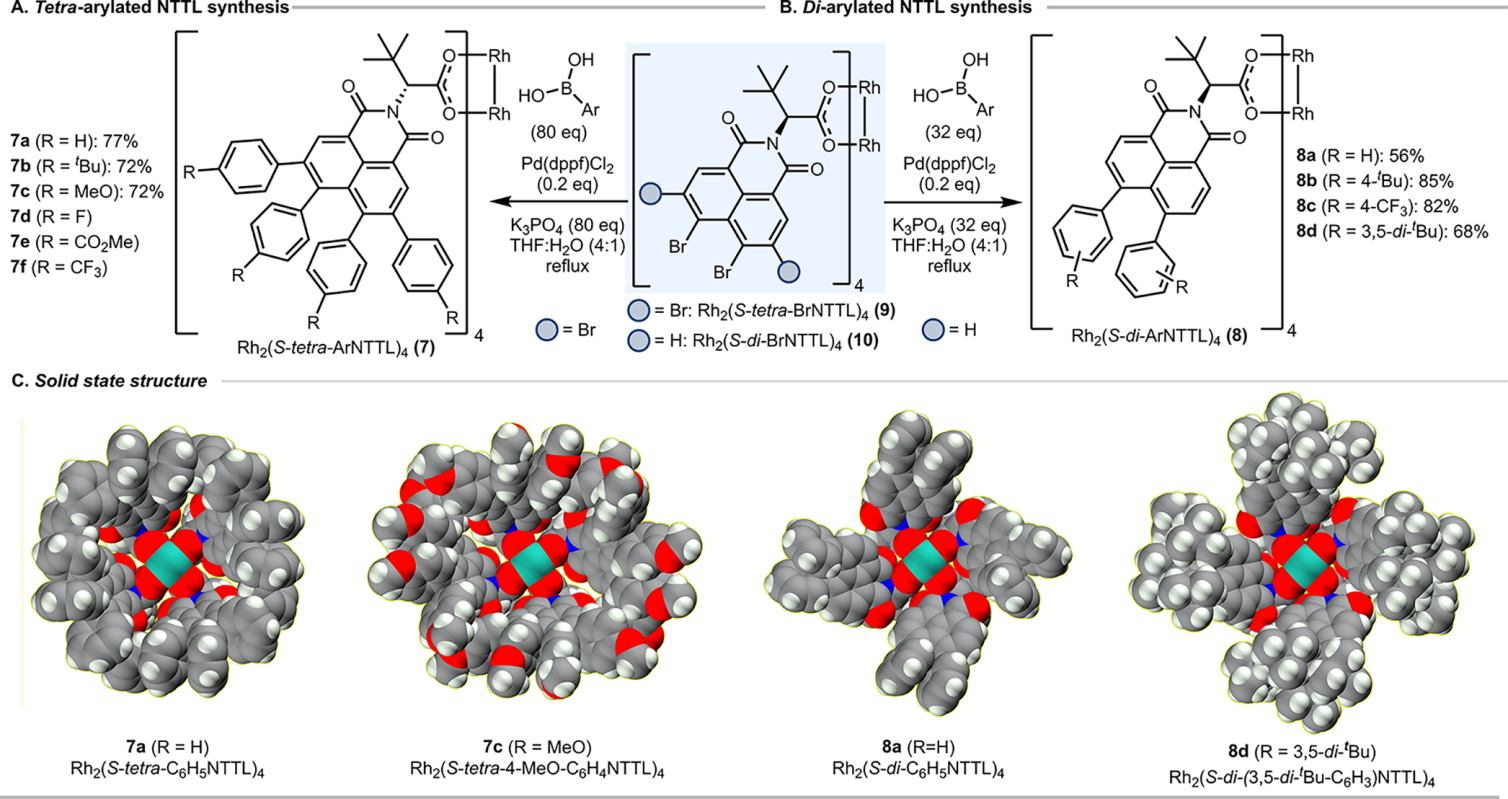
Catalyst synthesis. (A) Synthesis of a library of tetra-arylated Rh_2_(*S*-NTTL)_4_ derivatives **7** by a multifold Suzuki cross-coupling on preformed polybrominated complex **9**. In the case of **7d**–**f**, the Suzuki cross-coupling was conducted on the ligand prior to ligand exchange to form the catalyst. (B) Synthesis of a library of diarylated Rh_2_(*S*-NTTL)_4_ derivatives **8** by a multifold Suzuki cross-coupling on preformed polybrominated complex **10**. (C) X-ray structures of **7a**, **7c**, **8a** and **8d** illustrate that they all adopt bowl-shaped structures, the majority of which are very close to a perfect C4 symmetric structure (see SI for additional crystallographic data of other catalysts in the series).

### Reaction Optimization

As we envisioned π–π interactions between the approaching substrate and the multiple aromatic rings present in the ligands, we used the reaction of the well-established donor–acceptor carbene derived from *p*-bromophenyldiazoacetate (**2a)** with *p*-bromophenylcyclohexane (**5a**) as the substrate for the benchmark study (see Table S4). Even though this system did give reasonable site-selectivity favoring C4 (up to 8:1 rr), the enantioselectivity was very low (<20% ee) with the Rh_2_(*S*-*tetra*-ArNTTL)_4_ catalysts (**7**) and only moderate (68–83% ee) with the Rh_2_(*S*-*di-*ArNTTL)_4_ (**8**). Therefore, we switched to using *N*-(methyl sulfonyl)­triazole (**11**) as the carbene precursor because the parent catalyst Rh_2_(*S*-NTTL)_4_ (**6**) has been shown previously to be capable of high levels of asymmetric induction with this carbene source^35,36^ (Figure 3). The reactions with Rh_2_(*S*-TPPTTL)_4_ (**1**) and Rh_2_(*S*-NTTL)_4_ (**6**) were used as reference points for comparison with those of the new catalysts. The Rh_2_(*S*-TPPTTL)_4_ (**1**)-catalyzed reaction was unselective giving close to an equal mixture of C3, C4 and benzylic C1 functionalized products **12**–**14** (entry 1). In the Rh_2_(*S*-NTTL)_4_ (**6**)-catalyzed reaction, the site-selectivity favored C4 over C3 by a 5:1 ratio, and most promisingly, the asymmetric induction for the C4 product **13** was very high (96% ee) (entry 2). In these reactions, however, a significant amount of the benzylic C–H functionalization product was also formed. Extension of the study to the *tetra*-arylated catalysts **7a**–**b** resulted in even greater selectivity for C4 over C3 and high enantioselectivity across the board (entries 3 and 4). As we envisioned that π-stacking between the substrate and the catalyst wall was likely to be a key controlling factor for the C4 selectivity and the bromophenyl ring is relatively electron-deficient, we wondered whether Rh_2_(*S*-*tetra*-ArNTTL)_4_ catalysts (**7**) with electron-rich aryl groups would give better performance.^23^ We were delighted to find this is indeed the case, as Rh_2_(*S*-*tetra*-4-MeO-C_6_H_4_NTTL)_4_ (**7c**) resulted in a great improvement in site-selectivity, favoring C4 over C3 and benzylic C1 by a 3:92:5 ratio (entry 5). We also evaluated diarylated catalysts **8**. The catalysts **8a**–**c** all gave a preference for C4 over C3, but the benzylic C–H functionalization to form **14** was more pronounced (entries 6–8). Therefore, we decided to see if we could turn the 4,5-diarylated scaffold (**8**) into a catalyst selective for the C1 position. We reasoned that the diarylated catalysts **8a**–**8c** would still be capable of π-stacking with the *p*-bromophenylcyclohexane (**5a**) and that this would need to be blocked to diminish the C4 functionalization. Therefore, Rh_2_(*S*-*di*-(3,5-*di*-*^t^* Bu-C_6_H_3_)­NTTL)_4_ (**8d**) bearing sterically demanding *tert*-butyl groups was evaluated, and we were delighted to see that, in this case, benzylic C–H functionalization to form **14** was preferred by a ratio of 15:8:77 (entry 9).

**Figure 3:**
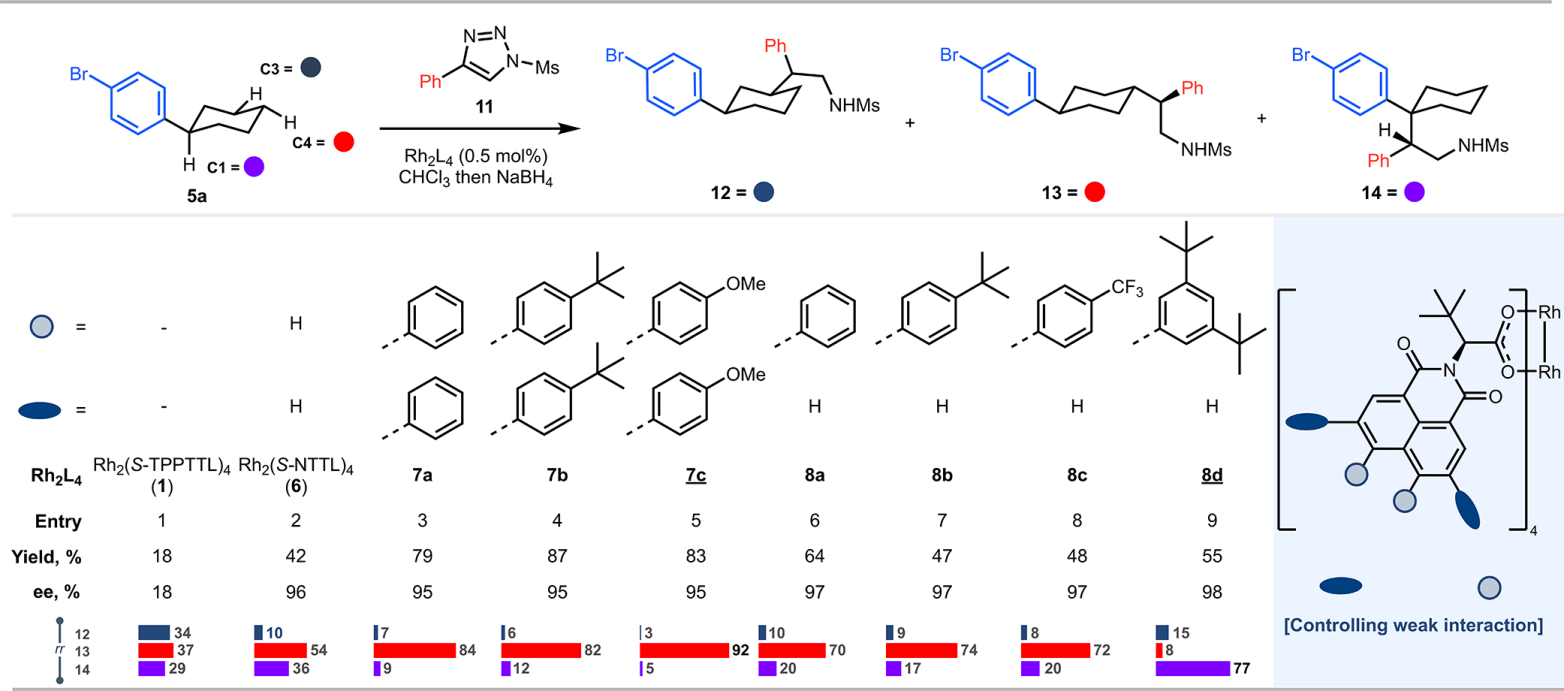
Optimization studies for the site-selective functionalization of 4-bromophenylcyclohexane. 1-(Methylsulfonyl)-4-phenyl-1*H*-1,2,3-triazole (**11**) was used as the carbene precursors. Reaction conditions: **11** (1.0 equiv), **5a** (2.5 equiv), Rh_2_L_4_ (0.5 mol %) in CHCl_3_ for 24 h then NaBH_4_ (2.5 equiv) in THF/MeOH (1:1). The products are color coded, blue for the C3 product **12**, red for the C4 product **13** and purple for the C1 benzylic product **14**. The ratio of the three products for each catalyst is visually represented. The reported enantioselectivity (ee) is for the major product of each reaction.

### Reaction Scope

Having identified the most suitable catalysts, the scope of the site-selective reactions was examined. Rh_2_(*S*-*tetra*-4-MeO-C_6_H_4_NTTL)_4_ (**7c**) is the optimal catalyst for electron-deficient aryl cyclohexane derivatives, as illustrated in the selectivity of this catalyst with various aryl cyclohexanes to form **15–28** (Figure 4A). All of the reactions proceeded with high levels of asymmetric induction (93–99% ee). When the arylcyclohexane substrate had even more electron-deficient aryl rings, as seen in the formation of **16–18**, the site-selectivity is >20:1. Electron-deficient heterocycles are also beneficial, as illustrated in the formation of **19–21**. Improved site-selectivity can also be obtained by making the aryl group of the carbene more electron-deficient, as can be seen with **22–24**. A particularly interesting feature of these reactions is that they can accommodate large *para* substituents on the aryl ring including groups with potentially competing C–H functionalization sites. A selective reaction is possible in a substrate containing two distinct cyclohexane rings, as illustrated in the formation of **25** in 81% yield with 93% ee. Similarly, high selectivity is observed with menthol and allofuranose derivatives to form **26** and **27**, respectively. The selective reaction to form the cholesterol derivative **28** in 85% yield with >20:1 rr and 96% ee is especially notable because a previous study with a simpler catalyst has shown that cholesteryl acetate is prone to C–H functionalization at the most accessible tertiary site,^32^ and no such reaction is observed here (see Figure S33 for more examples).

**Figure 4:**
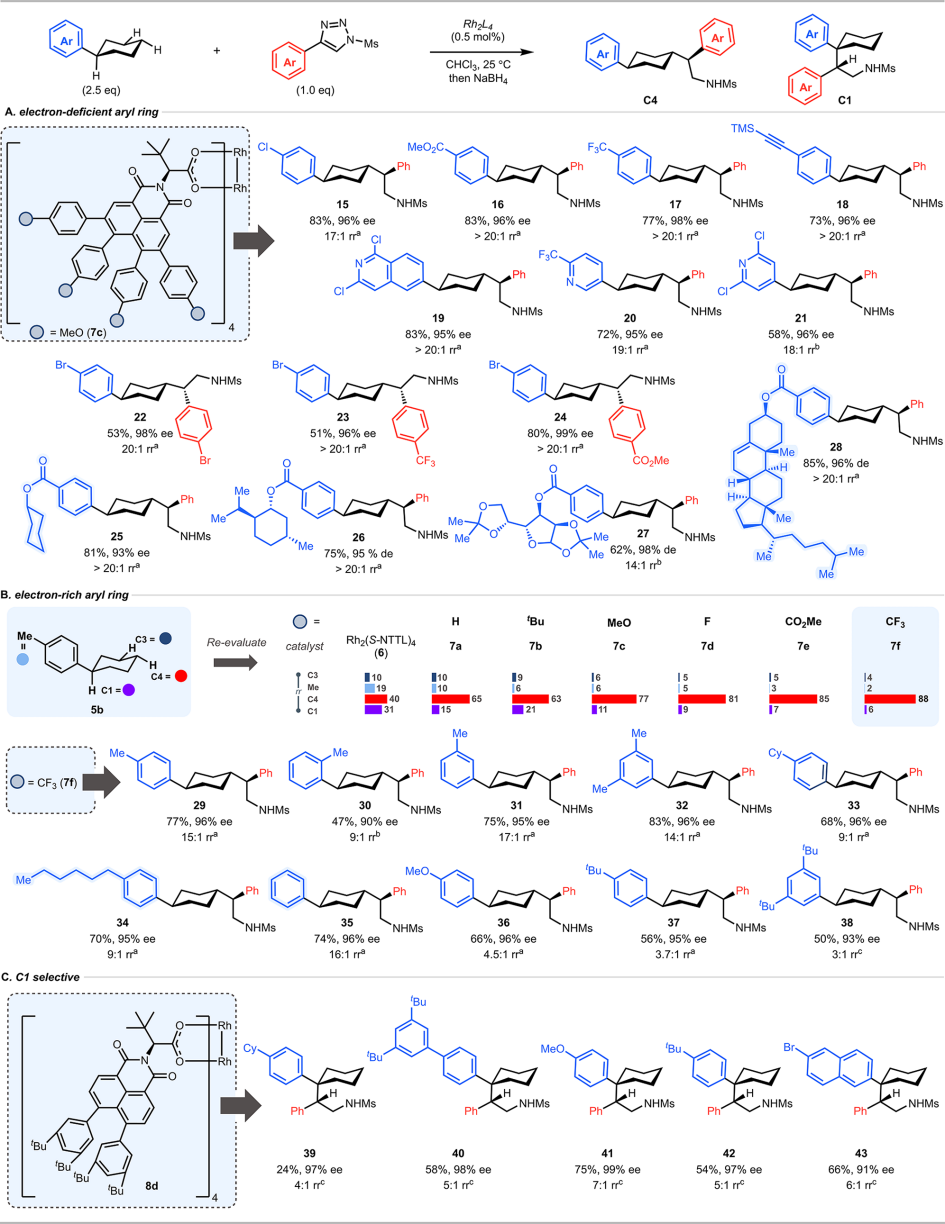
Substrate scope. (A) C4 selective C–H functionalization of electron-deficient arylcyclohexanes. (B) C4 selective C–H functionalization of electron-rich arylcyclohexanes. (C) C1 selective C–H functionalization of arylcyclohexanes. ^a^The regioselectivity (rr) is of the C4 product over the C1 product, whereas the ratio of C4 over C3 products is >20:1 rr. ^b^The regioselectivity (rr) is of the C4 product over C3 products with no observation of the C1 product. ^c^The regioselectivity (rr) is of the C4 (or C1) product over the C3 and C1 (or C4) products.

Rh_2_(*S*-*tetra*-4-MeO-C_6_H_4_NTTL)_4_ (**7c**) is highly C4 selective in all cases where either the arylcarbene or the arylcyclohexane had a strong electron-withdrawing group. As 4-cyclohexyltoluene (**5b**), lacking a strong electron-withdrawing aryl ring, is functionalized with relatively moderate site-selectivity for C4 over C1 (7:1), we examined whether Rh_2_(*S*-*tetra*-ArNTTL)_4_ (**7d**–**f**), with electron-deficient aryl rings, could enhance the C4 site-selectivity. Rh_2_(*S*-*tetra*-4-CF_3_–C_6_H_4_NTTL)_4_ (**7f**) was identified as the optimum catalyst, resulting in the formation of **29** with a C4 selectivity of 15:1 with a small amount of competing 1° and 3° benzylic functionalization (Figure 4B). The Rh_2_(*S*-*tetra*-4-CF_3_-C_6_H_4_NTTL)_4_ (**7f**) catalyzed C–H functionalization of electron-rich arylcyclohexanes was extended to a range of substrates (Figure 4B). A methyl substituent at the *para*, *meta* or *ortho* position and *p*-substituted primary alkyl or cyclohexyl are compatible with the chemistry resulting in the formation of **30–34** with high enantioselectivity (90–96% ee). Even an unsubstituted phenyl ring is compatible as seen in the formation of **35**, which is an impressive result because it is known that monosubstituted phenyl rings are prone to cyclopropanation with donor/acceptor carbenes.^37^ A highly electron-donating group such as *p*-MeO (**36**) and *p*-*^t^* Bu (**37**) does not give as high a site-selectivity for C4. Although the C4:C3 selectivity remains high, the competing benzylic C–H functionalization is now electronically more favored. We have also challenged the π interaction model by using 3,5-*di*-*^t^* Bu phenyl cyclohexane, which should be too sterically crowded for effective π-stacking, and, in this case, the resulting product **38** is formed with low site-selectivity for C4 versus C3 and C1 (3:1 ratio).

The utility of Rh_2_(*S*-*di*-(3,5-*di*-*^t^* Bu-C_6_H_3_)­NTTL)_4_ (**8d**), the catalyst designed for benzylic C–H functionalization, was also explored (Figure 4C). Although normally benzylic C–H functionalization at a tertiary site would be a favored site, in the case of arylcyclohexane, this would be a challenging reaction. In the dominant conformation of the arylcyclohexane, the benzylic hydrogen would be in an axial position and previous studies have shown that donor/acceptor carbene favor equatorial C–H functionalization over axial by a factor of 140.^34^ The benzylic C–H functionalization is effective with halo-, alkyl-, aryl-, and MeO-substituted aryl derivatives, as illustrated in the formation of **39–43**. The reactions are uniformly highly enantioselective (90–99% ee), and the regioselectivity is moderate (4–6:1 rr), except for the case of the *p*-MeO derivative **41**, where the electronic influence increases the benzylic site-selectivity to 7:1 rr.

### Computational Studies

At the onset of this study, the Rh_2_(*S*-*tetra*-ArNTTL)_4_ catalysts (**7**) were not expected to be highly enantioselective because they appeared to adopt a regular well-ordered bowl-shape, but they performed remarkably well in the reaction with the *N*-sulfonyltriazoles and displayed exceptional enantioselectivity, as did all of the Rh-NTTL catalysts **6–8**. Computational studies have been invaluable to gain a better understanding of rhodium-catalyzed carbene reactions^26,27,38–42^ but have not been applied extensively to the Rh-NTTL/triazole system.^43–45^ In order to understand the origin of the high enantioselectivity, density functional theory (DFT) studies were conducted on the C–H functionalization of cyclohexane (**5c**) by triazole **11** and Rh_2_(*S*-NTTL)_4_ (**6**) as a simplified model.^35^ The calculated energy profile for the C–H insertion at the CPCM­(CHCl_3_)-B3LYP-D3­(BJ)/Lan2ldz+6-31G­(d,p) level is depicted in Figure 5A. The coordination of the imino aryl carbene to **6** generates two diastereomeric structures **I** and **II** due to the high rotational barrier.^27,41,45^ Intermediate **II** is more stable than **I** by 1.4 kcal/mol, but it would result in the attack on the *Re* face of the carbene, leading to the wrong stereochemical outcome. However, the reaction proceeds under Curtin–Hammett conditions because the interconversion barrier of **II** to **I** (9.2 kcal/mol) is less than the barrier for C–H functionalization proceeding through **TS2** derived from **II** (12.7 kcal/mol). As a result, the 3.6 kcal/mol energy difference of **TS1** and **TS2**, instead of the ratio of **I** and **II**, will control the asymmetric outcome, reflecting the high enantioselectivity (96% ee).^35^ As the asymmetric induction of all the catalysts is routinely high in the C–H functionalization of cyclohexane and the substituted cyclohexanes, we propose that the cause of high enantioselectivity in the extended Rh_2_(*S*-NTTL)_4_ catalysts (**7**, **8**) involves a pathway similar to that of the parent Rh_2_(*S*-NTTL)_4_ (**6**).

**Figure 5:**
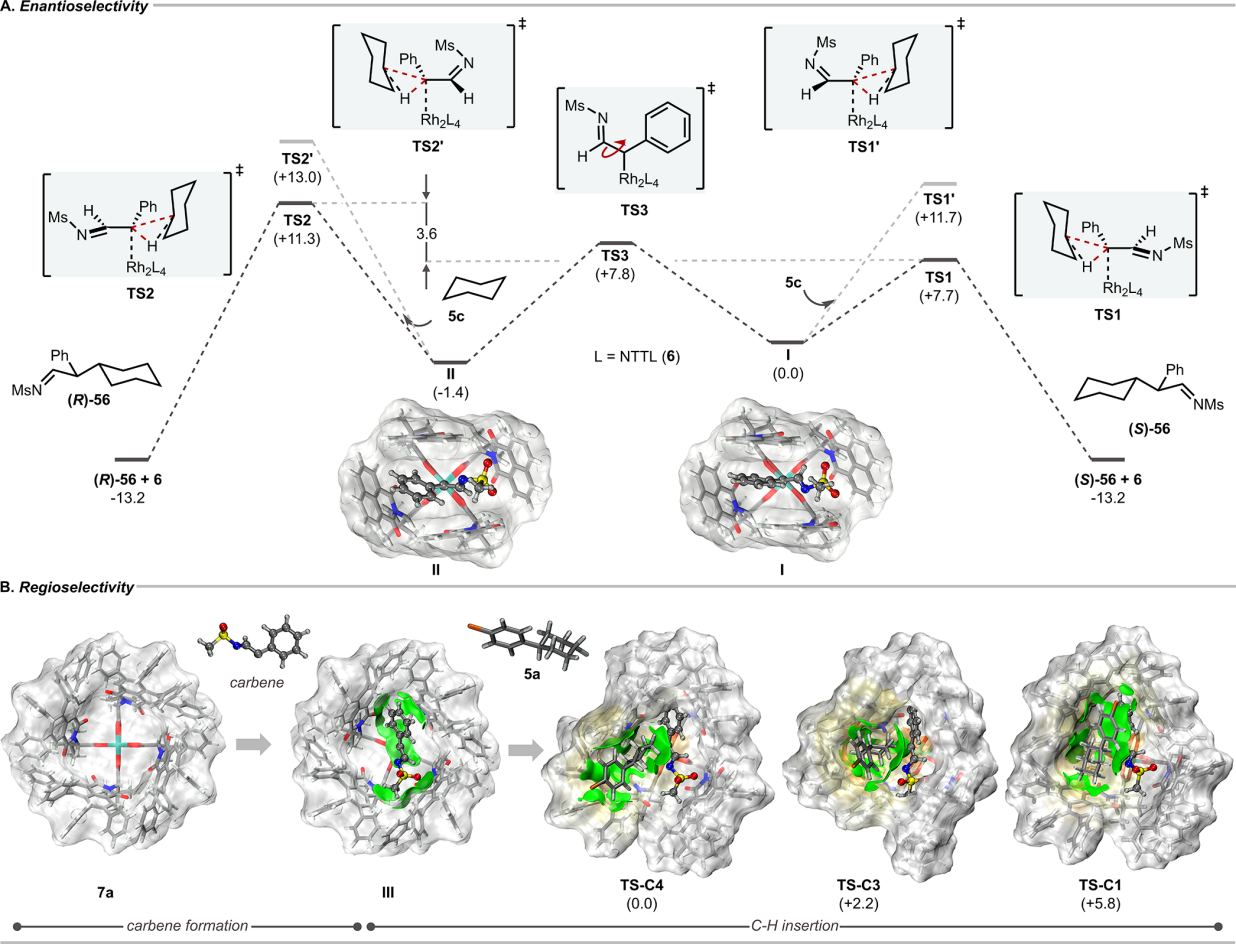
Computational studies. (A) The energy diagram for C–H insertion of metal-carbene intermediate derived from Rh_2_(*S*-NTTL)_4_ (**6**) and triazole **11** with cyclohexane as substrate (**5c**). (B) DFT optimized structure of Rh_2_(*S*-*tetra*-C_6_H_5_NTTL)_4_ (**7a**), metal-carbene intermediate (**III**) derived from **7a** and **11**, and the transition state structures for C4 (**TS-C4**), C3 (**TS-C3**) and C1 (**TS-C1**) functionalization with *p*-bromophenylcyclohexane (**5a**) as substrate. The catalyst structures are drawn by the VMD program^48^ as a surf model to visualize the catalyst shape while the carbene and substrate fragments are drawn as ball and stick model with the following atom coloring: H (light gray), carbon (black), oxygen (red), nitrogen (blue), sulfur (yellow), bromine (orange), and rhodium (light green). The green surfaces represent noncovalent interactions via IGMH method.^46,47^ Induced fitting occurs to accommodate the approaching substrate to the rhodium-bound carbene, and π-interactions exist between the bromophenyl ring of the arylcyclohexane and the wall of the catalyst, which is most extensive in **TS-C4**. The *p*-bromo substituent in TS-C4 points out the bowl, which is consistent with the experimental results showing that very large *p*-substituents can be accommodated during the C4 functionalization.

The next stage of the calculations focused on understanding the unprecedented site-selectivity exhibited in these reactions. The calculations were conducted on the C–H functionalization of 4-bromophenylcyclohexane (**5a**) by triazole **11** with Rh_2_(*S*-*tetra*-C_6_H_5_NTTL)_4_ (**7a**) (Figure 5B). The X-ray structure of **7a** was used as the starting point, and the DFT optimized structure of **7a** showed good alignment with the structure obtained from X-ray crystallography. It has a well-defined bowl-shape and is essentially C_4_-symmetric with the 16 phenyl rings at the periphery of the bowl tilting in one direction, generating a propeller chirality, similar to what had been previously seen with Rh_2_(S-TPPTTL)_4_.^34^ Thus, it appears that the catalyst in solution retains its general solid-state shape. Considerable changes in the shape of the catalyst occur on binding of the carbene to the catalysts (**III**) and during the subsequent approach of cyclohexane **5a** to the carbene complex. This induced-fitting occurs to accommodate extensive noncovalent interactions between the carbene and ligand in **III** and between the substrate and ligands in **TS-C4**, **TS-C3** and **TS-C1**, and these interactions greatly influence the C4, C3 and C1 benzylic site-selectivity. To visualize the noncovalent interactions, we applied the independent gradient model based on the Hirshfeld partition (IGMH) method^46^ at the B3LYP-D3­(BJ)/Lan2ldz+6–31G­(d,p) level of theory, using the Multiwfn program.^47^ For this purpose, intermediate **III** was divided into two fragments: the catalyst and the carbene. The solid green surfaces presented in Figure 5B represent interactions between the two fragments. Considerable π/π and CH/π interactions are seen between the aryl group of the carbene and the wall of the catalyst, leaving the *Si* face of the carbene open to the approach of the substrate. Each transition state structure, **TS-C4**, **TS-C3** and **TS-C1**, was similarly divided into two fragments: a carbene-catalyst and a substrate. The interaction regions were generally composed of a green isosurface, suggesting strong van der Waals interactions between cyclohexane **5a** and catalyst pocket, especially in **TS-C4** and **TS-C3**. The noncovalent interactions are less-pronounced in **TS-C1** because the benzylic C1 hydrogen is in the middle of the substrate, so it is not well positioned for nonbonding interactions with the catalyst wall. A closer analysis reveals that the aryl substituent of **5a** in **TS-C4** experiences much more π interactions than in **TS-C3** and **TS-C1**, which partially explains why it is more stable by 2.2 and 5.8 kcal/mol than the other two transition states. In all three transition states, the shape of the catalyst pocket has changed dramatically to accommodate the approach of the substrate to the carbene and to maximize noncovalent interactions between the catalyst wall and the aryl ring of the substrate. The observation of these weak interactions was the motivation for developing **7c** and **7f**, as it would be expected to maximize the interaction between the aryl groups on the substrate and the wall of catalysts, and indeed **7c** and **7f** are the optimal catalysts for C4 functionalization (Figure 4A-B). These observations also inspired the design of **8d**, which was expected to be less effective at π interactions and thus would be selective for C1 functionalization, as shown in Figure 4C. A further highly significant feature observed in **TS-C4** is the location of the *p*-bromo substituent of the arylcyclohexane outside of the bowl, which explains why a variety of *p*-substituents, even large groups such as a steroid, can be accommodated.

## Conclusion

Collectively, these findings underscore the exceptional controlling influence of bowl-shaped catalysts on the site-selectivity and stereoselectivity of C–H functionalization with donor/acceptor carbenes. Interactions of the approaching aryl cyclohexane substrates during the C–H functionalization with the catalyst wall lead to secondary π-bonding interactions which caused site-selective C4 functionalization. If these π-bonding interactions are blocked, then the catalysts can cause benzylic C1 functionalization to preferentially occur. A particularly interesting conclusion from the computational studies is the significance of induced fitting that occurs on carbene binding to the rhodium catalysts and as the substrate approaches the carbene during the C–H functionalization step. These insights into catalyst conformational mobility and the influence on the microenvironment inside the catalyst pocket open up new avenues for generating catalysts with even more subtle control elements for site-selective C–H functionalization.

## Supplementary Material



## Notes

Materials and methods, substrate and reagent details, computational studies, crystal structures, and spectroscopic data. (PDF)

## Abbreviations

TPPTTL: tetraphenylphthalimido-*tert*-leucine
NTTL: naphthalimido-*tert*-leucine
