# Supplementary material for: Impact of Induced Fitting and Secondary Noncovalent Interactions on Site-Selective and Enantioselective C–H Functionalization of Arylcyclohexanes
Source: J Am Chem Soc. 2025 Jun 24;147(27):23891–9. doi: 10.1021/jacs.5c06398 (PMC12257524; doi:10.1021/jacs.5c06398)
Supplement: Supplementary file 1 [file ja5c06398_si_001.pdf]

Impact of Induced Fitting and Secondary Noncovalent Interactions on Site-Selective and Enantioselective C–H Functionalization of Arylcyclohexanes

Duc Ly[1], Yannick T. Boni[1], Korkit Korvorapun[2], Volker Derdau[2], John Bacsa[1], Djamaladdin G. Musaev[1,3*], and Huw M. L. Davies[1*]
[1]*Department of Chemistry, Emory University, 1515 Dickey Drive, Atlanta, Georgia 30322, United States.*
[2]*Sanofi-Aventis Deutschland GmbH, R&D, Integrated Drug Discovery, Industriespark Höchst, 65926 Frankfurt am Main, Germany.*
[3]*Cherry L. Emerson Center for Scientific Computation, Emory University, 1521 Dickey Drive, Atlanta, Georgia, 30322, United States.*

Corresponding author: hmdavie@emory.edu

Supporting information

Complete experimental procedures, materials, computational details, and compound characterizations

Contents

1. General Information S3
2. Catalyst structure S4
3. Synthesis of triazoles S7
4. Acquisition and synthesis of Substrates S7
5. Synthesis of catalysts S18
   5.1. Synthesis of di-arylated NTTL derivatives S18
   5.2. Synthesis of tetra-arylated NTTL derivatives S22
6. Catalyst optimization study S35
   6.1. Study with diazo as Carbene Precursor S35
   6.2. Study with triazole as carbene precursor S40
   6.3. Scope of the site-selective C-H functionalization S58
7. Reaction monitoring and thermal dynamic study for C4 selective C-H functionalization S62
8. Crude NMR for regio-selectivity determination of C-H functionalization S67
   8.1. Crude NMR regio-selectivity determination of C4 selective C-H functionalization S67
   8.2. Crude NMR for regio-selectivity determination of C1 selective C-H functionalization S93
   8.3. Crude NMR for extra examples S99
9. Characterization of C-H functionalization products S111
10. Copies of NMR S165
   10.1. Spectra of prepared starting materials S165
   10.2. Spectra of catalyst synthesis S183
   10.3. Spectra of C-H functionalization products S212
11. Crystallography study S260
   Compound 7a S261
   Compound 7b S268
   Compound 7c S276
   Compound 8a S286
   Compound 8b S301
   Compound 8c S313
   Compound 8d S324
   Compound 10 ..... S337
   Compound 13 ..... S362
   Compound 13a S369
   Compound 42 ..... S375
12. DFT calculations S381
13. References S429

1. General Information

All reagents and solvents were used as purchased from commercial sources (Sigma) for substrate synthesis unless otherwise noted. Dichloromethane used in C–H insertion reactions was prepared from solvent purification system. Chloroform used in C-H insertion reaction was distilled over CaH_2 from alkene-stabilized dried chloroform obtained from Sigma followed by degassing by sparging with N_2 to remove alkene stabilizer and stored over 4 Å molecular sieves in dark cabinet. 4 Å molecular sieves were activated at 220 °C for 4 hours under vacuum and stored in an oven over 100 °C. All column chromatography was performed on silica gel (SiliaFlash® P60, 40-63 µm). Thin layer chromatographic (TLC) analysis was performed with aluminum-sheet silica gel plates. ^{1}H, ^{13}C and ^{19}F NMR spectra were recorded at 800MHz or 600 MHz on Bruker-800 spectrometer, Bruker-600 spectrometer or Varian IVONA-600 spectrometer (^{13}C at 151 MHz), 500 MHz on Varian INOVA-500 spectrometer, or 400 MHz (^{13}C at 101 MHz, ^{19}F at 376 MHz) on Bruker-400 spectrometer and all were reported in parts per million (ppm). Unless otherwise noted, ^{1}H, ^{13}C and ^{19}F NMR spectra were performed in solutions of deuterated chloroform ($CDCl_3$) with the residue chloroform set as an internal standard (7.26 ppm for 1 H, and 77.16 ppm for ^{13}C). Abbreviations for signal multiplicity are as follows: br = broad, s = singlet, d = doublet, t = triplet, q = quartet, m = multiplet, dd = doublet of doublet, tt = triplet of triplet, qt = quartet of triplet, dtd = doublet of triplet of doublet. Coupling constants (J values) were calculated directly from the spectra. Mass spectra were taken on a Thermo Finnigan LTQ-FTMS spectrometer with APCI, ESI or NSI. The enantiomeric excess (ee) was determined by High Performance Liquid Chromatography analysis was performed on either Varian Prostar model 410 HPLC, Agilent 1100 Technologies HPLC, Agilent Technologies 1290 Infinity UHPLC instruments, or Super Critical Fluid Chromatography using Water Acquity UPC^2 SFC system and the data outlined below varies in presentation based on the software used for each system. HPLC/SFC traces are reported based on the racemic retention times. The HPLC instruments used isopropanol/hexane gradient and commercial ChiralPak/ChiralCel columns from Daicel Chemical Industries, notably ChiralPak AD-H (5 µm particle size, 4.6 mm vs. 250 mm), ChiralCel OZ-H (5 µm particle size, 4.6 mm vs. 250 mm), and ChiralCel OD-H (5 µm particle size, 4.6 mm vs. 250 mm), ChiralCel AS-H (5 µm particle size, 4.6 mm vs. 250 mm), ChiralCel OJ-H (5 µm particle size, 4.6 mm vs. 250 mm), and Regis (S,S) Whelk-O1 5/100 Kromasil. The SFC system utilized supercritical fluid CO_2 with cosolvents of either HPLC-grade methanol, or acetonitrile, or ethanol, or isopropanol, or 1:1 MeOH:IPA with 0.2% formic acid, or 1:1 Ethanol:IPA with 0.2% formic acid, or 1:1 Ethanol:ACN with 0.2% formic acid, or 1:1:1 Ethanol:IPA:ACN with 20 mM ammonium formate with SFC columns: Trefoil AMY1 Column (2.5 µm, 3.0 mm X 150 mm), Trefoil CEL1 Column (2.5 µm, 3.0 mm X 150 mm), Trefoil CEL2 Column (2.5 µm, 3.0 mm X 150 mm), Regis (S,S) Whelk-O 1 Kromasil (3.5 µm, 3.0 mm X 150 mm), ChiralPak AD-3 (3.0 µm, 3.0 mm X 150 mm SFC), ChiralCel OZ-3 (3.0 µm, 3.0 mm X 150 mm), ChiralCel OD-3 (3.0 µm, 3.0 mm X 150 mm SFC), ChiralCel OX-3 (3.0 µm, 3.0 mm X 150 mm SFC); ChiralCel OJ-3 (3.0 µm, 3.0 mm X 150 mm SFC); ChiralPak AS-3 (3.0 µm, 3.0 mm X 150 mm SFC). In general, chiral HPLC or SFC conditions were determined by obtaining separation of the racemic products using Rh_2(R/S-NTTL)$_4$ as a catalyst (racemic Rh_2(NTTL)$_4$ catalyst was made by mixing equal amounts of R and S enantiomers).

2. Catalyst structure

Table S1. Structure of known catalysts

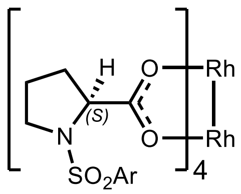

Ar = *p*-($C_{12}H_{25}$)C_6H_4

Class: Prolinato

Name: Rh_2(*S*-DOSP)$_4$[1]

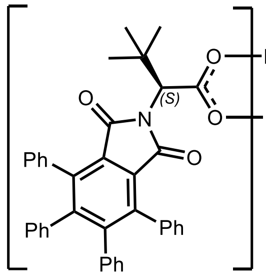

Class: Phthalimido

Name: Rh_2(*S*-TPPTTL)$_4$[2]

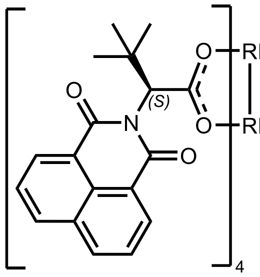

Class: Napthalimido

Name: Rh_2(*S*-NTTL)$_4$[3]

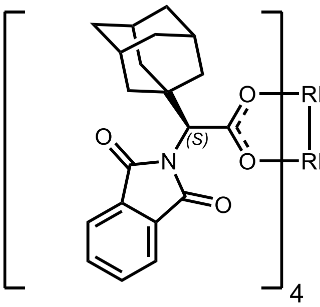

Class: Phthalimido

Name: Rh_2(*S*-PTAD)$_4$[4]

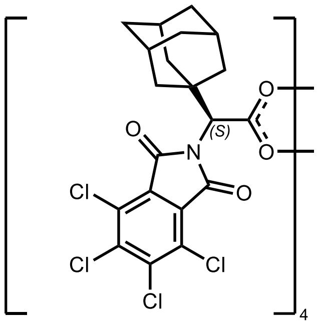

Class: Phthalimido

Name: Rh_2(*S*-TCPTAD)$_4$[4]

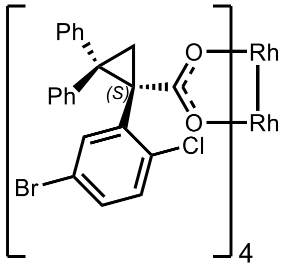

Class: TPCP

Name: Rh_2(*S*-*p*-2Cl5BrTPCP)$_4$[5]

Table S2. Summary of novel catalysts of napthalimido family prepared in this study

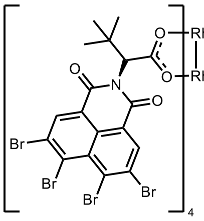

Class: Napthalimido
Name: Rh_2(*S*-*tetra*-BrNTTL)$_4$ (**9**)

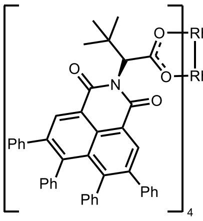

Class: Napthalimido
Name: Rh_2(S-*tetra*-C_6H_5NTTL)$_4$ (**7a**)

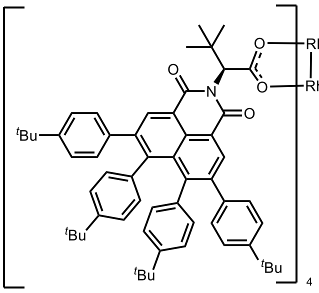

Class: Napthalimido
Name: Rh_2(S-*tetra*-4-^{t}BuC_6H_4NTTL)$_4$ (**7b**)

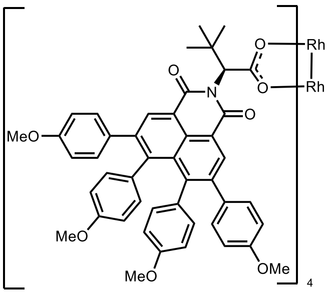

Class: Napthalimido
Name: Rh_2(S-*tetra*-4-MeOC_6H_4NTTL)$_4$ (**7c**)

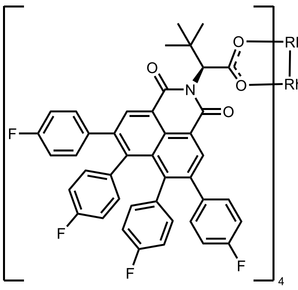

Class: Napthalimido
Name: Rh_2(S-*tetra*-4-FC_6H_4NTTL)$_4$ (**7d**)

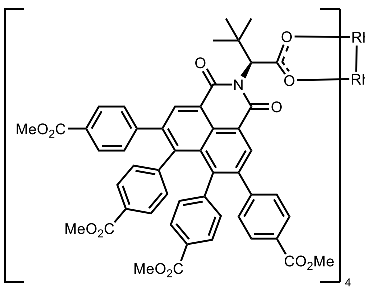

Class: Napthalimido
Name: Rh_2(S-*tetra*-4-CO_2MeC_6H_4NTTL)$_4$ (**7e**)

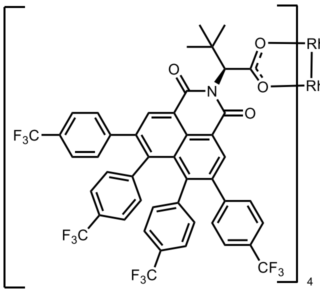

Class: Napthalimido
Name: Rh_2(S-*tetra*-4-$CF_3C_6H_4$NTTL)$_4$ (**7f**)

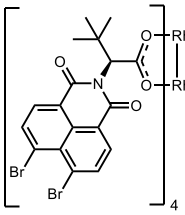

Class: Napthalimido
Name: Rh_2(*S*-*di*-BrNTTL)$_4$ (**10**)

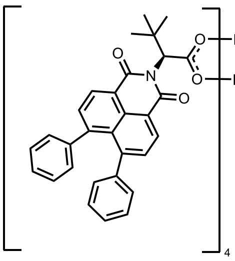

Class: Napthalimido
Name: Rh_2(S-*di*-C_6H_5NTTL)$_4$ (**8a**)

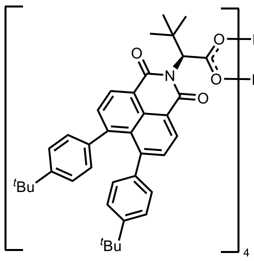

Class: Napthalimido
Name: Rh_2(S-*di*-4-^{t}BuC_6H_5NTTL)$_4$ (**8b**)

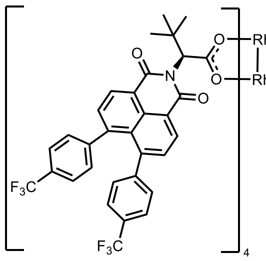

Class: Napthalimido
Name: Rh_2(S-*di*-4-$CF_3C_6H_5$NTTL)$_4$ (**8c**)

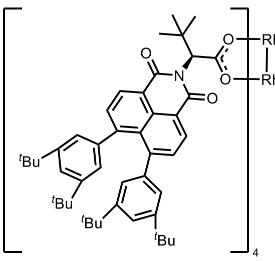

Class: Napthalimido
Name: Rh_2(S-*di*-3,5-*di*-^{t}BuC_6H_5NTTL)$_4$ (**8d**)

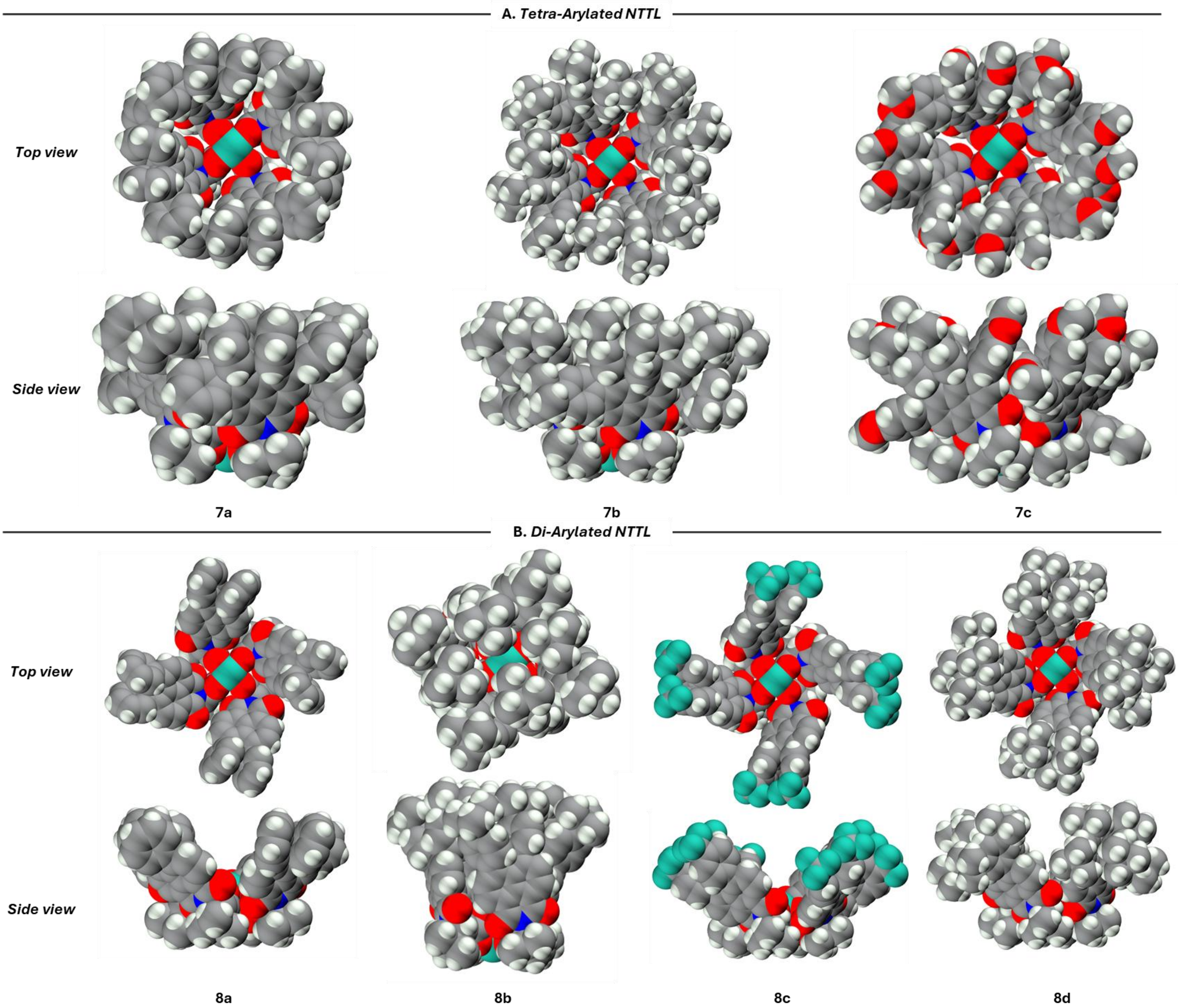

Figure S1. Solid-state structure of novel dirhodium tetracarboxylate catalysts. **A.** Solid state structures of Rh_2(*S*-*tetra*-ArNTTL)$_4$. **B.** Solid state structures of Rh_2(*S*-*di*-Ar-NTTL)$_4$. Axial coordinating ligands and solvent molecules were omitted for clarity. These images were rendered by VMD program with the coloring atoms as follows: hydrogen (white), carbon (black), oxygen (red), nitrogen (blue), fluorine (bright green), rhodium (green)

3. Synthesis of triazoles

Triazoles used in this study were synthesized according to the reported procedures.[6-10]

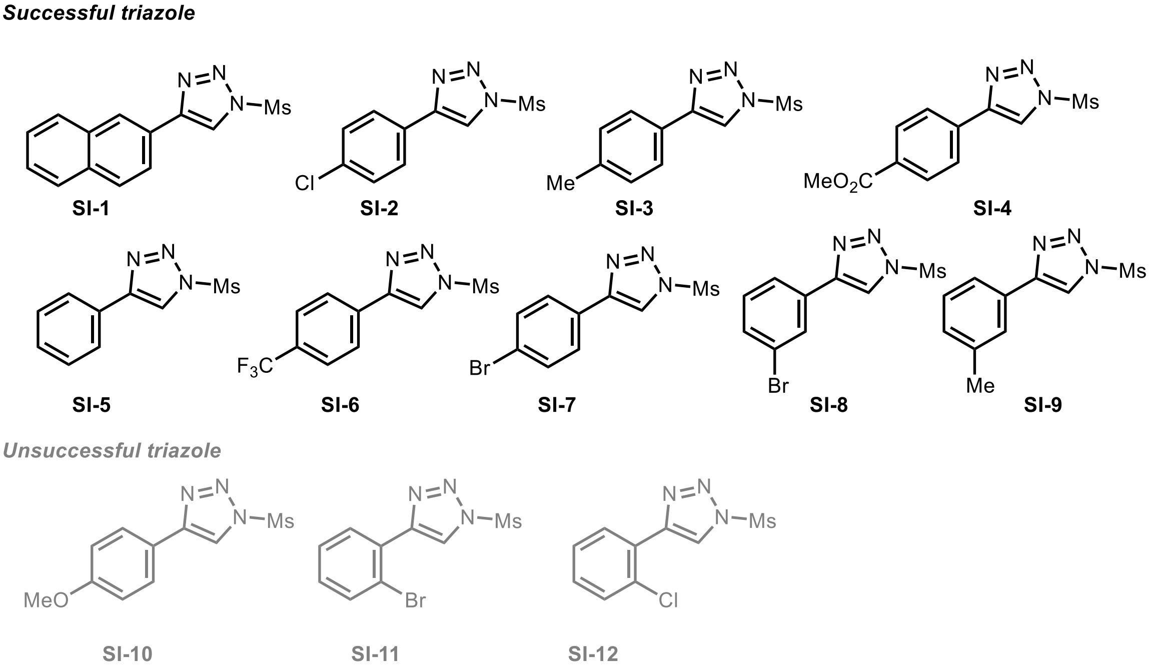

- Compound **SI-1** were synthesized according to the reported procedure.[8]
- Compound **SI-2**, **SI-3, SI-5**, **SI-6**, and **SI-12** were synthesized according to the reported procedure.[6]
- Compound **SI-4**, **SI-7**, **SI-8**, **SI-10**, and **SI-11** were synthesized according to the reported procedure.[9]
- Compound **SI-9** was synthesized according to the reported procedure.[11]

4. Acquisition and synthesis of Substrates

Table S3. Commercially available substrates and substrates from in-house suppliers of Sanofi are listed below:

No.	Name	Structure	Vendor	CAS
01	1-bromo-4-cyclohexylbenzene	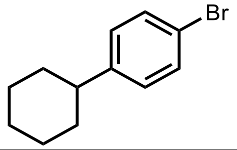	Ambeed	25109-28-8
02	4-Cyclohexylbenzoic acid	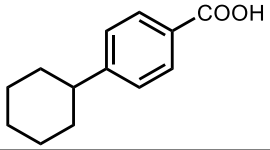	Ambeed	20029-52-1
03	4-Cyclohexylphenol	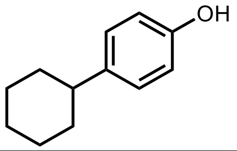	Ambeed	1131-60-8
04	1,4-Dicyclohexylbenzene	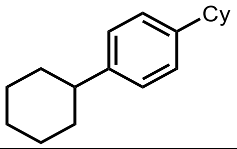	Ambeed	1087-02-1
05	1,3-dichloro-6-cyclohexylisoquinoline	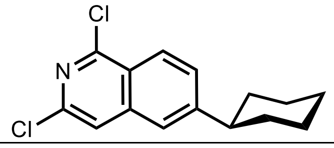	Sanofi	-
06	2-chloro-3-cyclohexyl-6-methylpyridine	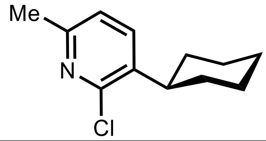	Sanofi	-

07	5-cyclohexyl-2-(trifluoromethyl)pyridine	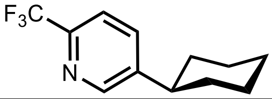	Sanofi	-
08	1-chloro-4-cyclohexylbenzene	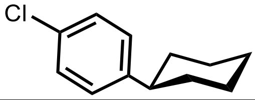	Sanofi	-
09	1-cyclohexyl-4-(trifluoromethyl)benzene	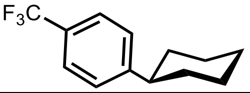	Sanofi	-
10	4-cyclohexyl toluene	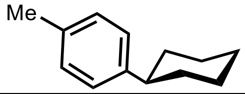	Ambeed	4501-36-4
11	cyclohexylbenzene	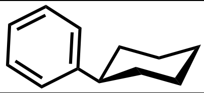	Aldrich	827-52-1
12	1,3-di-tert-butyl-5-cyclohexylbenzene	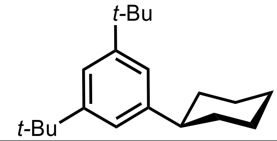	Sanofi	-
13	1-cyclohexyl-4-methoxybenzene	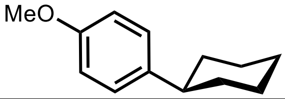	Sanofi	-
14	1-cyclohexyl-4-tert-butylbenzene	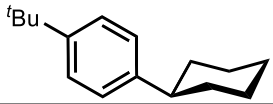	Sanofi	-
15	methyl 4-cyclohexylbenzoate	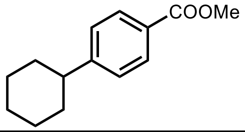	Sanofi	-

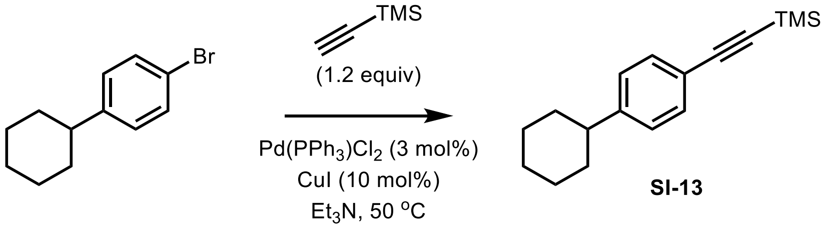

Under a nitrogen atmosphere, to a toluene:Et_3N solution (3:10, v/v, 17 mL, 0.6M) of $Pd(PPh_3)_2Cl_2$ (366 mg, 0.5 mmol, 5 mol%) and CuI (190 mg, 1.0 mmol, 10 mol%) was added 1-bromo-4-cyclohexylbenzene (1.86 ml, 2.39 g, 10.0 mmol, 1 equiv) and stirred for 10 min, then trimethylsilylacetylene (2.10 mL, 15.0 mmol, 1.5 equiv) was added dropwise. The resulting suspension was allowed to be stirred overnight at 50 °C. After completion of the reaction, the mixture was filtered through silica plug with a layer of celite and concentrated under reduced pressure. The residue was purified by flash chromatography (SiO_2, hexane) to give ((4-cyclohexylphenyl)ethynyl)trimethylsilane **S1** (1.43 g, 56% yield) as clear oil which solidifies after a few days.

R_f (hexane) = 0.5 (UV active)

^{1}H NMR (400 MHz, $CDCl_3$) δ 7.38 (d, J = 8.2 Hz, 2H), 7.13 (d, J = 8.2 Hz, 2H), 2.60 – 2.38 (m, 1H), 1.93 – 1.78 (m, 4H), 1.74 (d, J = 13.1 Hz, 1H), 1.49 – 1.30 (m, 4H), 1.29 – 1.14 (m, 1H), 0.24 (s, 9H). *NMR spectrum matched reported literature.*[12]

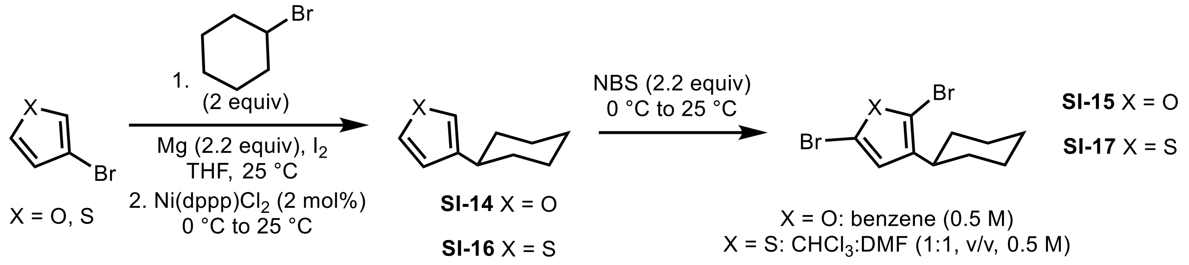

The synthesis for 2,5-dibromo-3-cyclohexylfuran and 2,5-dibromo-3-cyclohexylthiophene are adapted from literature:[5, 13, 14]

To a flamed-dried 50 ml round bottom flask was added magnesium turnings (2.2 equiv) and 1-2 beans of I_2. Dried THF (0.5M) was added bromocyclohexane (2 equiv) dropwise. The reaction mixture was stirred at room temperature for 2 hours. To another flamed-dried 50 ml flask under N_2 was added Ni(dppp)Cl_2 (2 mol%) and 3-bromofurane (or 3-bromothiophene, 1 equiv). The flask was vacuumed, refilled with N_2, and dried THF (0.5M) was added to dissolve the mixture. Then, the mixture was cooled to 0 ºC via ice bath followed by the 20 min slow addition of the above cyclohexylmagesium bromide solution. The reaction mixture was less warm up to room temperature and stirred overnight at room temperature. The crude reaction mixture was filtered through a silica plug wash with hexane. The combined organic filtrate was loaded on silica gel and purified by flash chromatography (SiO_2, 100% hexane) to provide a clear oil of 3-cyclohexylfuran **SI-14** in 43% yield (871.3 mg, 13.6 mmol scale) (or 3-cyclohexylthiophene **SI-16** in 71% yield (3.63 g, 30.7 mmol scale)). *Note:* After purification the products were used immediately for the next step.

The product from the last step was dissolved in the corresponding anhydrous solvents (DMF/$CHCl_3$ for thiophene, benzene for furan, 0.5M). Then, N-bromosuccinimide (2.2 equiv.) was added slowly at 0 ºC and the resulting mixture was stirred overnight at room temperature (23 ºC). The reaction solution was quenched by saturated solution of $Na_2S_2O_3$, followed by the extraction by hexane (3x50 mL), washing with H_2O and brine. After dried over Na_2SO_4, the product was concentrated under reduced pressure and purified by flash chromatography (SiO_2, hexane) to provide colorless oil of 2,5-dibromo-3-cyclohexylfuran **SI-15** in 77% yield (1.38 g, 5.8 mmol scale) (or 2,5-dibromo-3-cyclohexylthiophene **SI-17** in 93% yield (6.37 g, 21.0 mmol scale)). *Note:* these products decomposed at room temperature over times, storage at -20 ºC.

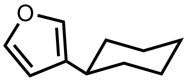

3-cyclohexylfuran (SI-14)

Appearance: clear oil (817.3 mg, 43% yield)

R_f (hexane) = 0.5 ($KMnO_4$)

^{1}H NMR (400 MHz, $CDCl_3$) δ 7.36 (t, *J* = 1.7 Hz, 1H), 7.21 (dt, *J* = 1.7, 0.9 Hz, 1H), 6.35 – 6.30 (m, 1H), 2.45 (td, *J* = 11.1, 5.6 Hz, 1H), 1.96 (s, 2H), 1.87 – 1.77 (m, 2H), 1.77 – 1.68 (m, 1H), 1.48 – 1.16 (m, 6H). *NMR spectrum matched reported literature.*[14]

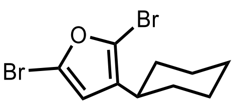

2,5-dibromo-3-cyclohexylfuran (SI-15)

Appearance: clear oil (1.38 g, 77% yield)

R_f (hexane) = 0.6 ($KMnO_4$)

^{1}H NMR (400 MHz, $CDCl_3$) δ 6.24 (s, 1H), 2.38 (tt, J = 11.7, 3.5 Hz, 1H), 1.77 (s, 5H), 1.41 – 1.17 (m, 5H).

^{13}C NMR (101 MHz, $CDCl_3$) δ 132.5, 121.2, 118.3, 113.1, 35.2, 32.9, 26.4, 26.0.

HRMS (+p APCI) calcd. for [$C_{10}H_{13}O^{79}Br_2$] ([M+H]$^+$) 306.9328 found 306.9326.

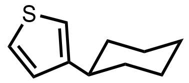

3-cyclohexylthiophene (SI-16)

Appearance: clear oil (3.63 g, 71% yield)

R_f (hexane) = 0.6-0.7 ($KMnO_4$)

^{1}H NMR (400 MHz, $CDCl_3$) δ 7.24 (dd, J = 5.0, 2.9 Hz, 1H), 6.99 (dd, J = 5.0, 1.3 Hz, 1H), 6.93 (dt, J = 2.9, 1.1 Hz, 1H), 2.61 (td, J = 8.2, 4.4 Hz, 1H), 1.97 (d, J = 6.4 Hz, 1H), 1.90 – 1.77 (m, 2H), 1.73 (dtd, J = 11.1, 3.2, 1.7 Hz, 1H), 1.44 – 1.31 (m, 4H), 1.29 – 1.20 (m, 1H). *NMR spectrum matched reported literature.*[13]

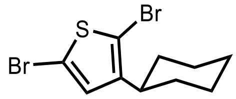

2,5-dibromo-3-cyclohexylthiophene (SI-17)

Appearance: clear oil (6.37 g, 93% yield)

R_f (hexane) = 0.6-0.7 ($KMnO_4$)

^{1}H NMR (400 MHz, $CDCl_3$) δ 6.81 (s, 1H), 2.65 (tt, J = 11.7, 3.3 Hz, 1H), 1.87 – 1.68 (m, 5H), 1.46 – 1.14 (m, 5H).

^{13}C NMR (101 MHz, $CDCl_3$) δ 147.9, 129.1, 110.6, 106.9, 39.3, 33.2, 26.6, 26.1.

HRMS (+p APCI) calcd. for [$C_{10}H_{12}{}^{79}Br_2{}^{32}S$] ([M+H]$^+$) 321.9021 found 321.9021.

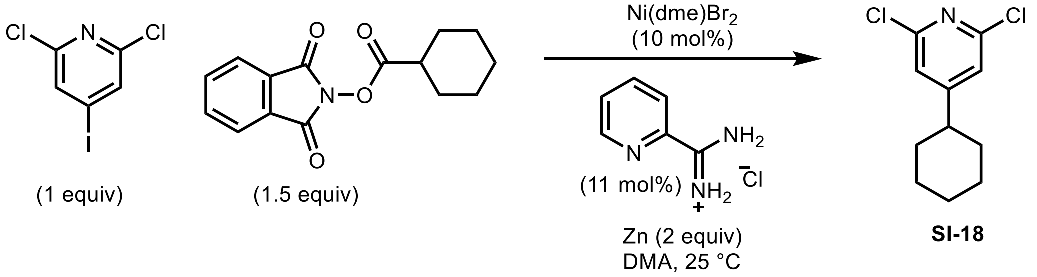

To a 25 mL round-bottom flask containing a Teflon-coated stir-bar was added NiBr2(diglyme) (154 mg, 0.5 mmol, 10 mol%) and amino(pyridin-2-yl)methaniminium chloride (86.7 mg, 0.55 mmol, 11 mol%). The reaction flask was vacuum and refilled with N_2 3 times. Anhydrous DMA (5 mL) was added via syringe and the resulting mixture was

stirred on the benchtop (~1200 rpm) at rt under Ar for 15 minutes to allow ligation of the nickel. The color of the reaction mixture became blue green. 2,6-dichloro-4-iodopyridine (1.37 g, 5.00 mmol, 1.0 equiv) was added and 1,3-dioxoisoindolin-2-yl cyclohexanecarboxylate (2.05 g, 7.50 mmol, 1.5 equiv) and zinc dust (654 mg, 10.0 mmol, 2 equiv) were added in one portion to the reaction vessel by quickly removing the septum and replacing it. The reaction mixture was warmed to 28 °C in an oil bath and stirred at that temperature under Ar for overnight. The reaction mixture was filtered through a plug of silica. The pad was washed with Et_2O (100 mL) and concentrated. The resulting residue was purified by flash chromatography (SiO_2, 0-25% Et_2O in hexane) to yield 2,6-dichloro-4-cyclohexylpyridine **SI-18** as an off-white solid (196.3 mg, 17% yield). *Note*: In case the product contaminated with the dehalogenated product, further purification by Kugerol distillation (130 ºC, 10 mmHg) was carried out.

R$_f$ (5H/1Et_2O) = 0.35

^{1}H NMR (400 MHz, $CDCl_3$) δ 7.10 (s, 2H), 2.57 – 2.42 (m, 1H), 1.93 – 1.81 (m, 4H), 1.80 – 1.67 (m, 1H), 1.47 – 1.30 (m, 4H), 1.30 – 1.16 (m, 1H).

^{13}C NMR (101 MHz, $CDCl_3$) δ 162.8, 150.6, 121.7, 43.8, 33.4, 26.4, 25.8.

HRMS (+p ESI) calcd. for [$C_{11}H_{14}N^{35}Cl_2$] ([M+H]$^+$) 230.0498 found 230.0496

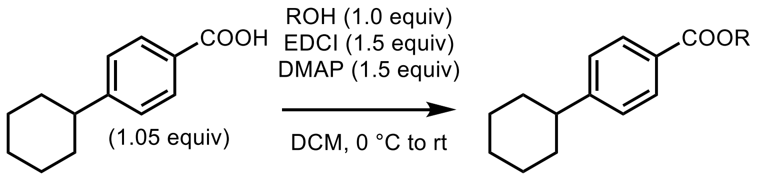

The alcohol (1 equiv, 5.0 mmol), 4-cyclohexylbenzoic acid (1.05 equiv), DMAP (1.5 equiv) and EDCI (1.44 g, 7.50 mmol, 1.5 equiv) were dissolved in DCM (0.25 M) and stirred at 0 ºC for 1 h, and then stirred at room temperature overnight. Upon completion, the reaction mixture was added 10 ml HCl (2M) and then the aqueous layer was extracted with DCM (10 mL) 2 times. The organic layer was combined and washed by water then brine, dried over anhydrous Na2SO4, and concentrated under vacuum. The residue was purified with flash chromatography (SiO_2, hexane/diethyl ether) to yield the corresponding ester.

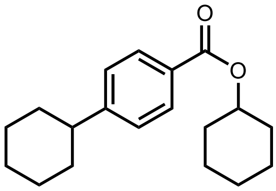

cyclohexyl 4-cyclohexylbenzoate (SI-19)

This compound was synthesized according to the above procedure for esterification to afford compound **SI-19** as a white solid (680.6 mg, 79% yield, 3.0 mmol scale).

R$_f$ (5H/1Et_2O) = 0.66

^{1}H NMR (600 MHz, $CDCl_3$) δ 7.97 (d, *J* = 7.9 Hz, 2H), 7.27 (d, *J* = 6.9 Hz, 2H), 5.02 (tt, *J* = 8.6, 3.9 Hz, 1H), 2.55 (td, *J* = 11.4, 3.4 Hz, 1H), 2.00 – 1.72 (m, 9H), 1.61 – 1.54 (m, 3H), 1.50 – 1.32 (m, 7H), 1.26 (tdd, *J* = 12.5, 8.9, 3.2 Hz, 1H).

^{13}C NMR (151 MHz, $CDCl_3$) δ 166.2, 153.4, 129.8, 128.8, 126.9, 72.9, 44.8, 34.3, 31.8, 26.9, 26.2, 25.6, 23.8.

HRMS (+p ESI) calcd. for [$C_{19}H_{27}O_2$] ([M+H]$^+$) 287.2006 found 287.2003.

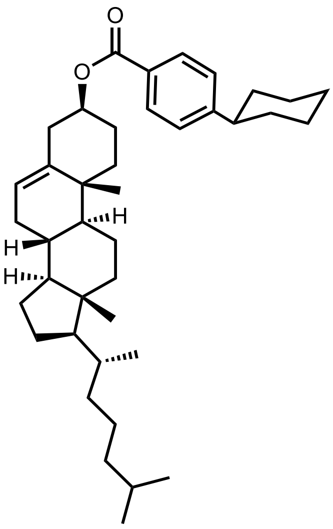

(3S,8S,9S,10R,13R,14S,17R)-10,13-dimethyl-17-((R)-6-methylheptan-2-yl)-2,3,4,7,8,9,10,11,12,13,14,15,16,17-tetradecahydro-1H-cyclopenta[a]phenanthren-3-yl 4-cyclohexylbenzoate (**SI-20**)

This compound was synthesized according to the above procedure for esterification to afford compound **SI-20** as a white solid (1.17 g, 84% yield, 2.5 mmol scale).

R$_f$ (2H/1DCM) = 0.2 (UV 254 nm)

^{1}H NMR (400 MHz, CDCl$_3$) δ 7.96 (d, *J* = 8.3 Hz, 2H), 7.26 (d, *J* = 8.4 Hz, 2H), 5.41 (d, *J* = 1.9 Hz, 1H), 4.84 (dtd, *J* = 12.3, 8.4, 4.5 Hz, 1H), 2.56 (dd, *J* = 7.2, 4.3 Hz, 1H), 2.44 (d, *J* = 7.6 Hz, 2H), 2.07 – 1.66 (m, 11H), 1.55 – 1.07 (m, 20H), 1.06 (s, 3H), 1.03 – 0.94 (m, 3H), 0.92 (d, *J* = 6.5 Hz, 3H), 0.87 (dd, *J* = 6.6, 1.9 Hz, 6H), 0.68 (s, 3H).

^{13}C NMR (101 MHz, CDCl$_3$) δ 166.2, 153.4, 139.9, 129.8, 128.5, 126.9, 122.9, 74.4, 56.8, 56.2, 50.2, 44.8, 42.5, 39.9, 39.7, 38.4, 37.2, 36.8, 36.3, 35.9, 34.3, 32.1, 32.0, 28.4, 28.2, 28.0, 26.9, 26.2, 24.4, 24.0, 23.0, 22.7, 21.2, 19.5, 18.9, 12.0.

HRMS (+p ESI) calcd. for [$C_{40}H_{61}O_2$] ([M+H]$^+$) 573.4666 found 573.4668.

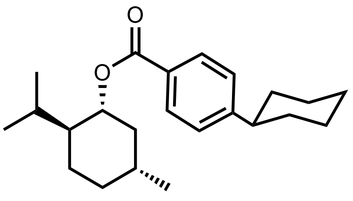

(1R,2S,5R)-2-isopropyl-5-methylcyclohexyl 4-cyclohexylbenzoate (SI-21)

This compound was synthesized according to the above procedure for esterification to afford compound **SI-21** as a white solid (1.61 g, 94% yield, 5.00 mmol scale).

R$_f$ (2H/1DCM) = 0.3 (UV 254 nm)

^{1}H NMR (400 MHz, CDCl$_3$) δ 7.96 (d, *J* = 8.3 Hz, 2H), 7.27 (d, *J* = 7.4 Hz, 2H), 4.91 (td, *J* = 10.8, 4.4 Hz, 1H), 2.63 – 2.47 (m, 1H), 2.13 – 2.04 (m, 1H), 1.96 (pd, *J* = 7.0, 2.8 Hz, 1H), 1.91 – 1.80 (m, 4H), 1.79 – 1.68 (m, 3H), 1.57 – 1.50 (m, 2H), 1.50 – 1.17 (m, 6H), 1.17 – 1.02 (m, 2H), 0.92 (d, *J* = 4.8 Hz, 3H), 0.90 (d, *J* = 5.2 Hz, 3H), 0.78 (d, *J* = 6.9 Hz, 3H). *NMR spectrum matched reported literature.*[15]

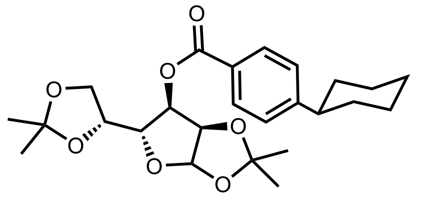

(5R,6R,6aR)-5-((R)-2,2-dimethyl-1,3-dioxolan-4-yl)-2,2-dimethyltetrahydrofuro[2,3-d][1,3]dioxol-6-yl 4-cyclohexylbenzoate (SI-22)

This compound was synthesized according to the above procedure for esterification to afford compound **S9** as a thick clear oil (1.61 g, 98% yield, 5.00 mmol scale).

$\mathbf{R_f}$ (3H/1EA) = 0.3 (UV 254 nm)

^{1}**H NMR (400 MHz, CDCl$_3$)** δ 7.97 (d, J = 8.4 Hz, 2H), 7.28 (d, J = 8.2 Hz, 2H), 5.89 (d, J = 3.8 Hz, 1H), 5.05 (dd, J = 8.4, 5.1 Hz, 1H), 4.97 (dd, J = 5.1, 3.8 Hz, 1H), 4.42 – 4.30 (m, 2H), 4.11 (dd, J = 8.6, 6.7 Hz, 1H), 3.98 (dd, J = 8.6, 5.8 Hz, 1H), 2.56 (ddd, J = 11.4, 7.9, 3.2 Hz, 1H), 1.91 – 1.80 (m, 4H), 1.76 (d, J = 12.5 Hz, 1H), 1.54 (s, 3H), 1.48 – 1.36 (m, 7H), 1.33 (s, 3H), 1.31 (s, 3H), 1.28 – 1.21 (m, 1H).

^{13}**C NMR (101 MHz, CDCl$_3$) δ** 165.8, 154.2, 130.1, 127.1, 127.0, 113.2, 110.1, 104.4, 78.0, 77.8, 75.2, 73.0, 65.7, 44.9, 34.3, 34.2, 26.86, 26.83, 26.81, 26.4, 26.1, 25.2.

HRMS (+p ESI) calcd. for [$C_{25}H_{34}O_7{}^{23}Na$] ([M+H]$^+$) 469.2197 found 469.2195.

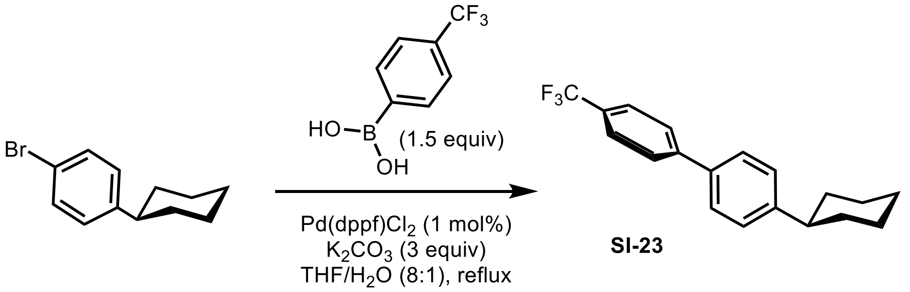

To 50 ml round-bottom flask fitted with a findenser, was added 1-bromo-4-cyclohexylbenzene (1.20 g, 5.00 mmol, 1.0 equiv), (4-(trifluoromethyl)phenyl)boronic acid (1.42 g, 7.50 mmol, 1.5 equiv), K_2CO_3 (2.07 g, 15.0 mmol, 3.0 equiv) and Pd(dppf)Cl_2 (36.6 mg, 50.0 μmol, 0.01 equiv). The flask was vacuumed and refilled with N_2. Then, A mixture of THF/Water (25 ml, 8:1 v/v) was added. The reaction was refluxed at 78℃ for overnight. The reaction mixture was extracted with CH_2Cl_2. The combined organic layer was washed with H_2O, dried over Na_2SO_4 and concentrated in vacuo. The crude mixture was purified by flash column chromatography (SiO_2, 100% hexane) to give **SI-23** as a white solid (1.37 g, 90% yield).

$\mathbf{R_f}$ (hexane) = 0.60 (UV 254 nm)

^{1}**H NMR (400 MHz, CDCl$_3$) δ** 7.68 (s, 4H), 7.54 (d, J = 8.3 Hz, 2H), 7.32 (d, J = 8.1 Hz, 2H), 2.63 – 2.49 (m, 1H), 1.99 – 1.83 (m, 4H), 1.78 (dtt, J = 12.6, 3.1, 1.5 Hz, 1H), 1.53 – 1.36 (m, 4H), 1.34 – 1.21 (m, 1H).

^{19}**F NMR (376 MHz, CDCl$_3$) δ** -62.31.

^{13}**C NMR (101 MHz, CDCl$_3$) δ** 148.5, 144.8, 137.4, 129.1 (q, J = 32.4 Hz), 127.6, 127.3, 127.3, 125.8 (q, J = 3.9 Hz), 123.1, 44.4, 34.6, 27.0, 26.3. *Note. The CF_3 carbon splitting overlapped with other peaks, preventing full analysis of the signal.*

HRMS (+p APCI) calcd. for [$C_{19}H_{19}F_3$] ([M]$^+$) 304.1433 found 304.1434.

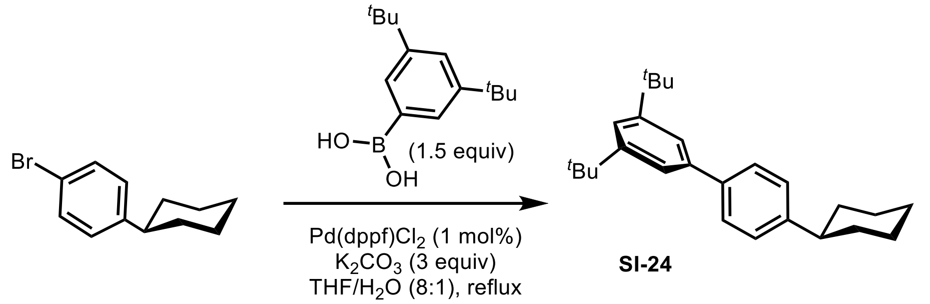

To 50 ml round-bottom flask fitted with a findenser, was added 1-bromo-4-cyclohexylbenzene (1.20 g, 5.00 mmol, 1.0 equiv), (3,5-di-tert-butylphenyl)boronic acid (1.76 g, 7.50 mmol, 1.5 equiv), K_2CO_3 (2.07 g, 15.0 mmol, 3.0 equiv) and Pd(dppf)Cl_2 (36.6 mg, 50.0 μmol, 0.01 equiv). The flask was vacuumed and refilled with N_2. Then, A mixture of THF/Water (25 ml, 8:1 v/v) was added. The reaction was refluxed at 78℃ for overnight. The reaction mixture was extracted with CH_2Cl_2. The combined organic layer was washed with H_2O, dried over Na_2SO_4 and concentrated in vacuo. The crude mixture was purified by flash column chromatography (SiO_2, 100% hexane) to give **SI-24** as a white solid (1.48 g, 85% yield).

R_f (hexane) = 0.70 (UV 254 nm)

^{1}H NMR (400 MHz, $CDCl_3$) δ δ 7.51 (d, J = 8.2 Hz, 2H), 7.44 – 7.35 (m, 3H), 7.28 (d, J = 8.1 Hz, 2H), 2.63 – 2.49 (m, 1H), 1.99 – 1.83 (m, 4H), 1.82 – 1.72 (m, 1H), 1.53 – 1.40 (m, 4H), 1.39 (s, 18H), 1.33 – 1.23 (m, 1H).

^{13}C NMR (101 MHz, $CDCl_3$) δ 151.1, 147.0, 140.8, 140.3, 127.5, 127.2, 121.8, 121.2, 44.4, 35.1, 34.7, 31.7, 27.1, 26.3.

HRMS (+p APCI) calcd. for [$C_{26}H_{36}$] ([M]$^+$) 348.2812 found 348.2813.

General procedure A. Synthesis of aryl cyclohexane derivatives.

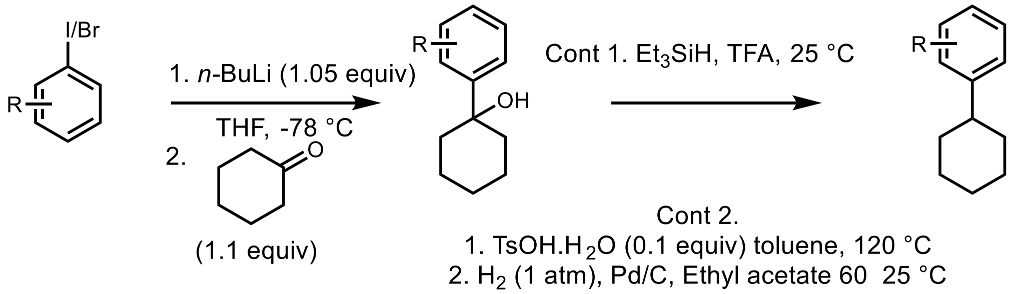

Nucleophilic addition. To a flamed dried round bottom flask charged with magnetic stir bar was added the corresponding aryl bromide or aryl iodide (1.0 equiv). Dried THF (0.4 M) was added to the above flask and the mixture was cooled down to -78℃ by a dry ice/acetone bath. Upon reaching the desired temperature, n-BuLi solution (2.5 M in hexane, 1.1 equiv) was added dropwise into the reaction mixture in 10-20 mins. The mixture kept stirring at -78℃ for 2 hours before cyclohexanone (1.05 equiv) was added in one portion via syringe. The mixture was then removed from the dry ice/acetone bath and slowly warmed up to room temperature. The reaction was then quenched with water and extracted with ethyl acetate 2 times. The combined organic layer was dried over anhydrous sodium sulfate, and purified by flash chromatography (SiO_2, 0-20% diethyl ether in hexane) to give the desired alcohol intermediate.

Deoxygenation. The deoxygenation is carried out by either by 1 step procedure (condition 1 and 2) or 2 step procedure.

Condition 1. *Reduction with Et_3SiH.* Under the N_2 atmosphere, the above alcohol intermediate (1.0 equiv) was then dissolved in TFA (0.5 M). The mixture was cooled down to 0℃ by an ice bath before triethyl silane (2.0 equiv) was

added in one portion via syringe. The mixture was let warm up to room temperature and stir overnight. Upon completion, water was added, and the mixture was extracted by hexane. The hexane layer was washed with $NaHCO_3$ followed by brine. The mixture was then filtered through a silica plug and wash with hexane. The combined organic layer was concentrated under vacuum and purified further by Kugelrohr distillation.

***Condition 2**. 2 step procedure.* The above alcohol intermediate (1.0 equiv) was dissolved in toluene (0.2 M). TsOH.H_2O (1.0 equiv) was added to the toluene solution, and the mixture was heated to reflux (120℃) for 4 hours. The reaction is cooled down to room temperature before a solution of 10% $NaHCO_3$ was added. The mixture was extracted with hexane and dried over anhydrous sodium sulfate. The solvent was then removed under vacuum to afford crude oil. The crude oil was passed through a silica plug using hexane as eluent. The solvent was again removed under vacuum to provide clear oil, which was used for the next step without further purification.

The obtained clear oil was dissolved in ethyl acetate (1.0 M) then Pd/C (0.05 equiv, 5 wt%) was added. The mixture was purging with hydrogen gas (1 atm) for 10 mins. After that, the mixture was heated to 60℃ under hydrogen balloon overnight. Upon completion, the crude mixture was filtered through a silica plug washing with hexane. The obtained organic solution was concentrated under vacuum to afford the desired product without further purification. In case the products obtained from silica plug are not clean, they can be further purified by Kugelrohr distillation.

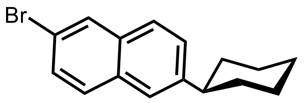

2-bromo-6-cyclohexylnaphthalene (SI-25)

This compound was synthesized according to the general procedure A using 2,6-dibromonaphthalene (2.86 g, 10.0 mmol, 1 equiv) followed by reduction by condition 1. The purified mixture was further purified by Kugelrohr distillation (165 ºC, 0.4 torr) to afford **SI-25** follow as a white solid (1.0 g, 47% yield).

$\mathbf{R_f}$ (100% hexane) = 0.6

^{1}**H NMR (400 MHz, CDCl$_3$) δ** 7.95 (d, *J* = 1.9 Hz, 1H), 7.66 (t, *J* = 9.1 Hz, 2H), 7.59 (s, 1H), 7.50 (dd, *J* = 8.7, 2.0 Hz, 1H), 7.39 (dd, *J* = 8.4, 1.8 Hz, 1H), 2.74 – 2.58 (m, 1H), 1.95 (d, *J* = 12.1 Hz, 2H), 1.88 (d, *J* = 12.3 Hz, 2H), 1.83 – 1.74 (m, 1H), 1.55 – 1.37 (m, 4H), 1.30 (dtd, *J* = 17.9, 7.8, 4.4 Hz, 1H).
^{13}**C NMR (101 MHz, CDCl$_3$) δ** 146.3, 133.3, 132.2, 129.7, 129.4, 129.2, 127.4, 127.0, 124.6, 118.9, 44.7, 34.4, 27.0, 26.
HRMS (+p APCI) calcd. for [$C_{16}H_{17}{}^{79}Br$] ([M]$^+$) 288.0508 found 288.0507.

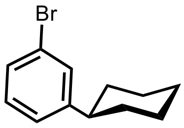

1-bromo-3-cyclohexylbenzene (SI-26)

This compound was synthesized according to general procedure A using 1,3-dibromobenzene (2.36 g, 10.0 mmol, 1 equiv) followed by reduction by condition 1. The compound was purified by Kugelrohr distillation (115 ºC, 0.5 torr) to afford **SI-26** follow as a white solid (1.3 g, 54% yield)

^{1}H NMR (400 MHz, $CDCl_3$) δ 7.36 (s, 1H), 7.31 (d, *J* = 7.0 Hz, 1H), 7.19 – 7.09 (m, 2H), 2.48 (ddd, *J* = 11.9, 8.5, 5.0 Hz, 1H), 1.95 – 1.79 (m, 4H), 1.76 (ddt, *J* = 14.2, 3.1, 1.6 Hz, 1H), 1.49 – 1.32 (m, 4H), 1.30 – 1.18 (m, 1H). NMR spectrum matched the reported literature. [16]

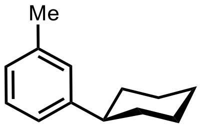

1-cyclohexyl-3-methylbenzene (SI-27)

This compound was synthesized according to general procedure A using 1-iodo-3-methylbenzene (3.27 g, 15.0 mmol, 1 equiv) followed by reduction by condition 2. The crude mixture was purified by flash chromatography (SiO_2, 100% hexane) to afford **SI-27** follow as a clear oil (1.4 g, 56% yield)

^{1}H NMR (400 MHz, $CDCl_3$) δ 7.21 (t, *J* = 7.5 Hz, 1H), 7.10 – 6.98 (m, 3H), 2.49 (ddt, *J* = 11.4, 6.7, 3.6 Hz, 1H), 2.37 (s, 3H), 1.97 – 1.82 (m, 4H), 1.78 (ddt, *J* = 12.6, 3.1, 1.5 Hz, 1H), 1.50 – 1.35 (m, 4H), 1.30 (d, *J* = 3.3 Hz, 1H). NMR spectrum matched the reported literature.[17]

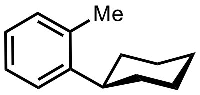

1-cyclohexyl-2-methylbenzene (SI-28)

This compound was synthesized according to general procedure A using 1-iodo-2-methylbenzene (3.27 g, 15.0 mmol, 1 equiv) followed by reduction by condition 2. The crude mixture was purified by flash chromatography (SiO_2, 100% hexane) to afford **SI-28** follow as a clear oil (1.0 g, 40% yield).

^{1}H NMR (400 MHz, $CDCl_3$) δ 7.24 (dd, *J* = 7.8, 1.6 Hz, 1H), 7.21 – 7.12 (m, 2H), 7.09 (td, *J* = 7.2, 1.6 Hz, 1H), 2.73 (tt, *J* = 8.4, 3.1 Hz, 1H), 2.35 (s, 3H), 1.95 – 1.72 (m, 5H), 1.51 – 1.36 (m, 4H), 1.36 – 1.28 (m, 1H). NMR spectrum matched the reported literature.[18]

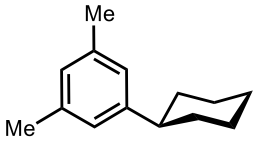

1-cyclohexyl-3,5-dimethylbenzene (SI-29)

This compound was synthesized according to general procedure A using 1-bromo-3,5-dimethylbenzene (5.55 g, 30.0 mmol, 1 equiv) followed by reduction by condition 2. The crude mixture was purified by flash chromatography (SiO_2, 100% hexane) to afford **DL-07-110-02** follow as a clear oil (4.14 g, 73% yield).

^{1}H NMR (400 MHz, $CDCl_3$) δ 6.90 (s, 3H), 2.53 – 2.45 (m, 1H), 2.36 (s, 6H), 1.98 – 1.85 (m, 4H), 1.85 – 1.74 (m, 1H), 1.58 – 1.39 (m, 4H), 1.32 (tdd, *J* = 12.5, 7.0, 3.7 Hz, 1H).

^{13}C NMR (101 MHz, $CDCl_3$) δ 148.3, 137.8, 127.6, 124.8, 44.7, 34.7, 27.1, 26.4, 21.5.

HRMS (+p APCI) calcd. for [$C_{14}H_{20}$] ([M]$^{+}$) 188.1560 found 188.1561.

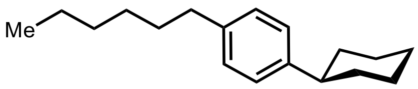

1-cyclohexyl-4-hexylbenzene (SI-30)

This compound was synthesized according to general procedure A using 1-bromo-4-cyclohexylbenzene (4.78 g, 20.0 mmol, 1.0 equiv) and hexanal (2.58 ml, 2.10 g, 21.0 mmol, 1.05 equiv) followed by reduction by condition 2. The crude mixture was purified by flash chromatography (SiO_2, 100% hexane) to afford **SI-30** follow as a clear oil (4.4 g, 90% yield).

^{1}H NMR (400 MHz, $CDCl_3$) δ 7.11 (d, J = 1.6 Hz, 4H), 2.57 (t, J = 7.0 Hz, 2H), 2.47 (td, J = 9.0, 5.1 Hz, 1H), 1.94 – 1.78 (m, 4H), 1.74 (d, J = 12.2 Hz, 1H), 1.60 (p, J = 7.6 Hz, 2H), 1.45 – 1.20 (m, 11H), 0.88 (t, J = 6.9 Hz, 3H).

^{13}C NMR (101 MHz, $CDCl_3$) δ 145.3, 140.3, 128.3, 126.7, 44.2, 35.6, 34.6, 31.8, 31.6, 29.2, 27.0, 26.2, 22.7, 14.2.

HRMS (+p APCI) calcd. for [$C_{18}H_{28}$] ([M]$^{+}$) 244.2186 found 244.2180.

5. Synthesis of catalysts

5.1. Synthesis of di-arylated NTTL derivatives

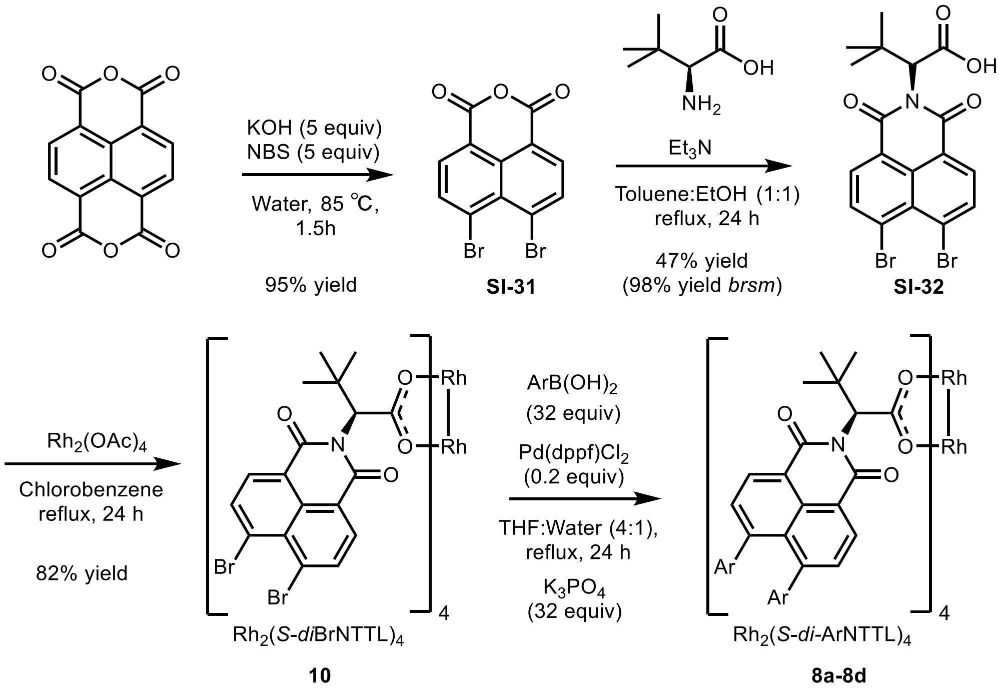

Scheme S1. Overall synthesis of Rh_2(*S-di*-ArNTTL)$_4$ by 8-fold Suzuki cross coupling

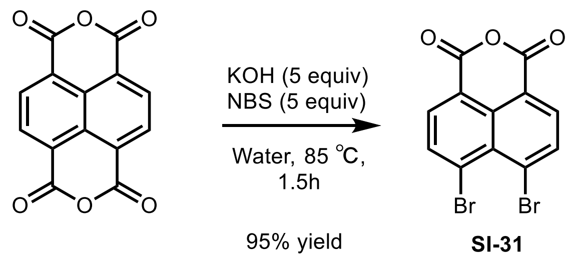

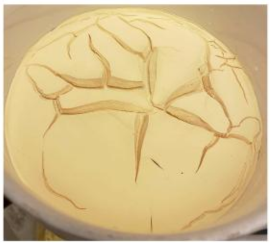

Di-bromination (Hunsdiecker reaction).[19] To a solution of isochromeno[6,5,4-def]isochromene-1,3,6,8-tetraone (10g, 37.3 mmol, 1.0 equiv) in water (373 ml, 0.1 M) at room temperature was added potassium hydroxide (12.3 g, 186 mmol, 5.0 equiv). The vigorously stirring suspension was then heated to 85-90 °C before NBS (33.2 g, 186 mmol, 5.0 equiv) was added in small increments. After the complete addition of NBS, the reaction mixture was stirred at 85-90 °C for about 1.5 hours. The reaction mixture was then allowed to cool down to room temperature before concentrated HCl was slowly added to neutralize any remaining excess base. The precipitate was filtered using a Buchner funnel and washed several times (at least 5x) with water and MeOH to afford the desired dibrominated naphthalic anhydride **SI-31** product in quantitative yield as milky-yellow powder solid (12.6 g, 95%) with very low solubility in aqueous or organic solvents. The milky-yellow powder was further dried overnight in a desiccator before being used in the next step without further purification and characterization due to poor solubility in most common solvents.

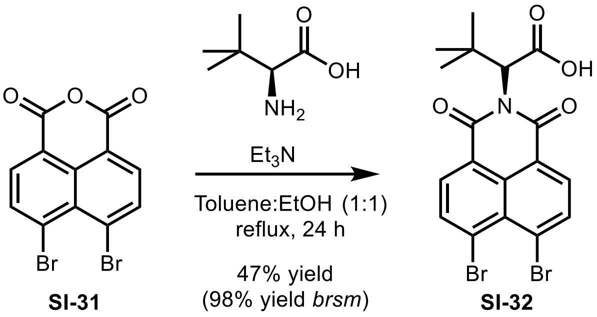

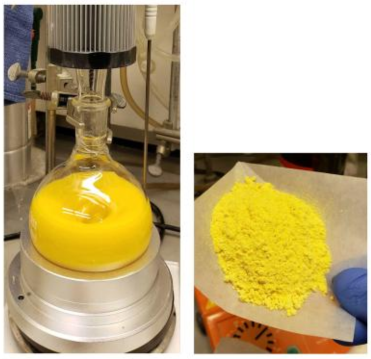

Condensation. To a 100 round-bottom-flask equipped with a magnetic stir-bar was added di-bromo-naphthalic anhydride (3.0 g, 8.4 mmol, 1.0 equiv) in a solution of toluene:ethanol (170 ml, 1:1 v/v, 0.05 M). *S*-tert-leucine (2.21 g, 16.9 mmol, 2.0 equiv) and triethylamine (2.35 ml, 16.9 mmol, 2.0 equiv) were added next. The flask was fitted with a findenser, and the reaction was then set to stir overnight under an inert atmosphere at reflux using a heating-block or an oil bath. After 24 hours, the reaction mixture was allowed to cool down to room temperature. The bright yellow solution was then transferred into a 250 ml separatory funnel and diluted with ethyl acetate (100 ml). It was then washed with 2 M HCl solution (20 ml or until the aqueous layer was clear) then brine (2x50 ml). The solvent was removed by reduced pressure and the crude material was purified by flash chromatography (SiO_2, 0-5% MeOH in DCM). The corresponding carboxylic ligand **SI-32** was obtained as a bright-yellow or light-yellow fluffy solid (1.86 g, 47% yield). The unreactive anhydride is recovered from the aqueous phase of the work up. *Note that the reaction is complete when it appears yellow as indicated in the images below. An undesired and unidentified red product is produced if the reaction is allowed to continue refluxing beyond 24 hours.*

$\mathbf{R_f}$ (5% MeOH in DCM) = 0.4 (UV 254 nm)

^{1}H NMR (600 MHz, CDCl3) δ 8.48 – 8.32 (m, 2H), 8.21 (d, *J* = 7.9 Hz, 2H), 5.54 (s, 1H), 1.17 (s, 9H).

^{13}C NMR (151 MHz, CDCl3) δ 174.0, 163.6, 163.1, 136.4, 132.5, 132.1, 131.3, 128.8, 128.7, 127.9, 122.9, 122.7, 60.1, 36.1, 28.5.

HRMS (+p APCI) calcd. for [$C_{18}H_{16}O_4N^{79}Br_2$] ([M+H]$^+$) 467.9441 found 467.9445.

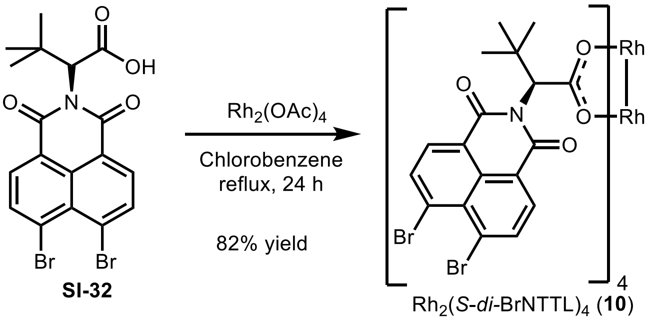

Ligand Exchange. To a 100 round-bottom-flask equipped with a magnetic stir-bar was added dirhodium tetraacetate (1 equiv) and the carboxylic acid ligand obtained from the previous step (8.0 equiv). Chlorobenzene (50 ml) was added, and the flask was fitted with a Soxhlet packed with anhydrous potassium carbonate. The Soxhlet was also fitted with a condenser and the reaction mixture was set to stir overnight at reflux (150-170 oC) under an inert atmosphere. After complete consumption of rhodium tetraacetate as indicated by TLC (1:1 DCM/hexanes), the reaction mixture was allowed to cool down to room temperature before removing the solvent by rotatory evaporation or under vacuum. The

Crude green material was then dry-loaded and purified by flash chromatography (SiO_2, 0%-15% ethyl acetate in hexane). The green fractions were then collected as desired Rh_2(S-*di*-BrNTTL)$_4$ (**10**) in 82% yield.

R$_f$ (4H/1EA) = 0.7

^{1}H NMR (800 MHz, $CDCl_3$) δ 8.53 (d, J = 7.9 Hz, 1H), 8.32 (t, J = 8.7 Hz, 2H), 8.13 (d, J = 7.9 Hz, 1H), 5.69 (s, 1H), 1.23 (s, 9H).

^{13}C NMR (201 MHz, $CDCl_3$) δ 187.2, 164.0, 162.4, 136.9, 136.0, 132.7, 131.6, 131.3, 128.0, 127.6, 123.3, 62.4, 36.3, 28.8.

HRMS (+p ESI) calcd. for [$C_{72}H_{56}O_{16}N_4Br_8Rh_2$] ([M+H]$^+$) 2069.5263 found 2069.5310. (Δ = 2.7 ppm)

General Procedure B. Eight (08)-fold Suzuki-Miyaura cross-coupling of Rh$_2$(S-di-BrNTTL)$_4$ (10). A 100 mL round-bottom flask was charged with Rh_2(*S*-DBNTTL)$_4$ (**2b**) (1.0 equiv), arylboronic acid (32.0 equiv), potassium phosphate (32 equiv) and THF:H_2O (1:1 v/v, 0.05M) was added. The flask was then degassed with nitrogen (or argon) for at least 5 minutes Pd(dppf)Cl_2 (0.200 equiv) was added. The resulting dark red solution was then heated to reflux overnight (24 hours). After the allotted time had passed and **2b** had disappeared on TLC, the solution was cooled down to room temperature and concentrated under reduced pressure. The residue was redissolved in DCM and washed with water (3x), brine (3x), dried over sodium sulfate and concentrated by rotary evaporation to afford the crude dark green product. The crude product was redissolved in either and transferred to a silica gel plug. All green residue was completely flushed down the silica plug with ether and collect. The green solution was concentrated and further purified by flash chromatography (SiO_2, ethyl acetate in hexane) to afford the eight-fold cross-coupled di-arylated-NTTL dirhodium catalyst products (**4a-4d**) (yields ranging from 39-87%).

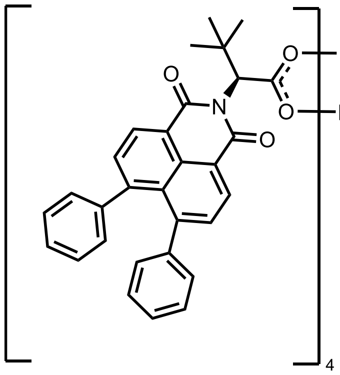

Rh_2(*S-di*-C_6H_5NTTL)$_4$ (8a)

Synthesized according to **General procedure B** using Rh_2(*S-di*-BrNTTL)$_4$ (**10**) (200.0 mg, 0.096 mmol, 1 equiv), phenylboronic acid (375.5 mg, 3.08 mmol, 32 equiv). The crude mixture was purified by flash chromatography (SiO_2, 0-20% ethyl acetate in hexane) to afford Rh_2(*S-di*-C_6H_5NTTL)$_4$ (**8a**) as green solid (110.8 mg, 56% yield).

R$_f$ (5H/1EA) = 0.40 (CAM)

^{1}H NMR (600 MHz, CDCl3) δ 8.92 (d, J = 7.5 Hz, 1H), 8.55 (d, J = 7.6 Hz, 1H), 7.84 (d, J = 7.6 Hz, 1H), 7.50 (d, J = 7.6 Hz, 1H), 7.07 – 6.86 (m, 10H), 5.92 (s, 1H), 1.35 (s, 9H).

^{13}C NMR (151 MHz, CDCl3) δ 187.55, 165.01, 163.28, 147.02, 146.76, 141.89, 141.80, 132.34, 131.80, 131.27, 130.63, 130.39, 129.90, 127.74, 127.57, 126.84, 126.80, 122.62, 122.43, 62.19, 36.48, 29.03.

HRMS (+p ESI) calcd. for [$C_{120}H_{97}O_{16}N_4Rh_2$] ($[M+H]^+$) 2055.5004 found 2055.5043. (Δ = 1.9 ppm)

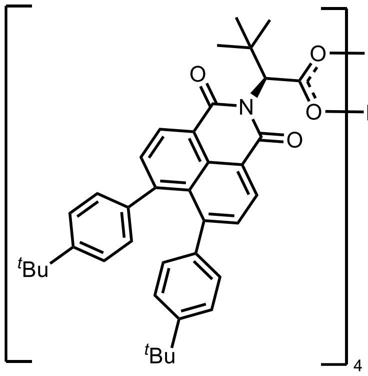

Rh2(*S*-*di*-4-^{t}BuC6H4NTTL)4 (8b)

Synthesized according to **general procedure B** using Rh_2(*S*-*di*-BrNTTL)$_4$ (**10**) (288.0 mg, 0.139 mmol, 1 equiv), (4-(tert-butyl)phenyl)boronic acid (790 mg, 4.43 mmol, 32 equiv). The crude mixture was purified by flash chromatography (SiO_2, 0-10% ethyl acetate in hexane) to afford Rh_2(*S*-*di*-4-^{t}BuC_6H_4NTTL)$_4$ **(8b)** as green solid (262 mg, 85% yield).

R$_f$ (7H/1EA) = 0.60 (CAM)

^{1}H NMR (400 MHz, CDCl$_3$) δ 8.88 (d, *J* = 7.5 Hz, 1H), 8.54 (d, *J* = 7.6 Hz, 1H), 7.82 (d, *J* = 7.6 Hz, 1H), 7.49 (d, *J* = 7.6 Hz, 1H), 6.96 (q, *J* = 7.6 Hz, 8H), 5.89 (s, 1H), 1.34 (s, 9H), 1.19 (s, 9H), 1.17 (s, 9H).

^{13}C NMR (101 MHz, CDCl$_3$) δ 187.5, 165.1, 163.3, 149.5, 149.4, 147.3, 147.0, 139.2, 139.2, 132.3, 131.7, 131.2, 130.6, 130.5, 129.6, 129.4, 127.9, 124.3, 124.3, 122.2, 122.0, 62.1, 36.5, 34.4, 31.4, 31.4, 29.1.

HRMS (+p ESI) calcd. for [$C_{152}H_{160}O_{16}N_4Rh_2$] ($[M+H]^+$) 2502.9934 found 2503.0032. (Δ = 3.9 ppm)

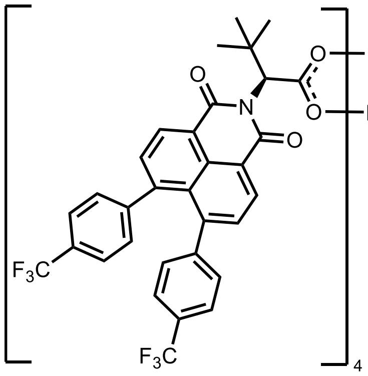

Rh2(*S*-*di*-4-CF3C6H4NTTL)4 (**8c**)

Synthesized according to **general procedure B** using Rh_2(*S*-*di*-BrNTTL)$_4$ (**10**) (200.0 mg, 0.096 mmol, 1 equiv), (4-(trifluoromethyl)phenyl)boronic acid (584.9 mg, 3.08 mmol, 32 equiv). The crude mixture was purified by flash chromatography (SiO_2, 0-10% ethyl acetate in hexane) to afford Rh_2(*S*-*di*-4-$CF_3C_6H_4$NTTL)$_4$ (**8c**) as green solid (205 mg, 82% yield).

R$_f$ (7H/1EA) = 0.55 (CAM)

^{1}H NMR (400 MHz, CDCl$_3$) δ 8.96 (d, *J* = 7.6 Hz, 1H), 8.57 (d, *J* = 7.6 Hz, 1H), 7.86 (d, *J* = 7.5 Hz, 1H), 7.53 (d, *J* = 7.6 Hz, 1H), 7.30 (d, *J* = 8.2 Hz, 1H), 7.19 (dt, *J* = 14.7, 6.5 Hz, 4H), 7.05 – 6.92 (m, 3H), 5.90 (s, 1H), 1.34 (s, 9H).

^{13}C NMR (101 MHz, CDCl$_3$) δ 187.48, 164.65, 162.93, 145.05, 144.89, 144.62, 132.38, 131.88, 131.36, 130.76, 130.26, 130.18, 129.82, 127.69, 124.71, 123.43, 123.27, 77.36, 62.23, 36.55, 28.94. (Due to the lack of resolution and complexity, C-F coupling was not analyzed)

^{19}F NMR (376 MHz, CDCl$_3$) δ -63.13, -63.20.

HRMS (+p ESI) calcd. for [$C_{128}H_{88}O_{16}N_4F_{24}Rh_2$] ([M+H]$^+$) 2598.3917 found 2598.3982. (Δ = 2.5 ppm)

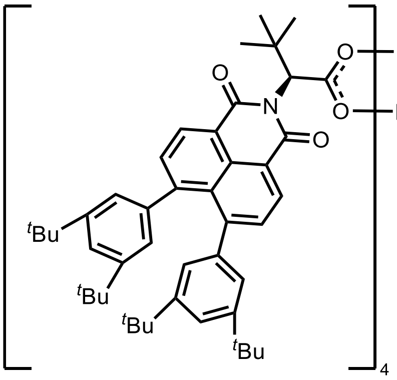

Rh$_2$(*S*-*di*-3,5-*di*-^{t}BuC$_6$H$_3$NTTL)$_4$ (8d)

Synthesized according to **general procedure B** using Rh$_2$(*S*-*di*-BrNTTL)$_4$ (**10**) (1.00 g, 0.481 mmol, 1 equiv), (3,5-di-tert-butylphenyl)boronic acid (3.61 g, 15.4 mmol, 32 equiv). The crude mixture was purified by flash chromatography (SiO_2, 0-8% ethyl acetate in hexane) to afford Rh$_2$(*S*-*di*-3,5-*di*-^{t}BuC$_6$H$_3$NTTL)$_4$ (**8d**) as green solid (963.8 mg, 68% yield).

R$_f$ (9H/1EA) = 0.80 (CAM)

^{1}H NMR (800 MHz, CDCl$_3$) δ 8.86 (d, *J* = 7.5 Hz, 1H), 8.50 (d, *J* = 7.5 Hz, 1H), 7.84 (d, *J* = 7.5 Hz, 1H), 7.43 (d, *J* = 7.5 Hz, 1H), 7.06 (s, 2H), 7.01 (s, 1H), 6.94 (s, 1H), 6.86 (s, 1H), 6.81 (s, 1H), 5.86 (s, 1H), 1.35 (s, 9H), 1.20 (s, 9H), 1.17 (s, 9H), 1.08 (s, 9H), 1.07 (s, 9H).

^{13}C NMR (201 MHz, CDCl$_3$) δ 187.2, 165.3, 163.4, 149.7, 149.6, 149.5, 148.3, 147.8, 141.9, 141.7, 133.7, 132.2, 132.1, 130.9, 130.2, 127.4, 123.6, 123.5, 123.4, 123.3, 122.2, 122.1, 122.0, 122.0, 62.6, 36.3, 34.8, 34.8, 34.7, 34.7, 31.6, 31.6, 31.5, 31.5, 29.1, 25.9.

HRMS (+p ESI) calcd. for [$C_{184}H_{224}O_{16}N_4Rh_2$] ([M+H]$^+$) 2951.4942 found 2951.5131.

5.2. Synthesis of tetra-arylated NTTL derivatives

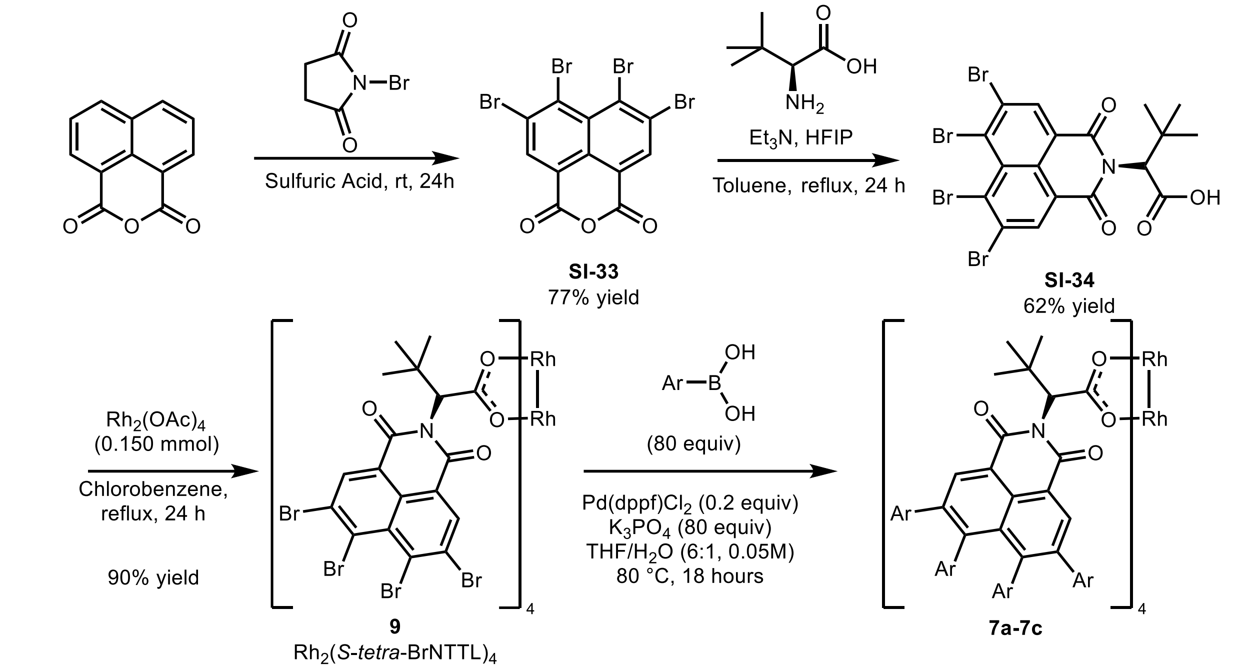

Scheme S2. Overall synthesis of Rh_2(*S-tetra*-ArNTTL)$_4$ by 16-fold Suzuki cross coupling

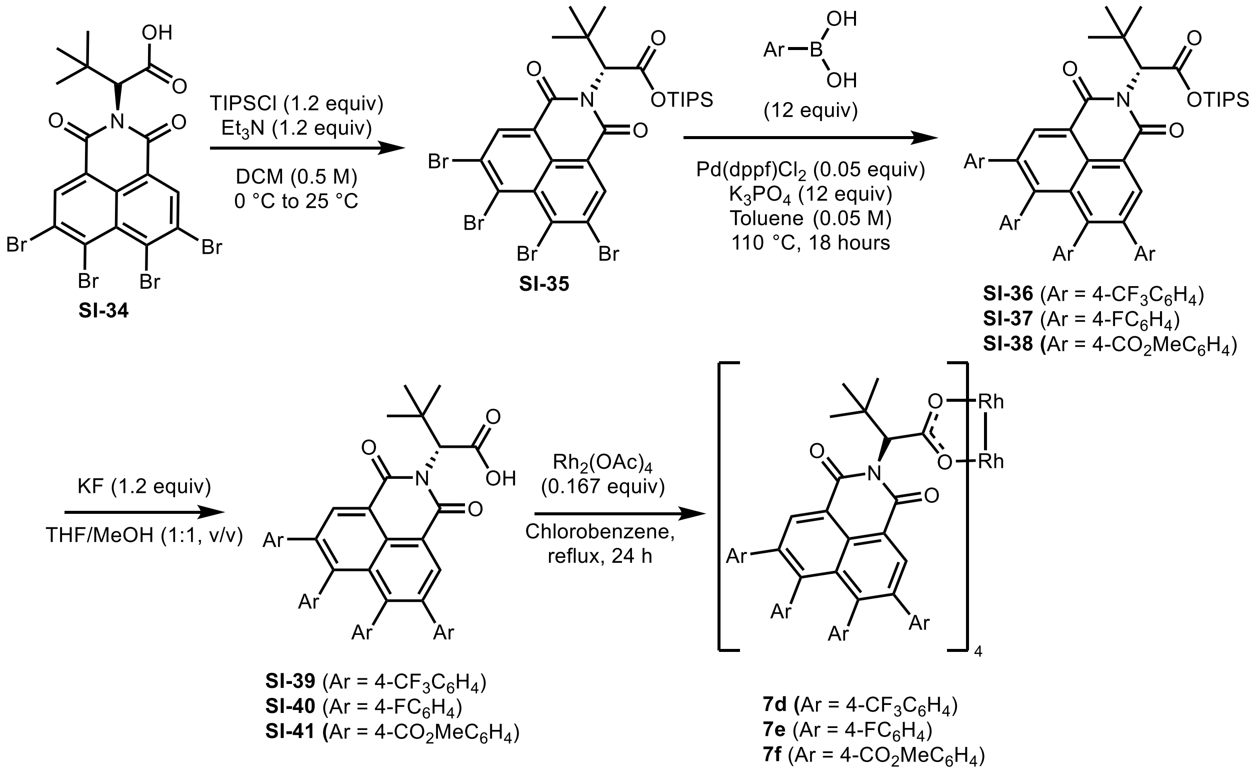

Scheme S3. Modified synthesis of Rh_2(*S-tetra*-ArNTTL)$_4$ derivatives via ligand exchange

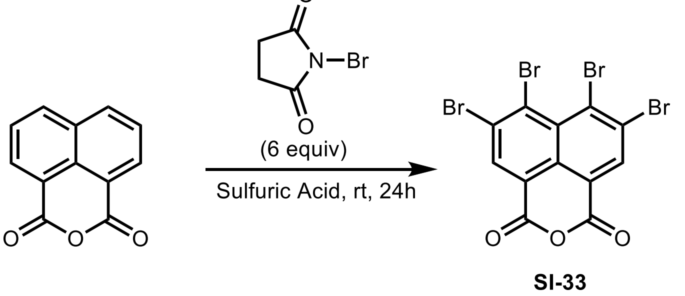

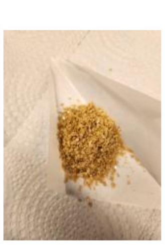

Tetra-bromination. To a solution of naphthalic anhydride (7.0 g, 35.3 mmol, 1.0 equiv) in concentrated sulfuric acid (250 mL, 95-97%, 0.1 M) at room temperature was added NBS (37.7 g, 212 mmol, 6.0 equiv). The reaction mixture turned from a clear solution to cloudy and thick solution after few hours stirring at room temperature. After 24 hours, the mixture was poured into ice. The precipitate was filtered and washed throughout with water and then methanol. The crude product which was then recrystallized from hot chlorobenzene to give the corresponding tetra-bromonaphthalic anhydride **SI-33** (9.65 g, 53 %) as a light-brown solid.

^{1}H NMR (400 MHz, DMSO) δ 8.70 (s, 2H). (spectroscopic data matching that reported in the literature)

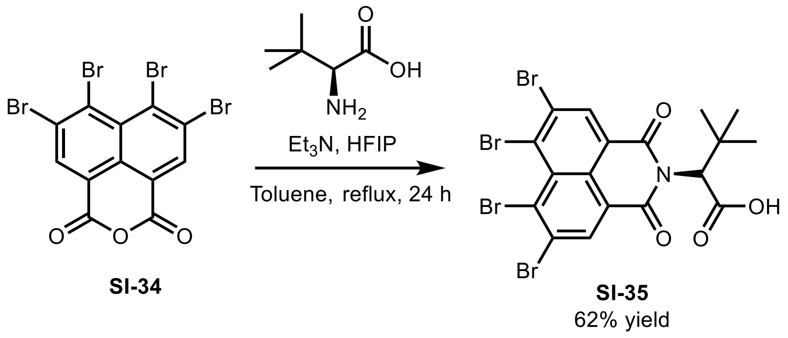

Condensation. To a 250 round-bottom-flask equipped with a magnetic stir-bar was added 5,6,7,8-tetrabromonaphthalic anhydride (12.7 g, 24.7 mmol, 1.05 equiv), (S)-tert-leucine (3.08 g, 23.5 mmol, 1.0 equiv) and triethylamine (3.28 ml, 23.5 mmol, 1.00 equiv) in toluene (235 mL, 0.1 M). 1,1,1,3,3,3-hexafluoropropan-2-ol (4.93 mL, 2 equiv, 47.0 mmol) was then added to facilitate the homogeneity of the reaction mixture. The flask was then fitted with a findenser, and the mixture was allowed to stir at reflux under a nitrogen atmosphere for around 16 hours. Unidentified red by-products were observed if the reaction was let stir longer. The reaction mixture was allowed to cool down to room temperature and a TLC (5% MeOH:DCM) revealed complete consumption of the amino acid. The red solution was then transferred into a 250 ml separatory funnel and diluted with ethyl acetate (100 ml). It was then washed with 2 M HCl solution (20 ml or until the aqueous layer was clear) then brine (2x50 ml). The solvent was removed by reduced pressure. The crude material was purified by flash chromatography (SiO_2, 0-5% MeOH in DCM) to afford the corresponding carboxylic ligand a brownish yellow solid (S)-3,3-dimethyl-2-(5,6,7,8-tetrabromo-1,3-dioxo-1H benzo[de]isoquinolin-2(3H)-yl) butanoic acid (9.32 g, 63% yield).

R_f (5% MeOH in DCM) = 0.4 (UV 254 nm)

^{1}H NMR (400 MHz, DMSO) δ 12.65 (s, 1H), 8.69 (s, 1H), 8.66 (s, 1H), 5.24 (s, 1H), 1.10 (s, 9H).

^{13}C NMR (101 MHz, DMSO) δ 168.7, 162.2, 161.4, 135.3, 134.8, 132.1, 130.7, 129.1, 129.0, 127.7, 122.8, 122.5, 59.8, 35.6, 28.2. *(Missing 1 carbon)*

HRMS (+p ESI) calcd. for [$C_{18}H_{14}O_4N^{79}Br_4$] ([M+H]$^+$) 623.7651 found 623.7653.

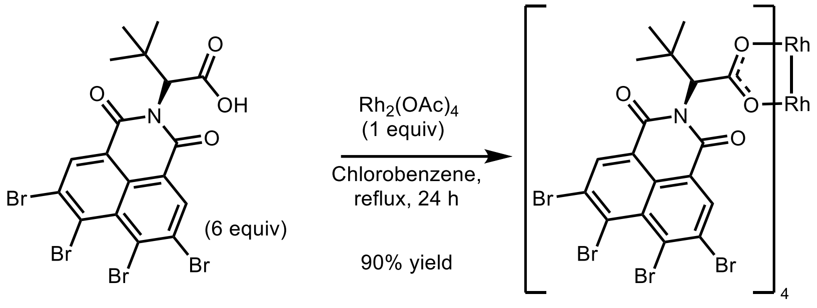

Rh$_2$(*S-tetra*-BrNTTL)$_4$ (**9**)

Ligand Exchange. To a 100 round-bottom-flask equipped with a magnetic stir-bar was added dirhodium tetraacetate (663 mg, 1.5 mmol, 1.0 equiv) and the carboxylic acid ligand obtained from the previous step (5.64 g, 9.0 mmol, 6 equiv). Chlorobenzene (40 ml) was added, and the flask was fitted with a Soxhlet packed with anhydrous potassium carbonate. The Soxhlet was also fitted with an inverted condenser and the reaction mixture was set to stir overnight at reflux. Upon completion, PhCl was removed under reduced pressure. The crude mixture was dissolved in dichloromethane and passed through neutral alumina plug to remove excess of the ligand using dichloromethane as eluent. The combined organic solution was removed under vacuum to afford Rh$_2$(*S-tetra*-BrNTTL)$_4$ (**9**) as green solid (2.70 g, 66% yield). If the product obtained from neutral alumina plug is still contaminated excess ligand, further purification by flash chromatography (SiO_2, 30-100% DCM in hexane, then 0-5% MeOH in DCM).

R$_f$ (100% DCM) = 0.7

^{1}H NMR (800 MHz, CDCl$_3$) δ 8.85 (s, 2H), 5.76 (s, 1H), 1.21 (s, 9H).

^{13}C NMR (201 MHz, CDCl$_3$) δ 187.2, 162.5, 161.1, 136.4, 136.3, 132.4, 131.8, 131.1, 129.4, 129.3, 127.9, 123.1, 122.8, 62.2, 36.2, 28.8.

HRMS (+p ESI) calcd. for [$C_{74}H_{51}O_{16}N_5{}^{79}Br_8{}^{81}Br_8{}^{103}Rh_2$] ([M+$CH_3CN$]$^+$) 2749.8206 found 2749.8339. (Δ = 4.8 ppm)

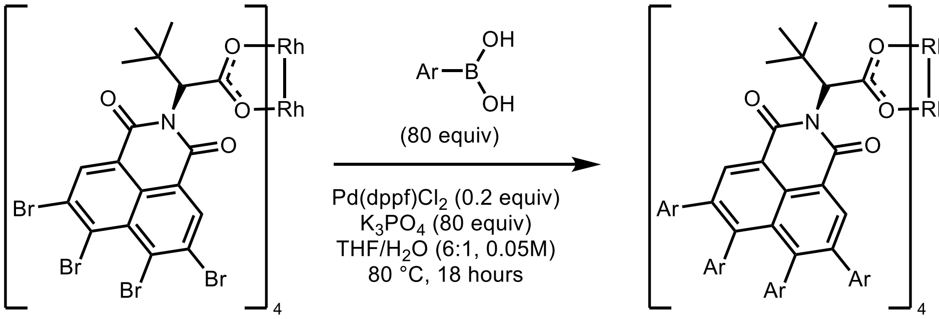

2a
Rh$_2$(*S*-TBNTTL)$_4$
3a-3c

General Procedure C. Sixteen (16) fold Suzuki-Miyaura cross-coupling. A 25 mL round-bottom flask was charged with Rh$_2$(*S-tetra*-BrNTTL)$_4$ (**9**) (1 equiv), aryl boronic acid (80.0 equiv), anhydrous K_3PO_4 (80.0 equiv) and degassed mixture of THF/H_2O (6:1 v/v, 0.05M) was added. The flask was then degassed by bubbling nitrogen/argon through it for at least 5 minutes. Pd(dppf)Cl2 (0.200 equiv) was added. The resulting red solution was then heated to reflux overnight (at least 18 hours). After the allotted time had passed and Rh$_2$(*S-tetra*-BrNTTL)$_4$ (**9**) had disappeared on TLC, the solution was cooled down to room temperature. The crude mixture was passed through silica plug using Et_2O as an eluent. The mixture was combined, and solvent was removed under reduced pressure. The green crude mixture was dry loaded and purified by flash chromatography (SiO_2, 0-30% ethyl acetate in hexane) to afford the (**3a-3c**) product as green solid.

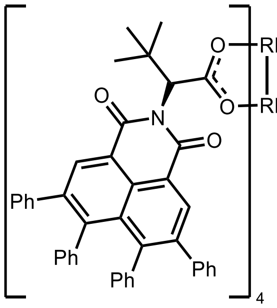

Rh_2(*S-tetra*-C_6H_5NTTL)$_4$ (7a)

Synthesized according to **general procedure C** using Rh_2(*S-tetra*-BrNTTL)$_4$ (**9**) (600 mg, 0.225 mmol, 1 equiv), phenyl boronic acid (2.16 g, 17.72 mmol, 80 equiv). The crude mixture was purified by flash chromatography (SiO_2, 0-15% ethyl acetate in hexane) to afford **Rh_2(*S-tetra*-C_6H_5NTTL)$_4$ (7a)** as green solid (454.4 mg, 77% yield).

R_f (3H/1EA) = 0.5 (UV 254 nm)

^{1}H NMR (400 MHz, CDCl3) δ 8.79 (s, 1H), 8.35 (s, 1H), 7.19 – 7.04 (m, 5H), 6.93 – 6.84 (m, 5H), 6.72 – 6.44 (m, 10H), 5.80 (s, 1H), 1.23 (s, 9H).

^{13}C NMR (151 MHz, CDCl3) δ 187.62, 164.19, 163.26, 145.20, 144.78, 142.89, 142.40, 141.15, 139.70, 139.60, 134.09, 133.15, 131.91, 131.64, 130.53, 130.41, 129.84, 129.73, 128.19, 127.58, 127.39, 127.27, 126.86, 126.60, 126.40, 126.22, 126.00, 125.86, 122.47, 121.94, 115.42, 61.36, 36.59, 28.90.

HRMS (+p ESI) calcd. for [$C_{168}H_{129}O_{16}N_4Rh_2$] ([M+H]$^+$) 2663.7508 found 2663.7627. (Δ = 4.5 ppm)

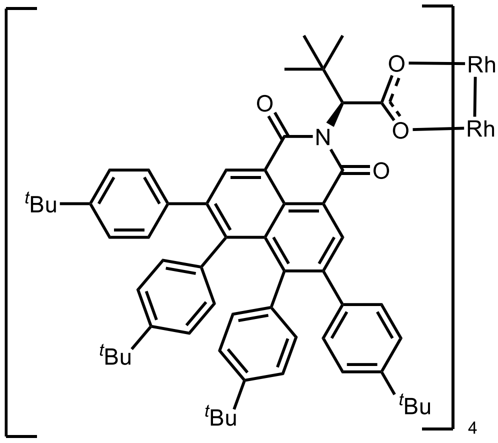

Rh_2(*S-tetra*-4-^{t}BuC_6H_4NTTL)$_4$ (7b)

Synthesized according to **general procedure C** using Rh_2(*S-tetra*-BrNTTL)$_4$ (**9**) (200 mg, 0.078 mmol, 1 equiv), (4-(tert-butyl)phenyl)boronic acid (1.05 g, 5.91 mmol, 80 equiv). The crude mixture was purified by flash chromatography (SiO_2, 0-8% ethyl acetate in hexane) to to afford Rh_2(*S-tetra*-4-^{t}BuC_6H_4NTTL)$_4$ (**7b**) as green solid (110.9 mg, 72% yield).

R_f (9H/1EA) = 0.3 (UV 254 nm)

^{1}H NMR (600 MHz, $CDCl_3$) δ 8.78 (s, 1H), 8.52 (s, 1H), 7.12 (d, *J* = 8.2 Hz, 2H), 7.02 (d, *J* = 7.0 Hz, 2H), 6.98 (d, *J* = 7.4 Hz, 1H), 6.78 (d, *J* = 8.2 Hz, 2H), 6.75 (dd, *J* = 8.3, 1.6 Hz, 2H), 6.67 – 6.62 (m, 2H), 6.54 (dd, *J* = 8.3, 2.1 Hz,

1H), 6.49 (dt, J = 8.2, 1.8 Hz, 1H), 6.44 (ddt, J = 14.9, 8.1, 1.8 Hz, 2H), 6.34 (dt, J = 8.2, 1.8 Hz, 1H), 5.79 (d, J = 1.1 Hz, 1H), 1.23 (s, 9H), 1.20 (s, 9H), 1.12 (s, 9H), 1.10 (s, 9H), 1.05 (s, 9H).

^{13}C NMR (151 MHz, CDCl$_3$) δ 187.6, 164.3, 163.4, 148.9, 148.7, 148.6, 148.32, 148.30, 148.0, 145.9, 145.0, 142.7, 142.4, 138.6, 138.3, 137.3, 137.2, 134.2, 132.8, 131.6, 131.5, 131.3, 131.2, 131.0, 130.1, 130.0, 129.6, 128.1, 124.0, 123.9, 123.3, 123.1, 123.0, 122.7, 122.2, 121.7, 61.3, 36.7, 34.4, 34.3, 34.2, 34.1, 31.47, 31.45, 31.43, 31.40, 31.37, 31.34, 31.32, 31.27, 28.88, 28.86, 28.84.

HRMS (+p ESI) calcd. for [$C_{232}H_{256}O_{16}N_4Rh_2$] ([M+H]$^+$) 3559.7446 found 3559.7846. (Δ = 11.2 ppm)

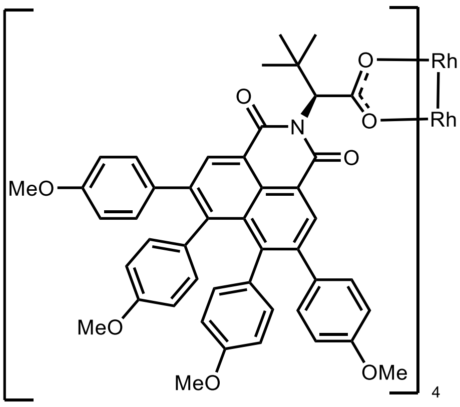

Rh$_2$(*S*-*tetra*-4-MeO-C$_6$H$_4$NTTL)$_4$ (7c)

Synthesized according to **general procedure C** using Rh$_2$(*S*-*tetra*-BrNTTL)$_4$ (**9**) (800 mg, 0.3 mmol, 1 equiv), (4-methoxyphenyl) boronic acid (3.589 g, 23.6 mmol, 80 equiv). The crude mixture was purified by flash chromatography (SiO$_2$, 0-55% ethyl acetate in hexane) to afford **Rh$_2$(*S*-*tetra*-4-MeO-C$_6$H$_4$NTTL)$_4$ (7c)** as green solid (655.8 mg, 72% yield).

R$_f$ (4H/3EA) = 0.2

^{1}H NMR (800 MHz, CDCl$_3$) δ 8.75 (s, 1H), 8.41 (s, 1H), 7.18 (d, J = 8.1 Hz, 2H), 6.79 (d, J = 6.6 Hz, 2H), 6.62 (d, J = 6.8 Hz, 2H), 6.57 (d, J = 7.6 Hz, 2H), 6.54 (d, J = 8.4 Hz, 1H), 6.42 (d, J = 8.5 Hz, 1H), 6.39 (d, J = 8.5 Hz, 1H), 6.37 (d, J = 8.8 Hz, 1H), 6.32 (d, J = 8.5 Hz, 1H), 6.23 (d, J = 8.5 Hz, 1H), 6.19 – 6.14 (m, 2H), 5.78 (d, J = 2.2 Hz, 1H), 3.72 (s, 3H), 3.64 – 3.62 (m, 6H), 3.60 (s, 3H), 1.24 (s, 9H).

^{13}C NMR (201 MHz, CDCl$_3$) δ 187.6, 164.3, 163.3, 158.0, 157.6, 157.4, 145.0, 144.3, 142.5, 142.1, 134.3, 134.1, 133.9, 133.3, 132.9, 132.82, 132.79, 132.7, 132.6, 131.7, 131.5, 131.0, 128.0, 122.3, 121.8, 113.00, 112.96, 112.7, 112.6, 112.5, 112.4, 61.3, 55.24, 55.22, 55.20, 55.18, 36.6, 28.9.

HRMS (+p ESI) calcd. For [$C_{184}H_{161}O_{32}N_4{}^{103}Rh_2$] ([M+H]$^+$) 3143.9199 found 3143.9474. (Δ = 8.8 ppm)

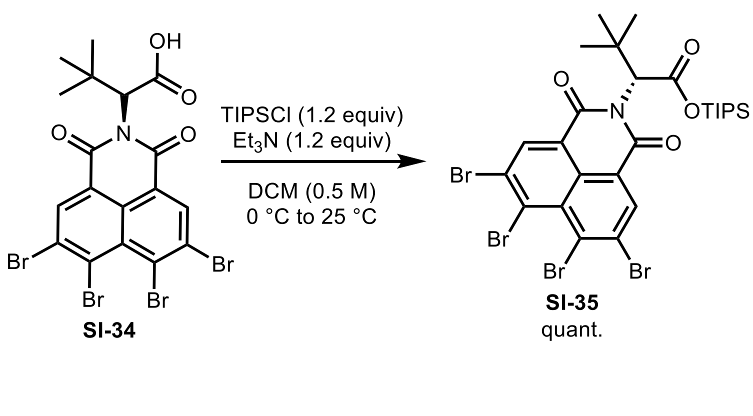

SI-34 **SI-35** quant.

TIPS protection. To a solution of **SI-34** ligand (1.00 g, 1.6 mmol, 1.0 equiv) in CH_2Cl_2 (17 ml, 0.1M) in a 20 ml vial was added TIPSCl (408 μL, 1.91 mmol, 1.2 equiv). A yellowish cloudy solution was stirred at room temperature. Then, Et_3N (267 μL, 1.91 mmol, 1.2 equiv) was added in one-portion at room temperature to the above suspension. The mixture was turned clear and let stir for 3 hours. Upon completion, the reaction was quenched with water and extracted with ethyl acetate. The organic phase was dried over sodium sulfate and concentrated under reduced pressure. The crude mixture was purified by flash chromatography (SiO_2, 0-10% diethyl ether in hexane) to afford **SI-35** as yellow solid (1.21 g, 97% yield).

R_f (9H/1Et_2O) = 0.5 (UV 254 nm)

^{1}H NMR (600 MHz, $CDCl_3$) δ 8.84 (s, 1H), 8.78 (s, 1H), 5.45 (s, 1H), 1.23 (p, J = 7.8 Hz, 3H), 1.19 (d, J = 1.8 Hz, 9H), 0.98 (d, J = 7.5 Hz, 18H).

^{13}C NMR (151 MHz, $CDCl_3$) δ 167.3, 162.6, 161.7, 136.4, 135.8, 132.7, 131.5, 131.5, 130.0, 129.9, 127.7, 122.9, 122.4, 61.9, 36.0, 28.7, 17.9, 17.8, 12.0.

HRMS (+p ESI) calcd. for [$C_{27}H_{34}O_4N^{79}Br_4{}^{28}Si$] ([M+H]$^+$) 779.8985 found 779.8993.

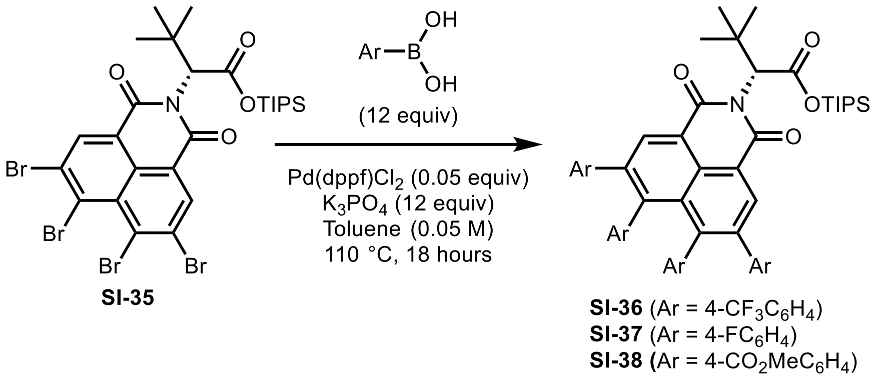

General Procedure D. Four (4) fold Suzuki-Miyaura cross-coupling. A round-bottom flask was charged with **SI-35** (1 equiv), aryl boronic acid (12.0 equiv), anhydrous K_3PO_4 (12.0 equiv) and degassed toluene (0.05M) was added. The flask was then degassed by bubbling nitrogen/argon through it for at least 5 minutes. Pd(dppf)Cl_2 (5 mol%) was added. The resulting red solution was then heated to reflux overnight (at least 24 hours). After that, the solution was cooled down to room temperature. The crude mixture was passed through silica plug using Et_2O as an eluent. The mixture was combined, and solvent was removed under reduced pressure. The crude mixture was dry load and purified by flash chromatography (SiO_2, 0-30% ethyl acetate in hexane) to afford **SI-36**, **SI-37**, and **SI-38** as a light-yellow solid.

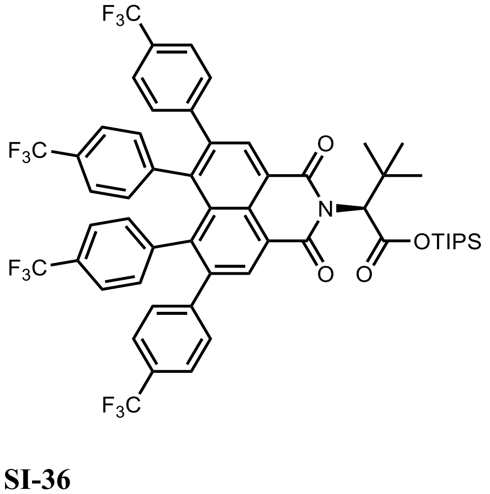

SI-36

This compound was synthesized according to **general procedure D** using (4-(trifluoromethyl)phenyl)boronic acid (2.91 g, 15.3 mmol, 12.0 equiv), and **SI-35** (1.0 g, 1.28 mmol, 1.0 equiv). The crude mixture was dry load and purified by flash chromatography (SiO_2, 0-10% diethyl ether in hexane) to obtain **SI-36** as yellow solid (957.4 mg, 72% yield)

R$_f$ (3H/1Et_2O) = 0.60 (UV 254 nm)

^{1}H NMR (600 MHz, CDCl$_3$) δ 8.68 (s, 1H), 8.63 (s, 1H), 7.40 (dd, *J* = 8.0, 5.8 Hz, 4H), 7.05 (d, *J* = 8.0 Hz, 2H), 7.03 – 6.96 (m, 6H), 6.77 – 6.59 (m, 4H), 5.62 (s, 1H), 1.34 – 1.24 (m, 12H), 1.05 (d, *J* = 7.5 Hz, 18H).

^{13}C NMR (151 MHz, CDCl$_3$) δ 168.1, 164.2, 163.2, 143.6, 143.5, 143.4, 143.3, 142.3, 142.0, 141.9, 133.9, 133.3, 131.73, 131.68, 130.4, 130.0, 129.97, 129.7 (q, *J* = 33.0 Hz), 129.4 (q, *J* = 33.3 Hz), 128.5, 124.95 (q, *J* = 3.9 Hz), 124.2 (m), 123.9 (q, *J* = 272.3 Hz), 123.5 (q, *J* =272.2 Hz), 122.8, 122.4, 121.2, 120.9, 61.8, 36.1, 28.9, 17.9, 17.9, 12.2.

^{19}F NMR (565 MHz, CDCl$_3$) δ -62.74 (s), -62.75 (s), -63.35 (s).

HRMS (+p APCI) calcd. for [$C_{55}H_{50}O_4NF_{12}{}^{28}Si$] ([M+H]$^+$) 1044.3312 found 1044.3319.

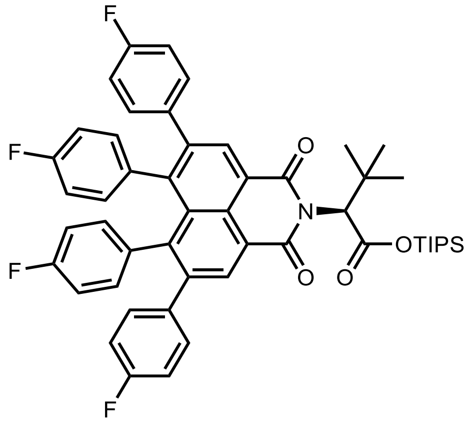

SI-37

This compound was synthesized according to **general procedure D** using (4-fluorophenyl)boronic acid (2.86 g, 20.4 mmol, 16.0 equiv), K_3PO_4 (4.34 g, 20.4 mmol, 16.0 equiv), and **SI-35** (1.0 g, 1.28 mmol, 1.0 equiv). The crude mixture was dry load and purified by flash chromatography (SiO_2, 0-10% diethyl ether in hexane) to obtain **SI-37** as yellow solid (963.2 mg, 80% yield). *Note. The boronic acid and K_3PO_4 loading increased from 12 equiv to 16 equiv.*

R$_f$ (9H/1Et_2O) = 0.15 (UV 254 nm)

^{1}H NMR (800 MHz, CDCl$_3$) δ 8.64 (s, 1H), 8.59 (s, 1H), 6.91 – 6.86 (m, 2H), 6.86 – 6.79 (m, 6H), 6.56 – 6.43 (m, 8H), 5.61 (d, *J* = 1.6 Hz, 1H), 1.32 – 1.26 (m, 12H), 1.03 (d, *J* = 7.6 Hz, 18H).

^{13}C NMR (201 MHz, CDCl$_3$) δ 168.17, 164.61, 163.67, 162.43, 162.42, 161.95, 161.20, 161.19, 160.71, 144.59, 144.46, 142.43, 142.38, 136.33, 136.31, 136.28, 136.26, 135.26, 135.24, 134.07, 133.51, 133.00, 132.95, 132.92, 131.35, 131.31, 131.28, 130.90, 128.23, 122.13, 121.66, 114.94, 114.92, 114.83, 114.81, 114.25, 114.23, 114.15, 114.12, 61.62, 36.07, 28.90, 17.93, 17.90, 12.16. *Due to the complexity and lack of resolution, the C-F coupling was not annotated. The reported signals are the number of peaks observed.*

^{19}F NMR (753 MHz, CDCl$_3$) δ -114.92 (t, *J* = 7.8 Hz), -114.98 (dd, *J* = 12.4, 6.7 Hz).

HRMS (+p APCI) calcd. for [$C_{51}H_{50}O_4NF_4{}^{28}Si$] ([M+H]$^+$) 844.3440 found 844.3444.

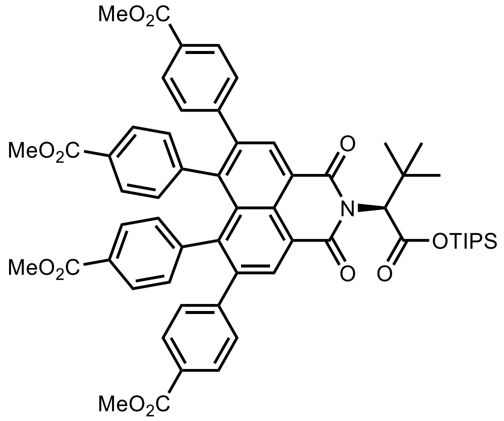

SI-38

This compound was synthesized according to **general procedure D** using (4-(methoxycarbonyl)phenyl)boronic acid (1.84 g, 10.2 mmol, 16.0 equiv), K_3PO_4 (2.17 g, 10.2 mmol, 16.0 equiv), and **SI-35** (0.50 g, 0.64 mmol, 1.0 equiv). The crude mixture was dry load and purified by flash chromatography (SiO_2, 0-20% ethyl acetate in hexane) to obtain **SI-38** as yellow solid (239.8 mg, 37% yield). *Note. The boronic acid and K_3PO_4 loading increased from 12 equiv to 16 equiv.*

$\mathbf{R_f}$ (1H/1EA) = 0.55 (UV 254 nm)

^{1}H NMR (600 MHz, CDCl$_3$) δ 8.68 (s, 1H), 8.63 (s, 1H), 7.77 (dd, *J* = 8.3, 6.1 Hz, 4H), 7.36 – 7.29 (m, 4H), 7.01 (d, *J* = 8.1 Hz, 2H), 6.97 (d, *J* = 8.3 Hz, 2H), 6.71 – 6.57 (m, 4H), 5.61 (s, 1H), 3.89 – 3.84 (m, 6H), 3.81 (s, 6H), 1.33 – 1.25 (m, 12H), 1.07 – 1.00 (m, 18H).

^{13}C NMR (151 MHz, CDCl$_3$) δ 168.04, 166.72, 166.35, 164.36, 163.43, 144.81, 144.76, 144.26, 144.14, 143.30, 142.27, 142.22, 133.82, 133.25, 131.67, 131.62, 131.61, 129.94, 129.77, 129.74, 129.13, 128.94, 128.39, 128.32, 128.30, 122.61, 122.15, 61.71, 52.30, 52.17, 36.09, 28.89, 17.93, 17.90, 12.15.

HRMS (+p APCI) calcd. for [$C_{59}H_{62}O_{12}N^{28}Si$] ([M+H]$^+$) 1004.4036 found 1004.4042.

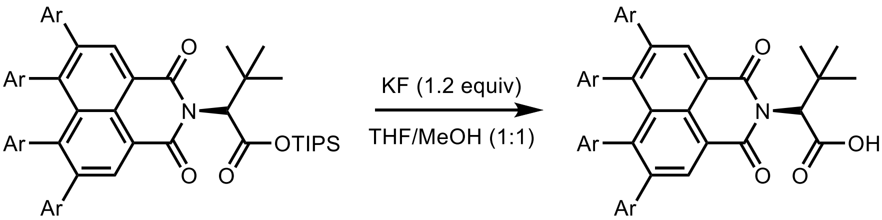

SI-36 (Ar = 4-$CF_3C_6H_4$)
SI-37 (Ar = 4-FC_6H_4)
SI-38 (Ar = 4-$CO_2MeC_6H_4$)

SI-39 (Ar = 4-$CF_3C_6H_4$)
SI-40 (Ar = 4-FC_6H_4)
SI-41 (Ar = 4-$CO_2MeC_6H_4$)

General procedure E. TIPS deprotection. KF (85 mg, 1.47 mmol) was added to a solution of TIPS protected ligand (**SI-36**, **SI-37**, or **SI-38**) in THF (6 mL) and MeOH (6 mL). The mixture was stirred for 1 h at an ambient temperature before it was diluted with ethyl acetate (20 mL) and acidified with 2N HCl (aq). The aqueous phase was extracted with ethyl acetate (3 x 20 mL), the combined organic layers were washed with brine (20 mL), dried over MgSO4, and concentrated under reduced pressure. The crude material was purified by flash chromatography (SiO_2, 0-5% MeOH in DCM) to give the ligand (**SI-39**, **SI-40**, or **SI-41**) as a yellow solid.

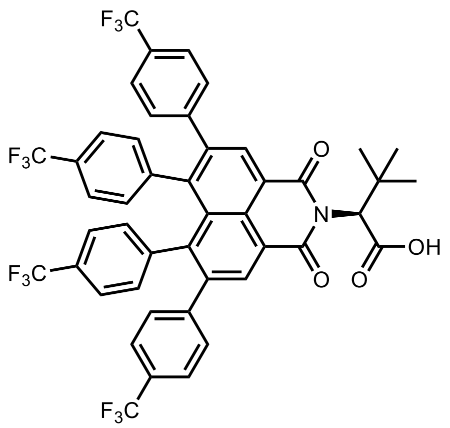

SI-39

This compound was synthesized according to **general procedure E** from **SI-36**. The crude mixture was purified by flash chromatography (SiO_2, 0-5% MeOH in DCM) to obtain **SI-39** as a yellow solid (777.4 mg, 96% yield).

$\mathbf{R_f}$ (5% MeOH/DCM) = 0.3 (UV 254 nm)

^{1}H NMR (600 MHz, $CDCl_3$) δ 11.44 (bs, 1H), 8.68 (d, *J* = 16.2 Hz, 2H), 7.49 – 7.31 (m, 4H), 7.11 – 6.93 (m, 8H), 6.69 (dq, *J* = 17.8, 8.2 Hz, 4H), 5.66 (d, *J* = 1.5 Hz, 1H), 1.26 (s, 9H).

^{13}C NMR (151 MHz, $CDCl_3$) δ 174.2, 164.0, 163.5, 143.9, 143.8, 143.3, 143.2, 142.2, 142.0, 134.1, 133.8, 131.7, 131.6, 130.5, 130.0, 129.8 (q, *J* = 32.6 Hz), 129.4 (q, *J* = 33.0 Hz), 128.6, 125.0, 124.2, 123.9 (q, *J* = 272.1 Hz), 123.5 (q, *J* = 272.3 Hz) 122.4, 122.2, 60.2, 36.2, 28.6.

^{19}F NMR (565 MHz, $CDCl_3$) δ -62.74, -62.77, -63.36.

HRMS (+p APCI) calcd. for [$C_{46}H_{30}O_4NF_{12}$] ([M+H]$^+$) 888.1978 found 888.1985.

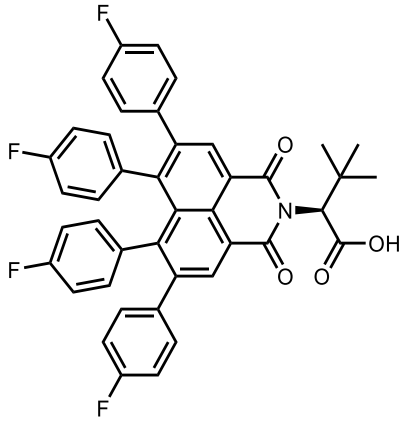

SI-40

This compound was synthesized according to **general procedure E** from **SI-37**. The crude mixture was purified by flash chromatography (SiO_2, 0-5% MeOH in DCM) to obtain **SI-40** as a yellow solid (707 mg, quant. yield).

$\mathbf{R_f}$ (5% MeOH/DCM) = 0.35 (UV 254 nm)

^{1}H NMR (600 MHz, $CDCl_3$) δ 10.90 (bs, 1H), 8.65 (s, 1H), 8.63 (s, 1H), 6.92 – 6.76 (m, 8H), 6.59 – 6.39 (m, 8H), 5.65 (s, 1H), 1.26 (s, 9H).

^{13}C NMR (151 MHz, CDCl$_3$) δ 173.54, 164.40, 163.97, 162.64, 162.16, 161.00, 160.51, 144.86, 144.76, 142.47, 136.21, 135.20, 135.18, 134.28, 133.97, 132.99, 132.93, 132.88, 131.31, 131.26, 131.01, 128.31, 121.77, 121.56, 114.97, 114.83, 114.29, 114.15, 60.01, 36.23, 28.63. *Due to the complexity and lack of resolution, the C-F coupling was not annotated. The reported signals are the number of peaks observed.*

^{19}F NMR (565 MHz, CDCl$_3$) δ -114.82 (p, *J* = 6.9 Hz), -114.91 (dt, *J* = 15.3, 7.7 Hz).

HRMS (+p APCI) calcd. for [$C_{42}H_{30}O_4NF_4$] ([M+H]$^+$) 688.2106 found 688.2103.

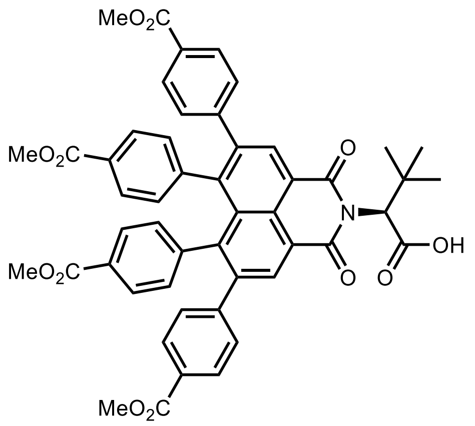

SI-41

This compound was synthesized according to **general procedure E** from **SI-38**. The crude mixture was purified by flash chromatography (SiO$_2$, 0-5% MeOH in DCM) to obtain **SI-41** as a yellow solid (164.0 mg, 85% yield).

R$_f$ (5% MeOH/DCM) = 0.25 (UV 254 nm)

^{1}H NMR (600 MHz, CDCl$_3$) δ ^{1}H NMR (600 MHz, CDCl$_3$) δ 10.94 (s, 1H), 8.68 (s, 1H), 8.66 (s, 1H), 7.75 (t, *J* = 8.7 Hz, 4H), 7.38 – 7.28 (m, 4H), 6.98 (d, *J* = 8.0 Hz, 4H), 6.70 – 6.56 (m, 4H), 5.65 (s, 1H), 3.85 (s, 6H), 3.80 (s, 6H), 1.25 (s, 9H).

^{13}C NMR (151 MHz, CDCl$_3$) δ 173.94, 166.69, 166.33, 164.16, 163.66, 144.70, 144.65, 144.46, 144.39, 143.24, 142.25, 133.96, 133.62, 131.61, 131.54, 130.04, 129.69, 129.11, 128.91, 128.46, 128.36, 128.30, 122.27, 122.03, 60.18, 52.27, 52.16, 36.17, 28.63.

HRMS (+p APCI) calcd. for [$C_{50}H_{42}O_{12}N$] ([M+H]$^+$) 848.2702 found 848.2700.

General procedure F. Ligand exchange. To a 100 round-bottom-flask equipped with a magnetic stir-bar was added $Rh_2(OAc)_4$ (1.0 equiv) and the corresponding carboxylic acid ligand obtained from the previous step (6 equiv). Chlorobenzene (0.05 M) was added, and the flask was fitted with a soxelet packed with anhydrous potassium carbonate. The soxelet was also fitted with an inverted condenser and the reaction mixture was set to stir overnight at reflux. Upon completion, PhCl was removed under reduced pressure. The crude mixture was dissolved in dichloromethane and passed through neutral alumina plug to remove excess of the ligand using dichloromethane as eluent. The combined organic solution was removed under vacuum. The crude product was further purified by flash chromatography (SiO$_2$, ethyl acetate in hexane) to afford (**3d-3f**) as a green solid.

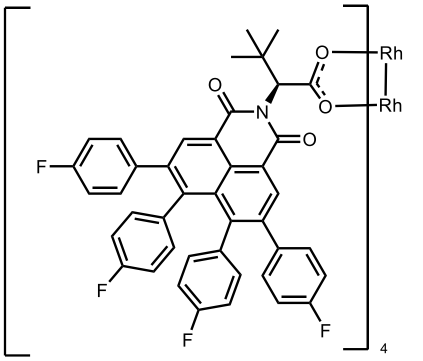

Rh_2(*S-tetra*-4-FC_6H_4NTTL)$_4$ (7d)

This compound was synthesized according to **general procedure F** using $Rh_2(OAc)_4$ (44.2 mg, 0.10 mmol, 1.0 equiv), and **SI-40** (413.0 mg, 0.6 mmol, 6.0 equiv). The crude mixture was purified by flash chromatography (SiO_2, 0-20% ethyl acetate in hexane) to yield **Rh_2(*S-tetra*-4-FC_6H_4NTTL)$_4$ (7d)** as green solid (251.5 mg, 85% yield).

R_f (4H/1EA) = 0.25 (UV 254 nm)

^{1}H NMR (800 MHz, $CDCl_3$) δ 8.75 (s, 1H), 8.28 (s, 1H), 7.13 (t, *J* = 6.7 Hz, 2H), 6.87 – 6.77 (m, 4H), 6.69 – 6.63 (m, 2H), 6.59 – 6.54 (m, 1H), 6.50 (t, *J* = 8.6 Hz, 1H), 6.47 – 6.36 (m, 6H), 5.72 (s, 1H), 1.22 (s, 9H).

^{13}C NMR (201 MHz, $CDCl_3$) δ 187.3, 164.1, 162.9, 162.3, 161.9, 161.8, 161.1, 160.7, 160.6, 144.1, 143.5, 142.4, 141.9, 136.79, 136.77, 136.69, 136.68, 135.51, 135.49, 135.42, 135.40, 133.99, 133.24, 133.20, 133.16, 133.12, 132.95, 132.93, 132.91, 132.89, 132.03, 131.99, 131.27, 131.23, 130.9, 128.20, 122.8, 122.3, 114.9, 114.8, 114.6, 114.5, 114.3, 114.2, 114.14, 114.08, 114.06, 114.03, 113.97, 61.5, 36.6, 28.8. *Due to the complexity of F coupling and lack of resolution, the C-F coupling constants (J) were not analyzed.*

^{19}F NMR (753 MHz, $CDCl_3$) δ -114.96, -115.12, -115.25, -115.84.

HRMS (+p ESI) calcd. for [$C_{168}H_{112}O_{16}N_4F_{16}{}^{103}Rh_2$] ($[M]^+$) 2950.5922 found 2950.6089. (Δ = 5.6 ppm)

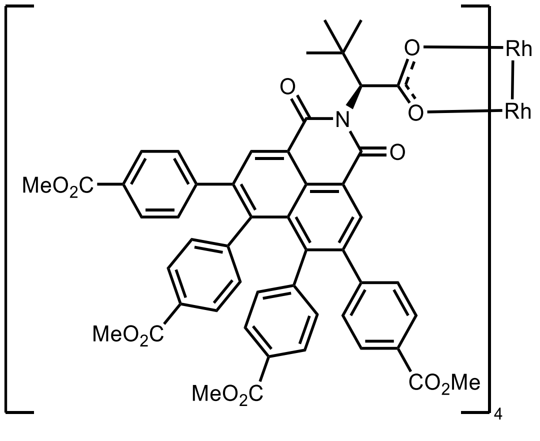

Rh_2(*S-tetra*-4-$CO_2MeC_6H_4$NTTL)$_4$ (7e)

This compound was synthesized according to **general procedure F** using $Rh_2(OAc)_4$ (8.4 mg, 0.019 mmol, 1.0 equiv), and **SI-41** (96.7 mg, 0.6 mmol, 6.0 equiv). The crude mixture was purified by flash chromatography (SiO_2, 0-5% MeOH in DCM) to yield **Rh_2(*S-tetra*-4-$CO_2MeC_6H_4$NTTL)$_4$ (7e)** as green solid (64.1 mg, 94% yield).

R_f (5% MeOH in DCM) = 0.30 (UV 254 nm)

^{1}H NMR (800 MHz, CDCl$_3$) δ 8.79 (s, 1H), 8.41 (s, 1H), 7.78 (d, J = 6.9 Hz, 2H), 7.67 (d, J = 7.9 Hz, 2H), 7.39 (d, J = 8.0 Hz, 1H), 7.32 (d, J = 8.1 Hz, 1H), 7.28 (d, J = 7.5 Hz, 2H), 7.23 (d, J = 8.1 Hz, 1H), 7.20 (d, J = 8.1 Hz, 1H), 6.97 (d, J = 7.9 Hz, 2H), 6.73 (d, J = 8.0 Hz, 1H), 6.61 (d, J = 8.0 Hz, 1H), 6.55 (t, J = 8.3 Hz, 2H), 5.73 (s, 1H), 3.87 (s, 3H), 3.82 (s, 3H), 3.81 (s, 3H), 3.79 (s, 3H), 1.20 (s, 9H).

^{13}C NMR (201 MHz, CDCl$_3$) δ 187.51, 166.94, 166.74, 166.41, 166.37, 163.85, 162.82, 145.25, 145.20, 143.86, 143.56, 143.49, 142.22, 141.85, 133.73, 132.92, 131.96, 131.79, 131.65, 131.63, 130.39, 130.02, 129.76, 129.12, 128.91, 128.88, 128.53, 128.41, 128.39, 128.32, 128.25, 128.23, 128.18, 123.17, 122.66, 61.57, 52.33, 52.17, 52.12, 36.59, 28.80.

HRMS (+p ESI) calcd. for [$C_{200}H_{161}O_{48}N_4{}^{103}Rh_2$] ([M+H]$^+$) 3591.8385 found 3591.8709. (Δ = 9.0 ppm)

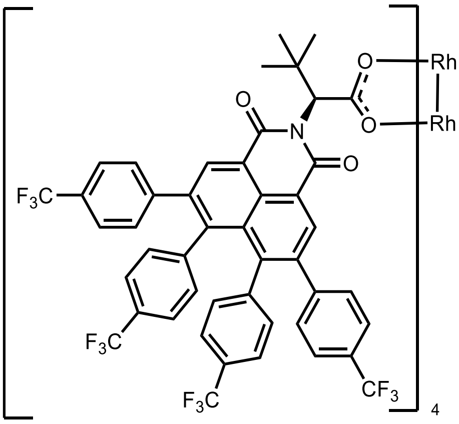

Rh$_2$(*S*-*tetra*-4-CF$_3$C$_6$H$_4$NTTL)$_4$ (7f)

This compound was synthesized according to **general procedure F** using $Rh_2(OAc)_4$ (44.2 mg, 0.10 mmol, 1.0 equiv), and **SI-39** (533.0 mg, 0.6 mmol, 6.0 equiv). The crude mixture was purified by flash chromatography (SiO_2, 0-20% ethyl acetate in hexane) to yield **Rh$_2$(*S*-*tetra*-4-CF$_3$C$_6$H$_4$NTTL)$_4$ (7f)** as green solid (331.7 mg, 88% yield).

R$_f$ (2H/1EA) = 0.4 (UV 254 nm)

^{1}H NMR (800 MHz, CDCl$_3$) δ 8.79 (s, 1H), 8.30 (s, 1H), 7.40 (d, J = 8.0 Hz, 2H), 7.28 (d, J = 8.0 Hz, 2H), 7.22 (d, J = 8.1 Hz, 2H), 7.06 (d, J = 8.1 Hz, 1H), 6.98 (dd, J = 7.2, 3.9 Hz, 3H), 6.93 (d, J = 8.2 Hz, 1H), 6.90 (d, J = 8.2 Hz, 1H), 6.78 (d, J = 8.1 Hz, 1H), 6.62 (q, J = 9.2 Hz, 3H), 5.74 – 5.67 (m, 1H), 1.20 (s, 9H).

^{13}C NMR (201 MHz, CDCl$_3$) δ 187.5, 163.8, 162.7, 143.7, 143.4, 143.0, 142.4, 142.3, 142.0, 141.6, 134.0, 132.8, 132.0, 131.8, 131.7, 131.5, 130.6, 130.5, 130.1, 129.93, 129.87, 129.77, 129.59, 129.57, 129.4, 129.2, 129.1, 128.4, 126.0, 125.8, 125.6, 125.5, 124.92, 124.90, 124.88, 124.68, 124.60, 124.58, 124.56, 124.54, 124.49, 124.24, 124.21, 124.18, 124.1, 123.33, 123.26, 123.1, 122.9, 122.83, 122.78, 122.0, 121.8, 121.51, 121.48, 61.7, 36.7, 28.7. *Due to the complexity of F coupling and lack of resolution, the C-F coupling constants (J) were not analyzed.*

^{19}F NMR (753 MHz, CDCl$_3$) δ -62.69, -62.87, -63.41.

HRMS (+p ESI) calcd. for [$C_{184}H_{112}O_{16}N_4F_{48}{}^{103}Rh_2$] ([M]$^+$) 3750.5412 found 3750.5764. (Δ = 9.4 ppm)

6. Catalyst optimization study

6.1. Study with diazo as Carbene Precursor

This study was initially started by the reaction of diazo as carbene precursor. The catalyst screening study is presented in **Table S4**. $Rh_2(NTTL)_4$ and its derivatives showed a preference for C_4 selectivity over C_3 using 3.4 ppm (d, 1H) signal. Although this signal was not perfectly resolved, it was enough to get a rough estimation of regio-selectivity ratio (rr). Tetra-arylated NTTL derivatives were not as selective as diarylated NTTL derivatives. The optimal catalyst was Rh_2(*S*-*di*-4-*t*-BuC_6H_4NTTL)$_4$ catalyst which gave 70% yield, 6:1 rr, and 81% ee. The addition of HFIP as an additive helps the reaction cleaner and helps slightly improve the enantioselectivity.

For most of the reaction the major regio-isomers were C_3-functionalized and C_4-functionalized products. However, C_1 (benzylic position) was also observed in some cases such as Rh_2(*S*-DOSP)$_4$ and Rh_2(*S*-TCPTAD)$_4$ with a characteristic signal was 3.7 ppm (s, 1H).

General procedure G. **C-H insertion with diazo as carbene precursor.** A clean oven dried and flame dried 8.0 ml scintillation vial (vial-A) equipped with a few (~10) activated 4-Angstrom molecular sieves and a small egg-shaped magnetic stir-bar was evacuated and purged with argon (2-3 times). After cooling down to room temperature, dirhodium tetracarboxylate catalyst (0.5 mol%) followed by the corresponding arylcyclohexane (0.600 mmol, 3 equiv.) were then added. The vial was once again evacuated and purged with argon (3-5 times) and 1.0 ml of dry dichloromethane was added. The vial and its content were then set to stir at room temperature under a nitrogen atmosphere. To a second oven dried vial (vial-B), that is evacuated and purged with argon, was added the aryldiazoacetate compound (0.200 mmol, 1 equiv.). Vial-B and its content were evacuated and purged with argon (2-3 times) and 1.0 ml of dry dichloromethane was then added. The diazo solution was transferred into a plastic syringe. Using a well calibrated syringe-pump, a slow addition of the diazo solution into the stirring solution of vial-A under an inert atmosphere was initiated. After complete addition (3 hours later), residual diazo compound in the plastic syringe was rinsed with ~0.5 ml dry dichloromethane and transferred dropwise into the stirring reaction mixture of vial-A. An additional 10-15 minutes was allowed before concentrating on the solution under reduced pressure. The crude product was analyzed by ^{1}H-NMR before being purified by flash chromatography (SiO_2, 0-10% diethyl ether in hexane).

Table S4. Catalyst screen with diazo as carbene precursor

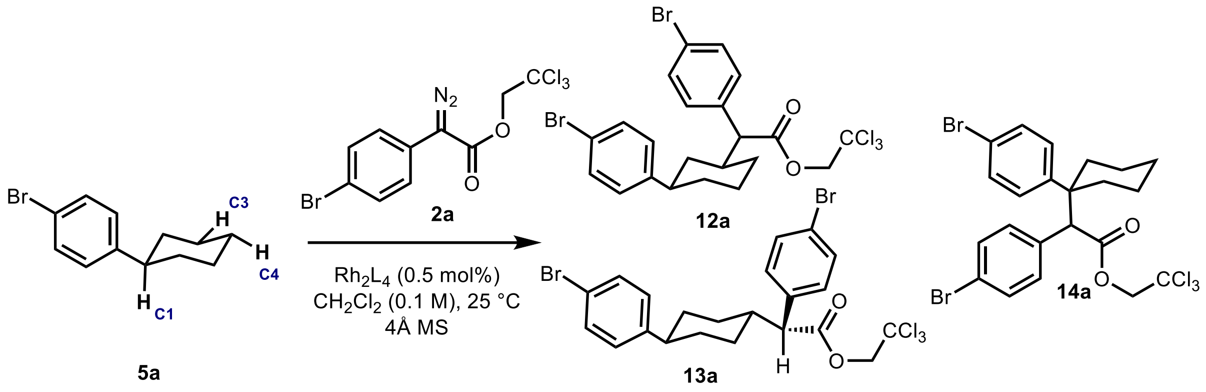

Entry[a]	Catalyst (0.5 mol%)	Yield, %[b]	13a:12a (C4:C3)[c]	13a:14a (C4:C1)[c]	14a ee ,%[d]
1	Rh_2(S-DOSP)$_4$	n.d.	1:2	4.8:1	n.d.
2	Rh_2(S-PTAD)$_4$	n.d.	1:1.5	6.6:1	n.d.
3	Rh_2(S-TCPAD)$_4$	n.d.	1:1.5	1:1	n.d.
4	Rh_2(S-2Cl5BrTPCP)$_4$	n.d.	1:1	>20:1	n.d.
5	Rh_2(S-TPPTTL)$_4$ (**1**)	41	1:2	36:1	n.d.
6	Rh_2(S-NTTL)$_4$ (**6**)	42	3:1	24:1	21
7	Rh_2(S-*tetra*-BrNTTL)$_4$ (**9**)	67	2.5:1	16:1	12
8	Rh_2(S-*tetra*-C_6H_5NTTL)$_4$ (**7a**)	81	4:1	22:1	13
9	Rh_2(S-*tetra*-4-^{t}BuC_6H_4NTTL)$_4$ (**7b**)	84	5:1	14:1	19
10	Rh_2(*S-di*-BrNTTL)$_4$ (**10**)	33	3:1	18:1	8
11	Rh_2(*S-di*-C_6H_5-NTTL)$_4$ (**8a**)	34	4:1	22:1	74
12	Rh_2(*S-di*-4-*C*$F_3C_6H_5$-NTTL)$_4$ (**8c**)	39	5:1	24:1	67
13	Rh_2(*S-di*-4-^{t}BuC_6H_5-NTTL)$_4$ (**8b**)	51	4:1	26:1	79
14	Rh_2(*S-di*-3,5-di-^{t}BuC_6H_5-NTTL)$_4$ (**8d**)	n.d.	1:3	1.5:1	n.d.
15[e]	Rh_2(*S-di*-C_6H_5-NTTL)$_4$ (**8a**)	44	6:1	34:1	76
16[e]	Rh_2(*S-di*-4-*C*$F_3C_6H_5$-NTTL)$_4$ (**8c**)	55	6:1	32:1	74
17[e]	Rh_2(*S-di*-4-^{t}BuC_6H_5-NTTL)$_4$ (**8b**)	70	6:1	30:1	81

[a]Reaction conditions: diazo **2a** (0.2 mmol, 1 equiv), **5a** (3 equiv), Rh_2L_4 (1 mol%), molecular sieves 4Å (100 wt%) in CH_2Cl_2 (0.1 M) at 25 ˚C. [b]Yields are isolated yields as a mixture of C_3 and C_4 products. [c]Regioisomers ratio C_4:C_3 was roughly determined by ^{1}H-NMR (3.3 – 3.4 ppm, hydrogen highlighted i n pink). [d]enantiomeric excess was determined by HPLC. [e]0.2 equiv of HFIP was added. The absolute stereocenter of C4 product was unambiguously determined by XRay.

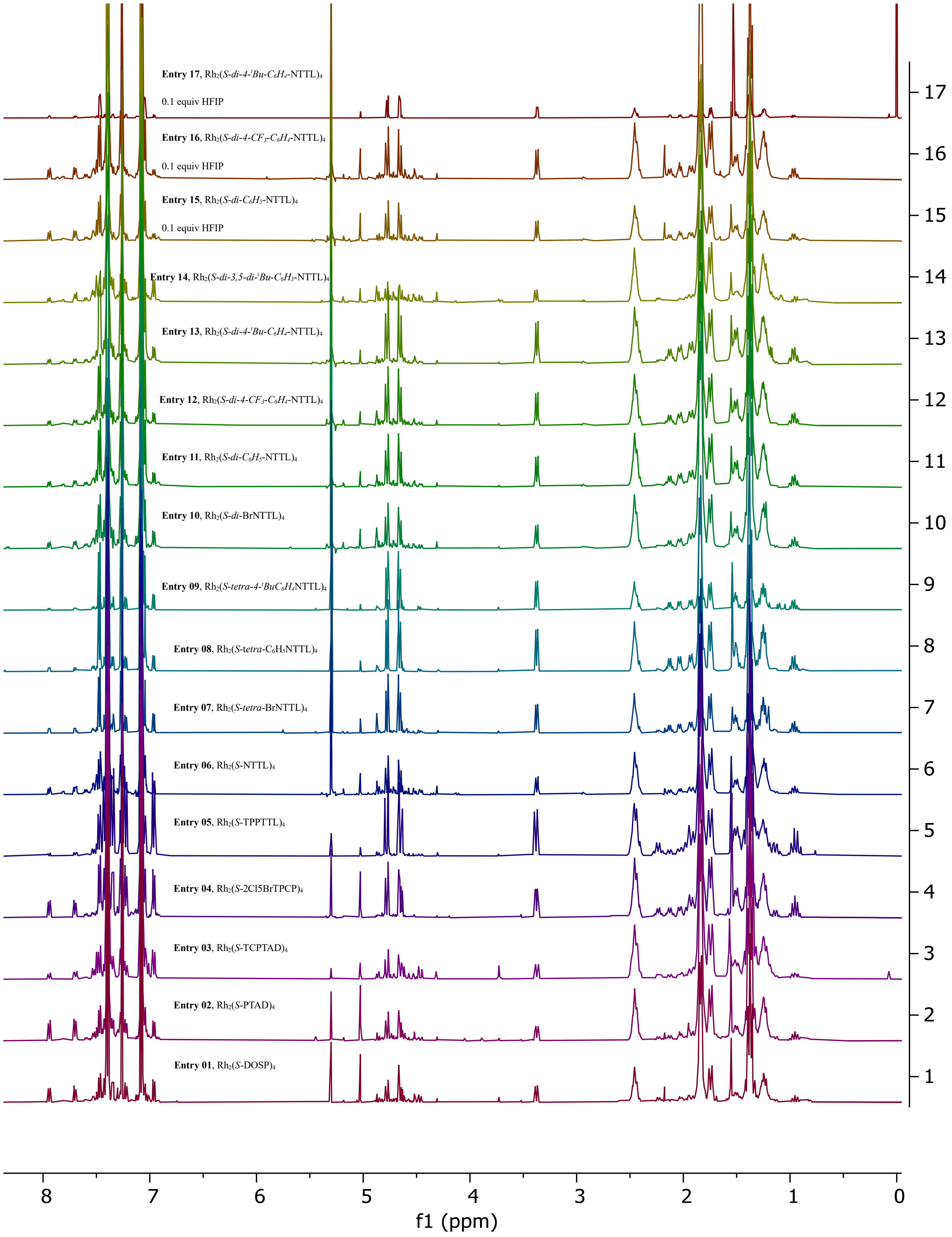

Figure S2. Stack crude NMR zoom in of reaction studies in Table S4 focusing on region of interest

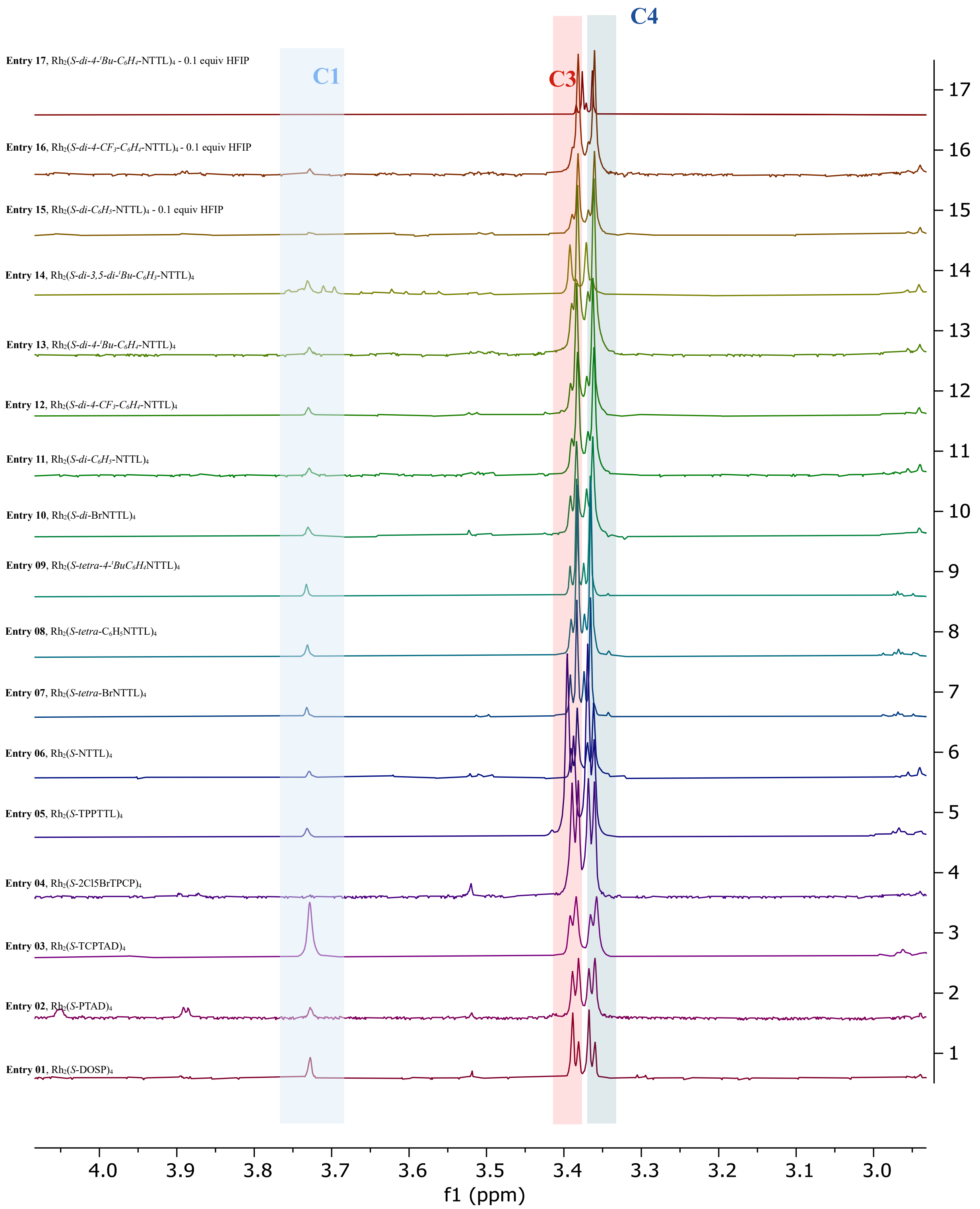

Figure S3. Stack crude NMR zoom in of reaction studies in Table S4 focusing on region of interest

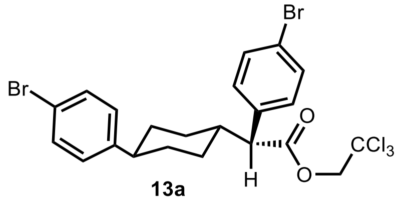

13a

2,2,2-trichloroethyl (R)-2-(4-bromophenyl)-2-(4-(4-bromophenyl)cyclohexyl)acetate (13a)

Prepared according to general procedures G. 1-bromo-4-cyclohexylbenzene (143 mg, 0.60 mmol, 3.0 equiv), Rh_2(*S-di-*4-^{t}BuC$_6$H$_4$NTTL)$_4$ (**8b**) (2.5 mg, 0.05 mol%, 0.005 equiv), and 2,2,2-trichloroethyl-2-(4-bromophenyl)-2-diazoacetate (74.5 mg, 0.20 mmol, 1.0 equiv) were used. The crude mixture was purified by flash chromatography (SiO_2, gradient

0%-10% Et_2O in hexane) afforded **13a** as an oil which slowly crystalized in hexane (81.6 mg, 70% yield, 81% ee, a 6:1 mixture of C4 and C3 products). The clean sample is further purified by HPLC. The absolute stereocenter is confirmed to be *(R)* configuration by Xray crystallography.

R_f (9H/1Et_2O) = 0.50 (CAM)

$[\alpha]^{20}_D$: -5.69° (c = 1.47 g/100 ml, $CHCl_3$, 80% ee)

^{1}H NMR (300 MHz, $CDCl_3$) δ 7.48 (d, *J* = 8.4 Hz, 2H), 7.40 (d, *J* = 8.4 Hz, 2H), 7.27 (d, *J* = 8.4 Hz, 2H), 7.06 (d, *J* = 8.5 Hz, 1H), 4.79 (d, *J* = 12.0 Hz, 1H), 4.67 (d, *J* = 12.0 Hz, 1H), 3.38 (d, *J* = 10.4 Hz, 1H), 2.44 (tt, *J* = 12.3, 3.5 Hz, 1H), 2.24 – 2.10 (m, 1H), 2.04 (dt, *J* = 12.5, 2.8 Hz, 1H), 1.94 (dt, *J* = 13.0, 2.9 Hz, 1H), 1.82 (dt, *J* = 13.1, 3.0 Hz, 1H), 1.64 – 1.46 (m, 2H), 1.41 – 1.18 (m, 2H), 0.97 (qd, *J* = 12.7, 3.5 Hz, 1H).

^{13}C NMR (75 MHz, $CDCl_3$) δ 171.7, 146.0, 135.9, 131.9, 131.6, 131.5, 130.5, 128.7, 121.9, 119.8, 94.9, 74.3, 58.2, 43.7, 40.5, 33.7, 33.7, 32.0, 30.6.

HRMS (+p APCI) calcd. for [$C_{22}H_{22}O_2Br_2Cl_3$] ([M+H]$^+$) 580.9047 found 580.9049.

HPLC (Chiralpak ODH column, 0.5% i-propanol in hexane, 0.5 mLmin^{-1}, 1.0 mgmL^{-1}, 45 min, UV 230 nm) retention times of 19.7 min (major) and 28.3 min (minor), 81% ee.

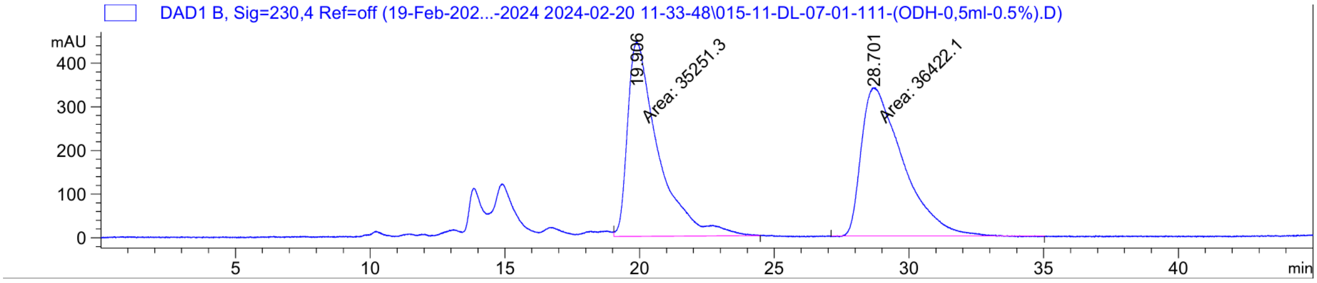

Signal 2: DAD1 B, Sig=230,4 Ref=off

Peak #	RetTime [min]	Type	Width [min]	Area [mAU*s]	Height [mAU]	Area %
1	19.906	MM	1.3199	3.52513e4	445.14026	49.1832
2	28.701	MM	1.7847	3.64221e4	340.13684	50.8168
Totals :				7.16734e4	785.27710	

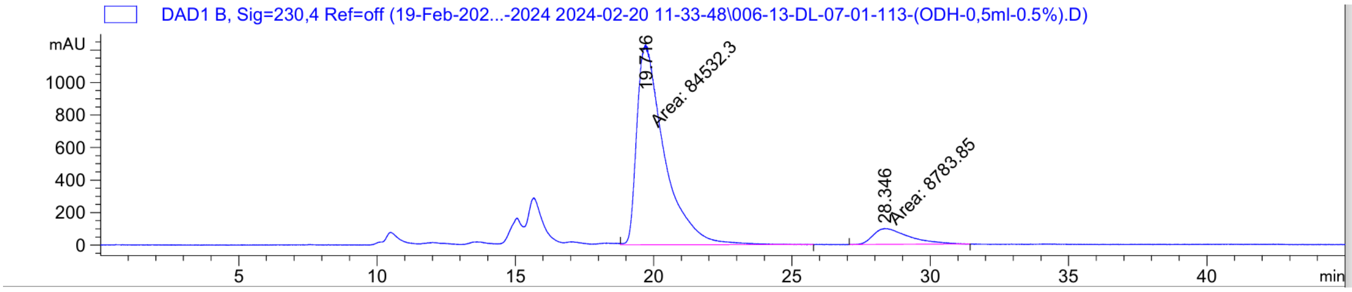

Signal 2: DAD1 B, Sig=230,4 Ref=off

Peak #	RetTime [min]	Type	Width [min]	Area [mAU*s]	Height [mAU]	Area %
1	19.716	MM	1.1423	8.45323e4	1233.31946	90.5870
2	28.346	MM	1.4914	8783.84863	98.16324	9.4130
Totals :				9.33162e4	1331.48270	

Figure S4. HPLC trace of 13a (racemic – top, chiral bottom)

6.2. Study with triazole as carbene precursor

General procedure H. ***C-H functionalization with triazole as carbene precursor***

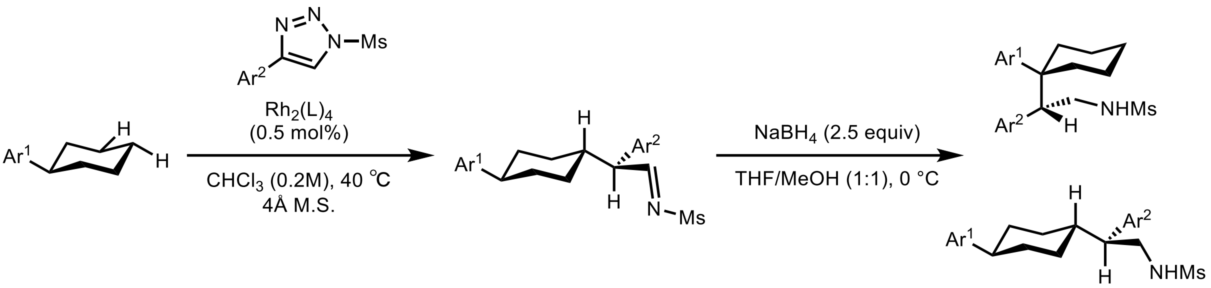

Scheme S4. C-H functionalization with triazole as carbene precursor

To an oven-dried 4 mL vial equipped with a stirring bar under nitrogen atmosphere, aryl cyclohexane (0.5 mmol, 2.5 equiv) was added to the vial first if it was a liquid. Otherwise, it will be added with triazole (0.2 mmol, 1.0 equiv), Rh_2L_4 (0.5 mol%) were added. The vial was vacuumed and refilled with N_2 before dried chloroform (1 ml, 0.2 M) was added. The resulting green reaction mixture was stirred at ambient temperature overnight. The reaction color often changes from green to yellow after 20 min and brown-red color after overnight. After reaction completion, the crude ^{1}H-NMR of the reaction mixture was taken to determine the regio-isomeric ratio. Then, the solvent was removed under vacuum followed by the addition of the mixture of THF/MeOH (1 ml, 1:1 v/v, 0.2 M). The reaction mixture then was cooled in ice-water bath, and $NaBH_4$ (18.9 mg, 0.5 mmol, 2.5 equiv) was added portion wise at 0 °C. The resulting mixture was stirred for 30 minutes at 0 °C, and then carefully quenched with water and extracted with ethyl acetate dried over anhydrous sodium sulfate. After removal of solvent, the residue was loaded on silica gel and purified by flash chromatography (SiO_2, 0-65% Et_2O in hexane) to get C_4-functionalized product as white solid. *It is worth noting that C_4 product was isolated as a mixture with C_3 product as they are inseparable by flash chromatography.*

Note: The imine product from C-H functionalization reaction is not stable on SiO_2. It can be hydrolyzed to the corresponding aldehyde by using K_2CO_3 and H_2O or wet silica, but the aldehyde will be racemized under hydrolysis conditions.

6.2.1. Determination of Regioisomers on crude NMR with triazole as carbene precursor

To measure the product ratios, the crude reaction mixtures were analyzed by ^{1}H NMR using the following settings:

Instrument: 400 MHz INOVA equipped with an ID probe with sensitivity of 1000:1

Number of scans: 16

Relaxation time: 5 seconds

The crude 1 H NMR spectra data was processed using MestReNova 15.0.0 (Mestrelab Research S.L.), applying an auto-phase correction as well as a Segments Smoother baseline correction. The baseline was manually inspected before integration.

Showing in **Scheme S5** is the model reaction with para-bromo phenyl cyclohexane and N-sulfonyl diazo. The crude NMR of the C-H functionalization before reduction is showed in **Figure S6** and crude NMR after reduction by $NaBH_4$ is shown in **Figure S7**. As demonstration in **Figure S8**, the resulting amine after reduction only 1 set of distinctive signals (4 singlets around 2.7 ppm, methyl of mesyl group) for distinguishing between C1, C3, C3', and C4 product. On

the other hand, there are three distinctive regions to determine rr with the crude NMR before reduction including imines (8.7 ppm), benzylic (3.5-3.7 ppm), and methyl of mesyl group (3.0 ppm). Although the imine regio was not able to resolve C1, C3, and C4 product, the combination of benzylic and mesyl group was able to help calculate the regioisomeric ratio. It is worth noting that the ratio of (C4:C3+C3') is often not well resolved for rr>8:1. In those cases, the rr value was determined by SFC trace of the purified product after reduction along with correction by response factor. **Figure S9** demonstrated an example of racemic SFC trace of crude reaction mixture, purified C1 product, and purified (C3,C4) products. As a result, the rr value was determined at the imine stage.

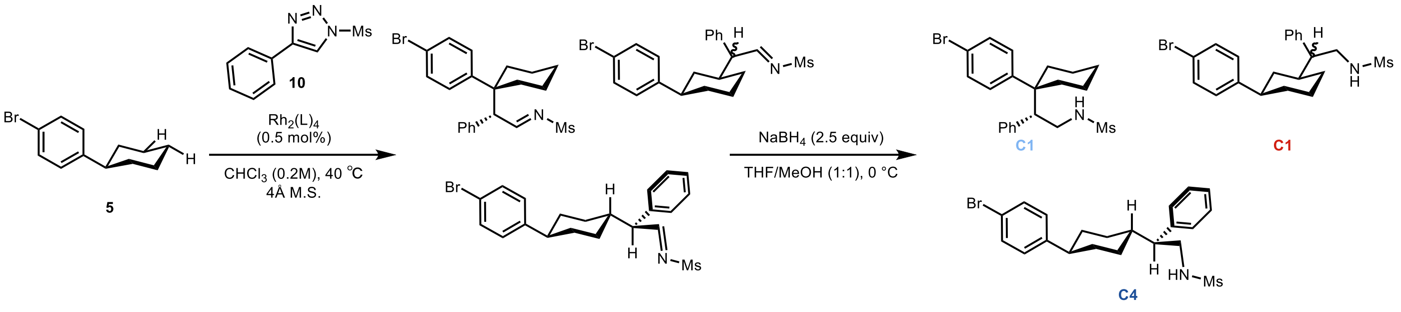

Scheme S5. Model reaction

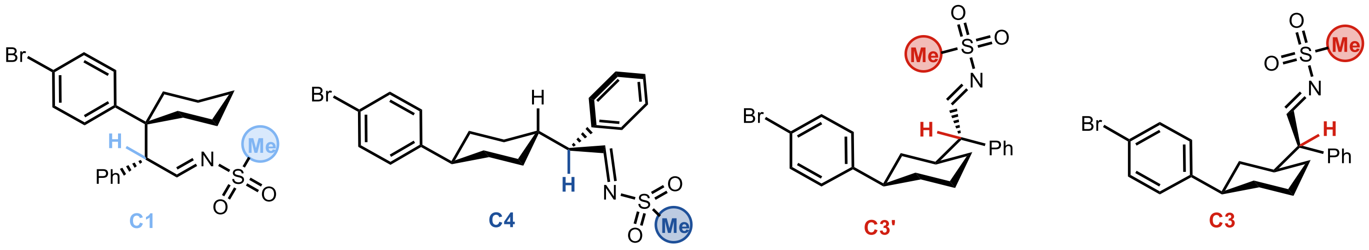

Figure S5. Highlighted signals for regioisomeric ratio measured by ^{1}H-NMR integration

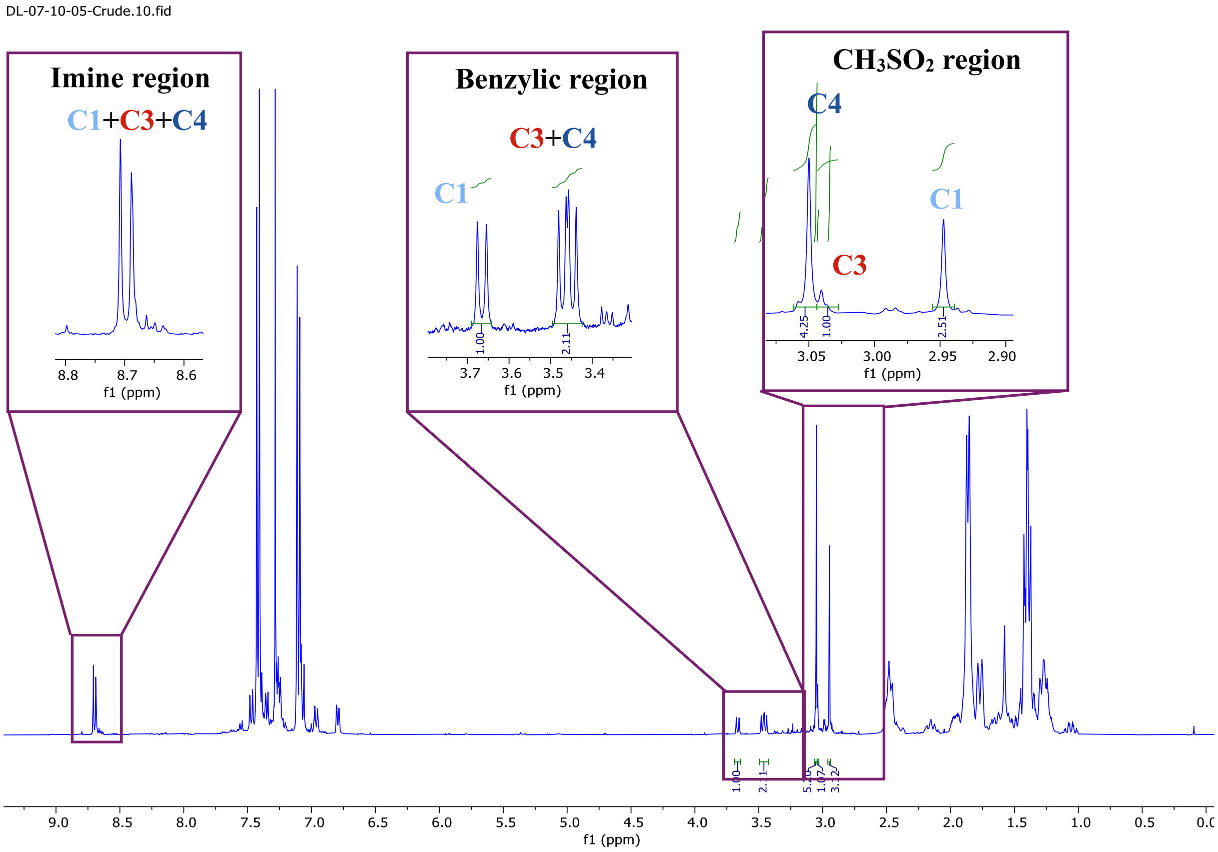

Figure S6. Example of crude NMR of triazole C-H functionalization with triazole. *Reaction described in step 1 of Scheme S5 with $Rh_2(S\text{-}NTTL)_4$ as a catalyst*

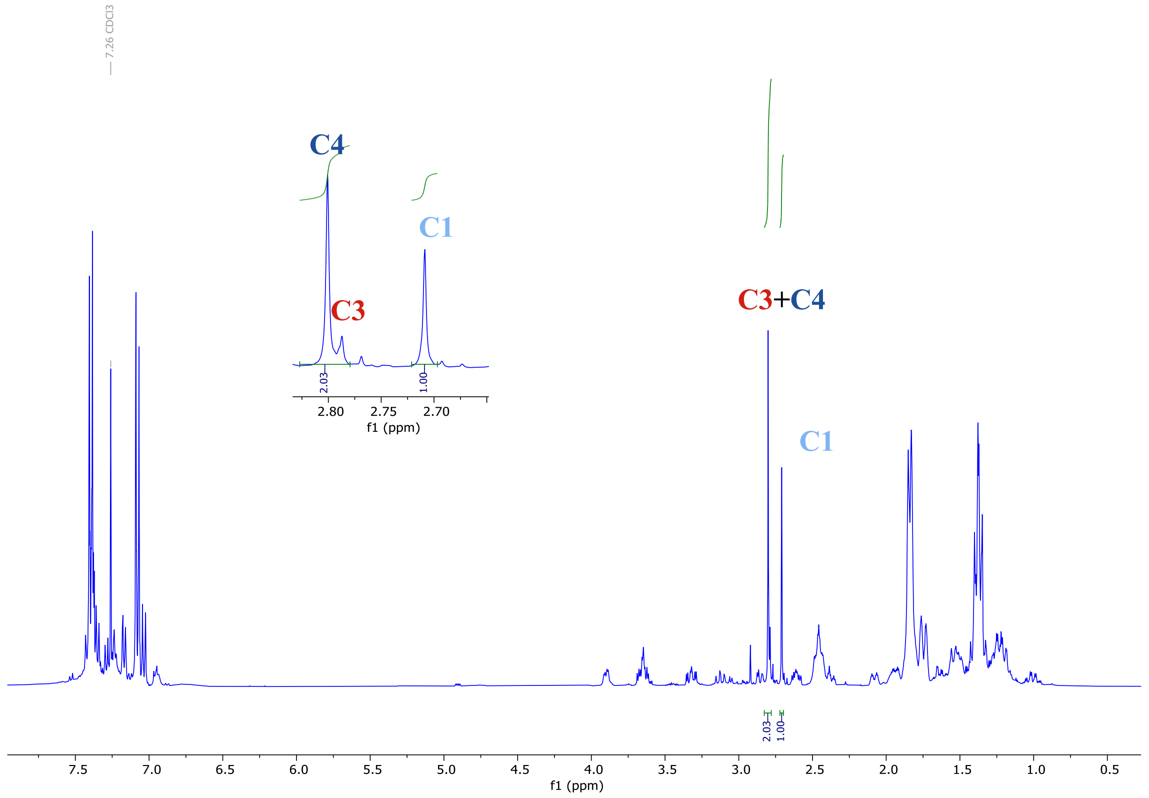

Figure S7. Example of crude NMR of triazole C-H functionalization with triazole after reduction. *Reaction described in step 2 of Scheme S5 with $Rh_2(S\text{-}NTTL)_4$ as a catalyst*

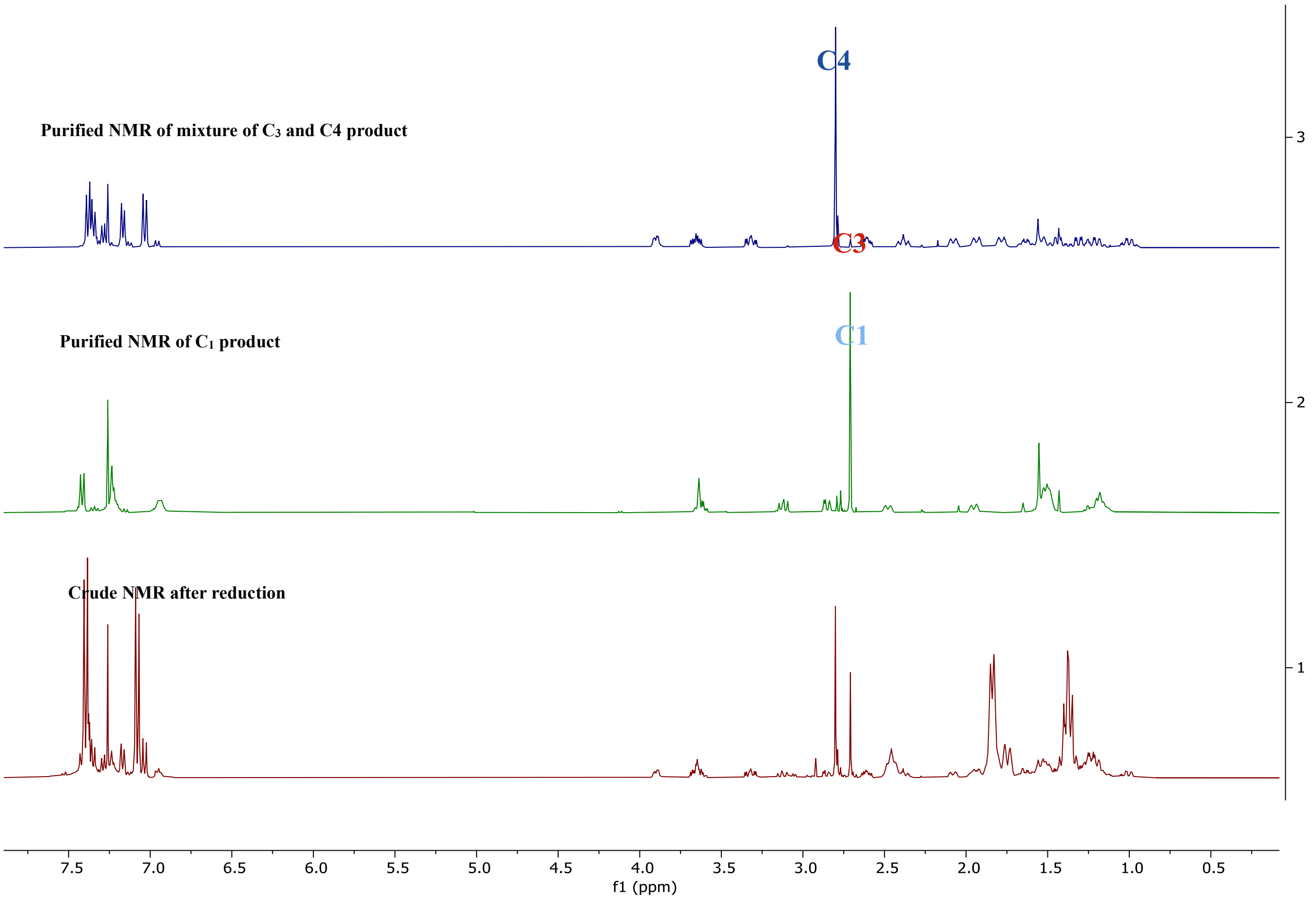

Figure S8. Stack of crude NMR after reduction and purified NMR of C_1 and C_4 products. *Reaction described in step 2 of Scheme S5 with $Rh_2(S\text{-}NTTL)_4$ as a catalyst*

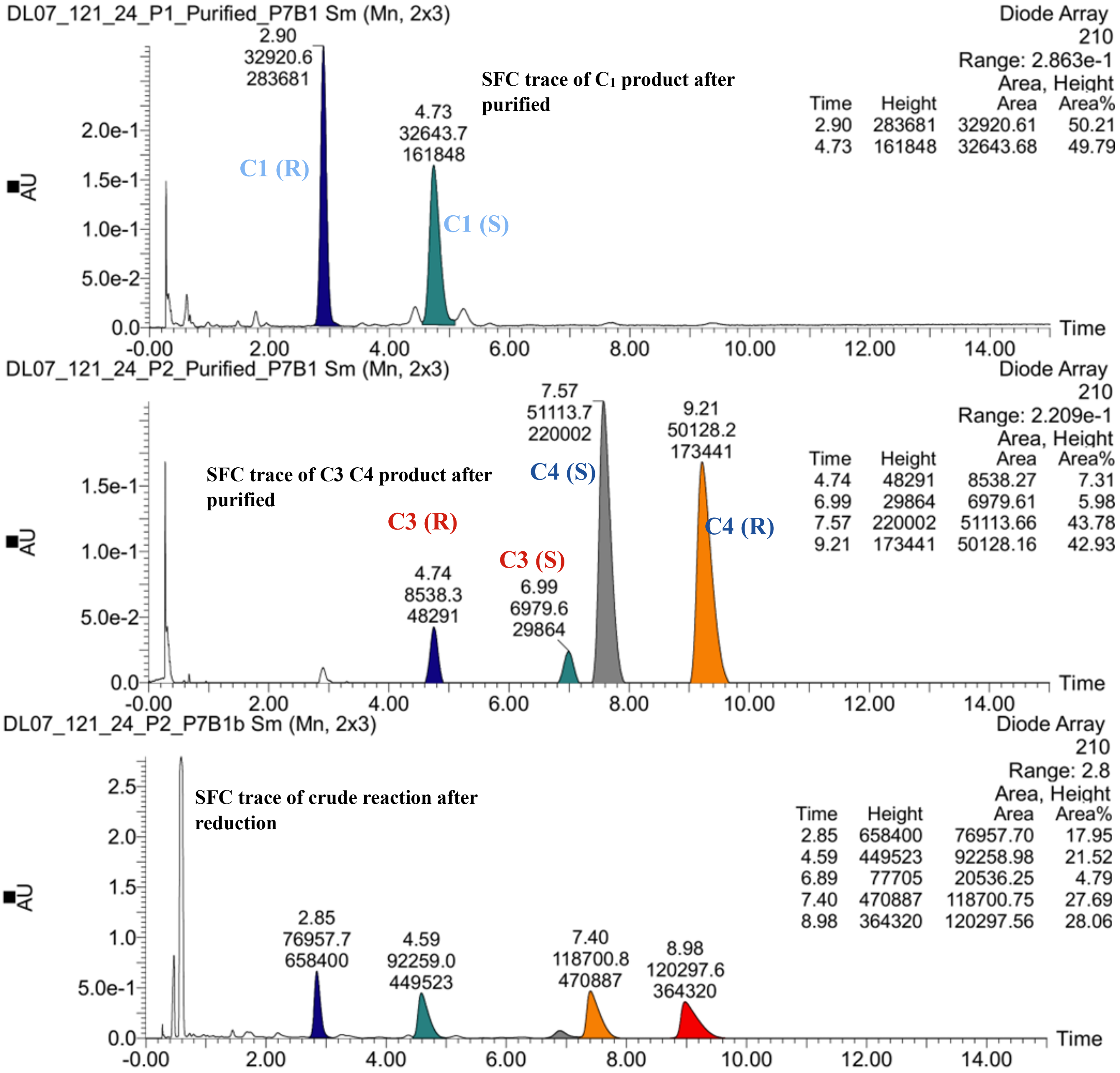

Figure S9. Stack of crude SFC analysis after reduction of reaction. *Reaction described in step 2 of Scheme S5 with Rh_2(R/S-NTTL)$_4$ as a catalyst. SFC condition:* OJ3, 10% (50% methanol in isopropanol with 0.2% Formic Acid) in CO_2, 2.5 mL/min, 1.0 mg/ml, UV 210 nm

Retention time (min)	Product
2.85	C1 (R isomer)
4.59	C1 (S isomer) and C3 (R isomer)
6.89	C3 (S isomer)
7.40	C4 (S isomer)
8.98	C4 (R isomer)

The assignment of the R/S product is based on the preferred product of chiral catalyst. For Rh_2(*S*-NTTL)$_4$ and its derivatives, the open face

6.2.2. Reaction optimization for electron deficient aryl cyclohexanes

Table S5. Reaction optimization with electron-deficient aryl cyclohexane and triazole as carbene precursor

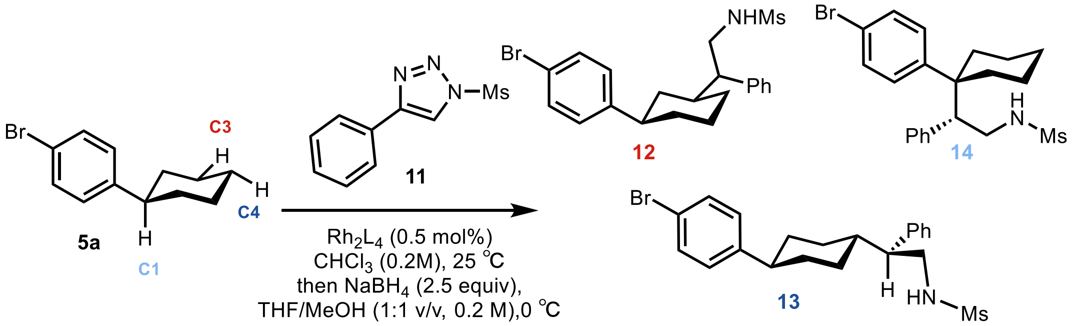

Entry[a]	*Catalyst* (0.5 mol%)	*Yield, %*[b]	*13:12* (C4:C3)[c]	*13:14* (C4:C1)[c]	*13 ee ,%*[d]	*14 ee ,%*[e]
1	Rh_2(*S*-TPPTTL)$_4$ (**1**)	18	1.1:1	1.3:1	18	-
2	Rh_2(*S*-NTTL)$_4$ (**6**)	42	5.4:1	1.5:1	96	93
3	Rh_2(*S-tetra*-BrNTTL)$_4$ (**9**)	trace	-	-	-	-
4	Rh_2(*S-di*-BrNTTL)$_4$ (**10**)	trace	-	-	-	-
5	Rh_2(*S-tetra*-C_6H_5NTTL)$_4$ (**7a**)	79	13:1[f]	9.2:1	95	40
6	Rh_2(*S-tetra*-4-^{t}BuC_6H_4NTTL)$_4$ (**7b**)	87	14:1[f]	6.9:1	95	8
7	Rh_2(*S-tetra-p*-MeOC_6H_4NTTL)$_4$ (**7c**)	83	26:1[f]	18.7:1	95	-
8	Rh_2(*S-tetra-p*-$CF_3C_6H_4$NTTL)$_4$ (**7f**)	63	11:1[f]	8.6:1	95	33
9	Rh_2(*S-di*-C_6H_5NTTL)$_4$ (**8a**)	64	7.4:1	3.5:1	97	87
10	Rh_2(*S-di*-4-^{t}BuC_6H_4NTTL)$_4$ (**8b**)	47	7.9:1	4.3:1	97	94
11	Rh_2(*S-di*-4-*CF_3*C_6H_4NTTL)$_4$ (**8c**)	48	9.3:1	3.5:1	97	86
12	Rh_2(*S-di*-3,5-di-^{t}BuC_6H_5NTTL)$_4$ (**8d**)	55[g]	1:1.7	1:8.9	-	98

[a]Reaction conditions: triazole **11** (0.2 mmol, 1 equiv), trap **5a** (2.5 equiv), Rh_2L_4 (0.5 mol%) in dry $CHCl_3$ (1.0 ml, 0.2 M) at 25 ˚C. [b]Yields are isolated yields and reported as combined yields of C_3 and C_4 products. [c]Ratio C_4:C_3 and C_4:C_1 were determined by crude ^{1}H-NMR before reduction. [d]Enantiomeric excess of **13** (C_4 product) was determined by SFC. [e]Enantiomeric excess of C_1 product was determined by HPLC. [f]Ratio of C4:C3 was determined based on SFC with response factor due to poor resolution of NMR (see below for more details). [g]Isolated yield of **14** (C_1 product).

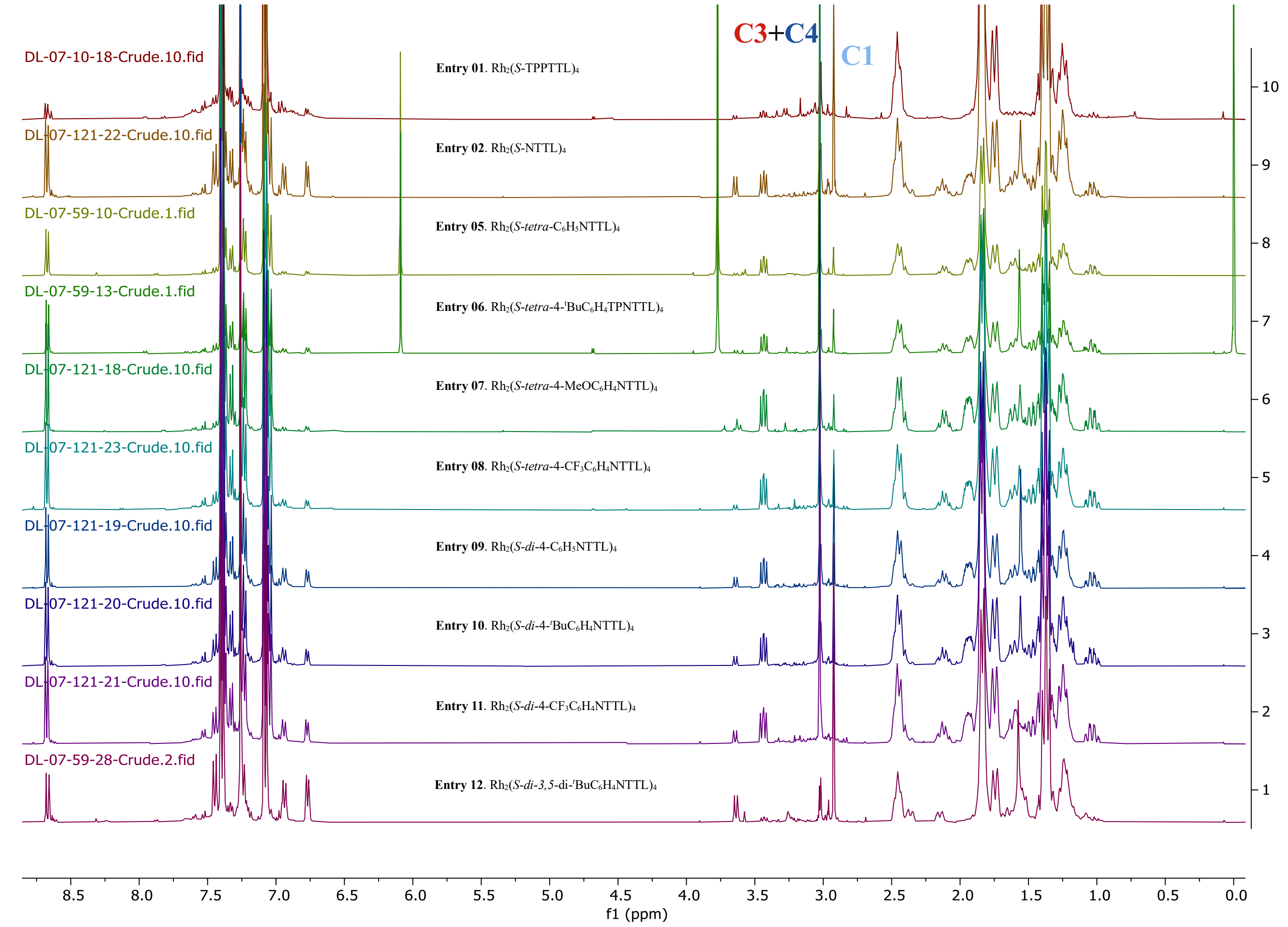

Figure S10. Stack overall crude NMR of reaction before reduction studied in Table S5

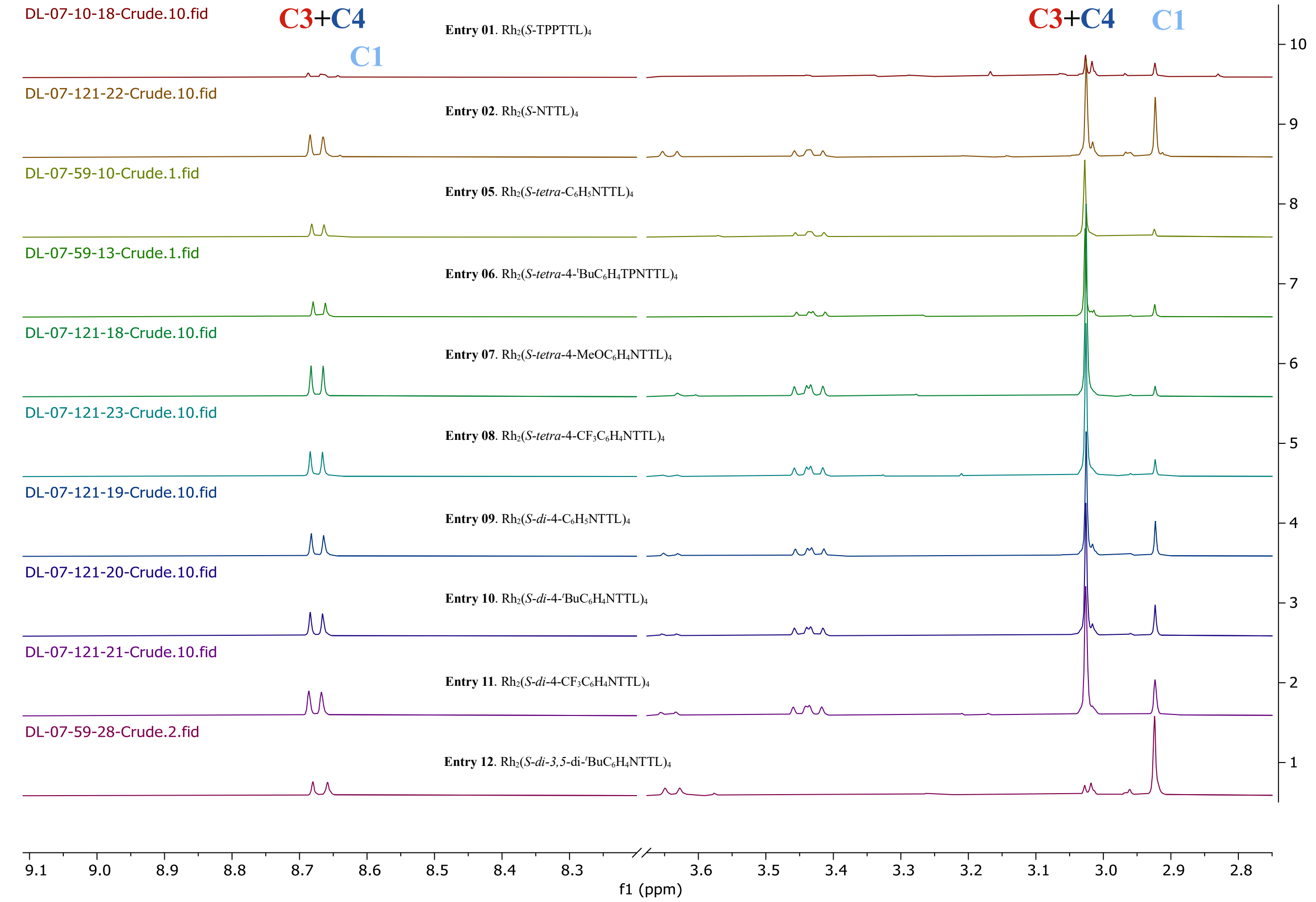

Figure S11. Stack overall crude NMR of reaction before reduction studied in Table S5

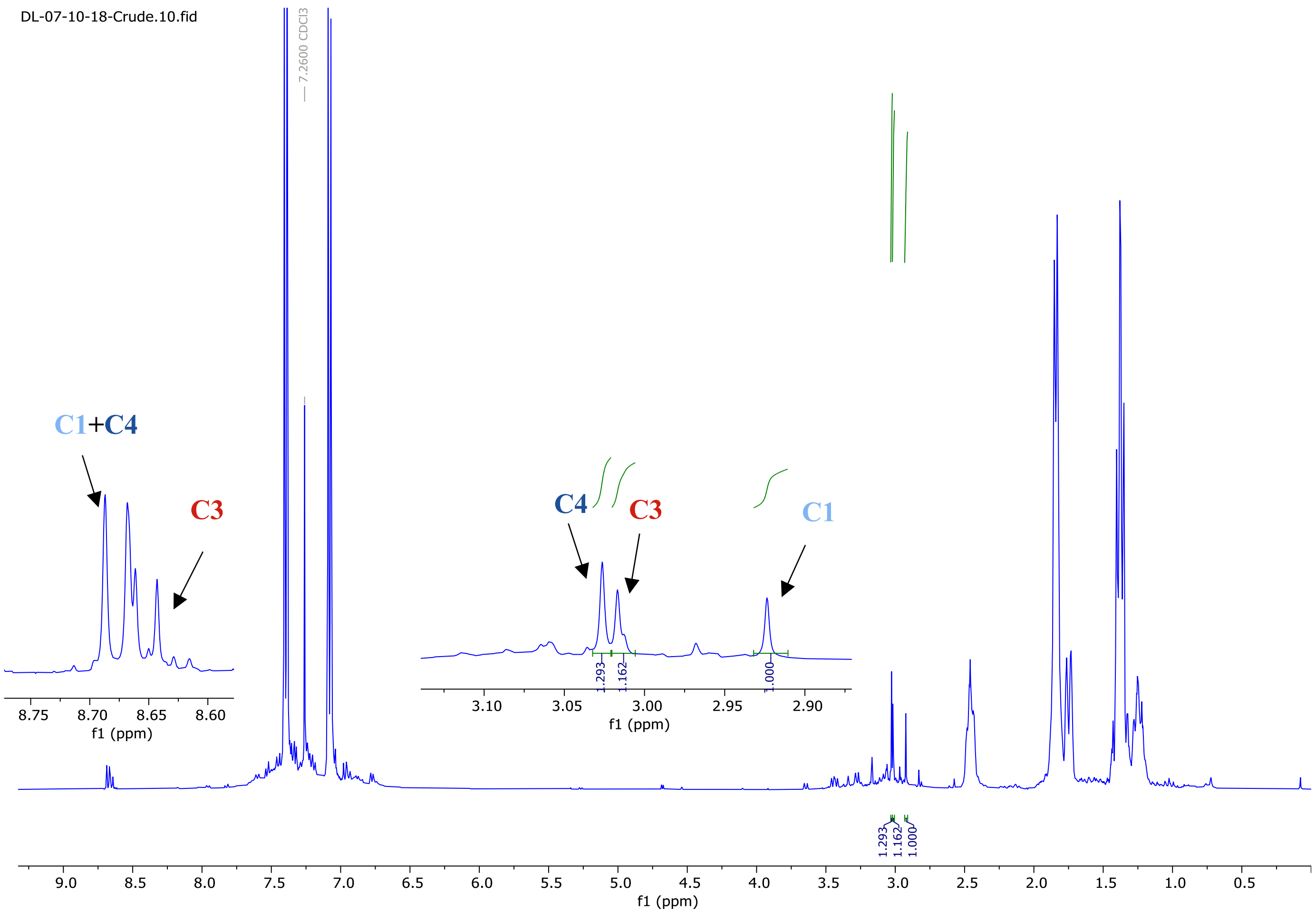

Figure S12. Crude NMR before reduction of entry 1 in Table S5 – Rh_2(*S*-TPPTTL)$_4$.

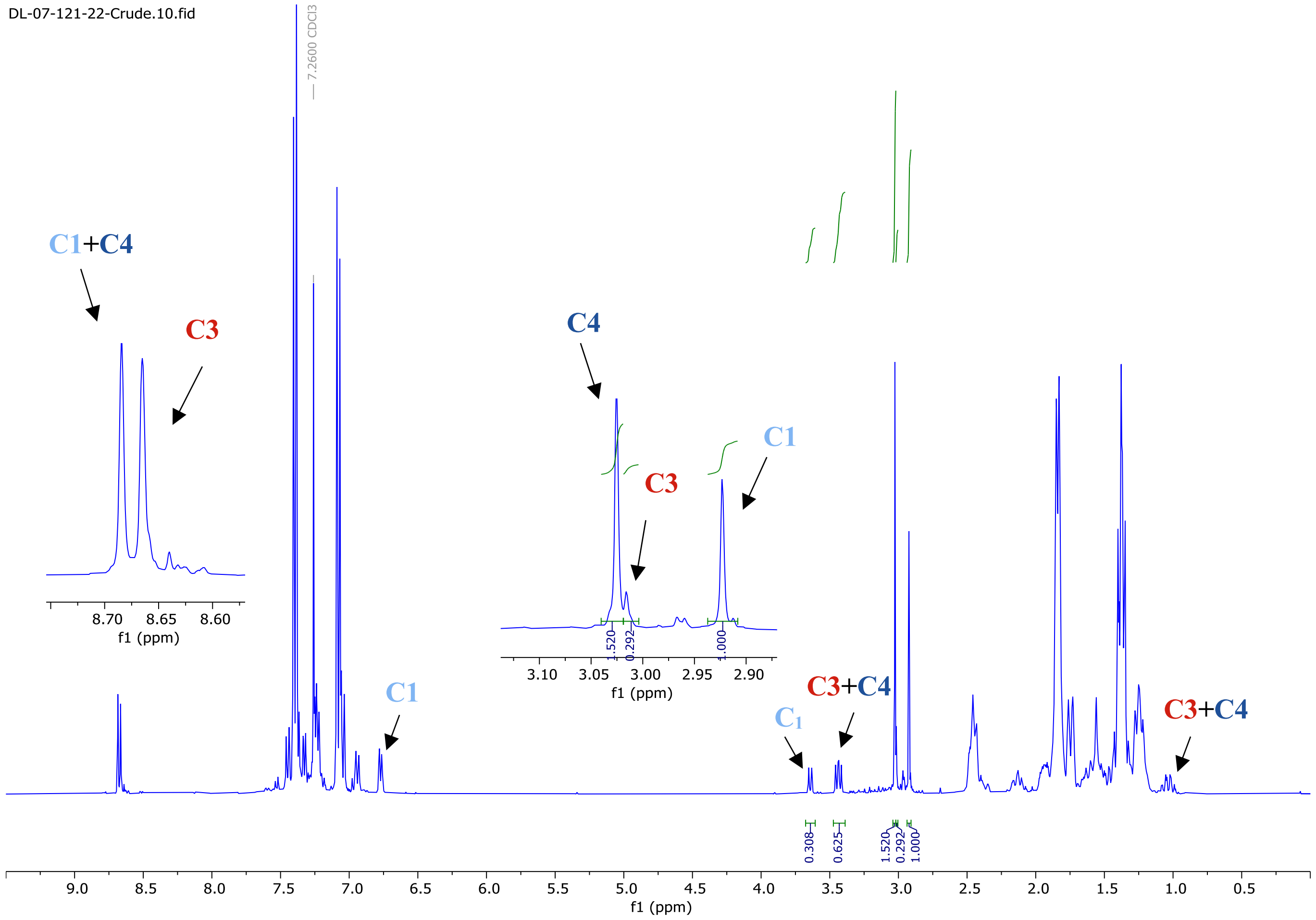

Figure S13. Crude NMR before reduction of entry 2 in Table S5 – Rh_2(*S*-NTTL)$_4$.

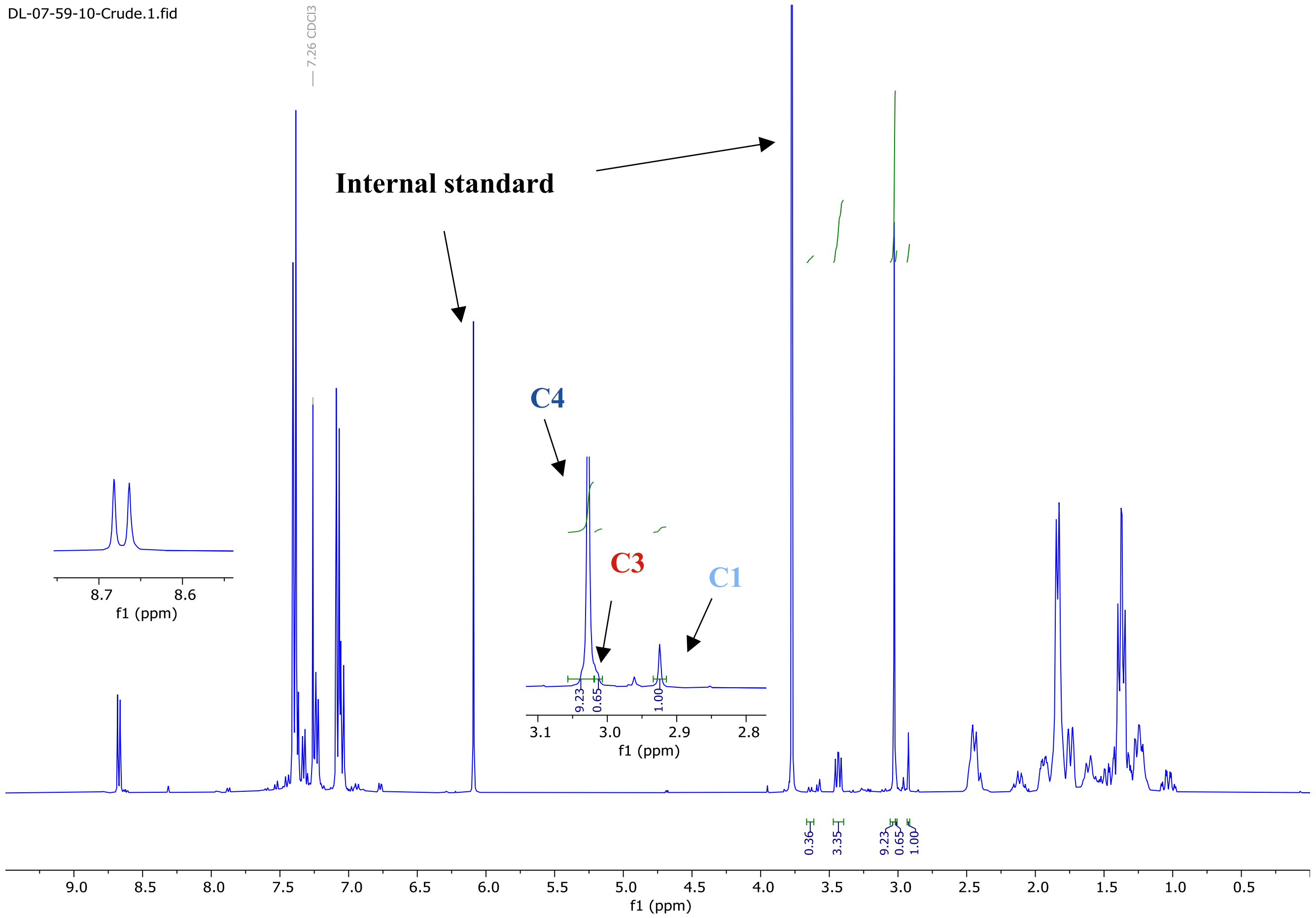

Figure S14. Crude NMR before reduction of entry 5 in Table S5 – Rh$_2$(*S-tetra*-C$_6$H$_5$NTTL)$_4$. Internal standard: 1,3,5-trimethoxybenzene

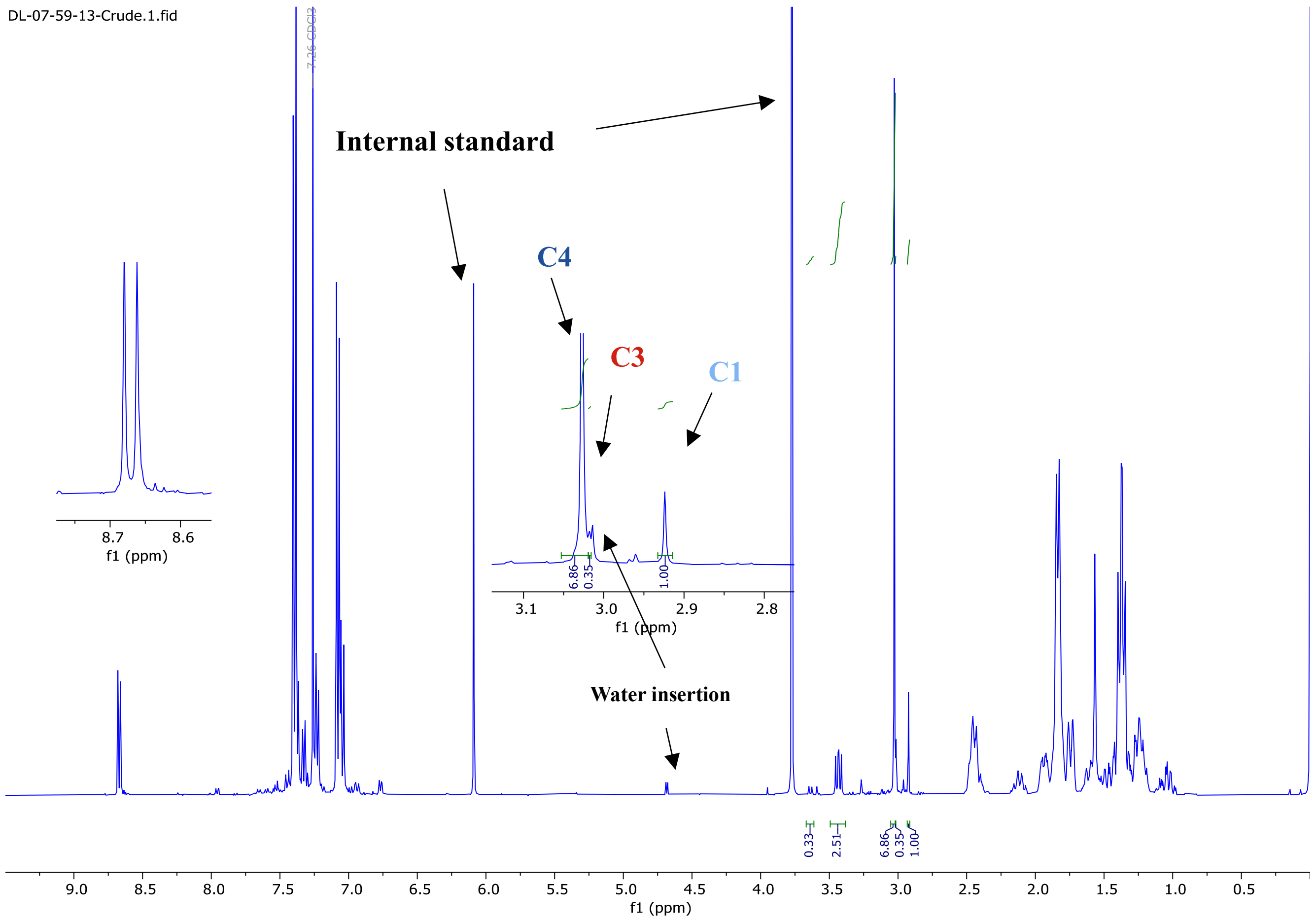

Figure S15. Crude NMR before reduction of entry 6 in Table S5 – Rh$_2$(*S-tetra*-4-^{t}BuC$_6$H$_4$NTTL)$_4$. Internal standard: 1,3,5-trimethoxybenzene. Water insertion was observed due to adventitious moisture

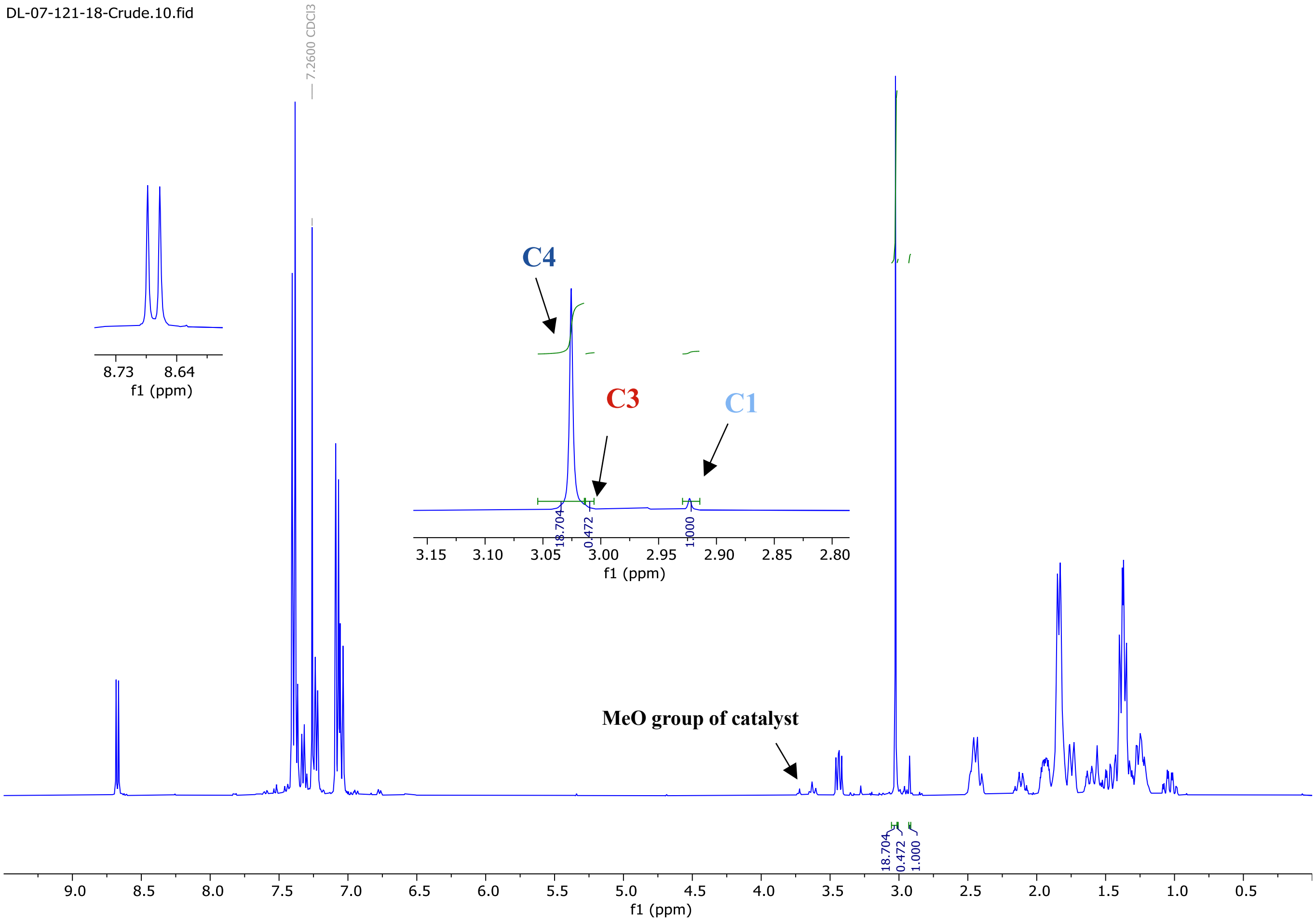

Figure S16. Crude NMR before reduction of entry 7 in Table S5 – Rh_2(*S-tetra*-4-$MeOC_6H_4$NTTL)$_4$.

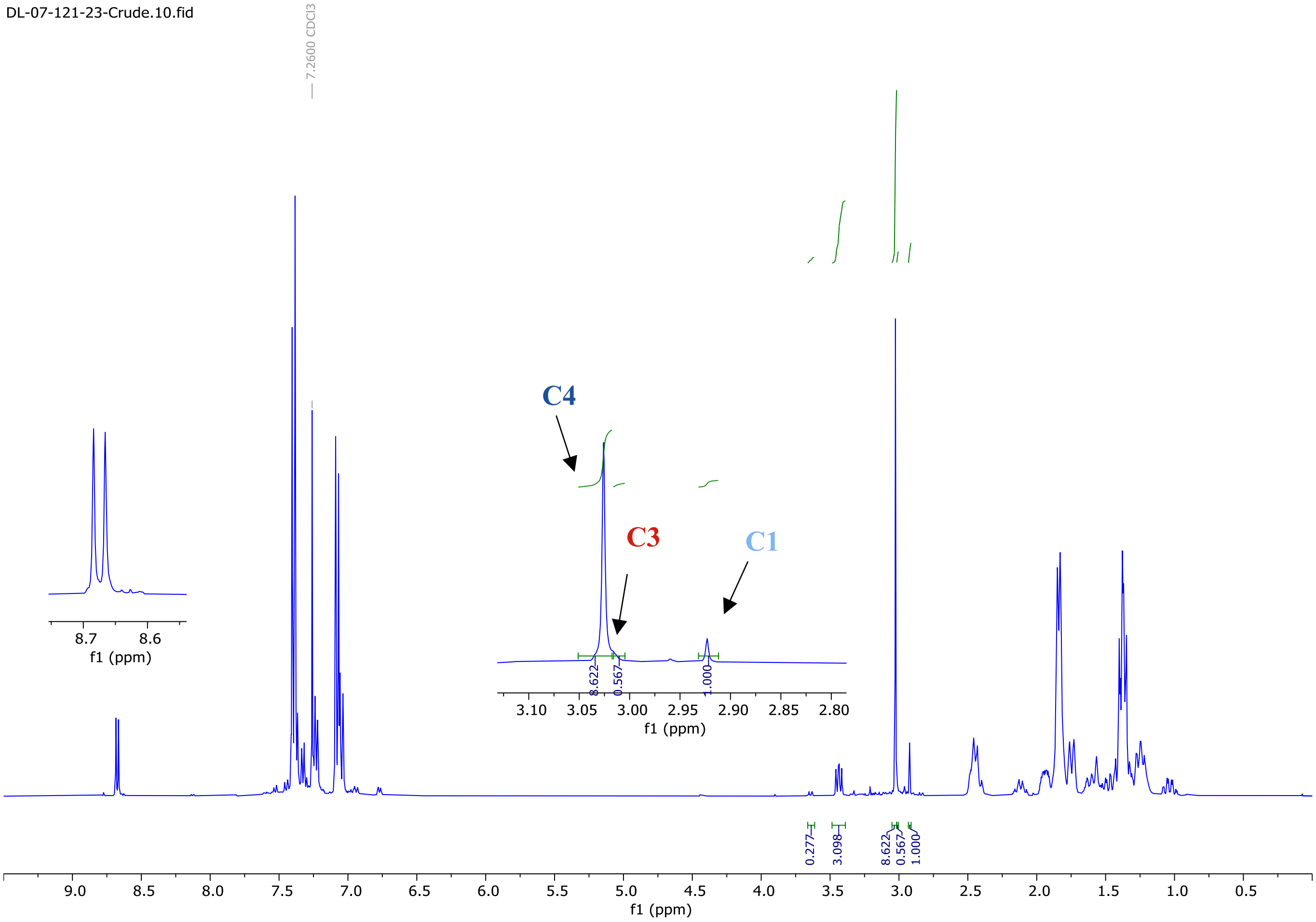

Figure S17. Crude NMR before reduction of entry 8 in Table S5 – Rh_2(*S-tetra*-4-$CF_3C_6H_4$NTTL)$_4$.

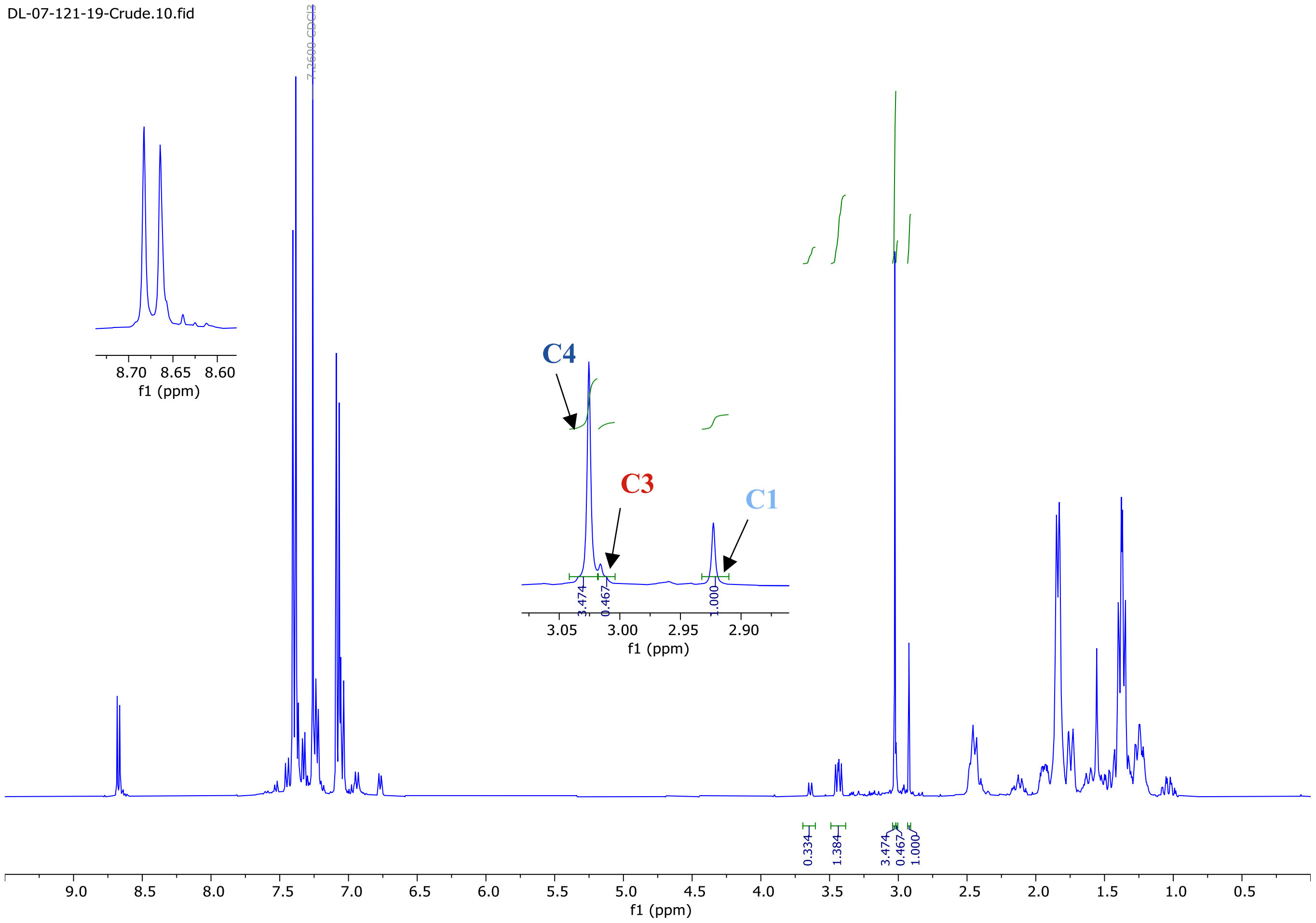

Figure S18. Crude NMR before reduction of entry 9 in Table S5 – Rh_2(*S*-*di*-4-C_6H_5NTTL)$_4$.

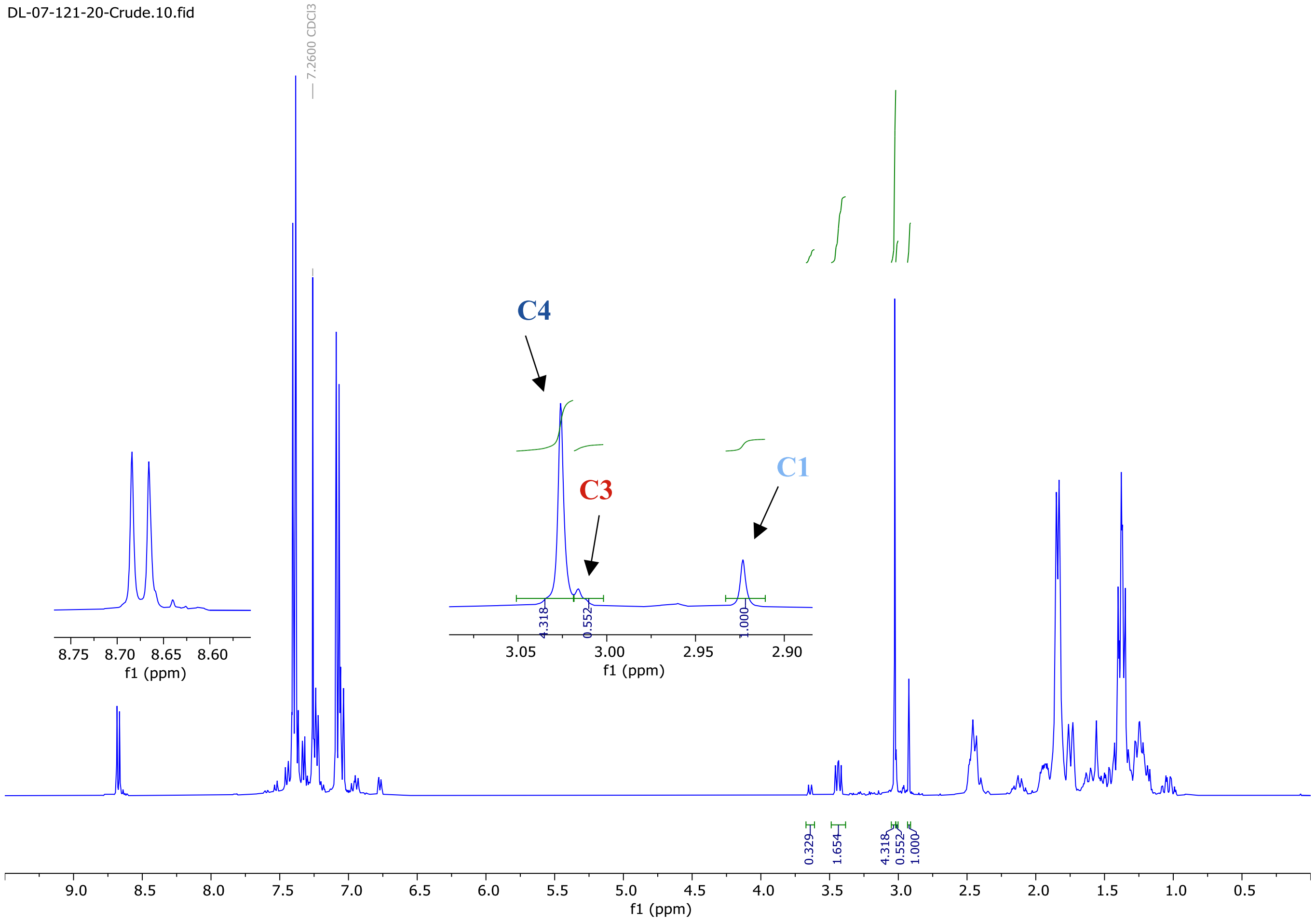

Figure S19. Crude NMR before reduction of entry 10 in Table S5 – Rh_2(*S*-*di*-4-^{t}BuC_6H_4NTTL)$_4$.

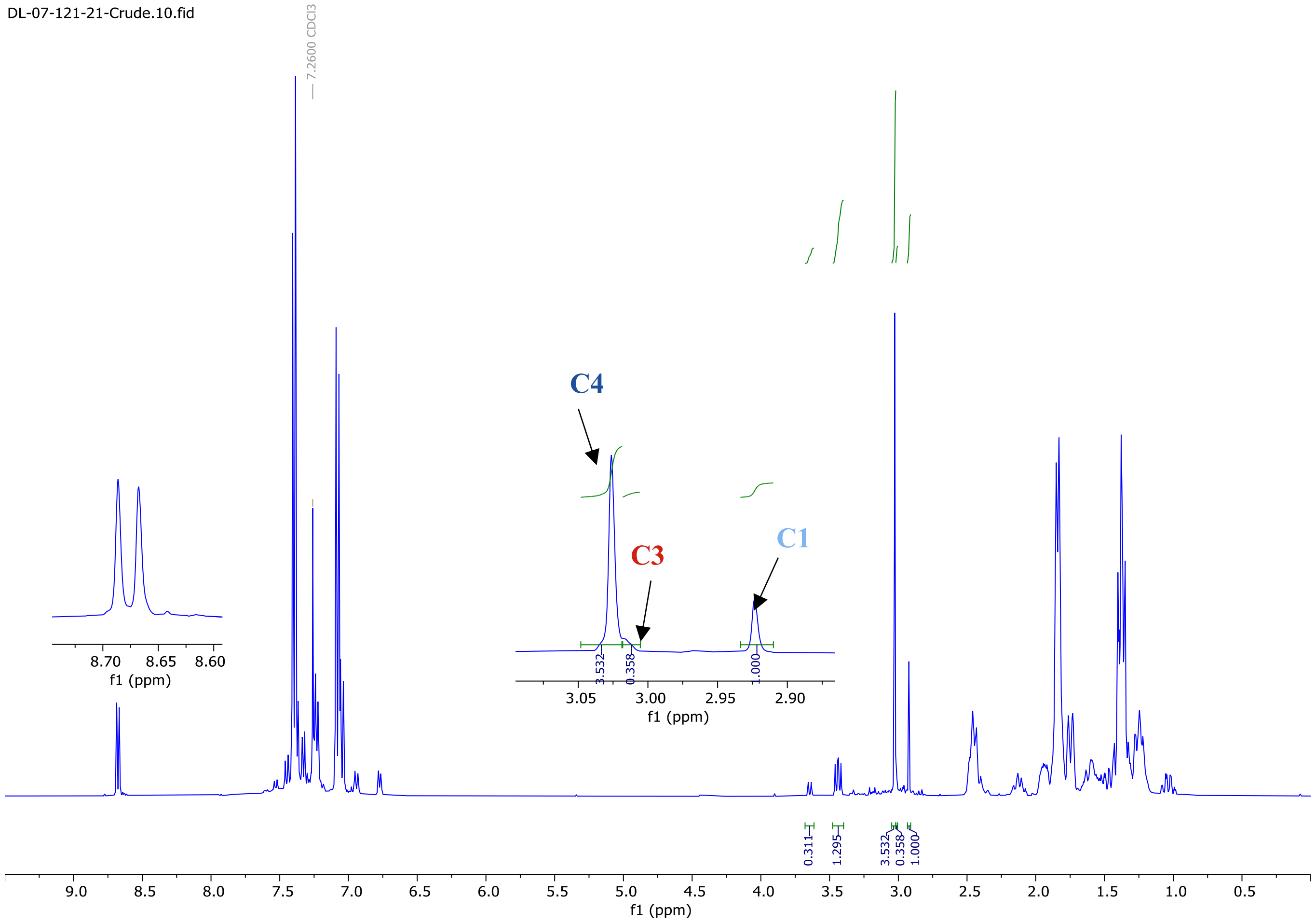

Figure S20. Crude NMR before reduction of entry 11 in Table S5 – Rh$_2$(*S*-*di*-4-CF$_3$C$_6$H$_4$NTTL)$_4$.

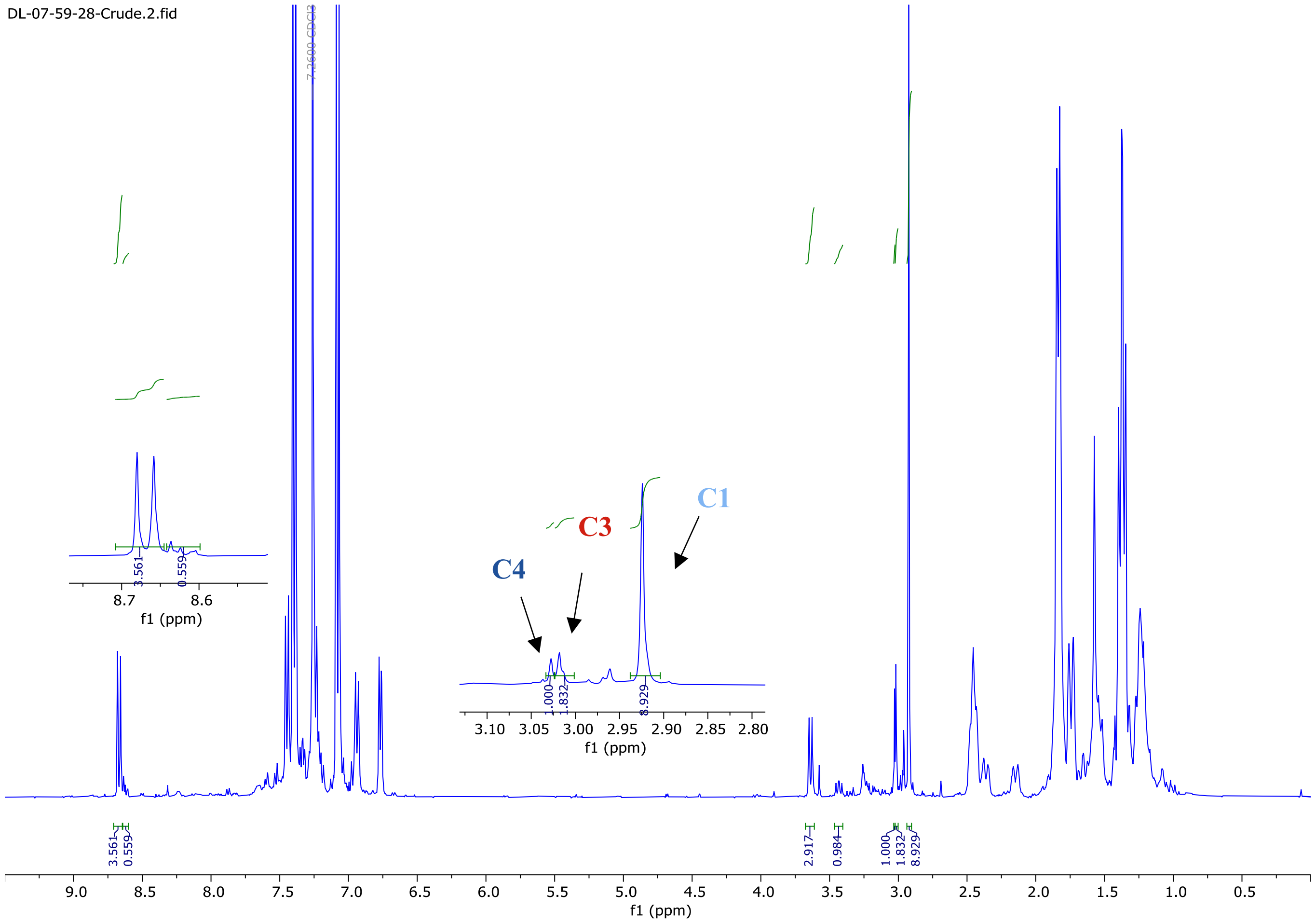

Figure S21. Crude NMR before reduction of entry 12 in Table S5 – Rh$_2$(*S*-*di*-3,5-di-^{t}BuC$_6$H$_4$NTTL)$_4$.

As the resolution of crude NMR is not good enough to determine the ratio of C4:C3 product, we used a combination of NMR with samples that were resolved and purified SFC to determine the response factor between SCF ratio and NMR ratio. (**Table S6**) This response factor was then used to calculate the ratio of C4:C3 product in entries 5-8 of **Table S5**. The SFC traces of the purified product after the reduction steps which are used to determine C4:C3 ratio are shown in **Figure S22**. The assignment of signal is demonstrated in **Figure S9**.

Table S6. Calculated the response factor between NMR and SFC ratio.

Entry from Table S2	C4:C3 (SFC)[a]	C4:C3 (NMR)[b]	Response factor[c]
2	7.0	5.2	1.35
9	9.8	7.4	1.32
10	10.5	7.8	1.34
11	12.2	9.9	1.23
Rh_2(*R*/S-NTTL)$_4$	6.6	4.9	1.35
Average			1.32

[a]C4:C3 ratio was determined by SFC. [b]C4:C3 ratio was determined by crude NMR. [c]Response factor = SFC ratio / NMR ratio

Table S7. Calculated ratio of C4:C3 and SFC ratio.

Entry from Table S2	C4:C3 (SFC)[a]	C4:C3 (calculated)[b]
5	17.0	12.9
6	17.8	13.5
7	35.2	26.7
8	15.0	11.4

[a]C4:C3 ratio was determined by SFC. [b]C4:C3 ratio was determined by dividing C4:C (SFC) ratio by the response factor of 1.32. [c]Response factor = SFC ratio / NMR ratio

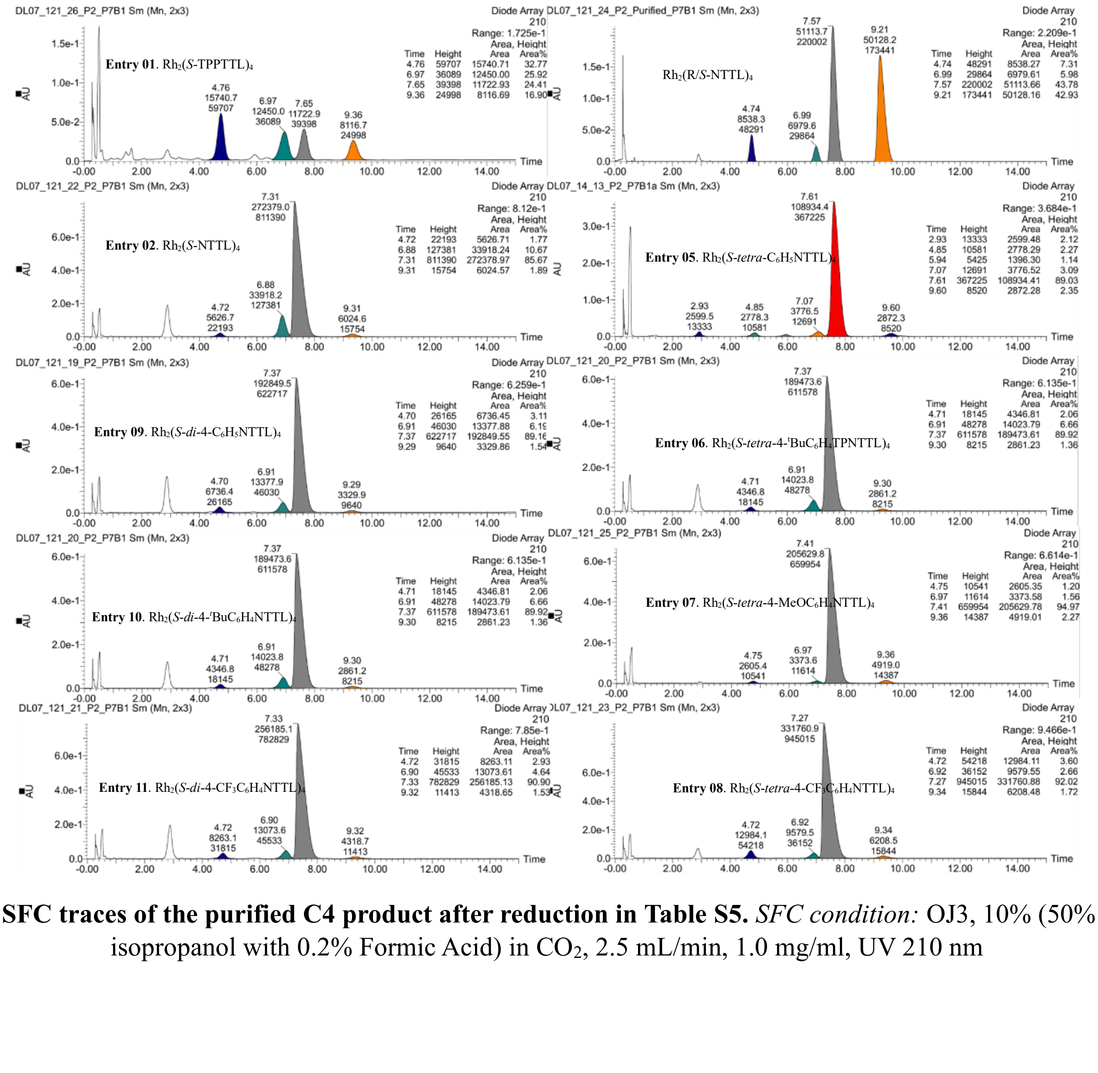

Figure S22. SFC traces of the purified C4 product after reduction in Table S5. *SFC condition:* OJ3, 10% (50% methanol in isopropanol with 0.2% Formic Acid) in CO_2, 2.5 mL/min, 1.0 mg/ml, UV 210 nm

6.2.3. Reaction optimization for electron rich aryl cyclohexane

As we moved from electron-deficient aryl cyclohexane (*p*-bromophenyl cyclohexane) to an electron-rich aryl cyclohexane such as 1-cyclohexyl-4-methylbenzene (**5b**), the selectivity of **7c** drastically decreased. Therefore, 3 more catalysts bearing electron deficient aryl groups including F (**7d**), CO_2Me (**7e**), and CF_3 (**7f**) were synthesized in order to maximize the π interaction between substrate and catalysts. The results with all Rh_2(*S*-*tetra*-Ar-NTTL)$_4$ are shown in **Table S8** below. The results showed that Rh_2(*S*-*tetra*-4-$CF_3C_6H_4$NTTL)$_4$ (**7f**) is the optimum catalyst which gave the highest selectivity for C4 product over C3, C1, and Me functionalized products. **7f** bearing CF_3 groups also gave higher selectivity than **7c** bearing OMe groups in most of electron rich substrate in this study.

Table S8. Reaction optimization with electron-rich aryl cyclohexane and triazole as carbene precursor

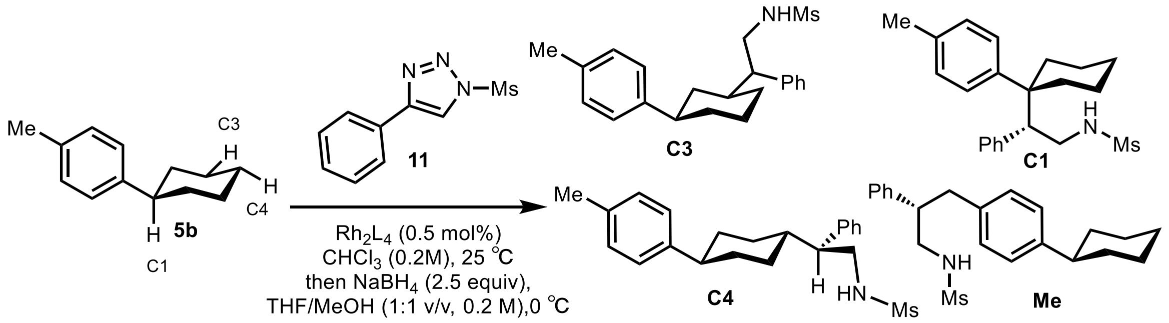

Entry[a]	*Catalyst (0.5 mol%)*	*Yield, %*[b]	(C4:C3)[c]	(C4:C1)[c]	(C4:Me)[c]	C4 *ee ,%*[d]
1	Rh_2(*R/S*-NTTL)$_4$ (**6**)	36	4:1	1.3:1	2.1:1	-
2	Rh_2(*S*-*tetra*-C_6H_5NTTL)$_4$ (**7a**)	79	6.4:1	4.3:1	6.2:1	95
3	Rh_2(*S*-*tetra*-4-^{t}BuC_6H_4NTTL)$_4$ (**7b**)	54	6.9:1	3:1	9.9:1	95
4	Rh_2(*S*-*tetra*-4-MeOC_6H_4NTTL)$_4$ (**7c**)	56	13:1	6.8:1	12.3:1	95
5	Rh_2(*S*-*tetra*-4-$CF_3C_6H_4$NTTL)$_4$ (**7f**)	77	22:1	15.3:1	36:1	95
6	Rh_2(*S*-*tetra*-4-FC_6H_4NTTL)$_4$ (**7d**)	72	16.4:1	9.2:1	15.6:1	95
7	Rh_2(*S*-*tetra*-4-$CO_2MeC_6H_4$NTTL)$_4$ (**7e**)	58	16.7:1	12:1	27:1	96

[a]Reaction conditions: triazole (0.2 mmol, 1 equiv), 1-cyclohexyl-4-methylbenzene (2.5 equiv), Rh_2L_4 (0.5 mol%) in dry $CHCl_3$ (0.2 M) at 25 °C. [b]Yields are isolated yields and reported as a combined yields of C_3 and C_4 products. [c]Regioisomeric ratio was determined by crude ^{1}H-NMR before reduction.[d]Enantiomeric excess of C_4 product was determined by SFC.

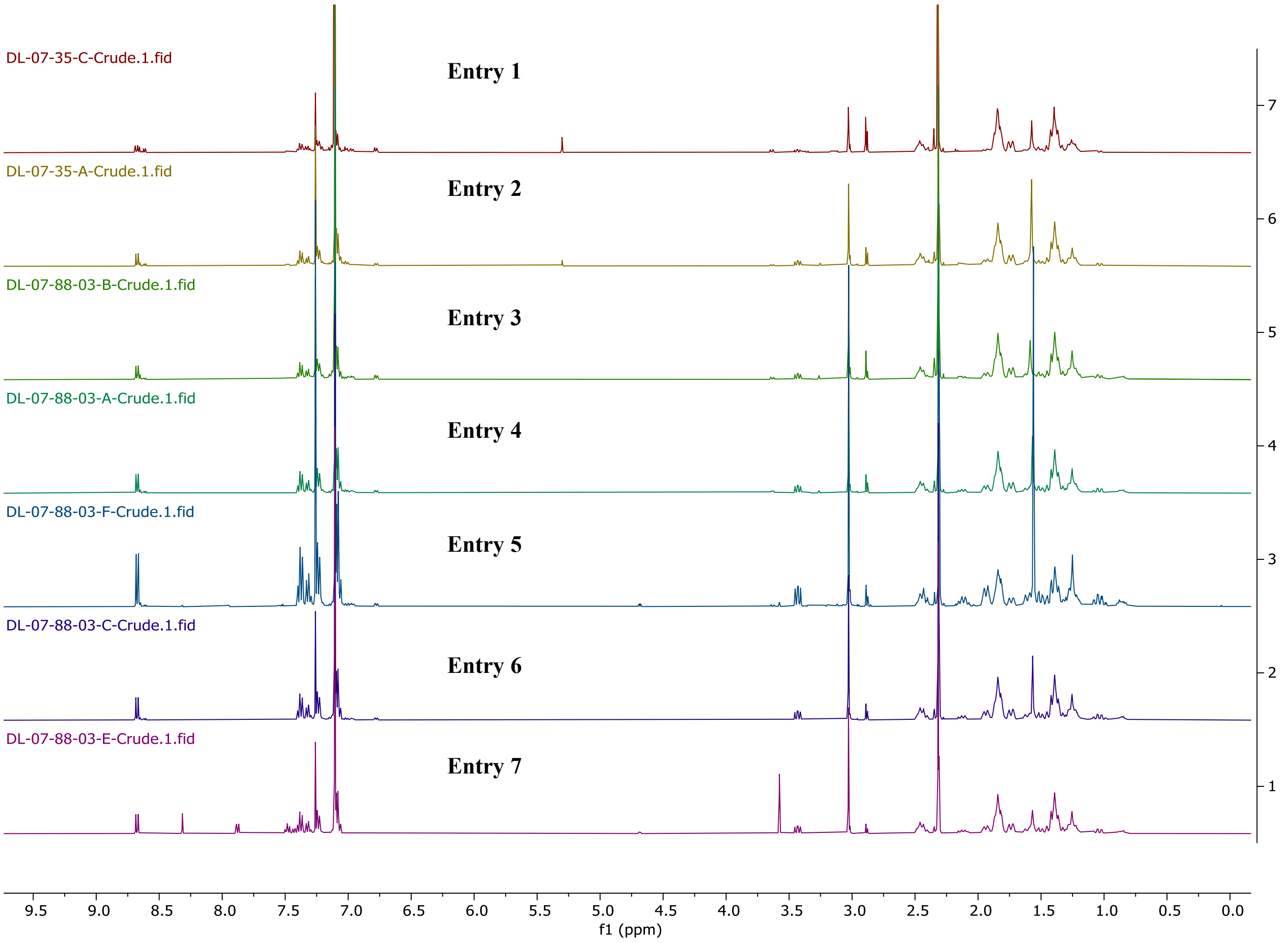

Figure S23. Stack crude NMR of Table S3.

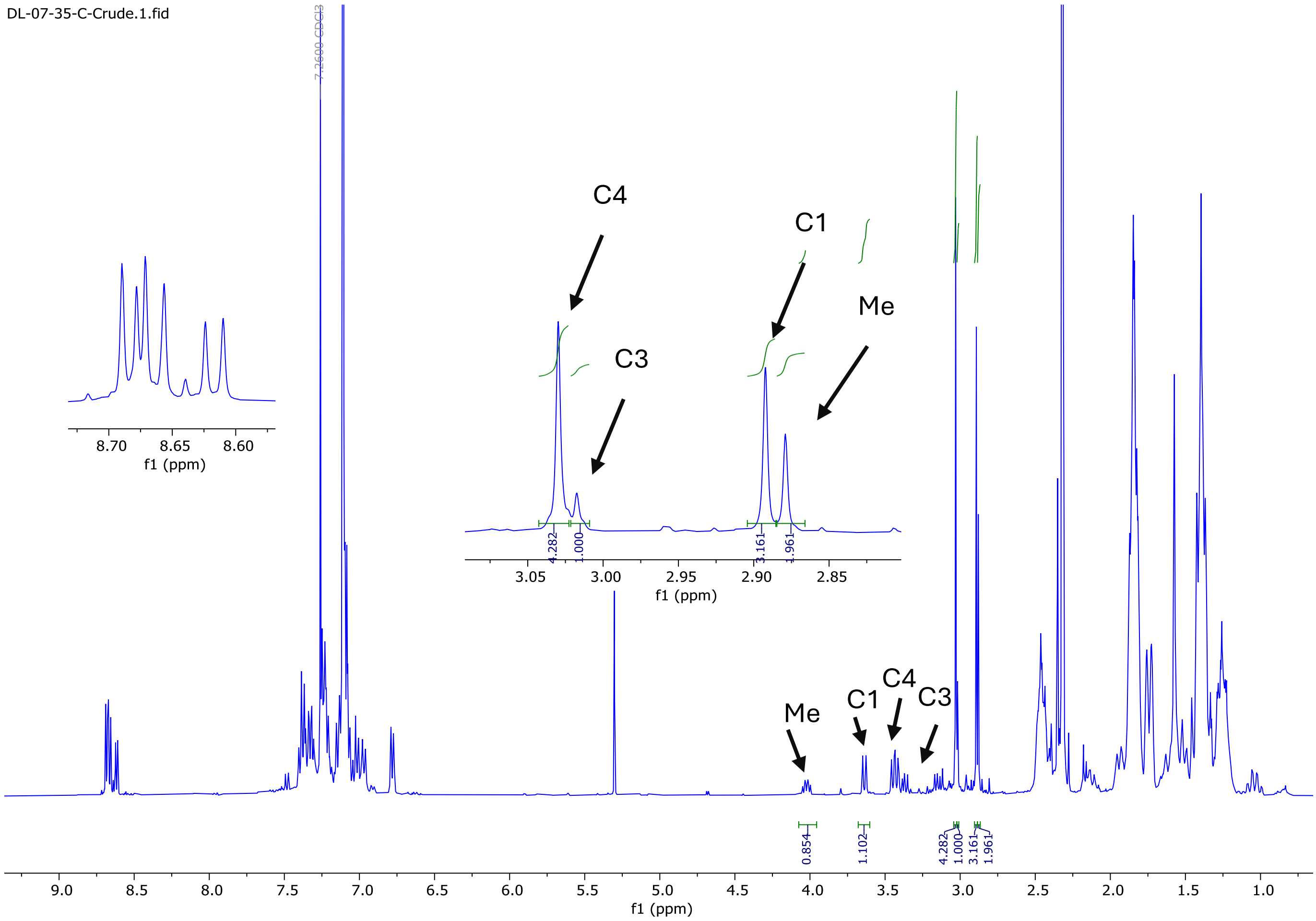

Figure S24. Crude NMR of entry 1 in Table S3 – Rh2(*R*/*S*-NTTL)4.

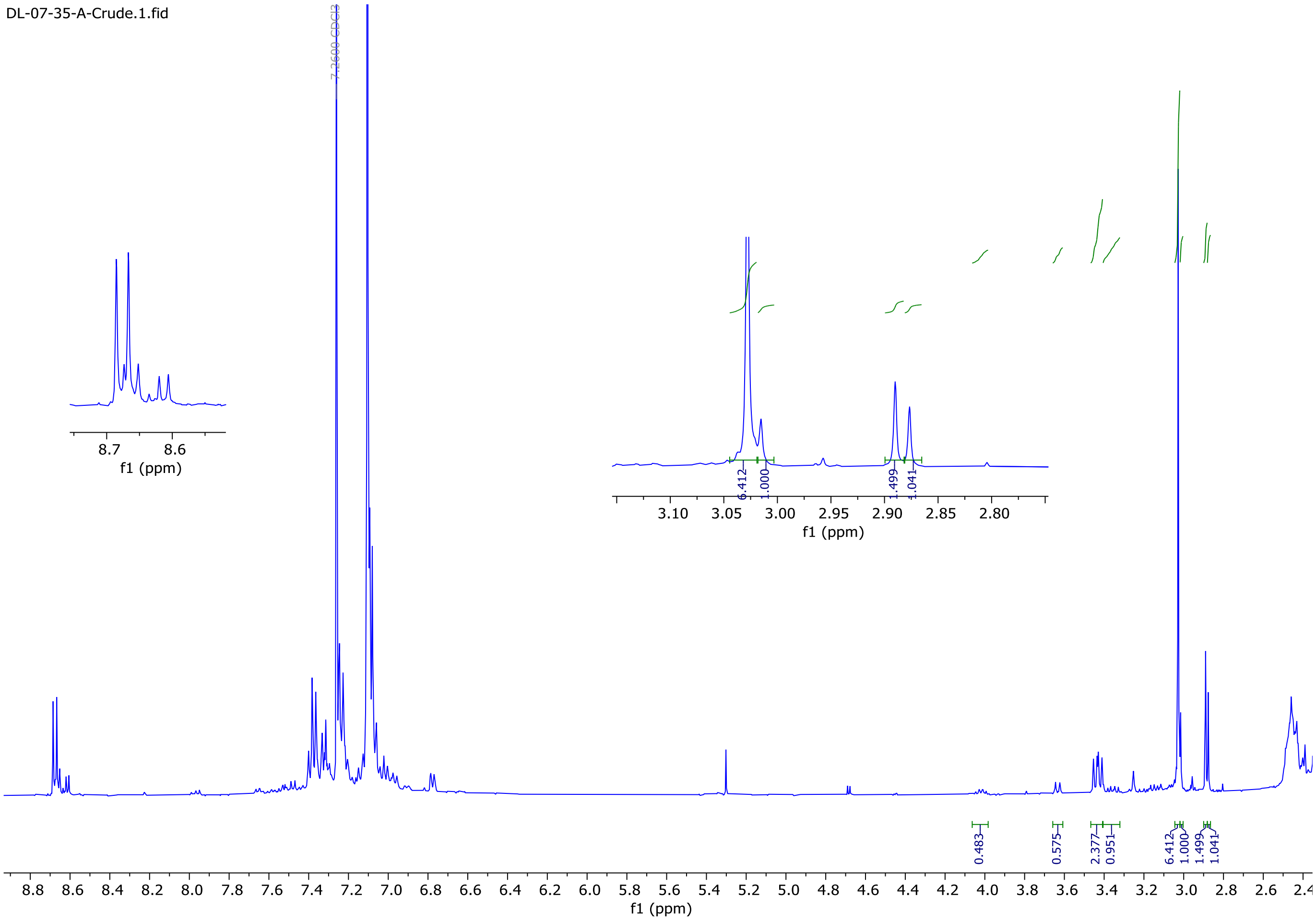

Figure S25. Crude NMR of entry 2 in Table S3 – Rh2(*S*-*tetra*-C6H5NTTL)4.

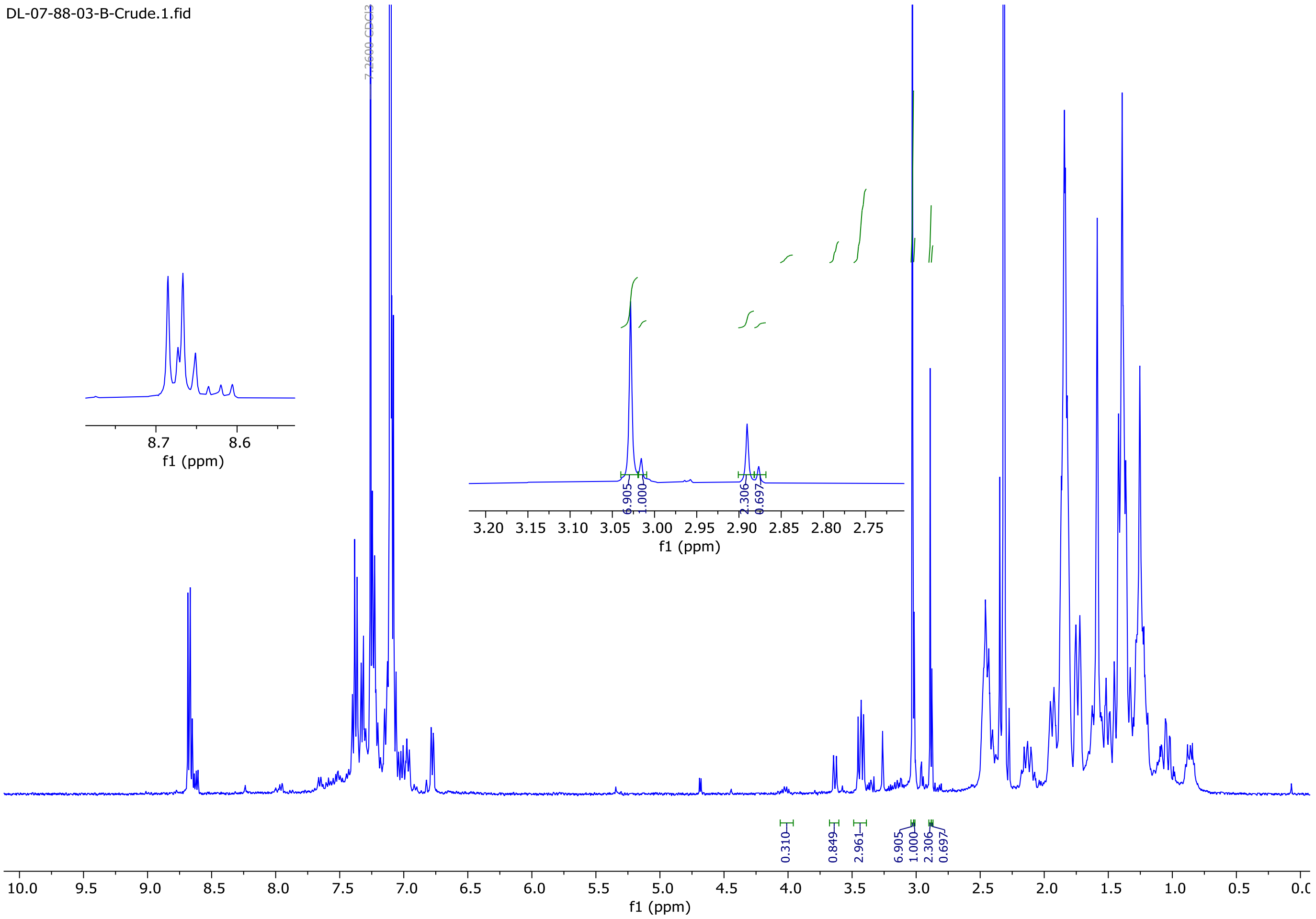

Figure S26. Crude NMR of entry 3 in Table S3 – Rh_2(*S*-*tetra*-4-^{t}BuC$_6$H$_4$NTTL)$_4$.

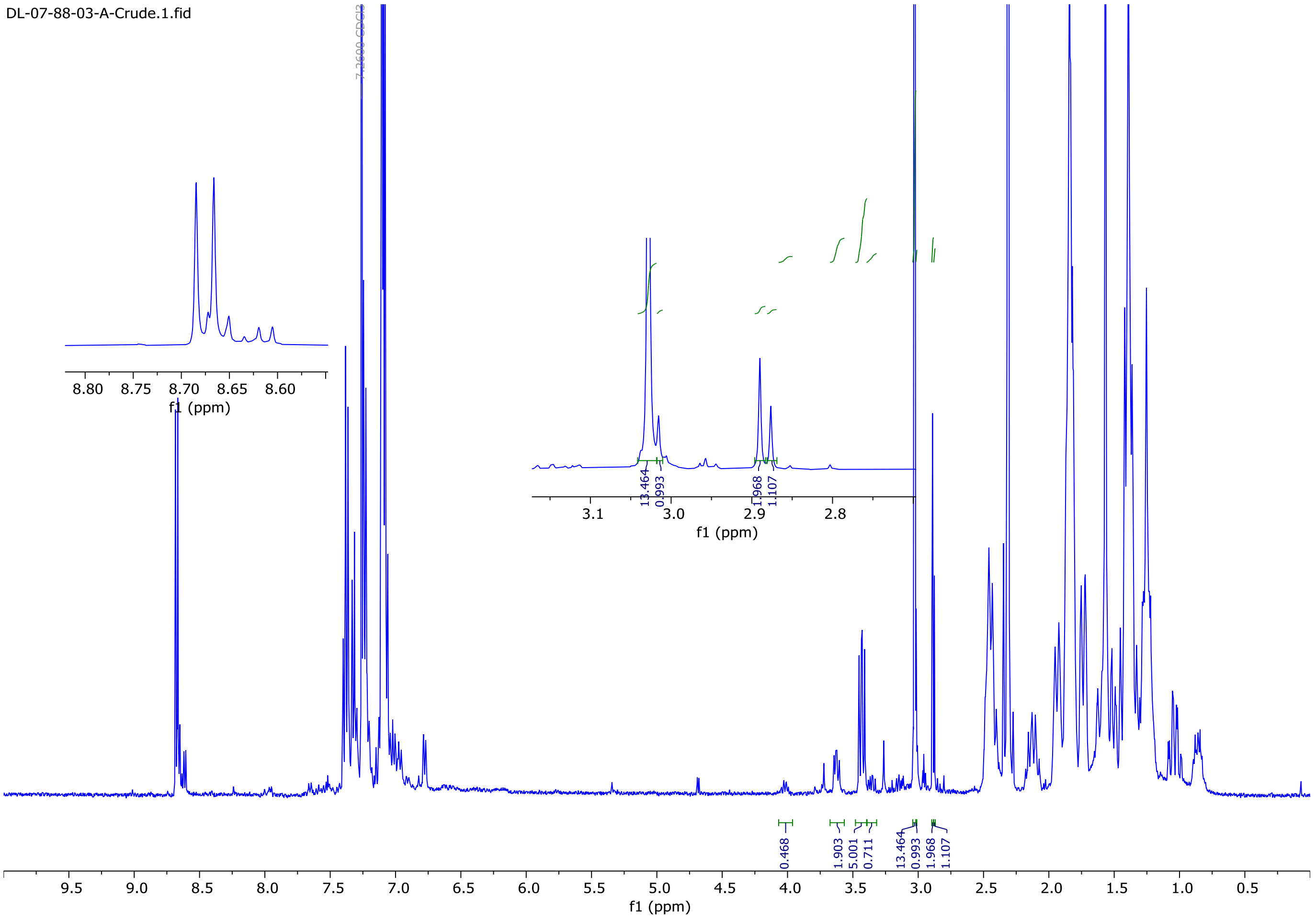

Figure S27. Crude NMR of entry 4 in Table S3 – Rh_2(*S*-*tetra*-4-MeOC$_6$H$_4$NTTL)$_4$.

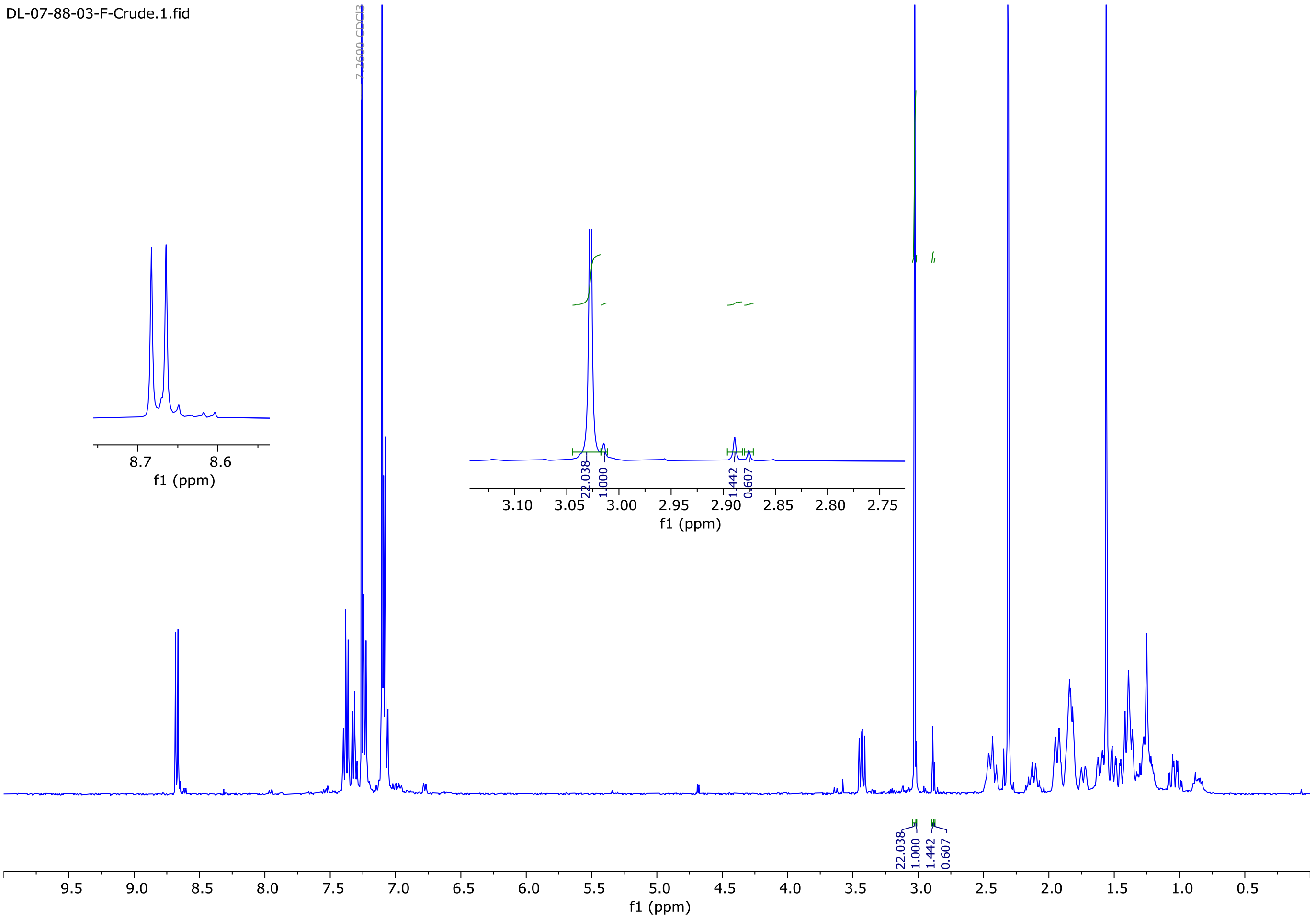

Figure S28. Crude NMR of entry 5 in Table S3 – Rh_2(*S-tetra*-4-$CF_3C_6H_4$NTTL)$_4$.

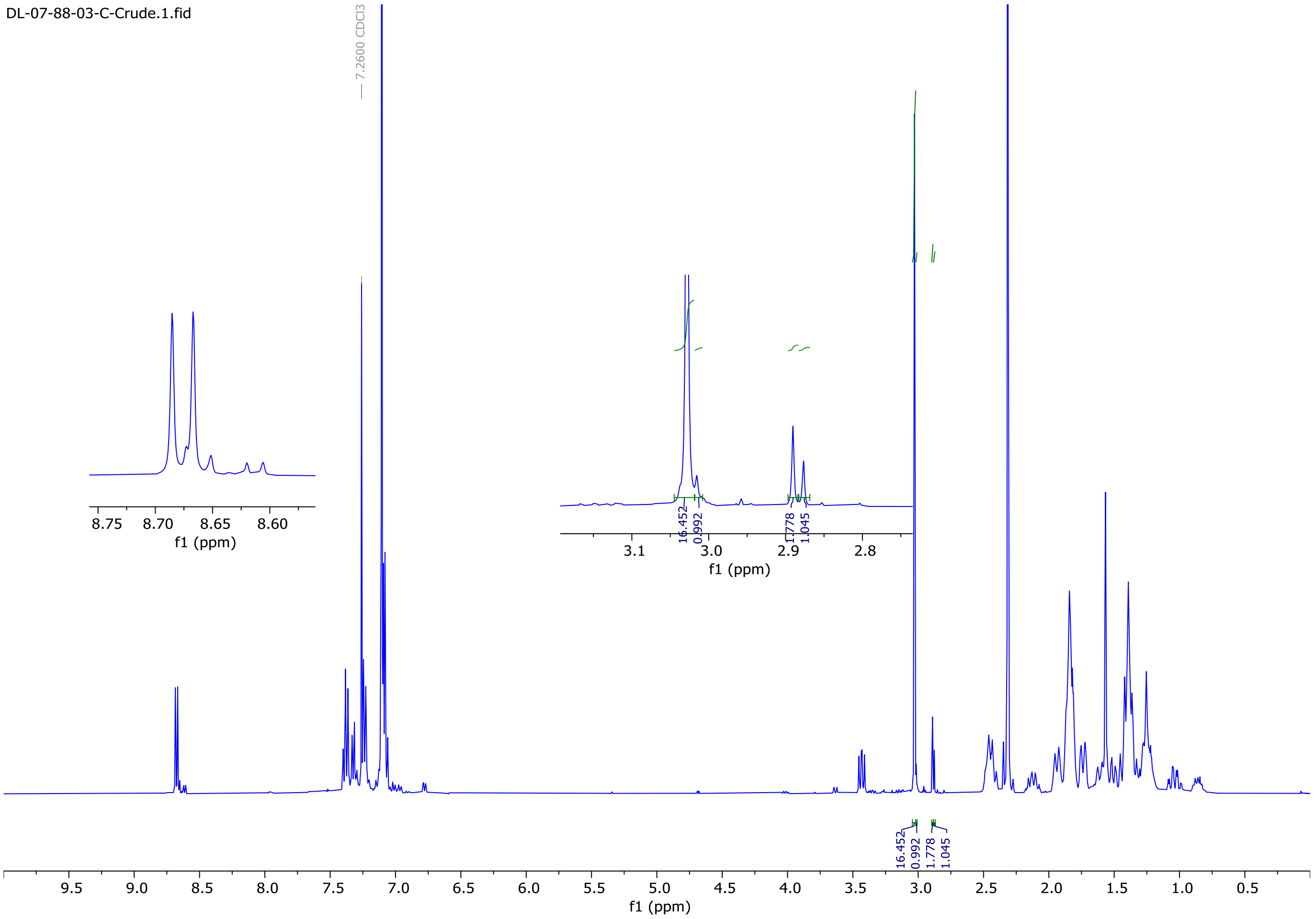

Figure S29. Crude NMR of entry 6 in Table S3 – Rh_2(*S-tetra*-4-FC_6H_4NTTL)$_4$.

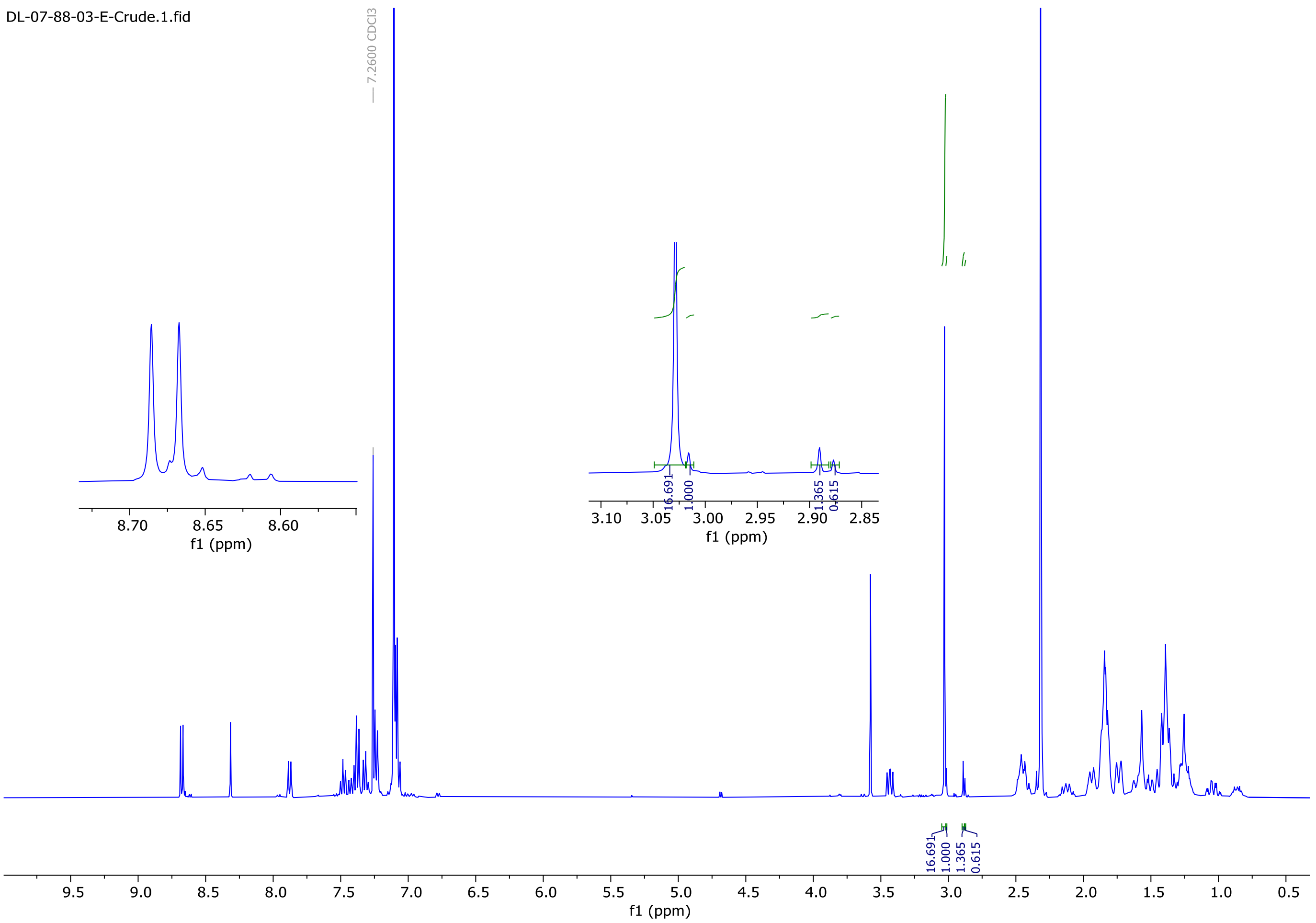

Figure S30. Crude NMR of entry 7 in Table S3 – Rh_2(*S-tetra*-4-$CO_2MeC_6H_4$NTTL)$_4$. *Reaction did not go to full conversion after 24 hours.*

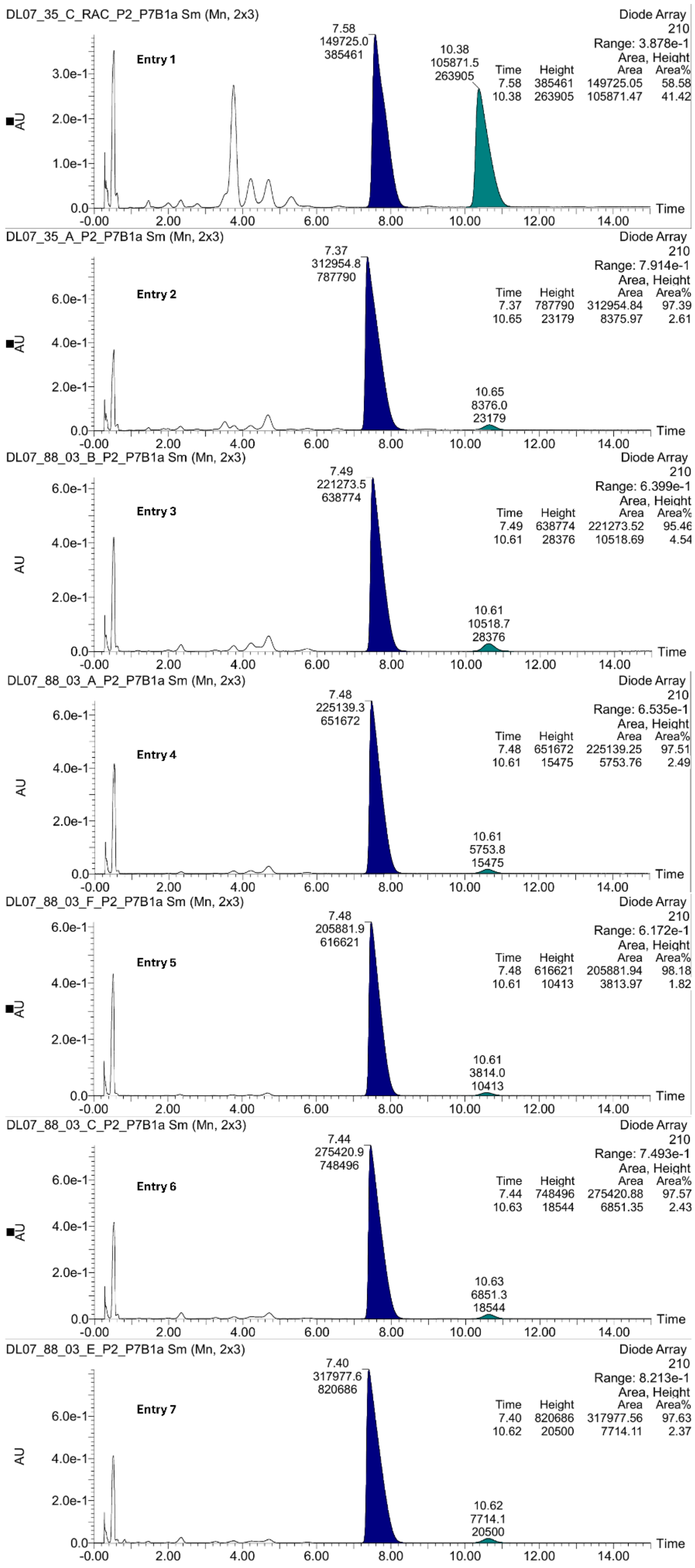

Figure S31. SFC trace for enantiomeric excess (ee) in Table S3. SFC conditions: CEL2, 10% (50% methanol in isopropanol with 0.2% Formic Acid) in CO_2, 2.5 mL/min, 1.0 mg/ml, UV 210 nm

6.3. Scope of the site-selective C-H functionalization

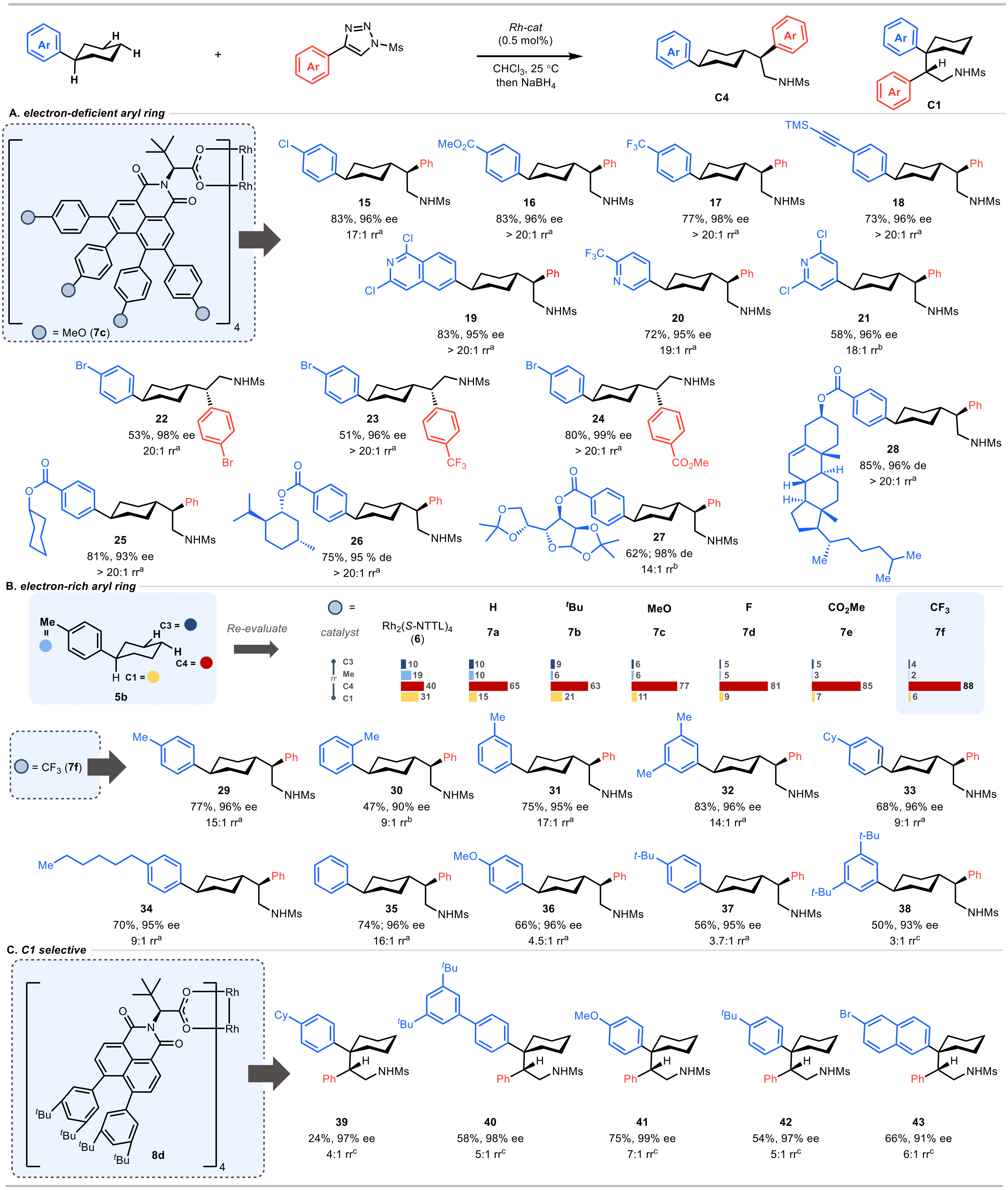

Figure S32. The overall Scope of this study. [a]The regioselectivity (rr) is of C4 product over C1 product with the ratio of C4 over C3 products is > 20:1 rr. [b]The regioselectivity (rr) is of C4 product over C3 products with no observation of C1 product. [c]The regioselectivity (rr) is of the C4 (or C1) product over C3 and C1 (or C4) products.

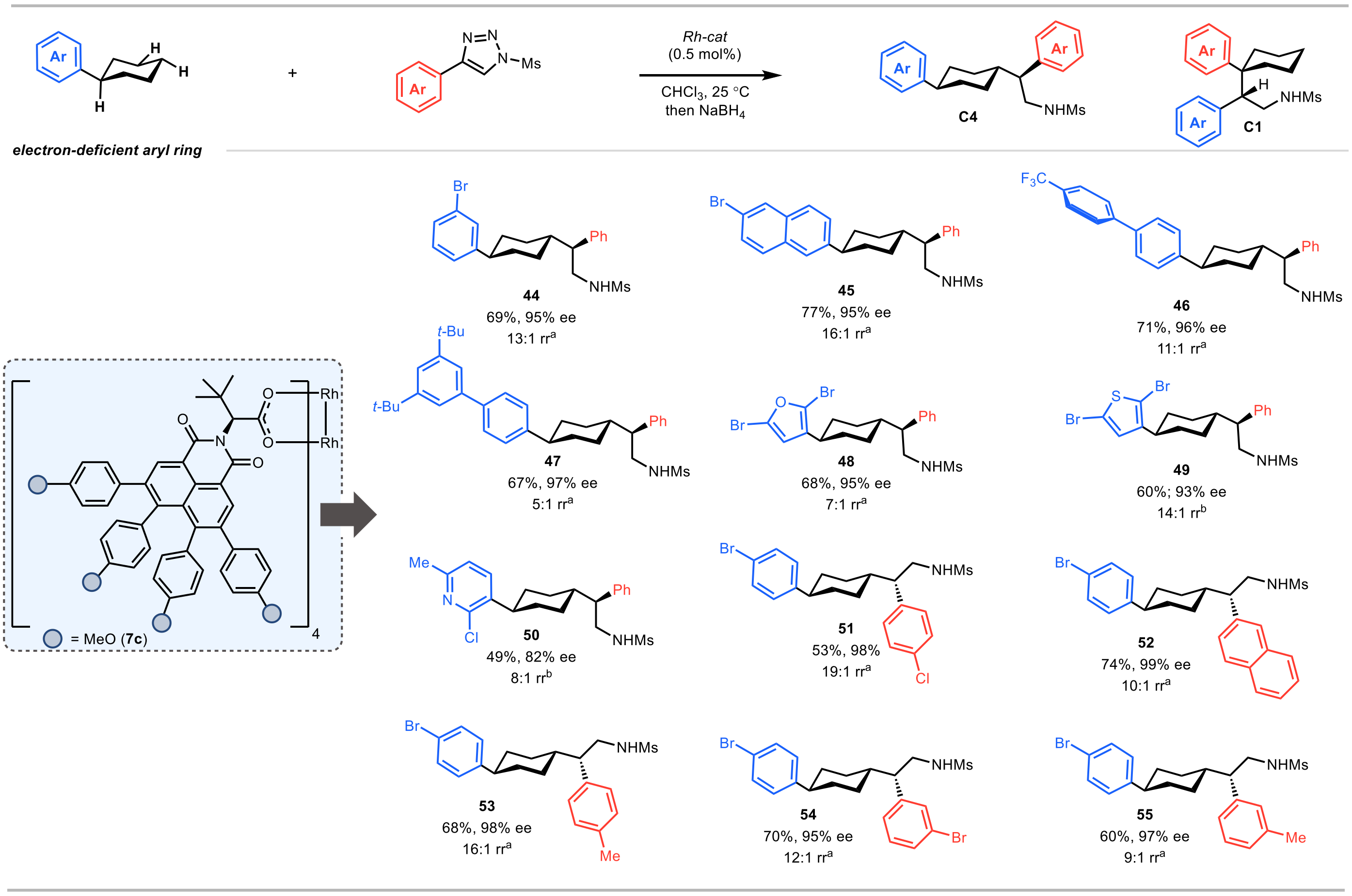

Figure S33. Extra example. This example used Rh_2(*S-tetra*-4-MeO-C_6H_4NTTL)$_4$ (**7c**) as a catalyst. [a]The regioselectivity (rr) is of C4 product over C1 product with the ratio of C4 over C3 products is > 20:1 rr. [b]The regioselectivity (rr) is of C4 product over C3 products with no observation of C1 product.

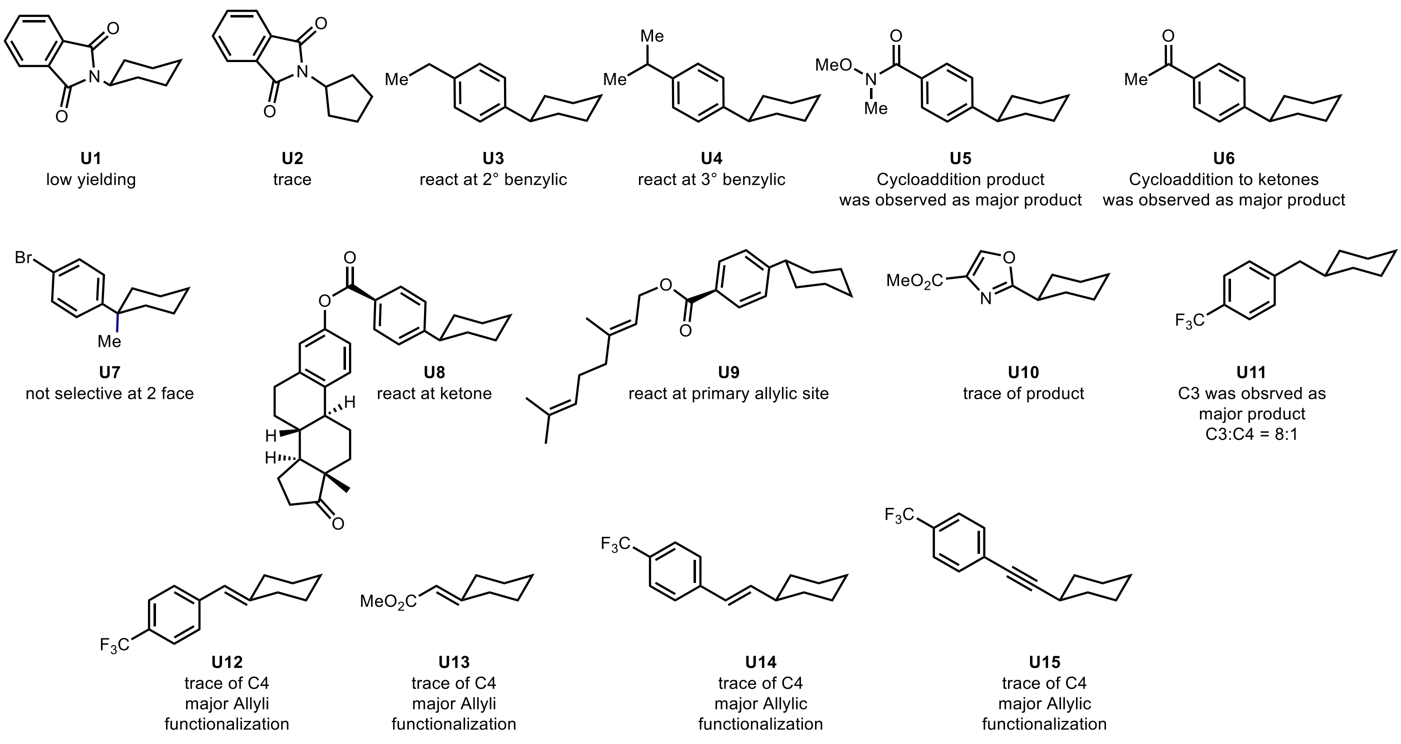

Figure S34. Unsuccessful cyclohexane scope

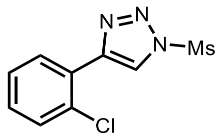

U12
triazole not decomposed

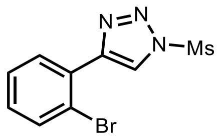

U13
triazole not decomposed

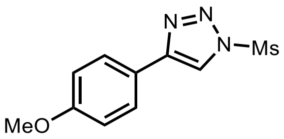

U14
low yielding

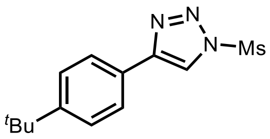

U15
low yielding

Figure S35. Unsuccessful triazole scope

7. Reaction monitoring and thermal dynamic study for C4 selective C-H functionalization

7.1. Reaction monitoring

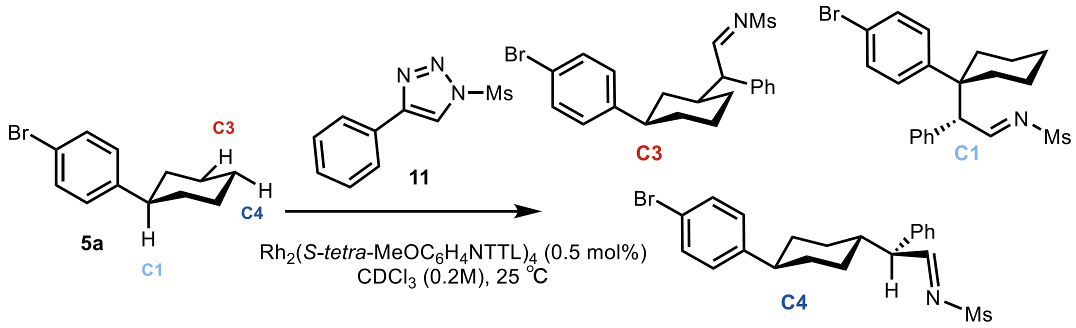

The kinetic study was carried out according to **general procedure H**. To a 1-dram vial was added 1-bromo-4-cyclohexylbenzene (**5a**) (120 mg, 0.5 mmol, 2.5 equiv), Rh$_2$(S-*tetra*-MeOC$_6$H$_4$NTTL)$_4$ (**7c**) (3.0 mg, 0.0001 mmol, 0.005 equiv), and 1-(methylsulfonyl)-4-phenyl-1H-1,2,3-triazole (**11**) (44.7 mg, 0.2 mmol, 1.0 equiv). Then, dried CDCl$_3$ (1.0 ml, 2.0 M) was added. The reaction was stirred until all the solid triazole **11** dissolves. The reaction mixture was then transferred to an NMR tube by cannular under N$_2$. The reaction mixture was then monitored by ^{1}H-NMR with 1 hour apart between each scan. The reaction seems to be sluggish under no stirring condition in the NMR tube. A detailed kinetic study of this reaction is a subject of future study.

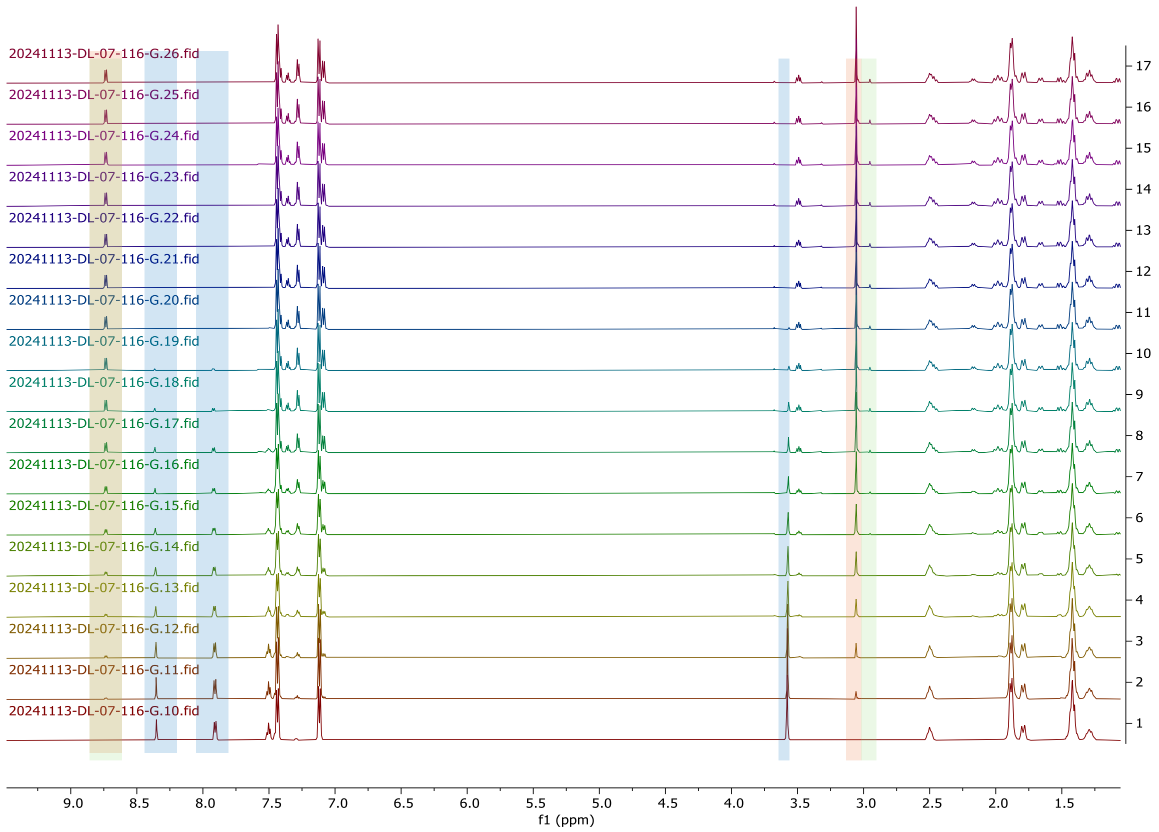

Figure S36. Reaction monitoring by ^{1}H-NMR every 1 hour. Blue: triazole **11**; Red: C4 product before reduction; Green: C1 product before reduction

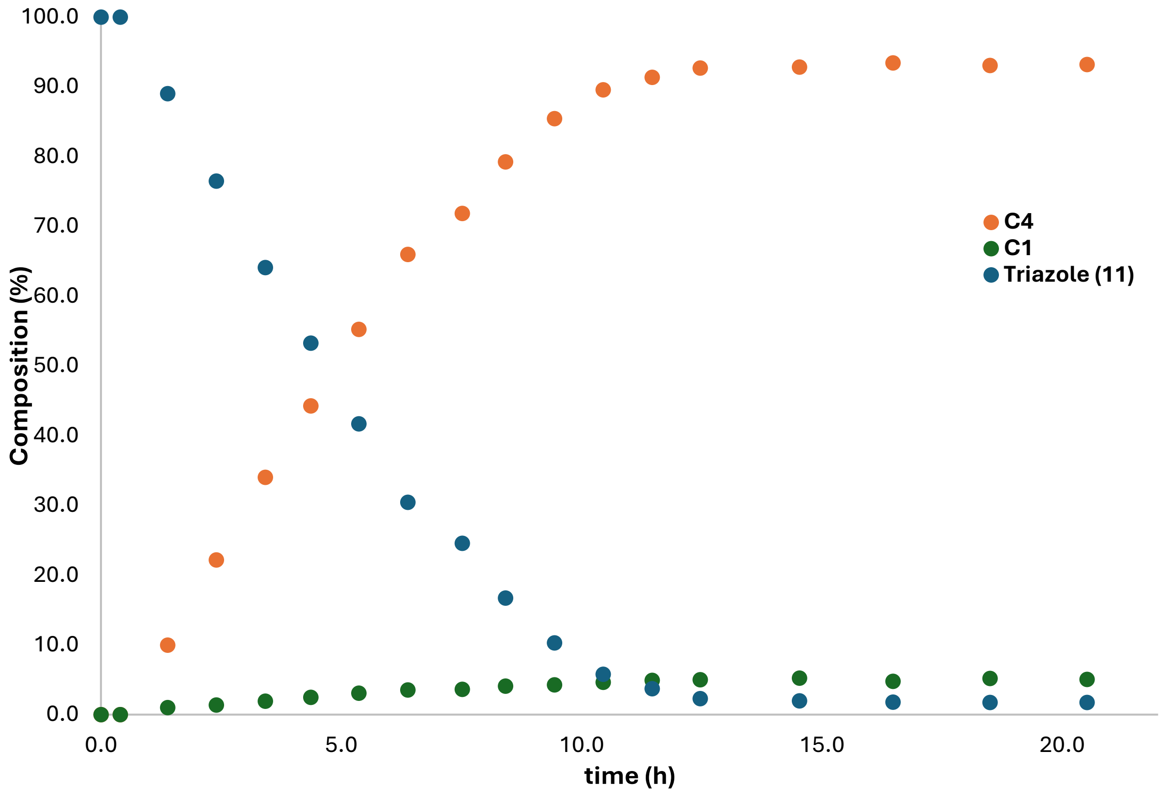

Figure S37. A composition of triazole and products over time.

7.2. Thermal dynamic study

In order to gain a better understanding of the thermodynamic aspects of the C4 selective C-H functionalization. We studied the reaction at different temperatures and applied the Eyring equation to gain thermodynamic value. It is noteworthy that in this analysis when we discussed the regioselectivity between C4, C3, and C1 in the calculation, both R and S products were included in the calculation. So, the energy obtained herein is an average of R and S pathways.

The results are illustrated in **Table S9, Figure S38,** and **Table S10**. The pathways leading to product C4 are enthalpically favorable compared to C1, C3, and enantiomer of C4 products, suggesting that there is a stabilized interaction in transition state leading to C4 product. On the other hand, the entropy aspect of C4 pathways is always disfavored compared to the other pathway, implying that the structure leading to C4 product is less disordered compared to the other pathways.

Eyring equation in which the $\Delta\Delta H^{\#}$ and $\Delta\Delta S^{\#}$ are the enthalpy and entropy difference between the transition states leading to the major C4 product and its enantiomer, C3 product, or C1 product, respectively.

$$\ln\left(\frac{8bS}{8bS'}\right)\ or\ \ln\left(\frac{8bS}{7bS}\right)\ or\ \ln\left(\frac{8bS}{8b'S}\right) = -\frac{\Delta\Delta H^{\#}}{RT} + \frac{\Delta\Delta S^{\#}}{R}$$

Table S9. The effect of temperature on selectivity including regioselectivity and enantioselectivity

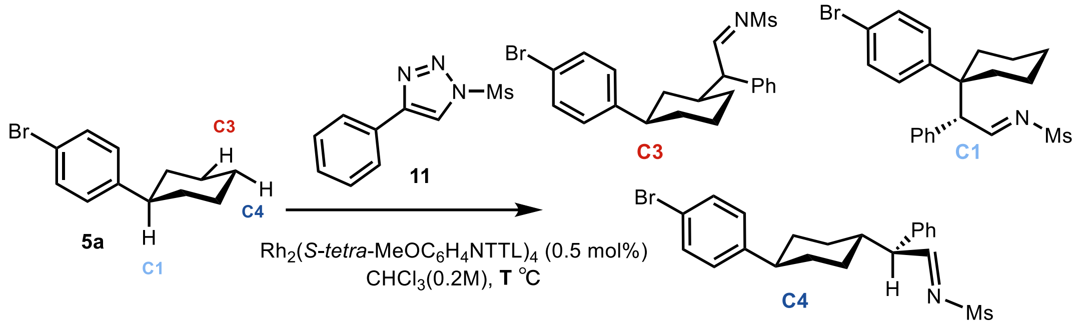

Entry	T	C4:C1[a]	C4:C3[b]	ee of C4[b]
01	25	18.5	26.7	95.3
02	35	15.7	25.2	94.7
03	40	14.3	17.8	93.6
04	50	12.7	15.7	92.6
05	60	10.6	12.4	91.0

The reactions were carried out according to general procedure H. [a]13S:14S was determined by crude ^{1}H-NMR. [b] Ratio was determined by SFC.

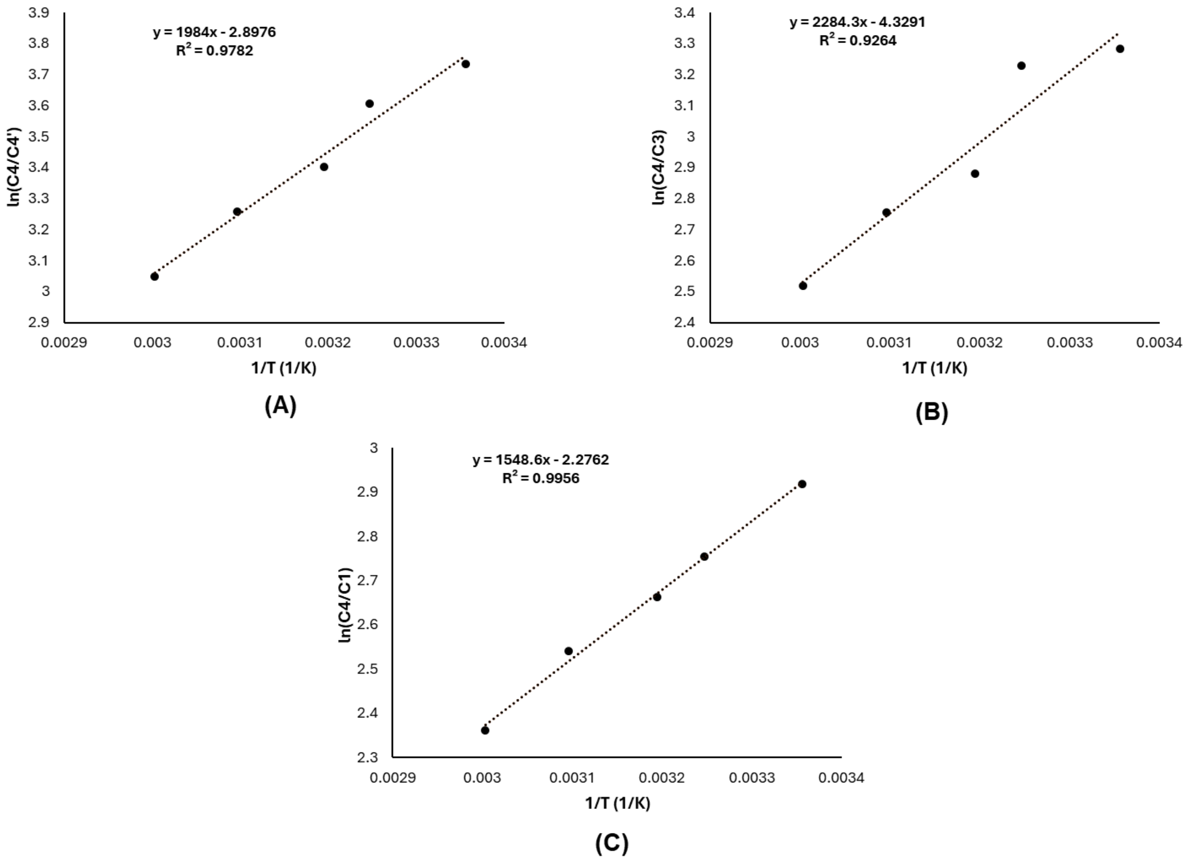

Figure S38. Eyring plots from Table S9. (A) The plot between C4 product and its enantiomer. (B) The plot between C4 product (both R and S) and C3 product (both R and S). (C) The plot between C4 product (both R and S) and C1 product (both R and S).

Table S10. Summary of thermodynamic values

	$\Delta\Delta H^{\#}$ (kcal/mol)	$-T\Delta\Delta S^{\#}$ (kcal/mol)	$\Delta\Delta G^{\#}$ (kcal/mol)
C4' compared to C4	3.95	-1.72	2.23
C3 compared to C4	4.54	-2.56	1.98
C1 compared to C4	3.08	-1.34	1.73

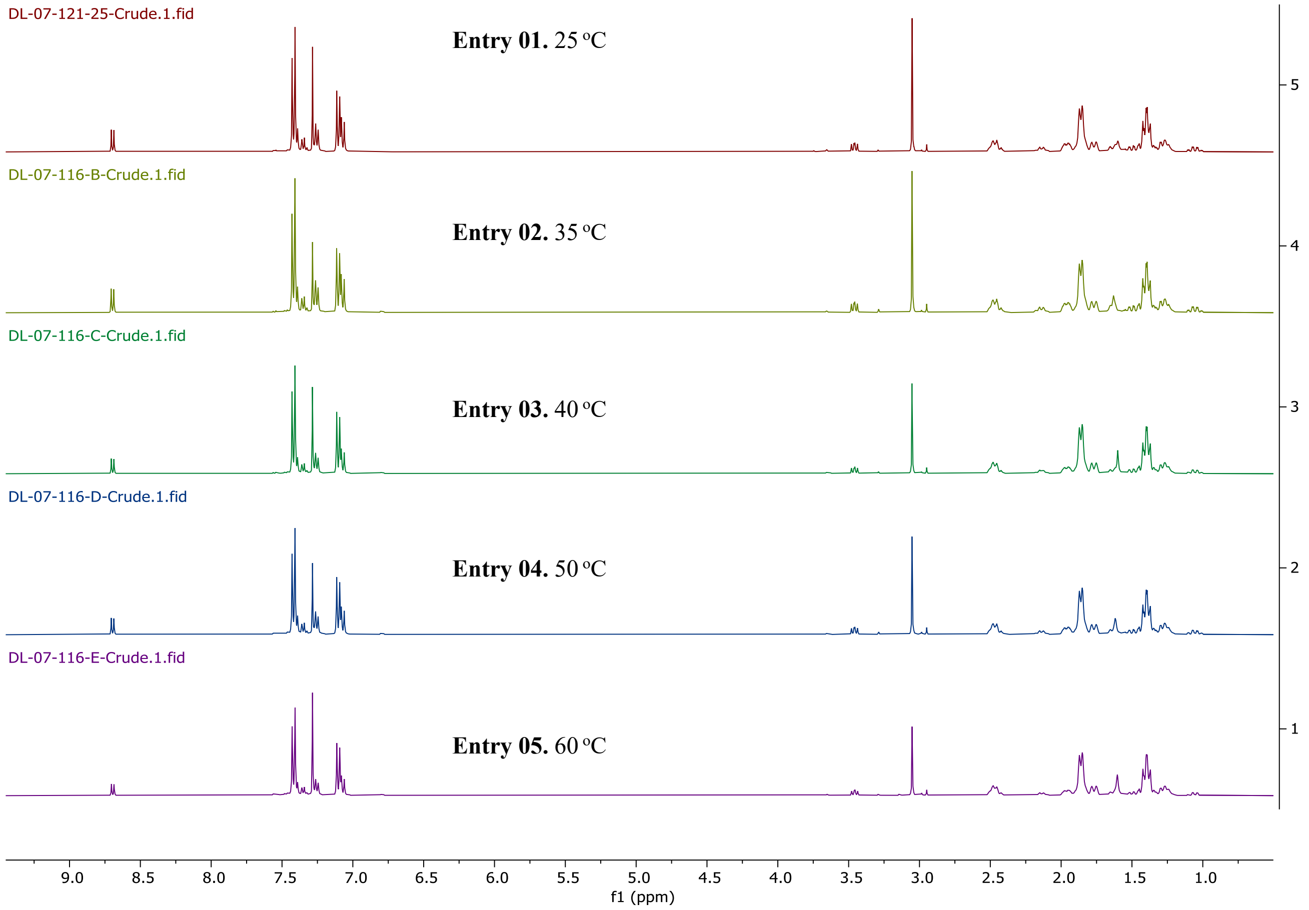

Figure S39. Crude ^{1}H-NMR of temperature effect study

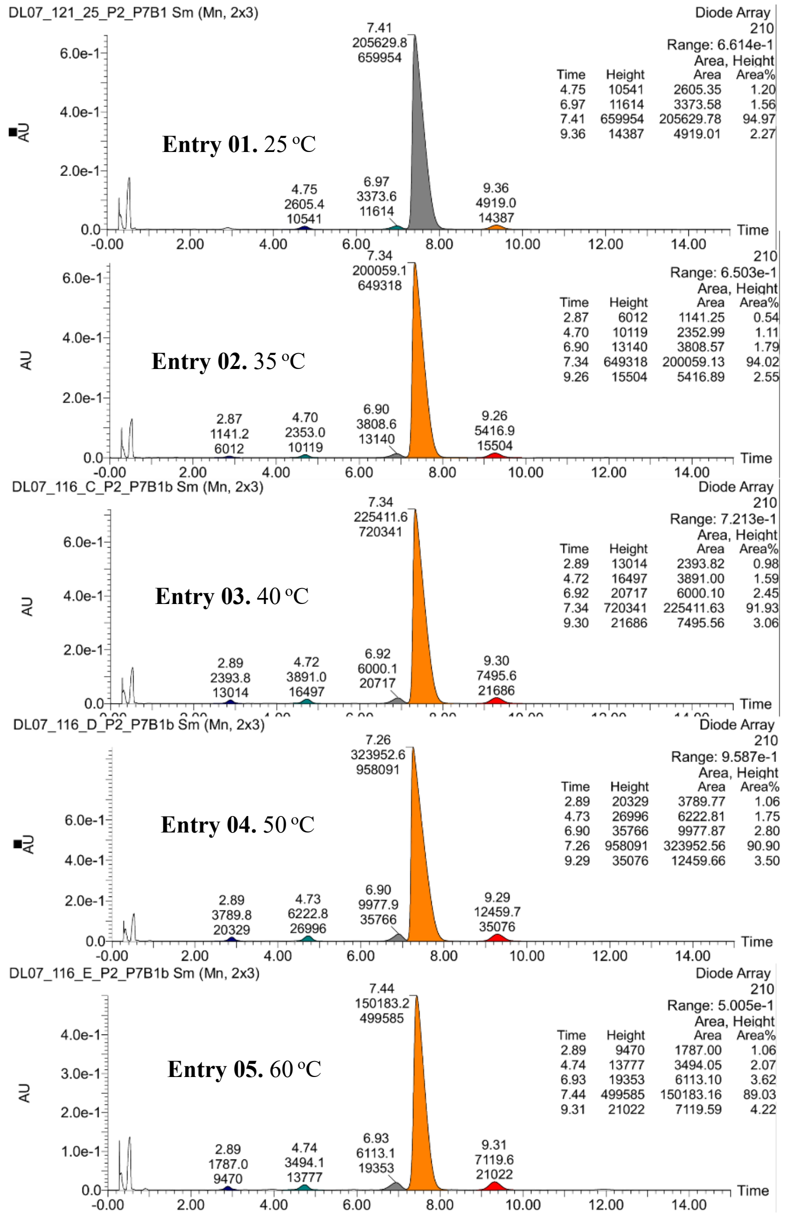

Figure S40. SFC trace of temperature effect study

8. Crude NMR for regio-selectivity determination of C-H functionalization

8.1. Crude NMR regio-selectivity determination of C4 selective C-H functionalization

Note: *The assignment of C3, C1, and C4 based on characteristics signal of the methyl of the mesylate and the H of imines. The assignment was also based on the unselective reaction of Rh₂(S-NTTL)₄ to identify C3 and C1.*

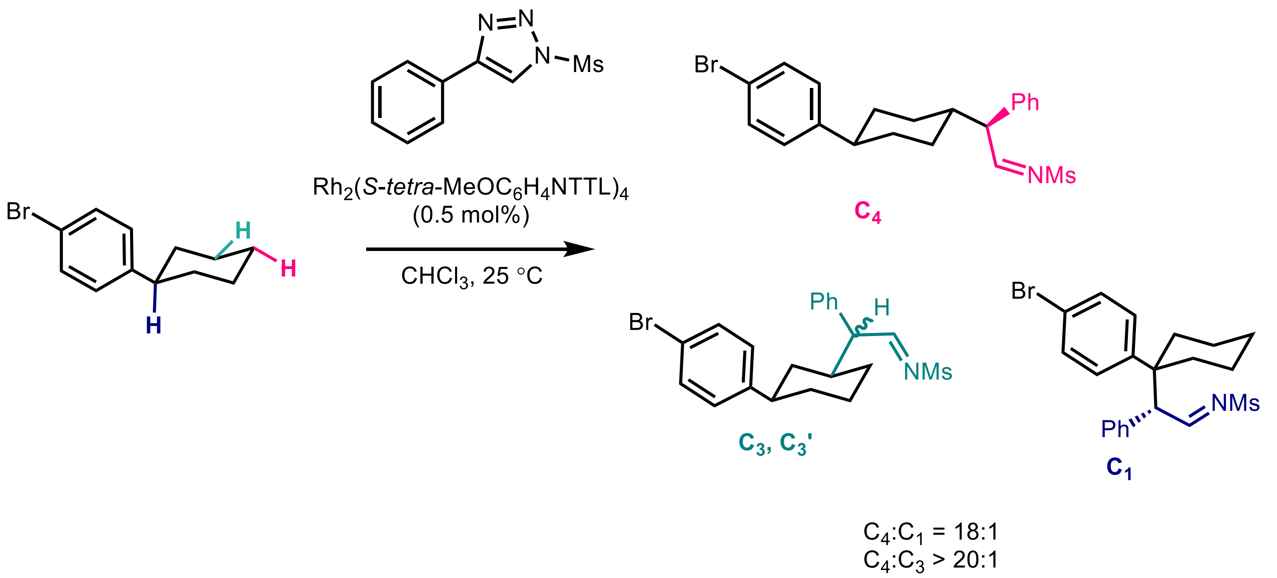

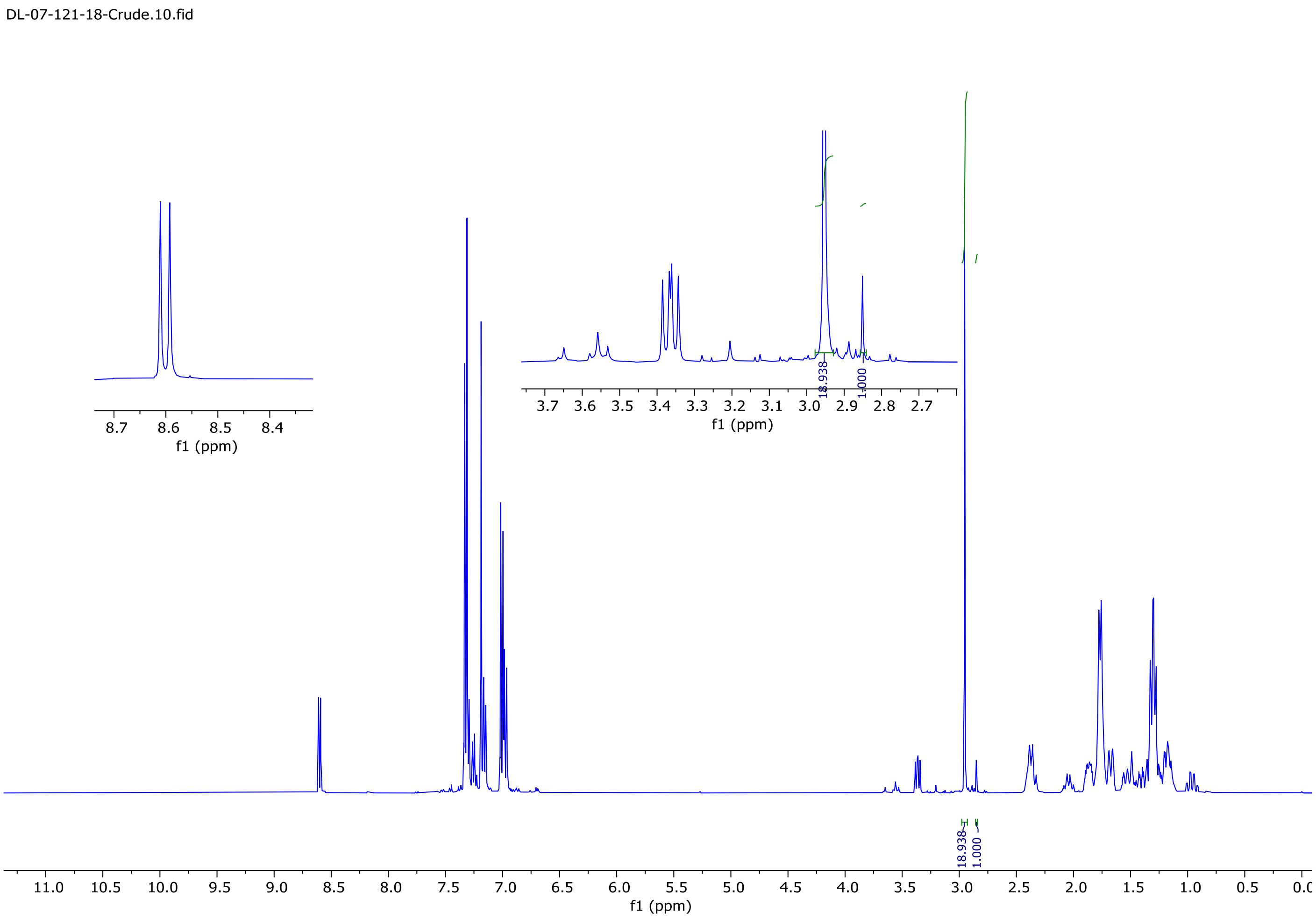

Figure S41. Crude ^{1}H-NMR before reduction of 13

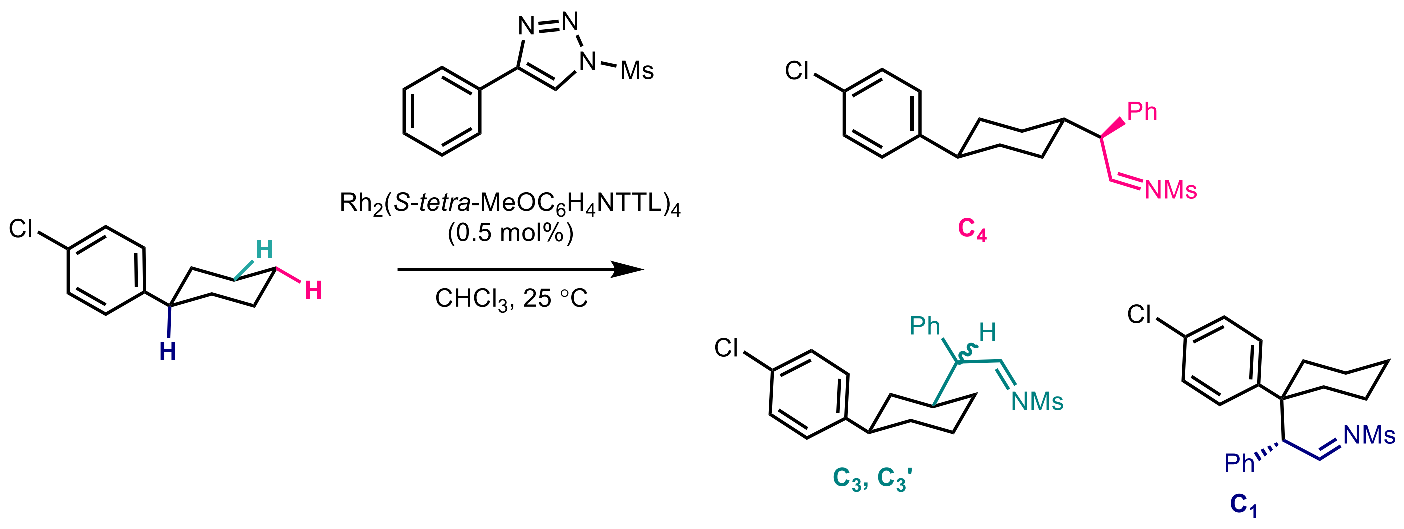

C_4:C_1 = 17:1
C_4:C_3 > 20:1

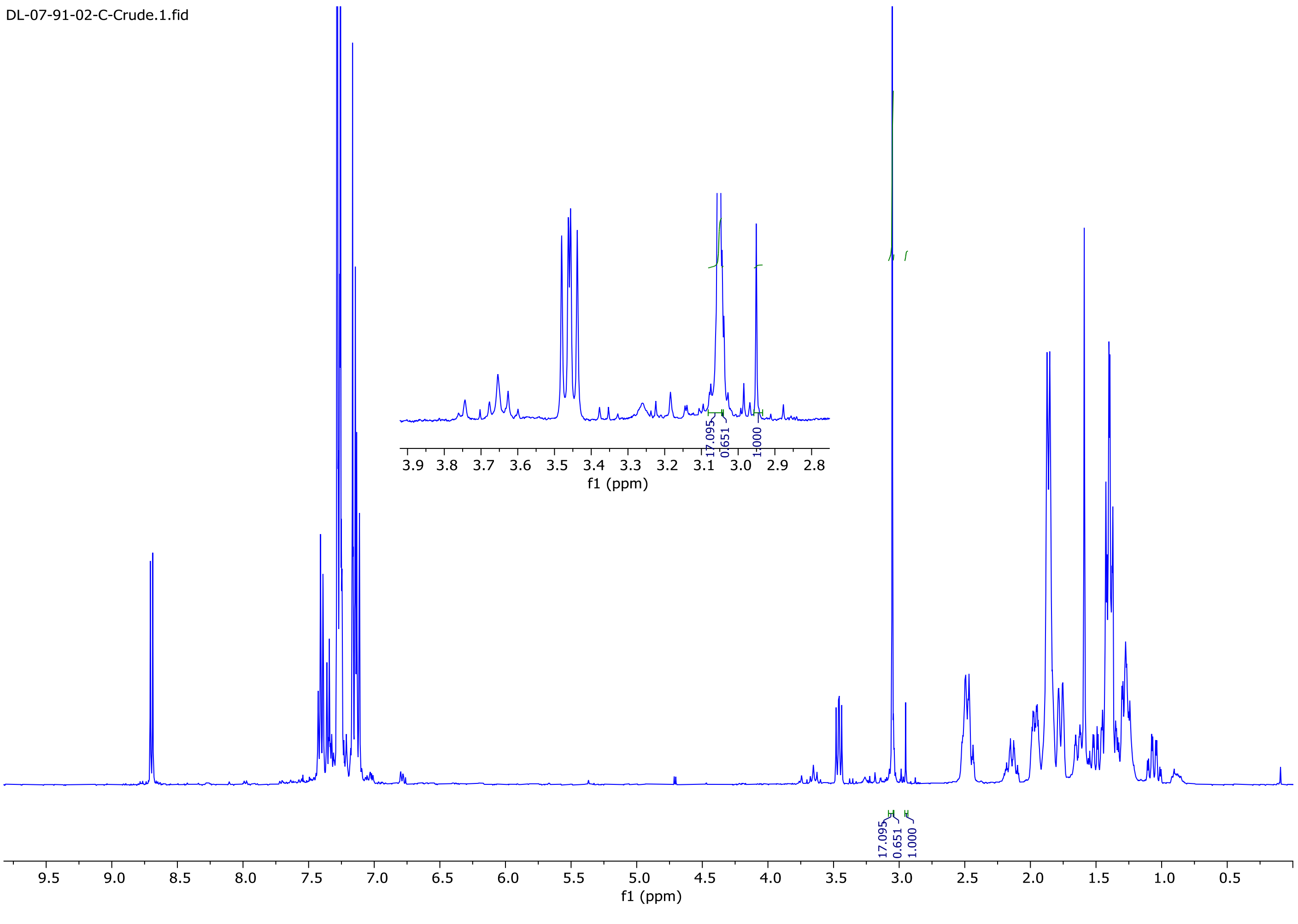

Figure S42. Crude ^{1}H-NMR before reduction of 15

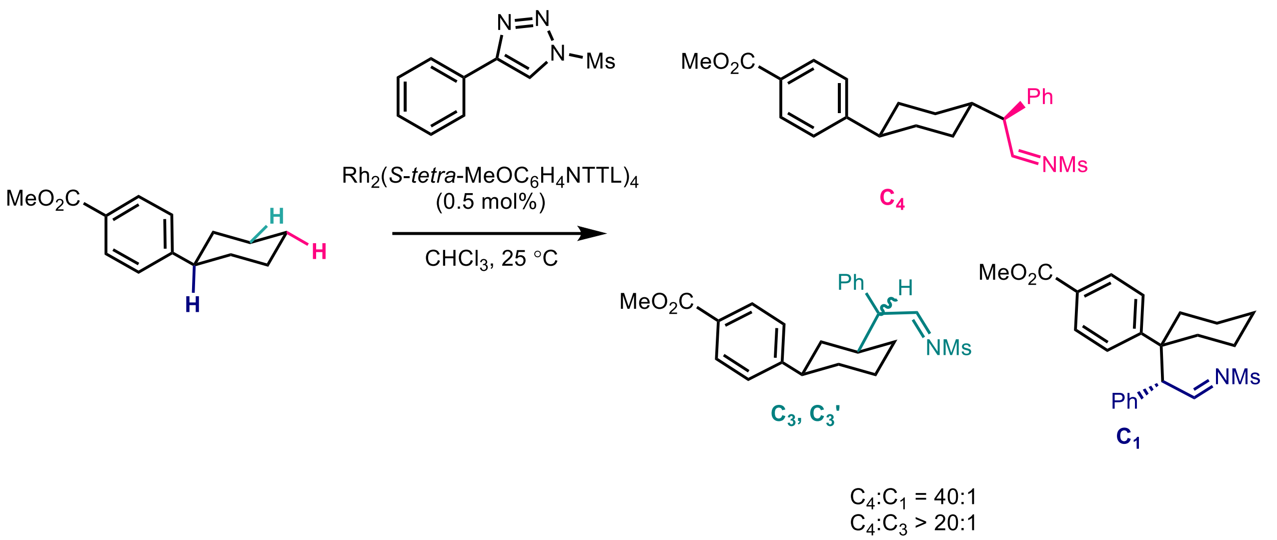

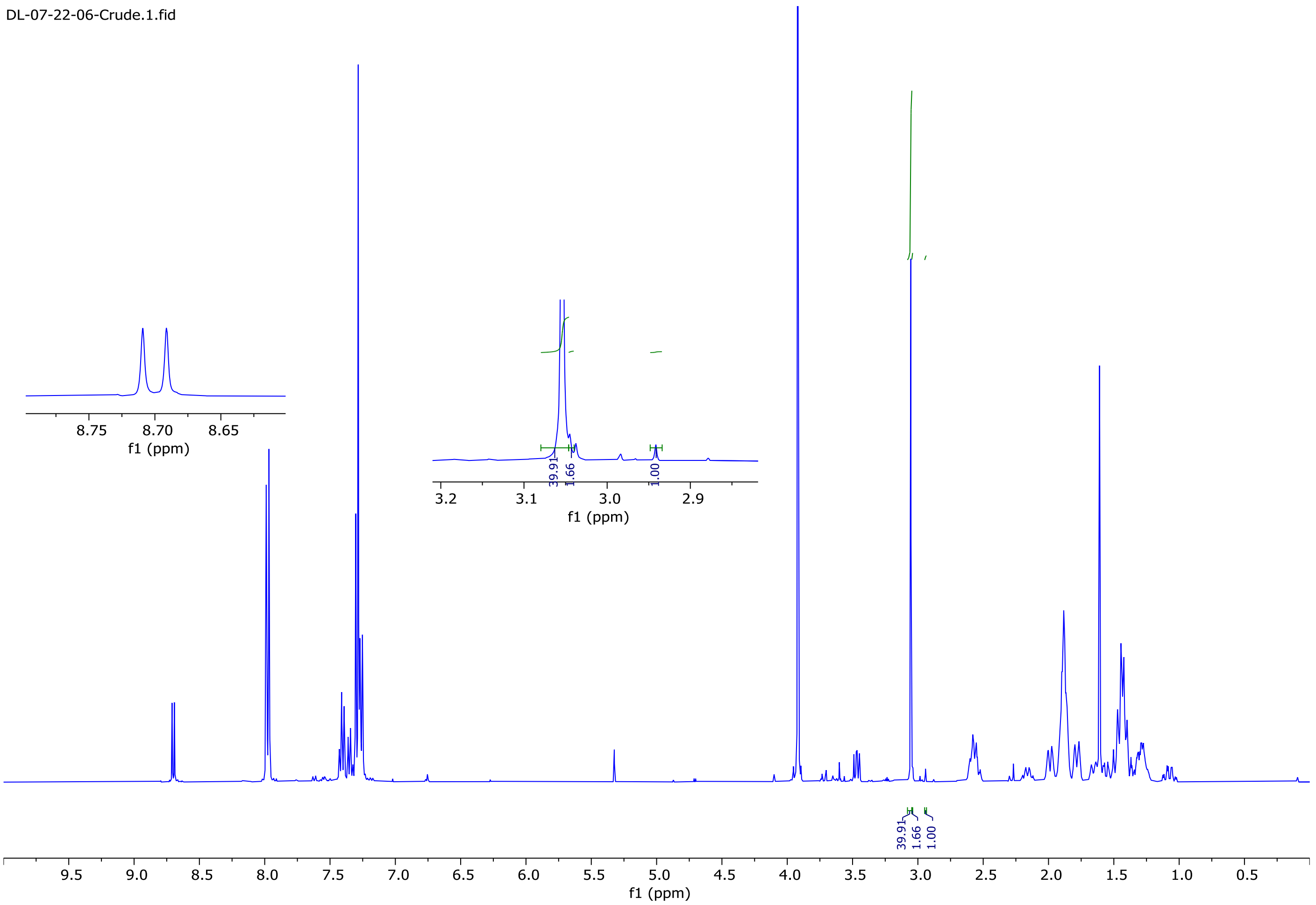

Figure S43. Crude ^{1}H-NMR before reduction of 16

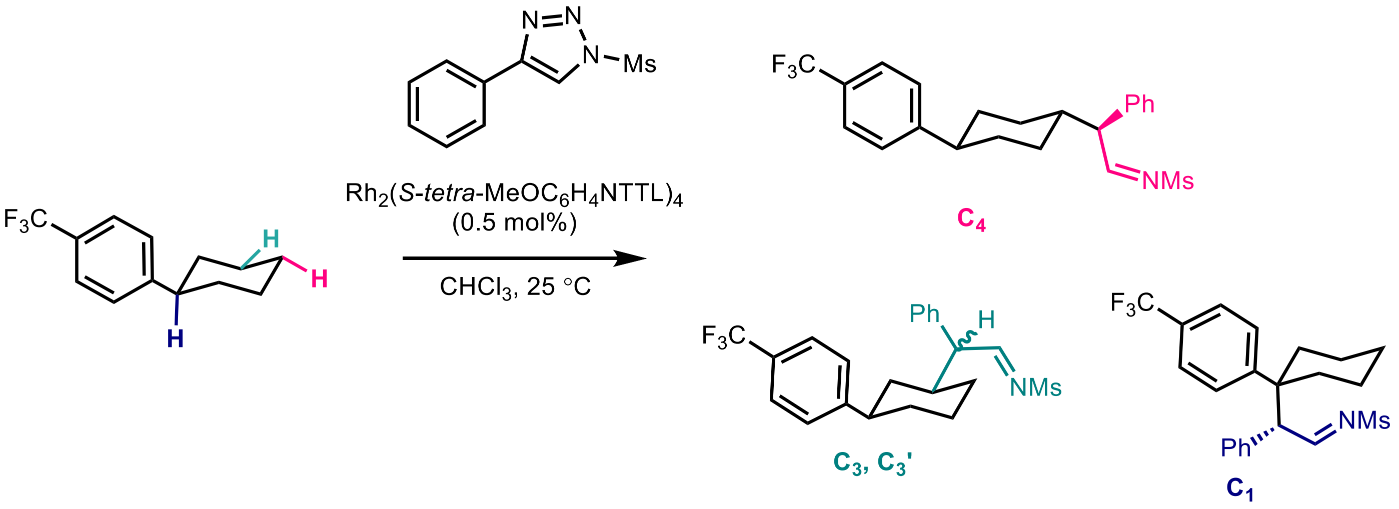

C_4:C_1 = 38:1
C_4:C_3 > 20:1

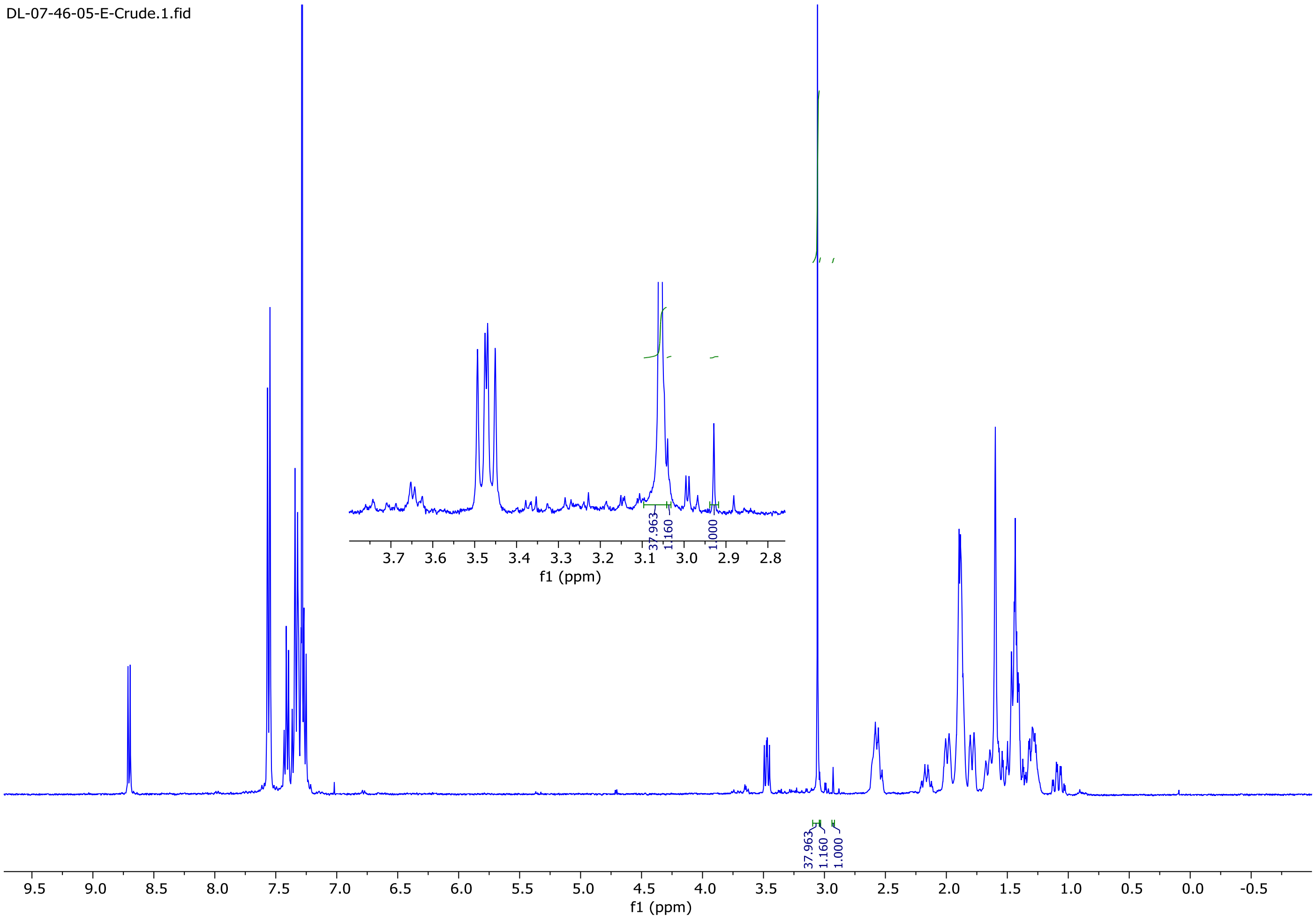

Figure S44. Crude ¹H-NMR before reduction of 17

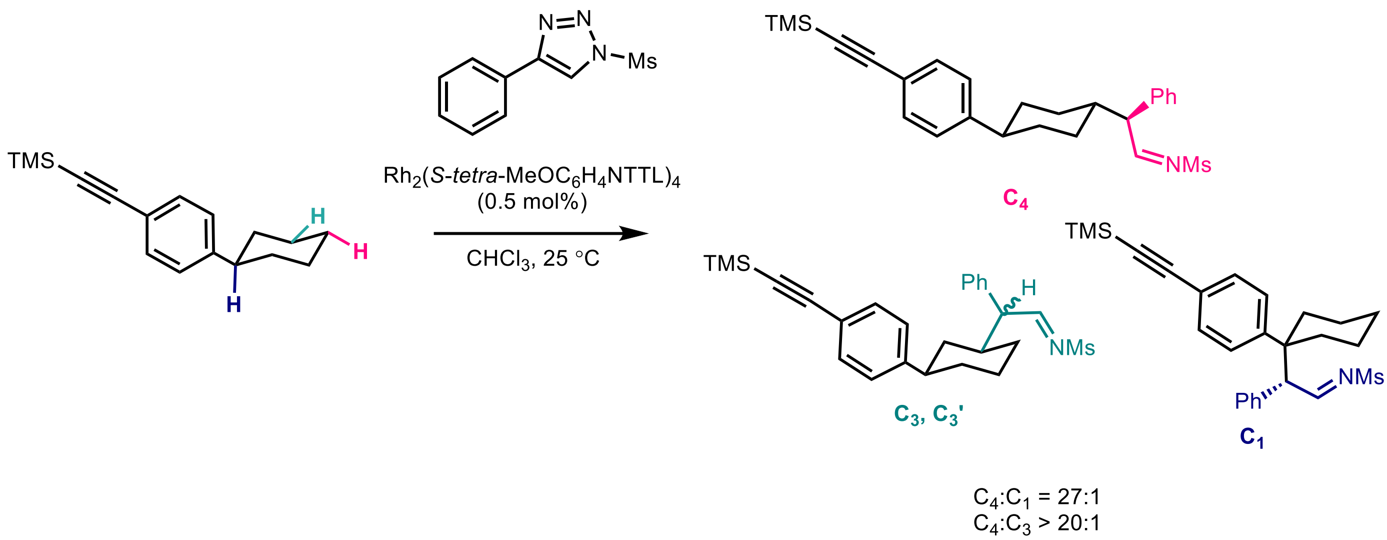

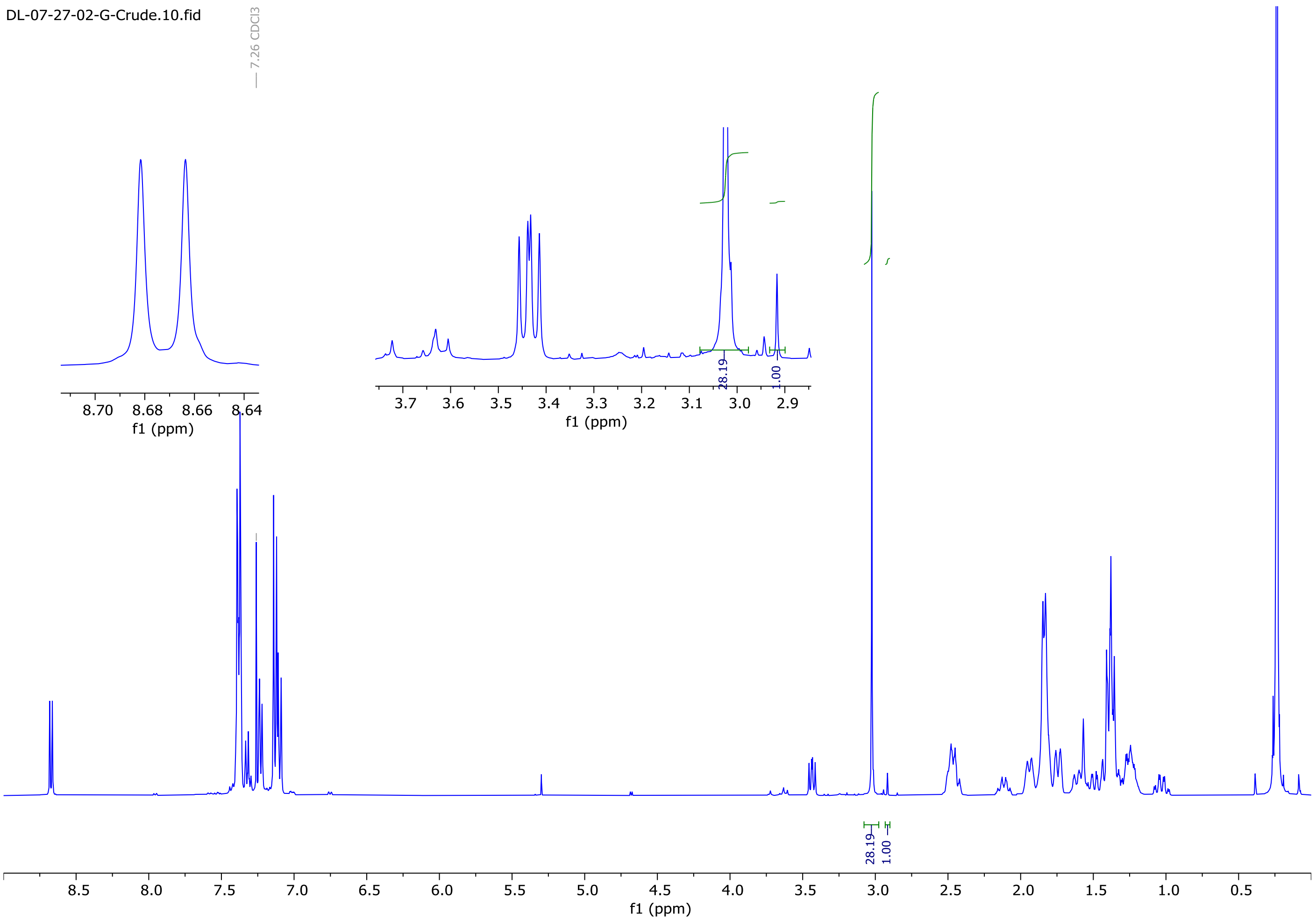

Figure S45. Crude ^{1}H-NMR before reduction of 18

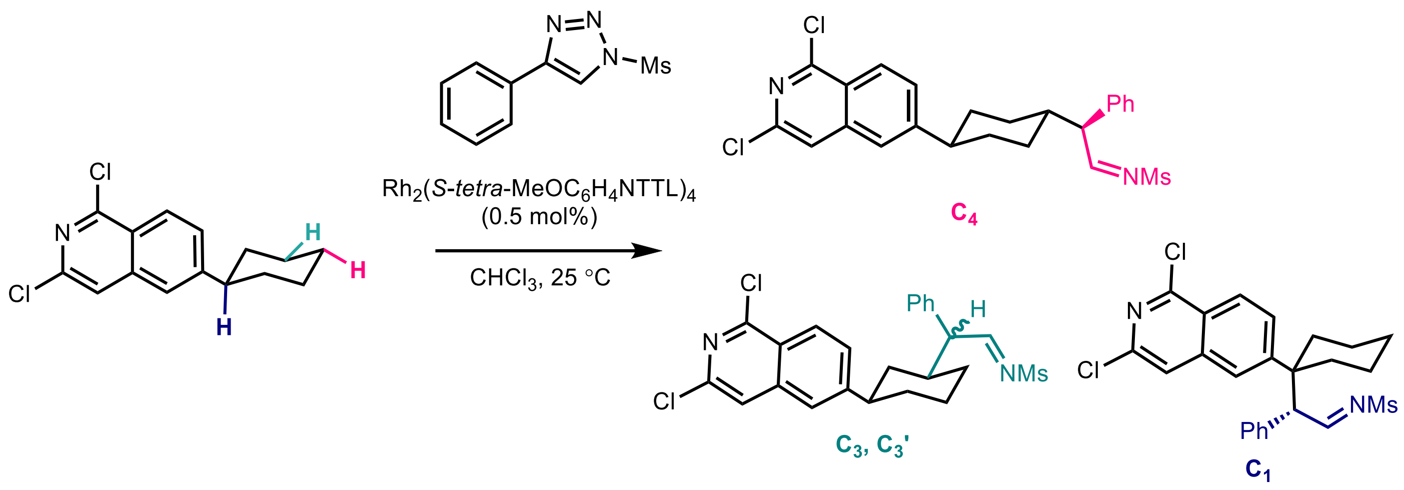

C_4:C_1 = 38:1
C_4:C_3 = > 20:1

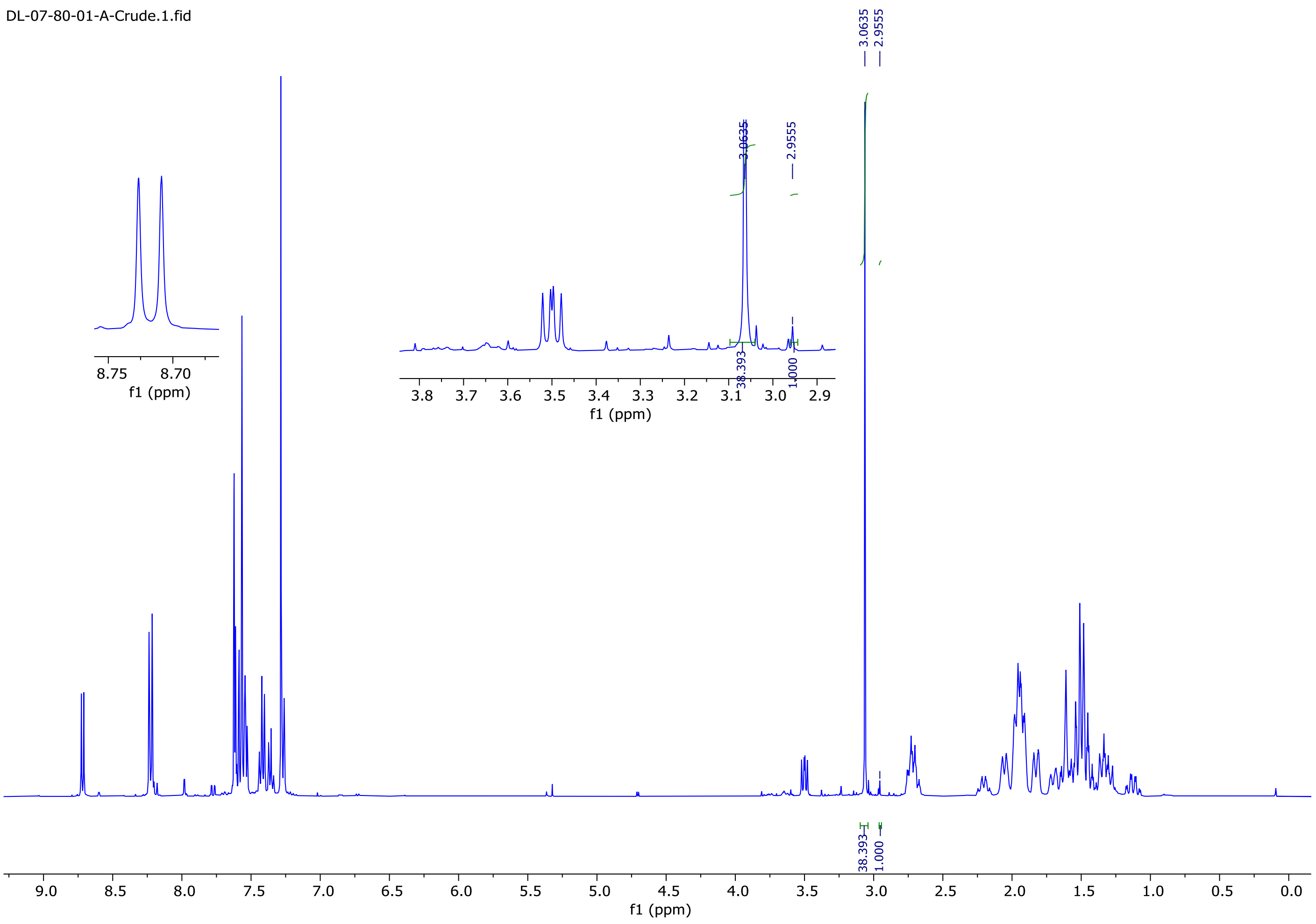

Figure S46. Crude ^{1}H-NMR before reduction of 19

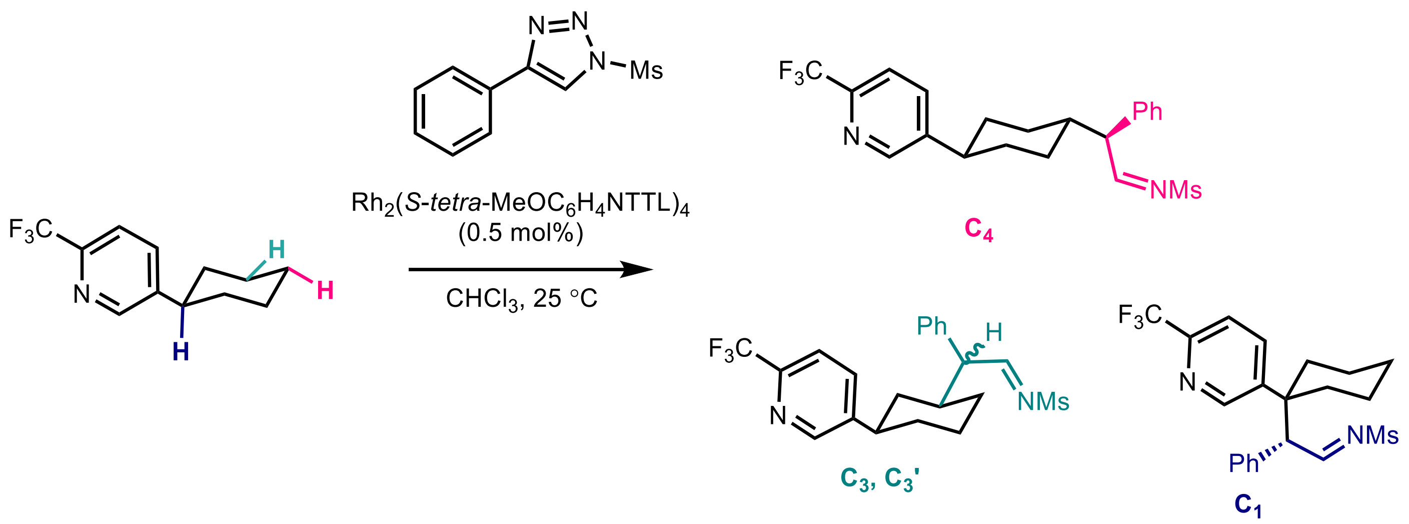

C_4:C_1 = 19:1
C_4:C_3 > 20:1

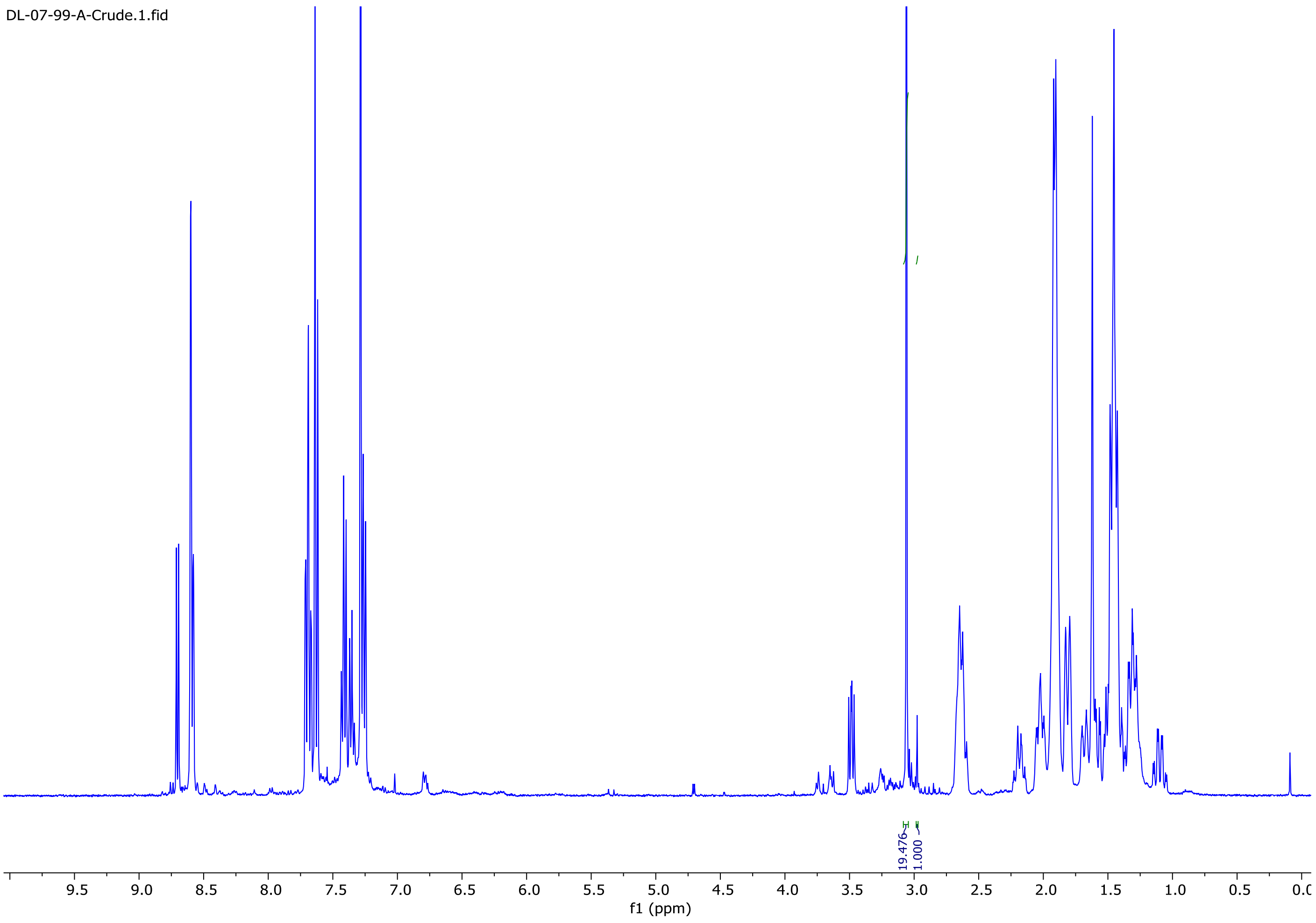

Figure S47. Crude ^{1}H-NMR of 20

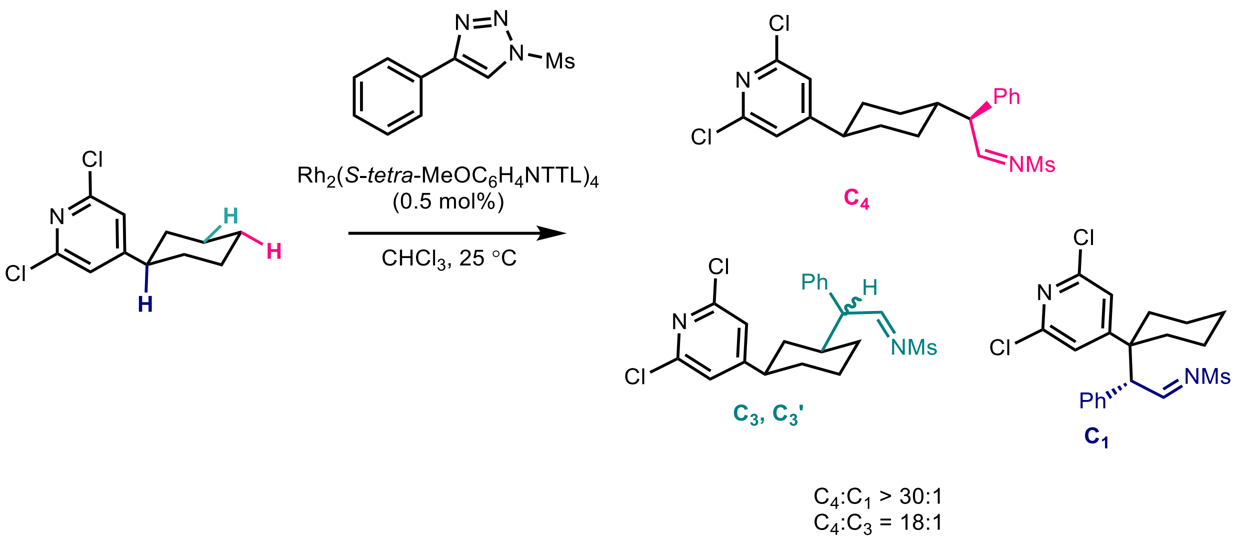

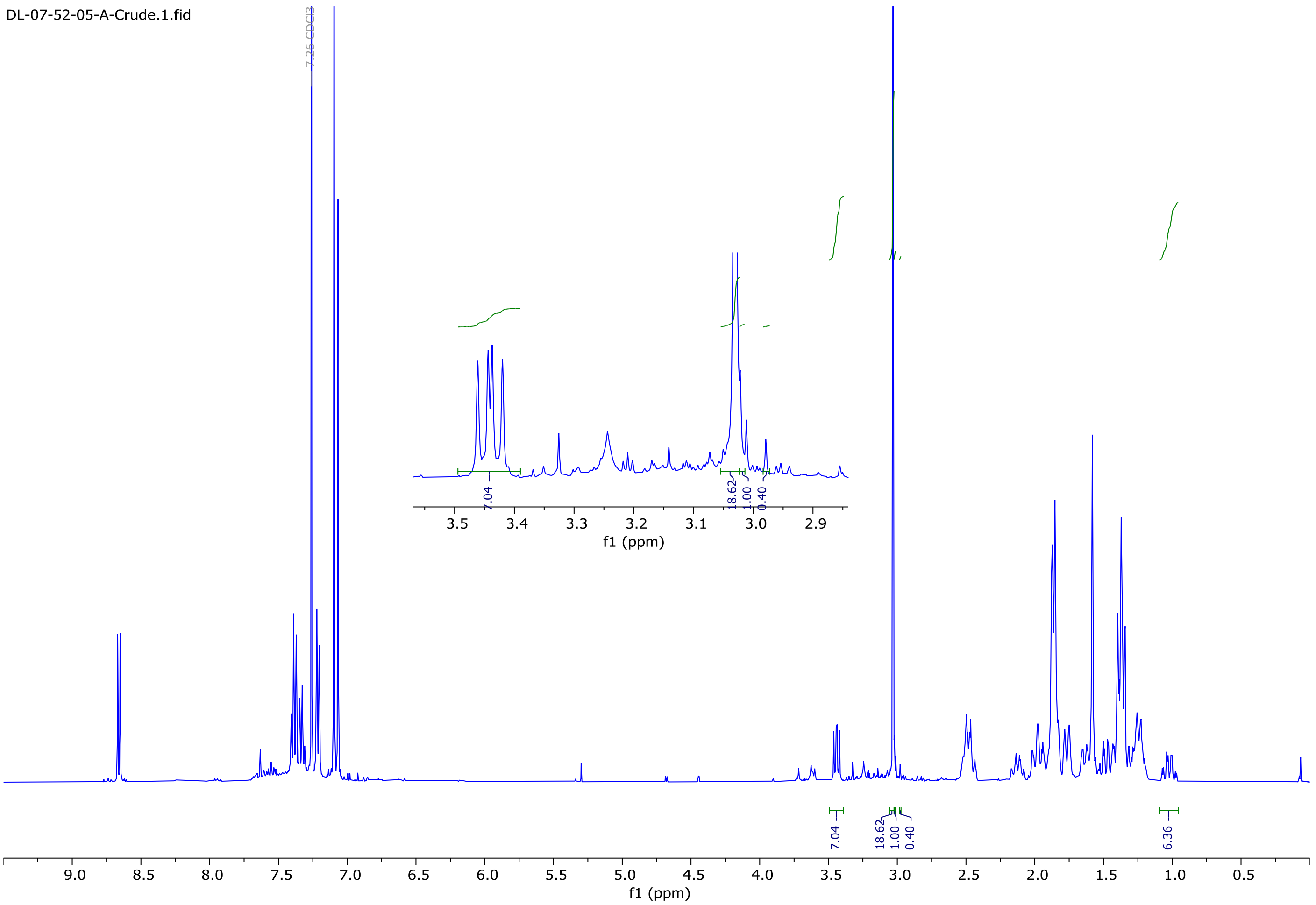

Figure S48. Crude ^{1}H-NMR before reduction of 21

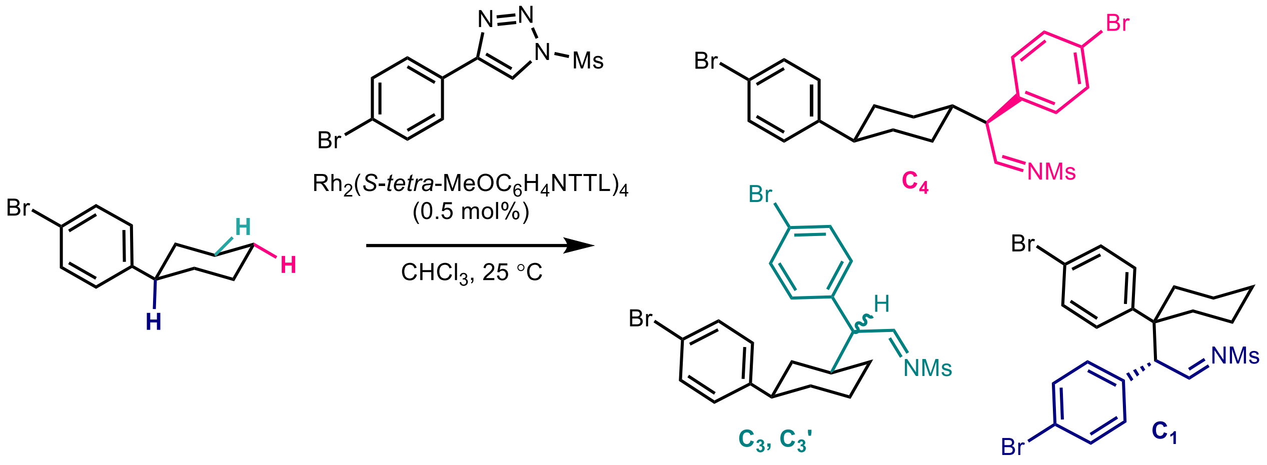

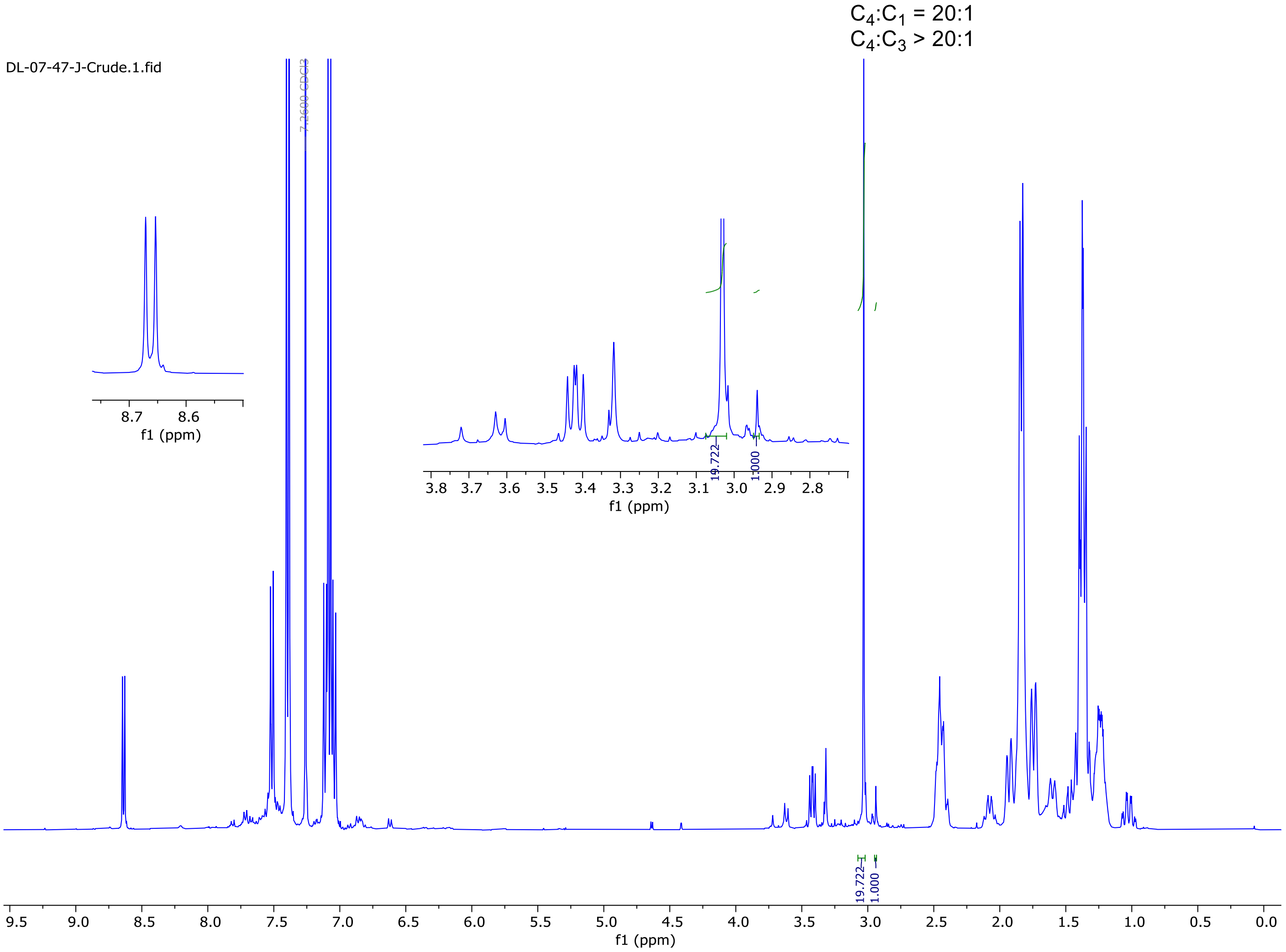

Figure S49. Crude ^{1}H-NMR before reduction of 22

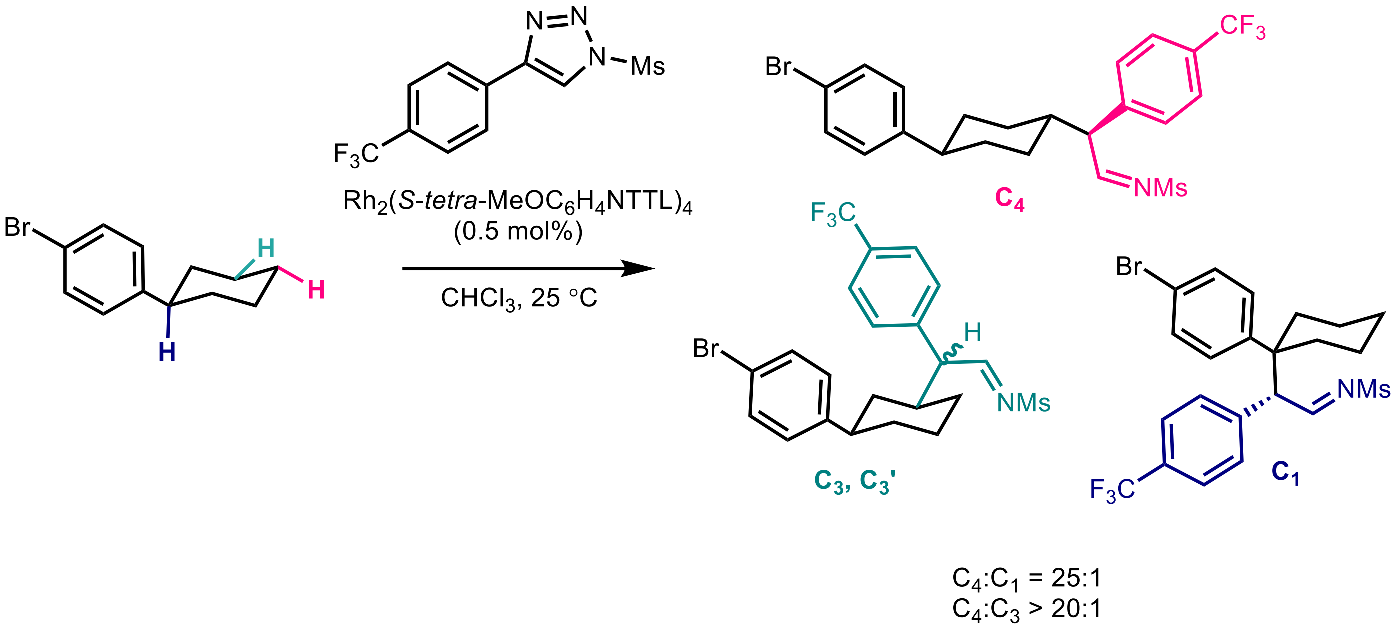

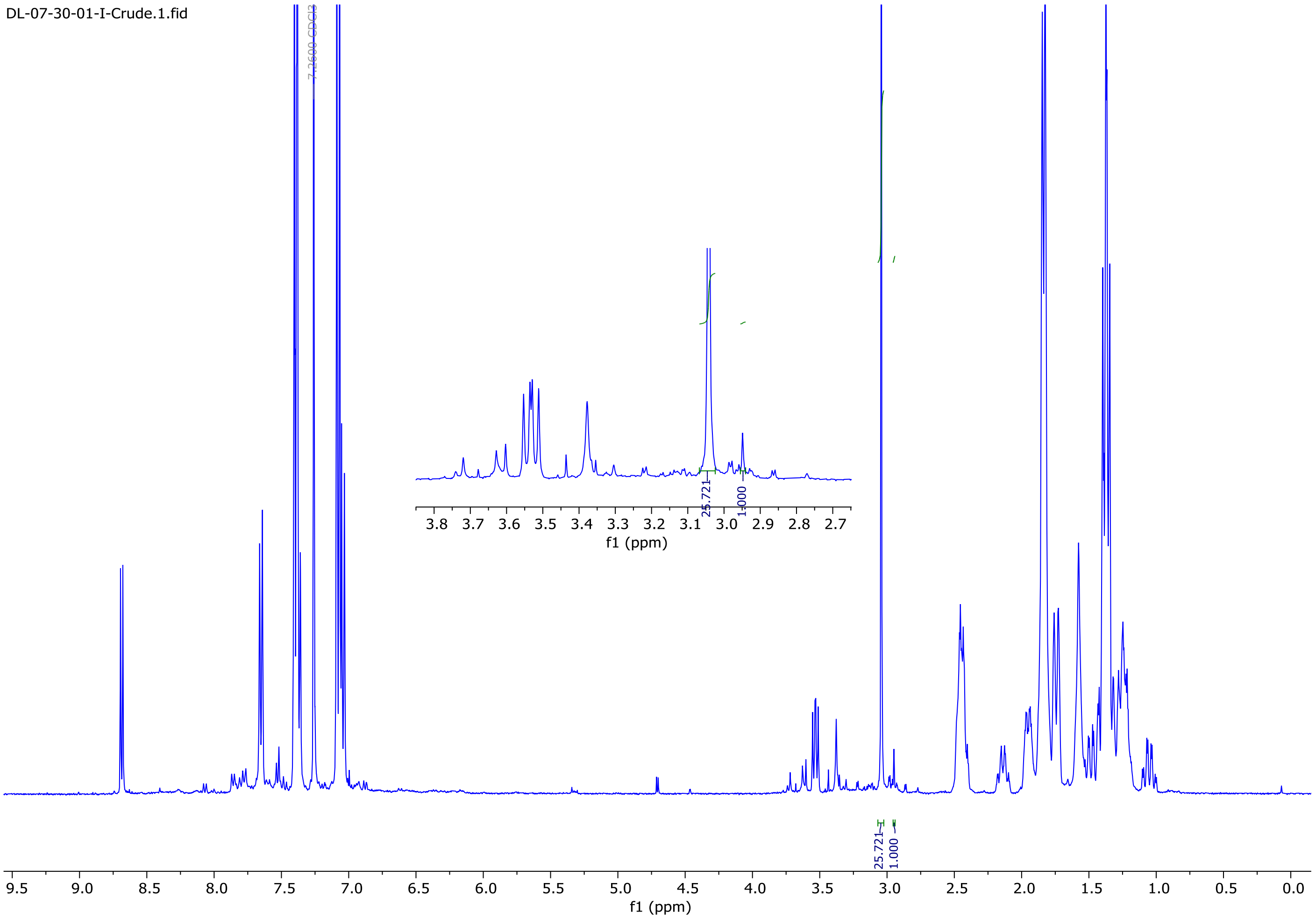

Figure S50. Crude ^{1}H-NMR before reduction of 23

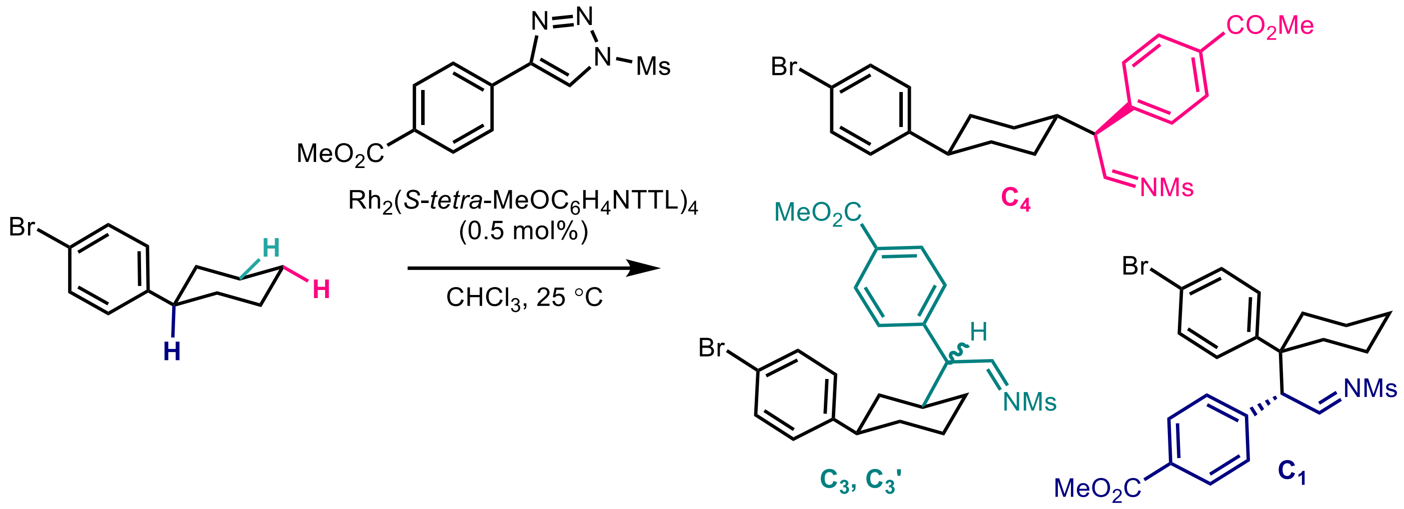

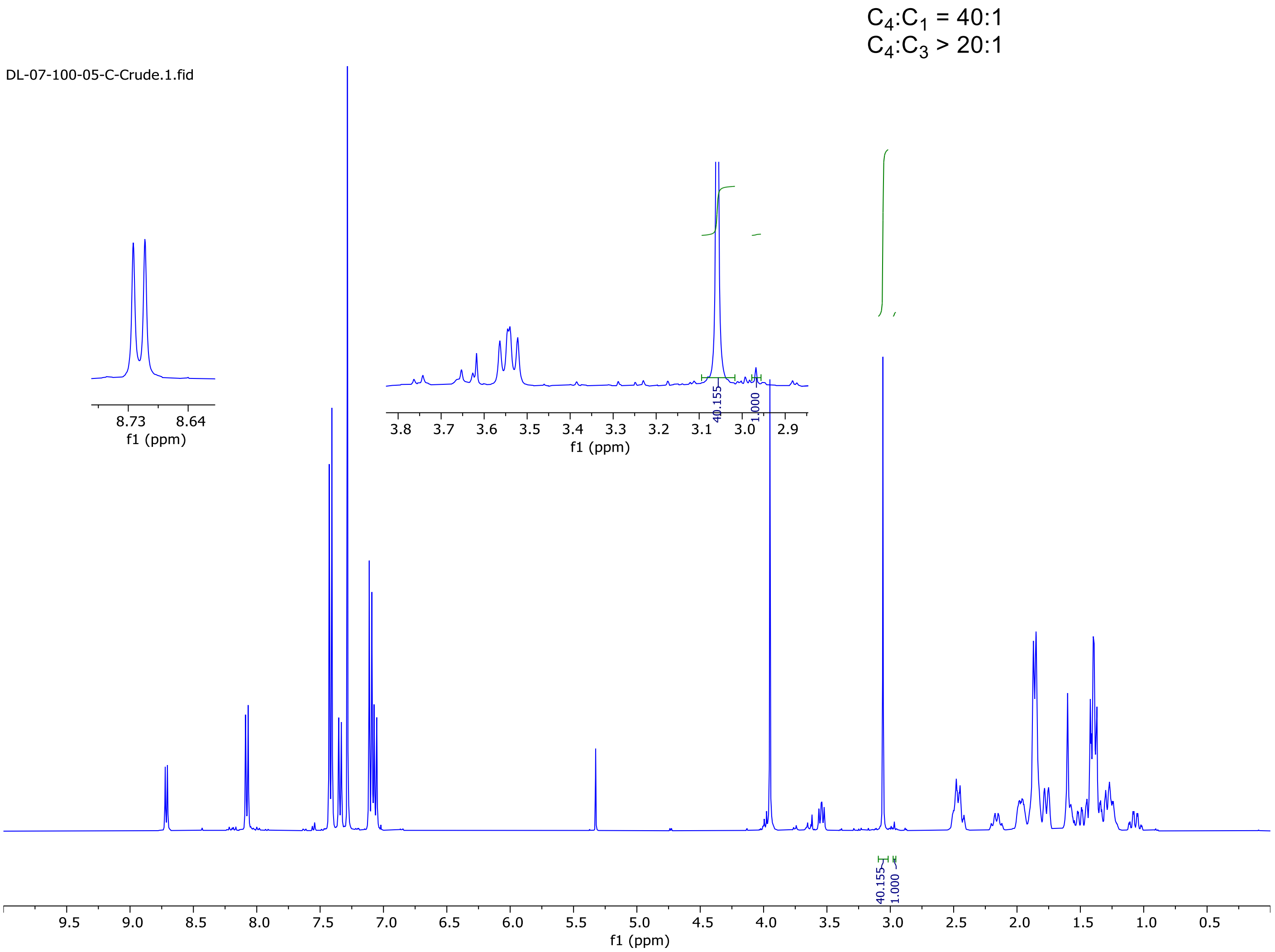

Figure S51. Crude ^{1}H-NMR before reduction of 24

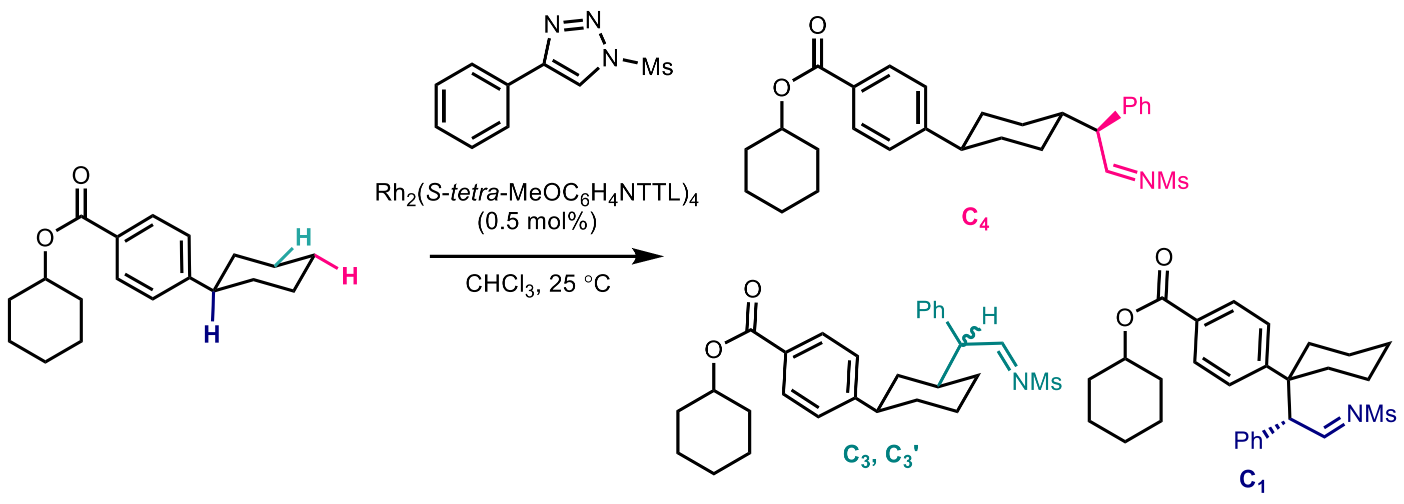

C_4:C_1 = 31:1
C_4:C_3 > 20:1

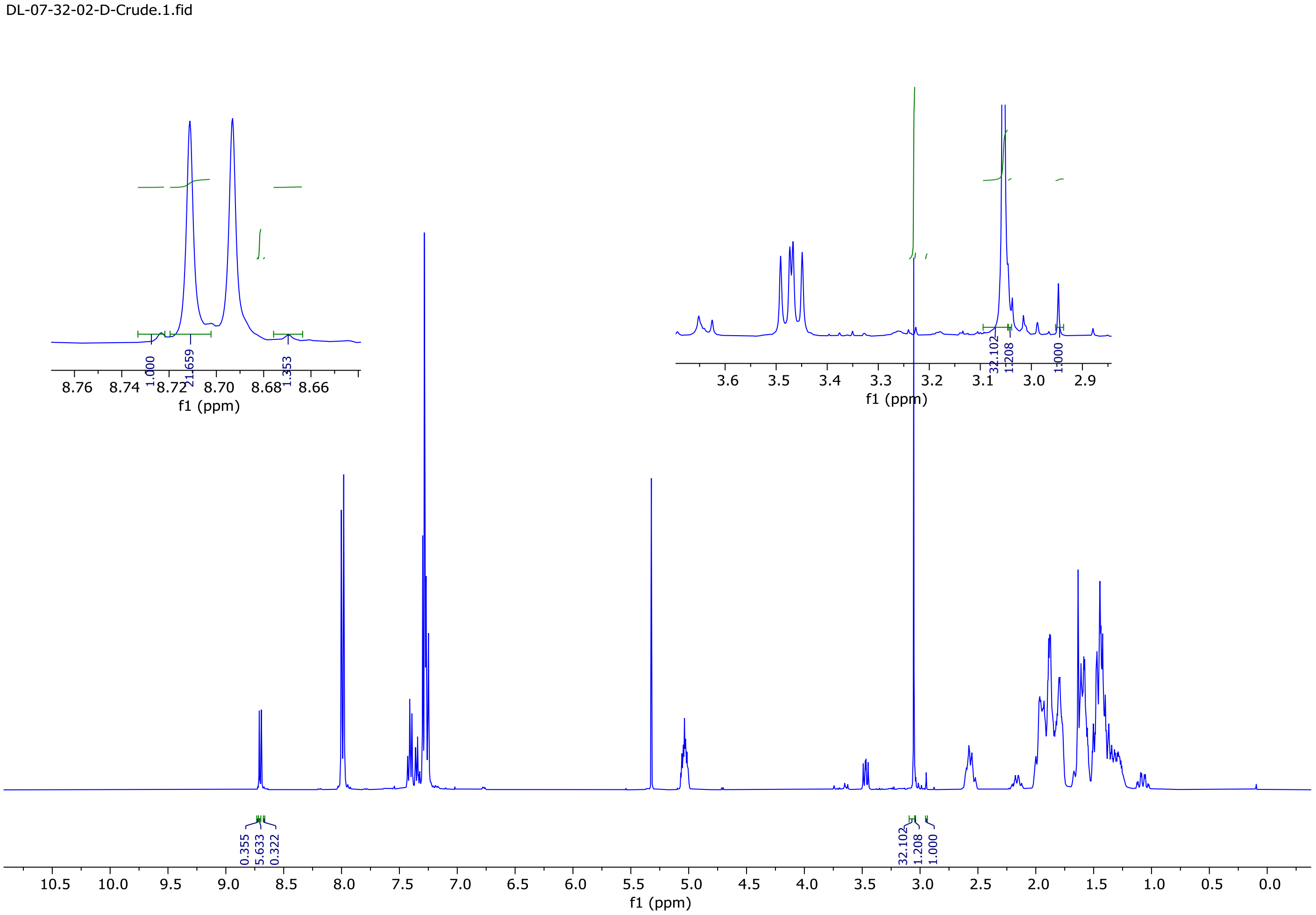

Figure S52. Crude ^{1}H-NMR before reduction of 25

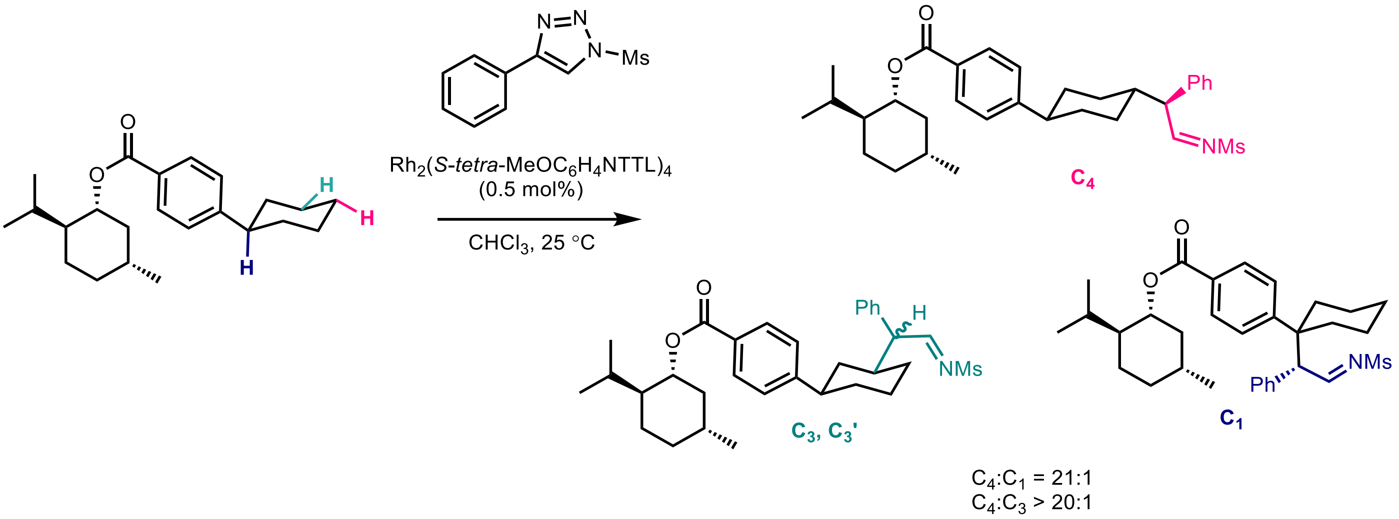

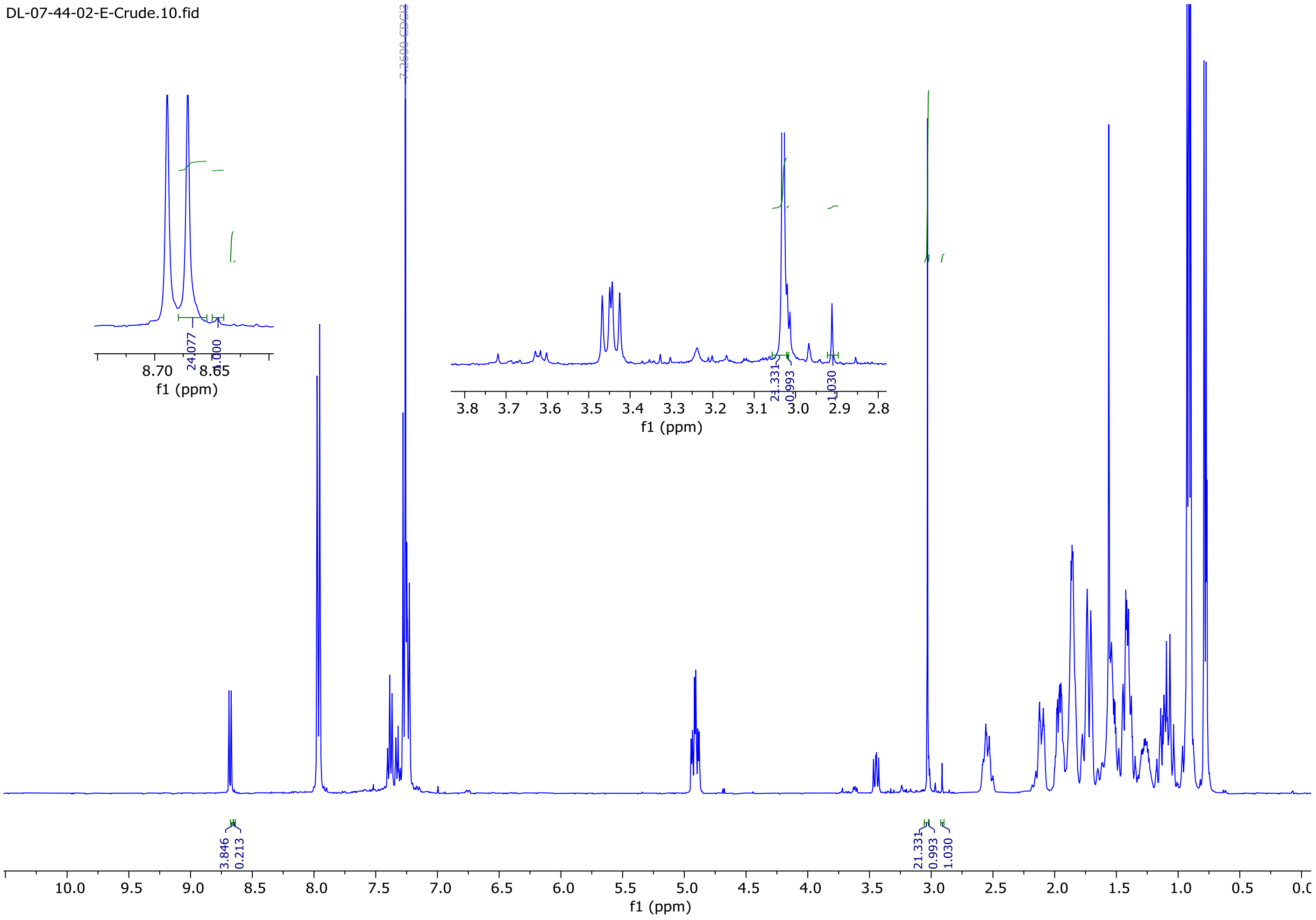

Figure S53. Crude ^{1}H-NMR before reduction of 26

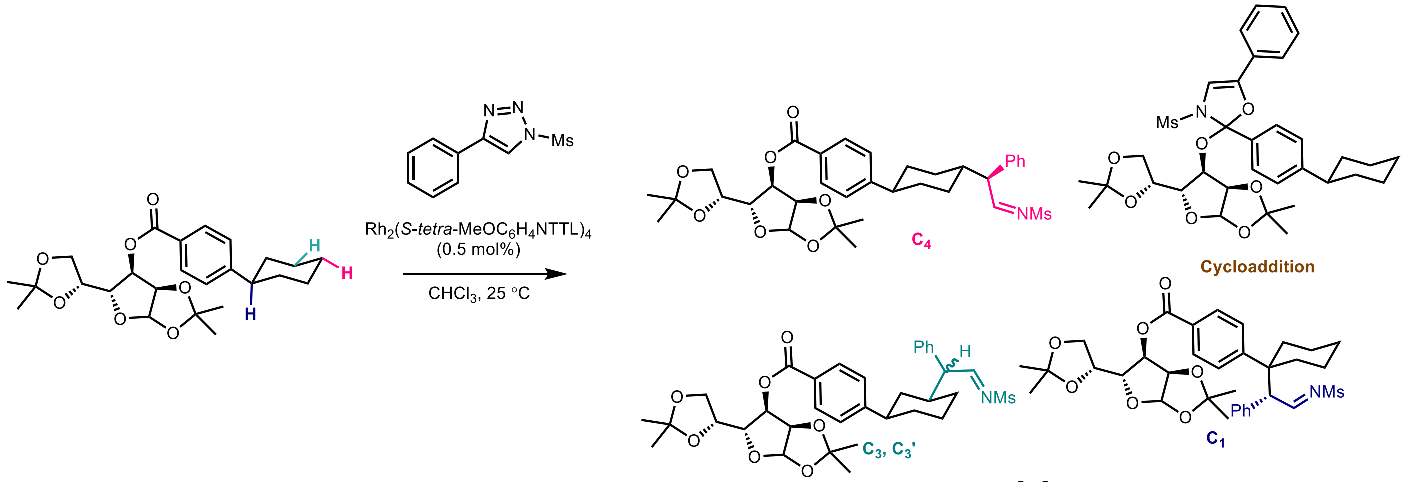

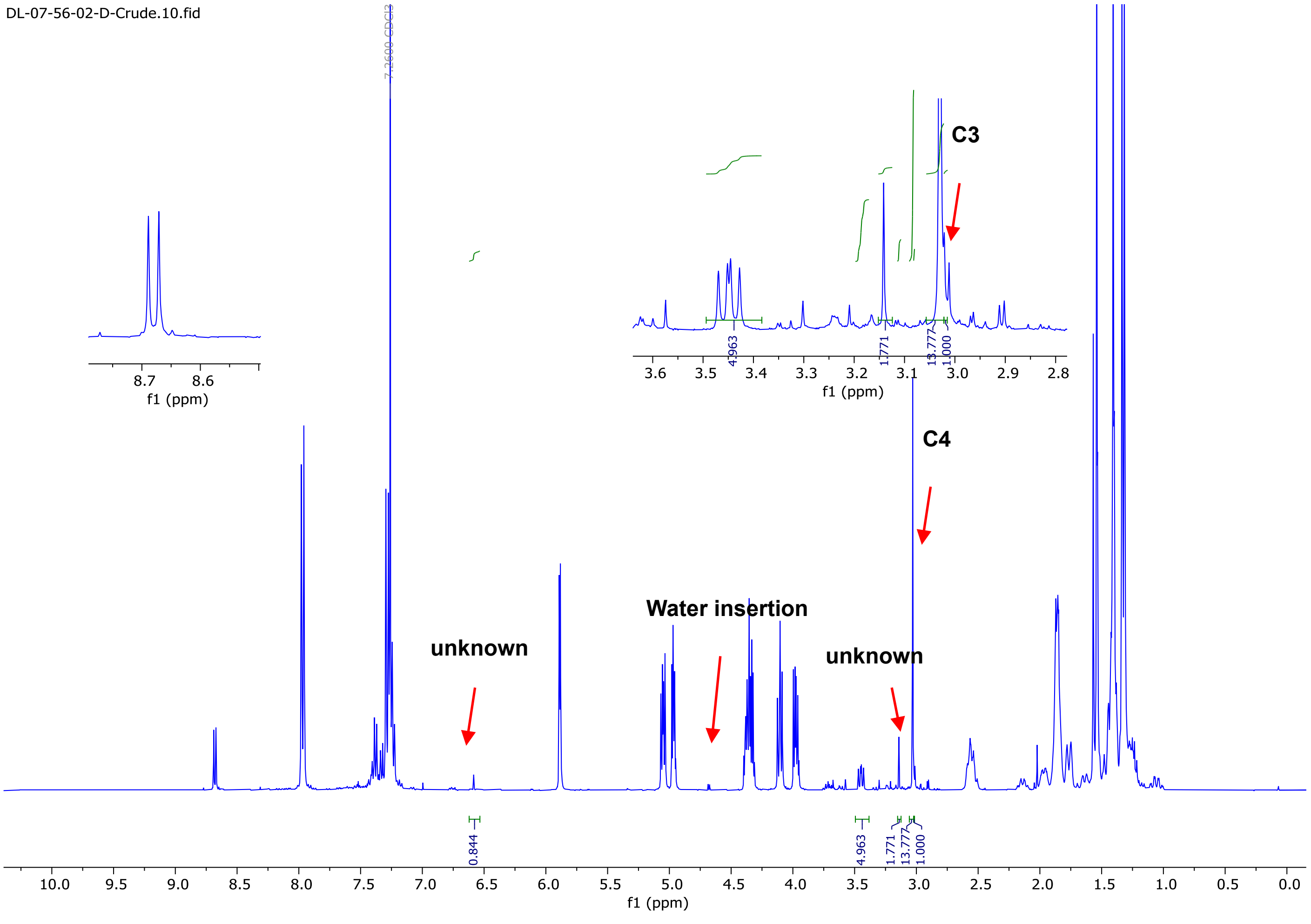

Figure S54. Crude ^{1}H-NMR before reduction of 27. No characteristic signal for C1 was observed. There is an unknown by-product at 6.5 ppm which is tentatively assigned to be the product of the cycloaddition to the ester group. We observed this type of product in the reaction catalyzed by Rh$_2$(*R*/*S*-NTTL)$_4$.

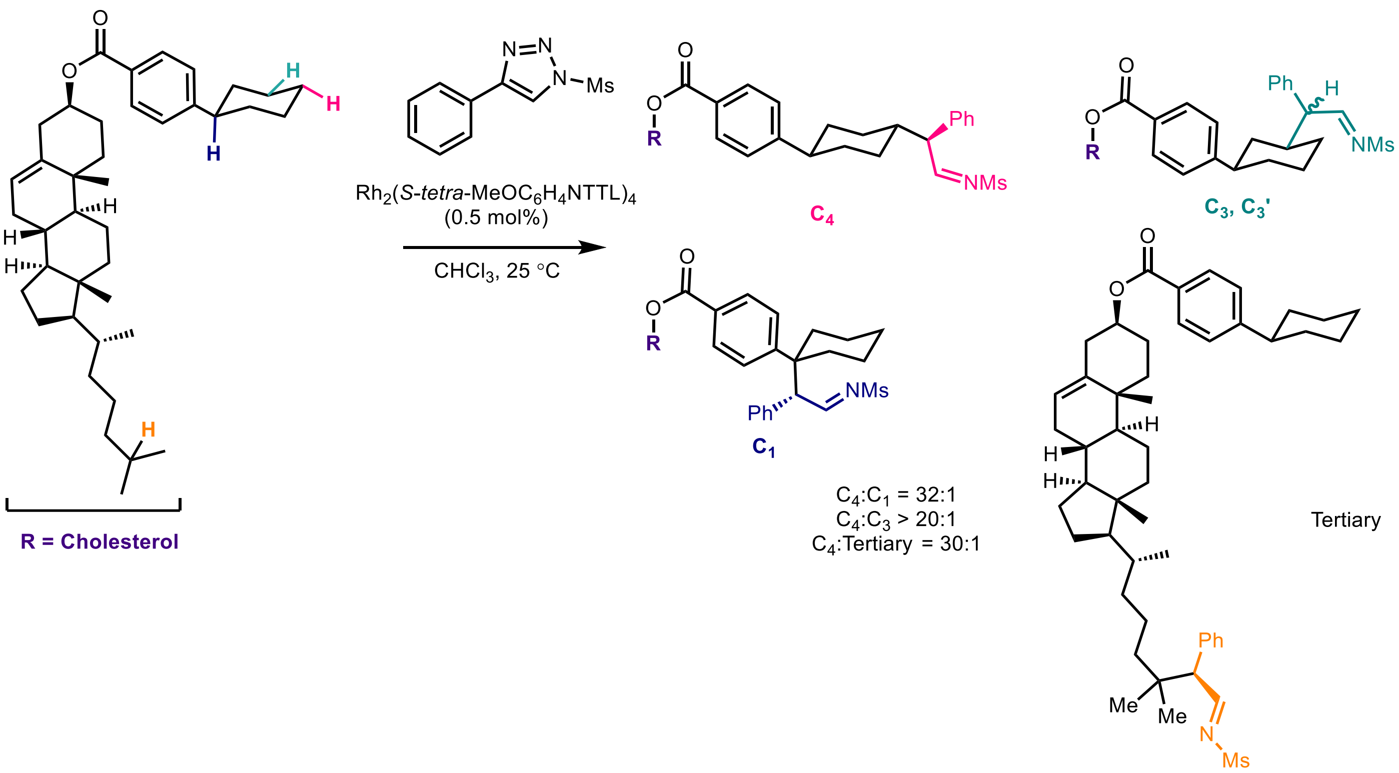

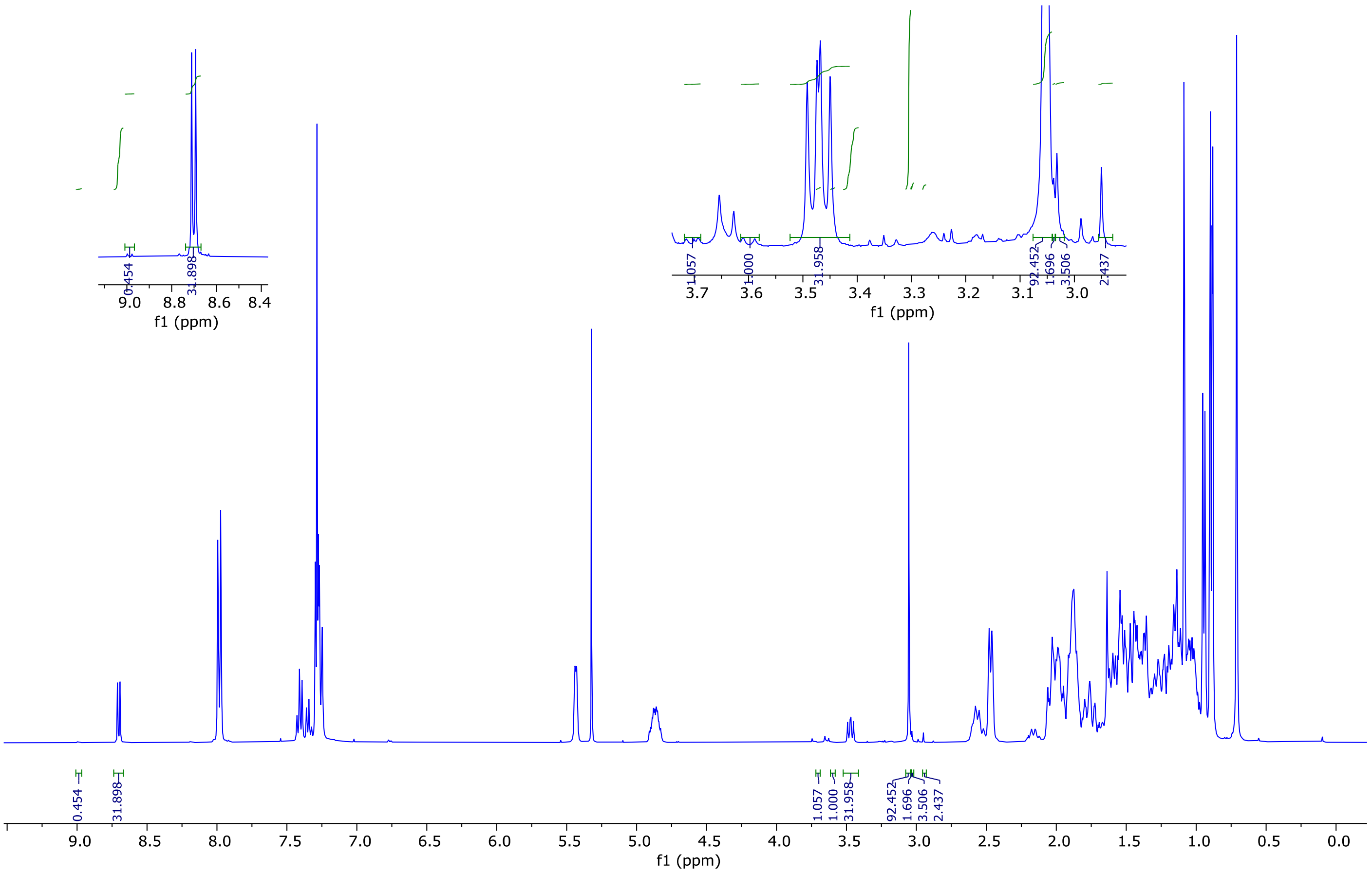

Figure S55. Crude ^{1}H-NMR before reduction of 28. This reaction gave not only C4, C1, and C3 functionalization but also gave reaction at tertiary C-H bond of cholesterol moiety. The assignment of all products was based on characteristic signals, as well as compared to unselective reaction with Rh$_2$(*S*-NTTL)$_4$. *See below for details NMR.*

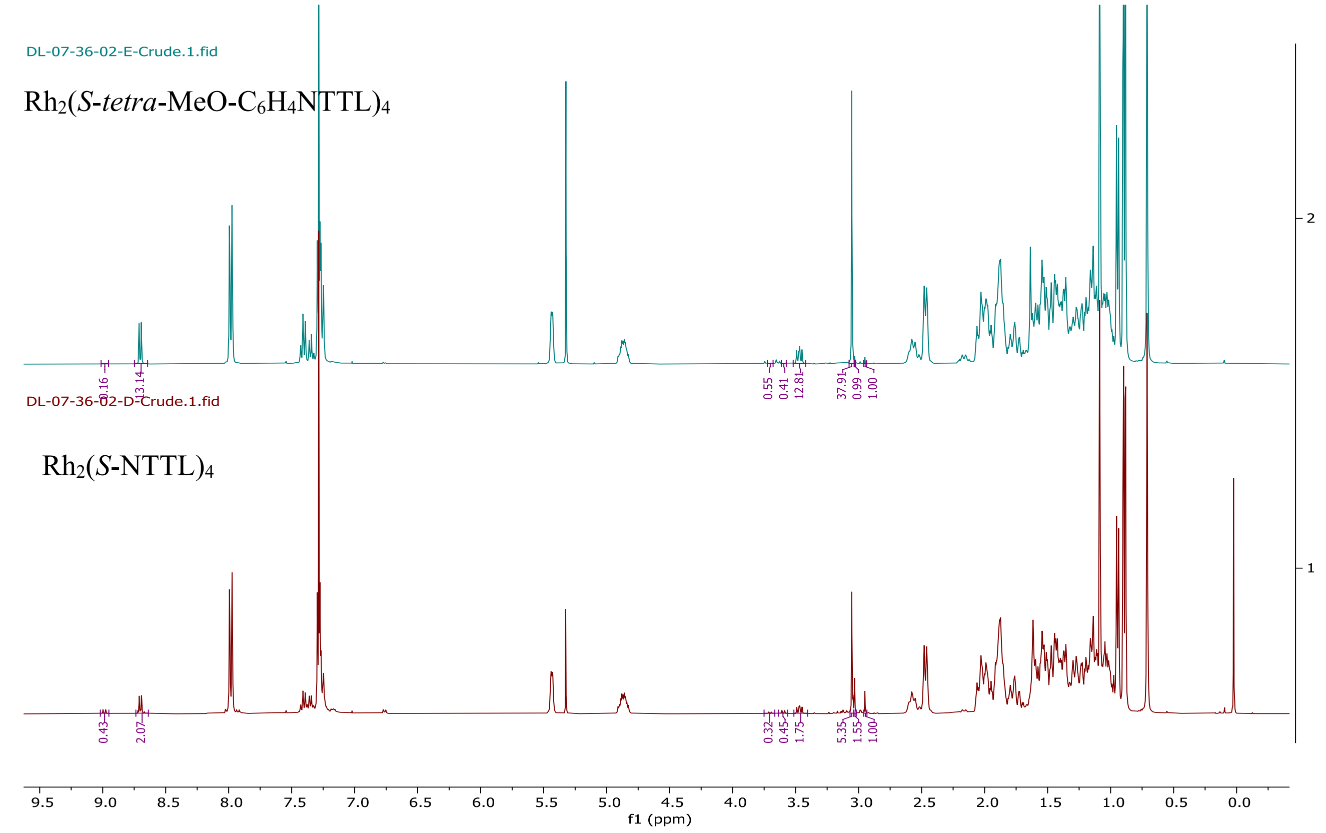

Figure S56. Compared crude ^{1}H-NMR before reduction of 28. Top with Rh_2(*S*-*tetra*-MeO-C_6H_4NTTL)$_4$ and bottom with Rh_2(*S*-NTTL)$_4$

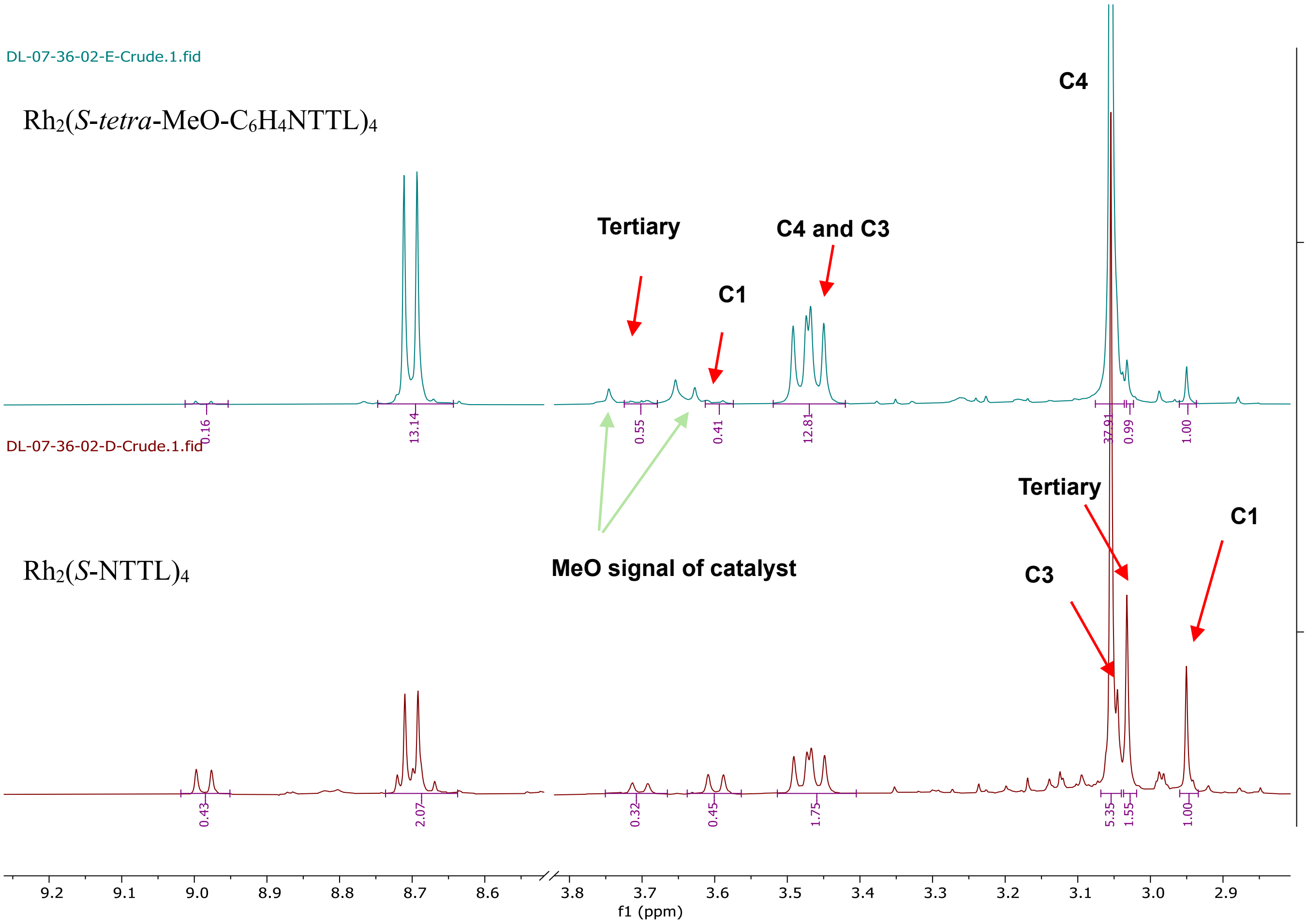

Figure S57. Stack crude ^{1}H-NMR before reduction of 28, Zoom in region of interest. Top with Rh_2(*S*-*tetra*-MeO-C_6H_4NTTL)$_4$ and bottom with Rh_2(*S*-NTTL)$_4$

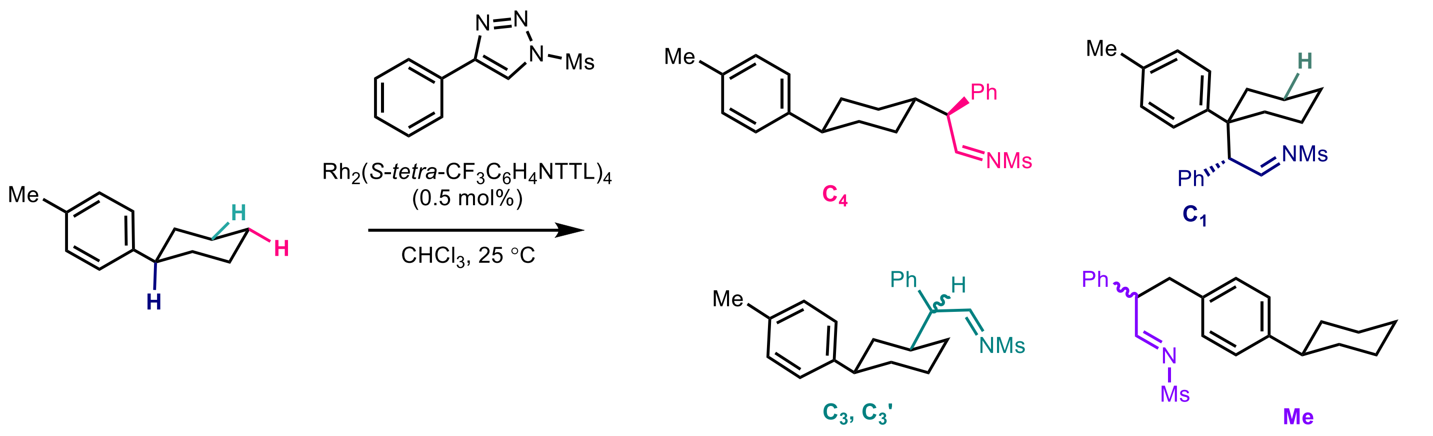

$C_4:C_1 = 15:1$
$C_4:C_3 > 20:1$
$C_4:Me = 20:1$

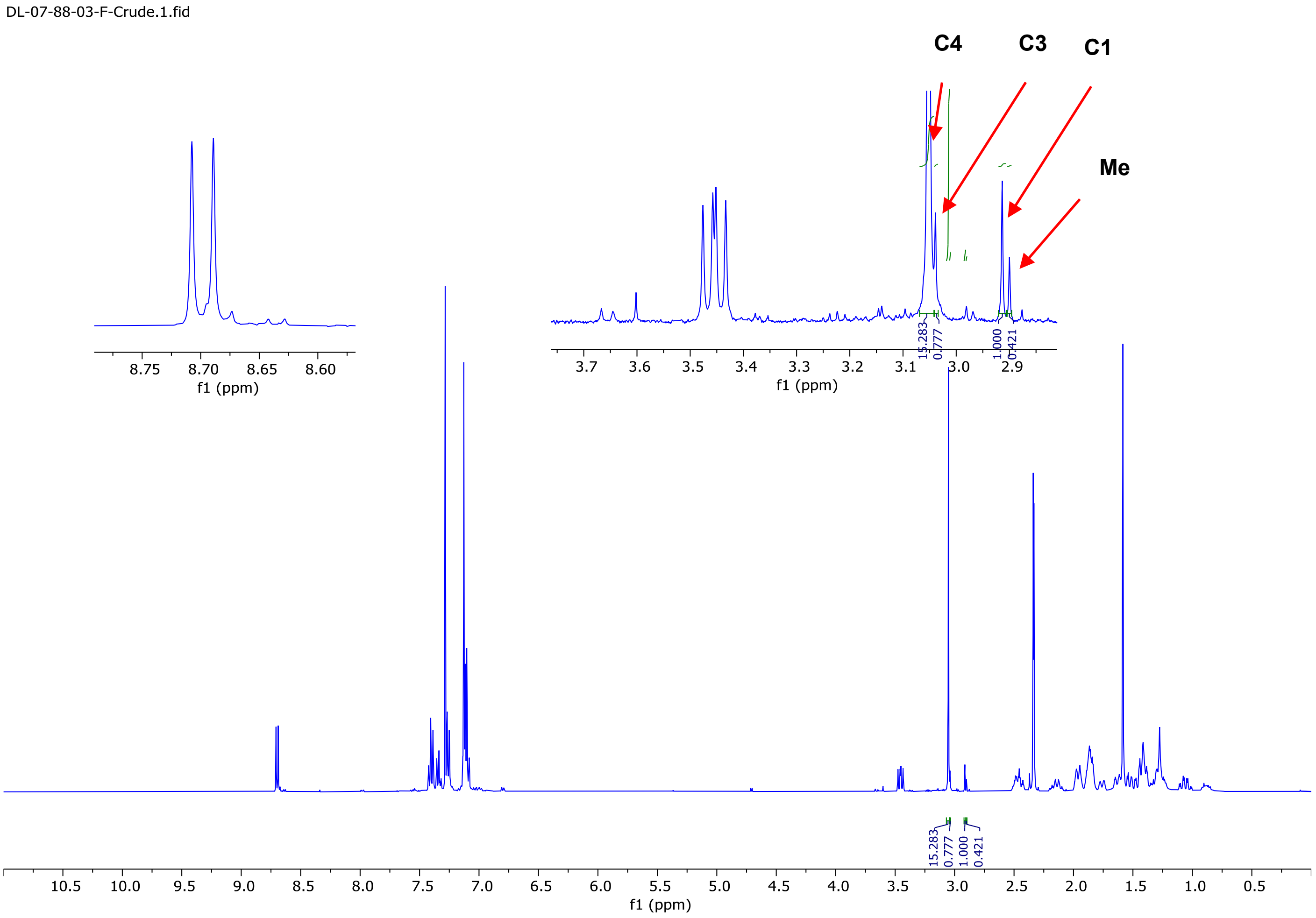

Figure S58. Crude ^{1}H-NMR before reduction of 29. *See optimization for more detail results with other catalysts.*

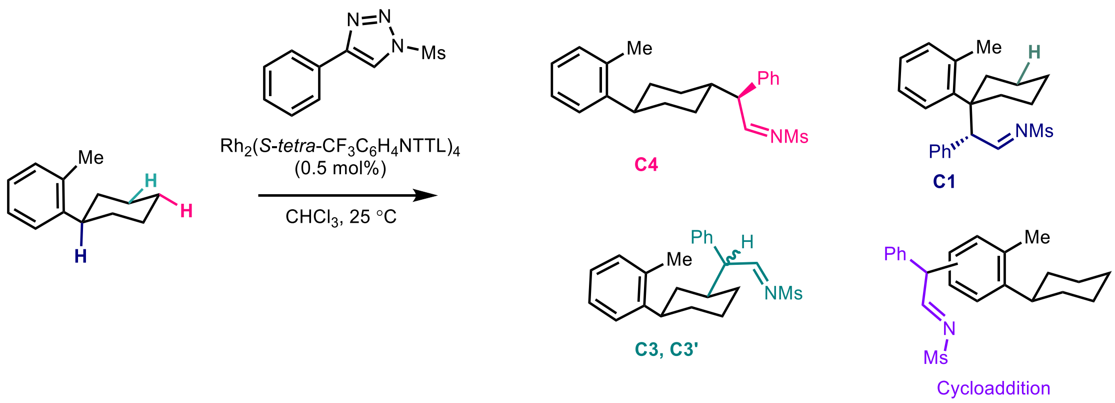

C4:C1 = na
C4:C3 = 9:1
and cycloaddition products

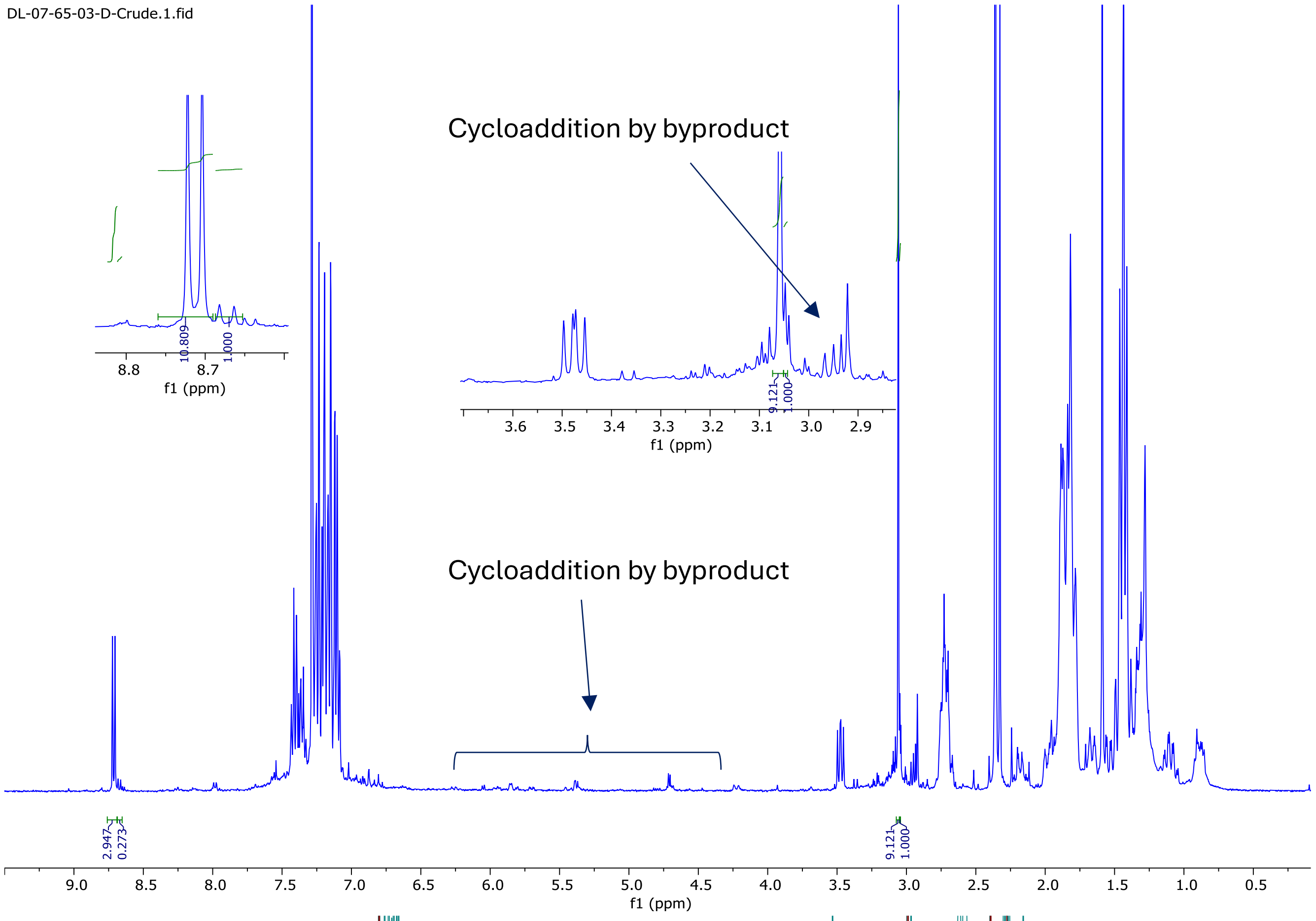

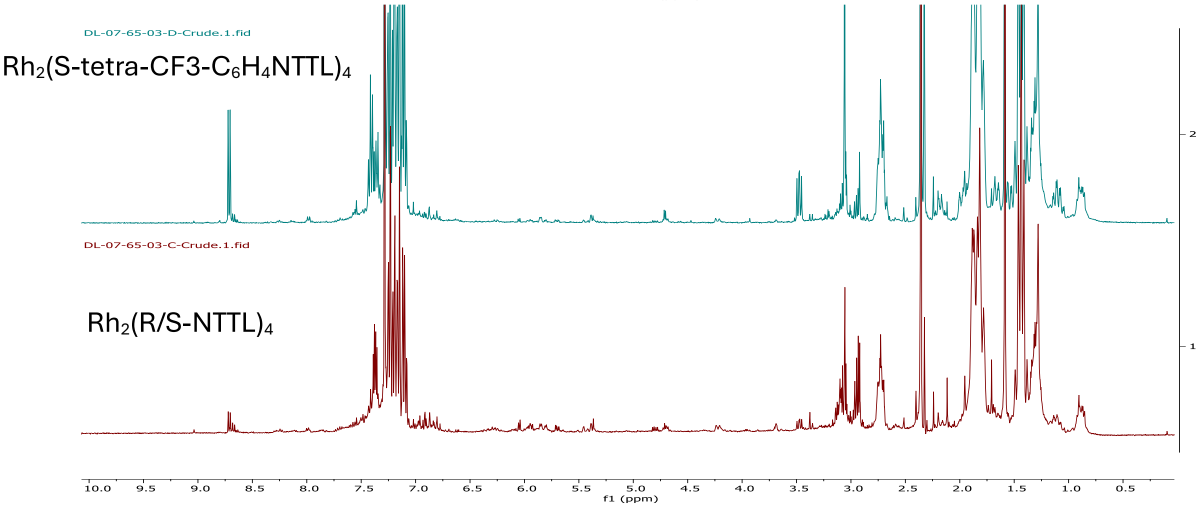

Figure S59. Crude ^{1}H-NMR before reduction of 30. *Not only C-H insertion product was observed, but there is also some trace undetermined cycloaddition products as compared to the reaction conducted with $Rh_2(R/S\text{-}NTTL)_4$*

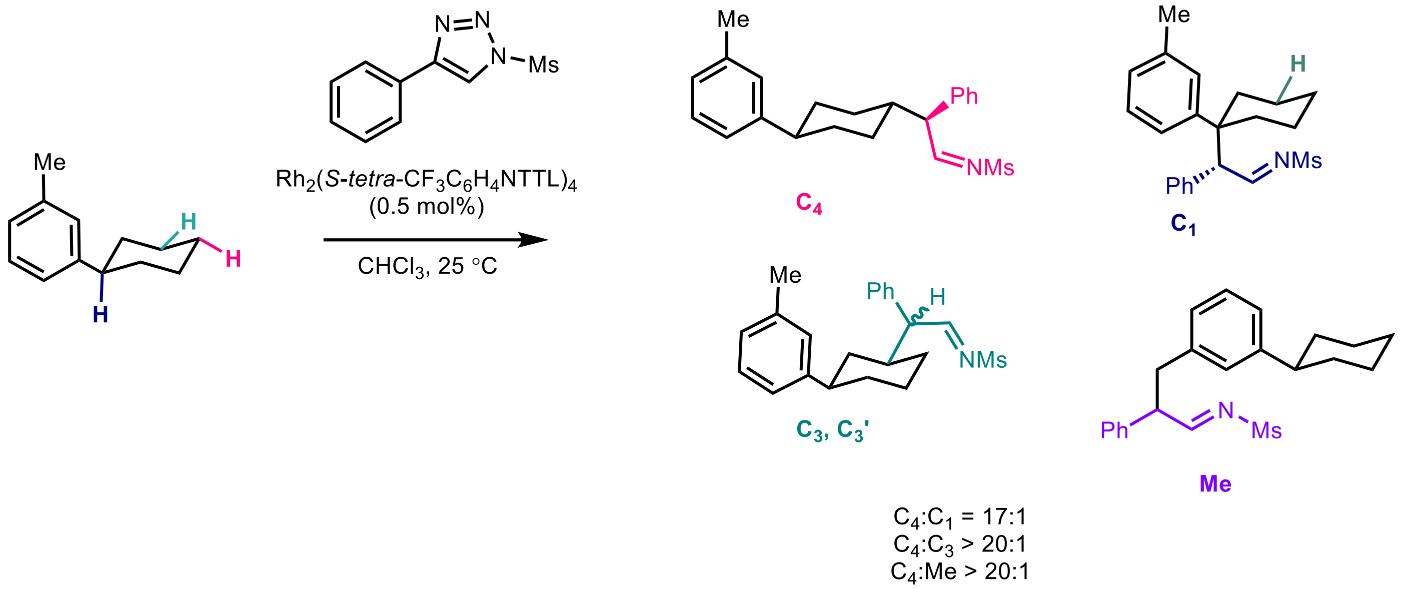

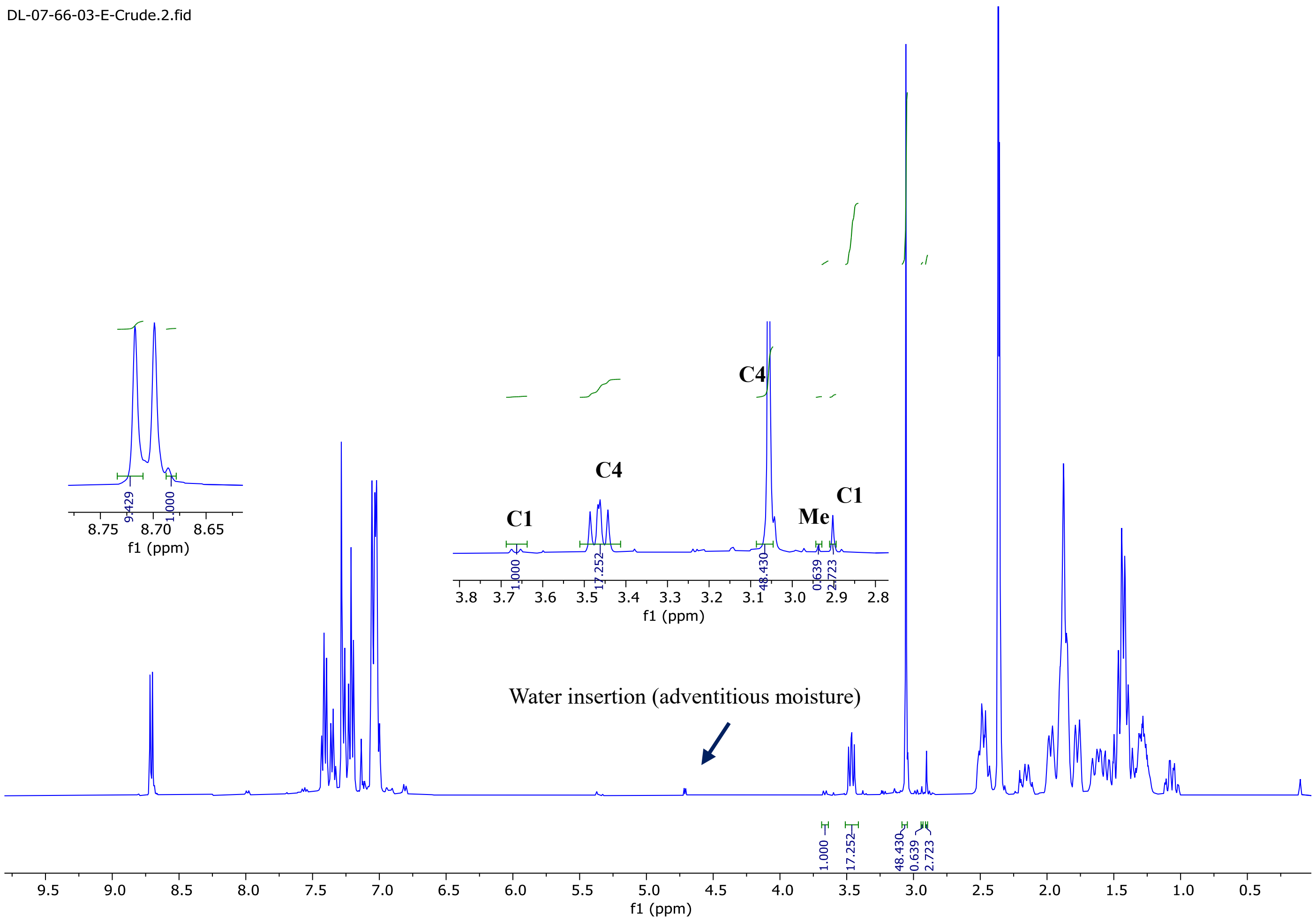

Figure S60. Crude ^{1}H-NMR before reduction of 31

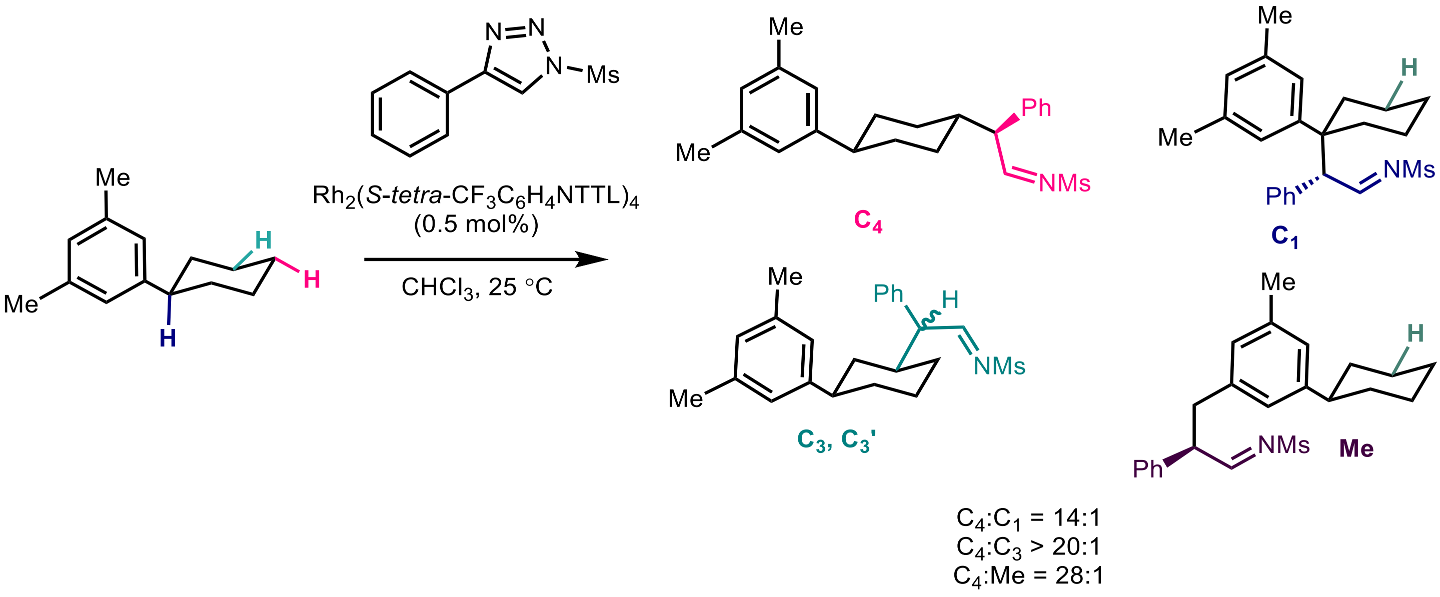

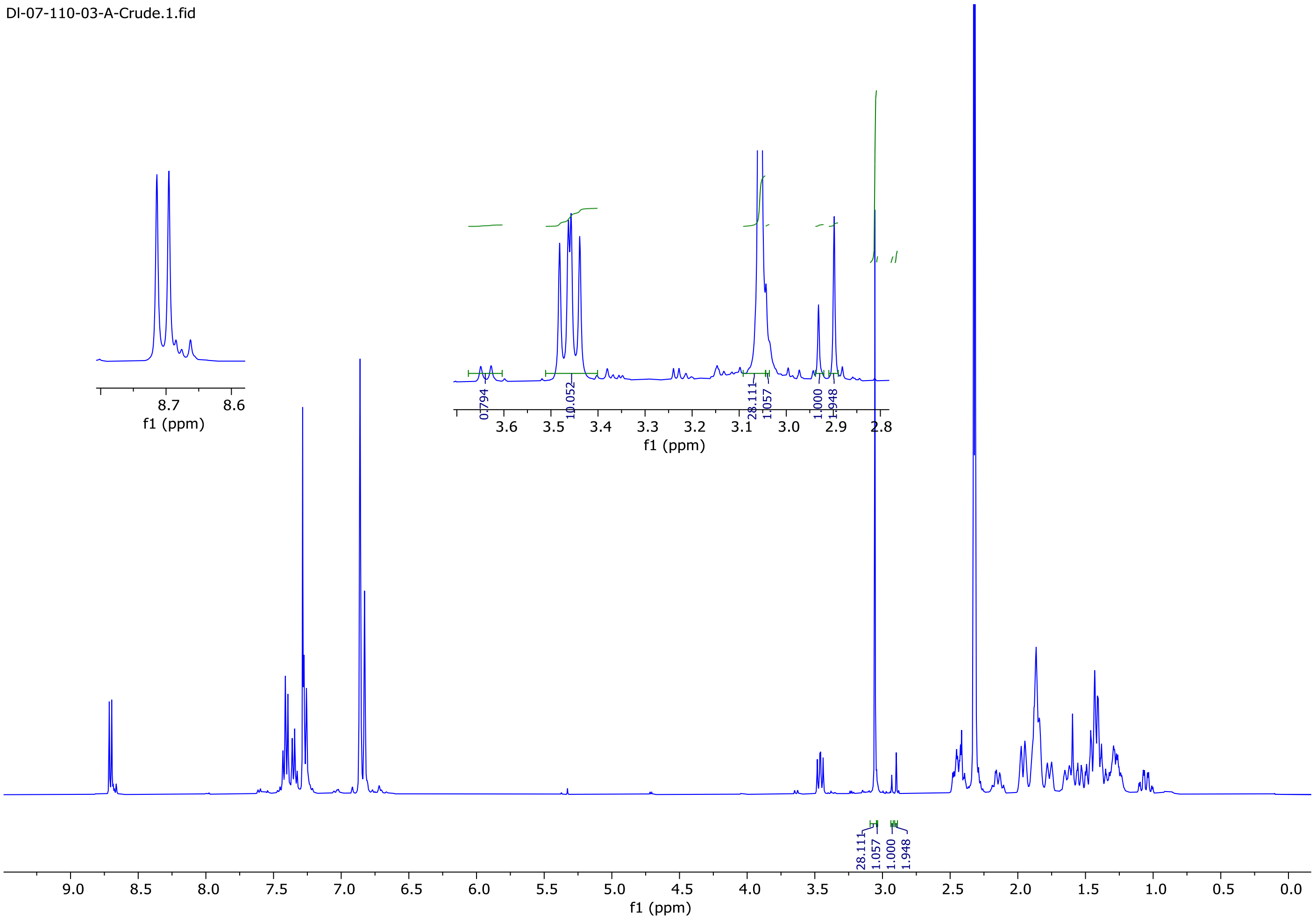

Figure S61. Crude ^{1}H-NMR before reduction of 32

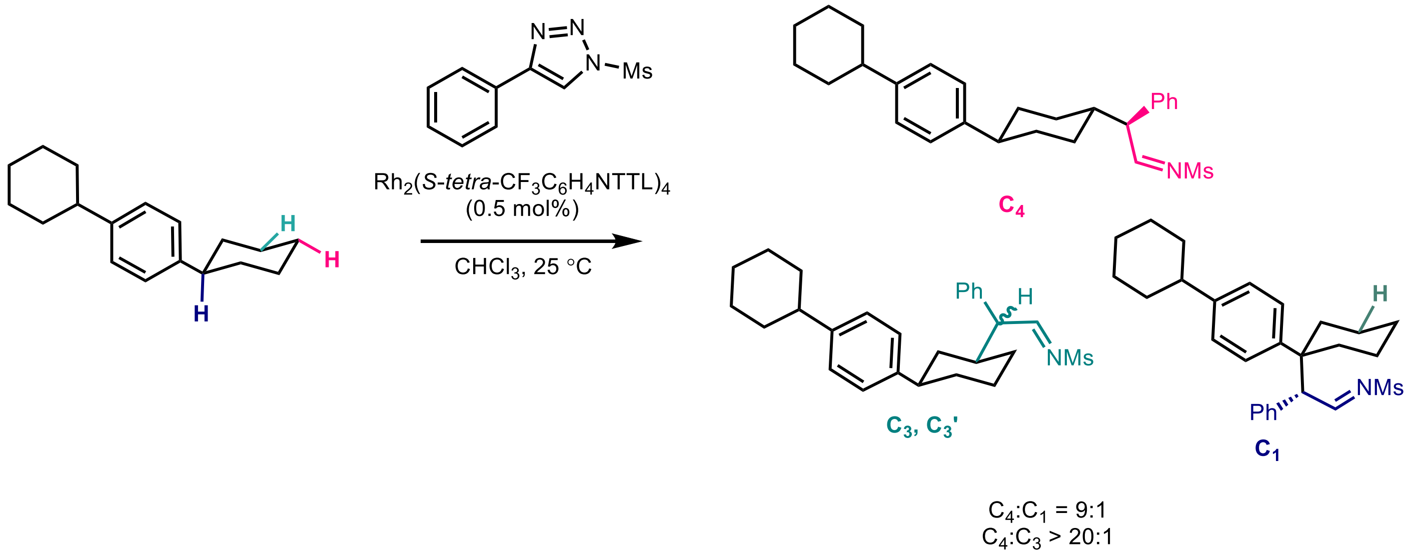

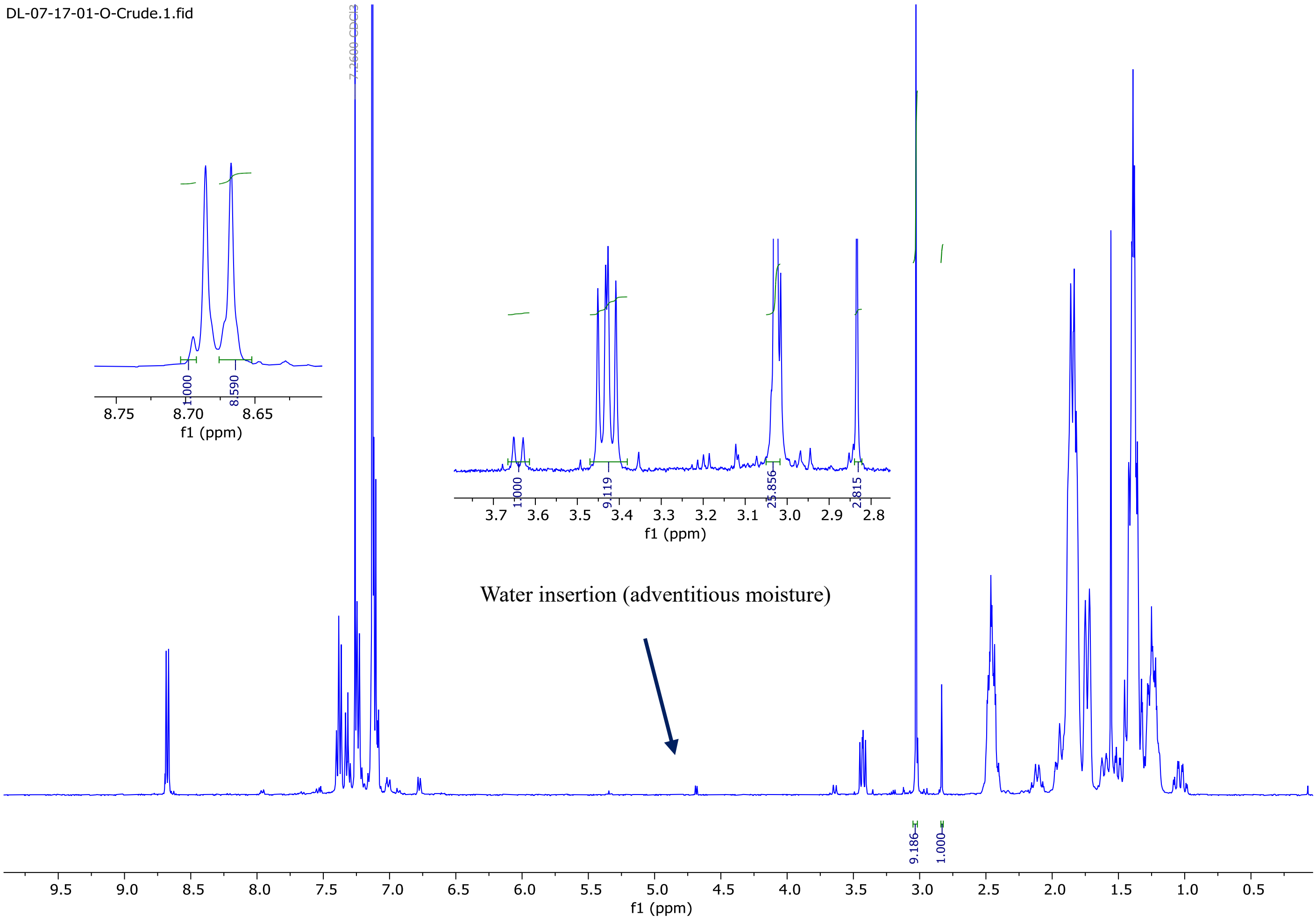

Figure S62. Crude ^{1}H-NMR before reduction of 33

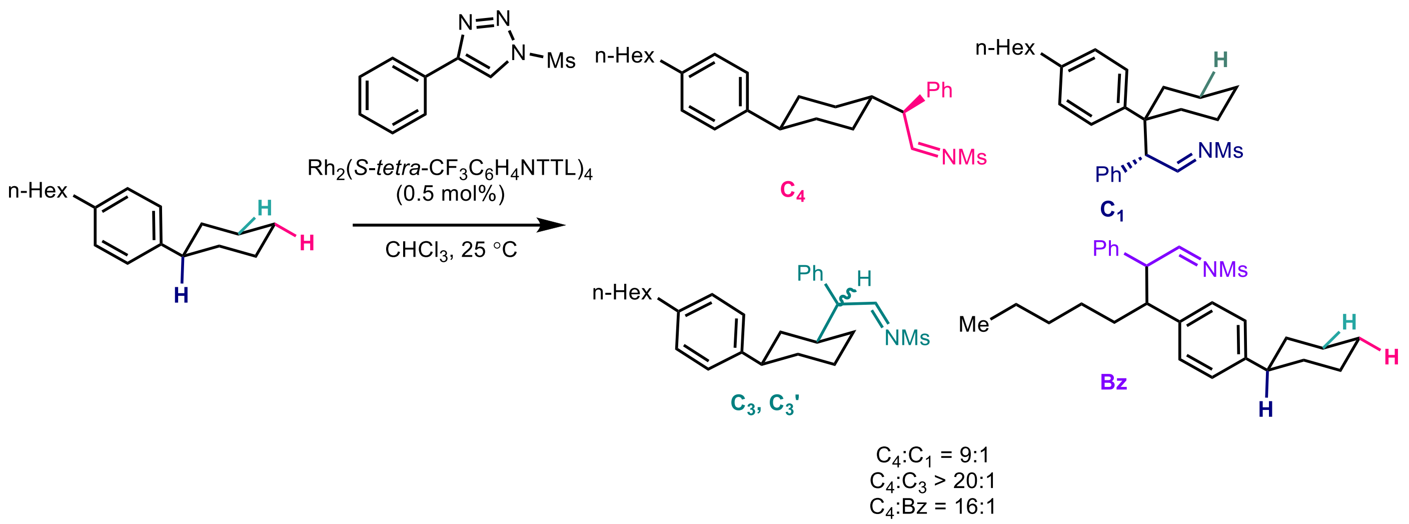

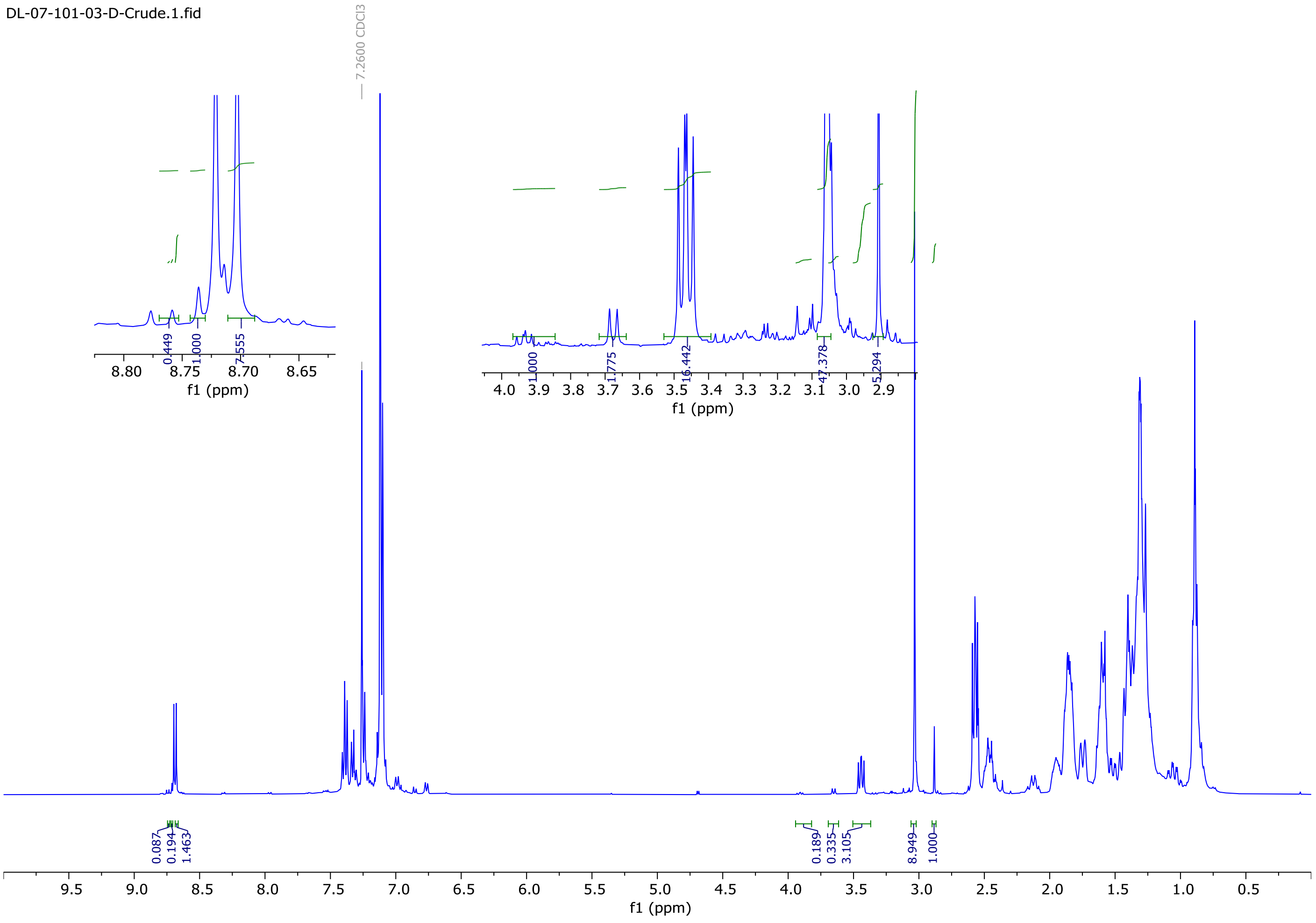

Figure S63. Crude ^{1}H-NMR before reduction of 34

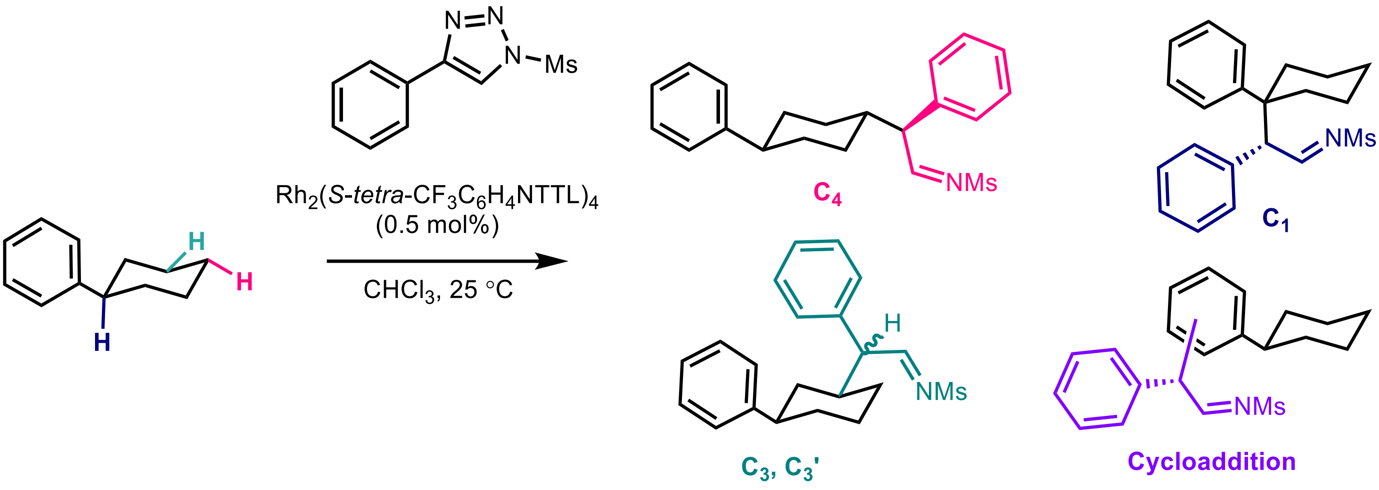

C_4:C_1 = 16:1
C_4:C_3 > 20:1
C_4:cycloaddition > 20:1

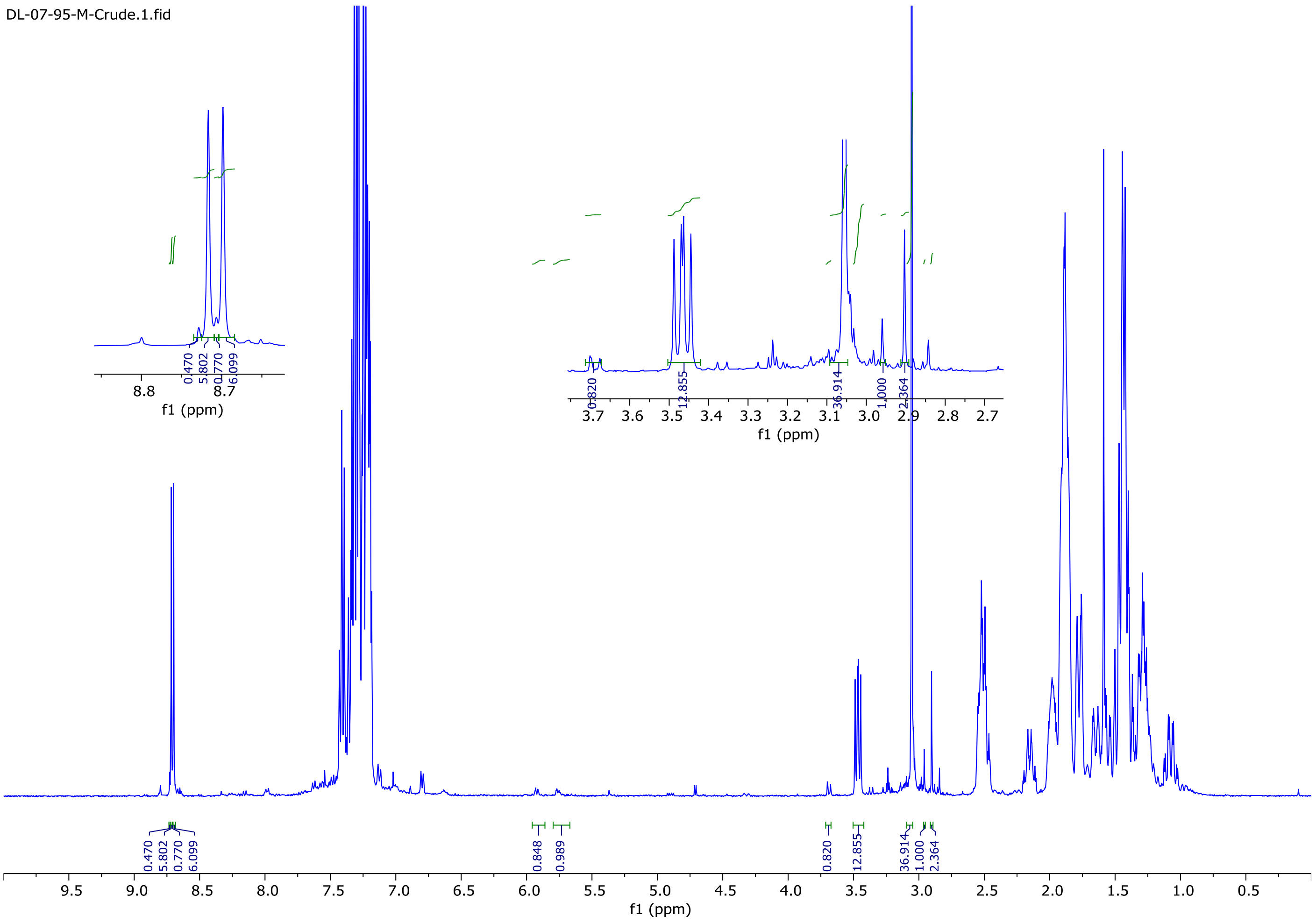

Figure S64. Crude ^{1}H-NMR before reduction of 35

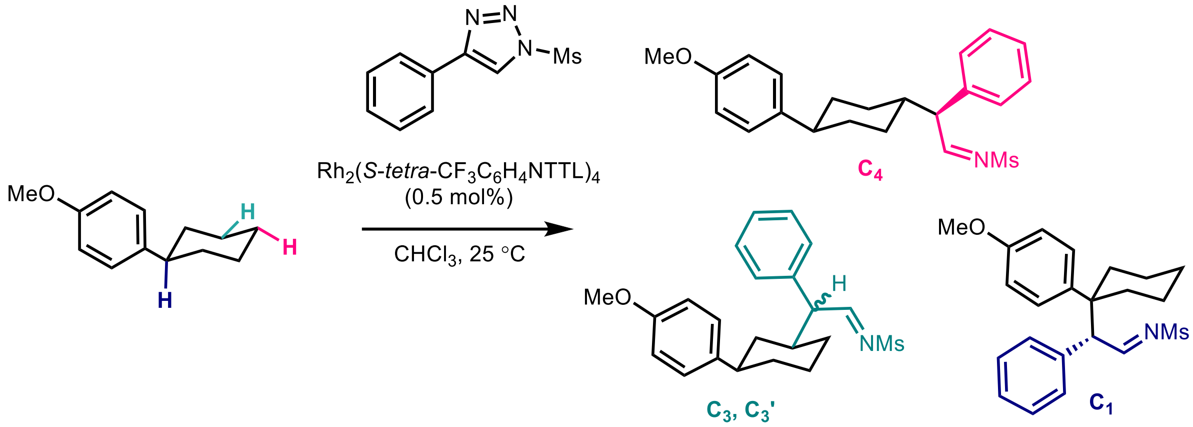

C_4:C_1 = 4.5:1
C_4:C_3 > 20:1

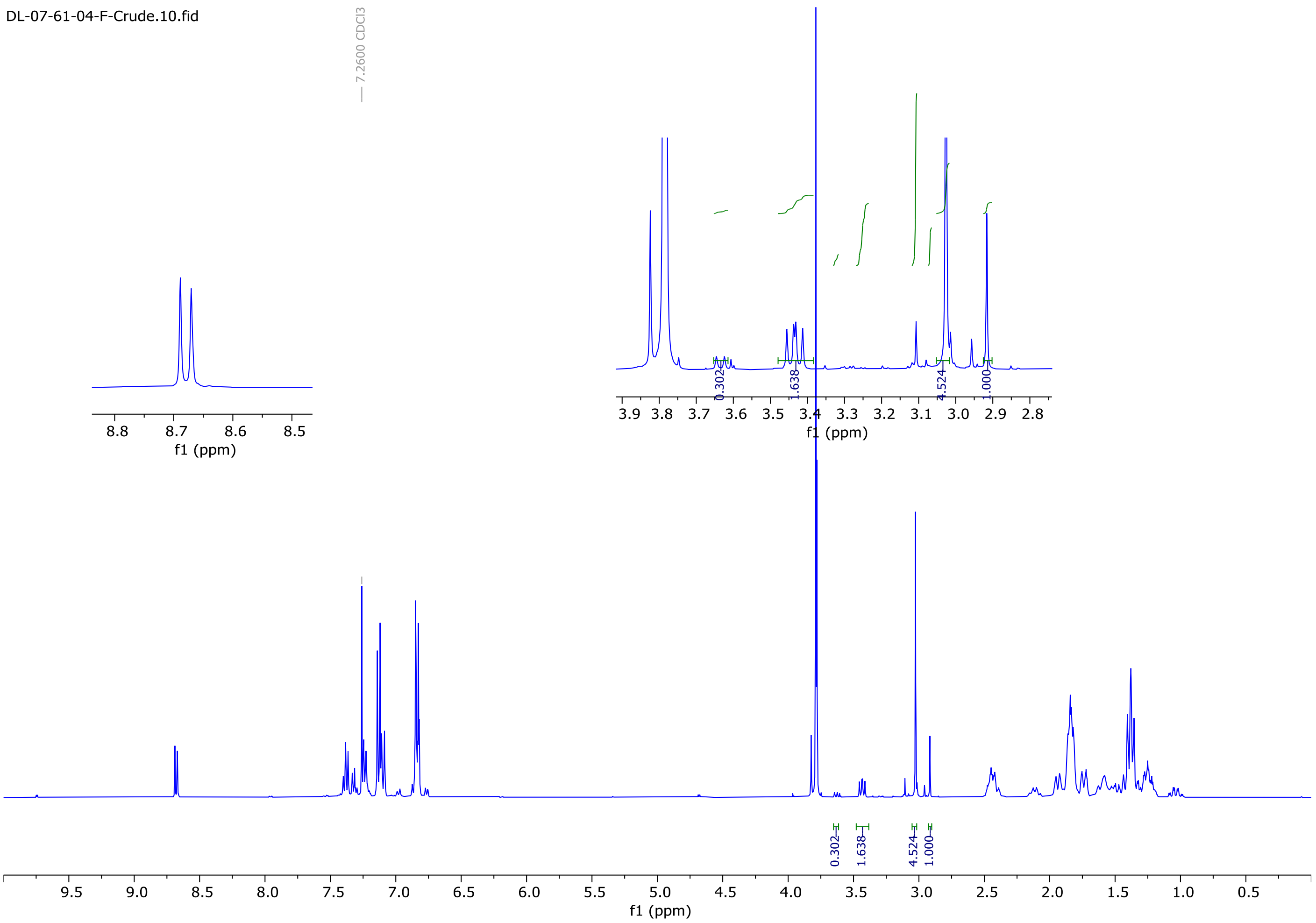

Figure S65. Crude ^{1}H-NMR before reduction of 36

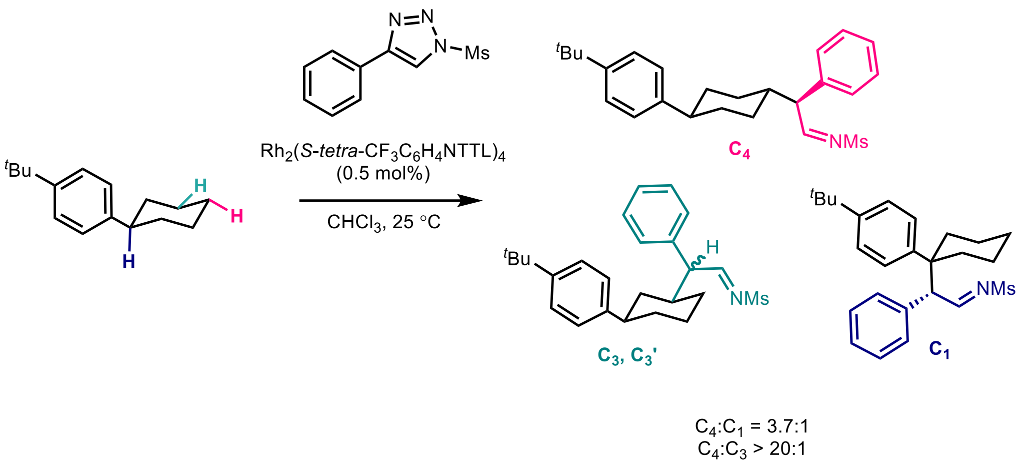

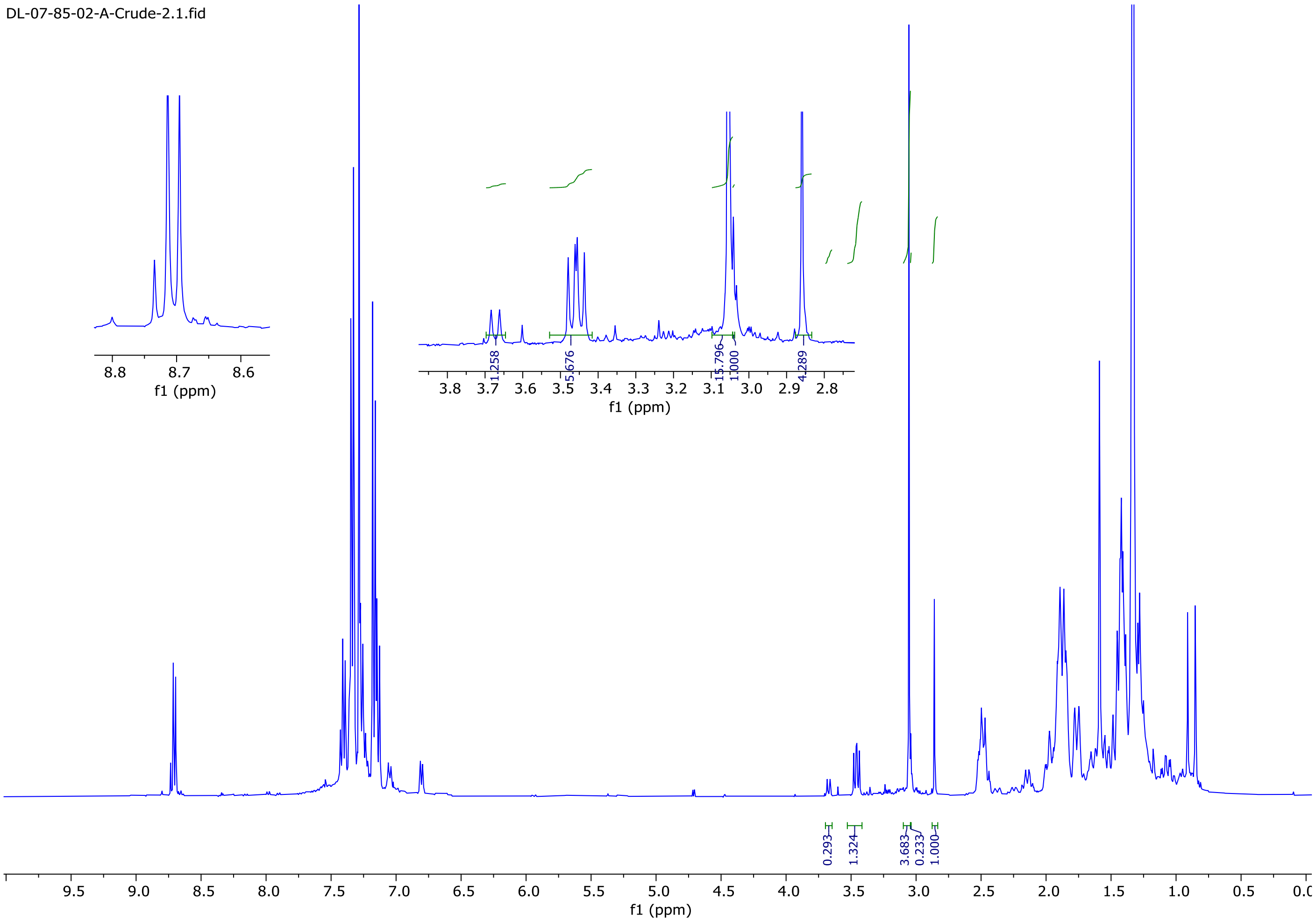

Figure S66. Crude ^{1}H-NMR before reduction of 37

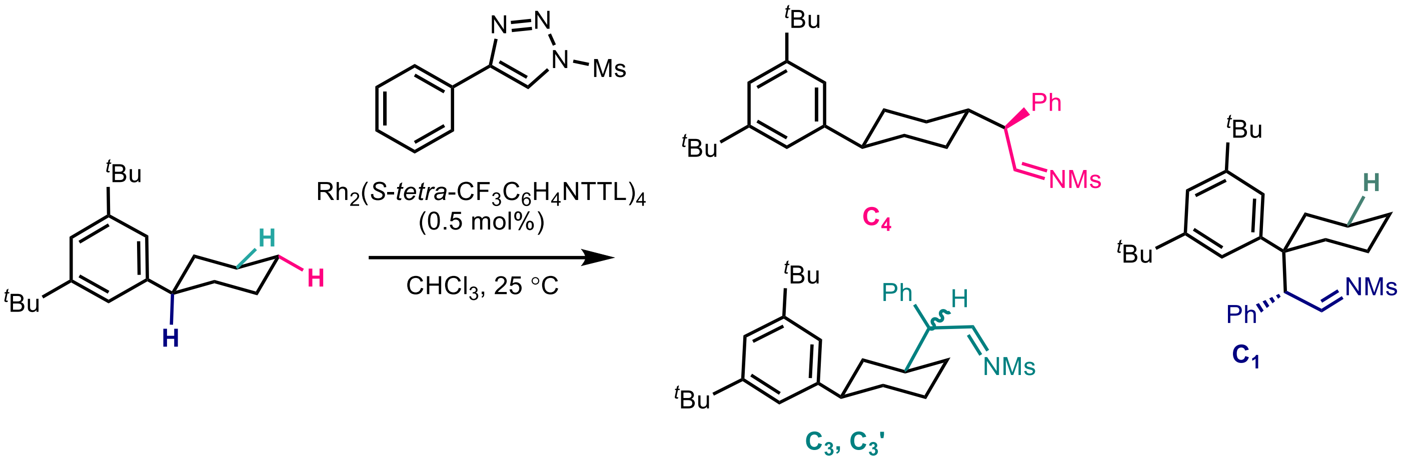

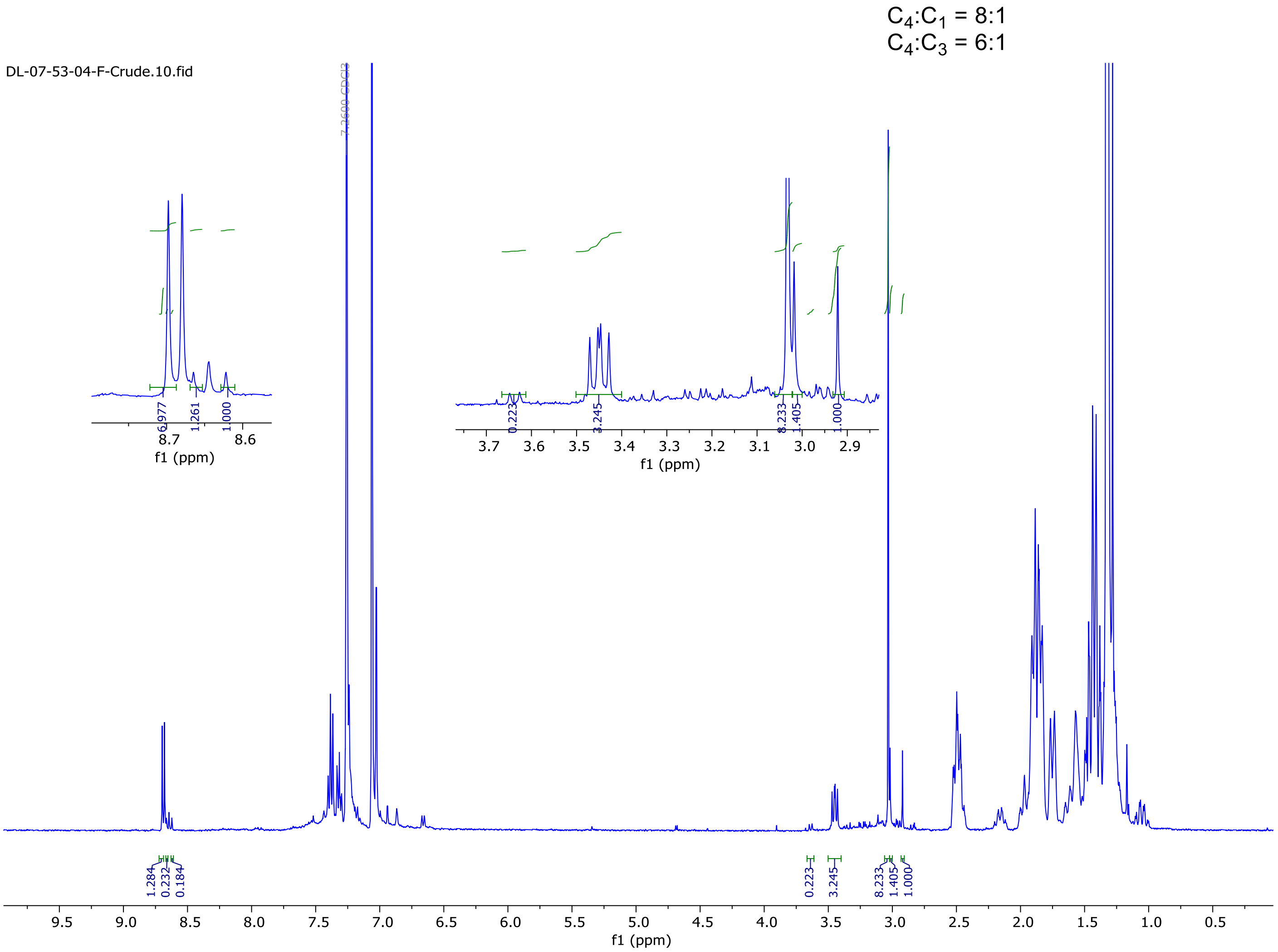

Figure S67. Crude ^{1}H-NMR before reduction of 38

8.2. Crude NMR for regio-selectivity determination of C1 selective C-H functionalization

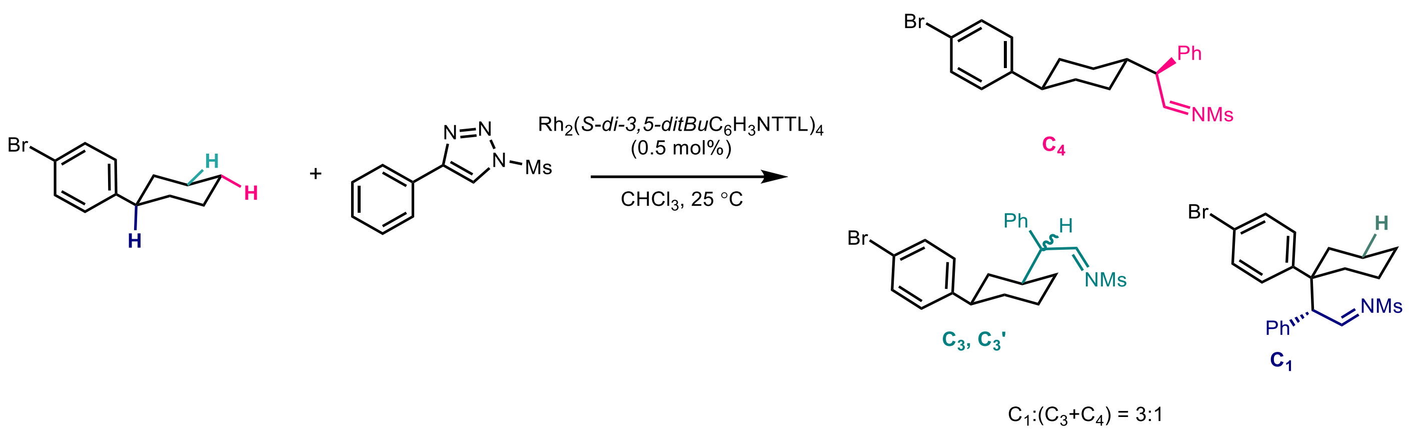

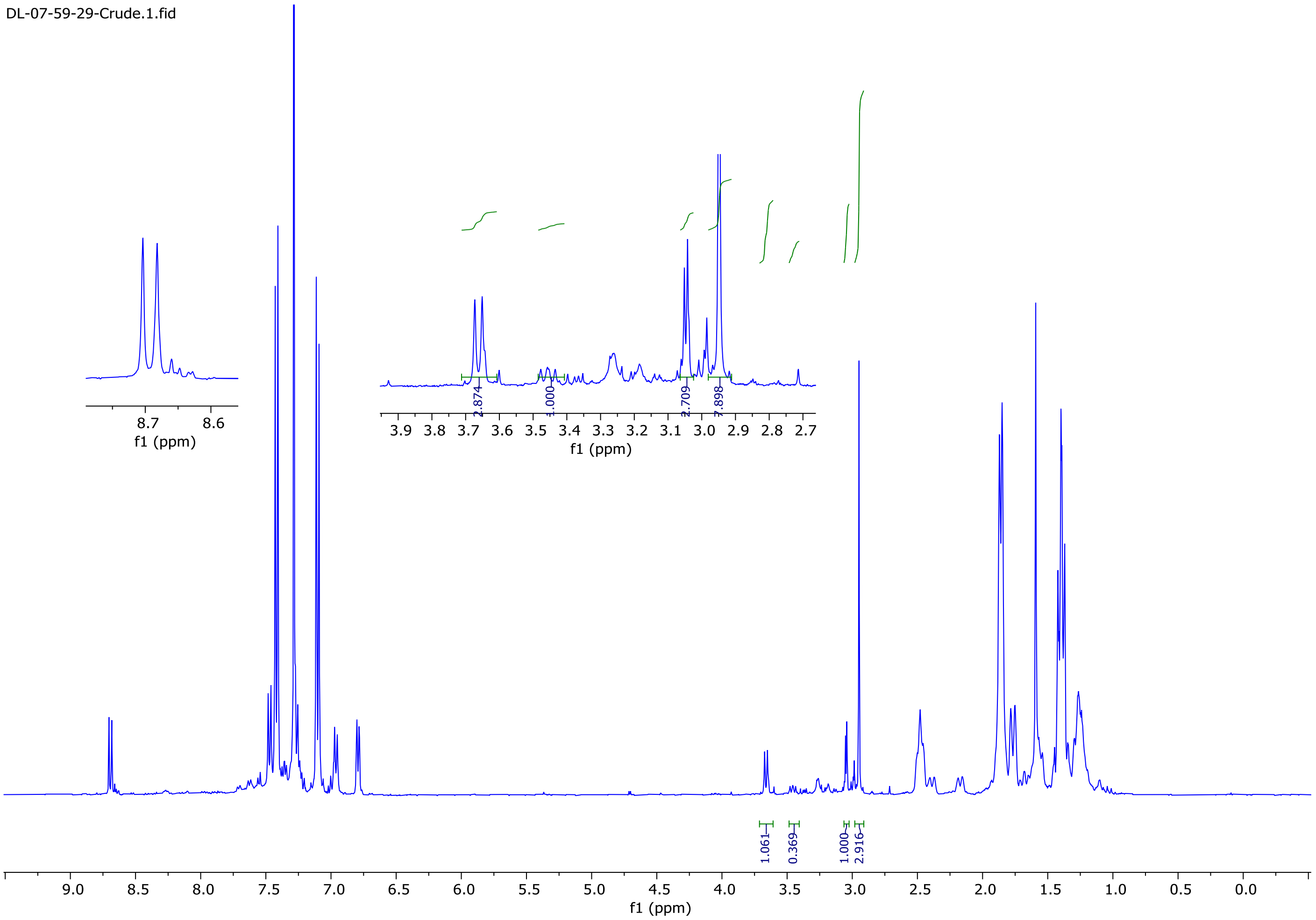

Figure S68. Crude ^{1}H-NMR before reduction of 14

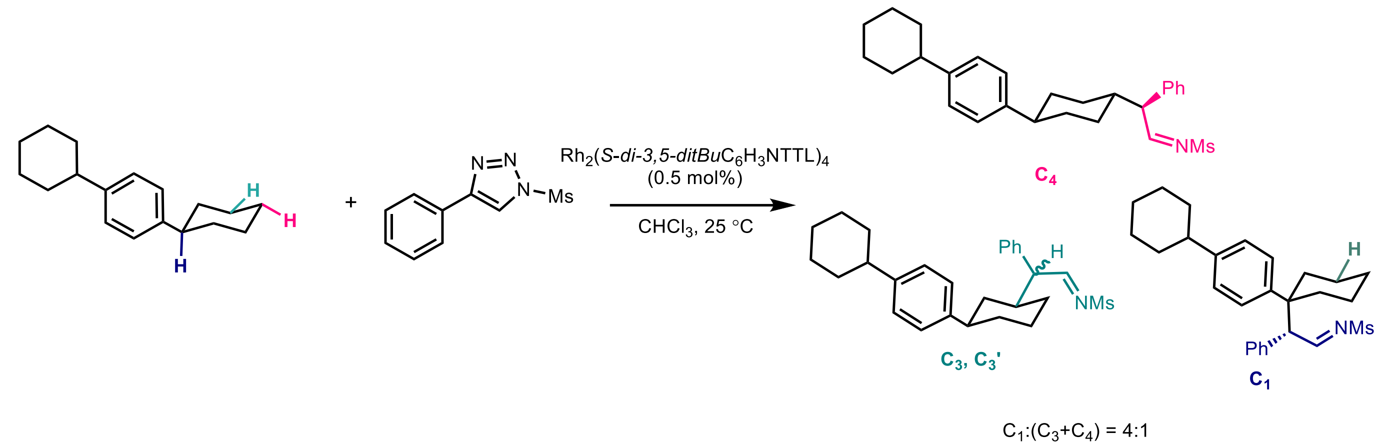

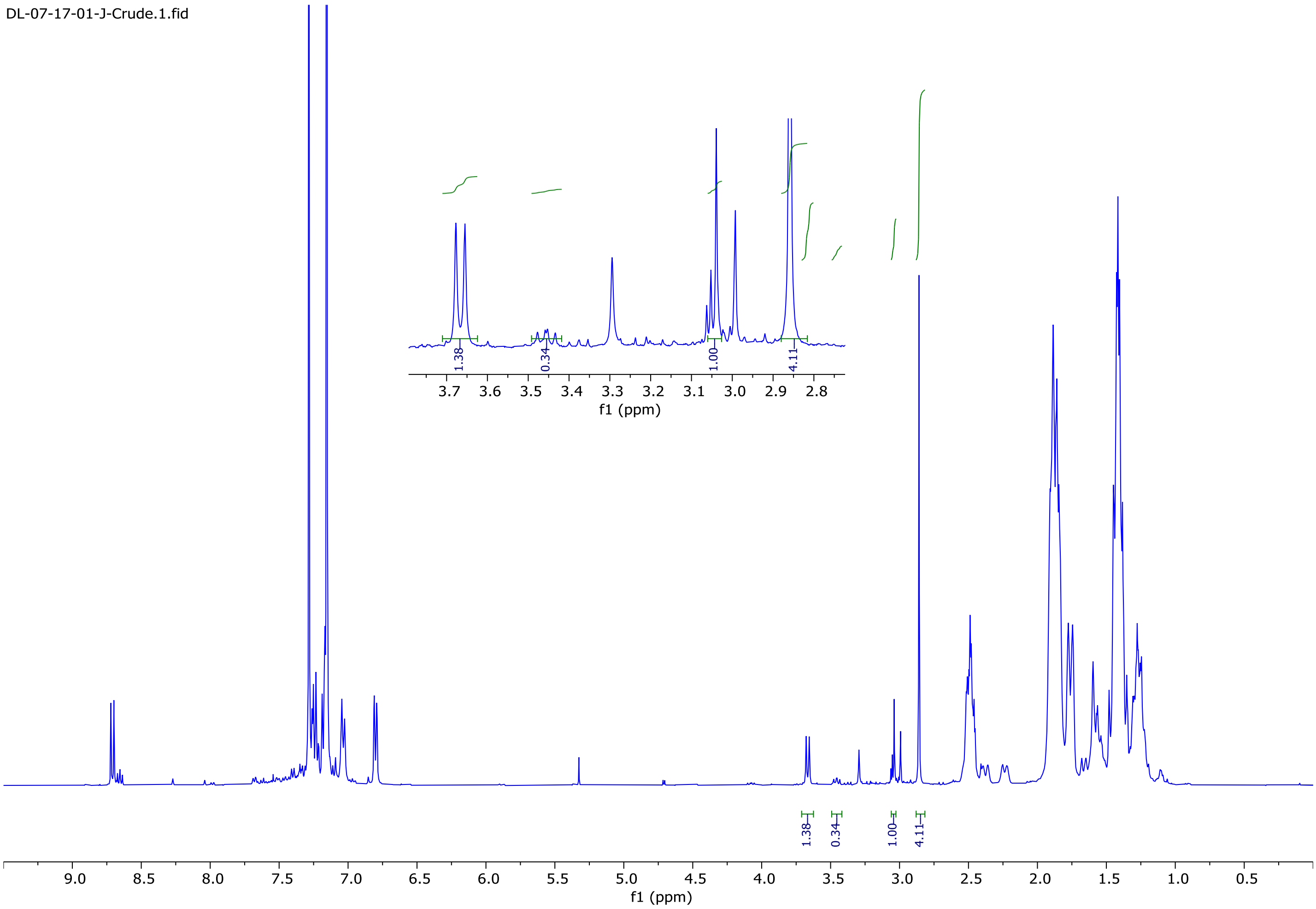

Figure S69. Crude ^{1}H-NMR before reduction of 39

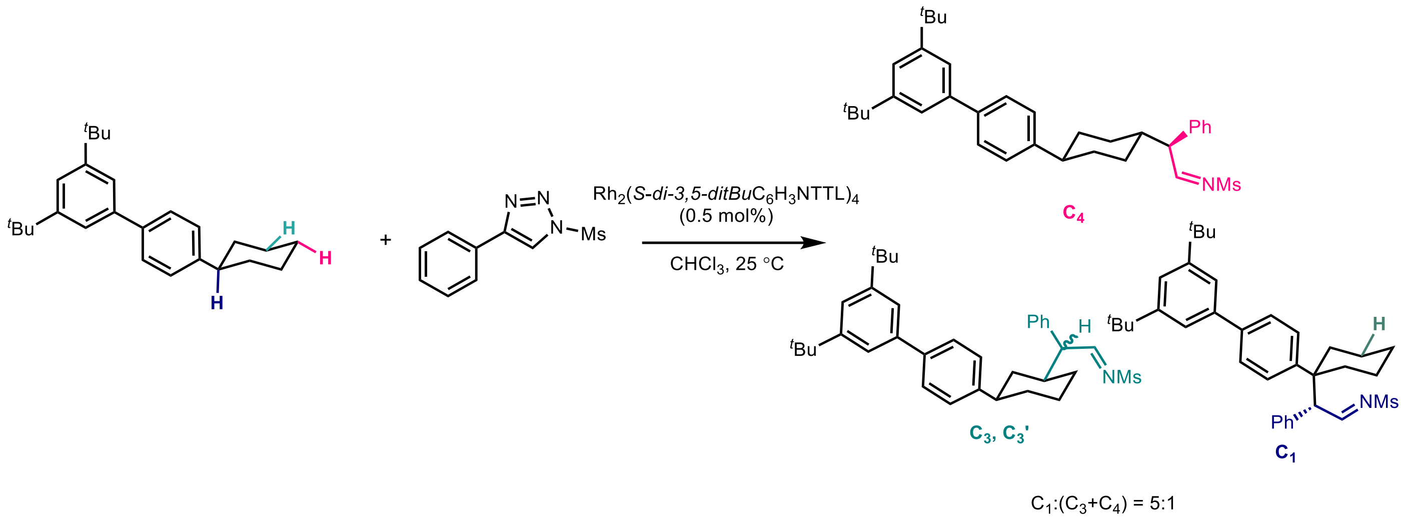

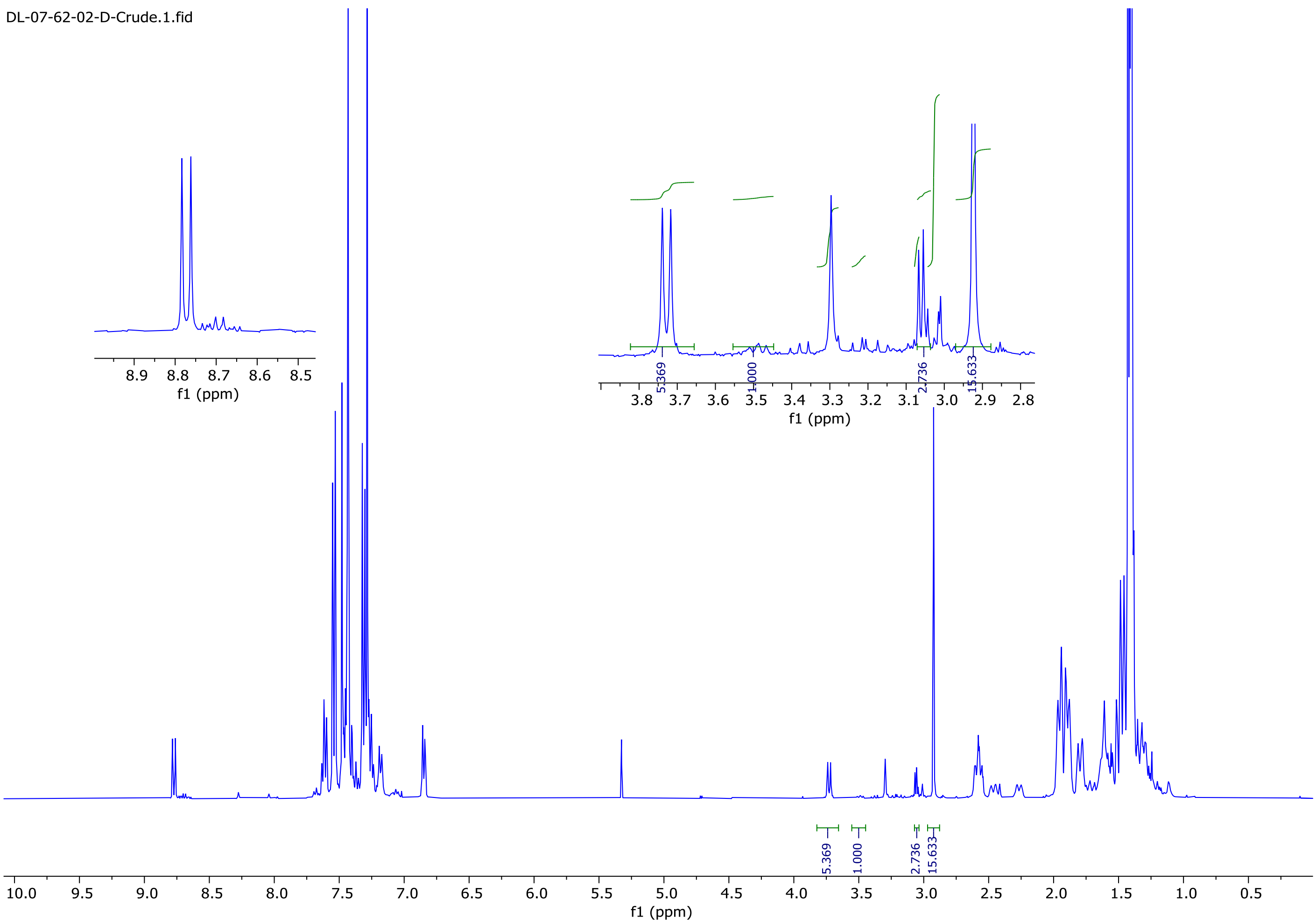

Figure S70. Crude ^{1}H-NMR before reduction of 40

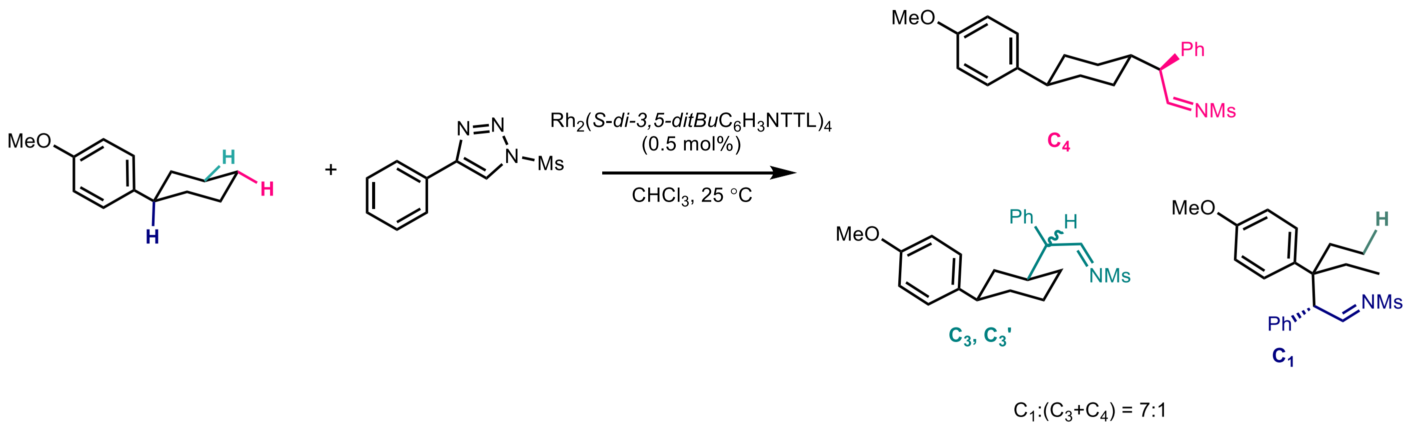

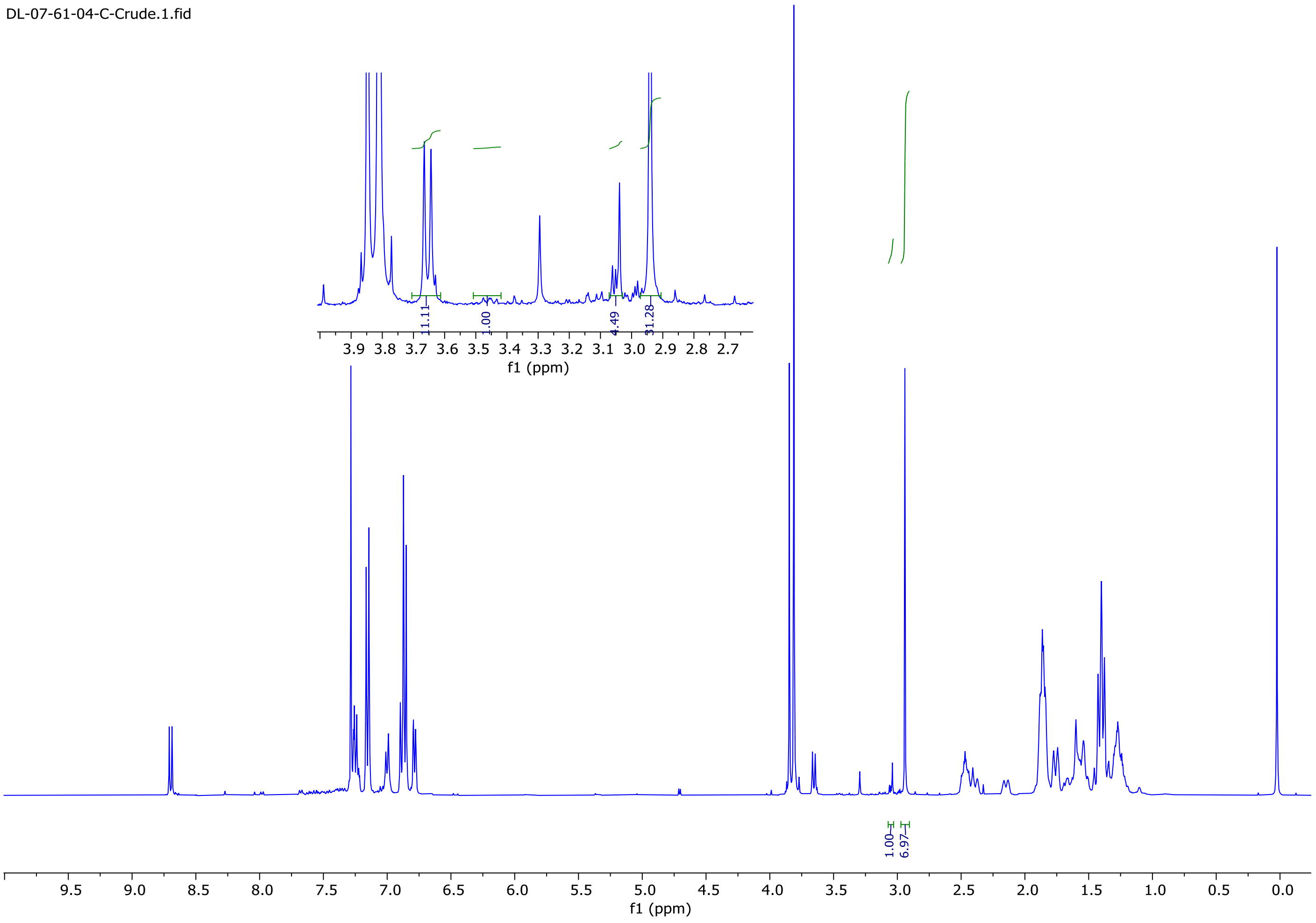

Figure S71. Crude ^{1}H-NMR before reduction of 41

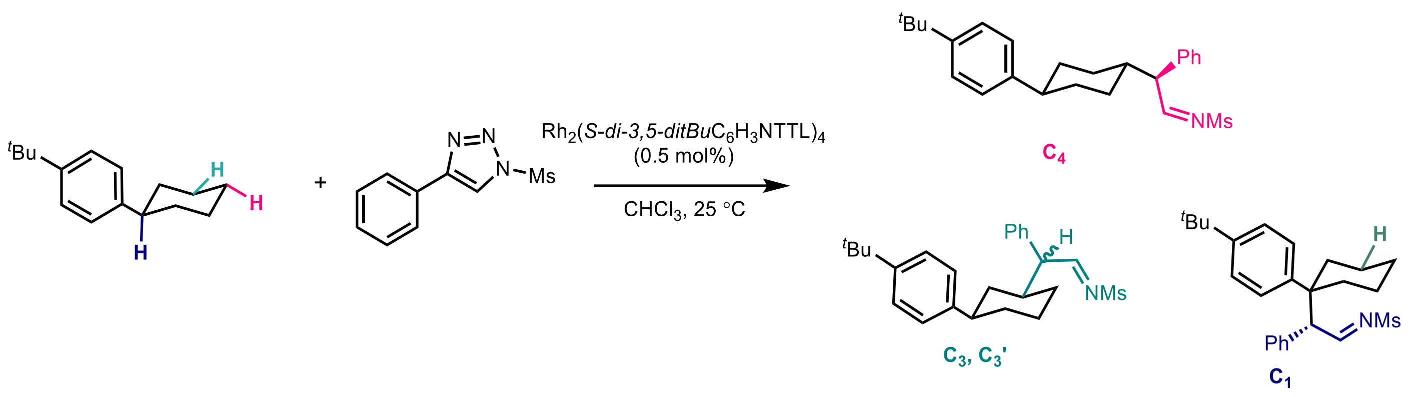

C1:(C3+C4) = 5:1

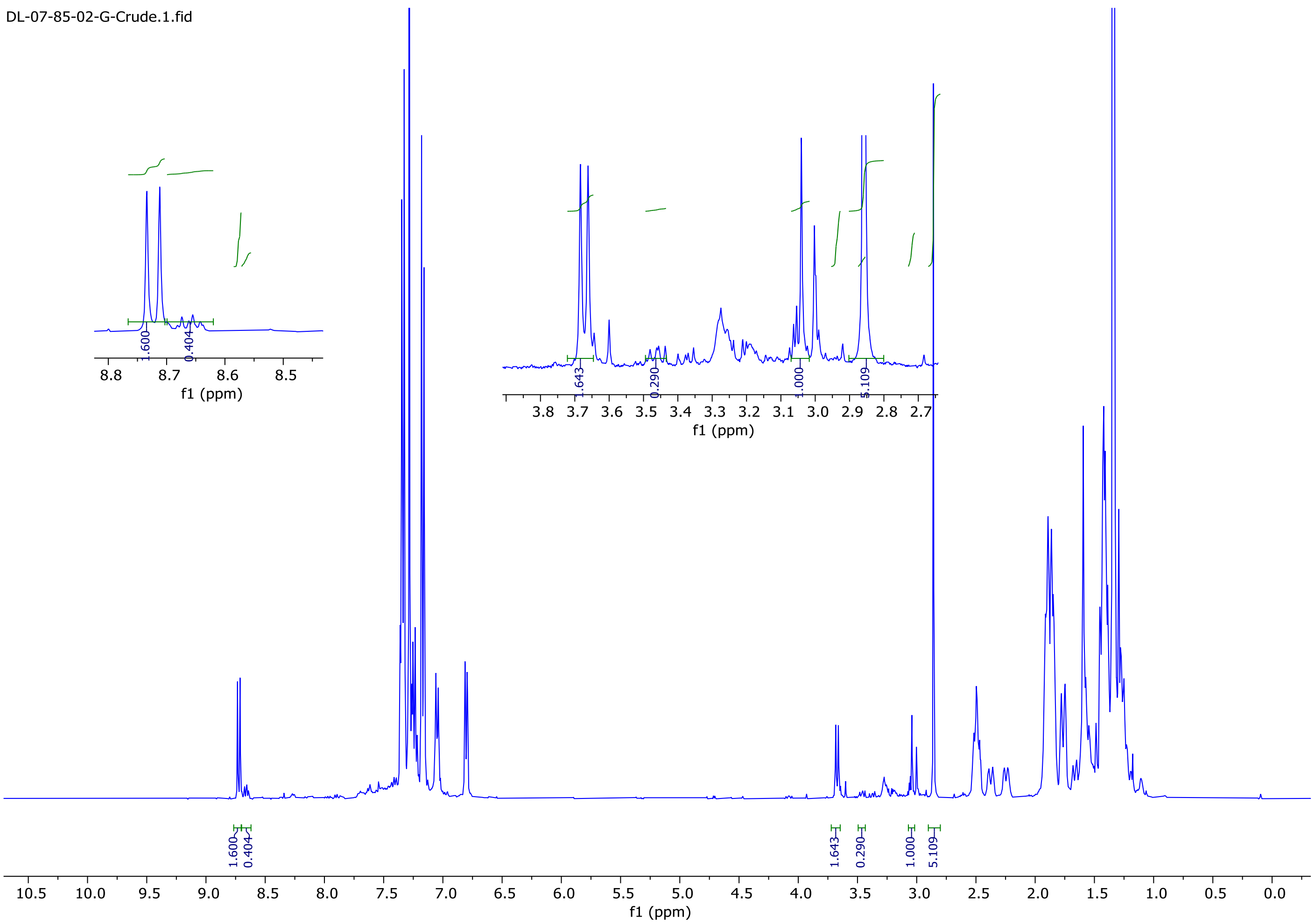

Figure S72. Crude ^{1}H-NMR before reduction of 42

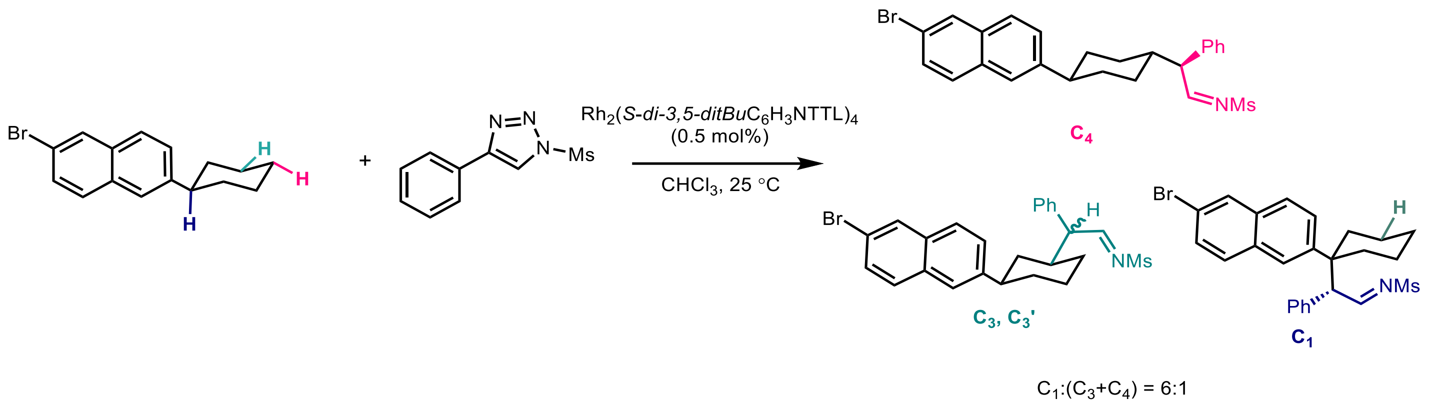

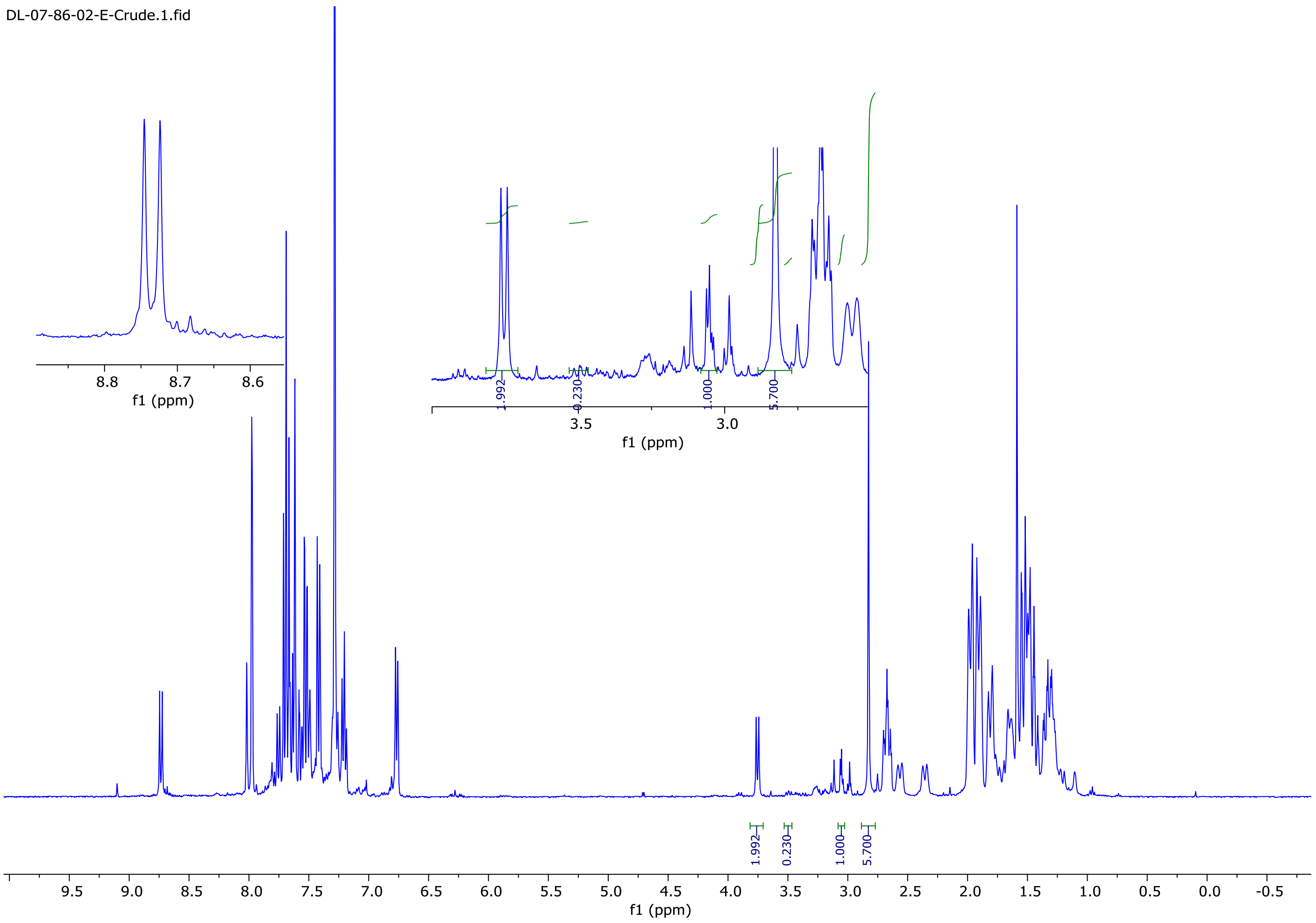

Figure S73. Crude ^{1}H-NMR before reduction of 43

8.3. Crude NMR for extra examples

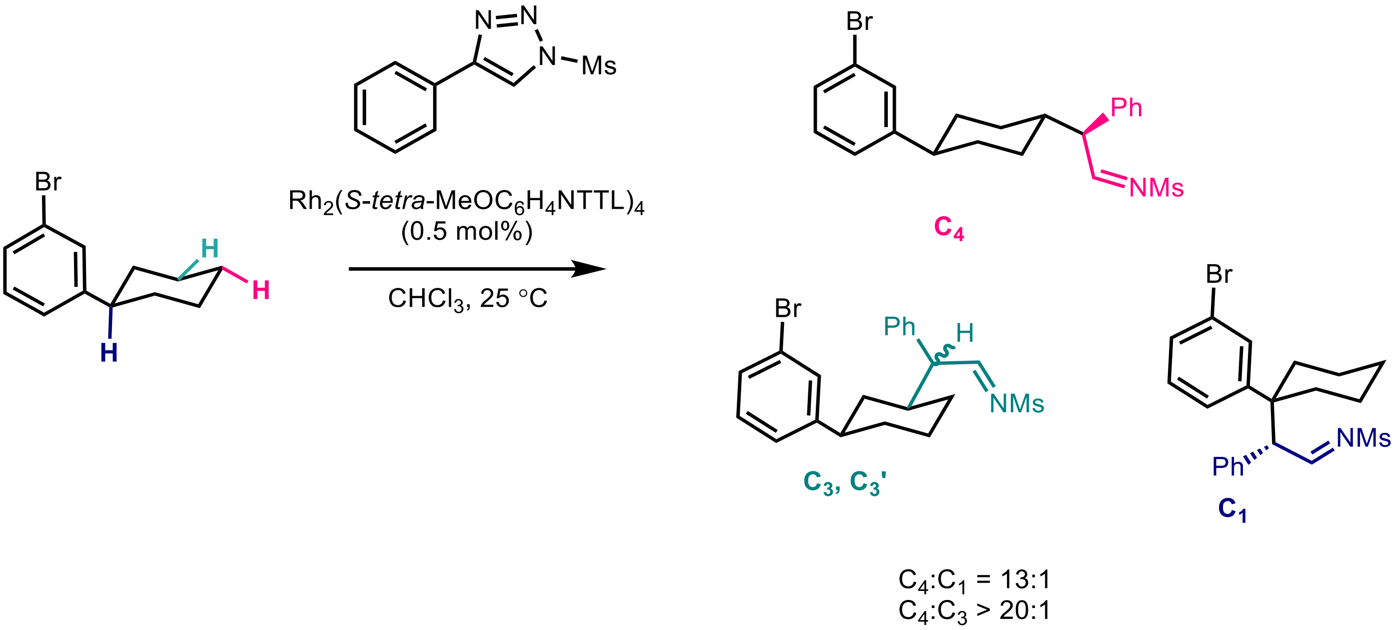

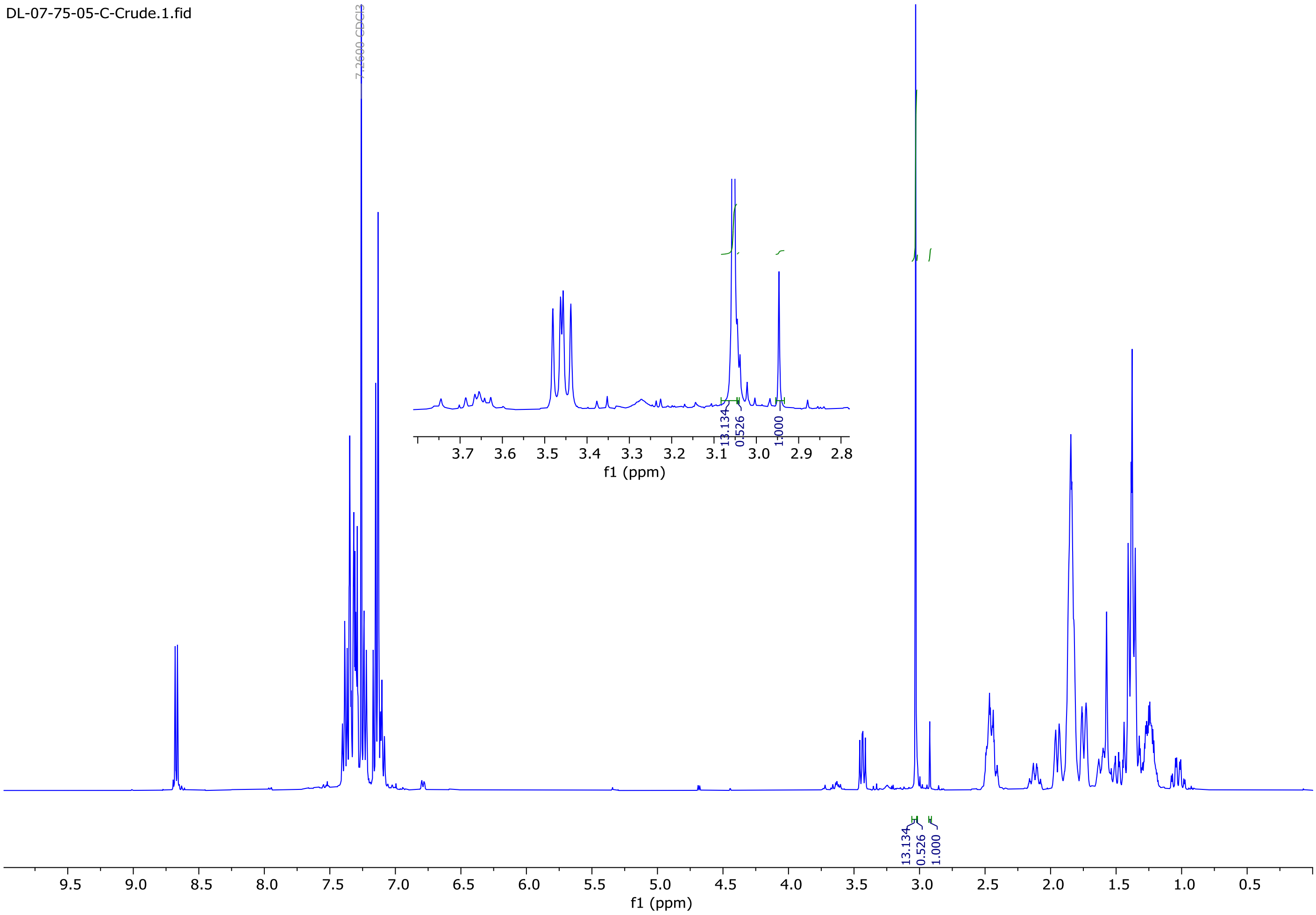

Figure S74. Crude ^{1}H-NMR before reduction of 44

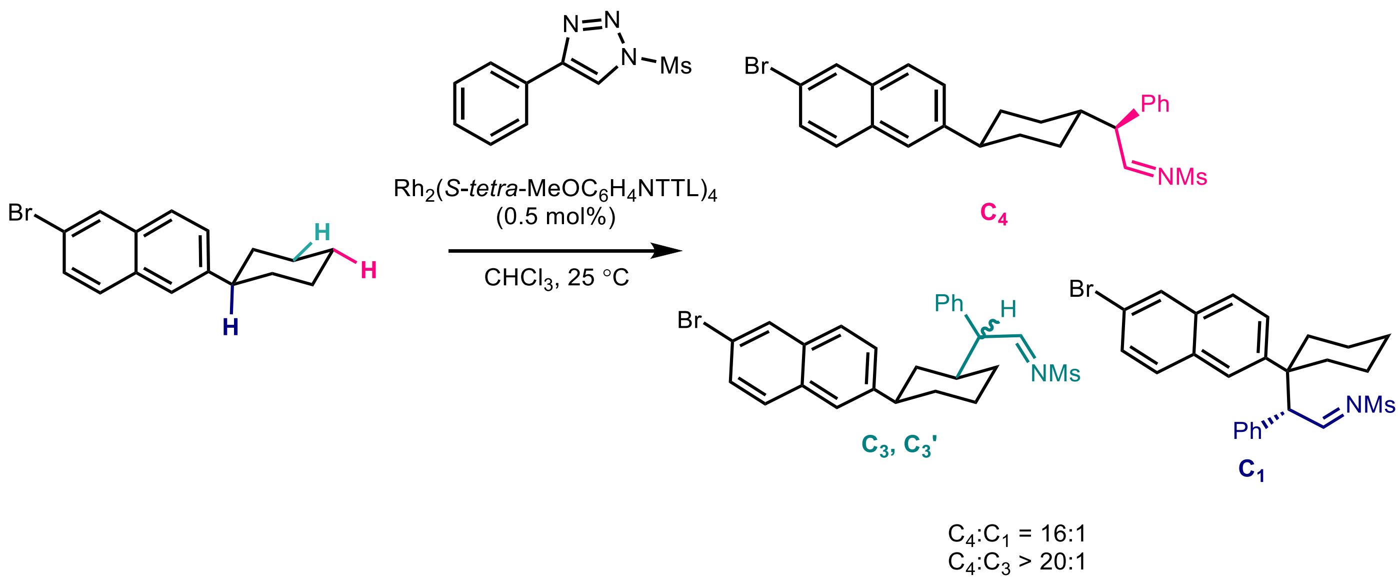

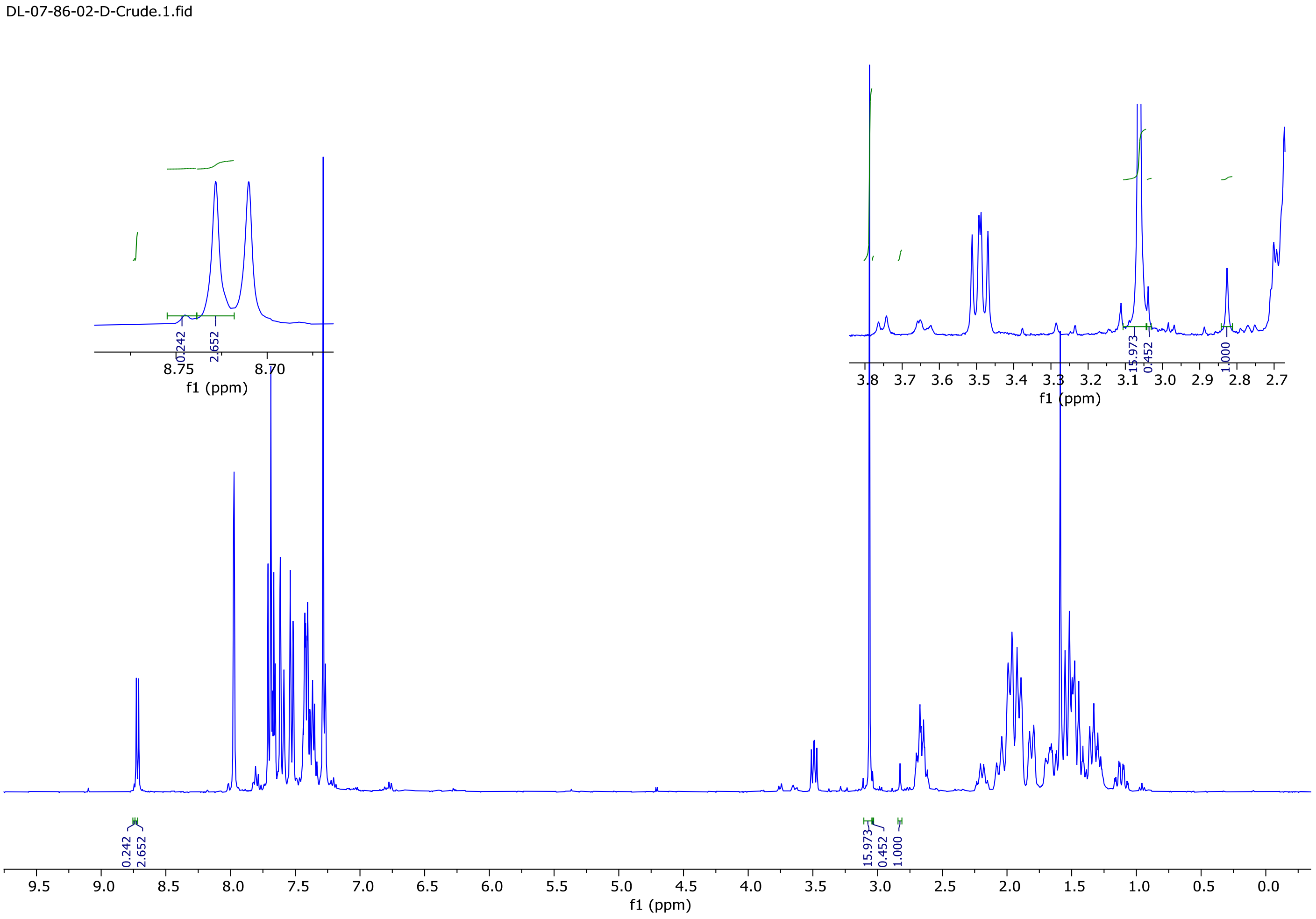

Figure S75. Crude ^{1}H-NMR before reduction of 45

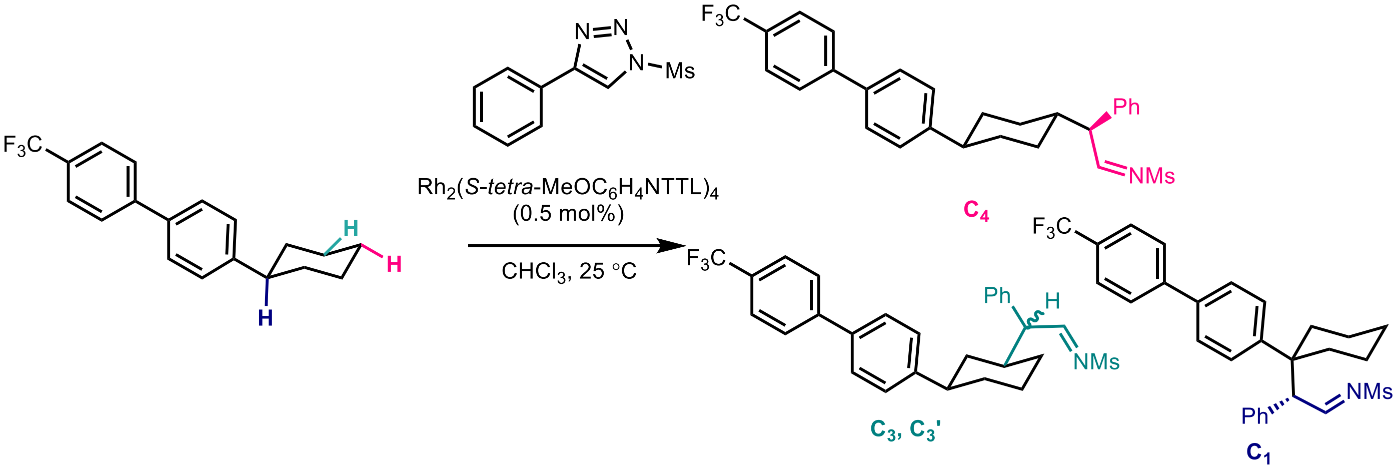

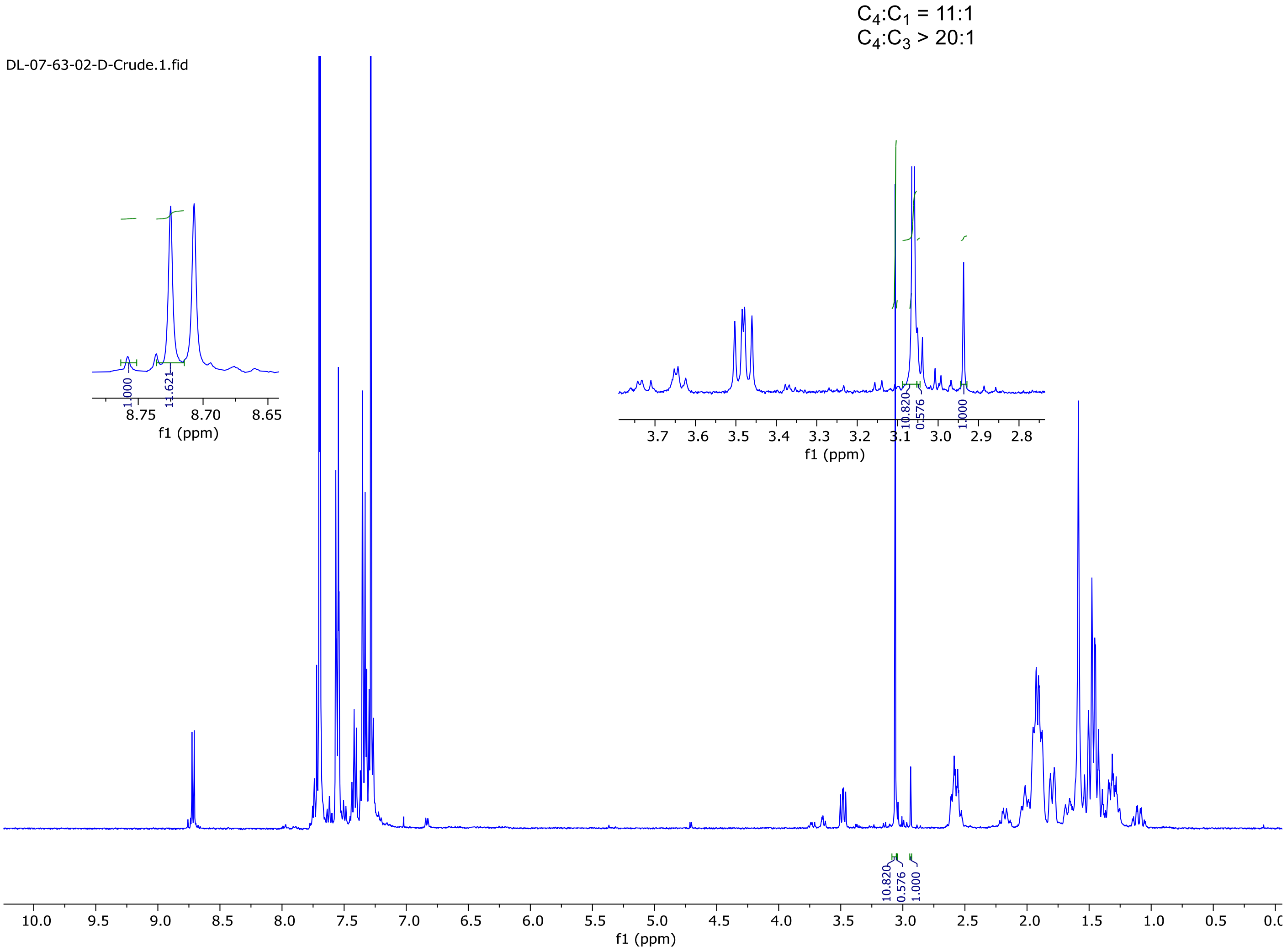

Figure S76. Crude ^{1}H-NMR of 46

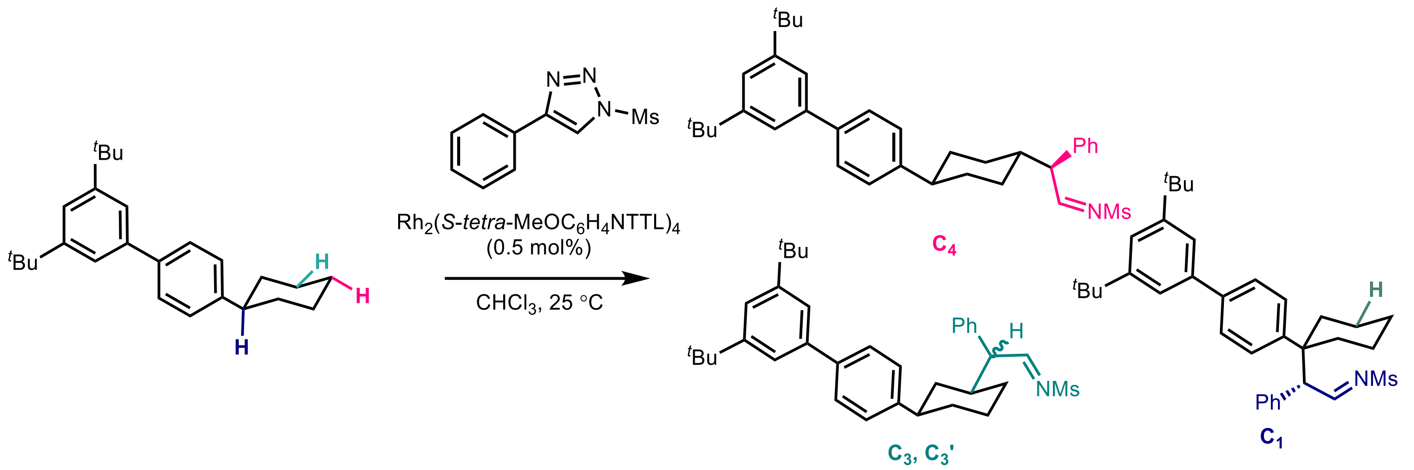

C$_4$:C$_1$ = 5:1
C$_4$:C$_3$ > 20:1

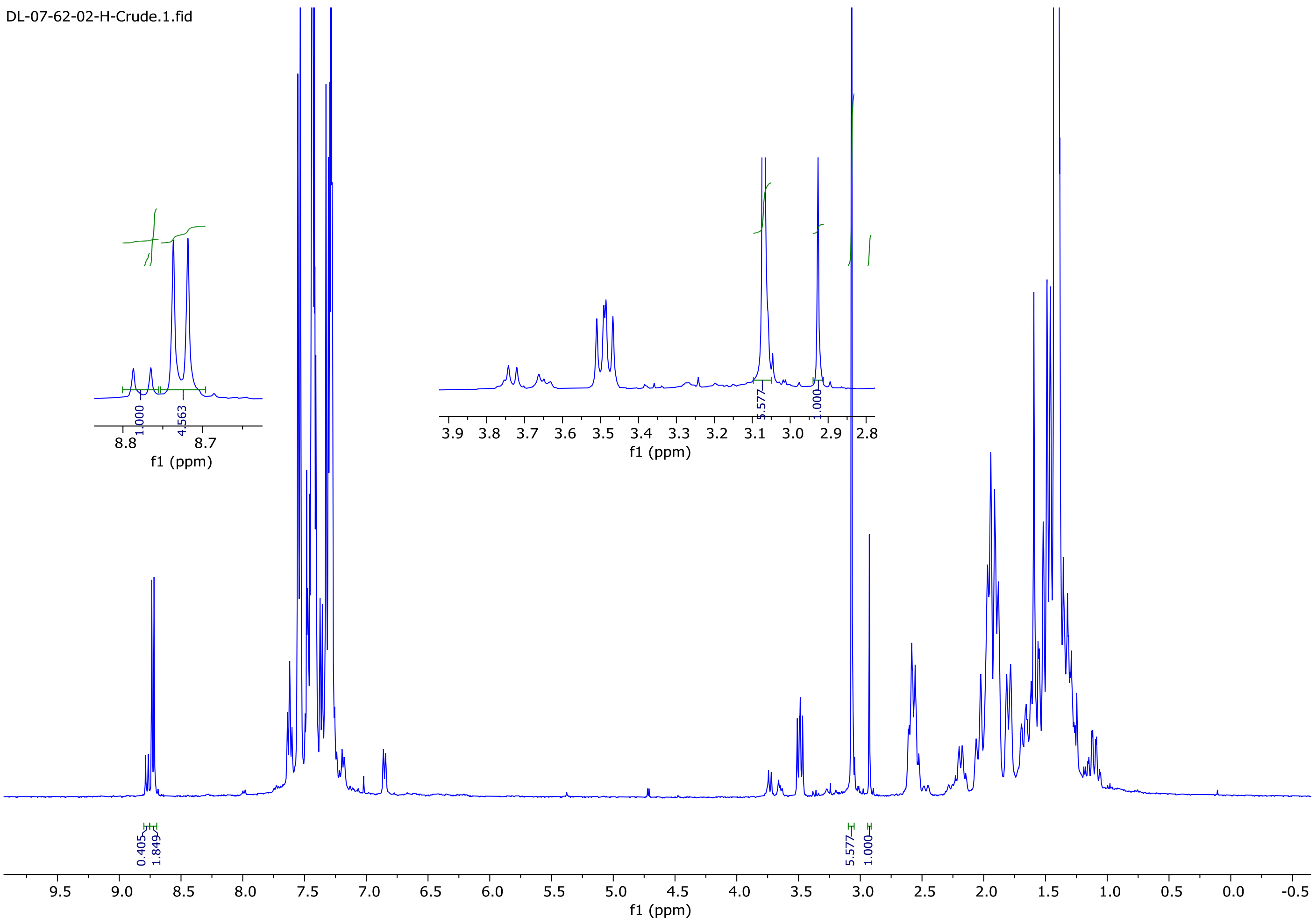

Figure S77. Crude ^{1}H-NMR before reduction of 47

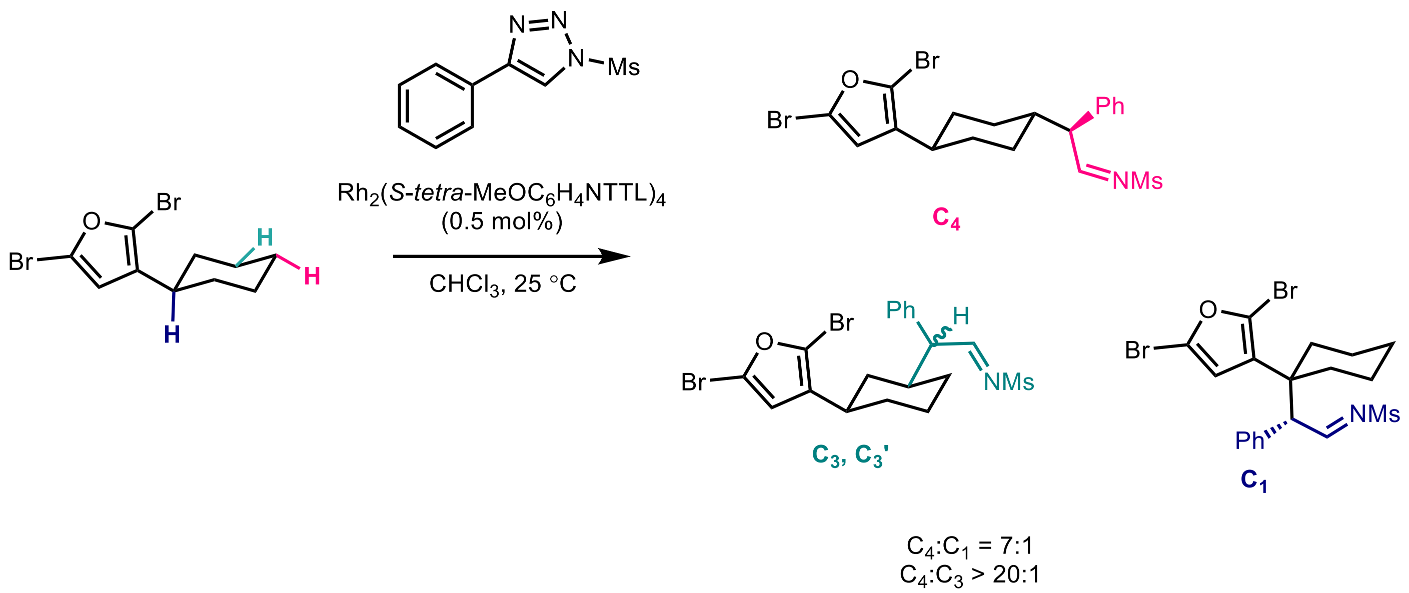

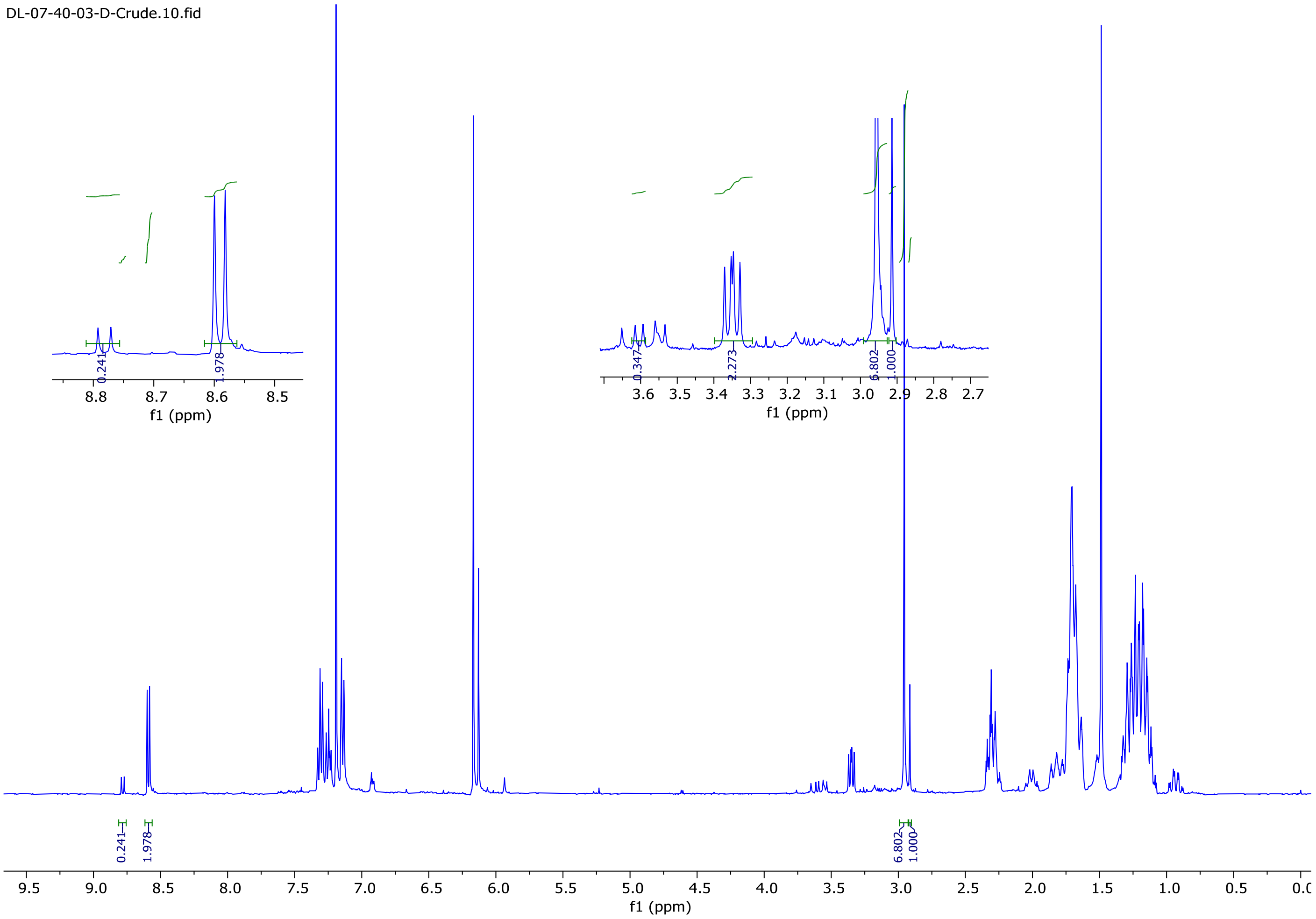

Figure S78. Crude ^{1}H-NMR before reduction of 48

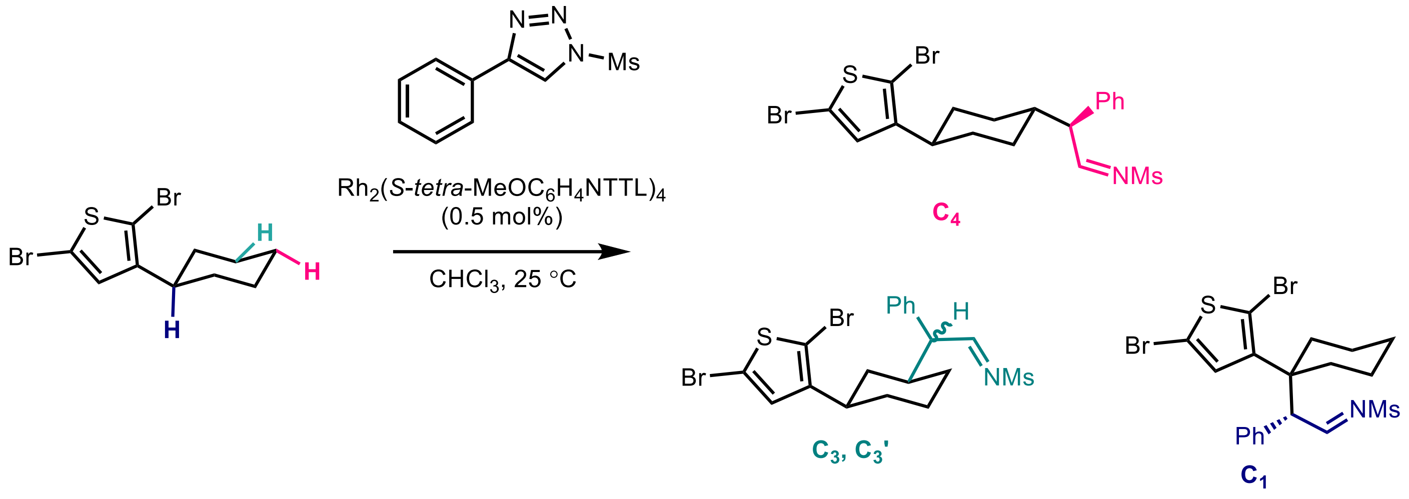

C_4:C_1 = na
C_4:C_3 = 14:1

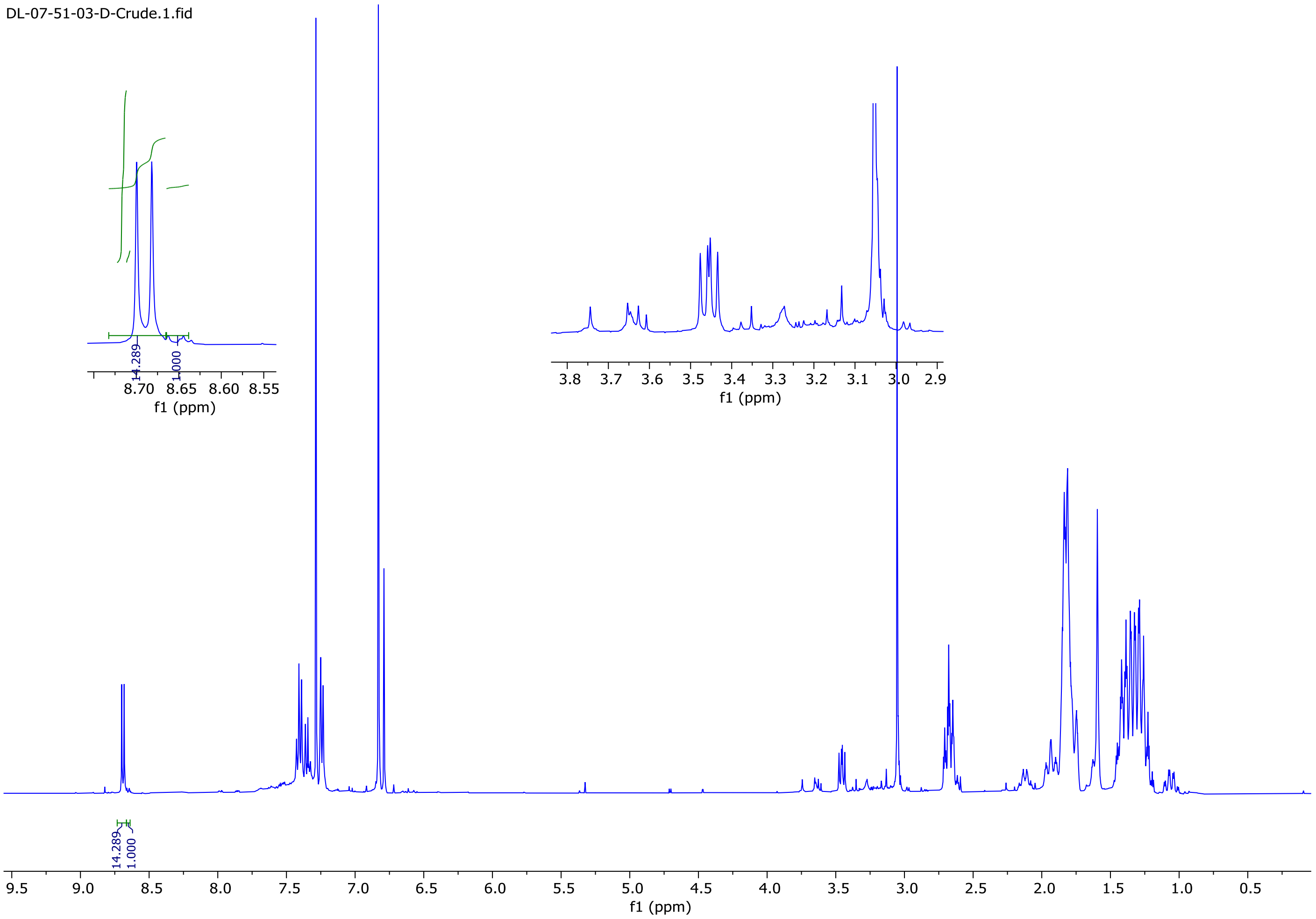

Figure S79. Crude ^{1}H-NMR before reduction of 49. The imine region was used in this case because the methyl of Ms group was not well resolved.

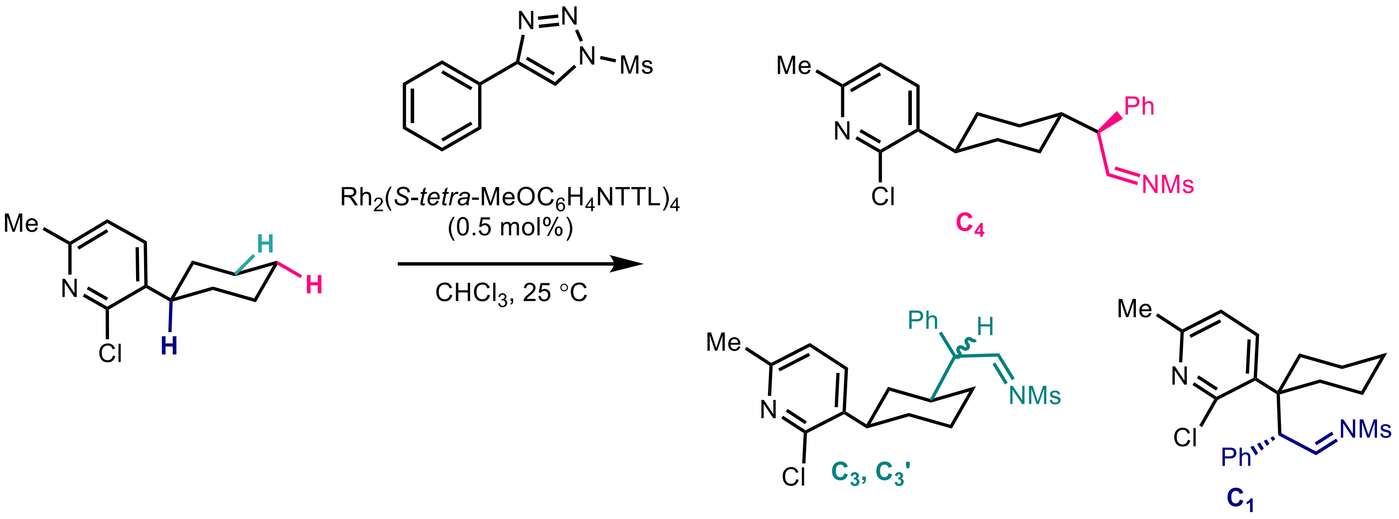

C_4:C_1 = na
C_4:C_3 = 8:1

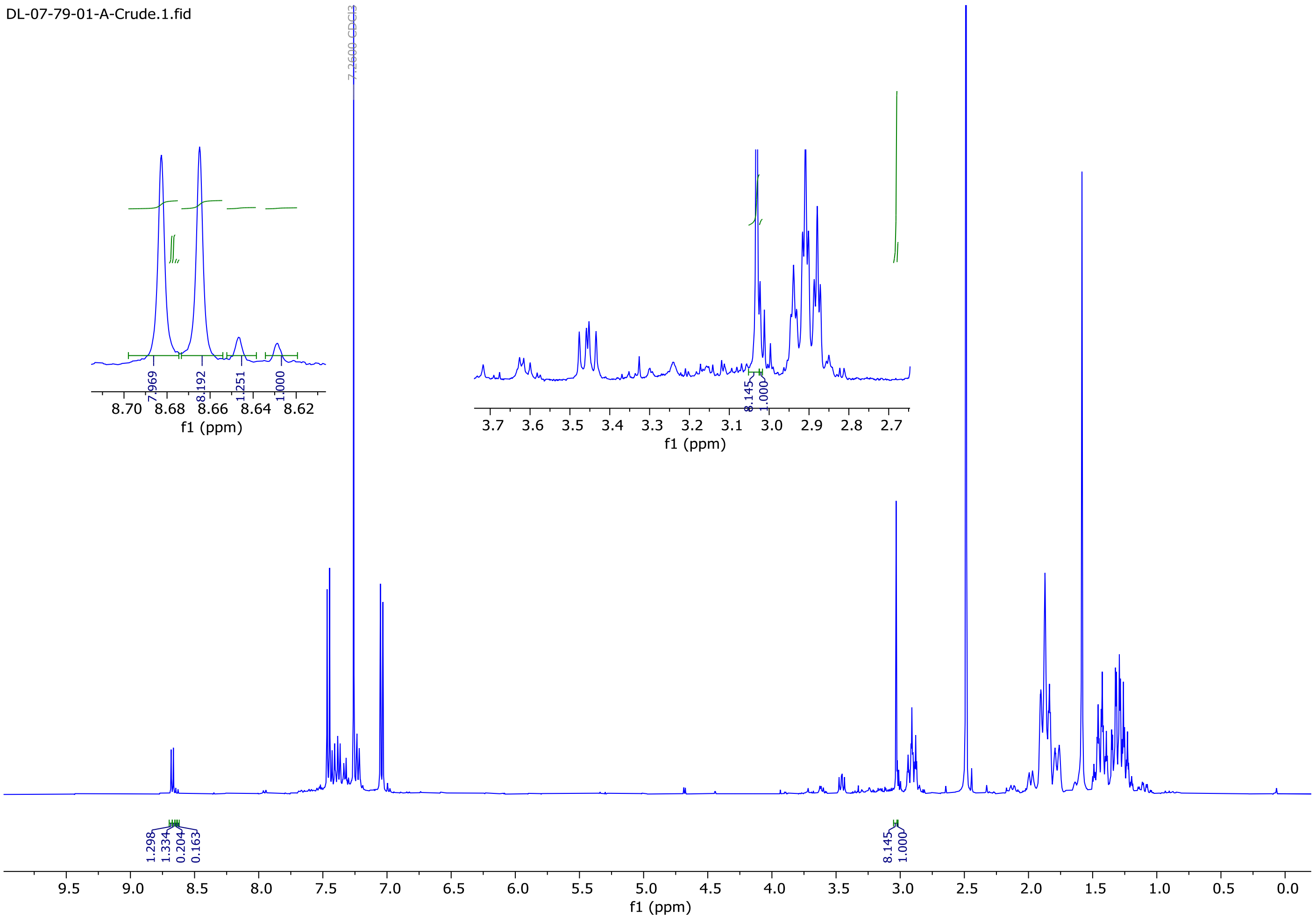

Figure S80. Crude ^{1}H-NMR before reduction of 50

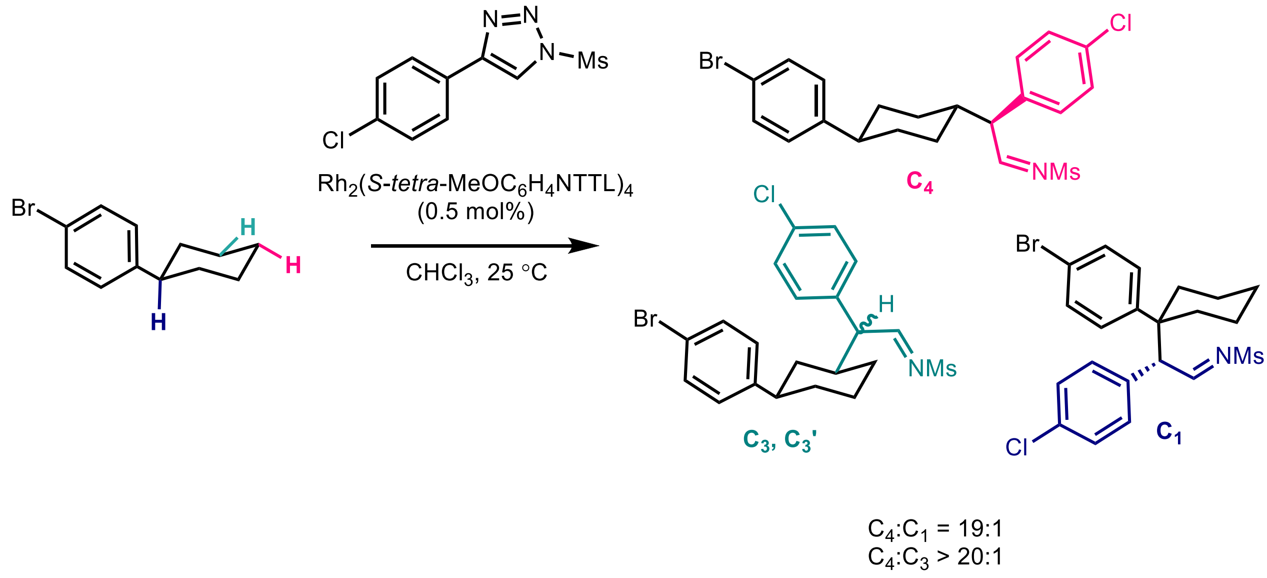

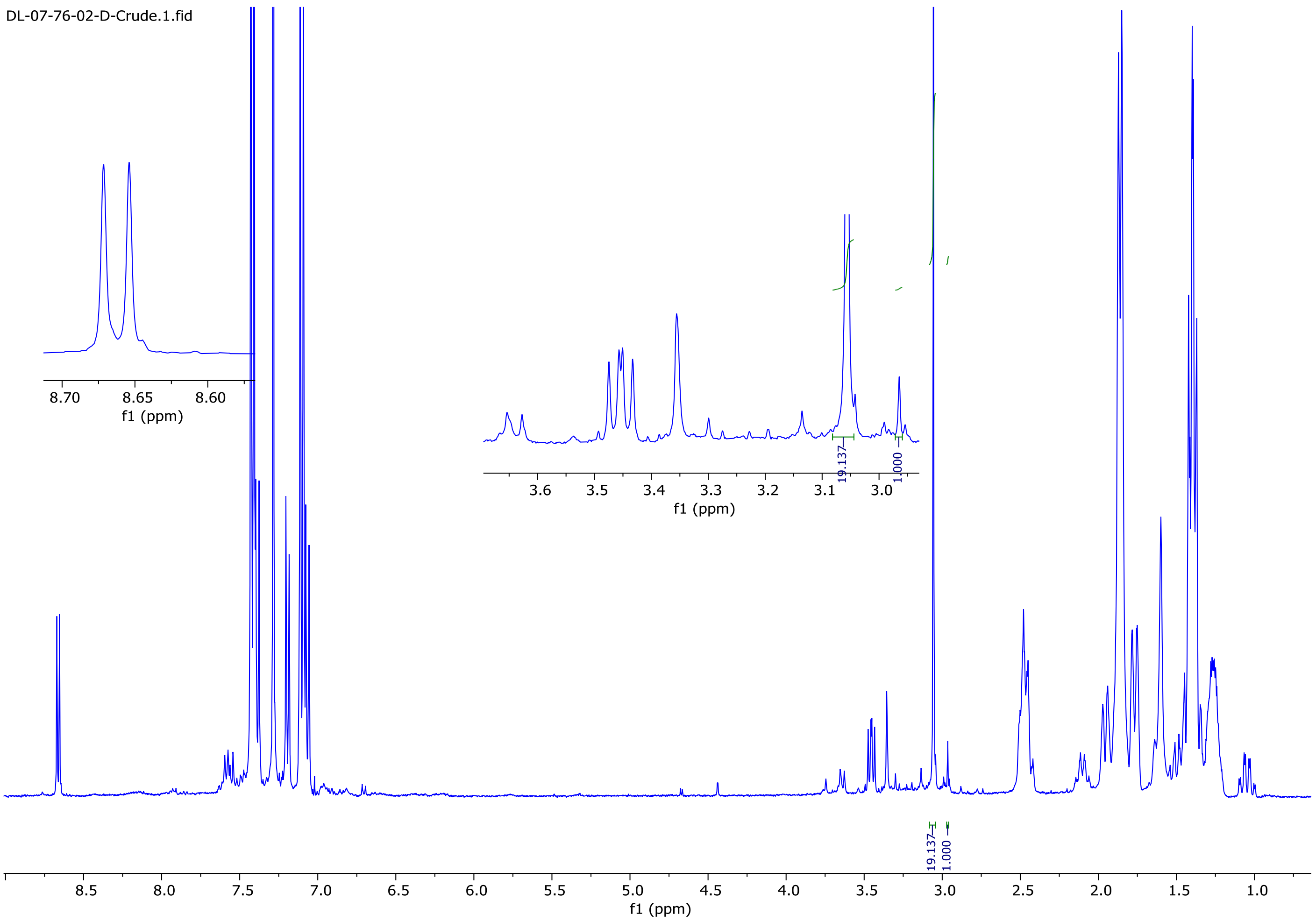

Figure S81. Crude ^{1}H-NMR before reduction of 51

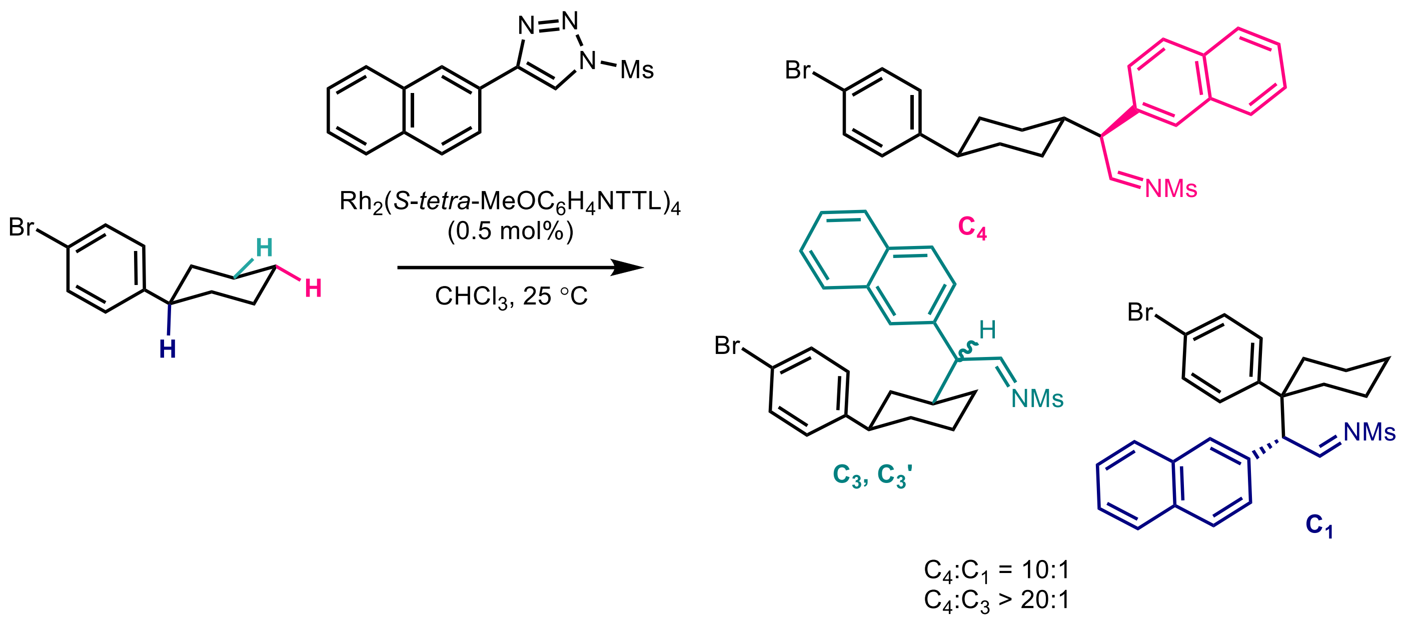

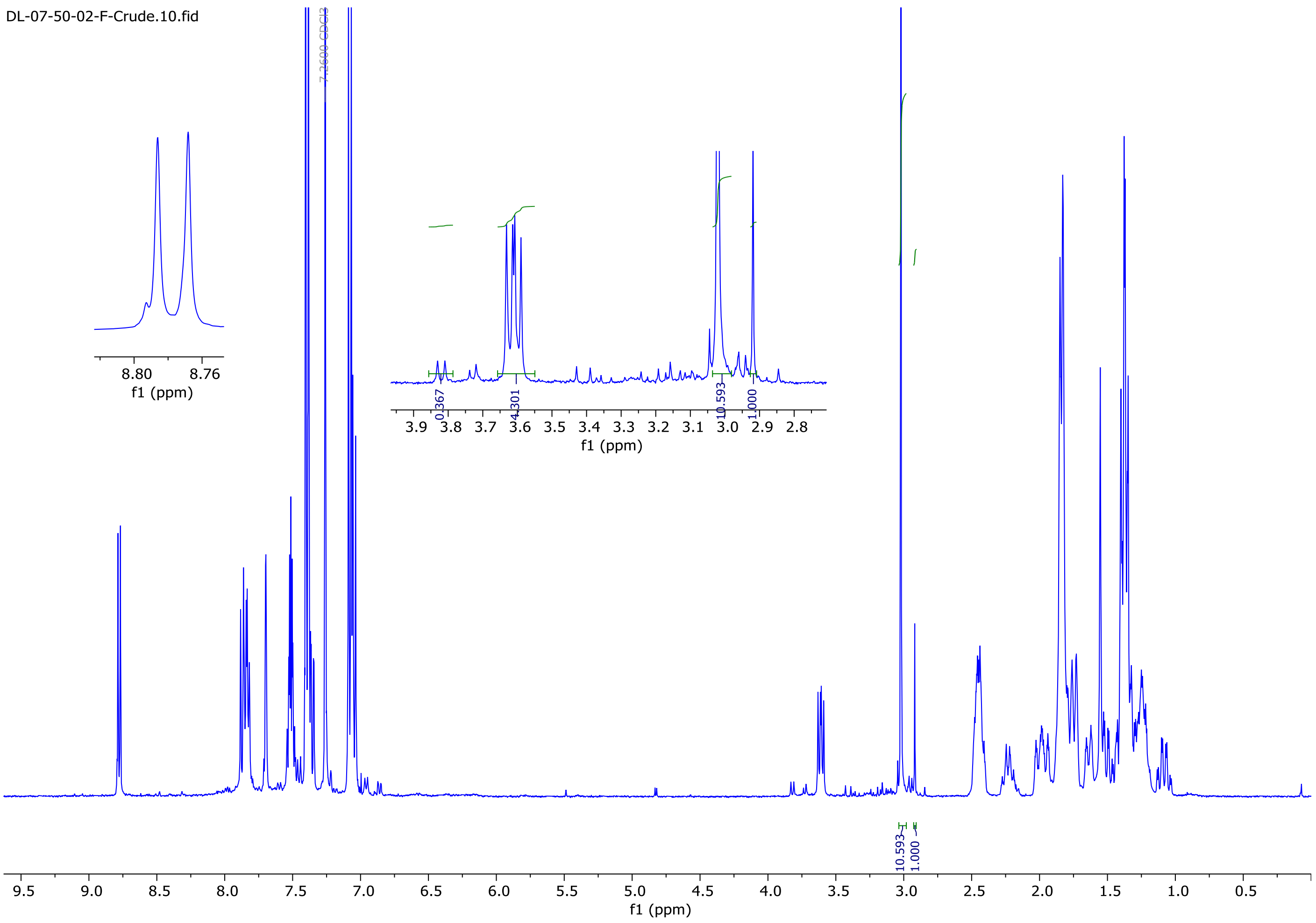

Figure S82. Crude ^{1}H-NMR before reduction of 52

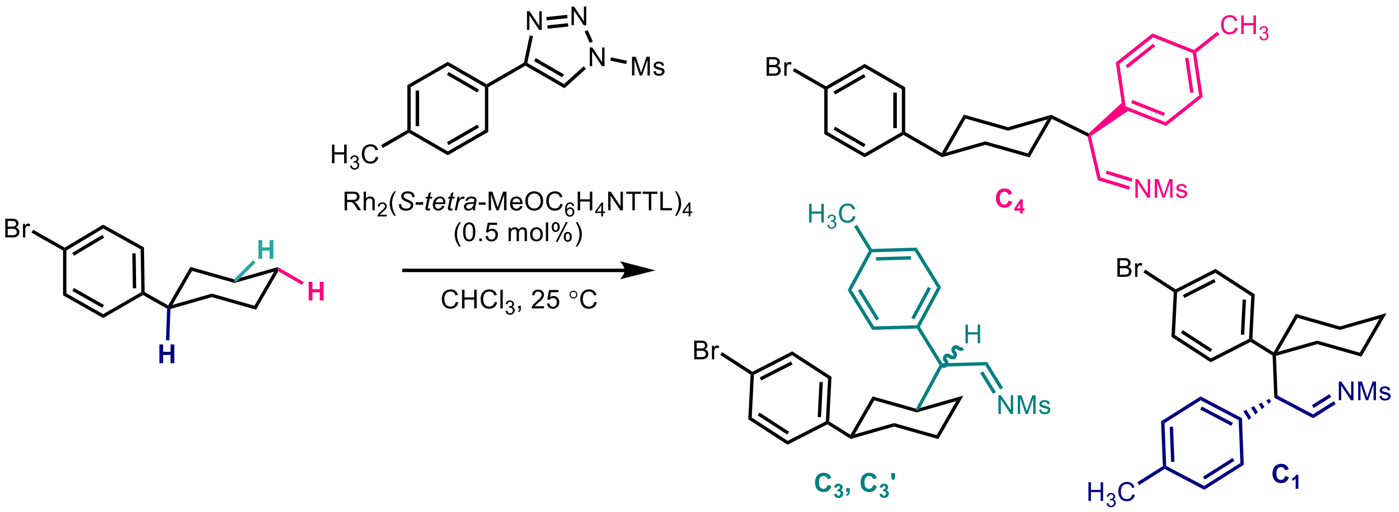

C$_4$:C$_1$ = 16:1
C$_4$:C$_3$ > 20:1

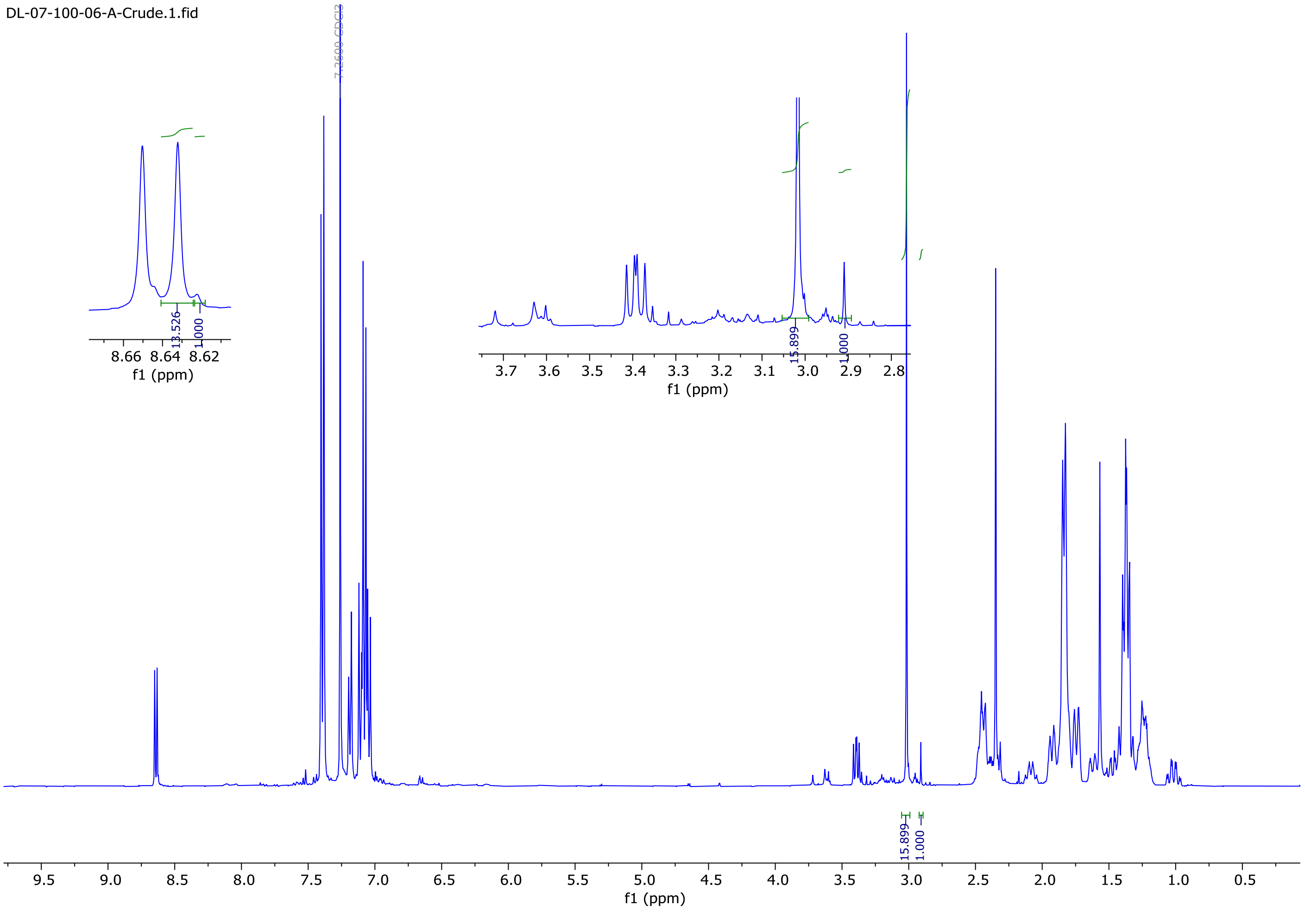

Figure S83. Crude ^{1}H-NMR before reduction of 53

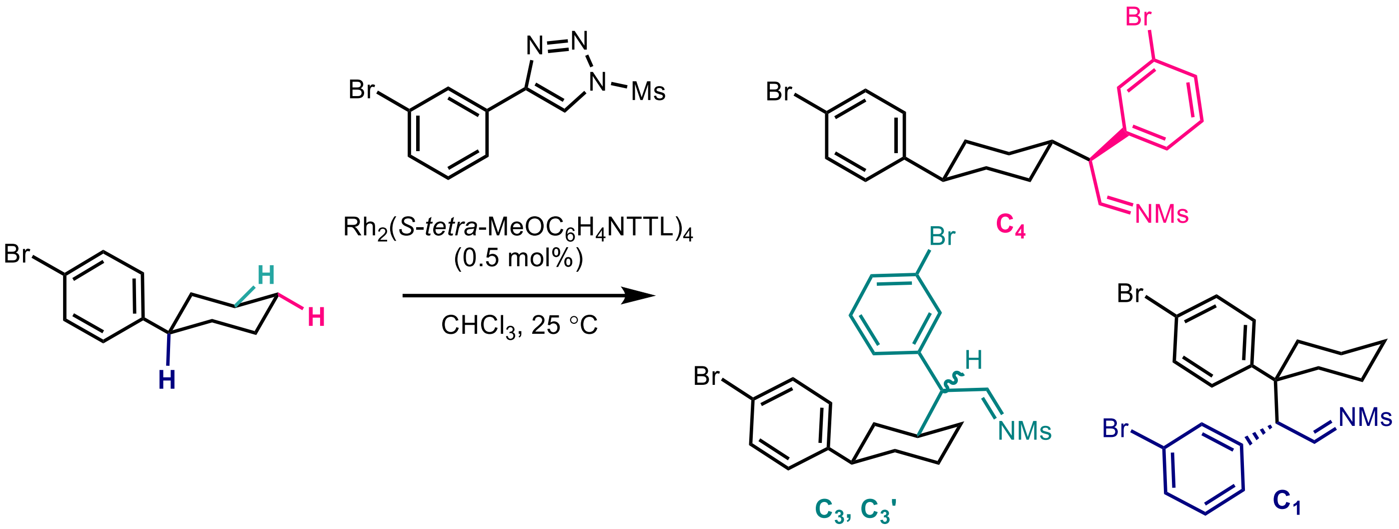

C$_4$:C$_1$ = 12:1
C$_4$:C$_3$ > 20:1

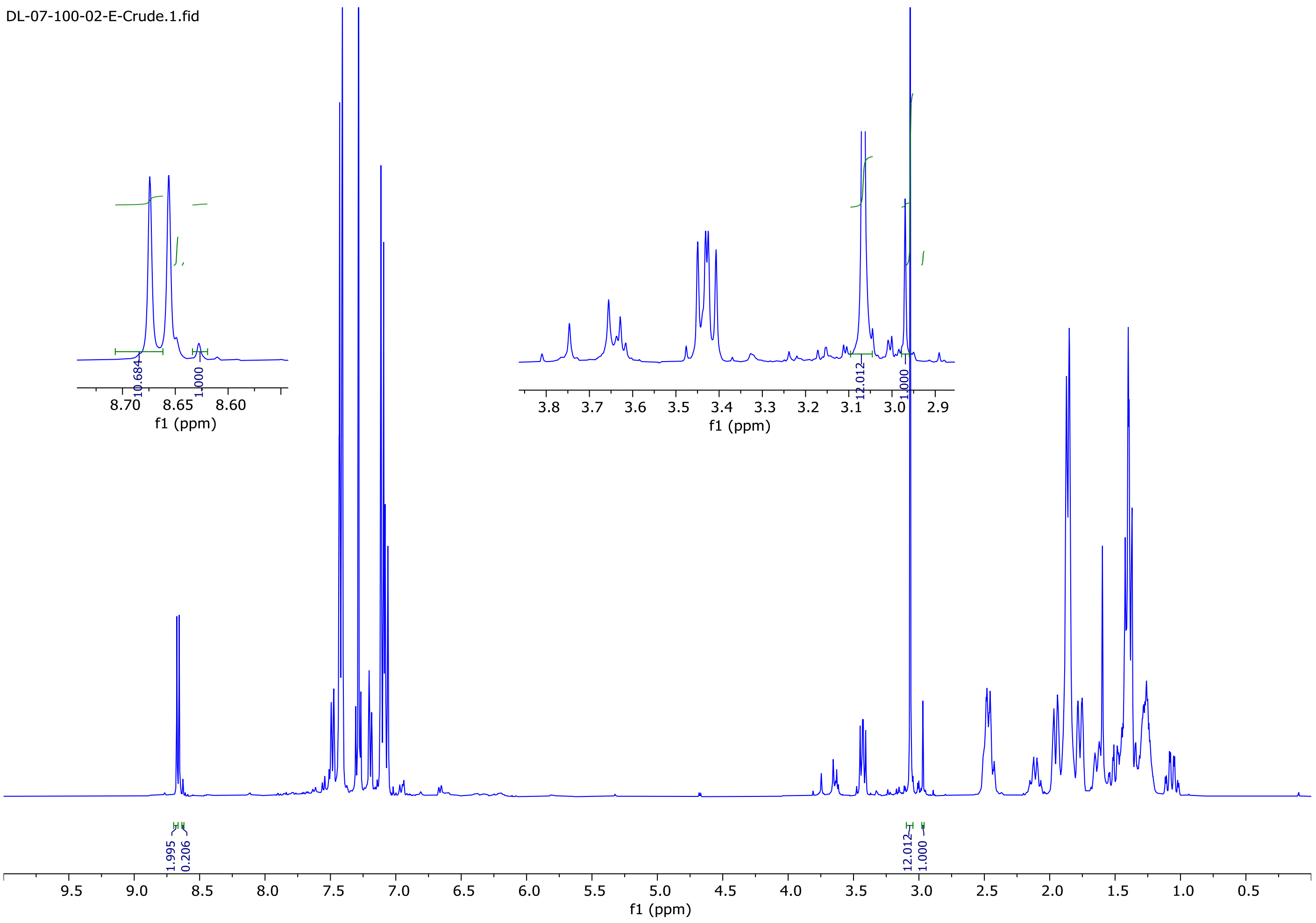

Figure S84. Crude ^{1}H-NMR before reduction of 54

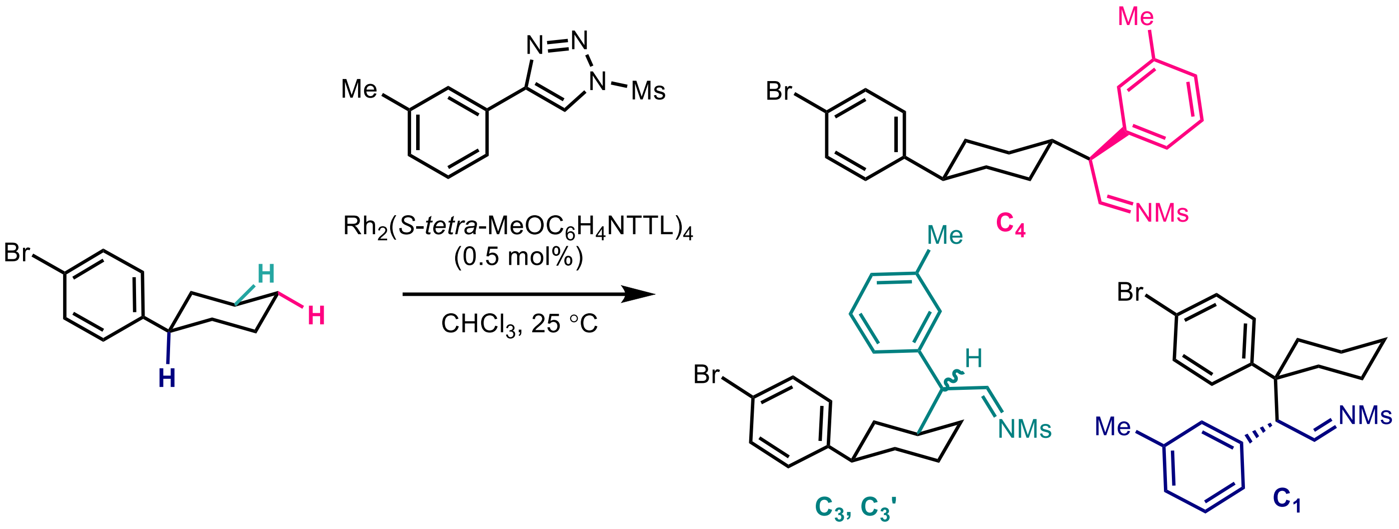

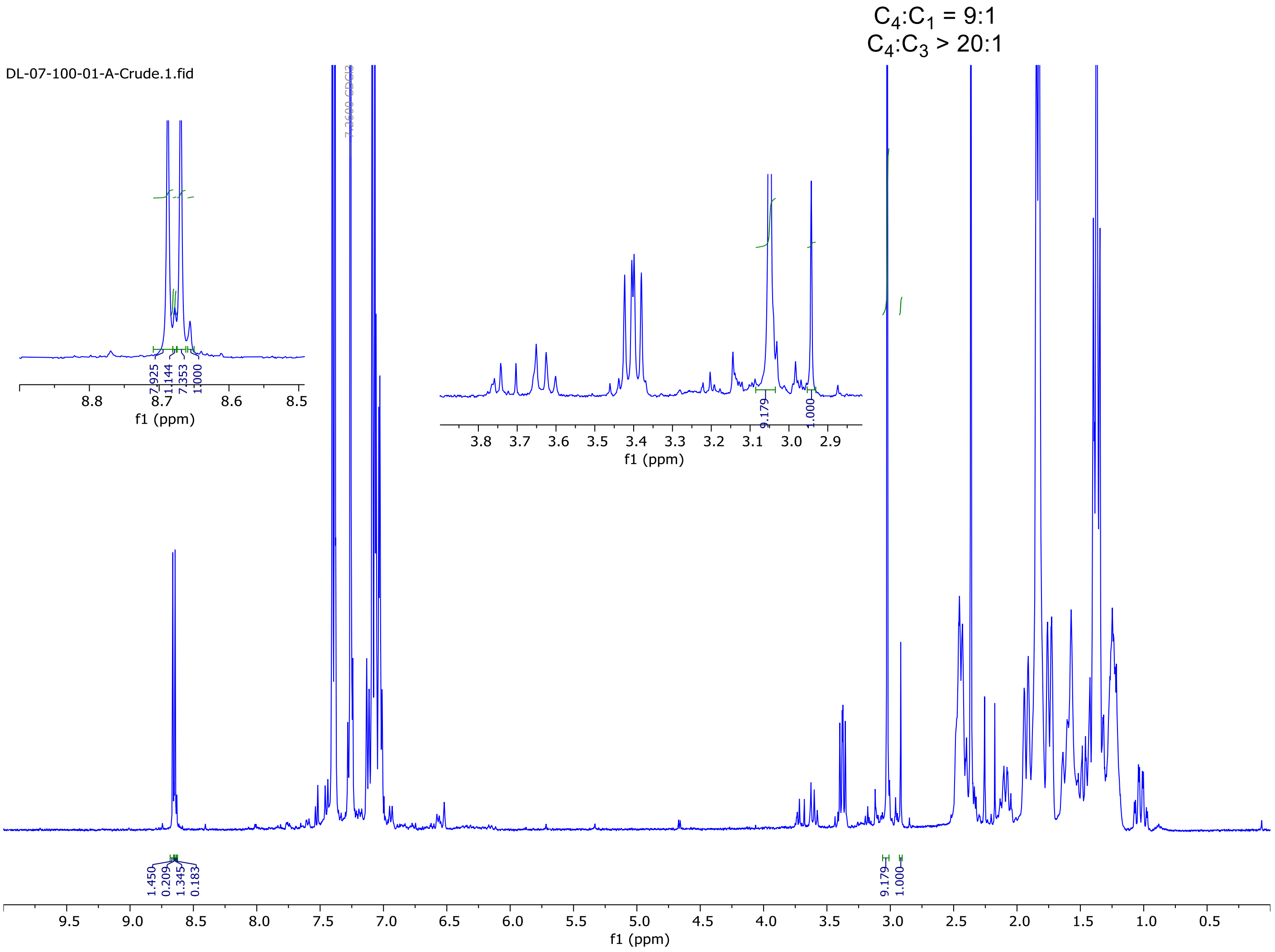

Figure S85. Crude ^{1}H-NMR before reduction of 55

9. Characterization of C-H functionalization products

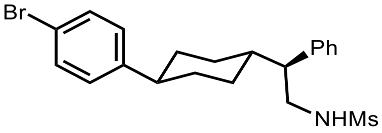

N-((S)-2-((1r,4S)-4-(4-bromophenyl)cyclohexyl)-2-phenylethyl)methanesulfonamide (13)

Prepared according to **general procedure H**, 1-bromo-4-cyclohexylbenzene (120 mg, 0.5 mmol, 2.5 equiv), Rh_2(S-*tetra*-$MeOC_6H_4$NTTL$)_4$ (3.0 mg, 0.0001 mmol, 0.005 equiv), and 1-(methylsulfonyl)-4-phenyl-1H-1,2,3-triazole (44.7 mg, 0.2 mmol, 1.0 equiv) were used. The crude mixture reduced by $NaBH_4$ (18.9 mg, 0.5 mmol, 2.5 equiv) at 0 °C then purified by flash chromatography (gradient 0%-65% Et_2O in hexane) afforded **13** as a white solid (72.7 mg, 83%, 95% ee).

R_f (3H/2EA) = 0.50 (CAM)

$[\alpha]^{20}_D$: -27.0° (c = 0.33 g/100 ml, $CHCl_3$, 95% ee)

^{1}H NMR (400 MHz, $CDCl_3$) δ 7.40 – 7.33 (m, 4H), 7.32 – 7.27 (m, 1H), 7.19 – 7.15 (m, 2H), 7.03 (d, *J* = 8.4 Hz, 2H), 3.88 (dd, *J* = 8.8, 3.6 Hz, 1H), 3.66 (ddd, *J* = 13.0, 8.8, 4.6 Hz, 1H), 3.32 (ddd, *J* = 12.5, 10.6, 3.7 Hz, 1H), 2.80 (s, 3H), 2.61 (ddd, *J* = 10.6, 8.5, 4.5 Hz, 1H), 2.38 (tt, *J* = 12.2, 3.5 Hz, 1H), 2.08 (dt, *J* = 12.8, 3.1 Hz, 1H), 1.99 – 1.89 (m, 1H), 1.78 (dt, *J* = 13.1, 3.1 Hz, 1H), 1.69 – 1.59 (m, 1H), 1.52 (t, *J* = 3.0 Hz, 1H), 1.44 (qd, *J* = 12.8, 3.4 Hz, 1H), 1.31 (qd, *J* = 12.9, 3.5 Hz, 1H), 1.18 (td, *J* = 12.4, 3.5 Hz, 1H), 1.00 (qd, *J* = 12.9, 3.5 Hz, 1H).

^{13}C NMR (101 MHz, $CDCl_3$) δ 146.2, 140.5, 131.5, 129.1, 128.7, 128.6, 128.6, 127.5, 119.7, 52.5, 46.2, 43.7, 40.5, 40.3, 34.0, 33.9, 31.4, 31.1.

HRMS (+p APCI) calcd. for $[C_{21}H_{27}O_2N^{79}Br^{32}S]$ ($[M+H]^+$) 436.0940 found 436.0950.

SFC (OJ3, 10% (50% methanol in isopropanol with 0.2% Formic Acid) in CO_2, 2.5 mL/min, 1.0 mg/ml, UV 230 nm) retention times of 7.39 (major) and 9.40 min (minor) 95% ee.

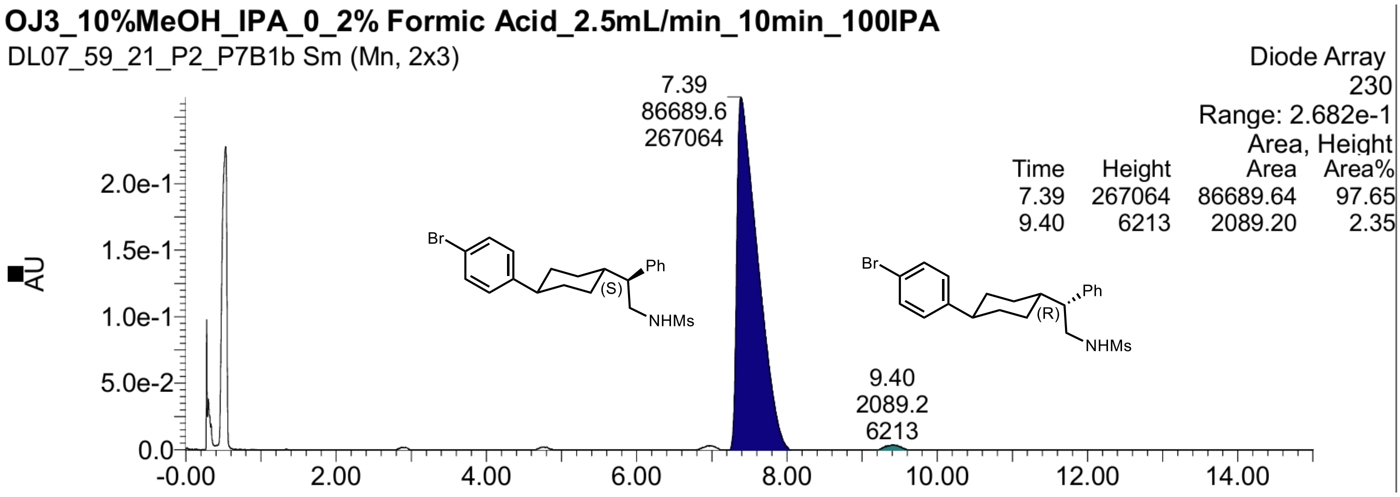

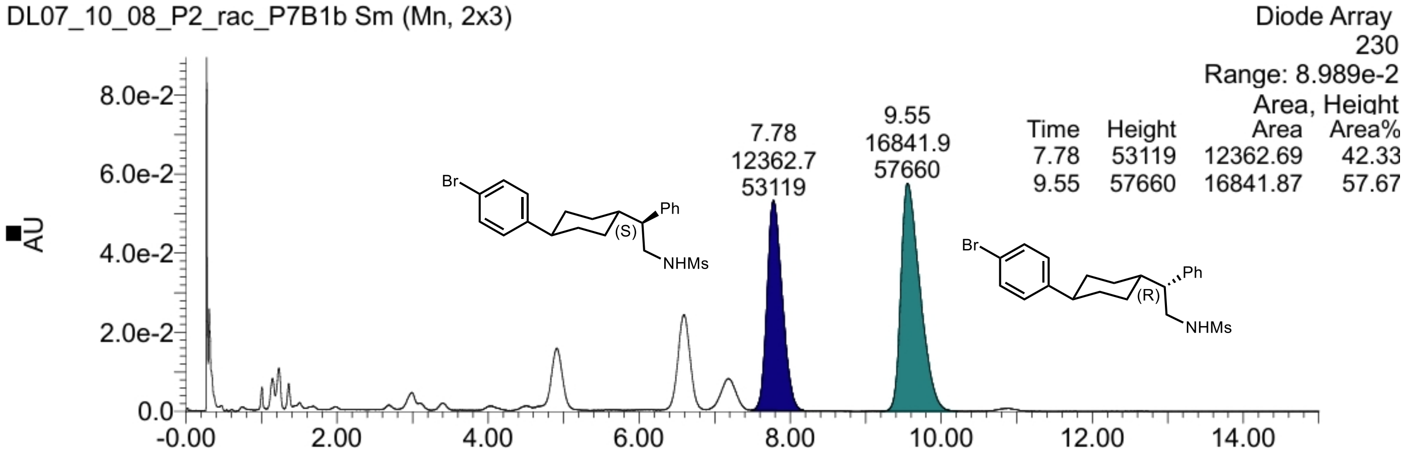

Figure S86. HPLC trace of 13 (chiral top, racemic bottom)

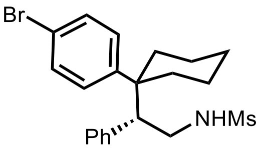

(R)-N-(2-(1-(4-bromophenyl)cyclohexyl)-2-phenylethyl)methanesulfonamide (14)

Prepared according to **general procedure H**, 1-bromo-4-cyclohexylbenzene (120 mg, 0.5 mmol, 2.5 equiv), Rh_2(*S*-*di*-3,5-di-*t*BuC_6H_4NTTL$)_4$ (3.0 mg, 0.0001 mmol, 0.005 equiv), and 1-(methylsulfonyl)-4-phenyl-1H-1,2,3-triazole (44.7 mg, 0.2 mmol, 1.0 equiv) were used. The crude mixture reduced by $NaBH_4$ (18.9 mg, 0.5 mmol, 2.5 equiv) at 0 ˚C then purified by flash chromatography (gradient 0%-65% Et_2O in hexane) afforded **14** as a clear oil (48.1 mg, 55%, 98% ee).

R$_f$ (3H/2EA) = 0.50 (CAM)

$[\alpha]^{20}_D$: -44.1° (c = 1.03 g/100 ml, $CHCl_3$, 98% ee)

^{1}H NMR (800 MHz, $CDCl_3$) δ 7.42 (d, *J* = 8.3 Hz, 2H), 7.23 (d, *J* = 9.7 Hz, 3H), 7.17 – 5.81 (m, 4H), 3.62 (d, *J* = 11.1 Hz, 2H), 3.12 (td, *J* = 11.2, 2.8 Hz, 1H), 2.85 (d, *J* = 11.6 Hz, 1H), 2.71 (s, 3H), 2.48 (d, *J* = 13.5 Hz, 1H), 1.96 (d, *J* = 13.7 Hz, 1H), 1.56 – 1.52 (m, 2H), 1.50 (d, *J* = 12.3 Hz, 3H), 1.18 (dq, *J* = 27.4, 13.3 Hz, 3H).

^{13}C NMR (201 MHz, $CDCl_3$) δ 140.4, 137.4, 131.3, 130.7, 128.3, 127.8, 120.4, 58.5, 44.8, 43.3, 40.4, 35.8, 34.4, 26.4, 22.3, 21.9. (Missing 1 aromatic carbon)

HRMS (-p APCI) calcd. [$C_{21}H_{25}O_2N^{79}Br^{32}S$] ($[M-H]^-$) for 434.0795 found 434.0786.
HPLC (Chiralpak ADH column, 1% i-propanol in hexane, 1.0 mLmin^{-1}, 1.0 mgmL^{-1}, 60 min, UV 230 nm) retention times of 17.8 min (major) and 21.2 min (minor), 98% ee.

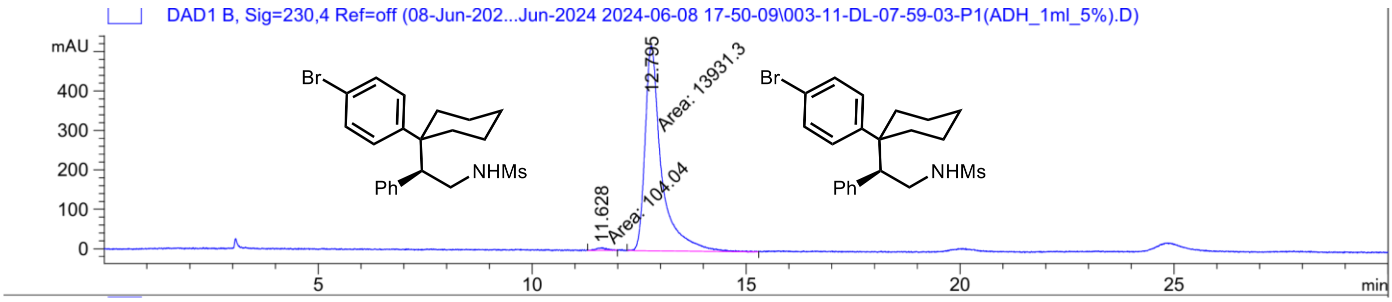

Signal 2: DAD1 B, Sig=230,4 Ref=off

Peak #	RetTime [min]	Type	Width [min]	Area [mAU*s]	Height [mAU]	Area %
1	11.628	MM	0.2527	104.04015	6.86168	0.7413
2	12.795	MM	0.4466	1.39313e4	519.88580	99.2587
Totals :				1.40353e4	526.74749	

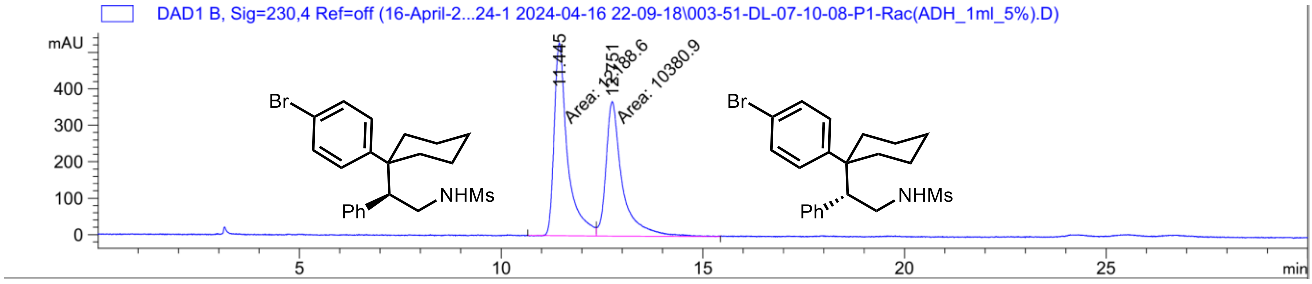

Signal 2: DAD1 B, Sig=230,4 Ref=off

Peak #	RetTime [min]	Type	Width [min]	Area [mAU*s]	Height [mAU]	Area %
1	11.445	MF	0.3840	1.21886e4	529.07813	54.0046
2	12.751	FM	0.4690	1.03809e4	368.90961	45.9954
Totals :				2.25695e4	897.98773	

Figure S87. HPLC trace of 14 (chiral – top, racemic – bottom)

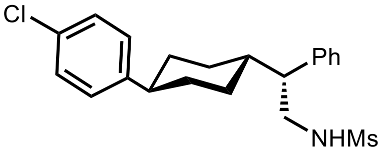

N-((R)-2-((1r,4R)-4-(4-chlorophenyl)cyclohexyl)-2-phenylethyl)methanesulfonamide (15)

Prepared according to **general procedure H**, 1-chloro-4-cyclohexylbenzene (97.4 mg, 0.5 mmol, 2.5 equiv), Rh_2(*S*-*tetra*-$MeOC_6H_4NTTL)_4$ (3.0 mg, 0.0001 mmol, 0.005 equiv), and 1-(methylsulfonyl)-4-phenyl-1H-1,2,3-triazole (44.7 mg, 0.2 mmol, 1.0 equiv) were used. The crude mixture reduced by $NaBH_4$ (18.9 mg, 0.5 mmol, 2.5 equiv) at 0 ˚C then purified by flash chromatography (SiO_2, gradient 0%-65% Et_2O in hexane) afforded **15** as a white solid (64.8 mg, 83% yield, 96% ee).

R_f (3H/2EA) = 0.50 (CAM, UV 210 nm)

$[\alpha]^{20}_D$: -39.0º (c = 0.27 g/100 ml, $CHCl_3$, 96% ee)

^{1}H NMR (800 MHz, $CDCl_3$) δ 7.36 (t, *J* = 7.1 Hz, 2H), 7.28 (t, *J* = 7.7 Hz, 1H), 7.23 (d, *J* = 7.0 Hz, 2H), 7.17 (d, *J* = 6.6 Hz, 2H), 7.09 (d, *J* = 7.1 Hz, 2H), 3.89 (d, *J* = 9.1 Hz, 1H), 3.65 (td, *J* = 9.0, 4.4 Hz, 1H), 3.32 (dd, *J* = 13.1, 10.1

Hz, 1H), 2.80 (d, J = 2.3 Hz, 3H), 2.61 (ddt, J = 13.9, 7.3, 3.3 Hz, 1H), 2.40 (td, J = 12.2, 3.3 Hz, 1H), 2.08 (dd, J = 13.0, 3.1 Hz, 1H), 1.94 (dd, J = 13.3, 3.2 Hz, 1H), 1.79 (dd, J = 13.2, 3.2 Hz, 1H), 1.63 (dh, J = 11.9, 4.0 Hz, 1H), 1.57 – 1.53 (m, 1H), 1.44 (q, J = 12.8 Hz, 1H), 1.31 (q, J = 12.8 Hz, 1H), 1.20 (q, J = 12.7 Hz, 1H), 1.01 (q, J = 13.0 Hz, 1H).

^{13}C NMR (201 MHz, $CDCl_3$) δ 145.69, 140.57, 131.67, 129.13, 128.62, 128.55, 128.22, 127.50, 52.53, 46.26, 43.68, 40.51, 40.37, 34.13, 34.02, 31.44, 31.18.

HRMS (+p APCI) calcd. for [$C_{21}H_{27}O_2N^{35}Cl^{32}S$] ([M+H]$^+$) 392.1446 found 392.1451.
SFC (OJ3, 10% (50% methanol in isopropanol with 0.2% Formic Acid) in CO_2, 2.5 mL/min, 1.0 mg/ml, UV 210 nm) retention times of 5.70 min (major) and 6.96 min (minor) 96% ee.

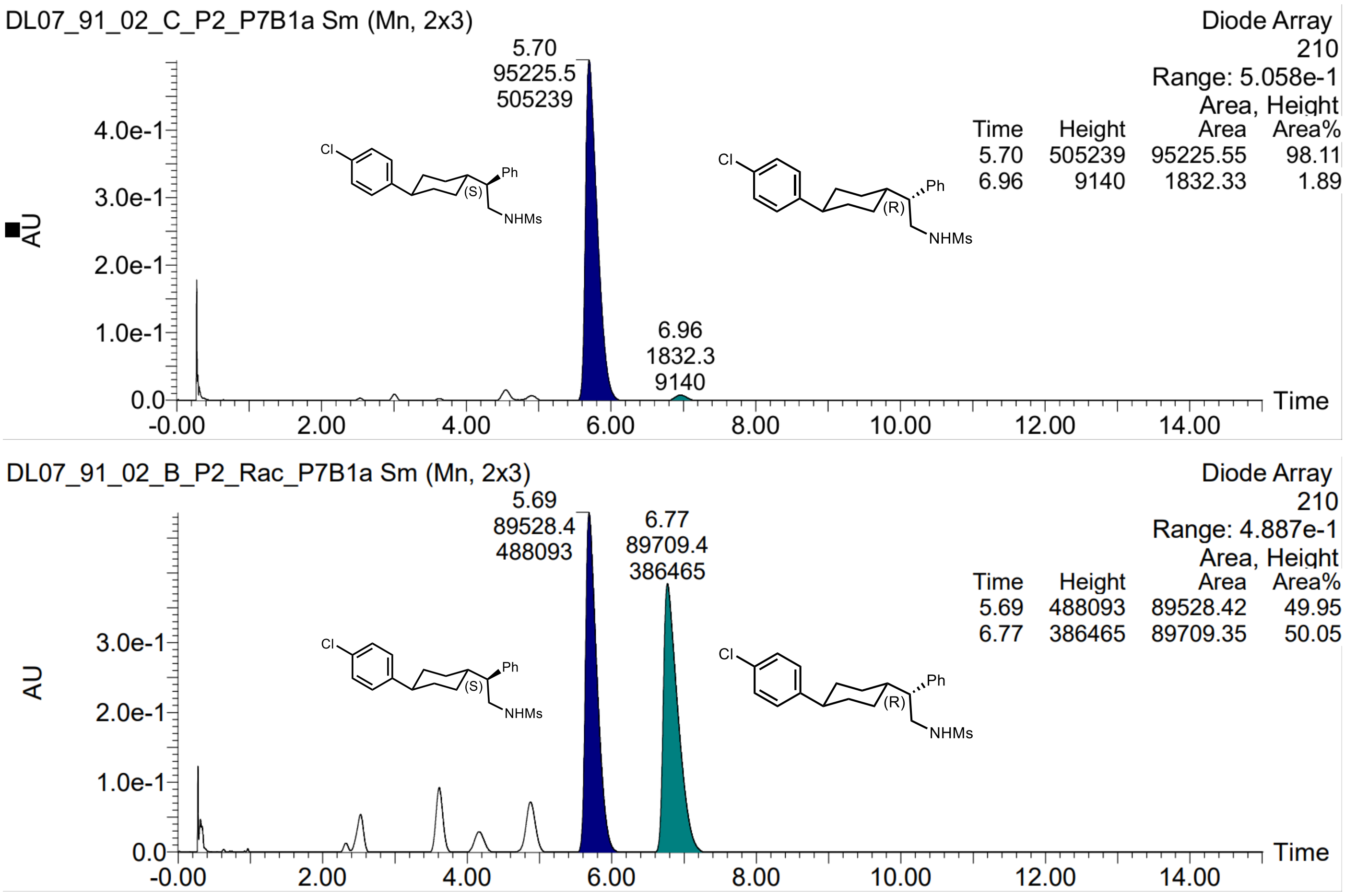

Figure S88. SFC trace of 15 (chiral top, racemic bottom)

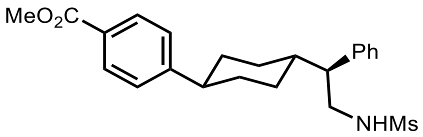

methyl 4-((1S,4r)-4-((S)-2-(methylsulfonamido)-1-phenylethyl)cyclohexyl)benzoate (16)

Prepared according to **general procedure H**, methyl 4-cyclohexylbenzoate (109 mg, 0.5 mmol, 2.5 equiv), Rh_2(*S-tetra*-$MeOC_6H_4NTTL)_4$ (3.0 mg, 0.0001 mmol, 0.005 equiv), and 1-(methylsulfonyl)-4-phenyl-1H-1,2,3-triazole (44.7 mg, 0.2 mmol, 1.0 equiv) were used. The crude mixture reduced by $NaBH_4$ (18.9 mg, 0.5 mmol, 2.5 equiv) at 0 ˚C then purified by flash chromatography (gradient 0%-65% Et_2O in hexane) afforded **16** as a white solid (68.9 mg, 83%, 96% ee).

R$_f$ (3H/2EA) = 0.35 (CAM)

[α]$^{20}_{D}$: -26.0° (c = 0.23 g/100 ml, $CHCl_3$, 96% ee)

^{1}H NMR (600 MHz, $CDCl_3$) δ 7.94 (d, J = 8.3 Hz, 2H), 7.36 (t, J = 7.5 Hz, 2H), 7.28 (t, J = 7.4 Hz, 1H), 7.22 (d, J = 8.3 Hz, 2H), 7.17 (d, J = 7.6 Hz, 2H), 3.89 (m, 4H), 3.66 (ddd, J = 13.0, 8.7, 4.5 Hz, 1H), 3.33 (td, J = 11.4, 3.6 Hz, 1H), 2.80 (s, 3H), 2.62 (td, J = 9.4, 4.5 Hz, 1H), 2.48 (td, J = 10.8, 6.3 Hz, 1H), 2.10 (d, J = 12.9 Hz, 1H), 1.97 (dd, J = 13.0, 3.1 Hz, 1H), 1.82 (dd, J = 13.1, 3.2 Hz, 1H), 1.71 – 1.62 (m, 1H), 1.55 (d, J = 2.9 Hz, 1H), 1.49 (qd, J = 13.0, 3.3 Hz, 1H), 1.37 (qd, J = 12.8, 3.4 Hz, 1H), 1.22 (qd, J = 12.5, 3.1 Hz, 1H), 1.02 (qd, J = 12.5, 3.1 Hz, 1H).

^{13}C NMR (151 MHz, $CDCl_3$) δ 167.2, 152.7, 140.5, 129.9, 129.1, 128.6, 128.1, 127.5, 126.9, 52.5, 52.1, 46.3, 44.4, 40.5, 40.4, 33.9, 33.8, 31.4, 31.1.

HRMS (+p ESI) calcd. for [$C_{23}H_{30}O_4N^{32}S$] ([M+H]$^+$) 416.1890 found 416.1888.
SFC (OJ3, 10% (50% methanol in isopropanol with 0.2% Formic Acid) in CO_2, 2.5 mL/min, 1.0 mg/ml, UV 230 nm) retention times 6.03 (minor) and 7.69 min (major) 96% ee.

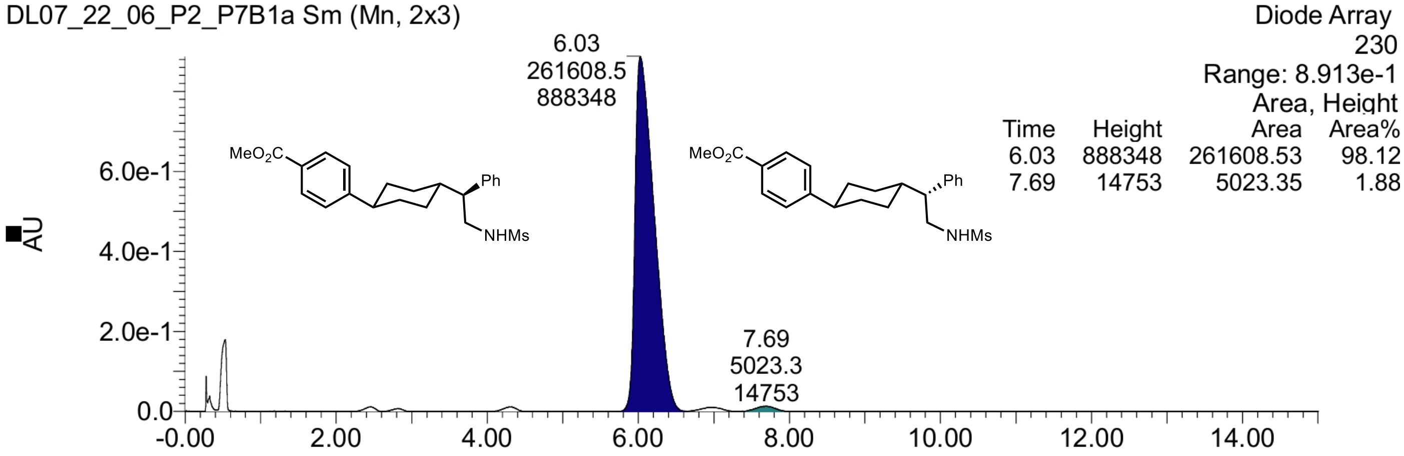

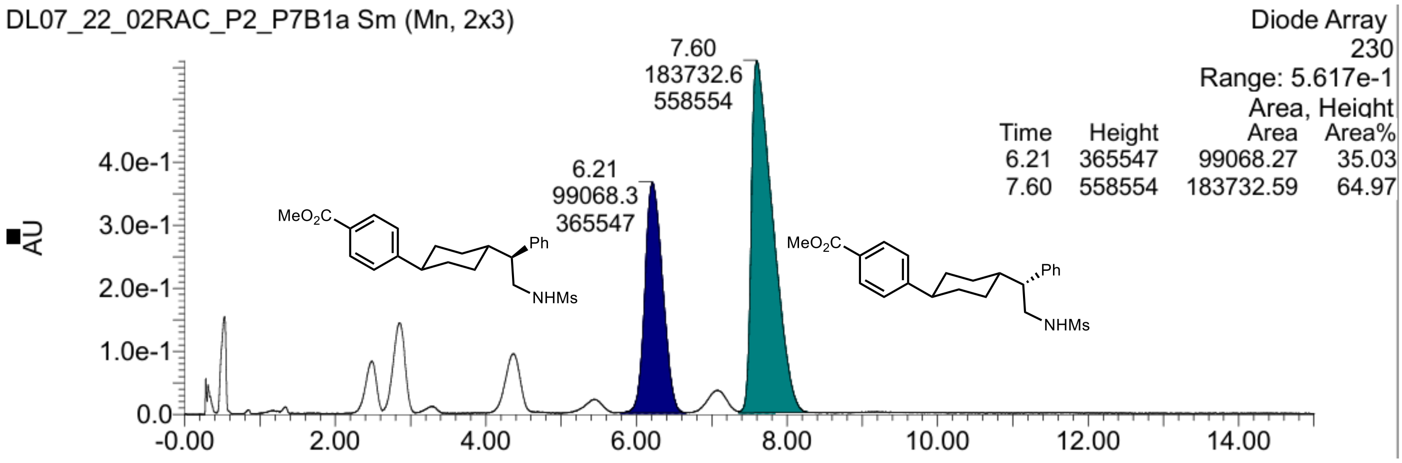

Figure S89. SFC trace of 16 (chiral top, racemic bottom)

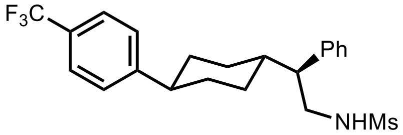

N-((S)-2-phenyl-2-((1r,4S)-4-(4-(trifluoromethyl)phenyl)cyclohexyl)ethyl)methanesulfonamide (17)

Prepared according to **general procedure H**, 1-cyclohexyl-4-(trifluoromethyl)benzene (114 mg, 0.5 mmol, 2.5 equiv), Rh_2(*S-tetra*-$MeOC_6H_4NTTL)_4$ (3.0 mg, 0.0001 mmol, 0.005 equiv), and 1-(methylsulfonyl)-4-phenyl-1H-1,2,3-triazole (44.7 mg, 0.2 mmol, 1.0 equiv) were used. The crude mixture reduced by $NaBH_4$ (18.9 mg, 0.5 mmol, 2.5 equiv) at 0 ˚C then purified by flash chromatography (gradient 0%-65% Et_2O in hexane) afforded **17** as a white solid (65.6 mg, 77% yield, 98% ee).

R_f (3H/2EA) = 0.50 (CAM)

$[\alpha]^{20}_D$: -22.4° (c = 0.33 g/100 ml, $CHCl_3$, 98% ee)

^{1}H NMR (600 MHz, $CDCl_3$) δ 7.52 (d, J = 8.0 Hz, 2H), 7.36 (t, J = 7.5 Hz, 2H), 7.31 – 7.26 (m, 3H), 7.18 (d, J = 7.5 Hz, 2H), 3.90 (dd, J = 8.9, 3.6 Hz, 1H), 3.66 (ddd, J = 13.0, 8.7, 4.6 Hz, 1H), 3.33 (ddd, J = 12.8, 10.6, 3.7 Hz, 1H), 2.80 (s, 3H), 2.62 (dq, J = 13.4, 4.6 Hz, 1H), 2.49 (tt, J = 12.2, 3.5 Hz, 1H), 2.10 (dq, J = 12.9, 3.2 Hz, 1H), 1.97 (dq, J = 12.9, 3.2 Hz, 1H), 1.82 (dq, J = 13.1, 3.2 Hz, 1H), 1.66 (dtt, J = 11.7, 7.4, 3.6 Hz, 1H), 1.57 (dq, J = 13.3, 3.2 Hz, 1H), 1.49 (qd, J = 12.8, 3.5 Hz, 1H), 1.37 (qd, J = 12.8, 3.4 Hz, 1H), 1.23 (qd, J = 12.7, 3.4 Hz, 1H), 1.03 (qd, J = 12.9, 3.6 Hz, 1H).

^{13}C NMR (151 MHz, $CDCl_3$) δ 151.2, 140.5, 129.1, 128.5, 128.4 (q, J = 32.2 Hz), 127.5, 127.2, 125.4 (q, J = 3.7 Hz), 124.5 (q, J = 217.6 Hz), 52.5, 46.2, 44.2, 40.5, 40.4, 33.9, 33.8, 31.4, 31.1.

^{19}F NMR (565 MHz, $CDCl_3$) δ -62.3.

HRMS (+p APCI) calcd. for $[C_{22}H_{27}O_2NF_3{}^{32}S]$ ($[M+H]^+$) 426.1709 found 426.1716.

HPLC (Chiralpak ADH column, 5% i-propanol in hexane, 0.5 $mLmin^{-1}$, 1.0 $mgmL^{-1}$, 60 min, UV 210 nm) retention times of 38.9 min (minor) and 41.1 min (major), 98% ee.

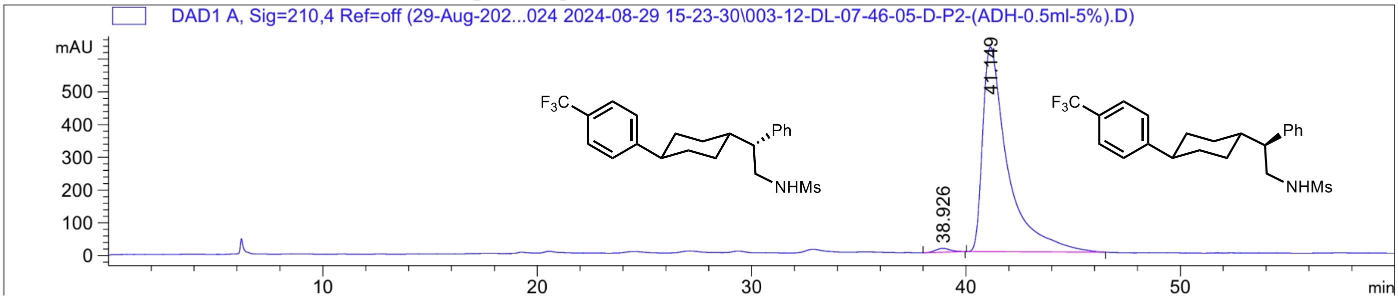

Signal 1: DAD1 A, Sig=210,4 Ref=off

Peak #	RetTime [min]	Type	Width [min]	Area [mAU*s]	Height [mAU]	Area %
1	38.926	BB	0.5783	580.44305	11.74034	1.1209
2	41.149	VV R	0.9591	5.12029e4	626.40698	98.8791
Totals :				5.17834e4	638.14733	

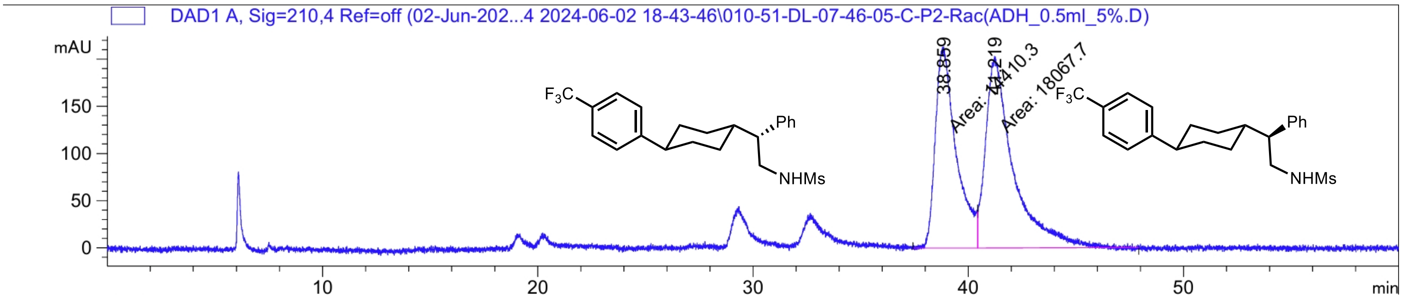

Signal 1: DAD1 A, Sig=210,4 Ref=off

Peak #	RetTime [min]	Type	Width [min]	Area [mAU*s]	Height [mAU]	Area %
1	38.859	MF	1.1266	1.44103e4	213.18413	44.3694
2	41.219	FM	1.4827	1.80677e4	203.09459	55.6306
Totals :				3.24779e4	416.27872	

Figure S90. HPLC trace of 17 (chiral top, racemic bottom)

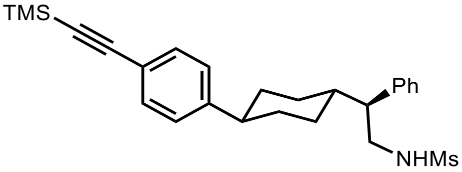

N-((S)-2-phenyl-2-((1r,4S)-4-(4-((trimethylsilyl)ethynyl)phenyl)cyclohexyl)ethyl)methanesulfonamide (18)

Prepared according to **general procedure H**, ((4-cyclohexylphenyl)ethynyl)trimethylsilane (128 mg, 0.5 mmol, 2.5 equiv), Rh_2(*S*-*tetra*-$MeOC_6H_4$NTTL)$_4$ (3.0 mg, 0.0001 mmol, 0.005 equiv), and 1-(methylsulfonyl)-4-phenyl-1H-1,2,3-triazole (44.7 mg, 0.2 mmol, 1.0 equiv) were used. The crude mixture reduced by $NaBH_4$ (18.9 mg, 0.5 mmol, 2.5 equiv) at 0 ˚C then purified by flash chromatography (gradient 0%-65% Et_2O in hexane) afforded **18** as a white solid (66.3 mg, 73%, 96% ee).

R$_f$ (3H/2EA) = 0.50 (CAM)

$[\alpha]^{20}_D$: -42.7° (c = 0.28 g/100 ml, $CHCl_3$, 96% ee)

^{1}H NMR (600 MHz, $CDCl_3$) δ 7.43 – 7.32 (m, 4H), 7.28 (d, *J* = 7.6 Hz, 1H), 7.17 (d, *J* = 7.0 Hz, 2H), 7.08 (d, *J* = 7.9 Hz, 2H), 3.89 (dd, *J* = 8.9, 3.6 Hz, 1H), 3.66 (ddd, *J* = 13.0, 8.7, 4.6 Hz, 1H), 3.32 (ddd, *J* = 13.3, 11.1, 3.6 Hz, 1H), 2.80 (s, 3H), 2.60 (td, *J* = 9.7, 4.4 Hz, 1H), 2.44 – 2.37 (m, 1H), 2.08 (d, *J* = 12.9 Hz, 1H), 1.94 (d, *J* = 13.3 Hz, 1H), 1.78 (d, *J* = 12.9 Hz, 1H), 1.63 (dtd, *J* = 11.8, 8.5, 4.0 Hz, 1H), 1.54 (d, *J* = 14.4 Hz, 1H), 1.49 – 1.41 (m, 1H), 1.32 (qd, *J* = 12.9, 3.5 Hz, 1H), 1.20 (qd, *J* = 12.6, 3.5 Hz, 1H), 1.08 – 0.94 (m, 1H), 0.23 (s, 9H).

^{13}C NMR (151 MHz, $CDCl_3$) δ 147.9, 140.6, 132.1, 129.1, 128.6, 127.5, 126.8, 120.8, 105.4, 93.5, 52.5, 46.3, 44.2, 40.5, 40.3, 33.9, 33.8, 31.4, 31.2, 0.2.

HRMS (-p APCI) calcd. for $[C_{26}H_{34}O_2N^{32}S^{28}Si]$ ($[M-H]^-$) 452.2085 found 452.2084.

SFC (OJ3, 5% (50% methanol in isopropanol with 0.2% Formic Acid) in CO_2, 2.5 mL/min, 1.0 mg/ml, UV 230 nm) retention times of 9.68 (major) and 11.55 min (minor) 96% ee.

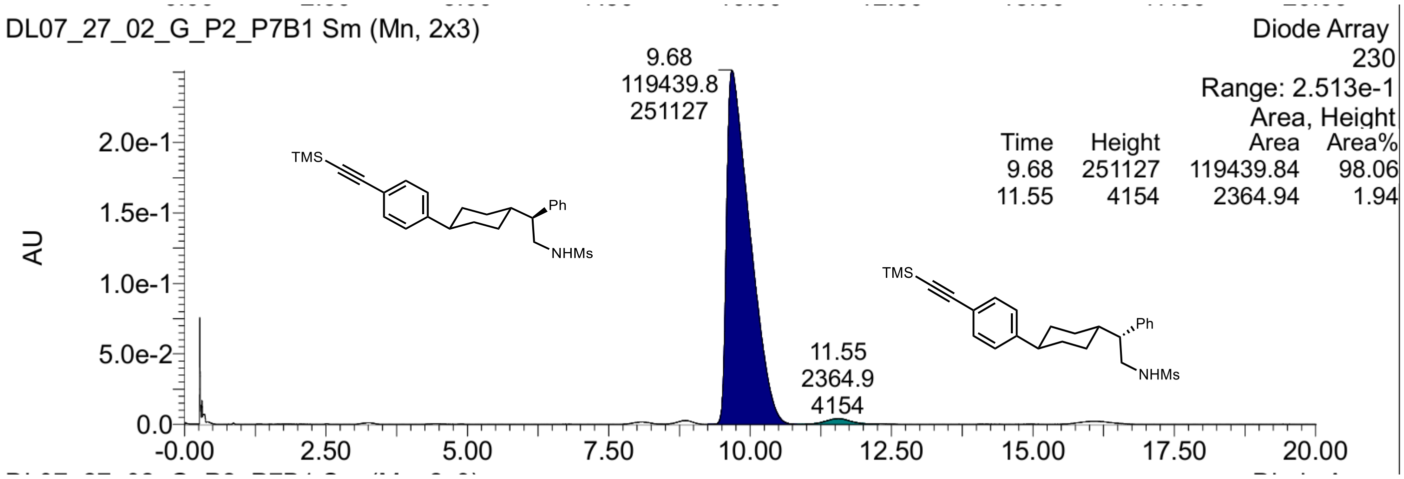

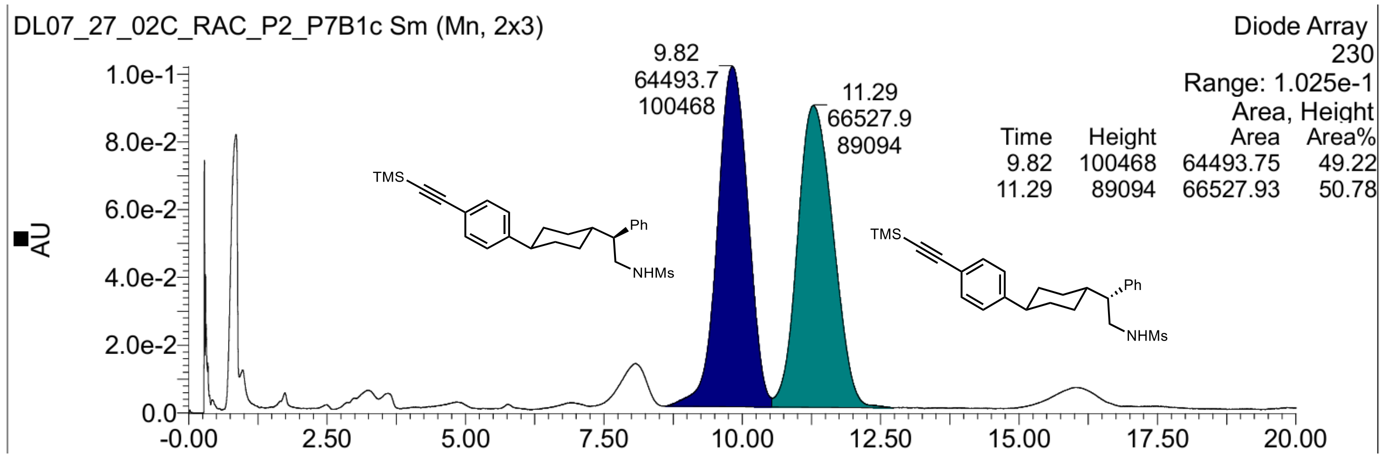

Figure S91. SFC trace of 18 (chiral top, racemic bottom)

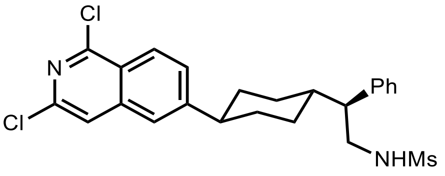

N-((S)-2-((1r,4S)-4-(1,3-dichloroisoquinolin-6-yl)cyclohexyl)-2-phenylethyl)methanesulfonamide (19)

Prepared according to **general procedure H**, 1,3-dichloro-6-cyclohexylisoquinoline (140 mg, 0.5 mmol, 2.5 equiv), Rh_2(*S*-*tetra*-$MeOC_6H_4NTTL)_4$ (3.0 mg, 0.0001 mmol, 0.005 equiv), and 1-(methylsulfonyl)-4-phenyl-1H-1,2,3-triazole (44.7 mg, 0.2 mmol, 1.0 equiv) were used. The crude mixture reduced by $NaBH_4$ (18.9 mg, 0.5 mmol, 2.5 equiv) at 0 ˚C then purified by flash chromatography (gradient 0%-65% Et_2O in hexane) afforded **19** as a white solid (78.9 mg, 83% yield, 95% ee).

R_f (3H/2EA) = 0.60 (CAM)

$[\alpha]^{20}_D$: -30.7° (c = 0.38 g/100 ml, $CHCl_3$, 95% ee)

^{1}H NMR (600 MHz, $CDCl_3$) δ 8.18 (d, *J* = 9.0 Hz, 1H), 7.57 (s, 1H), 7.53 – 7.47 (m, 2H), 7.37 (t, *J* = 7.5 Hz, 2H), 7.29 (t, *J* = 7.4 Hz, 1H), 7.19 (d, *J* = 7.4 Hz, 2H), 3.97 (dd, *J* = 9.0, 4.0 Hz, 1H), 3.67 (ddd, *J* = 13.2, 8.7, 4.7 Hz, 1H), 3.39 – 3.31 (m, 1H), 2.81 (s, 3H), 2.68 – 2.58 (m, 2H), 2.14 (d, *J* = 11.3 Hz, 1H), 2.04 (d, *J* = 12.0 Hz, 1H), 1.89 (d, *J* = 12.7 Hz, 1H), 1.76 – 1.65 (m, 1H), 1.61 (d, *J* = 13.7 Hz, 1H), 1.55 (td, *J* = 12.8, 3.4 Hz, 1H), 1.44 (qd, *J* = 12.4, 3.3 Hz, 1H), 1.27 (qd, *J* = 12.5, 3.4 Hz, 1H), 1.07 (qd, *J* = 12.9, 3.4 Hz, 1H).

^{13}C NMR (151 MHz, $CDCl_3$) δ 151.9, 150.7, 143.2, 140.4, 139.8, 129.1, 129.1, 128.6, 127.5, 126.7, 124.6, 123.1, 119.7, 52.4, 46.2, 44.4, 40.4, 33.7, 33.6, 31.3, 31.0.

HRMS (-p APCI) calcd. for $[C_{24}H_{25}O_2N_2{}^{35}Cl_2{}^{32}S]$ ($[M-H]^-$) 475.1019 found 475.1020.
SFC (CEL2, 30% (50% methanol in isopropanol with 0.2% Formic Acid) in CO_2, 2.5 mL/min, 1.0 mg/ml, UV 210 nm) retention times of 4.76 (major) and 6.19 min (minor) 95% ee.

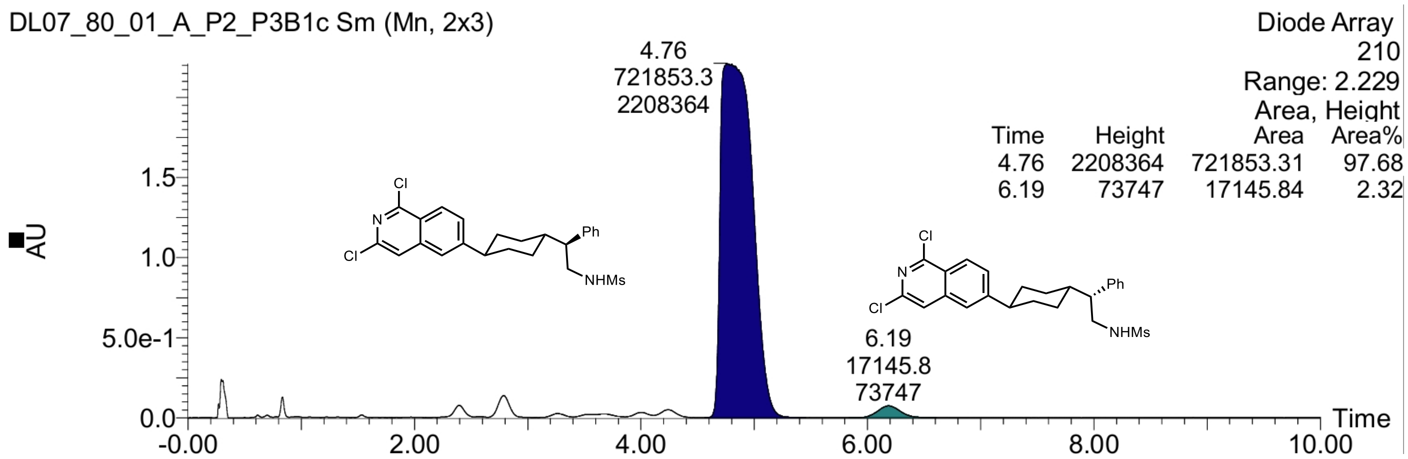

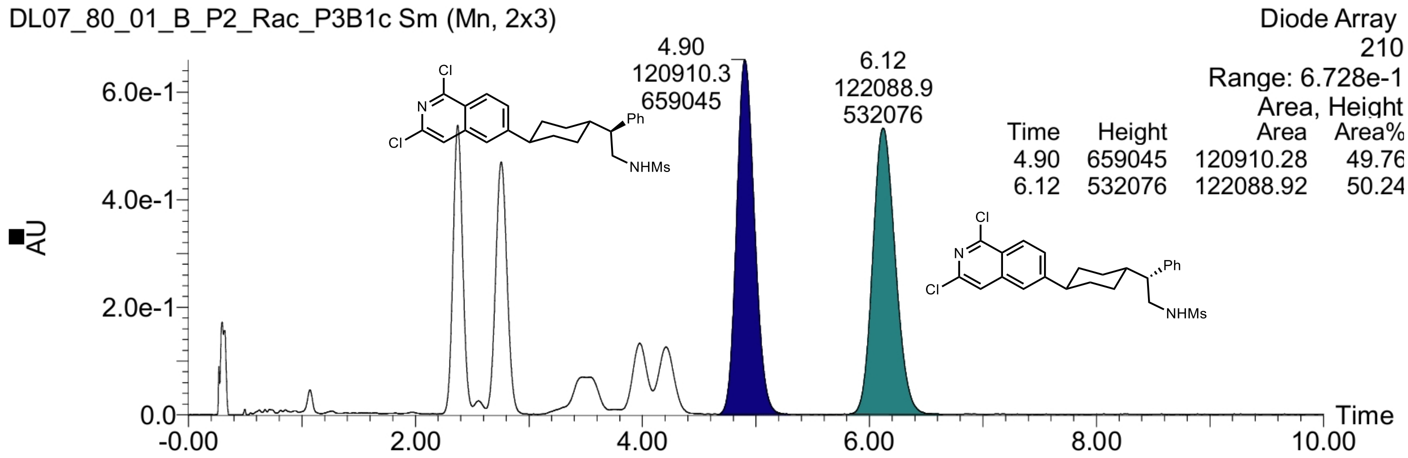

Figure S92. SFC trace of 19 (chiral top, racemic bottom)

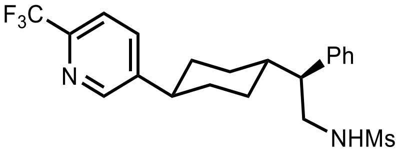

N-((S)-2-phenyl-2-((1r,4S)-4-(6-(trifluoromethyl)pyridin-3-yl)cyclohexyl)ethyl)methanesulfonamide (20)

Prepared according to **general procedure H**, 5-cyclohexyl-2-(trifluoromethyl)pyridine (115 mg, 0.5 mmol, 2.5 equiv), Rh_2(*S-tetra*-$MeOC_6H_4NTTL)_4$ (3.0 mg, 0.0001 mmol, 0.005 equiv), and 1-(methylsulfonyl)-4-phenyl-1H-1,2,3-triazole (44.7 mg, 0.2 mmol, 1.0 equiv) were used. The crude mixture reduced by $NaBH_4$ (18.9 mg, 0.5 mmol, 2.5 equiv) at 0 ˚C then purified by flash chromatography (SiO_2, gradient 0%-65% Et_2O in hexane) afforded **20** as a white solid (61.5 mg, 72% yield, 95% ee).

R_f (3H/1EA) = 0.50 (UV 254 nm)

$[\alpha]^{20}_D$: -20.8° (c = 0.39 g/100 ml, $CHCl_3$, 95% ee)

^{1}H NMR (800 MHz, CDCl$_3$) δ 8.54 (s, 1H), 7.64 (d, *J* = 8.2 Hz, 1H), 7.59 (d, *J* = 7.9 Hz, 1H), 7.38 – 7.34 (m, 2H), 7.30 – 7.27 (m, 1H), 7.17 (d, *J* = 8.5 Hz, 2H), 3.94 (dd, *J* = 8.9, 4.1 Hz, 1H), 3.65 (ddt, *J* = 12.7, 8.0, 3.5 Hz, 1H), 3.34 (dd, *J* = 13.2, 10.0 Hz, 1H), 2.80 (s, 3H), 2.67 – 2.60 (m, 1H), 2.55 (td, *J* = 12.4, 3.1 Hz, 1H), 2.13 (d, *J* = 12.9 Hz, 1H), 1.99 (d, *J* = 13.5 Hz, 1H), 1.84 (dd, *J* = 12.8, 3.2 Hz, 1H), 1.68 (dtd, *J* = 12.1, 8.4, 4.2 Hz, 1H), 1.62 – 1.56 (m, 1H), 1.51 (q, *J* = 13.0 Hz, 1H), 1.39 (q, *J* = 12.3 Hz, 1H), 1.25 (q, *J* = 12.7 Hz, 1H), 1.04 (q, *J* = 12.8 Hz, 1H).

^{13}C NMR (201 MHz, CDCl$_3$) δ 149.3, 146.2 (q, *J* = 34.7 Hz), 145.5, 140.3, 135.3, 129.2, 128.6, 127.6, 121.8 (q, *J* = 274 Hz), 120.3 (q, *J* = 3.0 Hz), 52.4, 46.2, 41.7, 40.4, 40.2, 33.7, 33.6, 31.2, 30.9.

^{19}F NMR (753 MHz, CDCl$_3$) δ -67.70.

HRMS (+p APCI) calcd. for [$C_{21}H_{26}O_2N_2F_3{}^{32}S$] ([M+H]$^+$) 427.1662 found 427.1666.
SFC (CEL2, 15% (50% methanol in isopropanol with 0.2% Formic Acid) in CO_2, 2.5 mL/min, 1.0 mg/ml, UV 210 nm) retention times of 3.09 min (major) and 3.93 min (minor) 95% ee.

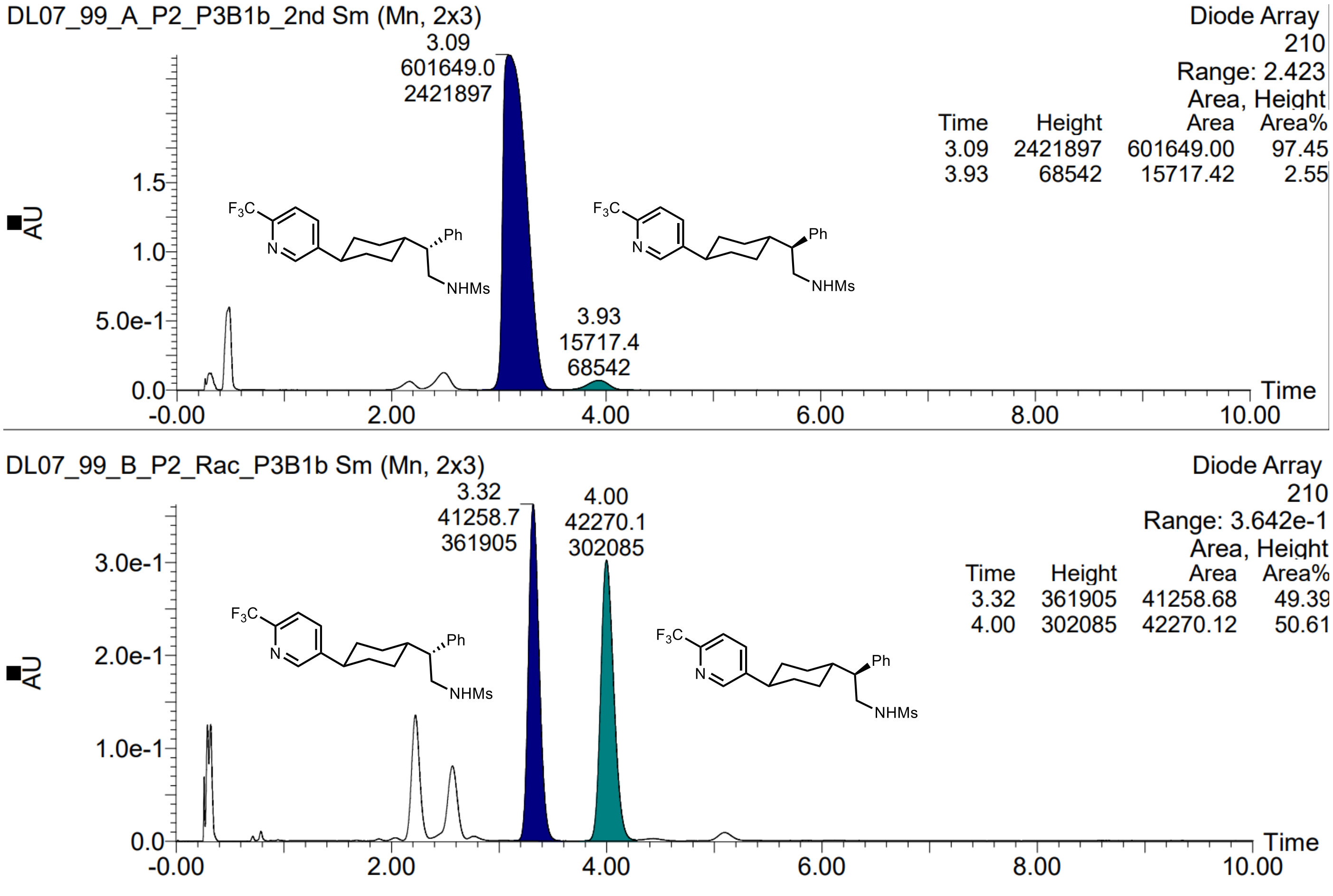

Figure S93. SFC trace of 20 (chiral top, racemic bottom)

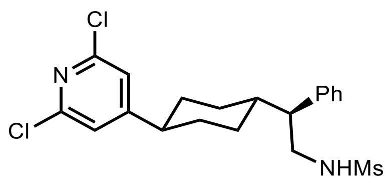

N-((S)-2-((1r,4S)-4-(2,6-dichloropyridin-4-yl)cyclohexyl)-2-phenylethyl)methanesulfonamide (21)

Prepared according to **general procedure H**, 2,6-dichloro-4-cyclohexylpyridine (115 mg, 0.5 mmol, 2.5 equiv), Rh$_2$(*S*-*tetra*-MeOC$_6$H$_4$NTTL)$_4$ (3.0 mg, 0.0001 mmol, 0.005 equiv), and 1-(methylsulfonyl)-4-phenyl-1H-1,2,3-triazole (44.7

mg, 0.2 mmol, 1.0 equiv) were used. The crude mixture reduced by $NaBH_4$ (18.9 mg, 0.5 mmol, 2.5 equiv) at 0 ˚C then purified by flash chromatography (gradient 0%-65% Et_2O in hexane) afforded **21** as a white solid (49.4 mg, 58%, 96% ee).

R$_f$ (3H/2EA) = 0.50 (CAM)

$[\alpha]^{20}_D$: -25.3° (c = 0.60 g/100 ml, $CHCl_3$, 96% ee)

^{1}H NMR (600 MHz, $CDCl_3$) δ 7.35 (t, *J* = 7.6 Hz, 2H), 7.31 – 7.26 (m, 1H), 7.15 (d, *J* = 8.2 Hz, 2H), 7.05 (s, 2H), 3.97 (dd, *J* = 9.0, 4.0 Hz, 1H), 3.63 (ddd, *J* = 13.2, 8.8, 4.7 Hz, 1H), 3.35 – 3.25 (m, 1H), 2.79 (d, *J* = 2.9 Hz, 3H), 2.61 (td, *J* = 9.6, 4.5 Hz, 1H), 2.42 (ddd, *J* = 15.6, 10.5, 3.4 Hz, 1H), 2.14 – 2.07 (m, 1H), 1.96 (dt, *J* = 13.4, 3.1 Hz, 1H), 1.80 (dt, *J* = 13.4, 3.0 Hz, 1H), 1.65 (ddt, *J* = 16.8, 11.6, 5.6 Hz, 1H), 1.59 – 1.53 (m, 1H), 1.48 – 1.39 (m, 1H), 1.31 (qd, *J* = 12.8, 3.3 Hz, 1H), 1.19 (qd, *J* = 12.7, 3.4 Hz, 1H), 0.99 (qd, *J* = 12.9, 3.4 Hz, 1H).

^{13}C NMR (151 MHz, $CDCl_3$) δ 161.9, 150.6, 140.2, 129.2, 128.6, 127.6, 121.6, 52.3, 46.1, 43.3, 40.4, 40.1, 33.0, 32.9, 30.9, 30.6.

HRMS (-p APCI) calcd. for [$C_{20}H_{23}O_2N_2{}^{35}Cl_2{}^{32}S$] ([M-H]$^-$) 425.0863 found 425.0860.

SFC (OJ3, 10% (50% methanol in isopropanol with 0.2% Formic Acid) in CO_2, 2.5 mL/min, 1.0 mg/ml, UV 210 nm) retention times of 5.54 (minor) and 5.81 min (major) 96% ee.

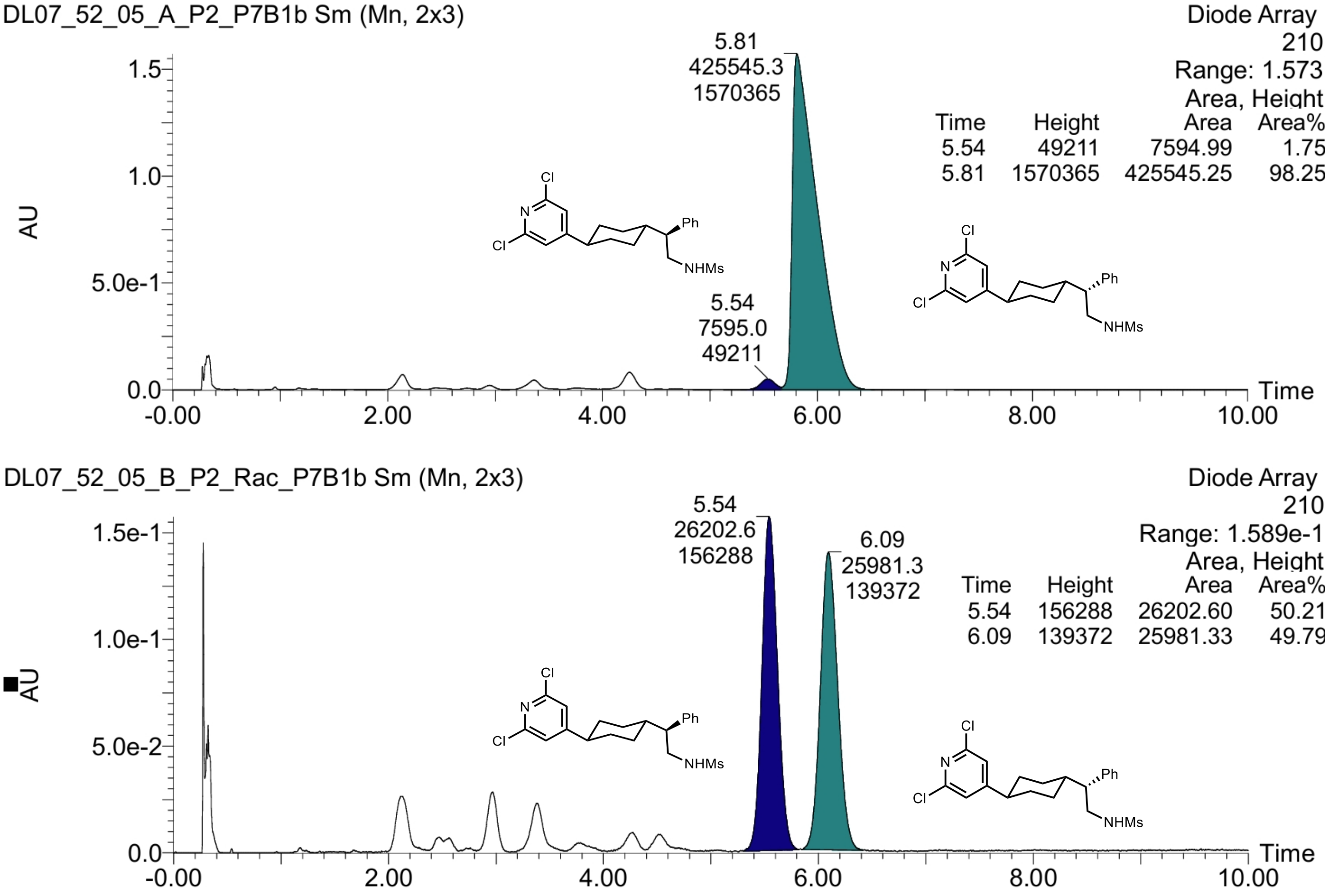

Figure S94. SFC trace of 21 (chiral top, racemic bottom)

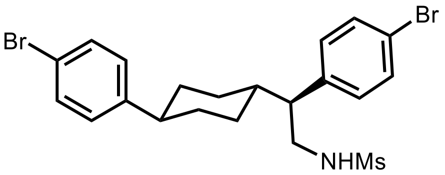

N-((S)-2-(4-bromophenyl)-2-((1r,4S)-4-(4-bromophenyl)cyclohexyl)ethyl)methanesulfonamide (22)

Prepared according to **general procedure H**, 1-bromo-4-cyclohexylbenzene (120 mg, 0.5 mmol, 2.5 equiv), Rh_2(*S*-*tetra*-$MeOC_6H_4$NTTL)$_4$ (3.0 mg, 0.0001 mmol, 0.005 equiv), and 4-(4-bromophenyl)-1-(methylsulfonyl)-1H-1,2,3-triazole (60.4 mg, 0.2 mmol, 1.0 equiv) were used. The crude mixture reduced by $NaBH_4$ (18.9 mg, 0.5 mmol, 2.5 equiv) at 0 ˚C then purified by flash chromatography (SiO_2, gradient 0%-65% Et_2O in hexane) afforded **22** as a white solid (54.3 mg, 53% yield, 98% ee).

R_f (3H/2EA) = 0.50 (CAM, UV 210 nm)

$[\alpha]^{20}_D$: -31.8° (c = 0.38 g/100 ml, $CHCl_3$, 98% ee)

^{1}H NMR (800 MHz, $CDCl_3$) δ 7.49 (d, *J* = 6.6 Hz, 2H), 7.38 (d, *J* = 6.6 Hz, 2H), 7.06 (d, *J* = 6.6 Hz, 2H), 7.03 (d, *J* = 7.1 Hz, 2H), 3.91 – 3.85 (m, 1H), 3.66 – 3.60 (m, 1H), 3.29 (td, *J* = 11.8, 3.6 Hz, 1H), 2.83 (s, 3H), 2.65 – 2.56 (m, 1H), 2.38 (td, *J* = 12.1, 3.1 Hz, 1H), 2.05 (d, *J* = 11.4 Hz, 1H), 1.94 (d, *J* = 13.2 Hz, 1H), 1.80 (d, *J* = 13.2 Hz, 1H), 1.61 (tdd, *J* = 11.8, 8.3, 3.3 Hz, 1H), 1.53 (d, *J* = 11.7 Hz, 1H), 1.43 (q, *J* = 12.7 Hz, 1H), 1.31 (q, *J* = 12.9 Hz, 1H), 1.19 (q, *J* = 12.8 Hz, 1H), 0.98 (q, *J* = 12.9 Hz, 1H).

^{13}C NMR (201 MHz, $CDCl_3$) δ 146.1, 139.6, 132.2, 131.5, 130.3, 128.6, 121.3, 119.8, 52.1, 46.0, 43.7, 40.6, 40.4, 34.0, 33.9, 31.4, 31.0.

HRMS (+p APCI) calcd. for $[C_{21}H_{26}O_2N^{79}Br_2{}^{32}S]$ ($[M+H]^+$) 514.0046 found 514.0045.
SFC (OJ3, 20% (50% methanol in isopropanol with 0.2% Formic Acid) in CO_2, 2.5 mL/min, 1.0 mg/ml, UV 230 nm) retention times of 3.57 min (major) and 5.08 min (minor) 98% ee.

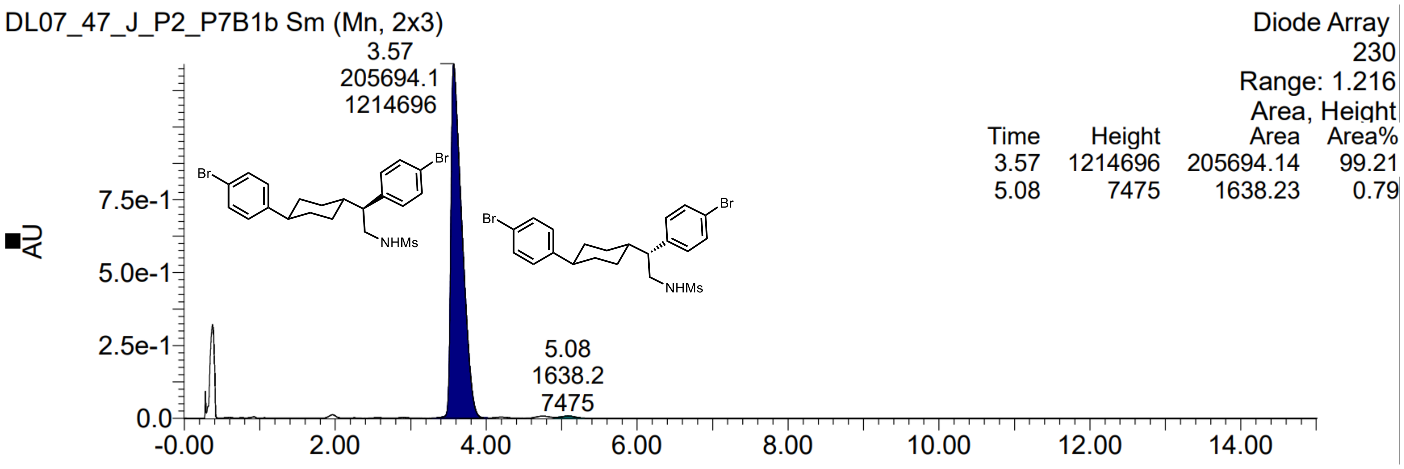

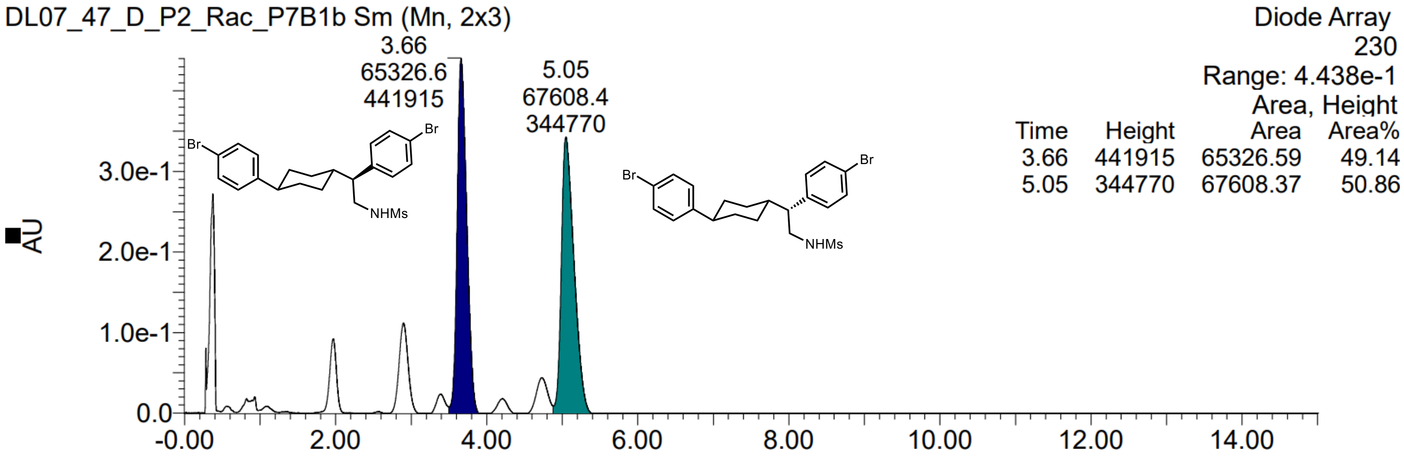

Figure S95. SFC trace of 22 (chiral – top, racemic – bottom).

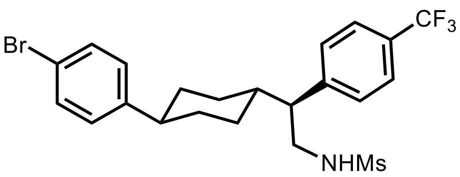

N-((S)-2-((1r,4S)-4-(4-bromophenyl)cyclohexyl)-2-(4-(trifluoromethyl)phenyl)ethyl)methanesulfonamide (23)

Prepared according to **general procedure H**, 1-bromo-4-cyclohexylbenzene (120 mg, 0.5 mmol, 2.5 equiv), Rh_2(*S*-*tetra*-$MeOC_6H_4NTTL)_4$ (3.0 mg, 0.0001 mmol, 0.005 equiv), and 1-(methylsulfonyl)-4-(4-(trifluoromethyl)phenyl)-1H-1,2,3-triazole (58.3 mg, 0.2 mmol, 1.0 equiv) were used. The crude mixture reduced by $NaBH_4$ (18.9 mg, 0.5 mmol, 2.5 equiv) at 0 ˚C then purified by flash chromatography (SiO_2, gradient 0%-65% Et_2O in hexane) afforded **23** as a white solid (51.9 mg, 51% yield, 96% ee).

R_f (3H/2EA) = 0.50 (CAM, UV 210 nm)

$[\alpha]^{20}_D$: -29.4° (c = 0.34 g/100 ml, $CHCl_3$, 96% ee)

^{1}H NMR (800 MHz, $CDCl_3$) δ 7.62 (d, *J* = 5.8 Hz, 2H), 7.38 (d, *J* = 6.8 Hz, 2H), 7.30 (d, *J* = 8.2 Hz, 2H), 7.03 (d, *J* = 6.9 Hz, 2H), 3.93 – 3.85 (m, 1H), 3.71 – 3.63 (m, 1H), 3.35 (ddd, *J* = 13.2, 10.5, 3.4 Hz, 1H), 2.83 (s, 3H), 2.73 (ddt, *J* = 10.1, 7.5, 4.3 Hz, 1H), 2.38 (td, *J* = 12.2, 3.1 Hz, 1H), 2.08 (d, *J* = 12.9 Hz, 1H), 1.95 (d, *J* = 13.3 Hz, 1H), 1.80 (d, *J* = 13.3 Hz, 1H), 1.70 – 1.61 (m, 1H), 1.52 (d, *J* = 16.3 Hz, 1H), 1.45 (q, *J* = 13.0 Hz, 1H), 1.32 (q, *J* = 12.8 Hz, 1H), 1.21 (q, *J* = 13.0 Hz, 1H), 1.00 (q, *J* = 12.9 Hz, 1H).

^{13}C NMR (201 MHz, CDCl$_3$) δ 145.8, 144.8, 131.4, 129.7 (q, J = 32.6 Hz), 128.9, 128.5, 125.9 (q, J = 3.7 Hz), 124.0 (q, J = 271.9 Hz), 119.7, 52.5, 45.8, 43.5, 40.5, 40.2, 33.8, 33.7, 31.2, 30.8.

^{19}F NMR (753 MHz, CDCl$_3$) δ -62.51.

HRMS (+p APCI) calcd. for [$C_{22}H_{26}O_2N^{79}BrF_3{}^{32}S$] ([M+H]$^+$) 504.0825 found 504.0817.
SFC (OJ3, 5% (50% methanol in isopropanol with 0.2% Formic Acid) in CO_2, 2.5 mL/min, 1.0 mg/ml, UV 230 nm) retention times of 3.94 min (major) and 5.88 min (minor) 96% ee.

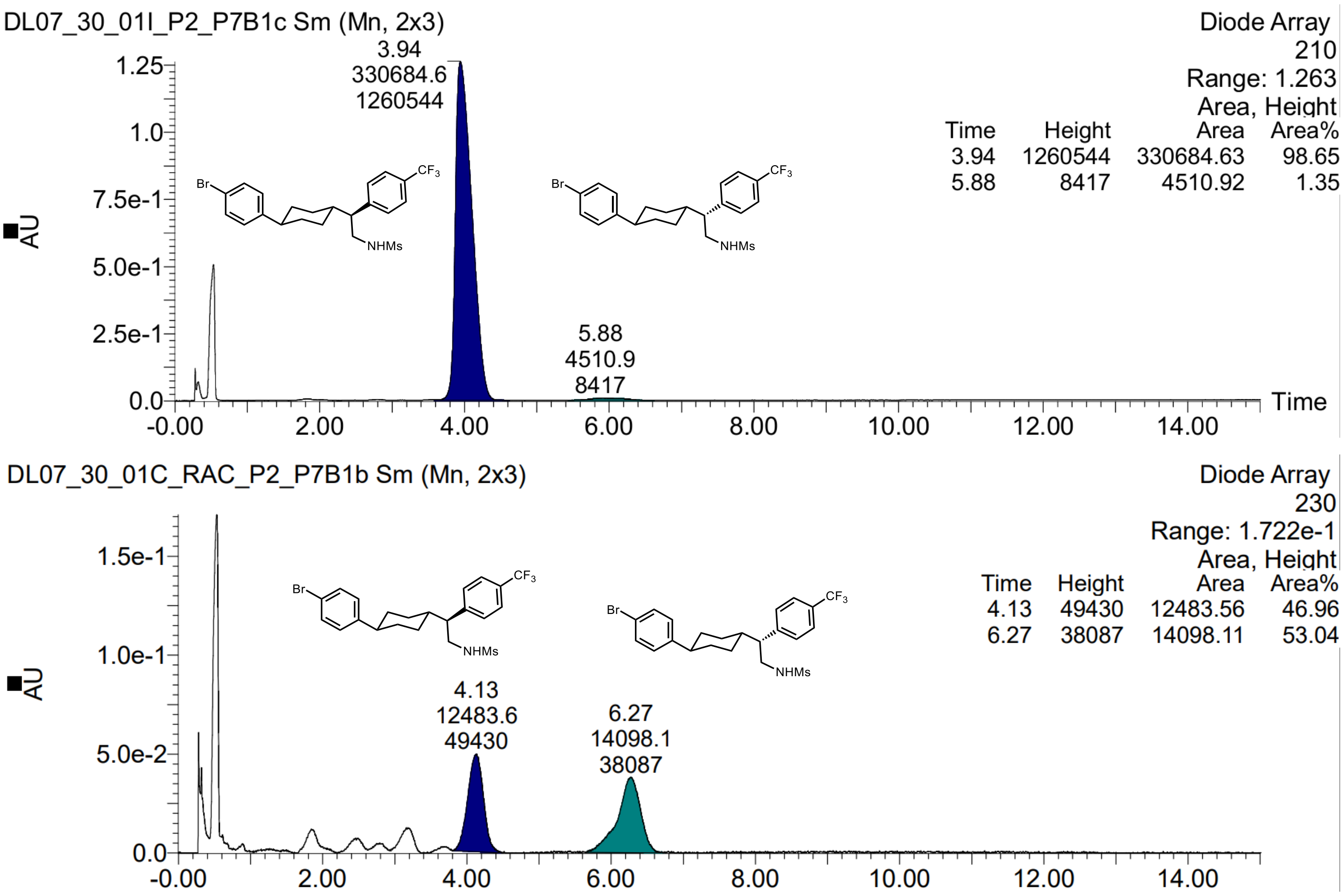

Figure S96. SFC trace of 23 (chiral – top, racemic – bottom).

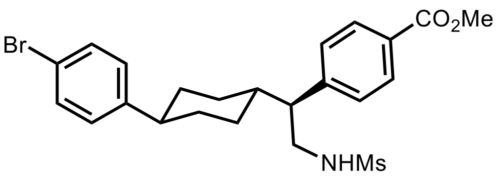

methyl 4-((S)-1-((1r,4S)-4-(4-bromophenyl)cyclohexyl)-2-(methylsulfonamido)ethyl)benzoate (24)

Prepared according to **general procedure H**, 1-bromo-4-cyclohexylbenzene (120 mg, 0.5 mmol, 2.5 equiv), Rh$_2$(S-$tetra$-MeOC$_6$H$_4$NTTL)$_4$ (3.0 mg, 0.0001 mmol, 0.005 equiv), and methyl 4-(1-(methylsulfonyl)-1H-1,2,3-triazol-4-yl)benzoate (56.3 mg, 0.2 mmol, 1.0 equiv) were used. The reaction was run for 24 hours. The crude mixture reduced by $NaBH_4$ (18.9 mg, 0.5 mmol, 2.5 equiv) at 0 ˚C then purified by flash chromatography (SiO_2, gradient 0%-75% Et_2O in hexane) afforded **24** as a white solid (79.4 mg, 80% yield, 99% ee).

R$_f$ (3H/2EA) = 0.30 (CAM, UV 210 nm)

[α]$^{20}_D$: -34.2° (c = 0.48 g/100 ml, $CHCl_3$, 99% ee)

^{1}H NMR (800 MHz, CDCl$_3$) δ 8.03 (d, *J* = 8.2 Hz, 2H), 7.38 (d, *J* = 8.4 Hz, 2H), 7.26 (d, *J* = 8.3 Hz, 2H), 7.03 (d, *J* = 8.3 Hz, 2H), 3.93 (s, 3H), 3.90 – 3.87 (m, 1H), 3.67 (ddd, *J* = 13.2, 8.3, 4.7 Hz, 1H), 3.39 – 3.33 (m, 1H), 2.81 (s, 3H), 2.74 – 2.68 (m, 1H), 2.37 (t, *J* = 12.3 Hz, 1H), 2.08 (d, *J* = 12.9 Hz, 1H), 1.94 (d, *J* = 12.2 Hz, 1H), 1.79 (d, *J* = 12.7 Hz, 1H), 1.70 – 1.63 (m, 1H), 1.51 (d, *J* = 13.4 Hz, 1H), 1.44 (q, *J* = 11.0 Hz, 1H), 1.31 (q, *J* = 12.1 Hz, 1H), 1.20 (q, *J* = 12.4 Hz, 1H), 1.00 (q, *J* = 12.8 Hz, 1H).

^{13}C NMR (201 MHz, CDCl$_3$) δ 166.8, 146.2, 146.0, 131.5, 130.3, 129.5, 128.7, 128.6, 119.8, 52.7, 52.3, 46.0, 43.7, 40.6, 40.4, 34.0, 33.9, 31.4, 31.0.

HRMS (-p APCI) calcd. for [$C_{23}H_{27}O_4N^{79}Br^{32}S$] ([M-H]$^-$) 492.0850 found 492.0853.
SFC (OJ3, 20% (50% methanol in isopropanol with 0.2% Formic Acid) in CO_2, 2.5 mL/min, 1.0 mg/ml, UV 230 nm) retention times of 4.19 min (major) and 5.55 min (minor) 99% ee.

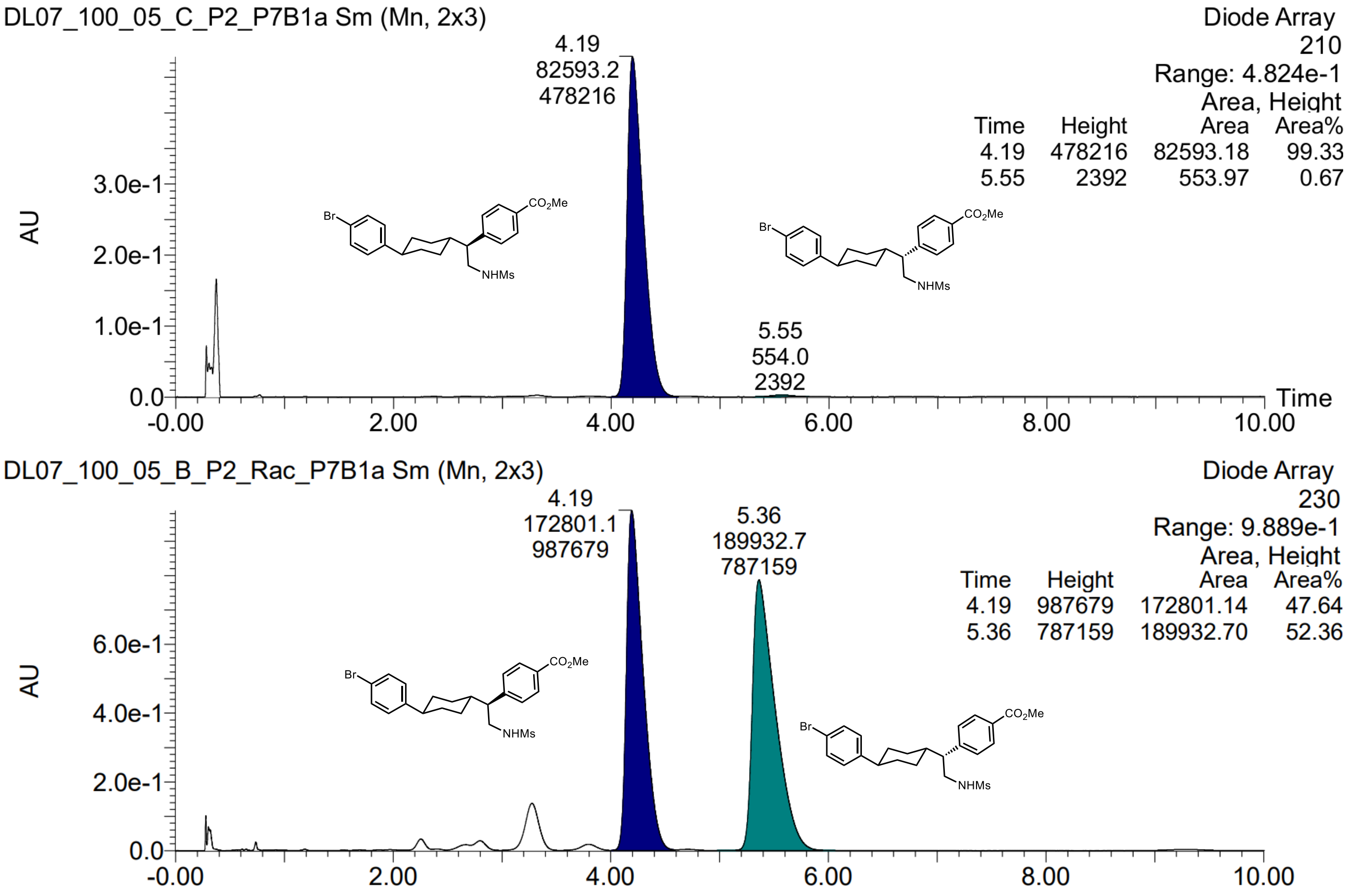

Figure S97. SFC trace of 24 (chiral – top, racemic – bottom).

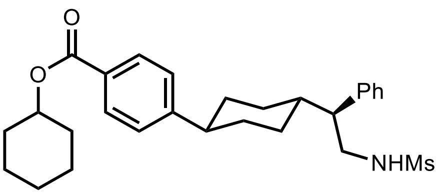

cyclohexyl 4-((1S,4r)-4-((S)-2-(methylsulfonamido)-1-phenylethyl)cyclohexyl)benzoate (25)

Prepared according to **general procedure H**, cyclohexyl 4-cyclohexylbenzoate (143 mg, 0.5 mmol, 2.5 equiv), Rh_2(*S-tetra*-$MeOC_6H_4NTTL)_4$ (3.0 mg, 0.0001 mmol, 0.005 equiv), and 1-(methylsulfonyl)-4-phenyl-1H-1,2,3-triazole (44.7 mg, 0.2 mmol, 1.0 equiv) were used. The crude mixture reduced by $NaBH_4$ (18.9 mg, 0.5 mmol, 2.5 equiv) at 0 ˚C then

purified by flash chromatography (gradient 0%-65% Et_2O in hexane) afforded **25** as a white solid (78.1 mg, 81% yield, 93% ee).

R_f (3H/2EA) = 0.45 (CAM)

$[\alpha]^{20}_D$: -25.8° (c = 0.39 g/100 ml, $CHCl_3$, 93% ee)

^{1}H NMR (600 MHz, $CDCl_3$) δ 7.95 (d, J = 8.1 Hz, 2H), 7.36 (t, J = 7.5 Hz, 2H), 7.28 (t, J = 7.5 Hz, 1H), 7.22 (d, J = 8.0 Hz, 2H), 7.17 (d, J = 6.9 Hz, 2H), 5.00 (dt, J = 8.9, 4.7 Hz, 1H), 3.90 (dd, J = 8.9, 3.6 Hz, 1H), 3.66 (ddd, J = 13.0, 8.8, 4.6 Hz, 1H), 3.33 (ddd, J = 12.4, 10.6, 3.7 Hz, 1H), 2.80 (s, 3H), 2.62 (ddd, J = 10.4, 8.5, 4.6 Hz, 1H), 2.48 (td, J = 10.5, 6.1 Hz, 1H), 2.10 (dt, J = 12.7, 3.0 Hz, 1H), 1.98 – 1.87 (m, 3H), 1.83 – 1.73 (m, 3H), 1.66 (dtt, J = 11.6, 7.1, 3.3 Hz, 1H), 1.58 (d, J = 6.0 Hz, 4H), 1.53 – 1.46 (m, 1H), 1.45 – 1.39 (m, 2H), 1.38 – 1.29 (m, 2H), 1.22 (qd, J = 12.5, 3.2 Hz, 1H), 1.02 (qd, J = 12.9, 3.5 Hz, 1H).

^{13}C NMR (151 MHz, $CDCl_3$) δ 166.1, 152.4, 140.5, 129.8, 129.1, 129.0, 128.6, 127.5, 126.8, 72.9, 52.5, 46.2, 44.4, 40.5, 40.4, 33.9, 33.8, 31.8, 31.4, 31.1, 25.6, 23.8.

HRMS (+p ESI) calcd. for [$C_{28}H_{37}O_4N^{23}Na^{32}S$] ($[M+Na]^+$) 506.2336 found 506.2338.
HPLC (Chiralpak ADH column, 10% i-propanol in hexane, 1.0 $mLmin^{-1}$, 1.0 $mgmL^{-1}$, 60 min, UV 230 nm) retention times of 30.7 min (minor) and 41.9 min (major), 93% ee.

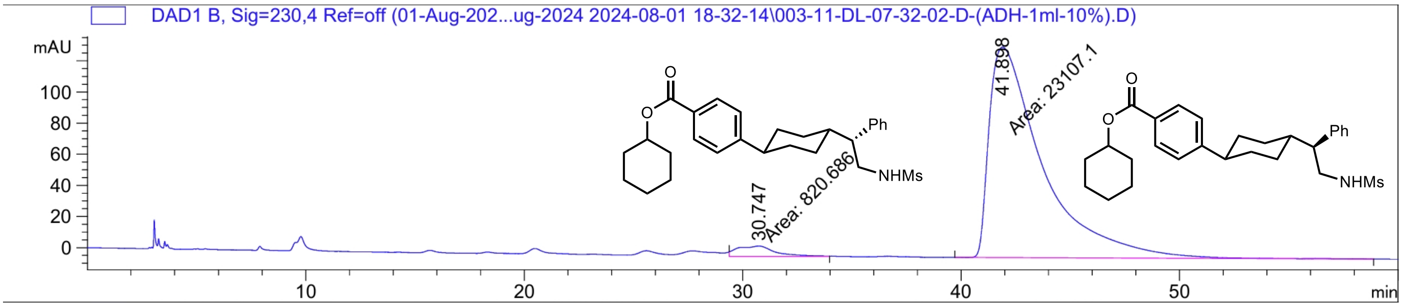

Signal 2: DAD1 B, Sig=230,4 Ref=off

Peak #	RetTime [min]	Type	Width [min]	Area [mAU*s]	Height [mAU]	Area %
1	30.747	MM	2.0354	820.68585	6.72024	3.4298
2	41.898	MM	2.8497	2.31071e4	135.14561	96.5702

Totals : 2.39278e4 141.86586

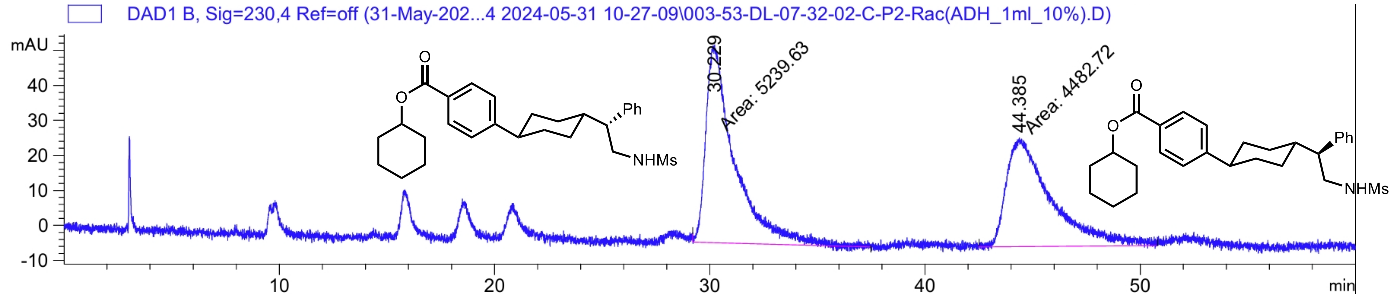

Signal 2: DAD1 B, Sig=230,4 Ref=off

Peak #	RetTime [min]	Type	Width [min]	Area [mAU*s]	Height [mAU]	Area %
1	30.229	MM	1.5459	5239.63232	56.48976	53.8926
2	44.385	MM	2.4045	4482.72314	31.07168	46.1074
Totals :				9722.35547	87.56144	

Figure S98. HPLC trace of 25 (chiral top, racemic bottom).

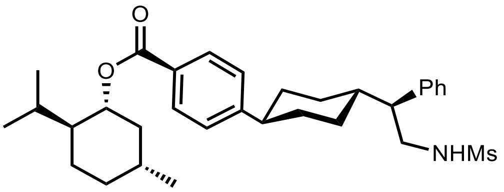

(1R,2S,5R)-2-isopropyl-5-methylcyclohexyl 4-((1S,4R)-4-((S)-2-(methylsulfonamido)-1-phenylethyl)cyclohexyl)benzoate (26)

Prepared according to **general procedure H**, (1R,2S,5R)-2-isopropyl-5-methylcyclohexyl 4-cyclohexylbenzoate (171 mg, 0.5 mmol, 2.5 equiv), Rh_2(*S*-*tetra*-$MeOC_6H_4NTTL)_4$ (3.0 mg, 0.0001 mmol, 0.005 equiv), and 1-(methylsulfonyl)-4-phenyl-1H-1,2,3-triazole (44.7 mg, 0.2 mmol, 1.0 equiv) were used. The crude mixture reduced by $NaBH_4$ (18.9 mg, 0.5 mmol, 2.5 equiv) at 0 ˚C then purified by flash chromatography (gradient 0%-65% Et_2O in hexane) afforded **26** as a white solid (80.8 mg, 75% yield, 95% de).

R_f (3H/2EA) = 0.50 (CAM)

$[\alpha]^{20}_D$: -62.3° (c = 0.40 g/100 ml, $CHCl_3$, 95% de)

^{1}H NMR (800 MHz, $CDCl_3$) δ 7.95 (d, *J* = 8.2 Hz, 2H), 7.36 (t, *J* = 7.3 Hz, 2H), 7.28 (t, *J* = 7.2 Hz, 1H), 7.23 (d, *J* = 7.1 Hz, 2H), 7.18 (d, *J* = 7.8 Hz, 2H), 4.91 (td, *J* = 10.9, 3.3 Hz, 1H), 3.91 (d, *J* = 8.9 Hz, 1H), 3.72 – 3.62 (m, 1H), 3.33 (ddd, *J* = 12.8, 8.0, 3.0 Hz, 1H), 2.80 (s, 3H), 2.62 (ddd, *J* = 13.6, 6.8, 2.9 Hz, 1H), 2.49 (t, *J* = 11.3 Hz, 1H), 2.10 (d, *J* = 9.9 Hz, 2H), 2.00 – 1.92 (m, 2H), 1.81 (d, *J* = 12.6 Hz, 1H), 1.75 – 1.69 (m, 2H), 1.69 – 1.62 (m, 1H), 1.56 – 1.52 (m, 2H), 1.52 – 1.46 (m, 1H), 1.37 (q, *J* = 12.9 Hz, 1H), 1.22 (q, *J* = 12.5 Hz, 1H), 1.12 (q, *J* = 11.7 Hz, 1H), 1.07 (q, *J* = 12.3 Hz, 1H), 1.02 (q, *J* = 11.7 Hz, 1H), 0.92 (d, *J* = 6.9 Hz, 3H), 0.90 (d, *J* = 7.2 Hz, 3H), 0.78 (d, *J* = 7.1 Hz, 3H).

^{13}C NMR (201 MHz, $CDCl_3$) δ 166.2, 152.4, 140.6, 129.9, 129.1, 128.8, 128.6, 127.5, 126.9, 74.7, 52.5, 47.4, 46.2, 44.4, 41.1, 40.5, 40.4, 34.5, 33.9, 33.8, 31.6, 31.4, 31.1, 26.6, 23.8, 22.2, 20.9, 16.6.

HRMS (-p APCI) calcd. for $[C_{32}H_{44}O_4N^{32}S]$ ($[M-H]^-$) 538.2997 found 538.2998.

HPLC (Chiralpak ADH column, 10% i-propanol in hexane, 1.0 $mLmin^{-1}$, 1.0 $mgmL^{-1}$, 60 min, UV 230 nm) retention times of 23.5 min (minor) and 29.4 min (major), 95% de.

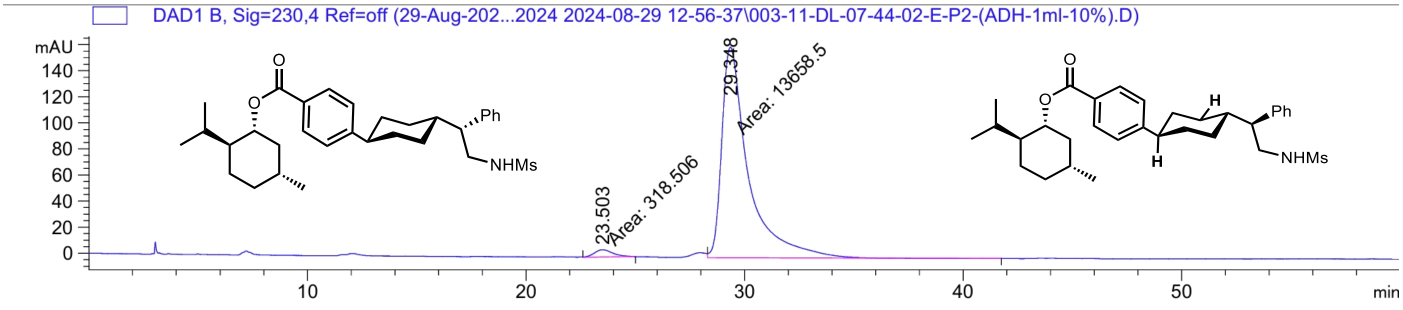

Signal 2: DAD1 B, Sig=230,4 Ref=off

Peak #	RetTime [min]	Type	Width [min]	Area [mAU*s]	Height [mAU]	Area %
1	23.503	MM	0.9568	318.50589	5.54838	2.2788
2	29.348	FM	1.4102	1.36585e4	161.42931	97.7212
Totals :				1.39770e4	166.97768	

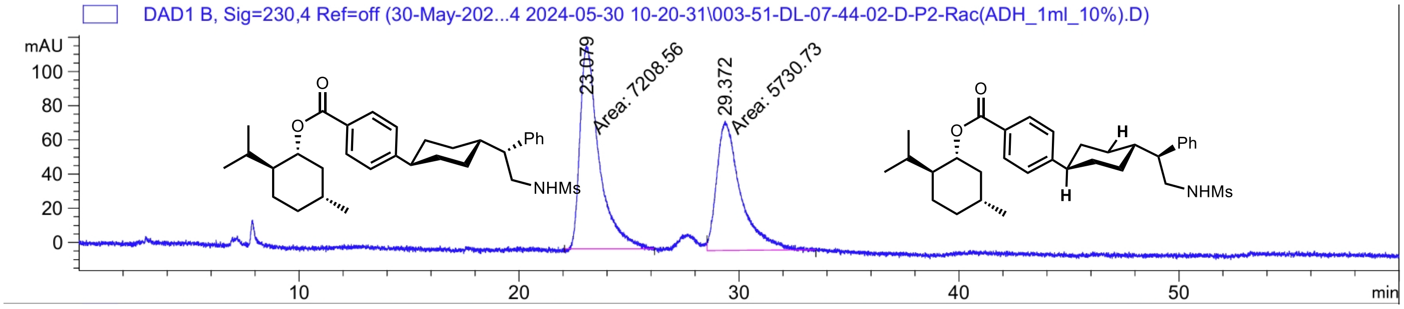

Signal 2: DAD1 B, Sig=230,4 Ref=off

Peak #	RetTime [min]	Type	Width [min]	Area [mAU*s]	Height [mAU]	Area %
1	23.079	MM	1.0125	7208.55957	118.65873	55.7106
2	29.372	MM	1.2633	5730.72852	75.60625	44.2894
Totals :				1.29393e4	194.26498	

Figure S99. HPLC trace of 26 (chiral top, racemic bottom).

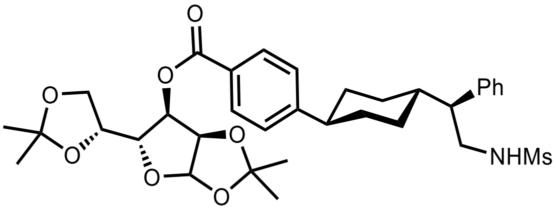

(5R,6R,6aR)-5-((R)-2,2-dimethyl-1,3-dioxolan-4-yl)-2,2-dimethyltetrahydrofuro[2,3-d][1,3]dioxol-6-yl 4-((1S,4R)-4-((S)-2-(methylsulfonamido)-1-phenylethyl)cyclohexyl)benzoate (27)

Prepared according to **general procedure H**, (3aR,5R,6S,6aR)-5-((R)-2,2-dimethyl-1,3-dioxolan-4-yl)-2,2-dimethyltetrahydrofuro[2,3-d][1,3]dioxol-6-yl 4-cyclohexylbenzoate (223 mg, 0.5 mmol, 2.5 equiv), Rh_2(*S-tetra*-$MeOC_6H_4$NTTL$)_4$ (3.0 mg, 0.0001 mmol, 0.005 equiv), and 1-(methylsulfonyl)-4-phenyl-1H-1,2,3-triazole (44.7 mg, 0.2 mmol, 1.0 equiv) were used. The crude mixture reduced by $NaBH_4$ (18.9 mg, 0.5 mmol, 2.5 equiv) at 0 ˚C then purified by flash chromatography (gradient 0%-40% ethyl acetate in hexane) afforded **27** as a white solid (80.1 mg, 62% yield, 98% de).

R_f (3H/2EA) = 0.30 (UV 254 nm, CAM)

$[\alpha]^{20}_D$: +60.7° (c = 0.17 g/100 ml, $CHCl_3$, 98% de)

^{1}H NMR (600 MHz, $CDCl_3$) δ 7.95 (d, J = 8.0 Hz, 2H), 7.36 (t, J = 7.5 Hz, 2H), 7.28 (t, J = 7.4 Hz, 1H), 7.24 (d, J = 8.0 Hz, 2H), 7.17 (d, J = 6.8 Hz, 2H), 5.88 (d, J = 3.8 Hz, 1H), 5.04 (dd, J = 8.6, 5.1 Hz, 1H), 4.96 (t, J = 4.5 Hz, 1H), 4.39 – 4.29 (m, 2H), 4.10 (dd, J = 8.6, 6.8 Hz, 1H), 3.97 (dd, J = 8.6, 5.8 Hz, 1H), 3.91 (dd, J = 8.8, 3.7 Hz, 1H), 3.66 (ddd, J = 13.0, 8.7, 4.6 Hz, 1H), 3.33 (ddd, J = 12.3, 10.5, 3.7 Hz, 1H), 2.80 (s, 3H), 2.62 (ddd, J = 13.5, 9.1, 4.6 Hz,

1H), 2.49 (tt, J = 12.2, 3.4 Hz, 1H), 2.10 (dt, J = 13.1, 3.1 Hz, 1H), 1.97 (dt, J = 13.2, 3.1 Hz, 1H), 1.81 (dt, J = 13.2, 3.1 Hz, 1H), 1.66 (dtd, J = 14.8, 9.4, 2.9 Hz, 1H), 1.61 – 1.54 (m, 2H), 1.53 (s, 3H), 1.49 (td, J = 12.9, 3.4 Hz, 1H), 1.40 (s, 3H), 1.39 – 1.35 (m, 1H), 1.33 (s, 3H), 1.31 (s, 3H), 1.23 (qd, J = 12.7, 3.5 Hz, 1H), 1.03 (qd, J = 12.9, 3.5 Hz, 1H).

^{13}C NMR (151 MHz, $CDCl_3$) δ 165.7, 153.2, 140.5, 130.1, 129.1, 128.6, 127.5, 127.3, 127.0, 113.2, 110.1, 104.4, 78.0, 77.9, 75.2, 73.1, 65.8, 52.5, 46.2, 44.4, 40.5, 40.4, 33.8, 33.8, 31.4, 31.1, 26.9, 26.8, 26.5, 25.2.

HRMS (-p APCI) calcd. for [$C_{34}H_{44}O_9N^{32}S$] ([M-H]$^-$) 642.2742 found 642.2737.

SFC (OJ3, 7% (50% methanol in isopropanol with 0.2% Formic Acid) in CO_2, 2.5 mL/min, 1.0 mg/ml, UV 230 nm) retention times of 9.96 min (major) and 10.7 min (minor) 98% de.

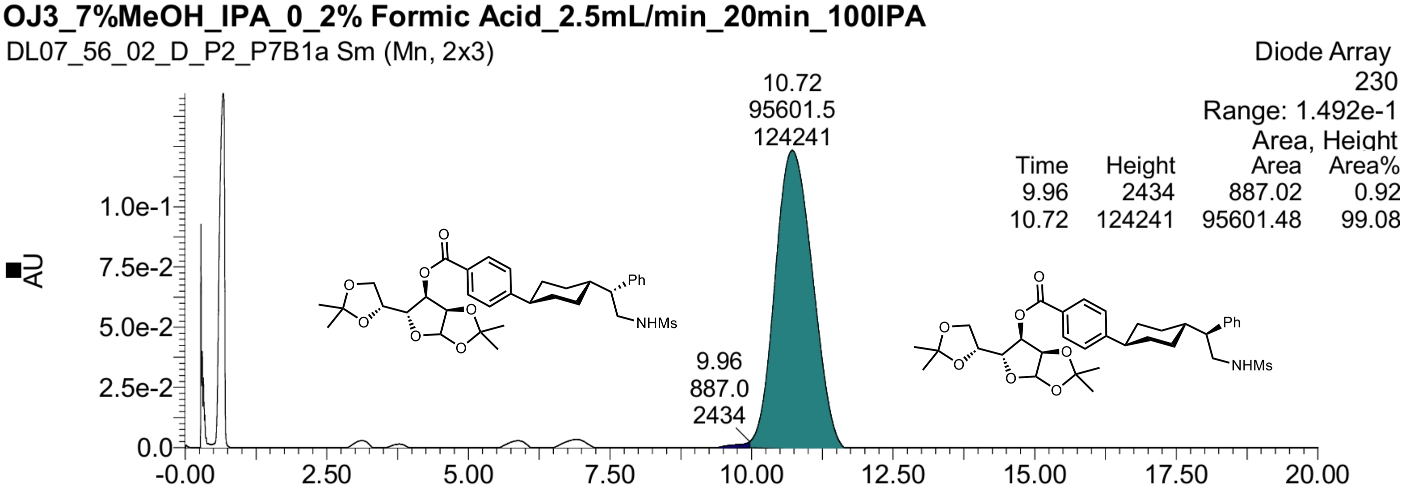

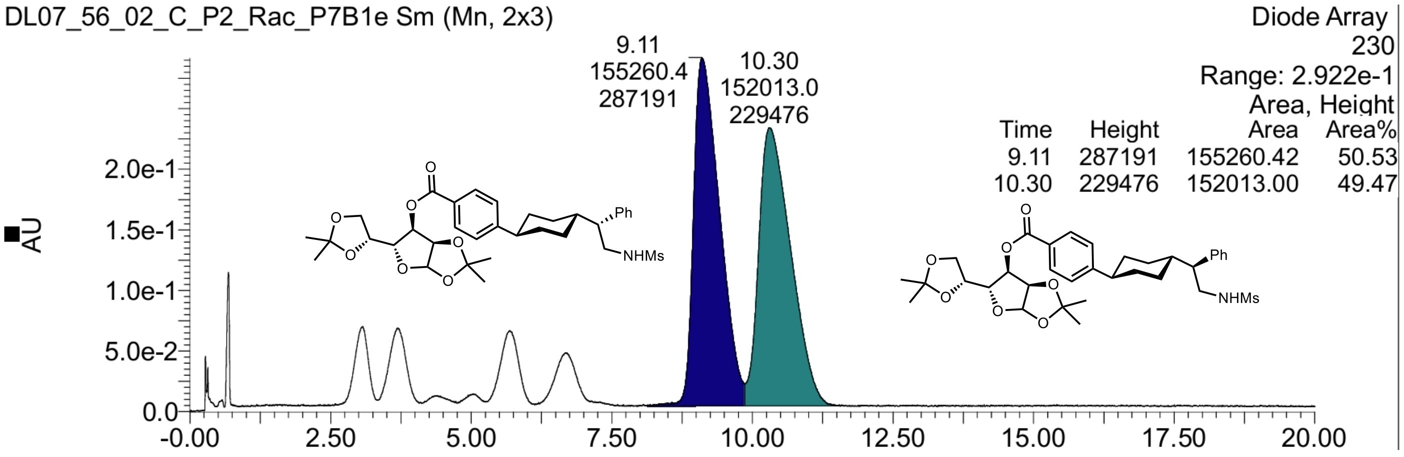

Figure S100. SFC trace of 27 (chiral top, racemic bottom).

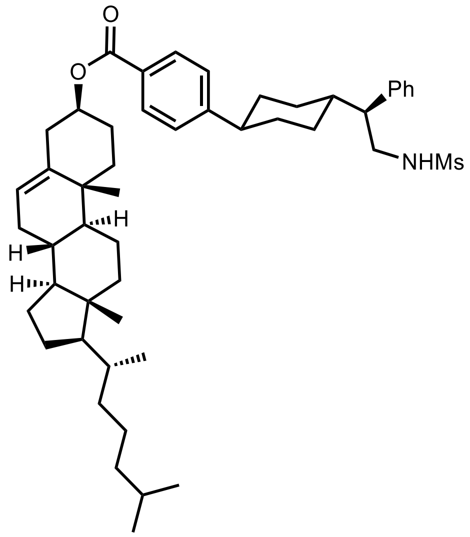

(3S,8S,9S,10R,13R,14S,17R)-10,13-dimethyl-17-((R)-6-methylheptan-2-yl)-2,3,4,7,8,9,10,11,12,13,14,15,16,17-tetradecahydro-1H-cyclopenta[a]phenanthren-3-yl 4-((1S,4S)-4-((S)-2-(methylsulfonamido)-1-phenylethyl)cyclohexyl)benzoate (28)

Prepared according to **general procedure H**, (3R,8S,9S,10R,13R,14S,17R)-17-((R)-heptan-2-yl)-10,13-dimethyl-2,3,4,7,8,9,10,11,12,13,14,15,16,17-tetradecahydro-1H-cyclopenta[a]phenanthren-3-yl 4-cyclohexylbenzoate (279 mg, 0.5 mmol, 2.5 equiv), Rh_2(*S-tetra*-MeOC_6H_4NTTL)$_4$ (3.0 mg, 0.0001 mmol, 0.005 equiv), and 1-(methylsulfonyl)-4-phenyl-1H-1,2,3-triazole (44.7 mg, 0.2 mmol, 1.0 equiv) were used. The crude mixture reduced by $NaBH_4$ (18.9 mg, 0.5 mmol, 2.5 equiv) at 0 ˚C then purified by flash chromatography (gradient 0%-65% Et_2O in hexane) afforded **28** as a white solid (129 mg, 85% yield, 96% de).

R$_f$ (3H/2EA) = 0.40 (CAM)

$[\alpha]^{20}_D$: -18.5^o (c = 0.76 g/100 ml, $CHCl_3$, 96% de)

^{1}H NMR (800 MHz, $CDCl_3$) δ 7.94 (d, *J* = 8.3 Hz, 2H), 7.36 (t, *J* = 7.5 Hz, 2H), 7.28 (t, *J* = 7.5 Hz, 1H), 7.22 (d, *J* = 8.0 Hz, 2H), 7.17 (d, *J* = 7.6 Hz, 2H), 5.41 (s, 1H), 4.84 (dq, *J* = 8.5, 3.9 Hz, 1H), 3.92 (dd, *J* = 8.8, 3.7 Hz, 1H), 3.66 (ddd, *J* = 13.0, 8.5, 4.6 Hz, 1H), 3.33 (dt, *J* = 12.2, 6.8 Hz, 1H), 2.80 (s, 3H), 2.65 – 2.58 (m, 1H), 2.48 (t, *J* = 12.4 Hz, 1H), 2.44 (d, *J* = 8.3 Hz, 2H), 2.10 (d, *J* = 13.0 Hz, 1H), 2.02 (d, *J* = 12.7 Hz, 1H), 2.00 – 1.94 (m, 3H), 1.90 (d, *J* = 14.2 Hz, 1H), 1.86 – 1.80 (m, 2H), 1.75 – 1.62 (m, 2H), 1.60 – 1.55 (m, 3H), 1.53 – 1.50 (m, 2H), 1.49 – 1.45 (m, 2H), 1.40 – 1.32 (m, 4H), 1.29 – 1.24 (m, 2H), 1.24 – 1.17 (m, 3H), 1.17 – 1.07 (m, 5H), 1.06 (s, 3H), 1.04 – 0.96 (m, 4H), 0.92 (d, *J* = 6.5 Hz, 3H), 0.88 – 0.85 (m, 6H), 0.69 (s, 3H).

^{13}C NMR (151 MHz, $CDCl_3$) δ 166.1, 152.4, 140.6, 139.8, 129.8, 129.1, 128.8, 128.6, 127.5, 126.8, 122.9, 74.5, 56.8, 56.3, 52.5, 50.2, 46.2, 44.4, 42.5, 40.5, 40.4, 39.9, 39.7, 38.4, 37.2, 36.8, 36.3, 35.9, 33.9, 33.8, 32.1, 32.0, 31.4, 31.1, 28.4, 28.2, 28.0, 24.4, 24.0, 23.0, 22.7, 21.2, 19.5, 18.9, 12.0.

HRMS (+p ESI) calcd. for [$C_{49}H_{71}O_4N^{23}Na^{32}S$] ([M+Na]$^+$) 792.4996 found 792.4996.
HPLC (Chiralpak ADH column, 5% i-propanol in hexane, 1.0 mLmin^{-1}, 1.0 mgmL^{-1}, 60 min, UV 230 nm) retention times of 24.8 min (minor) and 28.1 min (major), 96% de.

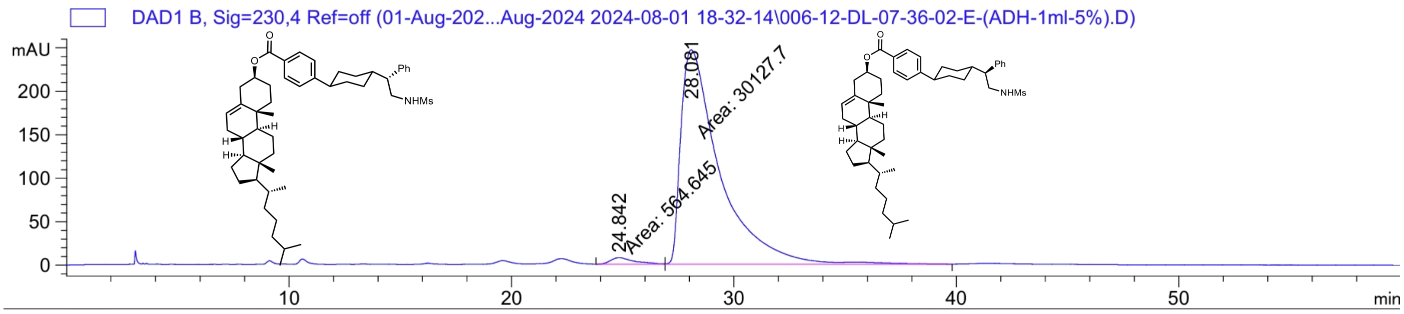

Signal 2: DAD1 B, Sig=230,4 Ref=off

Peak #	RetTime [min]	Type	Width [min]	Area [mAU*s]	Height [mAU]	Area %
1	24.842	MF	1.2573	564.64545	7.48506	1.8397
2	28.081	FM	2.0367	3.01277e4	246.53653	98.1603
Totals :				3.06923e4	254.02159	

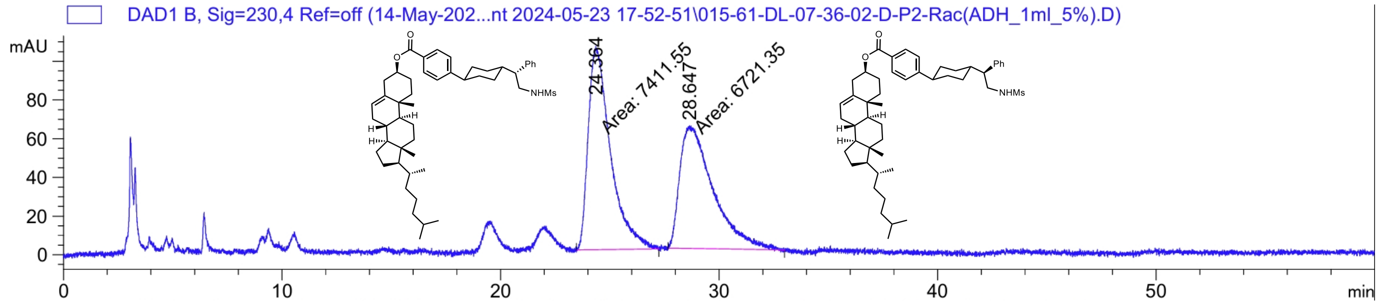

Signal 2: DAD1 B, Sig=230,4 Ref=off

Peak #	RetTime [min]	Type	Width [min]	Area [mAU*s]	Height [mAU]	Area %
1	24.364	MM	1.1771	7411.54980	104.94124	52.4418
2	28.647	MM	1.7612	6721.34912	63.60442	47.5582
Totals :				1.41329e4	168.54566	

Figure S101. HPLC trace of 28 (chiral top, racemic bottom).

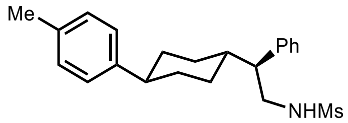

N-((S)-2-phenyl-2-((1r,4S)-4-(p-tolyl)cyclohexyl)ethyl)methanesulfonamide (29)

Prepared according to **general procedure H**, 1-cyclohexyl-4-methylbenzene (87.1 mg, 0.5 mmol, 2.5 equiv), Rh_2(*S*-*tetra*-$CF_3C_6H_4$NTTL)$_4$ (3.0 mg, 0.0001 mmol, 0.005 equiv), and 1-(methylsulfonyl)-4-phenyl-1H-1,2,3-triazole (44.7 mg, 0.2 mmol, 1.0 equiv) were used. The reaction was stirred for 24 hours. The crude mixture was reduced by $NaBH_4$ (18.9 mg, 0.5 mmol, 2.5 equiv) at 0 ˚C then purified by flash chromatography (SiO_2, gradient 0%-65% Et_2O in hexane) afforded **29** as a white solid (58.1 mg, 77% yield, 96% ee).

R_f (3H/2EA) = 0.50 (CAM, weak UV 254 nm)

$[\alpha]^{20}_D$: -31.8º (c = 0.17 g/100 ml, $CHCl_3$, 96% ee)

^{1}H NMR (800 MHz, CDCl$_3$) δ 7.36 (t, *J* = 7.6 Hz, 2H), 7.28 (t, *J* = 7.4 Hz, 1H), 7.18 (d, *J* = 7.5 Hz, 2H), 7.09 (d, *J* = 7.8 Hz, 2H), 7.06 (d, *J* = 8.7 Hz, 2H), 3.89 (dd, *J* = 8.9, 3.5 Hz, 1H), 3.66 (td, *J* = 11.2, 3.1 Hz, 1H), 3.33 (t, *J* = 11.6 Hz, 1H), 2.80 (s, 3H), 2.63 – 2.58 (m, 1H), 2.39 (t, *J* = 11.8 Hz, 1H), 2.30 (s, 3H), 2.07 (d, *J* = 10.8 Hz, 1H), 1.95 (d, *J* = 11.6 Hz, 1H), 1.80 (d, *J* = 12.0 Hz, 1H), 1.64 (q, *J* = 10.5 Hz, 1H), 1.54 (d, *J* = 13.2 Hz, 1H), 1.47 (q, *J* = 12.5 Hz, 1H), 1.34 (q, *J* = 12.4 Hz, 1H), 1.21 (q, *J* = 12.1 Hz, 1H), 1.01 (q, *J* = 12.2 Hz, 1H).

^{13}C NMR (201 MHz, CDCl$_3$) δ 144.3, 140.7, 135.6, 129.2, 129.1, 128.6, 127.5, 126.7, 52.6, 46.3, 43.9, 40.7, 40.3, 34.3, 34.2, 31.6, 31.3, 21.1.

HRMS (+p APCI) calcd. for [$C_{22}H_{30}O_2N^{32}S$] ([M+H]$^+$) 372.1992 found 372.1994.
SFC (OJ3, 10% (50% methanol in isopropanol with 0.2% Formic Acid) in CO_2, 2.5 mL/min, 1.0 mg/ml, UV 210 nm) retention times of 7.48 min (major) and 10.6 min (minor) 96% ee.

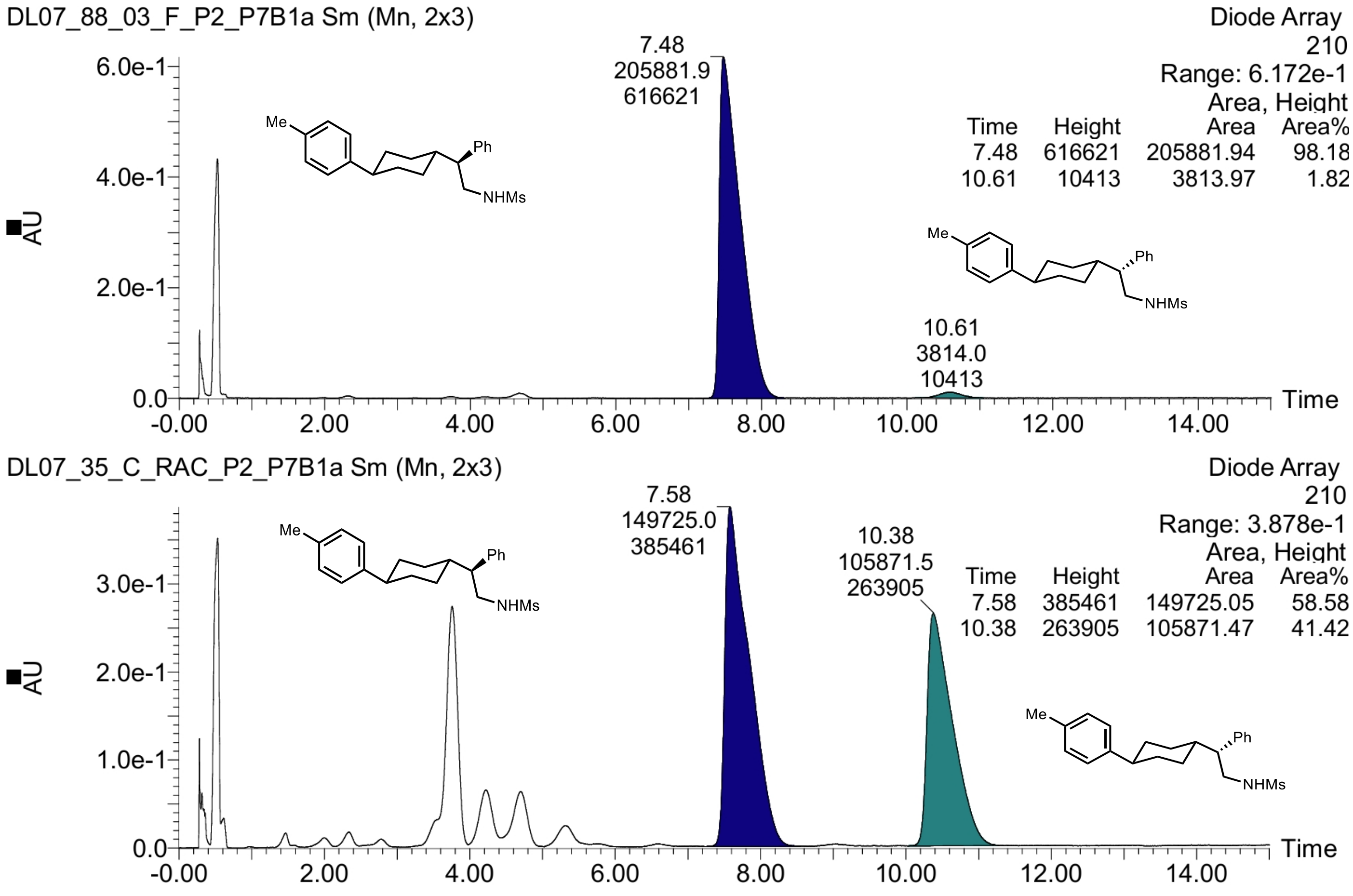

Figure S102. SFC trace of 29 (chiral – top, racemic – bottom).

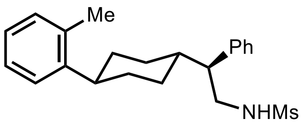

N-((S)-2-phenyl-2-((1r,4S)-4-(o-tolyl)cyclohexyl)ethyl)methanesulfonamide (30)

Prepared according to **general procedure H**, 1-cyclohexyl-2-methylbenzene (87.1 mg, 0.5 mmol, 2.5 equiv), Rh$_2$(*S*-*tetra*-$CF_3C_6H_4$NTTL)$_4$ (3.0 mg, 0.0001 mmol, 0.005 equiv), and 1-(methylsulfonyl)-4-phenyl-1H-1,2,3-triazole (44.7 mg, 0.2 mmol, 1.0 equiv) were used. The reaction was stirred for 24 hours. The crude mixture was reduced by $NaBH_4$

(18.9 mg, 0.5 mmol, 2.5 equiv) at 0 ˚C then purified by flash chromatography (SiO_2, gradient 0%-65% Et_2O in hexane) afforded **30** as a white solid (35.0 mg, 47% yield, 90% ee). *Isolated as a 9:1 mixture of C4 and C3 products.*

R$_f$ (3H/2EA) = 0.50 (CAM, UV 210 nm)

$[\alpha]^{20}_D$: -17.7° (c = 0.23 g/100 ml, $CHCl_3$, 90% ee)

^{1}H NMR (800 MHz, $CDCl_3$) δ 7.39 – 7.34 (m, 2H), 7.31 – 7.27 (m, 1H), 7.20 – 7.17 (m, 2H), 7.15 (d, J = 6.2 Hz, 2H), 7.12 (d, J = 7.5 Hz, 1H), 7.07 (t, J = 6.9 Hz, 1H), 3.90 (d, J = 8.6 Hz, 1H), 3.71 – 3.65 (m, 1H), 3.37 – 3.31 (m, 1H), 2.81 (s, 3H), 2.63 (td, J = 11.5, 3.1 Hz, 2H), 2.29 (s, 3H), 2.10 (dd, J = 12.9, 3.1 Hz, 1H), 1.90 (dd, J = 13.3, 3.0 Hz, 1H), 1.75 (dd, J = 13.2, 3.0 Hz, 1H), 1.70 – 1.65 (m, 1H), 1.59 – 1.53 (m, 1H), 1.48 (q, J = 12.9 Hz, 1H), 1.36 (q, J = 12.8 Hz, 1H), 1.26 – 1.22 (m, 1H), 1.05 (q, J = 12.8 Hz, 1H).

^{13}C NMR (201 MHz, $CDCl_3$) δ 145.1, 140.7, 135.3, 130.4, 129.1, 128.6, 127.5, 126.3, 125.8, 125.3, 52.7, 46.3, 40.8, 40.4, 39.8, 33.3, 33.2, 31.8, 31.5, 19.5.

HRMS (+p APCI) calcd. for $[C_{22}H_{30}O_2N^{32}S]$ ($[M+H]^+$) 372.1992 found 372.1995.
SFC (CEL2, 10% (50% methanol in isopropanol with 0.2% Formic Acid) in CO_2, 2.5 mL/min, 1.0 mg/ml, UV 210 nm) retention times of 7.01 min (major) and 8.26 min (minor) 90% ee.

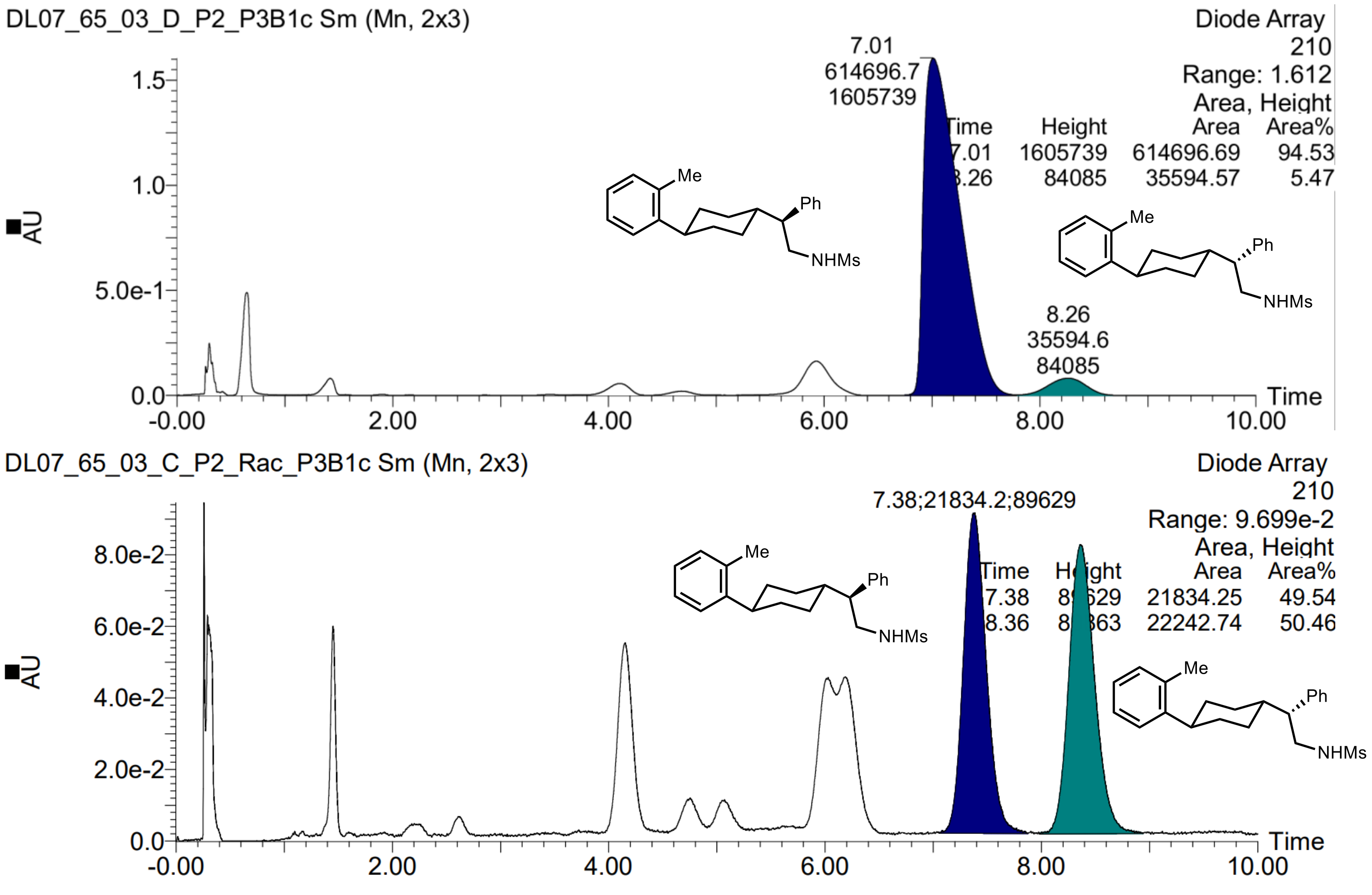

Figure S103. SFC trace of 30 (chiral – top, racemic – bottom).

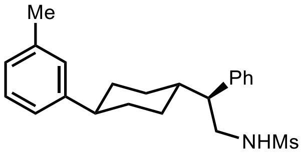

N-((S)-2-phenyl-2-((1r,4S)-4-(m-tolyl)cyclohexyl)ethyl)methanesulfonamide (31)

Prepared according to **general procedure H**, 1-cyclohexyl-3-methylbenzene (87.1 mg, 0.5 mmol, 2.5 equiv), Rh_2(*S-tetra*-$CF_3C_6H_4$NTTL$)_4$ (3.0 mg, 0.0001 mmol, 0.005 equiv), and 1-(methylsulfonyl)-4-phenyl-1H-1,2,3-triazole (44.7 mg, 0.2 mmol, 1.0 equiv) were used. The reaction was stirred for 48 hours. The crude mixture was reduced by $NaBH_4$ (18.9 mg, 0.5 mmol, 2.5 equiv) at 0 ˚C then purified by flash chromatography (SiO_2, gradient 0%-65% Et_2O in hexane) afforded **31** as a white solid (55.8 mg, 75% yield, 95% ee).

R_f (3H/2EA) = 0.50 (CAM, UV 210 nm)

$[\alpha]^{20}_D$: -24.5° (c = 0.31 g/100 ml, $CHCl_3$, 95% ee)

^{1}H NMR (800 MHz, $CDCl_3$) δ 7.36 (t, *J* = 7.6 Hz, 2H), 7.30 – 7.27 (m, 1H), 7.20 – 7.14 (m, 3H), 7.01 – 6.98 (m, 2H), 6.97 (d, *J* = 8.0 Hz, 1H), 3.93 (d, *J* = 8.5 Hz, 1H), 3.70 – 3.62 (m, 1H), 3.33 (dd, *J* = 13.1, 10.0 Hz, 1H), 2.80 (s, 3H), 2.65 – 2.57 (m, 1H), 2.39 (t, *J* = 12.2 Hz, 1H), 2.32 (s, 3H), 2.08 (dd, *J* = 12.9, 3.1 Hz, 1H), 1.96 (dd, *J* = 13.2, 3.1 Hz, 1H), 1.81 (dd, *J* = 13.1, 3.1 Hz, 1H), 1.68 – 1.62 (m, 1H), 1.55 (dd, *J* = 13.3, 3.1 Hz, 1H), 1.49 (q, *J* = 12.9 Hz, 1H), 1.36 (q, *J* = 12.8 Hz, 1H), 1.21 (q, *J* = 12.9 Hz, 1H), 1.01 (q, *J* = 12.8 Hz, 1H).

^{13}C NMR (201 MHz, $CDCl_3$) δ 147.3, 138.0, 129.1, 128.6, 128.4, 127.7, 127.4, 126.9, 123.8, 52.6, 46.3, 44.2, 40.6, 40.3, 34.2, 34.1, 31.6, 31.3, 21.6.

HRMS (+p APCI) calcd. for $[C_{22}H_{30}O_2N^{32}S]$ ($[M+H]^+$) 372.1992 found 372.1991.

SFC (OJ3, 10% (50% methanol in isopropanol with 0.2% Formic Acid) in CO_2, 2.5 mL/min, 1.0 mg/ml, UV 210 nm) retention times of 4.31 min (major) and 5.23 min (minor) 95% ee.

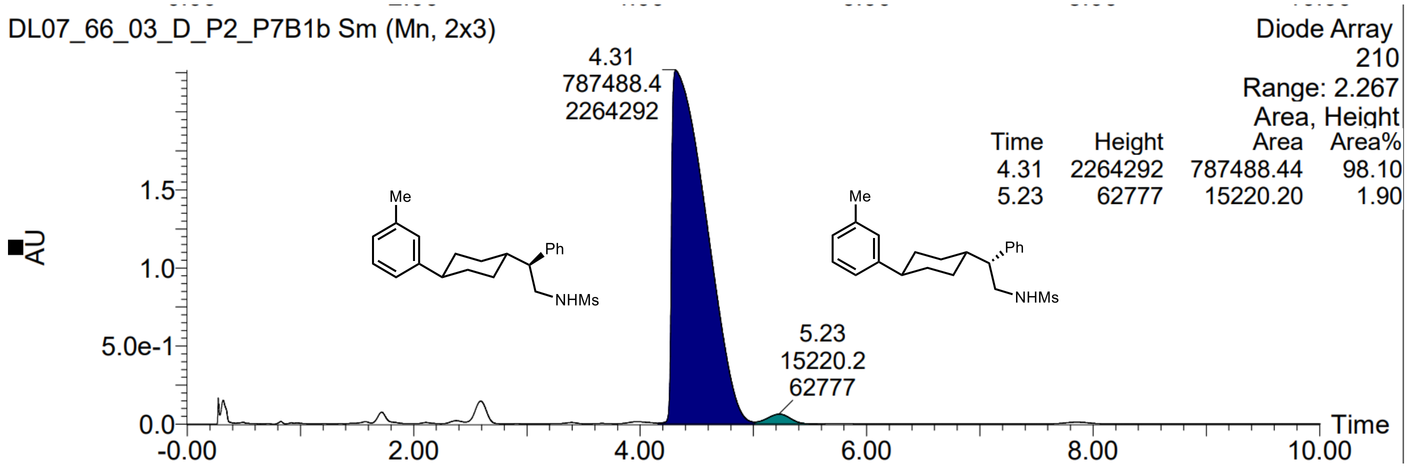

Time	Height	Area	Area%
4.31	2264292	787488.44	98.10
5.23	62777	15220.20	1.90

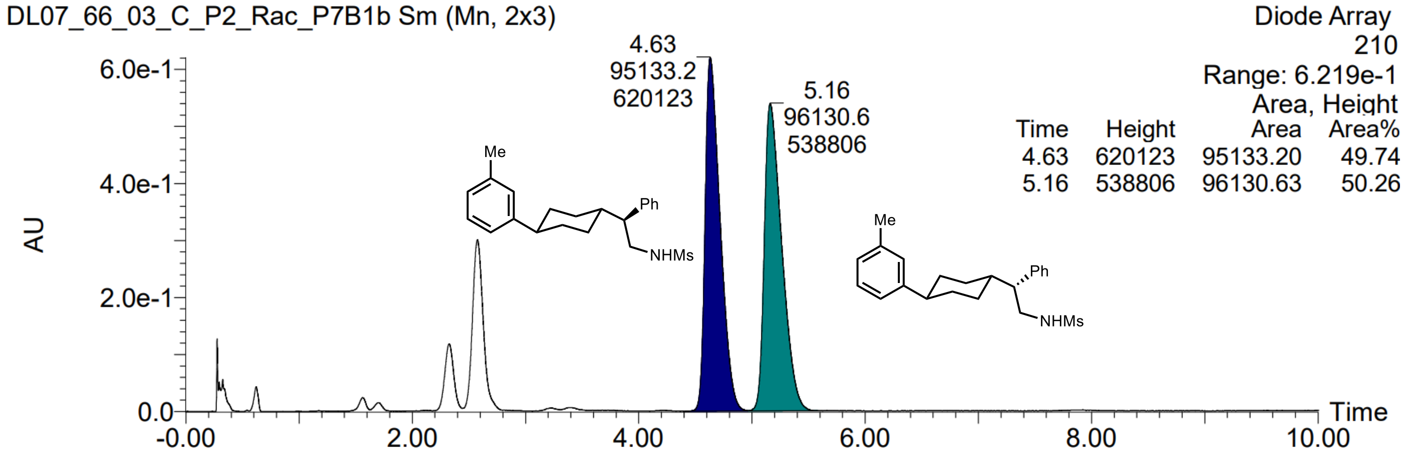

Time	Height	Area	Area%
4.63	620123	95133.20	49.74
5.16	538806	96130.63	50.26

Figure S104. SFC trace of 31 (chiral – top, racemic – bottom).

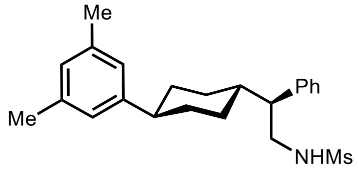

N-((S)-2-((1r,4S)-4-(3,5-dimethylphenyl)cyclohexyl)-2-phenylethyl)methanesulfonamide (32)

Prepared according to **general procedure H,** 1-cyclohexyl-3,5-dimethylbenzene (94.2 mg, 0.5 mmol, 2.5 equiv), Rh_2(*S-tetra*-$CF_3C_6H_4$NTTL)$_4$ (3.0 mg, 0.0001 mmol, 0.005 equiv), and 1-(methylsulfonyl)-4-phenyl-1H-1,2,3-triazole (44.7 mg, 0.2 mmol, 1.0 equiv) were used. The crude mixture was reduced by $NaBH_4$ (18.9 mg, 0.5 mmol, 2.5 equiv) at 0 ˚C then purified by flash chromatography (gradient 0%-65% Et_2O in hexane) afforded **32** as a white solid (64.3 mg, 83% yield, 96% ee).

R_f (3H/2EA) = 0.40 (CAM)

$[\alpha]^{20}_D$: -21.9° (c = 0.91 g/100 ml, $CHCl_3$, 96% ee)

^{1}H NMR (800 MHz, $CDCl_3$) δ 7.36 (t, *J* = 7.5 Hz, 2H), 7.28 (t, *J* = 7.6 Hz, 1H), 7.18 (d, *J* = 8.4 Hz, 2H), 6.82 (s, 1H), 6.79 (s, 2H), 3.96 – 3.86 (m, 1H), 3.72 – 3.62 (m, 1H), 3.33 (ddd, *J* = 15.1, 8.1, 3.1 Hz, 1H), 2.80 (s, 3H), 2.66 – 2.55 (m, 1H), 2.35 (t, *J* = 12.2 Hz, 1H), 2.28 (s, 6H), 2.08 (d, *J* = 12.8 Hz, 1H), 1.95 (d, *J* = 13.3 Hz, 1H), 1.80 (d, *J* = 12.5 Hz, 1H), 1.64 (q, *J* = 8.5 Hz, 1H), 1.54 (d, *J* = 13.0 Hz, 1H), 1.48 (q, *J* = 12.9 Hz, 1H), 1.36 (q, *J* = 12.9 Hz, 1H), 1.20 (q, *J* = 12.7 Hz, 1H), 1.00 (q, *J* = 12.8 Hz, 1H).

^{13}C NMR (201 MHz, $CDCl_3$) δ 147.3, 140.7, 137.9, 129.1, 128.6, 127.8, 127.4, 124.7, 52.6, 46.3, 44.2, 40.7, 40.3, 34.2, 34.1, 31.6, 31.4, 21.5.

HRMS (-p APCI) calcd. [$C_{23}H_{30}O_2N^{32}S$] ($[M-H]^-$) for 384.2003 found 384.1996.
SFC (CEL-2, 10% (50% methanol in isopropanol with 0.2% Formic Acid) in CO_2, 2.5 mL/min, 1.0 mg/ml, UV 210 nm) retention times of 5.94 min (major) and 7.29 min (minor) 96% ee.

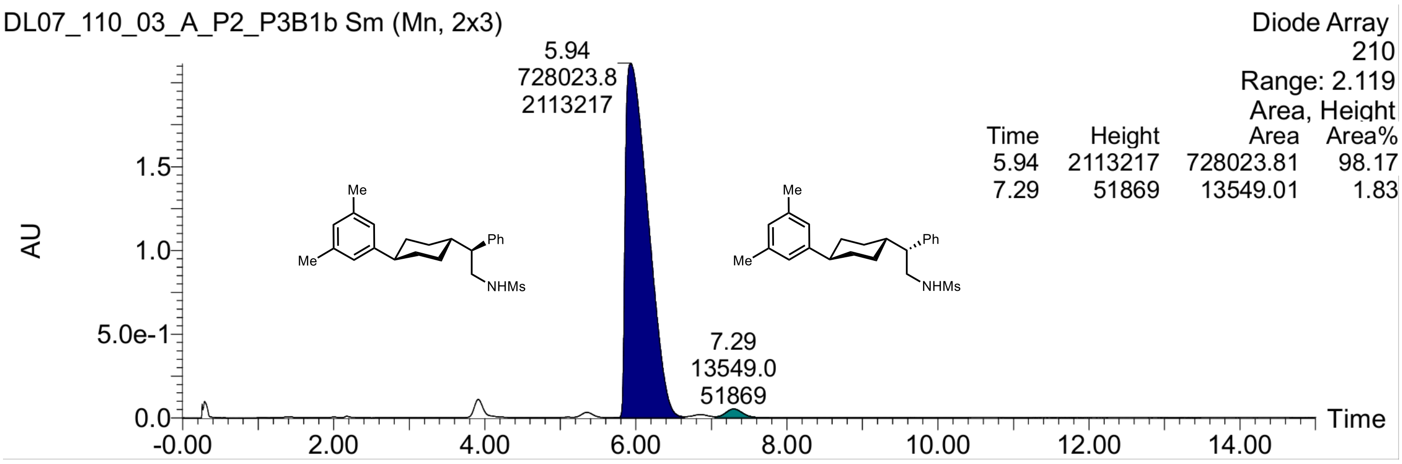

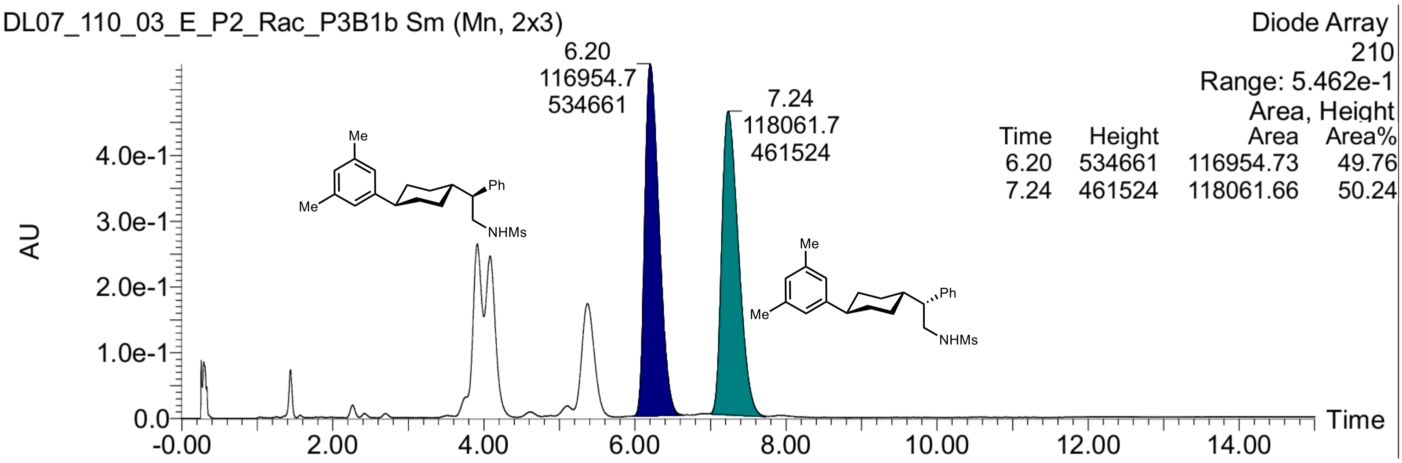

Figure S105. SFC trace of 32 (chiral – top, racemic – bottom)

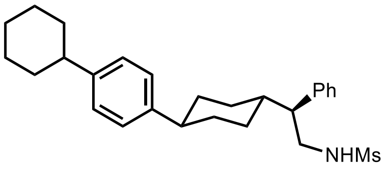

N-((S)-2-((1r,4S)-4-(4-cyclohexylphenyl)cyclohexyl)-2-phenylethyl)methanesulfonamide (33)

Prepared according to **general procedure H**, 1,4-dicyclohexylbenzene (121 mg, 0.5 mmol, 2.5 equiv), Rh_2(*S-tetra*-$CF_3C_6H_4$NTTL)$_4$ (3.0 mg, 0.0001 mmol, 0.005 equiv), and 1-(methylsulfonyl)-4-phenyl-1H-1,2,3-triazole (44.7 mg, 0.2 mmol, 1.0 equiv) were used. The crude mixture was reduced by $NaBH_4$ (18.9 mg, 0.5 mmol, 2.5 equiv) at 0 ˚C then purified by flash chromatography (gradient 0%-65% Et_2O in hexane) afforded **33** as a white solid (59.6 mg, 68%, 96% ee).

R_f (3H/2EA) = 0.40 (CAM)

$[\alpha]^{20}_D$: -29.1° (c = 0.26 g/100 ml, $CHCl_3$, 96% ee)

^{1}H NMR (800 MHz, $CDCl_3$) δ 7.36 (t, *J* = 7.1 Hz, 2H), 7.28 (t, *J* = 7.5 Hz, 1H), 7.17 (d, *J* = 7.5 Hz, 2H), 7.11 (d, *J* = 8.3 Hz, 2H), 7.08 (d, *J* = 8.2 Hz, 2H), 3.94 – 3.80 (m, 1H), 3.71 – 3.62 (m, 1H), 3.35 – 3.30 (m, 1H), 2.80 (s, 3H), 2.61 (td, *J* = 9.4, 4.1 Hz, 1H), 2.45 (t, *J* = 9.9 Hz, 1H), 2.39 (t, *J* = 12.1 Hz, 1H), 2.07 (d, *J* = 13.4 Hz, 1H), 1.97 (d, *J* = 13.3 Hz, 1H), 1.84 (dd, *J* = 29.0, 11.0 Hz, 5H), 1.73 (d, *J* = 12.2 Hz, 1H), 1.68 – 1.60 (m, 1H), 1.56 – 1.51 (m, 1H), 1.46 (q, *J* = 12.8 Hz, 1H), 1.42 – 1.31 (m, 5H), 1.26 – 1.18 (m, 2H), 1.01 (q, *J* = 12.9 Hz, 1H).

^{13}C NMR (101 MHz, CDCl$_3$) δ 145.84, 144.60, 140.68, 129.09, 128.63, 127.43, 126.84, 126.70, 52.57, 46.28, 44.22, 43.81, 40.63, 40.30, 34.61, 34.24, 34.04, 31.59, 31.33, 27.06, 26.30.

HRMS (-p APCI) calcd. [$C_{27}H_{36}O_2N^{32}S$] ($[M-H]^-$) for 438.2472 found 438.2476.

HPLC (Chiralpak ADH column, 1% i-propanol in hexane, 1.0 mLmin^{-1}, 1.0 mgmL^{-1}, 15 min, UV 230 nm) retention times of 17.0 min (major) and 23.3 min (minor), 96% ee.

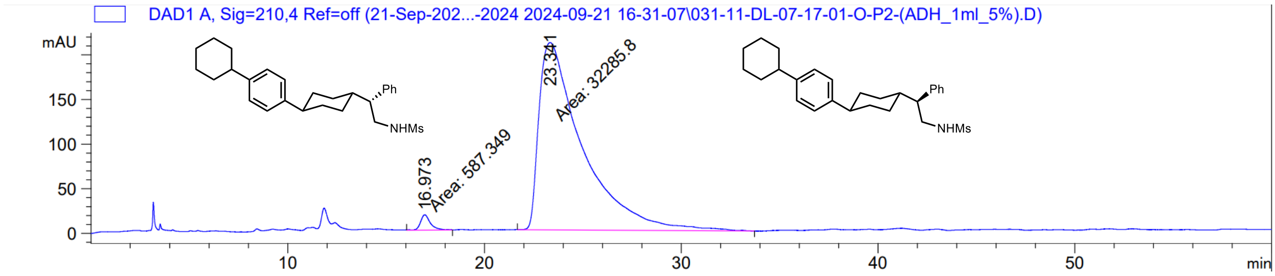

Signal 1: DAD1 A, Sig=210,4 Ref=off

Peak #	RetTime [min]	Type	Width [min]	Area [mAU*s]	Height [mAU]	Area %
1	16.973	MM	0.5722	587.34924	17.10732	1.7867
2	23.341	MM	2.5639	3.22858e4	209.87579	98.2133
Totals :				3.28732e4	226.98311	

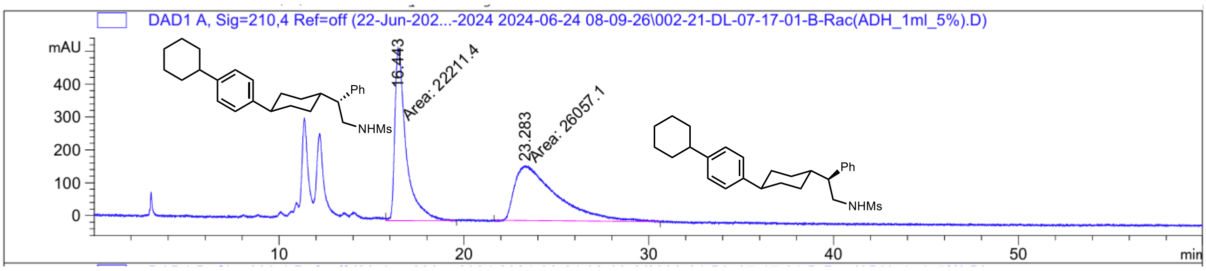

Signal 1: DAD1 A, Sig=210,4 Ref=off

Peak #	RetTime [min]	Type	Width [min]	Area [mAU*s]	Height [mAU]	Area %
1	16.443	MM	0.7007	2.22114e4	528.31763	46.0164
2	23.283	MM	2.5995	2.60571e4	167.06239	53.9836
Totals :				4.82685e4	695.38002	

Figure S106. HPLC trace of 33 (chiral – top, racemic – bottom)

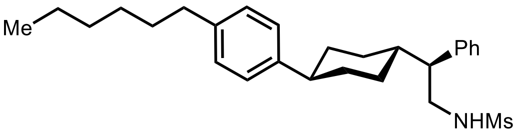

N-((S)-2-((1r,4S)-4-(4-hexylphenyl)cyclohexyl)-2-phenylethyl)methanesulfonamide (34)

Prepared according to **general procedure H**, 1-cyclohexyl-4-hexylbenzene (122 mg, 0.5 mmol, 2.5 equiv), Rh_2(*S-tetra-*$CF_3C_6H_4$NTTL)$_4$ (3.0 mg, 0.0001 mmol, 0.005 equiv), and 1-(methylsulfonyl)-4-phenyl-1H-1,2,3-triazole (44.7 mg, 0.2 mmol, 1.0 equiv) were used. The crude mixture was reduced by $NaBH_4$ (18.9 mg, 0.5 mmol, 2.5 equiv) at 0 ˚C then purified by flash chromatography (gradient 0%-65% Et_2O in hexane) afforded **34** as a white solid (62.2 mg, 70%, 95% ee).

R$_f$ (3H/2EA) = 0.40 (CAM)

[α]$^{20}_{D}$: -22.3° (c = 0.16 g/100 ml, $CHCl_3$, 95% ee)

^{1}H NMR (800 MHz, $CDCl_3$) δ 7.36 (t, J = 7.5 Hz, 2H), 7.30 – 7.26 (m, 1H), 7.18 (d, J = 7.6 Hz, 2H), 7.08 (dd, J = 8.4, 6.1 Hz, 4H), 3.94 (d, J = 8.4 Hz, 1H), 3.71 – 3.59 (m, 1H), 3.33 (dd, J = 13.0, 10.3 Hz, 1H), 2.79 (s, 3H), 2.65 – 2.59 (m, 1H), 2.56 (t, J = 7.9 Hz, 2H), 2.40 (td, J = 12.0, 3.1 Hz, 1H), 2.08 (d, J = 13.0 Hz, 1H), 1.97 (dd, J = 13.1, 3.1 Hz, 1H), 1.82 (dd, J = 13.1, 3.0 Hz, 1H), 1.64 (dtd, J = 11.8, 8.4, 3.8 Hz, 1H), 1.59 (p, J = 7.3 Hz, 2H), 1.55 (d, J = 13.1 Hz, 1H), 1.47 (q, J = 12.8 Hz, 1H), 1.39 – 1.25 (m, 7H), 1.21 (q, J = 12.7 Hz, 1H), 1.02 (q, J = 12.8 Hz, 1H), 0.90 – 0.86 (m, 3H).

^{13}C NMR (201 MHz, $CDCl_3$) δ 144.5, 140.7, 140.7, 129.1, 128.6, 128.4, 128.4, 127.4, 126.7, 52.6, 46.3, 43.8, 40.6, 40.3, 35.7, 34.3, 34.1, 31.9, 31.6, 31.6, 31.3, 29.2, 22.7, 14.2.

HRMS (+p APCI) calcd. [$C_{27}H_{40}O_2N^{32}S$] ($[M+H]^+$) for 442.2774 found 442.2772.
SFC (OJ3, 10% (50% methanol in isopropanol with 0.2% Formic Acid) in CO_2, 2.5 mL/min, 1.0 mg/ml, UV 210 nm) retention times of 7.05 min (major) and 8.88 min (minor) 95% ee.

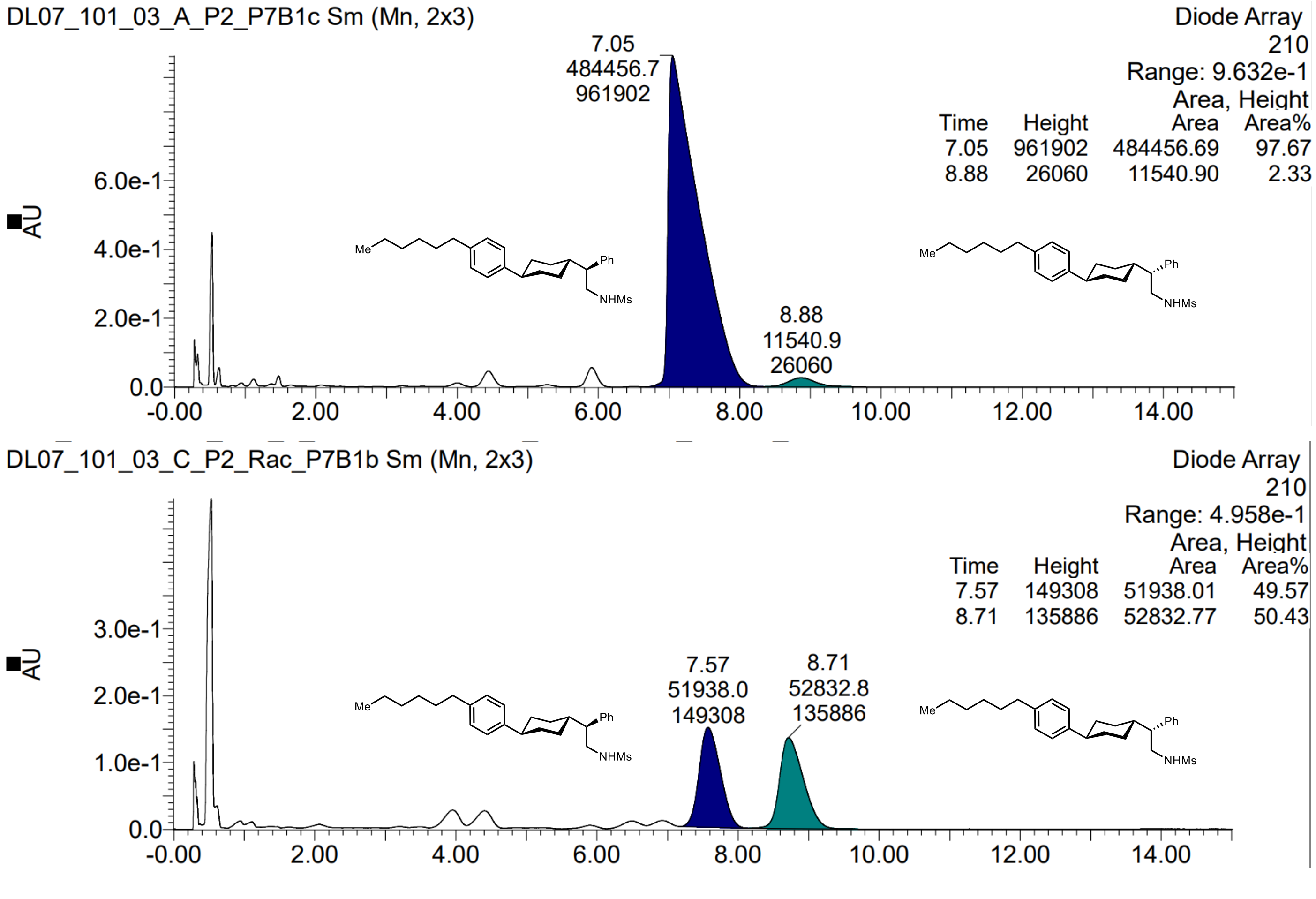

Figure S107. SFC trace of 34 (chiral – top, racemic – bottom)

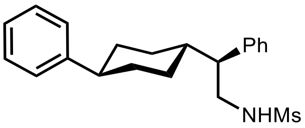

N-((S)-2-phenyl-2-((1r,4S)-4-phenylcyclohexyl)ethyl)methanesulfonamide (35)

Prepared according to **general procedure H**, cyclohexylbenzene (80.1 mg, 0.5 mmol, 2.5 equiv), Rh_2(*S-tetra*-$CF_3C_6H_4$NTTL)$_4$ (3.0 mg, 0.0001 mmol, 0.005 equiv), and 1-(methylsulfonyl)-4-phenyl-1H-1,2,3-triazole (44.7 mg, 0.2

mmol, 1.0 equiv) were used. The reaction was run for 36 hours. The crude mixture was reduced by $NaBH_4$ (18.9 mg, 0.5 mmol, 2.5 equiv) at 0 ˚C then purified by flash chromatography (gradient 0%-65% Et_2O in hexane) afforded **35** as a white solid (53.2 mg, 74% yield, 96% ee). *Note. Triazole was not fully consumed after 24 hours. The cyclohexylbenzene was obtained from Aldrich and was purified by distillation before use – The major impurities were benzene.*

R_f (3H/2EA) = 0.70 (CAM)

[α]$^{20}_D$: -26.1° (c = 0.30 g/100 ml, $CHCl_3$, 96% ee)

^{1}H NMR (800 MHz, $CDCl_3$) δ 7.36 (t, *J* = 7.9 Hz, 2H), 7.30 – 7.26 (m, 3H), 7.19 – 7.14 (m, 5H), 3.93 – 3.87 (m, 1H), 3.67 (ddd, *J* = 12.7, 8.6, 4.2 Hz, 1H), 3.36 – 3.30 (m, 1H), 2.80 (s, 3H), 2.62 (ddt, *J* = 12.9, 6.0, 3.1 Hz, 1H), 2.42 (t, *J* = 10.7 Hz, 1H), 2.08 (dd, *J* = 13.0, 3.0 Hz, 1H), 1.97 (dd, *J* = 13.2, 3.0 Hz, 1H), 1.82 (dd, *J* = 13.1, 3.0 Hz, 1H), 1.69 – 1.62 (m, 1H), 1.58 – 1.54 (m, 1H), 1.49 (q, *J* = 12.9 Hz, 1H), 1.37 (q, *J* = 12.8 Hz, 1H), 1.22 (q, *J* = 12.8 Hz, 1H), 1.02 (q, *J* = 12.8 Hz, 1H).

^{13}C NMR (201 MHz, $CDCl_3$) δ 147.3, 140.7, 129.1, 128.6, 128.5, 127.5, 126.9, 126.1, 52.6, 46.3, 44.3, 40.6, 40.4, 34.2, 34.0, 31.6, 31.3.

HRMS (+p APCI) calcd. [$C_{21}H_{28}O_2N^{32}S$] ([M+H]$^+$) for 358.1846 found 358.1838.
SFC (CEL2, 10% (50% methanol in isopropanol with 0.2% Formic Acid) in CO_2, 2.5 mL/min, 1.0 mg/ml, UV 210 nm) retention times of 7.07 min (major) and 8.36 min (minor) 96% ee.

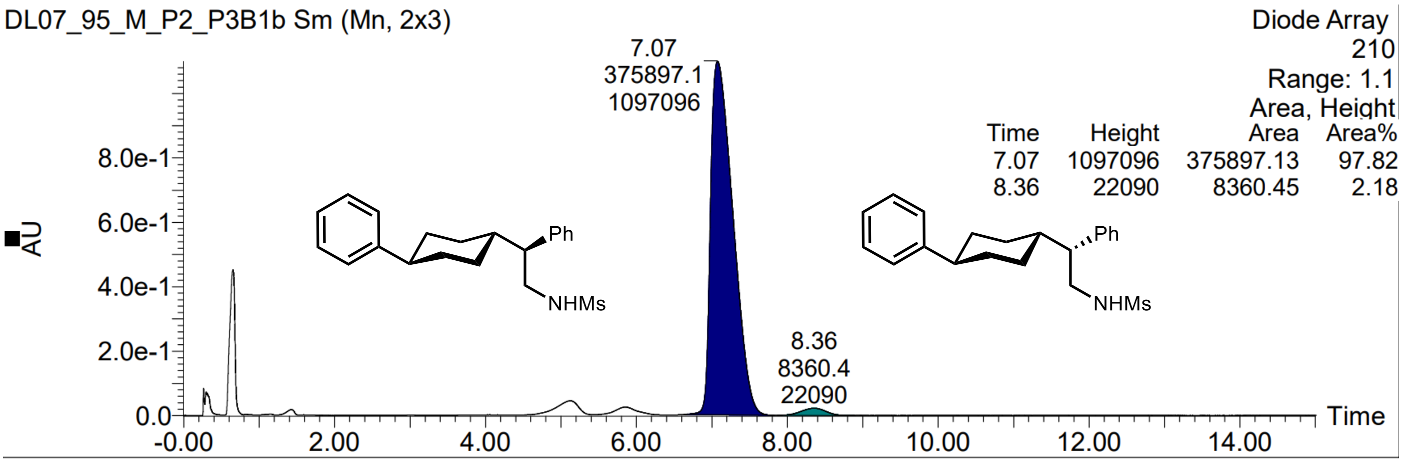

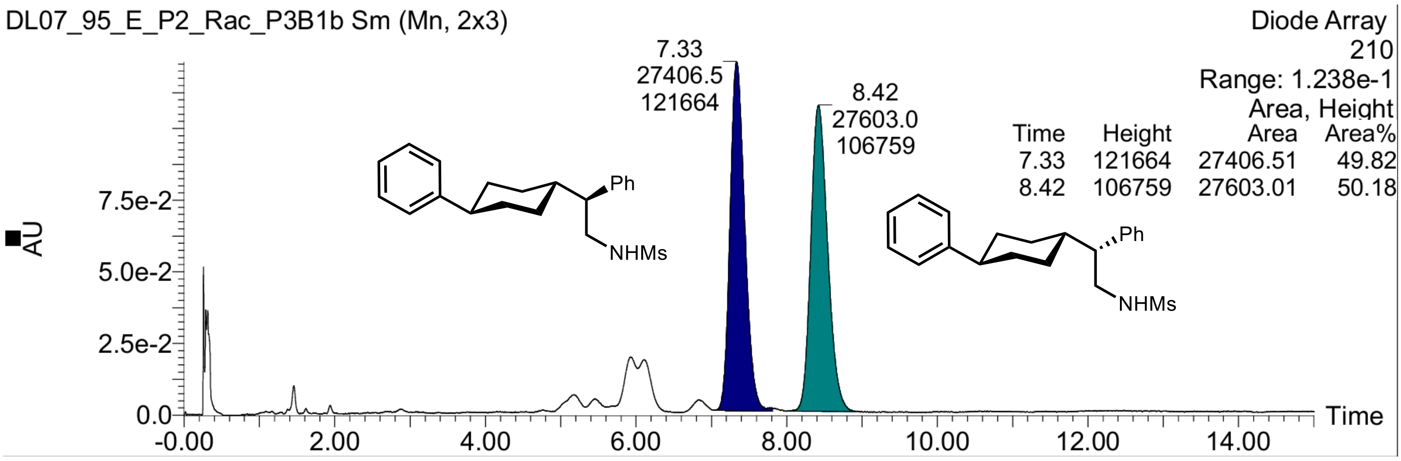

Figure S108. SFC trace of 35 (chiral – top, racemic – bottom)

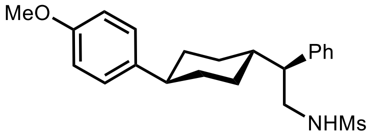

N-((S)-2-((1r,4S)-4-(4-methoxyphenyl)cyclohexyl)-2-phenylethyl)methanesulfonamide (36)

Prepared according to general procedures **H**, 1-cyclohexyl-4-methoxybenzene (95.1 mg, 0.5 mmol, 2.5 equiv), Rh_2(*S*-*tetra*-$CF_3C_6H_4$NTTL)$_4$ (3.0 mg, 0.0001 mmol, 0.005 equiv), and 1-(methylsulfonyl)-4-phenyl-1H-1,2,3-triazole (44.7 mg, 0.2 mmol, 1.0 equiv) were used. The reaction was run for 36 hours. The crude mixture was reduced by $NaBH_4$ (18.9 mg, 0.5 mmol, 2.5 equiv) at 0 ˚C then purified by flash chromatography (gradient 0%-65% Et_2O in hexane) afforded **36** as a white solid (50.9 mg, 66% yield, 96% ee). *Note. Triazole was not fully consumed after 24 hours.*

R_f (3H/2EA) = 0.40 (CAM)

$[\alpha]^{20}_D$: -29.5° (c = 0.28 g/100 ml, $CHCl_3$, 96% ee)

^{1}H NMR (800 MHz, $CDCl_3$) δ 7.36 (t, *J* = 6.8 Hz, 2H), 7.30 – 7.26 (m, 1H), 7.17 (d, *J* = 7.5 Hz, 2H), 7.08 (d, *J* = 6.8 Hz, 2H), 6.82 (d, *J* = 6.8 Hz, 2H), 3.90 (d, *J* = 5.4 Hz, 1H), 3.77 (s, 3H), 3.66 (ddd, *J* = 12.1, 7.8, 4.2 Hz, 1H), 3.37 – 3.28 (m, 1H), 2.80 (s, 3H), 2.64 – 2.58 (m, 1H), 2.37 (t, *J* = 12.2 Hz, 1H), 2.07 (d, *J* = 12.4 Hz, 1H), 1.95 (d, *J* = 13.3 Hz, 1H), 1.80 (d, *J* = 13.3 Hz, 1H), 1.68 – 1.59 (m, 1H), 1.55 – 1.51 (m, 1H), 1.44 (q, *J* = 12.2 Hz, 1H), 1.32 (q, *J* = 12.2 Hz, 1H), 1.20 (q, *J* = 12.3 Hz, 1H), 1.01 (q, *J* = 14.0 Hz, 1H).

^{13}C NMR (201 MHz, $CDCl_3$) δ 157.9, 140.7, 139.5, 129.1, 128.6, 127.7, 127.4, 113.8, 55.4, 52.6, 46.3, 43.4, 40.6, 40.3, 34.4, 34.3, 31.6, 31.3.

HRMS (-p APCI) calcd. [$C_{22}H_{28}O_3N^{32}S$] ($[M-H]^-$) for 386.1795 found 386.1798.
SFC (OJ3, 15% (50% methanol in isopropanol with 0.2% Formic Acid) in CO_2, 2.5 mL/min, 1.0 mg/ml, UV 210 nm) retention times of 4.72 min (major) and 8.14 min (minor) 96% ee.

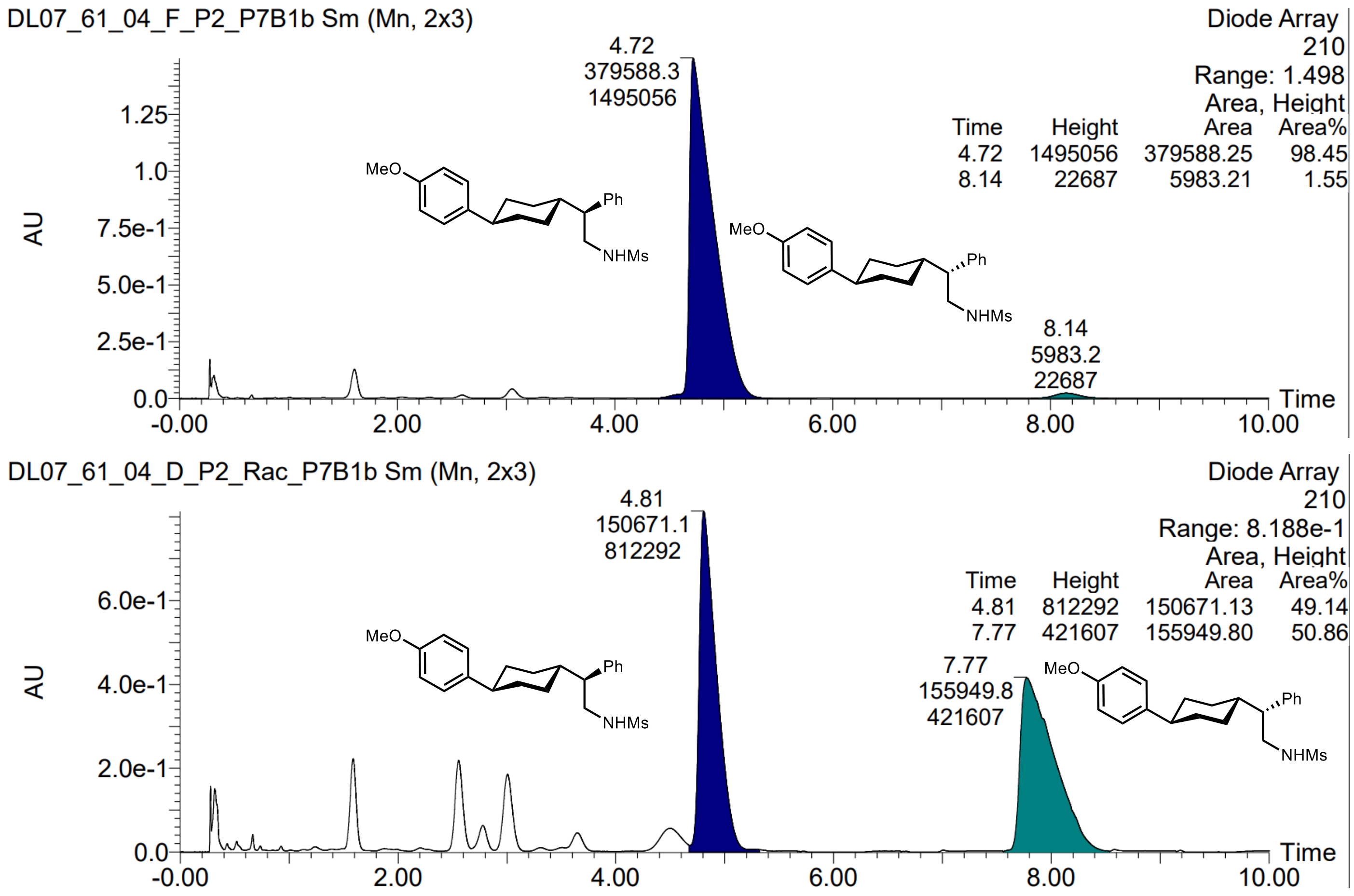

Figure S109. SFC trace of 36 (chiral – top, racemic – bottom)

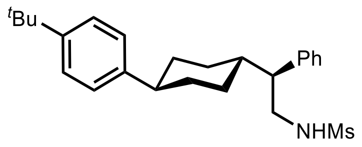

N-((S)-2-((1r,4S)-4-(4-(tert-butyl)phenyl)cyclohexyl)-2-phenylethyl)methanesulfonamide (37)

Prepared according to **general procedure H**, 1-(tert-butyl)-4-cyclohexylbenzene (108 mg, 0.5 mmol, 2.5 equiv), Rh_2(*S*-*tetra*-$CF_3C_6H_4$NTTL)$_4$ (3.0 mg, 0.0001 mmol, 0.005 equiv), and 1-(methylsulfonyl)-4-phenyl-1H-1,2,3-triazole (44.7 mg, 0.2 mmol, 1.0 equiv) were used. The reaction was run for 24 hours. The crude mixture was reduced by $NaBH_4$ (18.9 mg, 0.5 mmol, 2.5 equiv) at 0 ˚C then purified by flash chromatography (gradient 0%-65% Et_2O in hexane) afforded **37** as a white solid (46.0 mg, 56% yield, 95% ee).

R_f (3H/2EA) = 0.40 (CAM)

$[\alpha]^{20}_D$: -20.8° (c = 0.33 g/100 ml, $CHCl_3$, 95% ee)

^{1}H NMR (800 MHz, $CDCl_3$) δ 7.38 – 7.34 (m, 2H), 7.32 – 7.27 (m, 3H), 7.18 (d, *J* = 7.5 Hz, 2H), 7.10 (d, *J* = 8.6 Hz, 2H), 3.98 – 3.88 (m, 1H), 3.67 (ddt, *J* = 12.6, 8.1, 3.6 Hz, 1H), 3.33 (t, *J* = 11.7 Hz, 1H), 2.80 (s, 3H), 2.67 – 2.58 (m, 1H), 2.40 (t, *J* = 12.3 Hz, 1H), 2.08 (d, *J* = 9.6 Hz, 1H), 1.97 (d, *J* = 11.8 Hz, 1H), 1.83 (d, *J* = 11.3 Hz, 1H), 1.70 – 1.60 (m, 1H), 1.55 (d, *J* = 13.4 Hz, 1H), 1.47 (q, *J* = 12.9 Hz, 1H), 1.36 (q, *J* = 11.0 Hz, 1H), 1.30 (s, 9H), 1.21 (q, *J* = 12.7 Hz, 1H), 1.02 (q, *J* = 12.8 Hz, 1H).

^{13}C NMR (201 MHz, $CDCl_3$) δ 148.8, 144.2, 140.7, 129.1, 128.6, 127.4, 126.5, 125.3, 52.6, 46.3, 43.7, 40.7, 40.3, 34.5, 34.2, 34.0, 31.6, 31.5, 31.3.

HRMS (-p APCI) calcd. [$C_{25}H_{34}O_2N^{32}S$] ($[M-H]^-$) for 412.2316 found 412.2311.

SFC (OJ3, 10% (50% methanol in isopropanol with 0.2% Formic Acid) in CO_2, 2.5 mL/min, 1.0 mg/ml, UV 210 nm) retention times of 5.54 min (major) and 7.70 min (minor) 95% ee.

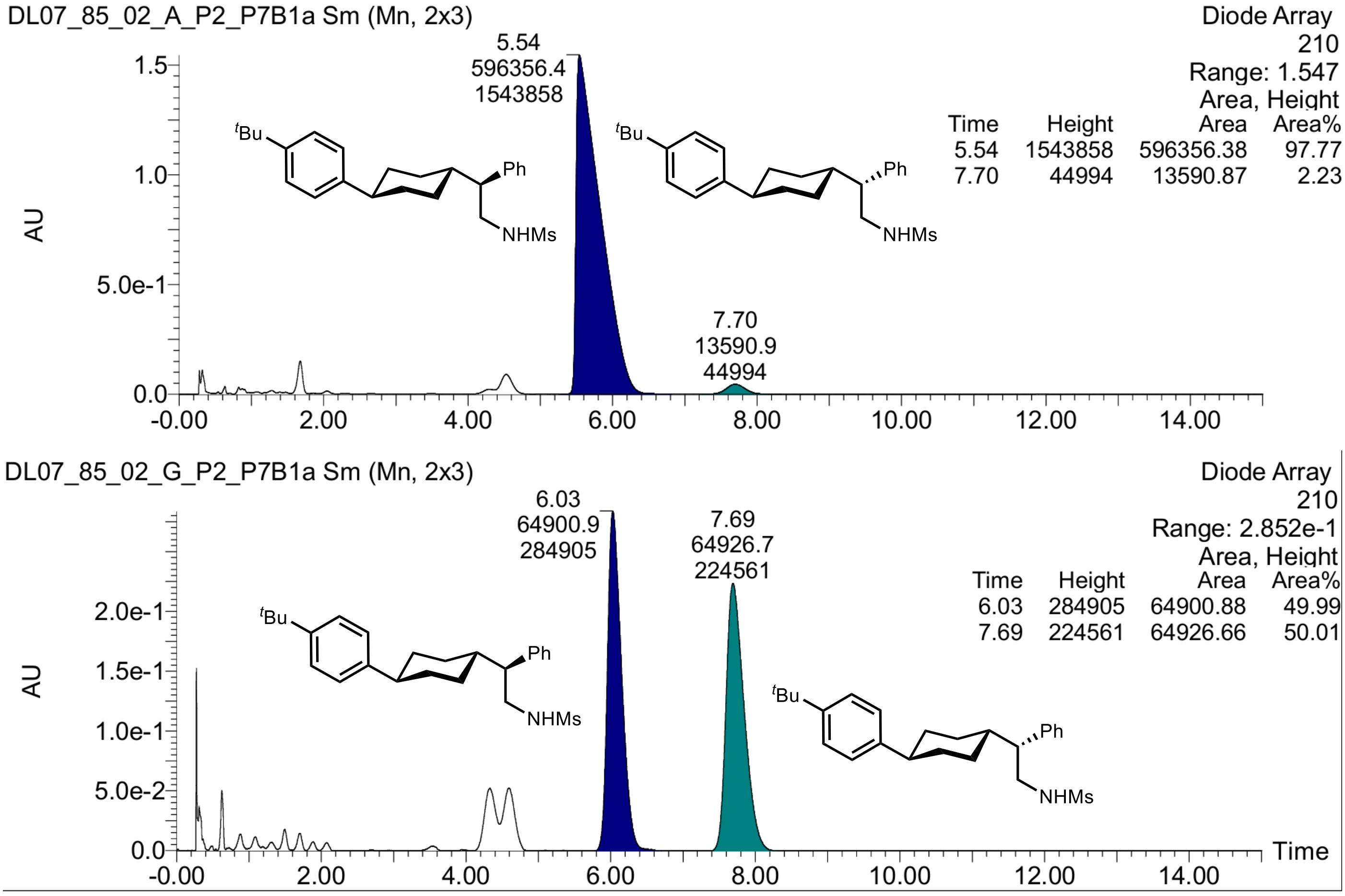

Figure S110. SFC trace of 37 (chiral – top, racemic – bottom)

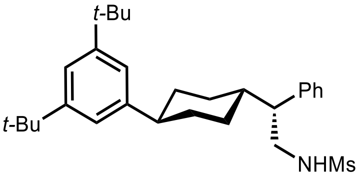

N-((S)-2-((1r,4S)-4-(3,5-di-tert-butylphenyl)cyclohexyl)-2-phenylethyl)methanesulfonamide (38)

Prepared according to **general procedure H**, 1,3-di-tert-butyl-5-cyclohexylbenzene (136 mg, 0.5 mmol, 2.5 equiv), Rh_2(*S-tetra*-$CF_3C_6H_4$NTTL)$_4$ (3.0 mg, 0.0001 mmol, 0.005 equiv), and 1-(methylsulfonyl)-4-phenyl-1H-1,2,3-triazole (44.7 mg, 0.2 mmol, 1.0 equiv) were used. The crude mixture was reduced by $NaBH_4$ (18.9 mg, 0.5 mmol, 2.5 equiv) at 0 °C then purified by flash chromatography (gradient 0%-65% Et_2O in hexane) afforded **38** as a white solid (46.9 mg, 50% yield, 93% ee). Note. Isolated as a mixture of 6:1 C4 to C3 products.

R_f (3H/2EA) = 0.70 (CAM)

$[\alpha]^{20}_D$: -19.8° (c = 0.13 g/100 ml, $CHCl_3$, 93% ee)

^{1}H NMR (800 MHz, $CDCl_3$) δ 7.60 (t, J = 7.5 Hz, 2H), 7.52 (t, J = 7.6 Hz, 1H), 7.51 – 7.48 (m, 2H), 7.45 – 7.41 (m, 2H), 4.15 (d, J = 8.5 Hz, 1H), 3.95 – 3.87 (m, 1H), 3.62 – 3.53 (m, 1H), 3.05 (s, 3H), 2.90 – 2.83 (m, 1H), 2.67 (t, J = 11.1 Hz, 1H), 2.33 (dd, J = 13.2, 2.9 Hz, 1H), 2.23 (dd, J = 13.3, 3.3 Hz, 1H), 2.09 (dd, J = 13.6, 3.2 Hz, 1H), 1.92 (ddd,

J = 11.9, 7.5, 4.5 Hz, 1H), 1.82 – 1.79 (m, 1H), 1.77 – 1.73 (m, 1H), 1.70 – 1.62 (m, 1H), 1.56 (s, 18H), 1.49 – 1.43 (m, 1H), 1.27 (q, J = 12.9 Hz, 1H).

^{13}C NMR (201 MHz, CDCl$_3$) δ 150.6, 146.3, 140.7, 129.1, 128.6, 127.4, 121.1, 120.2, 52.6, 46.3, 44.9, 40.7, 40.3, 35.0, 34.4, 34.2, 31.7, 31.7, 31.6, 31.4.

HRMS (-p APCI) calcd. [$C_{29}H_{42}O_2N^{32}S$] ([M-H]$^-$) for 468.2942 found 468.2946.
SFC (OJ3, 7% (50% methanol in isopropanol with 0.2% Formic Acid) in CO_2, 2.5 mL/min, 1.0 mg/ml, UV 210 nm) retention times of 3.10 min (major) and 3.97 min (minor) 93% ee.

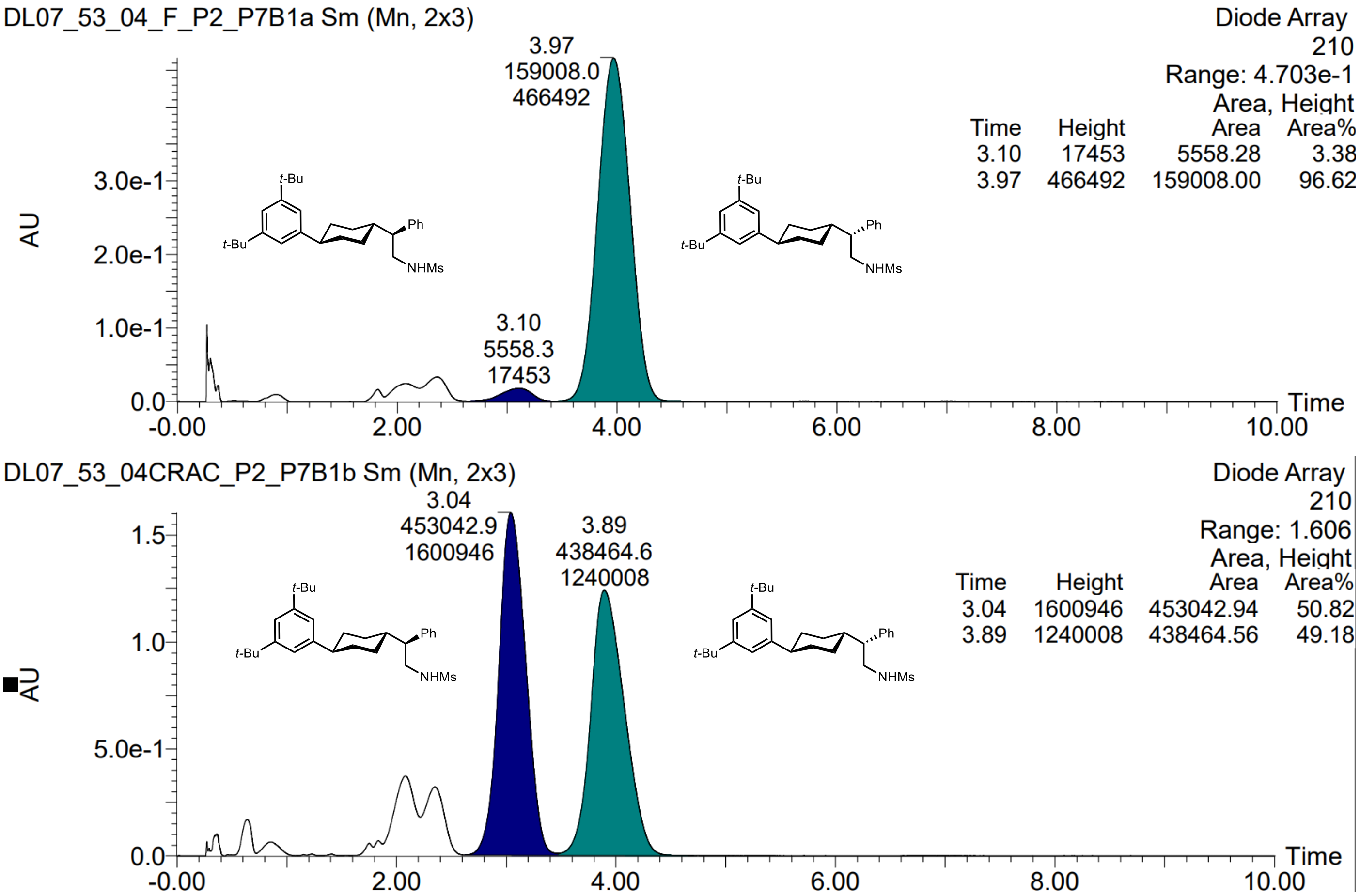

Figure S111. SFC trace of 38 (chiral – top, racemic – bottom).

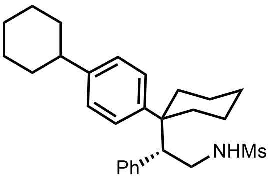

(R)-N-(2-(1-(4-cyclohexylphenyl)cyclohexyl)-2-phenylethyl)methanesulfonamide (39)

Prepared according to **general procedure H**, 1,4-dicyclohexylbenzene (121 mg, 0.5 mmol, 2.5 equiv), Rh$_2$(*S*-*di*-3,5-di-*t*BuC$_6$H$_4$NTTL)$_4$ (3.0 mg, 0.0001 mmol, 0.005 equiv), and 1-(methylsulfonyl)-4-phenyl-1H-1,2,3-triazole (44.7 mg, 0.2 mmol, 1.0 equiv) were used. The crude mixture reduced by NaBH$_4$ (18.9 mg, 0.5 mmol, 2.5 equiv) at 0 ˚C then purified by flash chromatography (gradient 0%-65% Et$_2$O in hexane) afforded **39** as a clear oil (21.0 mg, 24%, 97% ee).

R$_f$ (3H/2EA) = 0.45 (CAM)

[α]$^{20}_D$: -26.0° (c = 0.49 g/100 ml, CHCl$_3$, 97% ee)

^{1}H NMR (400 MHz, CDCl$_3$) δ 7.25 – 7.16 (m, 3H), 7.16 – 7.08 (m, 2H), 7.08 – 6.93 (m, 2H), 6.77 (bs, 2H), 3.67 – 3.48 (m, 2H), 3.17 (td, J = 11.7, 2.5 Hz, 1H), 2.81 (dd, J = 11.6, 3.6 Hz, 1H), 2.65 (s, 3H), 2.55 – 2.37 (m, 2H), 2.07 – 1.97 (m, 1H), 1.97 – 1.80 (m, 4H), 1.80 – 1.69 (m, 1H), 1.54 – 1.32 (m, 9H), 1.32 – 1.15 (m, 4H). *Hinder rotation was observed.*

^{13}C NMR (101 MHz, CDCl$_3$) δ 145.98, 138.69, 138.08, 128.45, 128.07, 127.42, 126.51, 58.87, 44.62, 44.03, 43.51, 40.12, 36.02, 34.61, 34.60, 33.54, 27.04, 26.51, 26.31, 22.33, 21.99. (*Missing 1 carbon)*

HRMS (-p APCI) calcd.[$C_{27}H_{36}O_2N^{32}S$] ([M-H]$^-$) for 438.2472 found 438.2474.

HPLC (Chiralpak ADH column, 2% i-propanol in hexane, 1.0 mLmin^{-1}, 1.0 mgmL^{-1}, 60 min, UV 230 nm) retention times of 16.4 min (major) and 19.1 min (minor), 97% ee.

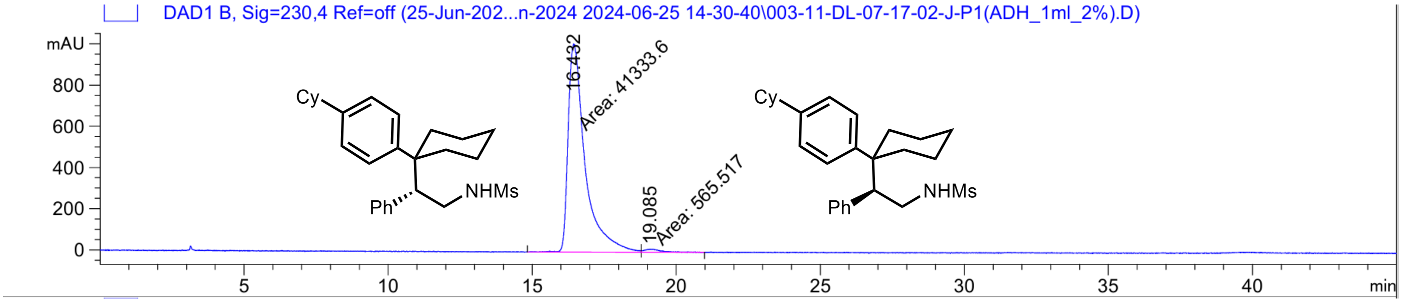

Signal 2: DAD1 B, Sig=230,4 Ref=off

Peak #	RetTime [min]	Type	Width [min]	Area [mAU*s]	Height [mAU]	Area %
1	16.432	MF	0.6817	4.13336e4	1010.55304	98.6503
2	19.085	FM	0.6241	565.51666	15.10152	1.3497
Totals :				4.18992e4	1025.65456	

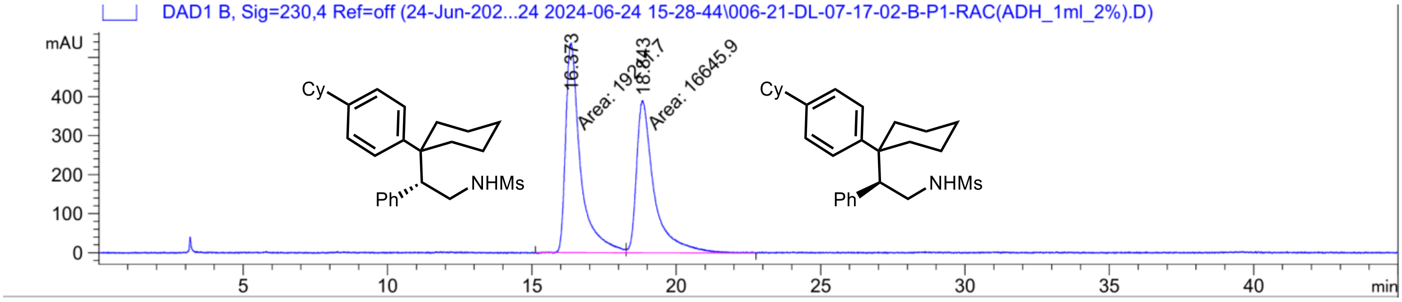

Signal 2: DAD1 B, Sig=230,4 Ref=off

Peak #	RetTime [min]	Type	Width [min]	Area [mAU*s]	Height [mAU]	Area %
1	16.373	MF	0.5961	1.92117e4	537.11713	53.5777
2	18.843	FM	0.7112	1.66459e4	390.09491	46.4223
Totals :				3.58576e4	927.21204	

Figure S112. HPLC trace of 39 (chiral – top, racemic – bottom)

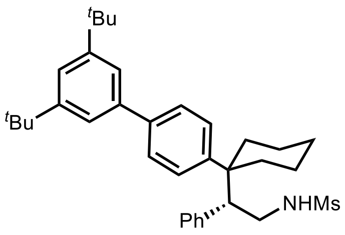

(R)-N-(2-(1-(3',5'-di-tert-butyl-[1,1'-biphenyl]-4-yl)cyclohexyl)-2-phenylethyl)methanesulfonamide (40)

Prepared according to **general procedure H**, 3,5-di-tert-butyl-4'-cyclohexyl-1,1'-biphenyl (174 mg, 0.5 mmol, 2.5 equiv), Rh_2(*S*-*di*-3,5-di-*t*BuC_6H_4NTTL)$_4$ (3.0 mg, 0.0001 mmol, 0.005 equiv), and 1-(methylsulfonyl)-4-phenyl-1H-1,2,3-triazole (44.7 mg, 0.2 mmol, 1.0 equiv) were used. The crude mixture reduced by $NaBH_4$ (18.9 mg, 0.5 mmol, 2.5 equiv) at 0 ˚C then purified by flash chromatography (gradient 0%-65% Et_2O in hexane) afforded **40** as a clear oil (63.3 mg, 58%, 98% ee).

R$_f$ (3H/2EA) = 0.45 (CAM)

$[\alpha]^{20}_D$: -40.4° (c = 2.16 g/100 ml, $CHCl_3$, 98% ee)

^{1}H NMR (600 MHz, $CDCl_3$) δ 7.56 (d, *J* = 8.6 Hz, 2H), 7.47 (d, *J* = 1.8 Hz, 2H), 7.45 (t, *J* = 1.8 Hz, 1H), 7.26 – 7.20 (m, 3H), 7.16 (bs, 2H), 6.82 (bs, 2H), 3.73 – 3.61 (m, 2H), 3.22 (dd, *J* = 11.7, 9.4 Hz, 1H), 2.89 (dd, *J* = 11.8, 3.6 Hz, 1H), 2.69 (s, 3H), 2.56 (d, *J* = 13.3 Hz, 1H), 2.05 (d, *J* = 13.4 Hz, 1H), 1.58 – 1.50 (m, 5H), 1.40 (s, 18H), 1.32 – 1.20 (m, 3H). *Hinder rotation was observed*

^{13}C NMR (151 MHz, $CDCl_3$) δ 151.3, 140.0, 137.8, 129.1, 128.2, 127.6, 127.0, 121.6, 121.6, 58.8, 44.8, 43.5, 40.3, 36.2, 35.1, 35.1, 34.2, 31.8, 31.7, 26.5, 22.4, 22.0. (Missing 1 aromatic carbon)

HRMS (-p APCI) calcd. [$C_{35}H_{46}O_2N^{32}S$] ([M-H]$^-$) for 544.3255 found 544.3258.
HPLC (Chiralpak ADH column, 1% i-propanol in hexane, 1.0 mLmin^{-1}, 1.0 mgmL^{-1}, 60 min, UV 230 nm) retention times of 17.8 min (major) and 21.2 min (minor), 98% ee.

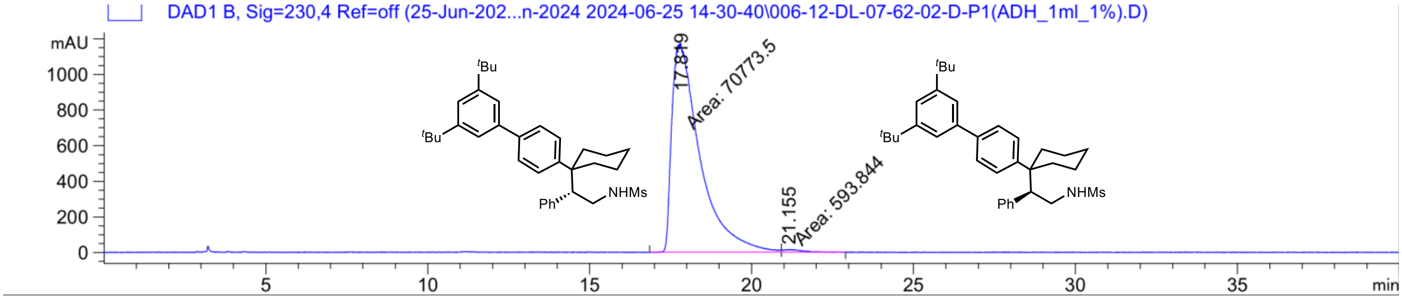


```
Signal 2: DAD1 B, Sig=230,4 Ref=off

Peak RetTime Type  Width     Area       Height      Area
  #    [min]        [min]    [mAU*s]     [mAU]        %
----|-------|----|-------|----------|----------|--------|
   1  17.819 MF    1.0038 7.07735e4  1175.13025  99.1679
   2  21.155 FM    0.6724  593.84381   14.71931   0.8321

Totals :                  7.13674e4  1189.84956
```

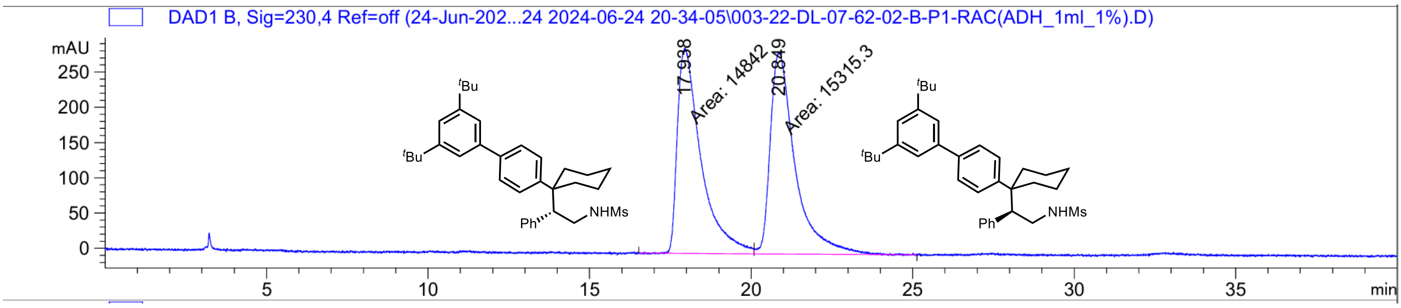

Signal 2: DAD1 B, Sig=230,4 Ref=off

Peak #	RetTime [min]	Type	Width [min]	Area [mAU*s]	Height [mAU]	Area %
1	17.938	MF	0.8503	1.48420e4	290.91125	49.2153
2	20.849	FM	0.8906	1.53153e4	286.62662	50.7847
Totals :				3.01573e4	577.53787	

Figure S113. HPLC trace of 40 (chiral – top, racemic – bottom)

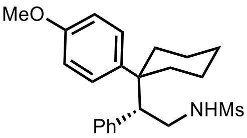

(R)-N-(2-(1-(4-methoxyphenyl)cyclohexyl)-2-phenylethyl)methanesulfonamide (41)

Prepared according to **general procedure H**, 1-cyclohexyl-4-methoxybenzene (95.1 mg, 0.5 mmol, 2.5 equiv), Rh_2(*S*-*di*-3,5-di-*t*BuC_6H_4NTTL)$_4$ (3.0 mg, 0.0001 mmol, 0.005 equiv), and 1-(methylsulfonyl)-4-phenyl-1H-1,2,3-triazole (44.7 mg, 0.2 mmol, 1.0 equiv) were used. The crude mixture reduced by $NaBH_4$ (18.9 mg, 0.5 mmol, 2.5 equiv) at 0 ˚C then purified by flash chromatography (gradient 0%-65% Et_2O in hexane) afforded **41** as a clear oil (58.1 mg, 75%, 99% ee).

R$_f$ (3H/2EA) = 0.45 (CAM)

$[\alpha]^{20}_D$: -50.2° (c = 0.19 g/100 ml, $CHCl_3$, 99% ee)

^{1}H NMR (400 MHz, $CDCl_3$) δ 7.25 – 7.16 (m, 3H), 7.10 – 6.91 (m, 2H), 6.90 – 6.27 (m, 4H), 3.82 (s, 3H), 3.69 – 3.50 (m, 2H), 3.15 (dt, *J* = 11.9, 8.4 Hz, 1H), 2.83 (dd, *J* = 10.8, 2.7 Hz, 1H), 2.68 (s, 3H), 2.48 (d, *J* = 12.8 Hz, 1H), 1.95 (d, *J* = 14.1 Hz, 1H), 1.55 – 1.37 (m, 5H), 1.33 – 1.09 (m, 3H). *Hinder rotation was observed*

^{13}C NMR (101 MHz, $CDCl_3$) δ 157.9, 137.9, 133.0, 129.8, 128.1, 127.5, 113.4, 58.8, 55.3, 44.3, 43.5, 40.2, 36.2, 34.3, 26.5, 22.3, 21.9. (Missing 1 aromatic carbon)

HRMS (-p APCI) calcd. [$C_{22}H_{28}O_3N^{32}S$] ($[M-H]^-$) for 386.1795 found 386.1787.

SFC (OJ3, 10% (50% methanol in isopropanol with 0.2% Formic Acid) in CO_2, 2.5 mL/min, 1.0 mg/ml, UV 210 nm) retention times of 2.83 min (major) and 9.25 min (minor) 99% ee.

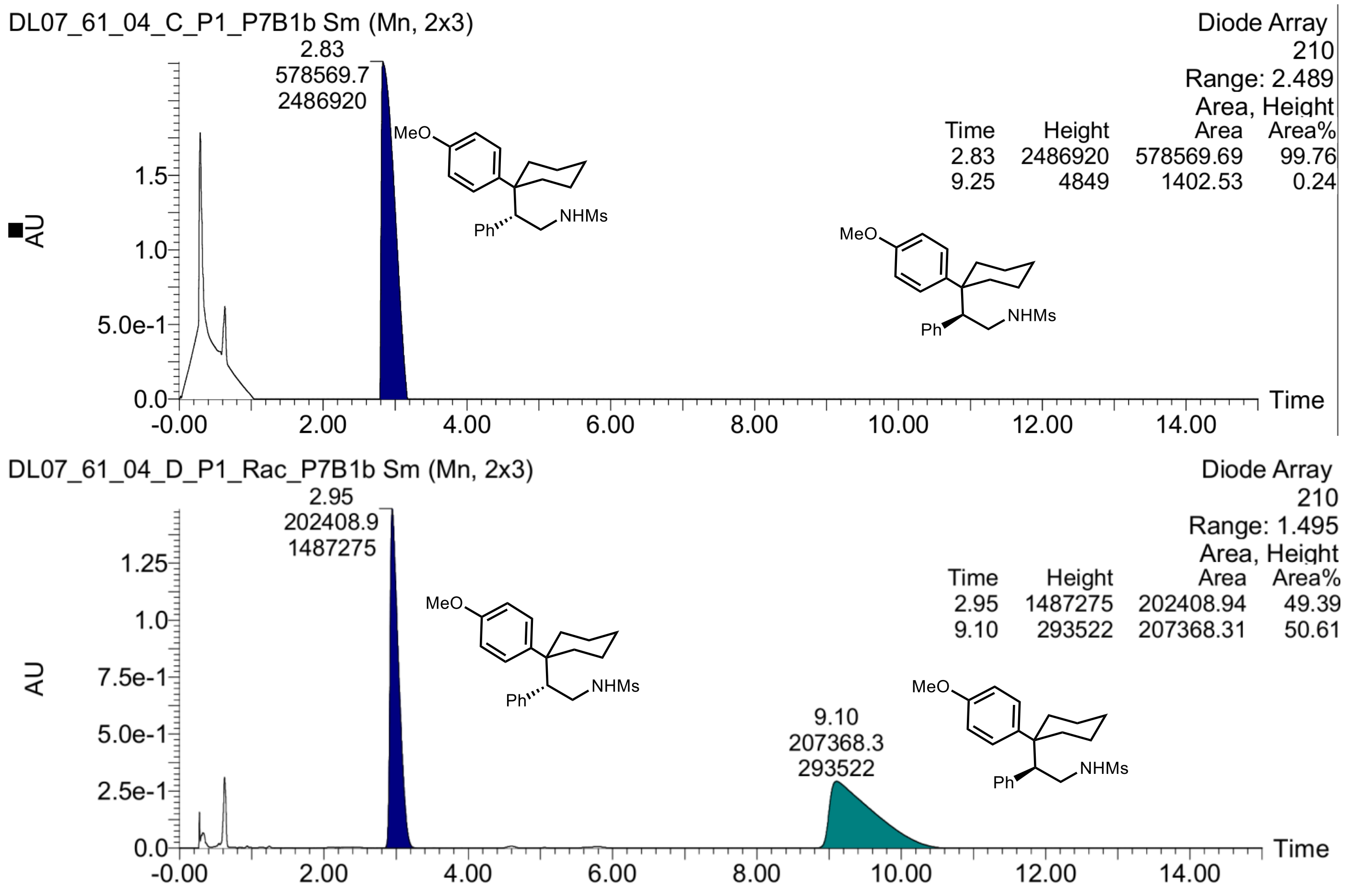

Figure S114. SFC trace of 41 (chiral – top, racemic – bottom)

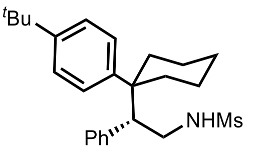

(R)-N-(2-(1-(4-(tert-butyl)phenyl)cyclohexyl)-2-phenylethyl)methanesulfonamide (42)

Prepared according to **general procedure H**, 1-(tert-butyl)-4-cyclohexylbenzene (108 mg, 0.5 mmol, 2.5 equiv), Rh_2(*S*-*di*-3,5-di-*t*BuC_6H_4NTTL)$_4$ (3.0 mg, 0.0001 mmol, 0.005 equiv), and 1-(methylsulfonyl)-4-phenyl-1H-1,2,3-triazole (44.7 mg, 0.2 mmol, 1.0 equiv) were used. The crude mixture reduced by $NaBH_4$ (18.9 mg, 0.5 mmol, 2.5 equiv) at 0 ˚C then purified by flash chromatography (gradient 0%-65% Et_2O in hexane) afforded **42** as a clear oil (44.7 mg, 54%, 97% ee).

$\mathbf{R_f}$ (3H/2EA) = 0.45 (CAM)

$\mathbf{[\alpha]^{20}_D}$**:** -32.2° (c = 0.70 g/100 ml, $CHCl_3$, 97% ee)

^{1}H NMR (800 MHz, $CDCl_3$) δ 7.30 (dd, *J* = 8.0, 2.8 Hz, 2H), 7.21 (p, *J* = 6.6 Hz, 3H), 7.03 (d, *J* = 8.1 Hz, 2H), 6.79 (bs, 2H), 3.56 (d, *J* = 10.4 Hz, 2H), 3.19 (t, *J* = 10.2 Hz, 1H), 2.82 (d, *J* = 11.4 Hz, 1H), 2.65 (s, 3H), 2.45 (d, *J* = 13.4 Hz, 1H), 2.02 (d, *J* = 13.5 Hz, 1H), 1.55 – 1.44 (m, 5H), 1.36 – 1.26 (m, 10H), 1.19 (q, *J* = 14.0 Hz, 2H). *Hinder rotation was observed.*

^{13}C NMR (201 MHz, $CDCl_3$) δ 149.1, 138.4, 138.1, 128.2, 128.1, 127.5, 125.0, 58.9, 44.6, 43.6, 40.2, 36.0, 34.5, 33.5, 31.5, 26.5, 22.4, 22.0. (Missing 1 aromatic carbon)

HRMS (-p APCI) calcd. [$C_{25}H_{34}O_2N^{32}S$] ($[M-H]^-$) for 412.2316 found 412.2312.

SFC (OJ3, 5% (50% methanol in isopropanol with 0.2% Formic Acid) in CO_2, 2.5 mL/min, 1.0 mg/ml, UV 210 nm) retention times of 1.94 min (major) and 2.88 min (minor) 97% ee.

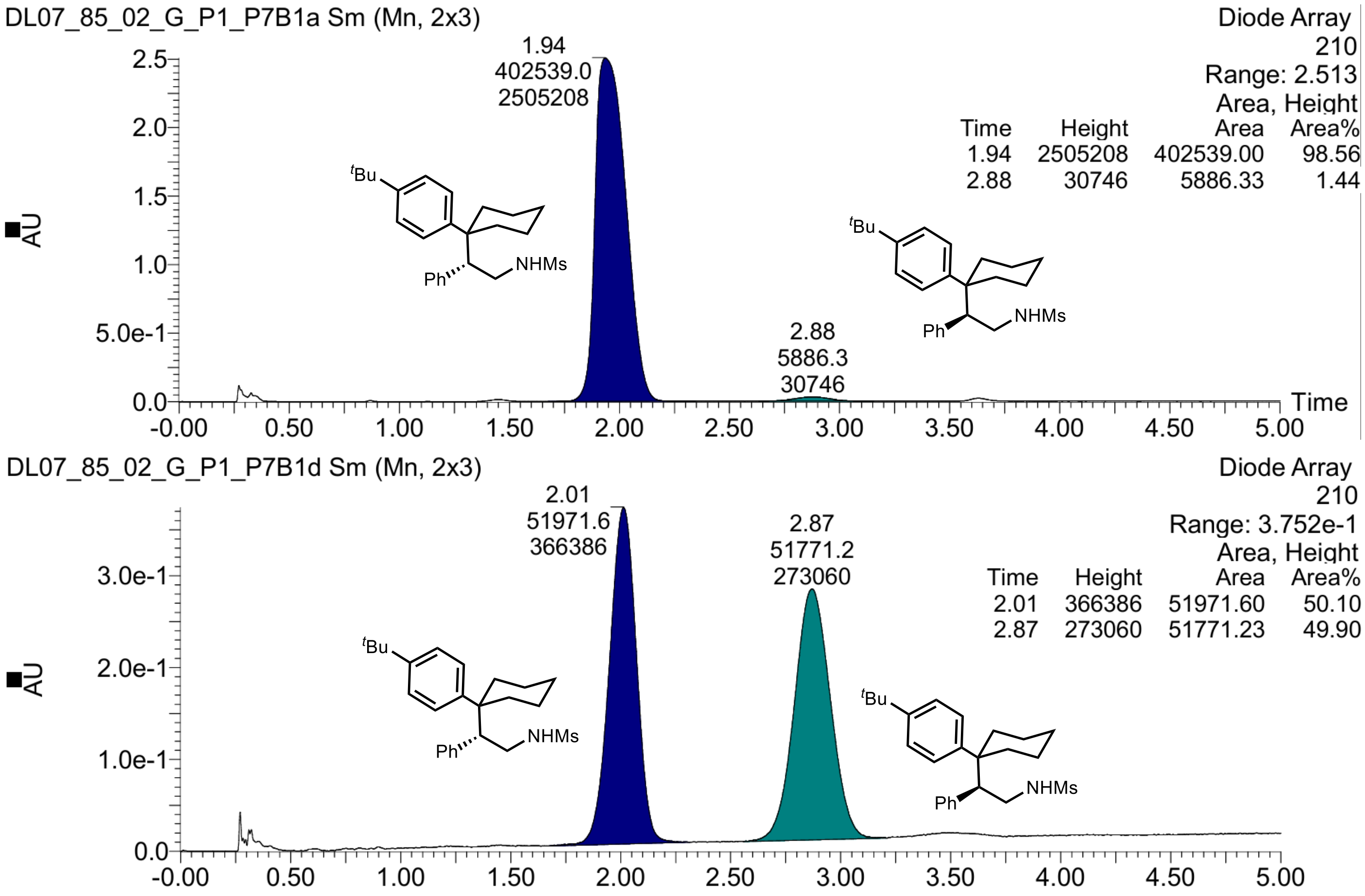

Figure S115. SFC trace of 42 (chiral – top, racemic – bottom)

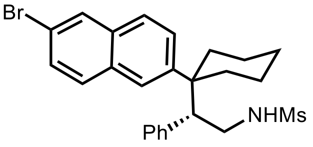

(R)-N-(2-(1-(6-bromonaphthalen-2-yl)cyclohexyl)-2-phenylethyl)methanesulfonamide (43)

Prepared according to **general procedure H**, 2-bromo-6-cyclohexylnaphthalene (145 mg, 0.5 mmol, 2.5 equiv), Rh_2(*S-di*-3,5-di-*t*BuC_6H_4NTTL$)_4$ (3.0 mg, 0.0001 mmol, 0.005 equiv), and 1-(methylsulfonyl)-4-phenyl-1H-1,2,3-triazole (44.7 mg, 0.2 mmol, 1.0 equiv) were used. The crude mixture reduced by $NaBH_4$ (18.9 mg, 0.5 mmol, 2.5 equiv) at 0 ˚C then purified by flash chromatography (gradient 0%-65% Et_2O in hexane) afforded **43** as a clear oil (63.8 mg, 66%, 91% ee).

R$_f$ (3H/2EA) = 0.50 (CAM)

$[\alpha]^{20}_D$: -52.7° (c = 1.0 g/100 ml, $CHCl_3$, 91% ee)

^{1}H NMR (600 MHz, CDCl$_3$) δ 7.99 (d, *J* = 1.9 Hz, 1H), 7.69 (d, *J* = 8.7 Hz, 1H), 7.60 (s, 1H), 7.54 (dd, *J* = 8.7, 1.9 Hz, 1H), 7.50 – 7.41 (m, 1H), 7.40 – 7.27 (m, 1H), 7.25 – 7.22 (m, 1H), 7.21 – 7.12 (m, 2H), 7.03 – 6.09 (bs, 2H), 3.76 – 3.55 (m, 2H), 3.17 (td, *J* = 12.0, 2.5 Hz, 1H), 2.95 (dd, *J* = 11.9, 3.6 Hz, 1H), 2.71 – 2.59 (m, 4H), 2.15 (d, *J* = 13.8 Hz, 1H), 1.63 – 1.53 (m, 4H), 1.52 – 1.45 (m, 1H), 1.29 – 1.17 (m, 3H). *Hinder rotation was observed*

^{13}C NMR (151 MHz, CDCl$_3$) δ 139.5, 137.5, 133.0, 131.7, 129.8, 129.5, 129.5, 128.2, 128.2, 128.2, 127.7, 126.7, 119.9, 58.7, 45.2, 43.3, 40.4, 35.9, 34.4, 26.5, 22.4, 22.1.

HRMS (-p APCI) calcd. [$C_{25}H_{27}O_2N^{79}Br^{32}S$] ([M-H]$^-$) for 484.0951 found 484.0949.

SFC (CEL2, 20% (50% methanol in isopropanol with 0.2% Formic Acid) in CO_2, 2.5 mL/min, 1.0 mg/ml, UV 210 nm) retention times of 4.28 min (minor) and 5.05 min (major) 91% ee.

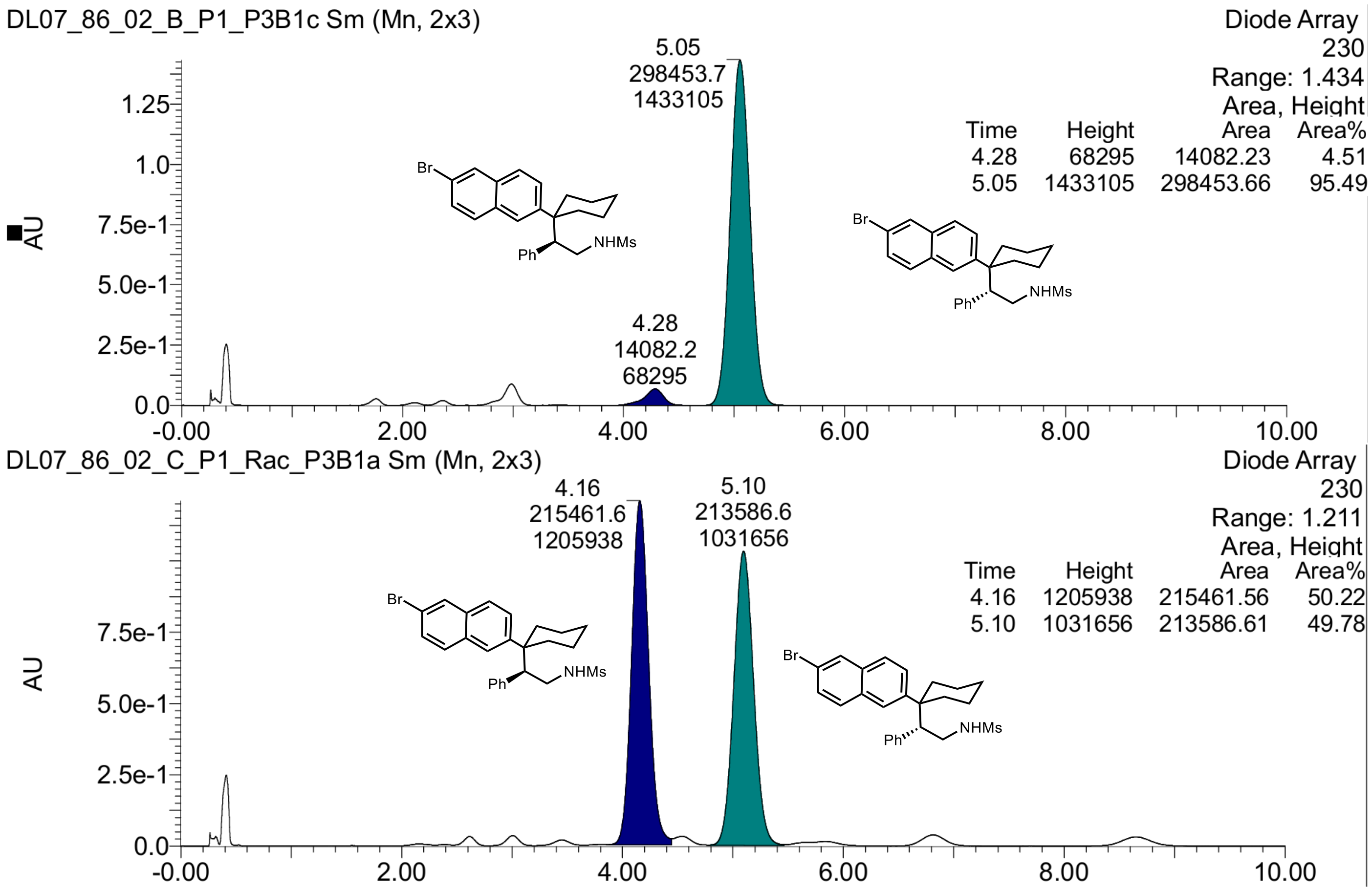

Figure S116. SFC trace of 43 (chiral – top, racemic – bottom)

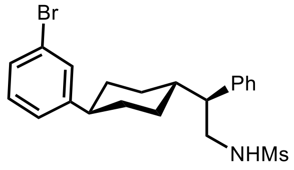

N-((S)-2-((1r,4S)-4-(3-bromophenyl)cyclohexyl)-2-phenylethyl)methanesulfonamide (44)

Prepared according to **general procedure H**, 1-bromo-3-cyclohexylbenzene (120 mg, 0.5 mmol, 2.5 equiv), Rh_2(*S-tetra*-$MeOC_6H_4$NTTL)$_4$ (3.0 mg, 0.0001 mmol, 0.005 equiv), and 1-(methylsulfonyl)-4-phenyl-1H-1,2,3-triazole (44.7 mg, 0.2 mmol, 1.0 equiv) were used. The crude mixture reduced by $NaBH_4$ (18.9 mg, 0.5 mmol, 2.5 equiv) at 0 °C then purified by flash chromatography (SiO_2, gradient 0%-65% Et_2O in hexane) afforded **44** as a white solid (60.4 mg, 69% yield, 95% ee).

R$_f$ (3H/2EA) = 0.50 (CAM, UV 210 nm)

[α]$^{20}_D$: -25.5° (c = 0.51 g/100 ml, $CHCl_3$, 95% ee)

^{1}H NMR (800 MHz, $CDCl_3$) δ 7.36 (t, *J* = 7.5 Hz, 2H), 7.32 – 7.27 (m, 3H), 7.17 (d, *J* = 5.8 Hz, 2H), 7.13 (t, *J* = 7.4 Hz, 1H), 7.08 (d, *J* = 7.9 Hz, 1H), 3.89 (d, *J* = 7.3 Hz, 1H), 3.70 – 3.63 (m, 1H), 3.32 (t, *J* = 11.7 Hz, 1H), 2.80 (s, 3H), 2.61 (ddd, *J* = 10.8, 7.5, 4.3 Hz, 1H), 2.40 (td, *J* = 12.2, 3.2 Hz, 1H), 2.09 (dd, *J* = 13.1, 3.1 Hz, 1H), 1.95 (dd, *J* = 13.2, 3.1 Hz, 1H), 1.80 (dd, *J* = 13.1, 3.1 Hz, 1H), 1.64 (dt, *J* = 11.7, 3.3 Hz, 1H), 1.54 (td, *J* = 8.1, 3.1 Hz, 1H), 1.45 (q, *J* = 11.8 Hz, 1H), 1.33 (q, *J* = 12.8 Hz, 1H), 1.20 (q, *J* = 12.7 Hz, 1H), 1.00 (q, *J* = 12.8 Hz, 1H).

^{13}C NMR (201 MHz, $CDCl_3$) δ 149.62, 140.55, 130.06, 130.04, 129.20, 129.14, 128.61, 127.51, 125.62, 122.59, 52.52, 46.26, 44.05, 40.51, 40.37, 34.01, 33.88, 31.41, 31.15.

HRMS (+p APCI) calcd. for [$C_{21}H_{27}O_2N^{79}Br^{32}S$] ([M+H]$^+$) 436.0940 found 436.0948.
SFC (CEL2, 15% (50% methanol in isopropanol with 0.2% Formic Acid) in CO_2, 2.5 mL/min, 1.0 mg/ml, UV 210 nm) retention times of 4.91 min (major) and 5.70 min (minor) 95% ee.

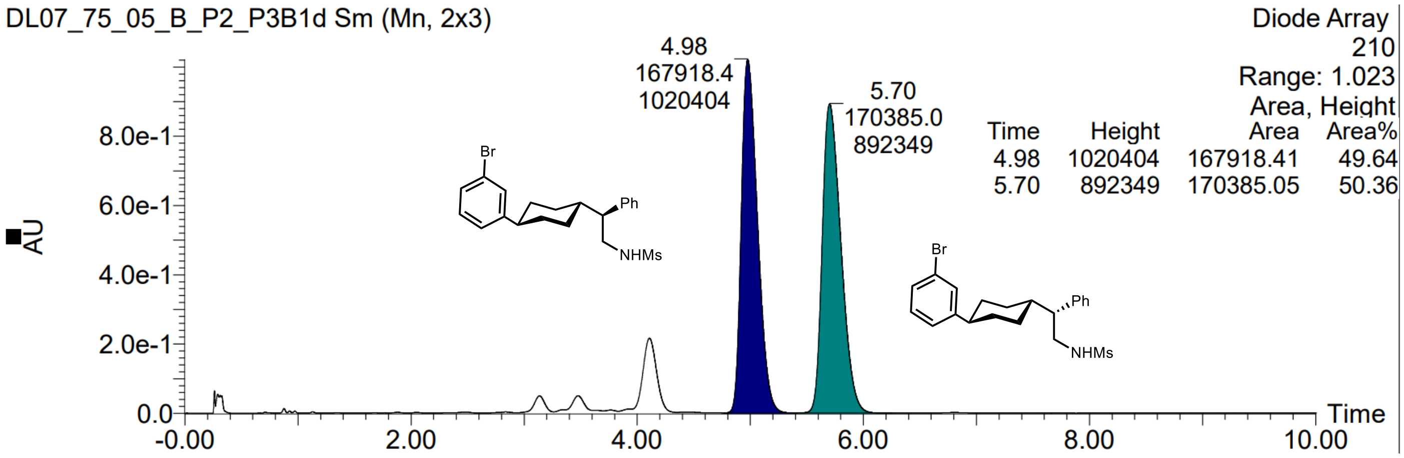

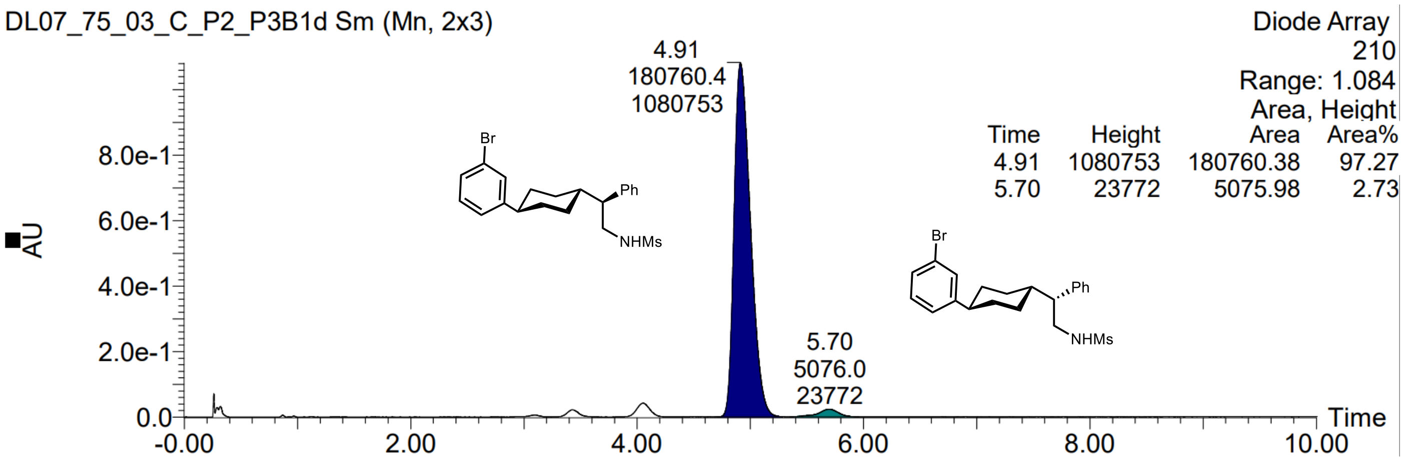

Figure S117. SFC trace of 44 (chiral top, racemic bottom)

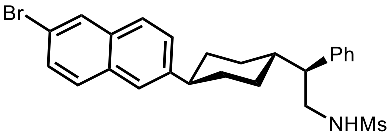

N-((S)-2-((1r,4S)-4-(6-bromonaphthalen-2-yl)cyclohexyl)-2-phenylethyl)methanesulfonamide (45)

Prepared according to **general procedure H**, 2-bromo-6-cyclohexylnaphthalene (145 mg, 0.5 mmol, 2.5 equiv), Rh_2(*S*-*tetra*-$MeOC_6H_4NTTL)_4$ (3.0 mg, 0.0001 mmol, 0.005 equiv), and 1-(methylsulfonyl)-4-phenyl-1H-1,2,3-triazole (44.7 mg, 0.2 mmol, 1.0 equiv) were used. The crude mixture reduced by $NaBH_4$ (18.9 mg, 0.5 mmol, 2.5 equiv) at 0 °C then

purified by flash chromatography (gradient 0%-65% Et_2O in hexane) afforded **45** as a white solid (75.3 mg, 77%, 95% ee).

R_f (3H/2EA) = 0.50 (CAM)

$[\alpha]^{20}_D$: -40.9° (c = 0.18 g/100 ml, $CHCl_3$, 95% ee)

^{1}H NMR (600 MHz, $CDCl_3$) δ 7.94 (s, 1H), 7.66 (d, *J* = 8.5 Hz, 1H), 7.63 (d, *J* = 8.7 Hz, 1H), 7.55 (s, 1H), 7.50 (d, *J* = 8.7 Hz, 1H), 7.39 – 7.36 (m, 2H), 7.34 (d, *J* = 8.3 Hz, 1H), 7.29 (t, *J* = 6.7 Hz, 1H), 7.19 (d, *J* = 8.1 Hz, 2H), 3.91 (dd, *J* = 9.0, 3.6 Hz, 1H), 3.68 (ddd, *J* = 13.1, 8.7, 4.5 Hz, 1H), 3.39 – 3.30 (m, 1H), 2.81 (d, *J* = 1.5 Hz, 3H), 2.64 (td, *J* = 9.6, 4.8 Hz, 1H), 2.58 (t, *J* = 12.1 Hz, 1H), 2.13 (d, *J* = 12.7 Hz, 1H), 2.05 (d, *J* = 13.1 Hz, 1H), 1.89 (d, *J* = 13.2 Hz, 1H), 1.69 (td, *J* = 11.9, 8.6 Hz, 1H), 1.58 (td, *J* = 12.6, 3.5 Hz, 2H), 1.45 (q, *J* = 12.9 Hz, 1H), 1.27 (q, *J* = 13.2 Hz, 1H), 1.06 (q, *J* = 12.8 Hz, 1H).

^{13}C NMR (151 MHz, $CDCl_3$) δ 145.3, 140.6, 133.3, 132.1, 129.7, 129.4, 129.3, 129.1, 128.6, 127.5, 127.2, 127.1, 124.6, 119.1, 52.6, 46.3, 44.3, 40.6, 40.4, 34.0, 33.9, 31.5, 31.3.

HRMS (+p APCI) calcd. for $[C_{25}H_{28}O_2N^{79}Br^{32}S]$ ($[M]^+$) 485.1019 found 485.1027.
SFC (CEL2, 25% (50% methanol in isopropanol with 0.2% Formic Acid) in CO_2, 2.5 mL/min, 1.0 mg/ml, UV 230 nm) retention times of 3.93 (major) and 4.93 min (minor) 95% ee.

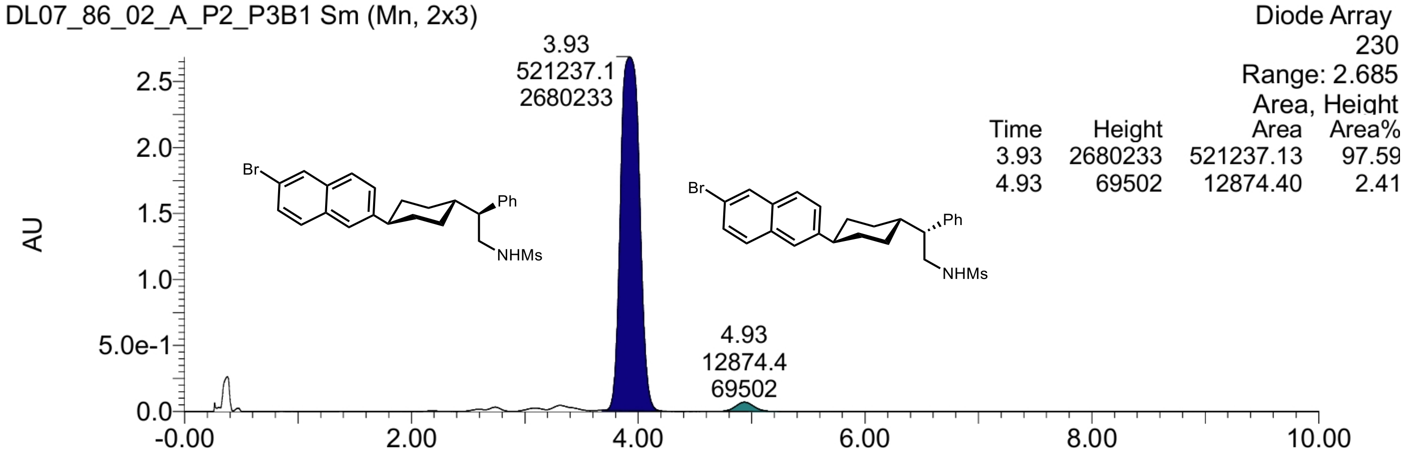

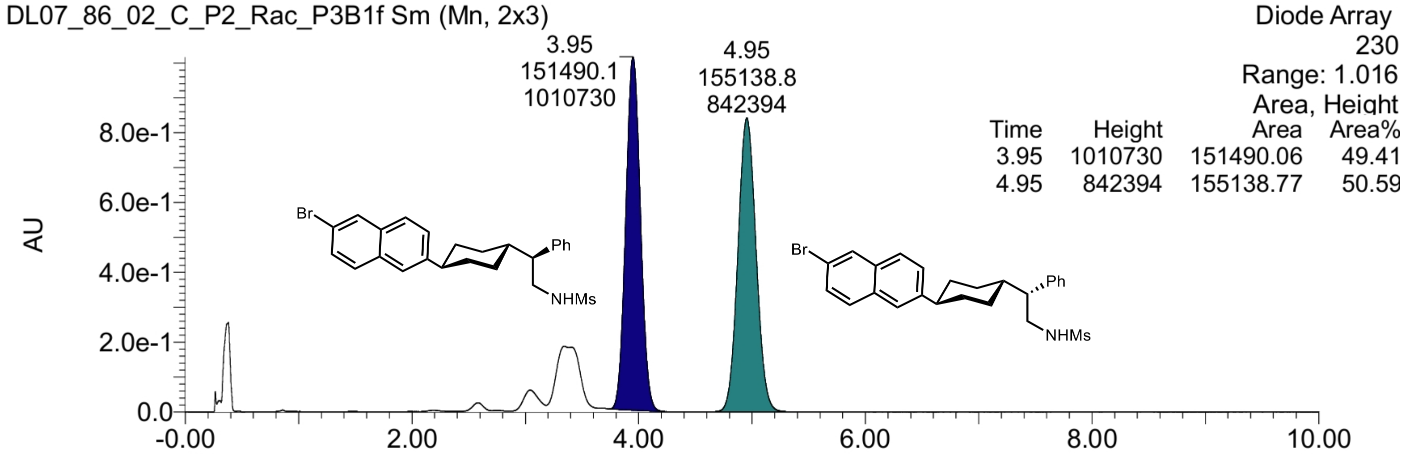

Figure S118. SFC trace of 45 (chiral top, racemic bottom)

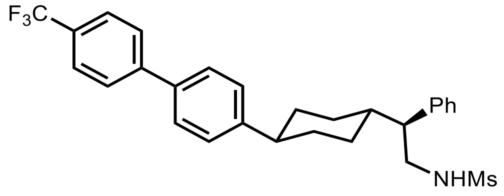

N-((S)-2-phenyl-2-((1r,4S)-4-(4'-(trifluoromethyl)-[1,1'-biphenyl]-4-yl)cyclohexyl)ethyl)methanesulfonamide (46)

Prepared according to **general procedure H**, 4-cyclohexyl-4'-(trifluoromethyl)-1,1'-biphenyl (152 mg, 0.5 mmol, 2.5 equiv), Rh_2(*S*-*tetra*-$MeOC_6H_4NTTL)_4$ (3.0 mg, 0.0001 mmol, 0.005 equiv), and 1-(methylsulfonyl)-4-phenyl-1H-1,2,3-triazole (44.7 mg, 0.2 mmol, 1.0 equiv) were used. The crude mixture reduced by $NaBH_4$ (18.9 mg, 0.5 mmol, 2.5 equiv) at 0 ˚C then purified by flash chromatography (gradient 0%-65% Et_2O in hexane) afforded **46** as a white solid (70.6 mg, 71% yield, 96% ee).

R$_f$ (3H/2EA) = 0.40 (UV 254 nm, CAM)

$[\alpha]^{20}_D$: -28.4° (c = 0.37 g/100 ml, $CHCl_3$, 96% ee)

^{1}H NMR (800 MHz, $CDCl_3$) δ 7.69 – 7.64 (m, 4H), 7.51 (d, *J* = 8.2 Hz, 2H), 7.37 (t, *J* = 7.1 Hz, 2H), 7.32 – 7.26 (m, 3H), 7.19 (d, *J* = 7.5 Hz, 2H), 3.91 (dt, *J* = 5.3, 2.9 Hz, 1H), 3.71 – 3.65 (m, 1H), 3.37 – 3.32 (m, 1H), 2.81 (s, 3H), 2.64 (qd, *J* = 7.0, 3.0 Hz, 1H), 2.49 (td, *J* = 11.2, 5.1 Hz, 1H), 2.12 (dq, *J* = 13.0, 3.1 Hz, 1H), 2.01 (dq, *J* = 13.2, 3.0 Hz, 1H), 1.86 (dq, *J* = 12.7, 3.0 Hz, 1H), 1.68 (tdd, *J* = 11.7, 9.5, 5.0 Hz, 1H), 1.61 – 1.56 (m, 1H), 1.53 (q, *J* = 13.1 Hz, 1H), 1.41 (q, *J* = 14.2 Hz, 1H), 1.28 – 1.21 (m, 1H), 1.08 – 1.01 (m, 1H).

^{13}C NMR (201 MHz, $CDCl_3$) δ 147.5, 144.7, 140.6, 137.6, 129.2 (q, *J* = 24.9 Hz), 129.1, 128.6, 127.5, 127.5, 127.4, 127.3, 125.8 (q, *J* = 3.7 Hz), 124.5 (q, *J* = 272.0 Hz), 52.6, 46.3, 44.0, 40.6, 40.4, 34.2, 34.0, 31.5, 31.2.

^{19}F NMR (753 MHz, $CDCl_3$) δ -62.4.

HRMS (+p APCI) calcd. for $[C_{28}H_{31}O_2NF_3{}^{32}S]$ ($[M+H]^+$) 502.2022 found 502.2020.
SFC (OJ3, 15% (50% methanol in isopropanol with 0.2% Formic Acid) in CO_2, 2.5 mL/min, 1.0 mg/ml, UV 254 nm) retention times of 5.38 min (major) and 7.36 min (minor) 96% ee.

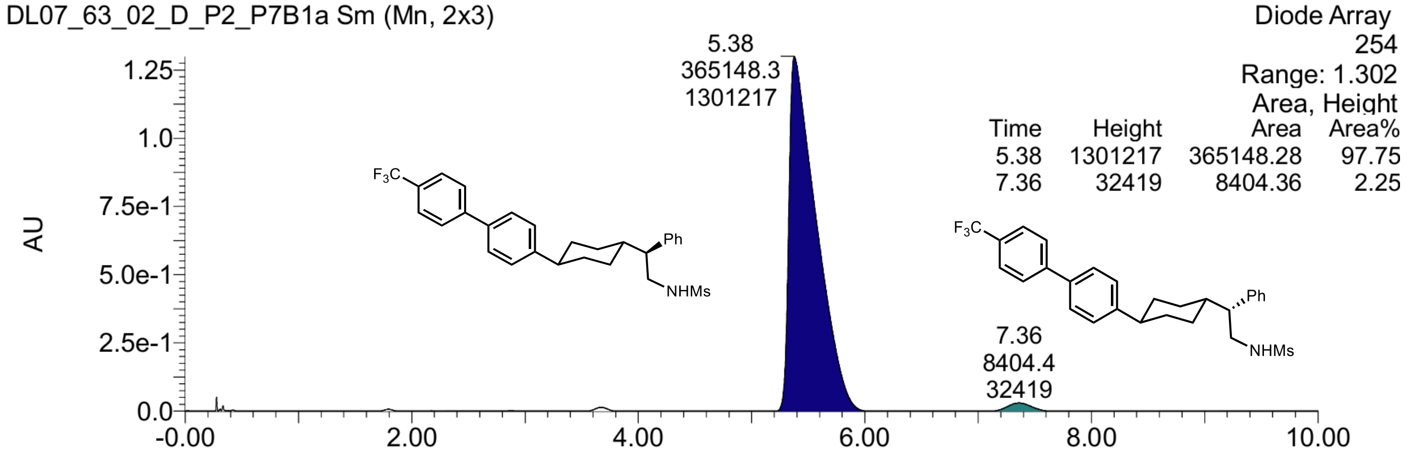

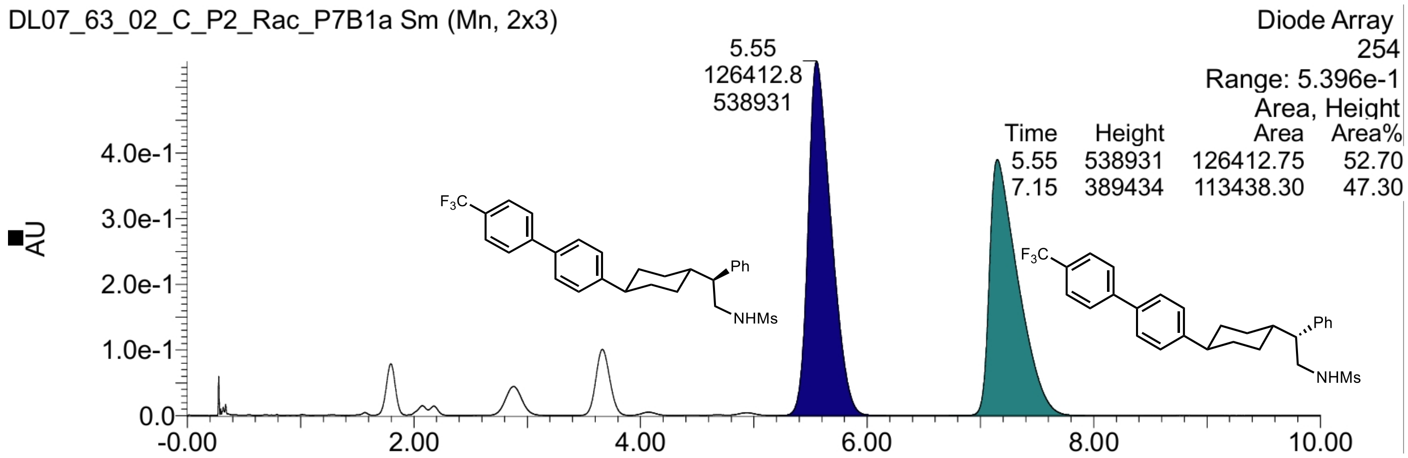

Figure S119. SFC trace of 46 (chiral top, racemic bottom)

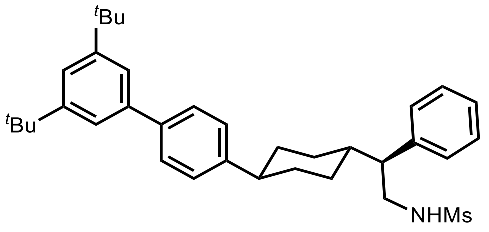

.

N-((S)-2-((1r,4S)-4-(3',5'-di-tert-butyl-[1,1'-biphenyl]-4-yl)cyclohexyl)-2-phenylethyl)methanesulfonamide (47)

Prepared according to **general procedure H**, 3,5-di-tert-butyl-4'-cyclohexyl-1,1'-biphenyl (174 mg, 0.5 mmol, 2.5 equiv), Rh_2(*S-tetra*-$MeOC_6H_4NTTL)_4$ (3.0 mg, 0.0001 mmol, 0.005 equiv), and 1-(methylsulfonyl)-4-(m-tolyl)-1H-1,2,3-triazole (47.5 mg, 0.2 mmol, 1.0 equiv) were used. The reaction was run for 24 hours. The crude mixture was reduced by $NaBH_4$ (18.9 mg, 0.5 mmol, 2.5 equiv) at 0 ˚C then purified by flash chromatography (SiO_2, gradient 0%-65% Et_2O in hexane) afforded **47** as a white solid (72.8 mg, 67% yield, 97% ee).

$\mathbf{R_f}$ (3H/2EA) = 0.40 (CAM, UV 254 nm)

$[\alpha]^{20}_D$**:** -23.9º (c = 0.73 g/100 ml, $CHCl_3$, 97% ee)

^{1}H NMR (400 MHz, $CDCl_3$) δ 7.50 (d, *J* = 8.2 Hz, 2H), 7.44 – 7.33 (m, 5H), 7.32 – 7.27 (m, 1H), 7.24 (d, *J* = 8.2 Hz, 2H), 7.20 (d, *J* = 6.8 Hz, 2H), 3.92 (dd, *J* = 8.7, 3.6 Hz, 1H), 3.69 (ddd, *J* = 13.0, 8.7, 4.6 Hz, 1H), 3.35 (ddd, *J* = 12.5, 10.6, 3.7 Hz, 1H), 2.81 (s, 3H), 2.64 (ddd, *J* = 10.4, 8.4, 4.6 Hz, 1H), 2.48 (tt, *J* = 12.2, 3.4 Hz, 1H), 2.12 (dt, *J* = 12.6, 3.0 Hz, 1H), 2.03 (dt, *J* = 13.2, 3.0 Hz, 1H), 1.88 (dt, *J* = 13.1, 3.0 Hz, 1H), 1.68 (dtd, *J* = 11.7, 8.4, 4.2 Hz, 1H), 1.63 – 1.48 (m, 2H), 1.47 – 1.33 (m, 19H), 1.25 (qd, *J* = 12.7, 3.4 Hz, 1H), 1.05 (qd, *J* = 12.9, 3.5 Hz, 1H).

^{13}C NMR (101 MHz, $CDCl_3$) δ 151.1, 146.0, 140.7, 140.7, 140.5, 129.1, 128.6, 127.6, 127.5, 127.1, 121.8, 121.3, 52.6, 46.3, 44.0, 40.6, 40.3, 35.1, 34.2, 34.1, 31.7, 31.6, 31.3.

HRMS (-p APCI) calcd. for [$C_{35}H_{46}O_2N^{32}S$] ([M-H]$^-$) 544.3255 found 544.3256.

HPLC (Chiralpak ADH column, 5% i-propanol in hexane, 1.0 mLmin^{-1}, 1.0 mgmL^{-1}, 30 min, UV 230 nm) retention times of 10.6 min (minor) and 14.4 min (major), 97% ee.

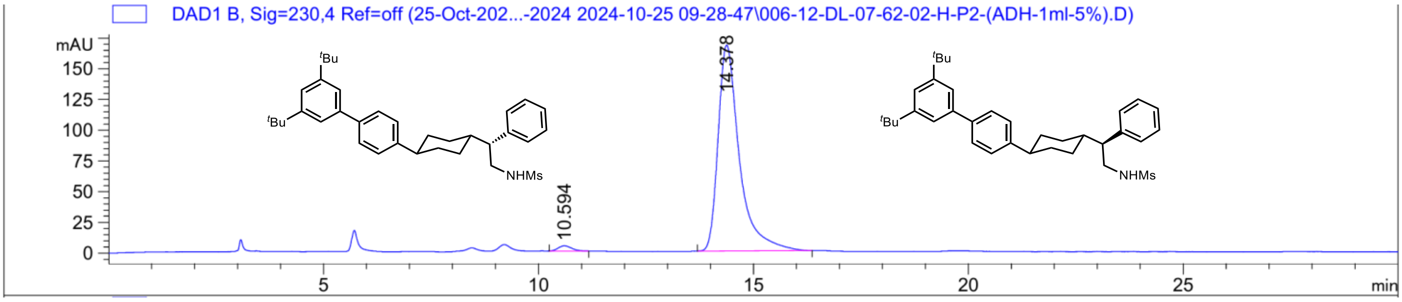

Signal 2: DAD1 B, Sig=230,4 Ref=off

Peak #	RetTime [min]	Type	Width [min]	Area [mAU*s]	Height [mAU]	Area %
1	10.594	BB	0.2576	92.49525	4.21304	1.5732
2	14.378	BB	0.4744	5786.81885	167.57245	98.4268
Totals :				5879.31409	171.78549	

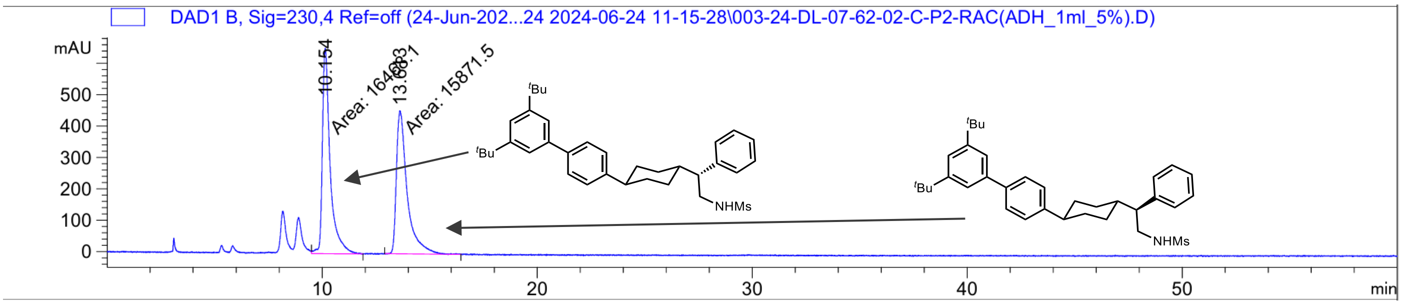

Signal 2: DAD1 B, Sig=230,4 Ref=off

Peak #	RetTime [min]	Type	Width [min]	Area [mAU*s]	Height [mAU]	Area %
1	10.154	MM	0.4186	1.64681e4	655.66034	50.9223
2	13.613	MM	0.5786	1.58715e4	457.20547	49.0777
Totals :				3.23396e4	1112.86581	

Figure S120. HPLC trace of 47 (chiral – top, racemic – bottom).

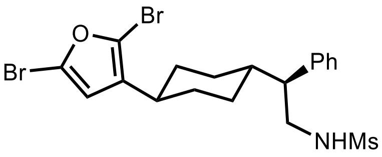

N-((S)-2-((1r,4S)-4-(2,5-dibromofuran-3-yl)cyclohexyl)-2-phenylethyl)methanesulfonamide (48)

Prepared according to **general procedure H**, 2,5-dibromo-3-cyclohexylfuran (154 mg, 0.5 mmol, 2.5 equiv), Rh_2(*S*-*tetra*-$MeOC_6H_4NTTL)_4$ (3.0 mg, 0.0001 mmol, 0.005 equiv), and 1-(methylsulfonyl)-4-phenyl-1H-1,2,3-triazole (44.7 mg, 0.2 mmol, 1.0 equiv) were used. The crude mixture reduced by $NaBH_4$ (18.9 mg, 0.5 mmol, 2.5 equiv) at 0 ˚C then purified by flash chromatography (gradient 0%-65% Et_2O in hexane) afforded **48** as a white solid (68.9 mg, 68% yield, 95% ee). *Note. This compound slowly decomposed under air at room temperature into yellow oil.*

R_f (3H/2EA) = 0.50 (UV 254 nm, CAM)

$[\alpha]^{20}_D$: -3.76° (c = 1.34 g/100 ml, $CHCl_3$, 95% ee)

^{1}H NMR (400 MHz, CDCl$_3$) δ 7.40 – 7.32 (m, 2H), 7.31 – 7.26 (m, 1H), 7.19 – 7.11 (m, 2H), 6.18 (s, 1H), 3.92 (dd, *J* = 8.7, 3.7 Hz, 1H), 3.63 (ddd, *J* = 13.0, 8.7, 4.6 Hz, 1H), 3.31 (ddd, *J* = 12.5, 10.5, 3.8 Hz, 1H), 2.79 (s, 3H), 2.59 (ddd, *J* = 10.4, 8.4, 4.6 Hz, 1H), 2.29 (tt, *J* = 12.2, 3.6 Hz, 1H), 2.03 (dp, *J* = 12.9, 3.2 Hz, 1H), 1.86 (dp, *J* = 12.8, 3.3 Hz, 1H), 1.71 (dp, *J* = 12.9, 3.3 Hz, 1H), 1.65 – 1.56 (m, 1H), 1.50 (dp, *J* = 12.7, 3.1 Hz, 1H), 1.33 (qd, *J* = 12.8, 3.4 Hz, 1H), 1.17 (ddt, *J* = 16.4, 12.8, 6.4 Hz, 2H), 0.96 (qd, *J* = 13.0, 3.5 Hz, 1H).

^{13}C NMR (201 MHz, CDCl$_3$) δ 140.4, 131.7, 129.1, 128.6, 127.5, 121.4, 118.6, 112.9, 52.5, 46.2, 40.4, 40.3, 34.9, 32.4, 32.4, 31.0, 30.7.

HRMS (-p APCI) calcd. for [$C_{19}H_{22}O_3N^{79}Br_2^{32}S$] ([M-H]$^-$) 501.9693 found 501.9680.
SFC (OJ3, 7% (50% methanol in isopropanol with 0.2% Formic Acid) in CO_2, 2.5 mL/min, 1.0 mg/ml, UV 230 nm) retention times of 6.44 (major) and 8.04 min (minor) 95% ee.

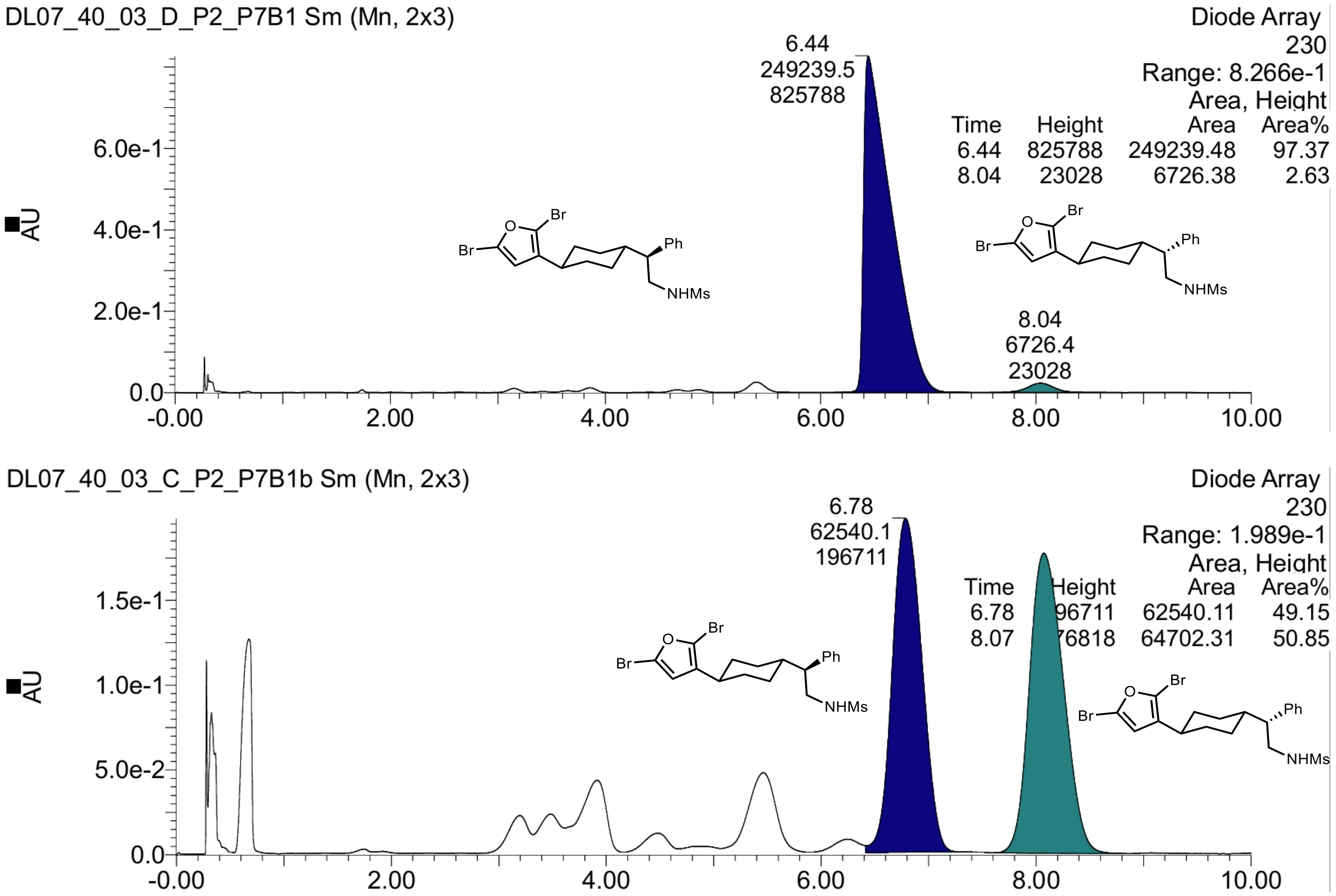

Figure S121. SFC trace of 48 (chiral top, racemic bottom).

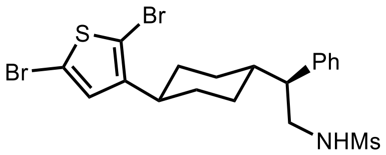

N-((S)-2-((1r,4S)-4-(2,5-dibromothiophen-3-yl)cyclohexyl)-2-phenylethyl)methanesulfonamide (49)

Prepared according to **general procedure H**, 2,5-dibromo-3-cyclohexylthiophene (162 mg, 0.5 mmol, 2.5 equiv), Rh$_2$(*S-tetra*-MeOC$_6$H$_4$NTTL)$_4$ (3.0 mg, 0.0001 mmol, 0.005 equiv), and 1-(methylsulfonyl)-4-phenyl-1H-1,2,3-triazole (44.7 mg, 0.2 mmol, 1.0 equiv) were used. The crude mixture reduced by NaBH$_4$ (18.9 mg, 0.5 mmol, 2.5 equiv) at 0

˚C then purified by flash chromatography (gradient 0%-65% Et_2O in hexane) afforded **49** as a white solid (62.5 mg, 60% yield, 93% ee).

R_f (3H/2EA) = 0.70 (UV 254 nm, CAM)

$[\alpha]^{20}_{D}$: -14.2° (c = 1.17 g/100 ml, $CHCl_3$, 93% ee)

^{1}H NMR (400 MHz, $CDCl_3$) δ 7.40 – 7.32 (m, 2H), 7.31 – 7.26 (m, 1H), 7.21 – 7.10 (m, 2H), 6.75 (s, 1H), 3.93 (dd, *J* = 8.6, 3.7 Hz, 1H), 3.64 (ddd, *J* = 13.0, 8.7, 4.6 Hz, 1H), 3.32 (ddd, *J* = 12.5, 10.6, 3.8 Hz, 1H), 2.79 (s, 3H), 2.66 – 2.51 (m, 2H), 2.05 (dt, *J* = 12.7, 3.2 Hz, 1H), 1.96 – 1.84 (m, 1H), 1.81 – 1.69 (m, 1H), 1.68 – 1.58 (m, 1H), 1.52 (dt, *J* = 13.2, 3.0 Hz, 1H), 1.35 (qd, *J* = 12.7, 3.3 Hz, 1H), 1.21 (pd, *J* = 12.8, 3.5 Hz, 2H), 1.00 (qd, *J* = 13.0, 3.5 Hz, 1H).

^{13}C NMR (201 MHz, $CDCl_3$) δ 147.0, 140.4, 129.1, 128.8, 128.6, 127.5, 110.8, 107.3, 52.5, 46.2, 40.4, 40.4, 38.9, 32.7, 32.7, 31.1, 30.8.

HRMS (-p APCI) calcd. for $[C_{19}H_{22}O_2N^{79}Br_2{}^{32}S_2]$ ($[M-H]^-$) 517.9464 found 517.9456.
SFC (OJ3, 10% (50% methanol in isopropanol with 0.2% Formic Acid) in CO_2, 2.5 mL/min, 1.0 mg/ml, UV 254 nm) retention times of 5.22 (major) and 6.00 min (minor) 93% ee.

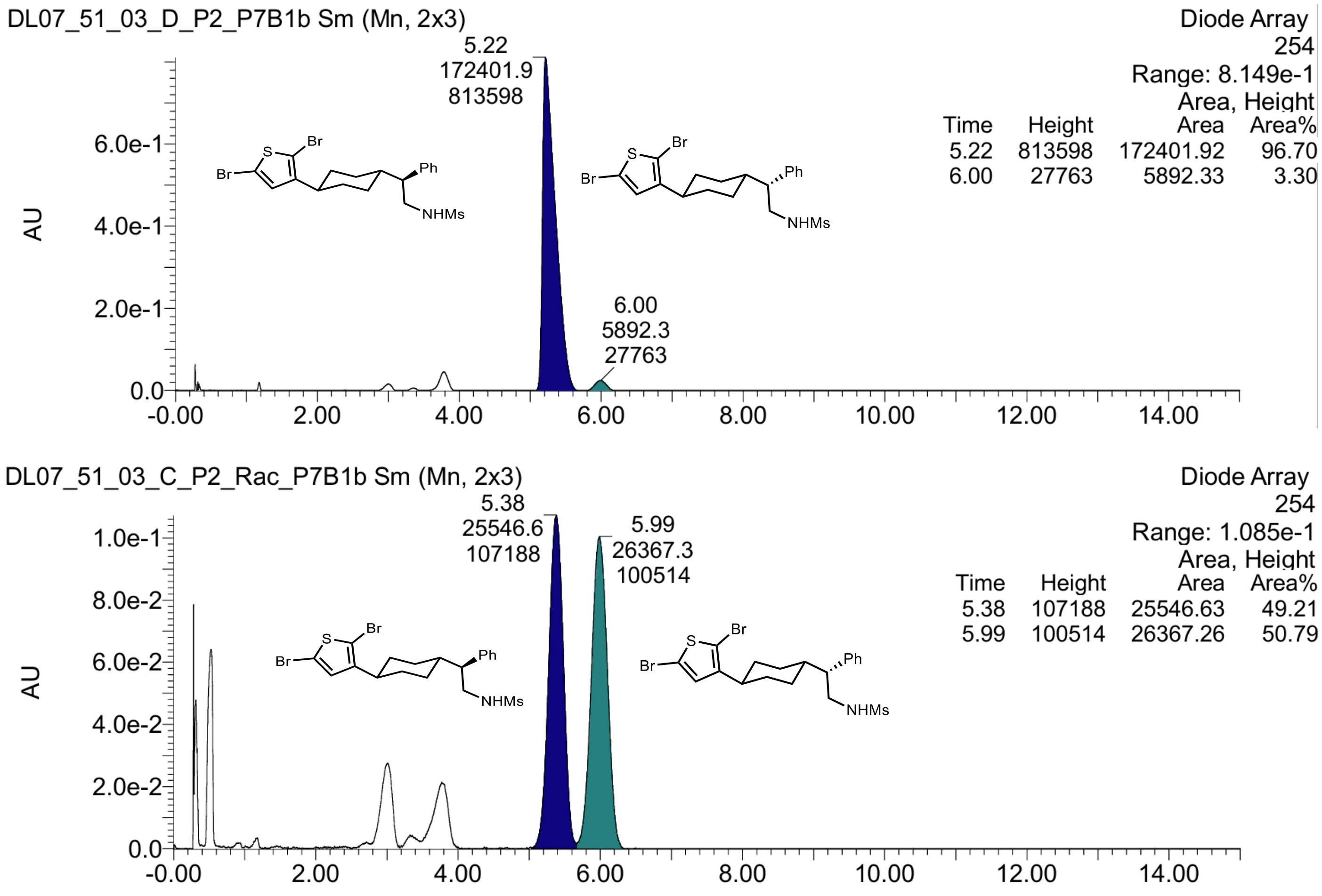

Figure S122. SFC trace of 49 (chiral top, racemic bottom).

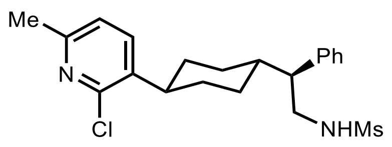

N-((S)-2-((1r,4S)-4-(2-chloro-6-methylpyridin-3-yl)cyclohexyl)-2-phenylethyl)methanesulfonamide (50)

Prepared according to **general procedure H**, 2-chloro-3-cyclohexyl-6-methylpyridine (105 mg, 0.5 mmol, 2.5 equiv), Rh_2(*S*-*tetra*-$MeOC_6H_4NTTL)_4$ (3.0 mg, 0.0001 mmol, 0.005 equiv), and 1-(methylsulfonyl)-4-phenyl-1H-1,2,3-triazole (44.7 mg, 0.2 mmol, 1.0 equiv) were used. The crude mixture reduced by $NaBH_4$ (18.9 mg, 0.5 mmol, 2.5 equiv) at 0 ˚C then purified by flash chromatography (gradient 0%-65% Et_2O in hexane) afforded **50** as a white solid (40 mg, 49%, 82% ee) – a mixture of C4 and C3 product (8:1).

R_f (3H/2EA) = 0.50 (CAM)

$[\alpha]^{20}_D$: -8.9° (c = 1.15 g/100 ml, $CHCl_3$, 82% ee)

^{1}H NMR (600 MHz, $CDCl_3$) δ 7.40 (d, *J* = 7.8 Hz, 1H), 7.36 (t, *J* = 7.5 Hz, 2H), 7.32 – 7.26 (m, 1H), 7.17 (d, *J* = 7.5 Hz, 2H), 7.03 (d, *J* = 7.8 Hz, 1H), 3.94 (dd, *J* = 8.7, 3.8 Hz, 1H), 3.65 (ddd, *J* = 12.9, 8.6, 4.6 Hz, 1H), 3.38 – 3.30 (m, 1H), 2.83 (td, *J* = 10.2, 5.9 Hz, 1H), 2.79 (d, *J* = 4.7 Hz, 3H), 2.63 (td, *J* = 9.4, 4.6 Hz, 1H), 2.47 (s, 3H), 2.10 (dt, *J* = 13.0, 3.1 Hz, 1H), 1.99 (dt, *J* = 12.9, 3.2 Hz, 1H), 1.84 (dt, *J* = 13.1, 3.2 Hz, 1H), 1.65 (dtd, *J* = 11.9, 8.4, 4.1 Hz, 1H), 1.59 – 1.52 (m, 1H), 1.42 – 1.32 (m, 1H), 1.31 – 1.21 (m, 2H), 1.07 (qd, *J* = 12.8, 3.4 Hz, 1H).

^{13}C NMR (151 MHz, $CDCl_3$) δ 156.5, 150.0, 140.5, 137.0, 136.0, 129.1, 128.6, 127.5, 122.4, 52.4, 46.2, 40.5, 40.4, 39.7, 32.4, 32.4, 31.3, 31.0, 23.7.

HRMS (-p APCI) calcd. for $[C_{21}H_{26}O_2N_2{}^{35}Cl^{32}S]$ ($[M-H]^-$) 405.1409 found 405.1403.
SFC (OJ3, 10% (50% methanol in isopropanol with 0.2% Formic Acid) in CO_2, 2.5 mL/min, 1.0 mg/ml, UV 210 nm) retention times of 3.90 (major) and 5.93 min (minor) 82% ee.

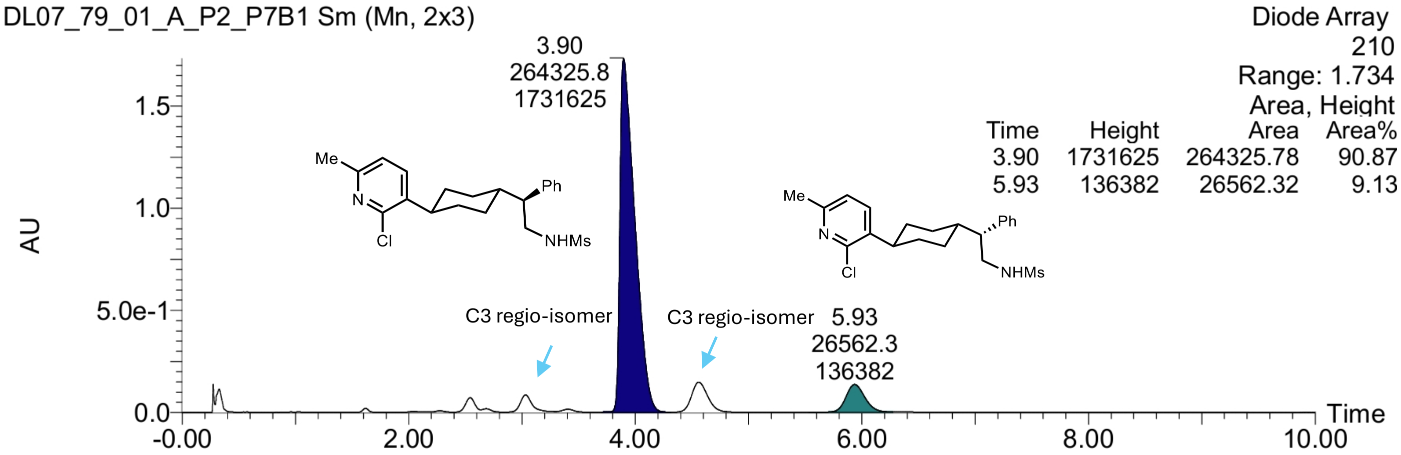

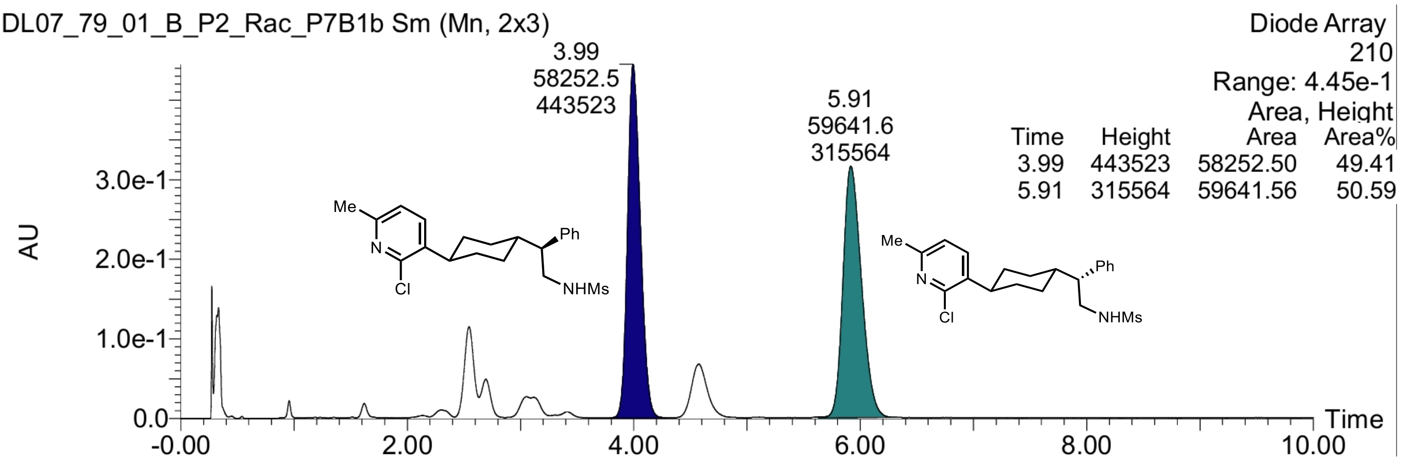

Figure S123. SFC trace of 50 (chiral top, racemic bottom). The product was isolated as a mixture of C3 (inseparable) and C4 products.

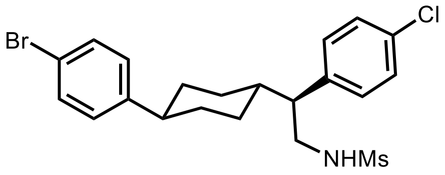

N-((S)-2-((1r,4S)-4-(4-bromophenyl)cyclohexyl)-2-(4-chlorophenyl)ethyl)methanesulfonamide (51)

Prepared according to **general procedure H**, 1-bromo-4-cyclohexylbenzene (120 mg, 0.5 mmol, 2.5 equiv), Rh_2(*S*-*tetra*-$MeOC_6H_4$NTTL)$_4$ (3.0 mg, 0.0001 mmol, 0.005 equiv), and 4-(4-chlorophenyl)-1-(methylsulfonyl)-1H-1,2,3-triazole (51.5 mg, 0.2 mmol, 1.0 equiv) were used. The crude mixture reduced by $NaBH_4$ (18.9 mg, 0.5 mmol, 2.5 equiv) at 0 ˚C then purified by flash chromatography (SiO_2, gradient 0%-65% Et_2O in hexane) afforded **51** as a white solid (49.6 mg, 53% yield, 98% ee).

R_f (3H/2EA) = 0.50 (CAM, UV 210 nm)

$[\alpha]^{20}_D$: -35.0° (c = 0.19 g/100 ml, $CHCl_3$, 98% ee)

^{1}H NMR (800 MHz, $CDCl_3$) δ 7.38 (d, *J* = 6.2 Hz, 2H), 7.34 (d, *J* = 6.2 Hz, 2H), 7.11 (d, *J* = 6.1 Hz, 2H), 7.03 (d, *J* = 6.2 Hz, 2H), 3.89 (d, *J* = 9.2 Hz, 1H), 3.69 – 3.59 (m, 1H), 3.29 (ddd, *J* = 12.3, 9.5, 3.1 Hz, 1H), 2.83 (s, 3H), 2.67 – 2.58 (m, 1H), 2.37 (dt, *J* = 12.4, 7.6 Hz, 1H), 2.05 (d, *J* = 10.0 Hz, 1H), 1.93 (d, *J* = 13.6 Hz, 1H), 1.80 (d, *J* = 12.7 Hz, 1H), 1.61 (dq, *J* = 11.7, 4.1 Hz, 1H), 1.55 – 1.50 (m, 1H), 1.43 (q, *J* = 12.8 Hz, 1H), 1.31 (q, *J* = 12.9 Hz, 1H), 1.19 (q, *J* = 12.6 Hz, 1H), 0.98 (q, *J* = 12.7 Hz, 1H).

^{13}C NMR (201 MHz, $CDCl_3$) δ 146.1, 139.1, 133.3, 131.5, 129.9, 129.3, 128.6, 119.8, 52.0, 46.1, 43.7, 40.5, 40.4, 34.0, 33.9, 31.4, 31.0.

HRMS (+p APCI) calcd. for [$C_{21}H_{26}O_2N^{79}Br^{35}Cl^{32}S$] ([M+H]$^+$) 470.0551 found 470.0562.

SFC (OJ3, 15% (50% methanol in isopropanol with 0.2% Formic Acid) in CO_2, 2.5 mL/min, 1.0 mg/ml, UV 210 nm) retention times of 5.45 min (major) and 7.39 min (minor) 98% ee.

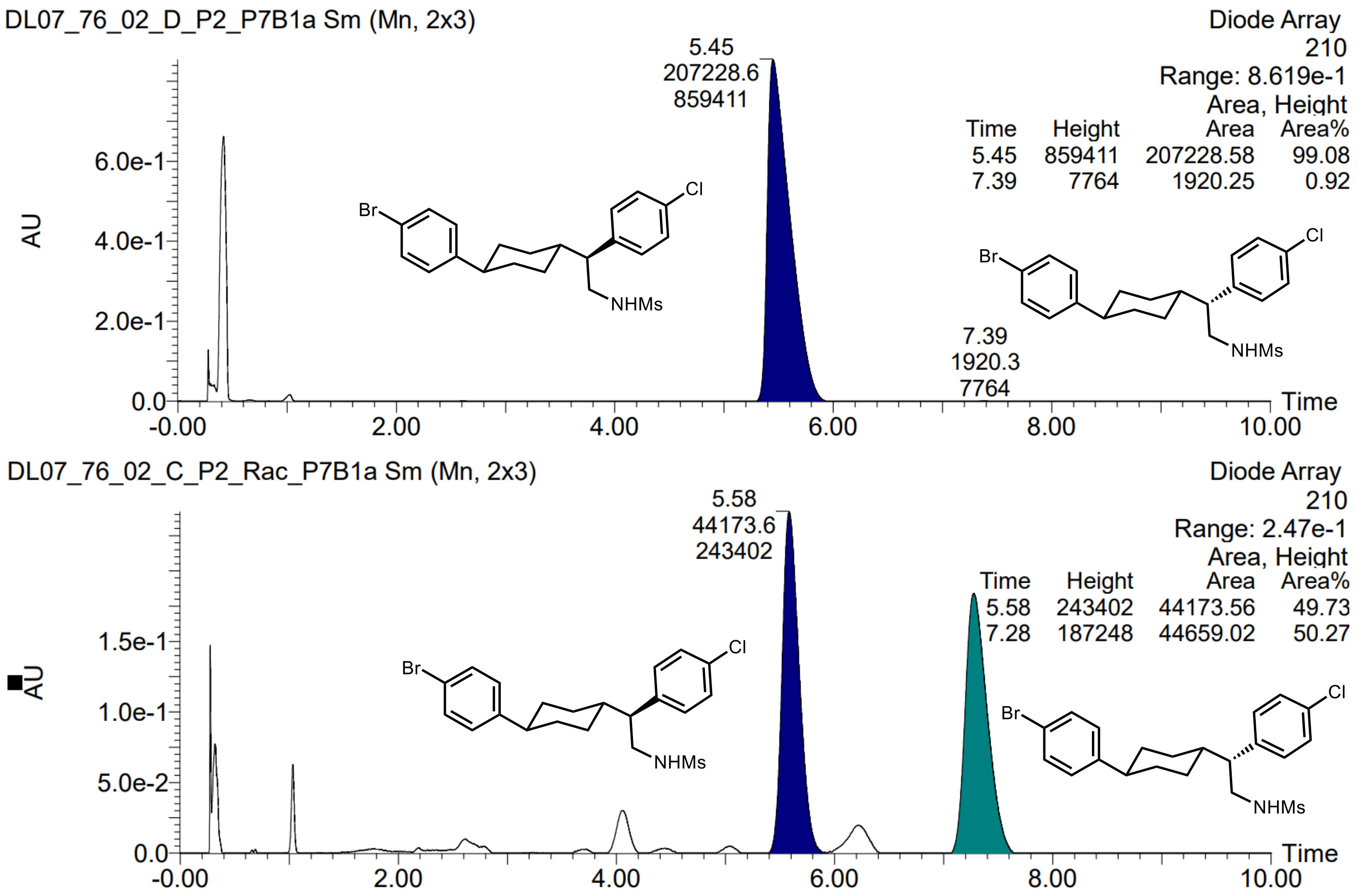

Figure S124. SFC trace of 51 (chiral – top, racemic – bottom).

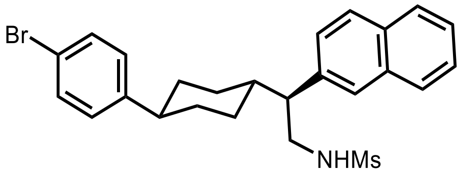

N-((S)-2-((1r,4S)-4-(4-bromophenyl)cyclohexyl)-2-(naphthalen-2-yl)ethyl)methanesulfonamide (52)

Prepared according to **general procedure H**, 1-bromo-4-cyclohexylbenzene (120 mg, 0.5 mmol, 2.5 equiv), Rh_2(*S*-*tetra*-MeOC_6H_4NTTL$)_4$ (3.0 mg, 0.0001 mmol, 0.005 equiv), and 1-(methylsulfonyl)-4-(naphthalen-2-yl)-1H-1,2,3-triazole (54.7 mg, 0.2 mmol, 1.0 equiv) were used. The crude mixture reduced by $NaBH_4$ (18.9 mg, 0.5 mmol, 2.5 equiv) at 0 ˚C then purified by flash chromatography (SiO_2, gradient 0%-65% Et_2O in hexane) afforded **52** as a white solid (71.8 mg, 74% yield, 99% ee).

R_f (3H/2EA) = 0.50 (UV 254 nm)

$[\alpha]^{20}_D$: -44.4° (c = 0.37 g/100 ml, $CHCl_3$, 99% ee)

^{1}H NMR (800 MHz, $CDCl_3$) δ 7.86 (d, *J* = 8.6 Hz, 1H), 7.84 (d, *J* = 9.5 Hz, 1H), 7.82 (d, *J* = 8.0 Hz, 1H), 7.52 (dd, *J* = 8.2, 6.5 Hz, 1H), 7.49 (dd, *J* = 8.3, 6.6 Hz, 1H), 7.38 (d, *J* = 6.8 Hz, 2H), 7.31 (d, *J* = 8.4 Hz, 1H), 7.03 (d, *J* = 8.4 Hz, 2H), 3.92 (d, *J* = 5.4 Hz, 1H), 3.77 – 3.66 (m, 1H), 3.42 (ddd, *J* = 14.4, 10.0, 2.9 Hz, 1H), 2.79 (s, 3H), 2.39 (t, *J* = 12.3 Hz, 1H), 2.14 (d, *J* = 12.2 Hz, 1H), 1.96 (d, *J* = 12.8 Hz, 1H), 1.80 – 1.72 (m, 2H), 1.56 (d, *J* = 14.7 Hz, 1H), 1.46 (q, *J* = 12.9 Hz, 1H), 1.32 (q, *J* = 11.9 Hz, 1H), 1.25 (q, *J* = 13.0 Hz, 1H), 1.05 (q, *J* = 13.1 Hz, 1H).

^{13}C NMR (201 MHz, $CDCl_3$) δ 146.2, 138.0, 133.6, 132.8, 131.5, 129.0, 128.6, 127.9, 127.8, 127.8, 126.7, 126.2, 125.8, 119.7, 52.7, 46.1, 43.7, 40.5, 40.4, 34.1, 33.9, 31.5, 31.3.

HRMS (+p APCI) calcd. for $[C_{25}H_{29}O_2N^{79}Br^{32}S]$ ($[M+H]^+$) 486.1097 found 486.1100.

SFC (OJ3, 20% (50% methanol in isopropanol with 0.2% Formic Acid) in CO_2, 2.5 mL/min, 1.0 mg/ml, UV 210 nm) retention times of 5.06 min (major) and 6.47 min (minor) 99% ee.

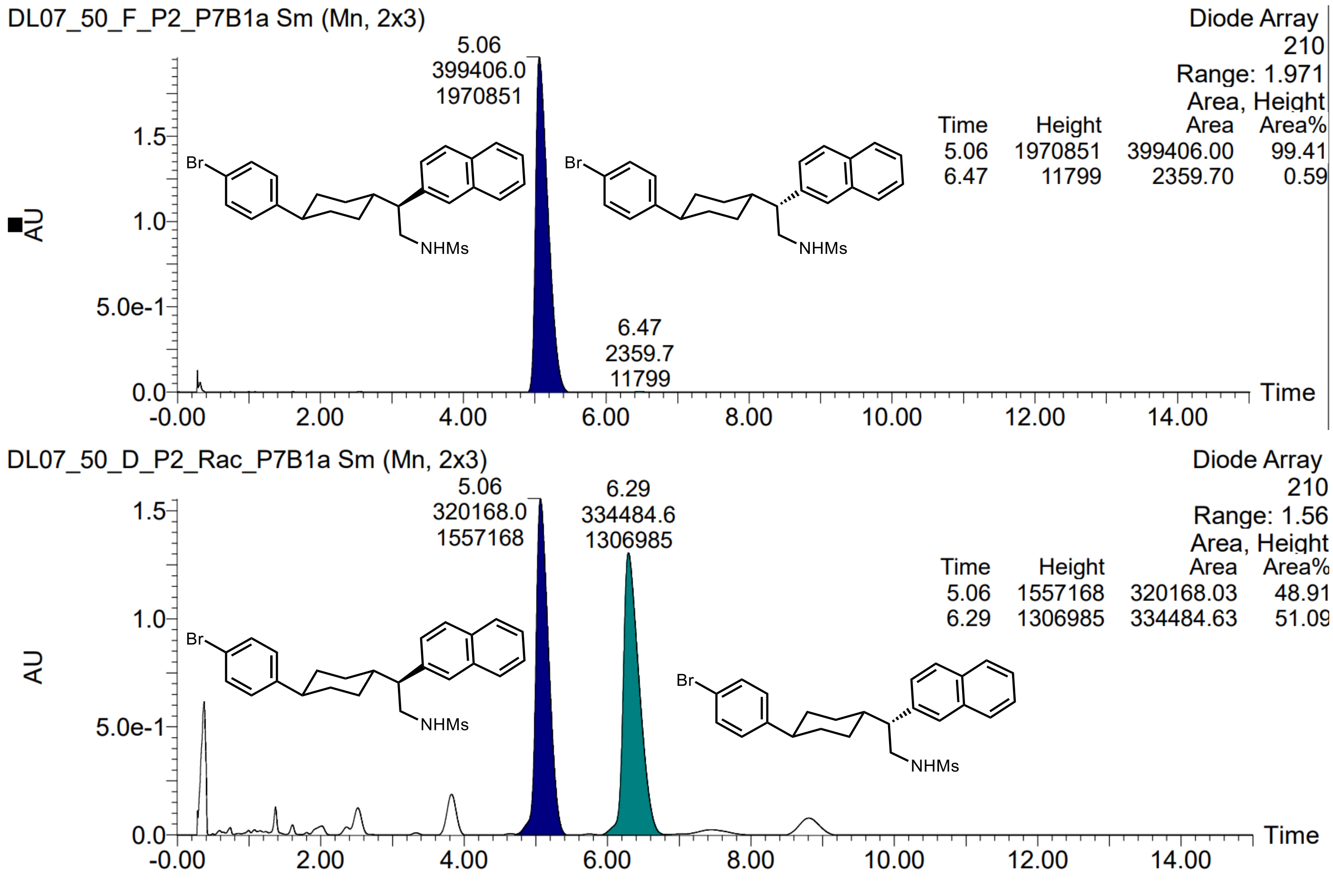

Figure S125. SFC trace of 52 (chiral – top, racemic – bottom).

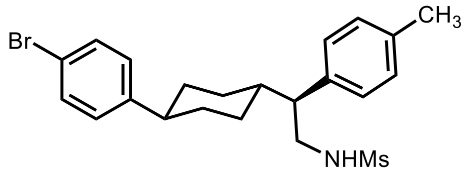

N-((S)-2-((1r,4S)-4-(4-bromophenyl)cyclohexyl)-2-(p-tolyl)ethyl)methanesulfonamide (53)

Prepared according to **general procedure H**, 1-bromo-4-cyclohexylbenzene (120 mg, 0.5 mmol, 2.5 equiv), Rh_2(*S*-*tetra*-$MeOC_6H_4NTTL)_4$ (3.0 mg, 0.0001 mmol, 0.005 equiv), and 1-(methylsulfonyl)-4-(p-tolyl)-1H-1,2,3-triazole (47.5 mg, 0.2 mmol, 1.0 equiv) were used. The crude mixture reduced by $NaBH_4$ (18.9 mg, 0.5 mmol, 2.5 equiv) at 0 ˚C then purified by flash chromatography (SiO_2, gradient 0%-65% Et_2O in hexane) afforded **53** as a white solid (61.3 mg, 68% yield, 98% ee).

R_f (3H/2EA) = 0.50 (CAM, UV 210 nm)

$[\alpha]^{20}_D$: -27.7° (c = 0.34 g/100 ml, $CHCl_3$, 98% ee)

^{1}H NMR (800 MHz, $CDCl_3$) δ 7.38 (d, J = 8.6 Hz, 2H), 7.16 (d, J = 7.5 Hz, 2H), 7.06 – 7.01 (m, 4H), 3.87 (d, J = 8.1 Hz, 1H), 3.63 (td, J = 8.7, 4.0 Hz, 1H), 3.28 (dd, J = 12.7, 10.4 Hz, 1H), 2.81 (d, J = 1.8 Hz, 3H), 2.57 (ddt, J = 13.1, 5.5, 3.1 Hz, 1H), 2.38 (td, J = 12.3, 3.2 Hz, 1H), 2.34 (d, J = 2.4 Hz, 3H), 2.07 (d, J = 13.0 Hz, 1H), 1.93 (d, J = 13.1 Hz, 1H), 1.78 (d, J = 13.2 Hz, 1H), 1.60 (qt, J = 8.9, 3.9 Hz, 1H), 1.55 (dd, J = 12.2, 3.3 Hz, 1H), 1.43 (q, J = 12.7 Hz, 1H), 1.31 (q, J = 12.9 Hz, 1H), 1.19 (q, J = 12.7 Hz, 1H), 0.99 (q, J = 13.0 Hz, 1H).

^{13}C NMR (201 MHz, $CDCl_3$) δ 146.3, 137.3, 137.2, 131.5, 129.8, 128.7, 128.5, 119.7, 52.0, 46.3, 43.8, 40.6, 40.3, 34.1, 34.0, 31.4, 31.2, 21.2.

HRMS (+p APCI) calcd. for [$C_{22}H_{29}O_2N^{79}Br^{32}S$] ($[M+H]^+$) 450.1097 found 450.1104.
SFC (OJ3, 10% (50% methanol in isopropanol with 0.2% Formic Acid) in CO_2, 2.5 mL/min, 1.0 mg/ml, UV 230 nm) retention times of 6.64 min (major) and 8.35 min (minor) 98% ee.

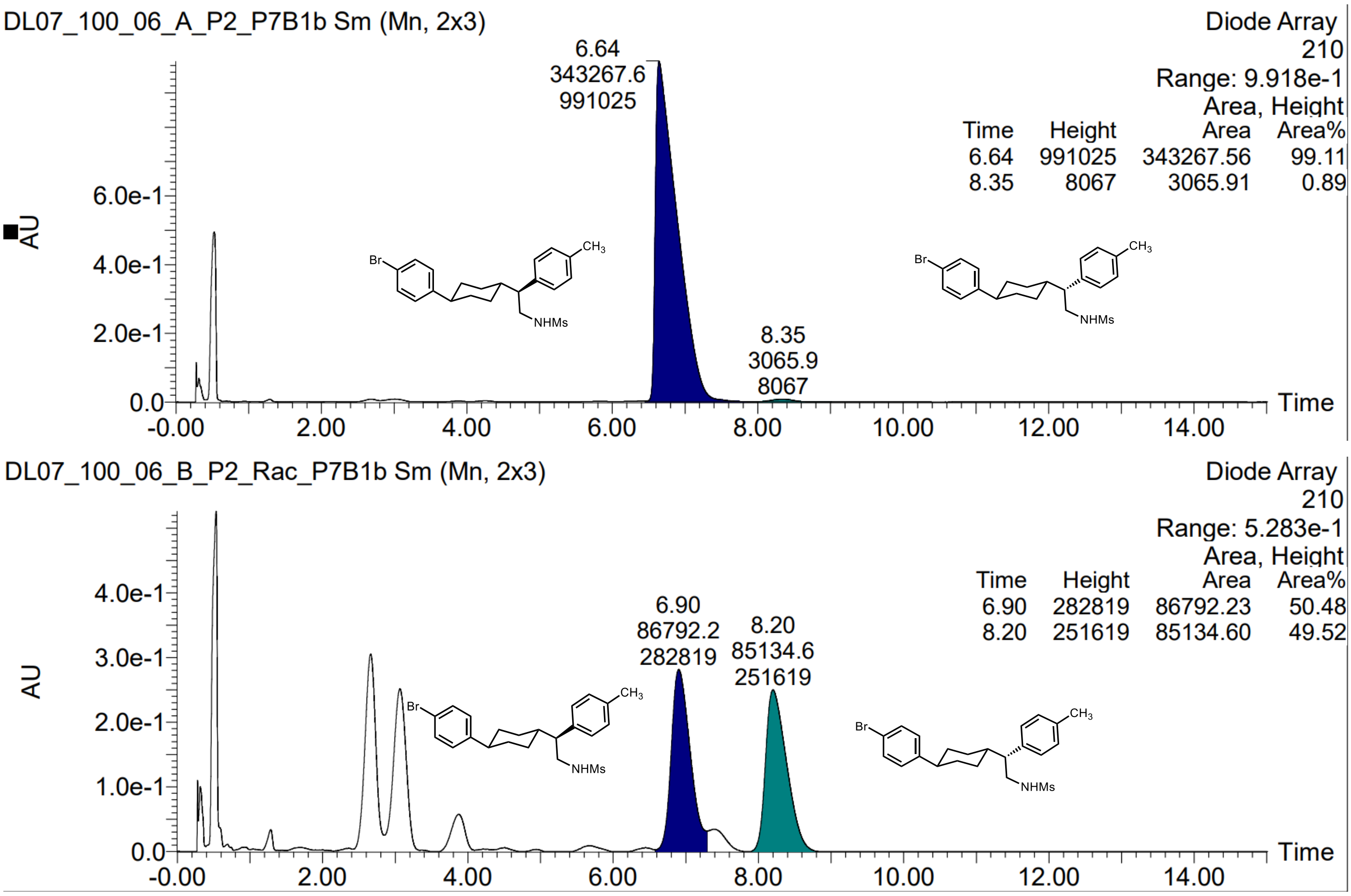

Figure S126. SFC trace of 53 (chiral – top, racemic – bottom).

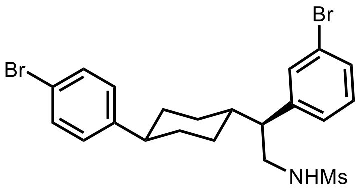

N-((S)-2-(3-bromophenyl)-2-((1r,4S)-4-(4-bromophenyl)cyclohexyl)ethyl)methanesulfonamide (54)

Prepared according to **general procedure H** with slight modification, 1-bromo-4-cyclohexylbenzene (120 mg, 0.5 mmol, 2.5 equiv), Rh_2(*S-tetra*-$MeOC_6H_4$NTTL)$_4$ (3.0 mg, 0.0001 mmol, 0.005 equiv), and methyl 4-(3-bromophenyl)-1-(methylsulfonyl)-1H-1,2,3-triazole (60.4 mg, 0.2 mmol, 1.0 equiv) were used. The reaction was run for 24 hours at 40 ºC. The crude mixture reduced by $NaBH_4$ (18.9 mg, 0.5 mmol, 2.5 equiv) at 0 ˚C then purified by flash

chromatography (SiO_2, gradient 0%-65% Et_2O in hexane) afforded **54** as a white solid (71.8 mg, 70% yield, 95% ee). *Note. 4-(3-bromophenyl)-1-(methylsulfonyl)-1H-1,2,3-triazole reacts slowly at room temperature with only 50% conversion after overnight with the regioselectivity of 16:1. However, slightly elevated temperature is required to achieve full conversion with a decrease in regioselectivity to 12:1.*

R_f (3H/2EA) = 0.50 (CAM, UV 210 nm)

$[\alpha]^{20}_D$: -24.5° (c = 0.24 g/100 ml, $CHCl_3$, 95% ee)

^{1}H NMR (800 MHz, $CDCl_3$) δ 7.42 (d, J = 8.0 Hz, 1H), 7.39 (d, J = 6.7 Hz, 2H), 7.32 (s, 1H), 7.24 (t, J = 7.8 Hz, 1H), 7.11 (d, J = 7.7 Hz, 1H), 7.03 (d, J = 6.9 Hz, 2H), 3.95 – 3.89 (m, 1H), 3.64 (td, J = 9.2, 4.3 Hz, 1H), 3.33 – 3.26 (m, 1H), 2.84 (s, 3H), 2.60 (ddd, J = 10.7, 7.5, 4.4 Hz, 1H), 2.39 (td, J = 12.0, 3.2 Hz, 1H), 2.06 (dd, J = 12.8, 3.0 Hz, 1H), 1.94 (dd, J = 13.3, 3.3 Hz, 1H), 1.81 (dd, J = 13.1, 3.1 Hz, 1H), 1.62 (dq, J = 12.1, 4.5 Hz, 1H), 1.54 (d, J = 13.3 Hz, 1H), 1.44 (q, J = 13.2 Hz, 1H), 1.33 (q, J = 12.8 Hz, 1H), 1.19 (q, J = 12.7 Hz, 1H), 1.00 (q, J = 12.9 Hz, 1H).

^{13}C NMR (201 MHz, $CDCl_3$) δ 146.1, 143.2, 131.5, 131.5, 130.7, 130.6, 128.6, 127.4, 123.3, 119.8, 52.5, 46.0, 43.7, 40.6, 40.4, 34.0, 33.9, 31.4, 31.0.

HRMS (+p APCI) calcd. for $[C_{21}H_{26}O_2N^{79}Br_2{}^{32}S]$ ($[M+H]^+$) 514.0046 found 514.0044.
SFC (CEL2, 20% (50% methanol in isopropanol with 0.2% Formic Acid) in CO_2, 2.5 mL/min, 1.0 mg/ml, UV 230 nm) retention times of 4.07 min (major) and 4.86 min (minor) 95% ee.

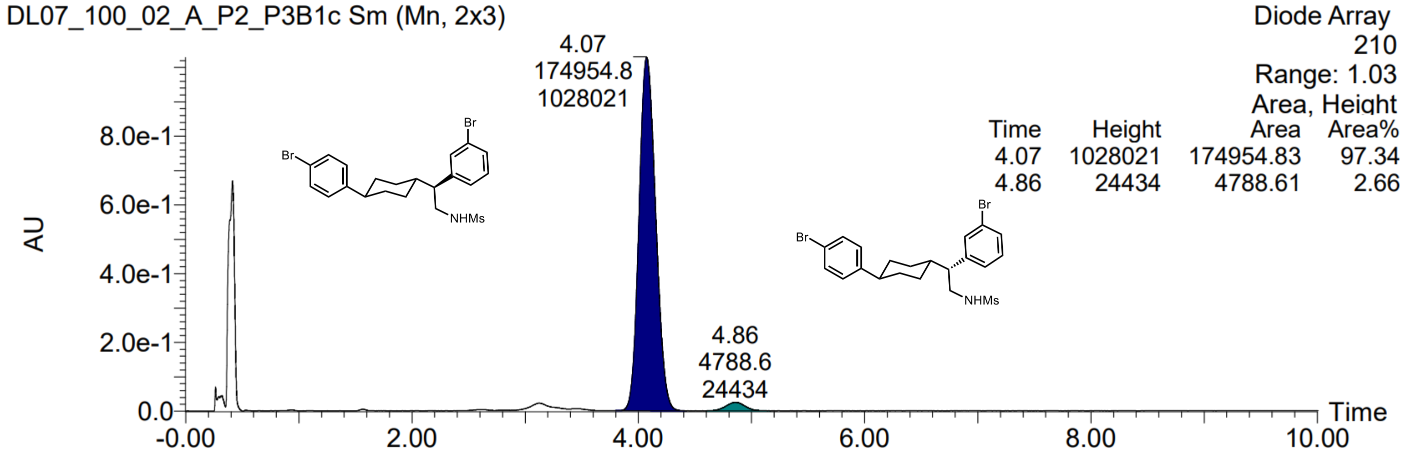

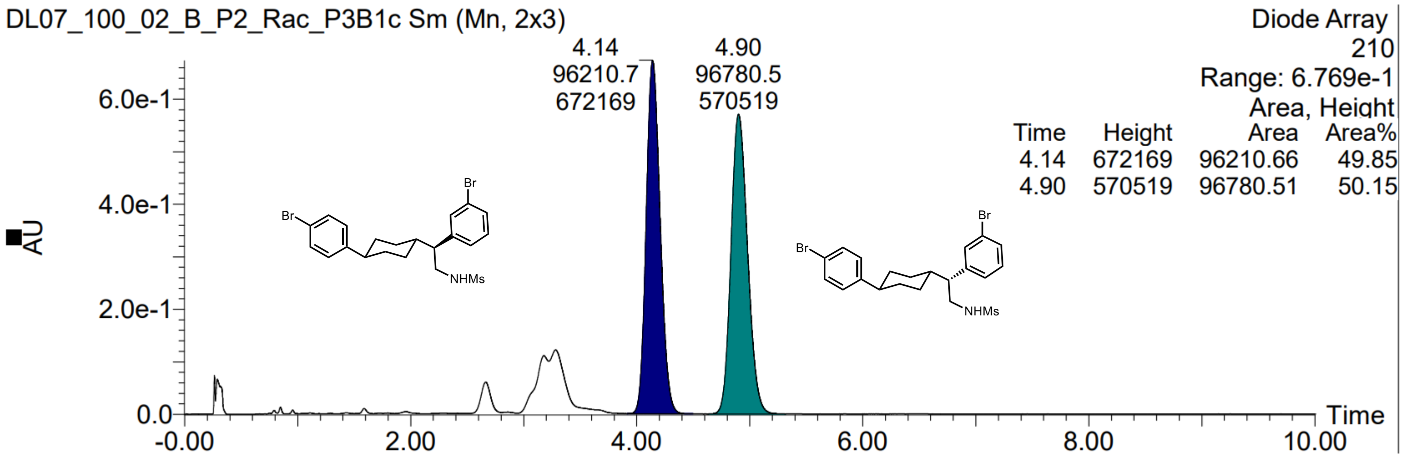

Figure S127. SFC trace of 54 (chiral – top, racemic – bottom).

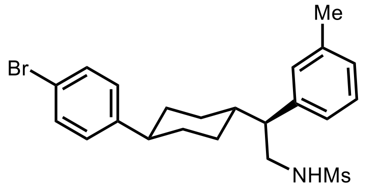

N-((S)-2-((1r,4S)-4-(4-bromophenyl)cyclohexyl)-2-(m-tolyl)ethyl)methanesulfonamide (55)

Prepared according to **general procedure H**, 1-bromo-4-cyclohexylbenzene (120 mg, 0.5 mmol, 2.5 equiv), Rh_2(*S*-*tetra*-$MeOC_6H_4NTTL)_4$ (3.0 mg, 0.0001 mmol, 0.005 equiv), and 1-(methylsulfonyl)-4-(m-tolyl)-1H-1,2,3-triazole (47.5 mg, 0.2 mmol, 1.0 equiv) were used. The reaction was run for 24 hours. The crude mixture was reduced by $NaBH_4$ (18.9 mg, 0.5 mmol, 2.5 equiv) at 0 ˚C then purified by flash chromatography (SiO_2, gradient 0%-75% Et_2O in hexane) afforded **55** as a white solid (54.4 mg, 60% yield, 97% ee).

R_f (3H/2EA) = 0.60 (CAM, UV 210 nm)

$[\alpha]^{20}_D$: -28.4° (c = 0.45 g/100 ml, $CHCl_3$, 97% ee)

^{1}H NMR (800 MHz, $CDCl_3$) δ 7.38 (d, *J* = 6.3 Hz, 2H), 7.24 (t, *J* = 7.8 Hz, 1H), 7.09 (d, *J* = 7.6 Hz, 1H), 7.03 (d, *J* = 6.2 Hz, 2H), 6.97 – 6.93 (m, 2H), 3.89 (d, *J* = 7.5 Hz, 1H), 3.64 (td, *J* = 9.8, 4.3 Hz, 1H), 3.30 (t, *J* = 11.5 Hz, 1H), 2.81 (s, 3H), 2.59 – 2.52 (m, 1H), 2.42 – 2.37 (m, 1H), 2.36 (s, 3H), 2.07 (dd, *J* = 13.0, 3.0 Hz, 1H), 1.97 – 1.89 (m, 1H), 1.79 (dd, *J* = 13.2, 3.1 Hz, 1H), 1.65 – 1.58 (m, 1H), 1.57 – 1.52 (m, 1H), 1.44 (q, *J* = 12.7 Hz, 1H), 1.32 (q, *J* = 12.9 Hz, 1H), 1.20 (q, *J* = 12.8 Hz, 1H), 1.00 (q, *J* = 12.9 Hz, 1H).

^{13}C NMR (201 MHz, $CDCl_3$) δ 146.3, 140.5, 138.8, 131.5, 129.4, 129.0, 128.7, 128.3, 125.5, 119.7, 52.4, 46.3, 43.8, 40.5, 40.3, 34.1, 34.0, 31.4, 31.2, 21.7.

HRMS (+p APCI) calcd. for $[C_{22}H_{29}O_2N^{79}Br^{32}S]$ ($[M+H]^+$) 450.1097 found 450.1100.
SFC (OJ3, 10% (50% methanol in isopropanol with 0.2% Formic Acid) in CO_2, 2.5 mL/min, 1.0 mg/ml, UV 230 nm) retention times of 5.79 min (major) and 8.31 min (minor) 97% ee.

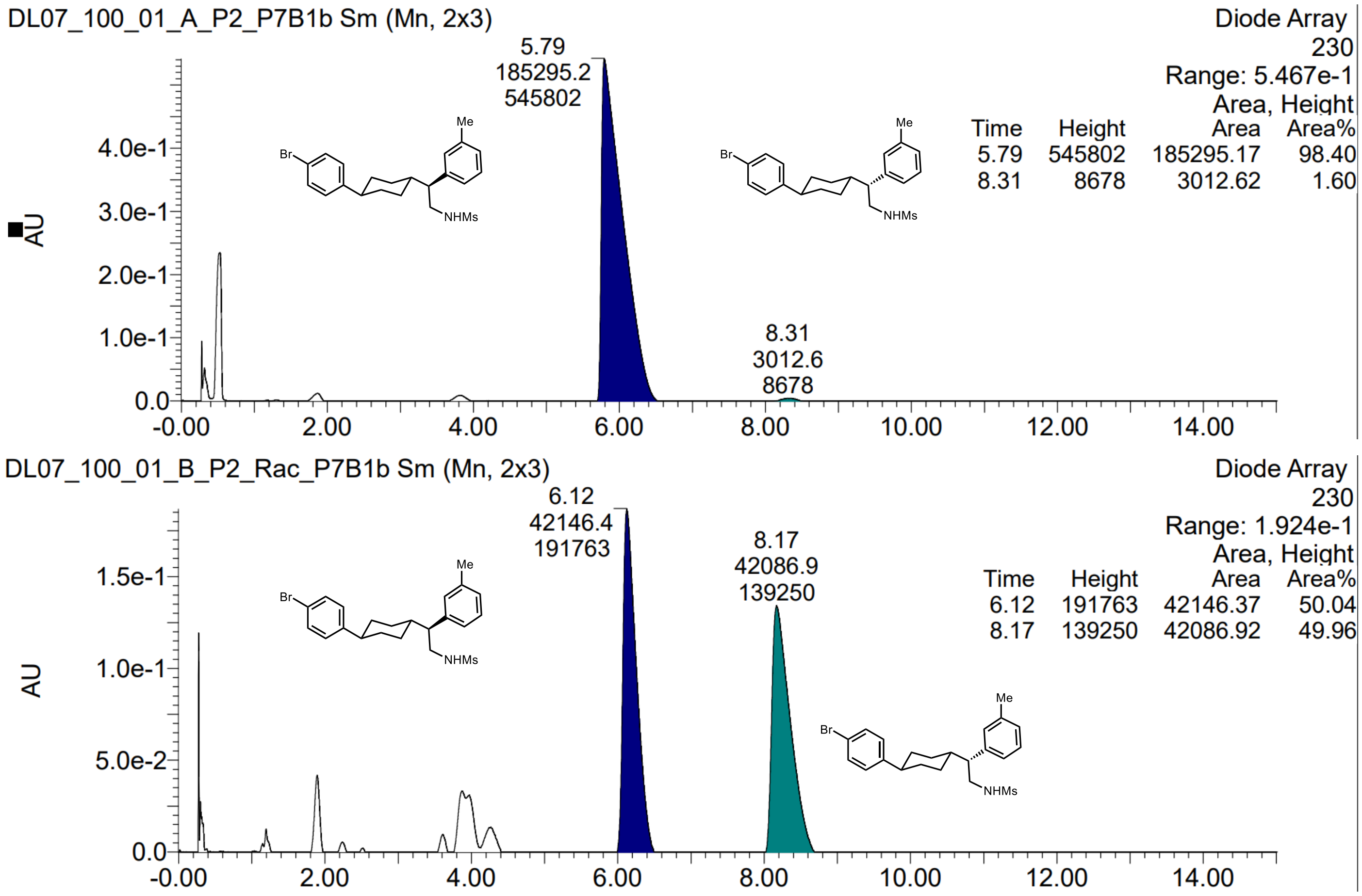

Figure S128. SFC trace of 55 (chiral – top, racemic – bottom).

10. Copies of NMR

10.1. Spectra of prepared starting materials

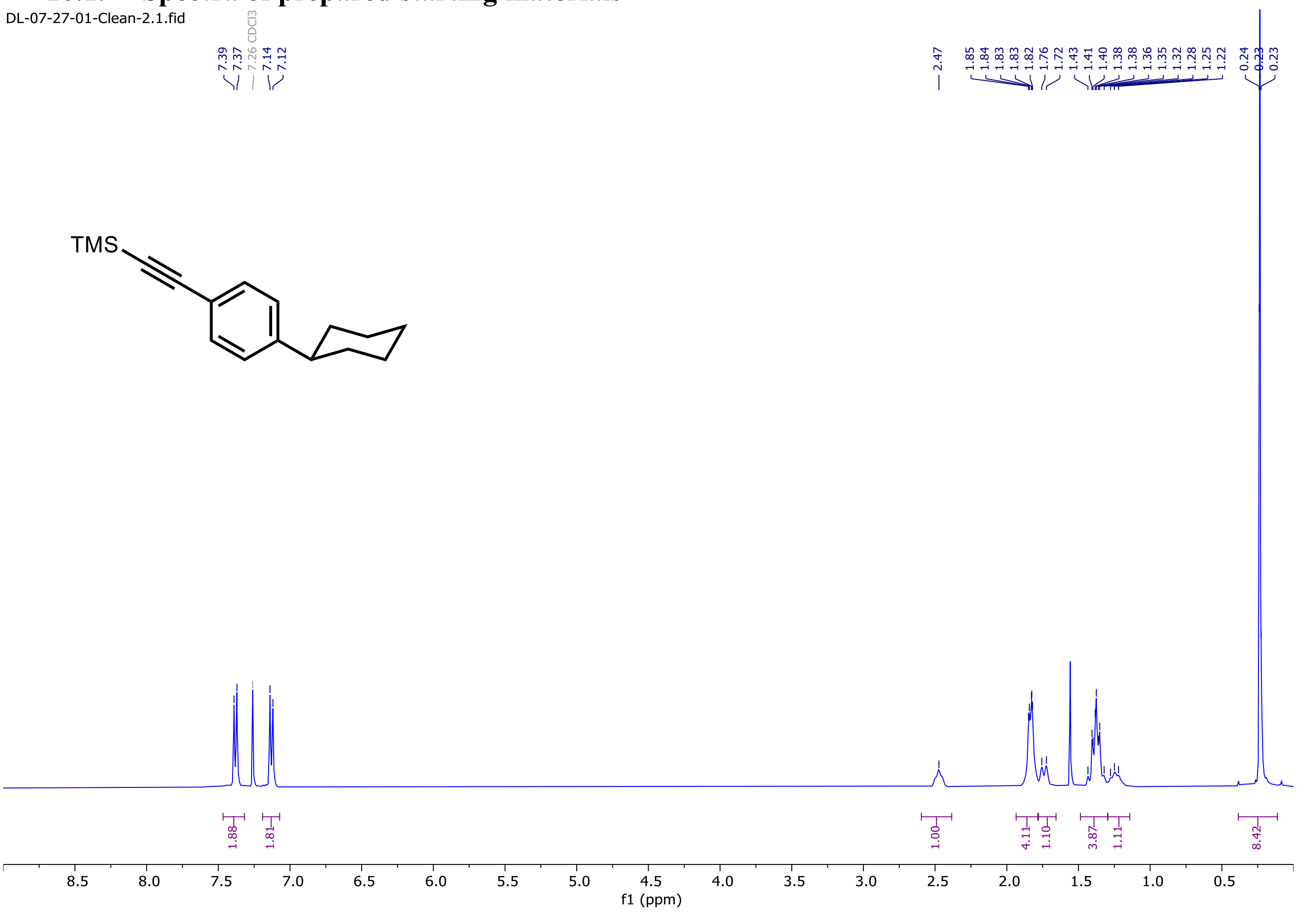

Figure S129. ^{1}H-NMR of SI-13

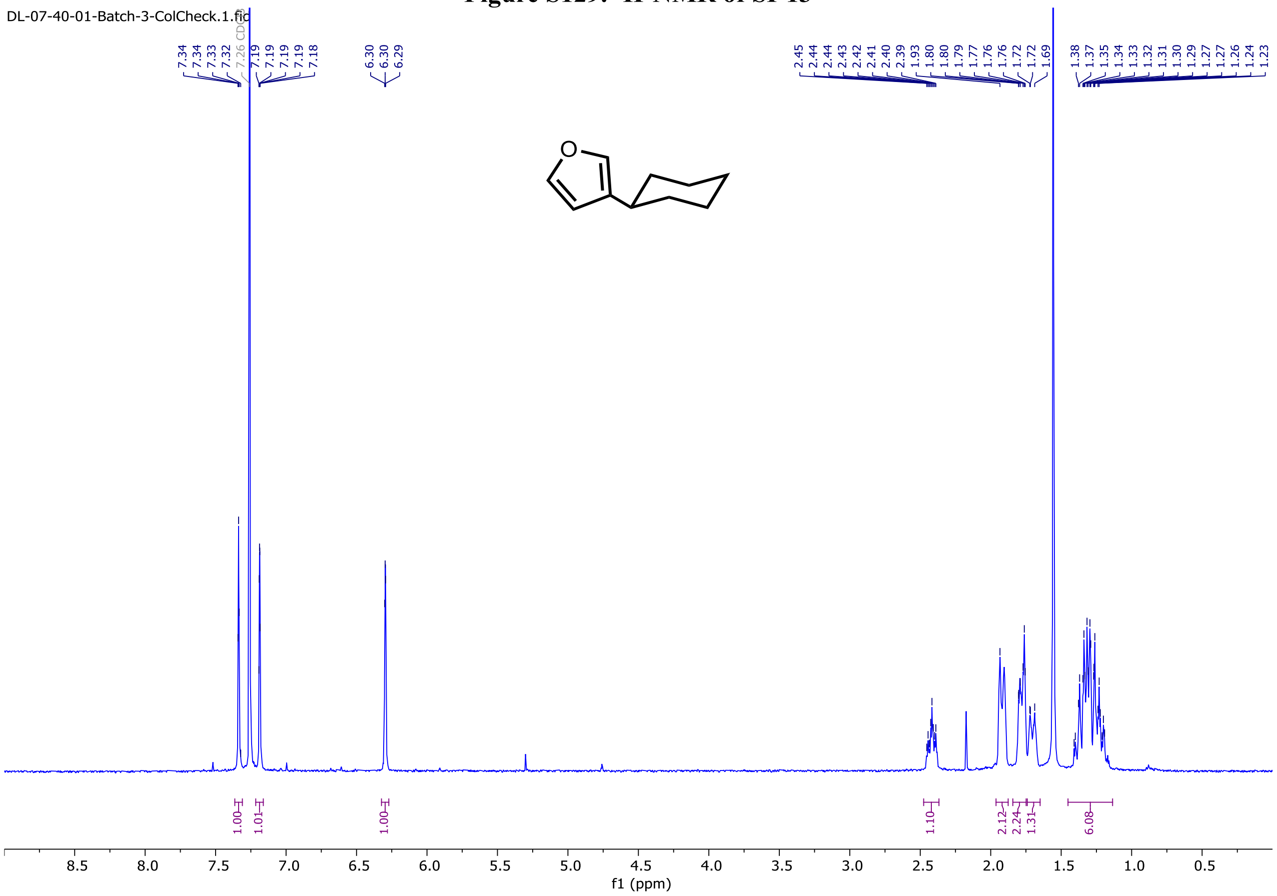

Figure S130. ^{1}H-NMR of SI-14

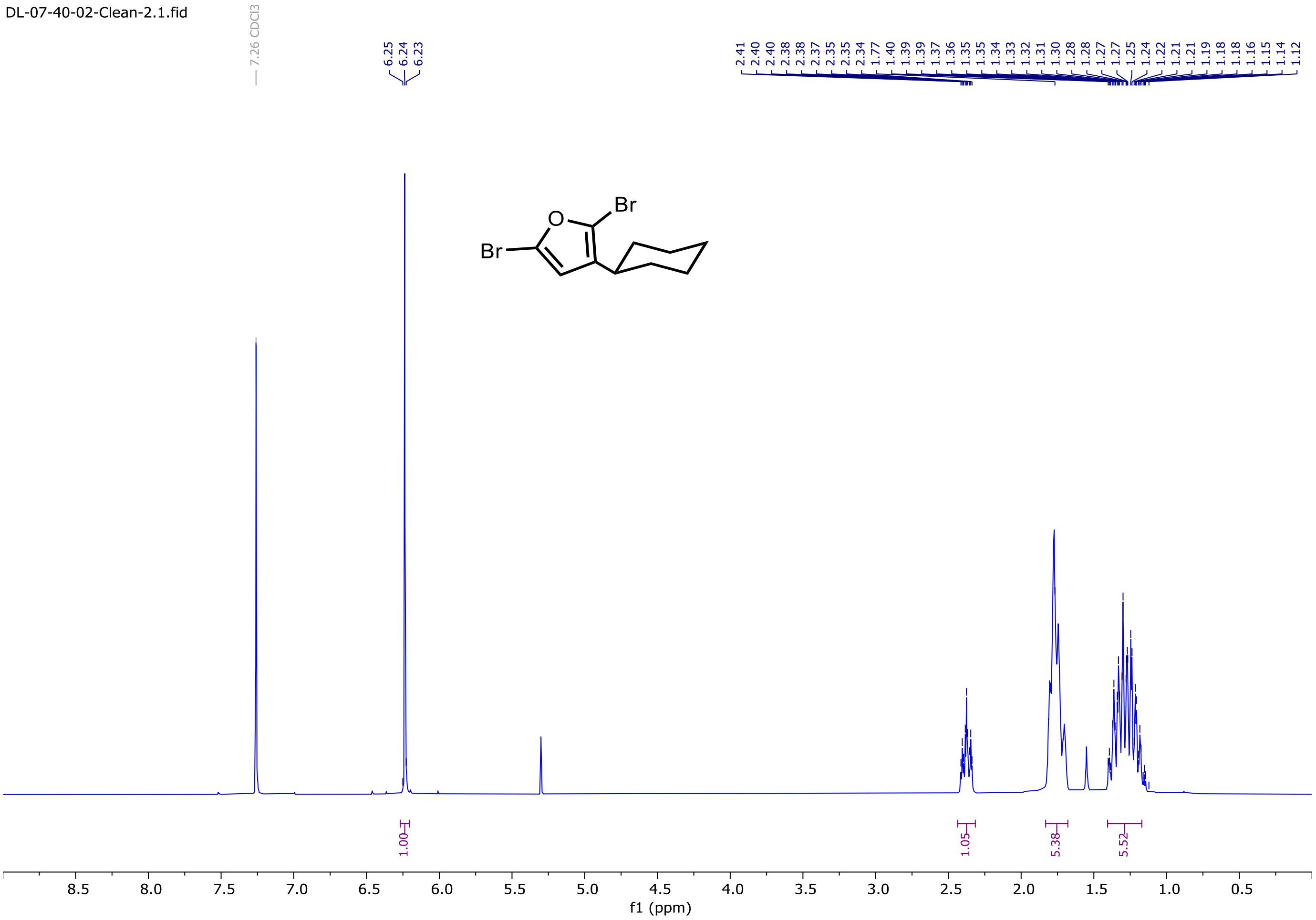

Figure S131. ^{1}H-NMR of SI-15

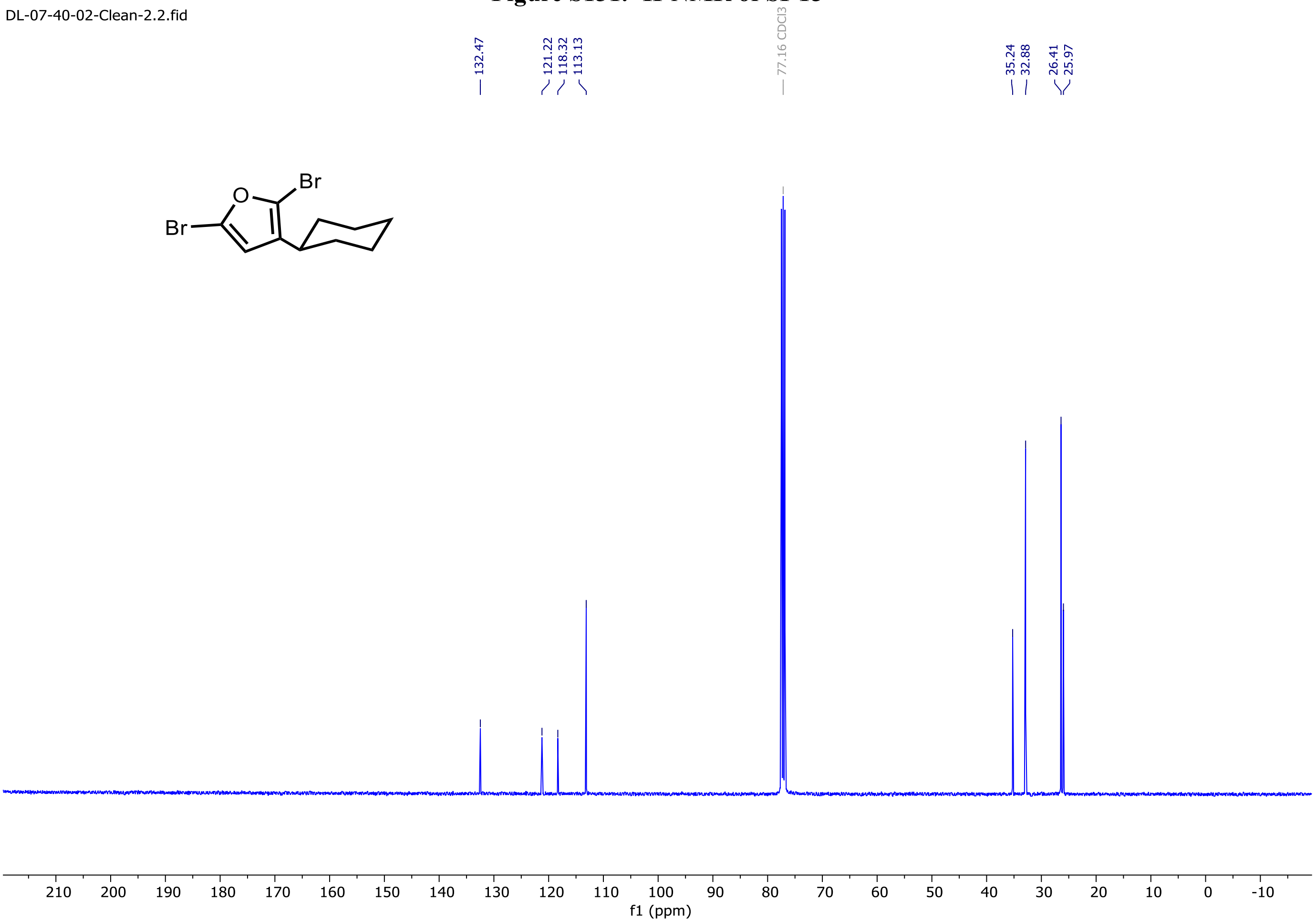

Figure S132. ^{13}C-NMR of SI-15

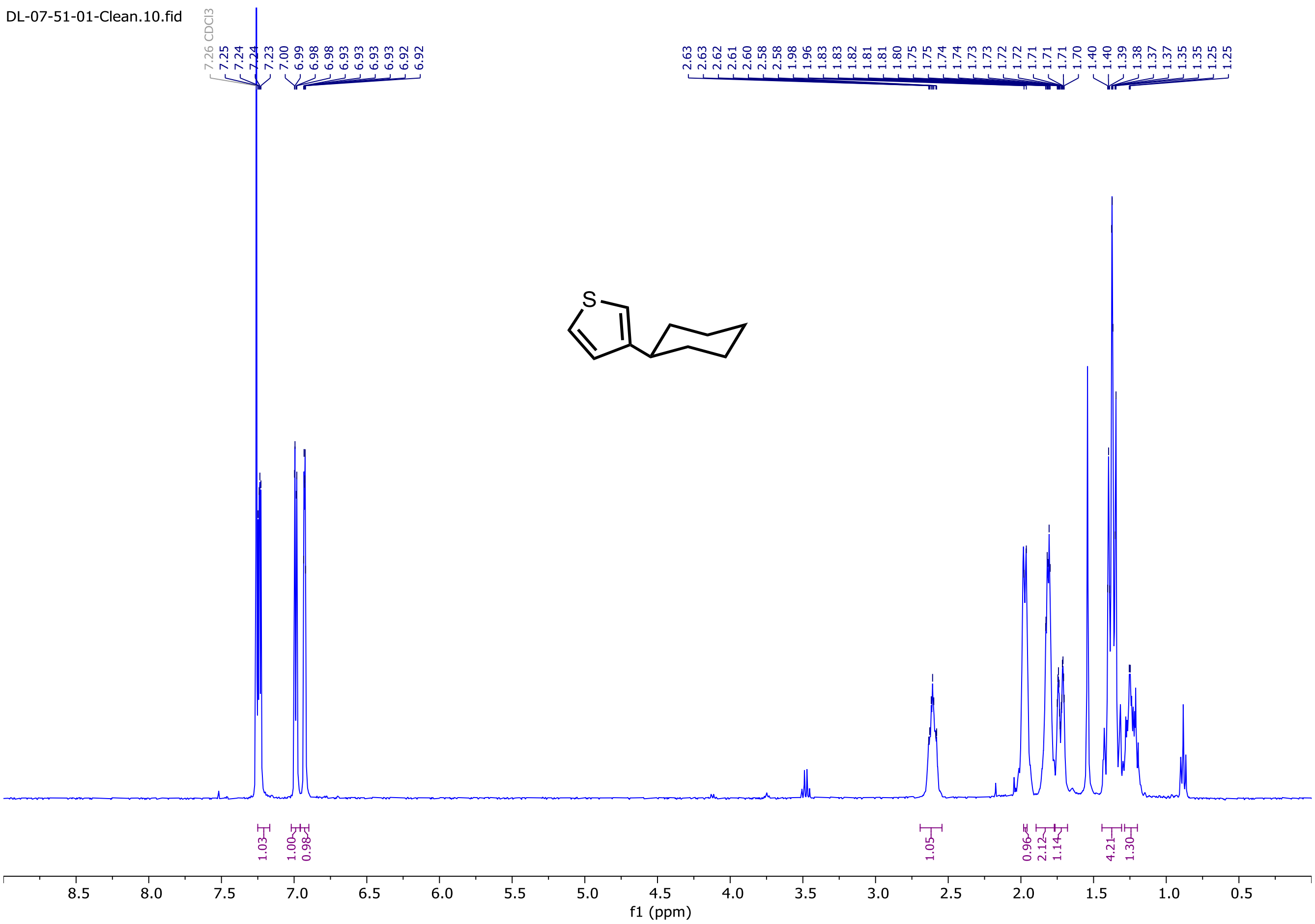

Figure S133. ^{1}H-NMR of SI-16

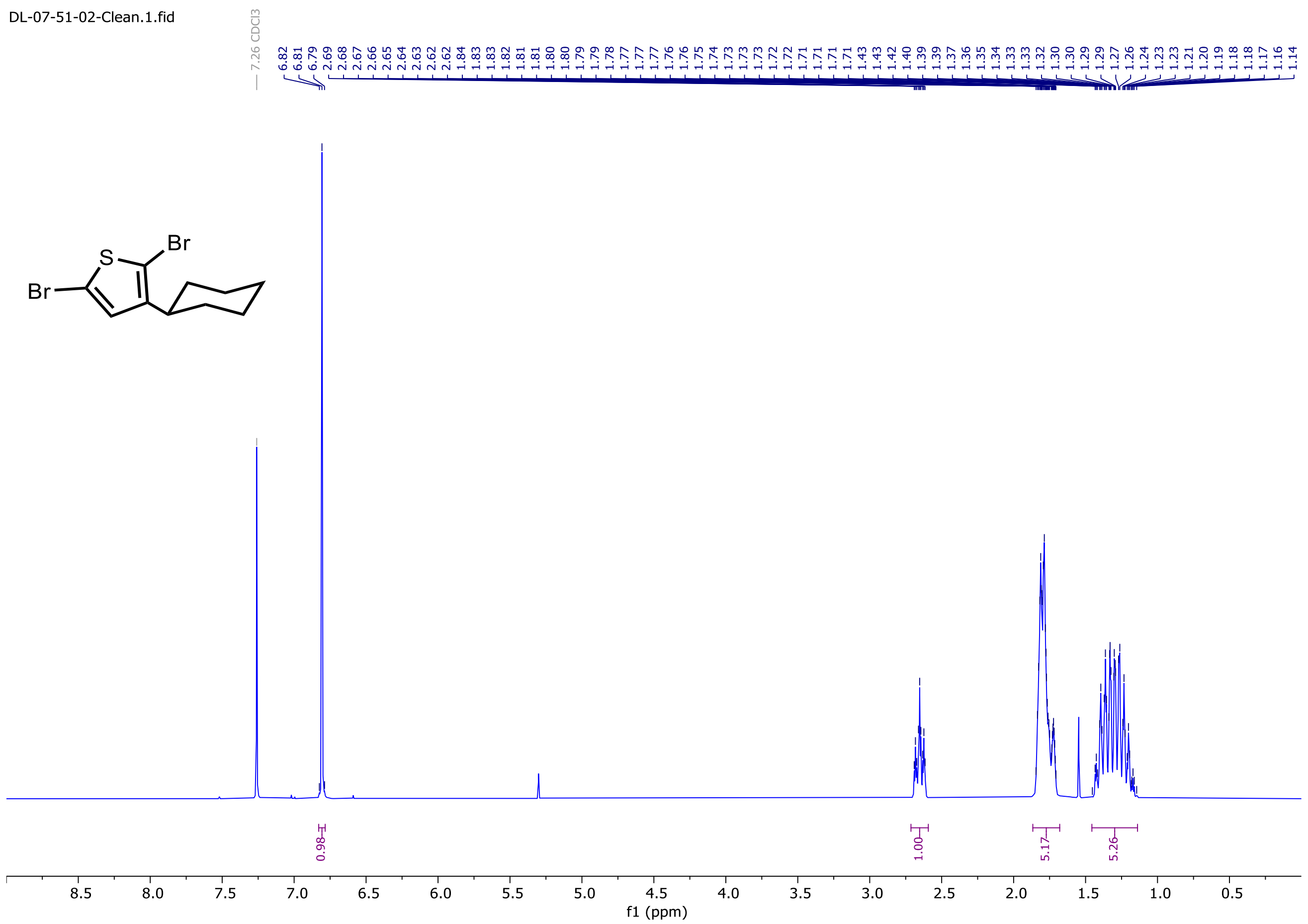

Figure S134. ^{1}H-NMR of SI-17

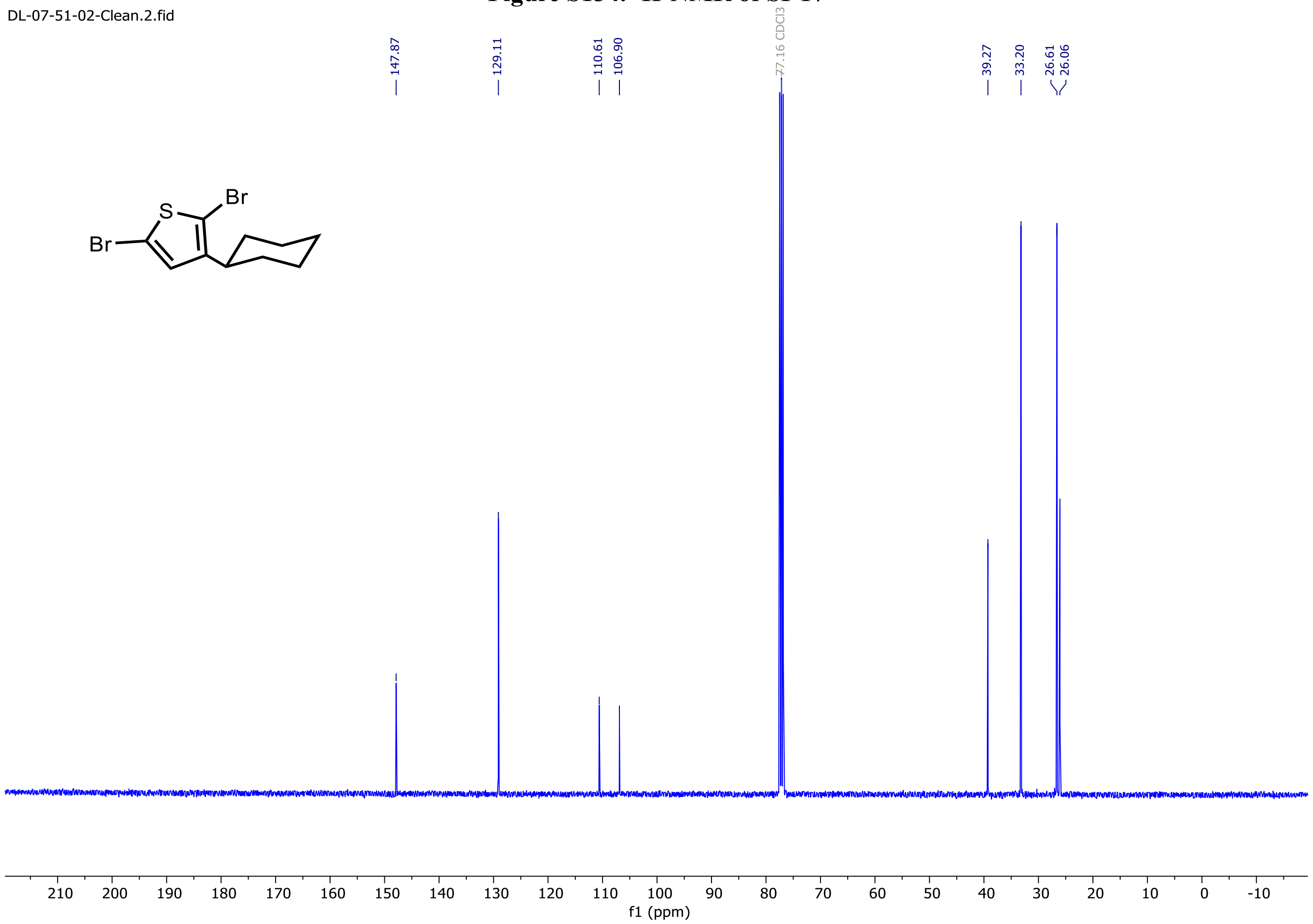

Figure S135. ^{13}C-NMR of SI-17

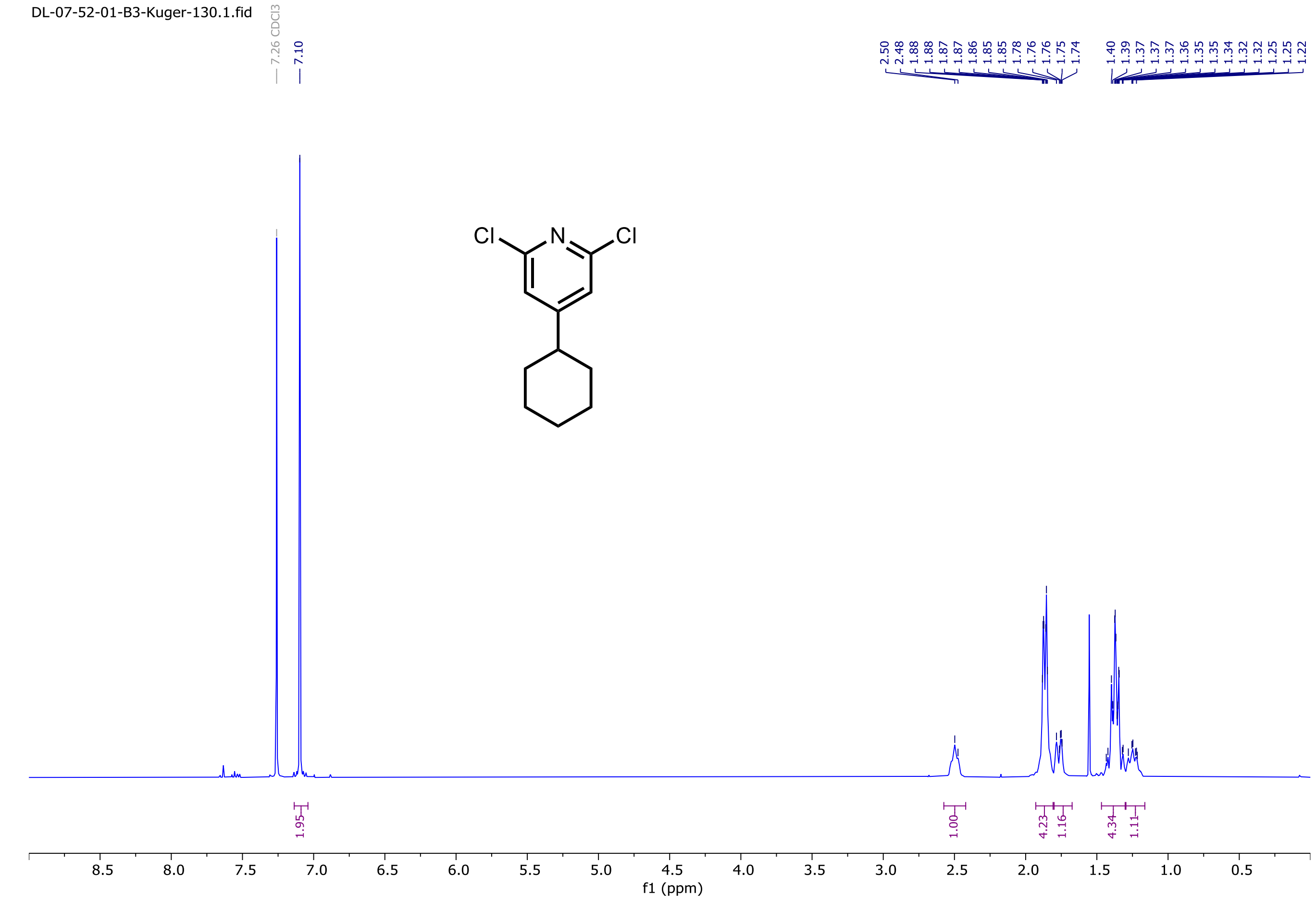

Figure S136. ^{1}H-NMR of SI-18

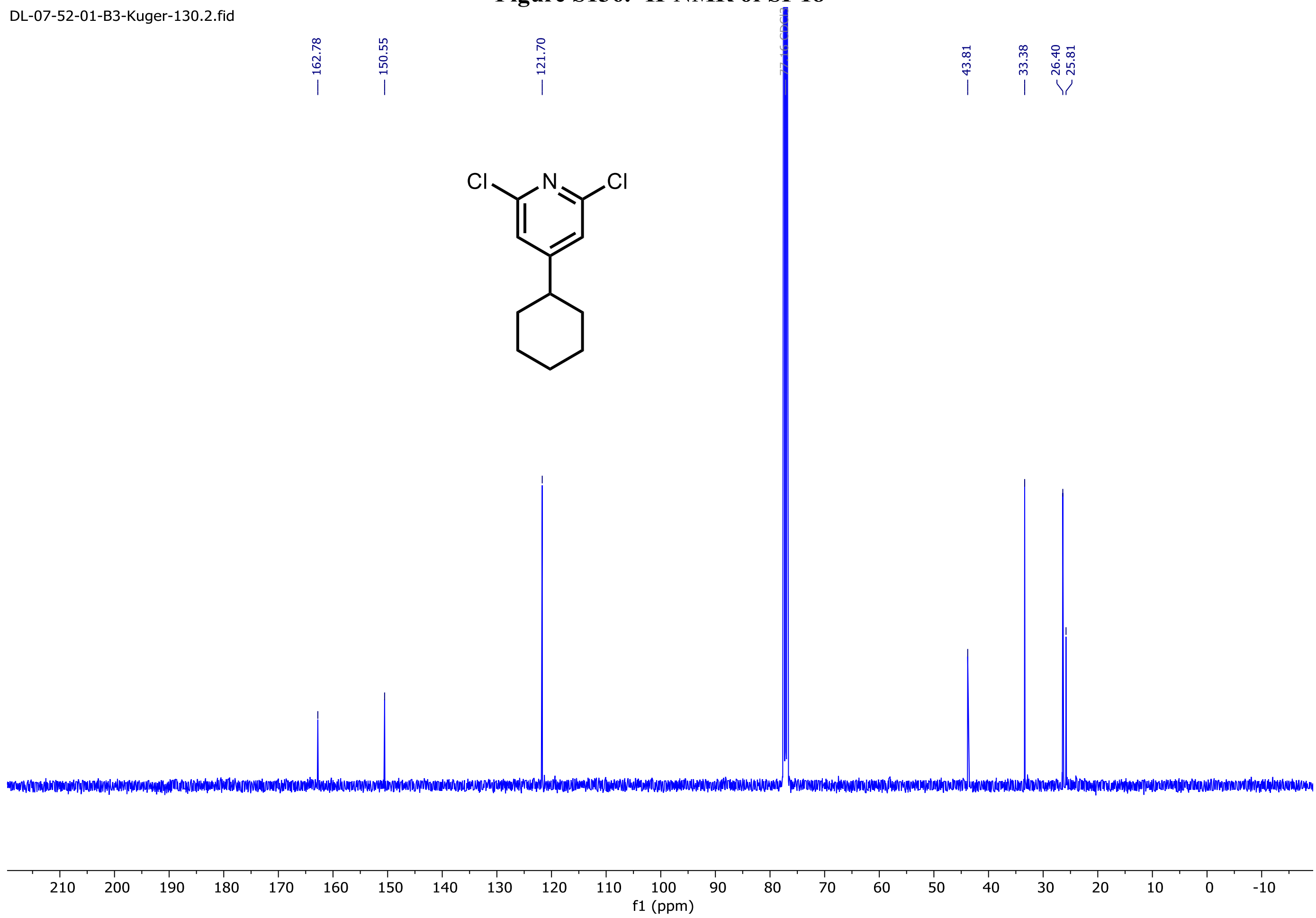

Figure S137. ^{13}C-NMR of SI-18

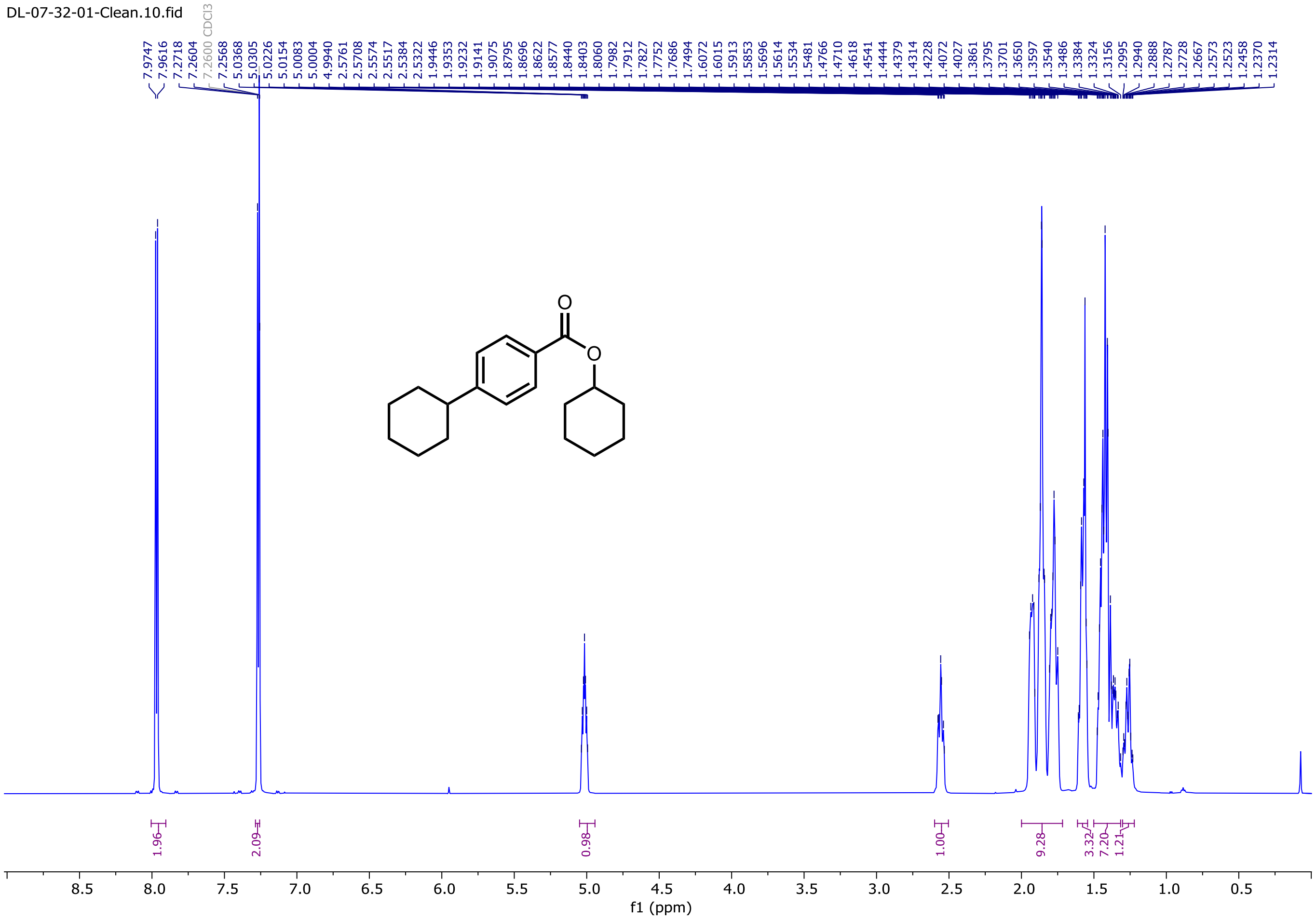

Figure S138. ^{1}H-NMR of SI-19

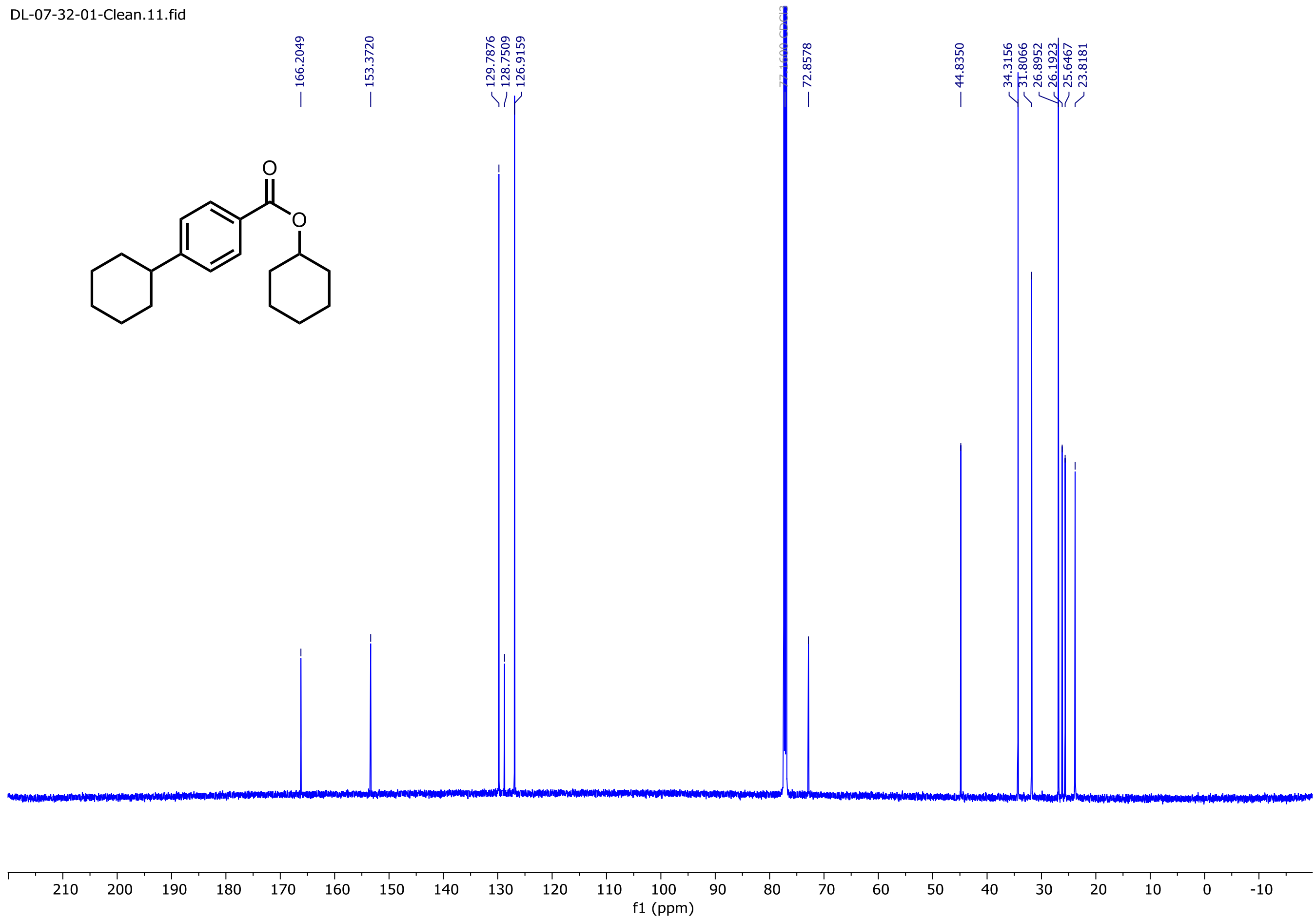

Figure S139. ^{13}C-NMR of SI-19

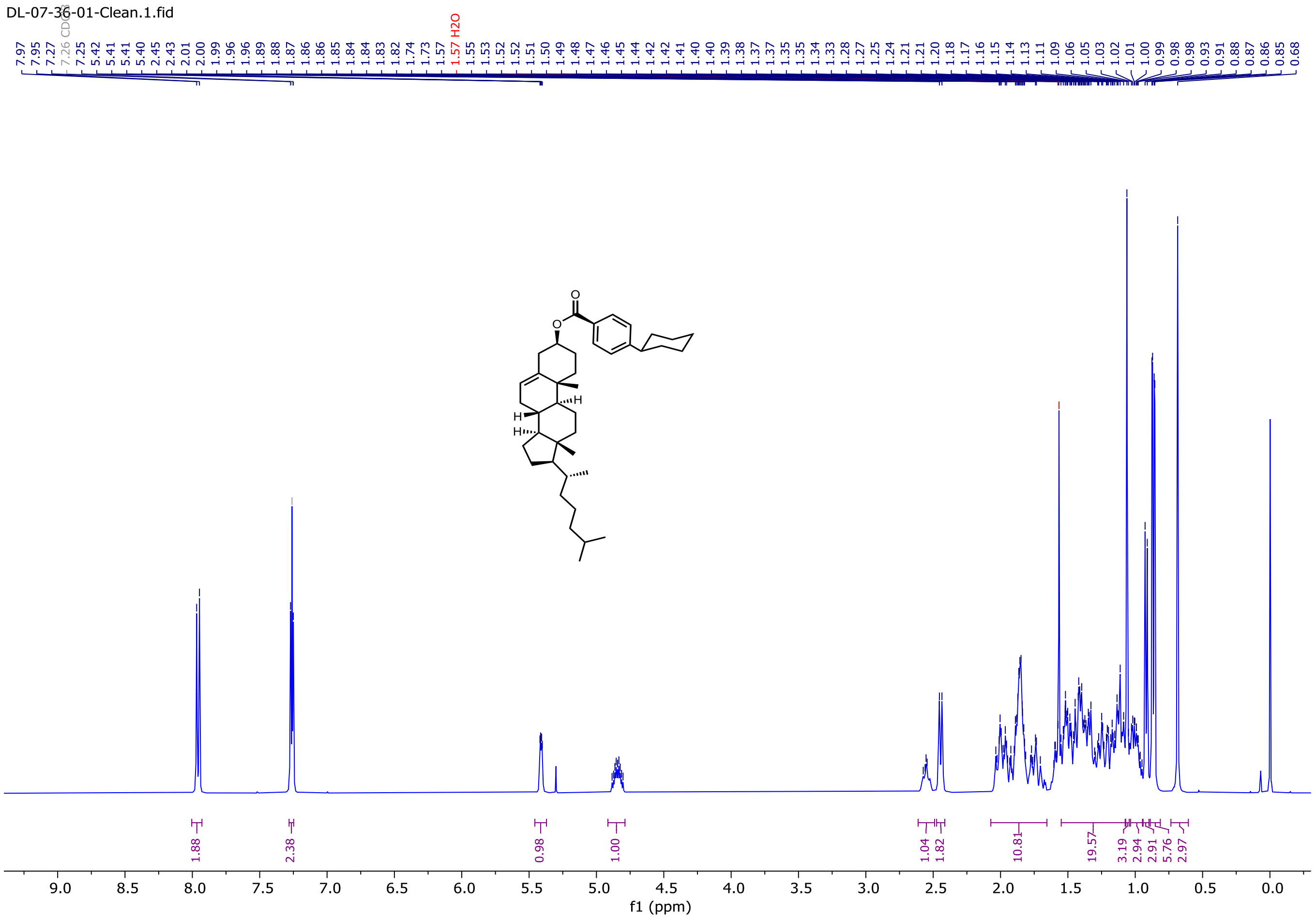

Figure S140. ^{1}**H-NMR of SI-20**

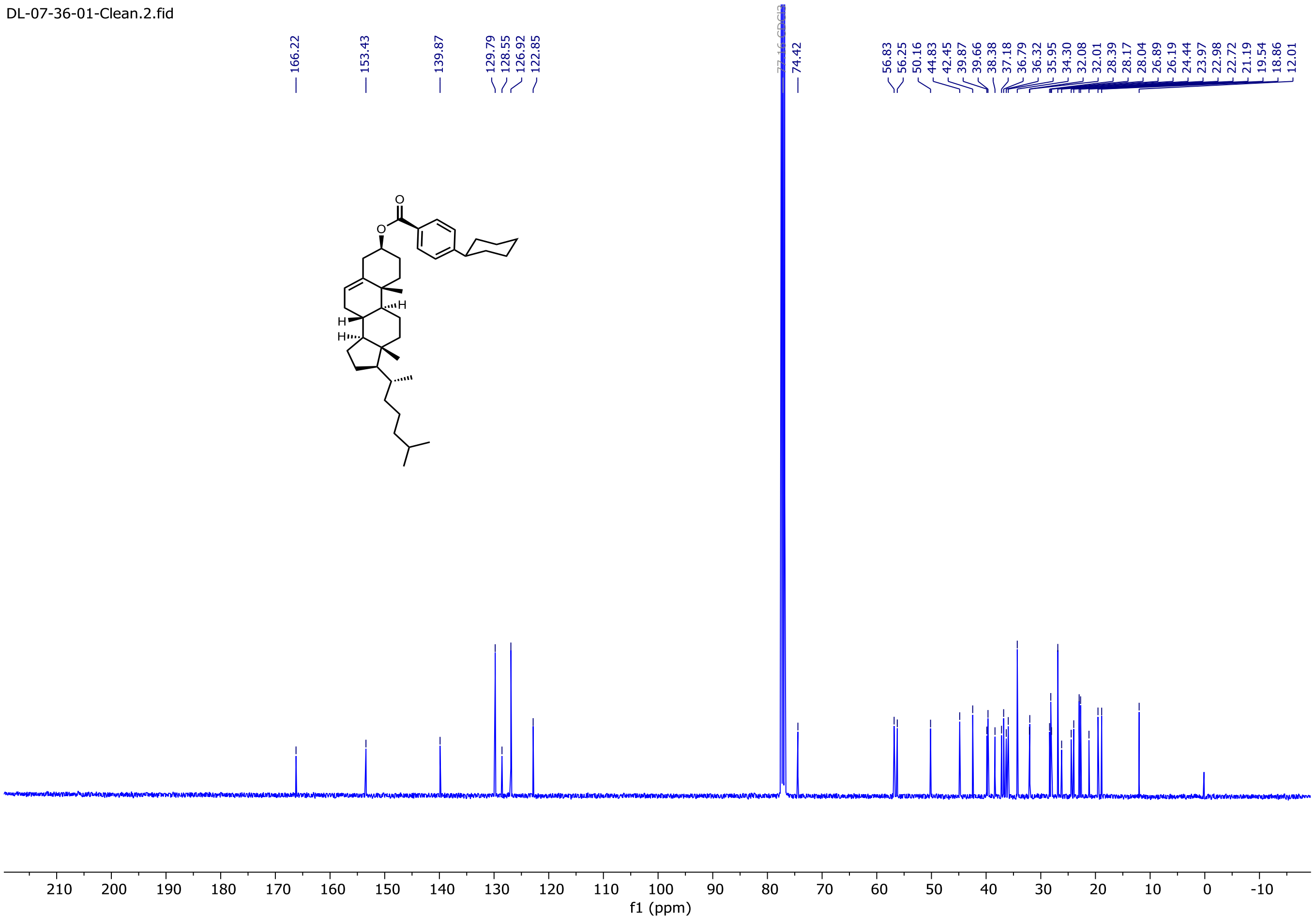

Figure S141. ^{13}**C-NMR of SI-20**

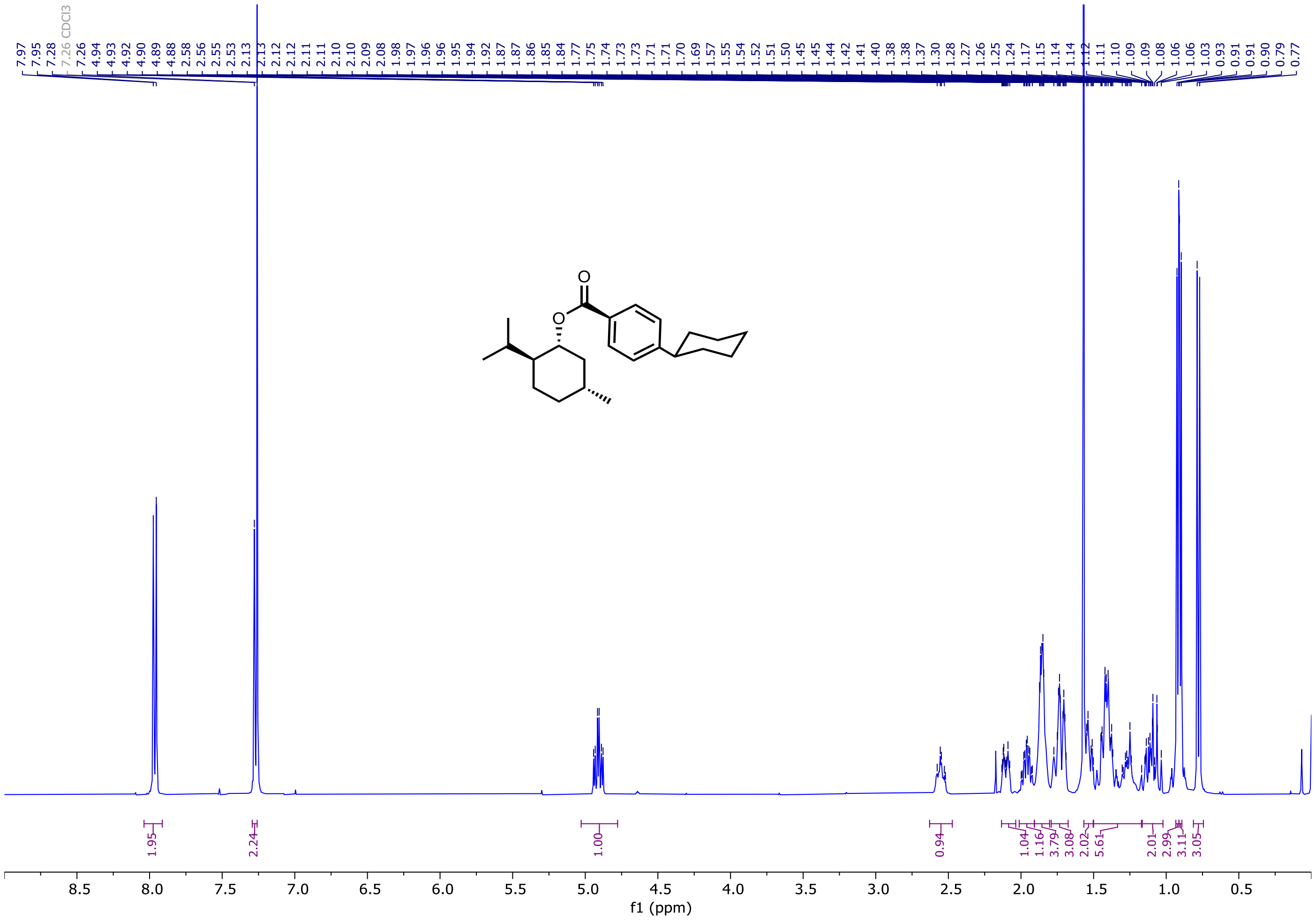

Figure S142. ^{1}H-NMR of SI-21

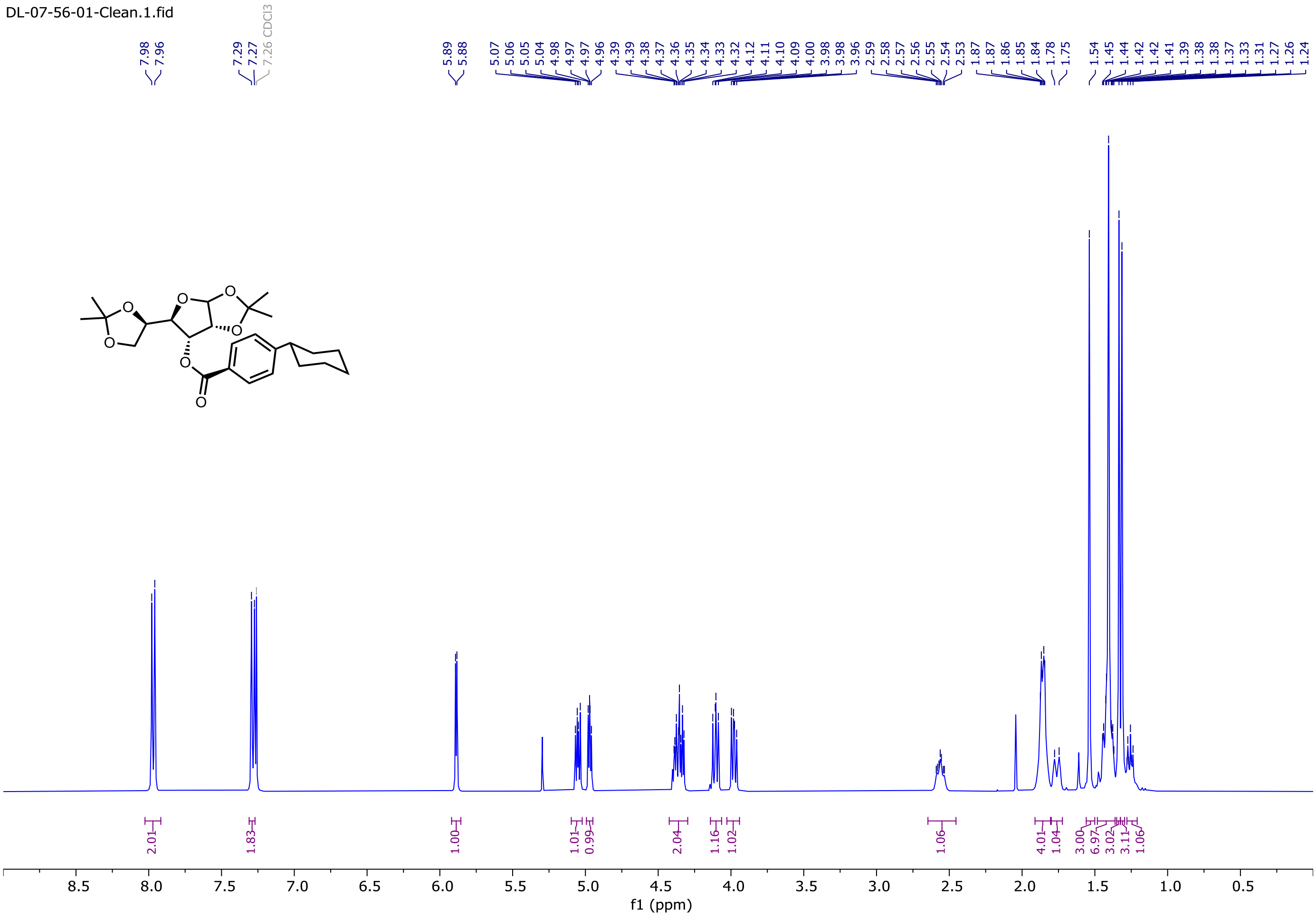

Figure S143. ^{1}H-NMR of SI-22

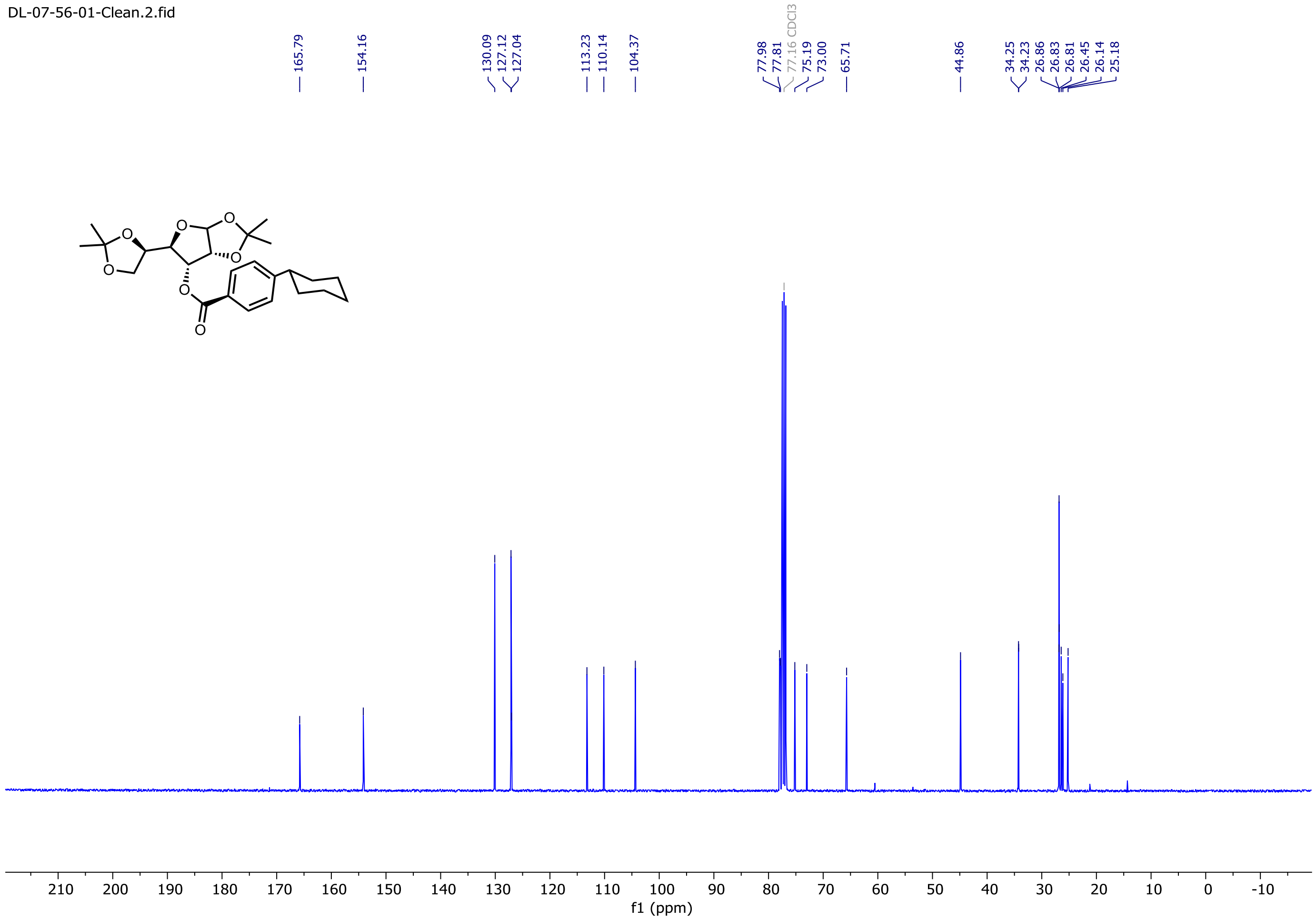

Figure S144. ^{13}C-NMR of SI-22

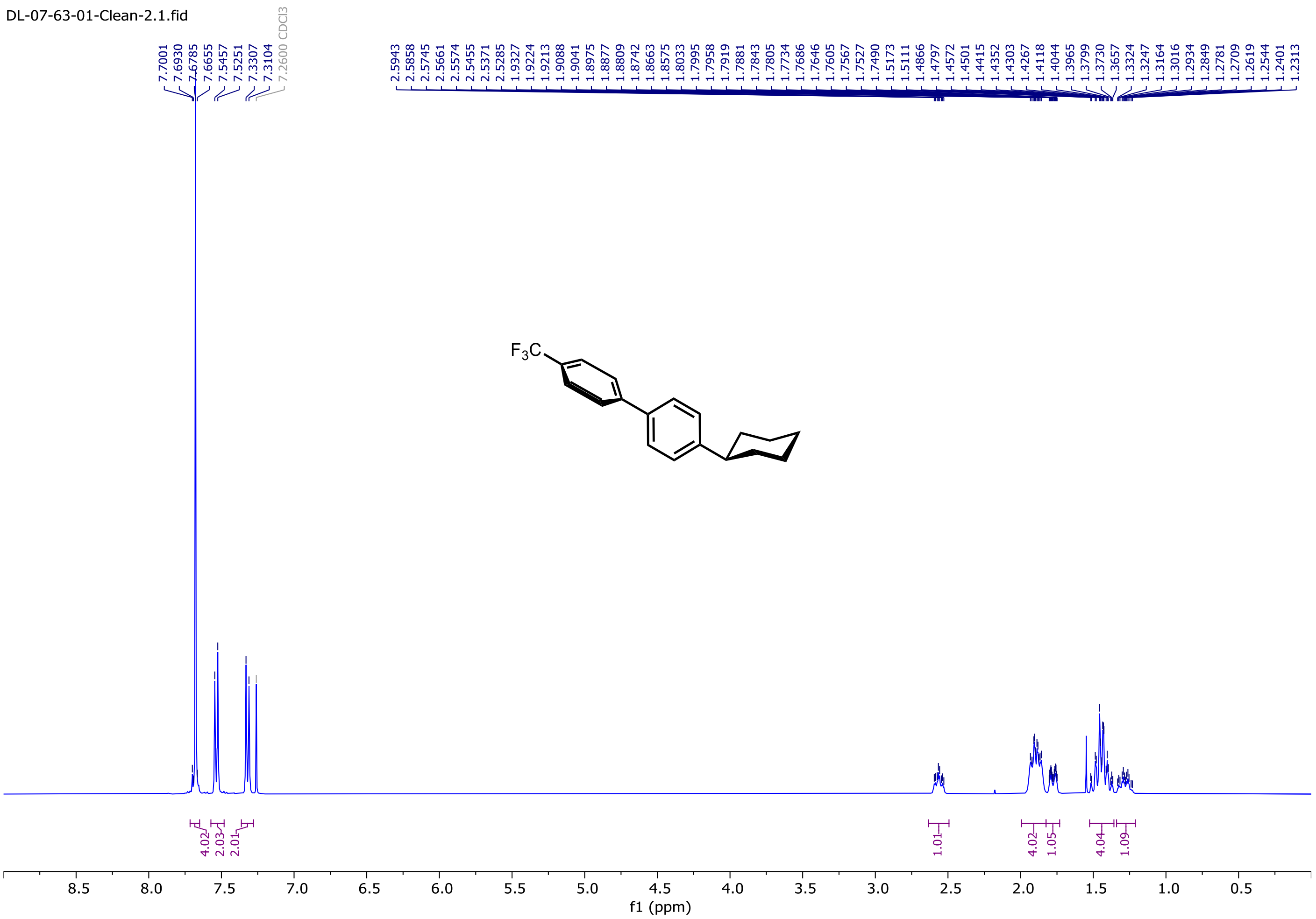

Figure S145. ^{1}H-NMR of SI-23

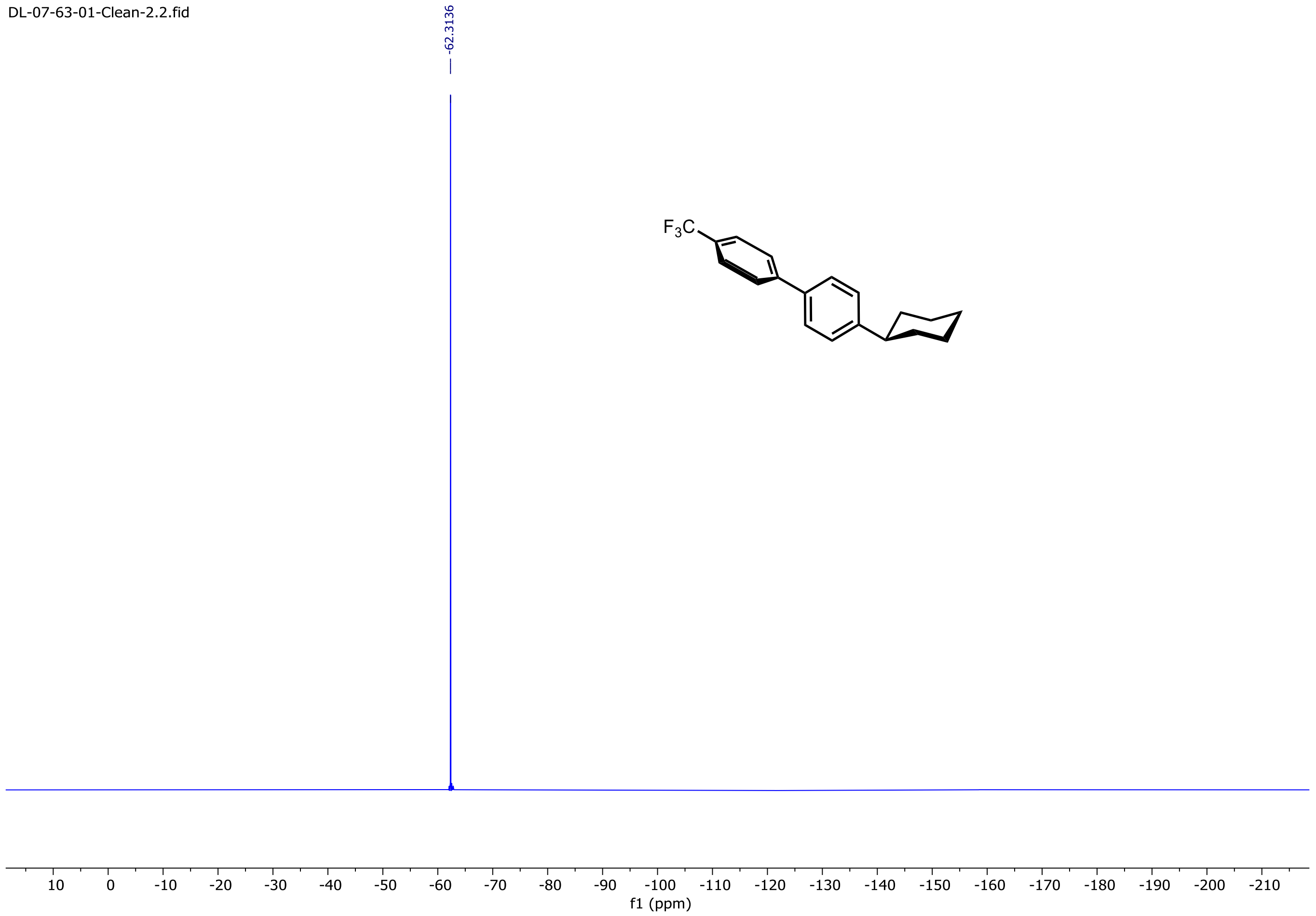

Figure S146. ^{19}F-NMR of SI-23

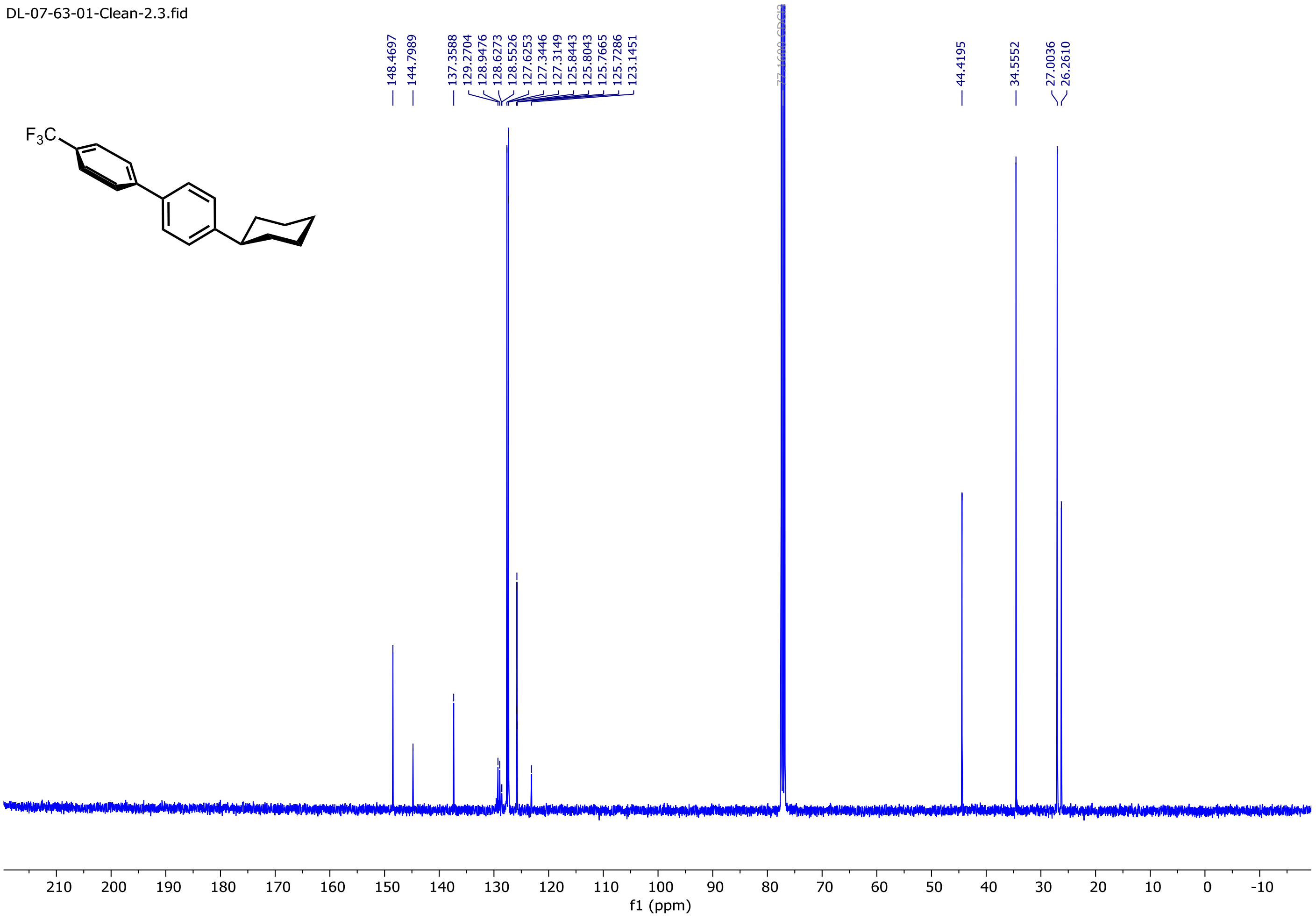

Figure S147. ^{13}C-NMR of SI-23

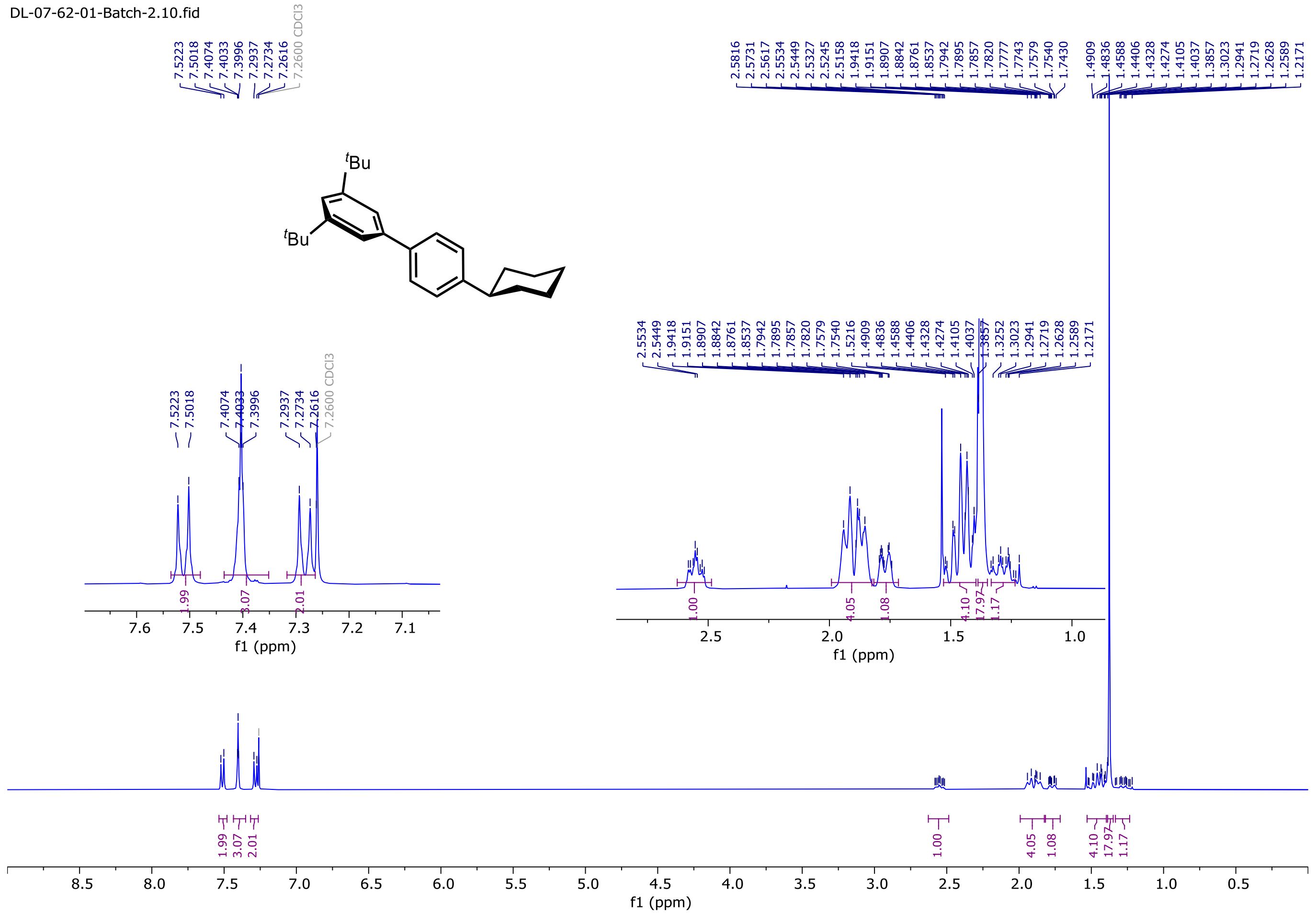

Figure S148. ^{1}H-NMR of SI-24

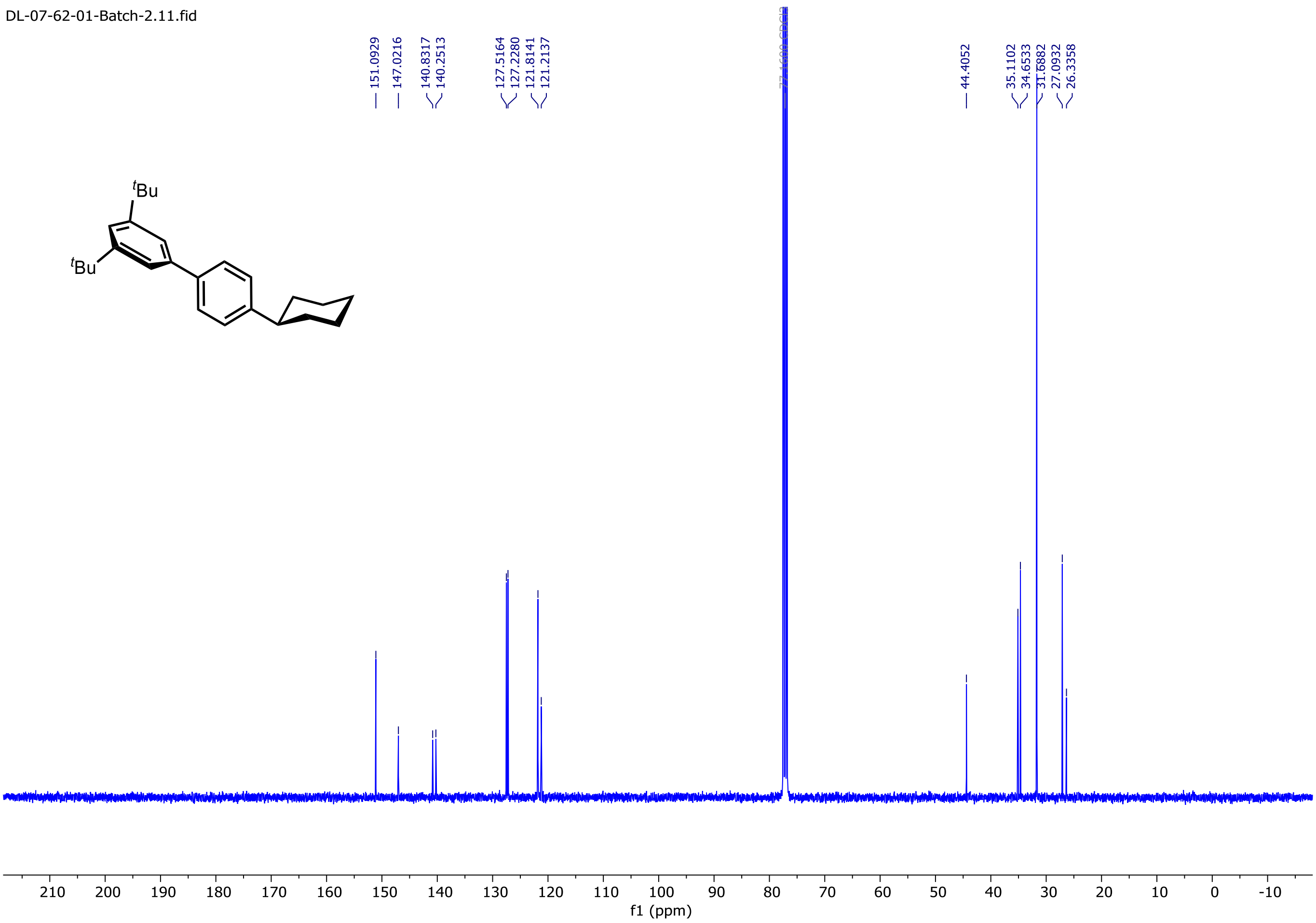

Figure S149. ^{13}C-NMR of SI-24

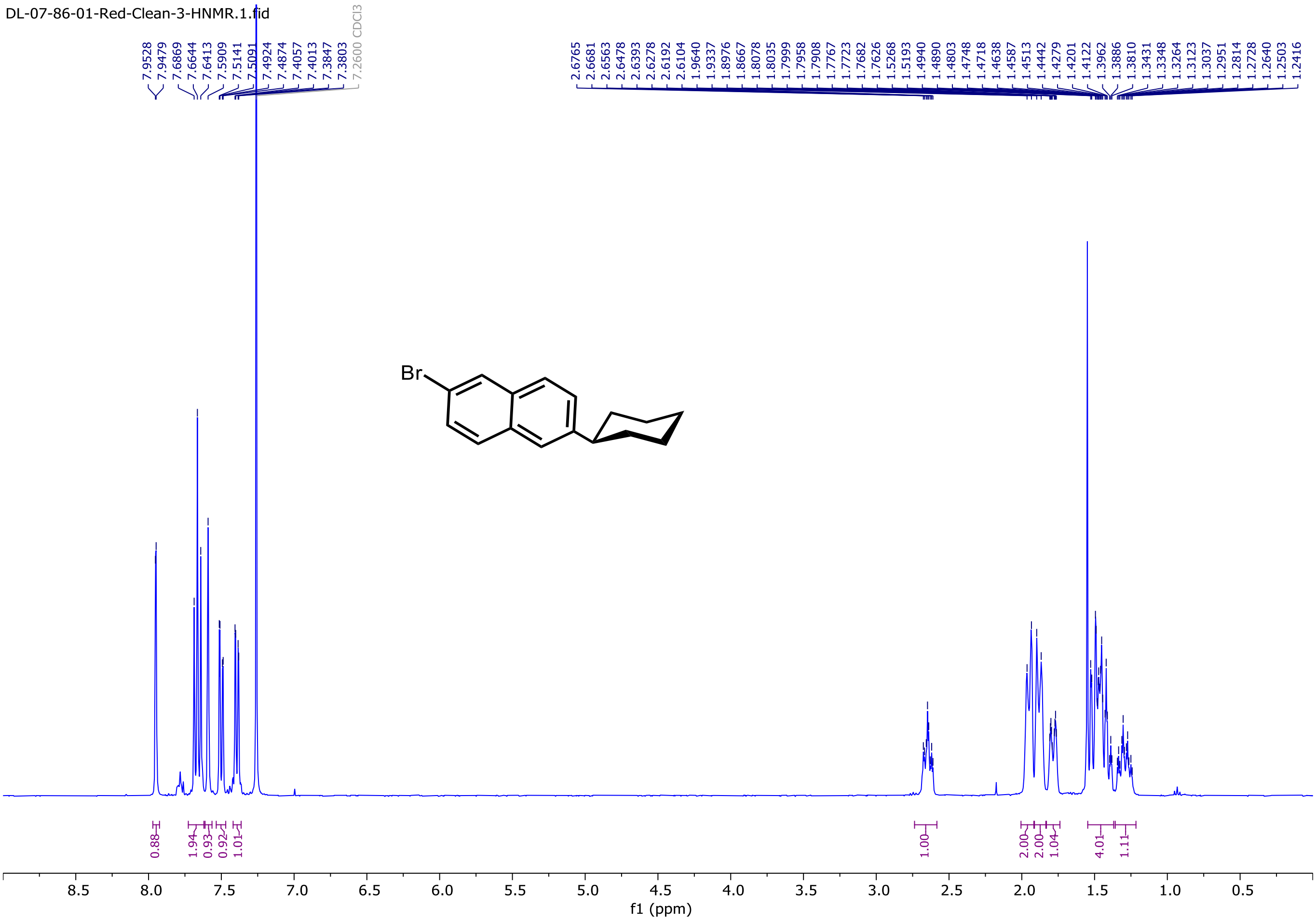

Figure S150. ^{1}H-NMR of SI-25

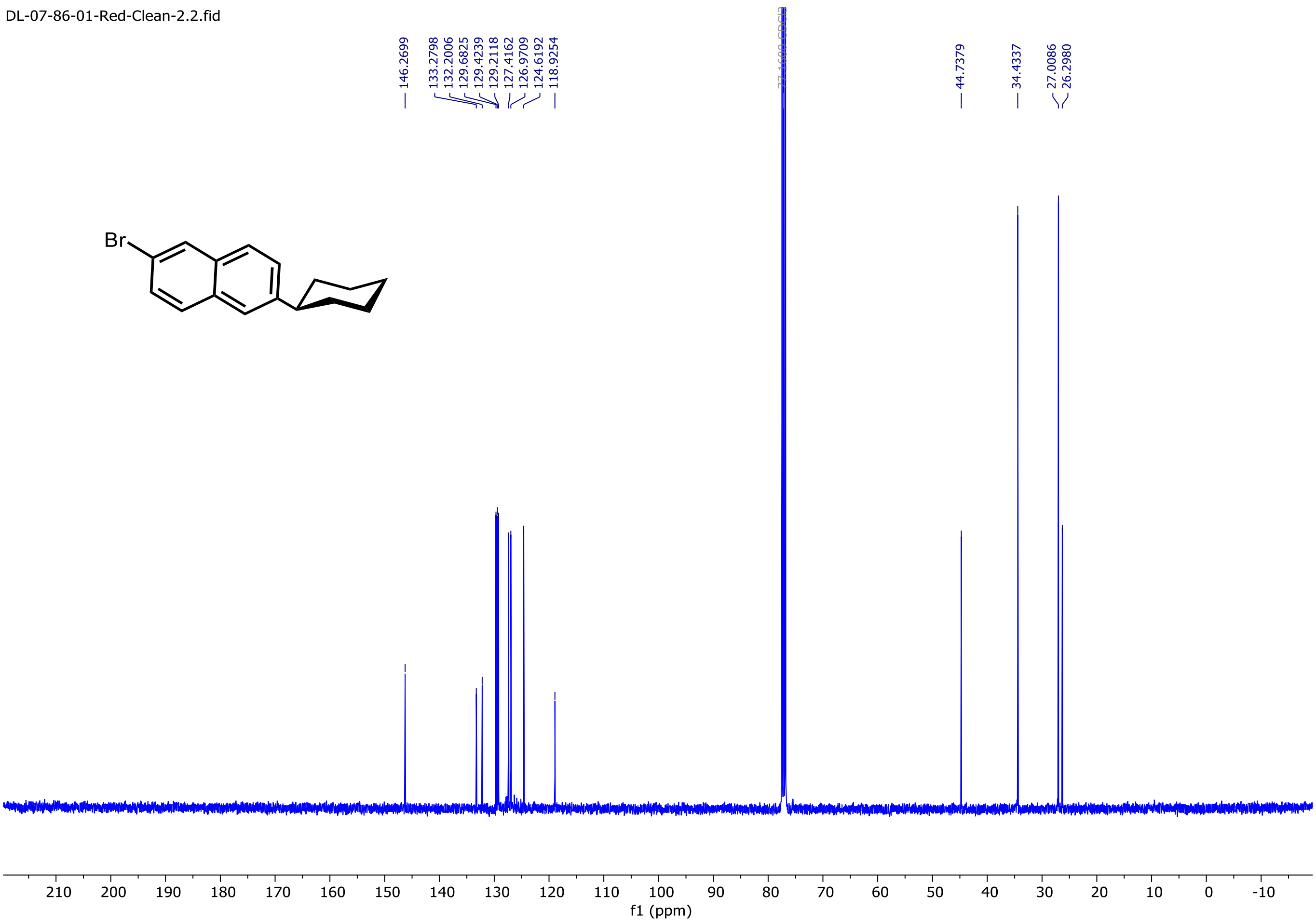

Figure S151. ^{13}C-NMR of SI-25

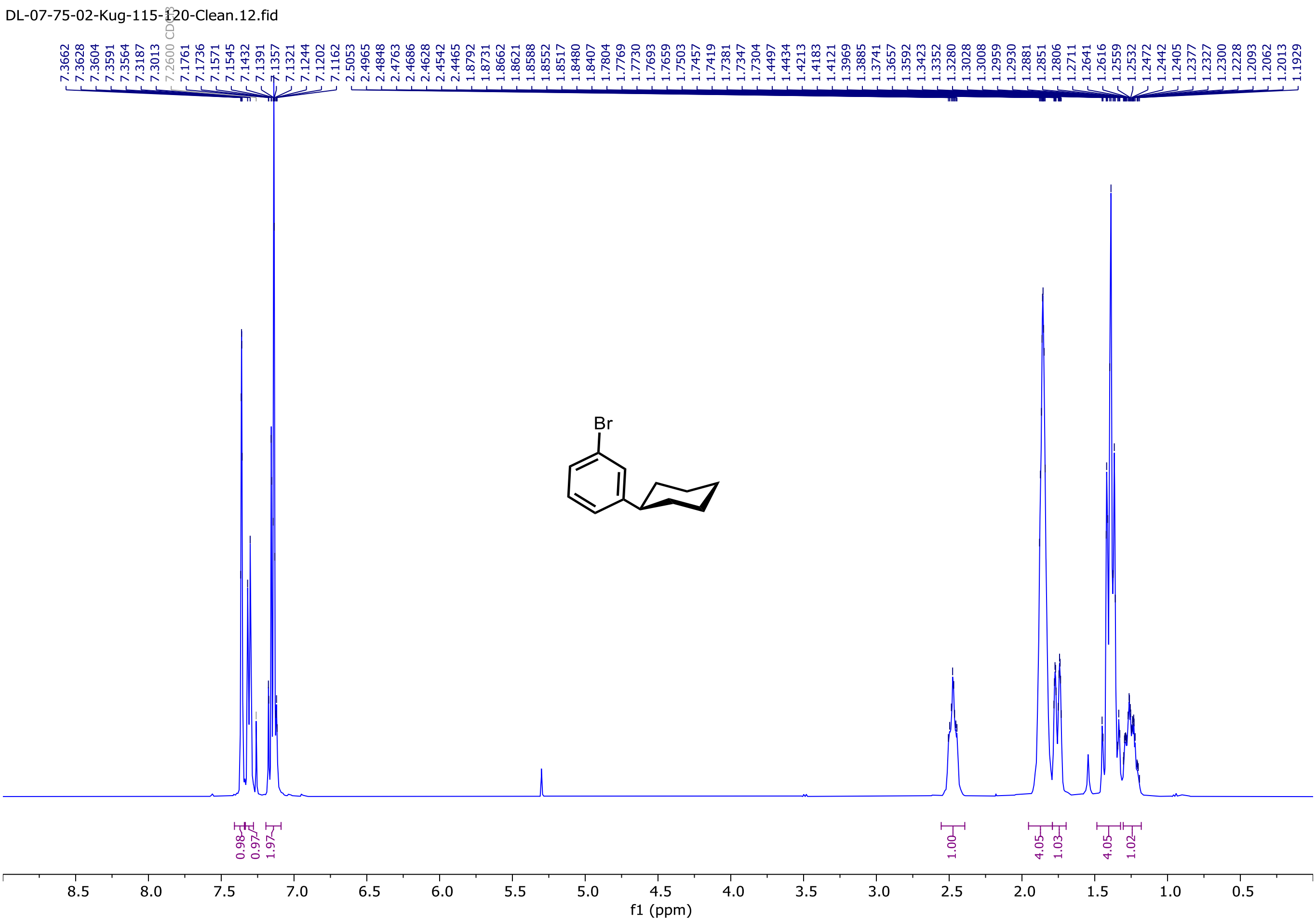

Figure S152. ¹H-NMR of SI-26

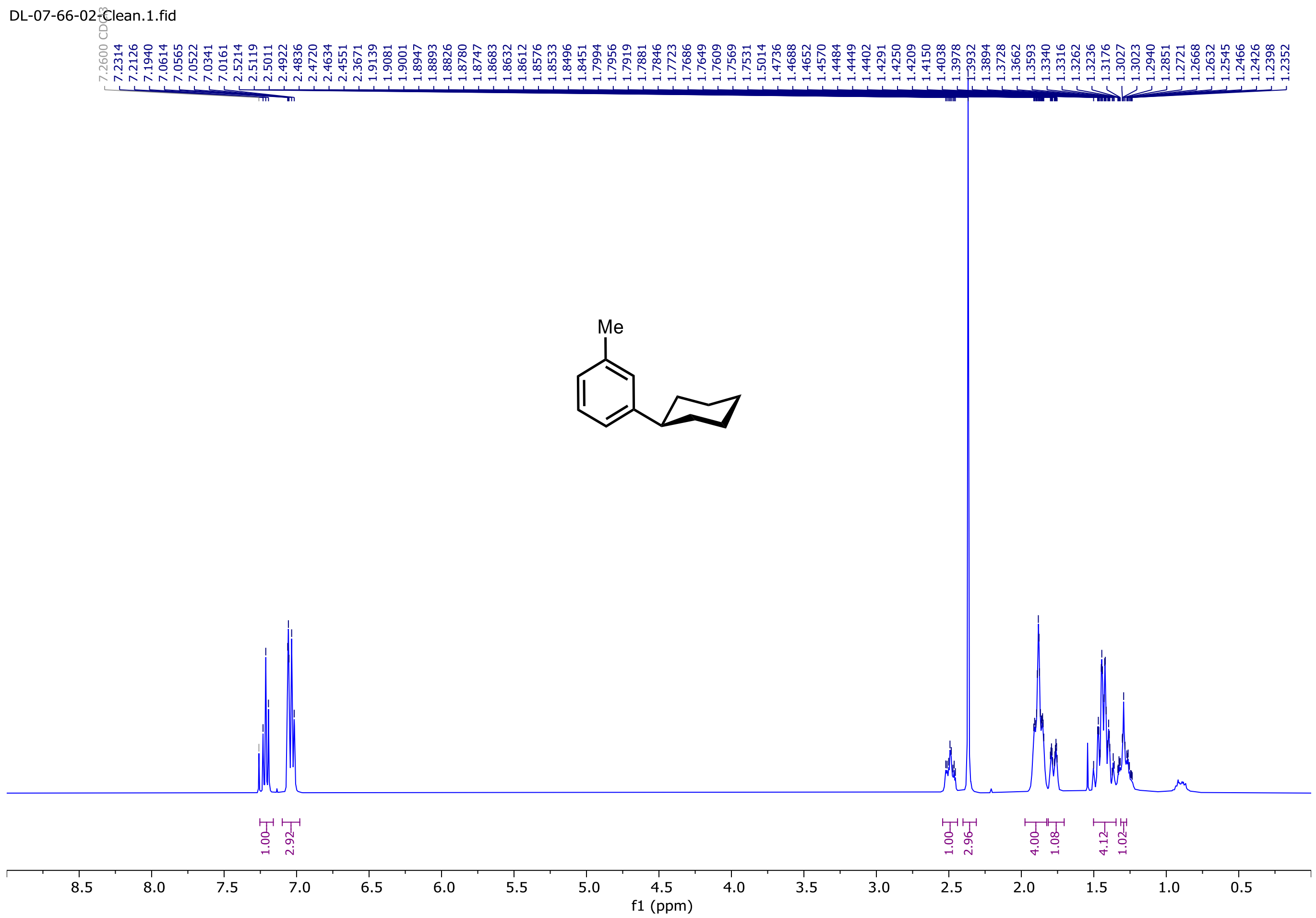

Figure S153. ¹H-NMR of SI-27

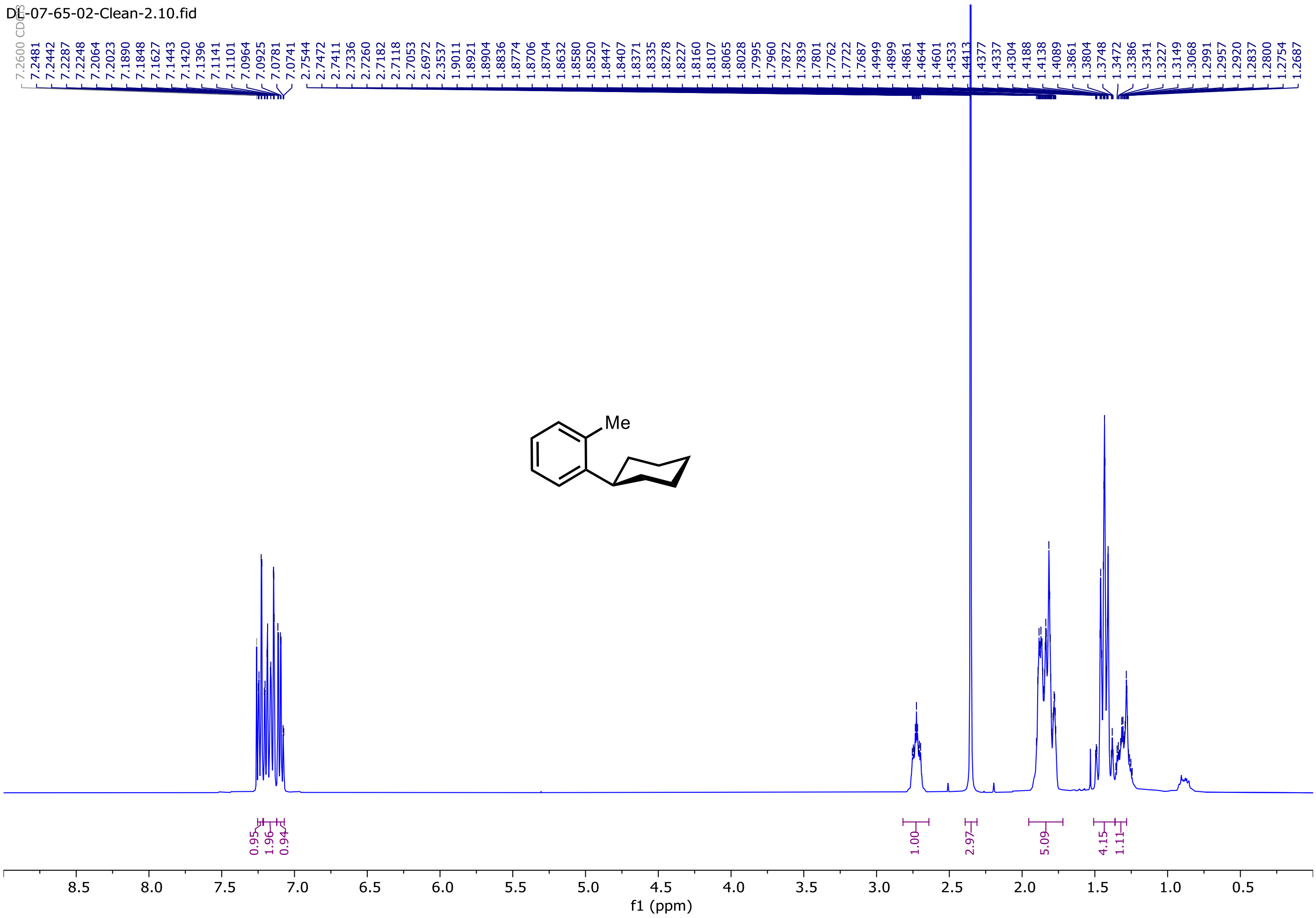

Figure S154. ^{1}H-NMR of SI-28

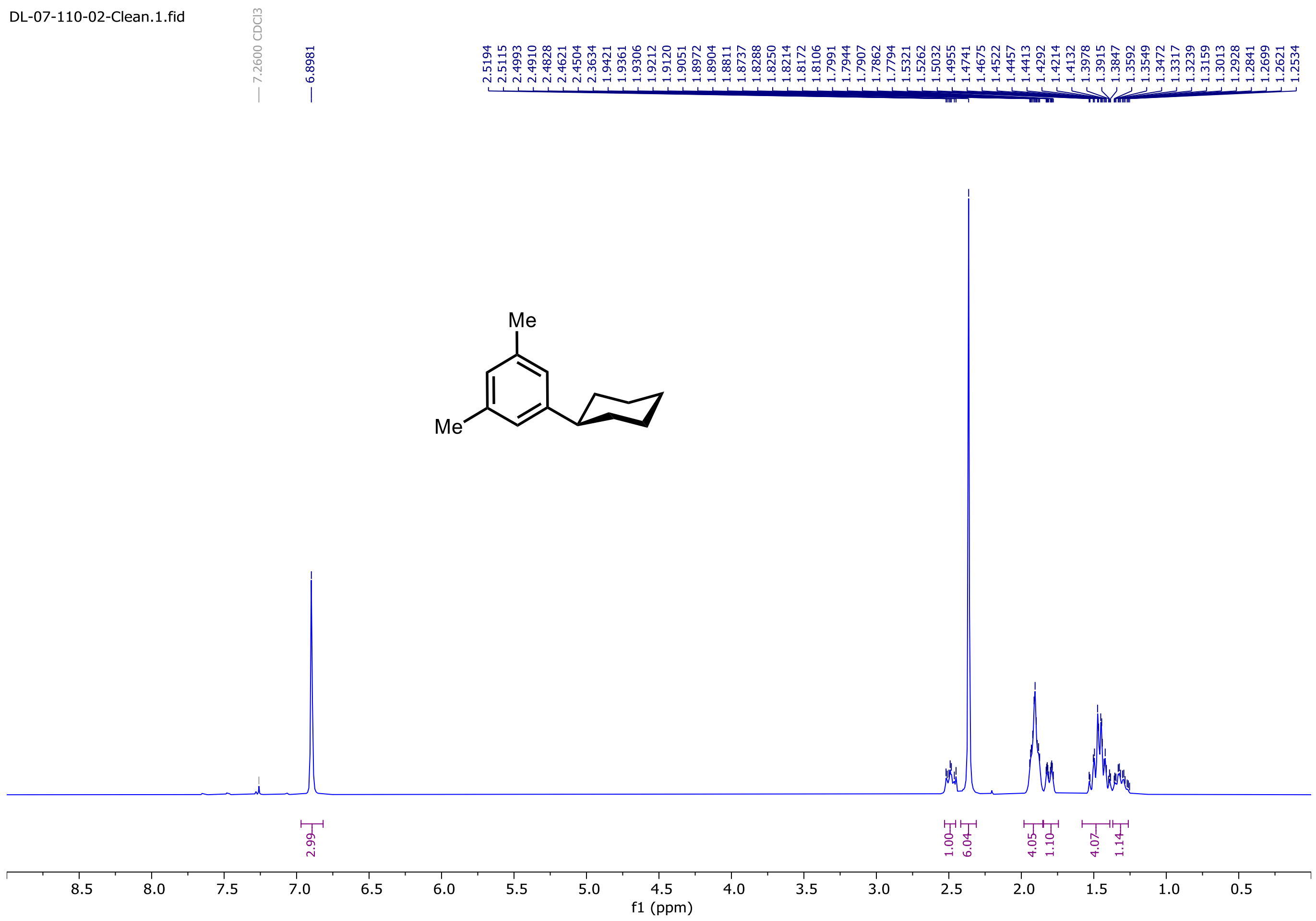

Figure S155. ¹H-NMR of SI-29

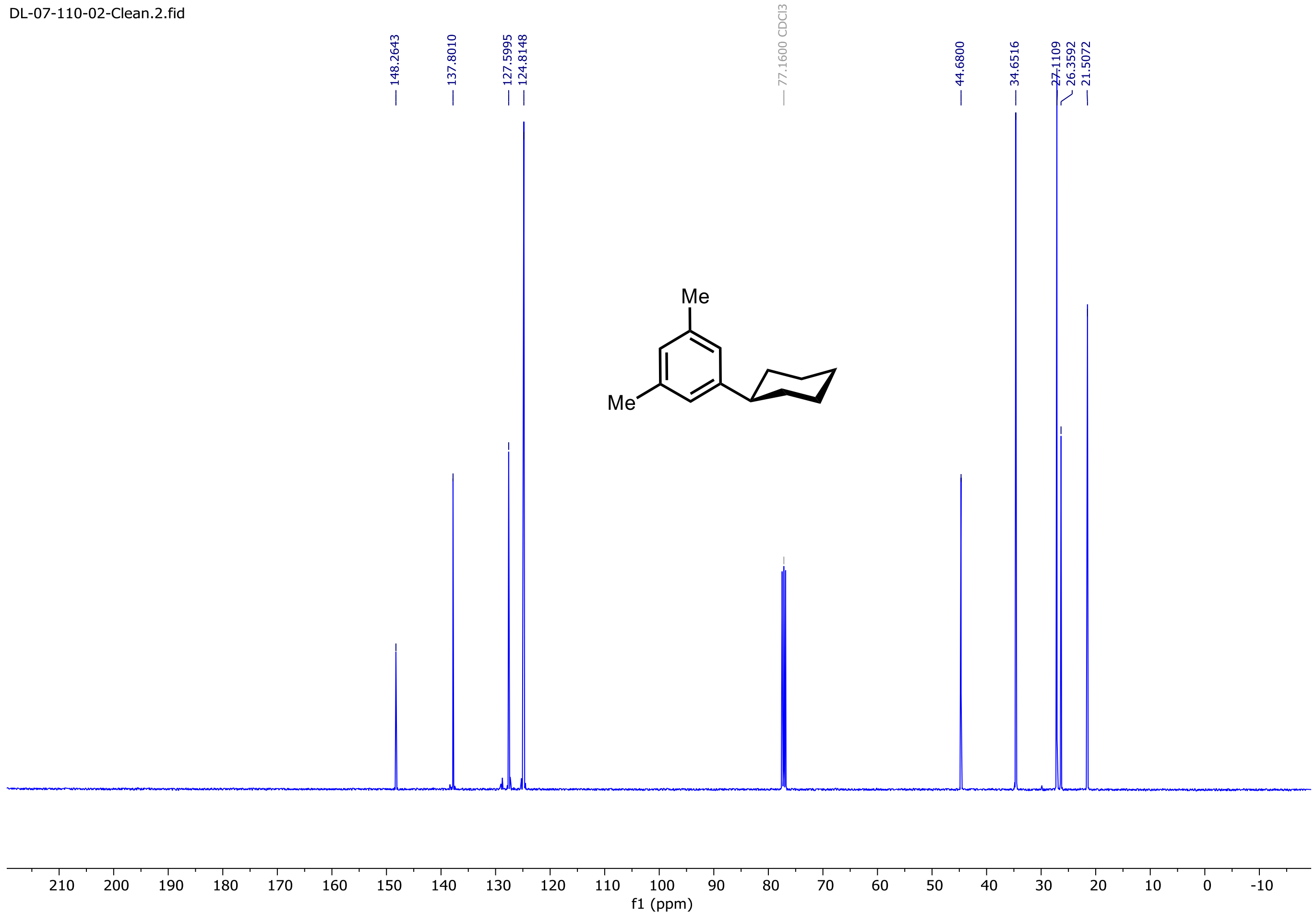

Figure S156. ¹³C-NMR of SI-29

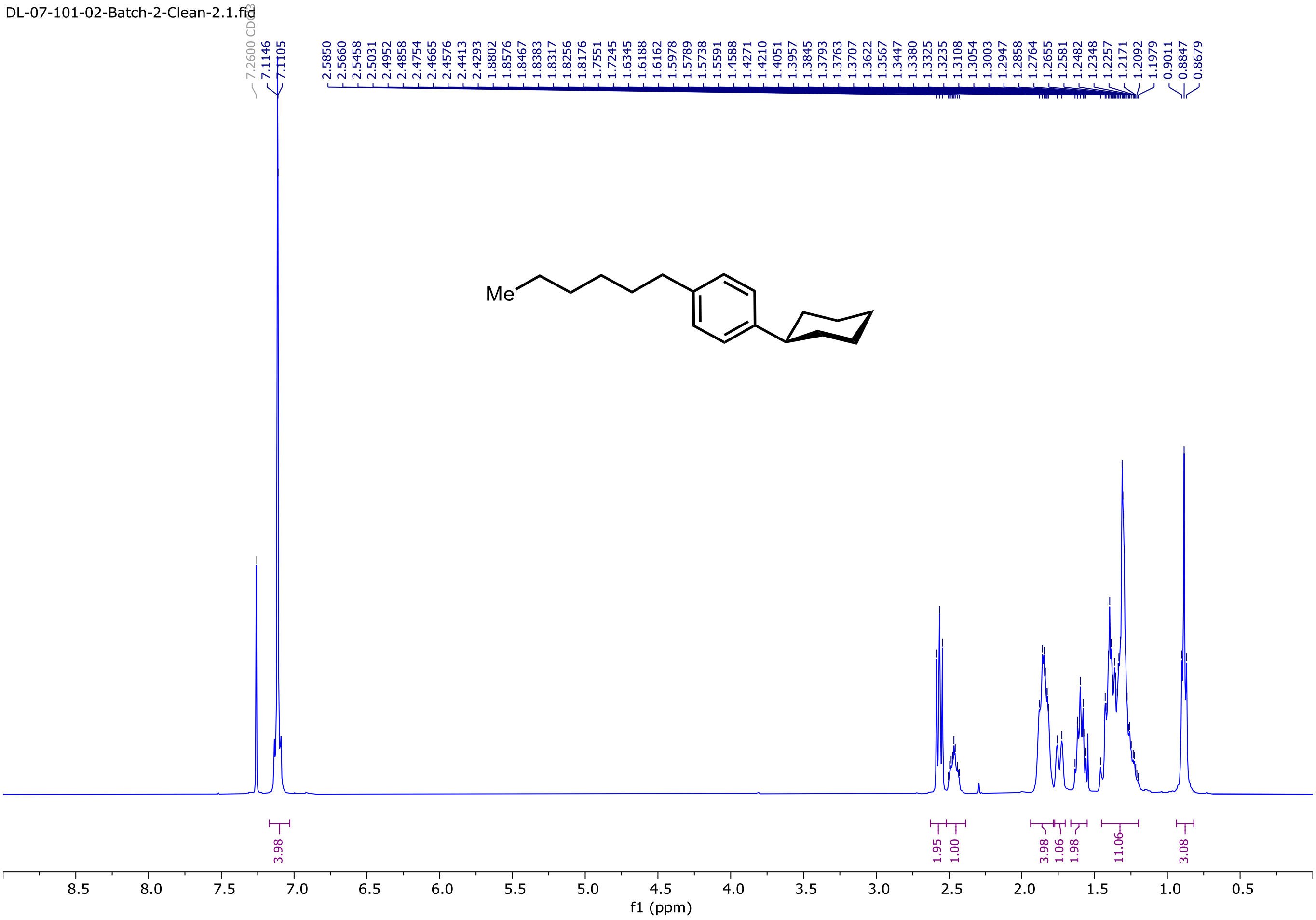

Figure S157. ^{1}H-NMR of SI-30

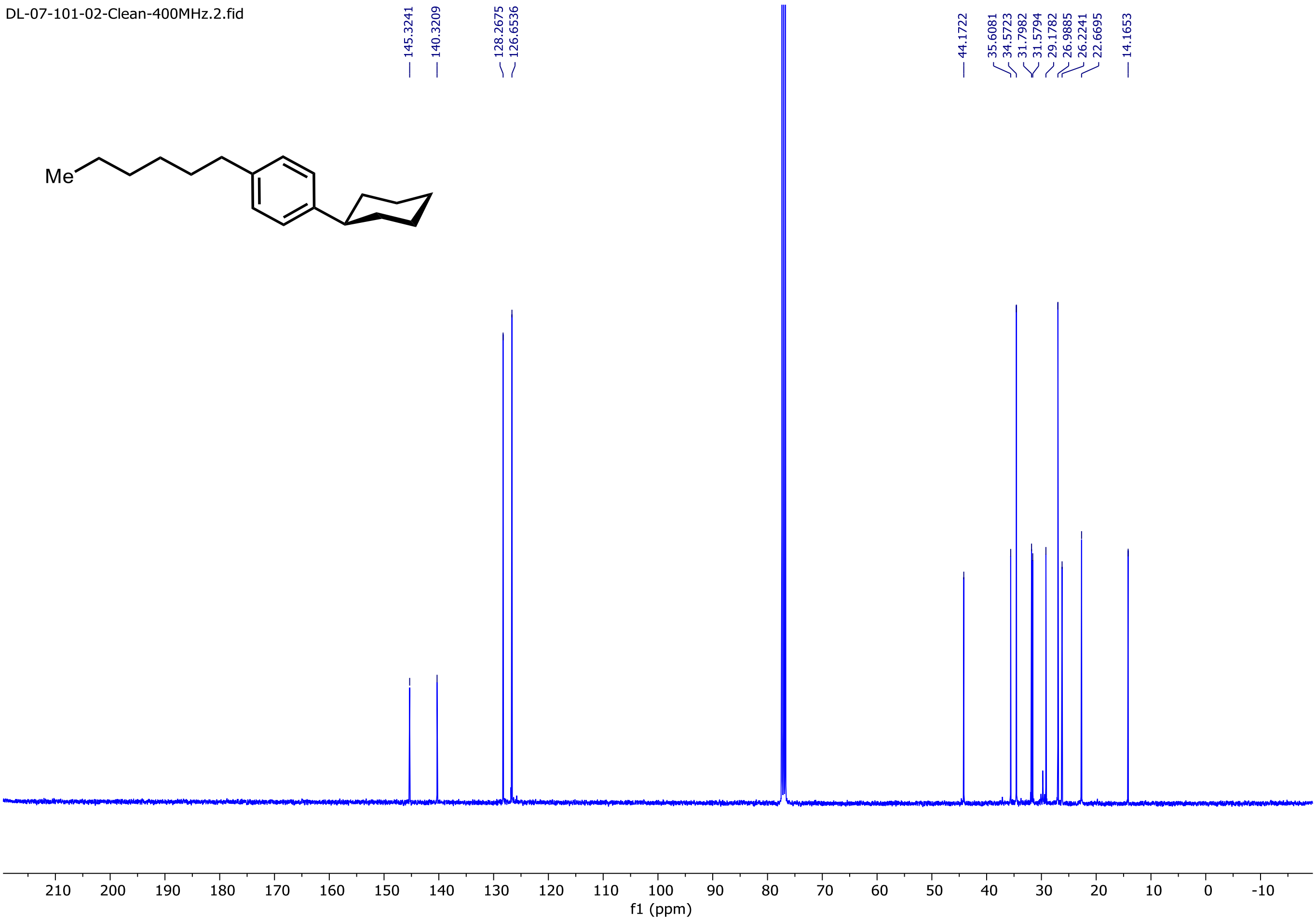

Figure S158. ^{13}C-NMR of SI-30

10.2. Spectra of catalyst synthesis

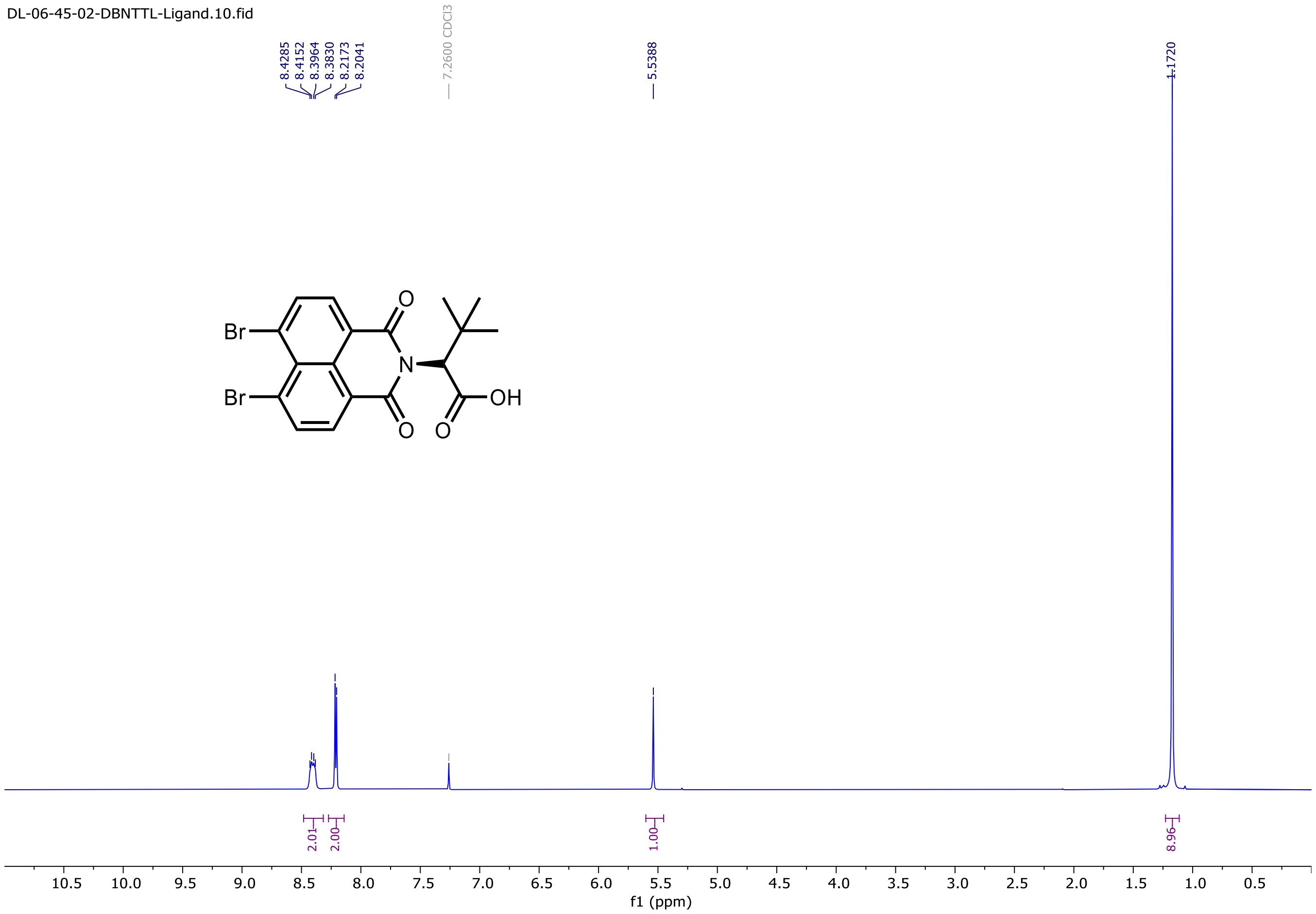

Figure S159. ^{1}H-NMR of SI-32

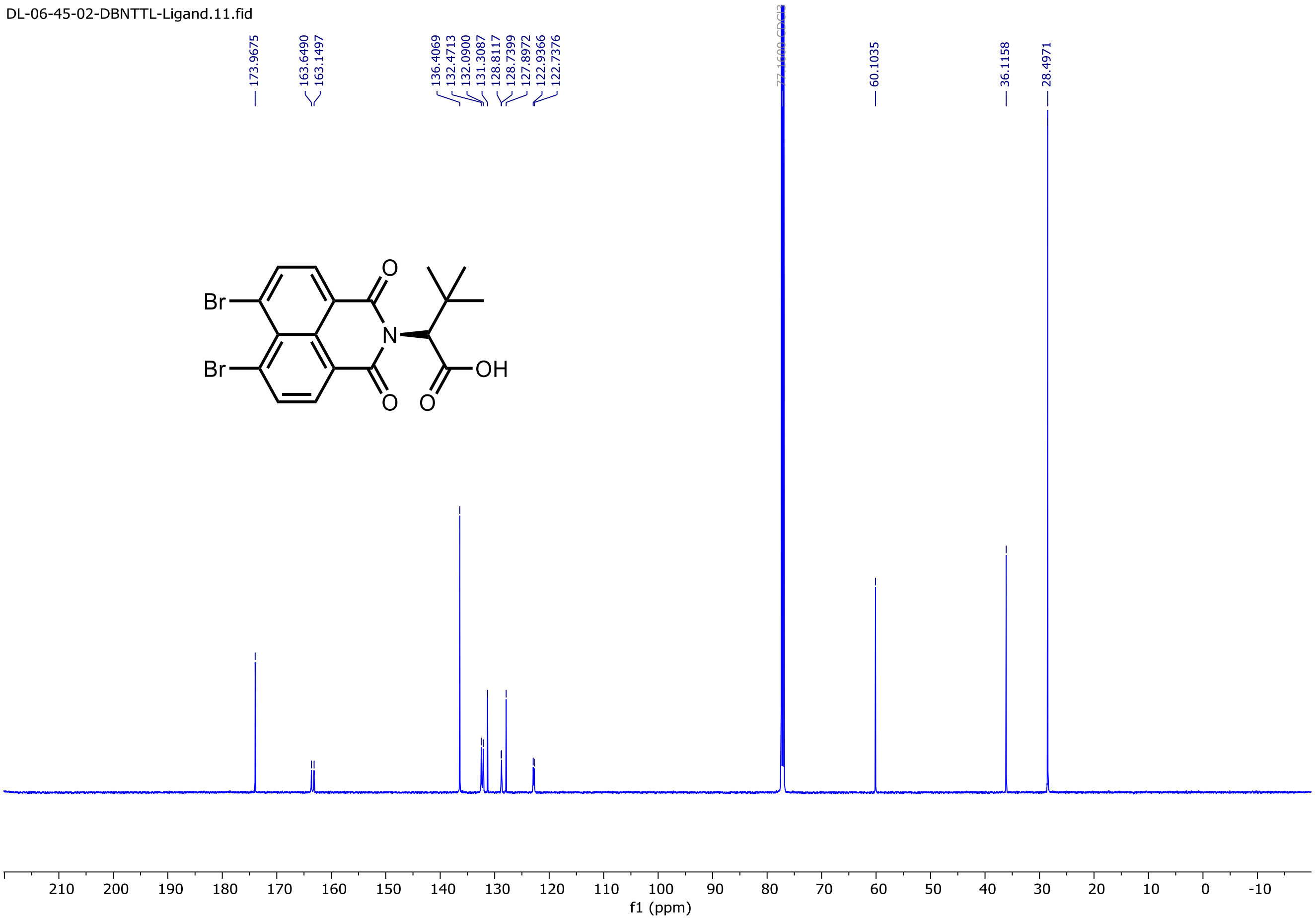

Figure S160. ^{13}C-NMR of SI-32

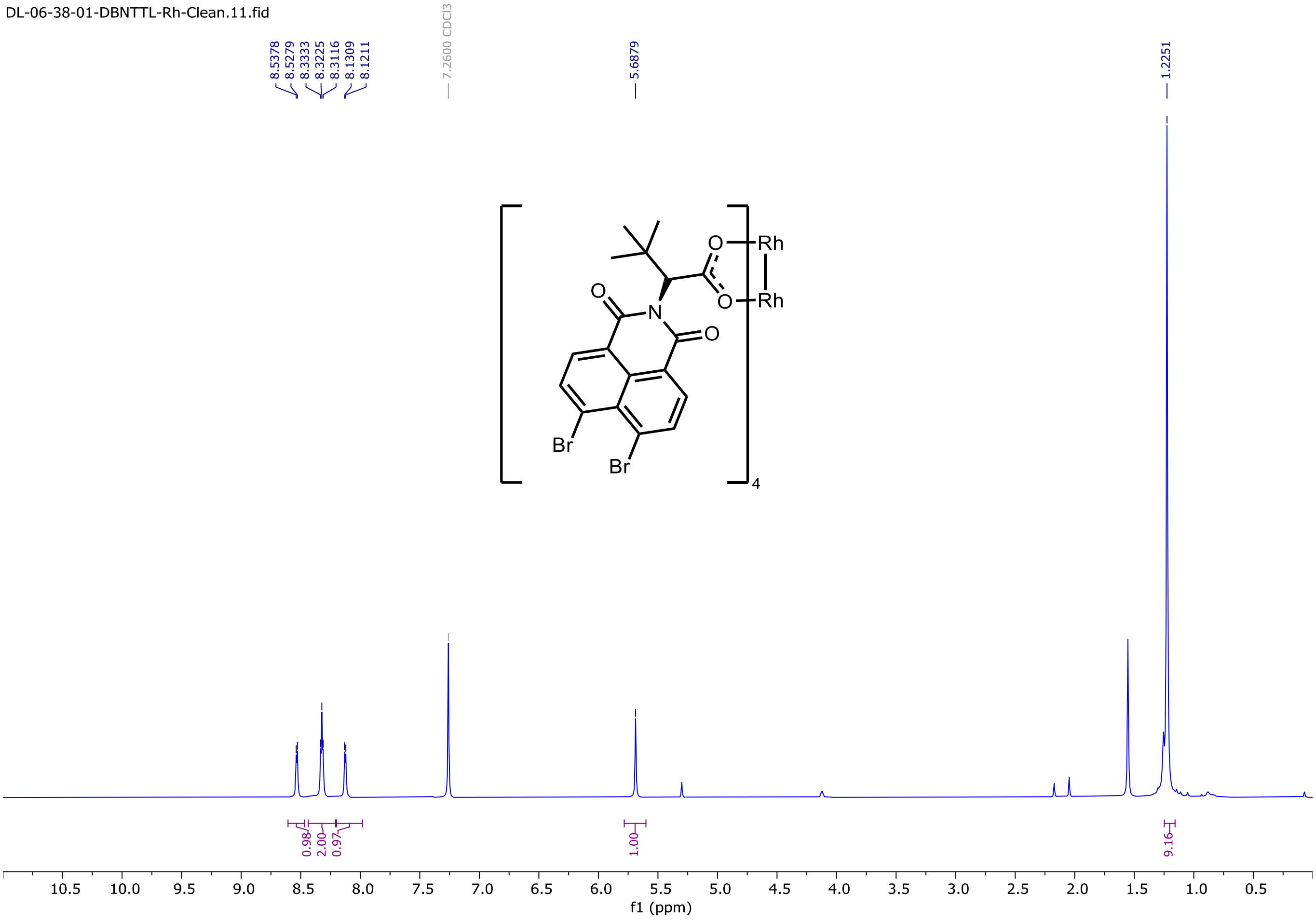

Figure S161. ^{1}**H-NMR of Rh$_2$(*S-di*-BrNTTL)$_4$ (10)**

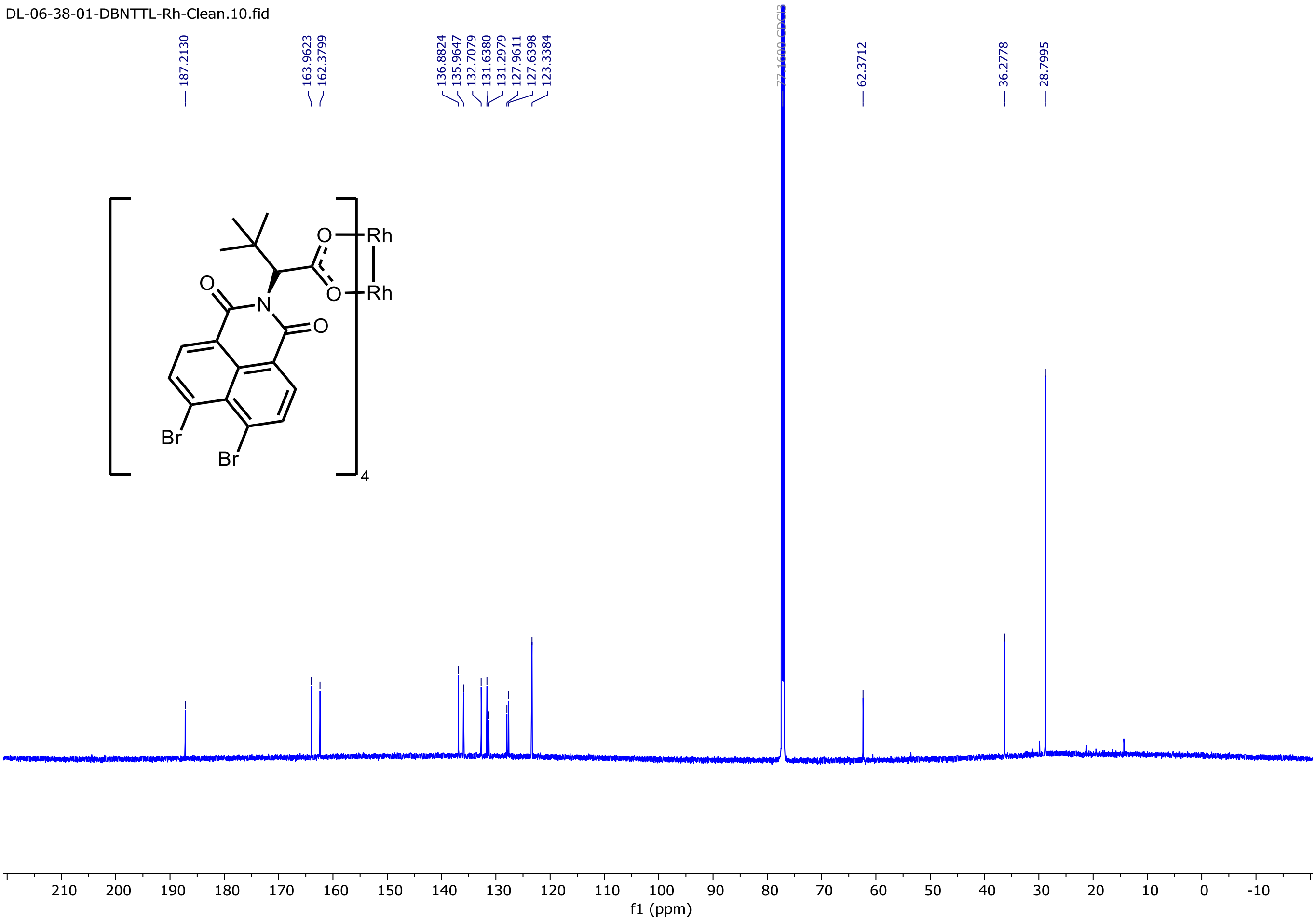

Figure S162. ^{13}**C-NMR of of Rh$_2$(*S*-di-BrNTTL)$_4$ (10)**

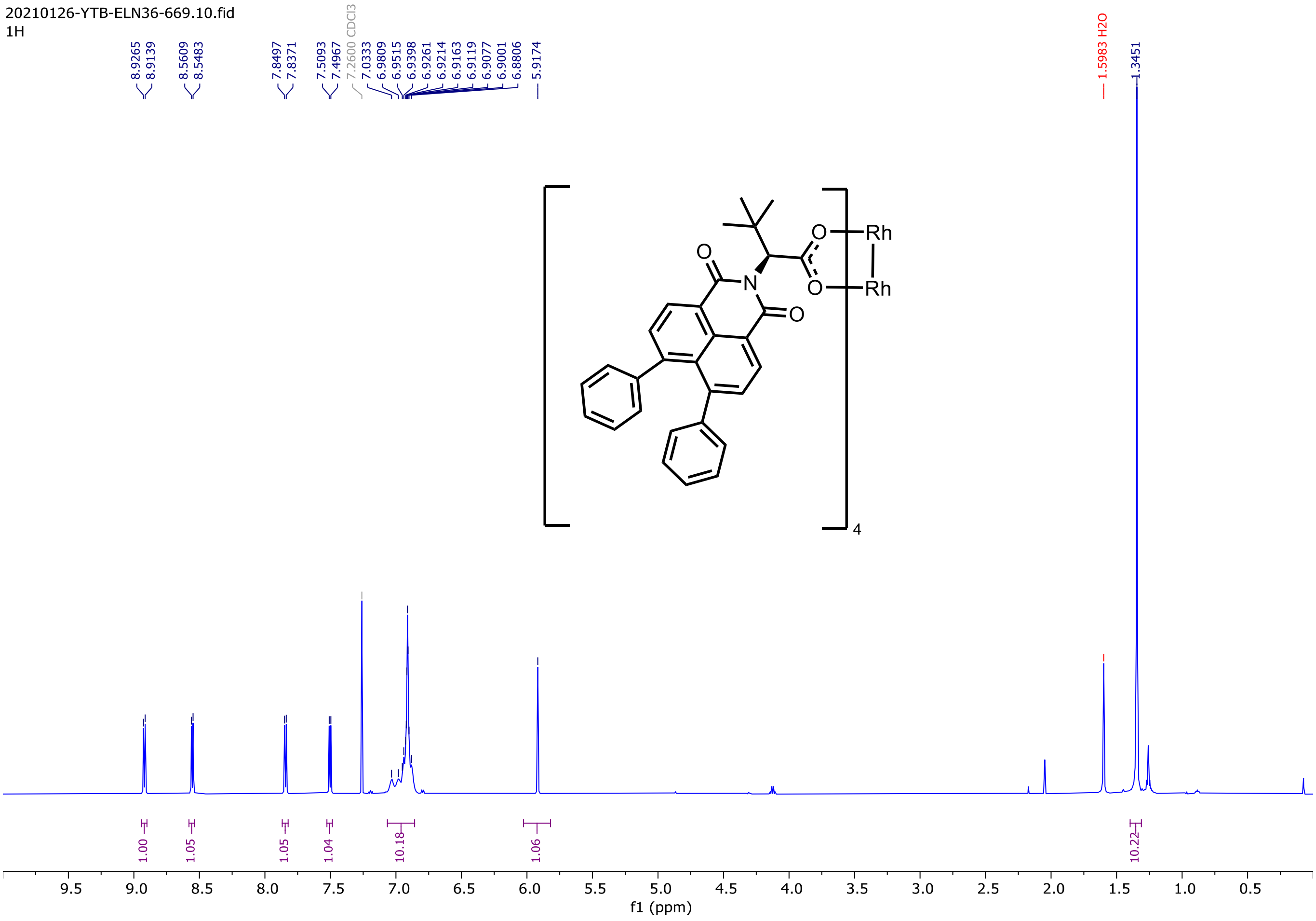

Figure S163. ^{1}H-NMR of Rh_2(*S*-di-C_6H_5NTTL)$_4$ (8a)

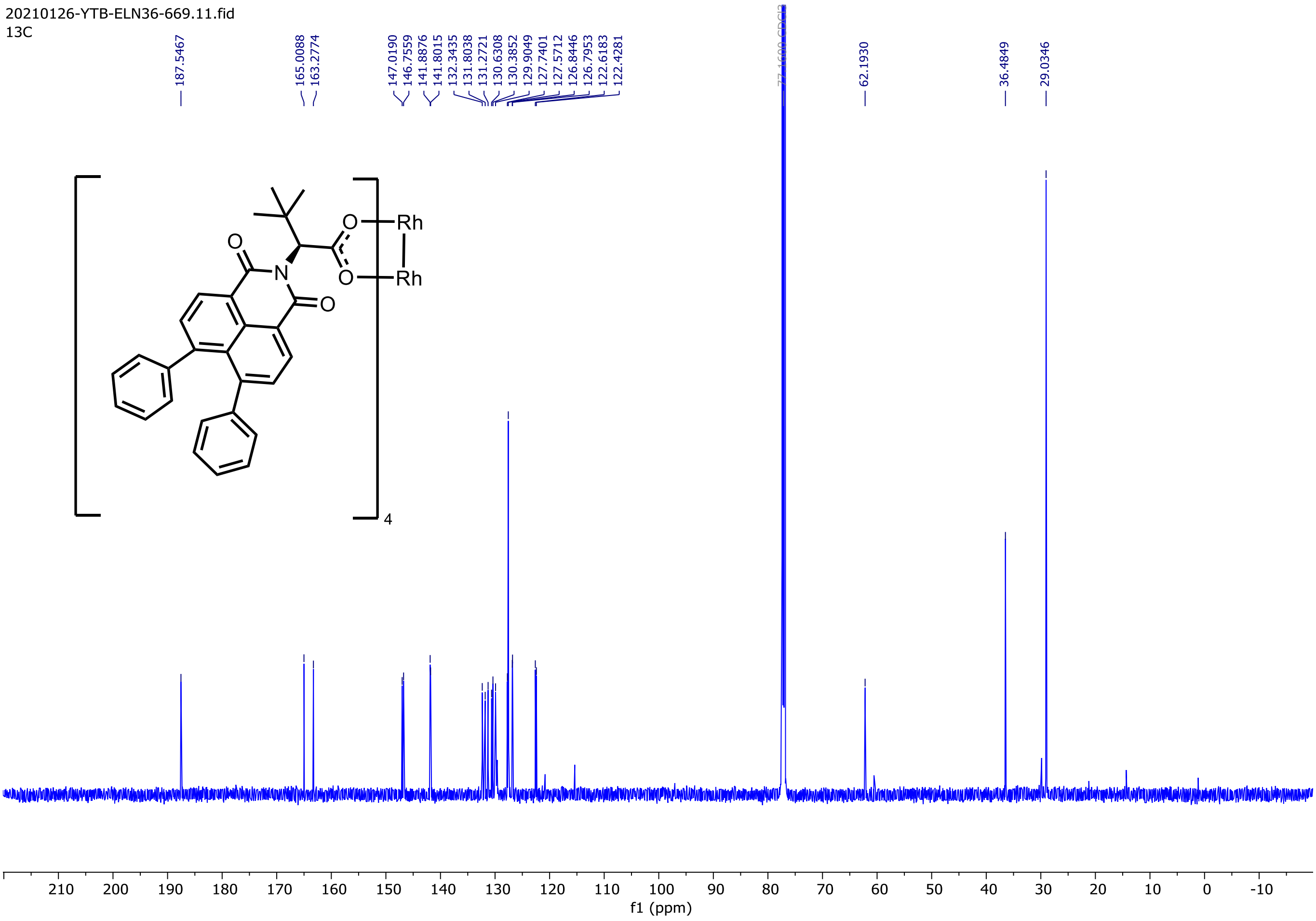

Figure S164. ^{13}C-NMR of Rh_2(*S*-di-C_6H_5NTTL)$_4$ (8a)

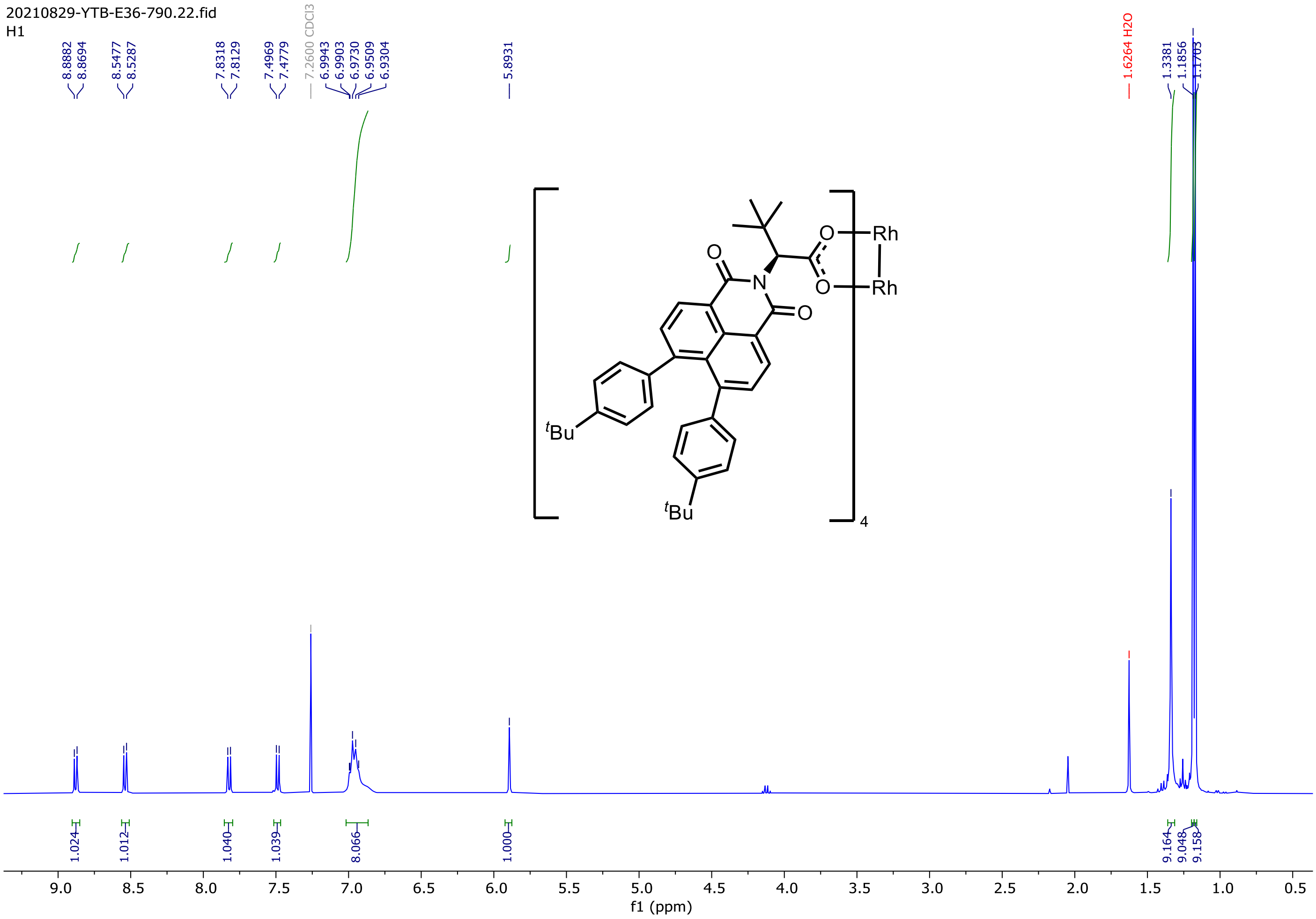

Figure S165. ^{1}H-NMR of Rh_2(S-*di*-4-^{t}BuC_6H_4NTTL)$_4$ (8b)

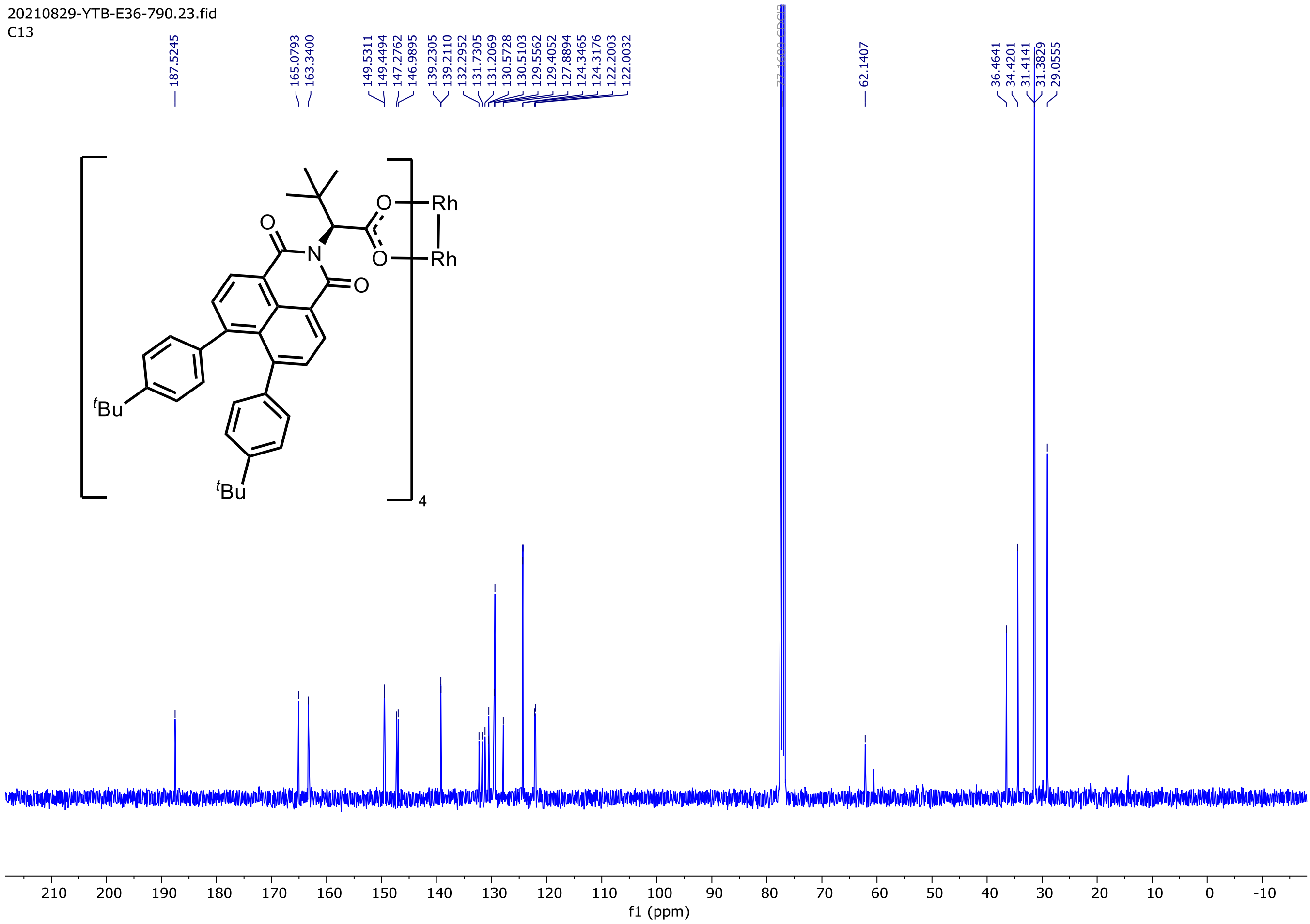

Figure S166. ^{13}C-NMR of Rh_2(S-*di*-4-^{t}BuC_6H_4NTTL)$_4$ (8b)

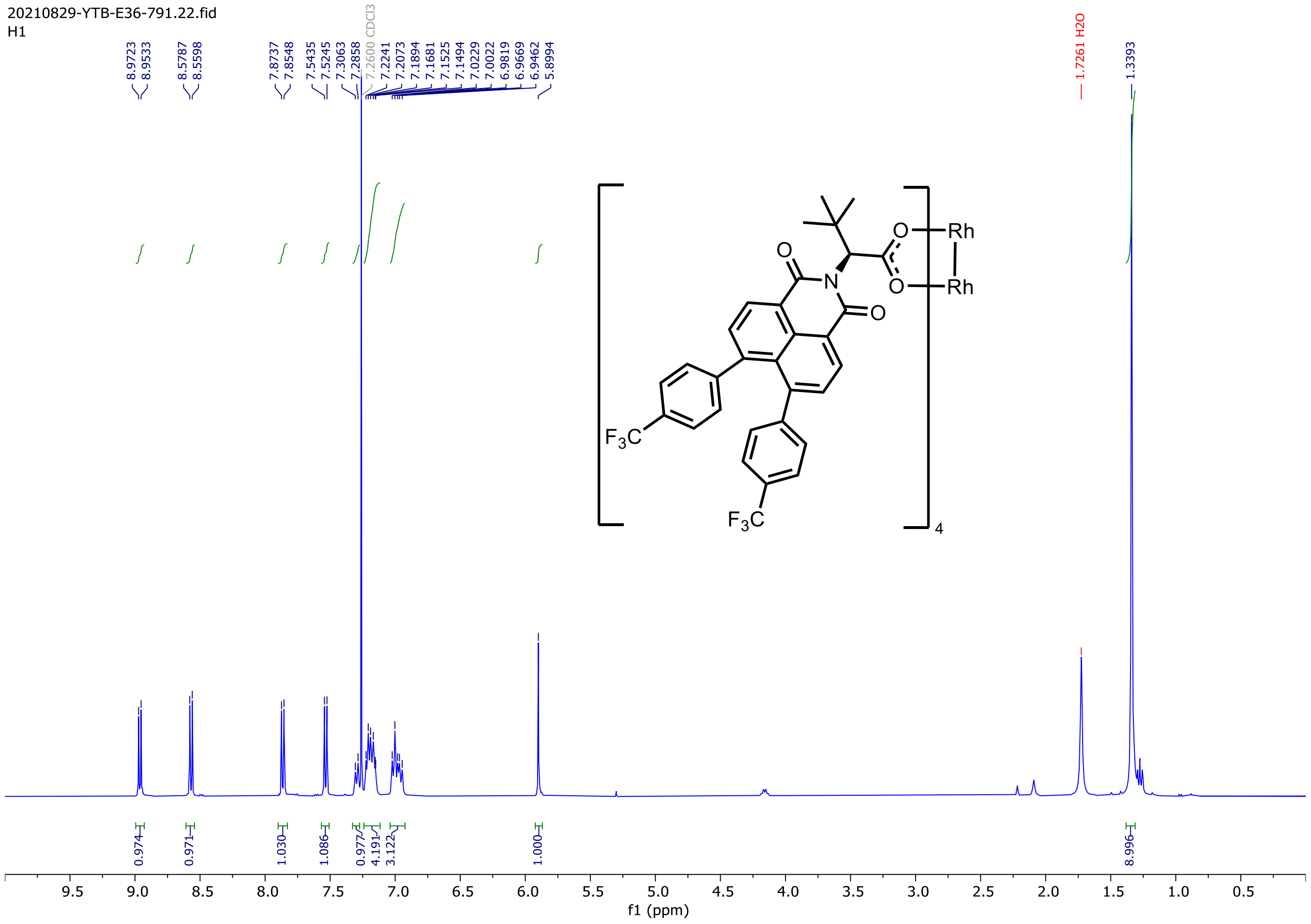

Figure S167. **^{1}H-NMR of Rh$_2$(*S-di*-4-CF$_3$C$_6$H$_4$NTTL)$_4$ (8c)**

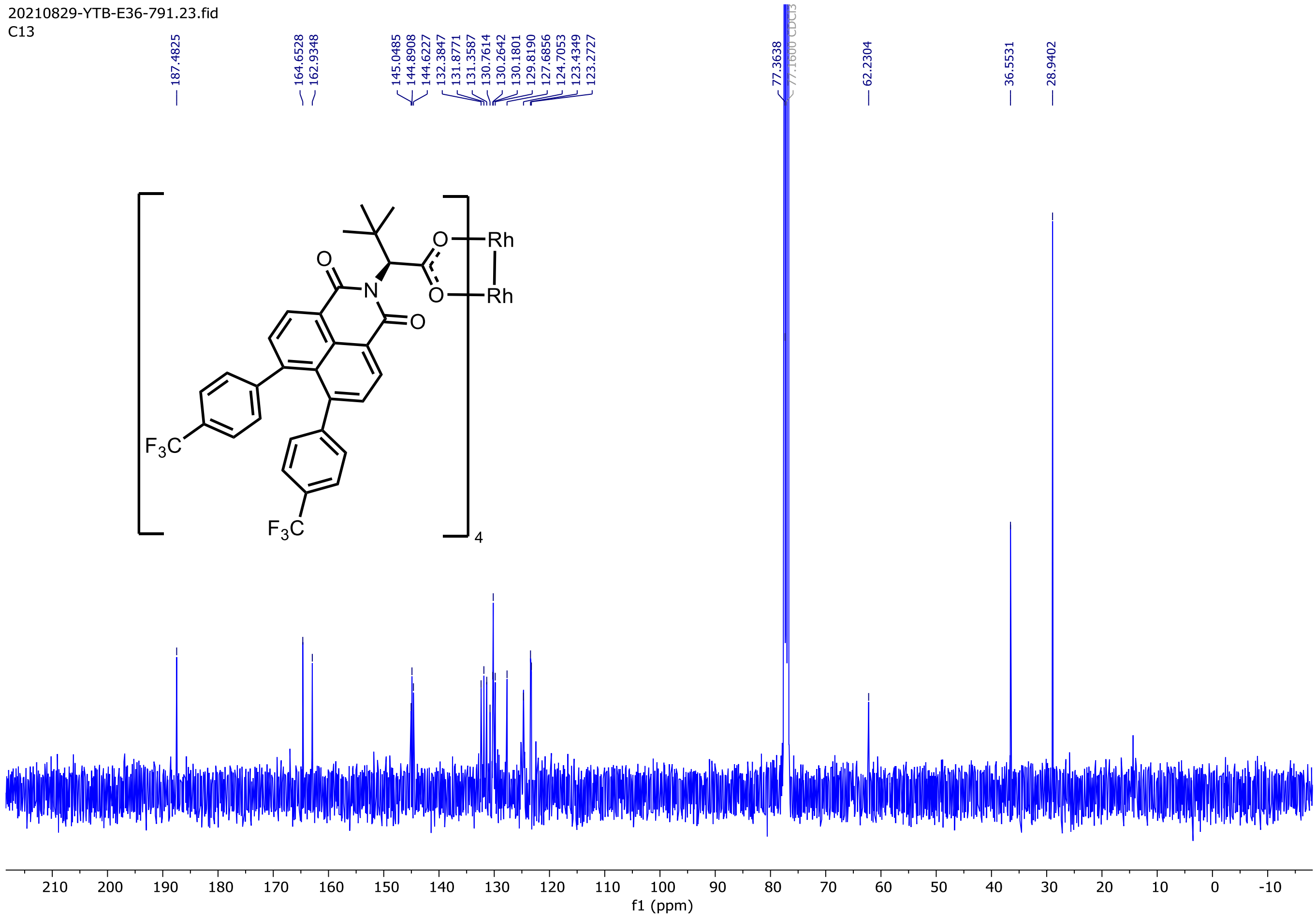

Figure S168. **^{13}C-NMR of Rh$_2$(*S-di*-4-CF$_3$C$_6$H$_4$NTTL)$_4$ (8c)**

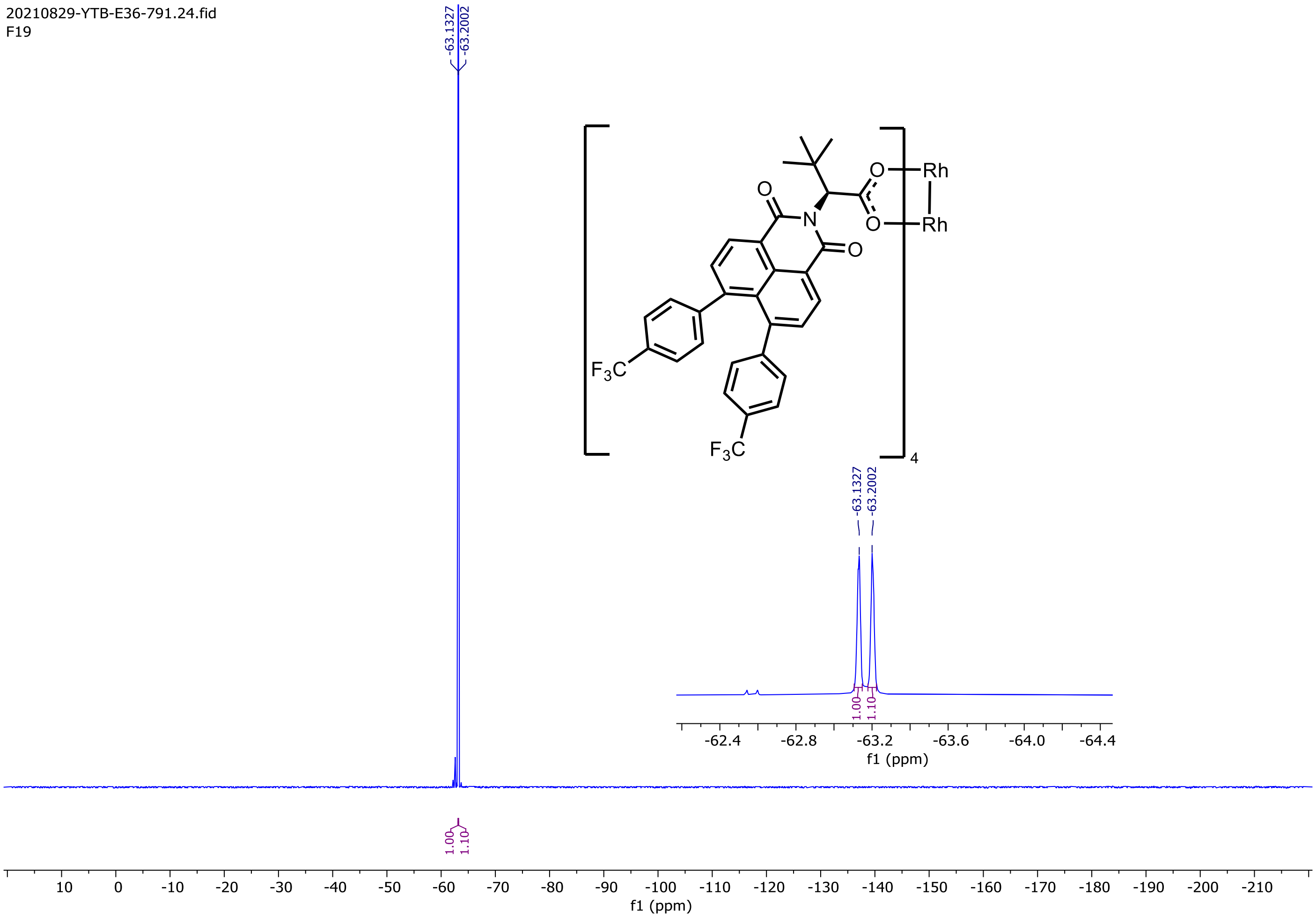

Figure S169. ^{19}**F-NMR of Rh$_2$(*S-di*-4-CF$_3$C$_6$H$_4$NTTL)$_4$ (8c)**

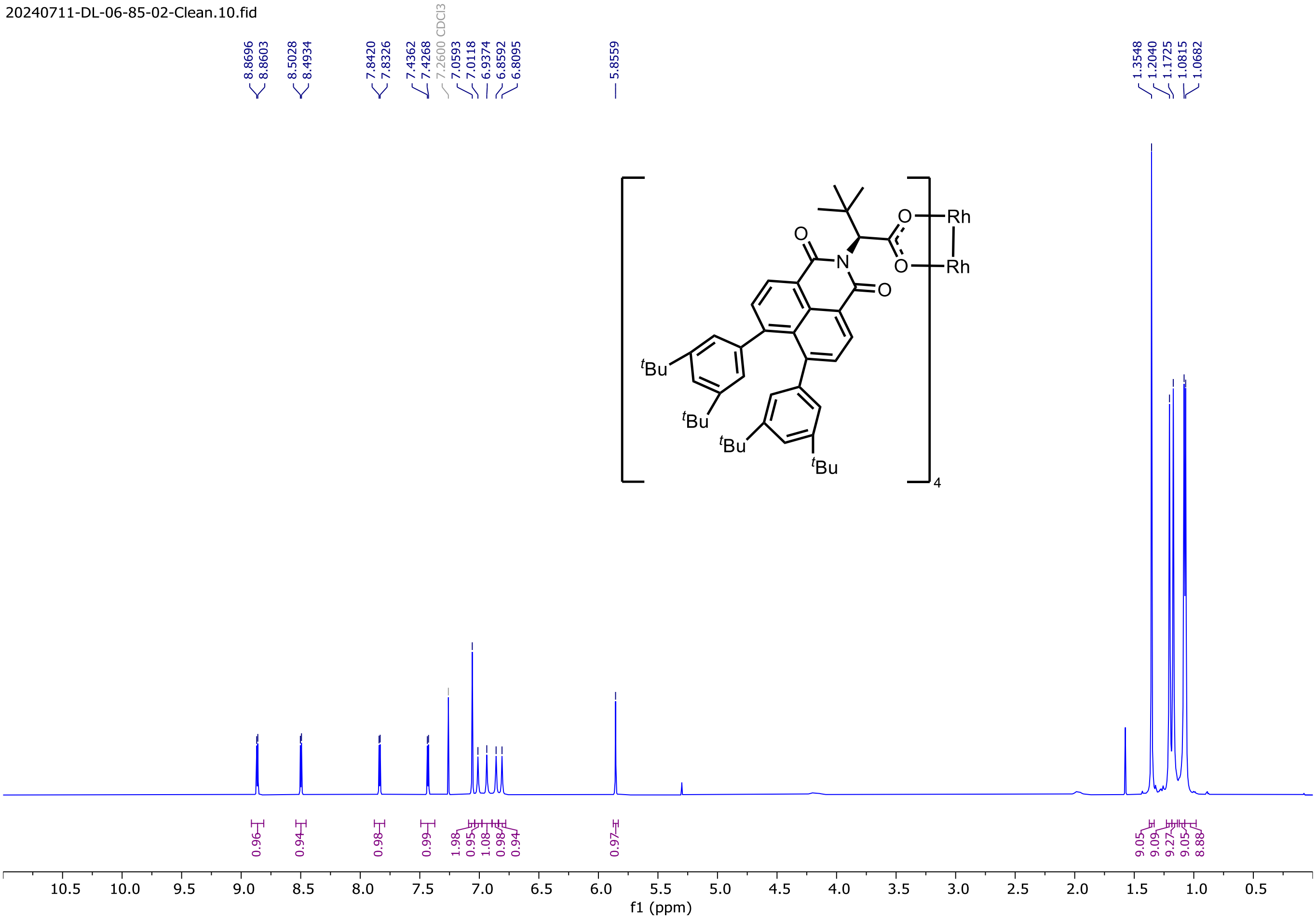

Figure S170. ^{1}H-NMR of Rh_2(*S*-*di*-3,5-*di*-^{t}BuC_6H_3NTTL)$_4$ (8d)

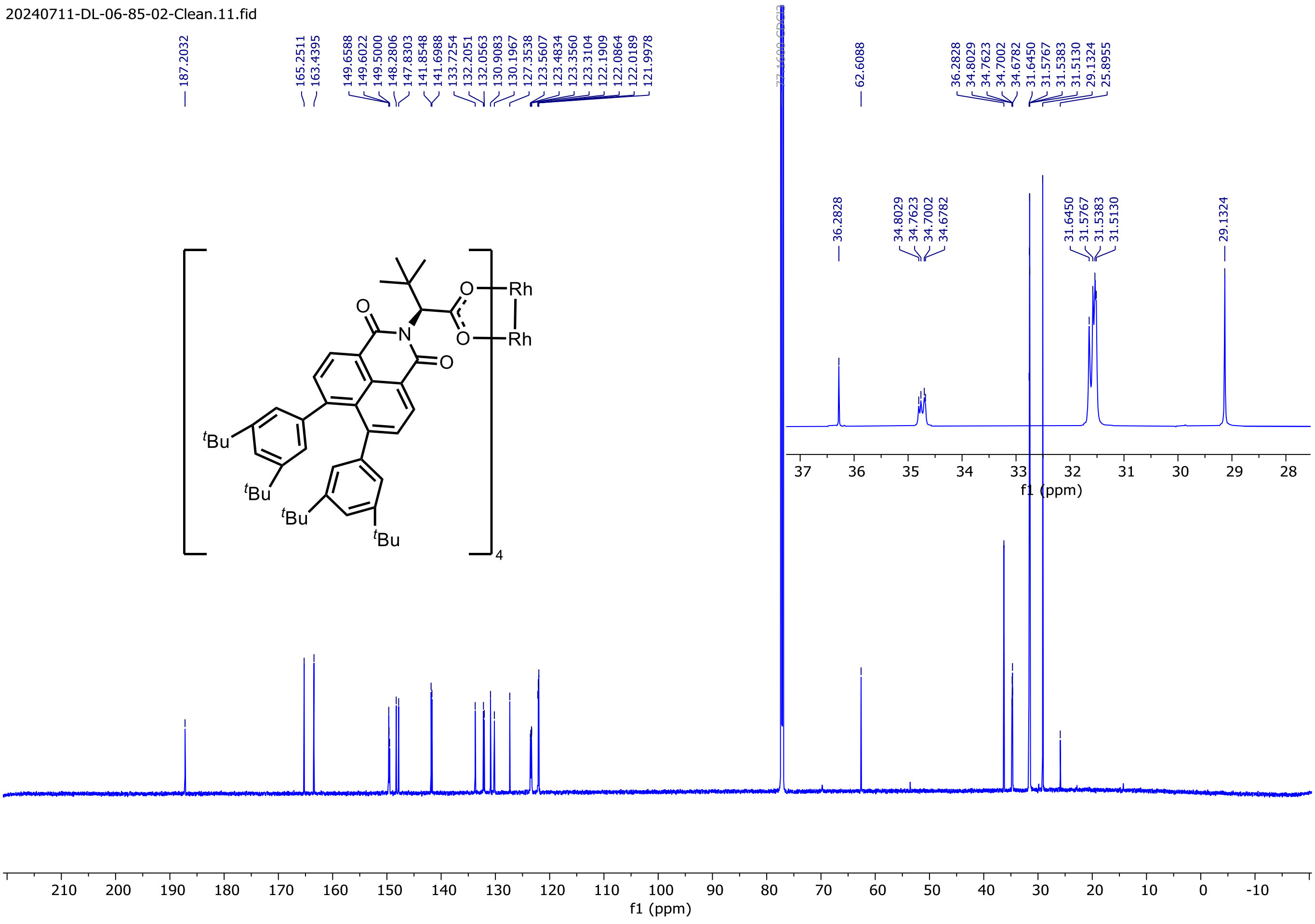

Figure S171. ^{13}C-NMR of Rh_2(*S*-*di*-3,5-*di*-^{t}BuC_6H_3NTTL)$_4$ (8d)

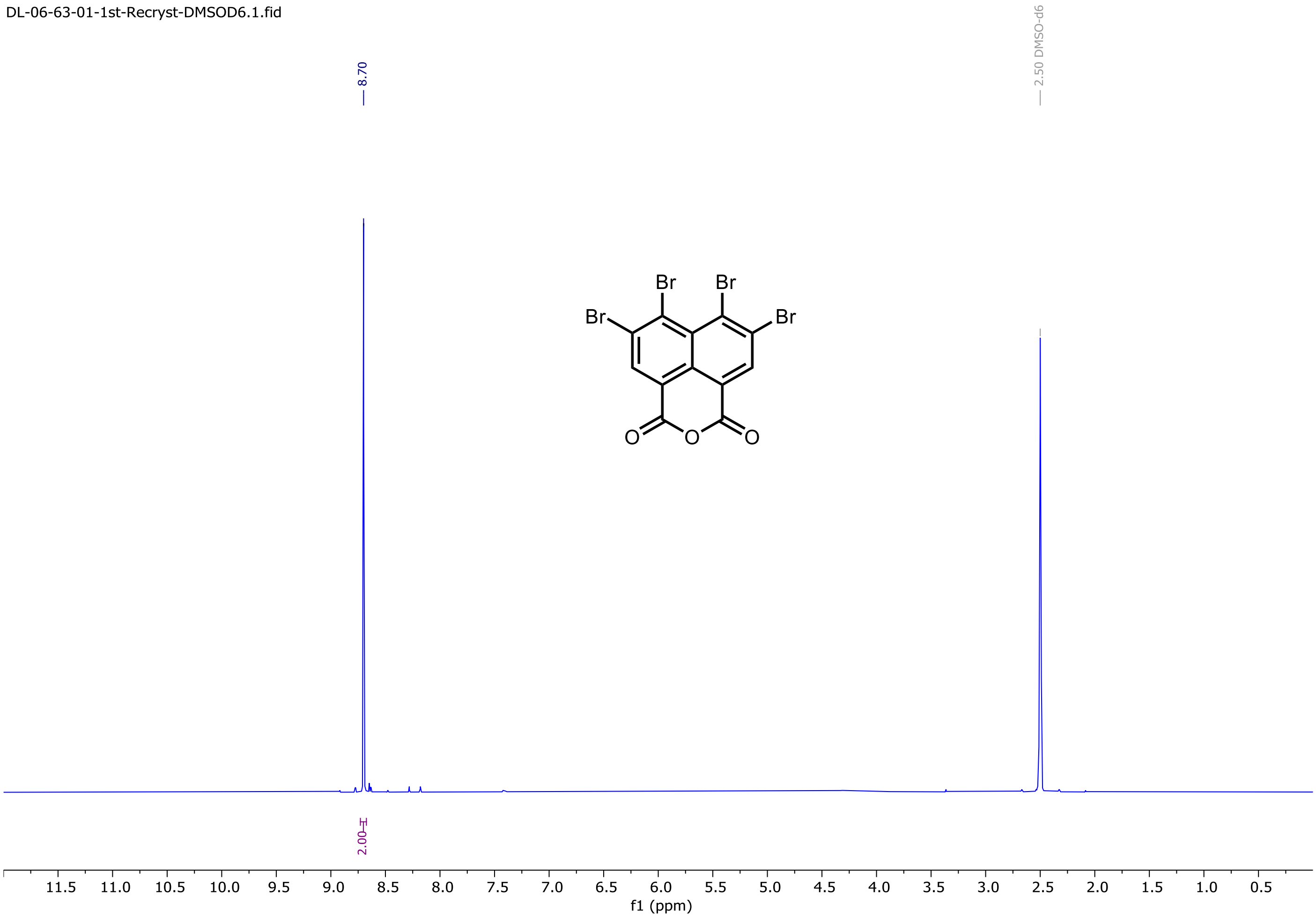

Figure S172. ^{1}H-NMR of SI-33

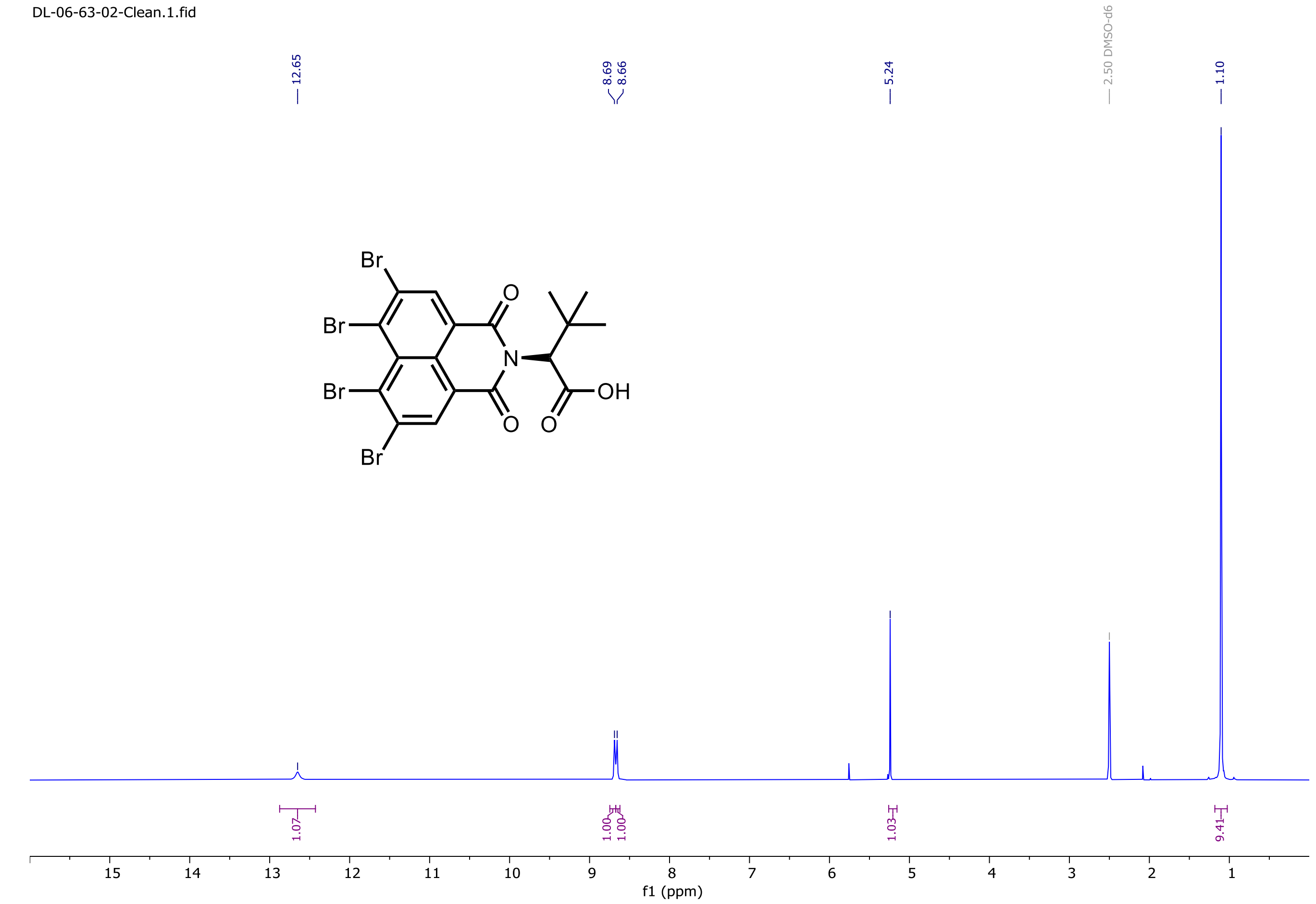

Figure S173. ^{1}H-NMR of SI-34

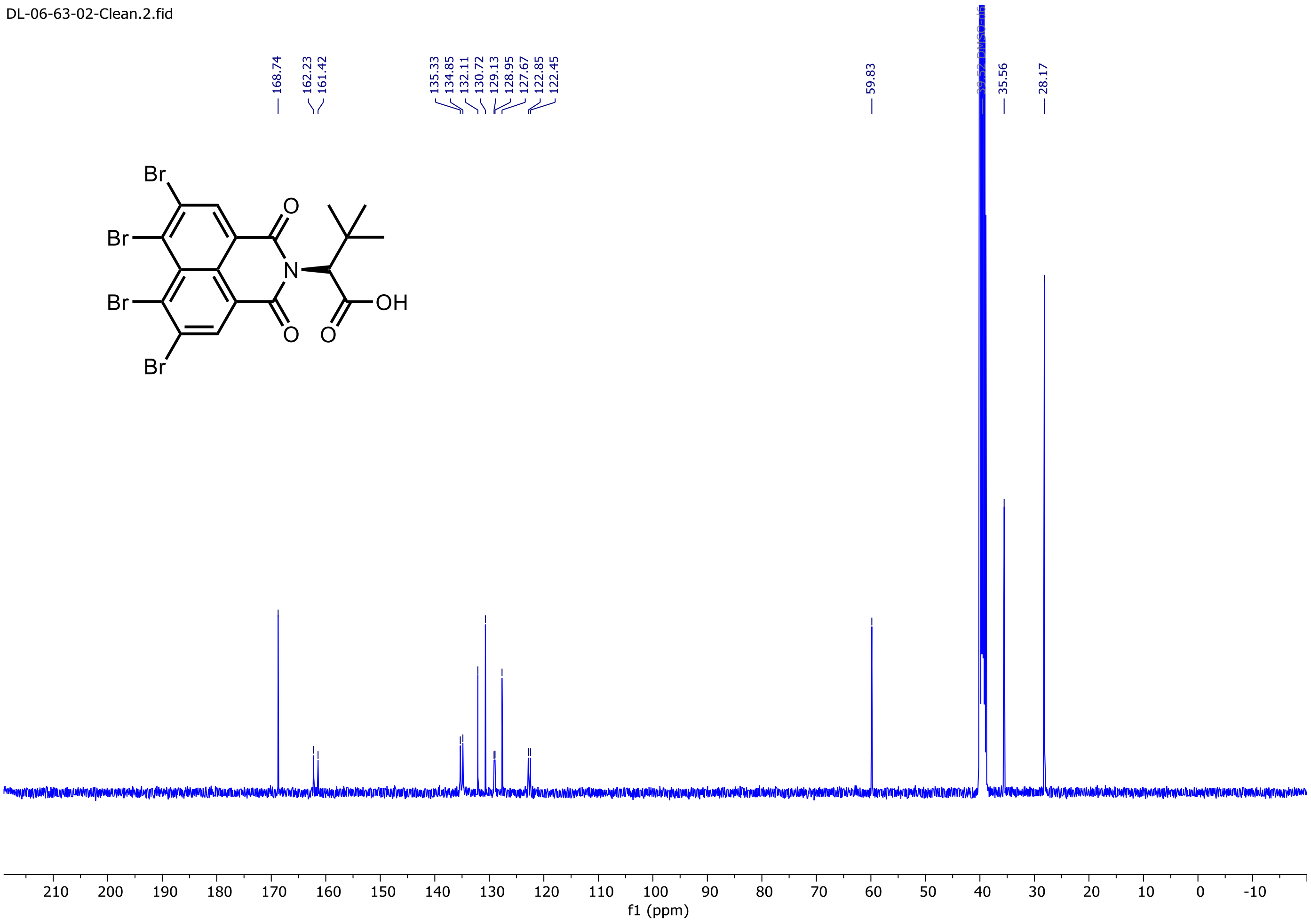

Figure S174. ^{13}C-NMR of SI-34

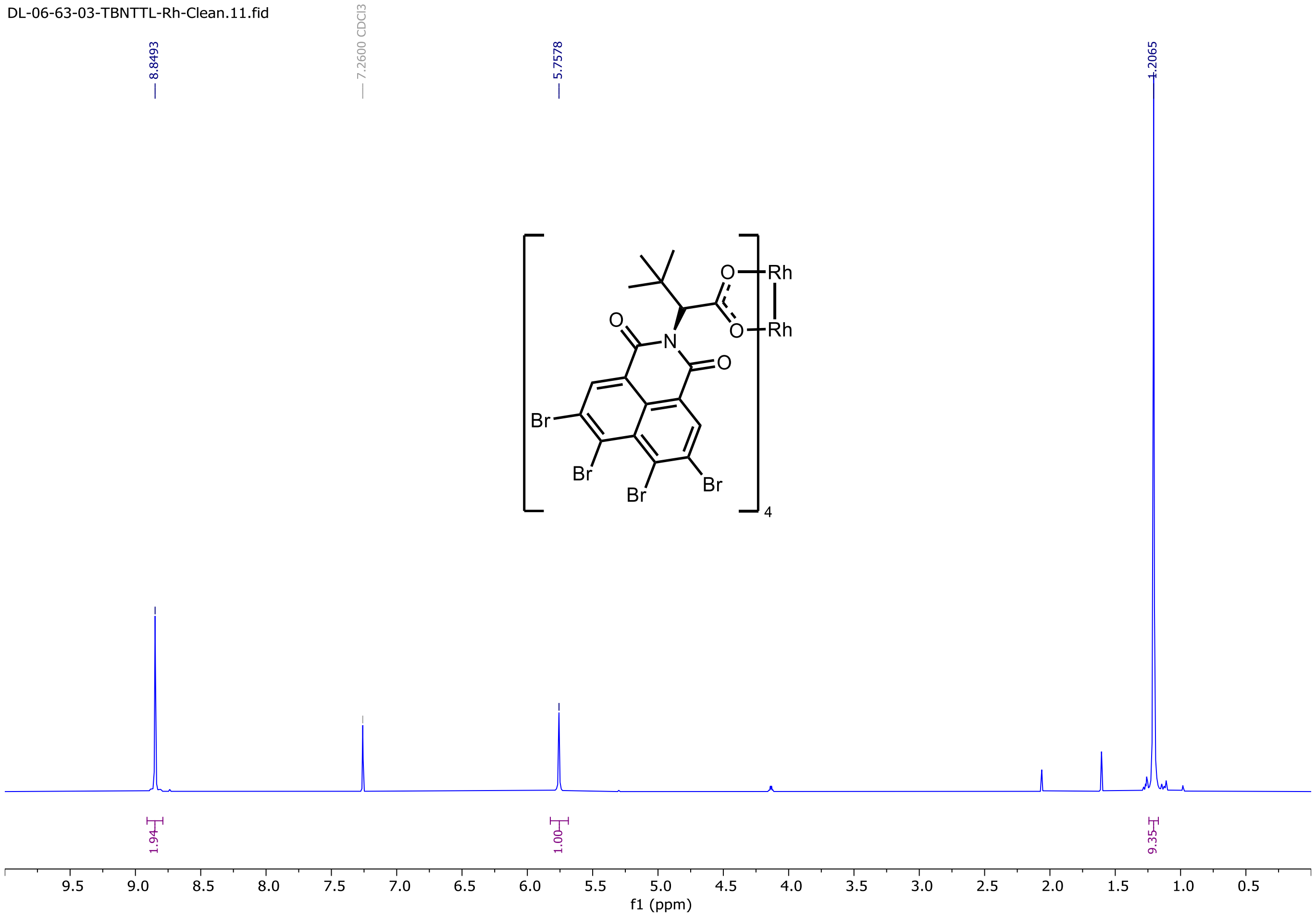

Figure S175. ^{1}**H-NMR of** Rh_2**(S-tetra-BrNTTL)**$_4$ **(9)**

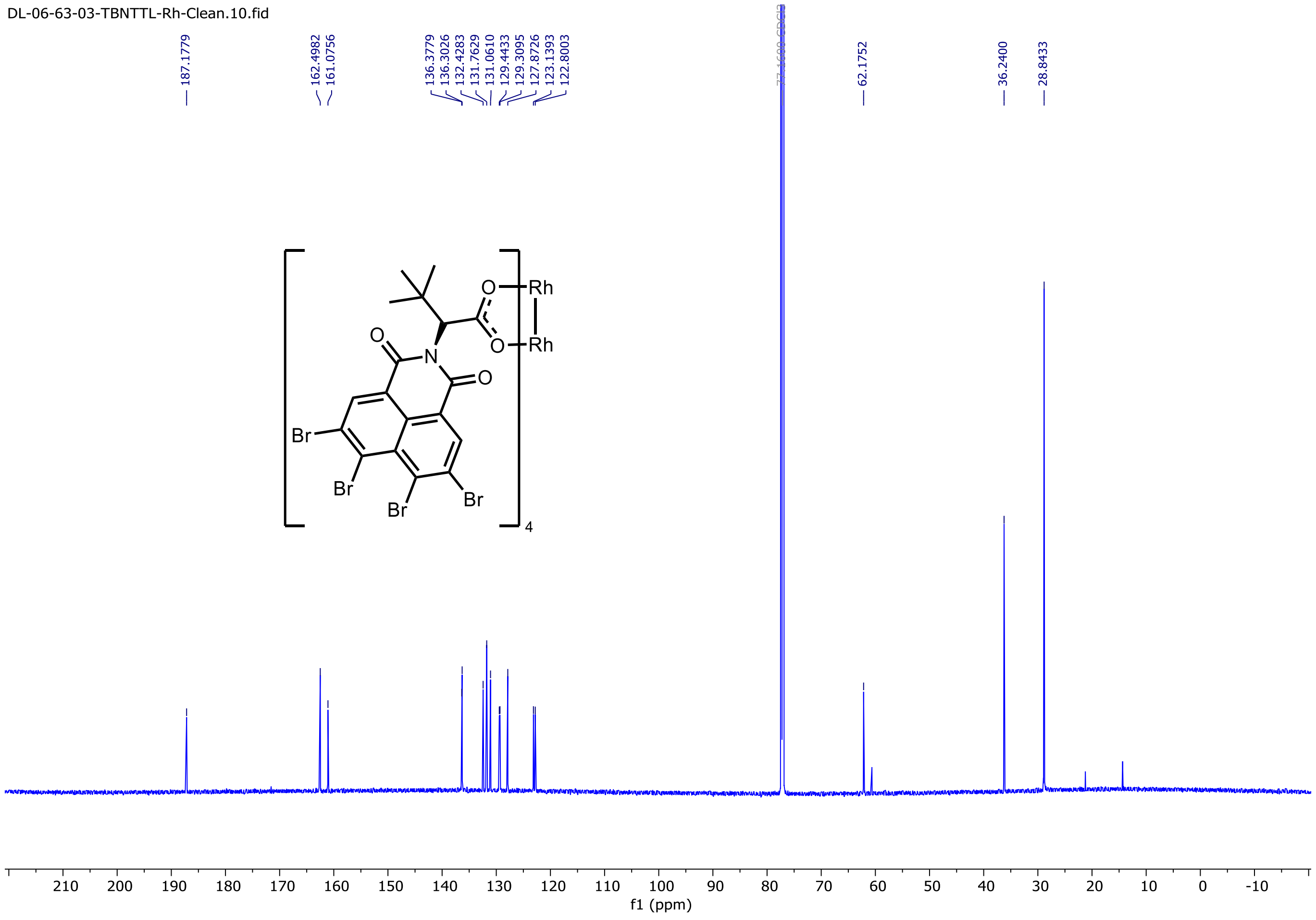

Figure S176. ^{13}**C-NMR of** Rh_2**(S-tetra-BrNTTL)**$_4$ **(9)**

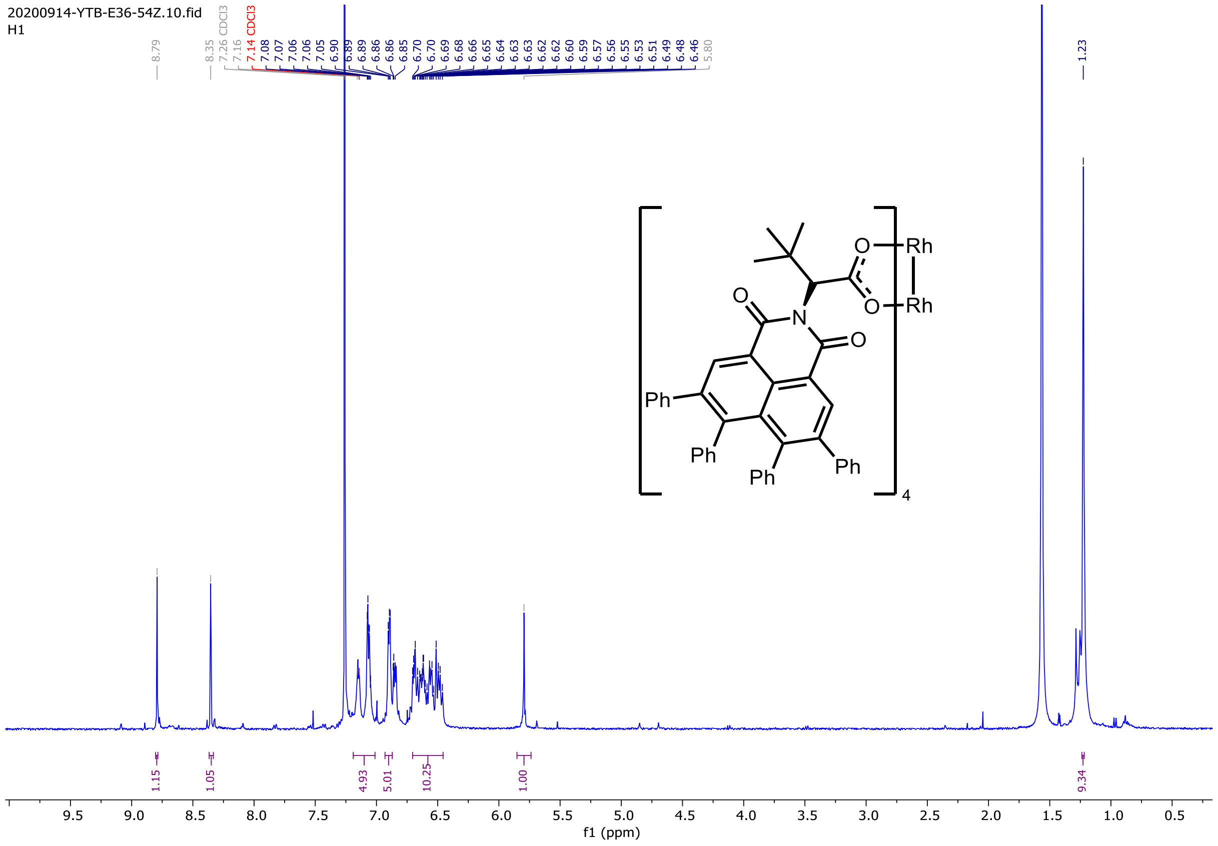

Figure S177. ^{1}H-NMR of Rh_2(*S*-*tetra*-C_6H_5NTTL)$_4$ (7a)

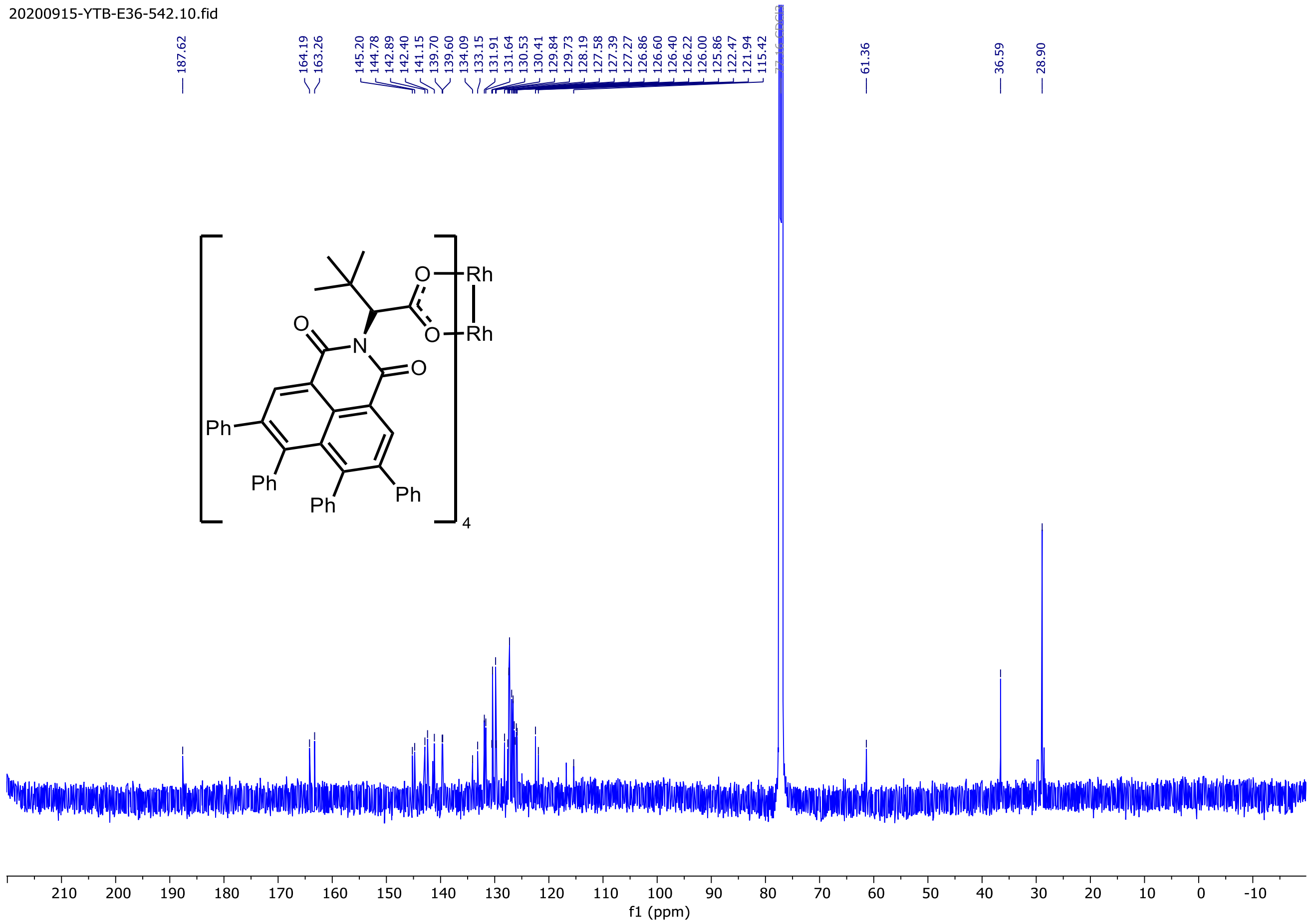

Figure S178. ^{13}C-NMR of Rh_2(S-*tetra*-C_6H_5NTTL)$_4$ (7a)

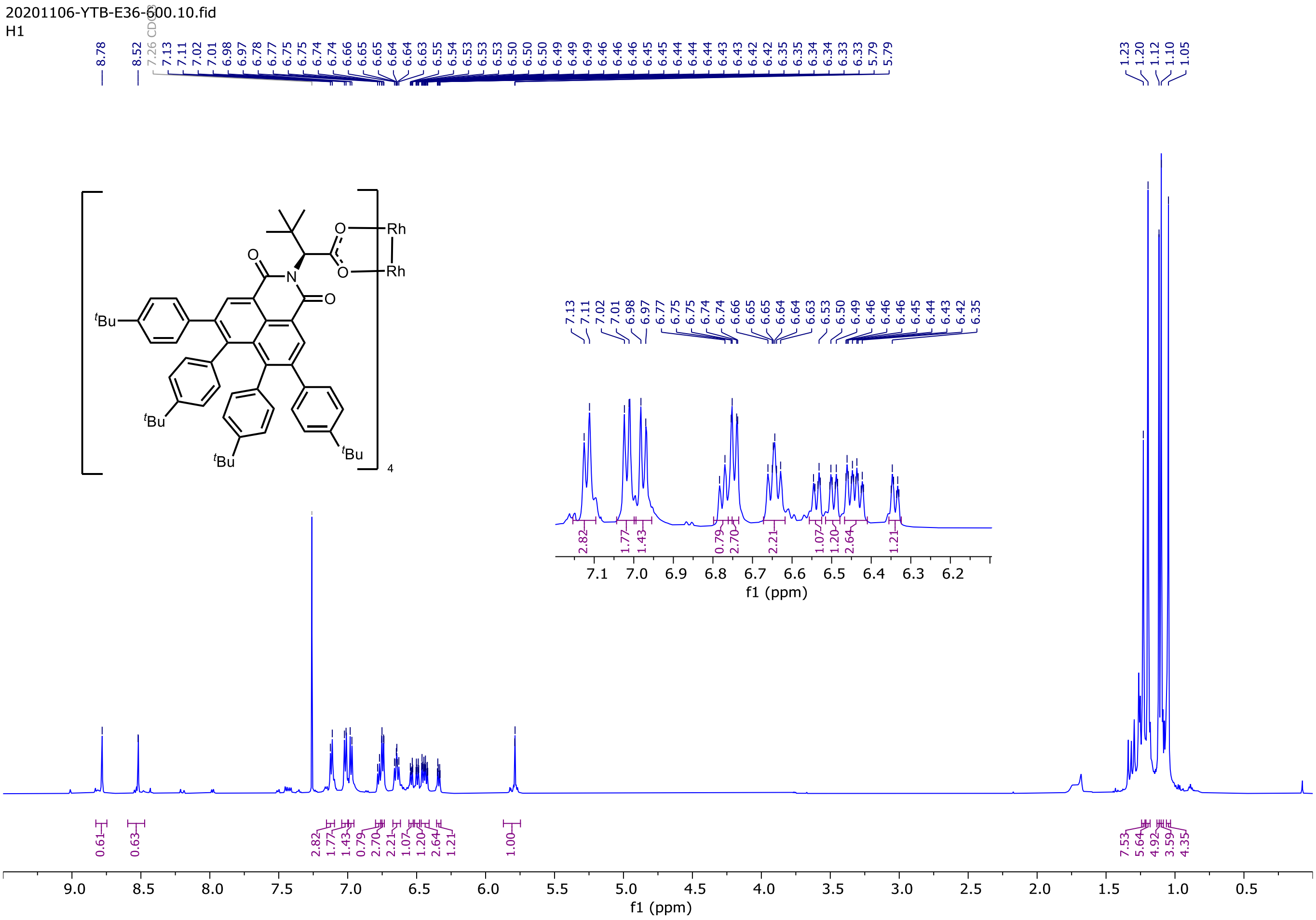

Figure S179. ^{1}H-NMR of Rh_2(S-*tetra*-4-^{t}Bu-C_6H_4NTTL)$_4$ (7b)

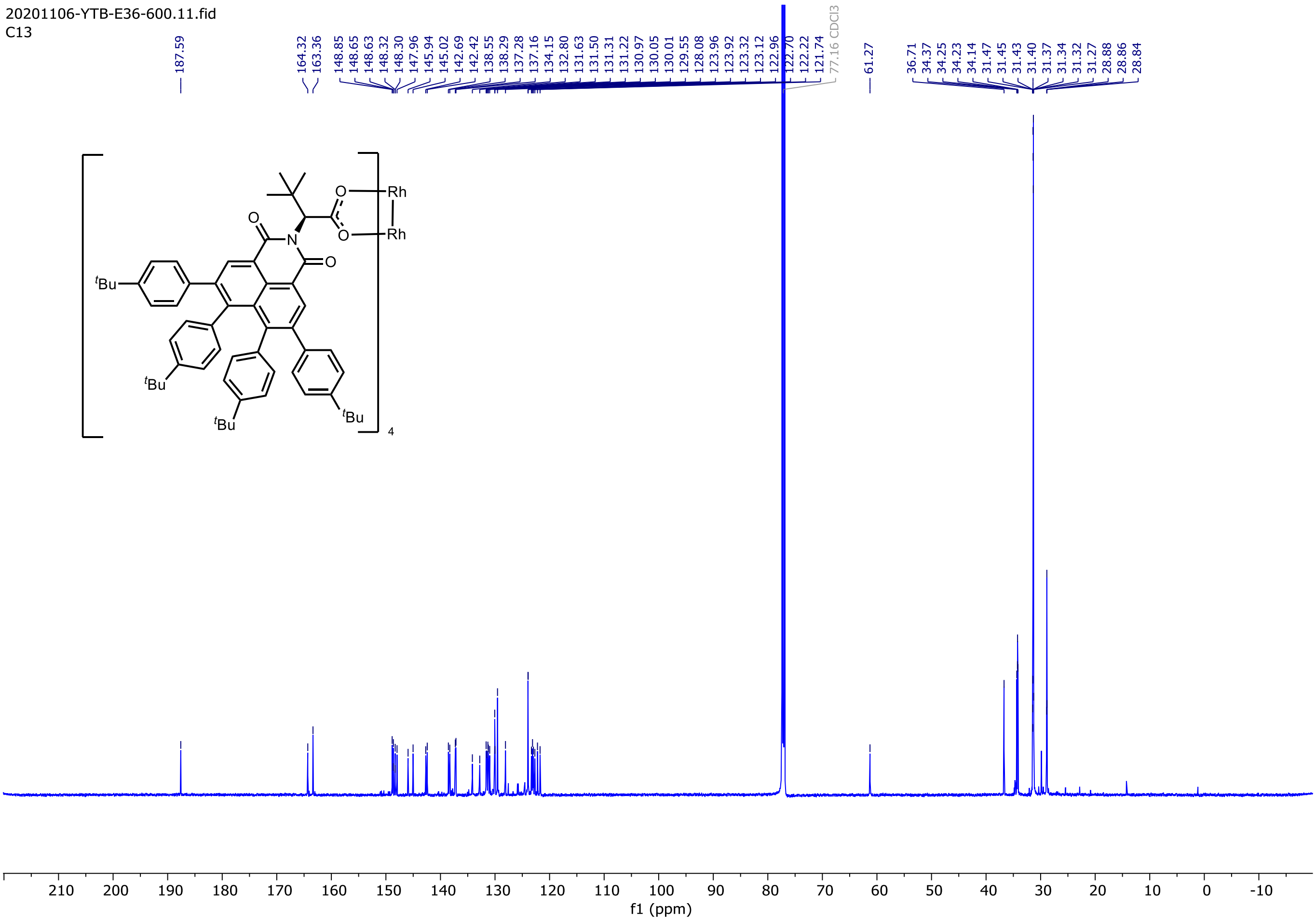

Figure S180. ^{13}C-NMR of Rh_2(S-*tetra*-4-^{t}Bu-C_6H_4NTTL)$_4$ (7b)

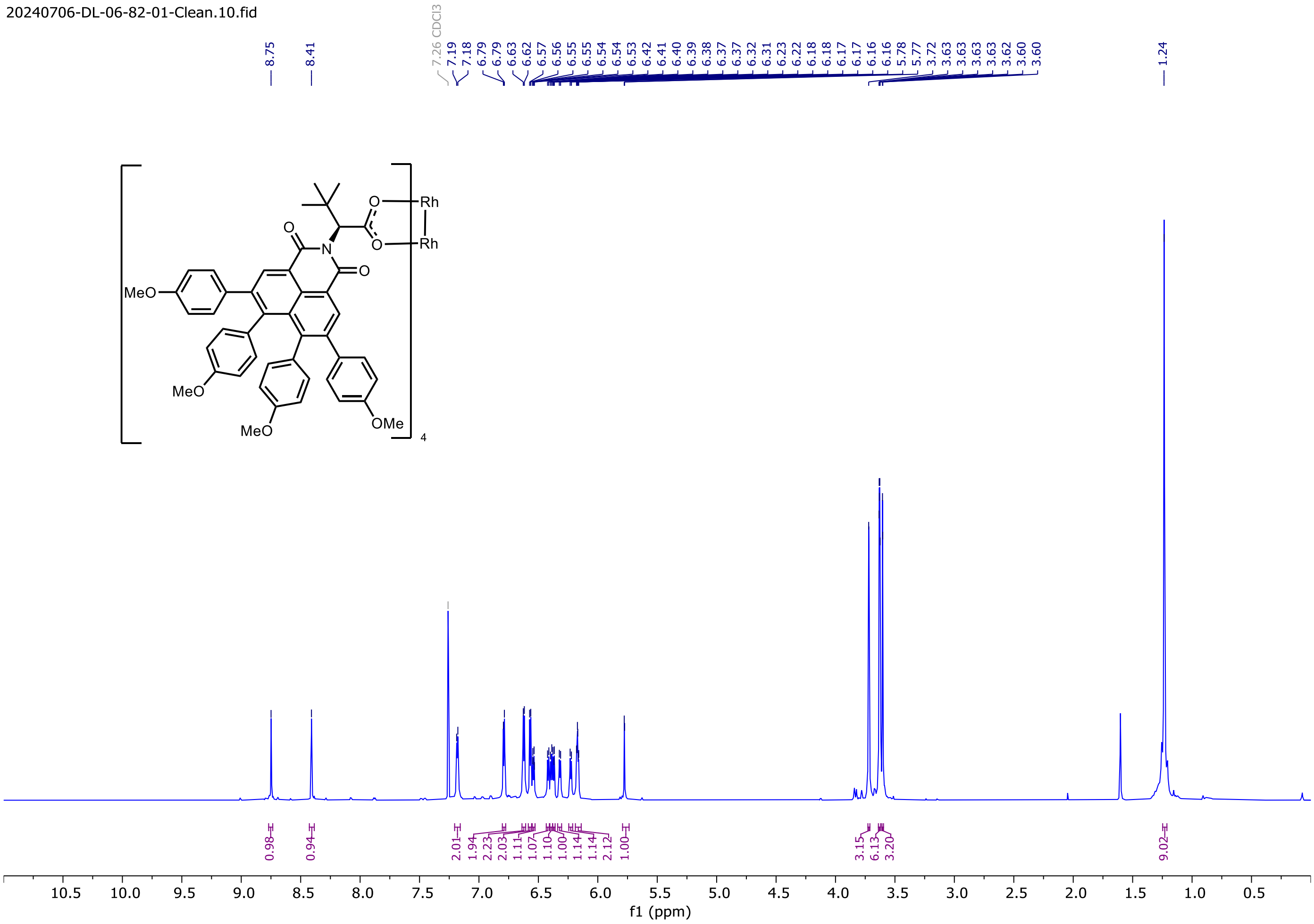

Figure S181. ^{1}H-NMR of Rh_2(S-*tetra*-4-MeO-C_6H_4NTTL)$_4$ (7c)

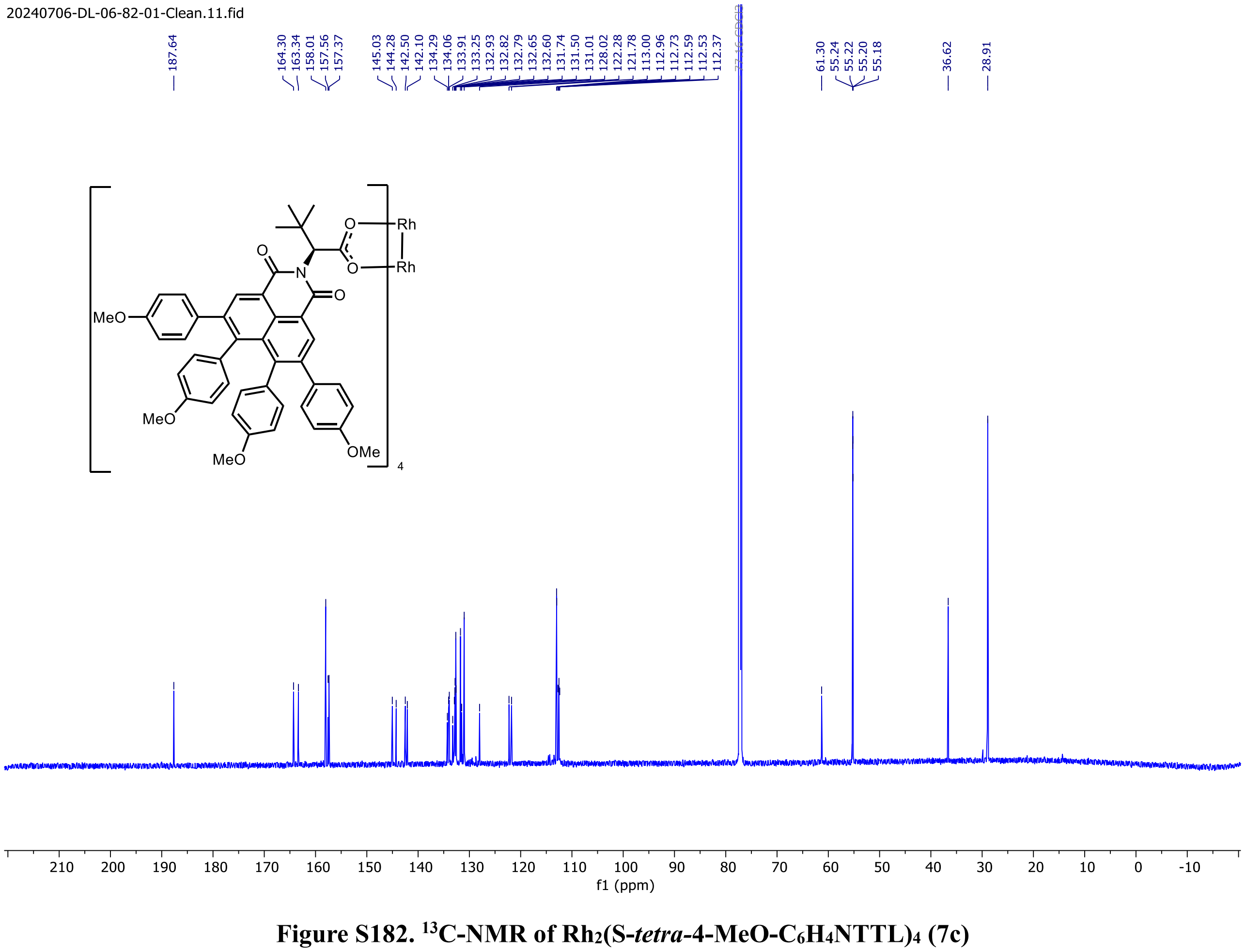

Figure S182. ^{13}C-NMR of Rh_2(S-*tetra*-4-MeO-C_6H_4NTTL)$_4$ (7c)

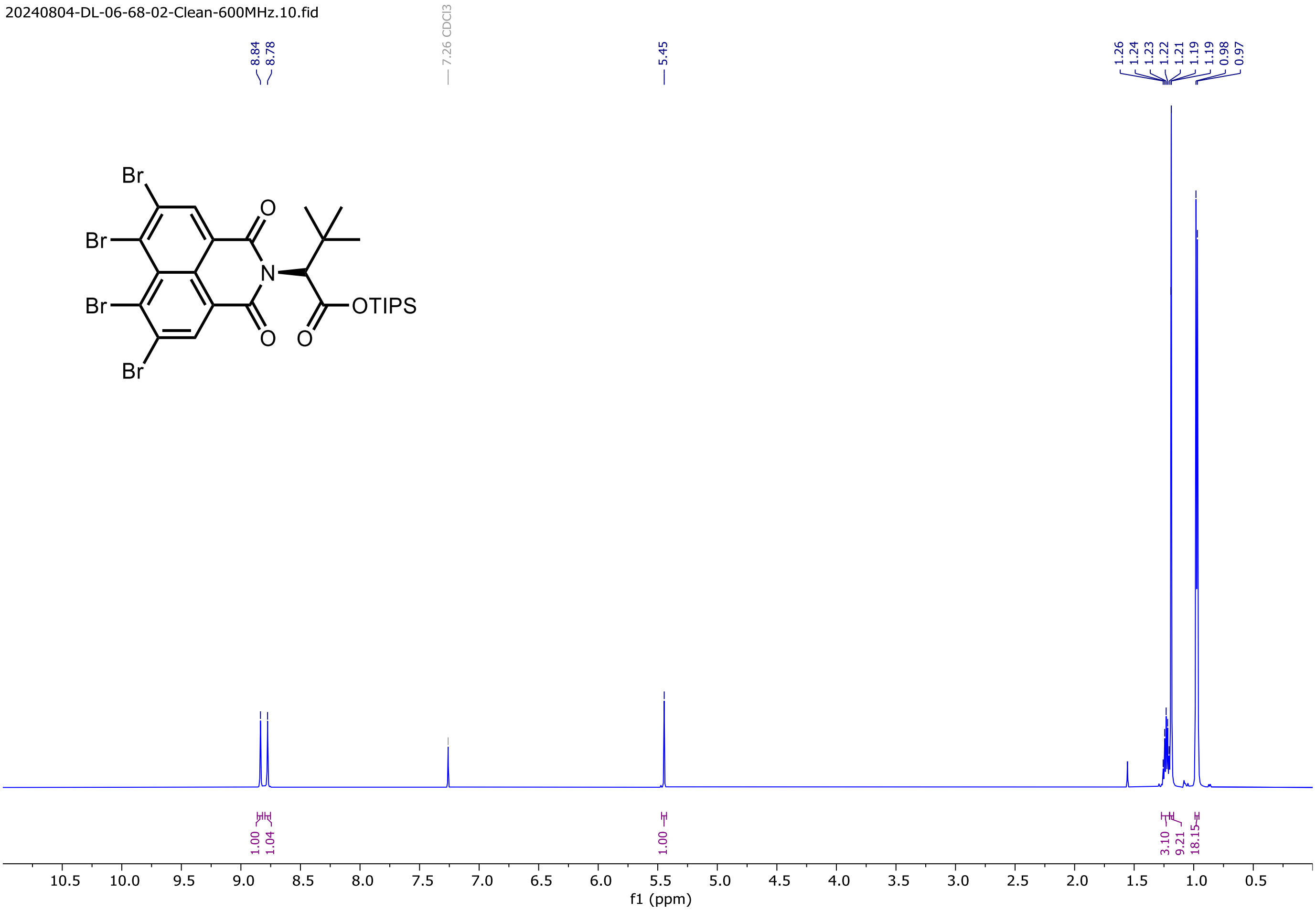

Figure S183. ^{1}H-NMR of SI-35

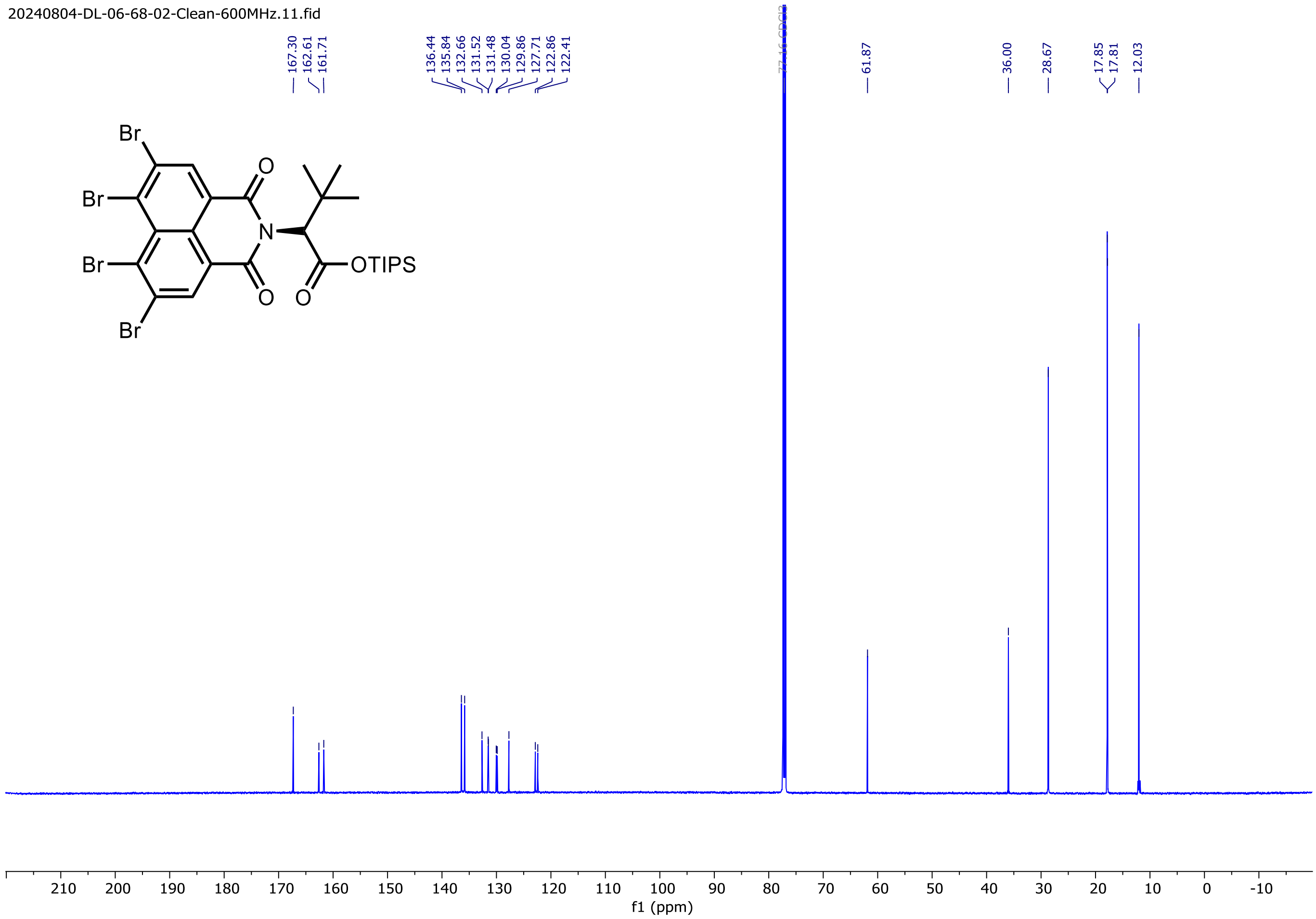

Figure S184. ^{13}C-NMR of SI-35

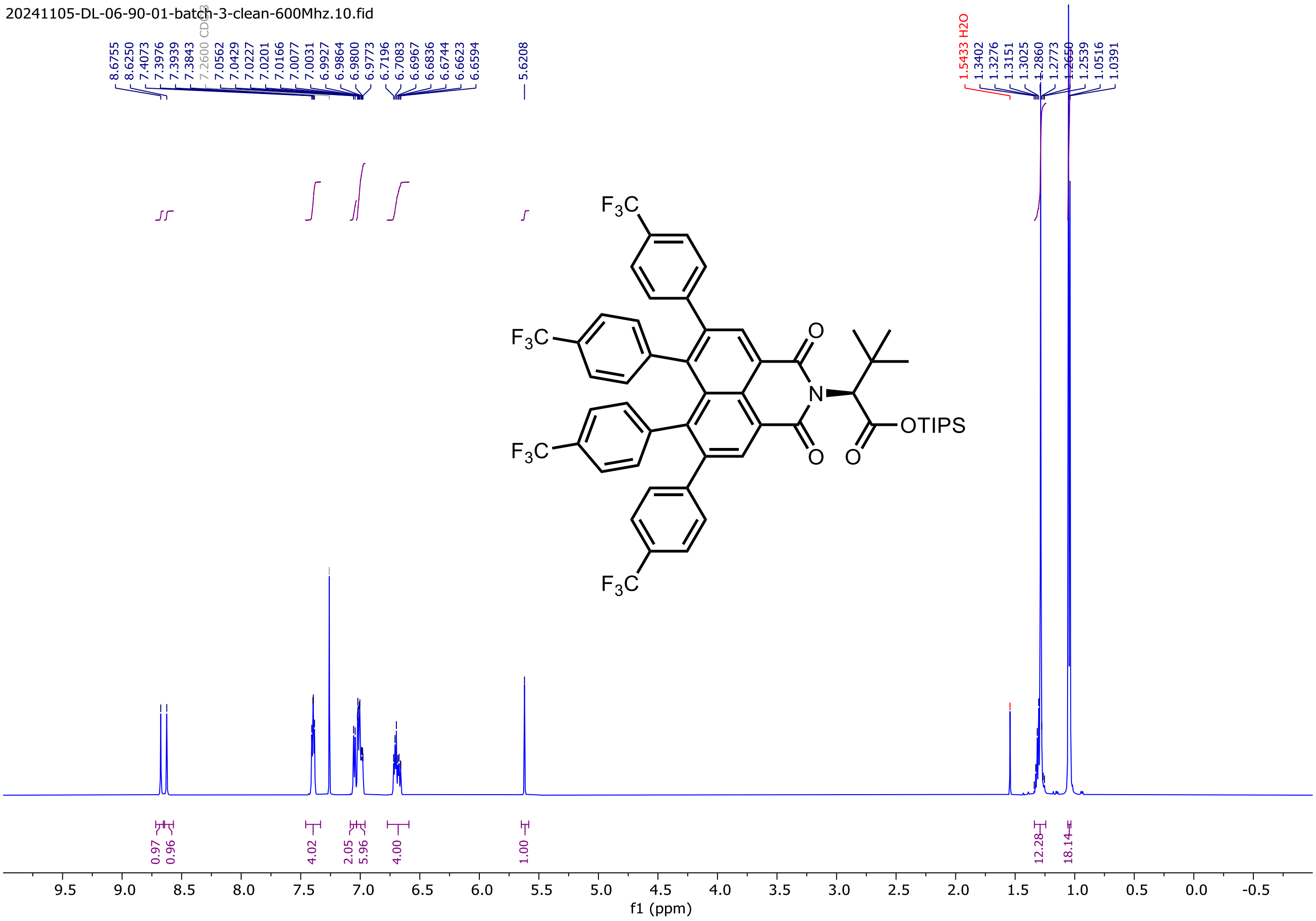

Figure S185. ^{1}H-NMR of SI-36

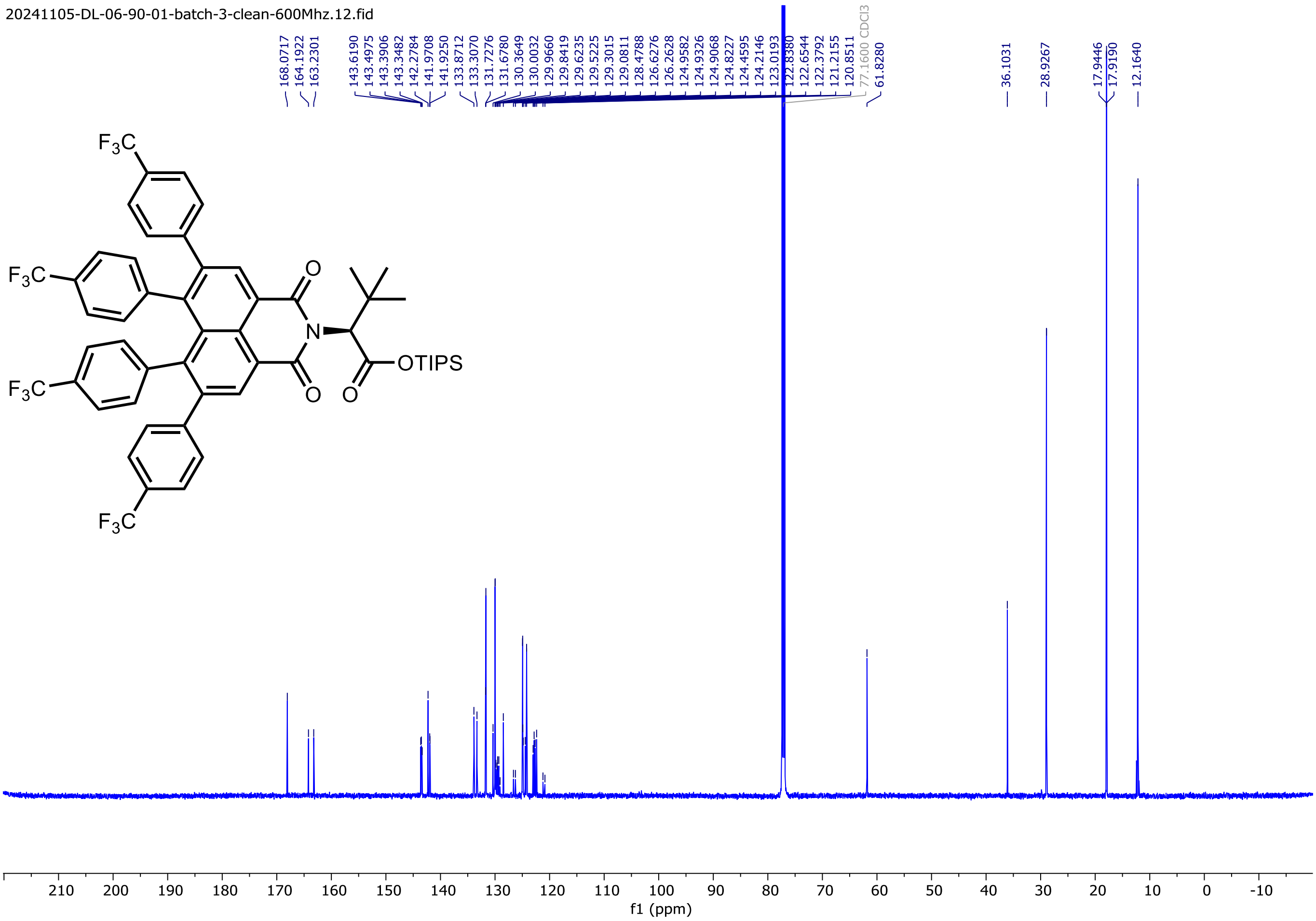

Figure S186. ^{13}C-NMR of SI-36

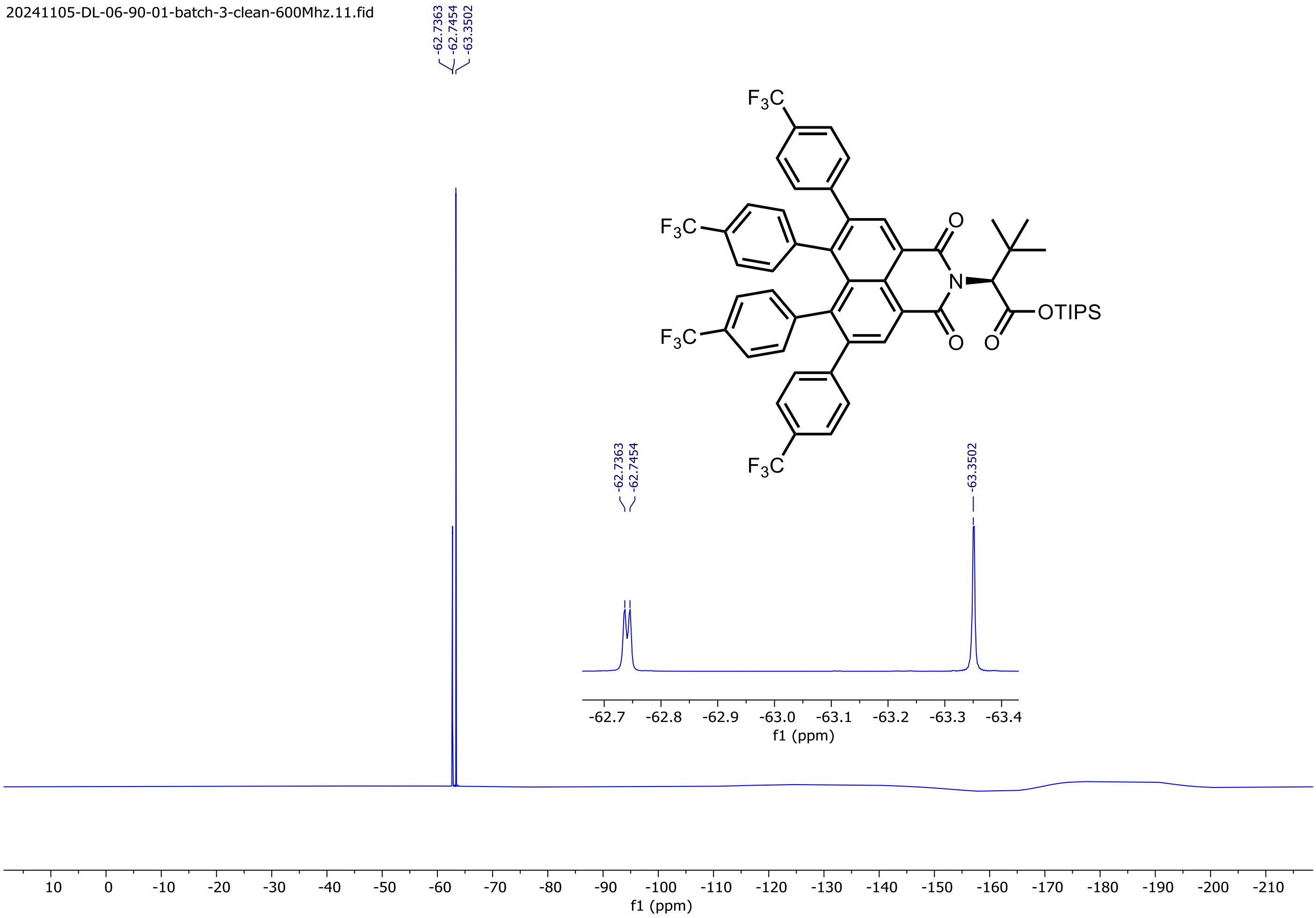

Figure S187. ^{19}F-NMR of SI-36

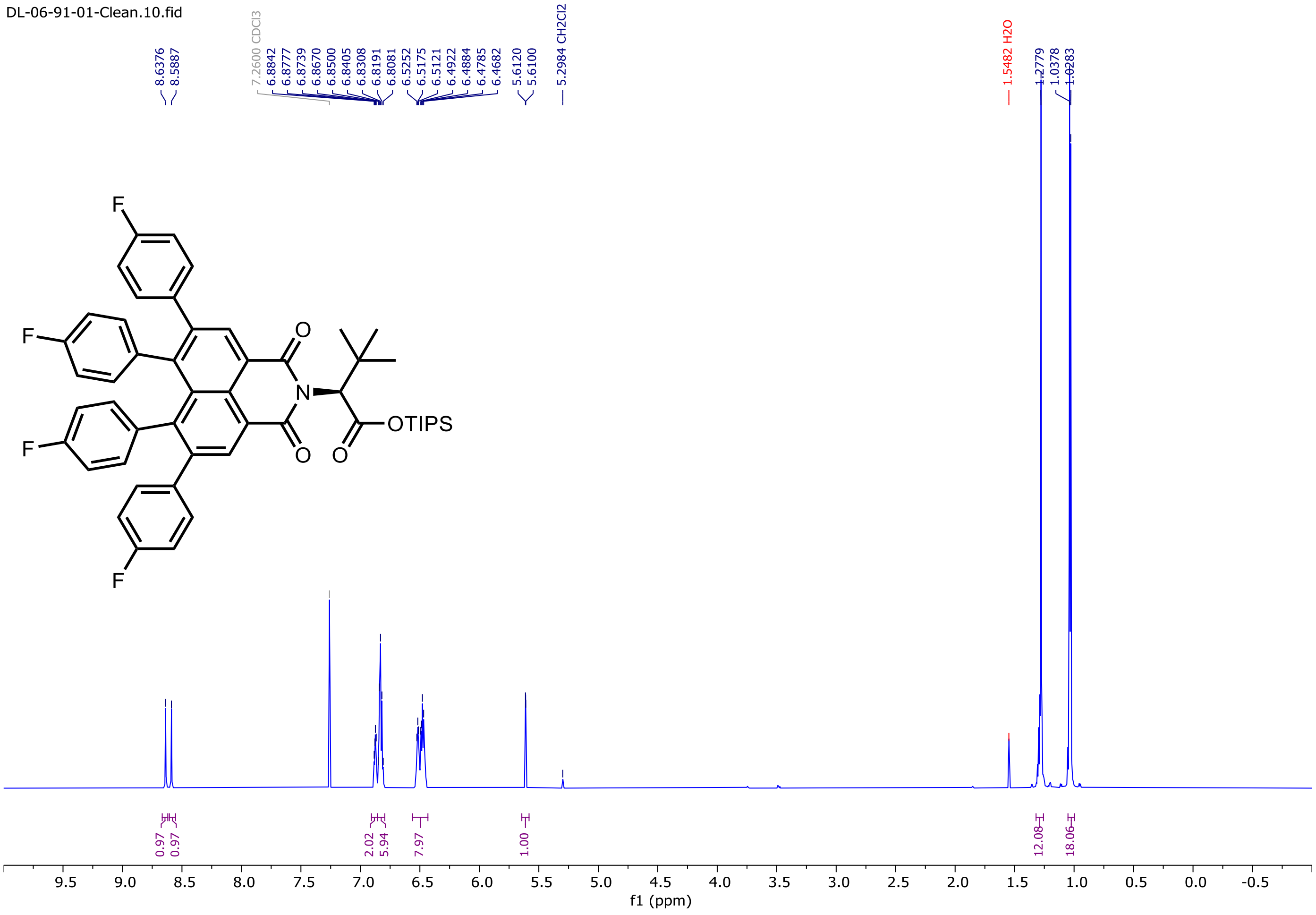

Figure S188. ^{1}H-NMR of SI-37

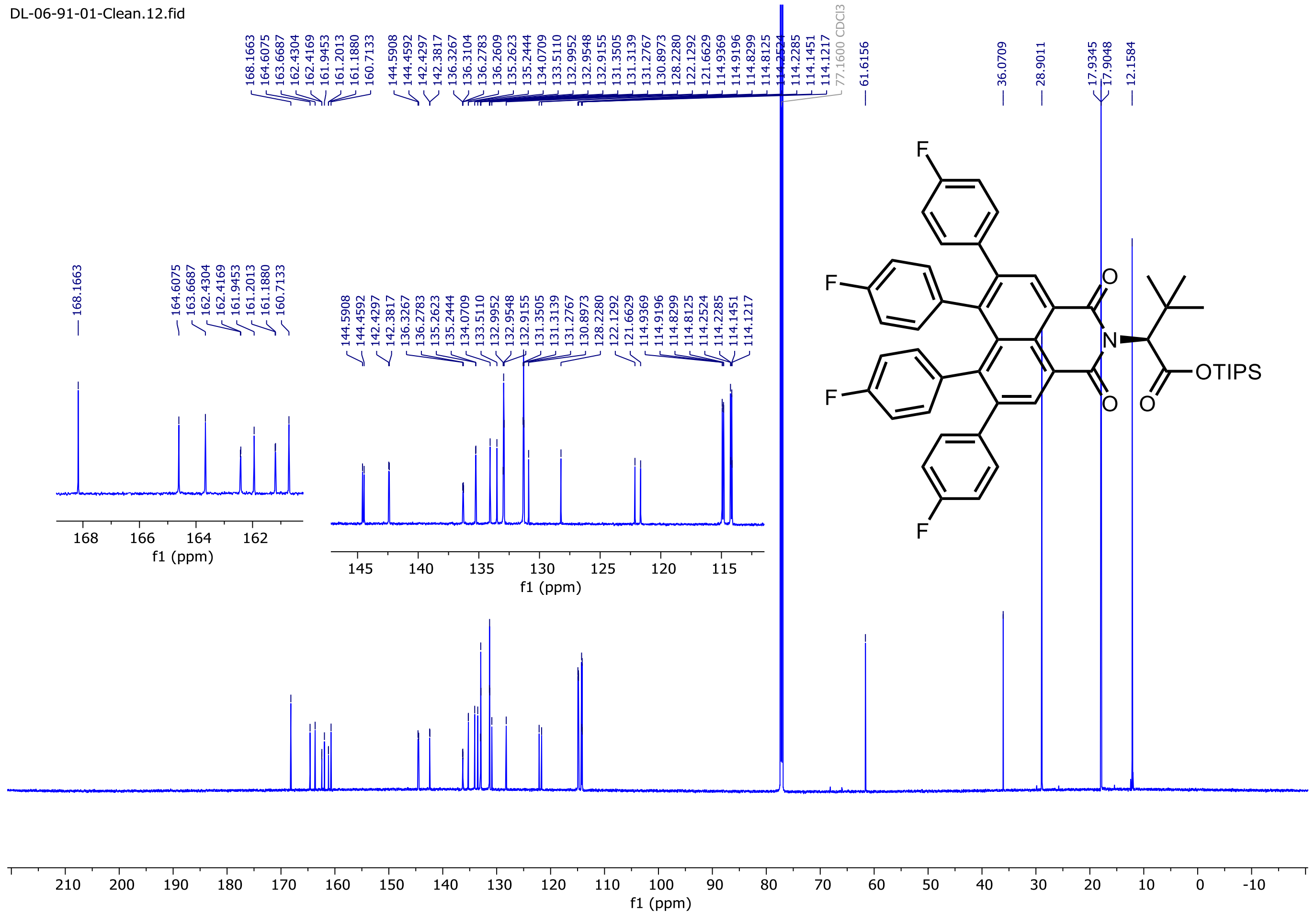

Figure S189. ^{13}C-NMR of *SI-37*

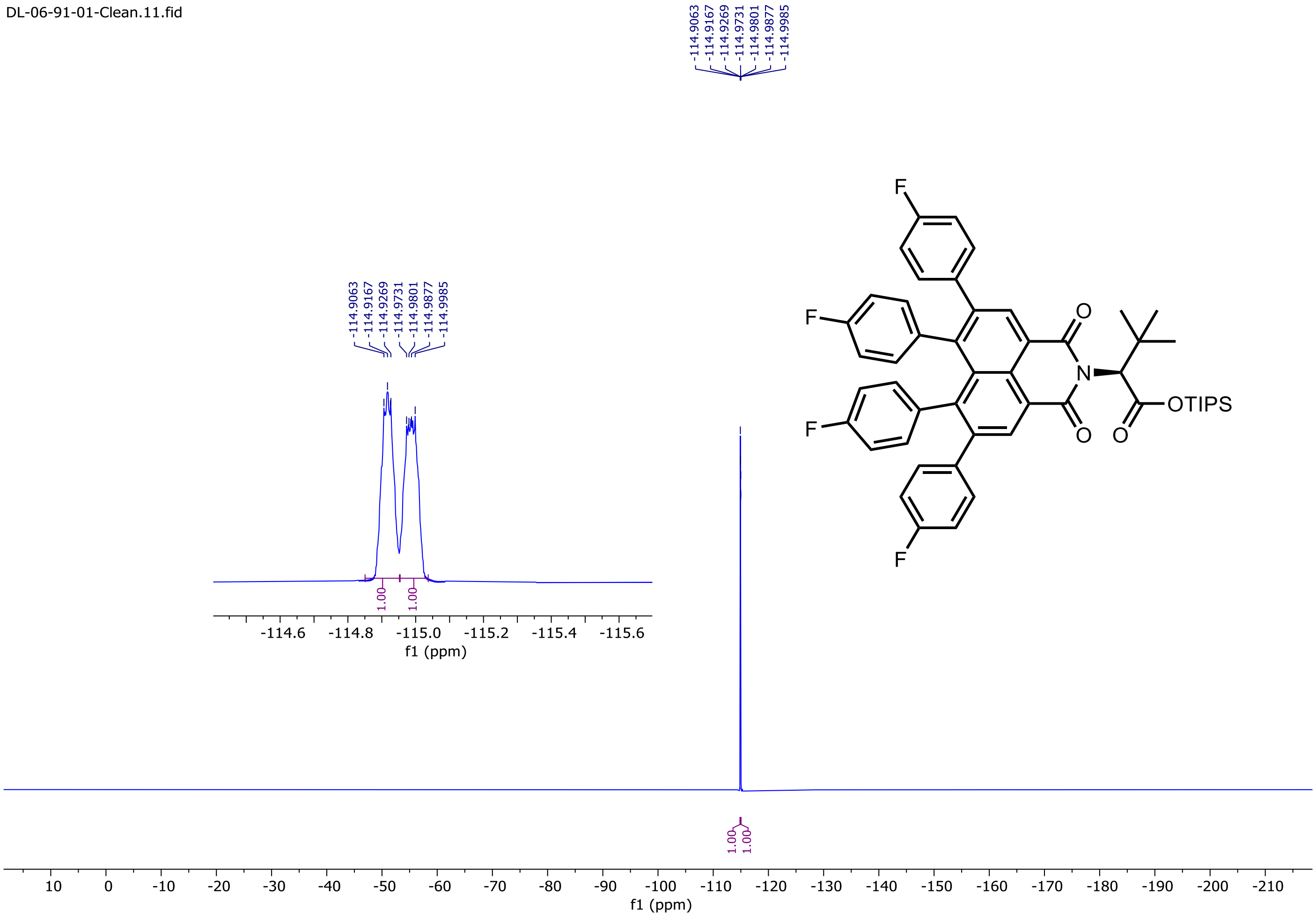

Figure S190. ^{19}F-NMR of SI-37

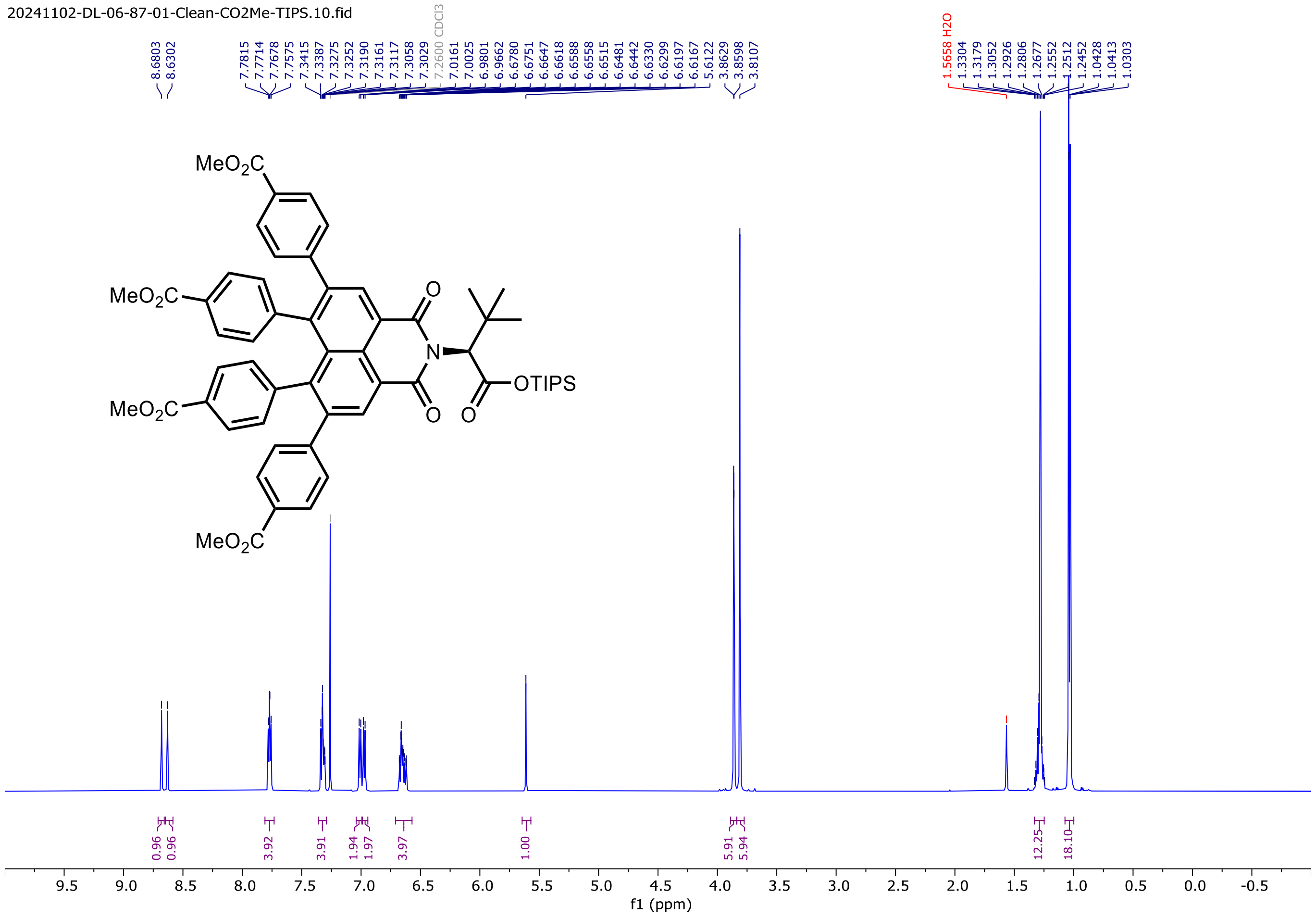

Figure S191. ^{1}**H-NMR of SI-38**

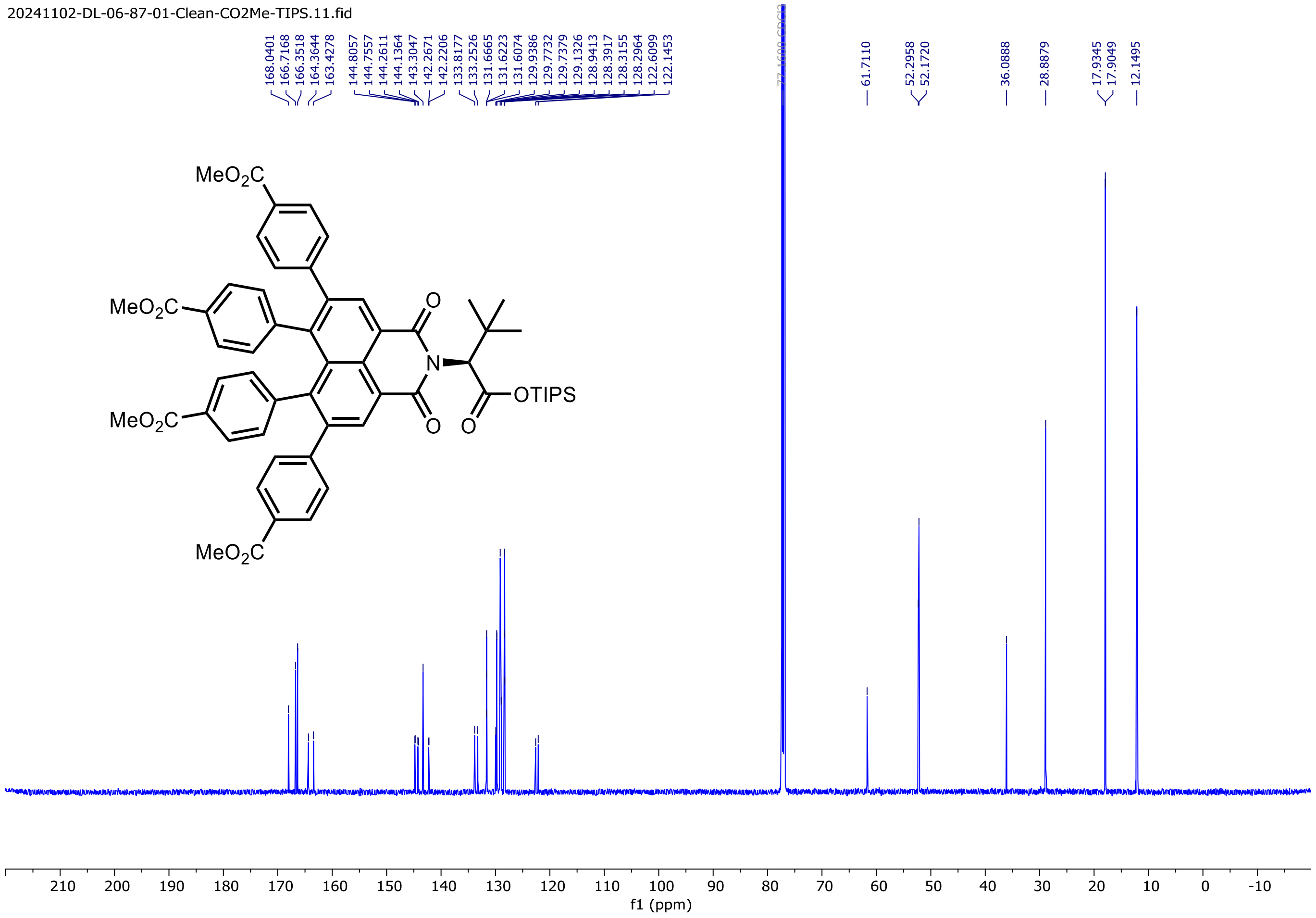

Figure S192. ^{13}**C-NMR of SI-38**

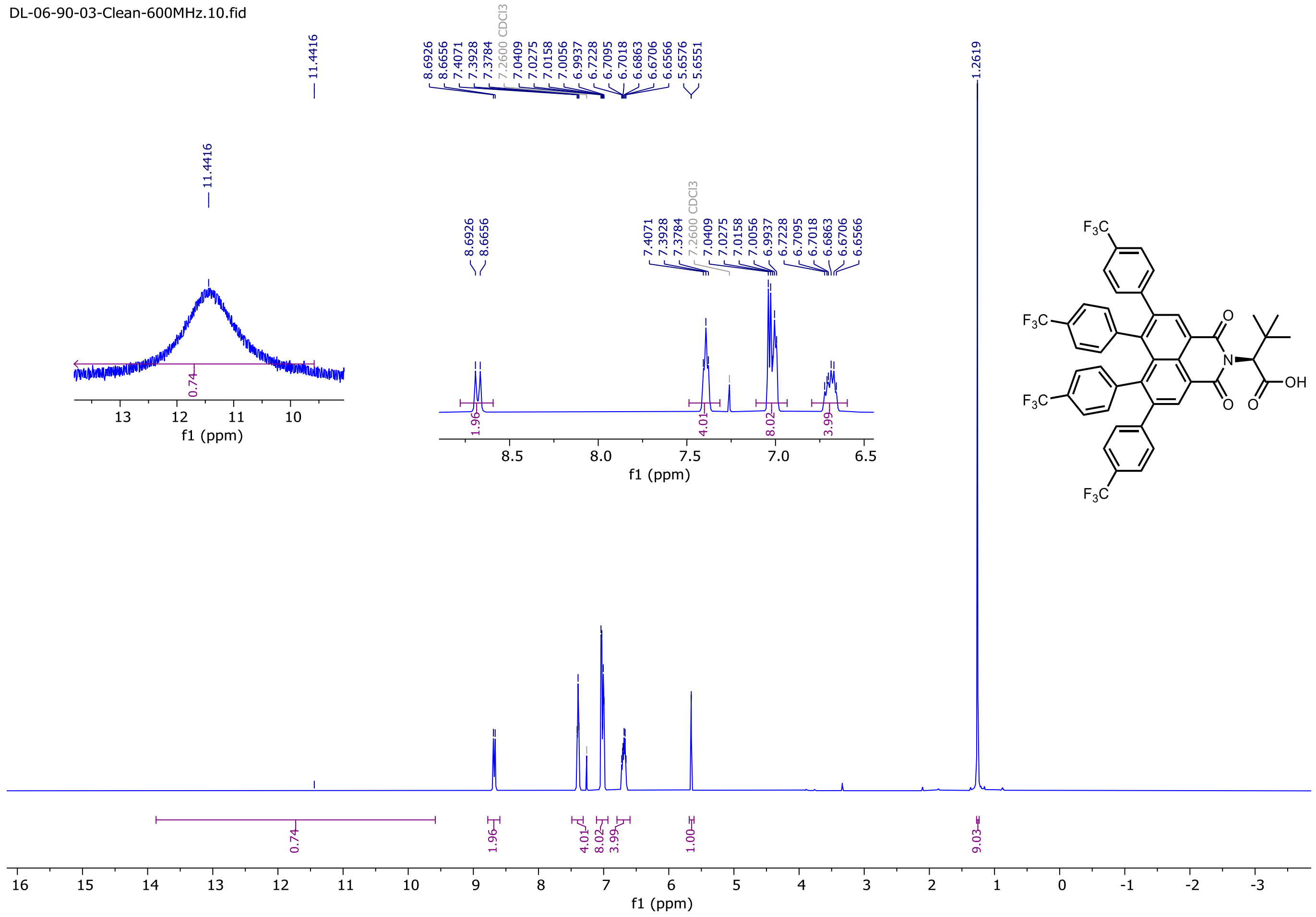

Figure S193. ^{1}H-NMR of SI-39

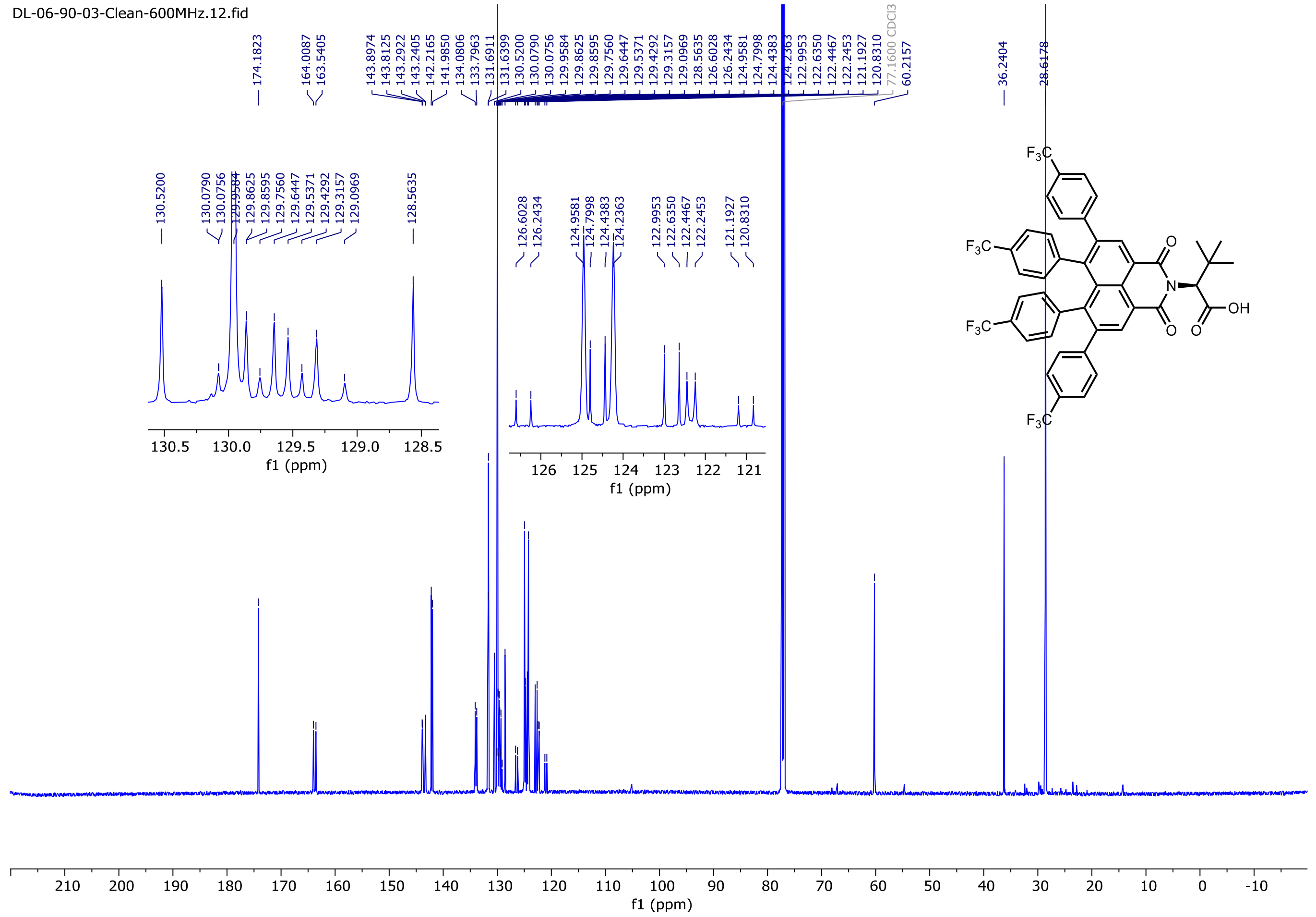

Figure S194. ^{13}C-NMR of SI-39

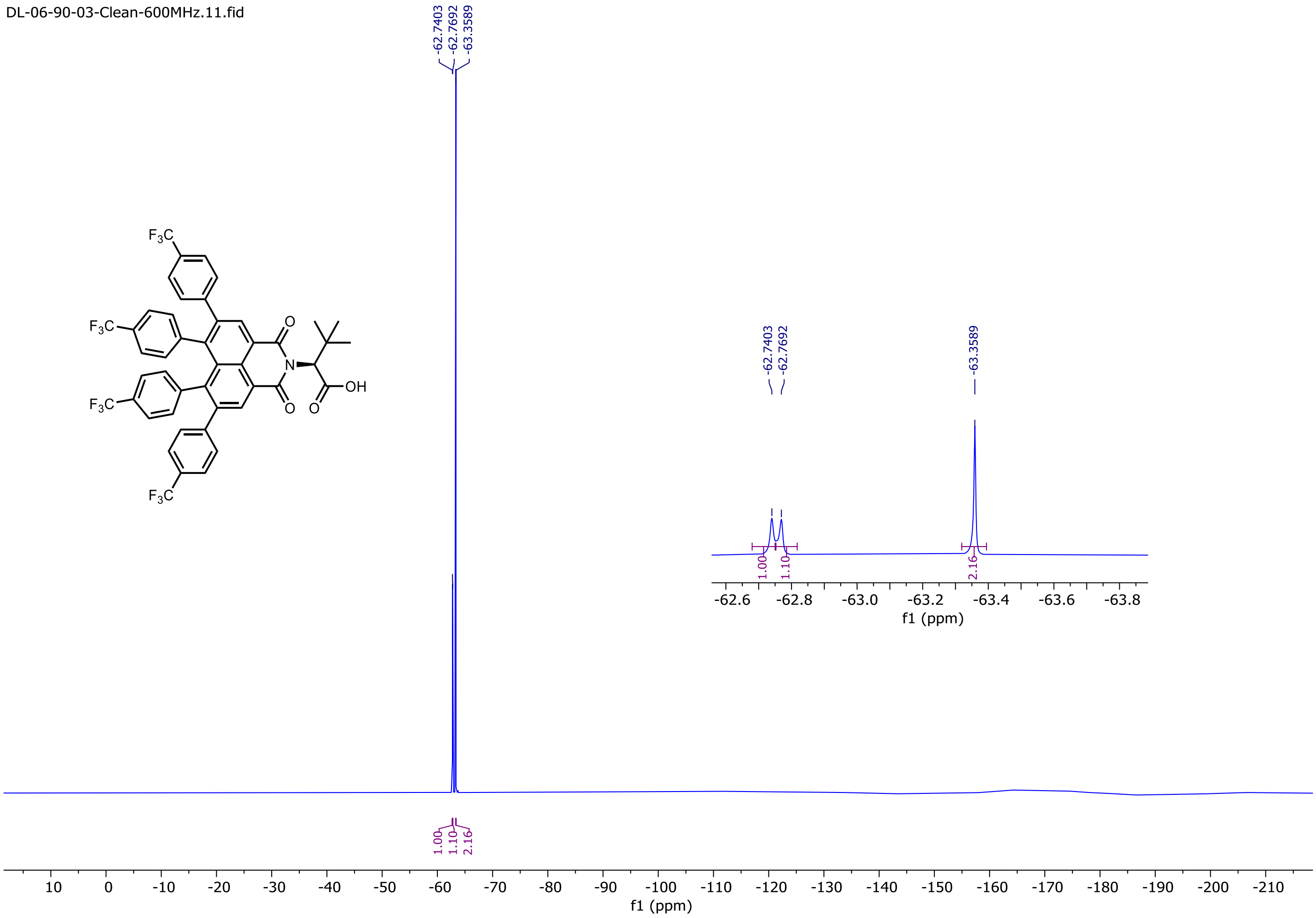

Figure S195. ^{19}F-NMR of SI-39

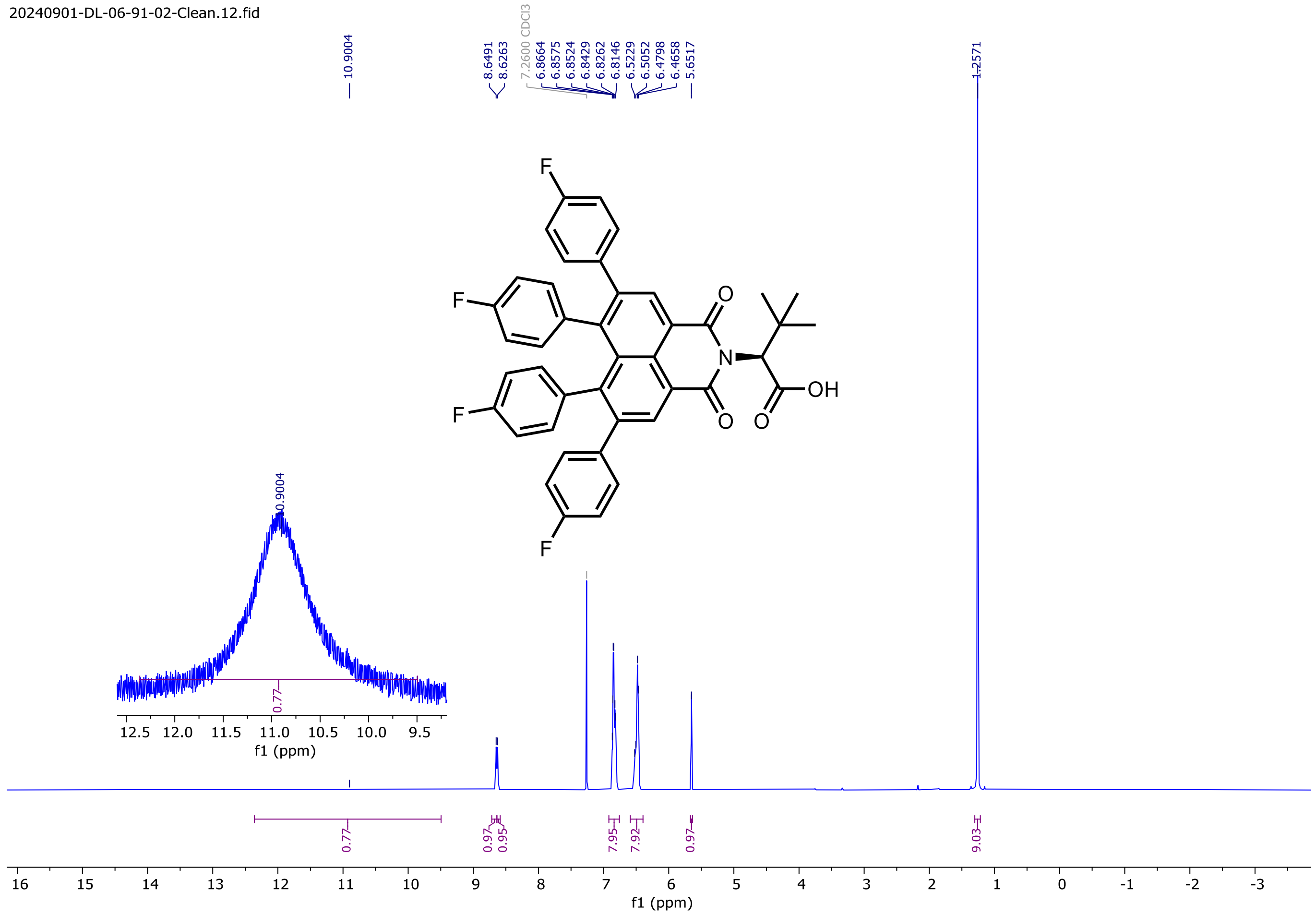

Figure S196. ^{1}H-NMR of SI-40

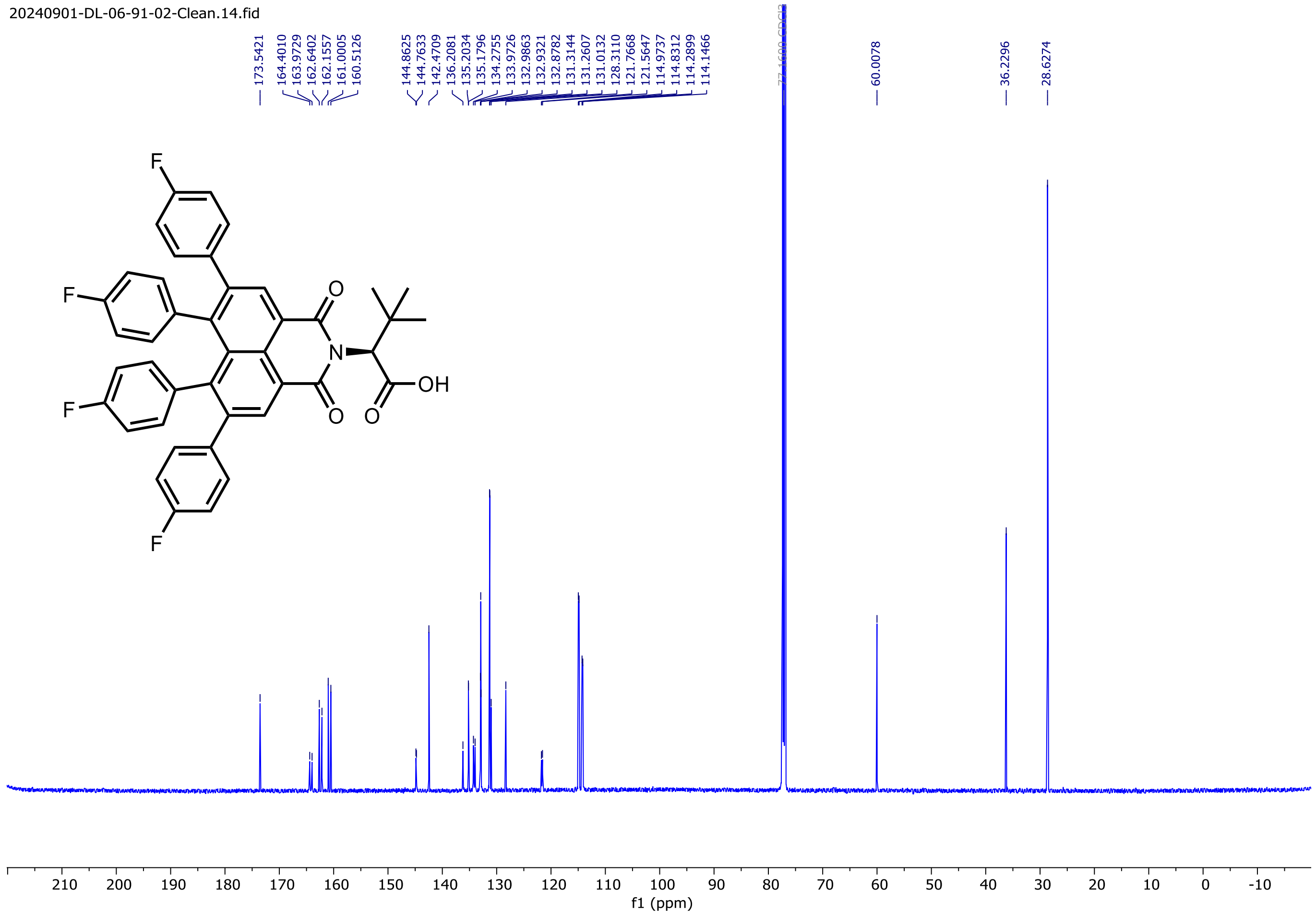

Figure S197. ^{13}C-NMR of SI-40

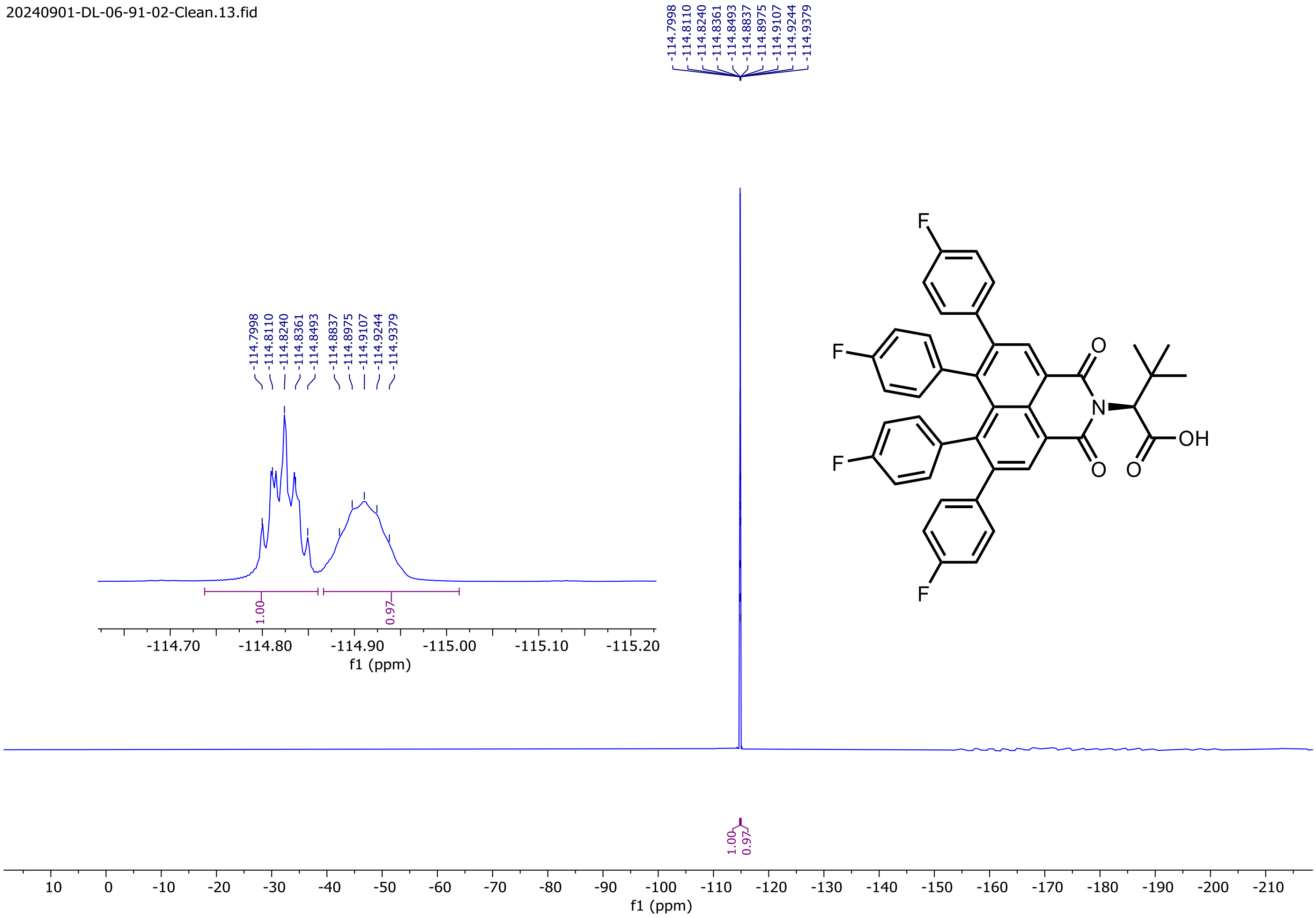

Figure S198. ^{19}F-NMR of SI-40

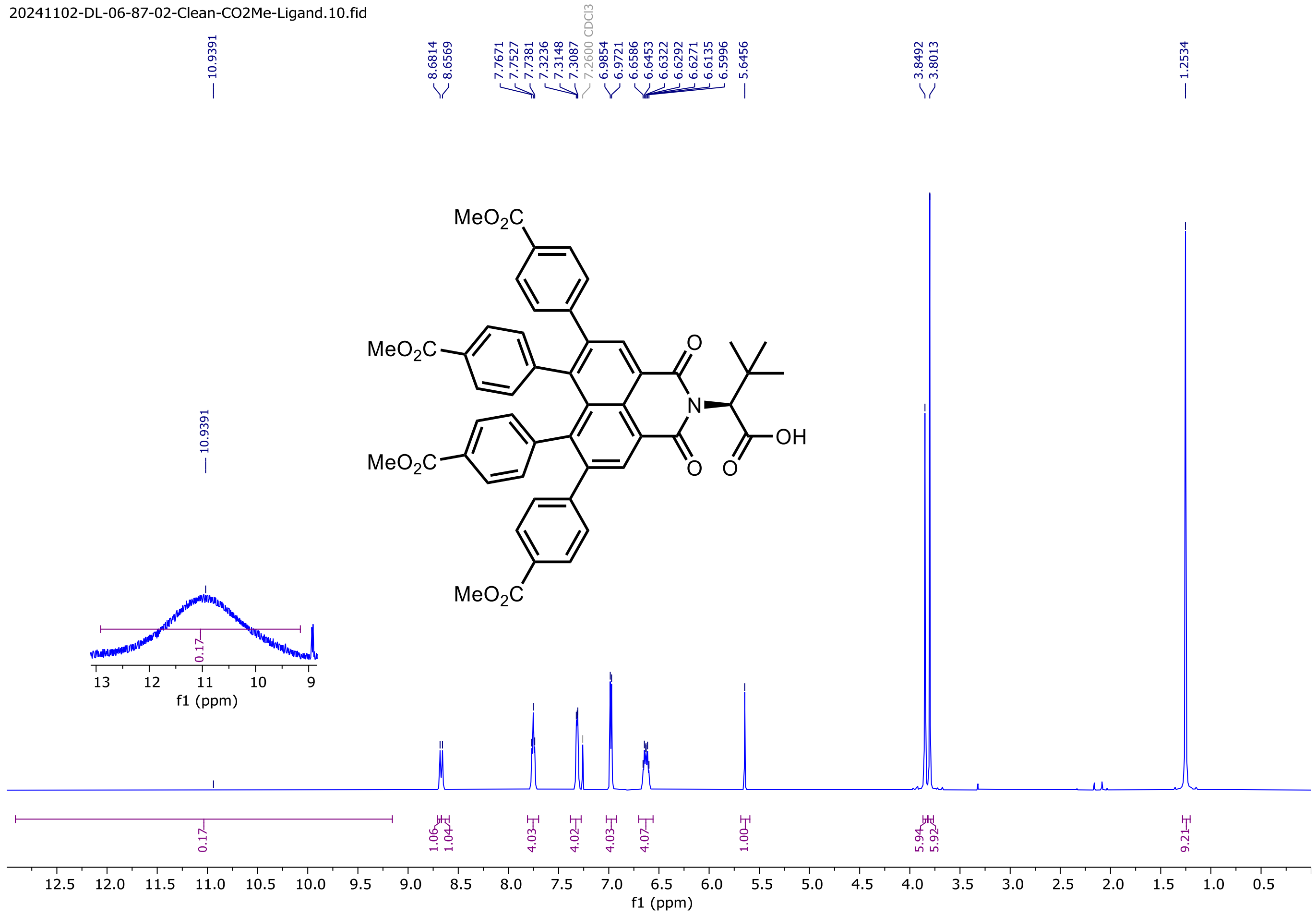

Figure S199. ^{1}H-NMR of SI-41

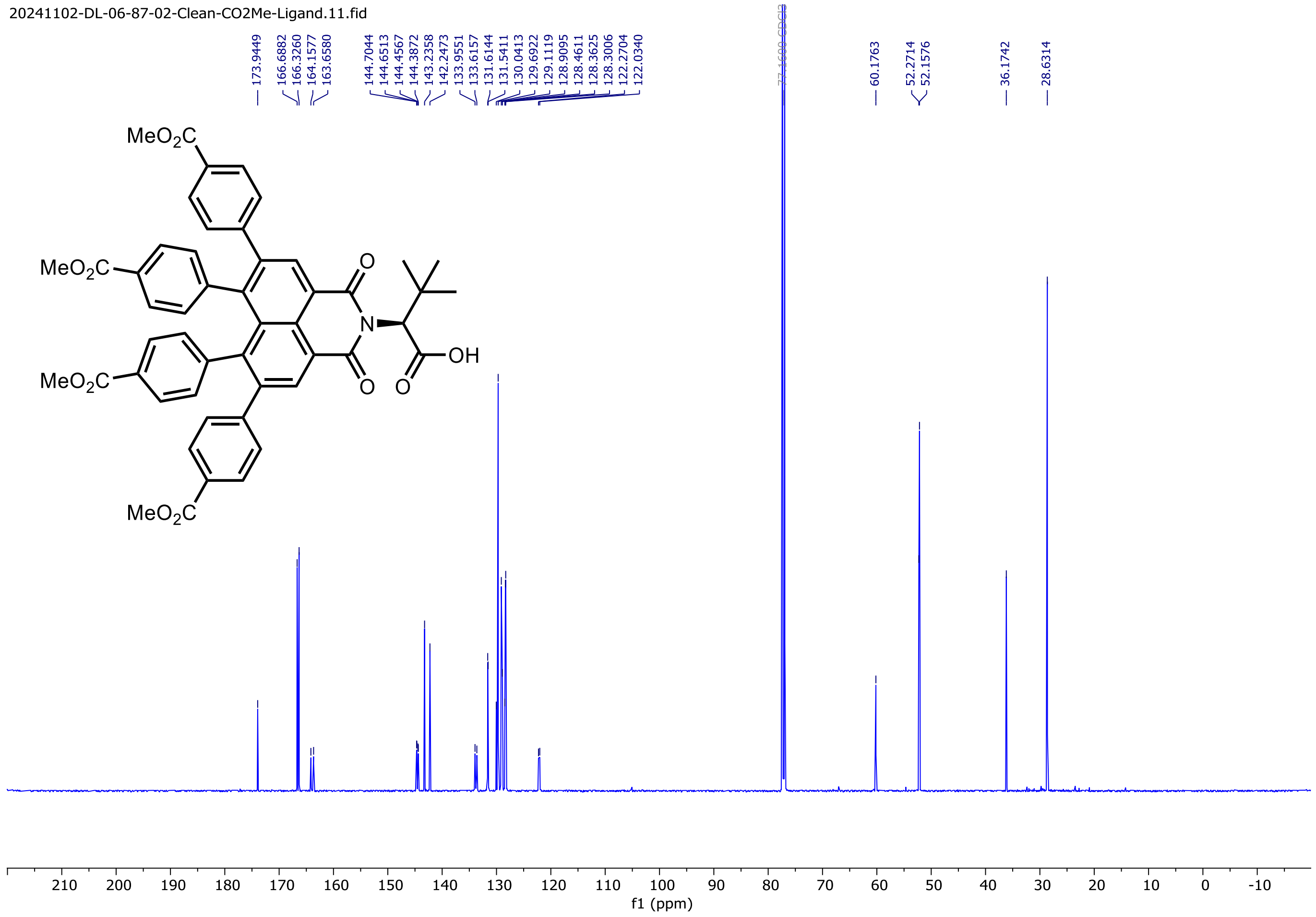

Figure S200. ^{13}C-NMR of SI-41

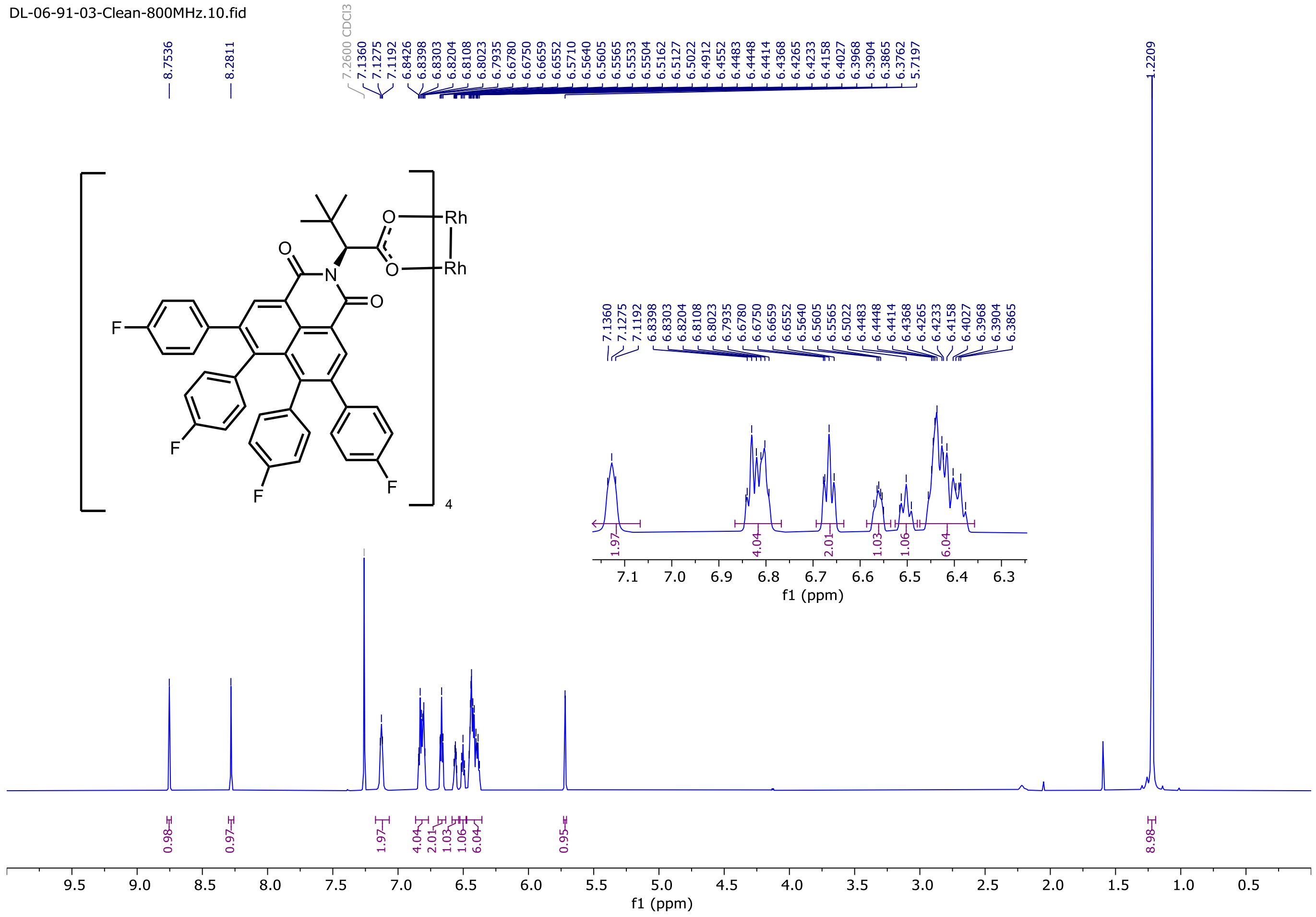

Figure S201. ^{1}H-NMR of Rh_2(*S-tetra*-4-FC_6H_4NTTL)$_4$ (7d)

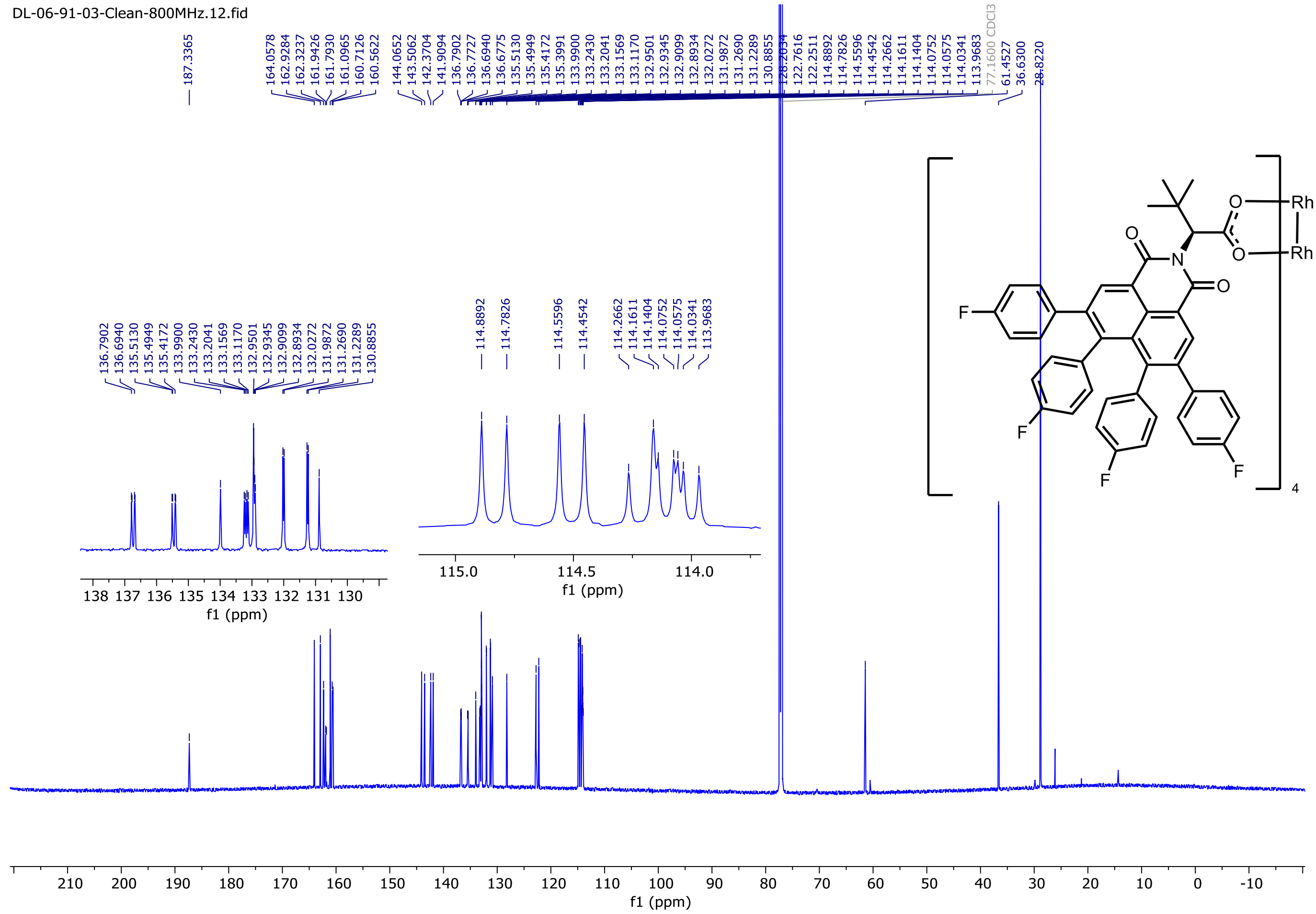

Figure S202. ^{13}C-NMR of Rh_2(*S-tetra*-4-FC_6H_4NTTL)$_4$ (7d)

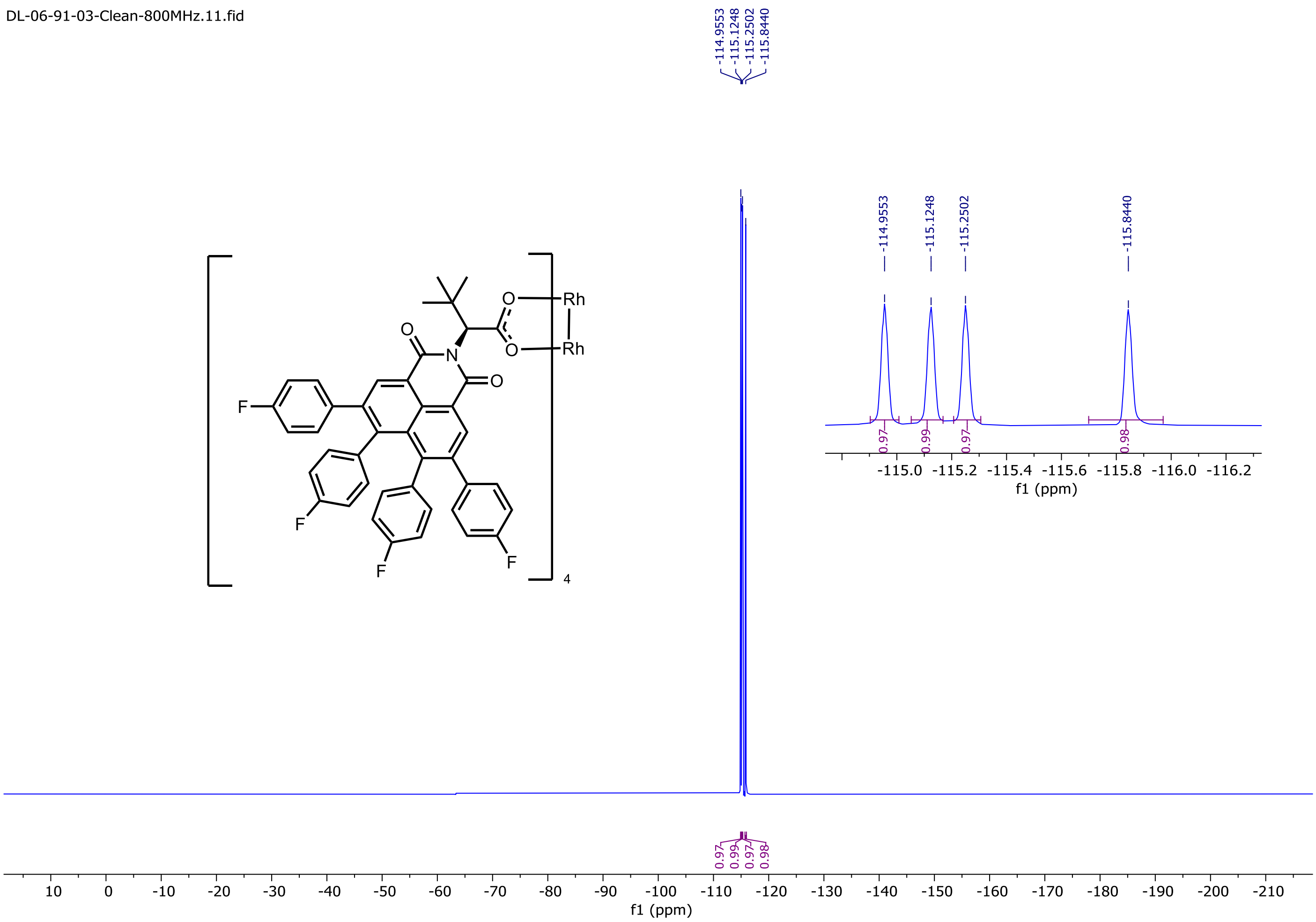

Figure S203. ^{19}F-NMR of Rh_2(*S-tetra*-4-FC_6H_4NTTL)$_4$ (7d)

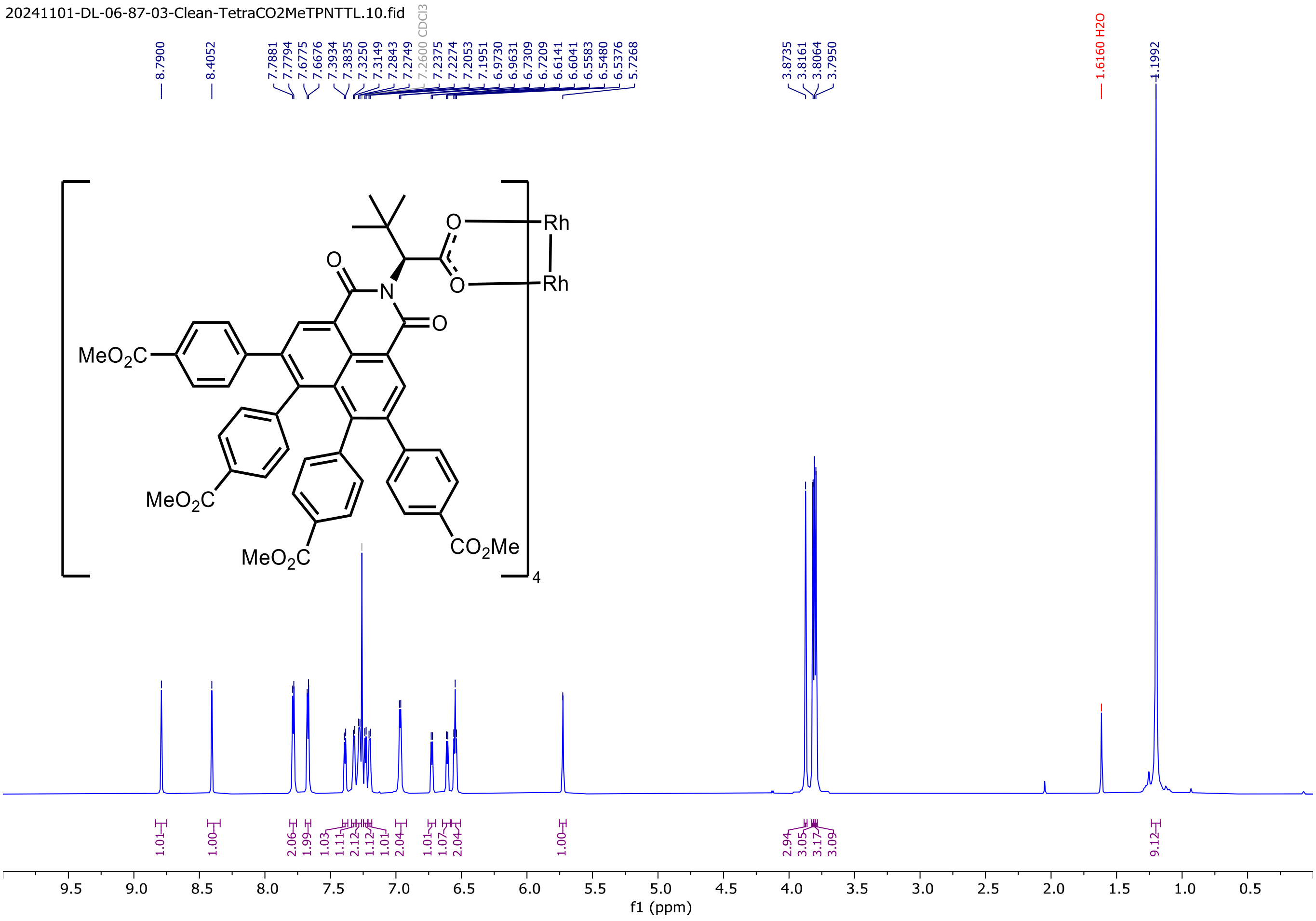

Figure S204. ^{1}**H-NMR of Rh$_2$(*S-tetra*-4-CO$_2$MeC$_6$H$_4$NTTL)$_4$ (7e)**

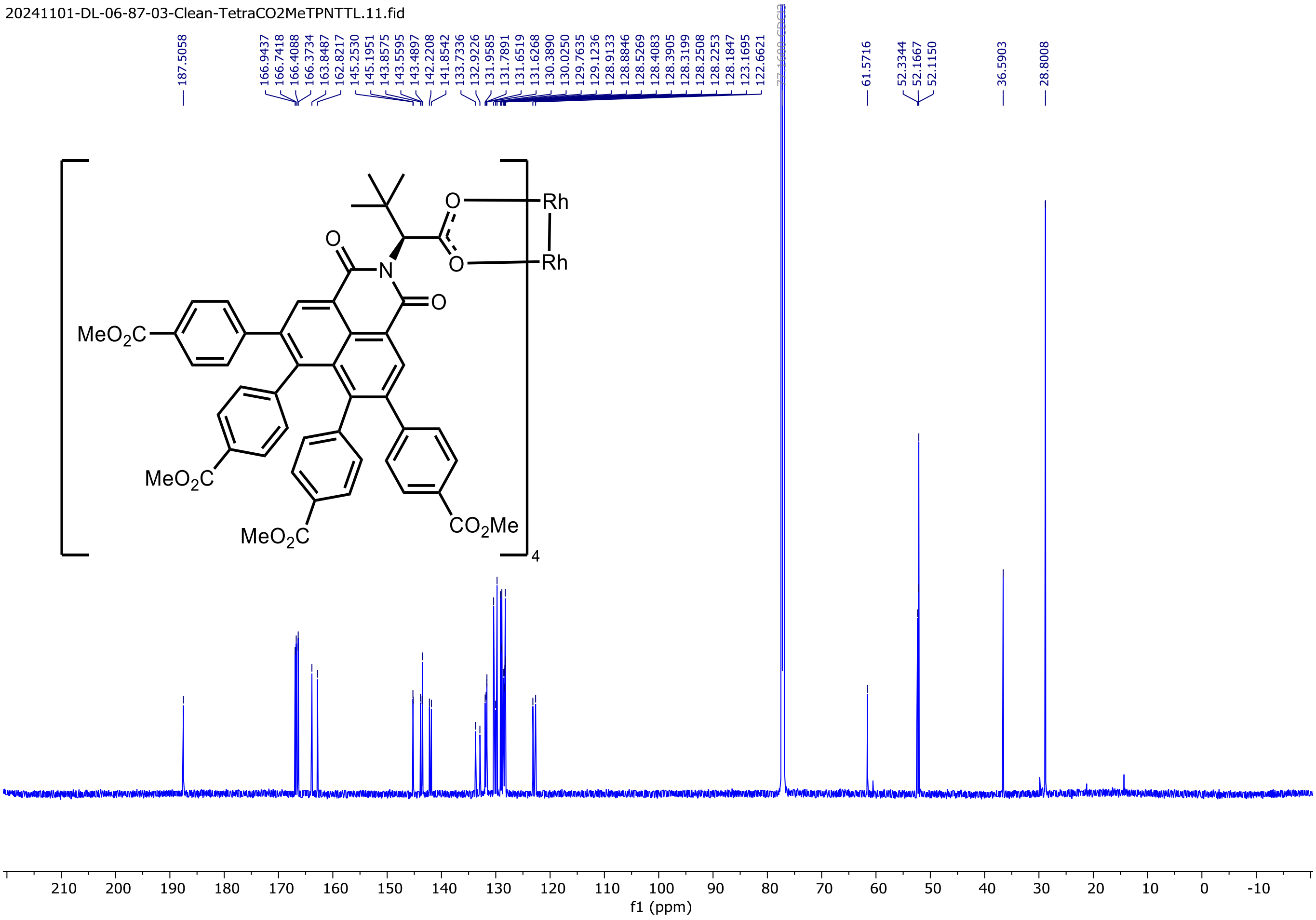

Figure S205. ^{13}**C-NMR of Rh$_2$(*S-tetra*-4-CO$_2$MeC$_6$H$_4$NTTL)$_4$ (7e)**

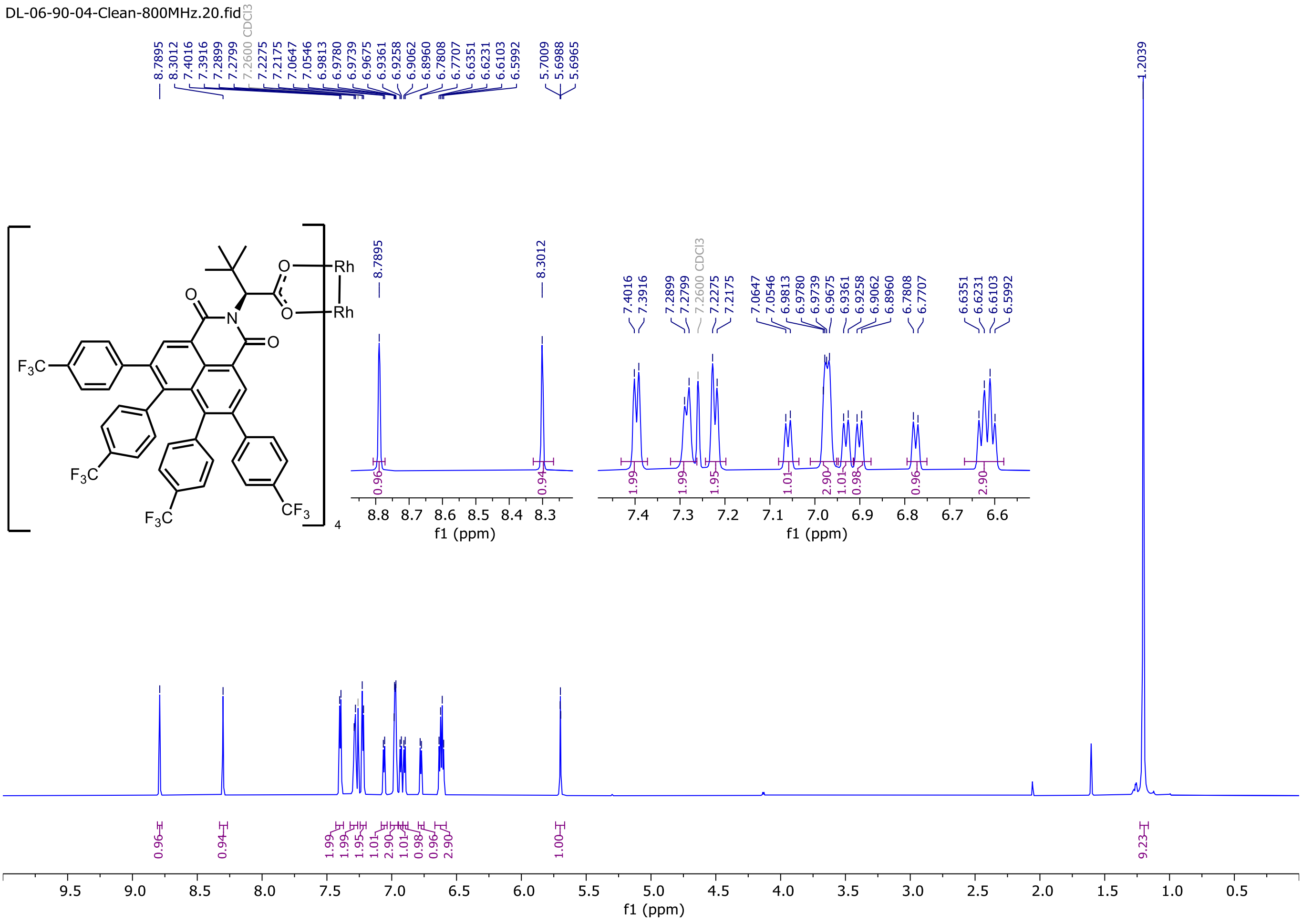

Figure S206. ^{1}H-NMR of Rh_2(*S-tetra*-4-$CF_3C_6H_4$NTTL)$_4$ (7f)

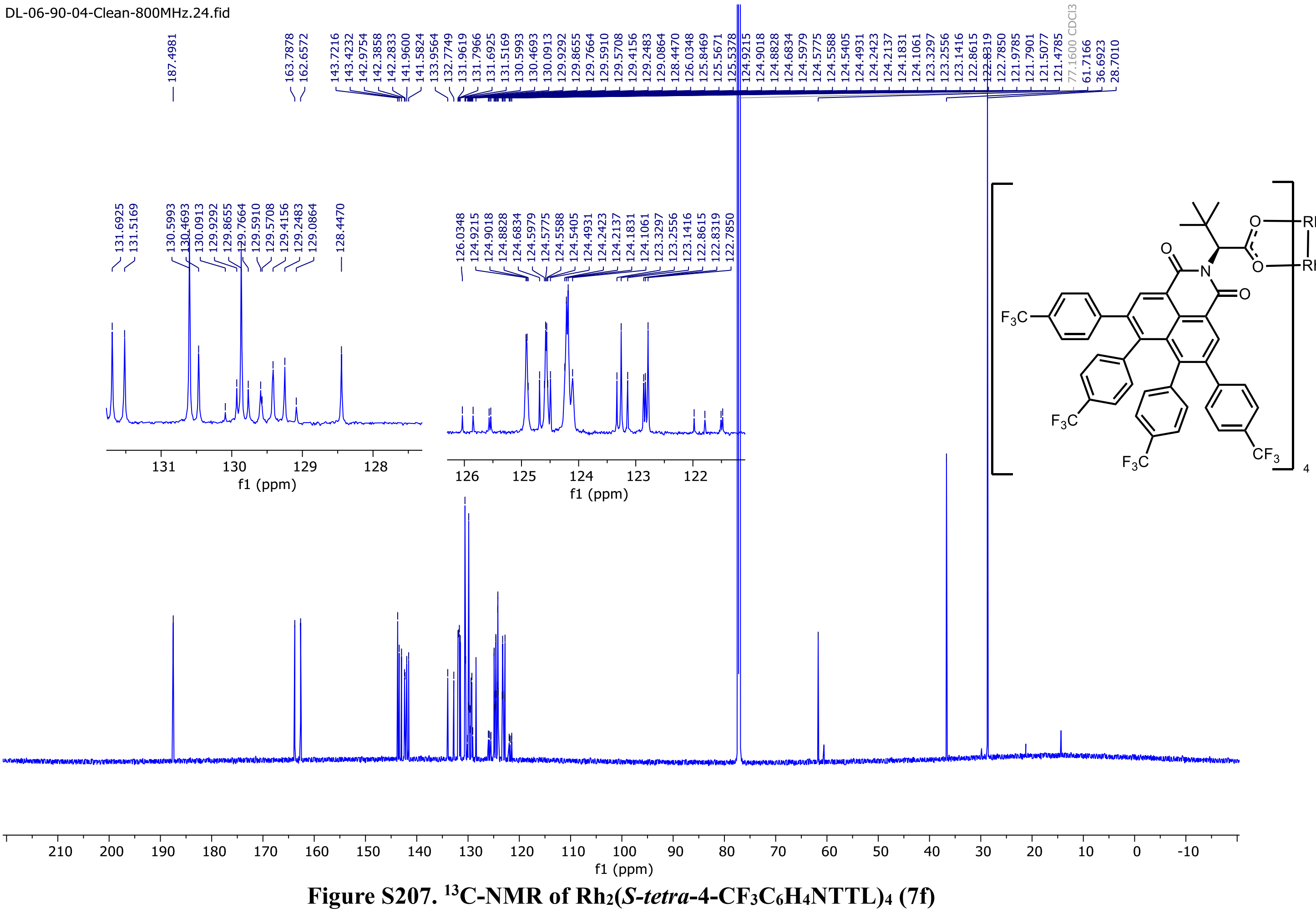

Figure S207. ^{13}C-NMR of Rh_2(*S-tetra*-4-$CF_3C_6H_4$NTTL)$_4$ (7f)

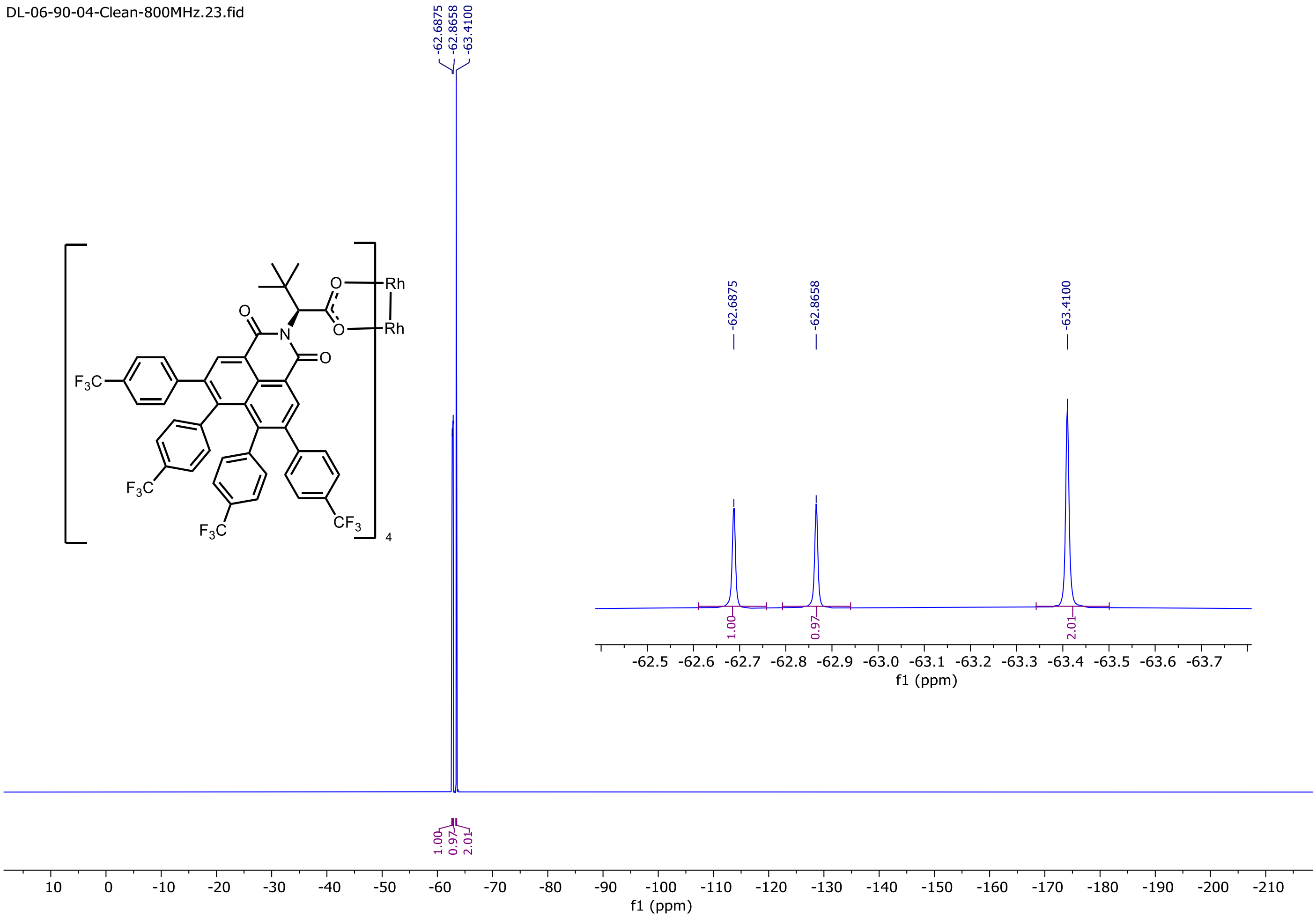

Figure S208. ^{19}F-NMR of Rh_2(*S*-*tetra*-4-$CF_3C_6H_4$NTTL)$_4$ (7f)

10.3. Spectra of C-H functionalization products

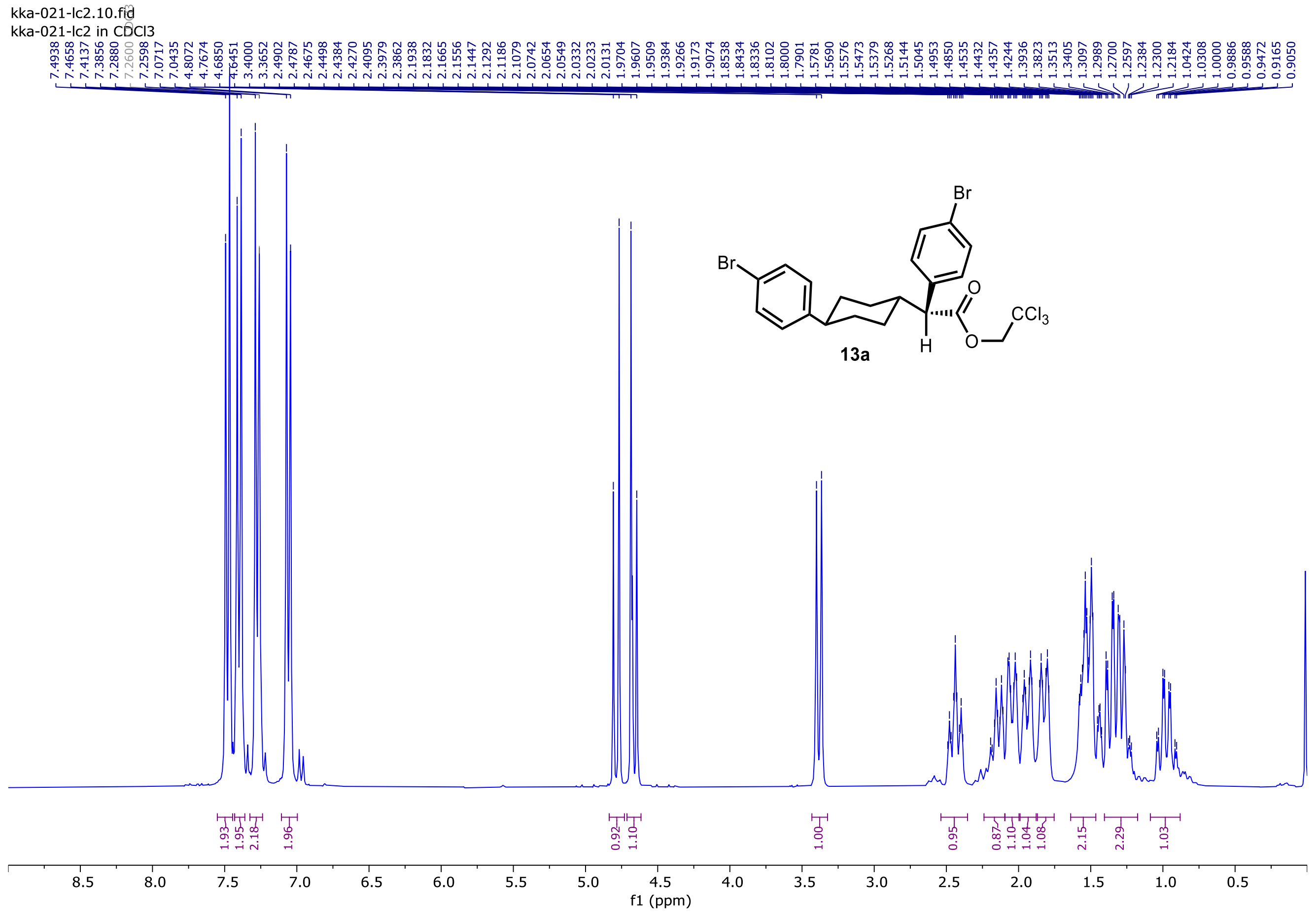

Figure S209. ^{13}**H-NMR of 13a**

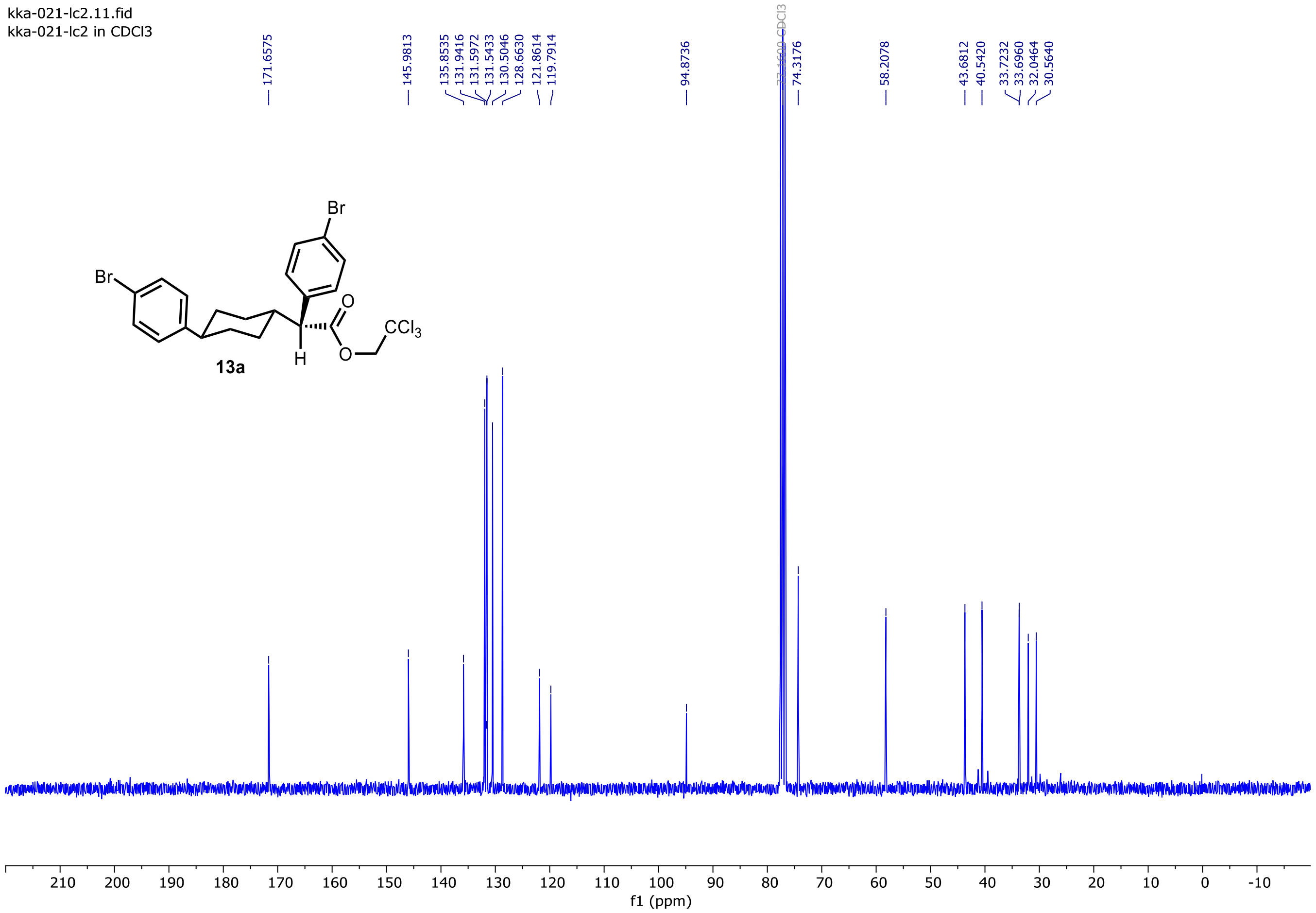

Figure S210. ^{13}**C-NMR of 13a**

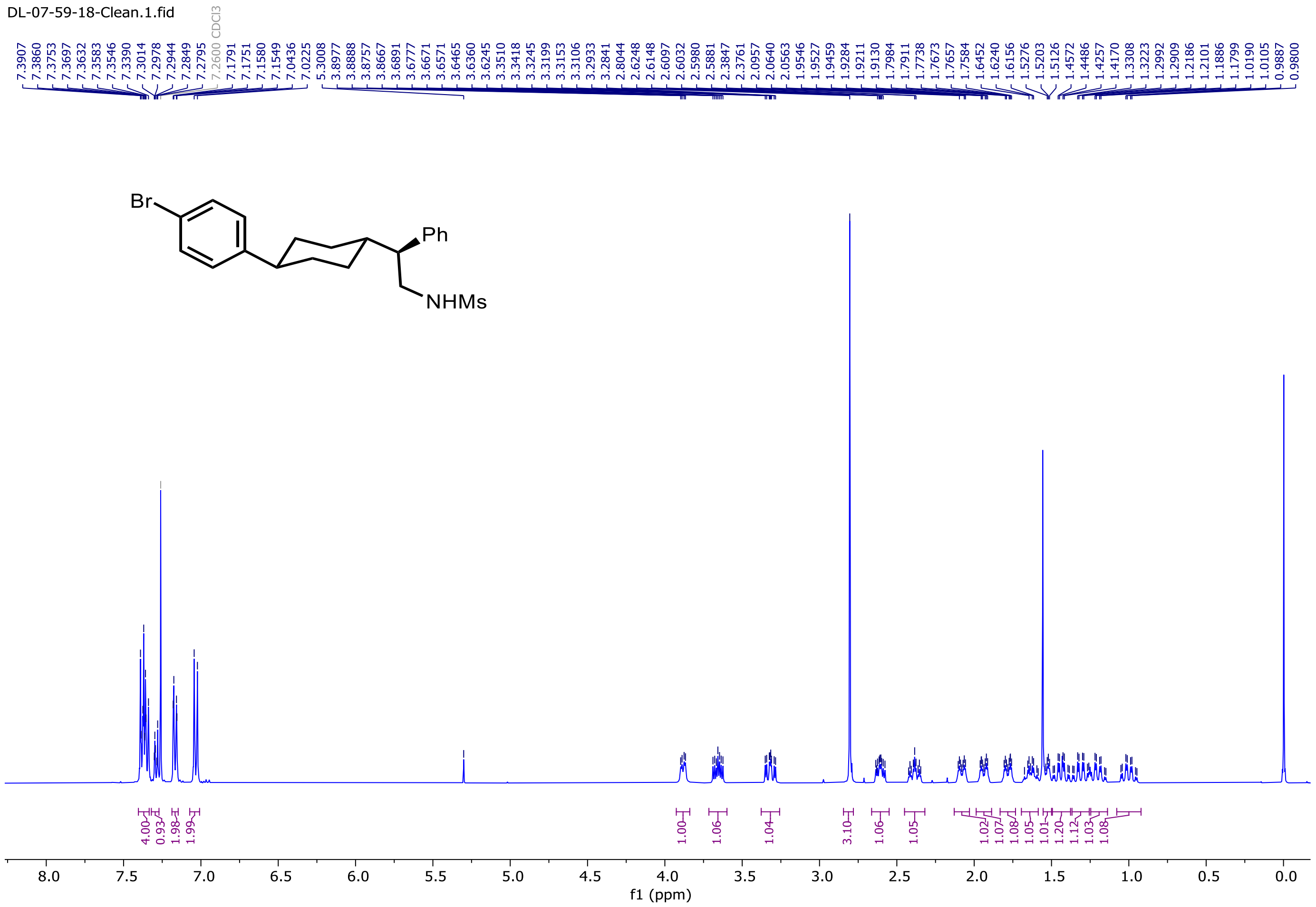

Figure S211. ^{1}H-NMR of 13

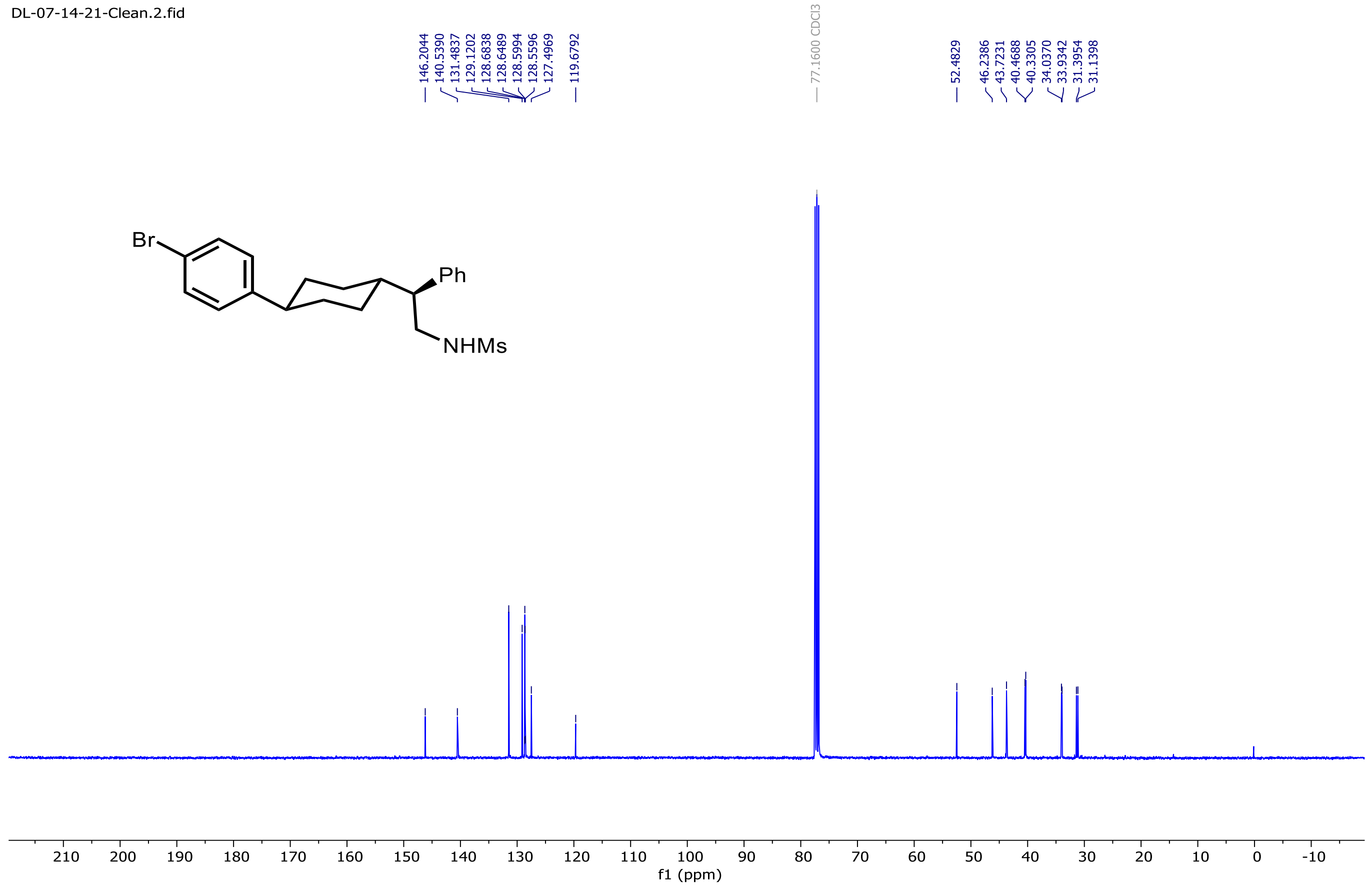

Figure S212. ^{13}C-NMR of 13

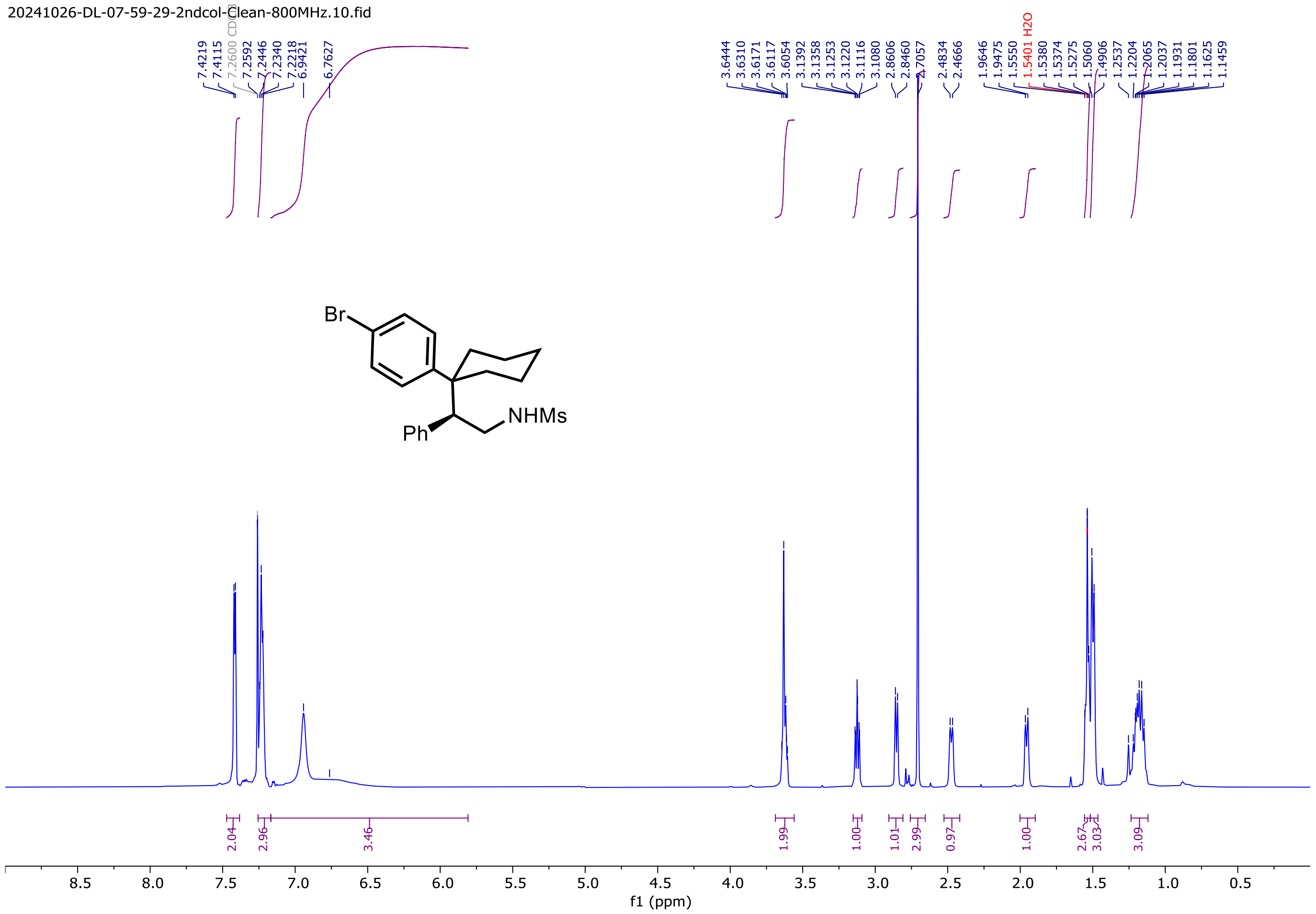

Figure S213. ^{1}H-NMR of 14

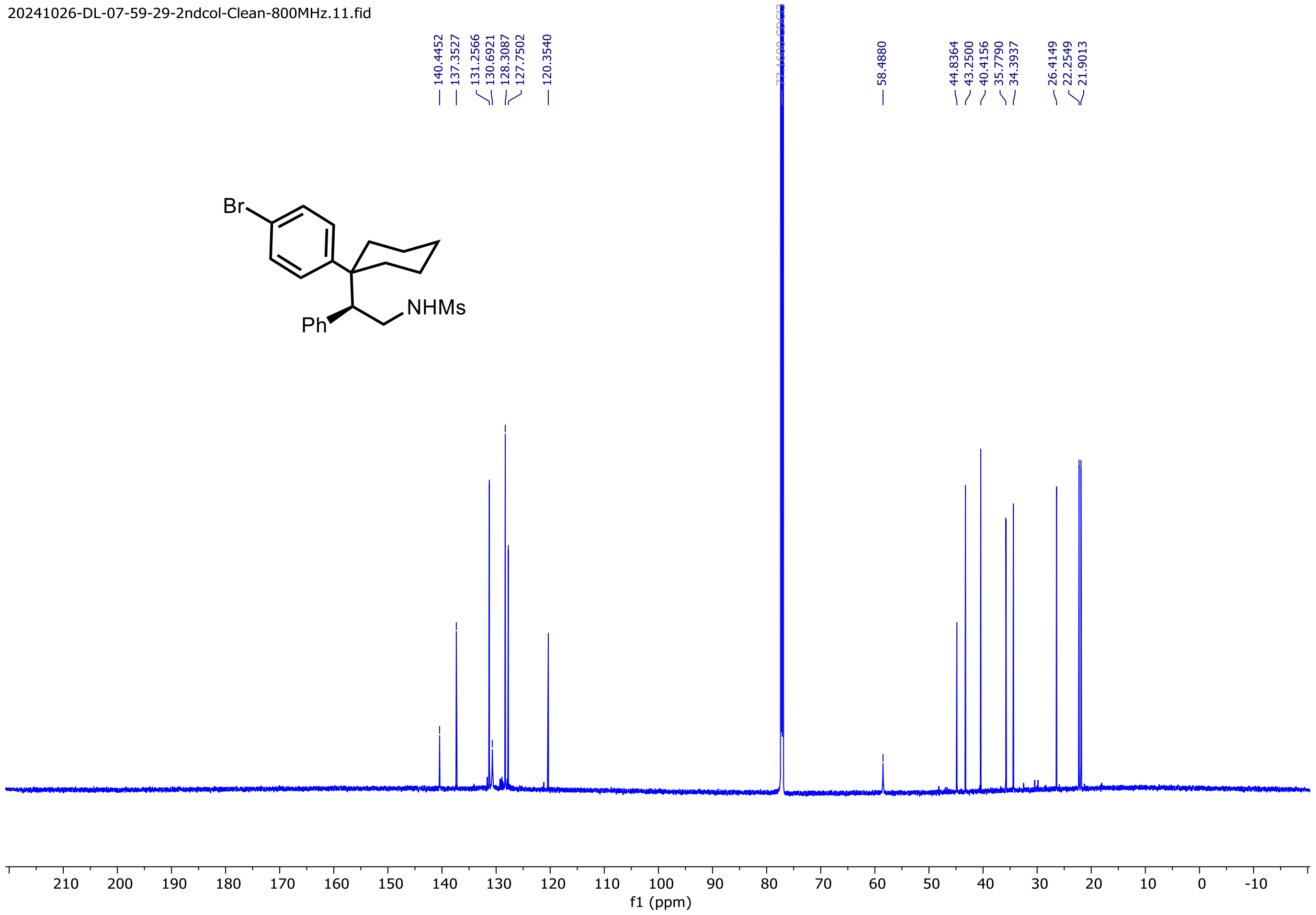

Figure S214. ^{13}C-NMR of 14

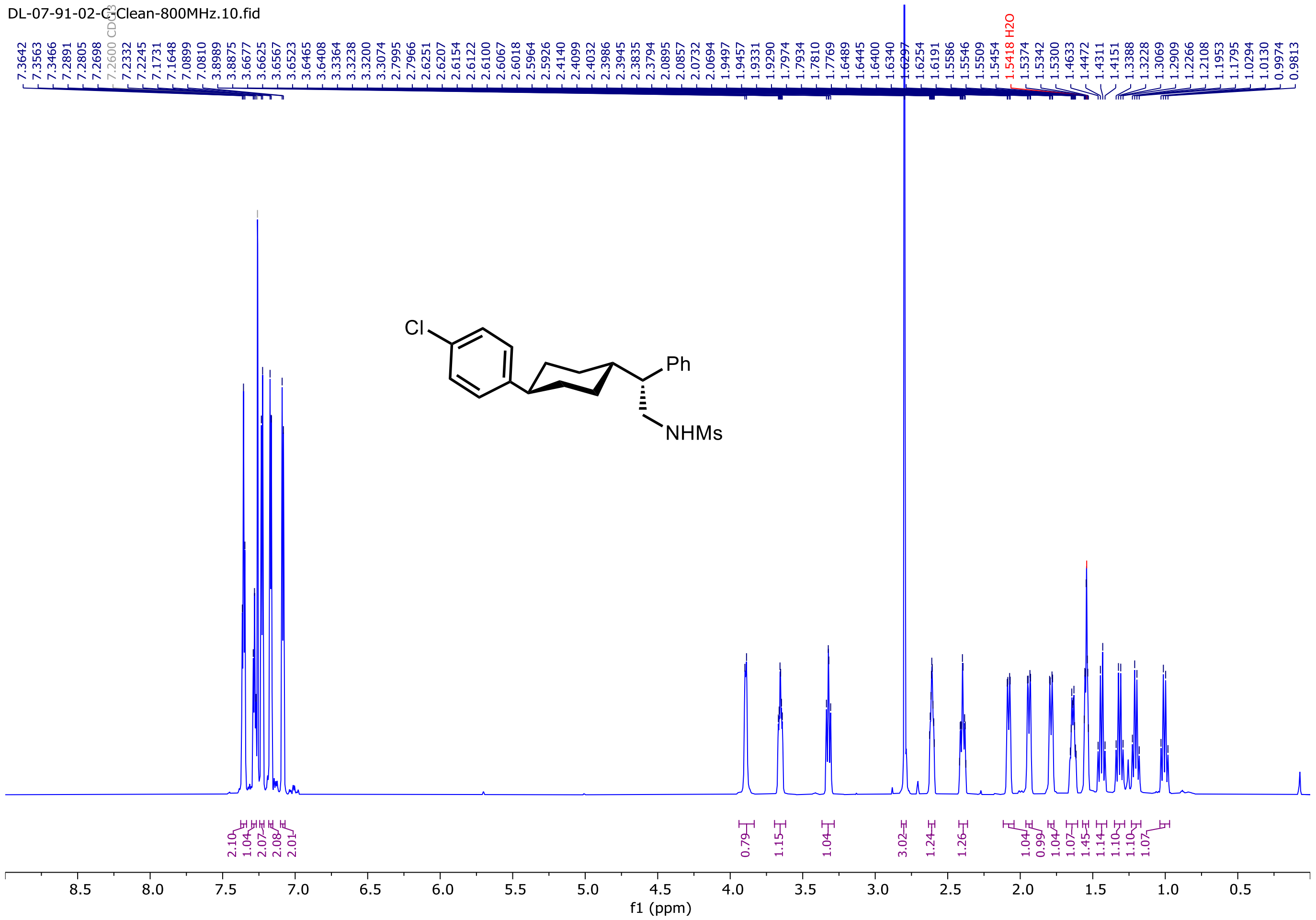

Figure S215. ^{1}H-NMR of 15

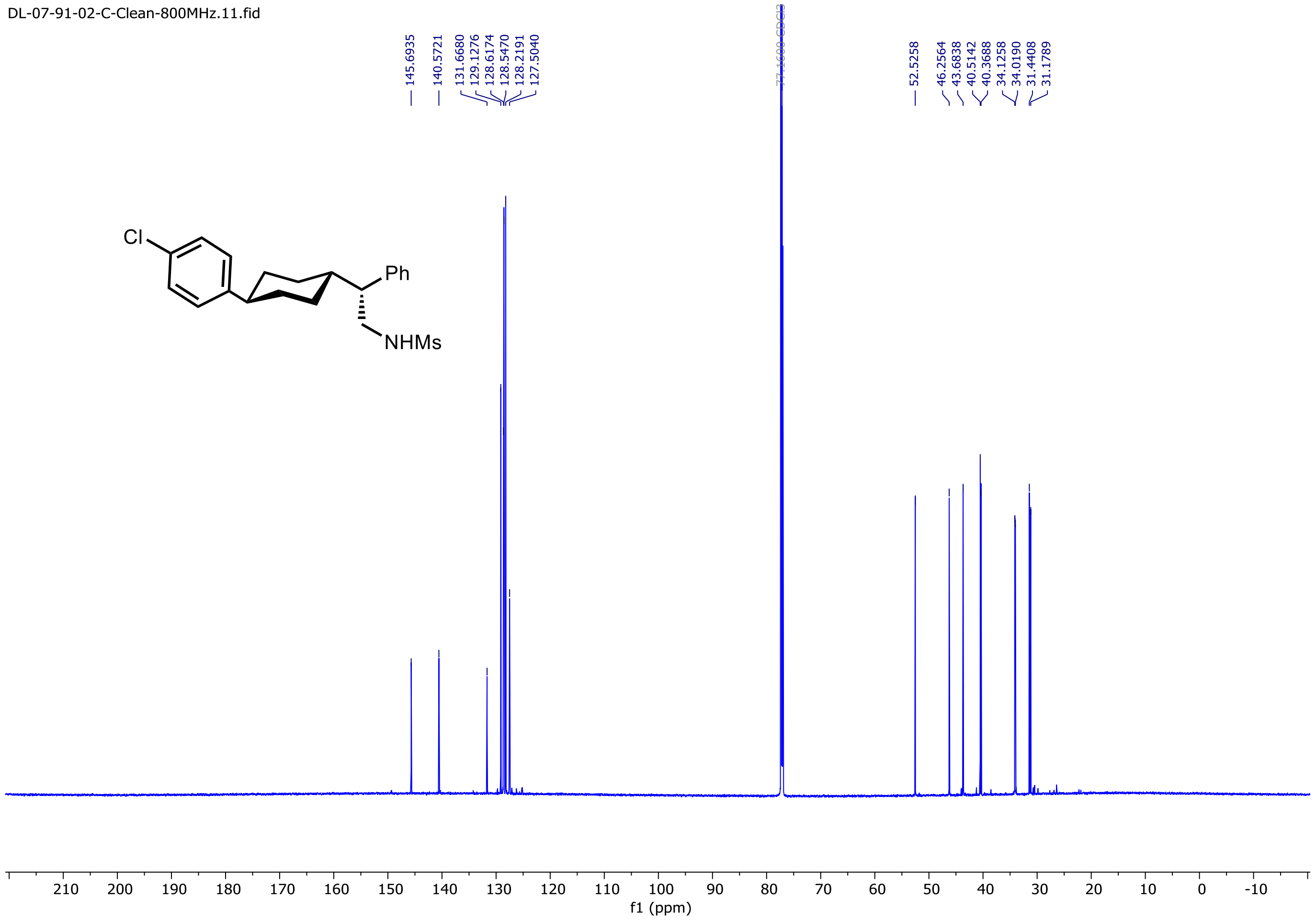

Figure S216. ^{13}C-NMR of 15

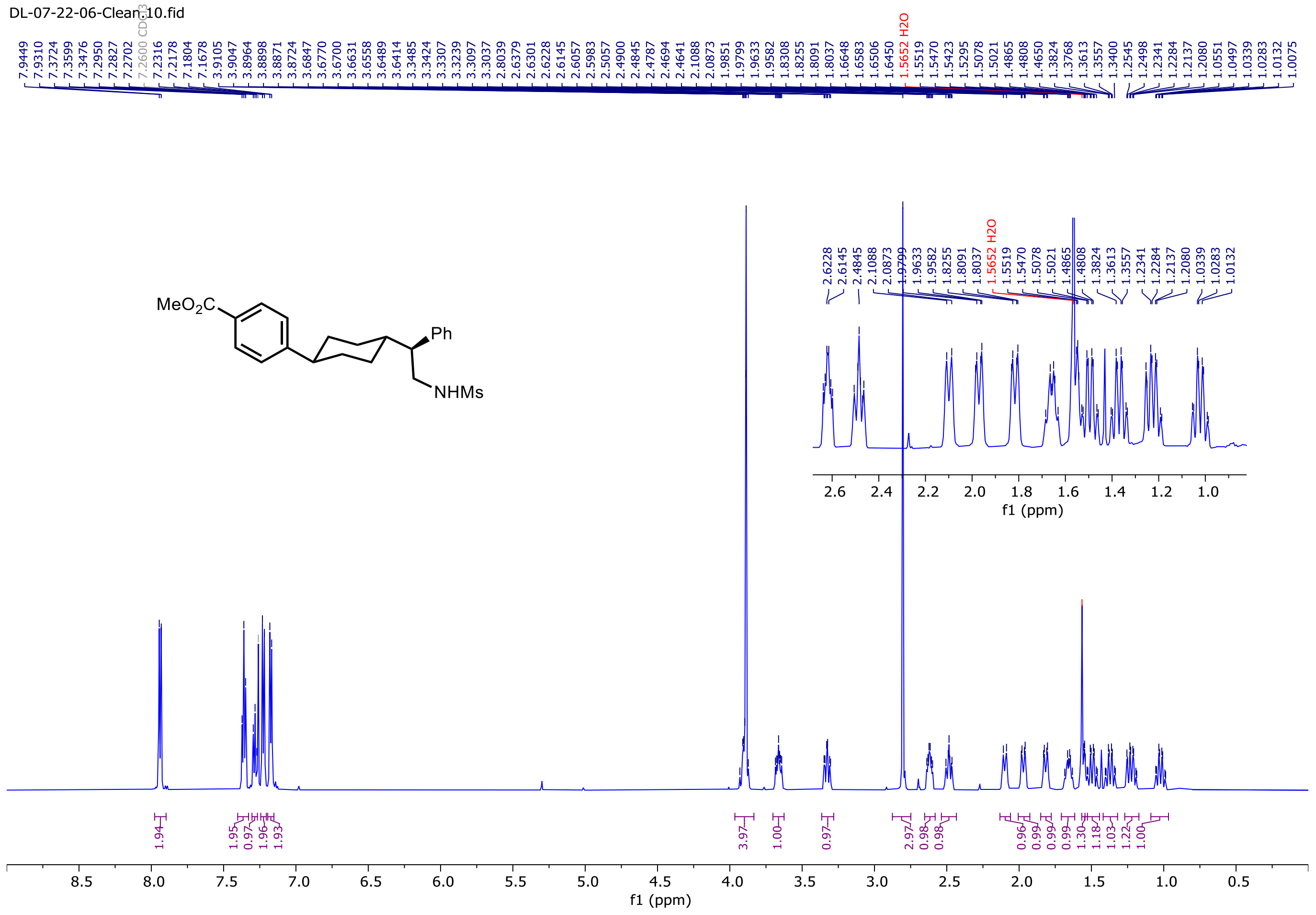

Figure S217. ^{1}H-NMR of 16

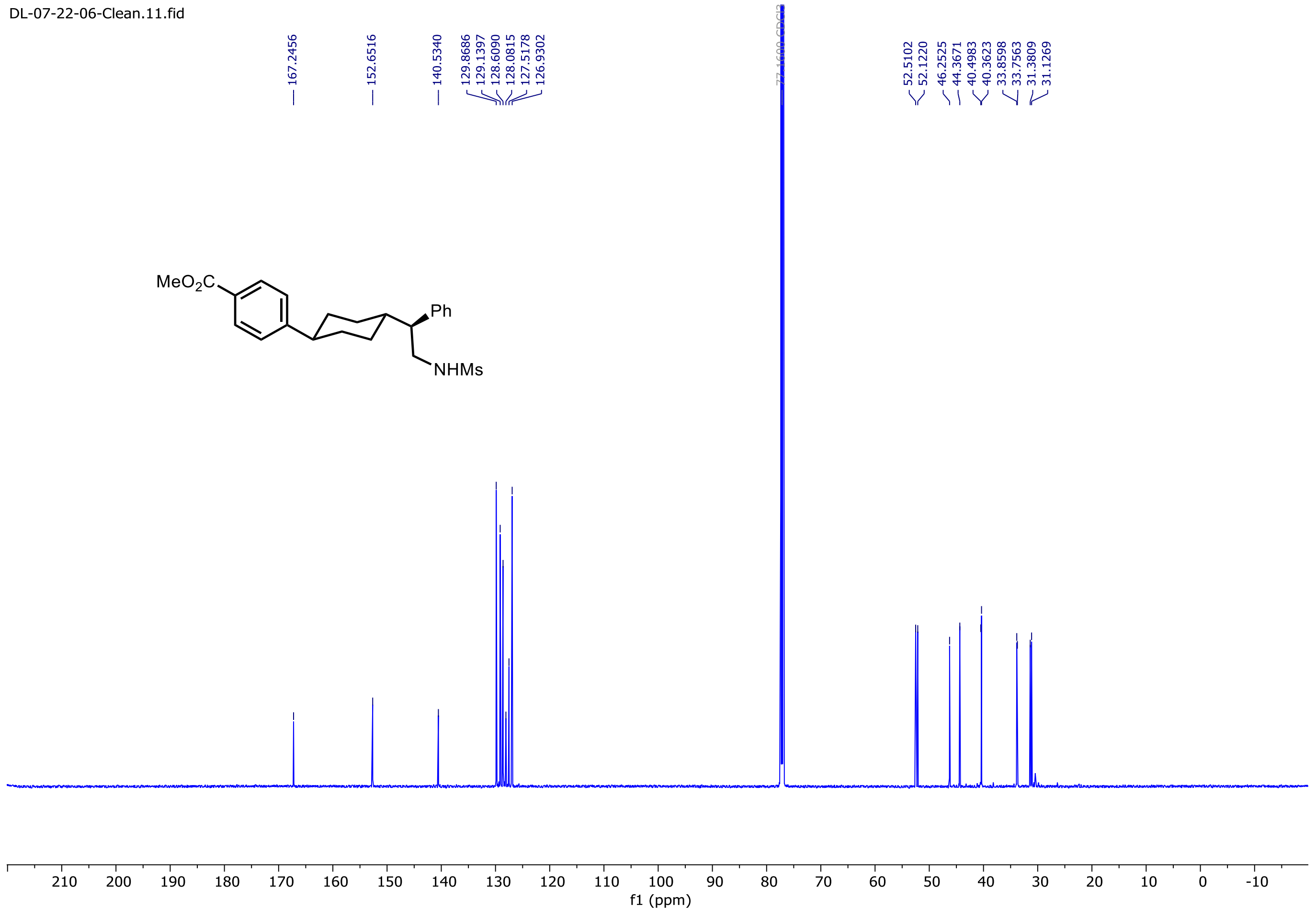

Figure S218. ^{13}C-NMR of 16

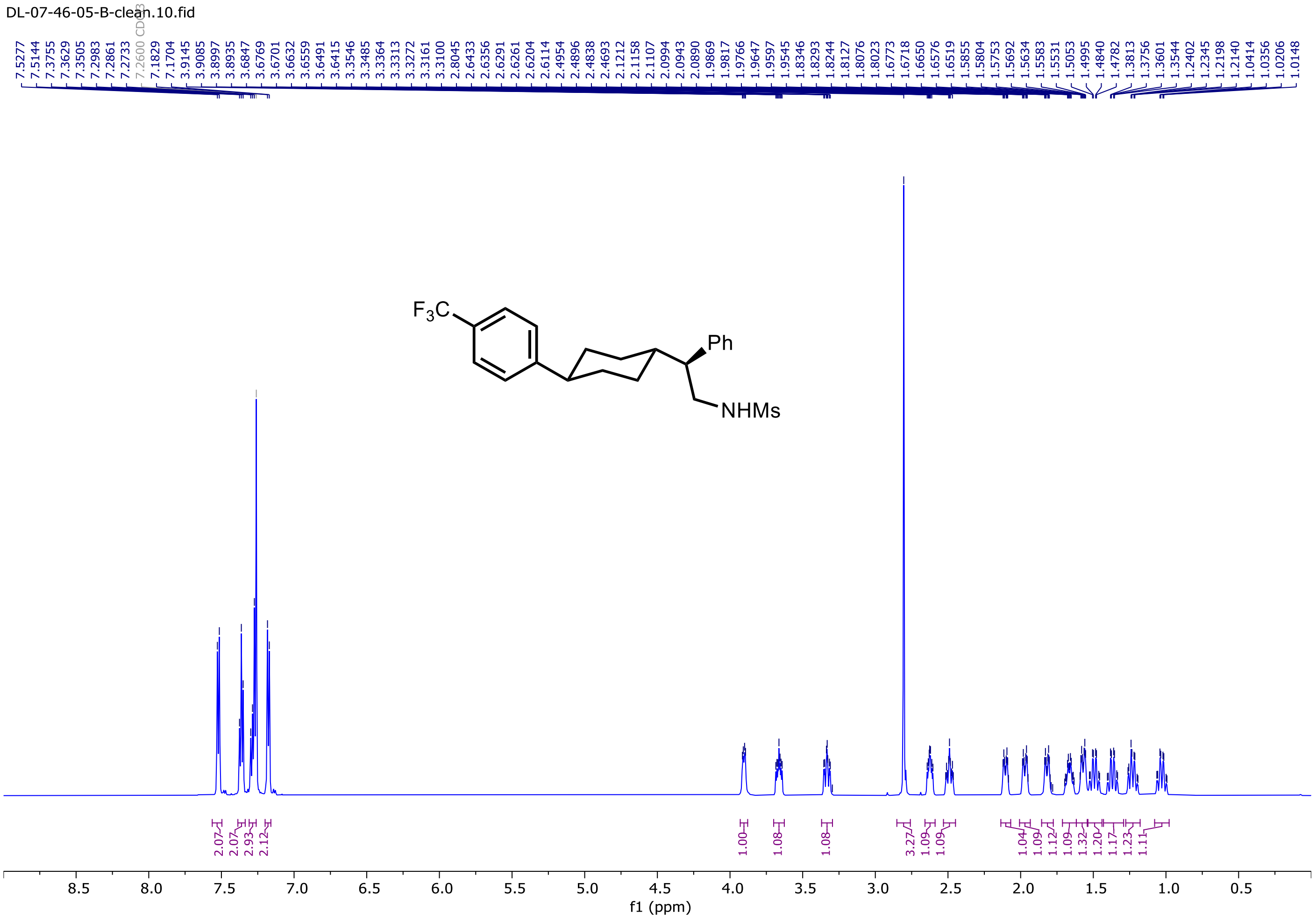

Figure S219. ^{1}H-NMR of 17

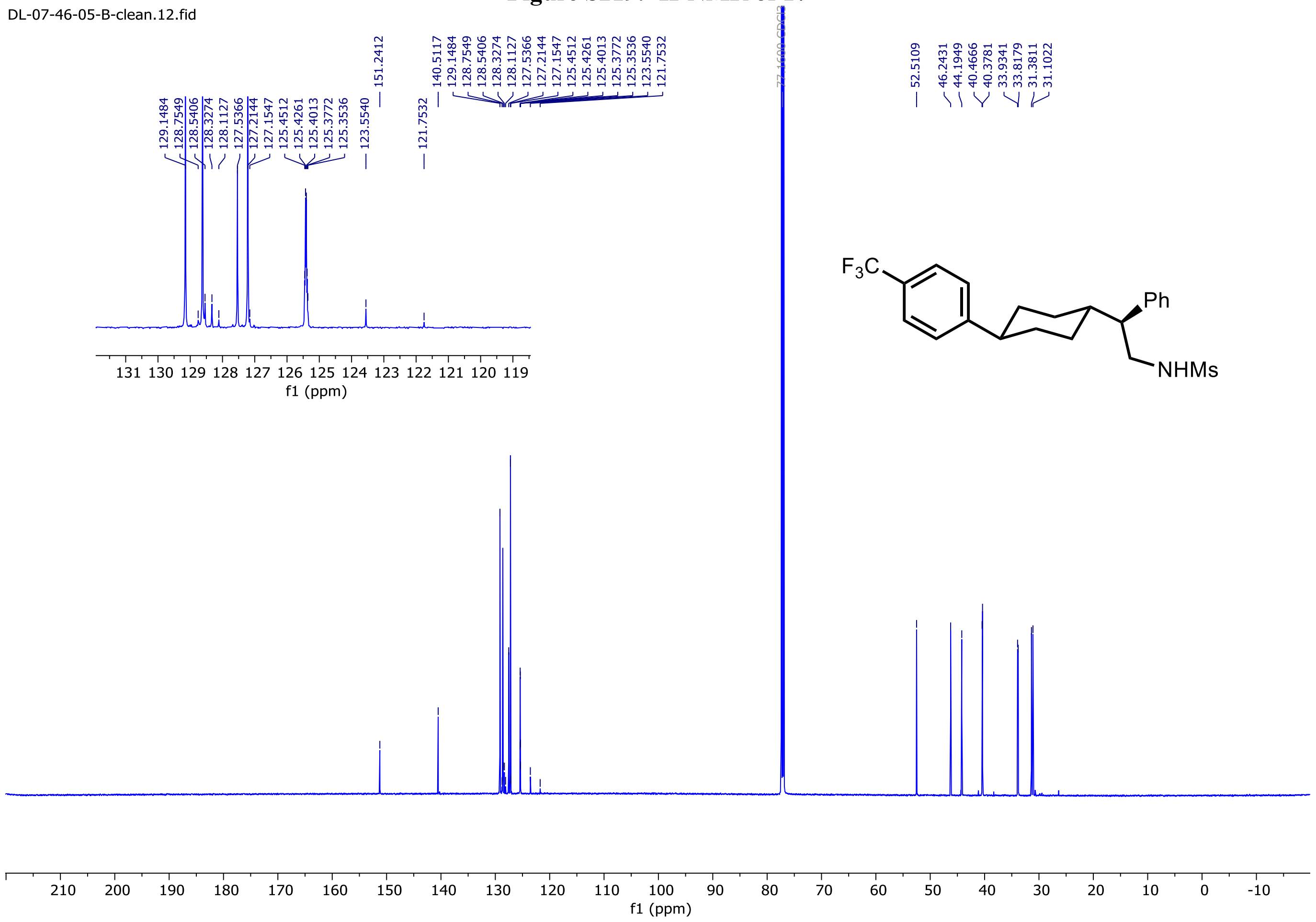

Figure S220. ^{13}C-NMR of 17

DL-07-46-05-B-clean.11.fid

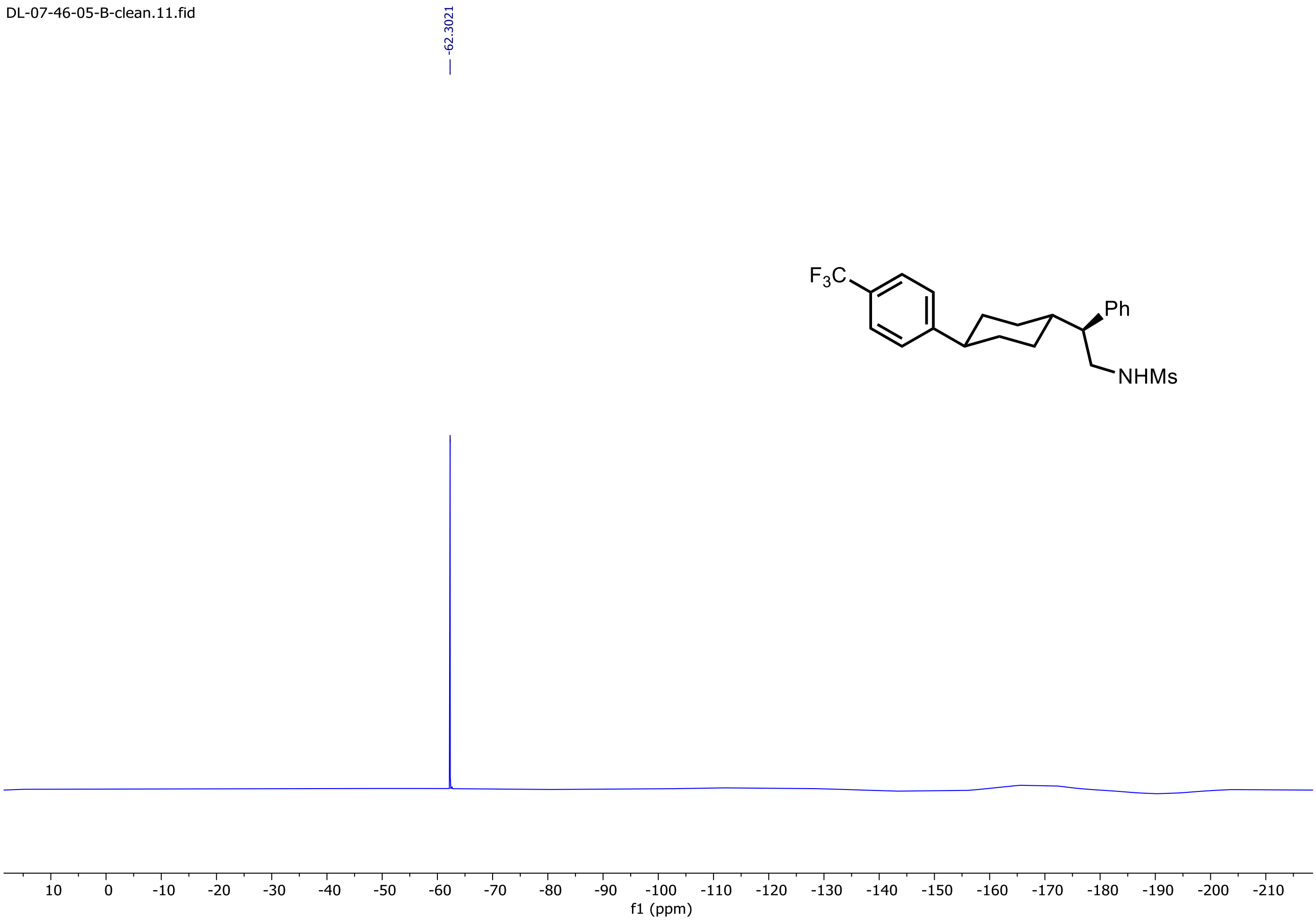

Figure S221. ^{19}F-NMR of 17

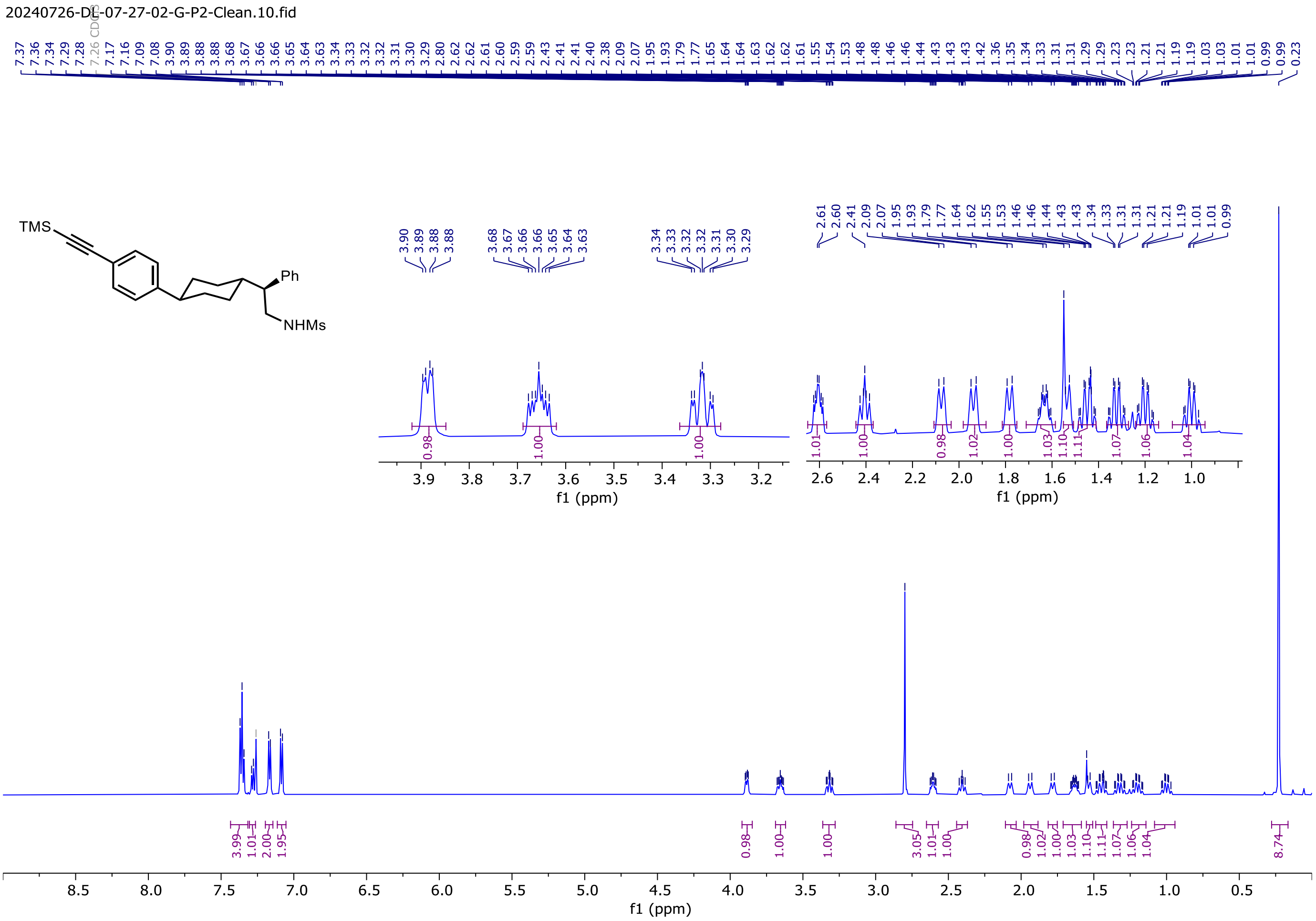

Figure S222. ^{1}H-NMR of 18

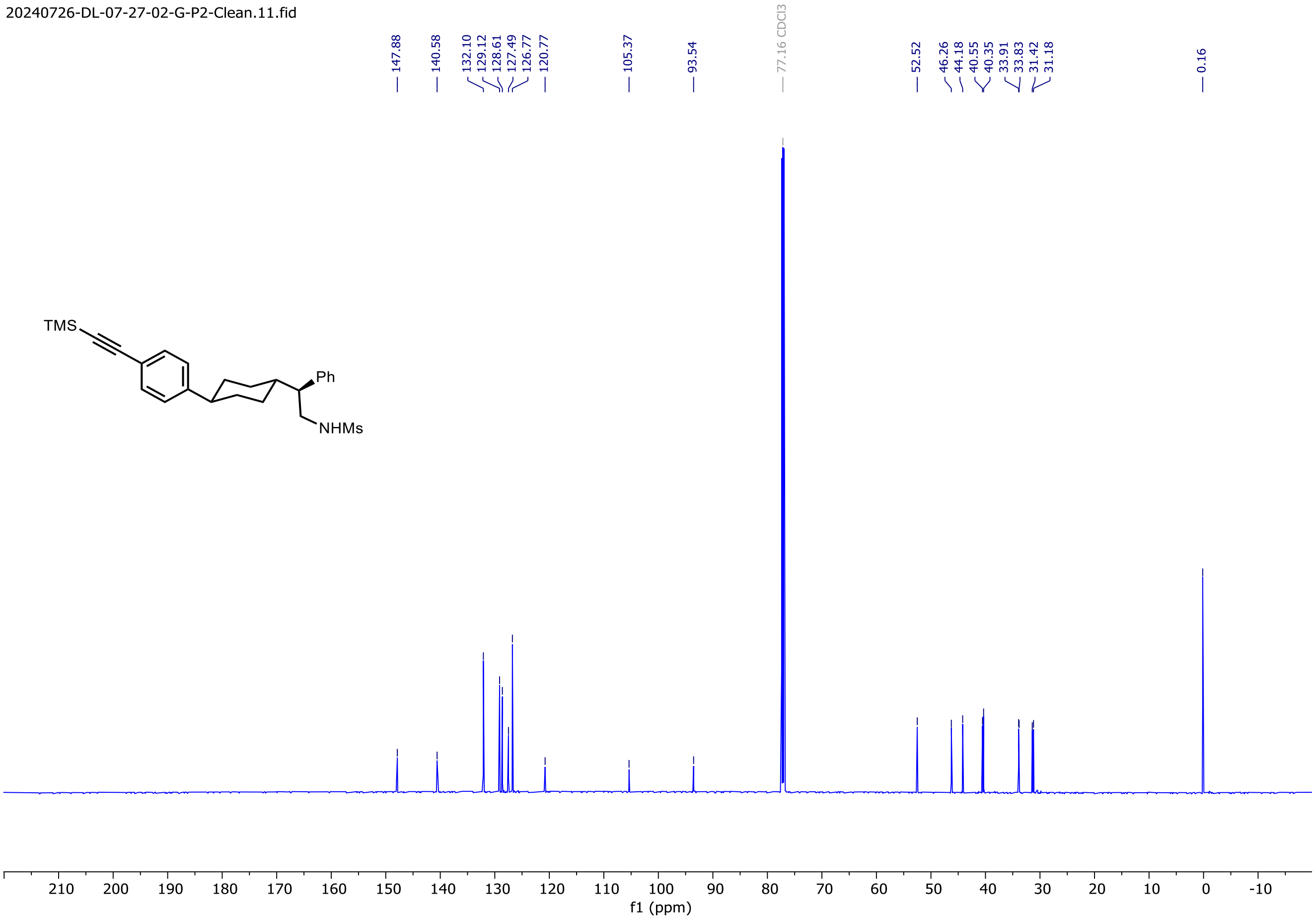

Figure S223. ^{13}C-NMR of 18

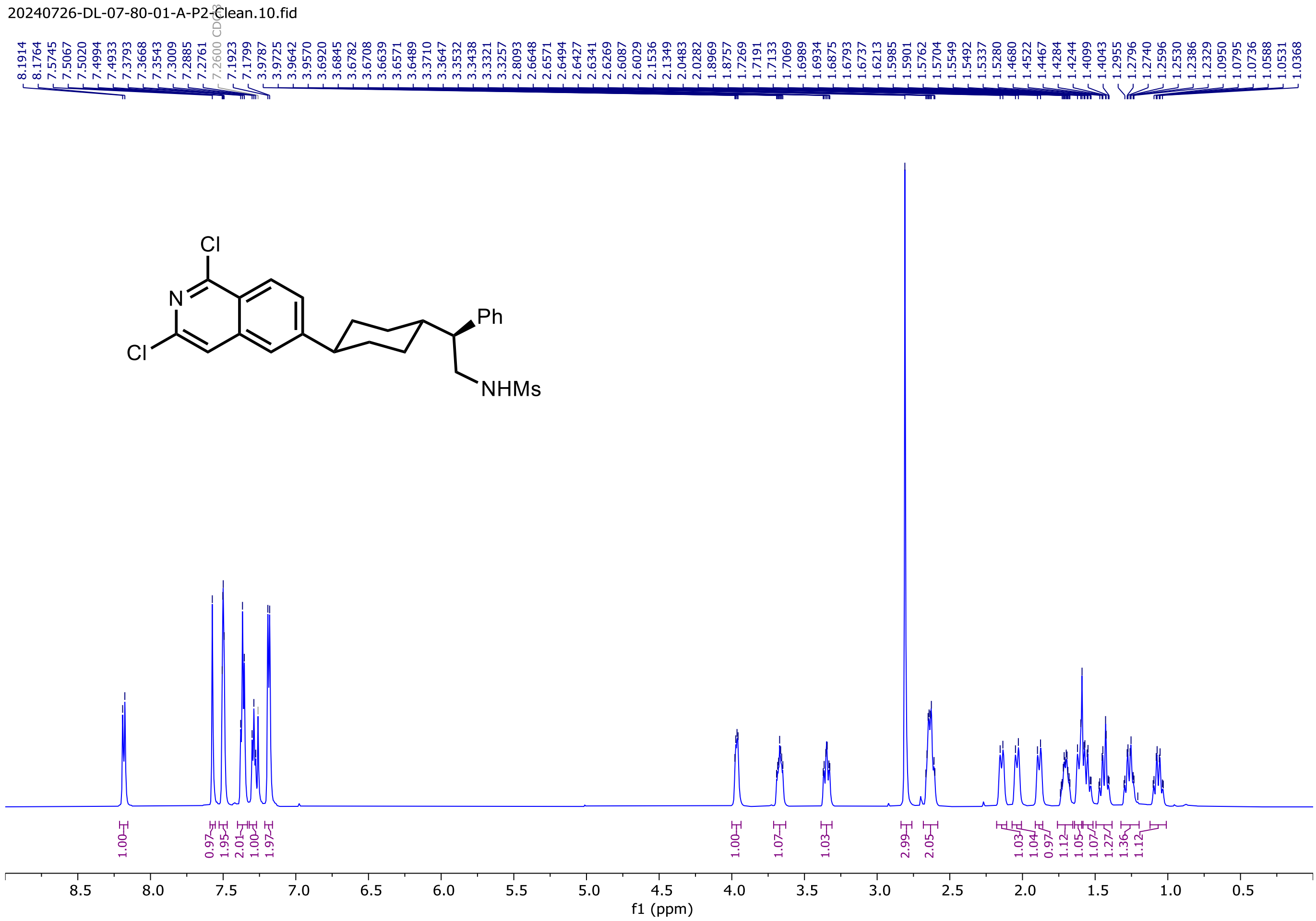

Figure S224. ^{1}**H-NMR of 19**

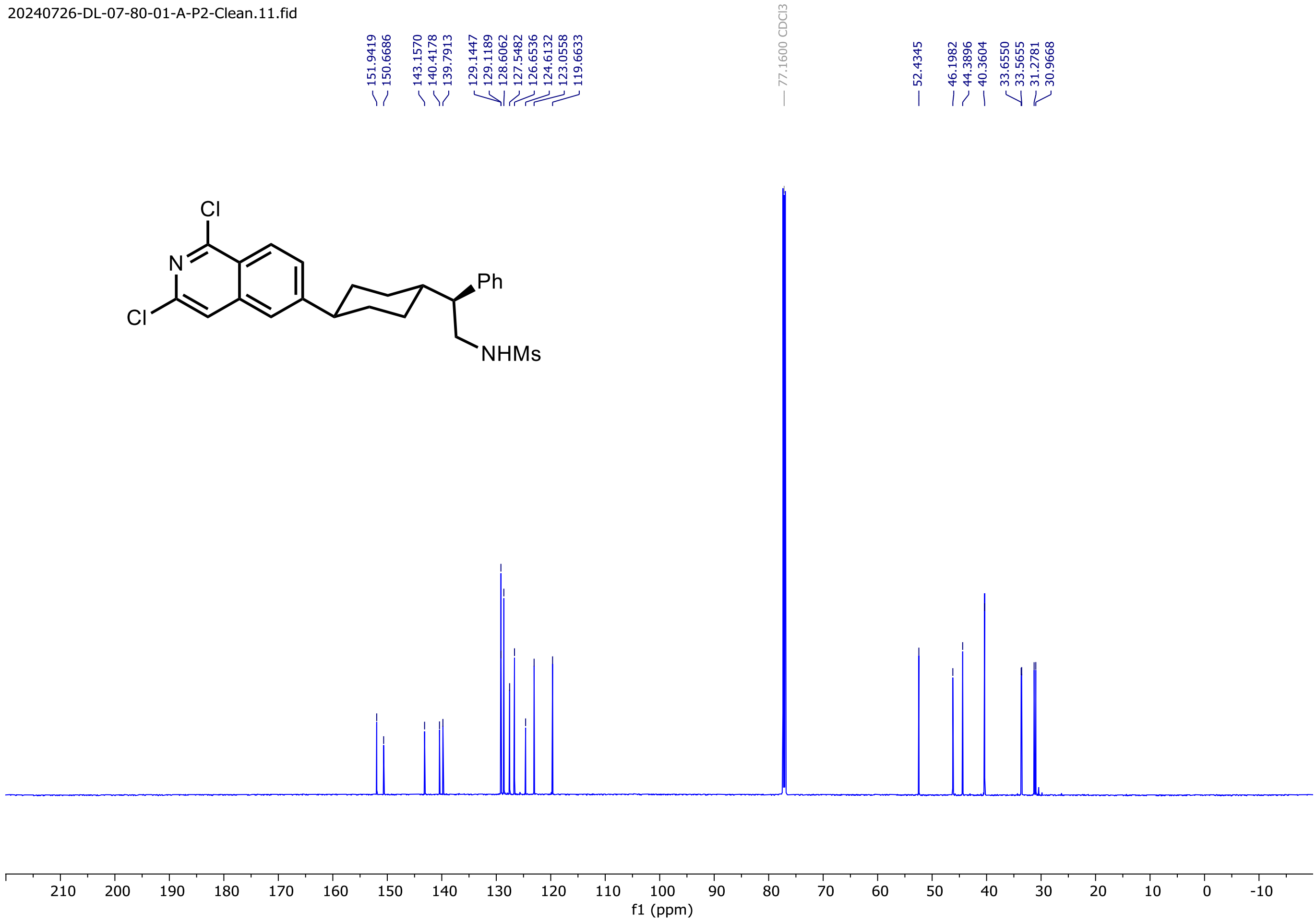

Figure S225. ^{13}**C-NMR of 19**

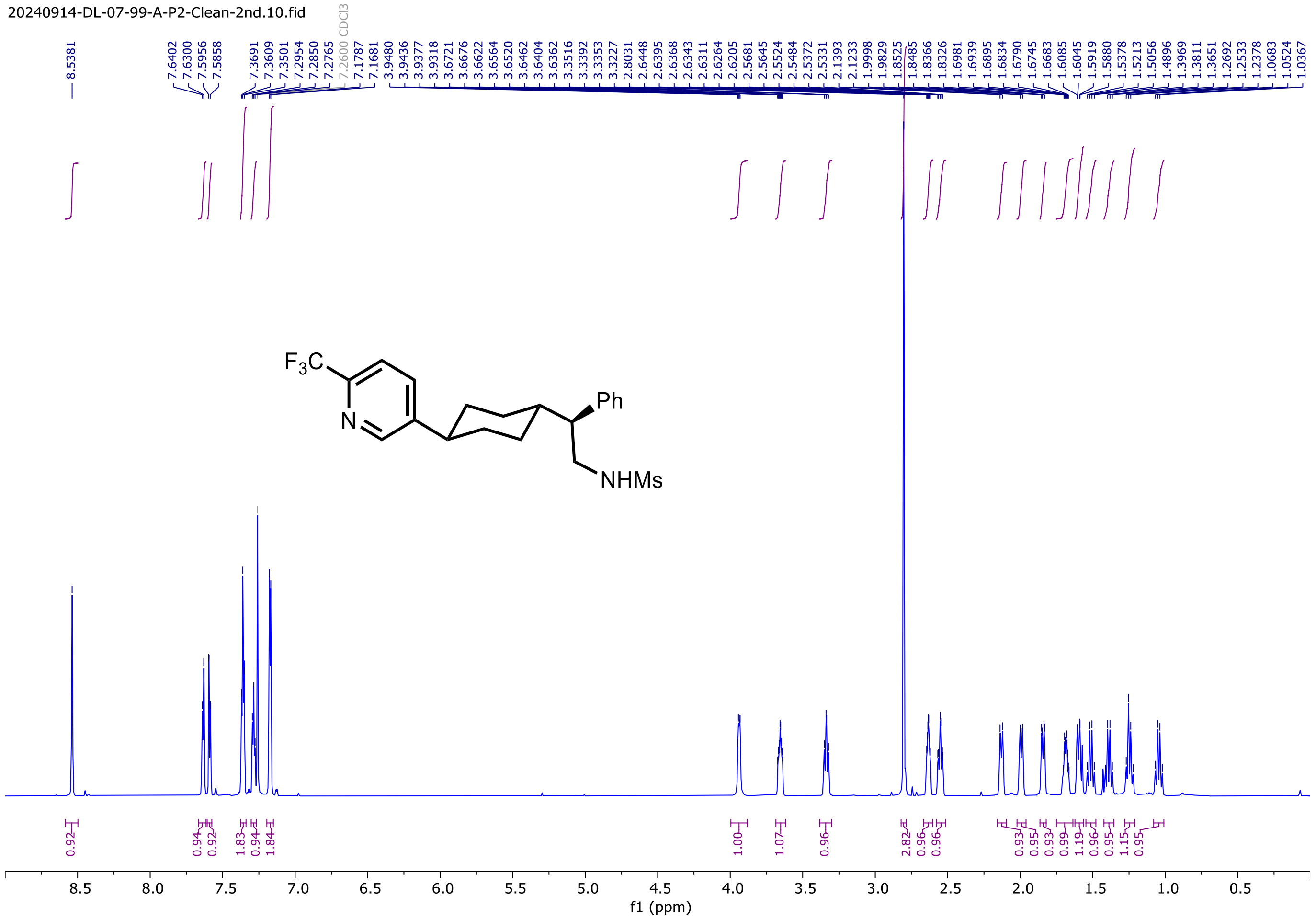

Figure S226. ^{1}H-NMR of 20

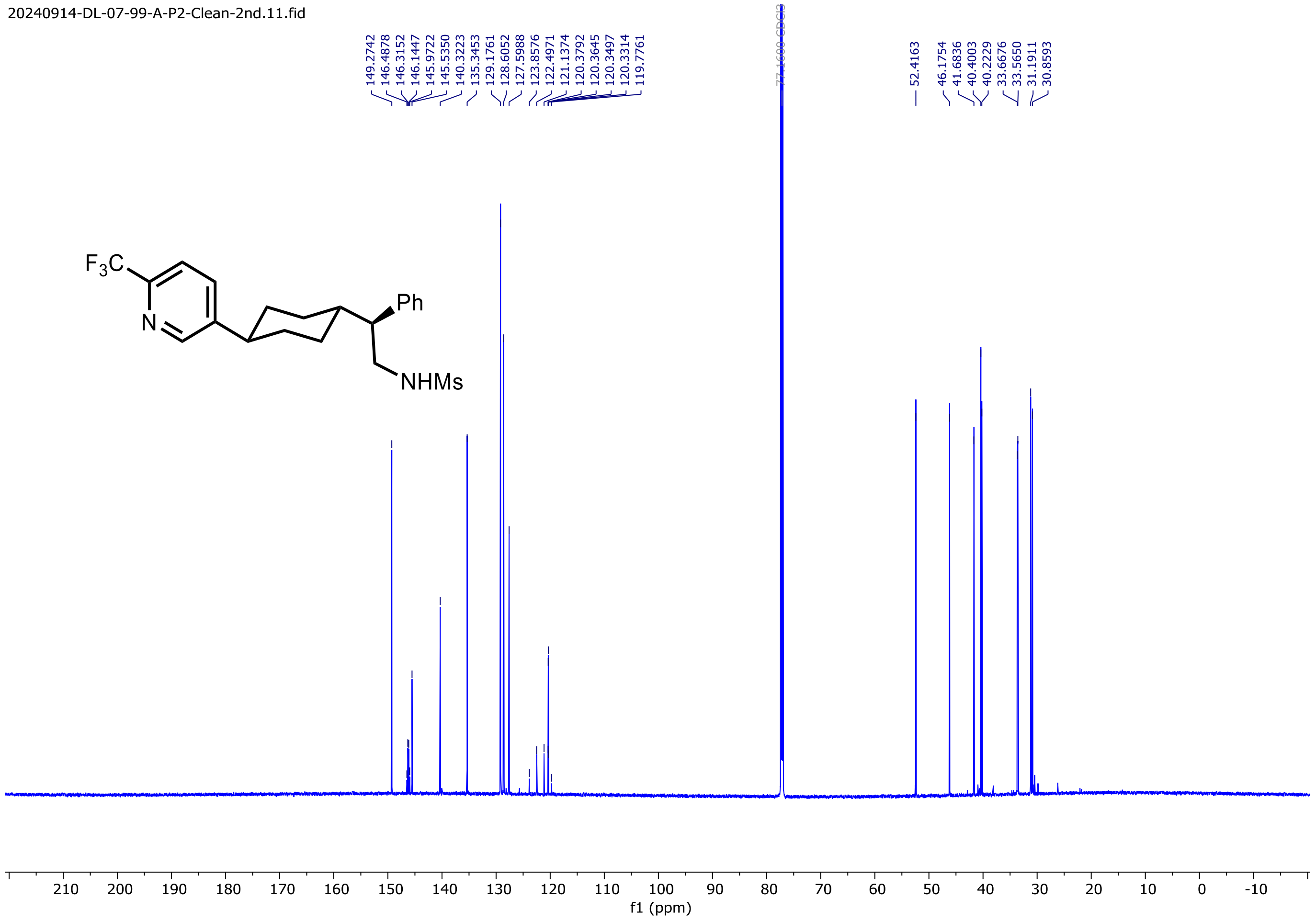

Figure S227. ^{13}C-NMR of 20

20240913-DL-07-99-A-P2-Clean.44.fid

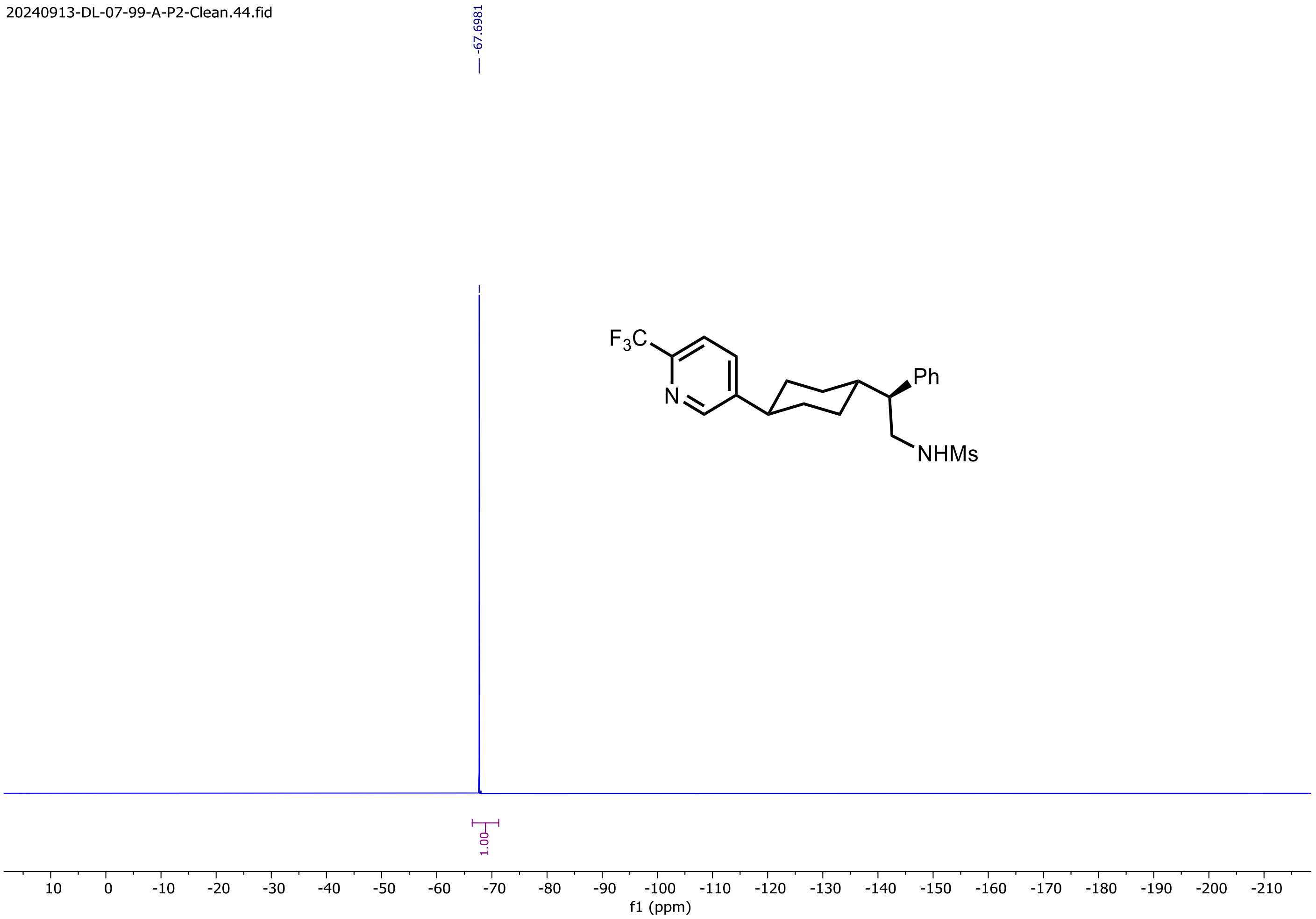

Figure S228. ^{19}F-NMR of 20

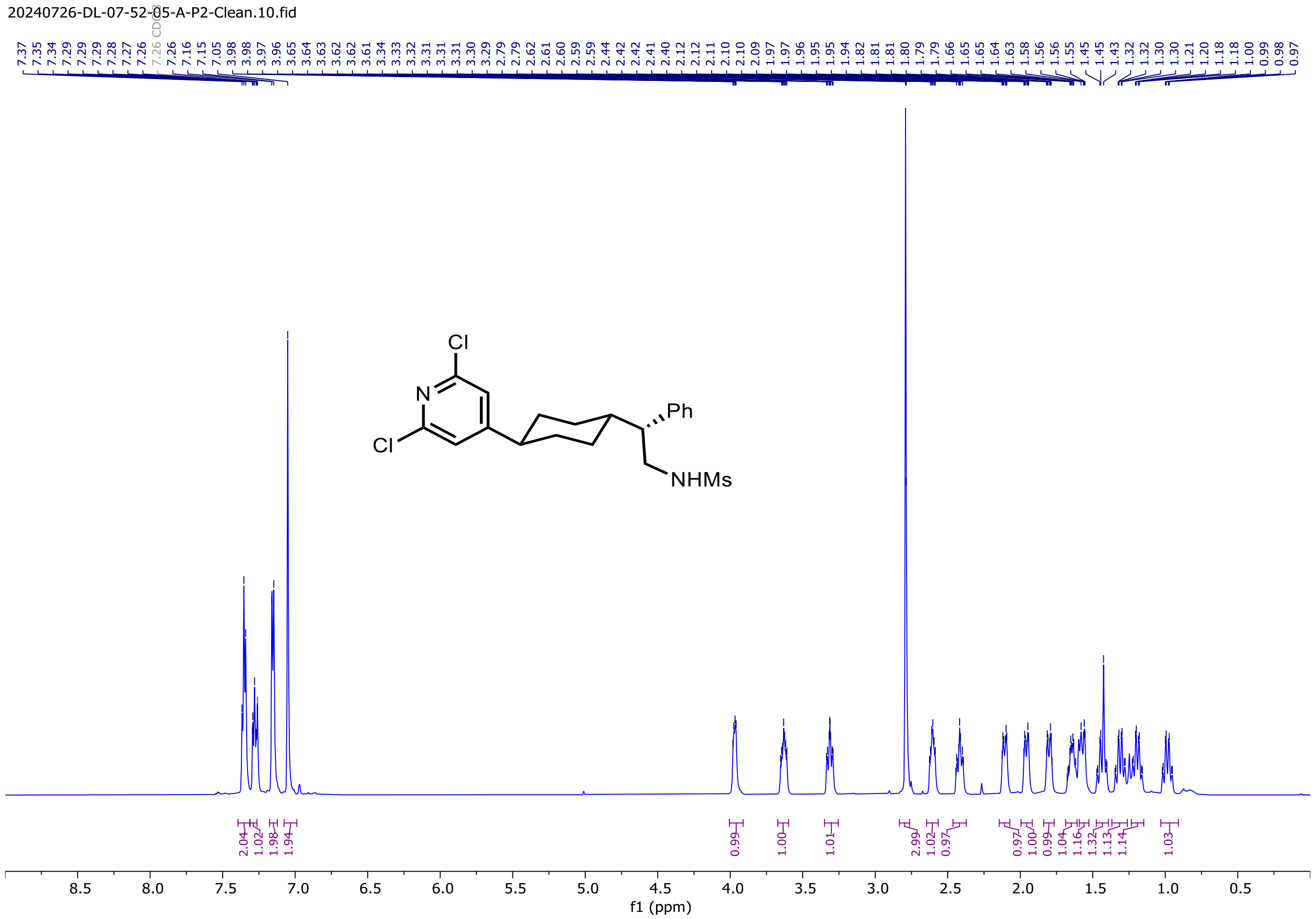

Figure S229. ^{1}H-NMR of 21

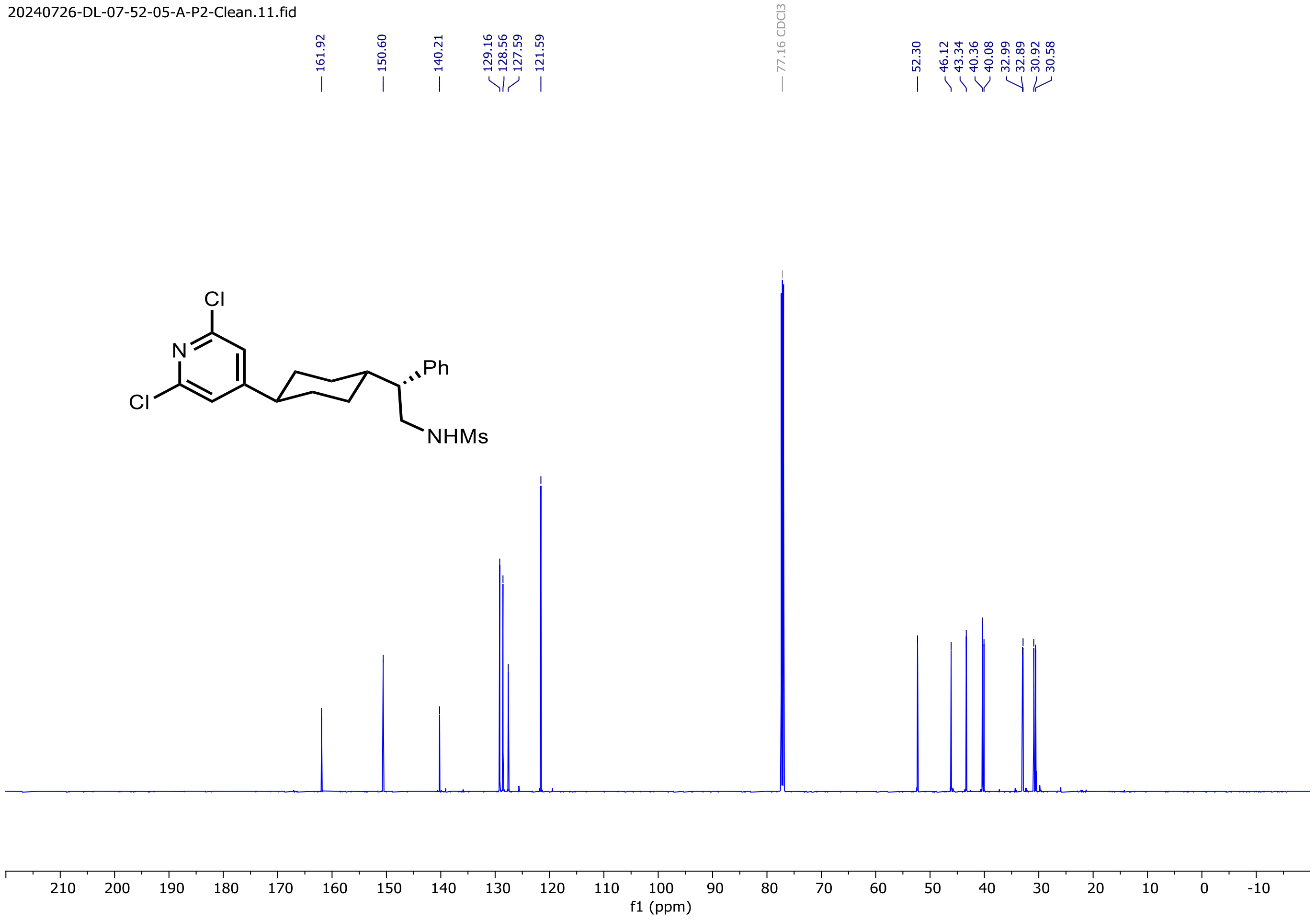

Figure S230. ^{13}C-NMR of 21

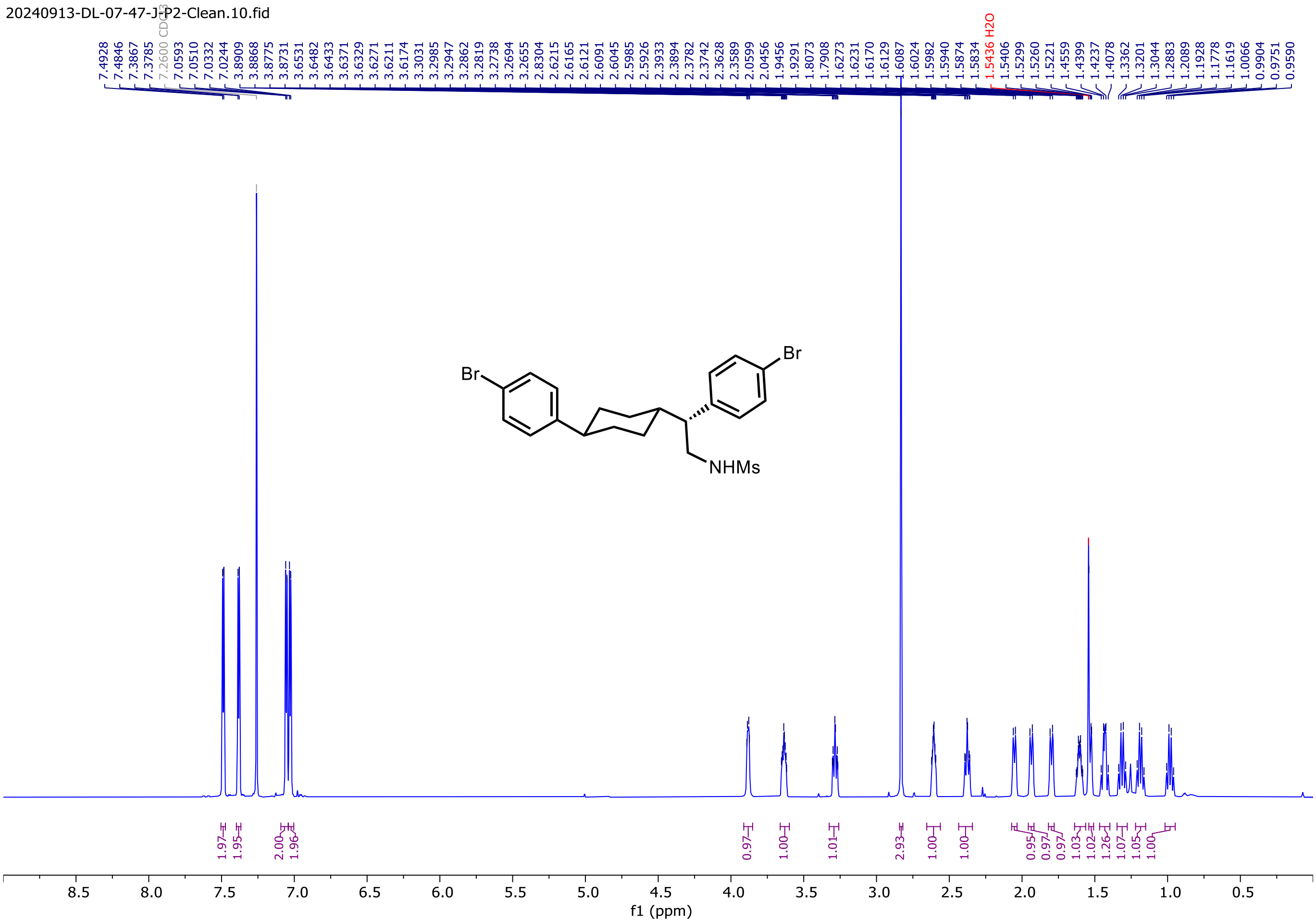

Figure S231. ^{1}H-NMR of 22

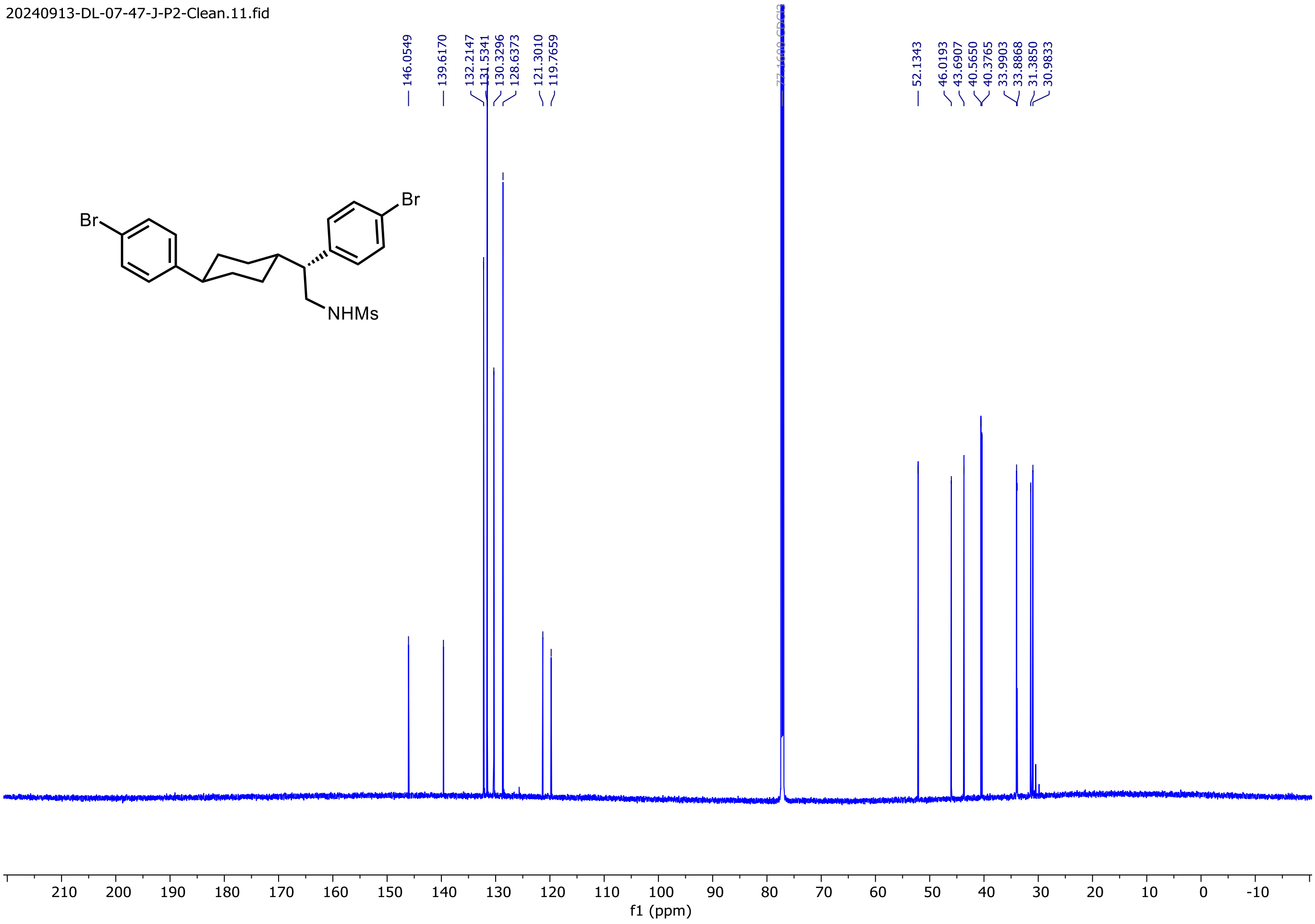

Figure S232. ^{13}C-NMR of 22

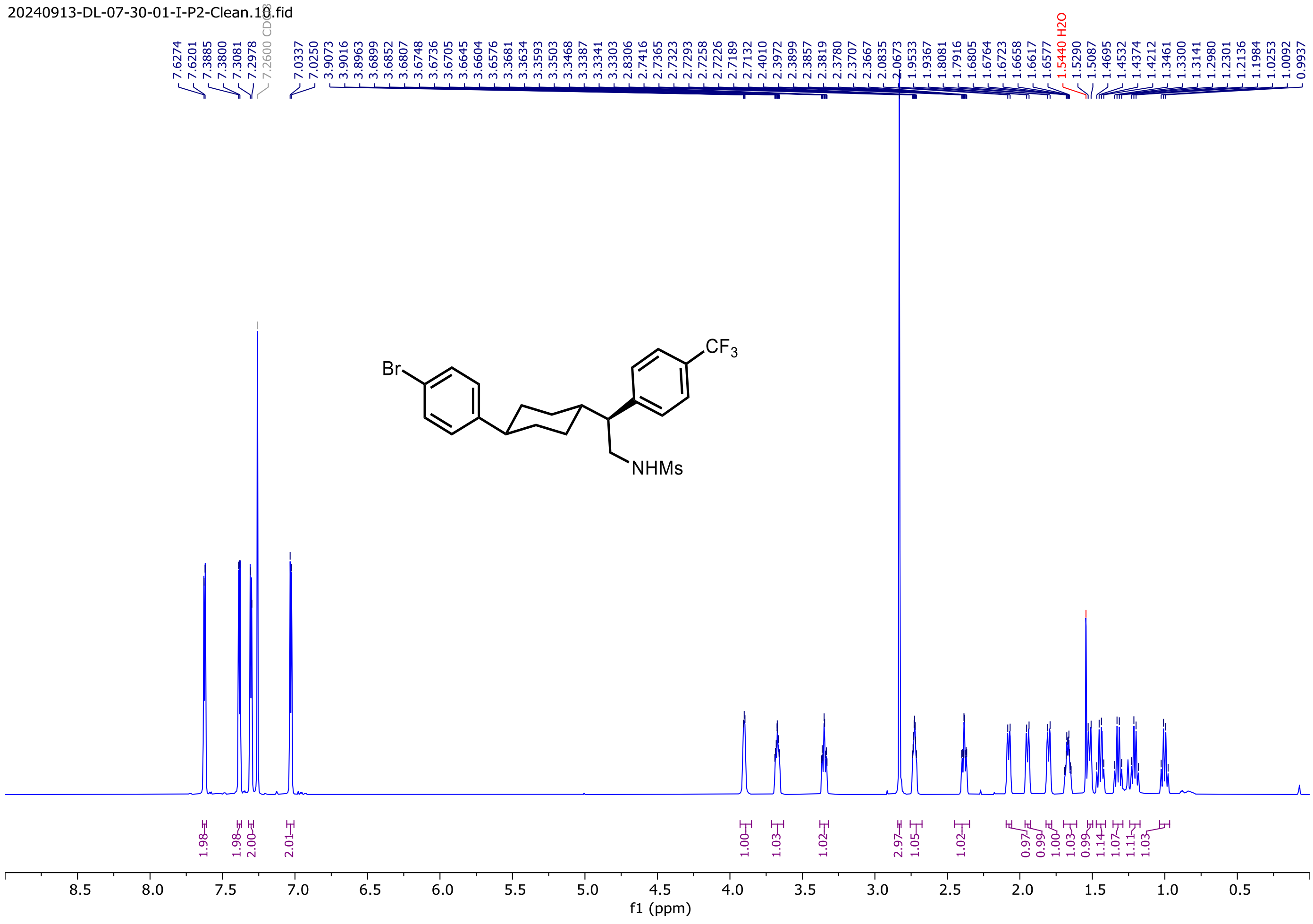

Figure S233. ^{1}H-NMR of 23

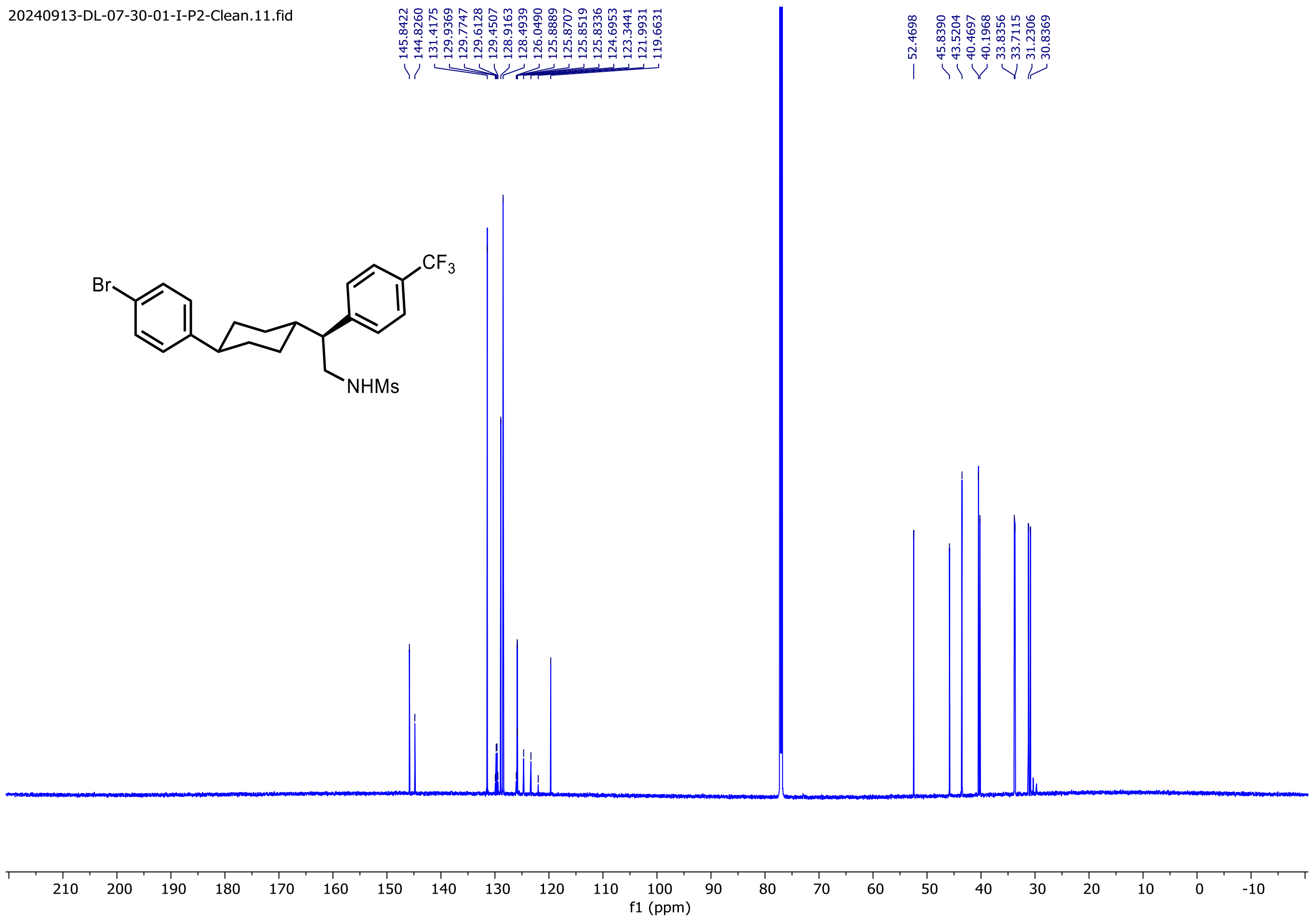

Figure S234. ^{13}C-NMR of 23

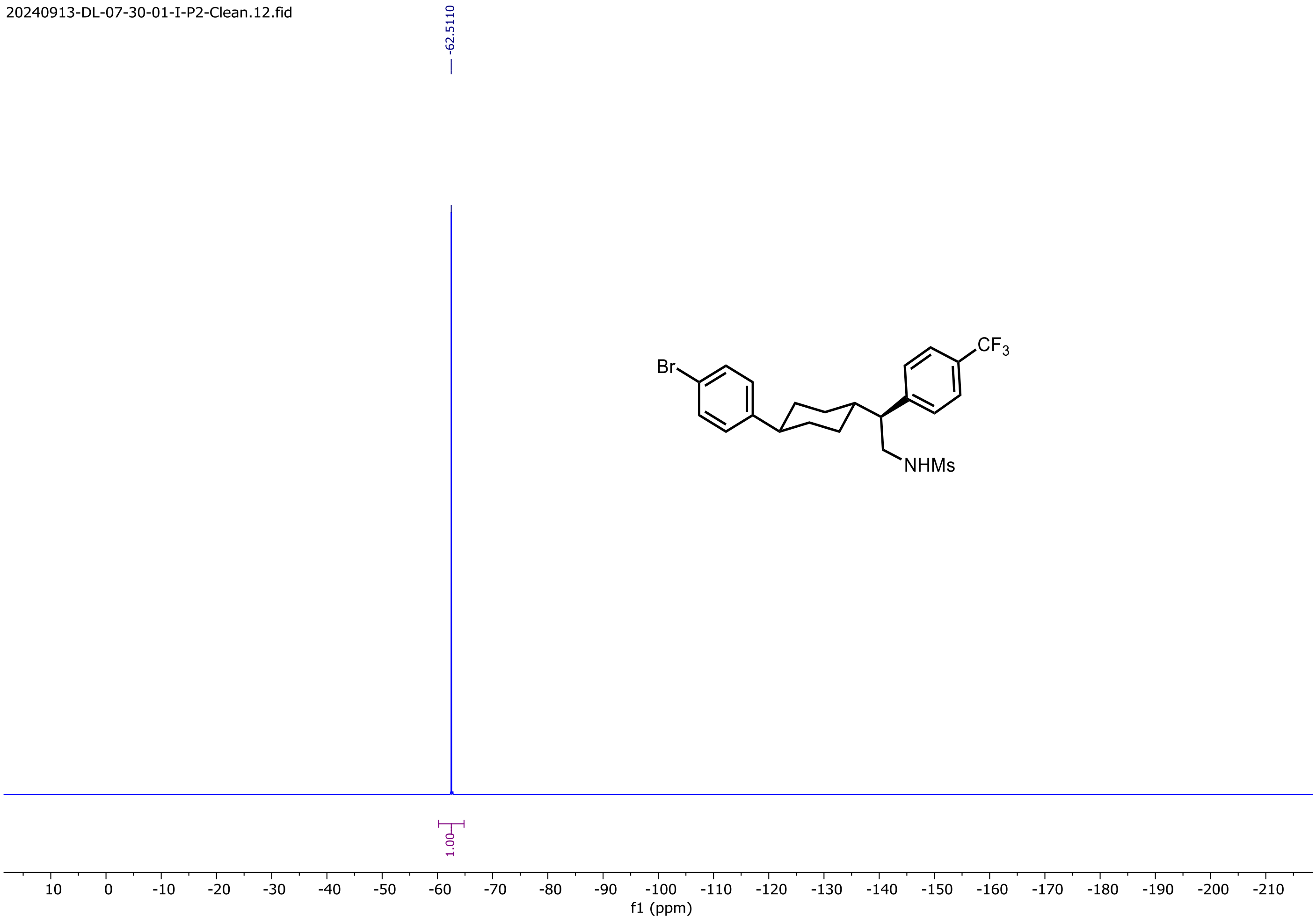

Figure S235. ^{19}F-NMR of 23

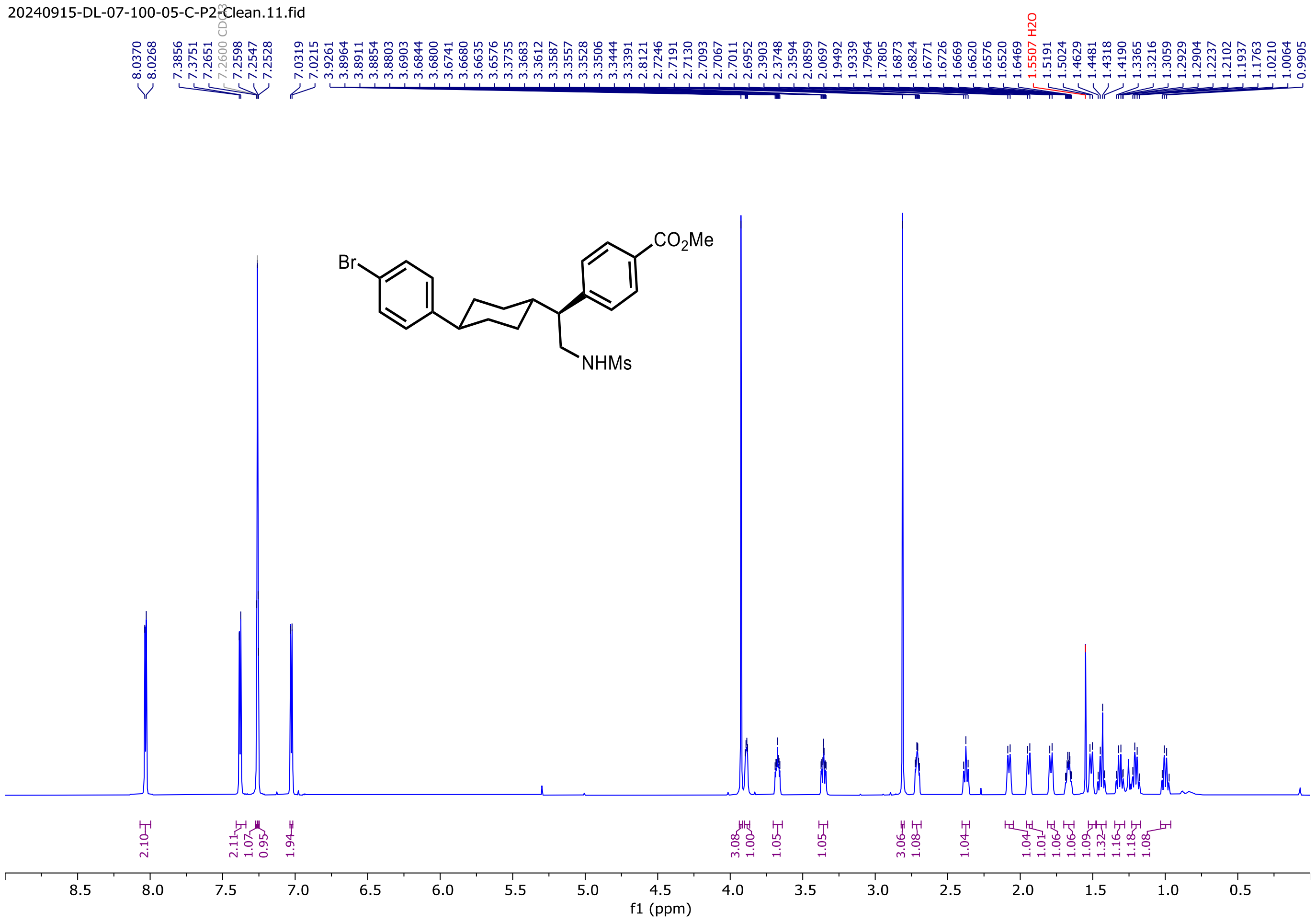

Figure S236. ^{1}H-NMR of 24

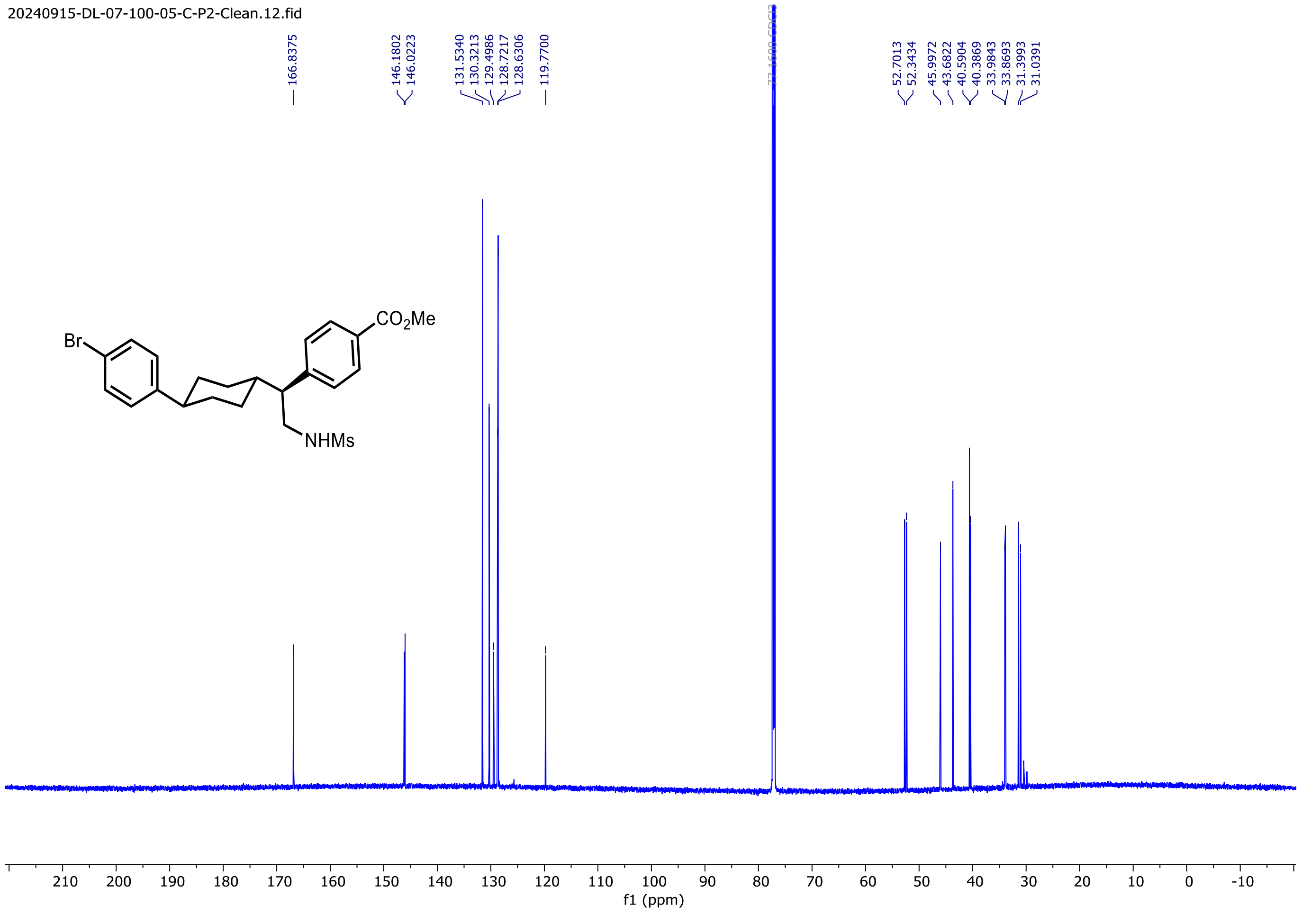

Figure S237. ^{13}C-NMR of 24

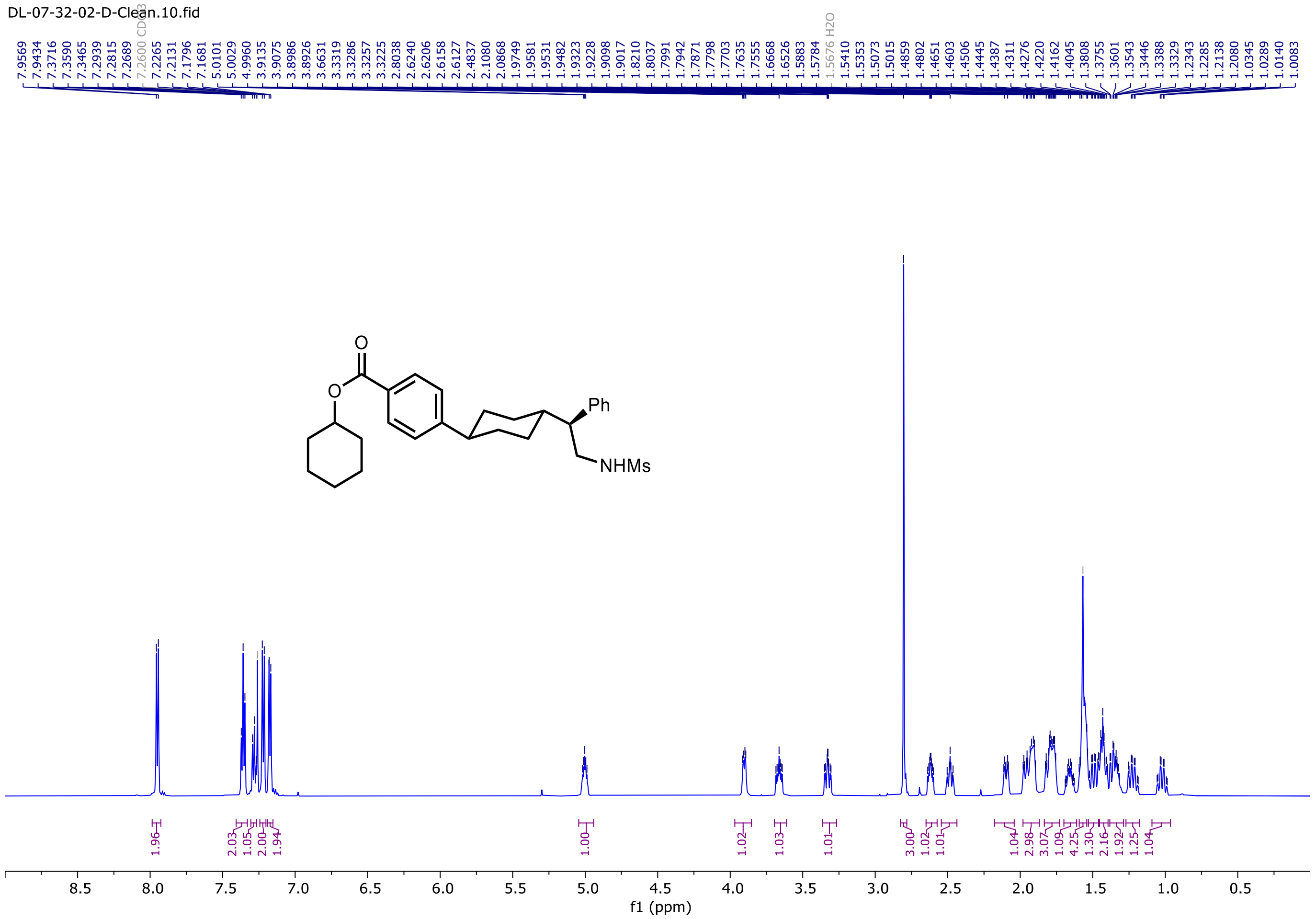

Figure S238. ^{1}H-NMR of 25

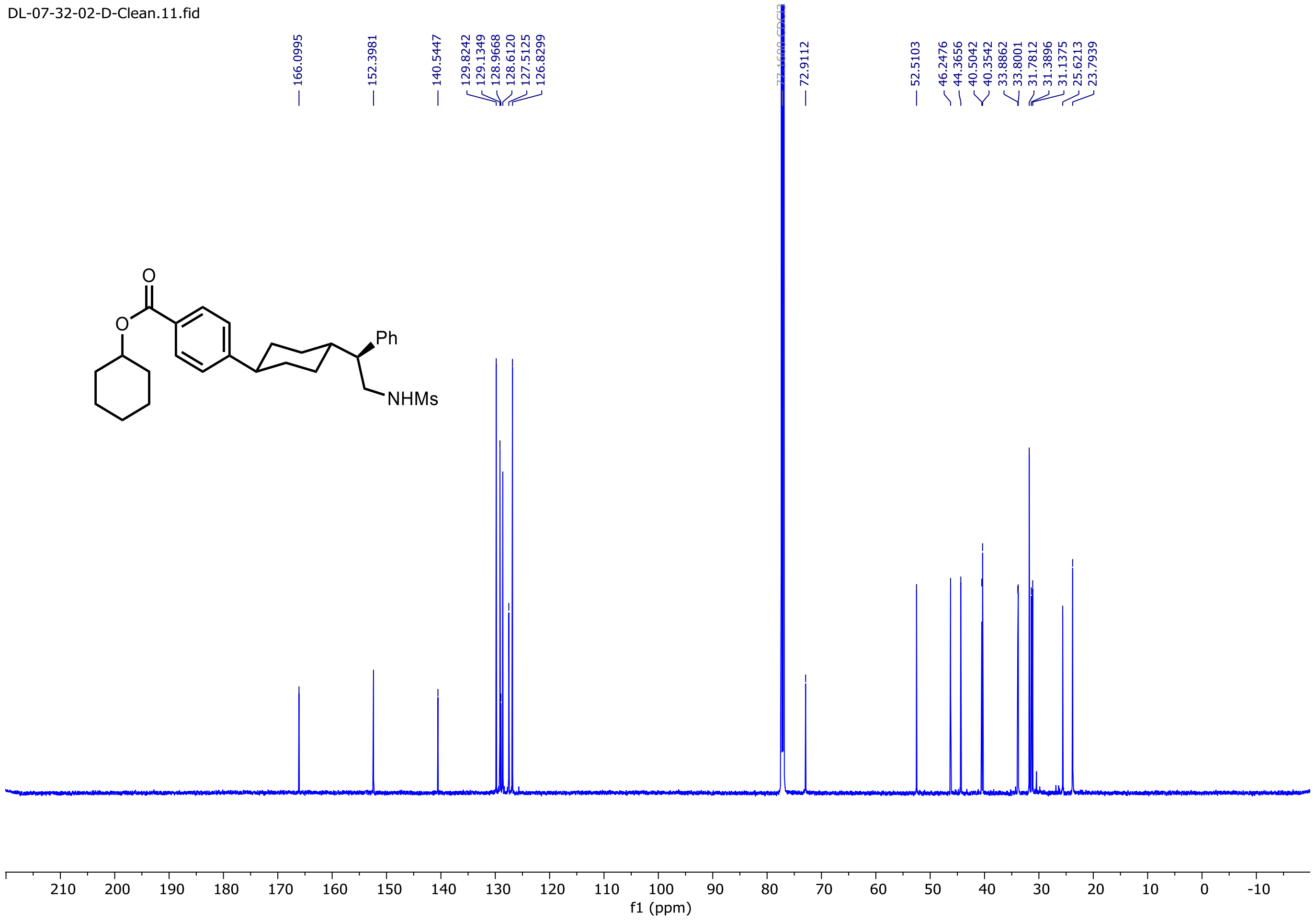

Figure S239. ^{13}C-NMR of 25

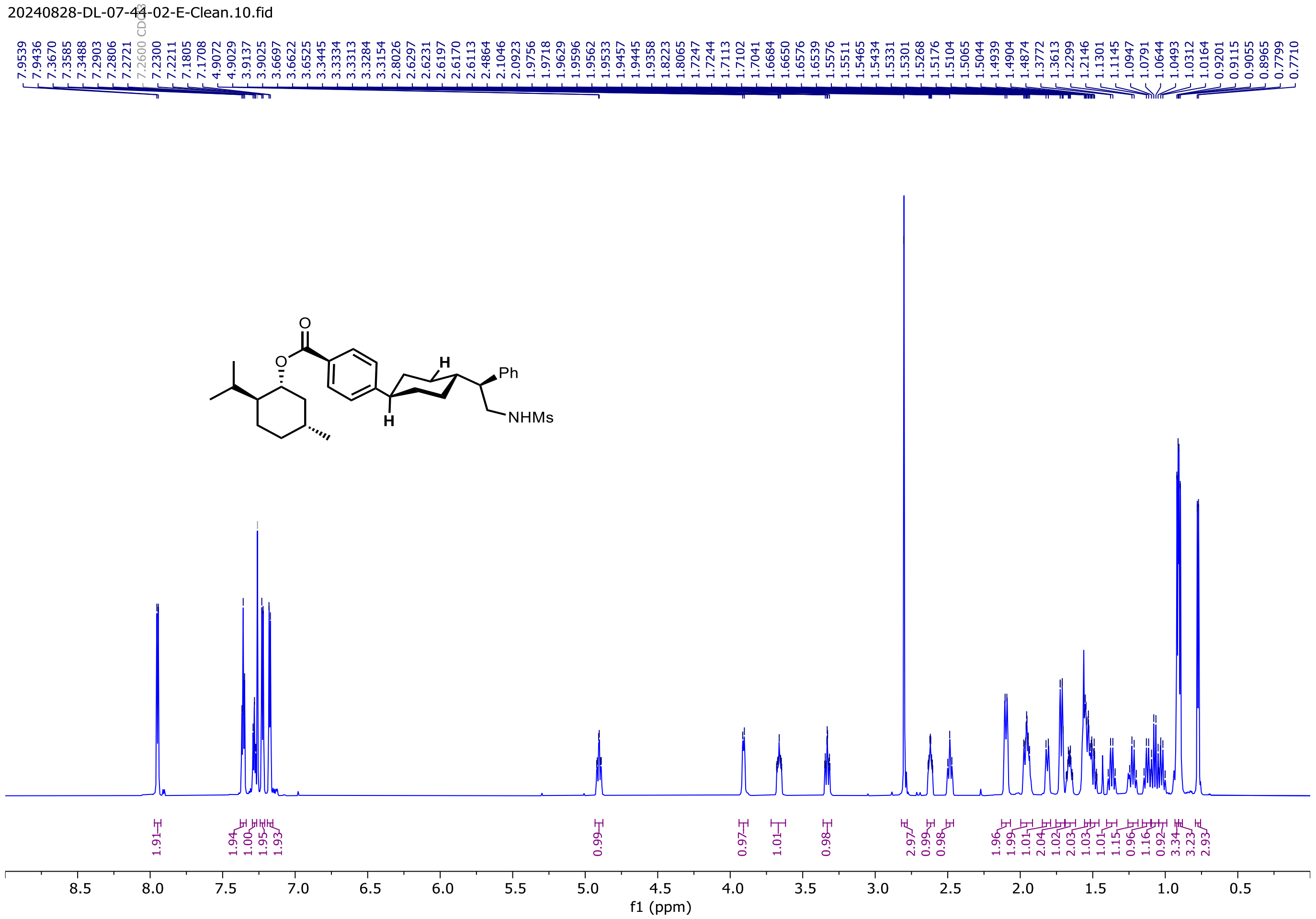

Figure S240. ^{1}H-NMR of 26

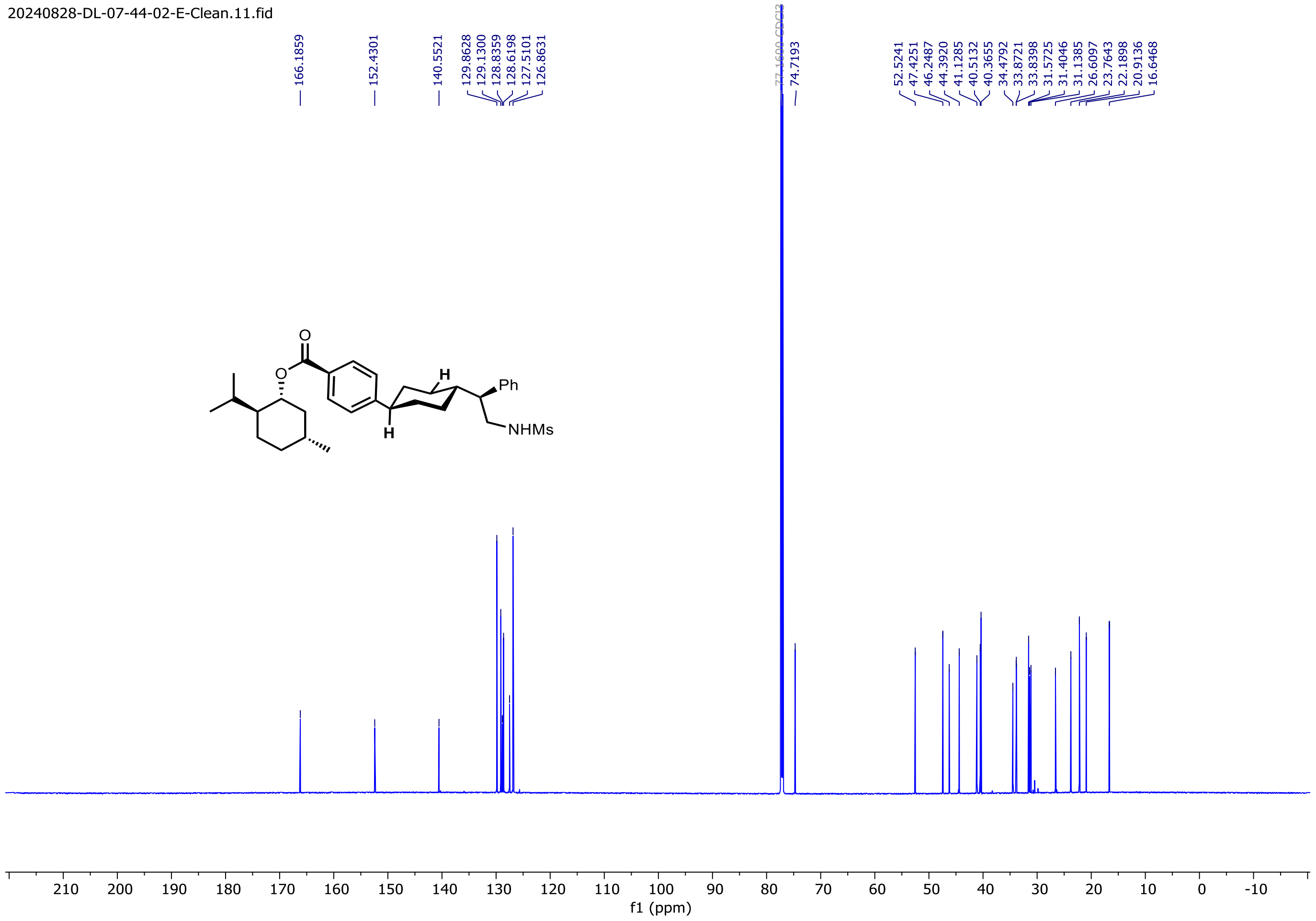

Figure S241. ^{13}C-NMR of 26

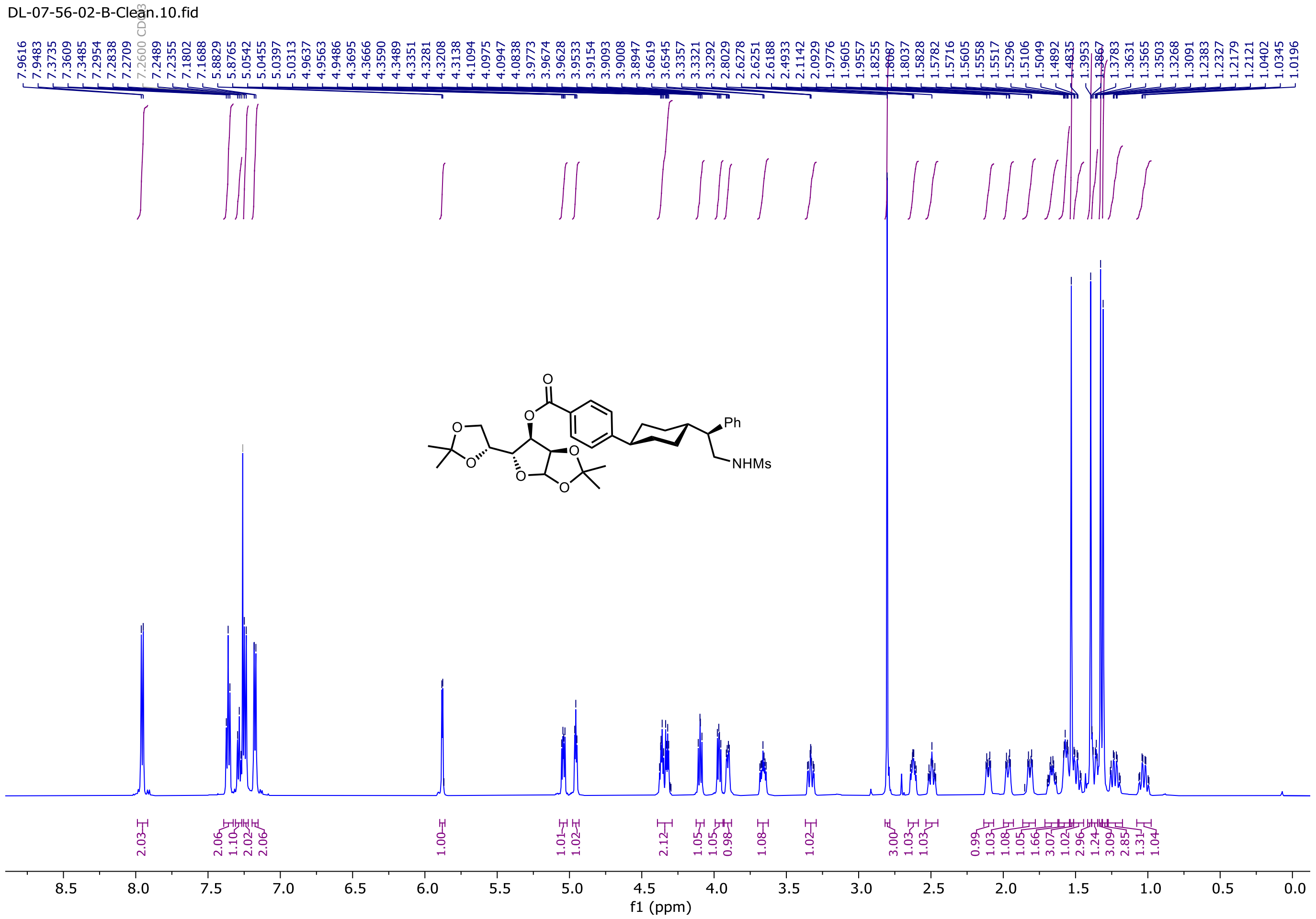

Figure S242. ^{1}**H-NMR of 27**

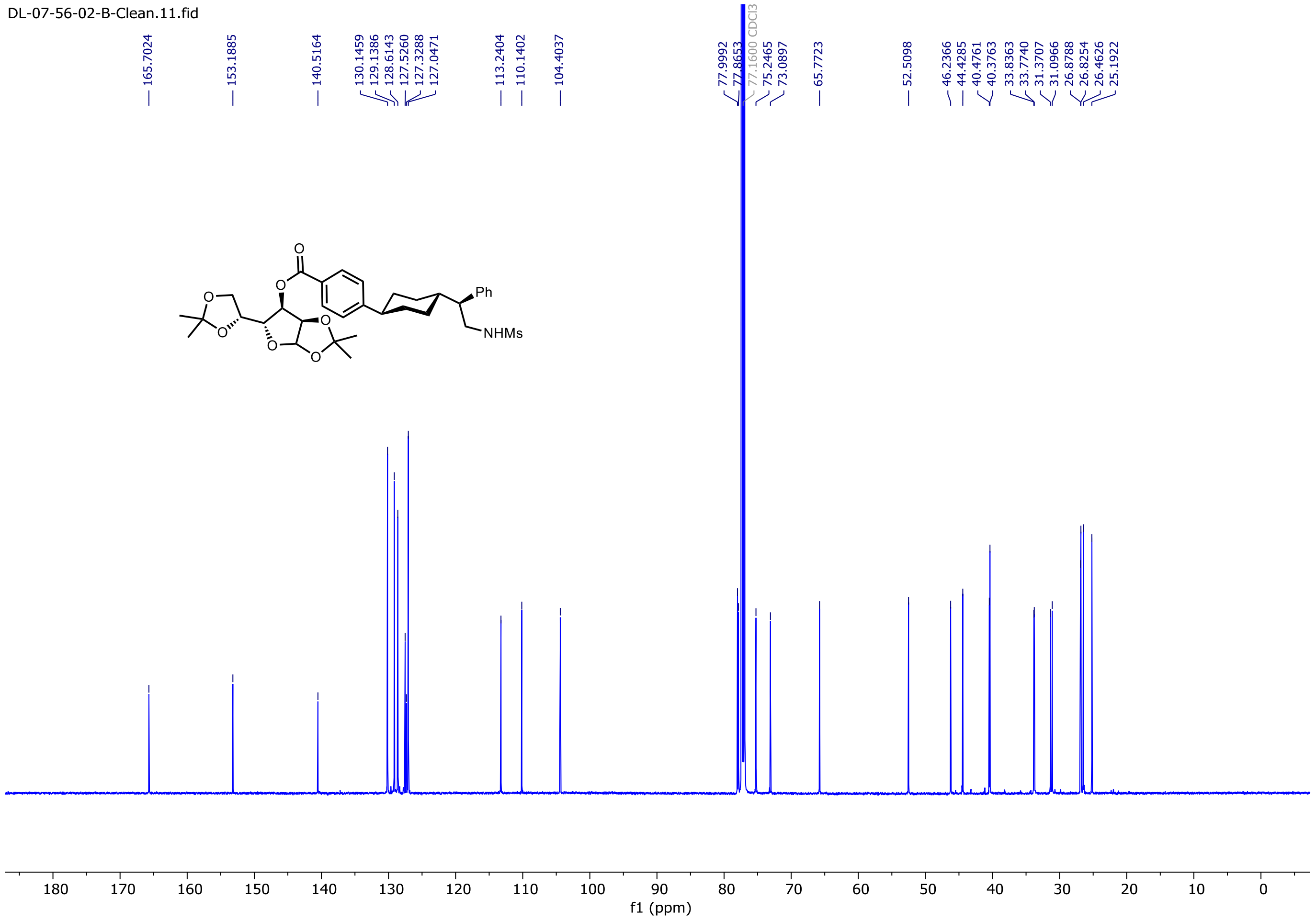

Figure S243. ^{13}**C-NMR of 27**

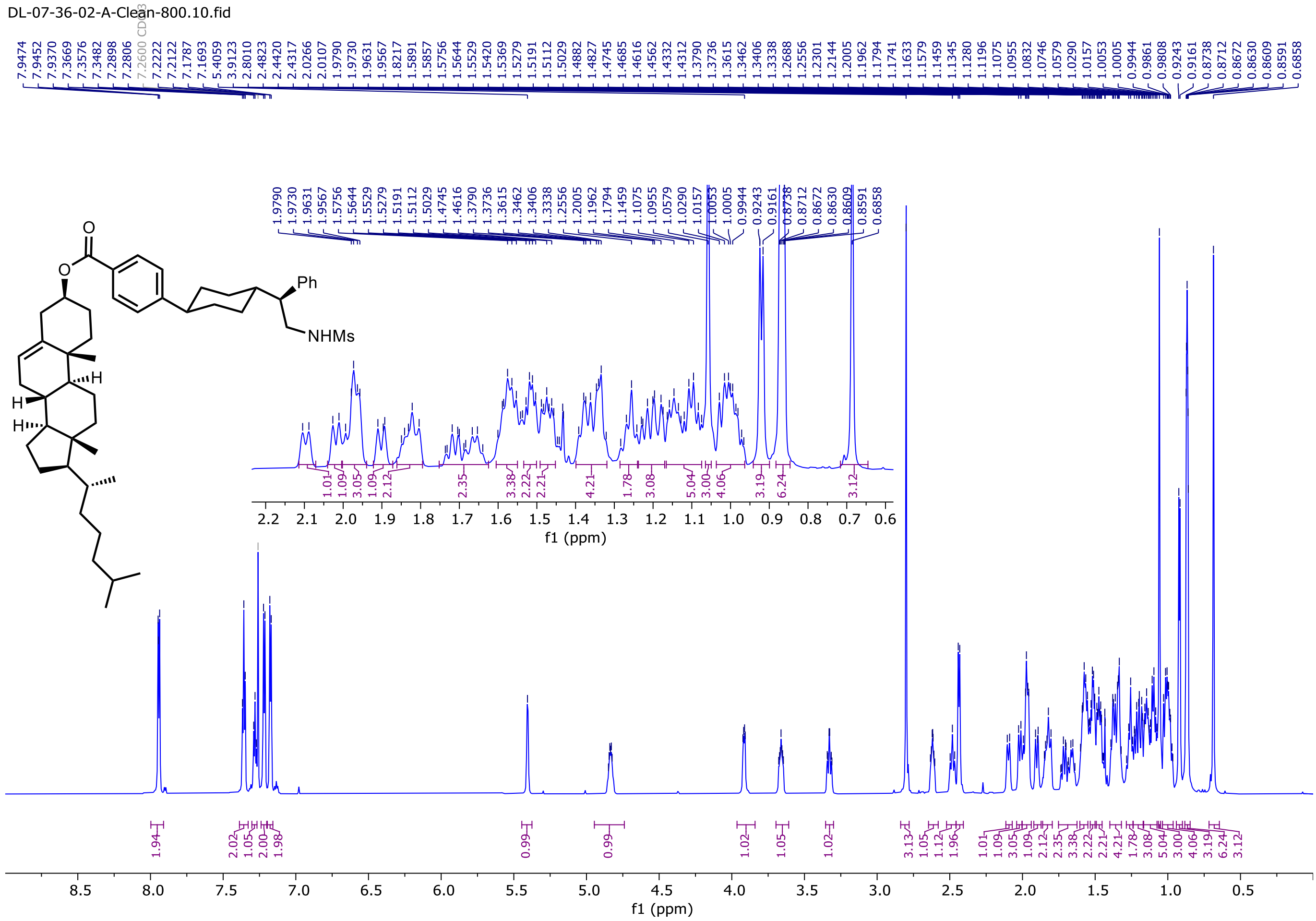

Figure S244. ^{1}H-NMR of 28

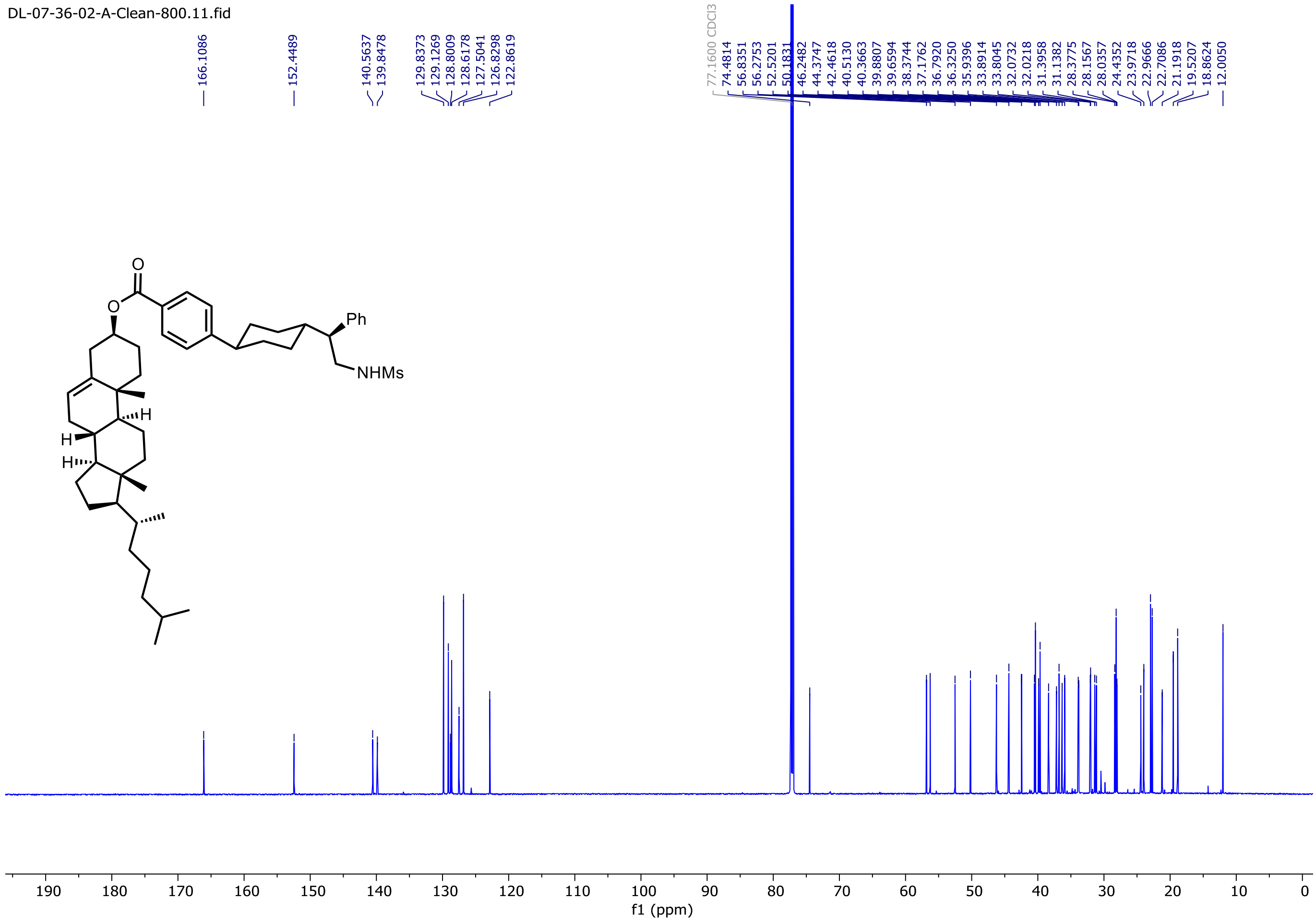

Figure S245. ^{13}C-NMR of 28

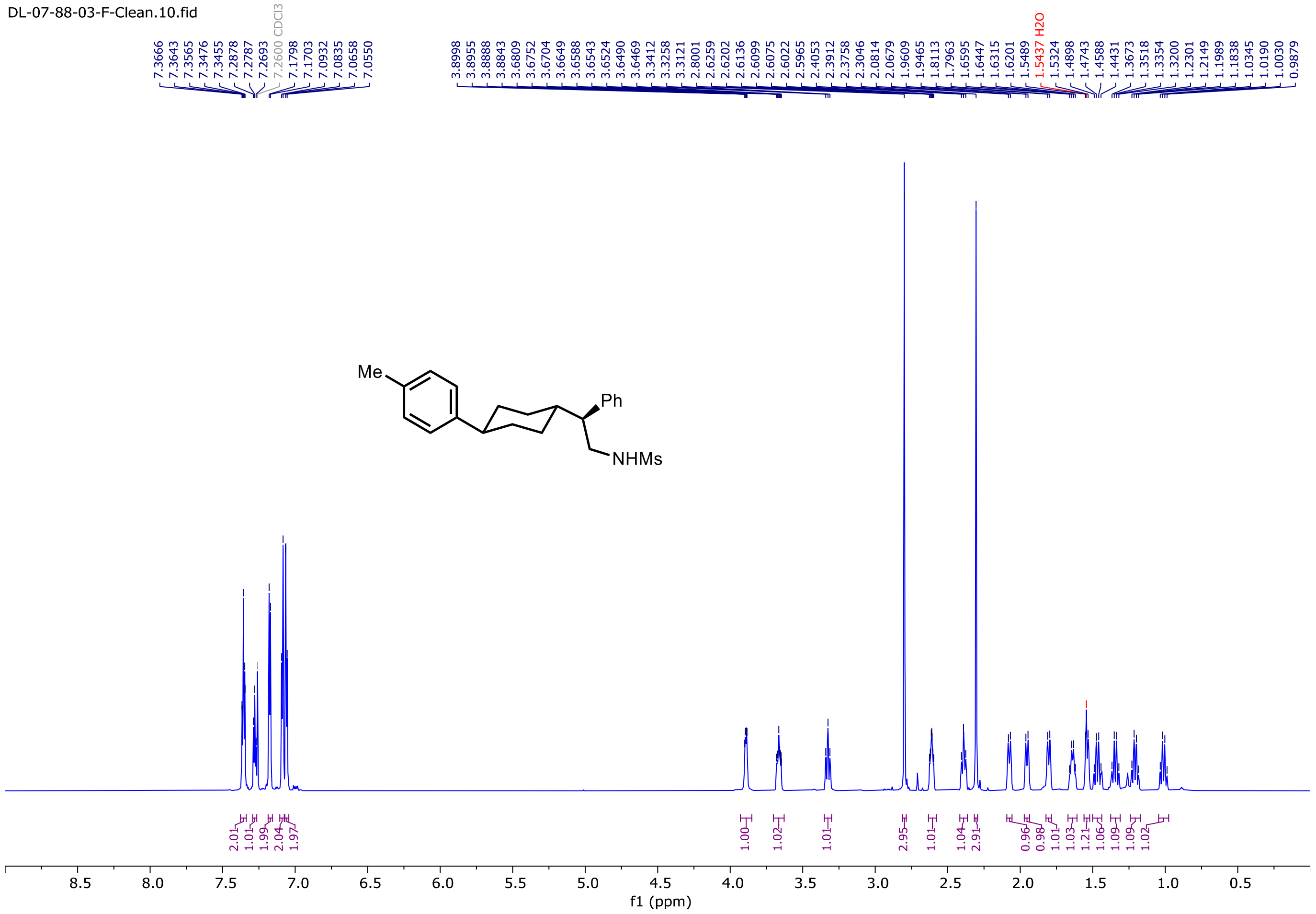

Figure S246. ^{1}H-NMR of 29

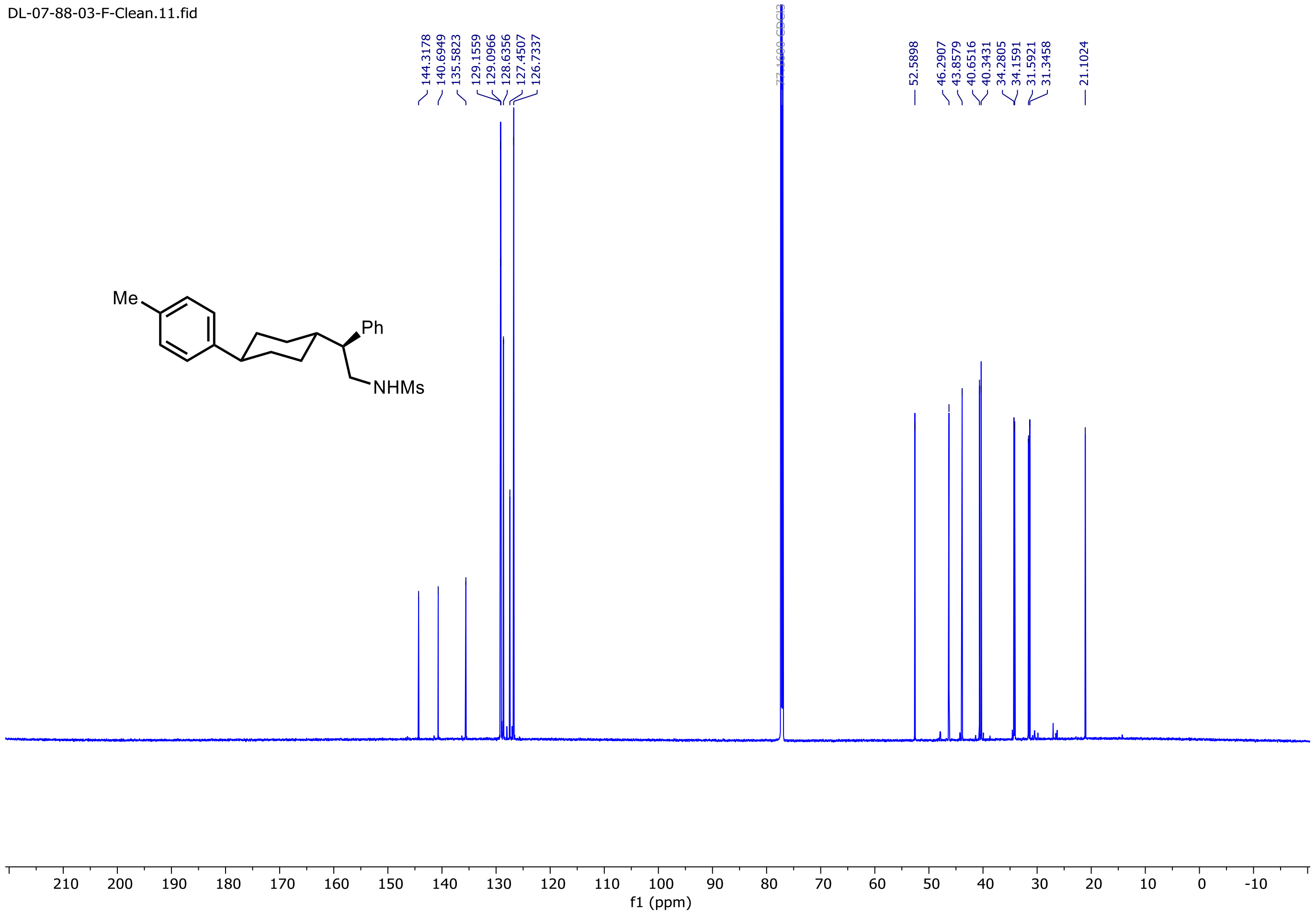

Figure S247. ^{13}C-NMR of 29

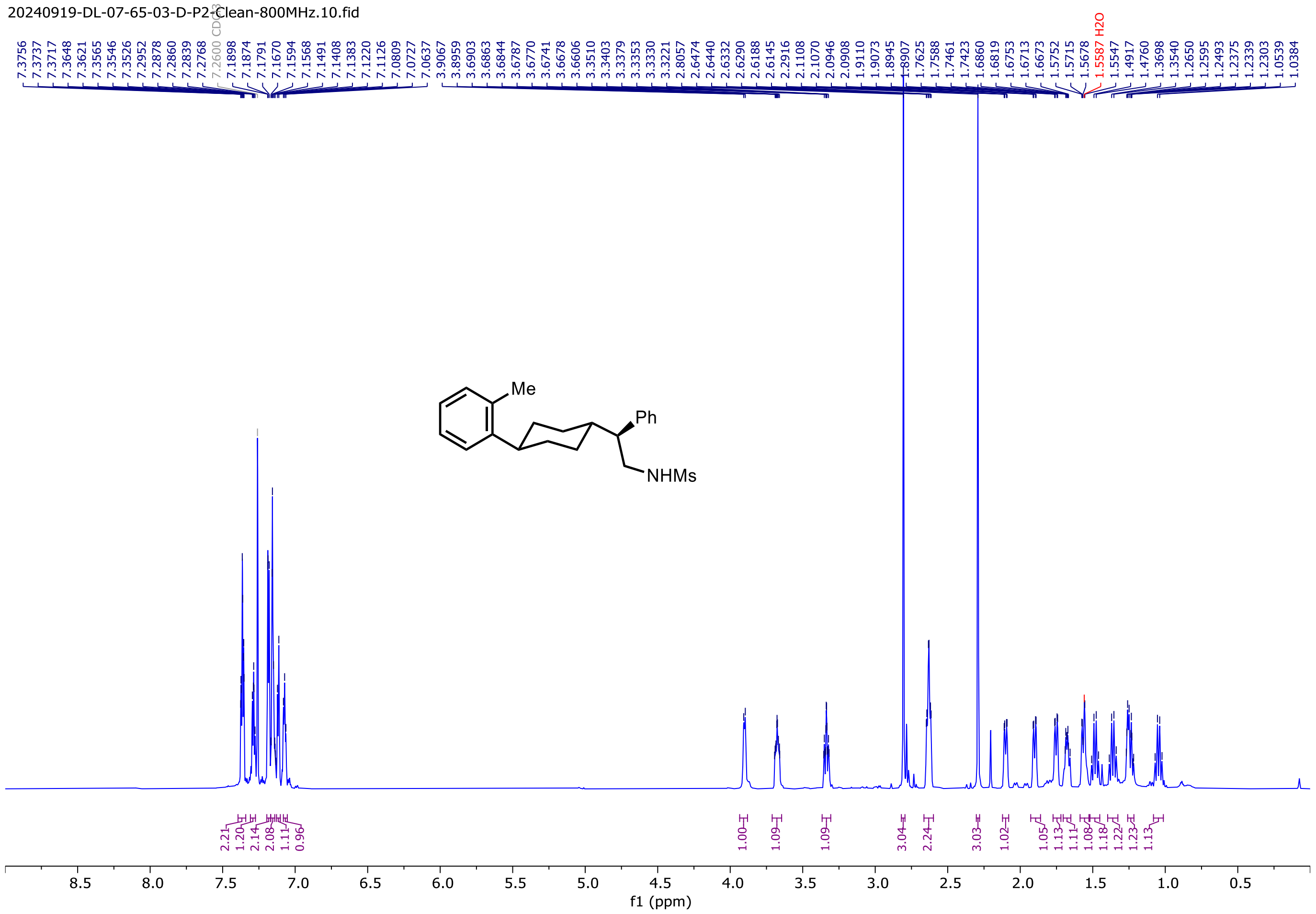

Figure S248. ¹H-NMR of 30

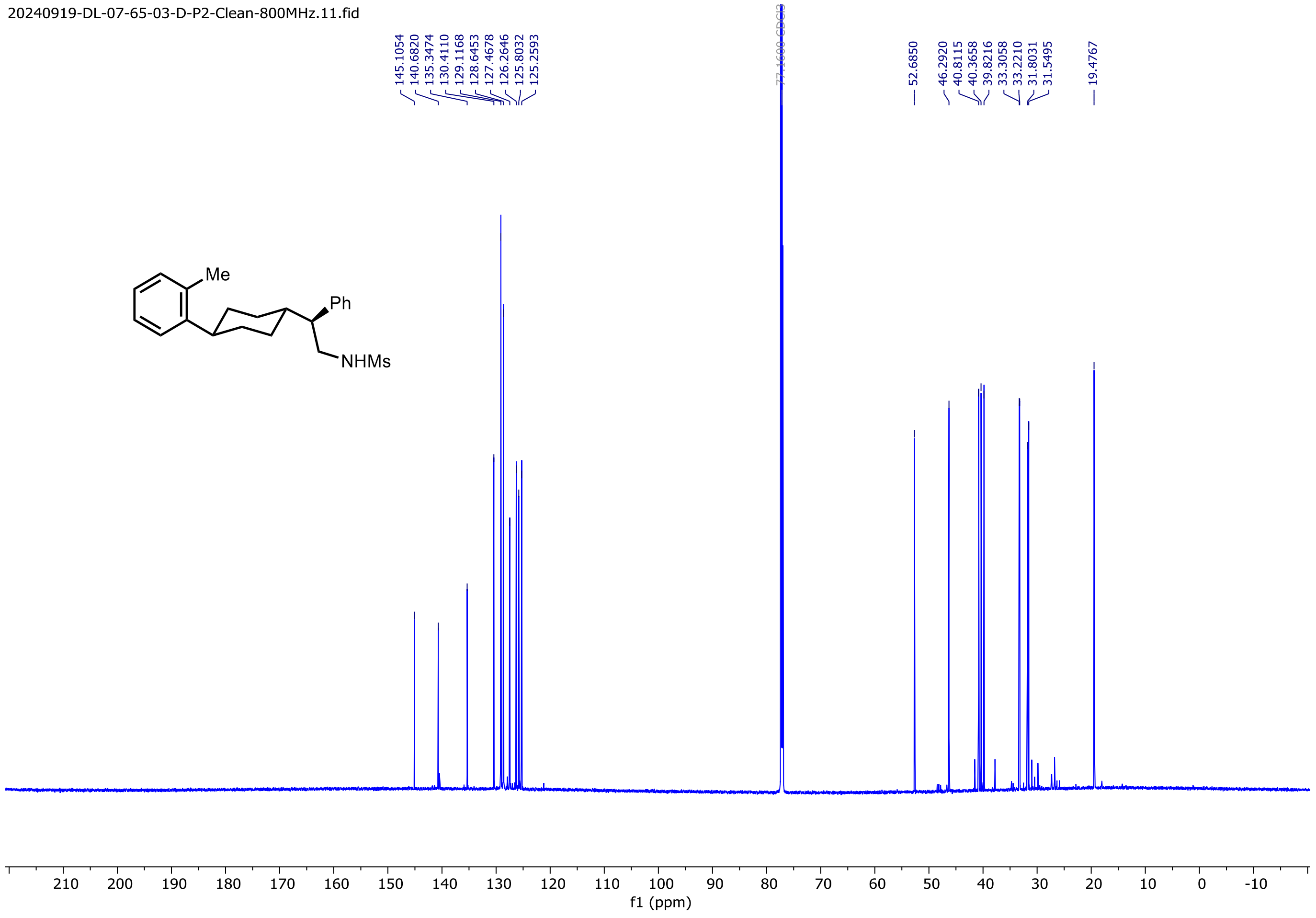

Figure S249. ¹³C-NMR of 30

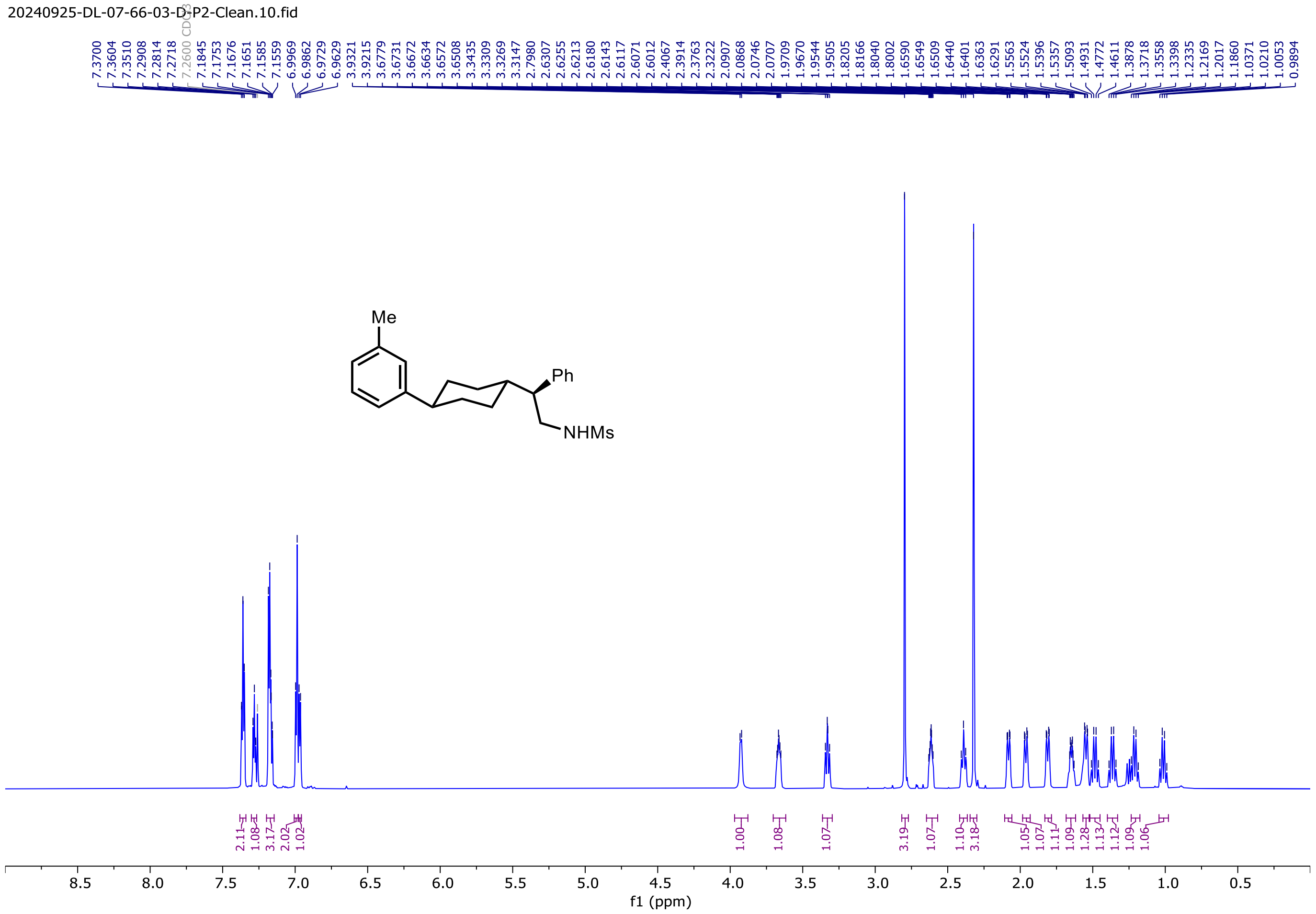

Figure S250. ^{1}H-NMR of 31

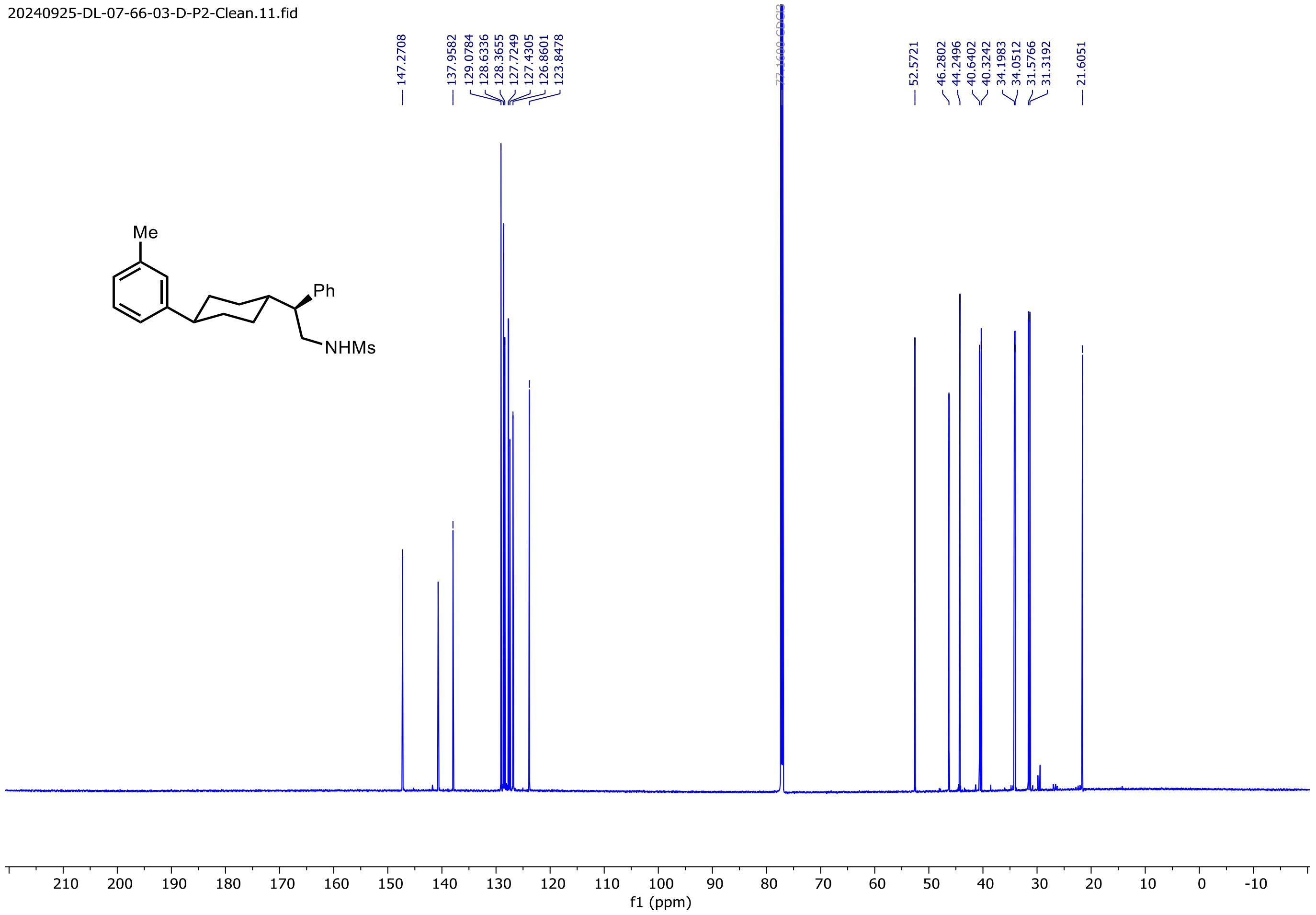

Figure S251. ^{13}C-NMR of 31

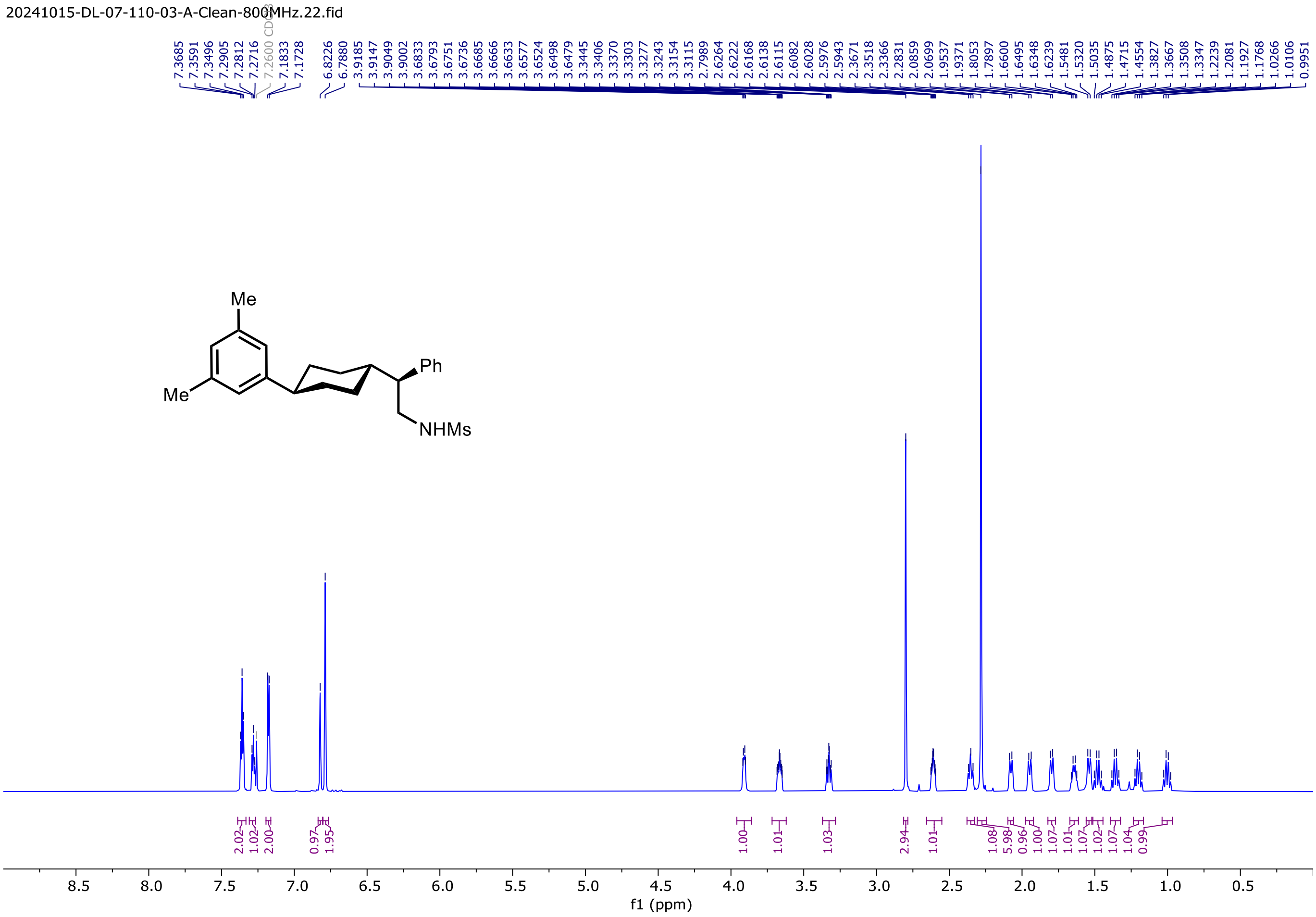

Figure S252. ^{1}**H-NMR of 32**

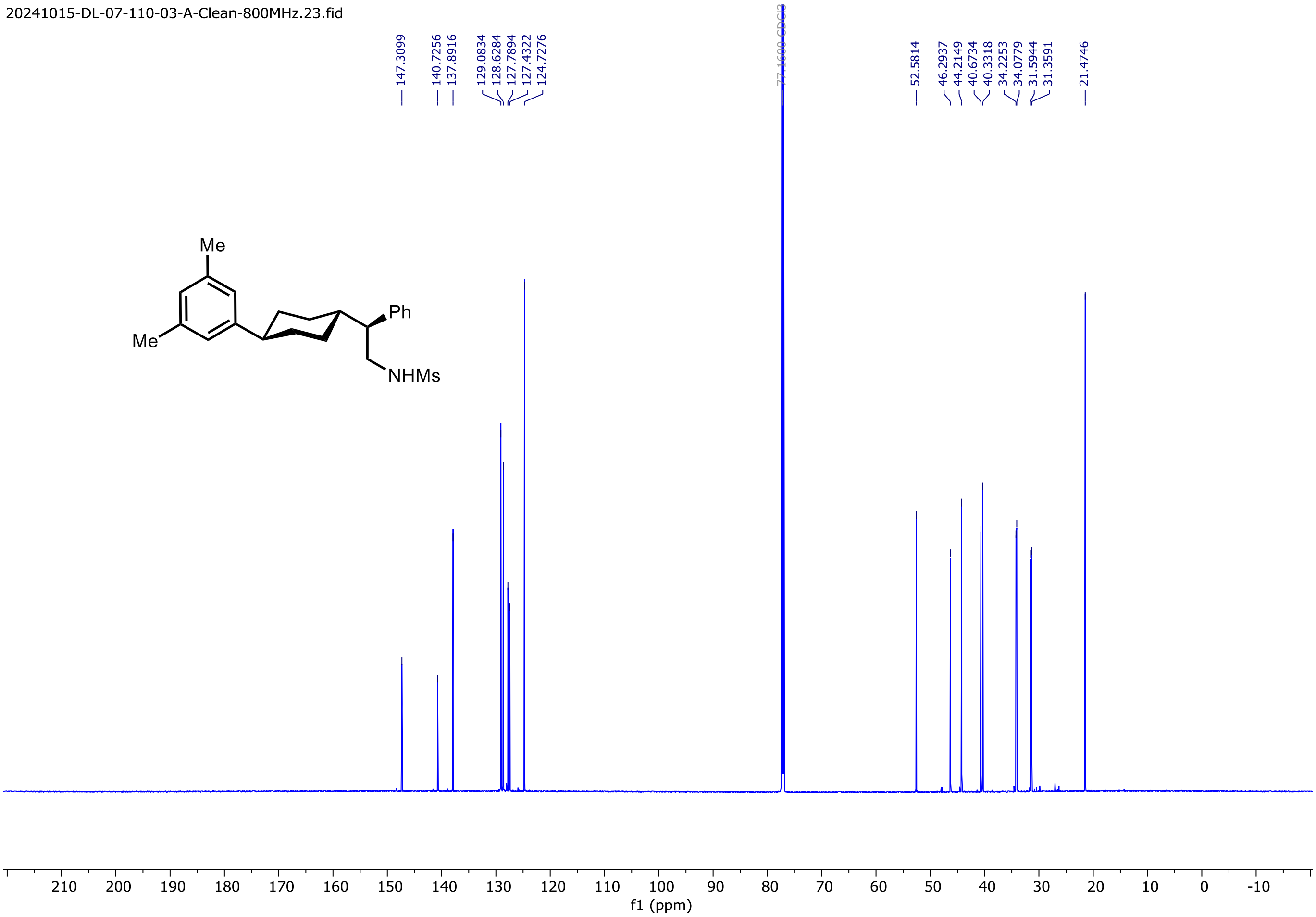

Figure S253. ^{13}**C-NMR of 32**

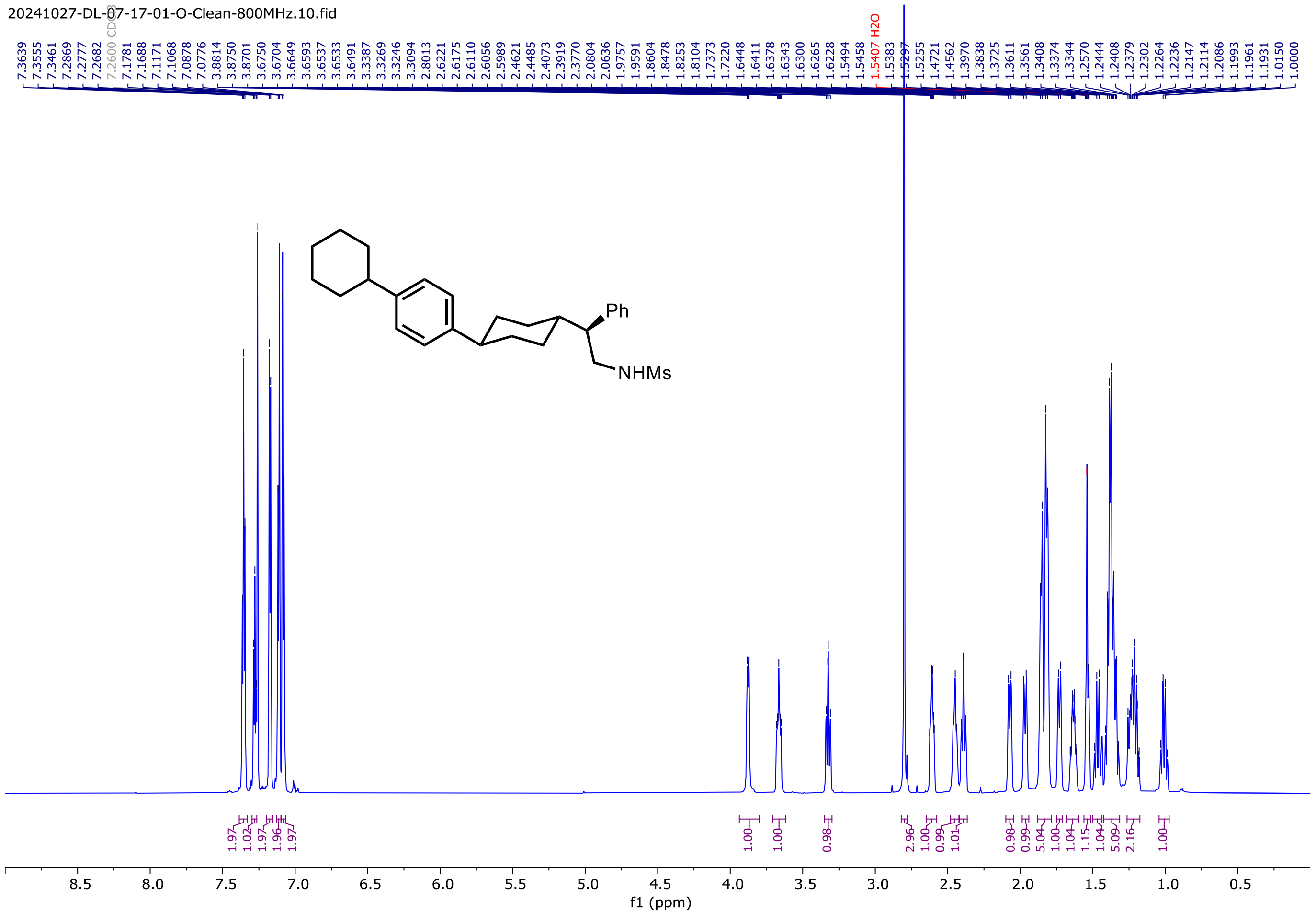

Figure S254. ^{1}H-NMR of 33

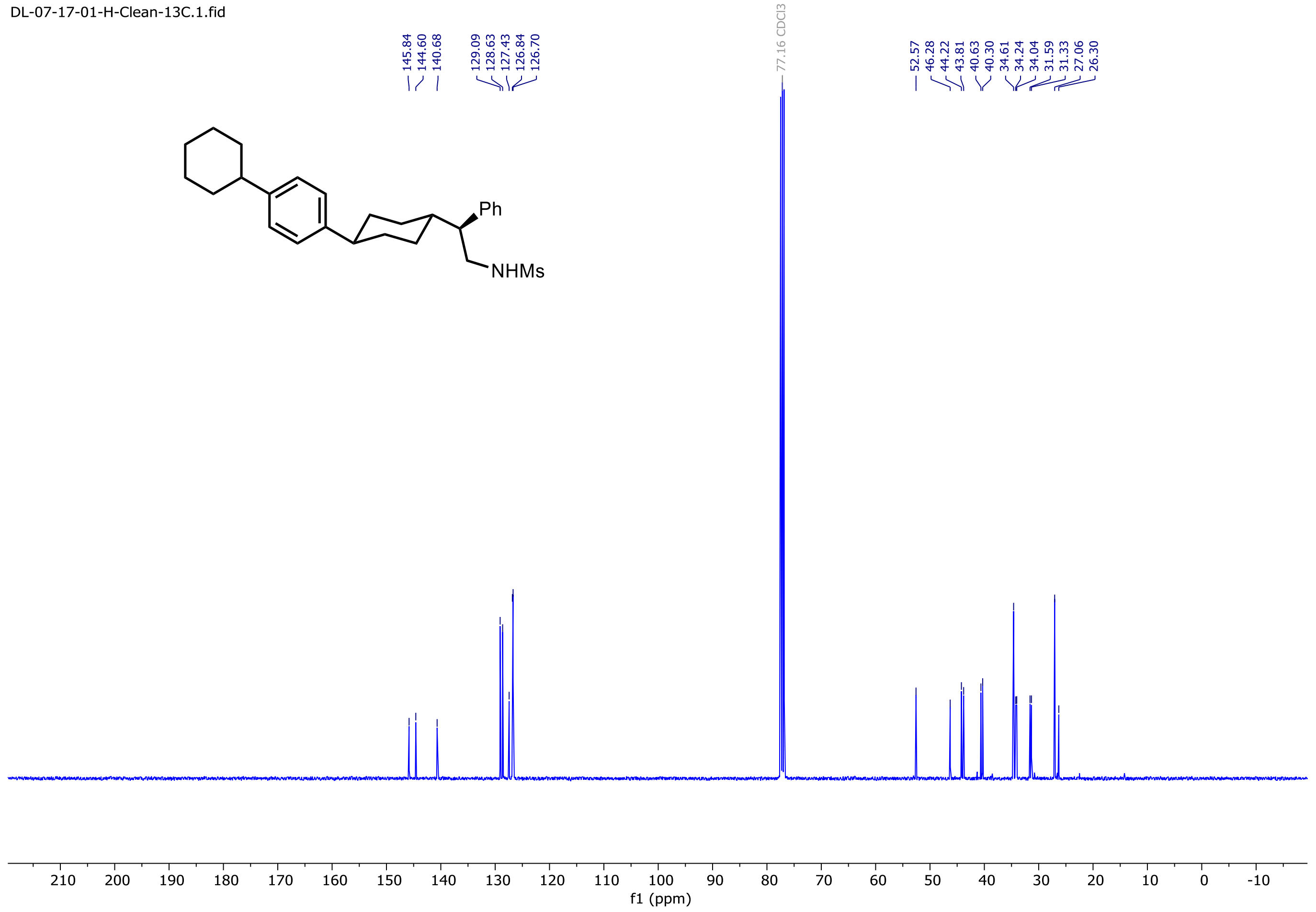

Figure S255. ^{13}C-NMR of 33

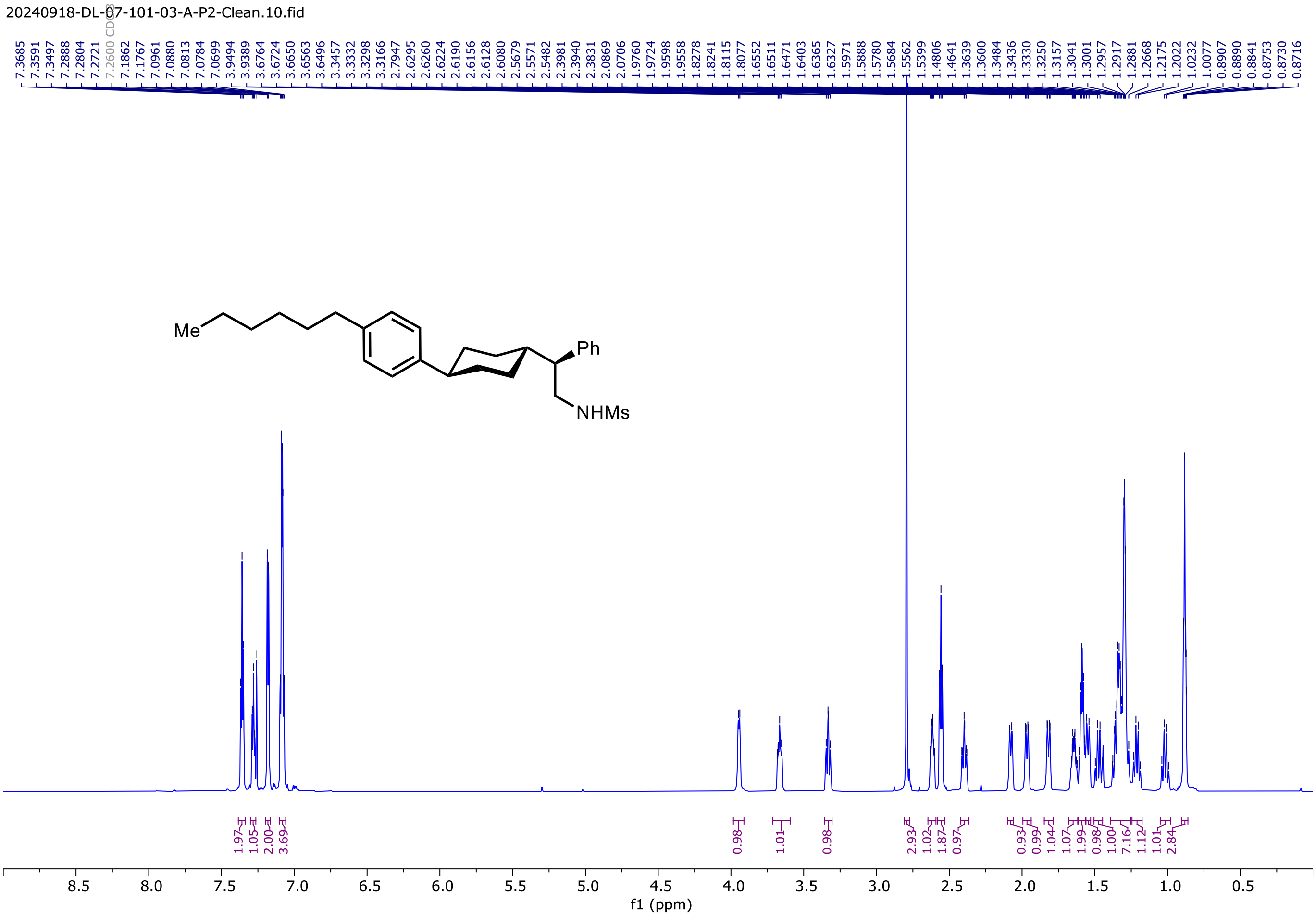

Figure S256. ^{1}H-NMR of 34

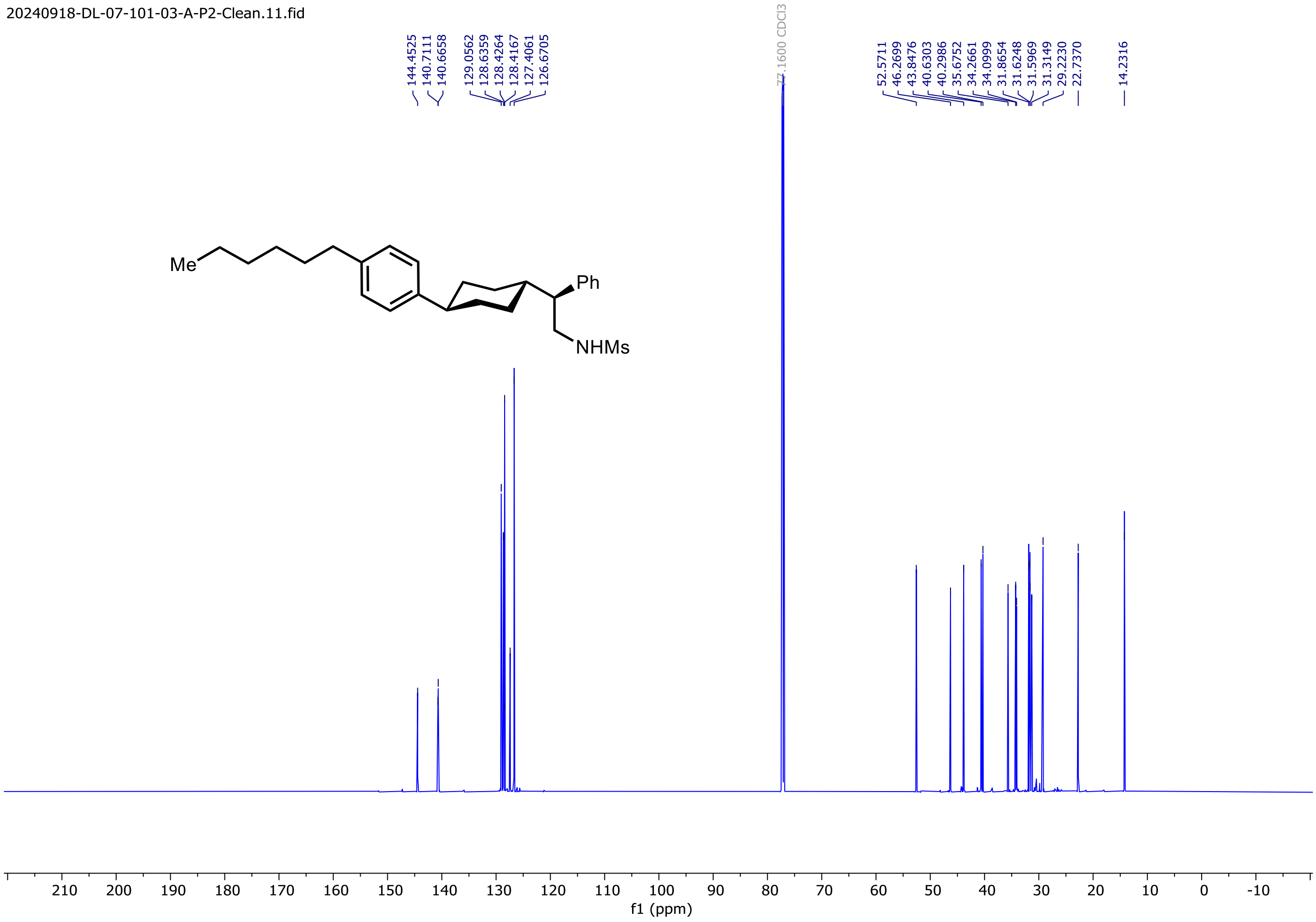

Figure S257. ^{13}C-NMR of 34

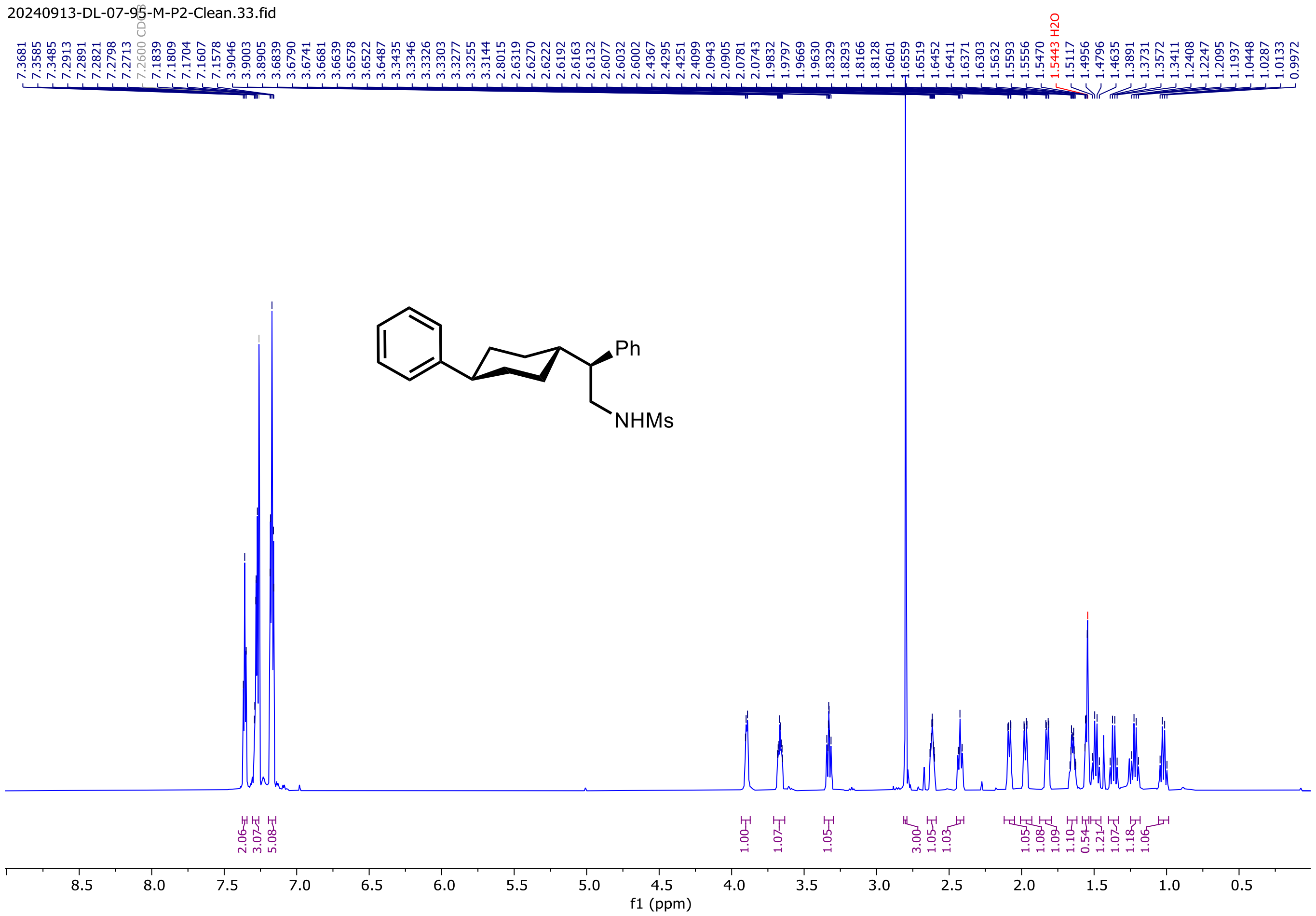

Figure S258. ^{1}H-NMR of 35

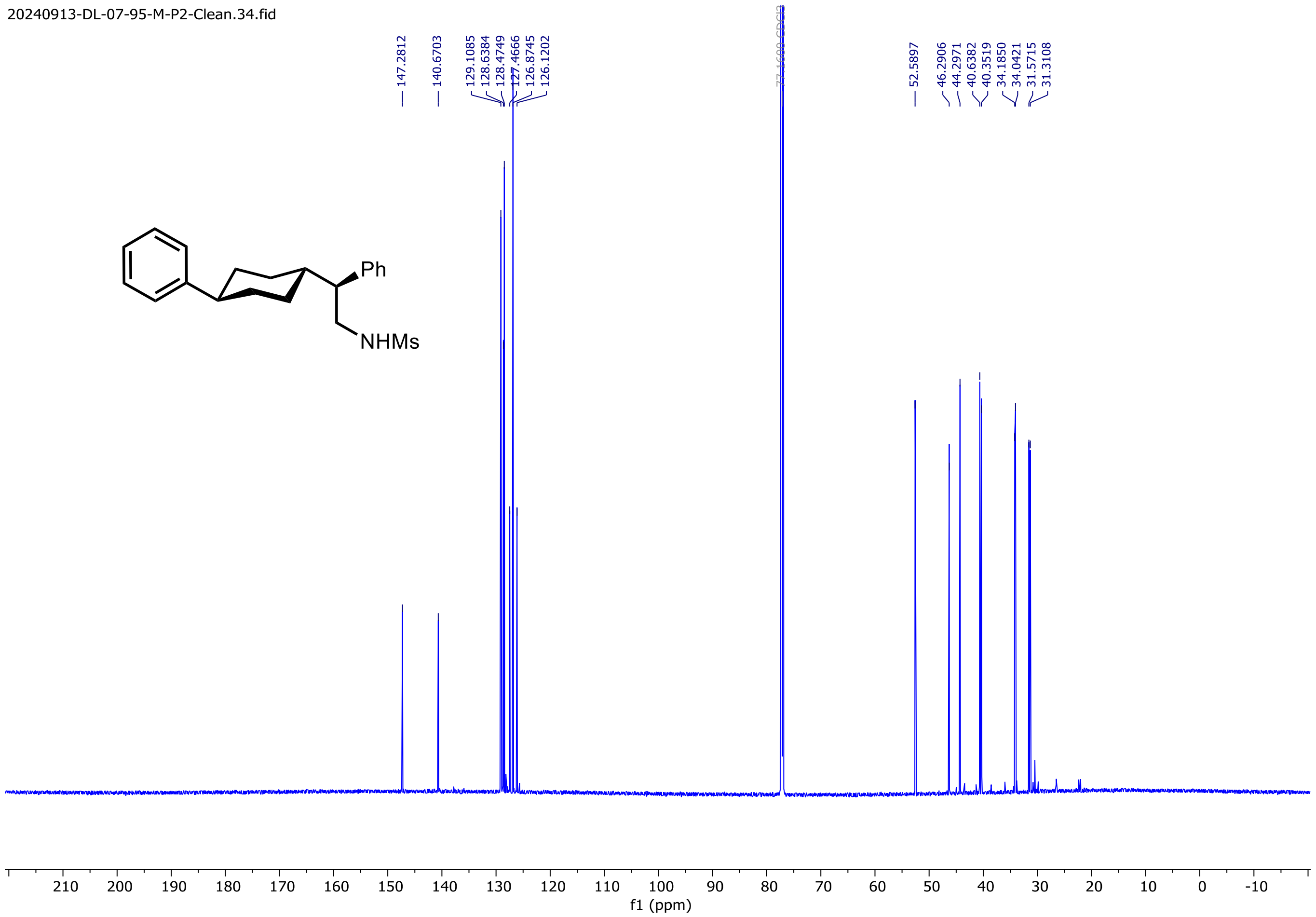

Figure S259. ^{13}C-NMR of 35

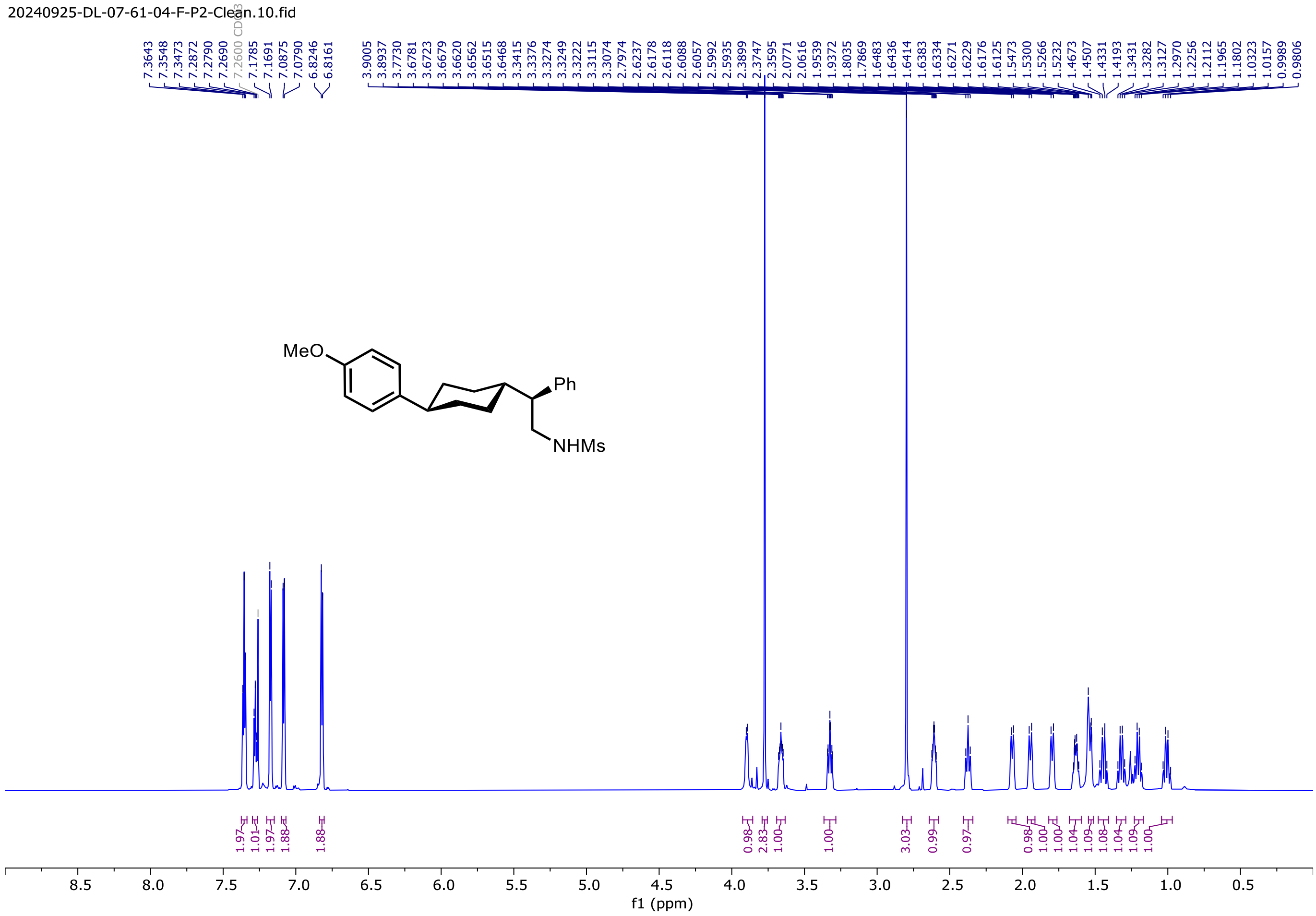

Figure S260. ^{1}**H-NMR of 36**

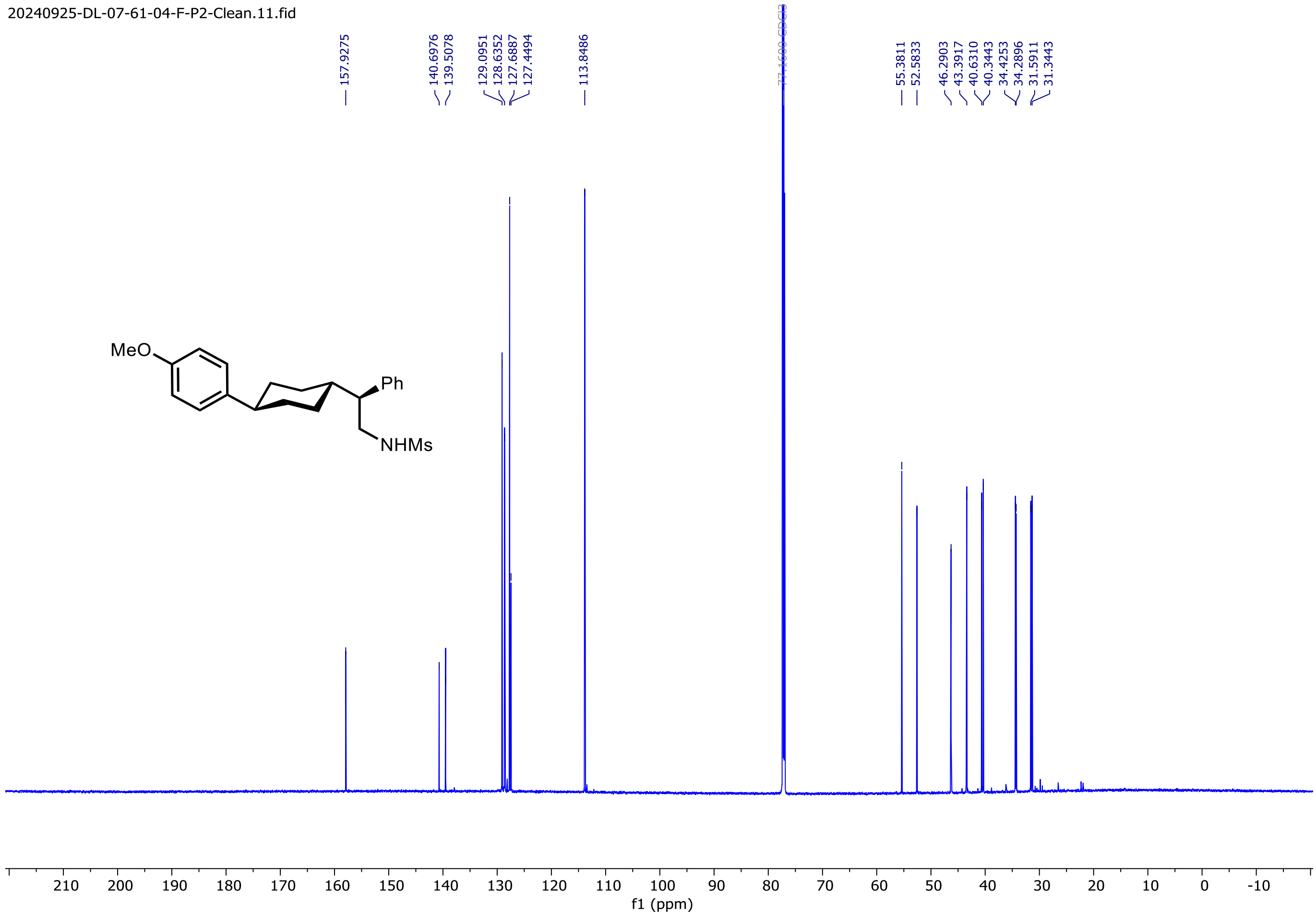

Figure S261. ^{13}**C-NMR of 36**

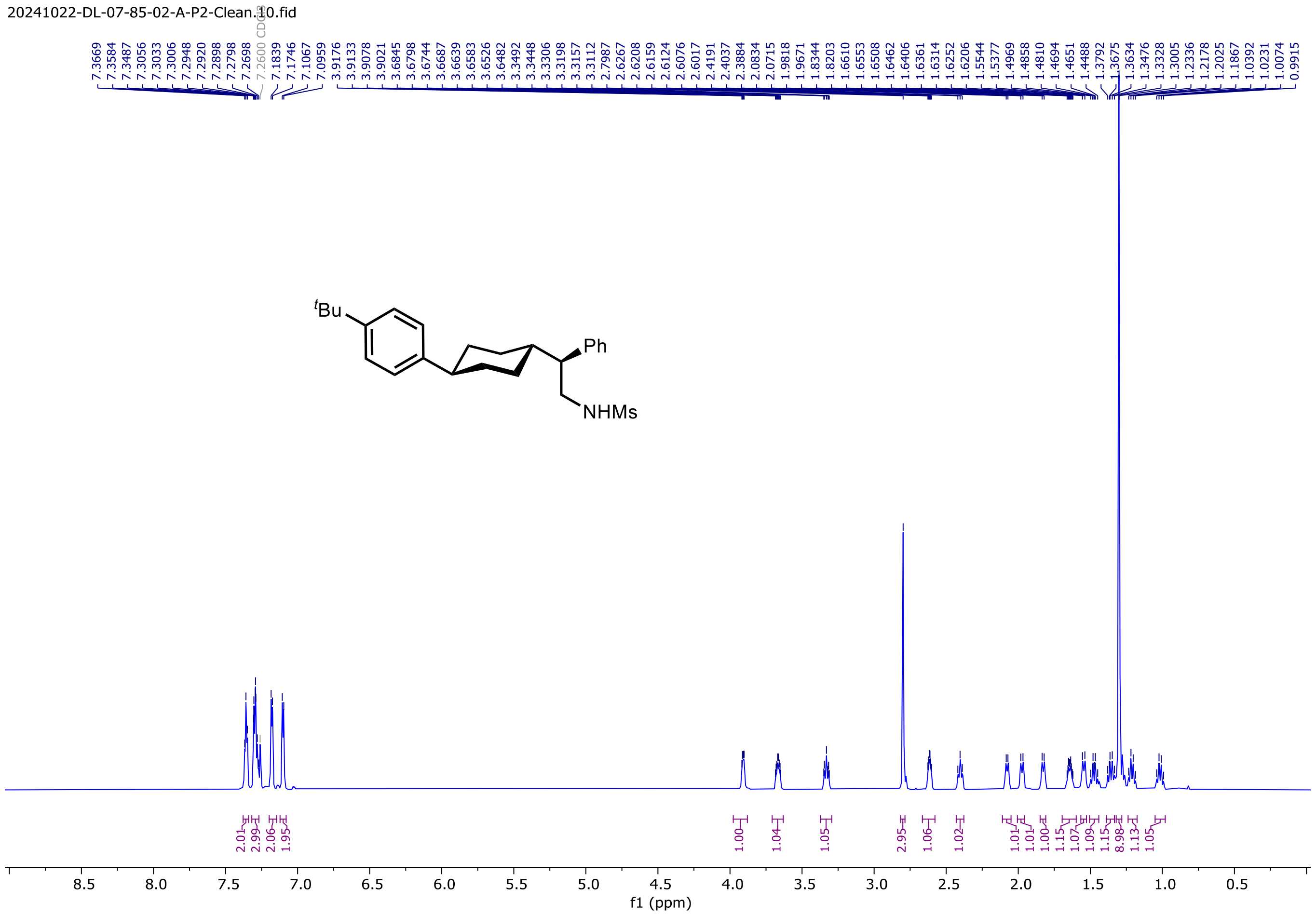

Figure S262. ^{1}H-NMR of 37

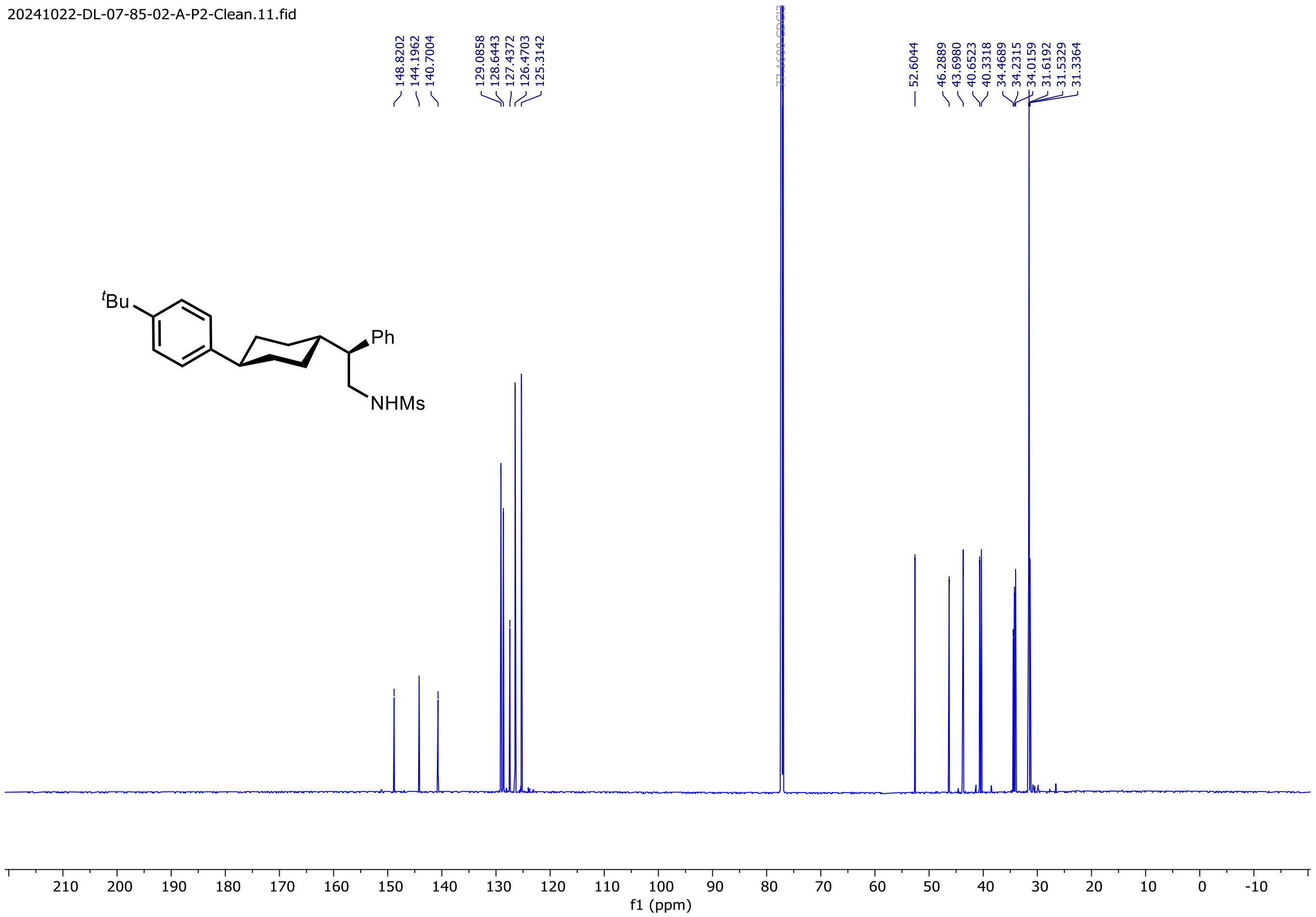

Figure S263. ^{13}C-NMR of 37

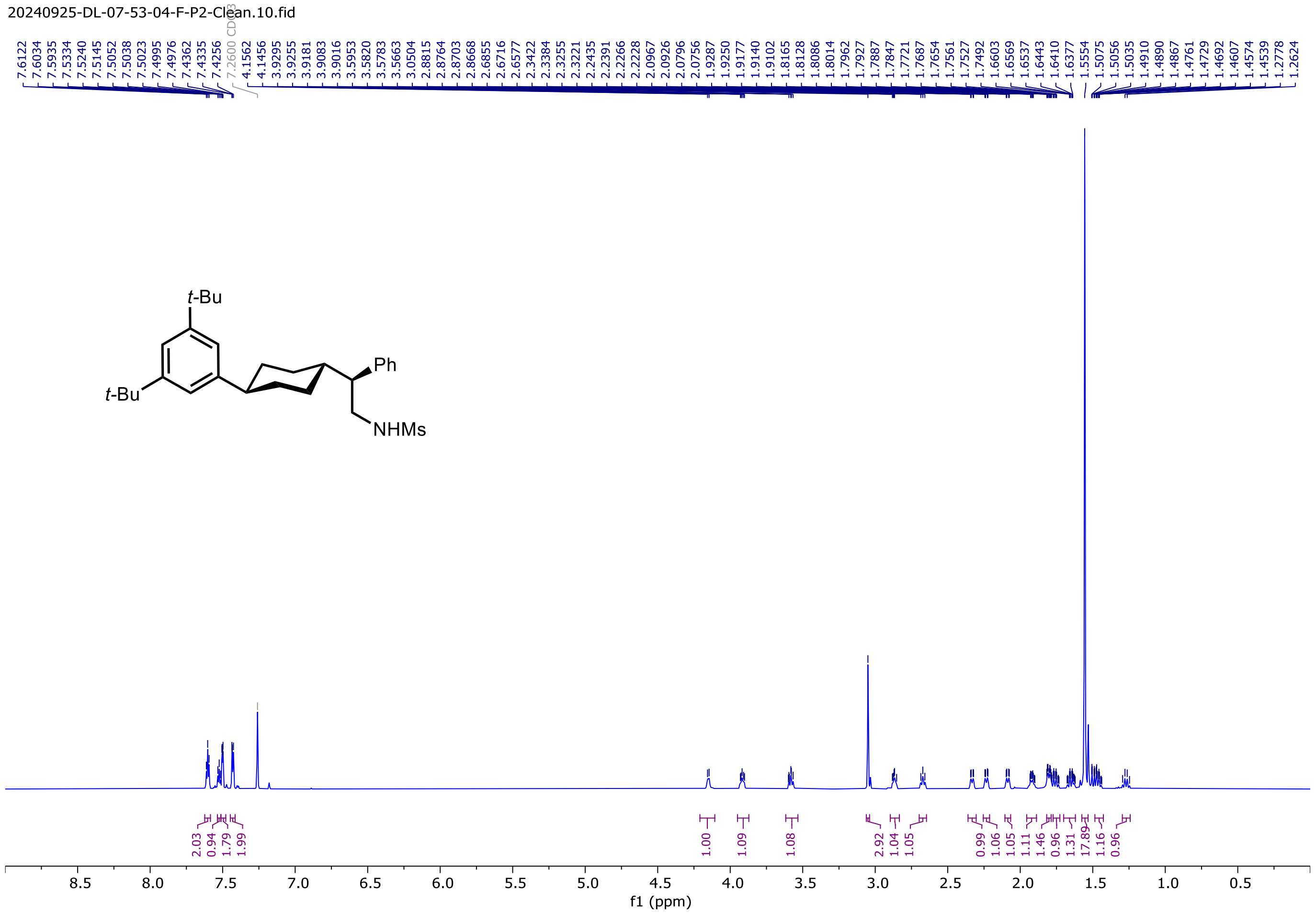

Figure S264. ^{1}H-NMR of 38

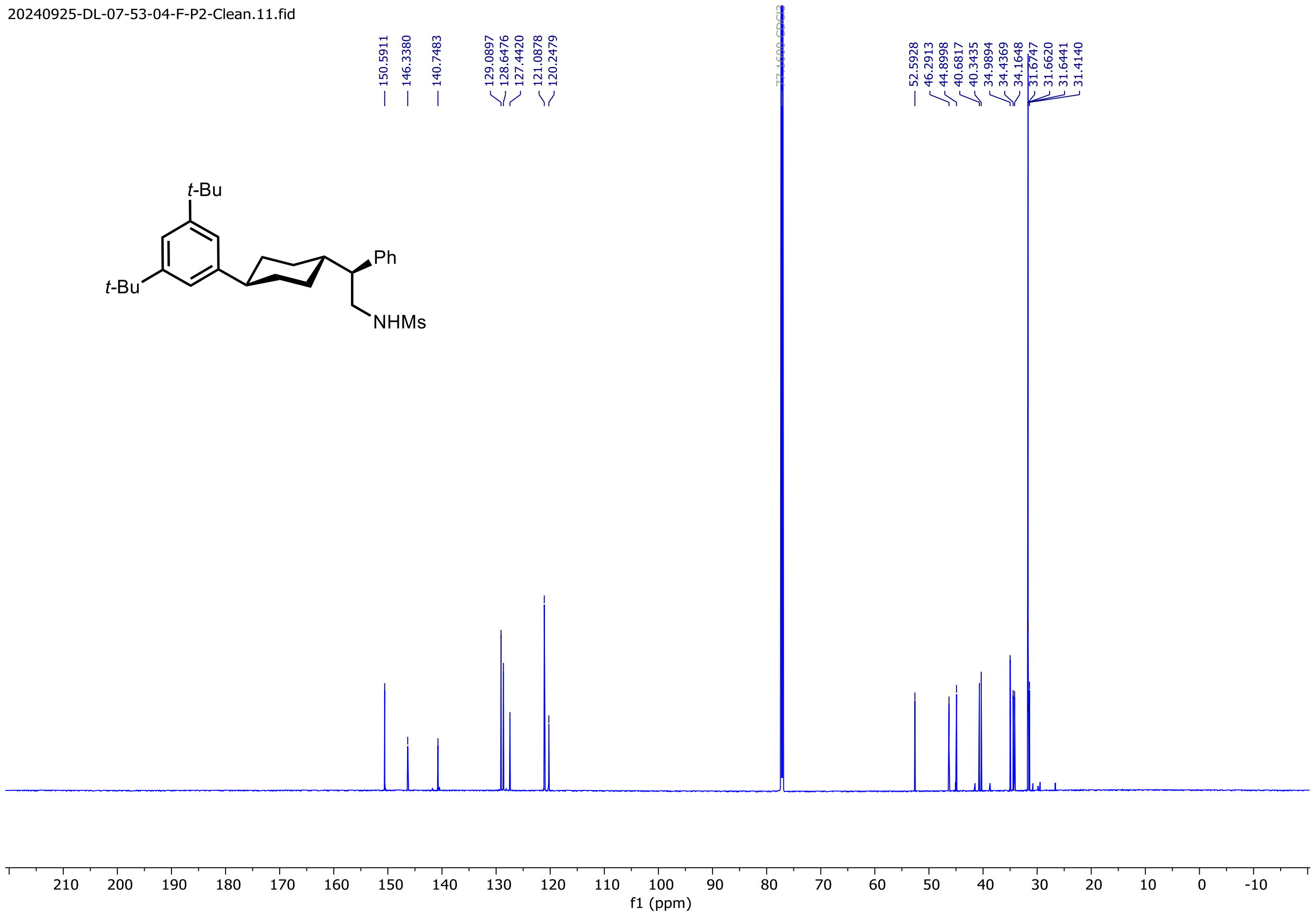

Figure S265. ^{13}C-NMR of 38

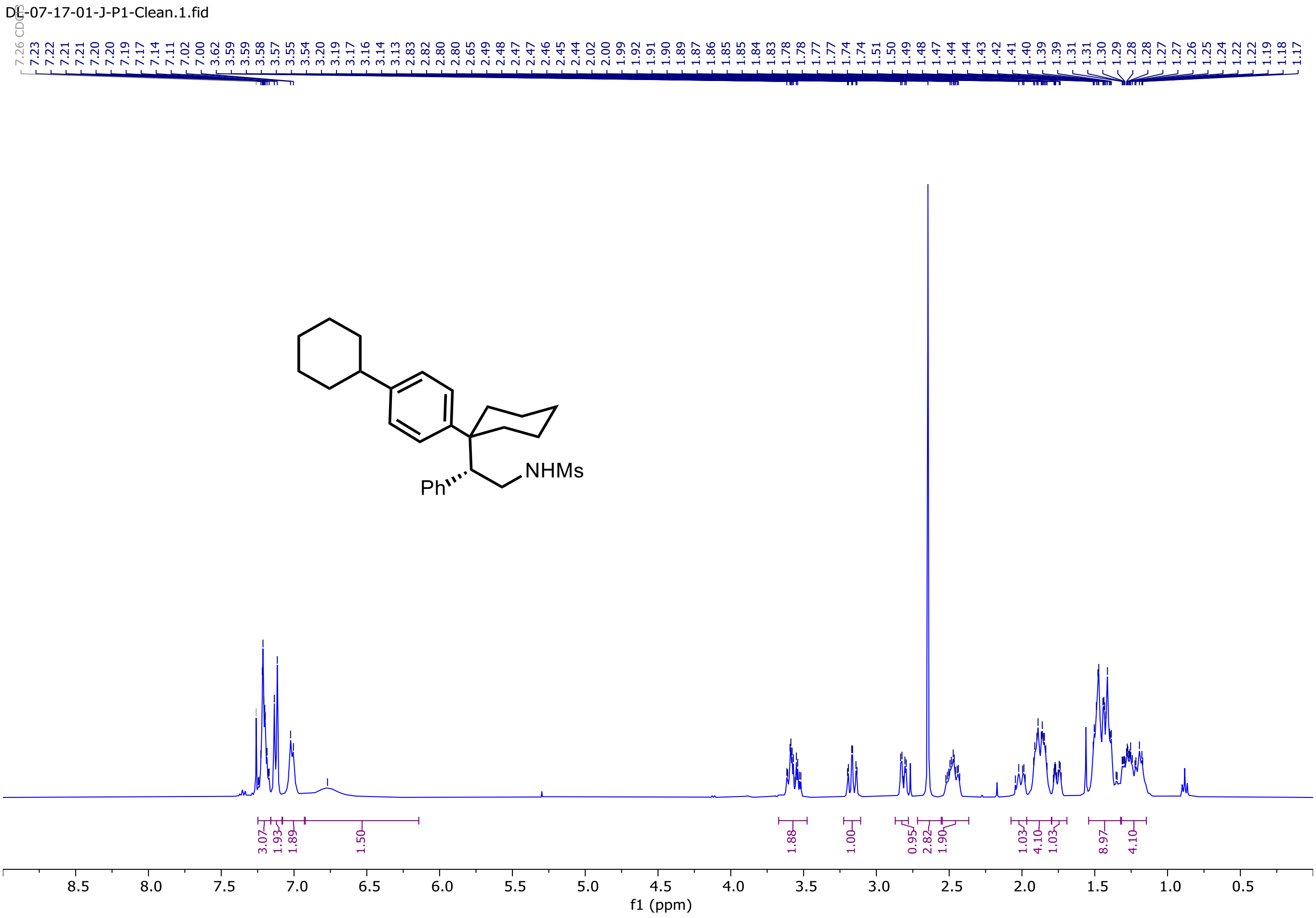

Figure S266. ^{1}H-NMR of 39

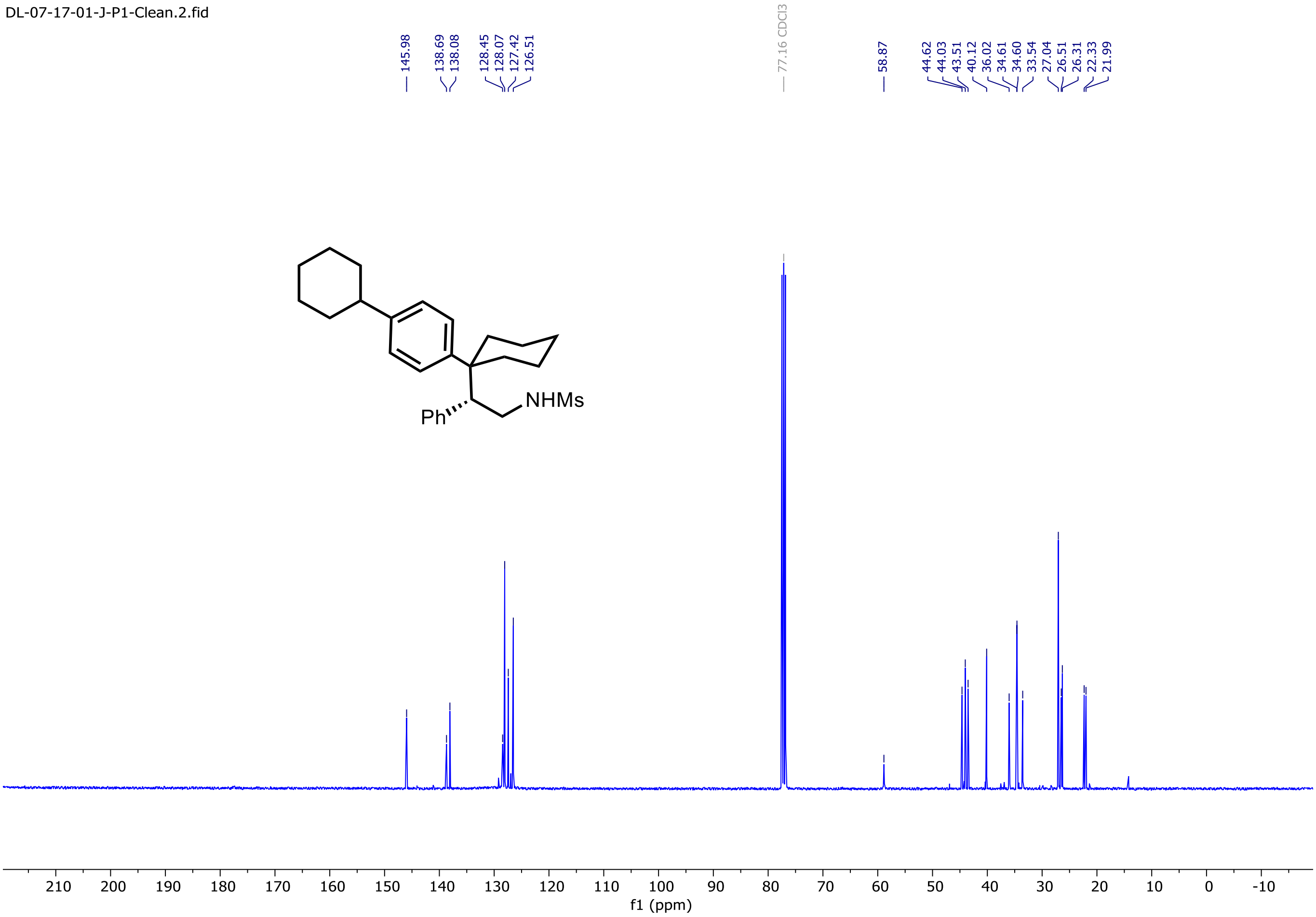

Figure S267. ^{13}C-NMR of 39

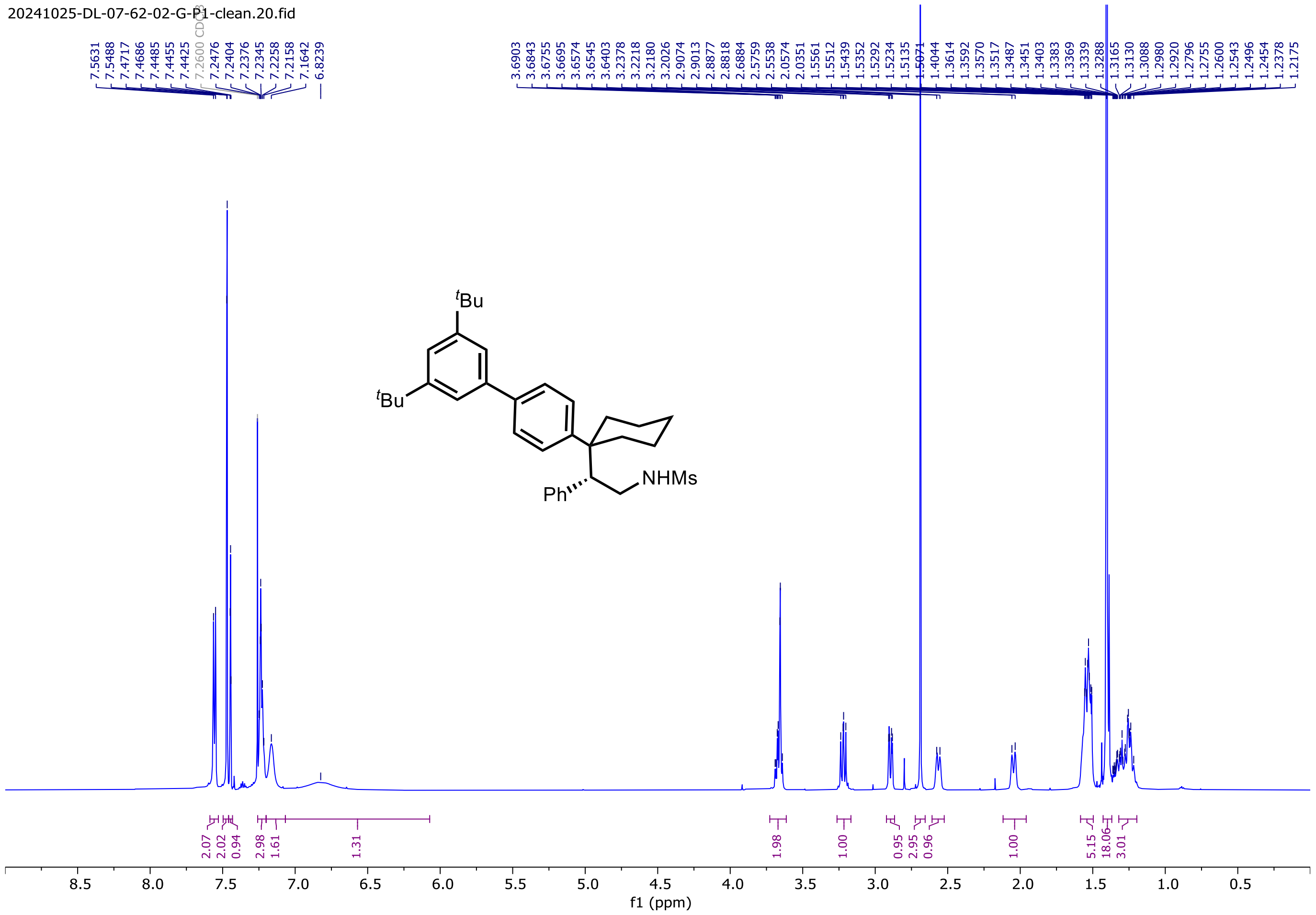

Figure S268. ^{1}**H-NMR of 40**

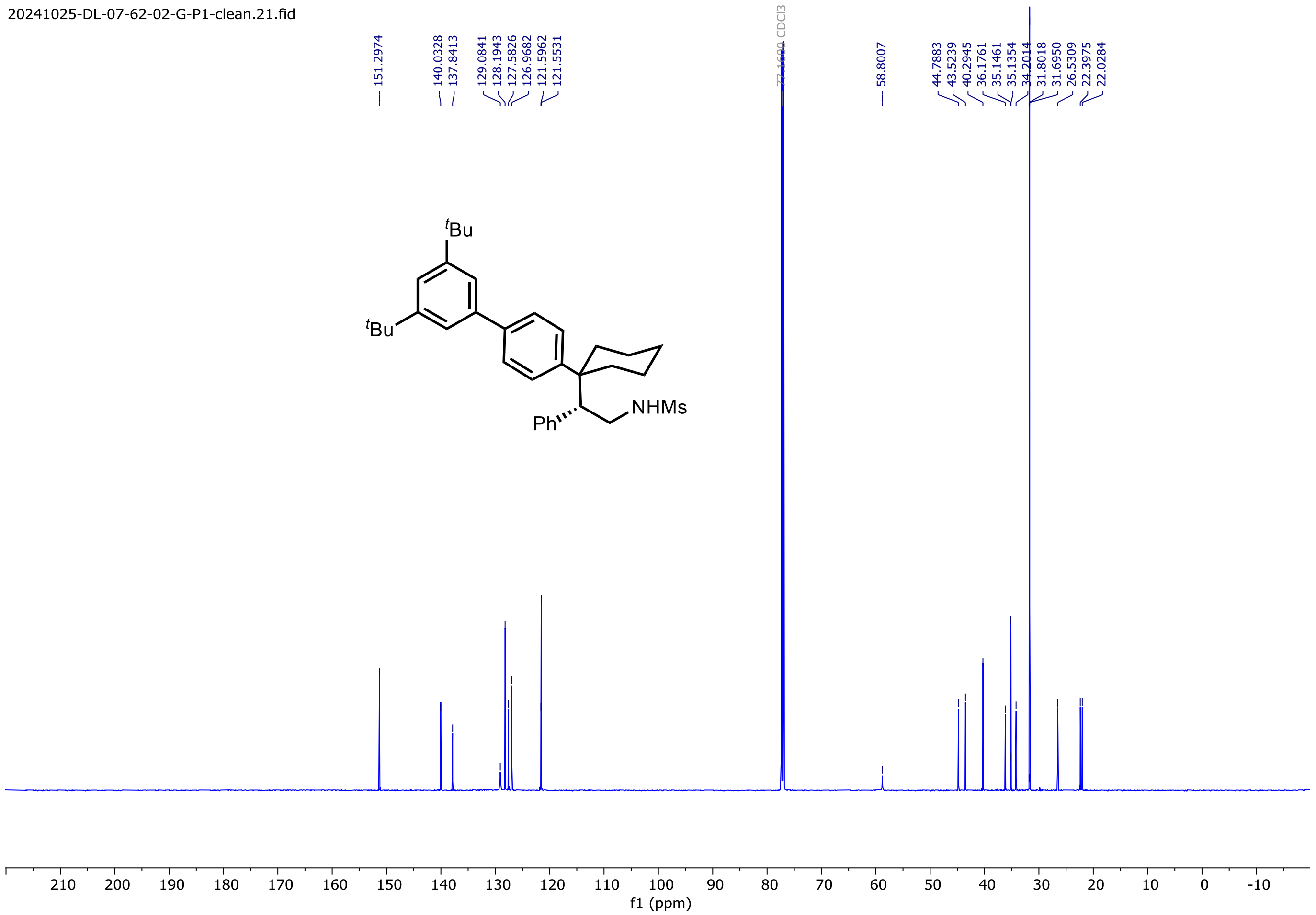

Figure S269. ^{13}**C-NMR of 40**

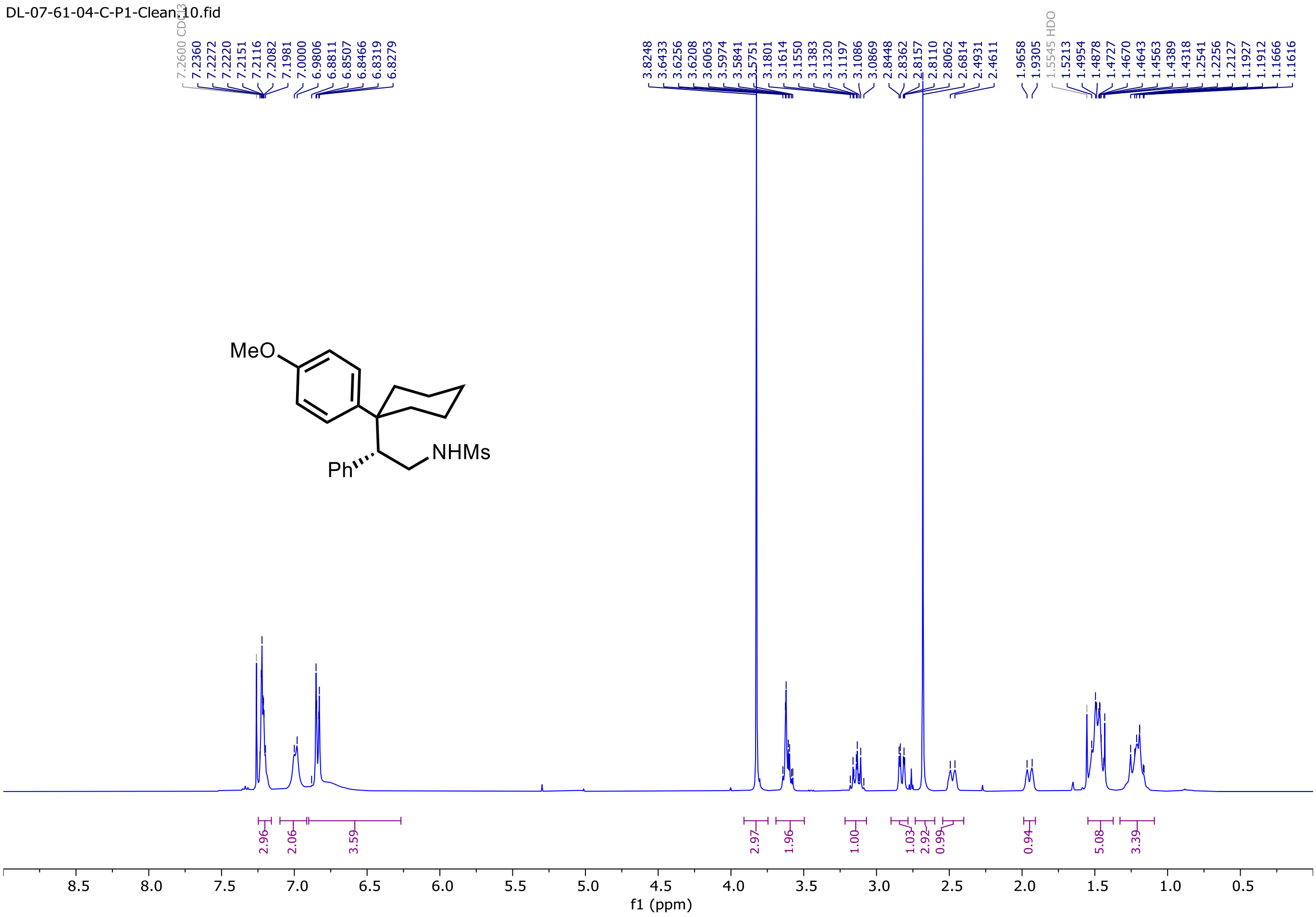

Figure S270. ^{1}**H-NMR of 41**

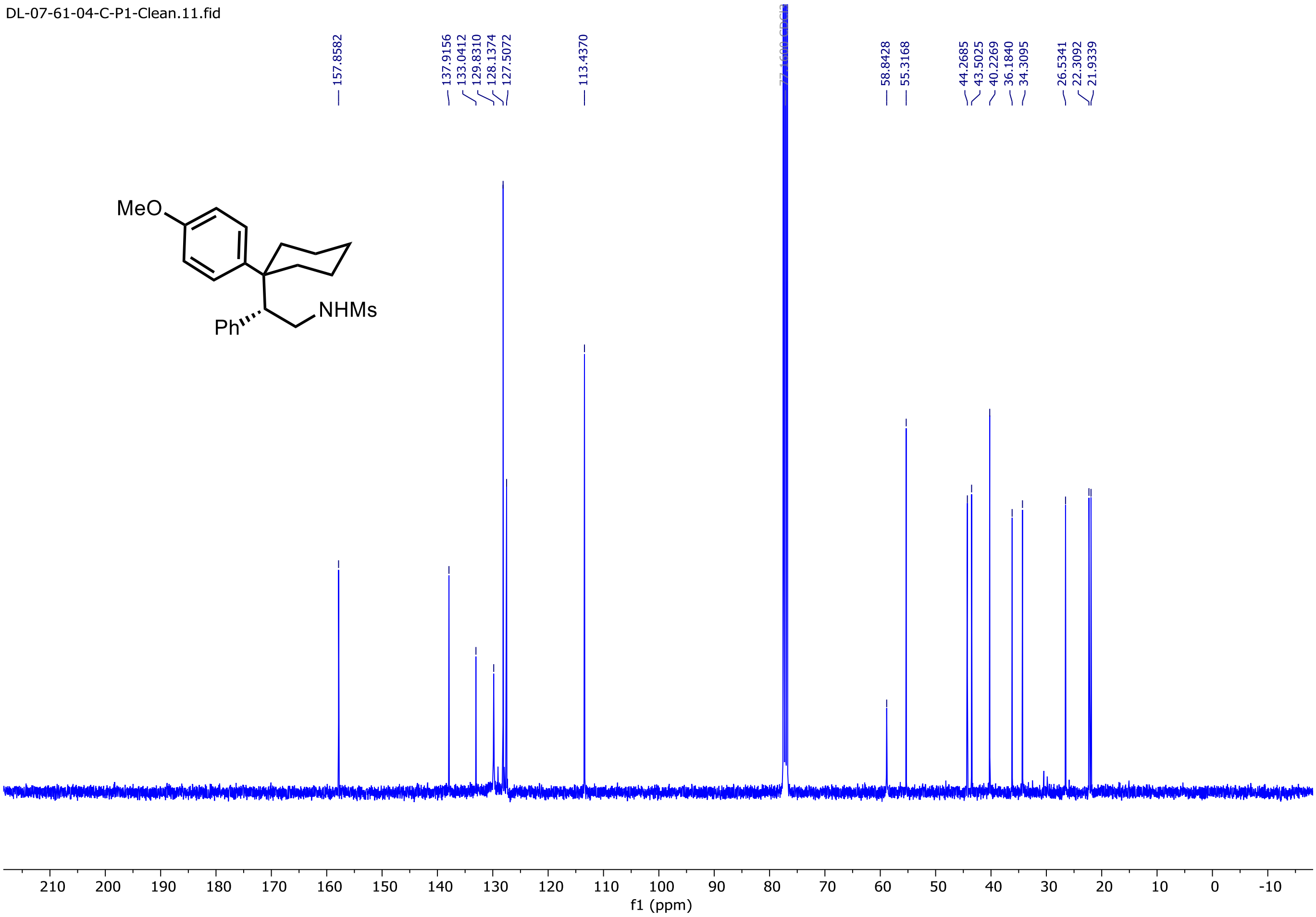

Figure S271. ^{13}**C-NMR of 41**

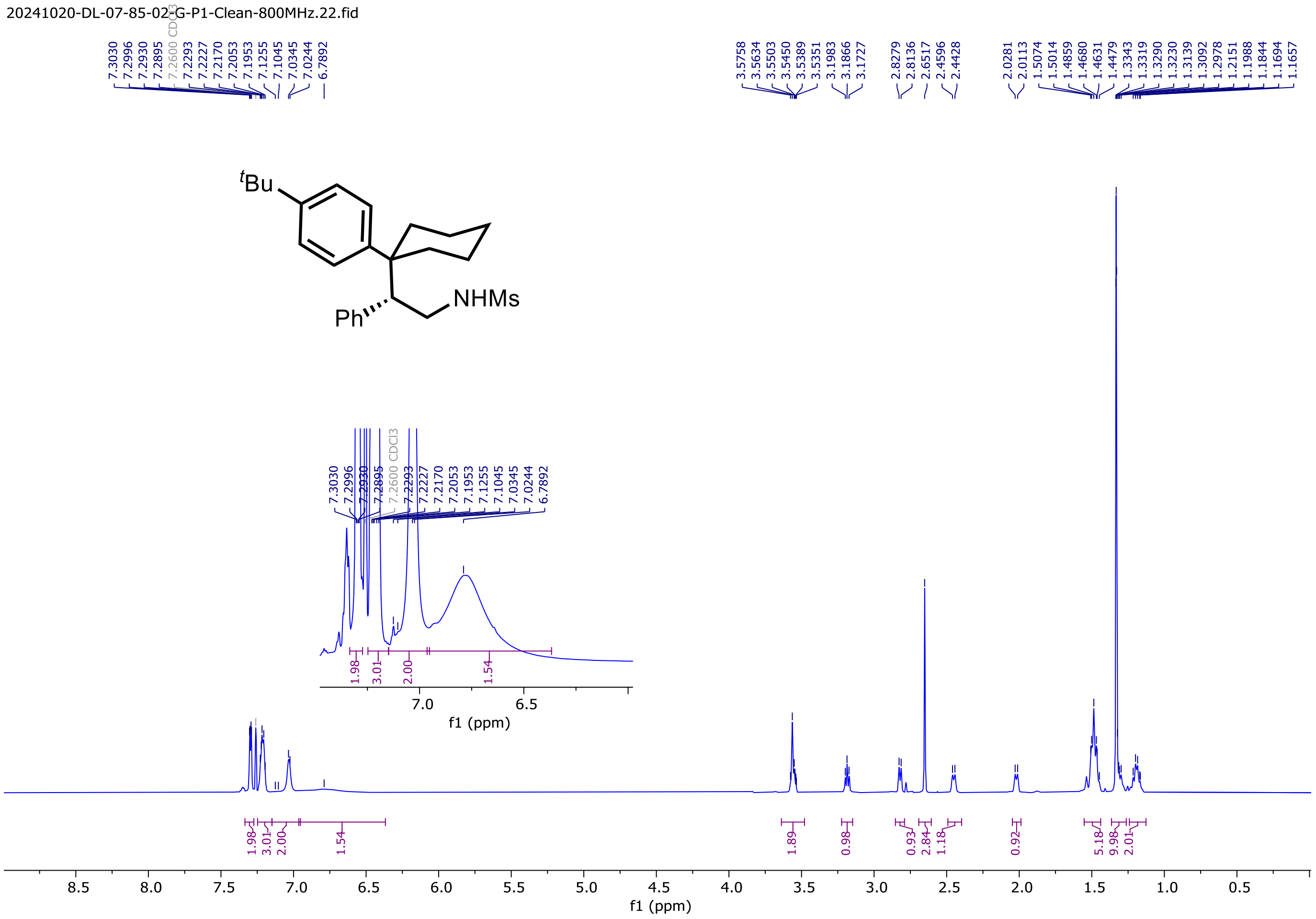

Figure S272. ^{1}**H-NMR of 42**

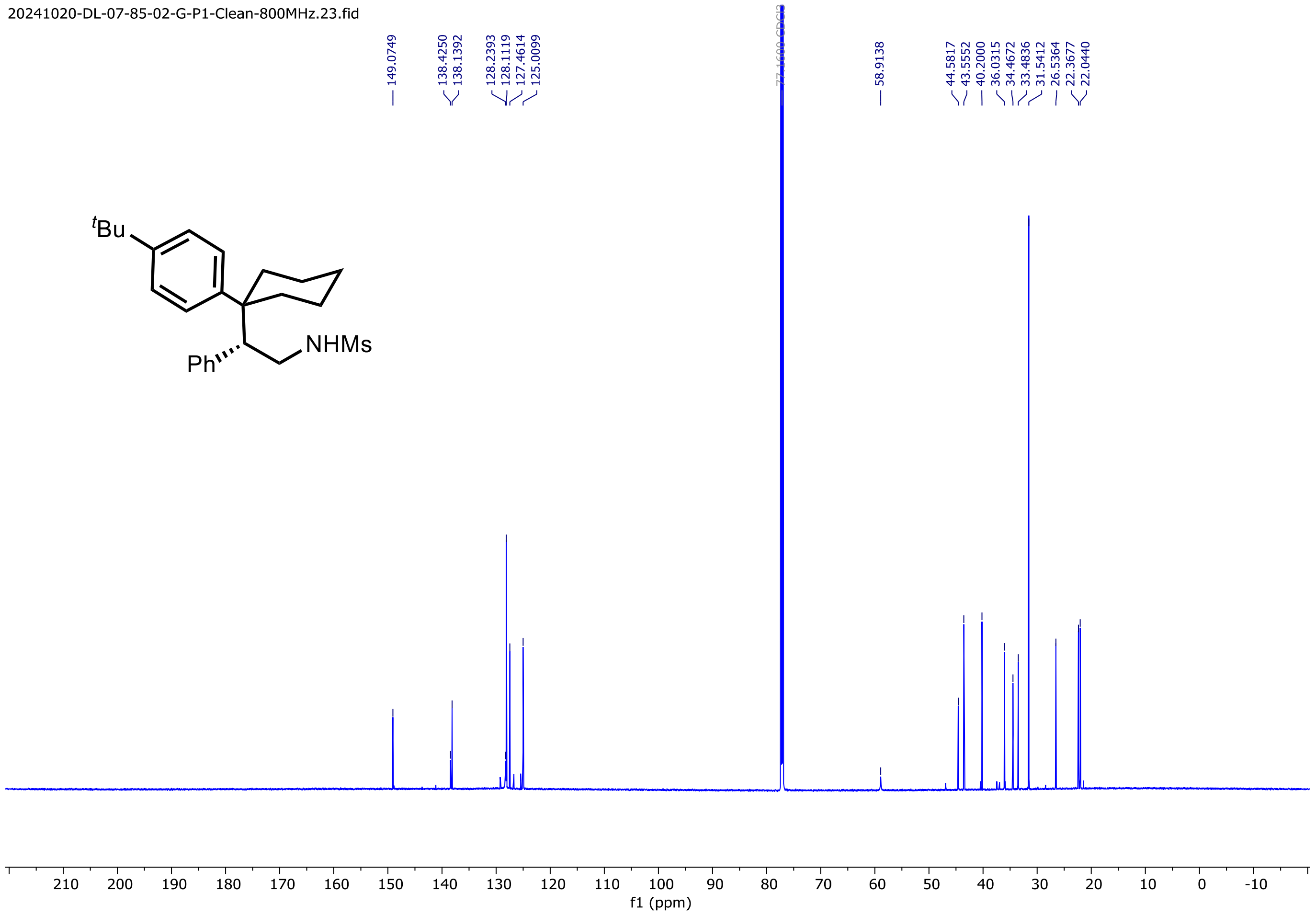

Figure S273. ^{13}**C-NMR of 42**

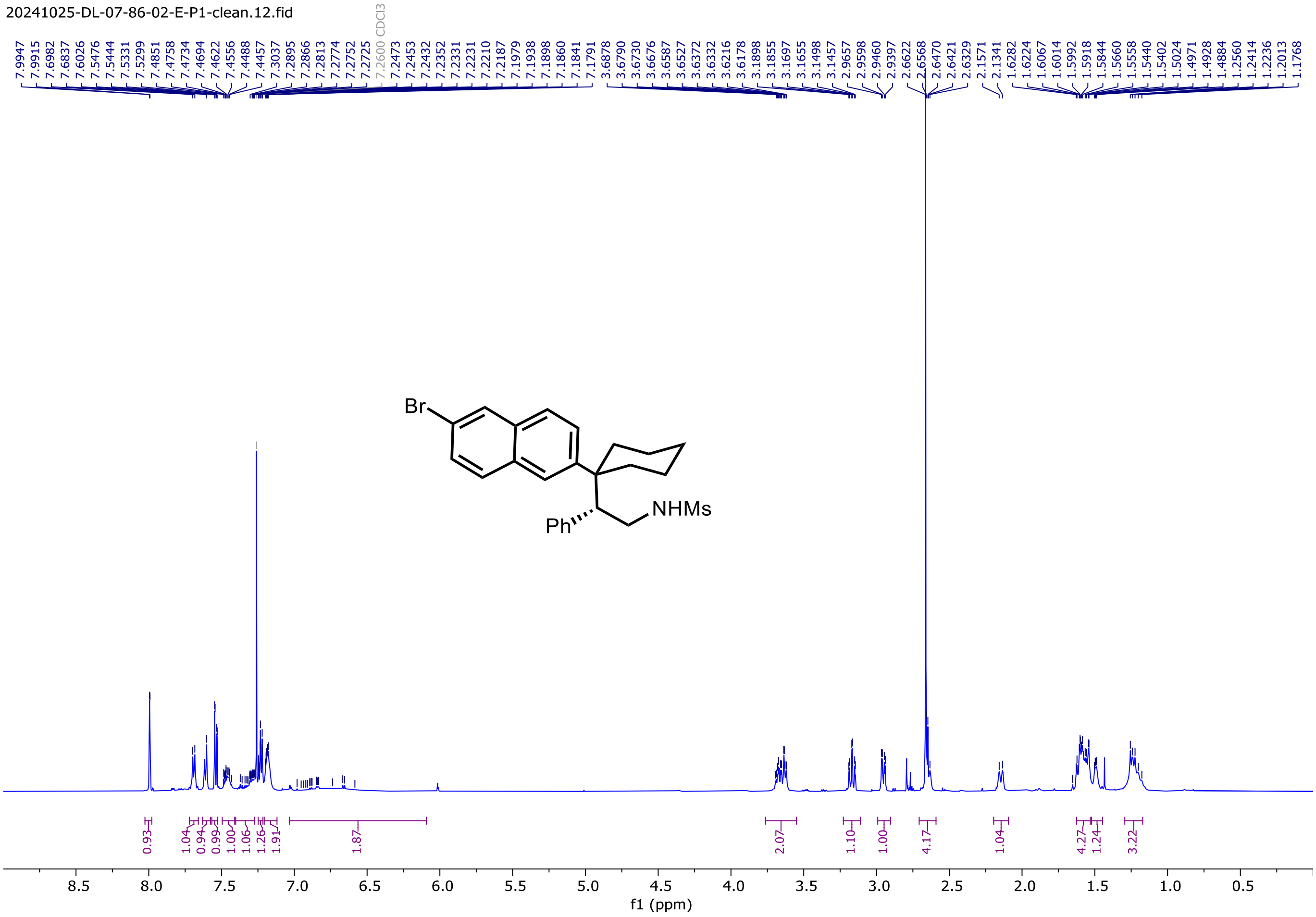

Figure S274. ^{1}H-NMR of 43

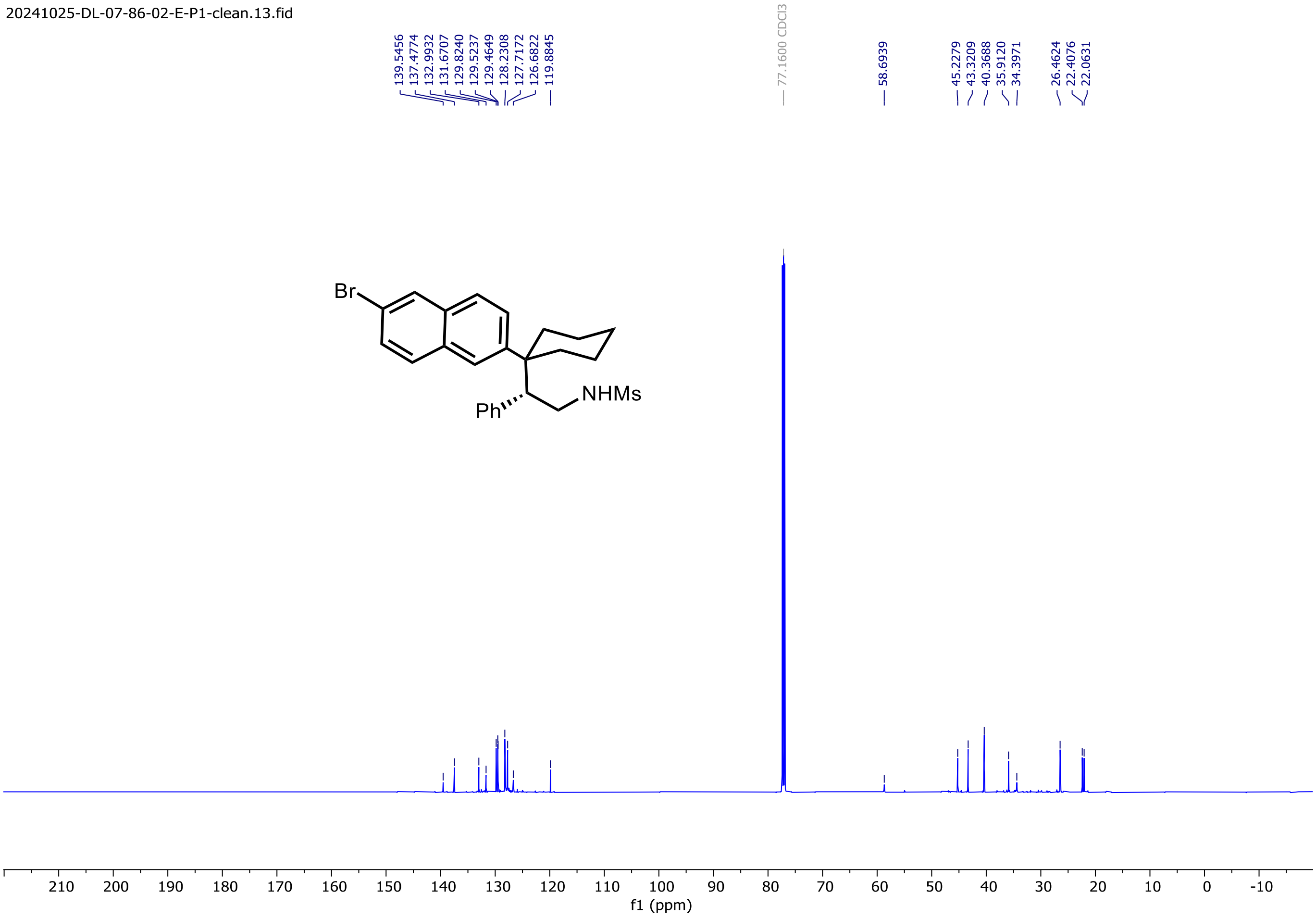

Figure S275. ^{13}C-NMR of 43

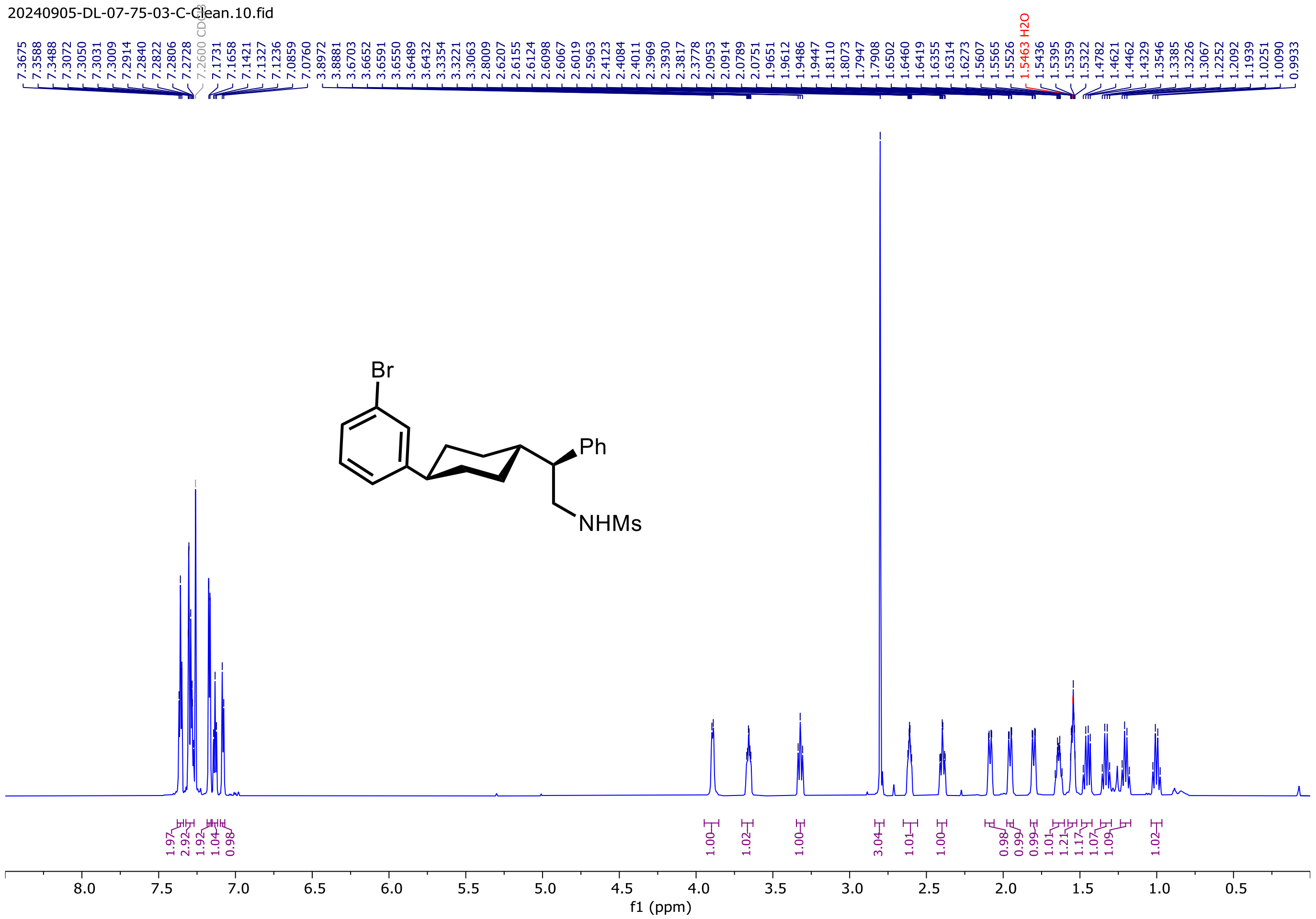

Figure S276. ^{1}H-NMR of 44

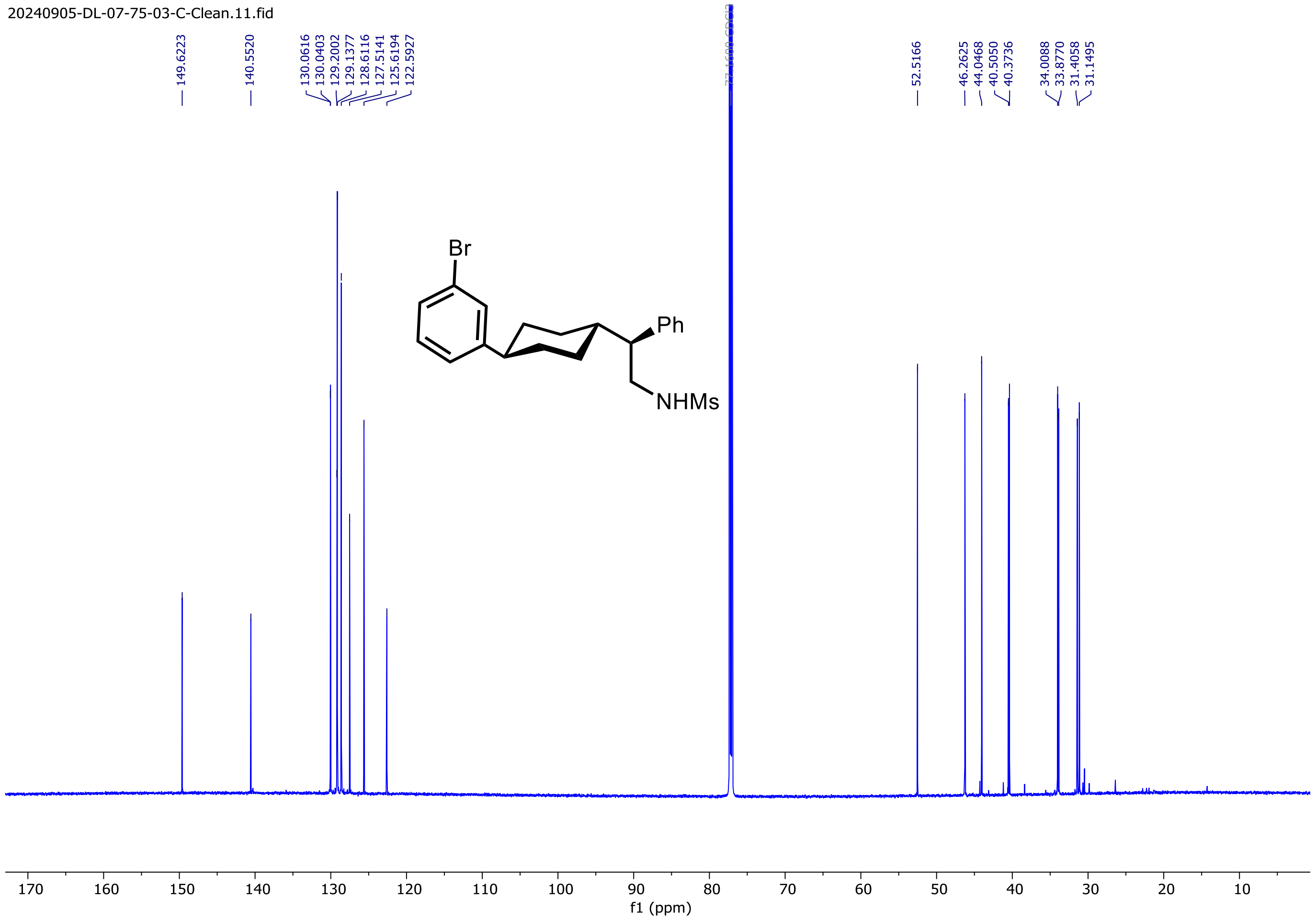

Figure S277. ^{13}C-NMR of 44

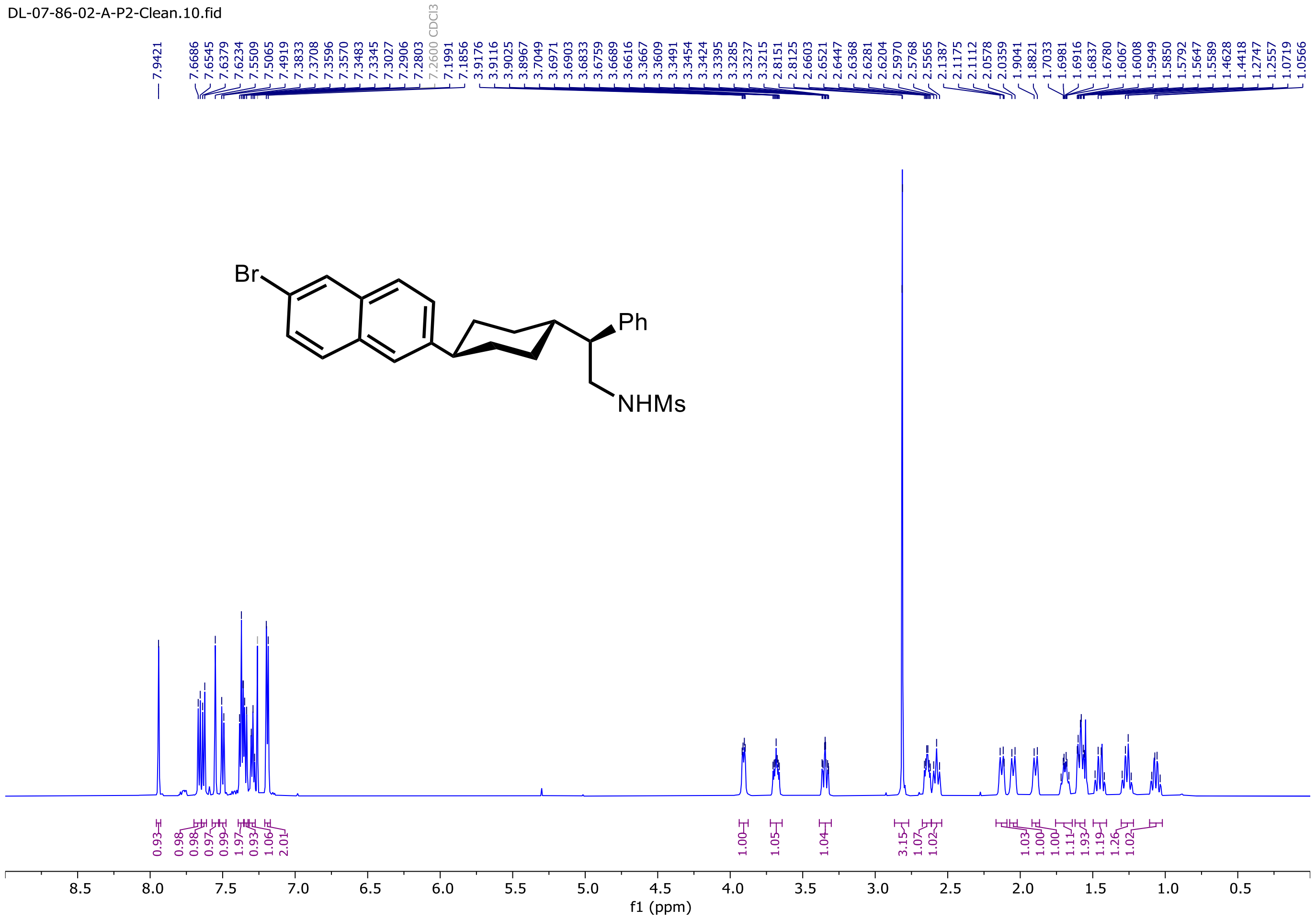

Figure S278. ^{1}H-NMR of 45

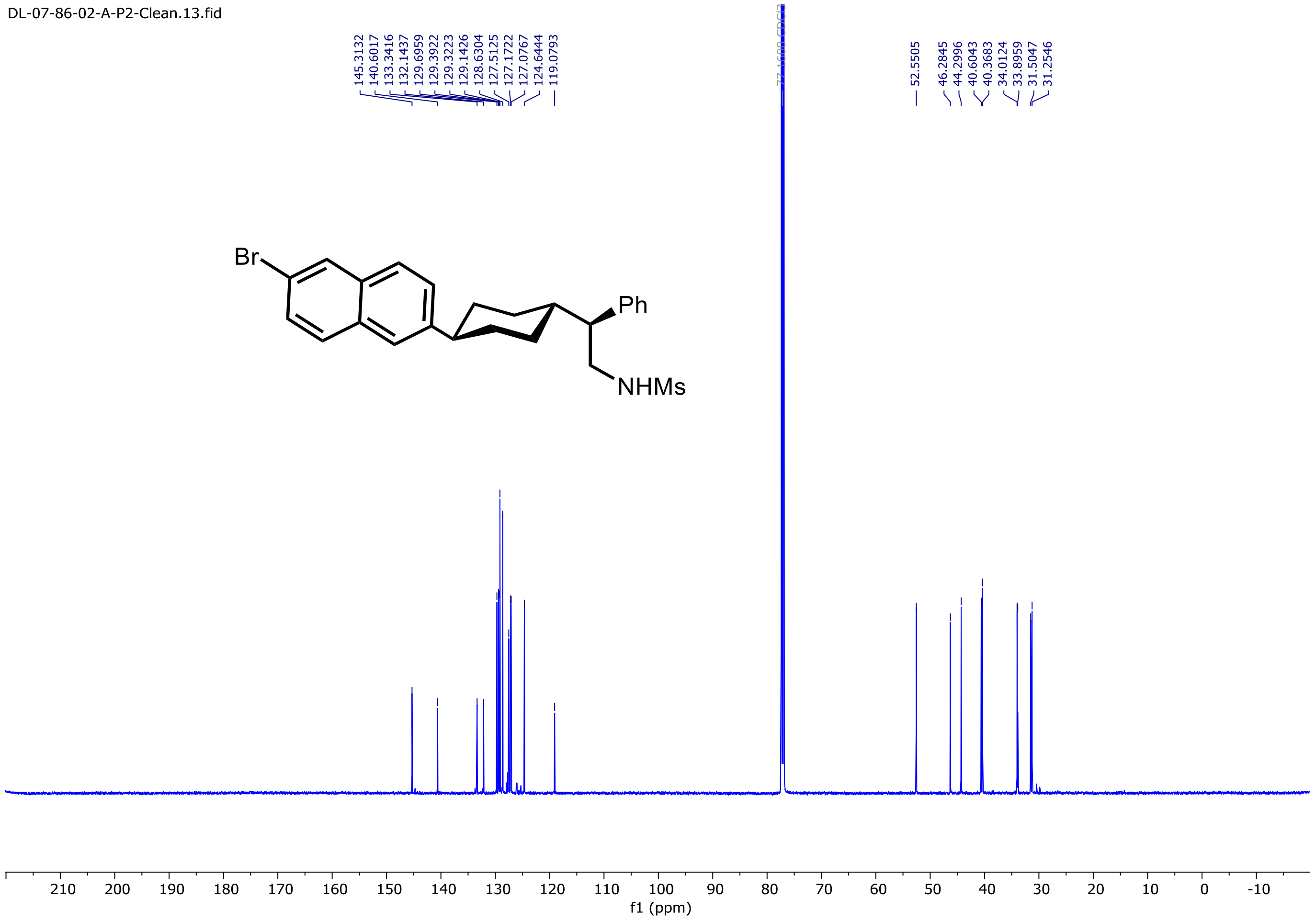

Figure S279. ^{13}C-NMR of 45

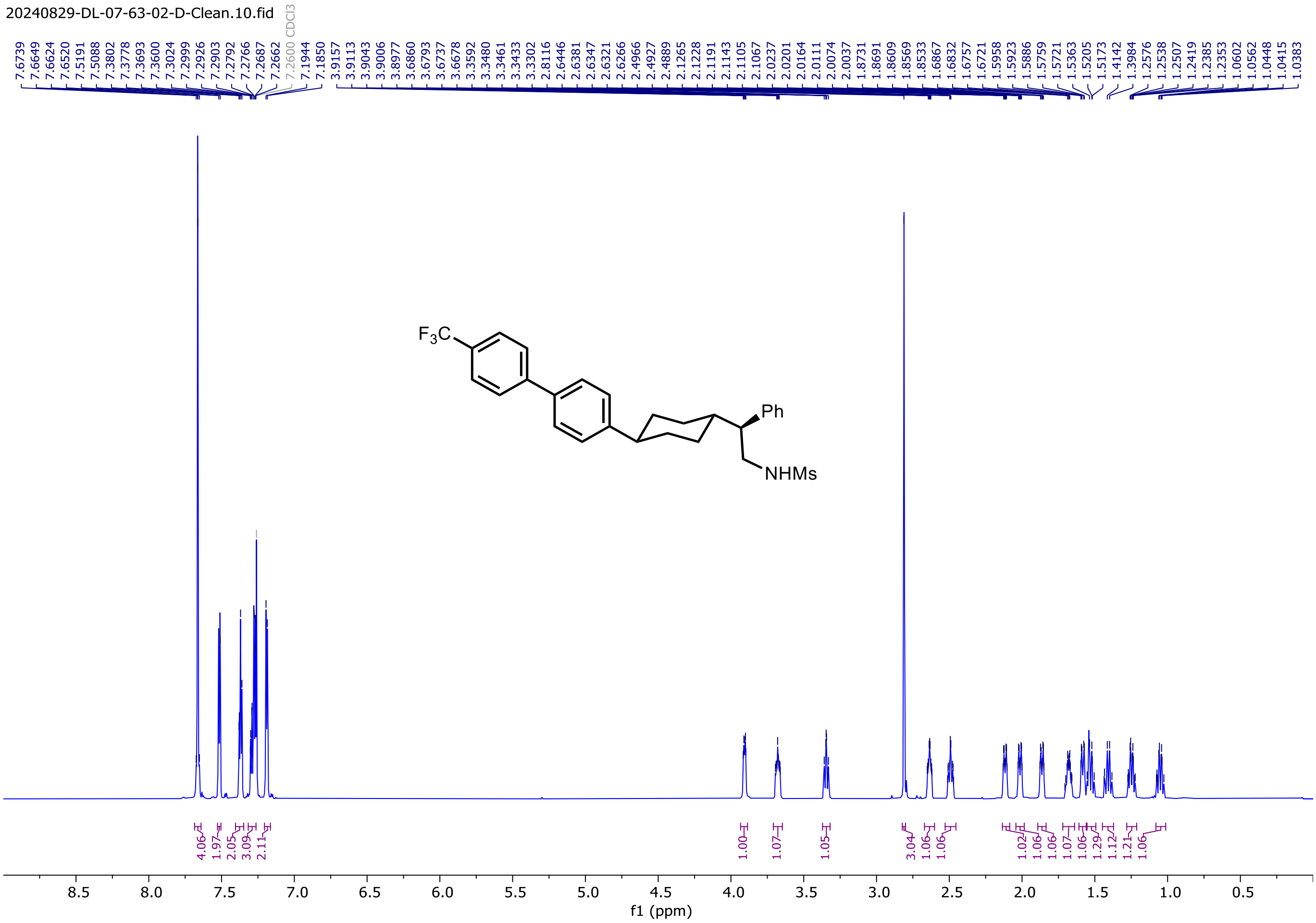

Figure S280. ^{1}H-NMR of 46

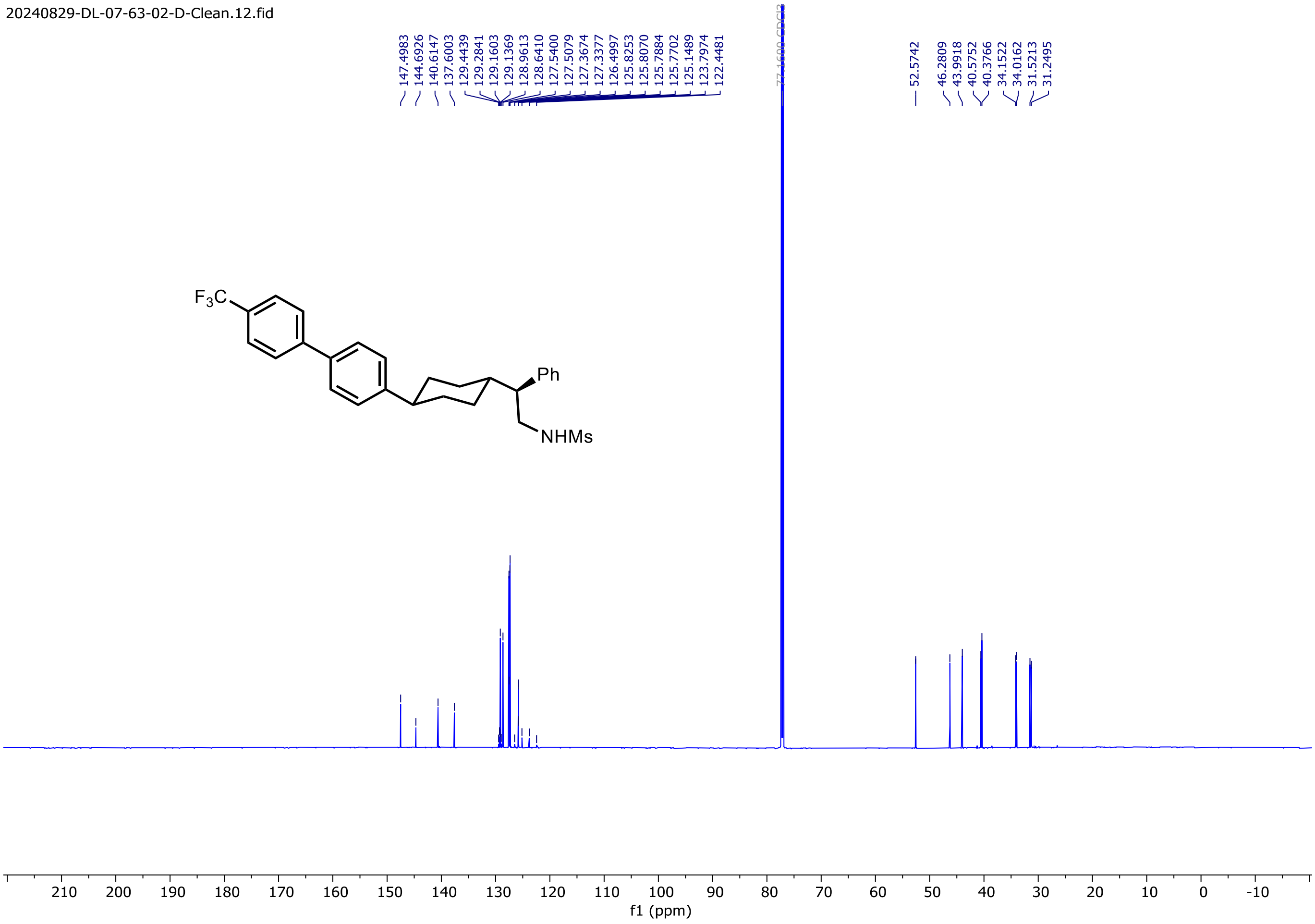

Figure S281. ^{13}C-NMR of 46

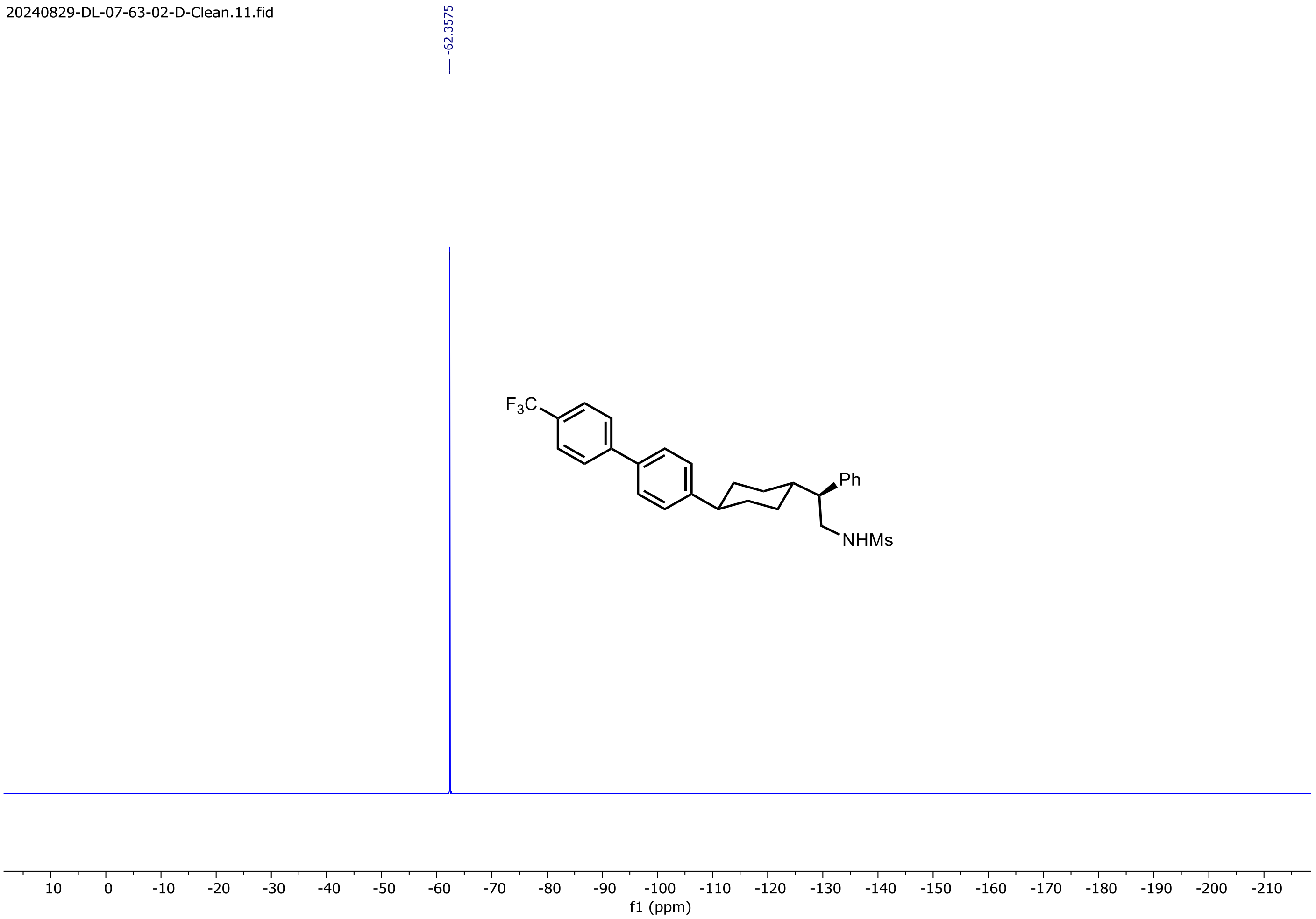

Figure S282. ^{19}F-NMR of 46

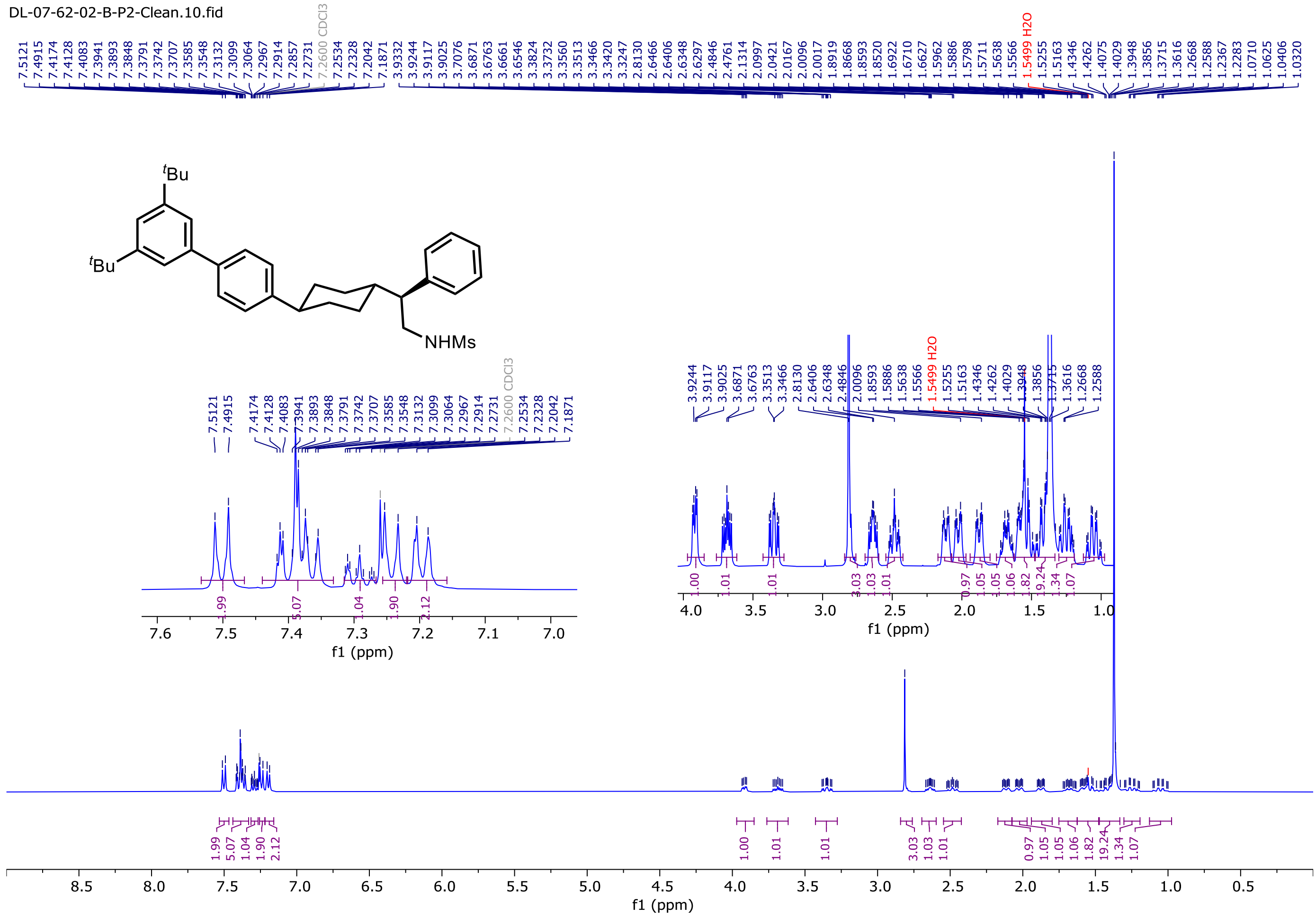

Figure S283. ^{1}**H-NMR of 47**

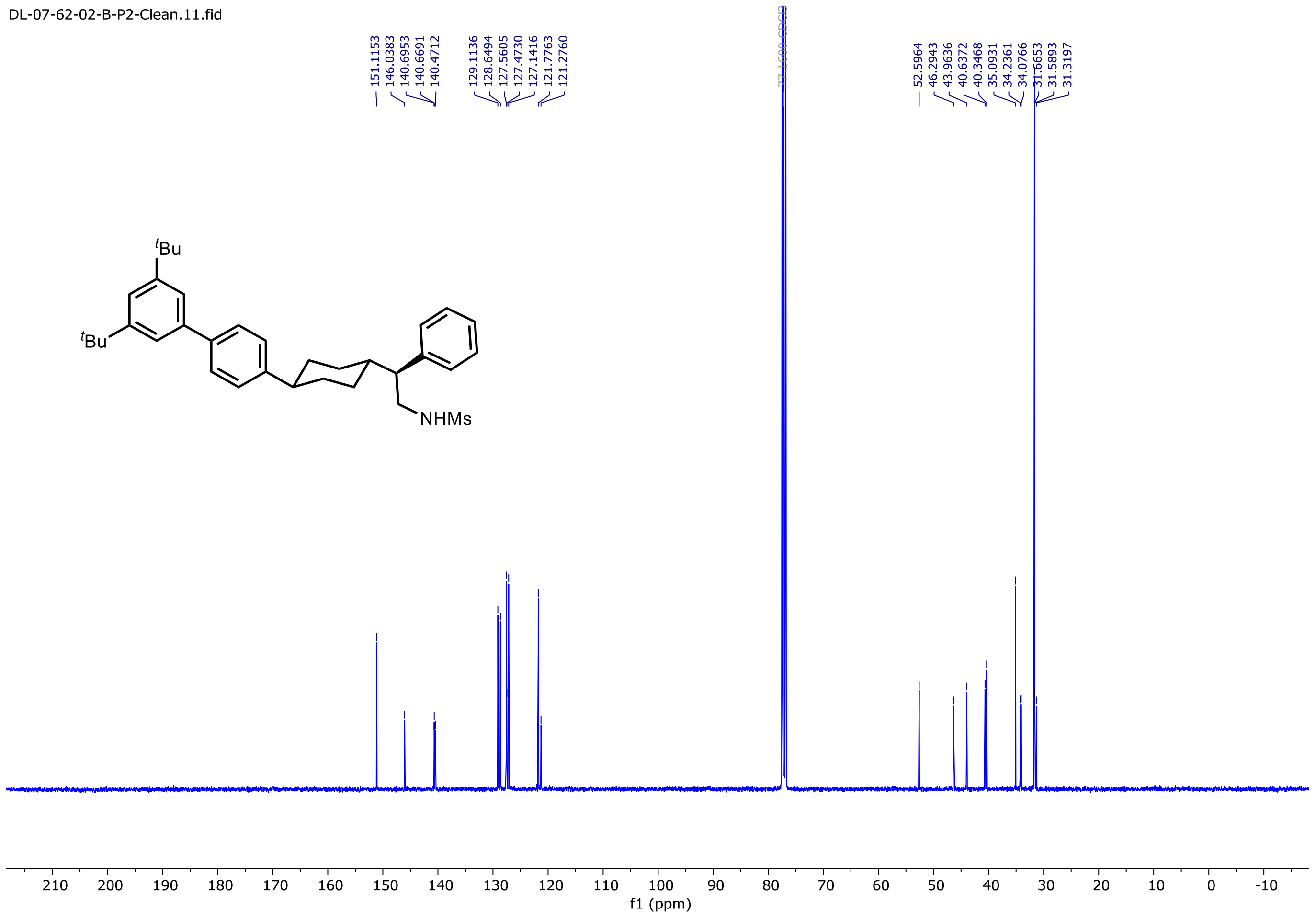

Figure S284. ^{13}**C-NMR of 47**

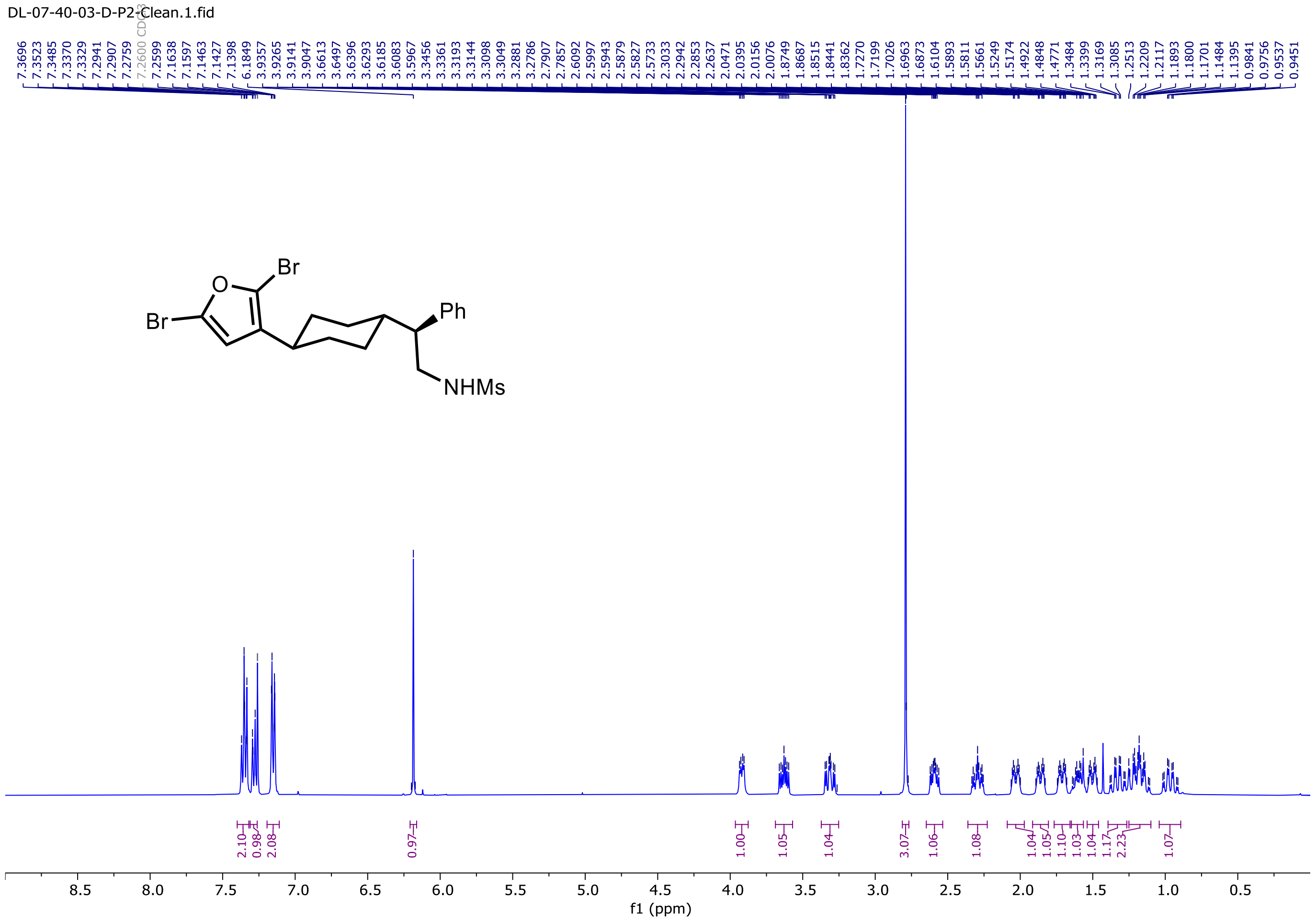

Figure S285. ^{1}H-NMR of 48

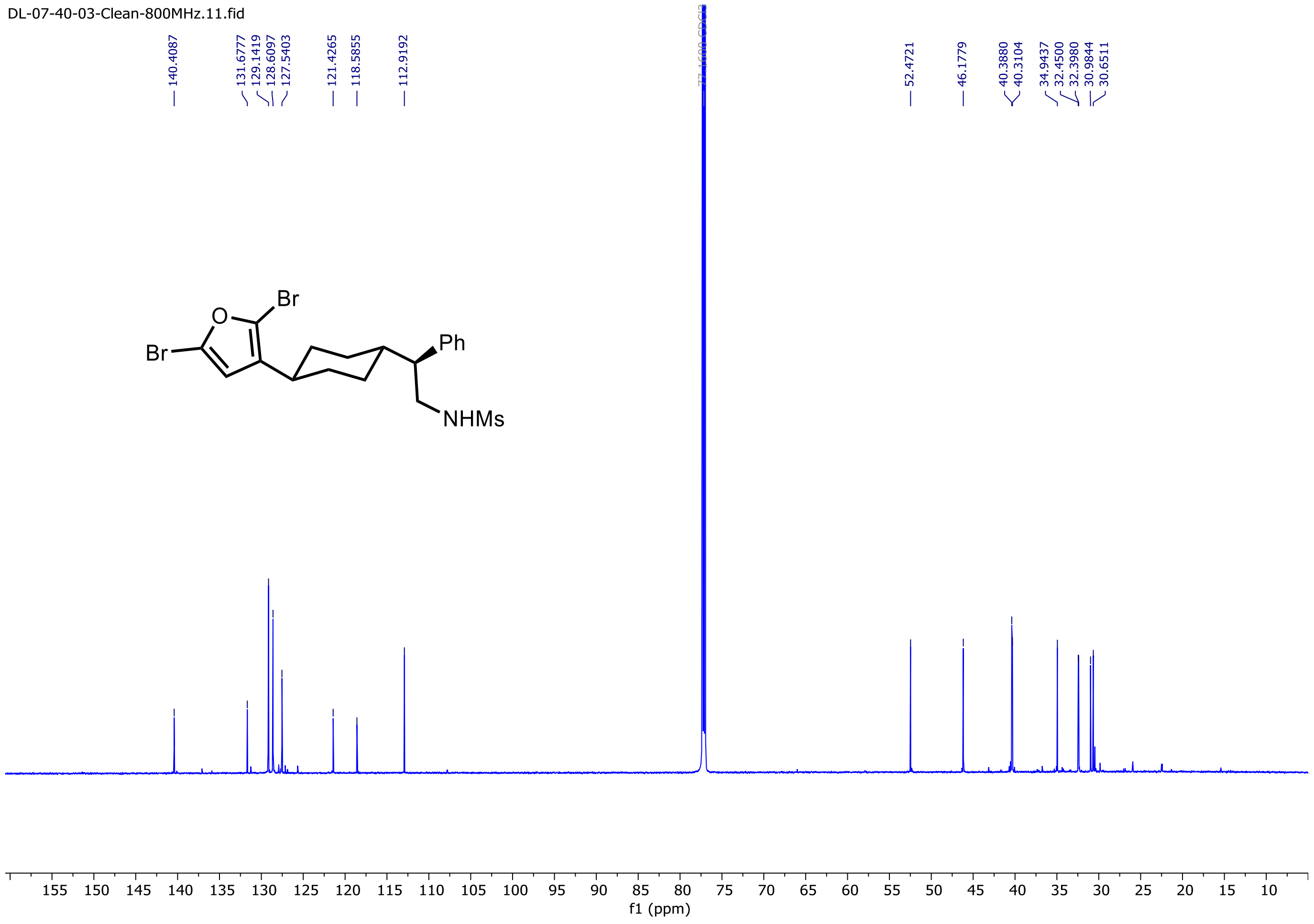

Figure S286. ^{13}C-NMR of 48

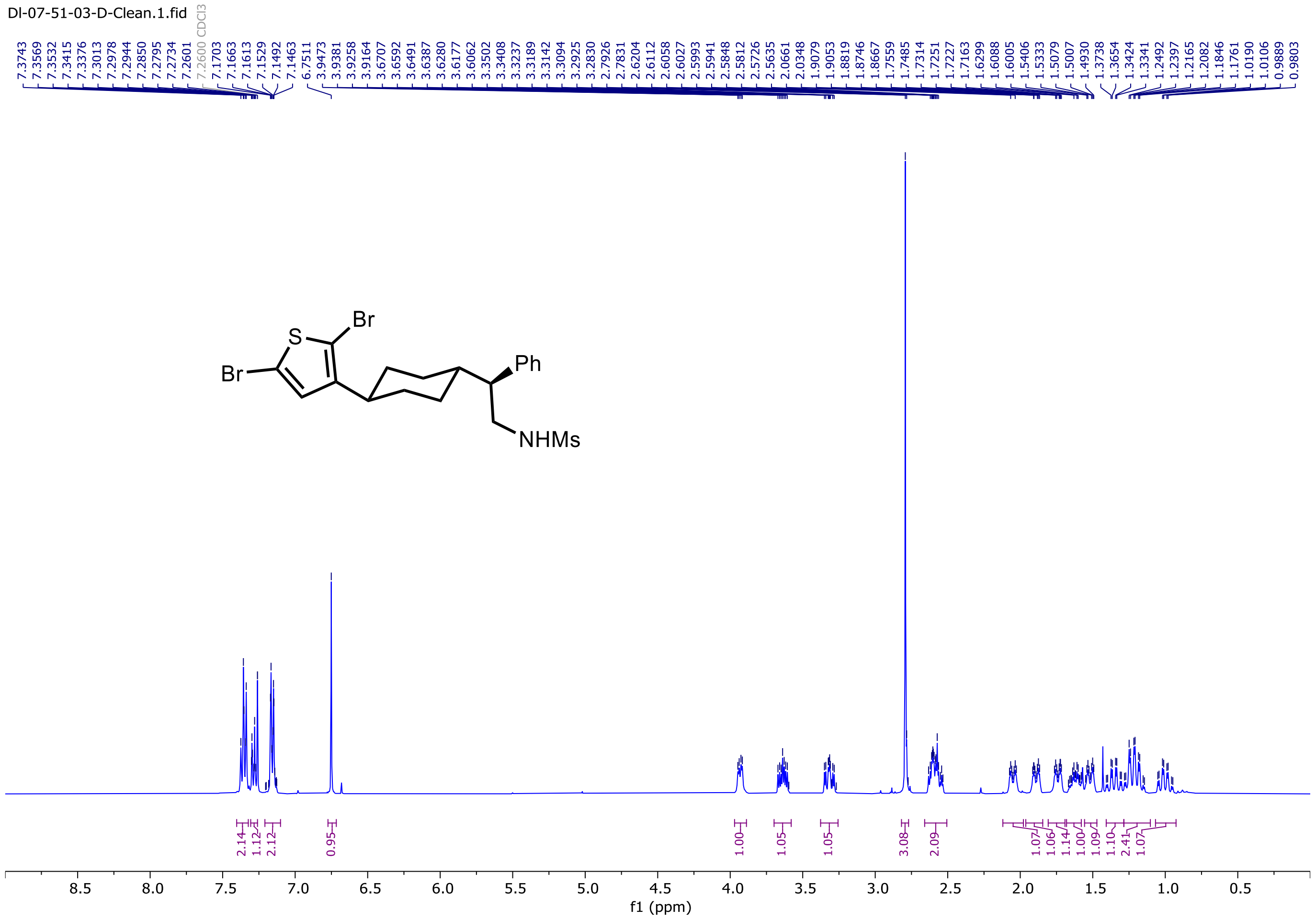

Figure S287. ^{1}H-NMR of 49

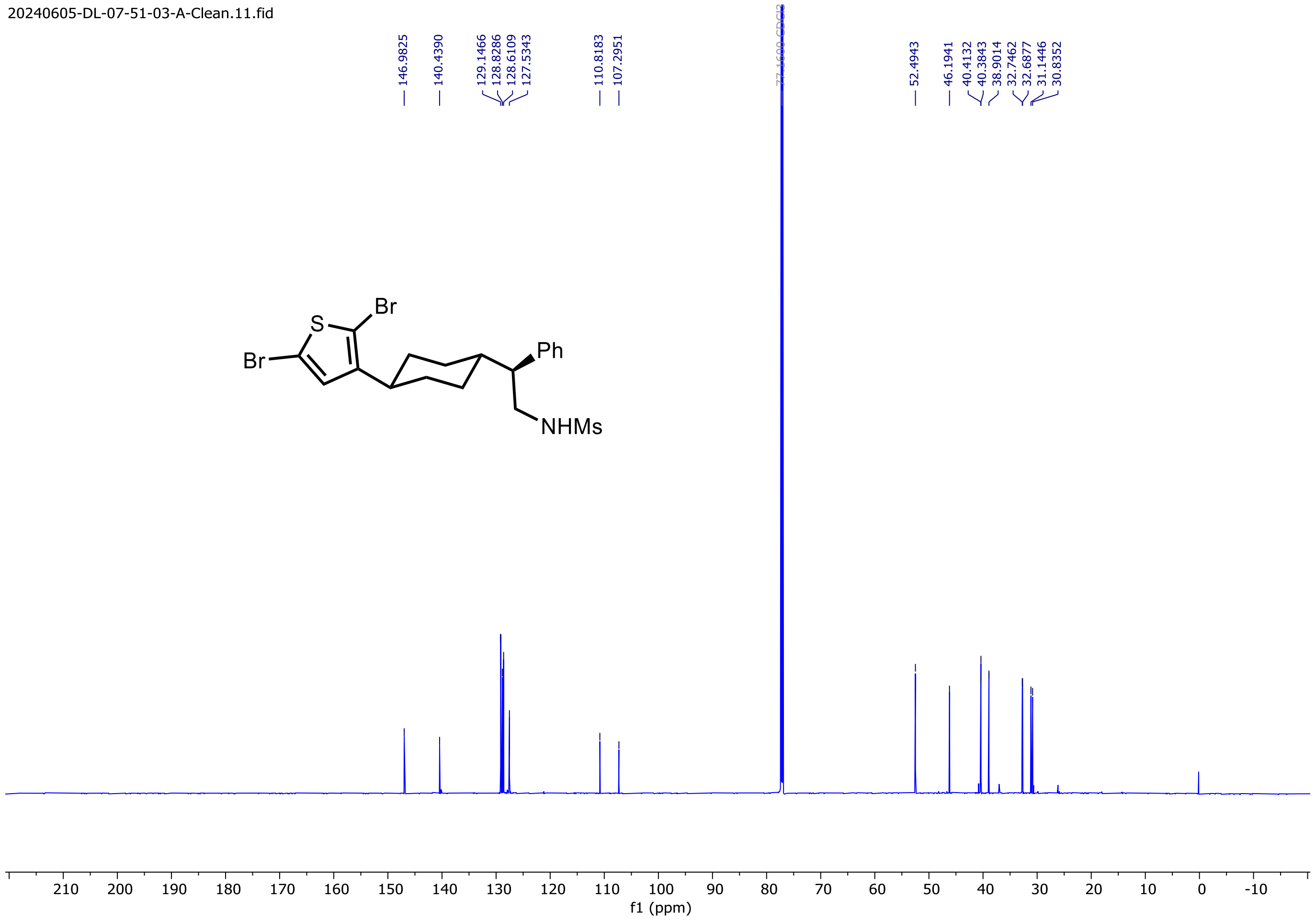

Figure S288. ^{13}C-NMR of 49

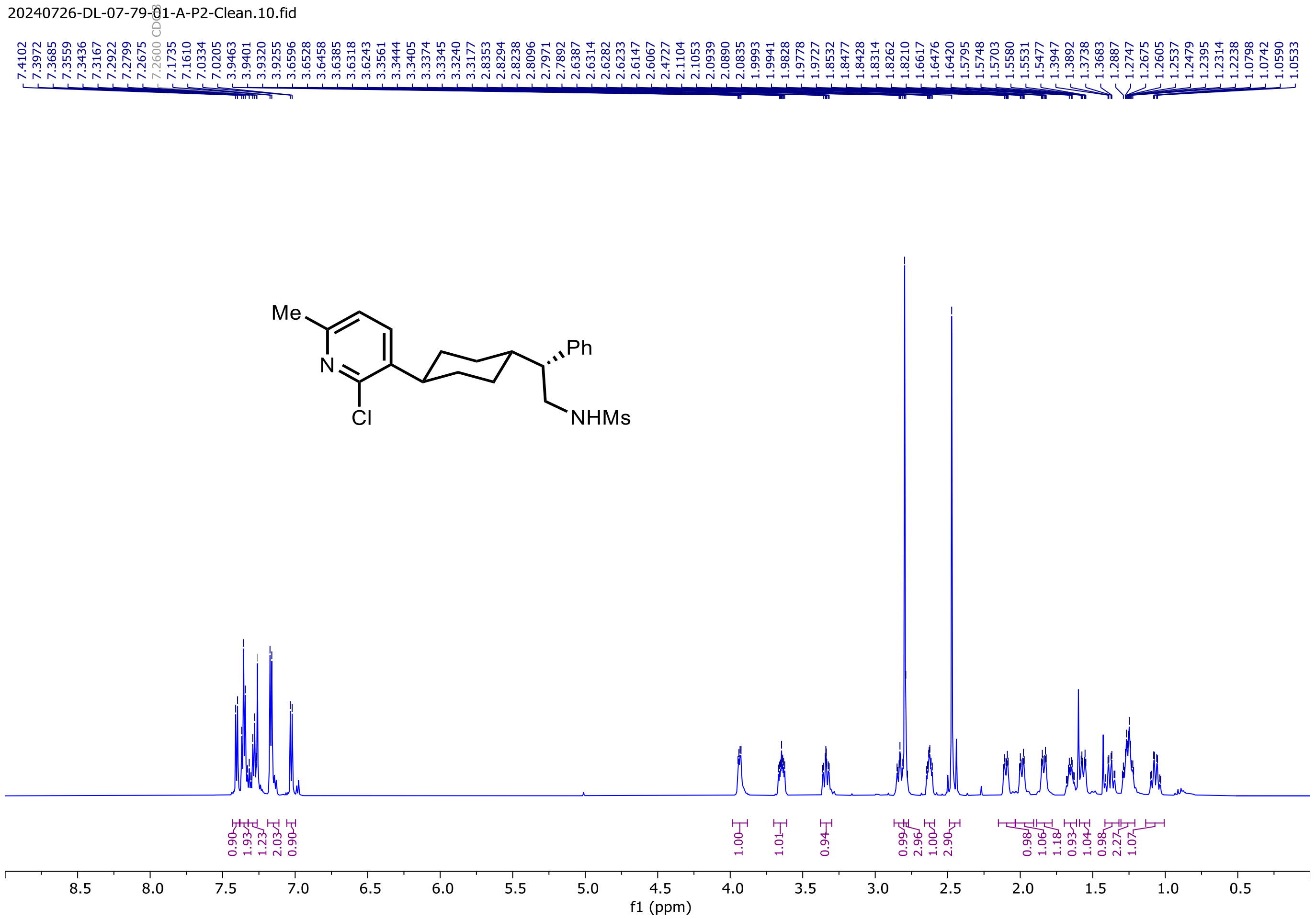

Figure S289. ^{1}**H-NMR of 50**

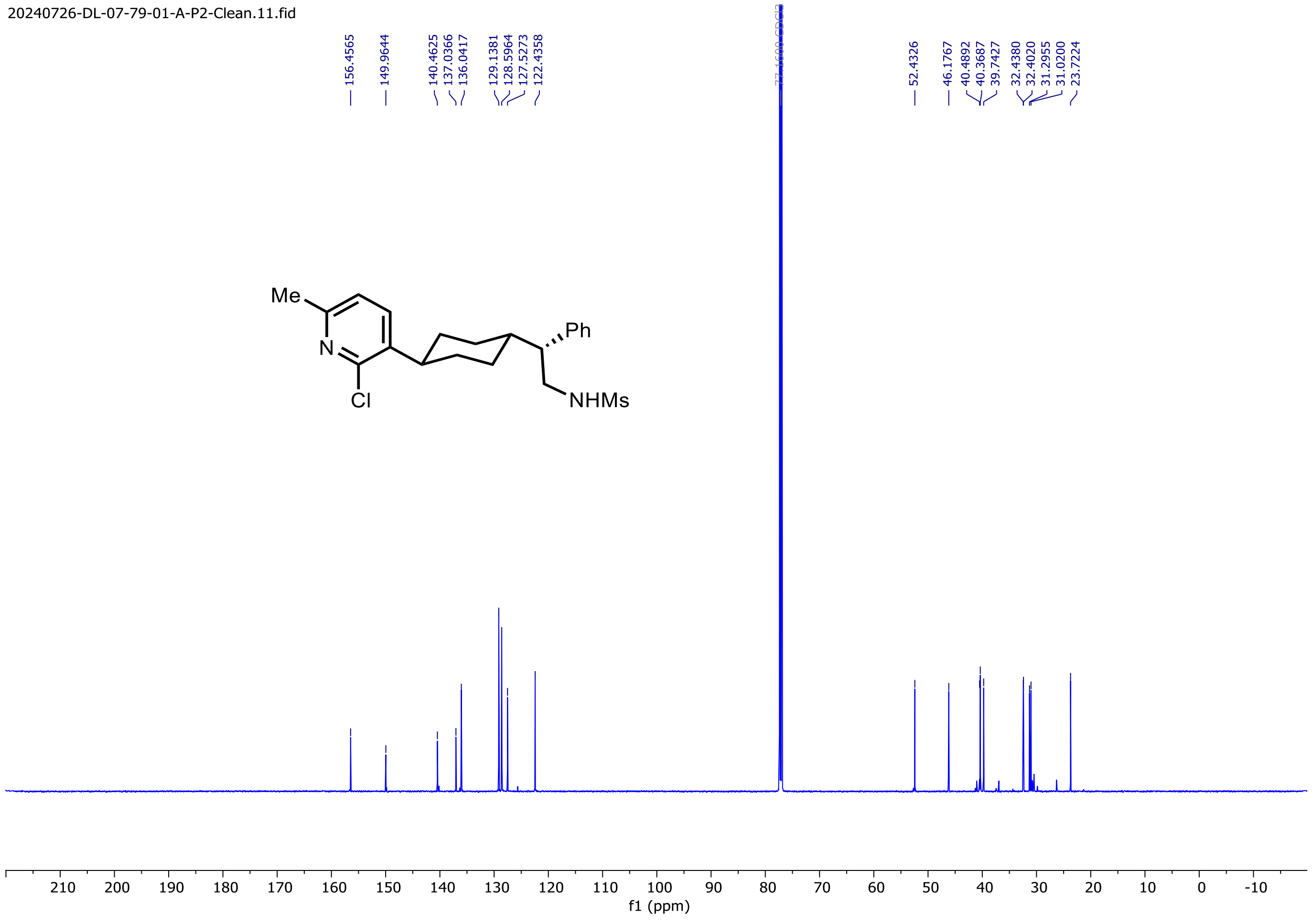

Figure S290. ^{13}**C-NMR of 50**

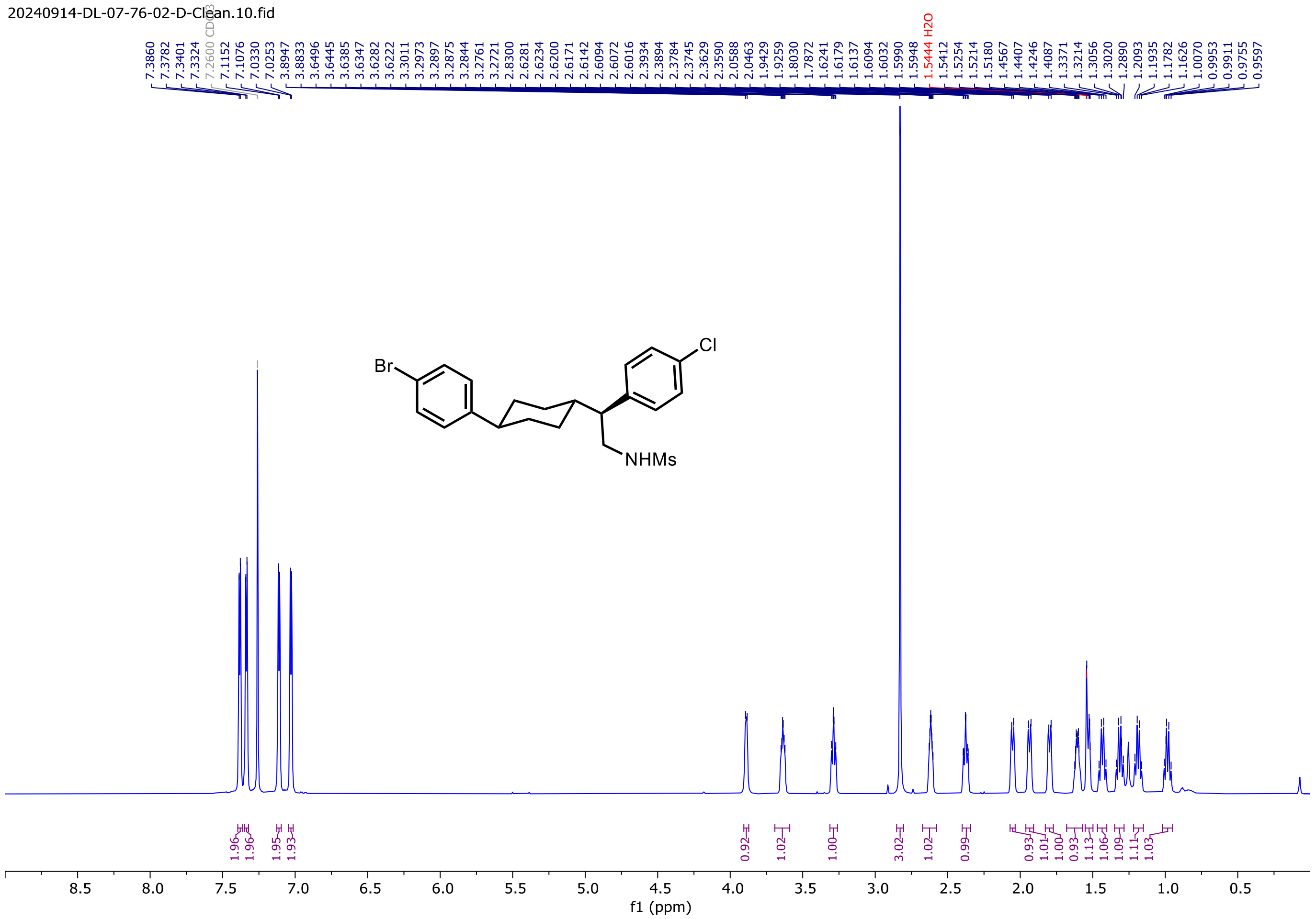

Figure S291. ^{1}**H-NMR of 51**

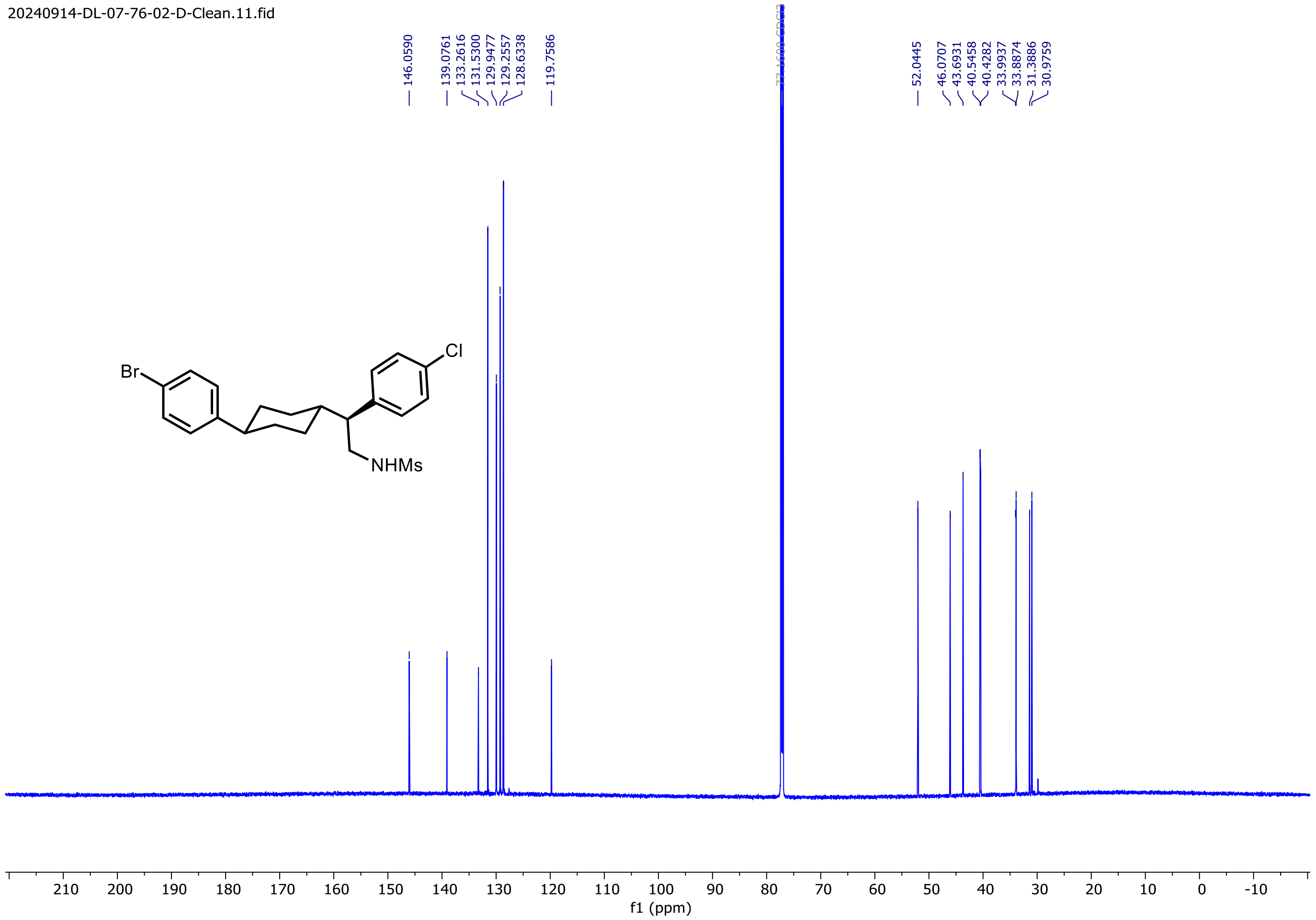

Figure S292. ^{13}**C-NMR of 51**

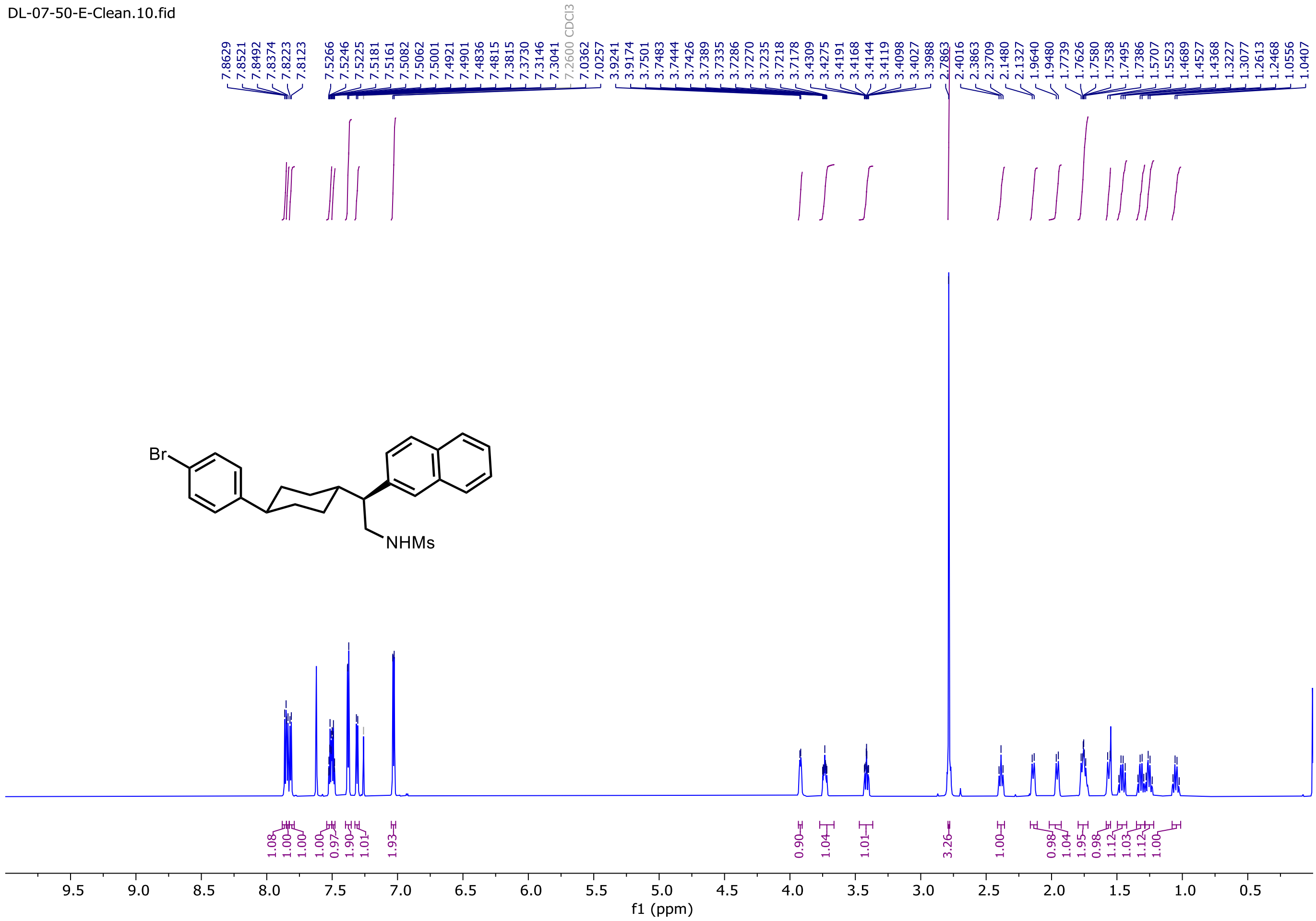

Figure S293. ^{1}H-NMR of 52

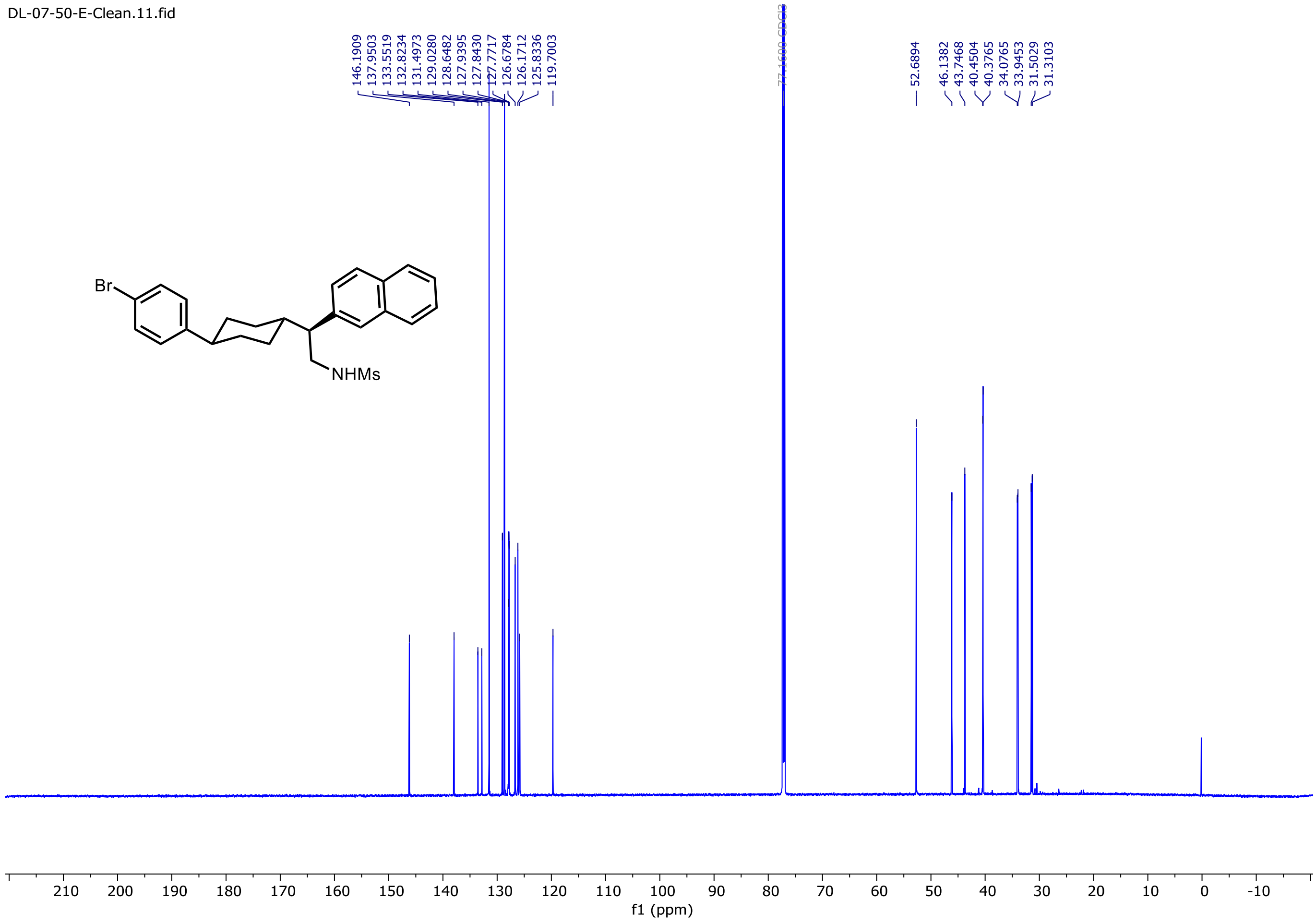

Figure S294. ^{13}C-NMR of 52

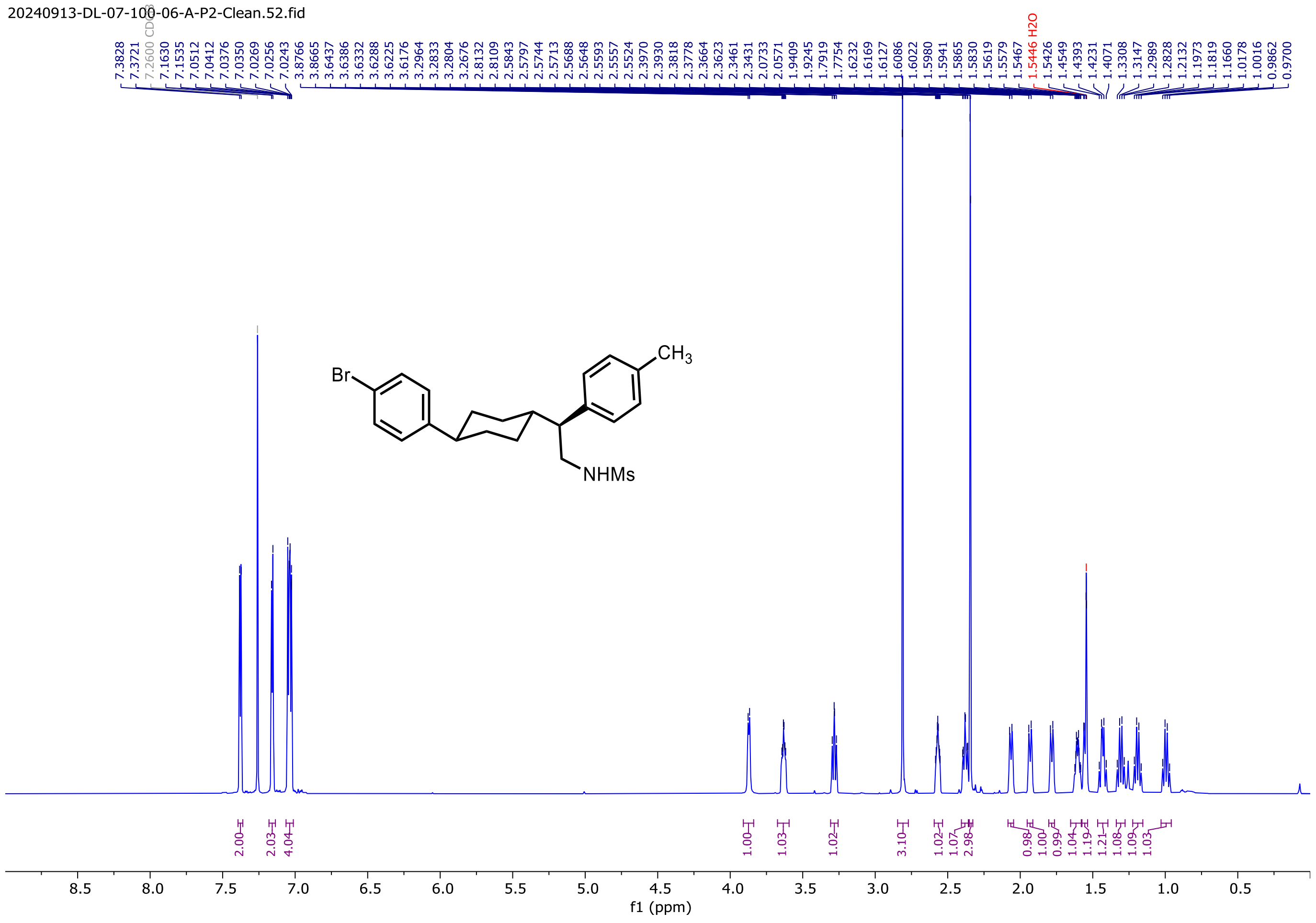

Figure S295. ¹H-NMR of 53

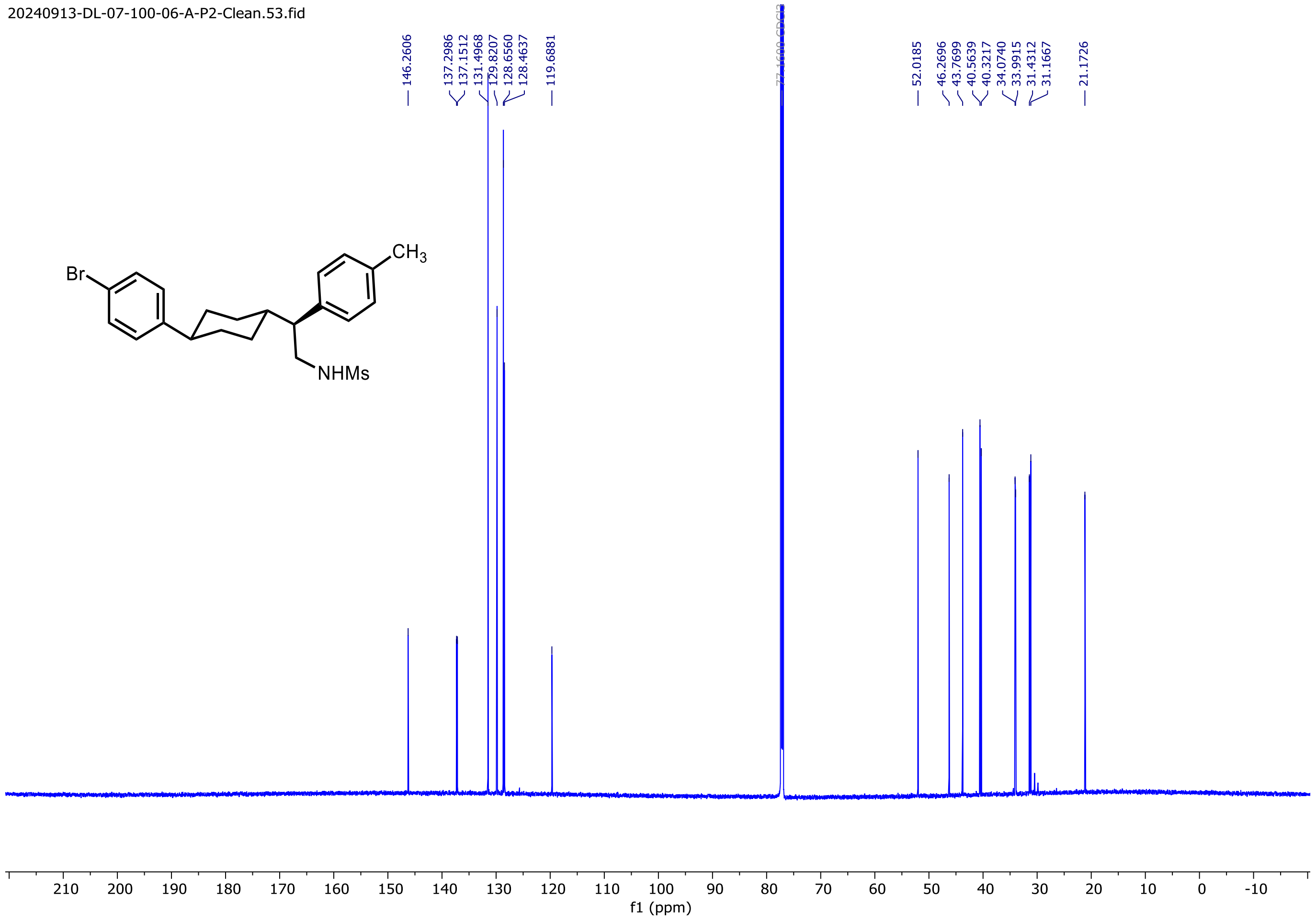

Figure S296. ¹³C-NMR of 53

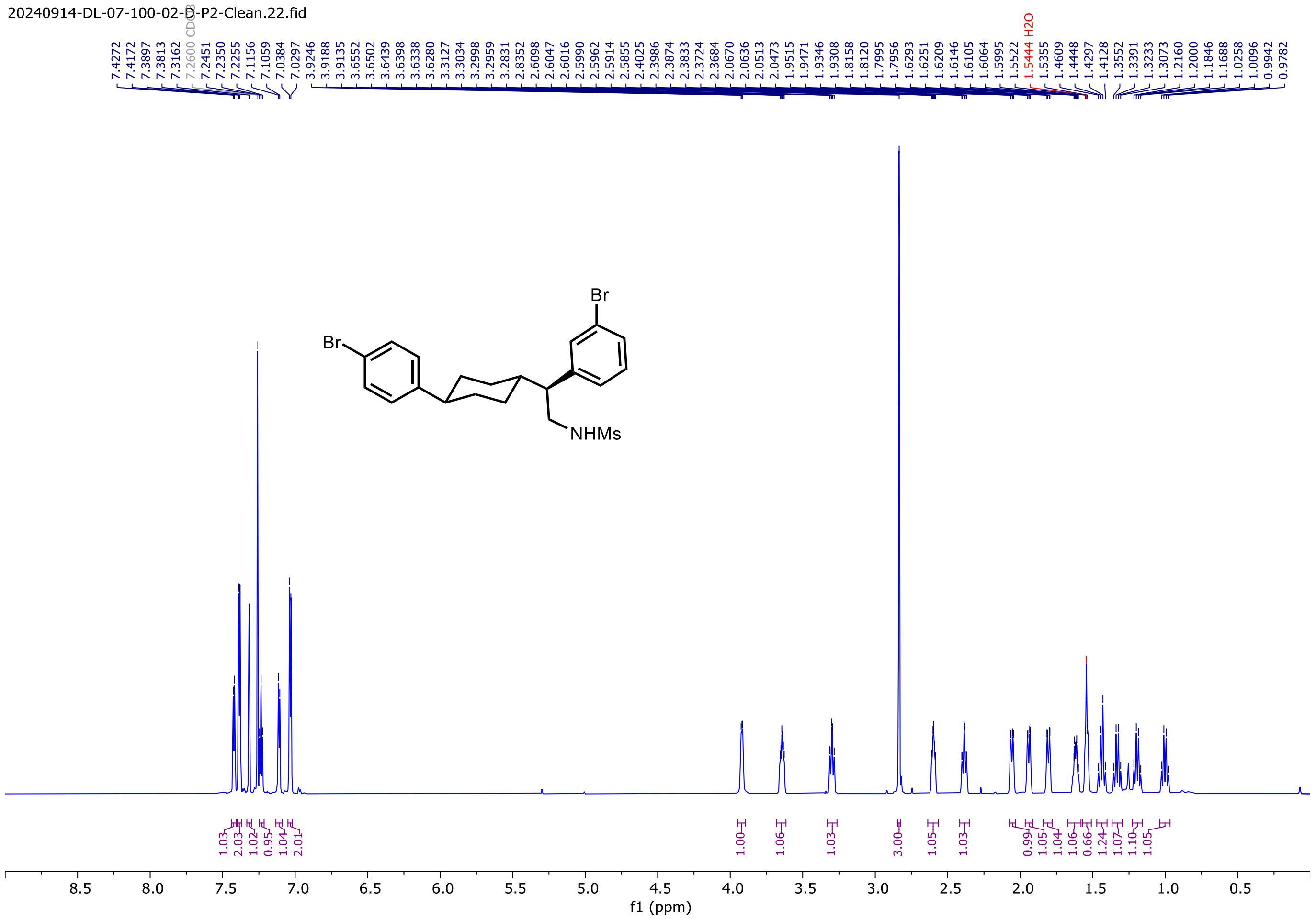

Figure S297. ^{1}H-NMR of 54

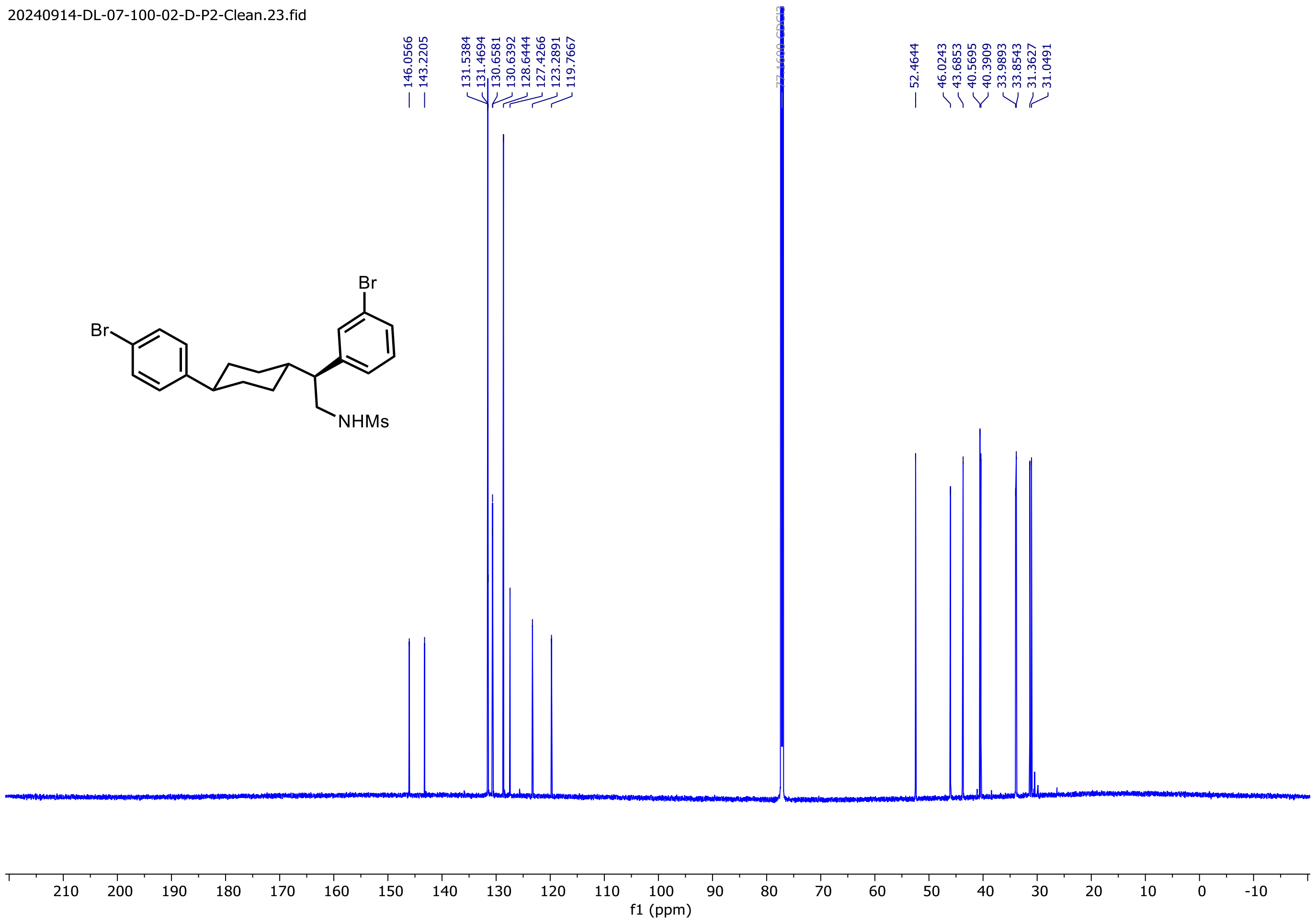

Figure S298. ^{13}C-NMR of 54

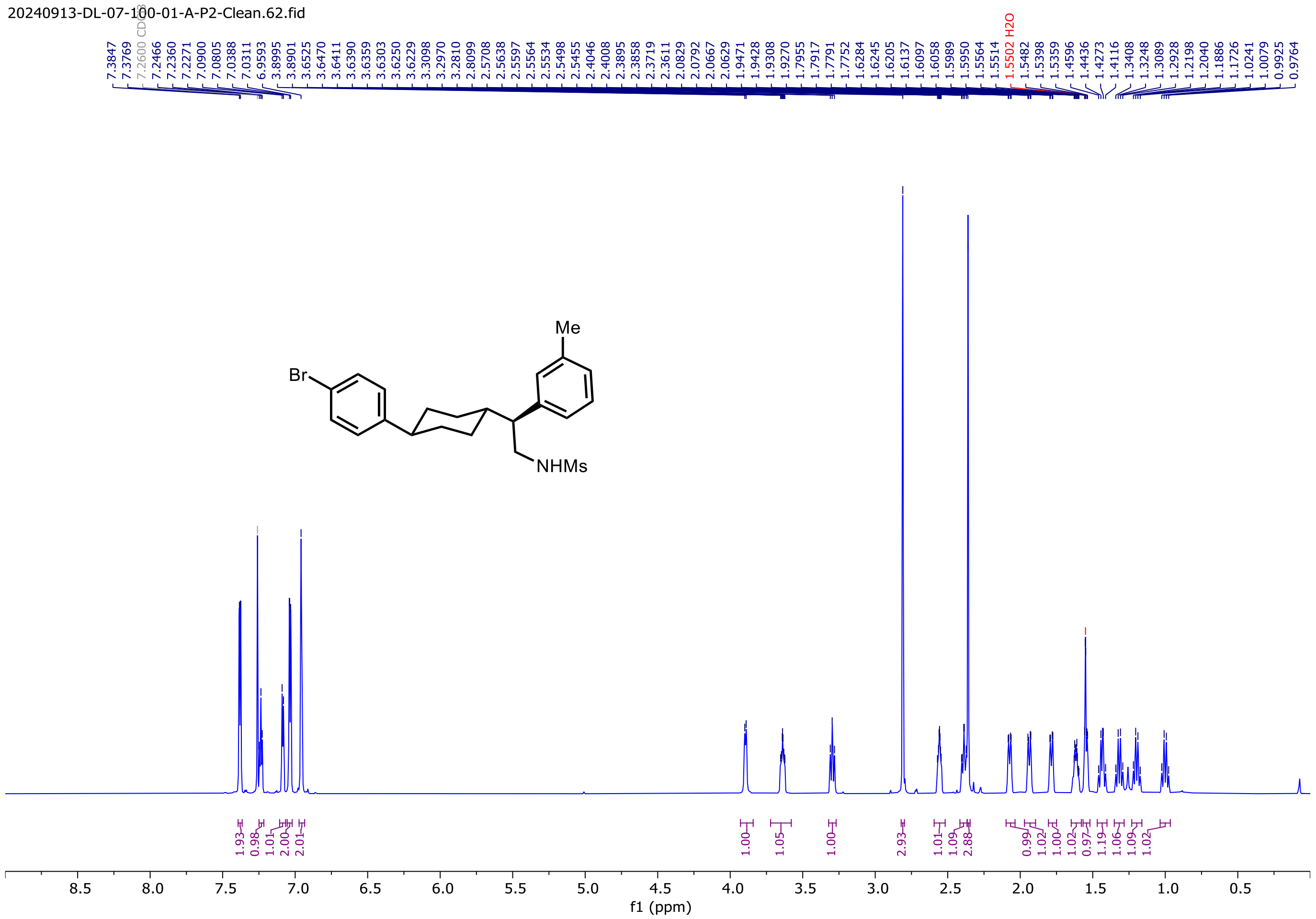

Figure S299. ^{1}H-NMR of 55

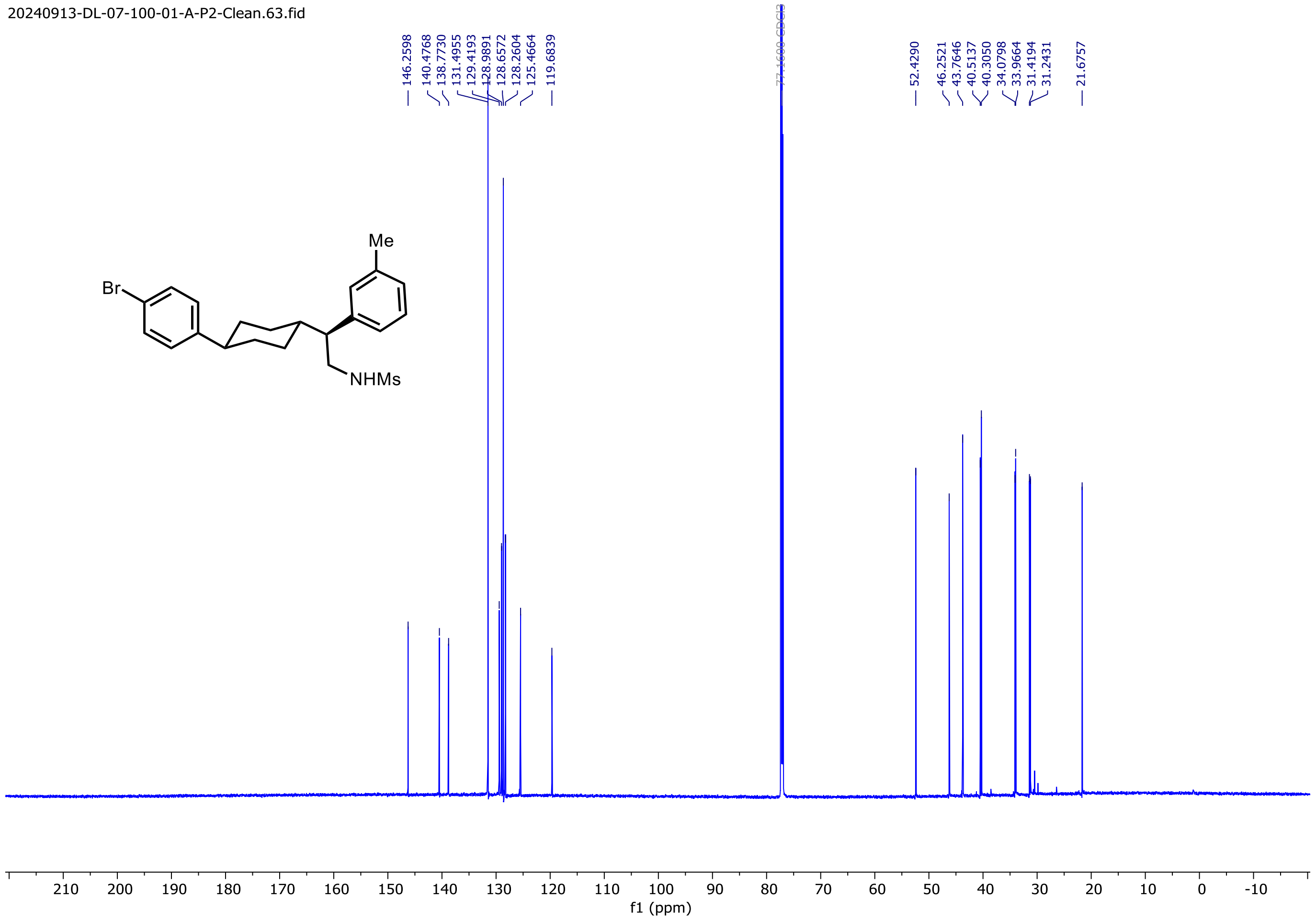

Figure S300. ^{13}C-NMR of 55

11. Crystallography study

Table S11. Summary of structure and CCDC number

Entry	Compound	CCDC
1	7a	2419298
2	7b	2419270
3	7c	2419549
4	8a	2419280
5	8b	2419272
6	8c	2419279
7	8d	2419960
8	10	2410264
9	13	2419540
10	13a	2419516
11	42	2410230

Compound 7a

Submitted by: **Yannick Boni**

Solved by: **John Bacsa**

R_1=5.58%

Crystal Data and Experimental

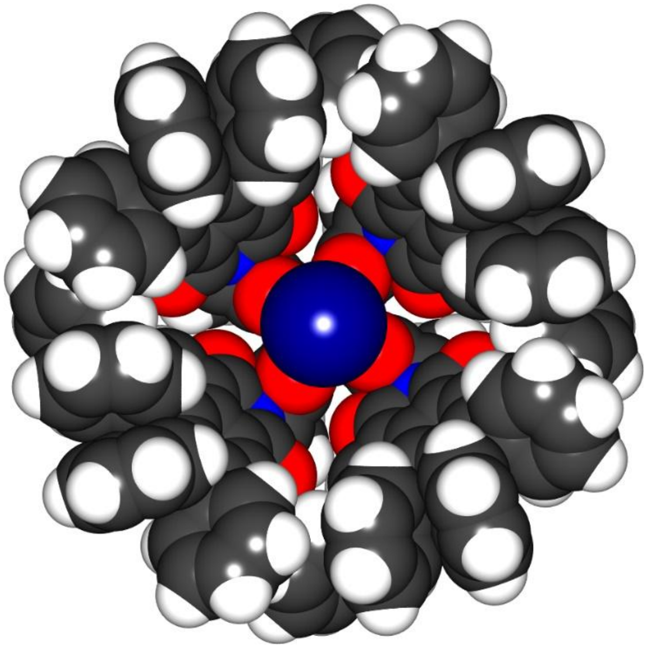

Experimental. Single green prism-shaped crystals of **Compound 7a** were chosen from the sample as supplied. A suiTable Scrystal with dimensions 0.34 × 0.22 × 0.18 mm^3 was selected and mounted on a XtaLAB Synergy-S diffractometer. The crystal was kept at a steady T = 103(4) K during data collection. The structure was solved with the ShelXT 2018/2 (Sheldrick, 2018) solution program using dual methods and by using Olex2 1.5-alpha (Dolomanov et al., 2009) as the graphical interface. The model was refined with ShelXL 2019/3 (Sheldrick, 2015) using full matrix least squares minimisation on $\boldsymbol{F}^2$.

Crystal Data. $C_{206}H_{167}N_5O_{16}Rh_2$, M_r = 3174.26, tetragonal, $P42_12$ (No. 90), a = 26.04010(10) Å, b = 26.04010(10) Å, c = 12.98300(10) Å, $\alpha = \beta = \gamma$ = 90°, V = 8803.60(10) Å^3, T = 103(4) K, Z = 2, Z' = 0.25, μ(Cu K$_\alpha$) = 2.021, 100815 reflections measured, 8248 unique (R_{int} = 0.0491) which were used in all calculations. The final wR_2 was 0.1653 (all data) and R_1 was 0.0558 (I≥2 σ(I)).

**Compound**	**7a**
Formula	$C_{206}H_{167}N_5O_{16}Rh_2$
$D_{calc.}$/ g cm^{-3}	1.197
μ/mm^{-1}	2.021
Formula Weight	3174.26
Colour	green
Shape	prism-shaped
Size/mm^3	0.34×0.22×0.18
T/K	103(4)
Crystal System	tetragonal
Flack Parameter	0.045(13)
Space Group	$P42_12$
a/Å	26.04010(10)
b/Å	26.04010(10)
c/Å	12.98300(10)
α/°	90
β/°	90
γ/°	90
V/Å^3	8803.60(10)
Z	2
Z'	0.25
Wavelength/Å	1.54184
Radiation type	Cu K$_\alpha$
Θ_{min}/°	3.404
Θ_{max}/°	72.914
Measured Refl's.	100815
Indep't Refl's	8248
Refl's I≥2 σ(I)	7402
R_{int}	0.0491
Parameters	448
Restraints	242
Largest Peak	2.192
Deepest Hole	-0.494
GooF	0.995
wR_2 (all data)	0.1653
wR_2	0.1601
R_1 (all data)	0.0599
R_1	0.0558

Structure Quality Indicators

**Reflections:**	d min (CuKα) 2Θ=145.8° 0.81	I/σ(I) 55.9	Rint m=12.24 4.91%	Full 135.4° 95% to 145.8° 98.6
**Refinement:**	Shift 0.001	Max Peak 2.2	Min Peak -0.5	GooF 0.995

A green prism-shaped-shaped crystal with dimensions 0.34 × 0.22 × 0.18 mm^3 was mounted. Data were collected using a XtaLAB Synergy, Dualflex, HyPix diffractometer operating at T = 103(4) K.

Data were measured using ω scans with Cu K_α radiation. The diffraction pattern was indexed and the total number of runs and images was based on the strategy calculation from the program CrysAlisPro 1.171.40.84a (Rigaku OD, 2020). The maximum resolution that was achieved was Θ = 72.914° (0.83 Å).

The unit cell was refined using CrysAlisPro 1.171.40.84a (Rigaku OD, 2020) on 46628 reflections, 46% of the observed reflections.

Data reduction, scaling and absorption corrections were performed using CrysAlisPro 1.171.40.84a (Rigaku OD, 2020). The final completeness is 98.60 % out to 72.914° in Θ. A numerical absorption correction based on gaussian integration over a multifaceted crystal model was performed using CrysAlisPro 1.171.41.108a (Rigaku Oxford Diffraction, 2021). An empirical absorption correction using spherical harmonics, implemented in SCALE3 ABSPACK scaling algorithm was also applied. The absorption coefficient μ of this material is 2.021 mm^{-1} at this wavelength (λ = 1.54184Å) and the minimum and maximum transmissions are 0.351 and 1.000.

The structure was solved and the space group $P42_12$ (# 90) determined by the ShelXT 2018/2 (Sheldrick, 2018) structure solution program using dual methods and refined by full matrix least squares minimisation on $\boldsymbol{F}^2$ using version 2018/3 of ShelXL 2019/3 (Sheldrick, 2015). All non-hydrogen atoms were refined anisotropically. Hydrogen atom positions were calculated geometrically and refined using the riding model. Hydrogen atom positions were calculated geometrically and refined using the riding model.

_refine_special_details: Refined as a 2-component inversion twin.

The value of Z' is 0.25. The moiety formula is C170 H131 N5 O16 Rh2, 4[C6], 4[H6].

The Flack parameter was refined to 0.045(13). Determination of absolute structure using Bayesian statistics on Bijvoet differences using the Olex2 results in None. The chiral atoms in this structure are: C14(S). Note: The Flack parameter is used to determine chirality of the crystal studied, the value should be near 0, a value of 1 means that the stereochemistry is wrong and the model should be inverted. A value of 0.5 means that the crystal consists of a racemic mixture of the two enantiomers.

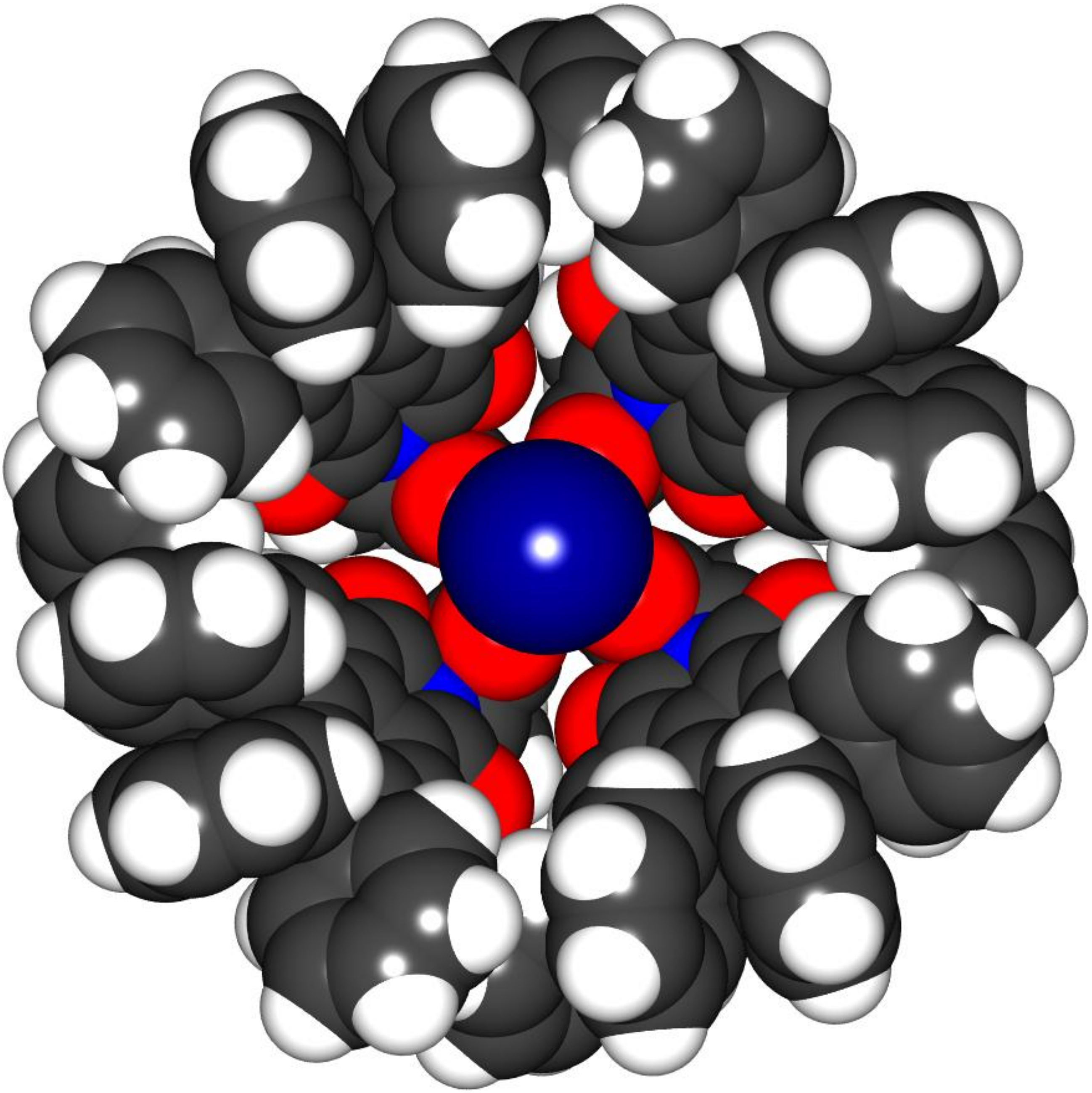

Figure S301. Van-der Waals representation of molecular structure showing the orientation and symmetry of the substituents.

Data Plots: Diffraction Data

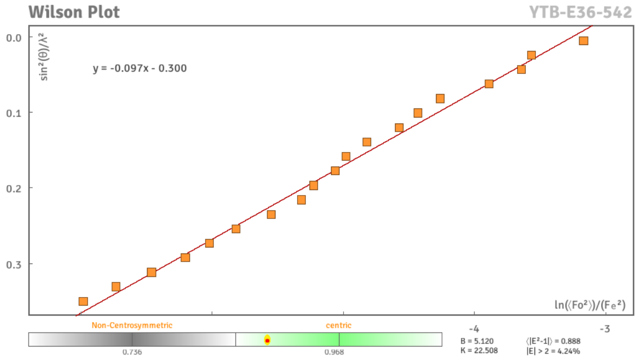

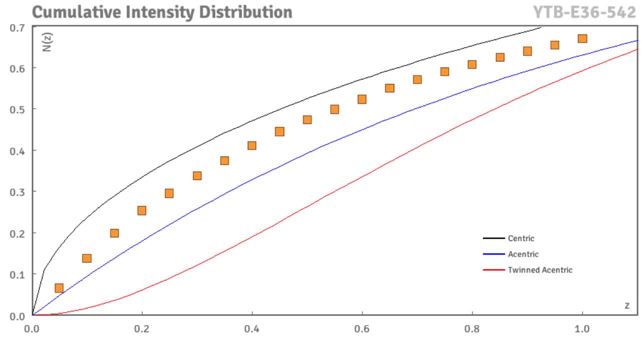

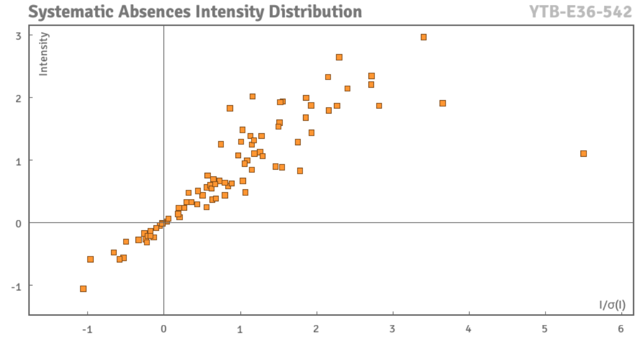

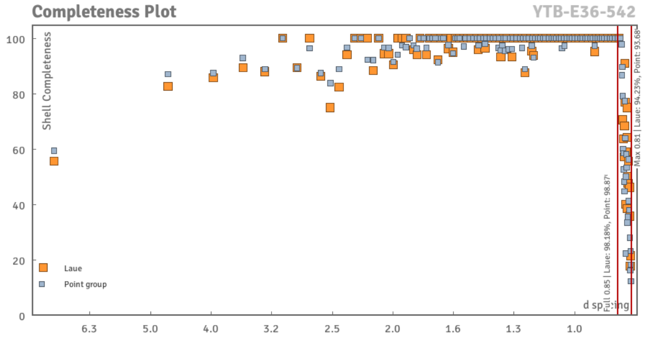

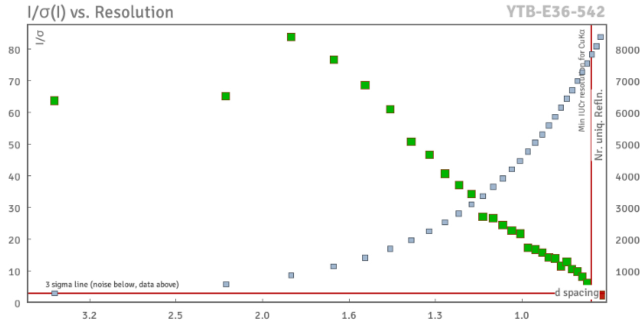

Data Plots: Refinement and Data

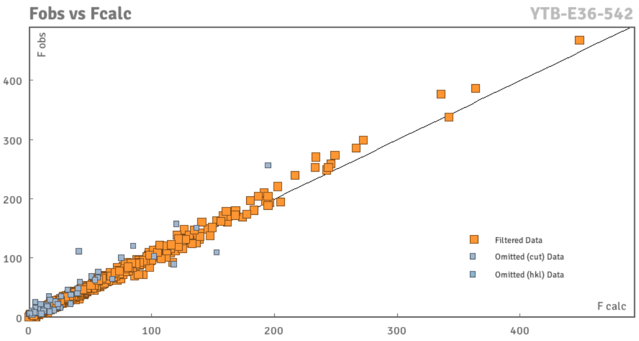

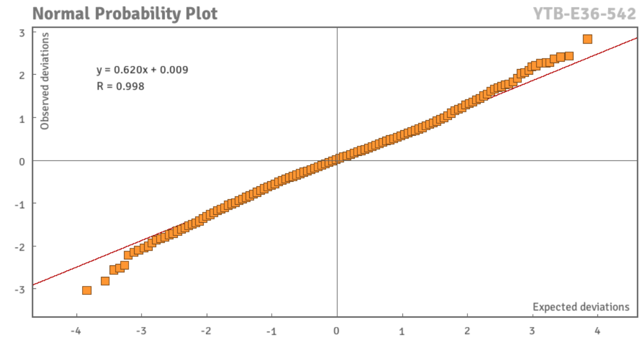

Reflection Statistics

Total reflections (after filtering)	100915	Unique reflections	8248

Completeness	0.937	Mean I/σ	31.3
hkl_{max} collected	(32, 31, 16)	hkl_{min} collected	(-30, -30, -15)
hkl_{max} used	(22, 32, 16)	hkl_{min} used	(-21, 0, 0)
Lim d_{max} collected	100.0	Lim d_{min} collected	0.77
d_{max} used	12.98	d_{min} used	0.81
Friedel pairs	14896	Friedel pairs merged	0
Inconsistent equivalents	1	R_{int}	0.0491
R_{sigma}	0.0179	Intensity transformed	0
Omitted reflections	0	Omitted by user (OMIT hkl)	1026
Multiplicity	(15548, 13006, 7459, 4268, 2228, 859, 358, 165, 69, 18, 1)	Maximum multiplicity	29
Removed systematic absences	100	Filtered off (Shel/OMIT)	0

Table S12. Fractional Atomic Coordinates (×10⁴) and Equivalent Isotropic Displacement Parameters (Å²×10³) for **Compound 7a.** U_{eq} is defined as 1/3 of the trace of the orthogonalised U_{ij}.

Atom	x	y	z	U_{eq}
Rh1	5000	0	7497.2(4)	56.3(2)
Rh2	5000	0	9336.2(4)	62.9(2)
O1	5653.1(16)	1492.2(15)	8002(3)	85.5(10)
C1	6009(2)	1459.4(19)	7412(5)	94(2)
C2	6057.6(18)	1804(2)	6492(5)	83.5(17)
C3	5705(2)	2198.3(19)	6395(4)	87.1(17)
C4	5704.3(18)	2510.9(16)	5527(4)	82.6(16)
C5	6049(2)	2430(2)	4718(4)	87.2(17)
O1B	7112.1(16)	669(2)	6914(5)	124(2)
C1B	6798(2)	1001(2)	6775(5)	90.6(19)
C2B	6806.0(19)	1339.0(19)	5843(5)	88.9(19)
C3B	7177(2)	1251(2)	5103(5)	91.2(18)
C4B	7212.1(19)	1568(2)	4242(4)	87.3(16)
C5B	6864.3(19)	1969.4(18)	4089(4)	79.3(14)
O2	5659.3(14)	413.0(14)	9301(3)	72.1(8)
O3	5684.2(12)	378.9(13)	7567(3)	66.4(8)
N1	6416.5(13)	1108.7(16)	7508(4)	87.1(15)
C14	6371(2)	786(3)	8459(5)	96.3(16)
C15	5854.3(16)	502.3(18)	8446(5)	73.0(12)
C16	6583(2)	1008(3)	9463(4)	114.5(18)
C17	7108(3)	1241(4)	9229(8)	140(3)
C18	6665(3)	570(3)	10229(7)	134(3)
C19	6242(3)	1412(3)	9971(7)	142(2)
N2A	5000	0	5783(8)	65(3)
C43A	5000	0	4888(8)	82(4)
C44A	5000	0	3769(8)	107(6)
N2B	5000	0	11037(7)	71(3)
C43B	5000	0	11931(7)	82(4)
C44B	5000	0	13050(7)	107(6)
C6	6447.2(14)	2045.5(18)	4808(4)	77.2(14)
C7	6436.1(14)	1732.2(18)	5723(4)	78.9(15)
C8	5308.0(19)	2927.6(17)	5529(4)	85.8(16)
C9	4917.3(18)	2982.3(18)	4806(4)	91.7(16)
C10	4559(2)	3375(2)	4886(5)	102(2)
C11	4593(3)	3714(2)	5705(5)	106(2)
C12	4976(3)	3662(2)	6437(5)	117(2)
C13	5338(2)	3272(2)	6347(5)	104(2)
C8B	5933(3)	2713.1(17)	3742(4)	101(2)
C9B	5760(3)	2447(2)	2880(5)	119(3)
C10B	5615(5)	2705(3)	1990(6)	165(5)
C11B	5655(4)	3236(3)	1960(6)	143(4)

Atom	x	y	z	U_{eq}
C12B	5854(3)	3503(2)	2790(6)	127(3)
C13B	6000(3)	3242.5(18)	3679(5)	116(3)
C8C	6965(2)	2304.0(14)	3175(3)	79.9(14)
C9C	6925(3)	2115.3(17)	2176(3)	106(2)
C10C	7069(3)	2410.9(19)	1335(4)	110(2)
C11C	7218(3)	2915.2(18)	1495(4)	100(2)
C12C	7258(3)	3110.0(17)	2483(3)	87.2(17)
C13C	7132(2)	2805.7(14)	3326(3)	74.8(12)
C8D	7646.6(18)	1461(2)	3518(4)	97.8(19)
C9D	7638(2)	1028(2)	2892(5)	111(2)
C10D	8036(3)	924(3)	2213(6)	139(3)
C11D	8454(3)	1253(3)	2181(7)	149(4)
C12D	8471(2)	1684(3)	2805(7)	135(4)
C13D	8070(2)	1787(2)	3483(5)	105(2)

Table S13: Anisotropic Displacement Parameters (×10[4]) for **Compound 7a**. The anisotropic displacement factor exponent takes the form: $-2\pi^2[h^2a^{*2} \times U_{11} + ... + 2hka^* \times b^* \times U_{12}]$

Atom	U_{11}	U_{22}	U_{33}	U_{23}	U_{13}	U_{12}
Rh1	57.3(2)	57.3(2)	54.3(3)	0	0	0
Rh2	67.4(3)	67.4(3)	53.9(3)	0	0	0
O1	79(2)	72(2)	106(3)	0(2)	-2(2)	-10.1(17)
C1	91(4)	57(3)	133(6)	12(3)	-27(4)	-28(3)
C2	63(3)	75(3)	112(5)	20(3)	-14(3)	-22(2)
C3	83(3)	61(3)	118(5)	0(3)	-18(3)	-12(2)
C4	78(3)	63(3)	107(4)	11(3)	-24(3)	-14(2)
C5	96(4)	69(3)	96(4)	10(3)	-27(3)	-21(3)
O1B	64(2)	124(4)	183(6)	72(4)	-3(3)	-1(2)
C1B	62(3)	94(4)	116(5)	33(4)	-18(3)	-14(3)
C2B	63(3)	67(3)	137(6)	16(3)	-21(3)	-22(2)
C3B	72(3)	93(4)	108(4)	27(3)	-11(3)	-13(3)
C4B	81(3)	71(3)	110(5)	14(3)	-10(3)	-16(3)
C5B	71(3)	67(3)	100(3)	9(3)	-22(3)	-19(2)
O2	74.3(19)	75.5(19)	66.5(19)	-0.5(15)	-11.4(15)	-8.3(16)
O3	60.6(17)	59.9(16)	79(2)	5.8(15)	1.4(15)	-0.8(13)
N1	70(3)	83(3)	108(4)	26(3)	-29(3)	-23(2)
C14	76(2)	99(3)	114(3)	24(3)	-32(2)	-23(2)
C15	65(2)	66(2)	87(4)	5(2)	-18(2)	-4.1(17)
C16	100(3)	129(4)	114(3)	22(2)	-33(2)	-49(3)
C17	106(3)	165(7)	150(6)	37(5)	-43(3)	-68(4)
C18	120(5)	157(5)	126(5)	42(4)	-49(4)	-52(4)
C19	133(5)	150(5)	142(6)	-14(4)	-37(4)	-40(4)
N2A	69(5)	69(5)	56(3)	0	0	0
C43A	95(6)	95(6)	56(3)	0	0	0
C44A	133(9)	133(9)	56(3)	0	0	0
N2B	79(5)	79(5)	56(3)	0	0	0
C43B	95(6)	95(6)	56(3)	0	0	0
C44B	133(9)	133(9)	56(3)	0	0	0
C6	72(3)	67(3)	92(4)	3(3)	-20(3)	-19(2)
C7	75(3)	67(3)	94(4)	16(3)	-29(3)	-18(2)
C8	103(4)	61(3)	94(4)	8(3)	-13(3)	-6(3)
C9	78(4)	77(3)	120(4)	5(3)	-8(3)	-10(3)
C10	108(5)	86(4)	111(5)	10(4)	-18(4)	4(3)
C11	119(5)	87(4)	111(5)	4(4)	-16(4)	17(4)
C12	140(6)	89(4)	123(5)	8(3)	-4(6)	46(5)

Atom	U_{11}	U_{22}	U_{33}	U_{23}	U_{13}	U_{12}
C13	119(5)	87(4)	107(5)	5(3)	-26(4)	7(4)
C8B	94(4)	81(4)	129(6)	12(4)	-22(4)	7(3)
C9B	119(6)	126(6)	111(6)	4(5)	-34(5)	9(5)
C10B	158(10)	195(12)	143(9)	43(9)	-38(8)	-6(9)
C11B	142(8)	160(9)	128(7)	59(7)	-38(6)	6(7)
C12B	102(5)	119(6)	161(9)	54(6)	-3(5)	-5(5)
C13B	95(4)	88(4)	163(7)	46(5)	-18(5)	-1(3)
C8C	90(3)	61(3)	89(4)	7(2)	-23(3)	-17(2)
C9C	167(7)	58(3)	93(4)	4(3)	-37(4)	-21(4)
C10C	160(7)	82(4)	88(4)	-5(3)	-25(4)	-9(4)
C11C	127(5)	76(3)	98(5)	11(3)	3(4)	-18(3)
C12C	98(4)	65(3)	99(4)	-7(3)	2(3)	-25(3)
C13C	75(3)	62(3)	87(3)	-5(2)	-3(3)	-16(2)
C8D	102(4)	82(4)	110(5)	23(3)	-12(4)	-22(3)
C9D	122(6)	115(6)	96(5)	21(4)	2(4)	-6(4)
C10D	179(10)	132(7)	105(6)	24(5)	25(6)	1(7)
C11D	160(9)	136(8)	152(9)	48(7)	19(7)	-22(7)
C12D	96(5)	128(7)	180(10)	54(7)	8(6)	-21(5)
C13D	80(4)	95(4)	141(7)	33(4)	9(4)	-12(3)

Compound 7b

R_1=7.84%

Submitted by: **Yannick Boni**

Solved by: **John Bacsa**

Crystal Data and Experimental

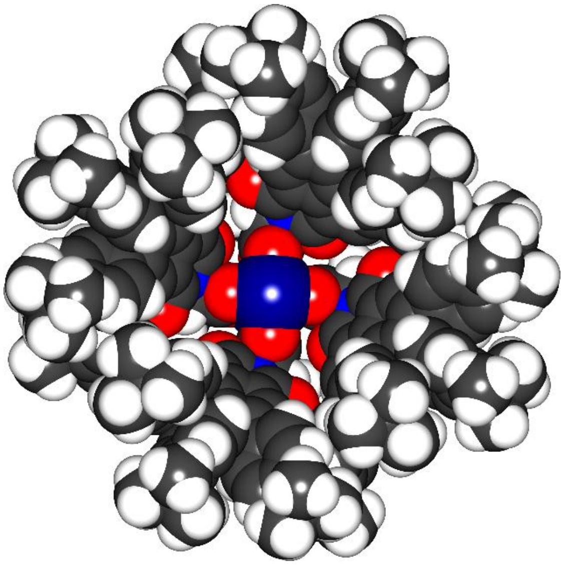

Experimental. Single green crystals of **Compound 7b** were crystallised from $CHCl_3$ and hexane by vapor diffusion. A suiTable Scrystal with dimensions 0.26 × 0.19 × 0.16 mm³ was selected and mounted on a loop with paratone on a Rigaku Synergy-S diffractometer. The crystal was kept at a steady T = 100.0(4) K during data collection. The structure was solved the ShelXT (Sheldrick, 2015) solution program using dual methods and by using Olex2 (Dolomanov et al., 2009) as the graphical interface. The model was refined with ShelXL 2018/3 (Sheldrick, 2015) using full matrix least squares minimisation on $\boldsymbol{F^2}$.

Crystal Data. $C_{232}H_{256}N_4O_{16}Rh_2$, M_r = 3562.21, tetragonal, $I4$ (No. 79), a = 32.79954(19) Å, b = 32.79954(19) Å, c = 11.77553(12) Å, $\alpha = \beta = \gamma =$ 90°, V = 12668.2(2) Å³, T = 100.0(4) K, Z = 2, Z' = 0.25, μ(Cu K$_\alpha$) = 1.440 mm⁻¹, 52250 reflections measured, 10808 unique (R_{int} = 0.0698) which were used in all calculations. The final wR_2 was 0.2066 (all data) and R_1 was 0.0784 (I≥2 σ(I)).

**Compound**	**7b**
Formula	$C_{232}H_{256}N_4O_{16}Rh_2$
$D_{calc.}$/ g cm^{-3}	0.934
μ/mm^{-1}	1.440
Formula Weight	3562.21
Colour	green
Shape	needle
Size/mm³	0.26×0.19×0.16
T/K	100.0(4)
Crystal System	tetragonal
Flack Parameter	0.312(14)
Hooft Parameter	0.162(3)
Space Group	$I4$
a/Å	32.79954(19)
b/Å	32.79954(19)
c/Å	11.77553(12)
α/°	90
β/°	90
γ/°	90
V/Å³	12668.2(2)
Z	2
Z'	0.25
Wavelength/Å	1.54184
Radiation type	Cu K$_\alpha$
Θ_{min}/°	2.694
Θ_{max}/°	65.079
Measured Refl's.	52250
Indep't Refl's	10808
Refl's I≥2 σ(I)	10742
R_{int}	0.0698
Parameters	584
Restraints	650
Largest Peak	1.601
Deepest Hole	-1.108
GooF	1.039
wR_2 (all data)	0.2066
wR_2	0.2059
R_1 (all data)	0.0787
R_1	0.0784

Structure Quality Indicators

**Reflections:**	d min (Cu) 0.85	I/σ(I) 24.8	Rint 6.98%	Compl.(full) 100% (IUCr) 100%
**Refinement:**	Shift -0.002	Max Peak 1.6	Min Peak -1.1	GooF 1.039

A green needle-shaped crystal with dimensions 0.26 × 0.19 × 0.16 mm^3 was mounted on a loop with paratone. Data were collected using a XtaLAB Synergy, Dualflex, HyPix diffractometer equipped with an Oxford Cryosystems low-temperature device operating at T = 100.0(4) K.

Data were measured using ω scans using Cu K_α radiation. The diffraction pattern was indexed and the total number of runs and images was based on the strategy calculation from the program CrysAlisPro (Rigaku, V1.171.40.84a, 2020). The maximum resolution that was achieved was Θ = 65.079° (0.85 Å).

The unit cell was refined using CrysAlisPro (Rigaku, V1.171.40.84a, 2020) on 30769 reflections, 59% of the observed reflections.

Data reduction, scaling and absorption corrections were performed using CrysAlisPro (Rigaku, V1.171.40.84a, 2020). The final completeness is 100.00 % out to 65.079° in Θ. A numerical absorption correction based on Gaussian integration over a multifaceted crystal model was performed using CrysAlisPro 1.171.40.84a (Rigaku Oxford Diffraction, 2020). An empirical absorption correction using spherical harmonics, implemented in SCALE3 ABSPACK scaling algorithm was also used. The absorption coefficient μ of this material is 1.440 mm^{-1} at this wavelength (λ = 1.54184Å) and the minimum and maximum transmissions are 0.603 and 1.000.

The structure was solved and the space group $I4$ (# 79) determined by the ShelXT (Sheldrick, 2015) structure solution program using dual methods and refined by full matrix least squares minimisation on $\boldsymbol{F}^2$ using version 2018/3 of ShelXL 2018/3 (Sheldrick, 2015). All non-hydrogen atoms were refined anisotropically. Hydrogen atom positions were calculated geometrically and refined using the riding model.

When viewed along the Rh-Rh axis, the complex has perfect 4-fold (C4) crystallographic symmetry with no deviations from C4 symmetry and a cyclic arrangement of t-butyl groups on the top periphery of the molecule. The molecule is extremely large with a molecular surface area of 3294 $Å^2$ and a molecular volume of 2979 $Å^3$. The void inside the interior of the molecule is very large and has a solvent accessible volume of 6334 $Å^3$ (i.e. over two-times the molecular volume). The diameter of the complex at its top periphery of the molecule is 2.5 nm (25 Å).

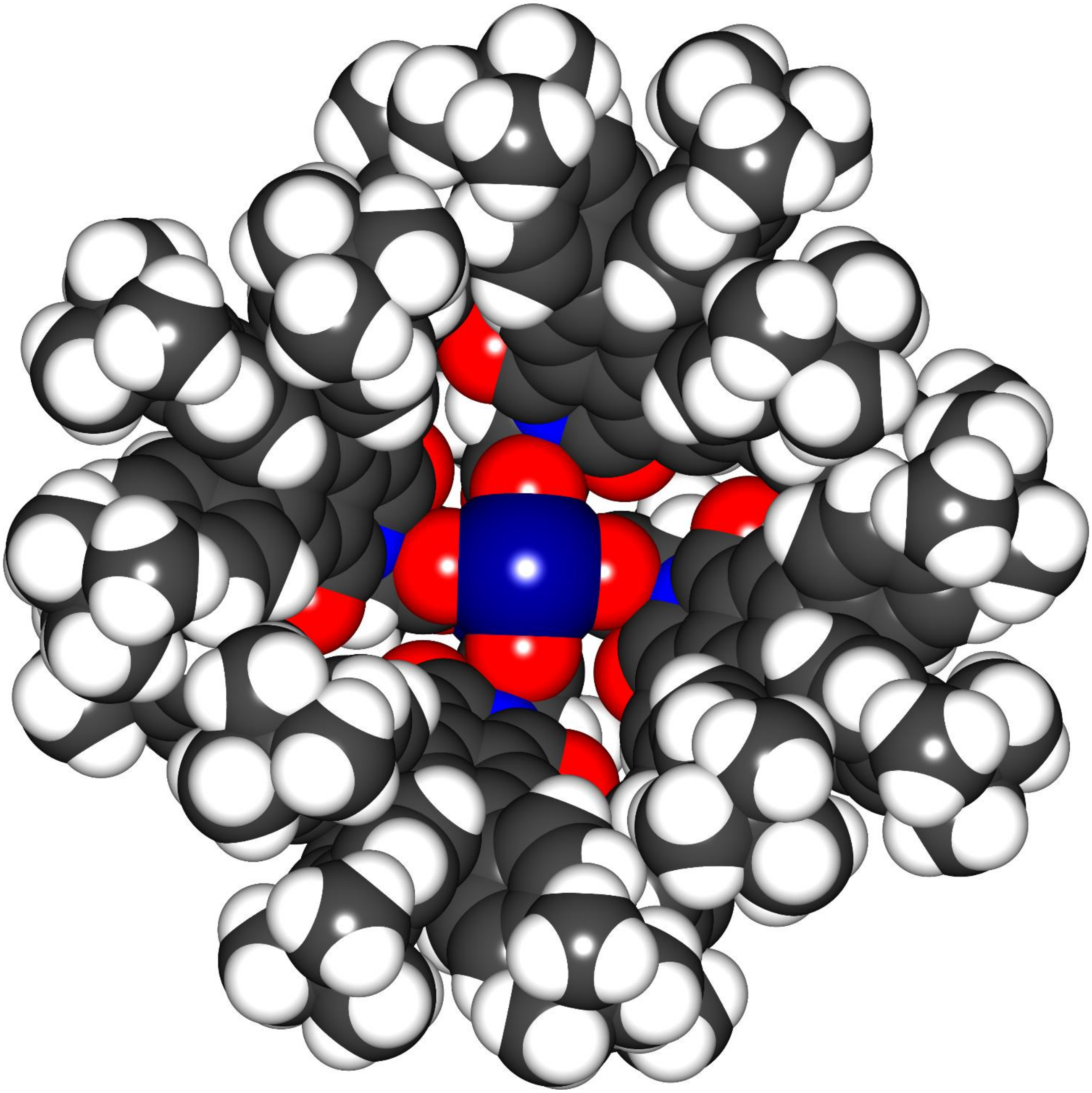

Figure S302. The molecular structure. When viewed along the Rh-Rh axis, the complex has perfect 4-fold (C4) crystallographic symmetry with no deviations from C4 symmetry and a cyclic arrangement of t-butyl groups on the top periphery of the molecule. The molecule is extremely large with a molecular surface area of 3294 Å^2 and a molecular volume of 2979 Å^3. The void inside the interior of the molecule is very large and has a solvent accessible volume of 6334 Å^3 (i.e. over two-times the molecular volume). The diameter of the complex at its top periphery of the molecule is 2.5 nm (25 Å).

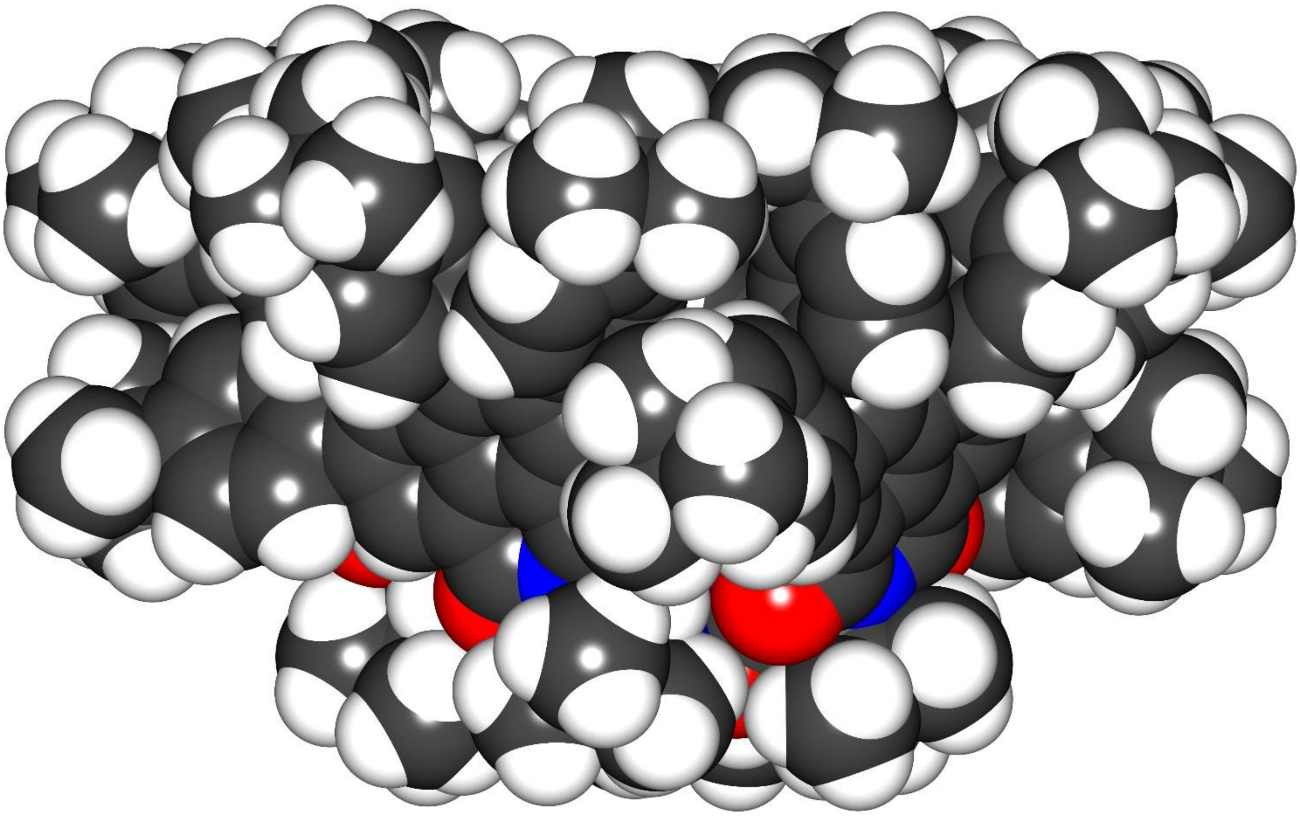

Figure S303. Side-on view of the molecular structure.

_refine_special_details: Refined as a 2-component inversion twin.

Data Plots: Diffraction Data

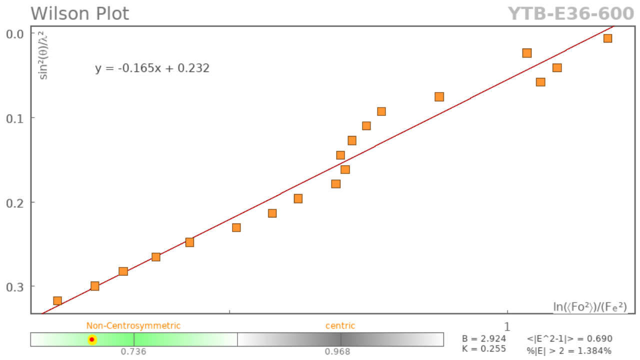

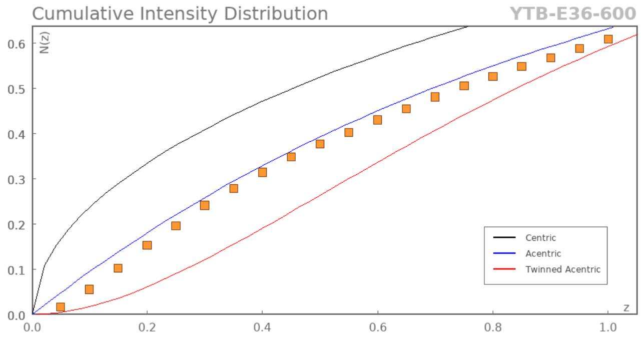

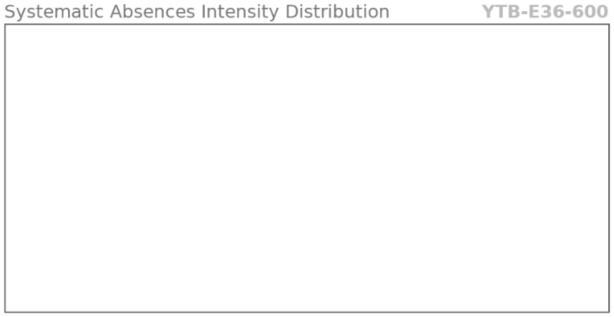

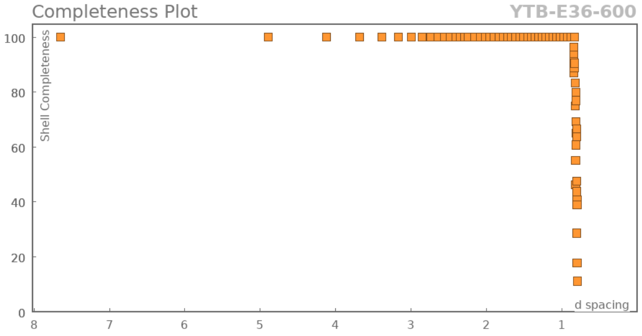

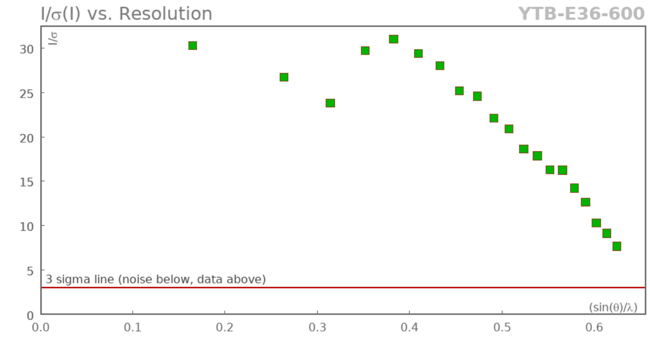

Data Plots: Refinement and Data

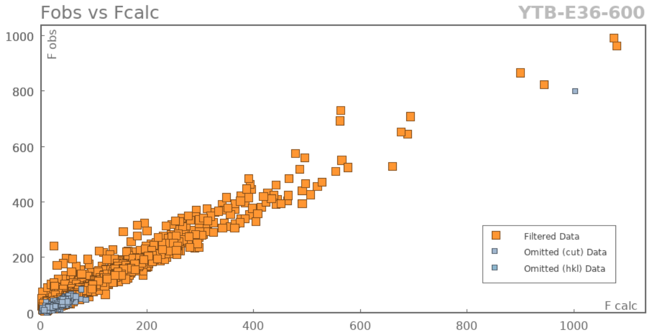

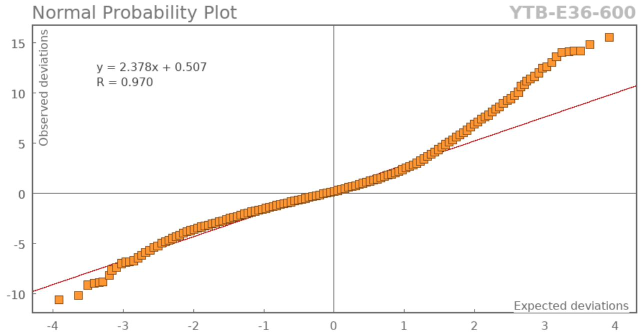

Reflection Statistics

Total reflections (after filtering)	52250	Unique reflections	10808
Completeness	1.0	Mean I/σ	23.23
hkl_{max} collected	(39, 40, 14)	hkl_{min} collected	(-39, -35, -13)
hkl_{max} used	(27, 38, 13)	hkl_{min} used	(-26, 0, -13)
Lim d_{max} collected	20.0	Lim d_{min} collected	0.85
d_{max} used	16.4	d_{min} used	0.85
Friedel pairs	6546	Friedel pairs merged	0
Inconsistent equivalents	23	R_{int}	0.0698
R_{sigma}	0.0404	Intensity transformed	0
Omitted reflections	0	Omitted by user (OMIT hkl)	0
Multiplicity	(12904, 7730, 4567, 2053, 548, 185, 98, 33, 4)	Maximum multiplicity	20
Removed systematic absences	0	Filtered off (Shel/OMIT)	2863

Images of the Crystal on the Diffractometer

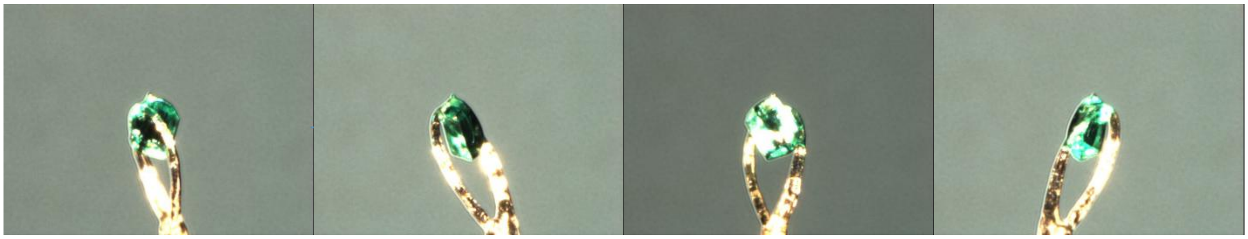

Table S14. Fractional Atomic Coordinates (×10^4) and Equivalent Isotropic Displacement Parameters (Å^2×10^3) for **compound 7b.** U_{eq} is defined as 1/3 of the trace of the orthogonalised U_{ij}.

Atom	x	y	z	U_{eq}
C1	139(2)	775(2)	-3801(5)	47.6(14)
C2	203(2)	1235(2)	-3716(8)	54.8(16)
C3	333(3)	1608(3)	-1904(9)	68(2)
C4	589(3)	1695(3)	-934(8)	62.8(18)
C5	978(3)	1490(3)	-824(8)	67(2)
C6	1131(3)	1191(3)	-1619(7)	61.3(18)
C7	840(3)	1123(3)	-2609(8)	63.8(18)
C8	1474(3)	1027(3)	-1534(8)	70(2)
C11	1235(3)	1585(2)	217(7)	54.5(16)
C14	470(3)	1981(3)	-143(8)	61.4(19)
C54	285(4)	1451(3)	-4805(9)	73(2)
C55	651(4)	1305(4)	-5405(11)	88(3)
C56	-90(4)	1428(4)	-5479(10)	87(3)
C57	335(6)	1909(4)	-4632(14)	104(4)
N1	482(3)	1339(2)	-2741(7)	63.7(16)
O1	113.1(17)	603.7(14)	-2744(4)	42.8(11)
O2	81(3)	604.8(19)	-4672(5)	71(2)
O3	-16(2)	1754.8(19)	-1983(6)	71.4(16)
O4	934.9(17)	883.1(18)	-3312(4)	56.8(13)
Rh1	0	0	-4607.2(14)	40.3(3)
Rh2	0	0	-2590.9(14)	28.7(2)
C9	1735(3)	1074(3)	-613(8)	63(2)
C10	1590(3)	1338(3)	286(8)	63.1(19)
C12	1132(3)	1905(3)	862(8)	65.9(19)
C13	733(3)	2101(3)	740(7)	68(2)
C15_2	1397(2)	2108(2)	1754(4)	64(2)
C16_2	1316(2)	2097(2)	2882(4)	78(3)
C17_2	1549.5(19)	2320(2)	3622(5)	70(2)
C18_2	1852(2)	2590(2)	3186(4)	107(4)
C19_2	1957(3)	2561(3)	2094(4)	97(4)
C20_2	1723(2)	2341(2)	1314(5)	87(3)
C21_2	2100.5(15)	2870.1(15)	3983(4)	85(3)
C22_2	1899(4)	3286(2)	3961(14)	124(4)
C23_2	2546.4(18)	2914(5)	3671(10)	120(5)
C24_2	2082(6)	2692(4)	5170(5)	130(6)
C15_3	1790.5(14)	1325.7(18)	1436(5)	71.7(19)
C16_3	2166.3(15)	1466.6(19)	1708(4)	67(2)
C17_3	2359(2)	1462(2)	2742(4)	102(4)
C18_3	2128.3(16)	1285(2)	3640(4)	84(3)
C19_3	1756.2(16)	1141(2)	3411(5)	89(4)
C20_3	1570(2)	1152(2)	2344(4)	74(2)
C21_3	2293.8(17)	1292.1(16)	4869(4)	119(3)
C22_3	2405(5)	853.2(19)	5123(9)	124(5)
C23_3	1967(3)	1436(4)	5685(6)	100(4)
C24_3	2651(3)	1582(3)	4996(9)	163(7)
C15_1	597.7(18)	2433.4(17)	1566(5)	69.6(19)
C16_1	789(3)	2798.7(15)	1638(5)	80(3)
C17_1	684(2)	3104(2)	2378(6)	111(3)
C18_1	338.7(19)	3019.6(16)	3073(5)	89(2)
C19_1	127(2)	2673.1(16)	2993(5)	83(2)
C20_1	243(2)	2371(2)	2222(5)	77(2)
C21_1	199.8(18)	3355.3(16)	3909(4)	117(4)
C22_1	233(11)	3166(4)	5078(6)	220(14)
C23_1	-236(2)	3501(4)	3783(14)	128(5)
C24_1	427(4)	3757(2)	3870(30)	218(13)
C15_4	2117.7(18)	868(2)	-527(4)	60.1(17)
C16_4	2296.0(17)	660(3)	334(6)	82(3)
C17_4	2678.1(16)	490(4)	296(5)	91(3)

Atom	x	y	z	U_{eq}
C18_4	2929.1(16)	532(3)	-684(4)	101(4)
C19_4	2749.9(18)	705(3)	-1582(5)	85(3)
C20_4	2364.7(16)	885(3)	-1508(5)	75(3)
C21_4	3317.0(16)	271.2(17)	-845(5)	121(4)
C22_4	3649(3)	561(3)	-1228(16)	174(9)
C23_4	3309(6)	-38(3)	-1800(8)	199(9)
C24_4	3409(5)	6(3)	177(7)	179(9)

Table S15. Anisotropic Displacement Parameters (×10^4) for **compound 7b**. The anisotropic displacement factor exponent takes the form: $-2\pi^2[h^2a^{*2} \times U_{11} + ... + 2hka^* \times b^* \times U_{12}]$

Atom	U_{11}	U_{22}	U_{33}	U_{23}	U_{13}	U_{12}
C1	59(4)	66(3)	18(3)	10(2)	1(2)	-1(3)
C2	59(4)	62(3)	43(4)	14(3)	-12(3)	-2(3)
C3	86(5)	65(5)	52(4)	-9(4)	-12(4)	1(4)
C4	72(4)	66(4)	50(4)	-4(3)	-5(3)	-16(3)
C5	77(5)	74(5)	50(4)	-8(3)	-12(3)	-11(4)
C6	80(4)	64(4)	40(4)	-3(3)	-11(3)	-24(3)
C7	72(4)	72(4)	47(4)	-13(3)	-8(3)	-5(3)
C8	83(5)	82(6)	44(4)	-13(4)	-15(4)	-15(4)
C11	74(4)	47(4)	43(4)	7(3)	-5(3)	-20(3)
C14	77(5)	55(4)	52(4)	1(3)	-6(3)	-5(4)
C54	98(6)	58(5)	63(5)	22(4)	-5(4)	-10(4)
C55	96(7)	104(8)	65(6)	-5(6)	1(5)	-18(6)
C56	123(8)	75(6)	63(6)	18(5)	-26(6)	-20(6)
C57	142(13)	64(6)	107(10)	-2(6)	-16(9)	-26(7)
N1	82(4)	63(4)	46(4)	-3(3)	-6(3)	-4(3)
O1	73(3)	41(2)	14(2)	10.3(18)	-1(2)	-4.1(19)
O2	139(6)	62(3)	13(2)	20(2)	-15(3)	-13(3)
O3	86(4)	56(3)	72(4)	-16(3)	-6(3)	3(3)
O4	61(3)	74(3)	36(3)	-8(2)	-9(2)	-10(2)
Rh1	49.3(4)	49.3(4)	22.2(4)	0	0	0
Rh2	28.8(3)	28.8(3)	28.7(4)	0	0	0
C9	72(5)	67(5)	51(4)	-15(4)	-18(4)	-10(4)
C10	73(5)	70(5)	46(4)	-5(3)	-12(3)	-14(4)
C12	80(5)	67(4)	51(4)	-10(3)	-4(4)	-32(3)
C13	89(5)	81(6)	34(4)	-2(4)	4(3)	-19(4)
C15_2	91(4)	59(4)	41(3)	-7(2)	-6(3)	-17(3)
C16_2	101(6)	90(6)	42(3)	-9(2)	-4(2)	-30(5)
C17_2	94(5)	76(5)	42(3)	-6(3)	-7(3)	-19(4)
C18_2	149(7)	142(7)	28(2)	-13(3)	-8(3)	-82(7)
C19_2	151(7)	111(7)	28(2)	-10(3)	-8(3)	-78(6)
C20_2	124(5)	107(6)	30(3)	-23(3)	0(3)	-59(5)
C21_2	122(5)	118(5)	15(3)	-7(3)	-3(3)	-62(4)
C22_2	156(9)	125(5)	91(10)	-18(5)	-27(8)	-47(6)
C23_2	124(5)	185(14)	52(6)	-11(7)	2(4)	-72(5)
C24_2	212(17)	157(11)	22(3)	9(4)	-9(5)	-87(11)
C15_3	83(3)	88(5)	45(3)	1(4)	-6(3)	-24(3)
C16_3	80(3)	92(6)	29(3)	-17(3)	3(2)	-22(3)
C17_3	102(5)	171(11)	35(3)	4(4)	-8(3)	-61(6)
C18_3	89(4)	118(8)	44(3)	3(3)	4(3)	-22(4)
C19_3	93(4)	110(9)	64(4)	34(4)	-12(3)	-25(5)
C20_3	70(4)	92(6)	59(3)	20(3)	0(3)	2(4)
C21_3	156(7)	148(7)	54(3)	-10(3)	-22(3)	10(5)
C22_3	195(16)	152(7)	25(4)	-9(4)	4(6)	27(7)

Atom	U_{11}	U_{22}	U_{33}	U_{23}	U_{13}	U_{12}
C23_3	158(8)	100(8)	41(4)	15(4)	-22(4)	12(6)
C24_3	222(11)	246(15)	19(4)	10(7)	-29(6)	-72(12)
C15_1	116(5)	76(3)	17(3)	6(3)	6(3)	-6(3)
C16_1	130(6)	80(3)	29(4)	2(3)	15(4)	-14(3)
C17_1	166(7)	109(4)	57(5)	-25(4)	9(5)	4(5)
C18_1	146(6)	85(3)	37(4)	-4(3)	-11(3)	25(4)
C19_1	145(7)	85(3)	19(3)	-3(3)	-11(3)	25(4)
C20_1	126(5)	72(3)	32(3)	-4(3)	19(3)	-11(3)
C21_1	178(9)	104(5)	69(5)	-27(5)	3(6)	24(6)
C22_1	440(40)	150(14)	74(6)	-14(7)	7(10)	80(20)
C23_1	181(9)	126(12)	78(9)	-14(9)	9(7)	33(7)
C24_1	246(19)	123(7)	290(40)	-64(13)	80(20)	-12(11)
C15_4	67(3)	89(5)	24(2)	-7(2)	-6(2)	-24(3)
C16_4	57(3)	145(7)	45(3)	30(4)	1(2)	-23(4)
C17_4	62(3)	168(10)	43(4)	25(4)	0(3)	-12(4)
C18_4	65(3)	190(9)	46(3)	32(4)	3(2)	-6(5)
C19_4	73(3)	145(8)	37(3)	10(4)	-1(2)	-6(4)
C20_4	70(3)	129(8)	26(3)	3(3)	-3(2)	-13(3)
C21_4	67(4)	198(9)	97(7)	36(6)	11(4)	-3(5)
C22_4	92(8)	194(12)	240(30)	32(13)	74(13)	0(8)
C23_4	100(12)	310(17)	188(14)	-65(15)	20(12)	-9(13)
C24_4	90(10)	290(20)	155(12)	109(14)	0(9)	-11(13)

Compound 7c

Submitted by: **Duc Ly**

Solved by: **John Bacsa**

***R_1*=4.69%**

Crystal Data and Experimental

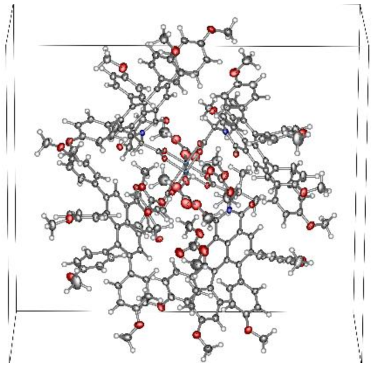

Experimental. Single green prism-shaped crystals of Compound 7c recrystallized from a mixture of chloroform and DMC. A suiTable Scrystal with dimensions 0.44 × 0.30 × 0.27 mm^3 was selected and mounted on a loop with paratone on a XtaLAB AFC11 (RCD3): quarter-chi single diffractometer. The crystal was kept at a steady *T* = 173.0(1) K during data collection. The structure was solved with the ShelXT (Sheldrick, 2015) solution program and by using and by using Olex2 1.5-alpha (Dolomanov et al., 2009) as the graphical interface. The model was refined with ShelXL 2018/3 (Sheldrick, 2015) using full matrix least squares minimisation on ***F*²**.

Crystal Data. $C_{201}H_{194}Cl_6N_4O_{48}Rh_2$, M_r = 3852.11, orthorhombic, *P*2$_1$2$_1$2 (No. 18), a = 27.4727(2) Å, b = 25.8856(2) Å, c = 13.01518(14) Å, $\alpha = \beta = \gamma = 90^\circ$, V = 9255.71(15) Å^3, T = 173.05(14) K, Z = 2, Z' = 0.5, μ(Cu K$_\alpha$) = 2.928, 134439 reflections measured, 17239 unique (R_{int} = 0.0270) which were used in all calculations. The final wR_2 was 0.1332 (all data) and R_1 was 0.0469 (I≥2 σ(I)).

**Compound**	Compound 7c
Formula	$C_{201}H_{194}Cl_6N_4O_{48}Rh_2$
$D_{calc.}$/ g cm^{-3}	1.382
μ/mm^{-1}	2.928
Formula Weight	3852.11
Colour	green
Shape	prism-shaped
Size/mm^3	0.44×0.30×0.27
T/K	173.05(14)
Crystal System	orthorhombic
Flack Parameter	-0.0089(19)
Hooft Parameter	-0.0081(9)
Space Group	*P*2$_1$2$_1$2
a/Å	27.4727(2)
b/Å	25.8856(2)
c/Å	13.01518(14)
α/°	90
β/°	90
γ/°	90
V/Å^3	9255.71(15)
Z	2
Z'	0.5
Wavelength/Å	1.54184
Radiation type	Cu K$_\alpha$
Θ_{min}/°	2.345
Θ_{max}/°	71.727
Measured Refl's.	134439
Indep't Refl's	17239
Refl's I≥2 σ(I)	16284
R_{int}	0.0270
Parameters	1201
Restraints	1235
Largest Peak	0.809
Deepest Hole	-0.828
GooF	1.041
wR_2 (all data)	0.1332
wR_2	0.1310
R_1 (all data)	0.0493
R_1	0.0469

Structure Quality Indicators

**Reflections:**	d min (CuKα) 2Θ=143.5° 0.81	I/σ(I) 57.5	Rint m=7.80 2.70%	Full 135.4° 98% to 143.5° 99.4	
**Refinement:**	Shift 0.000	Max Peak 0.8	Min Peak -0.8	GooF 1.041	Hooft -.0089(19)

A green prism-shaped-shaped crystal with dimensions 0.44 × 0.30 × 0.27 mm^3 was mounted on a loop with paratone. Data were collected using a XtaLAB AFC11 (RCD3): quarter-chi single diffractometer operating at T = 173.05(14) K.

Data were measured using ω scans with Cu K_{α} radiation. The diffraction pattern was indexed and the total number of runs and images was based on the strategy calculation from the program CrysAlisPro system (CCD 43.92a 64-bit (release 05-10-2023)). The maximum resolution that was achieved was Θ = 71.727° (0.83 Å).

The unit cell was refined using CrysAlisPro 1.171.43.92a (Rigaku OD, 2023) on 28794 reflections, 21% of the observed reflections.

Data reduction, scaling and absorption corrections were performed using CrysAlisPro 1.171.43.92a (Rigaku OD, 2023). The final completeness is 99.40 % out to 71.727° in Θ. A numerical absorption correction based on gaussian integration over a multifaceted crystal model was performed using CrysAlisPro 1.171.41.108a (Rigaku Oxford Diffraction, 2021). An empirical absorption correction using spherical harmonics, implemented in SCALE3 ABSPACK scaling algorithm was also applied. The absorption coefficient μ of this material is 2.928 mm^{-1} at this wavelength (λ = 1.54184Å) and the minimum and maximum transmissions are 0.326 and 1.000.

The structure was solved and the space group $P2_12_12$ (# 18) determined by the ShelXT (Sheldrick, 2015) structure solution program and refined by full matrix least squares minimisation on $\boldsymbol{F}^2$ using version 2018/3 of ShelXL 2018/3 (Sheldrick, 2015). All non-hydrogen atoms were refined anisotropically. Hydrogen atom positions were calculated geometrically and refined using the riding model.

The value of Z' is 0.5. This means that only half of the formula unit is present in the asymmetric unit, with the other half consisting of symmetry equivalent atoms. The moiety formula is C187 H168 N4 O36 Rh2, 2(C H Cl3), 4(C3 H6 O3).

The Flack parameter was refined to -0.0089(19). Determination of absolute structure using Bayesian statistics on Bijvoet differences using the Olex2 results in -0.0081(9). The chiral atoms in this structure are: C2(S), C2B(S). Note: The Flack parameter is used to determine chirality of the crystal studied, the value should be near 0, a value of 1 means that the stereochemistry is wrong and the model should be inverted. A value of 0.5 means that the crystal consists of a racemic mixture of the two enantiomers.

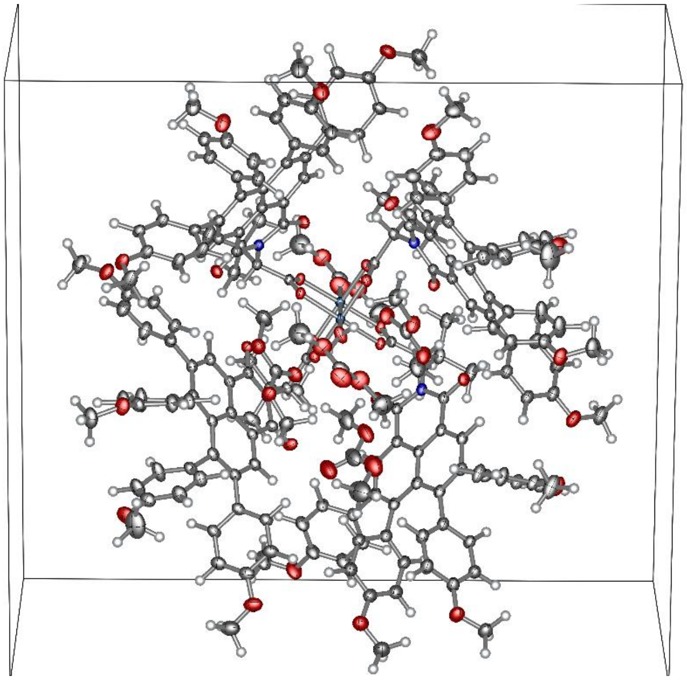

Figure S304. Thermal ellipsoidal representation (50% probability for all atoms, excluding hydrogens) of the molecular structure.

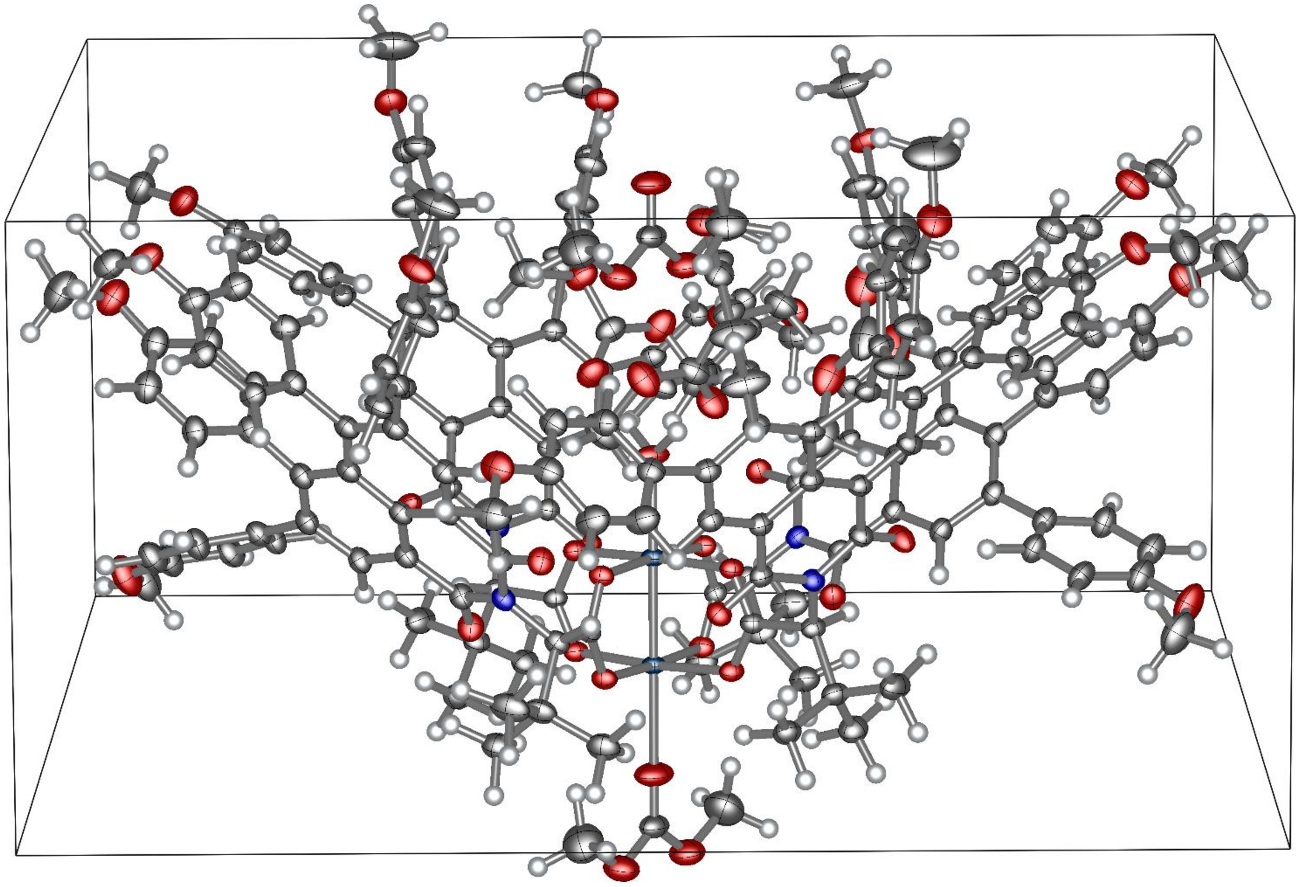

Figure S305. Thermal ellipsoidal representation (50% probability for all atoms, excluding hydrogens) of the molecular structure.

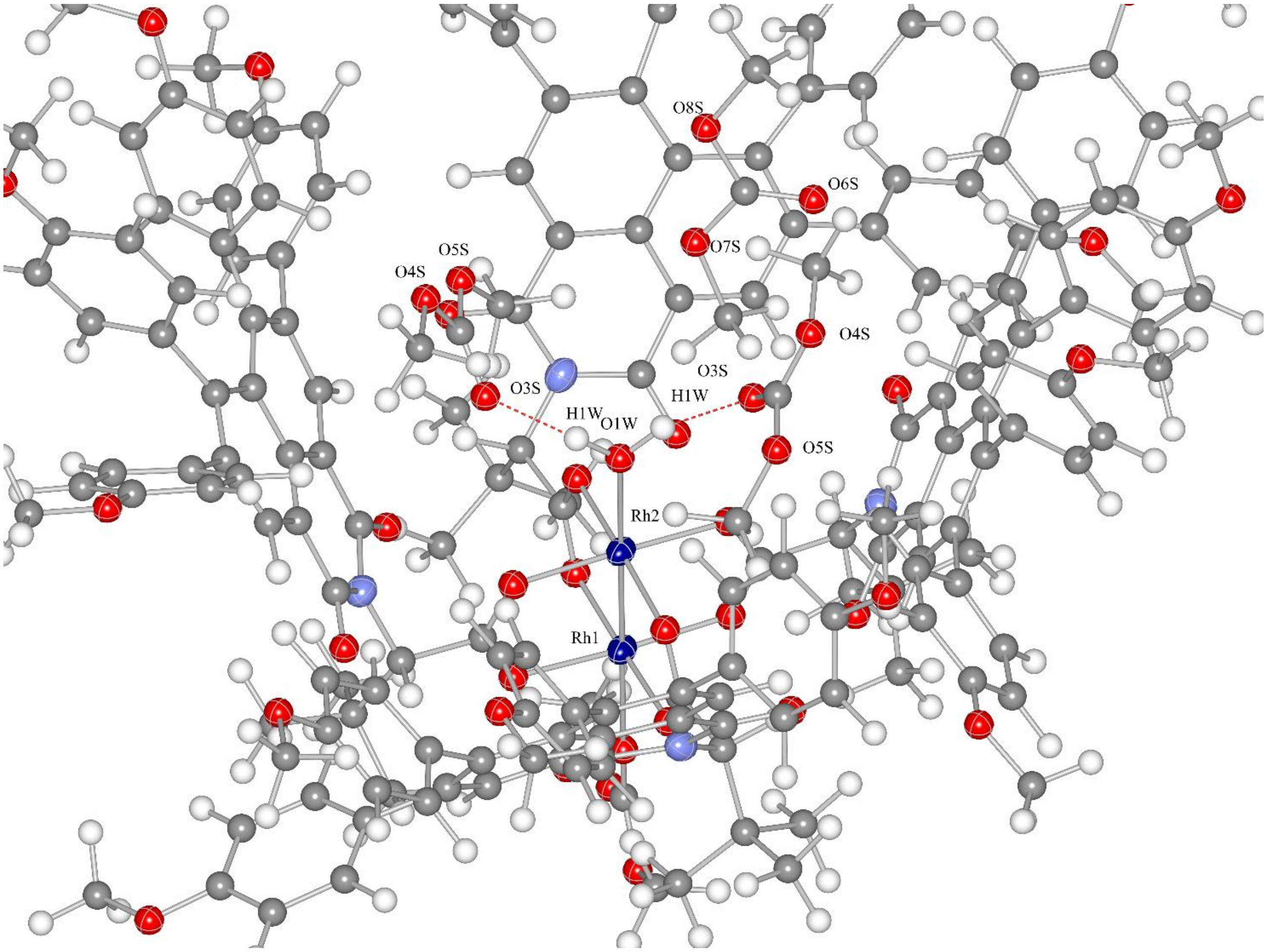

Figure S306. Close hydrogen contact

Data Plots: Diffraction Data

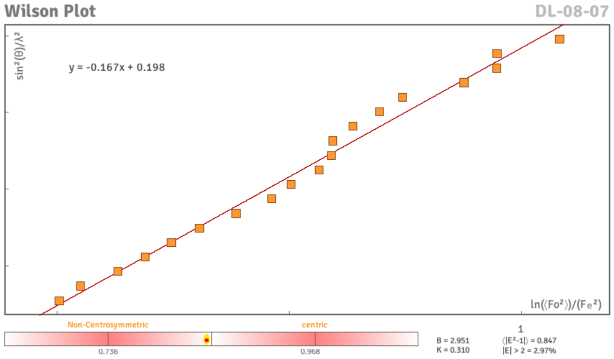

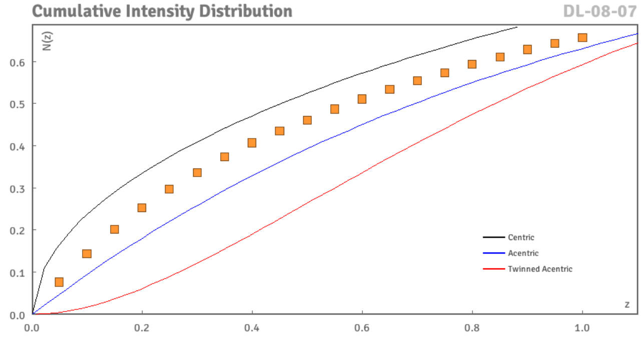

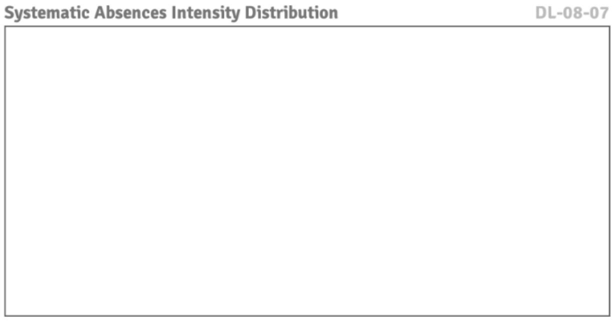

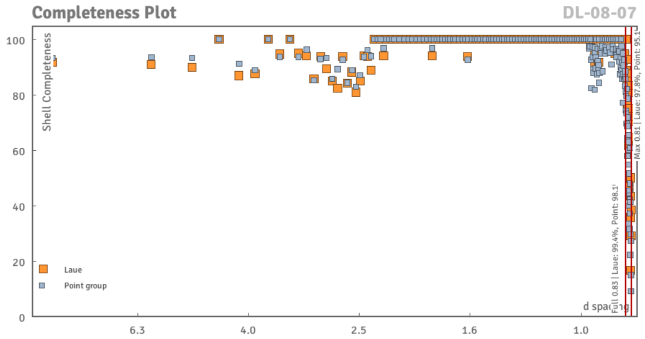

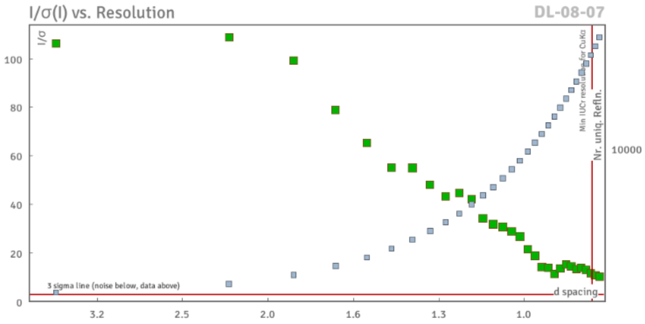

Data Plots: Refinement and Data

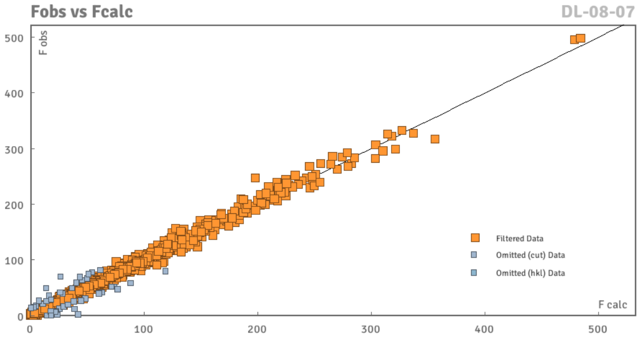

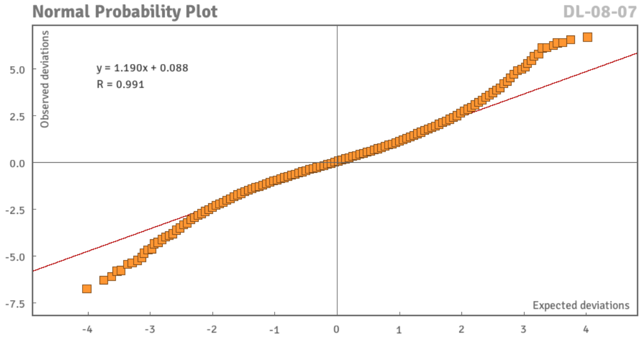

Reflection Statistics

Total reflections (after filtering)	134439	Unique reflections	17239
Completeness	0.951	Mean I/σ	37.59
hkl_{max} collected	(33, 31, 15)	hkl_{min} collected	(-33, -31, -14)
hkl_{max} used	(33, 31, 15)	hkl_{min} used	(-33, 0, 0)
Lim d_{max} collected	100.0	Lim d_{min} collected	0.77
d_{max} used	18.84	d_{min} used	0.81
Friedel pairs	19266	Friedel pairs merged	0
Inconsistent equivalents	37	R_{int}	0.027
R_{sigma}	0.0174	Intensity transformed	0
Omitted reflections	0	Omitted by user (OMIT hkl)	959
Multiplicity	(13185, 11599, 8778, 5995, 3960, 1935, 1127, 537, 302, 186, 48)	Maximum multiplicity	27
Removed systematic absences	0	Filtered off (Shel/OMIT)	0

Table S16. Fractional Atomic Coordinates (×10⁴) and Equivalent Isotropic Displacement Parameters (Å^2×10^3) for **Compound 7c**. U_{eq} is defined as 1/3 of the trace of the orthogonalised U_{ij}.

Atom	x	y	z	U_{eq}
C1S	5000	5000	-1912(6)	63.1(15)
C2S	4370(5)	5601(5)	-1934(10)	122(4)
O1S	5000	5000	-1025(5)	96(2)
O1W	5000	5000	4401(3)	55.0(12)
O2S	4716(2)	5280(2)	-2500(4)	82.4(14)
Rh1	5000	5000	839.2(3)	32.41(12)
Rh2	5000	5000	2668.5(3)	31.38(11)
C1	5799.4(13)	4471.1(15)	1739(3)	34.4(7)
C2	6298.0(13)	4207.2(14)	1776(3)	40.5(8)
C3	6553.4(16)	4075.2(19)	741(3)	55.2(10)
C4	6660(2)	4589(2)	184(4)	62.7(13)
C5	6270(2)	3714(2)	38(4)	62.0(14)
C6	7047(2)	3833(3)	1015(5)	81(2)
C7	6606.3(14)	3755.0(14)	3300(3)	40.5(8)
C8	6562.8(14)	3324.0(14)	4041(3)	40.1(8)
C9	6880.6(15)	3312.7(15)	4851(3)	44.0(9)
C10	6858.8(14)	2908.7(15)	5575(3)	45.1(9)
C11	6531.2(13)	2502.9(14)	5461(3)	43.6(8)
C12	5888.4(14)	3415.3(14)	2390(3)	42.3(9)
C13	5888.7(14)	2961.5(13)	3079(3)	42.0(8)
C14	5571.0(15)	2566.6(14)	2877(3)	45.0(9)
C15	5553.4(15)	2127.9(13)	3518(3)	45.0(9)
C16	5840.4(13)	2101.0(14)	4398(3)	45.7(8)
C17	6216.4(14)	2927.6(15)	3918(3)	40.6(8)
C18	6191.1(13)	2503.4(14)	4614(3)	42.1(8)
C19	7227.5(14)	2936.5(18)	6423(3)	46.0(10)
C20	7106.9(17)	3136(3)	7371(4)	62.2(15)
C21	7455.6(17)	3209(3)	8130(4)	65.2(16)
C22	7933.8(15)	3077(2)	7940(3)	51.2(11)
C23	8060.4(16)	2875(2)	7000(3)	47.3(10)
C24	7711.6(16)	2804(2)	6245(4)	49.7(11)
C25	8170(3)	3294(4)	9661(4)	95(3)
C26	6584.8(14)	2074.0(16)	6218(3)	45.7(9)
C27	6993.5(17)	1761.5(19)	6177(4)	48.2(10)
C28	7068.9(17)	1365.4(19)	6875(4)	51.5(11)
C29	6729.7(17)	1281.3(19)	7637(4)	56.6(11)
C30	6329(2)	1607(2)	7721(5)	62.0(13)
C31	6260.6(18)	1999(2)	7014(4)	53.5(11)
C32	5715.7(15)	1677.1(15)	5129(3)	49.7(10)
C33	5997.9(19)	1233.7(19)	5217(5)	56.7(12)
C34	5872.9(19)	842(2)	5904(5)	62.6(13)
C35	5469.7(19)	884.5(19)	6488(4)	63.8(13)
C36	5166.2(19)	1312(2)	6376(5)	61.8(13)
C37	5293.9(17)	1701.9(17)	5706(4)	51.8(11)
C38	5648(3)	127(3)	7421(8)	101(3)
C39	5211.7(15)	1706.9(14)	3191(4)	46.7(10)
C40	5370.4(18)	1203.6(17)	3081(5)	58.2(13)
C41	5070.6(19)	817(2)	2740(5)	67.6(14)
C42	4593.6(16)	930.3(17)	2477(5)	66.0(14)
C43	4424.9(18)	1434.4(18)	2546(5)	60.2(13)
C44	4739.9(16)	1818.0(17)	2898(5)	51.8(12)
C45	3818(2)	666(3)	1834(9)	106(3)
C46	7228(2)	629(3)	8395(6)	70.5(16)
N1	6269.9(10)	3768.9(12)	2496(2)	39.5(7)

Atom	x	y	z	U_{eq}
O1	5627.3(10)	4591.6(13)	879(2)	38.4(6)
O2	5620.9(10)	4571.9(12)	2610(2)	36.6(6)
O3	6912.3(12)	4089.6(12)	3393(3)	49.4(8)
O4	5575.8(12)	3482.6(13)	1744(3)	51.8(8)
O5	8287.8(13)	3140.0(19)	8668(3)	66.5(12)
O6	6789.9(14)	896.2(17)	8345(4)	73.6(12)
O7	5329.0(16)	529.7(17)	7208(4)	83.6(13)
O8	4292.5(15)	550.1(15)	2118(5)	94.9(17)
C1B	5526.7(14)	5826.2(15)	1768(3)	35.3(7)
C2B	5821.5(13)	6327.9(14)	1806(3)	38.5(8)
C3B	6078.1(17)	6504.4(17)	800(3)	49.0(9)
C4B	5678(2)	6665(2)	30(4)	61.1(14)
C5B	6406.2(19)	6100(2)	305(4)	55.1(12)
C6B	6371(2)	6991(2)	1056(5)	72.3(18)
C7B	6057.8(16)	6720.0(15)	3445(3)	42.1(9)
C8B	6353.7(14)	6709.2(14)	4394(3)	38.8(8)
C9B	6310.1(15)	7114.1(15)	5067(3)	43.4(9)
C10B	6617.0(14)	7148.3(14)	5933(3)	43.3(8)
C11B	6946.1(13)	6756.3(14)	6159(3)	42.2(8)
C12B	6424.9(14)	5892.5(14)	2927(3)	36.7(7)
C13B	6706.3(14)	5886.7(14)	3896(3)	38.4(8)
C14B	7008.2(15)	5475.9(15)	4089(3)	42.6(9)
C15B	7279.2(15)	5449.1(15)	5008(3)	44.4(9)
C16B	7255.4(15)	5846.7(14)	5726(3)	44.8(8)
C17B	6677.9(14)	6301.5(14)	4606(3)	38.5(8)
C18B	6965.9(14)	6300.8(11)	5517(3)	40.1(8)
C19B	6556.8(14)	7624.9(15)	6577(3)	44.7(9)
C20B	6125.6(17)	7713.3(17)	7088(4)	48.8(10)
C21B	6053.4(19)	8153.3(19)	7674(4)	58.6(12)
C22B	6416.7(17)	8522.5(16)	7736(4)	55.8(11)
C23B	6849.9(18)	8441.4(19)	7237(4)	57.6(11)
C24B	6925.6(17)	7990.0(18)	6679(4)	53.1(11)
C25B	6677(3)	9347(2)	8389(7)	91(2)
C26B	7282.4(14)	6858.0(18)	7035(3)	46.2(10)
C27B	7770.9(18)	6951(3)	6846(4)	73.2(19)
C28B	8087(2)	7092(3)	7623(4)	73.4(18)
C29B	7919.1(17)	7119(2)	8621(3)	58.3(12)
C30B	7437.6(19)	6997(3)	8834(4)	65.8(14)
C31B	7126.3(17)	6862(2)	8042(3)	55.3(12)
C32B	7534.5(14)	5761.2(18)	6694(3)	46.9(9)
C33B	8040.5(17)	5743(4)	6697(4)	86(2)
C34B	8299.1(19)	5648(4)	7598(5)	88(3)
C35B	8060.8(16)	5542(2)	8479(3)	54.0(11)
C36B	7554.8(18)	5525(3)	8484(4)	62.7(15)
C37B	7299.2(16)	5628(2)	7595(4)	53.5(12)
C38B	8763.9(19)	5599(4)	9510(6)	85(2)
C39B	7608.3(14)	4990.6(17)	5130(3)	48.2(9)
C40B	8003.0(19)	4923(2)	4487(4)	66.1(14)
C41B	8327(2)	4524(3)	4609(5)	71.4(16)
C42B	8256.1(16)	4169.8(18)	5391(4)	55.4(11)
C43B	7850.3(17)	4211.1(19)	6022(4)	52.5(11)
C44B	7534.7(16)	4627.3(18)	5891(4)	48.2(10)
C45B	9000(2)	3737(3)	4993(5)	77.1(19)
C46B	8084(4)	7226(6)	10410(4)	135(5)
N1B	6131.0(11)	6322.9(10)	2730(2)	37.8(7)
O1B	5407.7(11)	5652.3(12)	902(2)	38.8(6)
O2B	5398.1(11)	5656.1(11)	2634(2)	37.3(6)
O3B	5768.9(14)	7063.1(13)	3273(3)	57.3(9)

Atom	x	y	z	U_{eq}
O4B	6445.3(12)	5542.2(12)	2307(2)	43.6(7)
O5B	6327.7(17)	8965.1(15)	8277(4)	83.1(13)
O6B	8230.3(17)	7260(2)	9391(3)	85.3(14)
O7B	8286.9(13)	5429.2(19)	9388(3)	67.9(11)
O8B	8573.3(14)	3771.9(16)	5561(3)	70.5(11)
C9S	9868(4)	6600(4)	9778(9)	104(3)
Cl1	9313.6(12)	6927.2(12)	9585(2)	119.2(8)
Cl2	9845.5(10)	6162.8(12)	10712(3)	119.6(9)
Cl3	10337.4(15)	7046.7(16)	9901(3)	152.3(13)
C3S	5991(2)	5350(3)	6173(5)	57.1(11)
C4S	5823(2)	6060(3)	7205(6)	67.5(15)
O3S	5702.9(17)	5499.2(19)	5543(4)	71.2(11)
O4S	6089.6(16)	5593.8(18)	7042(3)	66.6(11)
C5S	6175(3)	4600(4)	5239(8)	95(3)
O5S	6258.9(16)	4933(2)	6129(4)	77.4(12)
C6S	4799(3)	6782(3)	6287(9)	94.5(18)
C7S	4908(3)	6407(4)	4673(8)	99(2)
C8S	4711(6)	7084(5)	7927(13)	162(6)
O6S	5174(2)	7007(3)	6249(8)	125(3)
O7S	4609(2)	6484(2)	5594(5)	93.9(15)
O8S	4500(3)	6799(3)	7077(7)	120(2)

Table S17. Anisotropic Displacement Parameters (×10⁴) for **Compound 7c**. The anisotropic displacement factor exponent takes the form: $-2\pi^2[h^2a^{*2} \times U_{11} + ... + 2hka^* \times b^* \times U_{12}]$

Atom	U_{11}	U_{22}	U_{33}	U_{23}	U_{13}	U_{12}
C1S	77(4)	71(4)	40.6(18)	0	0	-20(3)
C2S	128(7)	118(6)	120(8)	-24(6)	39(6)	7(5)
O1S	126(6)	122(6)	40.2(18)	0	0	-32(6)
O1W	48(3)	88(4)	29(2)	0	0	-20(3)
O2S	102(3)	99(3)	46(2)	2(2)	3.3(19)	10(3)
Rh1	28.59(19)	42.9(2)	25.8(2)	0	0	-1.8(2)
Rh2	27.07(19)	41.7(2)	25.4(2)	0	0	0.2(2)
C1	29.2(13)	39.2(17)	34.8(10)	-1.6(11)	1.6(9)	-0.6(12)
C2	32.5(14)	47.0(16)	41.8(16)	-4.0(13)	1.1(12)	5.0(12)
C3	43.0(19)	76(2)	46.5(17)	-8.9(15)	7.3(14)	9.0(16)
C4	50(3)	81(3)	57(3)	-2(2)	11(2)	8(2)
C5	62(3)	75(3)	49(3)	-11(2)	0(2)	10(2)
C6	56(2)	124(6)	61(4)	-14(4)	5(2)	36(3)
C7	33.0(13)	45.4(14)	43.1(15)	-6.0(12)	-2.5(12)	4.6(11)
C8	35.4(14)	42.9(14)	41.9(15)	-7.8(12)	-1.4(12)	5.3(11)
C9	40.1(18)	47.1(18)	44.8(16)	-6.3(14)	-5.1(14)	3.0(14)
C10	43.3(16)	48.2(15)	43.7(16)	-6.0(13)	-1.9(13)	6.3(13)
C11	40.7(15)	47.0(15)	43.2(16)	-7.2(12)	1.0(13)	7.9(12)
C12	36.6(13)	43.3(14)	47.0(17)	-6.0(13)	-5.8(12)	2.0(11)
C13	39.6(16)	40.7(13)	45.8(16)	-8.1(12)	-4.9(13)	3.2(12)
C14	43.5(18)	43.2(13)	48(2)	-8.7(13)	-5.4(16)	0.2(13)
C15	42.0(17)	42.5(14)	50.6(17)	-8.4(12)	1.3(14)	4.5(13)
C16	42.1(16)	44.2(14)	51.0(17)	-8.9(13)	1.0(12)	6.8(12)
C17	35.5(14)	42.6(13)	43.6(15)	-8.0(12)	-1.8(12)	5.5(11)
C18	39.6(15)	43.2(14)	43.4(15)	-7.6(12)	1.4(12)	8.2(11)
C19	47.5(15)	45(2)	45.6(16)	-6.0(15)	-4.7(12)	6.5(14)
C20	51(2)	87(4)	49.5(17)	-19(2)	-9.6(15)	22(2)
C21	51.7(16)	96(4)	48(2)	-20(3)	-8.9(14)	20.2(18)
C22	49.5(16)	62(3)	42.3(16)	-12.0(17)	-8.8(12)	12.5(16)

Atom	U_{11}	U_{22}	U_{33}	U_{23}	U_{13}	U_{12}
C23	49.0(18)	52(2)	41.3(16)	-7.1(16)	-5.7(14)	10.8(18)
C24	48.8(15)	59(3)	41.6(18)	-8.9(19)	-5.4(13)	10.3(15)
C25	79(4)	148(8)	58(2)	-40(3)	-21(2)	41(5)
C26	42.2(17)	48.6(18)	46.3(18)	-4.7(14)	-2.0(14)	4.7(14)
C27	44.1(18)	52(2)	48(2)	1.6(17)	2.1(16)	7.2(16)
C28	47(2)	55(2)	52(2)	5.1(17)	-1.8(16)	4.1(16)
C29	48.9(19)	68(2)	53(2)	7.8(18)	-1.2(16)	2.0(16)
C30	52(2)	72(2)	62(3)	9(2)	2.2(19)	5.4(19)
C31	47(2)	63(2)	51(2)	-0.7(17)	2.5(16)	5.7(17)
C32	44.4(17)	48.1(16)	57(2)	-4.0(15)	-3.0(15)	3.1(13)
C33	51(2)	50.7(17)	68(3)	-3.3(17)	-4(2)	7.5(15)
C34	56(2)	57(2)	75(3)	3(2)	-10(2)	0.3(17)
C35	56(2)	56(2)	79(3)	5.3(18)	-7.8(19)	-0.5(16)
C36	53(2)	58(2)	75(3)	8.5(19)	-2.9(19)	-0.7(16)
C37	46.6(18)	47(2)	62(2)	-3.2(17)	0.1(17)	3.8(15)
C38	80(4)	90(4)	134(8)	43(4)	-3(4)	1(3)
C39	47.2(15)	46.0(14)	47(3)	-7.5(16)	4.0(15)	-0.8(12)
C40	55(2)	46.9(14)	72(4)	-11.9(17)	1(2)	1.4(14)
C41	63.5(18)	59(2)	80(4)	-22(2)	9(2)	-9.0(16)
C42	62.4(18)	54.7(17)	81(4)	-20(2)	8(2)	-13.2(15)
C43	58(2)	56.7(17)	66(3)	-17.7(19)	4(2)	-10.7(15)
C44	48.8(15)	45.5(18)	61(3)	-12(2)	-1.4(17)	-0.1(14)
C45	80(3)	93(5)	144(8)	-43(5)	-2(4)	-28(3)
C46	66(2)	78(4)	68(4)	16(3)	-1(2)	6(2)
N1	33.5(12)	42.9(13)	42.0(14)	-6.3(11)	-2.2(11)	3.4(10)
O1	31.8(14)	49.0(17)	34.3(11)	-1.5(12)	2.4(10)	4.8(12)
O2	28.8(13)	47.0(16)	34.1(11)	-1.1(11)	0.4(10)	3.8(12)
O3	42.0(14)	52.5(15)	54(2)	1.7(14)	-11.0(14)	-3.6(12)
O4	45.7(15)	51.9(18)	57.9(17)	0.6(15)	-15.8(14)	-3.6(14)
O5	56.7(18)	88(3)	54.7(17)	-25.6(19)	-18.3(15)	22.2(19)
O6	65(2)	84(2)	71(2)	25(2)	2.7(19)	7.7(18)
O7	73(3)	78(2)	100(3)	26(2)	-8(2)	-6.8(19)
O8	79(2)	76(2)	130(4)	-38(3)	6(3)	-28(2)
C1B	30.4(15)	43.5(15)	31.9(10)	-0.8(10)	-1.2(10)	-0.1(12)
C2B	36.1(14)	45.0(15)	34.5(13)	2.5(12)	-3.5(11)	-3.5(13)
C3B	52.4(19)	55(2)	39.3(16)	5.4(14)	2.8(14)	-10.0(15)
C4B	65(2)	68(3)	50(3)	8(2)	-6(2)	-3(2)
C5B	58(3)	61(2)	47(3)	7(2)	9(2)	-6(2)
C6B	90(4)	72(3)	55(3)	2(3)	6(3)	-35(3)
C7B	46.1(17)	42.1(14)	38.0(13)	-3.0(12)	-6.4(13)	-0.2(13)
C8B	37.7(16)	43.8(14)	34.9(14)	0.3(11)	-1.3(12)	-6.3(12)
C9B	44.1(19)	46.8(17)	39.3(15)	-3.4(13)	-0.6(14)	-6.9(15)
C10B	43.0(16)	48.5(14)	38.4(16)	-2.6(12)	0.6(13)	-10.4(12)
C11B	40.9(15)	51.5(14)	34.2(14)	1.6(12)	4.7(13)	-10.2(12)
C12B	32.5(15)	41.2(12)	36.5(13)	1.4(10)	0.7(12)	-3.4(11)
C13B	33.5(16)	44.5(14)	37.2(13)	1.7(11)	-0.2(12)	-5.0(12)
C14B	39.0(17)	47.9(17)	40.8(16)	1.6(13)	-1.0(14)	-0.8(14)
C15B	37.9(17)	54.3(15)	40.9(15)	3.0(12)	-0.7(13)	-2.8(13)
C16B	37.8(17)	55.1(15)	41.4(14)	2.2(12)	-0.3(13)	-4.0(13)
C17B	35.3(15)	44.1(14)	36.3(13)	2.2(11)	0.3(12)	-7.1(12)
C18B	33.5(15)	51.8(14)	35.1(13)	1.3(11)	1.8(12)	-7.4(12)
C19B	50.2(16)	47.2(15)	36.8(19)	-0.9(14)	-1.9(14)	-7.0(12)
C20B	52.8(19)	48.2(18)	45(2)	-0.4(17)	2.7(16)	-6.1(14)
C21B	69(2)	53.0(17)	54(3)	-6.5(18)	0(2)	-0.1(16)
C22B	71(2)	51.2(17)	45(2)	0.5(16)	-9.3(17)	-0.6(15)
C23B	70(2)	55(2)	47(2)	-5.8(19)	-9.2(18)	-5.2(16)
C24B	56(2)	54.6(17)	49(3)	-5.6(18)	1.3(19)	-13.6(16)
C25B	113(4)	69(3)	92(6)	-18(4)	-19(4)	6(3)

Atom	*U11*	*U22*	*U33*	*U23*	*U13*	*U12*
C26B	51.4(17)	49(2)	38.3(14)	0.4(14)	-1.1(12)	-12.5(16)
C27B	54.4(17)	121(5)	44(2)	-7(3)	-0.6(15)	-29(2)
C28B	65(2)	106(5)	50.0(18)	-3(2)	-9.1(16)	-29(3)
C29B	68(2)	60(3)	46.7(18)	2.4(18)	-13.3(15)	-6.5(19)
C30B	70(2)	82(4)	45.5(19)	2(2)	-9.5(16)	-8(2)
C31B	55(2)	73(3)	38.3(15)	2.6(16)	-0.2(13)	-3(2)
C32B	44.3(15)	52(2)	44.8(15)	3.4(15)	-5.5(12)	-9.2(16)
C33B	44.4(16)	156(7)	57(2)	25(4)	-7.0(15)	-8.5(19)
C34B	50(2)	156(8)	58(2)	22(3)	-11.3(15)	-12(3)
C35B	51.0(17)	61(3)	49.6(17)	0.6(18)	-11.9(13)	-3.1(18)
C36B	51.0(17)	86(4)	51(2)	14(2)	-9.8(15)	-2.6(19)
C37B	42.1(18)	71(3)	47.4(17)	11.5(18)	-5.0(13)	-4(2)
C38B	67(2)	123(6)	64(4)	3(4)	-22(2)	-2(3)
C39B	43.8(16)	55.7(17)	45.0(18)	-2.1(15)	-6.7(14)	0.4(14)
C40B	58(2)	83(3)	58(3)	12(2)	6.7(19)	17(2)
C41B	64(3)	86(3)	64(3)	11(2)	5(2)	22(2)
C42B	46.4(18)	68(2)	52(2)	-3.8(17)	-6.9(16)	11.6(16)
C43B	45.2(17)	55(2)	57(2)	-2.7(17)	-5.4(17)	7.4(16)
C44B	41.0(19)	54.5(18)	49(2)	-0.5(16)	-2.3(16)	5.1(14)
C45B	64(2)	99(5)	68(3)	-6(3)	-6(2)	27(3)
C46B	116(6)	219(14)	72(2)	-1(4)	-36(3)	4(7)
N1B	36.4(14)	40.9(12)	36.1(13)	-1.5(10)	-4.7(11)	-2.4(11)
O1B	38.2(15)	47.3(16)	30.9(11)	0.2(11)	-1.0(11)	-5.3(13)
O2B	35.3(14)	45.0(15)	31.6(11)	-1.2(11)	-0.8(11)	-2.5(12)
O3B	67.1(19)	53.0(15)	52(2)	-12.0(16)	-20.9(18)	14.7(14)
O4B	41.5(16)	46.9(13)	42.5(15)	-4.5(12)	-2.4(13)	1.1(12)
O5B	108(3)	64.8(17)	76(3)	-18.9(19)	-15(2)	10.6(19)
O6B	100(3)	84(3)	72(2)	-8(2)	-39(2)	-6(2)
O7B	61.2(19)	92(3)	50.3(17)	-1.7(18)	-15.1(15)	10.3(19)
O8B	62.2(19)	79(2)	71(3)	-2.1(19)	-6.7(18)	25.4(17)
C9S	101(4)	101(4)	112(5)	3(4)	-5(3)	-5(4)
Cl1	126.5(19)	126.9(19)	104.1(18)	17.3(15)	13.2(15)	13.9(16)
Cl2	96.9(16)	123.3(19)	139(2)	29.5(17)	-2.3(14)	-4.9(13)
Cl3	156(3)	173(3)	128(2)	4(2)	-7(2)	-73(2)
C3S	45(2)	72(2)	54.0(19)	-12.9(15)	1.5(15)	-5.9(16)
C4S	61(3)	73(2)	68(4)	-15(2)	-1(3)	3(2)
O3S	64(2)	78(3)	71(2)	-9(2)	-16.6(19)	-7.8(19)
O4S	60(2)	77(2)	62(2)	-19.2(17)	-12.2(16)	6.5(17)
C5S	73(4)	109(5)	104(4)	-45(4)	4(4)	-13(4)
O5S	60(2)	80(2)	93(3)	-25.7(19)	-9(2)	4.5(18)
C6S	77(3)	71(3)	135(4)	22(3)	-47(2)	-3(2)
C7S	76(4)	98(5)	123(4)	32(3)	-32(3)	-8(4)
C8S	181(12)	117(7)	189(8)	-5(6)	-101(9)	32(7)
O6S	85(3)	92(4)	198(8)	10(4)	-40(3)	-16(2)
O7S	75(3)	84(3)	123(3)	24(3)	-33(2)	-20(2)
O8S	101(4)	114(5)	144(4)	13(3)	-32(3)	-7(3)

Compound 8a

Submitted by: **Yannick Boni**

Solved by: **John Bacsa**

R_1=5.01%

Crystal Data and Experimental

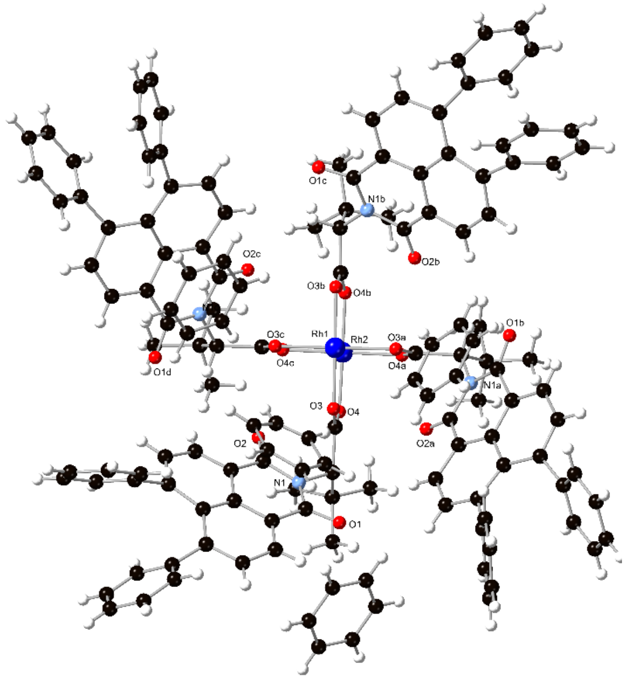

Experimental. Single violet needle-shaped crystals of **compound 8a** were chosen from the sample as supplied. A suiTable Scrystal with dimensions 0.20 × 0.09 × 0.08 mm³ was selected and mounted on a loop with paratone on a XtaLAB Synergy-S diffractometer. The crystal was kept at a steady *T* = 107(5) K during data collection. The structure was solved with the **ShelXT** (Sheldrick, 2015) solution program and by using **Olex2** 1.3-alpha (Dolomanov et al., 2009) as the graphical interface. The model was refined with **ShelXL** 2018/3 (Sheldrick, 2015) using full matrix least squares minimisation on ***F*²**.

Crystal Data. $C_{157}H_{134}N_6O_{16}Rh_2$, M_r = 2566.51, tetragonal, $P4_12_12$ (No. 92), a = 25.1296(2) Å, b = 25.1296(2) Å, c = 46.4882(8) Å, $\alpha = \beta = \gamma =$ 90°, V = 29357.1(8) Å³, T = 107(5) K, Z = 8, Z' = 1, μ(Cu K$_\alpha$) = 2.308, 174589 reflections measured, 22909 unique (R_{int} = 0.0722) which were used in all calculations. The final wR_2 was 0.1285 (all data) and R_1 was 0.0501 (I≥2 σ(I)).

**Compound**	**8a**
Formula	$C_{157}H_{134}N_6O_{16}Rh_2$
$D_{calc.}$/ g cm^{-3}	1.161
μ/mm^{-1}	2.308
Formula Weight	2566.51
Colour	violet
Shape	needle-shaped
Size/mm³	0.20×0.09×0.08
T/K	107(5)
Crystal System	tetragonal
Flack Parameter	-0.010(3)
Hooft Parameter	0.010(2)
Space Group	$P4_12_12$
a/Å	25.1296(2)
b/Å	25.1296(2)
c/Å	46.4882(8)
α/°	90
β/°	90
γ/°	90
V/Å³	29357.1(8)
Z	8
Z'	1
Wavelength/Å	1.54184
Radiation type	Cu K$_\alpha$
Θ_{min}/°	1.998
Θ_{max}/°	63.116
Measured Refl's.	174589
Indep't Refl's	22909
Refl's I≥2 σ(I)	16897
R_{int}	0.0722
Parameters	1508
Restraints	1469
Largest Peak	0.621
Deepest Hole	-0.399
GooF	1.042
wR_2 (all data)	0.1285
wR_2	0.1200
R_1 (all data)	0.0730
R_1	0.0501

Structure Quality Indicators

**Reflections:**	d min (Cu\a) 2θ=126.2° 0.86	I/σ(I) 17.2	Rint 7.22%	CAP 88.9° 97% to 126.2° 99.8	
**Refinement:**	Shift -0.004	Max Peak 0.6	Min Peak -0.4	GooF 1.042	Flack -.010(3)

A violet needle-shaped-shaped crystal with dimensions 0.20 × 0.09 × 0.08 mm^3 was mounted on a loop with paratone. Data were collected using a XtaLAB Synergy, Dualflex, HyPix diffractometer equipped with an Oxford Cryosystems low-temperature device operating at T = 107(5) K.

Data were measured using ω scans using Cu K_α radiation. The diffraction pattern was indexed and the total number of runs and images was based on the strategy calculation from the program CrysAlisPro 1.171.41.98a (Rigaku OD, 2021). The maximum resolution that was achieved was Θ = 63.116° (0.86 Å).

The unit cell was refined using CrysAlisPro 1.171.41.98a (Rigaku OD, 2021) on 15981 reflections, 9% of the observed reflections.

Data reduction, scaling and absorption corrections were performed using CrysAlisPro 1.171.41.98a (Rigaku OD, 2021). The final completeness is 97.10 % out to 63.116° in Θ. A numerical absorption correction based on gaussian integration over a multifaceted crystal model was performed using CrysAlisPro 1.171.41.108a (Rigaku Oxford Diffraction, 2021). An empirical absorption correction using spherical harmonics, implemented in SCALE3 ABSPACK scaling algorithm was also applied. The absorption coefficient μ of this material is 2.308 mm^{-1} at this wavelength (λ = 1.54184Å) and the minimum and maximum transmissions are 0.744 and 1.000.

The structure was solved and the space group $P4_12_12$ (# 92) determined with the **ShelXT** (Sheldrick, 2015) and refined by full matrix least squares minimisation on $\boldsymbol{F^2}$ using version 2018/3 of **ShelXL** 2018/3 (Sheldrick, 2015). All non-hydrogen atoms were refined anisotropically. Hydrogen atom positions were calculated geometrically and refined using the riding model.

A solvent mask was calculated and 1148 electrons were found in a volume of 12247$Å^3$ in 1 void per unit cell. This is consistent with the presence of 4[C_6H_6] per Asymmetric Unit which account for 1344 electrons per unit cell.

There is a single molecule in the asymmetric unit, which is represented by the reported sum formula. In other words: Z is 8 and Z' is 1.

The Flack parameter was refined to -0.010(3). Determination of absolute structure using Bayesian statistics on Bijvoet differences using the Olex2 results in 0.010(2). Note: The Flack parameter is used to determine chirality of the crystal studied, the value should be near 0, a value of 1 means that the stereochemistry is wrong and the model should be inverted. A value of 0.5 means that the crystal consists of a racemic mixture of the two enantiomers.

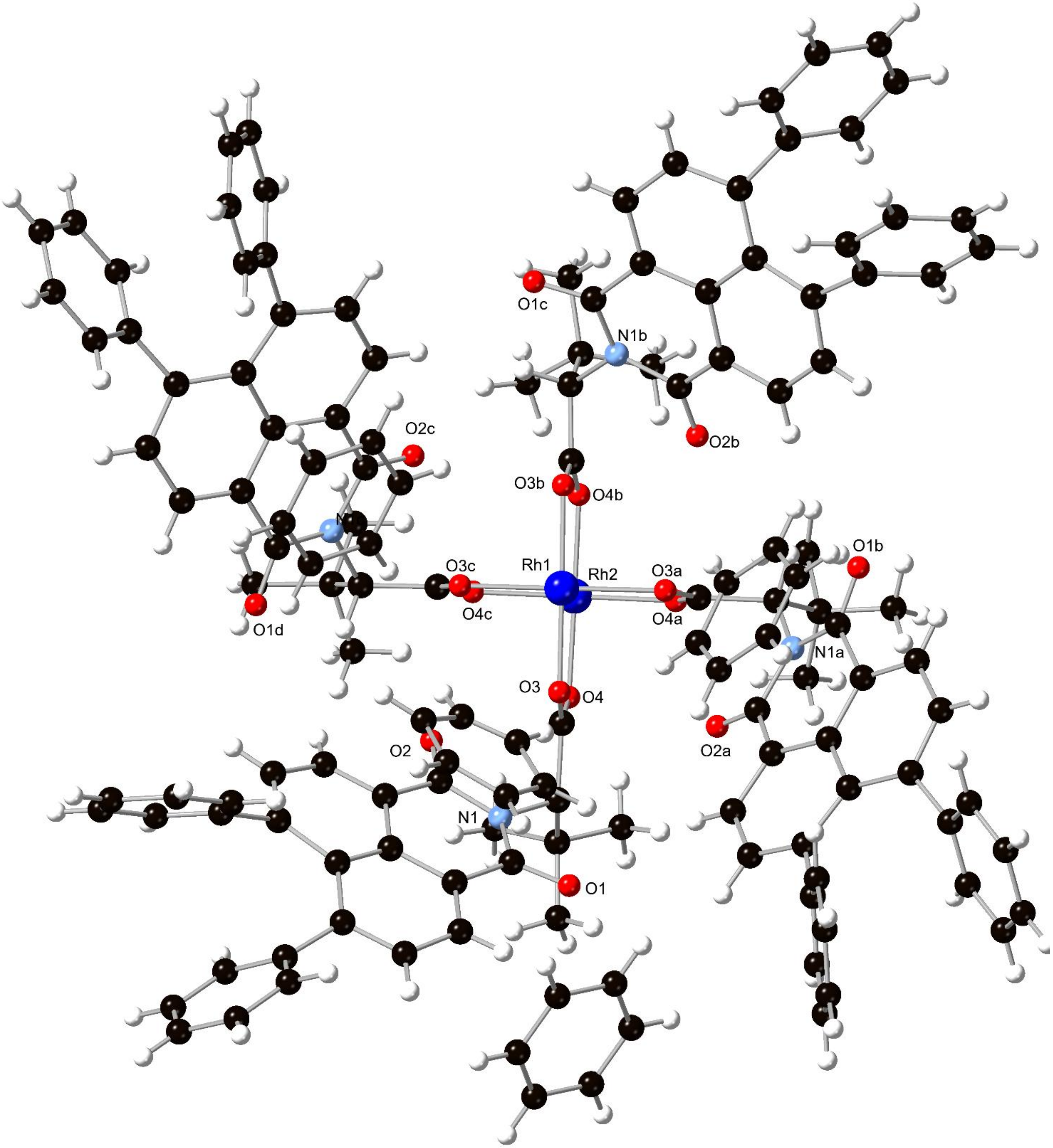

Figure S307. Ball-and-stick representation of molecular structure and one benzene molecule showing the orientation of the substituents. There are six benzene molecule and 1 catalyst molecule in the asymmetric unit, which is represented by the reported sum formula.

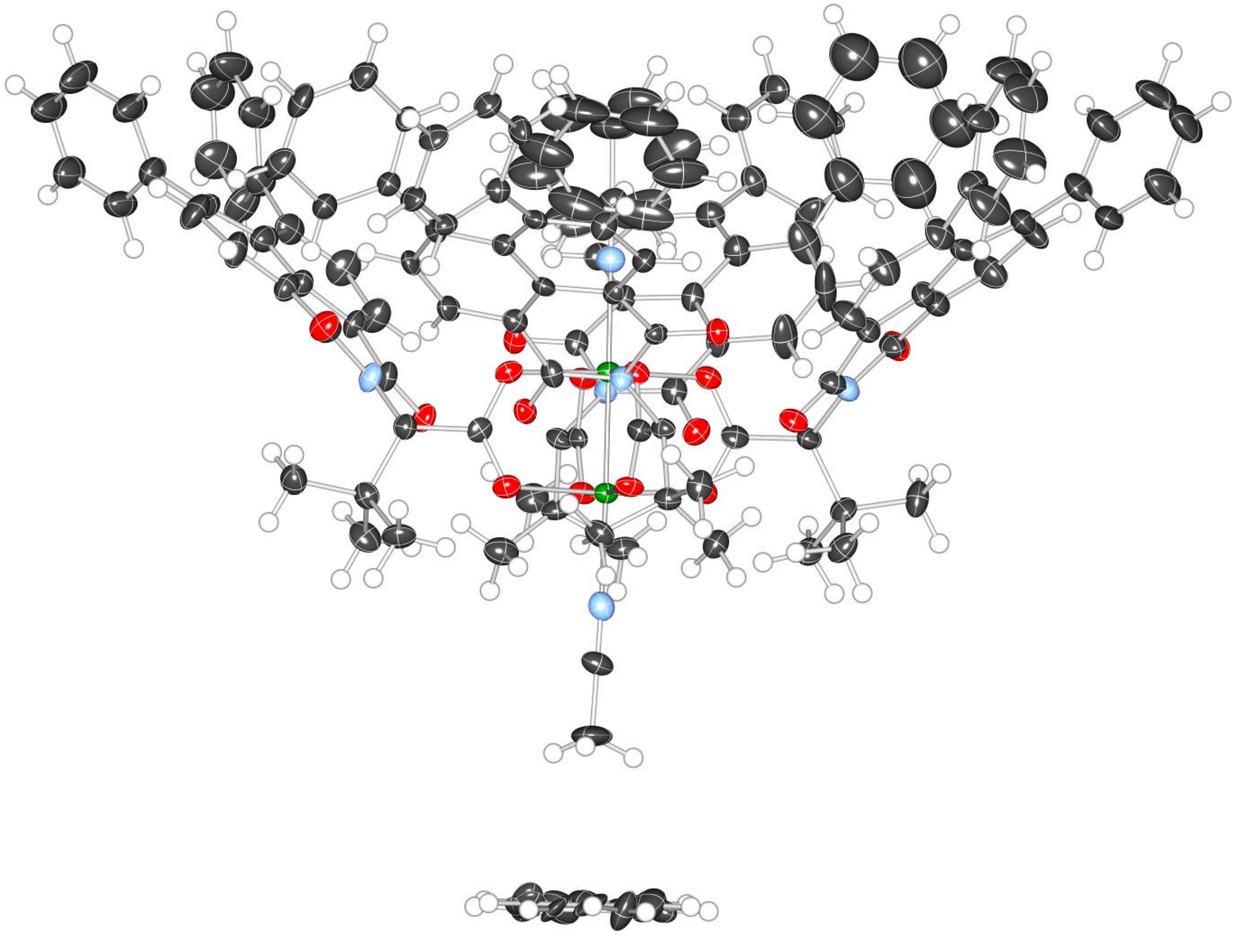

Figure S308. A thermal ellipsoid representation of molecular structure showing the encapsulation of the benzene molecule by the catalyst molecule and a benzene interacting with the bound acetonitrile on the opposite side of the cavity.

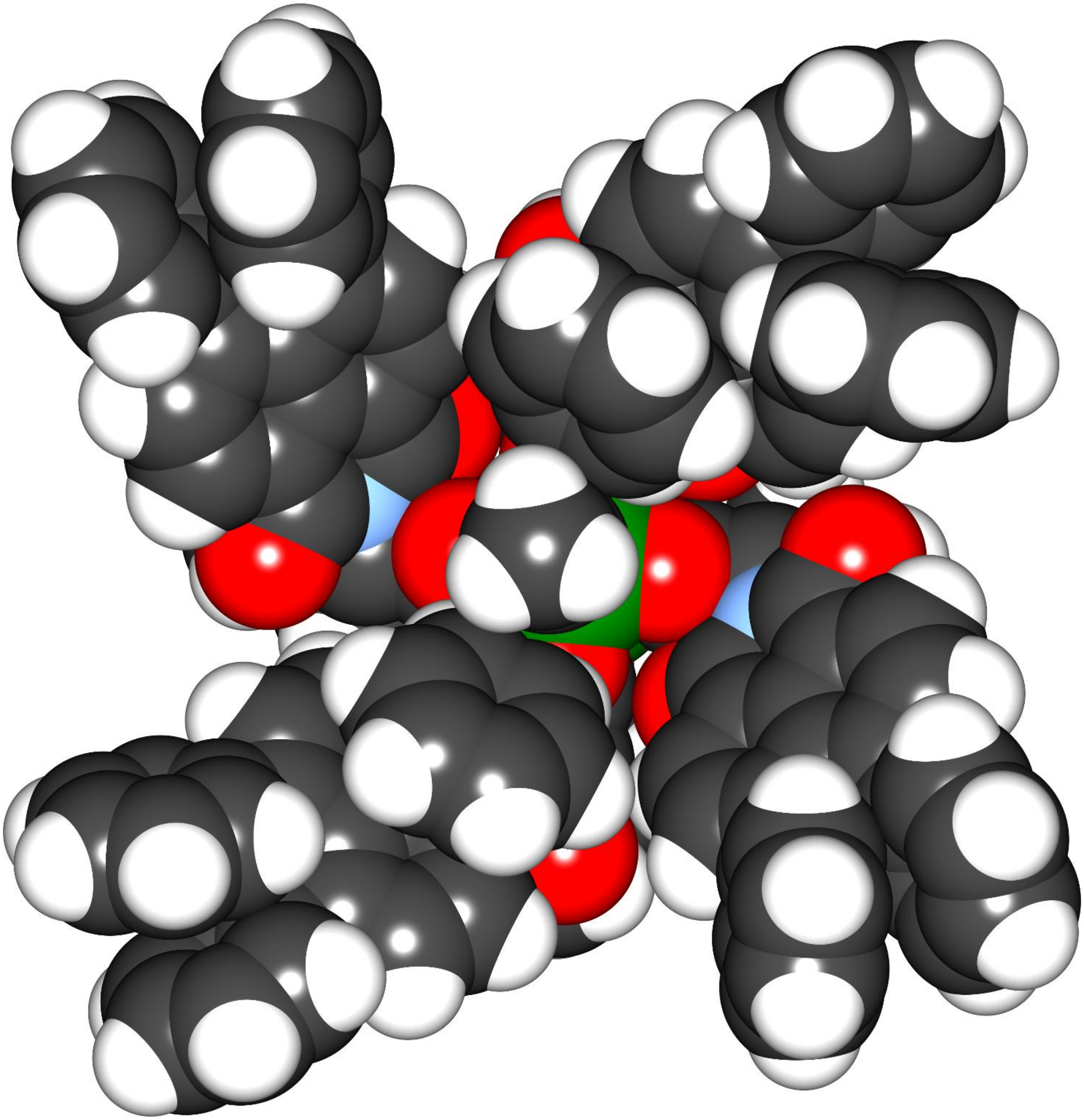

Figure S309. A space-filling representation of molecular structure and one benzene guest molecule showing the large bowl shaped cavity.

Data Plots: Diffraction Data

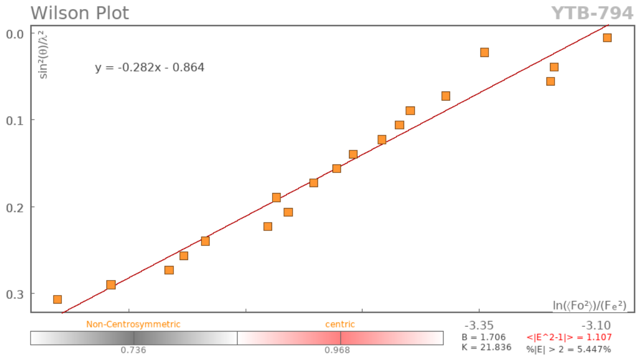

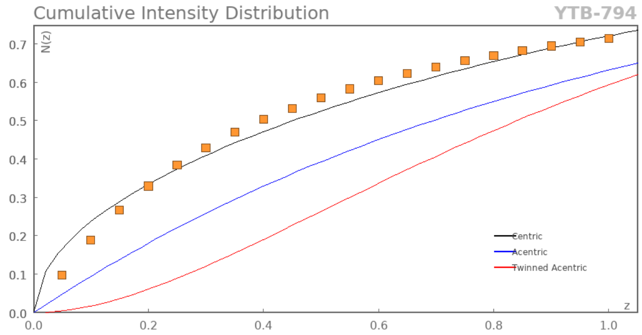

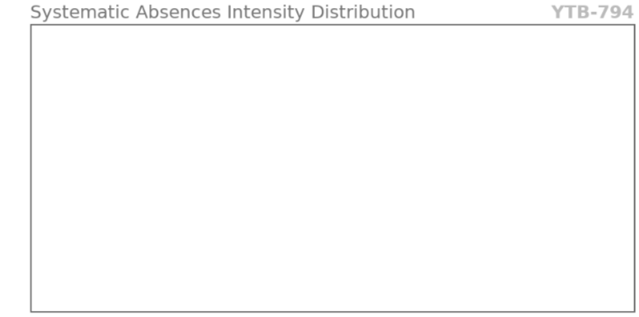

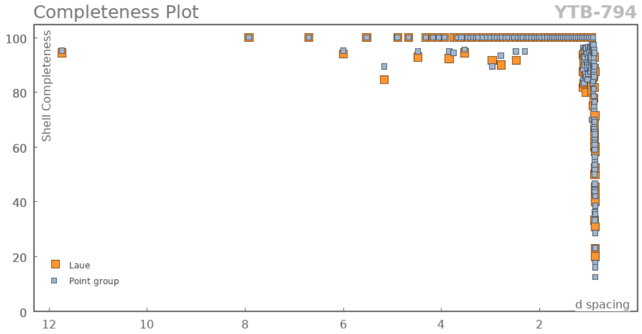

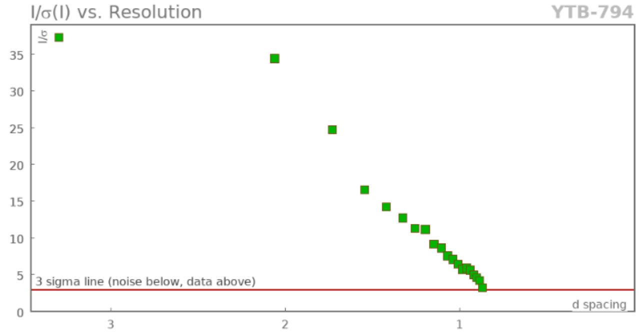

Data Plots: Refinement and Data

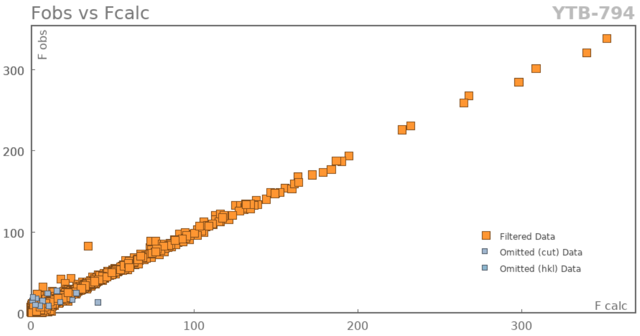

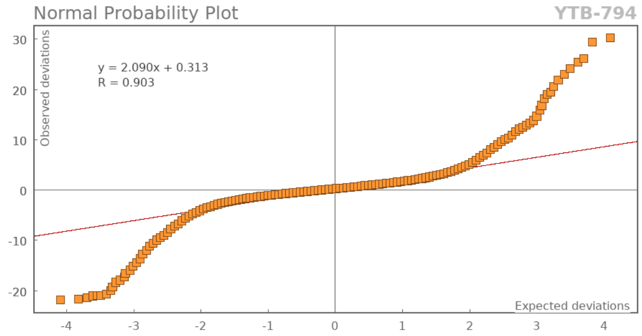

Images of the Crystal on the Diffractometer

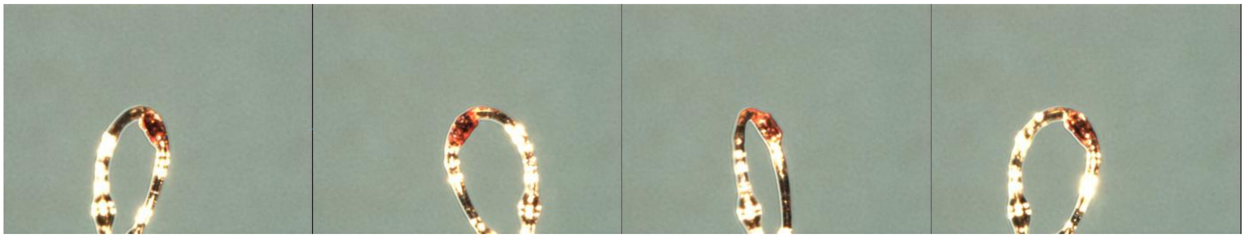

Table S18: Fractional Atomic Coordinates (×10^4) and Equivalent Isotropic Displacement Parameters (Å^2×10^3) for **compound 8a.** U_{eq} is defined as 1/3 of the trace of the orthogonalised U_{ij}.

Atom	x	y	z	U_{eq}
O1	1753.2(16)	5278.8(17)	5083.9(9)	30.0(11)
O2	3189.0(16)	6124.6(16)	5480.3(9)	30.9(10)
O3	2285.8(16)	6742.3(15)	5254.9(9)	25.6(9)
O4	2287.9(16)	6784.7(15)	5739.8(8)	25.3(10)
N1	2467.8(17)	5673.1(17)	5307.5(10)	24.0(10)
C1	2107(2)	5954(2)	5514.0(14)	24.3(11)
C2	2244(2)	6545(2)	5506.5(13)	23.1(12)
C3	2070(3)	5673(2)	5811.4(14)	28.8(12)
C4	1890(3)	5093(2)	5768.4(14)	35.8(17)
C5	1646(3)	5947(3)	5989.2(14)	34.5(16)
C6	2603(3)	5662(2)	5974.8(13)	30.5(15)
C7	3006.7(18)	5817(2)	5304.9(14)	24.4(12)
C8	2245(2)	5334(2)	5102.7(13)	23.5(12)
C9	2600(3)	5033(2)	4909.5(13)	26.5(12)
C10	3161(2)	5150(2)	4899.3(13)	23.5(11)
C11	3352.6(19)	5557(2)	5090.8(14)	29.4(13)
C15	3498.7(15)	4903.5(18)	4710.6(13)	38.2(14)
C16	3300.0(16)	4444.1(19)	4557.4(12)	29.9(13)
C17	2762.1(17)	4341(2)	4566.7(13)	28.4(13)
C18	2418(2)	4652(2)	4735.0(13)	29.6(13)
C25	3635(2)	4036(2)	4412.3(13)	33.5(14)
C26	4081(2)	3817(2)	4550.4(14)	42.7(17)
C27	4375(3)	3417(2)	4430.6(14)	51(2)
C28	4230(2)	3214(2)	4160.7(13)	34.0(15)
C29	3797(2)	3418(2)	4019.3(14)	39.2(17)
C30	3505(2)	3825(2)	4145.9(14)	35.8(16)
C12A	3891(2)	5668(6)	5128(2)	32.4(15)
C13A	4220(4)	5464(4)	4916.9(19)	48(2)
C14A	4040.5(18)	5097(4)	4715.4(17)	46.4(16)
C19A	4454(3)	5003(5)	4494.0(19)	50.0(17)
C20A	4958(4)	4847(6)	4583(2)	53.0(15)
C21A	5355(3)	4750(7)	4381(3)	58(2)
C22A	5247(5)	4810(7)	4090(3)	55(2)
C23A	4742(5)	4965(6)	4000.5(17)	54.1(19)
C24A	4346(4)	5062(6)	4203(2)	53.0(15)
C12B	3871.8(19)	5731(3)	5046.6(17)	32.4(15)
C13B	4189(3)	5512(3)	4835.2(16)	48(2)
C14B	4016.9(19)	5121(3)	4644.6(13)	46.4(16)
C19B	4338(2)	5031(3)	4381.6(11)	50.0(17)
C20B	4887(2)	4975(3)	4397.3(12)	53.0(15)
C21B	5191.3(18)	4989(3)	4147.6(15)	58(2)
C22B	4946(3)	5058(3)	3882.3(12)	55(2)
C23B	4396(3)	5113(3)	3866.6(11)	54.1(19)
C24B	4092.2(19)	5100(3)	4116.2(14)	53.0(15)
Rh1	2492.1(2)	7528.4(2)	5225.9(2)	21.33(12)
Rh2	2468.7(2)	7560.5(2)	5741.6(2)	20.97(12)

Atom	x	y	z	U_{eq}
C38_4	2359(3)	7763(3)	6773.9(14)	52(2)
N2_4	2431.1(19)	7635.0(18)	6225.6(11)	28.5(11)
C37_4	2403(3)	7688(2)	6463.1(14)	32.1(15)
O1_1	252.4(17)	8304.1(17)	5033.2(10)	34.6(11)
O2_1	1041.3(16)	6844.7(16)	5418.3(9)	29.1(10)
O3_1	1702.0(16)	7735.2(15)	5227.0(9)	24.2(8)
O4_1	1685.3(16)	7741.4(16)	5711.8(9)	27.2(9)
N1_1	613.6(19)	7577.1(17)	5246.4(11)	27.9(11)
C1_1	879(2)	7930(2)	5462.9(13)	24.9(11)
C2_1	1477(2)	7785(2)	5468.3(13)	20.4(11)
C3_1	577(3)	7977(2)	5747.2(14)	29.1(12)
C4_1	12(3)	8153(3)	5687.3(15)	40.5(18)
C5_1	835(3)	8417(3)	5925.7(15)	37.4(17)
C6_1	564(3)	7471(3)	5924.5(14)	35.4(16)
C7_1	743(2)	7034.7(19)	5245.6(14)	27.5(13)
C8_1	284(2)	7826(3)	5042.6(14)	28.9(13)
C9_1	-53(2)	7458(3)	4866.8(14)	32.8(12)
C10_1	66(3)	6917(3)	4849.0(14)	32.4(12)
C11_1	494(2)	6708.8(19)	5016.2(15)	29.6(12)
C12_1	657(2)	6183.0(18)	4983.7(14)	42.8(16)
C13_1	394(2)	5860(2)	4779.2(12)	42.5(16)
C14_1	-25(2)	6040.0(17)	4615.0(11)	38.6(12)
C15_1	-227.1(18)	6572.2(16)	4660.0(13)	33.2(13)
C16_1	-687(2)	6801.6(18)	4520.7(13)	39.4(12)
C17_1	-774(2)	7341.3(18)	4541.0(14)	45.7(17)
C18_1	-454(2)	7683(3)	4709.1(13)	40.7(15)
C19_1	-237.5(19)	5644(2)	4408.8(13)	42.8(15)
C20_1	-401(2)	5147(3)	4513.2(16)	54.0(18)
C21_1	-603(2)	4767(3)	4321.5(17)	64(2)
C22_1	-639(2)	4883(3)	4038.1(18)	68(2)
C23_1	-485(2)	5357(3)	3931.2(18)	70(2)
C24_1	-281(2)	5746(3)	4116.1(16)	53.2(18)
C25_1	-1122(2)	6498(3)	4374.1(15)	47.8(17)
C26_1	-1370(2)	6077(3)	4507.9(15)	45.4(16)
C27_1	-1767(3)	5794(3)	4375.8(15)	51.9(19)
C28_1	-1922(3)	5932(3)	4102.7(16)	60(2)
C29_1	-1678(3)	6353(3)	3965.4(17)	73(3)
C30_1	-1274(3)	6647(3)	4095.2(16)	54(2)
C28_2	1112(2)	12204(2)	4230.2(13)	35.0(16)
O1_2	3351.5(15)	9765.0(15)	5053.3(9)	24.8(10)
C8_2	2863(2)	9763(2)	5063.0(12)	21.7(12)
N1_2	2597.3(19)	9420.2(18)	5251.9(10)	23.5(10)
C9_2	2537(3)	10120(2)	4886.9(12)	23.8(11)
C1_2	2922(2)	9119(2)	5466.1(13)	23.3(11)
C7_2	2049(2)	9312(2)	5232.3(13)	23.1(12)
C10_2	1976(2)	10044(2)	4860.4(13)	24.0(12)
C18_2	2782(3)	10545(2)	4750.4(14)	29.9(14)
C2_2	2744(2)	8541(2)	5473.7(14)	24.7(12)
C3_2	2962(3)	9410(2)	5762.6(14)	27.5(12)
O2_2	1829.8(16)	8995.8(16)	5392.6(9)	26.9(10)
C11_2	1748(2)	9587(2)	5009.8(14)	23.5(12)
C15_2	1662.8(16)	10375.3(18)	4683.2(13)	23.2(12)
C17_2	2460(2)	10908(2)	4601.6(12)	25.3(12)
O3_2	2709.9(16)	8311.3(15)	5226.9(8)	23.6(9)
O4_2	2654.9(16)	8334.9(15)	5714.7(8)	24.9(9)
C4_2	3206(2)	9956(2)	5708.1(13)	29.0(15)
C5_2	3339(3)	9104(2)	5961.5(13)	29.2(15)
C6_2	2416(3)	9487(2)	5912.7(14)	30.4(15)

Atom	x	y	z	U_{eq}
C12_2	1227(2)	9460(3)	4959.1(14)	28.0(13)
C14_2	1120.9(19)	10205(2)	4627.8(13)	25.9(12)
C16_2	1912(2)	10857(2)	4575.8(12)	24.0(12)
C13_2	926(2)	9755(2)	4762.6(13)	26.5(13)
C19_2	749(2)	10464.3(19)	4423.4(13)	30.4(13)
C25_2	1633(2)	11324(2)	4452.4(12)	26.0(12)
C20_2	242(2)	10616(2)	4522.3(15)	34.7(15)
C24_2	893(2)	10612.5(19)	4143.1(13)	29.3(13)
C26_2	1198(2)	11553(2)	4598.5(11)	21.5(12)
C30_2	1799(2)	11545(2)	4195.1(12)	29.7(14)
C21_2	-99(3)	10906(2)	4342.6(15)	40.8(16)
C23_2	559(2)	10900(2)	3963.3(15)	37.0(15)
C27_2	946(2)	11992(2)	4482.4(13)	31.4(14)
C29_2	1536(2)	11985(2)	4085.5(13)	31.4(14)
C22_2	55(3)	11043(2)	4072.5(15)	40.4(16)
O1_3	4757.6(18)	6726.7(15)	5112.3(9)	36.2(12)
C8_3	4726(2)	7208.3(18)	5110.1(12)	28.1(13)
N1_3	4379.5(18)	7472(2)	5296.4(10)	25.6(10)
C9_3	5084(2)	7535(2)	4932.2(12)	33.1(13)
C1_3	4075(2)	7151(3)	5510.0(14)	27.3(12)
C7_3	4258(2)	8020(2)	5266.8(11)	27.1(13)
C10_3	4980(3)	8076(3)	4899.2(14)	30.4(13)
C18_3	5494(3)	7314(2)	4796.1(14)	45.5(18)
C2_3	3488(2)	7305(2)	5504.4(14)	24.0(12)
C3_3	4349(3)	7135(3)	5813.4(14)	28.5(12)
O2_3	3950.0(16)	8233.6(15)	5431.5(8)	27.4(10)
C11_3	4546(2)	8313(2)	5041.0(13)	36.4(16)
C15_3	5294(3)	8401(3)	4700.8(14)	31.3(13)
C17_3	5845(3)	7620(3)	4635.7(16)	49.3(18)
O3_3	3274.9(16)	7330.6(16)	5250.7(9)	28.1(10)
O4_3	3251.1(15)	7372.6(15)	5735.5(9)	26.7(10)
C4_3	4930(3)	6961(3)	5773.9(15)	39.9(18)
C5_3	4077(3)	6695(3)	5988.5(15)	38.5(18)
C6_3	4341(3)	7662(2)	5976.0(14)	32.4(16)
C12_3	4377(3)	8832(3)	4986.5(16)	44.4(18)
C14_3	5111(3)	8926(3)	4640.4(15)	37.8(15)
C16_3	5758(3)	8158(2)	4589.6(14)	40.9(15)
C13_3	4663(3)	9120(3)	4779.4(15)	43.1(17)
C19_3	5343(2)	9305(3)	4423.4(16)	49.9(18)
C25_3	6209(3)	8438(3)	4448.0(15)	50.8(19)
C20_3	5494(2)	9811(3)	4514.5(18)	67(2)
C24_3	5419(2)	9169(3)	4137.3(17)	57(2)
C26_3	6451(2)	8879(3)	4570.3(15)	47.7(19)
C30_3	6403(3)	8264(3)	4174.7(15)	53(2)
C21_3	5718(3)	10183(4)	4325(2)	86(3)
C23_3	5642(3)	9538(4)	3948.6(19)	74(3)
C27_3	6865(3)	9138(3)	4431.3(16)	59(2)
C29_3	6803(3)	8522(3)	4047.2(17)	68(2)
C22_3	5779(3)	10018(4)	4047(2)	79(3)
C28_3	7046(3)	8966(3)	4170.6(16)	58(2)
C38_5	2526(3)	7504(3)	4187.7(15)	55(2)
N2_5	2509.5(19)	7514.2(18)	4747.1(13)	33.3(13)
C37_5	2520(2)	7513(2)	4504.1(17)	38.0(16)
C34_7	3966(8)	7112(9)	4547(4)	82.6(19)
C31_7	4466(10)	7594(9)	4115(5)	82.6(19)
C32_7	4086(9)	7861(9)	4300(5)	82.6(19)
C36_7	4495(9)	7034(9)	4164(5)	82.6(19)
C33_7	3873(10)	7568(9)	4515(5)	82.6(19)

Atom	x	y	z	U_{eq}
C35_7	4273(9)	6810(9)	4388(5)	82.6(19)
C35_8	4311(6)	7070(4)	4411(4)	82.6(19)
C36_8	4571(6)	7286(6)	4174(4)	82.6(19)
C31_8	4455(7)	7801(6)	4085(3)	82.6(19)
C32_8	4078(7)	8100(5)	4233(3)	82.6(19)
C33_8	3818(6)	7884(6)	4469(3)	82.6(19)
C34_8	3935(6)	7369(6)	4558(3)	82.6(19)
C33_9	2883(7)	8900(7)	4556(4)	50.8(16)
C31_9	2096(7)	9061(7)	4314(4)	50.8(16)
C32_9	2356(7)	8835(7)	4530(4)	50.8(16)
C36_9	2443(7)	9435(7)	4123(4)	50.8(16)
C35_9	2908(7)	9407(7)	4156(4)	50.8(16)
C34_9	3160(7)	9185(7)	4393(4)	50.8(16)
C33_10	2151(4)	8798(5)	4488(3)	50.8(16)
C32_10	2683(5)	8892(5)	4552(2)	50.8(16)
C31_10	2976(4)	9250(5)	4388(3)	50.8(16)
C36_10	2736(5)	9514(5)	4159(3)	50.8(16)
C35_10	2204(5)	9420(5)	4095(3)	50.8(16)
C34_10	1912(4)	9062(5)	4260(3)	50.8(16)
C31_11	803(6)	8178(4)	4369(3)	71.0(19)
C32_11	1131(6)	7841(5)	4526(3)	71.0(19)
C33_11	1199(6)	7315(5)	4440(3)	71.0(19)
C34_11	939(7)	7127(4)	4197(3)	71.0(19)
C35_11	611(6)	7463(5)	4039(3)	71.0(19)
C36_11	543(6)	7989(5)	4125(3)	71.0(19)
C31_12	725(6)	7960(4)	4358(3)	71.0(19)
C32_12	1109(6)	7658(5)	4498(3)	71.0(19)
C33_12	1216(6)	7143(5)	4407(3)	71.0(19)
C34_12	941(7)	6929(5)	4175(3)	71.0(19)
C35_12	557(6)	7231(5)	4034(3)	71.0(19)
C36_12	449(5)	7747(5)	4126(3)	71.0(19)
C36_13	1833(7)	5908(6)	4367(4)	52.3(14)
C31_13	2111(7)	5713(6)	4135(4)	52.3(14)
C32_13	2587(7)	5747(5)	4100(4)	52.3(14)
C33_13	2900(7)	6022(5)	4325(4)	52.3(14)
C35_13	2132(7)	6163(6)	4574(4)	52.3(14)
C34_13	2672(8)	6220(6)	4552(5)	52.3(14)
C36_14	2041(7)	5891(8)	4369(5)	52.3(14)
C31_14	2258(8)	5696(8)	4110(5)	52.3(14)
C32_14	2780(7)	5821(7)	4075(4)	52.3(14)
C33_14	3079(7)	6109(7)	4258(4)	52.3(14)
C35_14	2373(8)	6186(7)	4563(4)	52.3(14)
C34_14	2846(7)	6252(6)	4517(5)	52.3(14)
C33_15	1436(5)	3525(5)	4421(4)	55.8(18)
C32_15	1619(4)	3792(6)	4179(3)	55.8(18)
C31_15	1428(5)	4297(7)	4115(3)	55.8(18)
C36_15	1053(5)	4536(6)	4293(3)	55.8(18)
C35_15	871(5)	4269(5)	4536(3)	55.8(18)
C34_15	1062(5)	3764(5)	4600(3)	55.8(18)
C33_16	1478(13)	3611(12)	4328(12)	55.8(18)
C32_16	1677(13)	3917(16)	4104(9)	55.8(18)
C31_16	1518(14)	4444(18)	4073(7)	55.8(18)
C36_16	1159(15)	4664(15)	4267(7)	55.8(18)
C35_16	959(14)	4358(13)	4491(7)	55.8(18)
C34_16	1119(13)	3832(12)	4522(10)	55.8(18)
C33_6	1860(6)	7932(5)	7490(2)	39.9(17)
C31_6	2310(6)	7094(8)	7489(2)	39.9(17)
C32_6	1853(7)	7389(6)	7485.7(17)	39.9(17)

Atom	x	y	z	U_{eq}
C36_6	2798(5)	7368(5)	7496(3)	39.9(17)
C35_6	2840(8)	7910(6)	7501(2)	39.9(17)
C34_6	2336(6)	8191(7)	7497(2)	39.9(17)

Table S19: Anisotropic Displacement Parameters (×10[4]) for **compound 8a**. The anisotropic displacement factor exponent takes the form: $-2\pi^2[h^2a^{*2} \times U_{11} + ... +2hka^* \times b^* \times U_{12}]$

Atom	U_{11}	U_{22}	U_{33}	U_{23}	U_{13}	U_{12}
O1	25.3(15)	30(3)	35(3)	-5(2)	-2.3(14)	0.7(15)
O2	32(2)	25(2)	35(2)	-0.9(16)	-3.2(18)	-2.1(18)
O3	31(2)	24(2)	22.1(16)	-0.9(14)	1.6(15)	0.3(19)
O4	34(2)	20.6(19)	21.5(17)	-0.1(14)	4.3(16)	3.0(18)
N1	22.9(16)	21.0(19)	28(2)	-2.8(15)	-0.4(13)	2.2(14)
C1	27(2)	21.4(17)	25(2)	0.8(12)	-0.3(16)	6.2(16)
C2	25(3)	21.6(17)	22.2(16)	-1.1(11)	3.4(15)	6.1(17)
C3	38(3)	24(2)	24(2)	0.6(15)	-2.9(16)	0.5(18)
C4	54(4)	25(2)	28(4)	1(2)	-5(3)	-4(2)
C5	43(3)	32(3)	29(3)	-1(3)	0(2)	4(3)
C6	40(3)	26(3)	26(3)	1(3)	-5(2)	1(2)
C7	22.9(16)	19(2)	32(2)	2.4(18)	-2.3(13)	2.6(14)
C8	25.3(15)	18(2)	27(2)	0.8(18)	-2.5(13)	1.3(14)
C9	26.7(16)	20(2)	33(3)	-1.2(18)	0.8(15)	0.2(15)
C10	26.5(16)	15(2)	29(3)	5.1(17)	-1.2(14)	1.0(15)
C11	24.7(18)	24(2)	39(2)	-4.7(19)	1.1(16)	-0.8(16)
C15	30.4(18)	35(2)	50(3)	-15(2)	6.2(17)	-4.9(16)
C16	32.5(19)	26(2)	31(3)	-2(2)	-1.0(18)	-1.5(14)
C17	32.7(19)	25(3)	27(3)	-1(2)	-2.3(18)	-2.0(16)
C18	35(2)	22(2)	32(3)	-1.0(19)	3(2)	-5.2(19)
C25	38(3)	30(3)	32(3)	-1.7(19)	-0.2(18)	3(2)
C26	47(3)	51(4)	30(3)	-10(2)	-3(2)	19(3)
C27	49(4)	63(4)	40(3)	-21(3)	-12(3)	26(3)
C28	38(3)	34(3)	30(3)	-5(2)	-1(2)	11(3)
C29	42(3)	43(3)	32(3)	-8(2)	-5(2)	16(3)
C30	36(3)	38(3)	33(3)	-6(2)	-1(2)	10(3)
C12A	24.9(18)	27(4)	45(3)	-5(2)	1.1(17)	-1.6(18)
C13A	27(3)	57(4)	61(4)	-26(3)	6(2)	-7(3)
C14A	33.6(18)	52(4)	53(3)	-19(2)	8.4(17)	-12(2)
C19A	42(2)	48(4)	60(3)	-22(2)	16.0(19)	-16(2)
C20A	42(2)	58(4)	59(3)	-22(2)	16.4(18)	-14(2)
C21A	46(2)	68(6)	61(3)	-18(3)	19.0(19)	-9(3)
C22A	45(2)	59(6)	61(3)	-18(3)	19.2(19)	-11(3)
C23A	45(2)	56(5)	61(3)	-19(3)	18.6(19)	-12(3)
C24A	42(2)	58(4)	59(3)	-22(2)	16.4(18)	-14(2)
C12B	24.9(18)	27(4)	45(3)	-5(2)	1.1(17)	-1.6(18)
C13B	27(3)	57(4)	61(4)	-26(3)	6(2)	-7(3)
C14B	33.6(18)	52(4)	53(3)	-19(2)	8.4(17)	-12(2)
C19B	42(2)	48(4)	60(3)	-22(2)	16.0(19)	-16(2)
C20B	42(2)	58(4)	59(3)	-22(2)	16.4(18)	-14(2)
C21B	46(2)	68(6)	61(3)	-18(3)	19.0(19)	-9(3)
C22B	45(2)	59(6)	61(3)	-18(3)	19.2(19)	-11(3)
C23B	45(2)	56(5)	61(3)	-19(3)	18.6(19)	-12(3)
C24B	42(2)	58(4)	59(3)	-22(2)	16.4(18)	-14(2)
Rh1	26.9(3)	18.2(2)	18.9(2)	0.11(17)	0.16(18)	-0.7(3)
Rh2	25.8(3)	18.3(2)	18.8(2)	-0.24(18)	0.89(18)	-1.5(3)
C38_4	51(5)	79(5)	27.8(19)	-3(2)	6(2)	-10(4)

Atom	U_{11}	U_{22}	U_{33}	U_{23}	U_{13}	U_{12}
N2_4	34(3)	25(3)	26.9(18)	4.4(18)	3.6(19)	-3(2)
C37_4	38(4)	31(3)	26.9(18)	3.7(18)	4.2(19)	-5(3)
O1_1	44(3)	28.9(14)	31(3)	1.8(15)	-3(2)	7.5(15)
O2_1	26(2)	23(2)	38(2)	-3.5(17)	-8.8(16)	5.6(18)
O3_1	30.1(18)	18(2)	24.9(15)	-0.7(15)	-2.9(12)	2.2(17)
O4_1	30.6(18)	26(2)	24.9(15)	-4.3(15)	-4.7(13)	5.2(18)
N1_1	28(2)	24.0(17)	31(2)	-3.3(14)	-7.4(18)	7.0(16)
C1_1	27.9(16)	21(3)	26(2)	1.6(18)	-2.9(13)	0.9(16)
C2_1	27.0(16)	9(3)	24.8(15)	-2.1(14)	-3.8(11)	-2.3(16)
C3_1	34(2)	24(2)	29(2)	-0.7(17)	1.2(15)	-1.8(19)
C4_1	37(2)	47(4)	38(4)	-6(3)	-2(2)	7(2)
C5_1	39(4)	34(3)	40(4)	-10(3)	2(3)	-5(3)
C6_1	40(4)	28(3)	39(4)	5(2)	9(3)	3(2)
C7_1	23(3)	23.8(16)	36(2)	-2.7(14)	-5.6(19)	5.7(15)
C8_1	25(2)	29.0(14)	33(3)	1.7(14)	-5(2)	6.0(14)
C9_1	28(2)	38.8(13)	32(3)	0.7(14)	-1.6(18)	-2.3(13)
C10_1	28(2)	38.7(13)	31(2)	-0.3(13)	-0.2(18)	-3.6(13)
C11_1	27(2)	31.4(16)	31(2)	-1.3(15)	0.5(18)	-5.2(15)
C12_1	42(3)	33.5(17)	52(3)	-8.1(17)	-10(2)	0.1(17)
C13_1	41(3)	34.6(18)	52(3)	-8.8(17)	-8(2)	-1.7(17)
C14_1	33(2)	41.4(14)	41(2)	-4.4(15)	0.3(19)	-4.4(14)
C15_1	28.1(19)	41.2(14)	30(3)	-1.1(14)	0.3(18)	-4.9(13)
C16_1	31.8(18)	48.4(16)	38(3)	-1.0(17)	-5.3(18)	-3.0(13)
C17_1	37(3)	48.9(17)	51(4)	-1.5(18)	-15(2)	0.1(15)
C18_1	34(2)	45.2(17)	43(3)	1.5(17)	-10(2)	-0.2(15)
C19_1	31(3)	51.8(19)	46(2)	-12.5(15)	3.7(18)	-7(2)
C20_1	61(5)	54(2)	47(2)	-14.6(18)	1(2)	-19(2)
C21_1	79(6)	64(2)	50(3)	-19(2)	1(3)	-28(3)
C22_1	81(6)	72(3)	50(2)	-18(2)	0(3)	-34(3)
C23_1	85(6)	76(3)	50(2)	-15(2)	-4(2)	-36(3)
C24_1	50(4)	63(3)	46(2)	-11.2(17)	-1.3(19)	-20(3)
C25_1	40(3)	67(3)	37(2)	-1(2)	-4.5(19)	-16(2)
C26_1	39(3)	62(3)	35(2)	-5(2)	0(2)	-13(2)
C27_1	47(3)	74(3)	35(3)	-2(2)	-2(2)	-23(3)
C28_1	54(4)	87(4)	39(3)	5(3)	-8(3)	-33(3)
C29_1	72(4)	104(5)	45(3)	16(3)	-20(3)	-51(4)
C30_1	46(3)	78(4)	39(2)	4(2)	-9(2)	-25(3)
C28_2	39(3)	33(3)	32(3)	10(2)	0(2)	12(2)
O1_2	21.3(13)	22(2)	31(3)	0.9(19)	0.7(13)	0.6(14)
C8_2	21.2(13)	22(2)	21(2)	-0.9(18)	0.2(13)	0.2(13)
N1_2	19.7(14)	24(2)	27(2)	2.9(16)	1.1(12)	1.9(14)
C9_2	23.2(15)	24(2)	24(3)	-0.2(17)	-2.5(15)	0.2(14)
C1_2	20(2)	25.3(17)	24(2)	1.1(12)	2.4(16)	4.5(15)
C7_2	19.7(15)	24(2)	26(2)	-3.2(18)	2.3(13)	2.1(15)
C10_2	23.4(15)	25(2)	23(3)	-3.5(18)	-2.4(15)	-0.2(14)
C18_2	27(2)	27(2)	35(3)	5(2)	2(2)	0.4(17)
C2_2	26(3)	26.0(17)	22.0(16)	1.2(11)	3.6(15)	2.6(18)
C3_2	31(2)	26(2)	25(2)	0.2(15)	3.5(16)	0.2(18)
O2_2	24(2)	28(2)	29(2)	-0.3(16)	1.8(16)	-1.0(17)
C11_2	21.8(17)	23(2)	26(2)	-4.5(18)	1.2(15)	2.7(15)
C15_2	19.0(15)	27.6(19)	23(3)	-0.6(17)	0.9(15)	-1.0(14)
C17_2	23.9(17)	22(2)	30(3)	0.6(19)	1.4(17)	-2.5(14)
O3_2	33(2)	18.4(19)	19.9(16)	5.3(13)	3.8(15)	1.7(18)
O4_2	33(2)	22.2(19)	19.9(16)	-2.2(13)	2.3(15)	-2.9(19)
C4_2	30(3)	27(2)	30(4)	4(2)	-3(3)	-1(2)
C5_2	34(3)	22(3)	31(3)	0(2)	-3(2)	-3(3)
C6_2	34(3)	25(3)	32(4)	-1(3)	8(2)	1(2)
C12_2	22.5(17)	34(3)	27(3)	-4(2)	0.8(17)	-0.3(16)

Atom	U_{11}	U_{22}	U_{33}	U_{23}	U_{13}	U_{12}
C14_2	18.7(15)	31(2)	28(3)	-4.2(19)	1.6(16)	-1.3(14)
C16_2	23.6(16)	27.4(18)	21(3)	-2.5(17)	3.0(16)	-2.3(13)
C13_2	20(2)	32(2)	27(3)	-4(2)	0.7(19)	-2.9(17)
C19_2	26.4(19)	31(3)	34(2)	-5.8(19)	-5.5(15)	0(2)
C25_2	26(2)	28(2)	24(2)	-2.1(17)	2.1(17)	-1.3(18)
C20_2	27(2)	37(3)	40(3)	-7(2)	-3.7(18)	1(2)
C24_2	22(3)	32(3)	34(2)	-5.4(19)	-6.4(17)	1(2)
C26_2	24(2)	25(2)	16(3)	1.6(18)	-1.2(18)	-0.9(19)
C30_2	29(3)	32(3)	27(2)	2.1(19)	6.2(19)	6(2)
C21_2	40(3)	41(4)	41(3)	-12(2)	-10(2)	10(3)
C23_2	31(2)	40(4)	40(3)	-4(2)	-11.2(19)	6(2)
C27_2	35(3)	30(3)	29(3)	6(2)	-5(2)	4(2)
C29_2	32(3)	29(3)	34(3)	-1.1(19)	-4(2)	3(2)
C22_2	30(2)	51(4)	40(3)	-10(3)	-13(2)	6(2)
O1_3	41(3)	30.9(15)	36(3)	2.4(15)	5(2)	3.2(16)
C8_3	30(2)	30.9(15)	24(3)	2.3(15)	4(2)	2.5(16)
N1_3	25(2)	25.8(16)	26(2)	0.1(14)	4.3(16)	-4.0(15)
C9_3	33(2)	38.3(18)	28(3)	6.2(18)	5(2)	0.1(16)
C1_3	24.3(17)	31(3)	26(2)	3.3(17)	2.0(12)	-5.2(16)
C7_3	28(3)	25.9(16)	28(3)	-0.1(14)	2.0(19)	-3.9(16)
C10_3	25(2)	37.5(18)	29(3)	5.2(17)	-0.9(19)	-2.7(16)
C18_3	42(3)	51(3)	44(4)	4(3)	14(3)	6(2)
C2_3	24.5(17)	21(3)	26.8(16)	-3.2(15)	2.2(11)	-6.3(18)
C3_3	29(2)	28(2)	28(2)	2.6(17)	-1.0(15)	-0.6(19)
O2_3	33(2)	21(2)	28(2)	4.6(17)	5.9(16)	-2.6(17)
C11_3	32(3)	37(2)	40(3)	10.5(19)	9(2)	0.4(19)
C15_3	31(2)	38(2)	25(3)	0.4(18)	-0.7(19)	-8.9(17)
C17_3	45(3)	56(2)	47(4)	5(2)	16(3)	3.7(19)
O3_3	27(2)	30(2)	27.2(16)	-3.3(16)	1.3(14)	-5(2)
O4_3	27(2)	25(2)	27.5(17)	-3.1(17)	3.4(14)	-4.2(19)
C4_3	30(2)	36(4)	54(5)	-2(3)	0(2)	0(2)
C5_3	33(3)	38(3)	45(4)	15(3)	1(3)	0(3)
C6_3	32(4)	31(3)	34(4)	-2(2)	-13(3)	5(2)
C12_3	42(3)	39(2)	53(4)	15(2)	16(3)	4(2)
C14_3	37(3)	39(2)	37(3)	4.8(19)	4(2)	-6.5(18)
C16_3	36(2)	54(2)	33(4)	0(2)	2(2)	-0.8(18)
C13_3	44(3)	39(3)	46(4)	10(2)	12(3)	-3(2)
C19_3	49(4)	57(3)	43(3)	14(2)	-1(2)	-20(3)
C25_3	46(3)	67(3)	39(3)	-5(2)	8(2)	-12(3)
C20_3	84(6)	64(3)	52(4)	8(3)	13(4)	-35(3)
C24_3	58(5)	68(4)	44(3)	12(2)	2(3)	-27(4)
C26_3	41(3)	66(3)	36(4)	-5(3)	11(3)	-11(3)
C30_3	44(4)	75(5)	39(3)	-6(3)	7(2)	-9(3)
C21_3	113(8)	82(4)	63(4)	18(3)	17(4)	-48(5)
C23_3	81(6)	83(4)	56(4)	21(3)	7(4)	-37(4)
C27_3	50(4)	76(5)	51(4)	-4(3)	20(3)	-16(3)
C29_3	58(4)	86(5)	59(5)	-10(3)	21(3)	-18(4)
C22_3	90(7)	86(4)	62(4)	18(4)	12(4)	-41(4)
C28_3	47(4)	78(5)	49(4)	-2(3)	17(3)	-9(3)
C38_5	81(6)	62(5)	21(4)	-9(3)	-7(3)	26(6)
N2_5	40(4)	28(3)	31(4)	-2(2)	0(3)	-2(3)
C37_5	40(4)	28(4)	46(5)	-8(3)	-1(3)	4(4)
C34_7	82(2)	83(2)	83(2)	0.3(11)	-1.5(11)	-0.8(11)
C31_7	82(2)	83(2)	83(2)	0.3(11)	-1.5(11)	-0.8(11)
C32_7	82(2)	83(2)	83(2)	0.3(11)	-1.5(11)	-0.8(11)
C36_7	82(2)	83(2)	83(2)	0.3(11)	-1.5(11)	-0.8(11)
C33_7	82(2)	83(2)	83(2)	0.3(11)	-1.5(11)	-0.8(11)
C35_7	82(2)	83(2)	83(2)	0.3(11)	-1.5(11)	-0.8(11)

Atom	U_{11}	U_{22}	U_{33}	U_{23}	U_{13}	U_{12}
C35_8	82(2)	83(2)	83(2)	0.3(11)	-1.5(11)	-0.8(11)
C36_8	82(2)	83(2)	83(2)	0.3(11)	-1.5(11)	-0.8(11)
C31_8	82(2)	83(2)	83(2)	0.3(11)	-1.5(11)	-0.8(11)
C32_8	82(2)	83(2)	83(2)	0.3(11)	-1.5(11)	-0.8(11)
C33_8	82(2)	83(2)	83(2)	0.3(11)	-1.5(11)	-0.8(11)
C34_8	82(2)	83(2)	83(2)	0.3(11)	-1.5(11)	-0.8(11)
C33_9	54(3)	50.2(19)	49(2)	0.3(16)	3(2)	8(2)
C31_9	54(3)	50.2(19)	49(2)	0.3(16)	3(2)	8(2)
C32_9	54(3)	50.2(19)	49(2)	0.3(16)	3(2)	8(2)
C36_9	54(3)	50.2(19)	49(2)	0.3(16)	3(2)	8(2)
C35_9	54(3)	50.2(19)	49(2)	0.3(16)	3(2)	8(2)
C34_9	54(3)	50.2(19)	49(2)	0.3(16)	3(2)	8(2)
C33_10	54(3)	50.2(19)	49(2)	0.3(16)	3(2)	8(2)
C32_10	54(3)	50.2(19)	49(2)	0.3(16)	3(2)	8(2)
C31_10	54(3)	50.2(19)	49(2)	0.3(16)	3(2)	8(2)
C36_10	54(3)	50.2(19)	49(2)	0.3(16)	3(2)	8(2)
C35_10	54(3)	50.2(19)	49(2)	0.3(16)	3(2)	8(2)
C34_10	54(3)	50.2(19)	49(2)	0.3(16)	3(2)	8(2)
C31_11	69(2)	73(3)	71(3)	0(3)	4.6(18)	-4(3)
C32_11	69(2)	73(3)	71(3)	0(3)	4.6(18)	-4(3)
C33_11	69(2)	73(3)	71(3)	0(3)	4.6(18)	-4(3)
C34_11	69(2)	73(3)	71(3)	0(3)	4.6(18)	-4(3)
C35_11	69(2)	73(3)	71(3)	0(3)	4.6(18)	-4(3)
C36_11	69(2)	73(3)	71(3)	0(3)	4.6(18)	-4(3)
C31_12	69(2)	73(3)	71(3)	0(3)	4.6(18)	-4(3)
C32_12	69(2)	73(3)	71(3)	0(3)	4.6(18)	-4(3)
C33_12	69(2)	73(3)	71(3)	0(3)	4.6(18)	-4(3)
C34_12	69(2)	73(3)	71(3)	0(3)	4.6(18)	-4(3)
C35_12	69(2)	73(3)	71(3)	0(3)	4.6(18)	-4(3)
C36_12	69(2)	73(3)	71(3)	0(3)	4.6(18)	-4(3)
C36_13	52.8(18)	51.6(15)	52.5(16)	-0.4(10)	0.2(11)	0.1(11)
C31_13	52.8(18)	51.6(15)	52.5(16)	-0.4(10)	0.2(11)	0.1(11)
C32_13	52.8(18)	51.6(15)	52.5(16)	-0.4(10)	0.2(11)	0.1(11)
C33_13	52.8(18)	51.6(15)	52.5(16)	-0.4(10)	0.2(11)	0.1(11)
C35_13	52.8(18)	51.6(15)	52.5(16)	-0.4(10)	0.2(11)	0.1(11)
C34_13	52.8(18)	51.6(15)	52.5(16)	-0.4(10)	0.2(11)	0.1(11)
C36_14	52.8(18)	51.6(15)	52.5(16)	-0.4(10)	0.2(11)	0.1(11)
C31_14	52.8(18)	51.6(15)	52.5(16)	-0.4(10)	0.2(11)	0.1(11)
C32_14	52.8(18)	51.6(15)	52.5(16)	-0.4(10)	0.2(11)	0.1(11)
C33_14	52.8(18)	51.6(15)	52.5(16)	-0.4(10)	0.2(11)	0.1(11)
C35_14	52.8(18)	51.6(15)	52.5(16)	-0.4(10)	0.2(11)	0.1(11)
C34_14	52.8(18)	51.6(15)	52.5(16)	-0.4(10)	0.2(11)	0.1(11)
C33_15	53(2)	57(3)	57(3)	3(2)	-9(2)	-5(2)
C32_15	53(2)	57(3)	57(3)	3(2)	-9(2)	-5(2)
C31_15	53(2)	57(3)	57(3)	3(2)	-9(2)	-5(2)
C36_15	53(2)	57(3)	57(3)	3(2)	-9(2)	-5(2)
C35_15	53(2)	57(3)	57(3)	3(2)	-9(2)	-5(2)
C34_15	53(2)	57(3)	57(3)	3(2)	-9(2)	-5(2)
C33_16	53(2)	57(3)	57(3)	3(2)	-9(2)	-5(2)
C32_16	53(2)	57(3)	57(3)	3(2)	-9(2)	-5(2)
C31_16	53(2)	57(3)	57(3)	3(2)	-9(2)	-5(2)
C36_16	53(2)	57(3)	57(3)	3(2)	-9(2)	-5(2)
C35_16	53(2)	57(3)	57(3)	3(2)	-9(2)	-5(2)
C34_16	53(2)	57(3)	57(3)	3(2)	-9(2)	-5(2)
C33_6	47(3)	42(4)	31(3)	3(3)	-4(3)	5(5)
C31_6	47(3)	42(4)	31(3)	3(3)	-4(3)	5(5)
C32_6	47(3)	42(4)	31(3)	3(3)	-4(3)	5(5)
C36_6	47(3)	42(4)	31(3)	3(3)	-4(3)	5(5)

Atom	U_{11}	U_{22}	U_{33}	U_{23}	U_{13}	U_{12}
C35_6	47(3)	42(4)	31(3)	3(3)	-4(3)	5(5)
C34_6	47(3)	42(4)	31(3)	3(3)	-4(3)	5(5)

Compound 8b

Submitted by: **Duc Ly**

Solved by: **John Bacsa**

R_1=6.65%

Crystal Data and Experimental

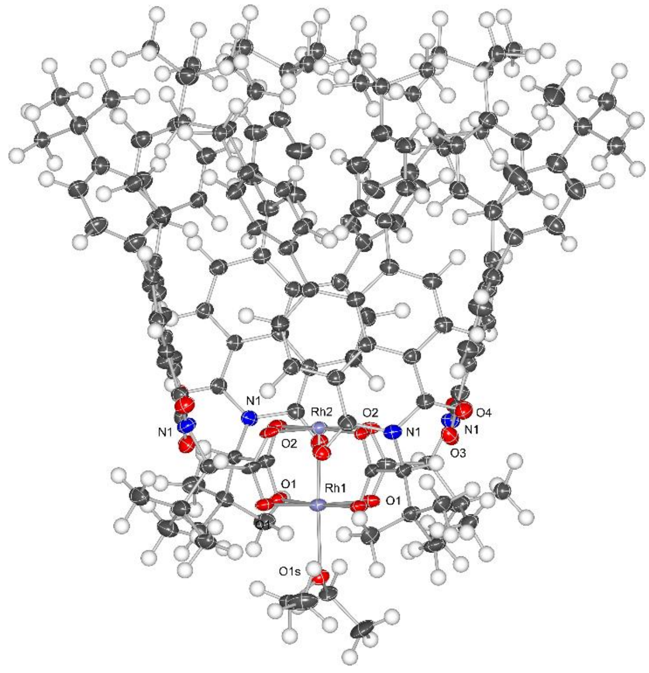

Experimental. Single green plate-shaped crystals of **Compound 8b** were chosen from the sample as supplied. A suiTable Scrystal with dimensions 0.22 × 0.17 × 0.12 mm³ was selected and mounted on a loop with paratone on a XtaLAB AFC11 (RCD3): quarter-chi single diffractometer. The crystal was kept at a steady T = 173.0(2) K during data collection. The structure was solved with the ShelXT (Sheldrick, 2015) solution program and by using Olex2 1.5-alpha (Dolomanov et al., 2009) as the graphical interface. The model was refined with ShelXL 2018/3 (Sheldrick, 2015) using full matrix least squares minimisation on $\boldsymbol{F}^2$.

Crystal Data. $C_{162}H_{182}N_4O_{23}Rh_2$, M_r = 2758.93, orthorhombic, $P2_12_12_1$ (No. 19), a = 18.3865(3) Å, b = 22.5167(4) Å, c = 36.4429(4) Å, $\alpha = \beta = \gamma =$ 90°, V = 15087.5(4) Å³, T = 173.0(2) K, Z = 4, Z' = 1, μ(Cu K$_\alpha$) = 2.306, 56773 reflections measured, 17440 unique (R_{int} = 0.0665) which were used in all calculations. The final wR_2 was 0.1428 (all data) and R_1 was 0.0665 (I≥2 σ(I)).

**Compound**	**8b**
Formula	$C_{162}H_{182}N_4O_{23}Rh_2$
$D_{calc.}$/ g cm^{-3}	1.215
μ/mm^{-1}	2.306
Formula Weight	2758.93
Colour	green
Shape	plate-shaped
Size/mm³	0.22×0.17×0.12
T/K	173.0(2)
Crystal System	orthorhombic
Flack Parameter	-0.007(5)
Hooft Parameter	-0.007(5)
Space Group	$P2_12_12_1$
a/Å	18.3865(3)
b/Å	22.5167(4)
c/Å	36.4429(4)
α/°	90
β/°	90
γ/°	90
V/Å³	15087.5(4)
Z	4
Z'	1
Wavelength/Å	1.54184
Radiation type	Cu K$_\alpha$
Θ_{min}/°	2.307
Θ_{max}/°	55.071
Measured Refl's.	56773
Indep't Refl's	17440
Refl's I≥2 σ(I)	12792
R_{int}	0.0665
Parameters	1650
Restraints	2438
Largest Peak	1.256
Deepest Hole	-0.686
GooF	0.854
wR_2 (all data)	0.1428
wR_2	0.1322
R_1 (all data)	0.0909
R_1	0.0665

Structure Quality Indicators

**Reflections:**	d min (CuKα) 2Θ=110.1° 0.94	I/σ(I) 12.2	Rint m=3.26 6.65%	Full 110.1° 97.2	
**Refinement:**	Shift 0.003	Max Peak 1.3	Min Peak -0.7	GooF 0.854	Hooft -.007(5)

A green plate-shaped-shaped crystal with dimensions 0.22 × 0.17 × 0.12 mm^3 was mounted on a loop with paratone. Data were collected using a XtaLAB AFC11 (RCD3): quarter-chi single diffractometer operating at T = 173.0(2) K.

Data were measured using ω scans with Cu K_α radiation. The diffraction pattern was indexed and the total number of runs and images was based on the strategy calculation from the program CrysAlisPro system (CCD 43.92a 64-bit (release 05-10-2023)). The maximum resolution that was achieved was Θ = 55.071° (0.94 Å).

The unit cell was refined using CrysAlisPro 1.171.43.103a (Rigaku OD, 2023) on 9585 reflections, 17% of the observed reflections.

Data reduction, scaling and absorption corrections were performed using CrysAlisPro 1.171.43.103a (Rigaku OD, 2023). The final completeness is 97.30 % out to 55.071° in Θ. A numerical absorption correction based on gaussian integration over a multifaceted crystal model was performed using CrysAlisPro 1.171.41.108a (Rigaku Oxford Diffraction, 2021). An empirical absorption correction using spherical harmonics, implemented in SCALE3 ABSPACK scaling algorithm was also applied. The absorption coefficient μ of this material is 2.306 mm^{-1} at this wavelength (λ = 1.54184Å) and the minimum and maximum transmissions are 0.692 and 0.802.

The structure was solved and the space group $P2_12_12_1$ (# 19) determined by the ShelXT (Sheldrick, 2015) structure solution program and refined by full matrix least squares minimisation on $\boldsymbol{F}^2$ using version 2018/3 of ShelXL 2018/3 (Sheldrick, 2015). All non-hydrogen atoms were refined anisotropically. Hydrogen atom positions were calculated geometrically and refined using the riding model.

There is a single formula unit in the asymmetric unit, which is represented by the reported sum formula. In other words: Z is 4 and Z' is 1. The moiety formula is C156 H170 N4 O17 Rh2, 2[C3], 2[O3], 2[H6].

The Flack parameter was refined to -0.007(5). Determination of absolute structure using Bayesian statistics on Bijvoet differences using the Olex2 results in -0.007(5). The chiral atoms in this structure are: C13_3(S), C13_4(S), C13_5(S), C13_7(S). Note: The Flack parameter is used to determine chirality of the crystal studied, the value should be near 0, a value of 1 means that the stereochemistry is wrong and the model should be inverted. A value of 0.5 means that the crystal consists of a racemic mixture of the two enantiomers.

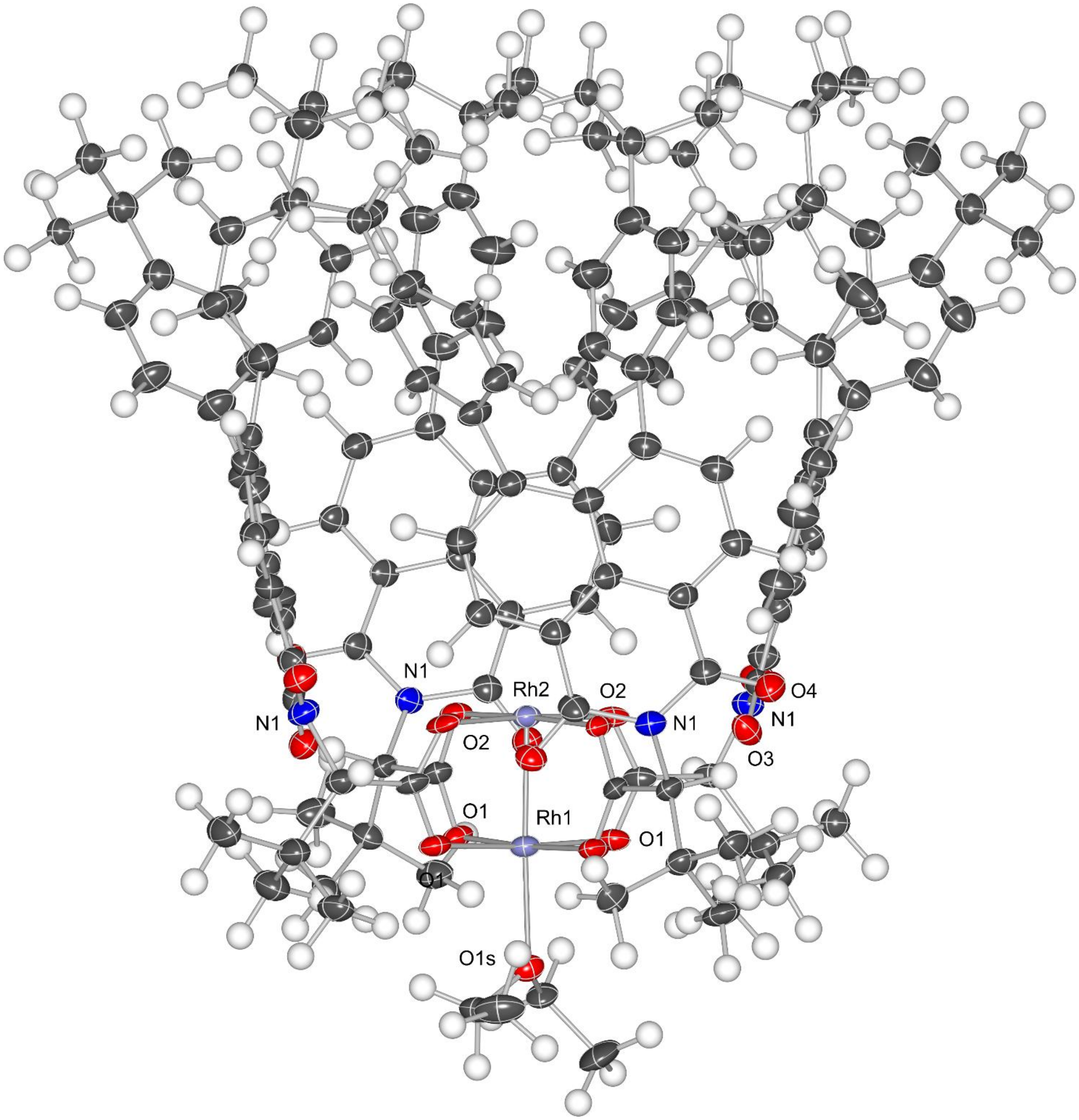

Figure S310. Thermal ellipsoidal representation (50% probability for all atoms, excluding hydrogens) of the molecular structure.

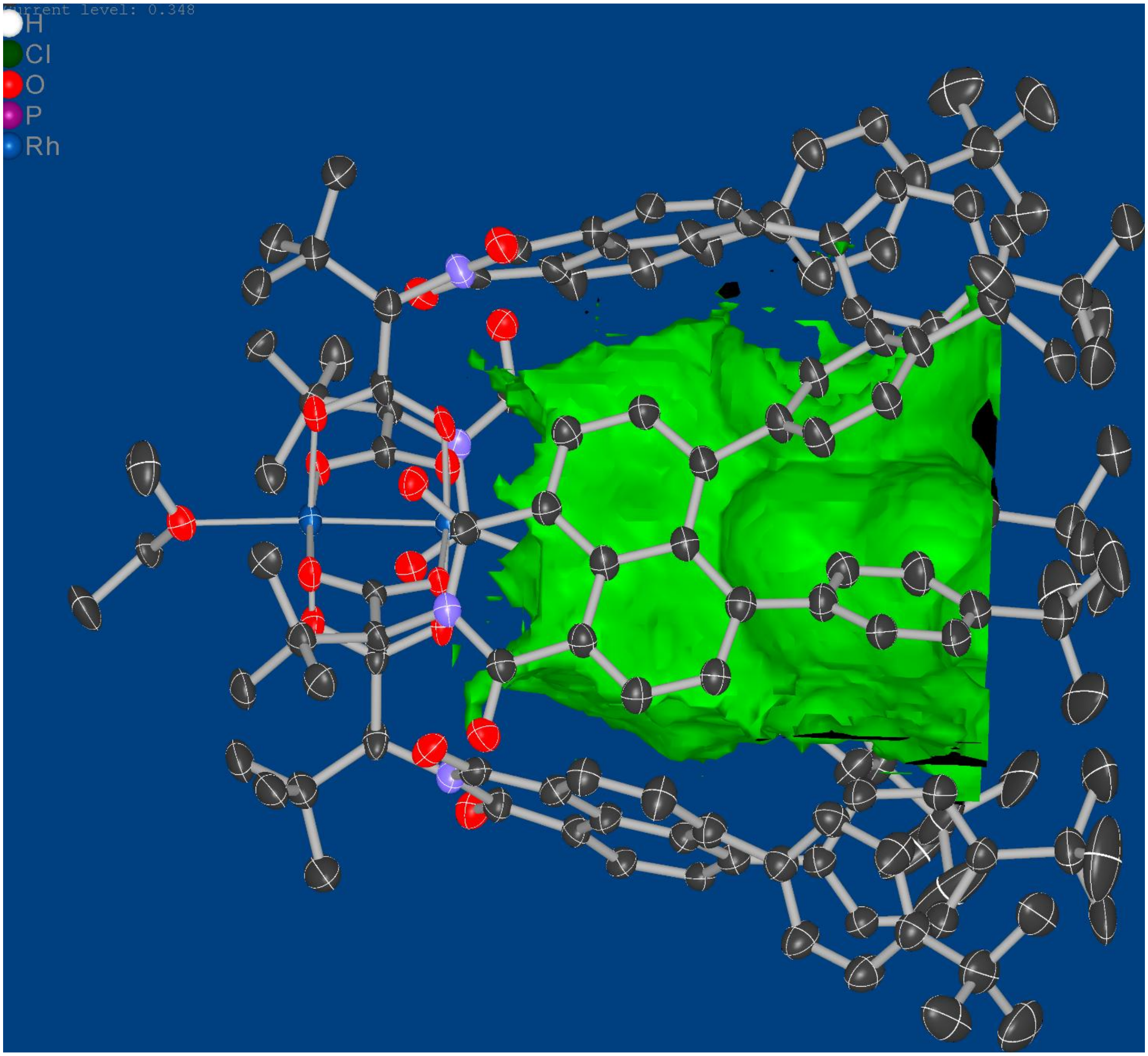

Figure S311. The molecule has significant void space inside its bowl shape cavity,

Data Plots: Diffraction Data

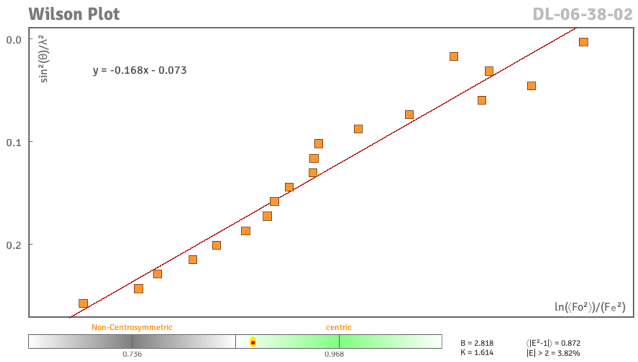

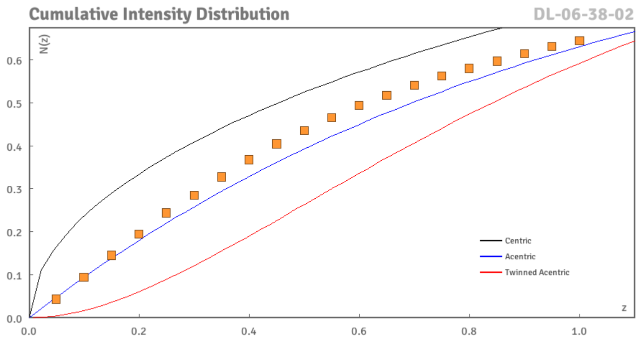

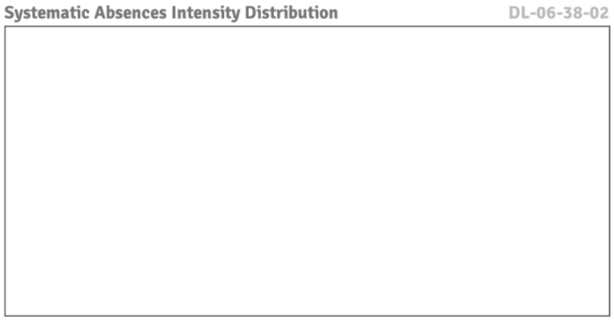

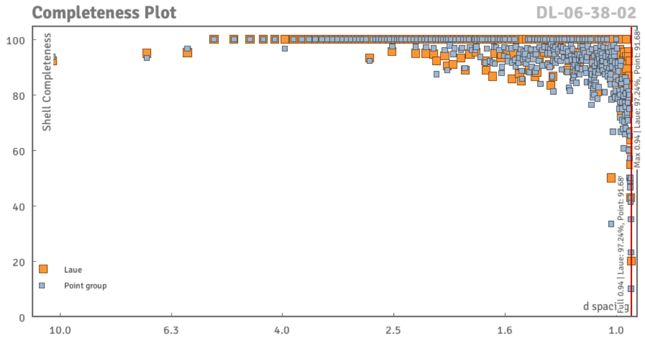

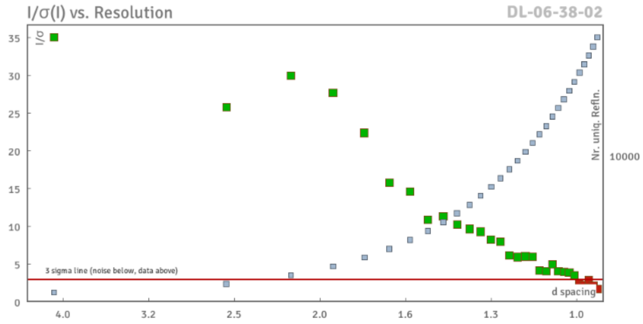

Data Plots: Refinement and Data

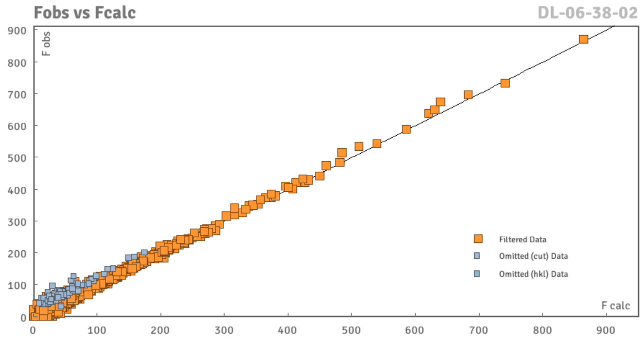

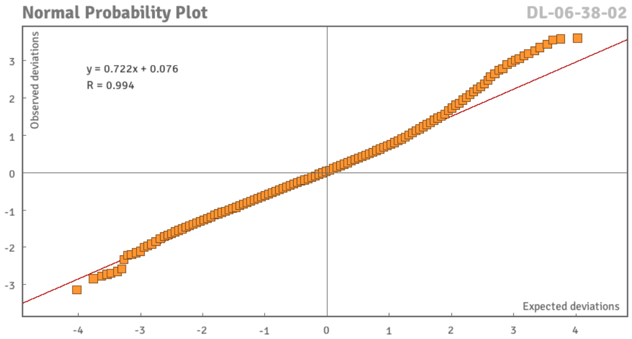

Reflection Statistics

Total reflections (after filtering)	56773	Unique reflections	17440
Completeness	0.917	Mean I/σ	10.51
hkl_{max} collected	(18, 20, 37)	hkl_{min} collected	(-19, -23, -38)
hkl_{max} used	(19, 23, 38)	hkl_{min} used	(-19, 0, 0)
Lim d_{max} collected	100.0	Lim d_{min} collected	0.77
d_{max} used	19.16	d_{min} used	0.94
Friedel pairs	8797	Friedel pairs merged	0
Inconsistent equivalents	21	R_{int}	0.0665
R_{sigma}	0.0823	Intensity transformed	0
Omitted reflections	0	Omitted by user (OMIT hkl)	439
Multiplicity	(19899, 12307, 3717, 300, 60, 8)	Maximum multiplicity	14
Removed systematic absences	0	Filtered off (Shel/OMIT)	0

Table S20. Fractional Atomic Coordinates (×10[4]) and Equivalent Isotropic Displacement Parameters (Å^2×10^3) for

Compound 8b. U_{eq} is defined as 1/3 of the trace of the orthogonalised U_{ij}.

Atom	x	y	z	U_{eq}
Rh1	2476.0(7)	2861.2(4)	4246.4(2)	40.3(2)
Rh2	2523.2(7)	2952.5(4)	3598.9(2)	41.0(2)
O1_3	1429(3)	2610(4)	4190.5(14)	47(2)
O2_3	1466(3)	2744(4)	3580.7(15)	51(2)
O3_3	1218(4)	1457(3)	3682.9(15)	55(2)
O4_3	-679(4)	2599(3)	3306.5(17)	53(2)
N1_3	295(3)	2057(3)	3500.6(15)	46(2)
C1_3	869(5)	1630(4)	3426.6(18)	45(3)
C2_3	949(5)	1440(5)	3050.3(18)	47(3)
C3_3	1576(5)	1124(6)	2963(2)	66(4)
C4_3	1672(5)	940(6)	2601(2)	60(4)
C5_3	1194(5)	1061(4)	2320.0(19)	53(3)
C6_3	587(5)	1469(5)	2392.4(17)	48(3)
C7_3	137(4)	1720(4)	2119.9(17)	47(3)
C8_3	-495(5)	2013(5)	2228.8(19)	55(3)
C9_3	-613(5)	2151(5)	2595.8(18)	51(3)
C10_3	-135(5)	1980(5)	2862.3(18)	45(3)
C11_3	-217(5)	2218(5)	3234.7(19)	51(3)
C12_3	476(5)	1625(5)	2769.0(18)	46(3)
C13_3	337(4)	2421(3)	3836.7(17)	45(2)
C14_3	-53(4)	2169(3)	4183.2(17)	47(2)
C15_3	-835(4)	2022(6)	4067(2)	66(4)
C16_3	-95(6)	2672(4)	4467(2)	51(3)
C17_3	302(6)	1620(4)	4357(3)	60(4)
C18_3	1345(4)	738(4)	1974(2)	60(4)
C19_3	789(5)	431(5)	1802(3)	66(4)
C20_3	932(5)	69(6)	1506(3)	73(4)
C21_3	1622(5)	22(5)	1359(3)	80(5)
C22_3	2164(5)	385(6)	1513(3)	72(4)
C23_3	2019(5)	742(5)	1812(3)	64(4)
C24_3	1781(6)	-415(5)	1049(3)	104(5)
C25_3	1447(10)	-188(8)	697(3)	164(9)
C26_3	1425(10)	-1015(6)	1136(5)	163(9)
C27_3	2589(6)	-510(8)	998(5)	189(10)
C28_3	286(5)	1775(4)	1721.8(17)	59(3)
C29_3	-197(5)	1642(6)	1449(2)	69(4)
C30_3	-48(5)	1747(6)	1084(2)	69(4)
C31_3	625(5)	1944(5)	973.1(19)	70(4)
C32_3	1142(6)	2081(7)	1244(2)	87(5)
C33_3	980(5)	1983(6)	1612(2)	70(4)
C34_3	818(5)	2041(4)	570(2)	80(4)
C35_3	897(9)	1450(5)	378(3)	102(6)
C36_3	237(7)	2409(6)	386(3)	95(5)
C37_3	1526(6)	2369(7)	520(3)	97(5)
C38_3	1148(4)	2605(4)	3875.0(18)	42(3)
O1_4	2137(4)	3722(3)	4270.9(15)	48(2)
O2_4	2265(4)	3807(3)	3661.3(14)	55(3)
O3_4	664(4)	3923(3)	3773.0(16)	59(2)
O4_4	2024(4)	5542(3)	3489.2(17)	57(2)
N1_4	1360(4)	4723(3)	3638.1(16)	51(2)
C1_4	828(5)	4273(4)	3539.8(18)	48(3)
C2_4	636(6)	4238(4)	3156.0(19)	50(3)
C3_4	141(7)	3799(5)	3057(2)	73(5)
C4_4	-81(7)	3768(5)	2693(2)	67(4)
C5_4	98(5)	4178(4)	2427(2)	62(4)

Atom	x	y	z	U_{eq}
C6_4	600(6)	4660(4)	2525.2(18)	54(3)
C7_4	899(5)	5067(4)	2271.6(17)	51(3)
C8_4	1284(6)	5552(4)	2406.4(19)	46(3)
C9_4	1450(6)	5599(4)	2777.6(19)	45(3)
C10_4	1276(6)	5157(4)	3018.8(19)	46(3)
C11_4	1574(6)	5167(4)	3392(2)	47(3)
C12_4	825(6)	4681(4)	2900.9(19)	52(3)
C13_4	1784(4)	4647(3)	3978.8(17)	53(3)
C14_4	1426(4)	4873(3)	4340.3(18)	61(3)
C15_4	1117(7)	5493(4)	4258(3)	74(4)
C16_4	2037(6)	4953(5)	4624(2)	65(4)
C17_4	826(6)	4476(5)	4501(3)	70(4)
C18_4	-339(5)	4131(4)	2086(2)	65(4)
C19_4	-723(6)	4621(4)	1963(3)	69(4)
C20_4	-1146(7)	4589(4)	1652(3)	66(4)
C21_4	-1152(6)	4089(4)	1431(3)	79(4)
C22_4	-734(8)	3604(5)	1552(3)	84(5)
C23_4	-334(8)	3630(5)	1871(3)	78(4)
C24_4	-1651(6)	4052(4)	1098(2)	91(4)
C25_4	-1585(9)	4614(5)	875(3)	117(6)
C26_4	-2443(6)	4001(7)	1226(4)	122(6)
C27_4	-1472(7)	3518(5)	861(3)	87(5)
C28_4	950(5)	5000(4)	1867.7(17)	56(3)
C29_4	692(8)	5400(5)	1618(2)	64(4)
C30_4	751(7)	5311(4)	1245(2)	54(3)
C31_4	1012(7)	4786(4)	1104.2(19)	64(4)
C32_4	1247(7)	4348(4)	1352(2)	59(4)
C33_4	1198(7)	4448(4)	1727(2)	57(3)
C34_4	1063(5)	4661(4)	693(2)	79(4)
C35_4	743(9)	5168(5)	476(3)	111(6)
C36_4	1847(6)	4562(8)	586(4)	122(6)
C37_4	653(9)	4107(6)	585(4)	116(6)
C38_4	2078(5)	4000(3)	3971.9(18)	47(3)
O1_5	3532(3)	3104(4)	4261.8(15)	48(2)
O2_5	3568(3)	3138(3)	3646.9(14)	42(2)
O3_5	3857(4)	4382(3)	3845.4(15)	52(2)
O4_5	5722(4)	3229(3)	3446.6(18)	59(2)
N1_5	4765(4)	3780(3)	3650.3(16)	50(2)
C1_5	4224(5)	4240(4)	3588.4(18)	52(3)
C2_5	4130(5)	4431(5)	3213.2(18)	47(3)
C3_5	3502(5)	4750(5)	3133(2)	54(3)
C4_5	3400(5)	4945(5)	2773(2)	53(3)
C5_5	3847(5)	4801(4)	2483.1(19)	54(3)
C6_5	4467(5)	4405(4)	2551.3(17)	45(3)
C7_5	4896(5)	4142(4)	2274.0(17)	47(3)
C8_5	5532(5)	3851(5)	2376.5(19)	50(3)
C9_5	5666(5)	3713(5)	2742.2(19)	55(3)
C10_5	5193(5)	3877(5)	3012.7(18)	46(3)
C11_5	5270(5)	3619(5)	3381(2)	53(3)
C12_5	4581(5)	4235(5)	2925.5(18)	49(3)
C13_5	4666(4)	3372(3)	3963.7(18)	46(3)
C14_5	5007(4)	3568(3)	4336.1(18)	57(3)
C15_5	5797(4)	3745(5)	4255(3)	61(3)
C16_5	5028(7)	3019(4)	4585(2)	68(4)
C17_5	4616(5)	4079(4)	4534(3)	55(3)
C18_5	3695(4)	5137(4)	2142(2)	51(3)
C19_5	4254(5)	5445(5)	1973(3)	64(4)
C20_5	4123(5)	5796(5)	1670(3)	54(4)

Atom	x	y	z	U_{eq}
C21_5	3434(5)	5849(5)	1521(2)	61(4)
C22_5	2887(5)	5492(5)	1677(3)	57(4)
C23_5	3026(5)	5132(5)	1975(3)	57(4)
C24_5	3303(5)	6231(4)	1182(2)	65(3)
C25_5	3612(7)	5921(6)	849(2)	84(4)
C26_5	3705(7)	6824(5)	1224(4)	87(5)
C27_5	2502(5)	6356(5)	1125(3)	75(4)
C28_5	4737(5)	4095(5)	1876.6(17)	60(3)
C29_5	5225(5)	4202(6)	1602(2)	60(3)
C30_5	5077(5)	4075(6)	1240(2)	67(4)
C31_5	4407(5)	3865(6)	1133(2)	80(4)
C32_5	3869(5)	3793(7)	1404(2)	83(5)
C33_5	4031(5)	3910(6)	1769(2)	65(4)
C34_5	4229(6)	3707(5)	736(2)	113(5)
C35_5	4177(11)	4263(6)	507(3)	156(9)
C36_5	4806(9)	3298(8)	584(4)	179(10)
C37_5	3513(8)	3385(8)	699(4)	148(7)
C38_5	3848(4)	3198(4)	3960.5(18)	41(3)
O1_7	2812(4)	2020(3)	4190.8(14)	50(2)
O2_7	2788(3)	2085(3)	3577.3(14)	40.6(19)
O3_7	4346(4)	1948(3)	3629.8(16)	61(2)
O4_7	3011(5)	326(3)	3329.6(17)	60(2)
N1_7	3638(4)	1161(3)	3482.4(15)	56(3)
C1_7	4161(7)	1620(5)	3388.8(19)	56(4)
C2_7	4381(6)	1652(4)	3009.1(19)	52(3)
C3_7	4815(7)	2129(5)	2906(2)	67(4)
C4_7	5064(8)	2156(5)	2546(2)	78(5)
C5_7	4913(5)	1738(4)	2280(2)	57(3)
C6_7	4405(6)	1257(4)	2371.3(17)	52(3)
C7_7	4116(5)	852(4)	2114.4(17)	51(3)
C8_7	3752(6)	352(4)	2245.5(19)	49(3)
C9_7	3568(7)	302(4)	2614.4(19)	53(3)
C10_7	3741(6)	738(4)	2860.5(19)	53(3)
C11_7	3430(6)	723(4)	3231(2)	56(3)
C12_7	4188(6)	1219(4)	2747.9(19)	49(3)
C13_7	3258(4)	1188(3)	3838.2(17)	55(3)
C14_7	3667(4)	930(3)	4177.7(18)	70(3)
C15_7	4038(7)	355(4)	4051(3)	83(5)
C16_7	3091(6)	758(5)	4464(2)	75(4)
C17_7	4231(6)	1345(5)	4354(3)	75(4)
C18_7	5364(5)	1791(4)	1945(2)	60(3)
C19_7	5759(7)	1304(4)	1826(3)	73(4)
C20_7	6204(7)	1340(4)	1523(3)	69(4)
C21_7	6259(6)	1855(4)	1318(3)	76(4)
C22_7	5848(7)	2344(4)	1440(3)	80(5)
C23_7	5388(7)	2299(4)	1737(3)	65(4)
C24_7	6820(5)	1907(4)	1010(2)	99(5)
C25_7	6809(8)	1347(5)	782(3)	108(5)
C26_7	7583(6)	1965(7)	1177(4)	111(5)
C27_7	6678(7)	2441(6)	769(3)	96(5)
C28_7	4135(5)	890(4)	1708.7(17)	53(3)
C29_7	4359(7)	448(4)	1479(2)	57(3)
C30_7	4332(8)	500(4)	1102(2)	70(4)
C31_7	4080(7)	1009(4)	937.0(19)	64(4)
C32_7	3852(8)	1479(5)	1163(2)	67(4)
C33_7	3890(7)	1423(4)	1542(2)	63(4)
C34_7	4039(6)	1085(4)	521(2)	88(4)
C35_7	4628(10)	1501(8)	391(4)	156(9)

Atom	x	y	z	U_{eq}
C36_7	4110(10)	490(5)	334(3)	116(6)
C37_7	3322(8)	1346(9)	399(3)	164(9)
C38_7	2946(5)	1827(3)	3873.0(18)	43(3)
O1_1	2446(5)	2711(3)	4851.7(17)	50.3(19)
C1_1	2605(10)	1874(6)	5262(4)	97(6)
C2_1	2140(6)	2165(4)	4970(3)	55(3)
C3_1	2271(6)	3206(5)	5076(3)	61(4)
C4_1	2901(8)	3640(6)	5093(4)	77(5)

Table S21. Anisotropic Displacement Parameters (×10[4]) for **Compound 8b**. The anisotropic displacement factor exponent takes the form: $-2\pi^2[h^2a^{*2} \times U_{11} + ... + 2hka^* \times b^* \times U_{12}]$

Atom	U_{11}	U_{22}	U_{33}	U_{23}	U_{13}	U_{12}
Rh1	48.0(5)	46.4(5)	26.3(4)	1.7(4)	-3.4(6)	-1.6(7)
Rh2	44.9(5)	50.8(5)	27.1(4)	1.5(4)	-1.9(6)	0.3(7)
O1_3	51(5)	63(6)	26(3)	16(4)	-7(3)	-2(4)
O2_3	57(5)	70(6)	27(4)	8(4)	-7(4)	0(5)
O3_3	59(6)	58(6)	48(4)	0(4)	-3(4)	9(5)
O4_3	51(6)	65(6)	42(5)	-1(4)	-11(4)	3(4)
N1_3	42(5)	58(6)	37(4)	0(4)	-1(3)	-1(4)
C1_3	45(7)	45(7)	45(4)	3(5)	3(4)	-7(5)
C2_3	40(6)	53(8)	47(4)	-4(5)	0(4)	-9(5)
C3_3	61(8)	86(11)	51(6)	-13(7)	-9(6)	18(7)
C4_3	36(7)	90(11)	53(5)	-14(6)	-2(5)	9(6)
C5_3	47(7)	60(8)	53(5)	-9(5)	-5(4)	1(5)
C6_3	48(7)	50(8)	45(4)	-4(5)	-3(4)	-5(5)
C7_3	44(6)	52(8)	47(4)	1(5)	-4(4)	-10(5)
C8_3	47(7)	74(9)	44(4)	-1(6)	-4(5)	-2(6)
C9_3	53(7)	57(8)	42(4)	0(6)	-6(4)	-3(6)
C10_3	38(6)	56(8)	41(4)	1(5)	0(4)	-11(5)
C11_3	53(7)	59(8)	41(4)	0(5)	-6(4)	-1(5)
C12_3	42(6)	51(8)	45(4)	4(5)	4(4)	-10(5)
C13_3	47(5)	53(7)	34(4)	4(4)	1(4)	1(5)
C14_3	47(6)	52(6)	43(5)	5(4)	3(5)	-4(5)
C15_3	48(6)	99(11)	51(8)	9(7)	-2(5)	-17(7)
C16_3	48(8)	66(7)	40(6)	0(5)	7(6)	5(6)
C17_3	77(9)	55(7)	49(8)	6(5)	-8(7)	-1(7)
C18_3	38(6)	86(9)	56(6)	-20(6)	-9(5)	4(6)
C19_3	44(7)	95(11)	58(8)	-24(7)	5(6)	-11(7)
C20_3	67(7)	97(11)	55(8)	-27(7)	9(6)	-13(7)
C21_3	72(7)	106(10)	61(8)	-33(7)	19(6)	-16(7)
C22_3	63(8)	83(10)	71(8)	-29(7)	6(6)	5(6)
C23_3	44(6)	80(10)	69(8)	-28(7)	-1(6)	-3(6)
C24_3	77(9)	140(12)	96(9)	-71(9)	21(8)	-17(9)
C25_3	176(19)	230(20)	82(9)	-92(12)	10(11)	51(18)
C26_3	162(19)	134(12)	190(20)	-105(12)	65(17)	-41(13)
C27_3	73(9)	280(20)	210(20)	-166(19)	27(11)	-7(12)
C28_3	76(7)	53(9)	47(4)	-3(5)	2(4)	-5(6)
C29_3	91(9)	71(10)	45(5)	-8(6)	-1(5)	-14(8)
C30_3	96(8)	67(10)	45(5)	-5(7)	1(6)	0(8)
C31_3	100(8)	66(10)	43(5)	-2(6)	6(5)	2(7)
C32_3	111(10)	106(13)	44(5)	-5(8)	11(5)	-29(10)
C33_3	83(8)	81(11)	46(5)	-4(7)	6(5)	-20(8)
C34_3	101(9)	94(10)	44(5)	2(6)	4(6)	-3(7)
C35_3	146(17)	102(10)	59(9)	-9(8)	25(11)	-4(10)
C36_3	117(11)	107(12)	60(9)	17(9)	-7(9)	-3(9)

Atom	U_{11}	U_{22}	U_{33}	U_{23}	U_{13}	U_{12}
C37_3	111(10)	122(13)	57(9)	12(9)	9(8)	-17(9)
C38_3	52(5)	45(7)	30(4)	6(5)	-6(4)	-1(5)
O1_4	58(5)	57(5)	29(3)	10(4)	-6(4)	1(4)
O2_4	93(8)	46(5)	24(3)	9(3)	-16(4)	5(5)
O3_4	64(6)	55(6)	59(5)	10(4)	4(5)	9(5)
O4_4	62(6)	63(6)	46(5)	3(4)	-2(4)	-5(4)
N1_4	58(6)	54(6)	42(4)	5(4)	-6(4)	5(4)
C1_4	35(7)	54(7)	55(5)	6(5)	-1(5)	14(5)
C2_4	44(8)	50(7)	56(5)	7(5)	-2(5)	7(5)
C3_4	83(11)	74(9)	62(6)	15(7)	-19(7)	-19(7)
C4_4	69(10)	70(9)	61(6)	14(6)	-14(6)	-17(7)
C5_4	71(9)	58(8)	58(6)	8(5)	-13(6)	-12(6)
C6_4	55(8)	53(7)	54(5)	3(5)	-4(5)	-1(5)
C7_4	48(8)	52(7)	52(4)	3(4)	-5(5)	3(5)
C8_4	38(7)	53(7)	48(5)	1(5)	3(5)	5(5)
C9_4	46(8)	45(7)	45(5)	1(4)	6(5)	9(5)
C10_4	49(7)	49(6)	40(5)	2(4)	3(5)	7(5)
C11_4	53(8)	48(7)	41(5)	0(4)	1(5)	9(5)
C12_4	51(8)	51(6)	54(5)	5(4)	-4(5)	4(5)
C13_4	65(8)	54(6)	41(5)	2(4)	-5(4)	2(5)
C14_4	79(8)	59(7)	44(5)	7(5)	5(5)	10(6)
C15_4	97(11)	62(7)	61(9)	1(7)	5(8)	18(7)
C16_4	90(9)	60(10)	46(7)	-7(6)	-1(6)	8(7)
C17_4	81(9)	78(9)	52(9)	8(7)	11(7)	7(7)
C18_4	72(9)	67(8)	57(6)	-1(5)	-12(6)	0(6)
C19_4	76(10)	66(8)	65(8)	-1(6)	-27(7)	-6(7)
C20_4	52(9)	83(9)	61(7)	-8(6)	-15(6)	-2(7)
C21_4	94(10)	83(9)	61(8)	-9(6)	-24(7)	-4(7)
C22_4	96(12)	79(9)	76(9)	-6(7)	-30(8)	-7(8)
C23_4	78(11)	81(8)	74(8)	-14(6)	-23(7)	0(8)
C24_4	98(8)	97(9)	78(9)	-25(6)	-39(7)	7(9)
C25_4	165(17)	110(10)	76(11)	-14(8)	-70(11)	9(12)
C26_4	98(8)	145(14)	122(13)	-68(10)	-27(9)	14(11)
C27_4	62(10)	107(10)	93(11)	-33(8)	-44(8)	8(9)
C28_4	59(9)	60(7)	50(4)	3(4)	-2(5)	6(6)
C29_4	84(11)	55(8)	52(5)	-2(5)	-13(7)	13(7)
C30_4	57(9)	52(7)	52(5)	-4(5)	-13(6)	2(6)
C31_4	88(10)	52(7)	53(5)	-2(5)	-5(6)	6(6)
C32_4	72(10)	54(7)	52(5)	-2(5)	-6(6)	3(7)
C33_4	58(9)	59(7)	53(5)	1(5)	-8(6)	4(7)
C34_4	110(10)	73(8)	54(5)	-7(6)	-3(7)	20(7)
C35_4	173(18)	104(11)	54(9)	4(8)	-11(11)	41(12)
C36_4	124(11)	162(18)	79(11)	6(12)	20(9)	34(10)
C37_4	181(17)	99(10)	69(11)	-27(9)	-12(12)	-10(11)
C38_4	66(8)	51(6)	26(4)	3(4)	-19(5)	-7(5)
O1_5	41(5)	75(6)	28(3)	1(4)	-8(3)	2(4)
O2_5	35(4)	63(6)	29(3)	0(4)	-2(3)	1(4)
O3_5	54(5)	57(6)	46(4)	1(4)	8(4)	-4(4)
O4_5	57(6)	66(6)	54(5)	9(4)	-2(4)	9(4)
N1_5	55(6)	55(6)	40(5)	7(4)	-2(4)	0(4)
C1_5	62(8)	47(8)	47(5)	11(5)	12(5)	1(5)
C2_5	39(6)	57(8)	45(4)	7(5)	2(4)	-4(5)
C3_5	35(6)	77(10)	51(5)	14(6)	4(5)	1(6)
C4_5	50(8)	65(9)	45(5)	1(6)	-3(5)	0(6)
C5_5	62(7)	56(8)	43(5)	5(5)	-2(4)	1(5)
C6_5	47(7)	47(8)	40(4)	5(5)	-5(4)	-11(5)
C7_5	54(7)	39(7)	46(4)	8(5)	4(4)	-10(5)
C8_5	50(7)	55(8)	46(5)	3(6)	3(5)	-10(5)

Atom	U_{11}	U_{22}	U_{33}	U_{23}	U_{13}	U_{12}
C9_5	54(8)	63(9)	48(5)	5(6)	3(5)	1(6)
C10_5	35(6)	58(8)	45(5)	2(5)	-5(4)	-6(5)
C11_5	52(8)	56(8)	51(5)	9(5)	3(5)	0(5)
C12_5	42(6)	63(8)	42(4)	5(5)	0(4)	-3(5)
C13_5	43(5)	56(7)	39(5)	9(4)	-10(4)	5(5)
C14_5	55(6)	70(8)	46(6)	0(5)	-14(5)	1(6)
C15_5	50(6)	82(10)	51(8)	-5(7)	-14(6)	3(6)
C16_5	87(11)	83(8)	34(6)	5(6)	-18(7)	2(8)
C17_5	37(7)	73(8)	54(8)	-7(6)	-11(6)	-13(6)
C18_5	49(6)	63(8)	42(6)	6(5)	-6(5)	-4(6)
C19_5	46(6)	87(11)	58(8)	22(6)	-8(6)	-9(7)
C20_5	28(5)	78(10)	57(7)	20(6)	-3(5)	-5(6)
C21_5	30(5)	97(10)	56(7)	30(6)	-1(5)	-4(6)
C22_5	32(6)	85(10)	54(7)	23(6)	-8(5)	-10(6)
C23_5	45(6)	81(10)	45(7)	17(6)	-1(5)	-10(6)
C24_5	52(6)	88(8)	56(7)	31(6)	-1(5)	-2(6)
C25_5	80(11)	110(11)	61(7)	33(7)	8(8)	14(9)
C26_5	61(9)	84(8)	115(13)	36(8)	-7(9)	0(7)
C27_5	56(6)	103(10)	65(8)	33(8)	-7(7)	3(8)
C28_5	68(7)	69(10)	43(4)	9(6)	2(4)	-2(7)
C29_5	69(8)	67(9)	43(5)	12(6)	4(5)	12(7)
C30_5	86(8)	68(10)	47(5)	4(7)	-1(6)	22(8)
C31_5	89(8)	98(12)	52(6)	3(7)	-9(5)	16(8)
C32_5	79(8)	114(13)	57(6)	-5(8)	-12(5)	9(9)
C33_5	67(7)	73(10)	54(6)	6(7)	-4(5)	1(7)
C34_5	138(13)	152(15)	49(6)	-9(8)	-18(8)	21(10)
C35_5	240(30)	167(16)	66(11)	6(11)	-77(14)	-8(14)
C36_5	197(18)	260(20)	84(13)	-77(14)	-48(13)	84(18)
C37_5	178(15)	190(20)	80(13)	-20(13)	-46(12)	-15(13)
C38_5	43(5)	50(7)	30(4)	-1(5)	-6(3)	10(5)
O1_7	68(6)	52(5)	28(3)	-1(4)	1(3)	-9(4)
O2_7	38(4)	59(5)	25(3)	3(3)	6(3)	-24(4)
O3_7	62(6)	67(6)	54(5)	-16(4)	2(5)	0(5)
O4_7	73(7)	61(6)	48(5)	-3(4)	6(4)	-7(4)
N1_7	75(7)	58(6)	35(4)	-2(4)	-1(4)	0(4)
C1_7	63(9)	56(8)	49(5)	-7(5)	1(5)	5(6)
C2_7	60(9)	46(7)	51(5)	-10(4)	2(5)	0(5)
C3_7	84(10)	59(8)	57(6)	-6(6)	0(6)	-16(7)
C4_7	88(11)	86(10)	62(6)	-10(6)	6(7)	-33(9)
C5_7	63(8)	55(7)	55(6)	2(5)	1(5)	1(5)
C6_7	65(8)	45(7)	45(5)	-2(4)	2(5)	6(5)
C7_7	56(8)	45(6)	53(4)	-4(4)	0(5)	8(5)
C8_7	56(8)	46(7)	43(5)	-5(5)	-5(5)	5(5)
C9_7	73(10)	44(7)	43(5)	-9(4)	-4(6)	-2(6)
C10_7	78(9)	42(6)	40(5)	-6(4)	0(5)	-5(6)
C11_7	79(9)	51(7)	39(5)	-2(4)	2(5)	-3(6)
C12_7	64(8)	38(6)	47(4)	-2(4)	0(5)	7(5)
C13_7	92(9)	42(6)	32(5)	-3(4)	-4(4)	-10(5)
C14_7	106(10)	61(7)	44(6)	3(5)	-9(5)	13(6)
C15_7	123(14)	72(8)	53(9)	-2(7)	-8(8)	28(8)
C16_7	118(11)	63(10)	44(7)	7(7)	-8(7)	7(8)
C17_7	110(11)	63(9)	53(9)	12(7)	-26(7)	12(8)
C18_7	70(9)	50(7)	60(7)	0(5)	5(6)	-8(6)
C19_7	102(12)	56(7)	62(8)	-5(6)	19(7)	1(7)
C20_7	76(10)	68(8)	64(8)	3(6)	11(6)	0(7)
C21_7	85(9)	72(8)	71(8)	7(6)	14(7)	0(7)
C22_7	105(12)	54(8)	82(10)	12(7)	33(8)	-8(7)
C23_7	67(10)	62(8)	65(8)	11(6)	7(6)	-2(7)

Atom	U_{11}	U_{22}	U_{33}	U_{23}	U_{13}	U_{12}
C24_7	96(9)	113(10)	86(10)	21(6)	30(7)	7(9)
C25_7	113(14)	118(10)	94(12)	14(8)	55(10)	8(11)
C26_7	89(8)	126(13)	119(12)	39(10)	33(9)	10(11)
C27_7	93(12)	118(11)	75(10)	19(8)	32(9)	7(10)
C28_7	53(9)	53(7)	53(4)	-1(4)	2(5)	0(6)
C29_7	61(9)	55(7)	54(5)	-2(5)	8(6)	1(7)
C30_7	95(11)	62(8)	53(5)	-3(6)	7(7)	6(7)
C31_7	67(9)	69(8)	57(5)	2(5)	5(6)	2(7)
C32_7	74(10)	72(8)	56(5)	3(5)	4(7)	10(8)
C33_7	80(10)	53(7)	56(5)	1(5)	5(7)	4(7)
C34_7	106(11)	105(11)	53(6)	7(6)	2(8)	17(9)
C35_7	218(17)	184(18)	67(12)	11(12)	38(14)	-59(17)
C36_7	175(19)	123(12)	49(9)	-2(8)	1(11)	22(11)
C37_7	163(14)	270(30)	63(11)	3(15)	-13(11)	105(16)
C38_7	60(7)	39(5)	30(4)	1(4)	2(5)	-18(5)
O1_1	54(5)	59(4)	38(4)	5(3)	-5(5)	-10(5)
C1_1	119(14)	97(11)	75(9)	41(8)	-38(10)	-5(12)
C2_1	58(8)	67(6)	39(6)	22(6)	9(5)	2(6)
C3_1	63(10)	79(7)	41(7)	-7(6)	8(6)	-7(6)
C4_1	93(10)	86(10)	51(9)	-6(8)	-5(8)	-22(8)

Compound 8c

Submitted by: **Yannick Boni**

Solved by: **John Bacsa**

R_1=11.4%

Crystal Data and Experimental

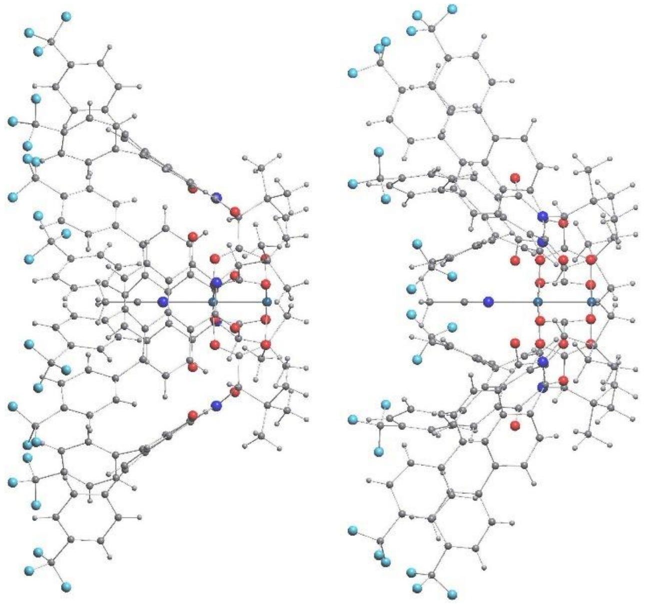

Experimental. Single green prism-shaped crystals of **Compound 8c** were chosen from the sample as supplied. A suiTable Scrystal with dimensions 0.40 × 0.27 × 0.17 mm^3 was selected and mounted on a loop with paratone on a XtaLAB Synergy-S diffractometer. The crystal was kept at a steady T = 100(1) K during data collection. The structure was solved with the ShelXT (Sheldrick, 2015) solution program and by using Olex2 1.5-dev (Dolomanov et al., 2009) and by using Olex2 1.5-alpha (Dolomanov et al., 2009) as the graphical interface. The model was refined with ShelXL 2018/3 (Sheldrick, 2015) using full matrix least squares minimisation on $\boldsymbol{F^2}$.

Crystal Data. $C_{144}H_{105}Cl_8F_{24}N_5O_{16}Rh_2$, M_r = 3106.74, tetragonal, $P42_12$ (No. 90), a = 21.47152(16) Å, b = 21.47152(16) Å, c = 31.9602(4) Å, $\alpha = \beta = \gamma =$ 90°, V = 14734.5(3) Å^3, T = 100(1) K, Z = 4, Z' = 0.5, μ(Cu K$_\alpha$) = 3.942, 87892 reflections measured, 14305 unique (R_{int} = 0.0943) which were used in all calculations. The final wR_2 was 0.3388 (all data) and R_1 was 0.1140 (I≥2 σ(I)).

**Compound**	**8c**
Formula	$C_{144}H_{105}Cl_8F_{24}N_5O_{16}Rh_2$
$D_{calc.}$/ g cm^{-3}	1.400
μ/mm^{-1}	3.942
Formula Weight	3106.74
Colour	green
Shape	prism-shaped
Size/mm^3	0.40×0.27×0.17
T/K	100(1)
Crystal System	tetragonal
Flack Parameter	0.18(2)
Hooft Parameter	-0.036(4)
Space Group	$P42_12$
a/Å	21.47152(16)
b/Å	21.47152(16)
c/Å	31.9602(4)
α/°	90
β/°	90
γ/°	90
V/Å^3	14734.5(3)
Z	4
Z'	0.5
Wavelength/Å	1.54184
Radiation type	Cu K$_\alpha$
Θ_{min}/°	2.479
Θ_{max}/°	73.994
Measured Refl's.	87892
Indep't Refl's	14305
Refl's I≥2 σ(I)	11621
R_{int}	0.0943
Parameters	809
Restraints	1134
Largest Peak	3.054
Deepest Hole	-0.858
GooF	1.439
wR_2 (all data)	0.3388
wR_2	0.3201
R_1 (all data)	0.1253
R_1	0.1140

Structure Quality Indicators

**Reflections:**	d min (CuKα) 2Θ=148.0° 0.80	I/σ(I) 20.0	Rint m=6.14 9.43%	Full 135.4° 97% to 148.0° 99.5
**Refinement:**	Shift 0.002	Max Peak 3.1	Min Peak -0.9	GooF 1.439

A green prism-shaped-shaped crystal with dimensions 0.40 × 0.27 × 0.17 mm^3 was mounted on a loop with paratone. Data were collected using a XtaLAB Synergy, Dualflex, HyPix diffractometer equipped with an Oxford Cryosystems low-temperature device operating at T = 100(1) K.

Data were measured using ω scans with Cu K_α radiation. The diffraction pattern was indexed and the total number of runs and images was based on the strategy calculation from the program CrysAlisPro system (CCD 41.108a 64-bit (release 27-04-2021)). The maximum resolution that was achieved was Θ = 73.994° (0.83 Å). The unit cell was refined using CrysAlisPro 1.171.44.57a (Rigaku OD, 2024) on 21484 reflections, 24% of the observed reflections.

Data reduction, scaling and absorption corrections were performed using CrysAlisPro 1.171.44.57a (Rigaku OD, 2024). The final completeness is 99.50 % out to 73.994° in Θ. A numerical absorption correction based on gaussian integration over a multifaceted crystal model was performed using CrysAlisPro 1.171.41.108a (Rigaku Oxford Diffraction, 2021). An empirical absorption correction using spherical harmonics, implemented in SCALE3 ABSPACK scaling algorithm was also applied. The absorption coefficient μ of this material is 3.942 mm^{-1} at this wavelength (λ = 1.54184Å) and the minimum and maximum transmissions are 0.313 and 1.000.

The structure was solved and the space group $P42_12$ (# 90) determined by the ShelXT (Sheldrick, 2015) structure solution program and refined by full matrix least squares minimisation on $\boldsymbol{F}^2$ using version 2018/3 of ShelXL 2018/3 (Sheldrick, 2015). All non-hydrogen atoms were refined anisotropically. Hydrogen atom positions were calculated geometrically and refined using the riding model. Hydrogen atom positions were calculated geometrically and refined using the riding model.

_refine_special_details: Refined as a 2-component inversion twin.

The value of Z' is 0.5. This means that only half of the formula unit is present in the asymmetric unit, with the other half consisting of symmetry equivalent atoms. The moiety formula is C130 H91 F24 N5 O16 Rh2, 1[C6], 1[H6], 4[C2], 4[Cl2], 4[H2].

The Flack parameter was refined to 0.18(2). Determination of absolute structure using Bayesian statistics on Bijvoet differences using the Olex2 results in -0.036(4). The chiral atoms in this structure are: C27(S), C27B(S). Note: The Flack parameter is used to determine chirality of the crystal studied, the value should be near 0, a value of 1 means that the stereochemistry is wrong and the model should be inverted. A value of 0.5 means that the crystal consists of a racemic mixture of the two enantiomers.

The Flack parameter was refined to 0.18(2). Determination of absolute structure using Bayesian statistics on Bijvoet differences using the Olex2 results in -0.036(4). The chiral atoms in this structure are: C27(S), C27B(S). Note: The Flack parameter is used to determine chirality of the crystal studied, the value should be near 0, a value of 1 means that the stereochemistry is wrong and the model should be inverted. A value of 0.5 means that the crystal consists of a racemic mixture of the two enantiomers.

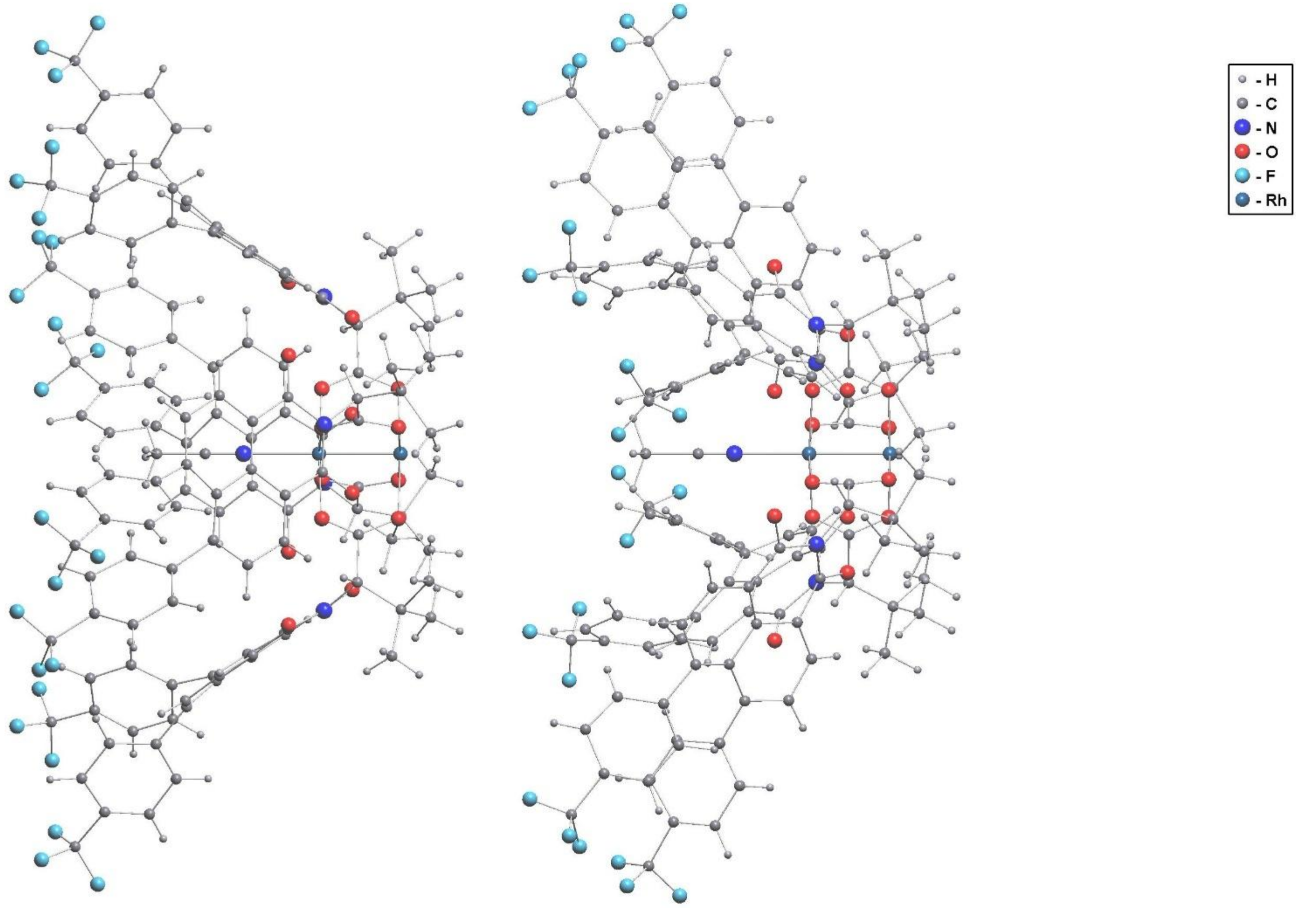

Figure S312. The symmetry expanded asymmetric unit in the crystal giving two different Rh2-complexes (but chemically equivalent) in the crystal structure. The chiral atoms in this structure have S-configuration.

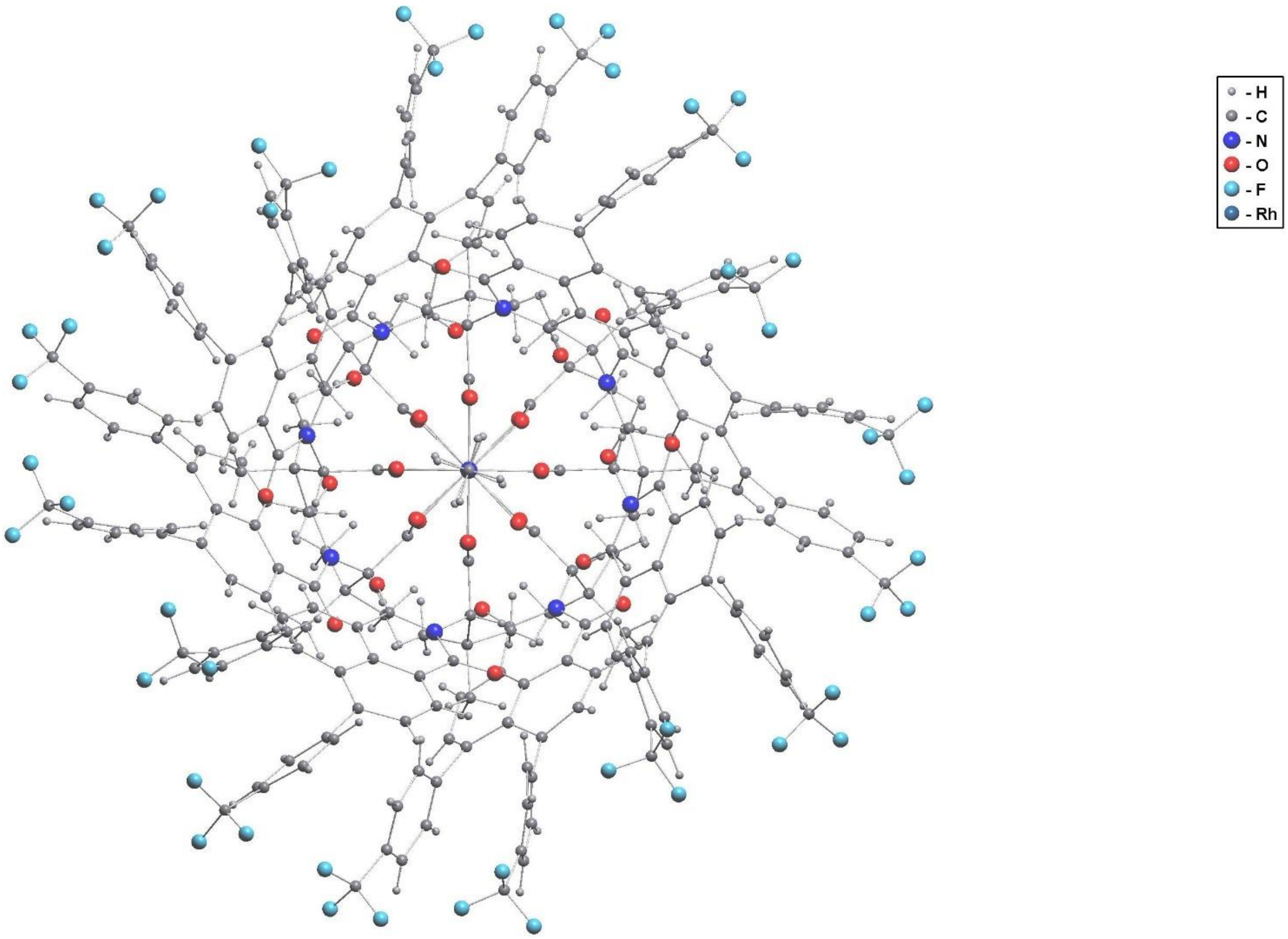

Figure S313. The symmetry expanded asymmetric unit in the crystal giving two different Rh2-complexes (but chemically equivalent) in the crystal structure (viewed along the c-axis and along the Rh2 vectors).

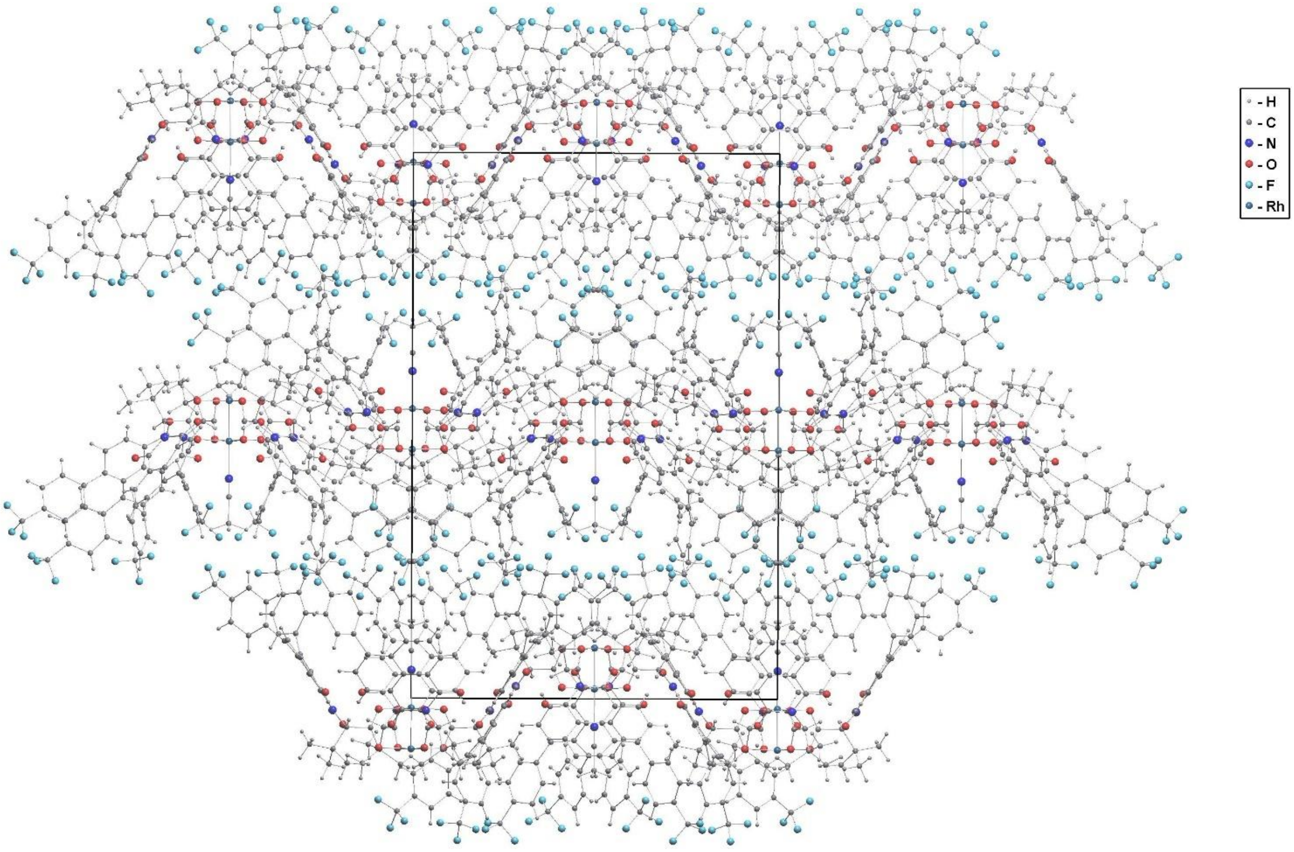

Figure S314. Molecular packing in the crystal viewed along the a-axis

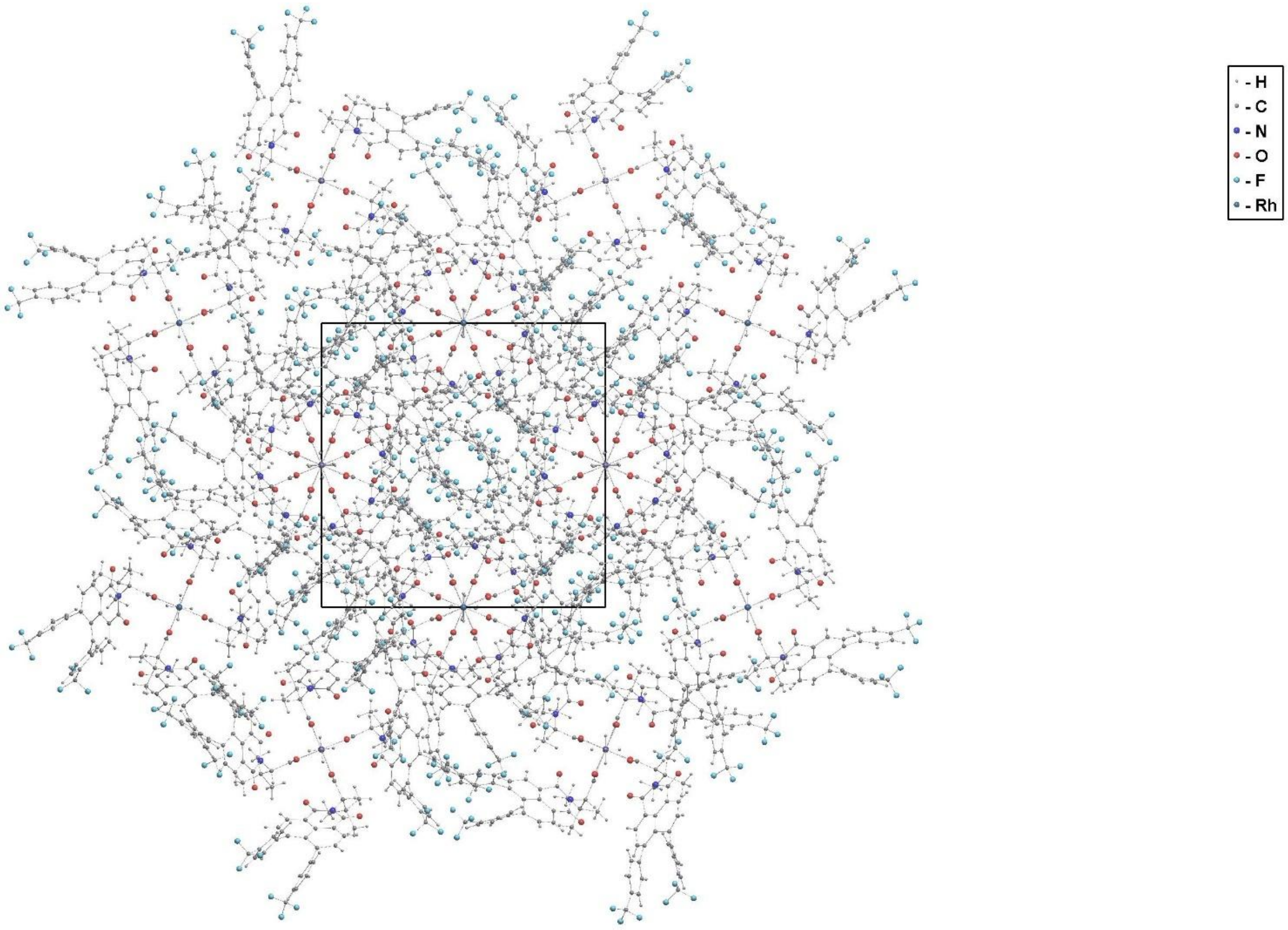

Figure S315. Molecular packing in the crystal viewed along the c-axis (along the Rh2 bonds).

Data Plots: Diffraction Data

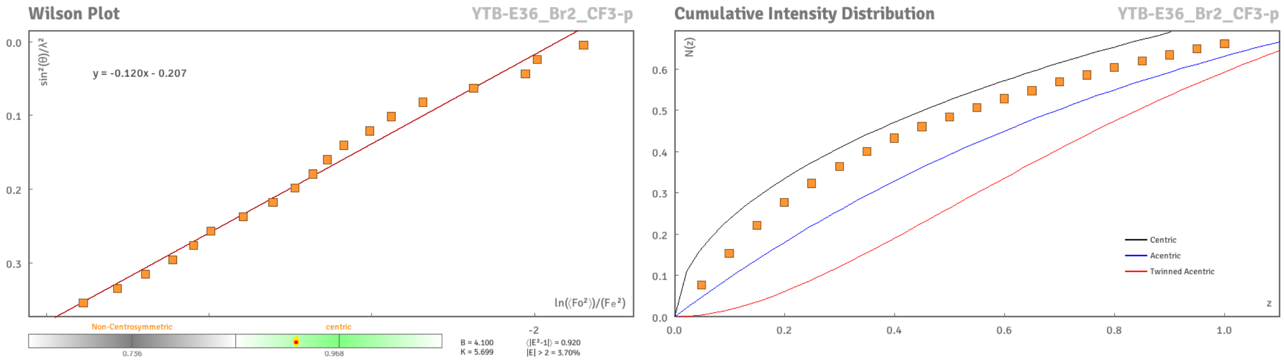

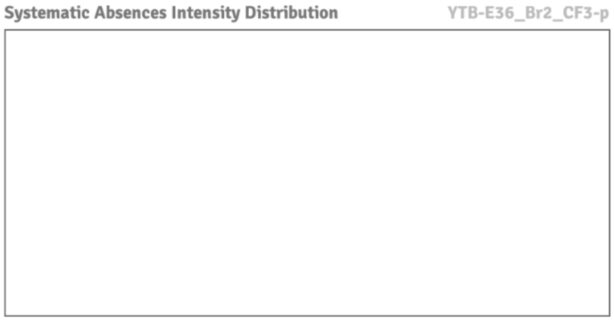

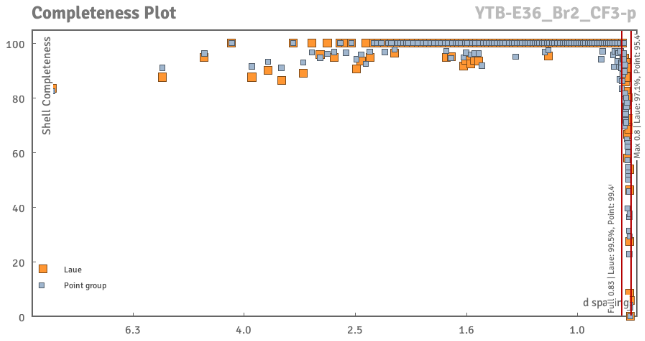

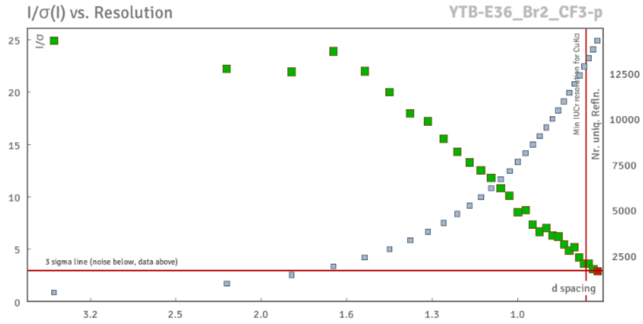

Data Plots: Refinement and Data

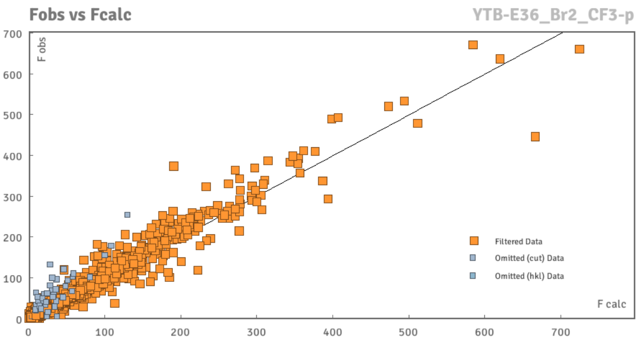

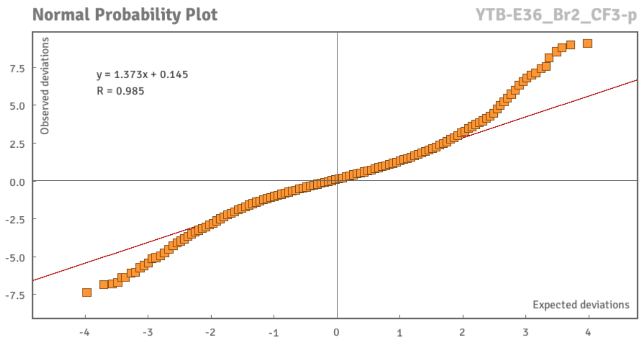

Reflection Statistics

Total reflections (after filtering)	87892	Unique reflections	14305
Completeness	0.954	Mean I/σ	11.74
hkl_{max} collected	(26, 23, 38)	hkl_{min} collected	(-26, -26, -39)
hkl_{max} used	(18, 26, 39)	hkl_{min} used	(-18, 0, 0)
Lim d_{max} collected	100.0	Lim d_{min} collected	0.77
d_{max} used	17.82	d_{min} used	0.8
Friedel pairs	10559	Friedel pairs merged	0
Inconsistent equivalents	12	R_{int}	0.0943
R_{sigma}	0.0501	Intensity transformed	0
Omitted reflections	0	Omitted by user (OMIT hkl)	320
Multiplicity	(35156, 14042, 4797, 1758, 602, 84, 5)	Maximum multiplicity	19
Removed systematic absences	0	Filtered off (Shel/OMIT)	0

Table S22. Fractional Atomic Coordinates (×10⁴) and Equivalent Isotropic Displacement Parameters (Å²×10³) for **Compound 8c.** U_{eq} is defined as 1/3 of the trace of the orthogonalised U_{ij}.

Atom	x	y	z	U_{eq}
Rh1	0	5000	5304.5(5)	87.8(6)
Rh2	0	5000	4559.1(5)	103.7(8)
Rh3	5000	10000	10178.9(4)	42.0(3)
Rh4	5000	10000	10929.9(4)	43.1(3)
C1	4226(3)	7597(4)	9893(2)	58.4(18)
C2	4557(3)	7242(4)	9567(2)	57.8(18)
C3	4204(3)	6961(5)	9261(3)	69(2)
C4	4503(3)	6618(5)	8948(3)	67(2)
C5	5148(3)	6536(4)	8959(3)	59.4(17)
O1	3657(2)	7653(3)	9898(2)	58.7(14)
C1B	5253(3)	7860(4)	10210(2)	48.9(14)
C2B	5566(2)	7525(3)	9866.9(19)	45.1(12)
C3B	6207(3)	7535(4)	9857(2)	47.2(14)
C4B	6519(3)	7256(4)	9522(2)	51.0(14)
C5B	6185(3)	6960(4)	9204.7(19)	56.5(13)
O1B	5540(2)	8139(3)	10484.6(18)	49.7(11)
C1C	1291(4)	7195(5)	5574(3)	111(3)
C2C	1825(5)	7162(6)	5862(4)	135(4)
C3C	1834(5)	7567(6)	6197(4)	134(3)
C4C	2339(5)	7534(6)	6476(3)	132(3)
C5C	2887(4)	7238(7)	6349(4)	165(4)
O1C	854(4)	7557(5)	5622(3)	123(3)
C1D	1705(5)	6291(7)	5203(5)	155(5)
C2D	2307(5)	6402(9)	5413(5)	167(6)
C3D	2809(6)	6053(11)	5281(6)	193(9)
C4D	3376(5)	6118(10)	5493(4)	167(6)
C5D	3384(5)	6366(7)	5897(3)	162(4)
O1D	1629(5)	5865(6)	4952(4)	148(4)
C6	5550(4)	6894(3)	9235(2)	47.1(12)
C11	5214(4)	7217(4)	9556(3)	49.6(14)
C6B	2880(5)	6752(8)	6040(4)	180(6)
C11B	2328(5)	6749(8)	5789(4)	161(5)
C13	5379(4)	6026(3)	8678(2)	68.1(17)
C14	5722(4)	5549(3)	8863(2)	65.1(17)
C15	5971(4)	5067(3)	8618(2)	69.6(18)
C16	5896(6)	5096(5)	8204(3)	120(3)
C17	5523(6)	5557(5)	7993(2)	152(7)
C18	5289(8)	6035(6)	8247(2)	111(5)
C19	6255(4)	4622(3)	7931(2)	123(2)
F1	6410(5)	4106(3)	8139(2)	128(2)
F2	6776(4)	4824(4)	7749(3)	131(2)
F3	5878(4)	4441(4)	7622(3)	136(2)
C20B	3963(4)	6179(5)	6128(3)	159(5)
C21B	4544(5)	6276(11)	5945(4)	190(9)
C22B	5092(4)	6155(14)	6170(5)	261(18)
C23B	5041(6)	5916(7)	6554(4)	567(12)
C24B	4457(6)	5827(15)	6769(5)	430(40)
C25B	3922(5)	5908(10)	6523(5)	215(12)
C26B	5644(6)	5721(8)	6783(5)	562(10)
F4B	5918(10)	6194(11)	6983(7)	591(12)
F5B	5594(9)	5261(11)	7063(7)	570(10)
F6B	6051(8)	5525(14)	6496(6)	570(11)
C13B	3423(4)	7317(7)	6645(3)	184(8)
C14B	3992(5)	7549(7)	6505(3)	136(4)

Atom	x	y	z	U_{eq}
C15B	4486(7)	7640(17)	6786(4)	227(16)
C16B	4377(6)	7561(11)	7194(4)	289(14)
C17B	3774(7)	7405(18)	7365(3)	350(20)
C18B	3327(7)	7214(15)	7071(3)	199(12)
C19B	4940(6)	7551(7)	7494(4)	304(8)
F1B	5233(9)	8098(8)	7516(6)	381(13)
F2B	5379(8)	7125(10)	7420(5)	305(9)
F3B	4727(7)	7435(11)	7879(3)	288(10)
C20	6577(3)	6840(4)	8825.1(18)	63.5(15)
C21	7022(4)	6367(4)	8832(2)	63.0(17)
C22	7340(4)	6201(4)	8465(2)	71(2)
C23	7188(7)	6489(7)	8110(3)	188(4)
C24	6682(7)	6927(9)	8071(2)	117(5)
C25	6402(6)	7110(6)	8448(2)	109(5)
C26	7563(4)	6329(5)	7711(3)	173(4)
F4	7959(5)	6779(5)	7600(3)	191(4)
F5	7895(5)	5803(5)	7719(3)	177(4)
F6	7161(5)	6270(6)	7395(3)	182(4)
C27	4247(3)	8239(3)	10533(2)	53.4(10)
C28	4544(3)	8891(3)	10543(3)	50.6(15)
C29	4139(3)	7866(3)	10936(2)	59.9(12)
C30	3677(4)	8258(4)	11192(3)	63.9(16)
C31	4717(4)	7759(5)	11211(3)	61.7(16)
C32	3833(5)	7231(3)	10850(3)	63.3(16)
N1	4602(3)	7853(3)	10226(2)	51.8(10)
O3	4650(2)	9111(3)	10205.2(18)	44.8(12)
O4	4637(3)	9112(3)	10910.3(19)	49.1(13)
C27B	704(5)	6767(4)	4940(3)	119.2(18)
C28B	416(6)	6110(5)	4937(3)	118(3)
C29B	898(5)	7088(4)	4530(3)	122(2)
C30B	286(6)	7187(8)	4290(4)	136(3)
C31B	1336(8)	6711(7)	4245(4)	137(4)
C32B	1192(9)	7730(5)	4607(4)	129(3)
N1B	1241(5)	6763(4)	5238(2)	114(2)
O3B	393(5)	5866(4)	5275(2)	93(2)
O4B	354(6)	5871(5)	4571(2)	110(3)
C33_3	0	5000	6327(7)	118(11)
C34_3	0	5000	6811(7)	124(13)
N2_3	0	5000	5993(4)	71(5)
C33B_3	5000	10000	9130(9)	66(6)
C34B_3	5000	10000	8684(6)	66(5)
N2B_3	5000	10000	9484(5)	48(3)

Table S23. Anisotropic Displacement Parameters (×10^4) for **Compound 8c**. The anisotropic displacement factor exponent takes the form: $-2\pi^2[h^2a^{*2} \times U_{11} + ... + 2hka^* \times b^* \times U_{12}]$

Atom	U_{11}	U_{22}	U_{33}	U_{23}	U_{13}	U_{12}
Rh1	112.4(10)	112.4(10)	38.8(7)	0	0	0
Rh2	135.0(12)	135.0(12)	41.1(7)	0	0	0
Rh3	28.4(4)	28.4(4)	69.3(7)	0	0	0
Rh4	30.8(4)	30.8(4)	67.7(7)	0	0	0
C1	41.3(14)	47(4)	86(2)	-12(3)	-7.0(12)	-2.5(14)
C2	44.0(14)	46(4)	83(2)	-11(3)	-8.2(12)	-1.6(13)
C3	47.6(18)	67(5)	93(3)	-24(3)	-10.7(16)	-2.1(18)
C4	52.7(18)	59(4)	89(3)	-19(3)	-9.8(17)	-2.6(17)

Atom	U_{11}	U_{22}	U_{33}	U_{23}	U_{13}	U_{12}
C5	52.8(18)	48(3)	77(3)	-9(3)	-6.5(16)	-2.5(15)
O1	41.0(14)	44(3)	92(3)	-9(3)	-7.0(13)	-3.4(14)
C1B	40.1(13)	36(3)	71(2)	-1(2)	-3.8(12)	-3.6(12)
C2B	41.9(14)	28(3)	65.8(18)	5.2(18)	-5.3(12)	-0.6(13)
C3B	42.0(14)	32(3)	67(2)	1(2)	-4.6(12)	-0.2(14)
C4B	45.3(17)	39(3)	69(2)	-1(2)	-3.8(13)	2.3(16)
C5B	49.4(15)	49(3)	71.4(19)	-7(2)	-2.6(12)	-1.5(14)
O1B	39.6(19)	39(2)	70(2)	-2.0(17)	-2.3(17)	-5.3(18)
C1C	157(5)	107(5)	70(3)	11(3)	40(3)	-26(4)
C2C	185(5)	110(7)	111(5)	16(5)	5(4)	-33(5)
C3C	194(6)	103(7)	106(5)	22(5)	7(4)	-40(5)
C4C	194(6)	97(7)	106(5)	23(5)	7(4)	-41(5)
C5C	202(5)	162(9)	130(5)	-10(6)	-4(4)	-17(5)
O1C	166(6)	114(5)	88(5)	5(4)	41(4)	-19(5)
C1D	187(5)	151(7)	127(9)	-37(6)	-7(5)	16(5)
C2D	195(5)	164(12)	144(7)	-18(8)	-18(5)	6(6)
C3D	202(6)	209(16)	166(10)	-43(12)	-29(6)	25(8)
C4D	197(6)	165(15)	139(6)	-7(8)	-17(5)	10(7)
C5D	197(5)	152(10)	136(5)	-1(5)	-9(4)	-9(6)
O1D	193(10)	140(6)	112(7)	-25(5)	13(6)	4(6)
C6	48.8(15)	27(2)	65.3(19)	6.0(18)	-6.5(12)	0.9(13)
C11	44.2(14)	35(3)	69.9(19)	0(2)	-6.7(11)	-0.9(12)
C6B	205(5)	181(10)	153(7)	-31(7)	-22(5)	4(6)
C11B	198(5)	148(10)	138(6)	-8(7)	-12(5)	-10(6)
C13	57(3)	56(3)	91(3)	-21(2)	-3(2)	-6(2)
C14	48(4)	54(3)	93(3)	-17(2)	4(3)	-9(3)
C15	58(4)	54(3)	97(3)	-16(2)	12(3)	-9(3)
C16	161(7)	101(4)	97(3)	-24(2)	3(3)	51(5)
C17	227(15)	132(8)	96(3)	-37(3)	-20(5)	91(10)
C18	141(12)	101(6)	92(3)	-28(2)	-14(3)	46(7)
C19	170(5)	95(4)	104(3)	-26(2)	15(3)	42(3)
F1	173(6)	96(4)	117(4)	-22(3)	24(4)	46(4)
F2	172(5)	104(5)	118(5)	-17(3)	21(3)	45(4)
F3	181(6)	116(5)	111(3)	-38(3)	10(4)	41(4)
C20B	196(4)	115(13)	166(8)	17(9)	-18(5)	-30(7)
C21B	197(5)	160(20)	209(12)	64(13)	-8(6)	-17(8)
C22B	196(5)	360(50)	228(13)	120(20)	-6(7)	-9(11)
C23B	198(6)	1150(30)	354(12)	434(16)	-21(8)	-30(13)
C24B	201(7)	820(100)	254(14)	260(30)	-35(7)	-62(13)
C25B	198(6)	250(30)	192(10)	78(14)	-21(7)	-28(13)
C26B	207(7)	1150(20)	329(13)	389(13)	-33(8)	-23(13)
F4B	260(20)	1180(20)	333(17)	373(19)	-29(14)	-53(19)
F5B	194(14)	1180(20)	341(18)	410(16)	-45(12)	-23(15)
F6B	209(13)	1180(30)	321(15)	386(16)	-41(12)	-9(17)
C13B	210(6)	210(20)	132(5)	4(7)	-8(4)	-38(8)
C14B	192(6)	88(10)	129(7)	-1(8)	-6(5)	10(7)
C15B	230(8)	310(40)	144(7)	64(12)	-31(6)	-84(15)
C16B	265(9)	460(40)	143(6)	68(12)	-38(6)	-164(14)
C17B	292(10)	610(60)	136(6)	13(10)	-18(5)	-220(20)
C18B	224(8)	240(30)	131(5)	5(8)	-11(5)	-69(15)
C19B	327(10)	351(17)	234(10)	61(14)	-113(8)	-155(11)
F1B	440(30)	376(17)	330(30)	109(19)	-210(20)	-209(18)
F2B	372(16)	392(18)	150(10)	123(13)	-82(11)	-107(14)
F3B	268(15)	360(30)	234(10)	97(15)	-120(10)	-42(19)
C20	56(2)	62(4)	73(2)	-9.3(19)	0.7(17)	0(2)
C21	54(3)	56(4)	79(3)	-14(2)	2(2)	-4(3)
C22	54(4)	81(5)	79(3)	-19(3)	0(2)	1(3)
C23	184(7)	288(9)	93(3)	40(4)	45(3)	155(7)

Atom	U_{11}	U_{22}	U_{33}	U_{23}	U_{13}	U_{12}
C24	95(7)	177(11)	79(2)	15(4)	14(3)	54(8)
C25	90(7)	157(9)	80(2)	20(4)	15(3)	51(7)
C26	162(6)	266(8)	92(3)	18(4)	39(3)	113(5)
F4	173(6)	270(8)	128(6)	12(5)	45(4)	103(6)
F5	178(6)	269(8)	83(4)	13(5)	37(5)	121(6)
F6	182(6)	261(10)	104(3)	19(5)	24(4)	111(6)
C27	43(2)	35.1(18)	81.8(19)	-3.9(14)	2.7(16)	-6.4(14)
C28	35(3)	33.6(19)	83(5)	-3.4(18)	1(3)	-2.5(19)
C29	51(2)	42(2)	86(2)	2.0(18)	6.3(17)	-5.1(18)
C30	57(3)	40(3)	95(3)	9(3)	18(3)	-4(2)
C31	52(3)	48(4)	85(3)	7(3)	6(2)	-8(2)
C32	58(4)	42(2)	90(5)	5(2)	4(3)	-8(2)
N1	39.9(13)	36(2)	79.6(18)	-5.4(17)	-3.3(11)	-4.5(12)
O3	31(3)	39(3)	65(3)	-8(3)	-6(2)	-1(2)
O4	38(3)	39(3)	71(4)	0(3)	-2(3)	5(3)
C27B	158(4)	121(4)	79(3)	9(3)	27(3)	-18(3)
C28B	164(8)	121(4)	68(3)	8(3)	18(3)	-20(4)
C29B	162(5)	127(5)	77(3)	9(4)	26(3)	-23(4)
C30B	164(6)	142(8)	104(6)	41(7)	18(4)	-36(6)
C31B	191(9)	129(6)	91(6)	17(5)	47(6)	-14(6)
C32B	175(9)	129(5)	85(7)	12(5)	27(5)	-29(6)
N1B	155(4)	112(4)	75(3)	6(3)	31(3)	-19(3)
O3B	95(6)	115(5)	68(3)	7(3)	24(3)	-1(4)
O4B	133(8)	129(6)	69(3)	5(3)	18(4)	-9(5)
C33_3	156(18)	156(18)	41(10)	0	0	0
C34_3	160(20)	160(20)	47(11)	0	0	0
N2_3	97(8)	97(8)	18(5)	0	0	0
C33B_3	38(5)	38(5)	120(20)	0	0	0
C34B_3	70(7)	70(7)	58(9)	0	0	0
N2B_3	34(4)	34(4)	75(9)	0	0	0

Compound 8d

Submitted by: **Duc Ly**

Solved by: **John Bacsa**

R_1=6.88%

Crystal Data and Experimental

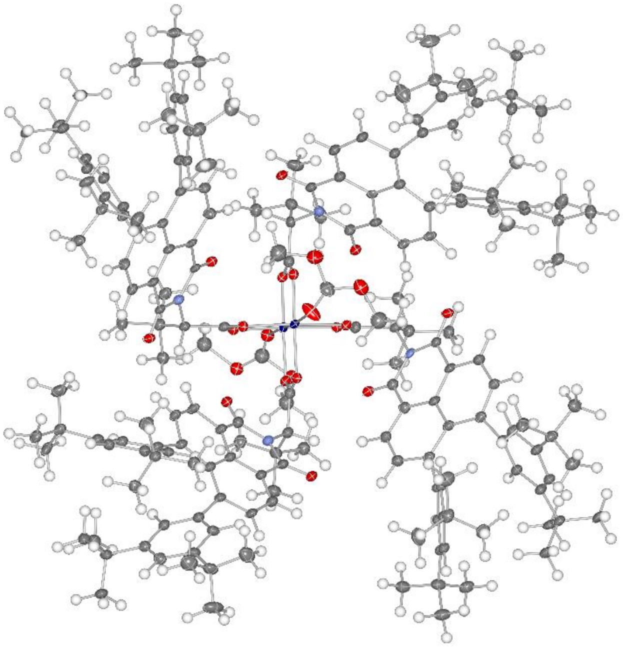

Experimental. Single green plate-shaped crystals of DY-65 were recrystallized from a mixture of heptane and DMC by solvent layering. A suiTable Scrystal with dimensions 0.17 × 0.05 × 0.04 mm³ was selected and mounted on a XtaLAB Synergy-S diffractometer. The crystal was kept at a steady T = 100(1) K during data collection. The structure was solved with the Superflip (Palatinus et al., 2012) solution program using iterative methods and by using Olex2 1.5-alpha (Dolomanov et al., 2009) as the graphical interface. The model was refined with ShelXL 2018/3 (Sheldrick, 2015) using full matrix least squares minimisation on $\boldsymbol{F}^2$.

Crystal Data. $C_{196}H_{248}N_4O_{28}Rh_2$, M_r = 3313.79, triclinic, P1 (No. 1), a = 9.4592(2) Å, b = 22.1361(4) Å, c = 23.1216(5) Å, α = 69.9035(17)°, β = 89.4550(17)°, γ = 78.1332(16)°, V = 4439.65(16) Å³, T = 100.00(10) K, Z = 1, Z' = 1, μ(Cu K_α) = 2.063, 67285 reflections measured, 22203 unique (R_{int} = 0.0632) which were used in all calculations. The final wR_2 was 0.1867 (all data) and R_1 was 0.0688 (I≥2 σ(I)).

**Compound**	Compound 8d
Formula	$C_{196}H_{248}N_4O_{28}Rh_2$
$D_{calc.}$/ g cm^{-3}	1.239
μ/mm^{-1}	2.063
Formula Weight	3313.79
Colour	green
Shape	plate-shaped
Size/mm³	0.17×0.05×0.04
T/K	100.00(10)
Crystal System	triclinic
Flack Parameter	0.154(10)
Hooft Parameter	0.016(5)
Space Group	P1
a/Å	9.4592(2)
b/Å	22.1361(4)
c/Å	23.1216(5)
α/°	69.9035(17)
β/°	89.4550(17)
γ/°	78.1332(16)
V/Å³	4439.65(16)
Z	1
Z'	1
Wavelength/Å	1.54184
Radiation type	Cu K_α
Θ_{min}/°	2.408
Θ_{max}/°	74.296
Measured Refl's.	67285
Indep't Refl's	22203
Refl's I≥2 σ(I)	19777
R_{int}	0.0632
Parameters	1834
Restraints	3060
Largest Peak	1.680
Deepest Hole	-1.301
GooF	1.058
wR_2 (all data)	0.1867
wR_2	0.1809
R_1 (all data)	0.0753
R_1	0.0688

Structure Quality Indicators

**Reflections:**	d min (CuKα) 2Θ=148.6° 0.80	I/σ(I) 15.9	Rint m=3.03 6.32%	Full 135.4° 93% to 148.6° 97.0
**Refinement:**	Shift -0.001	Max Peak 1.7	Min Peak -1.3	GooF 1.058

A green plate-shaped-shaped crystal with dimensions 0.17 × 0.05 × 0.04 mm^3 was mounted. Data were collected using a XtaLAB Synergy, Dualflex, HyPix diffractometer equipped with an Oxford Cryosystems low-temperature device operating at T = 100.00(10) K.

Data were measured using ω scans with Cu K_α radiation. The diffraction pattern was indexed and the total number of runs and images was based on the strategy calculation from the program CrysAlisPro system (CCD 43.92a 64-bit (release 05-10-2023)). The maximum resolution that was achieved was Θ = 74.296° (0.80 Å). The unit cell was refined using CrysAlisPro 1.171.43.121a (Rigaku OD, 2024) on 21747 reflections, 32% of the observed reflections.

Data reduction, scaling and absorption corrections were performed using CrysAlisPro 1.171.43.121a (Rigaku OD, 2024). The final completeness is 97.60 % out to 74.296° in Θ. A numerical absorption correction based on gaussian integration over a multifaceted crystal model was performed using CrysAlisPro 1.171.41.108a (Rigaku Oxford Diffraction, 2021). An empirical absorption correction using spherical harmonics, implemented in SCALE3 ABSPACK scaling algorithm was also applied. The absorption coefficient μ of this material is 2.063 mm^{-1} at this wavelength (λ = 1.54184Å) and the minimum and maximum transmissions are 0.696 and 1.000.

The structure was solved and the space group P1 (# 1) determined by the Superflip (Palatinus & Chapuis, 2007;Palatinus & van der Lee, 2008;Palatinus et al., 2012) structure solution program using using iterative methods and refined by full matrix least squares minimisation on $\boldsymbol{F}^2$ using version 2018/3 of ShelXL 2018/3 (Sheldrick, 2015). All non-hydrogen atoms were refined anisotropically. Hydrogen atom positions were calculated geometrically and refined using the riding model. Hydrogen atom positions were calculated geometrically and refined using the riding model.

_refine_special_details: Refined as a 2-component inversion twin.

There is a single formula unit in the asymmetric unit, which is represented by the reported sum formula. In other words: Z is 1 and Z' is 1. The moiety formula is C190 H236 N4 O22 Rh1.832, 0.084(Rh2), 2[C3], 2[O3], 2[H6].

The Flack parameter was refined to 0.154(10). Determination of absolute structure using Bayesian statistics on Bijvoet differences using the Olex2 results in 0.016(5). The chiral atoms in this structure are: C2_1(S), C2_2(S), C2_3(S), C2_4(S). Note: The Flack parameter is used to determine chirality of the crystal studied, the value should be near 0, a value of 1 means that the stereochemistry is wrong and the model should be inverted. A value of 0.5 means that the crystal consists of a racemic mixture of the two enantiomers.

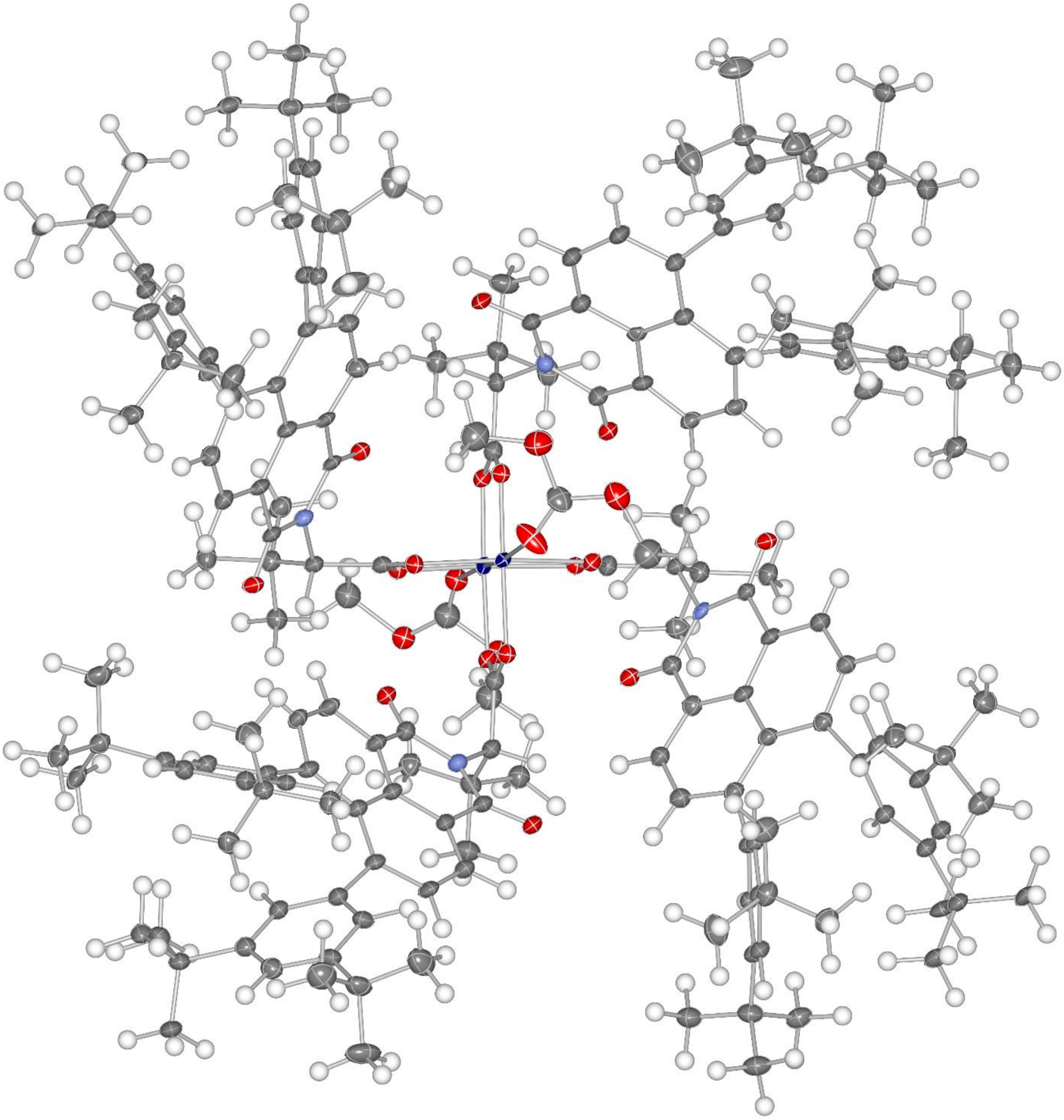

Figure S316

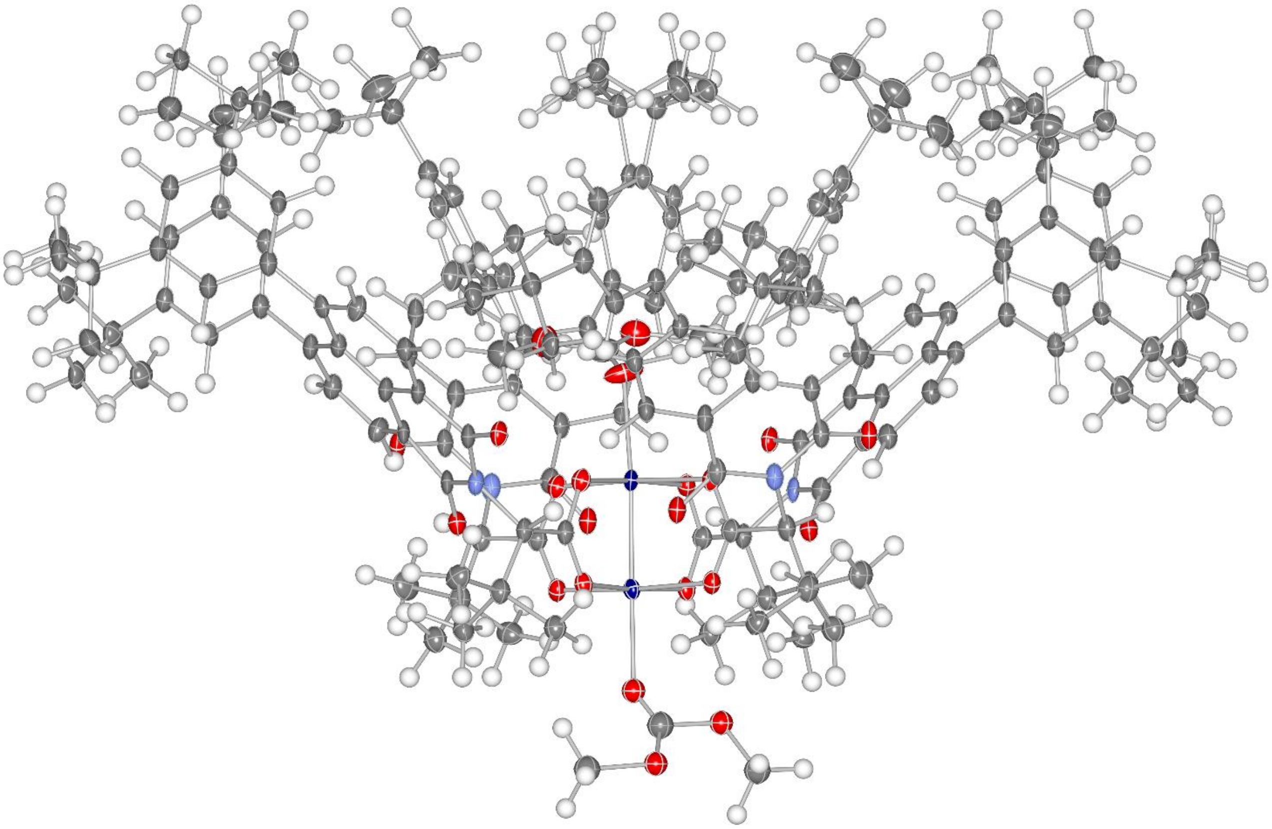

Figure S317

Data Plots: Diffraction Data

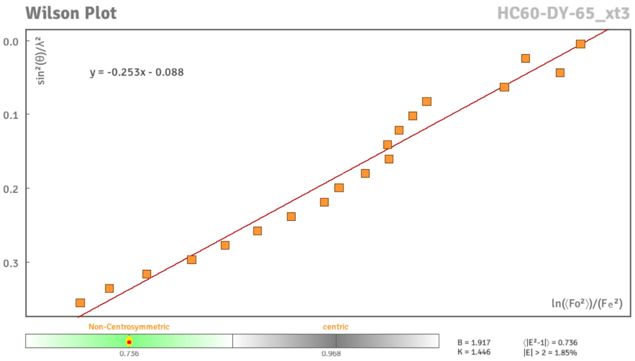

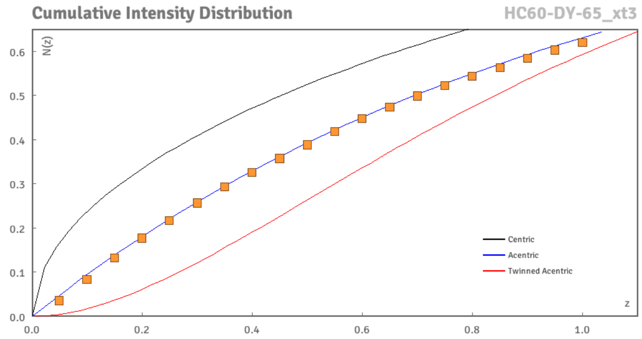

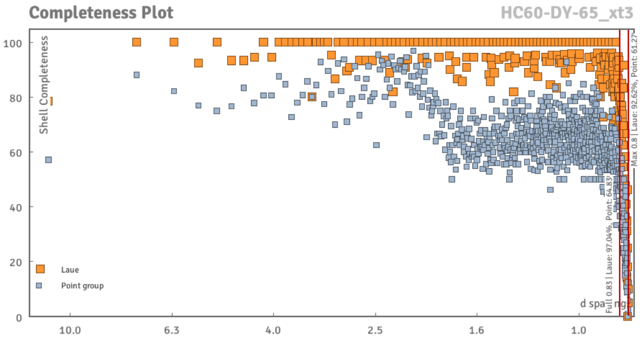

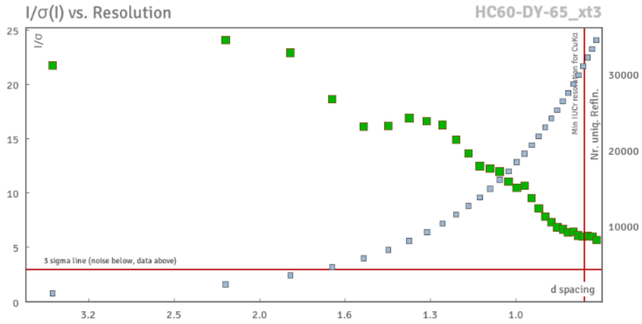

Data Plots: Refinement and Data

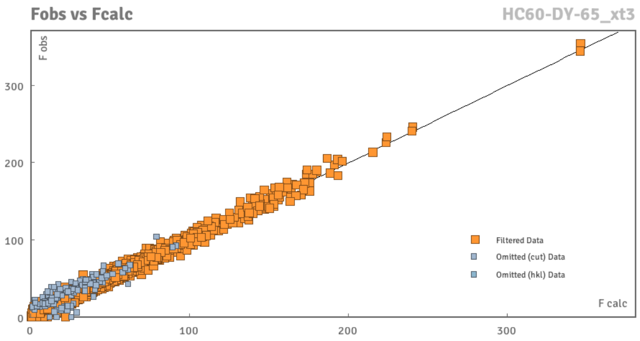

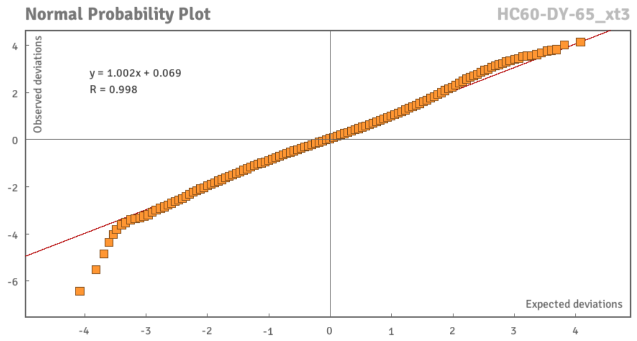

Reflection Statistics

Total reflections (after filtering)	67285	Unique reflections	22203
Completeness	0.613	Mean I/σ	12.63
hkl_{max} collected	(11, 22, 27)	hkl_{min} collected	(-11, -26, -28)
hkl_{max} used	(11, 22, 27)	hkl_{min} used	(-11, -26, -28)
Lim d_{max} collected	100.0	Lim d_{min} collected	0.77
d_{max} used	18.35	d_{min} used	0.8
Friedel pairs	5428	Friedel pairs merged	0
Inconsistent equivalents	94	R_{int}	0.0632
R_{sigma}	0.0629	Intensity transformed	0
Omitted reflections	0	Omitted by user (OMIT hkl)	878
Multiplicity	(6810, 5209, 2967, 2196, 1903, 1383, 978, 540, 263, 117, 52, 9, 3, 0, 1)	Maximum multiplicity	15
Removed systematic absences	0	Filtered off (Shel/OMIT)	0

Table S24. Fractional Atomic Coordinates (×10^4) and Equivalent Isotropic Displacement Parameters (Å^2×10^3) for **Compound 8d**. U_{eq} is defined as 1/3 of the trace of the orthogonalised U_{ij}.

Atom	x	y	z	U_{eq}
Rh1	4073.8	1796	7545.22	20.41(16)
Rh2	6554.2(8)	1840.0(3)	7480.2(3)	21.88(16)
Rh2B	5377(10)	1859(4)	7536(3)	21.1(15)
Rh1B	2964(9)	1885(3)	7455(3)	27.2(19)
O1_1	6363(5)	1847(3)	6582(2)	30.3(6)
O2_1	4007(5)	1802(3)	6634(2)	30.6(5)
O3_1	3009(6)	1657(2)	5011(2)	36.6(11)
O4_1	4047(5)	3116(2)	5843(2)	33.1(6)
N1_1	3625(5)	2397(2)	5397(2)	32.7(8)
C1_1	5155(6)	1864(4)	6360(2)	30.9(4)
C2_1	5037(6)	1933(2)	5674(2)	33.8(7)
C3_1	6344(6)	2083(3)	5290(2)	36.6(7)
C4_1	7584(6)	1474(3)	5543(3)	39.8(9)
C5_1	6826(8)	2702(3)	5298(3)	38.9(14)
C6_1	5955(8)	2172(4)	4618(2)	43.2(15)
C7_1	2717(6)	2203(2)	5052(3)	33.2(6)
C8_1	1417(6)	2701(3)	4721(3)	31.3(11)
C9_1	624(7)	2569(3)	4305(3)	35.0(13)
C10_1	-548(8)	3044(3)	3961(3)	36.4(14)
C11_1	-1038(6)	3644(2)	4052(2)	30.5(10)

Atom	x	y	z	U_{eq}
C12_1	3226(6)	2974(3)	5533(3)	33.6(12)
C13_1	1788(6)	3394(3)	5291(3)	34.8(13)
C14_1	1324(7)	3923(3)	5478(3)	36.7(14)
C15_1	-1(7)	4356(3)	5232(3)	34.2(13)
C16_1	-809(6)	4311(2)	4754(3)	31.9(11)
C17_1	939(6)	3296(3)	4846(3)	31.5(11)
C18_1	-336(6)	3768(3)	4536(3)	32.5(5)
C19_1	-2124(6)	4132(2)	3563(2)	32.1(4)
C20_1	-3323(7)	3947(3)	3392(3)	34.5(13)
C21_1	-4290(6)	4386(3)	2890(3)	38.7(13)
C22_1	-4016(7)	5004(3)	2605(3)	37.5(14)
C23_1	-2800(6)	5202(2)	2760(3)	34.5(10)
C24_1	-1870(7)	4764(3)	3250(3)	32.2(4)
C25_1	-5576(5)	4174(3)	2672(2)	46.8(13)
C26_1	-5825(9)	3505(3)	3106(4)	56.7(19)
C27_1	-5287(10)	4130(5)	2030(3)	60(2)
C28_1	-7015(6)	4670(4)	2619(4)	55.1(17)
C29_1	-2595(5)	5905(2)	2410(2)	35.2(11)
C30_1	-1155(6)	6010(3)	2621(3)	38.5(14)
C31_1	-3865(7)	6395(3)	2532(3)	38.6(14)
C32_1	-2576(9)	6021(3)	1715(2)	38.7(15)
C33_1	-2123(5)	4853(2)	4545(3)	32.1(4)
C34_1	-1952(5)	5491(2)	4443(4)	34.4(13)
C35_1	-3159(6)	6025(2)	4316(3)	35.0(11)
C36_1	-4508(6)	5906(2)	4266(4)	33.6(12)
C37_1	-4721(5)	5264(2)	4347(3)	34.0(10)
C38_1	-3520(5)	4743(2)	4486(3)	29.8(7)
C39_1	-2937(6)	6703(2)	4290(3)	36.0(11)
C40_1	-1638(8)	6881(3)	3914(4)	42.0(15)
C41_1	-2646(9)	6675(4)	4951(3)	46.4(17)
C42_1	-4271(7)	7250(3)	4001(4)	40.8(14)
C43_1	-6276(5)	5176(3)	4282(3)	34.6(7)
C44_1	-6303(7)	4461(3)	4367(4)	38.6(14)
C45_1	-6932(8)	5614(4)	3634(3)	40.7(14)
C46_1	-7202(8)	5379(4)	4766(3)	42.1(15)
O1_2	7056(5)	818(2)	7788(3)	28.4(6)
O2_2	4687(5)	792(2)	7828(3)	29.4(10)
O3_2	4590(6)	-1089(3)	8584.5(16)	33.8(7)
O4_2	5884(5)	354(2)	6838(2)	32.5(6)
N1_2	5378(5)	-413(2)	7710.9(18)	29.1(8)
C1_2	6020(6)	536(2)	7871(3)	30.9(4)
C2_2	6368(6)	-224(2)	8091(2)	30.0(4)
C3_2	7963(6)	-584(2)	8146(2)	33.2(6)
C4_2	8734(7)	-457(3)	8661(3)	34.7(8)
C5_2	8737(7)	-374(4)	7545(3)	35.1(13)
C6_2	8036(7)	-1328(2)	8350(3)	37.0(13)
C7_2	4510(7)	-848(3)	8022(2)	30.1(11)
C8_2	3543(7)	-1028(3)	7638(2)	30.5(5)
C9_2	2848(8)	-1527(3)	7920(2)	32.8(13)
C10_2	2001(8)	-1732(3)	7568(2)	32.6(13)
C11_2	1797(7)	-1446(3)	6925(2)	31.9(11)
C12_2	5176(7)	-57(3)	7077(2)	31.4(12)
C13_2	4075(7)	-191(3)	6721(2)	32.4(12)
C14_2	3801(8)	195(3)	6108(2)	33.5(13)
C15_2	2828(8)	58(3)	5743(2)	34.4(13)
C16_2	2201(7)	-487(3)	5967(2)	31.8(11)
C17_2	3328(7)	-700(3)	6984(2)	31.3(5)
C18_2	2411(7)	-885(3)	6616(2)	30.8(12)

Atom	x	y	z	U_{eq}
C19_2	980(6)	-1785(3)	6630(2)	32.1(4)
C20_2	-371(6)	-1888(4)	6827(3)	34.7(13)
C21_2	-1090(6)	-2278(3)	6609(3)	35.0(11)
C22_2	-406(6)	-2534(3)	6190(3)	32.8(6)
C23_2	983(6)	-2464(3)	6002(3)	35.0(11)
C24_2	1649(7)	-2070(3)	6211(3)	32.2(4)
C25_2	-2494(5)	-2462(2)	6886(2)	37.6(12)
C26_2	-3645(7)	-1866(3)	6901(4)	43.5(15)
C27_2	-2101(9)	-2949(4)	7550(3)	44.6(16)
C28_2	-3228(7)	-2794(4)	6524(3)	43.3(16)
C29_2	1711(5)	-2825(2)	5579(2)	38.9(11)
C30_2	3310(6)	-2779(4)	5516(4)	42.4(14)
C31_2	886(8)	-2513(4)	4940(3)	43.1(15)
C32_2	1657(9)	-3559(3)	5857(3)	43.2(15)
C33_2	1343(6)	-571(3)	5474(2)	32.1(4)
C34_2	2044(6)	-602(4)	4949(2)	32.0(12)
C35_2	1273(6)	-583(4)	4420(2)	36.3(11)
C36_2	-190(6)	-542(4)	4437(2)	32.8(6)
C37_2	-940(5)	-514(4)	4967(2)	35.2(11)
C38_2	-162(5)	-525(4)	5480(2)	31.1(12)
C39_2	2081(6)	-570(3)	3836(2)	39.1(10)
C40_2	3331(7)	-1177(3)	3976(3)	41.0(14)
C41_2	2701(8)	53(3)	3613(3)	41.8(9)
C42_2	1077(7)	-554(4)	3315(3)	43.2(15)
C43_2	-2583(5)	-476(3)	4956(3)	41.2(11)
C44_2	-3192(7)	-439(4)	5562(3)	43.2(15)
C45_2	-2872(8)	-1098(3)	4875(4)	43.0(15)
C46_2	-3371(8)	136(4)	4417(3)	49.1(17)
O1_3	6615(5)	1815(3)	8350(2)	30.3(6)
O2_3	4258(5)	1775(3)	8398(2)	30.6(5)
O3_3	3264(6)	1981(2)	10011(3)	37.8(12)
O4_3	5694(5)	503(2)	9189(2)	33.1(6)
N1_3	4576(5)	1234(2)	9624(2)	32.3(9)
C1_3	5476(7)	1768(4)	8620(2)	30.9(4)
C2_3	5507(6)	1707(2)	9305(2)	33.0(7)
C3_3	6982(6)	1582(3)	9649(2)	36.6(7)
C4_3	7600(7)	2201(3)	9364(3)	39.8(9)
C5_3	8068(7)	968(3)	9633(4)	40.3(14)
C6_3	6736(8)	1501(4)	10328(2)	46.5(15)
C7_3	3473(7)	1436(3)	9968(3)	33.2(6)
C8_3	2583(8)	956(3)	10283(3)	33.7(13)
C9_3	1637(8)	1096(3)	10694(3)	36.0(15)
C10_3	917(10)	621(3)	11048(3)	38.0(17)
C11_3	976(7)	25(2)	10954(2)	32.0(12)
C12_3	4696(7)	662(3)	9480(3)	33.5(13)
C13_3	3637(7)	250(3)	9723(3)	32.1(13)
C14_3	3611(7)	-256(3)	9511(3)	29.2(12)
C15_3	2700(8)	-690(3)	9767(3)	34.6(15)
C16_3	1850(7)	-646(3)	10252(3)	33.1(12)
C17_3	2672(6)	358(2)	10164(3)	25.7(10)
C18_3	1800(8)	-100(3)	10466(3)	32.5(5)
C19_3	328(6)	-462(2)	11441(2)	32.1(4)
C20_3	-1066(6)	-276(3)	11605(3)	33.9(13)
C21_3	-1621(5)	-705(3)	12119(3)	36.7(13)
C22_3	-777(6)	-1326(3)	12404(3)	37.7(15)
C23_3	648(6)	-1522(2)	12261(3)	32.9(11)
C24_3	1185(6)	-1086(3)	11770(3)	32.2(4)
C25_3	-3125(5)	-492(2)	12334(2)	41.5(13)

Atom	x	y	z	U_{eq}
C26_3	-3995(8)	175(3)	11898(4)	57(2)
C27_3	-2910(9)	-445(5)	12975(3)	53.0(19)
C28_3	-4084(8)	-991(4)	12390(4)	49.1(17)
C29_3	1514(5)	-2224(2)	12613(2)	35.1(11)
C30_3	3073(6)	-2324(3)	12410(3)	37.3(14)
C31_3	736(8)	-2715(3)	12484(3)	38.7(14)
C32_3	1589(8)	-2341(3)	13308(2)	39.4(15)
C33_3	1050(6)	-1189(2)	10470(3)	32.1(4)
C34_3	1825(6)	-1826(2)	10566(4)	38.3(15)
C35_3	1128(6)	-2362(2)	10697(3)	34.3(11)
C36_3	-343(6)	-2240(2)	10733(3)	31.9(12)
C37_3	-1168(5)	-1599(2)	10655(3)	31.4(10)
C38_3	-461(5)	-1078(2)	10514(3)	29.8(7)
C39_3	2002(6)	-3041(2)	10726(3)	38.1(11)
C40_3	3465(7)	-3212(4)	11093(4)	45.7(15)
C41_3	2273(9)	-3020(4)	10066(3)	43.4(16)
C42_3	1186(8)	-3587(3)	11028(4)	43.8(15)
C43_3	-2811(5)	-1511(3)	10719(3)	34.6(7)
C44_3	-3524(7)	-797(3)	10636(4)	38.7(15)
C45_3	-3076(8)	-1948(4)	11368(3)	40.1(15)
C46_3	-3524(8)	-1715(4)	10237(3)	43.5(16)
O1_4	5940(5)	2823(2)	7144(3)	28.4(6)
O2_4	3577(5)	2796(2)	7208(3)	30.6(5)
O3_4	1554(5)	4682(3)	6444.7(16)	33.8(7)
O4_4	4353(5)	3284(2)	8162.0(19)	32.5(6)
N1_4	3033(5)	4011(2)	7297.4(18)	31.5(9)
C1_4	4630(6)	3079(2)	7123(3)	30.0(4)
C2_4	4202(6)	3838(2)	6902(2)	30.0(4)
C3_4	5413(6)	4224(2)	6824(2)	33.2(6)
C4_4	6285(7)	4123(3)	6289(3)	34.7(8)
C5_4	6416(7)	4026(4)	7408(3)	38.7(14)
C6_4	4720(8)	4963(3)	6634(4)	39.5(14)
C7_4	1745(6)	4447(3)	7006(2)	28.6(11)
C8_4	641(6)	4631(3)	7409(2)	30.5(5)
C9_4	-568(6)	5107(3)	7138(2)	31.9(12)
C10_4	-1604(7)	5303(3)	7505(2)	35.1(15)
C11_4	-1485(6)	5027(2)	8150(2)	29.9(10)
C12_4	3223(6)	3671(3)	7933(2)	29.0(11)
C13_4	2015(6)	3801(3)	8310(2)	29.8(11)
C14_4	2163(7)	3430(3)	8928(2)	36.7(15)
C15_4	1105(7)	3575(3)	9315(2)	34.5(14)
C16_4	-79(6)	4102(3)	9100(2)	31.4(11)
C17_4	773(6)	4306(3)	8063(2)	31.3(5)
C18_4	-312(6)	4479(3)	8447(2)	31.0(12)
C19_4	-2633(6)	5357(3)	8457(2)	32.1(4)
C20_4	-4076(5)	5454(4)	8267(3)	34.0(13)
C21_4	-5171(5)	5867(3)	8465(3)	35.0(11)
C22_4	-4746(6)	6145(3)	8865(3)	32.8(6)
C23_4	-3297(6)	6067(3)	9056(3)	34.9(11)
C24_4	-2246(6)	5669(3)	8849(3)	32.2(4)
C25_4	-6747(5)	6027(2)	8203(2)	37.3(11)
C26_4	-7292(8)	5415(3)	8213(4)	44.7(16)
C27_4	-6838(9)	6501(4)	7531(2)	44.1(16)
C28_4	-7813(6)	6361(4)	8565(3)	38.4(14)
C29_4	-2927(5)	6442(2)	9465(2)	39.1(11)
C30_4	-1287(6)	6402(4)	9515(4)	43.9(15)
C31_4	-3456(9)	6137(4)	10110(3)	44.8(16)
C32_4	-3685(9)	7173(3)	9179(3)	43.5(15)

Atom	x	y	z	U_{eq}
C33_4	-1025(6)	4167(3)	9603(2)	32.1(4)
C34_4	-387(6)	4230(4)	10113(2)	32.3(13)
C35_4	-1153(6)	4211(3)	10645(2)	33.6(11)
C36_4	-2576(6)	4170(4)	10632(3)	32.8(6)
C37_4	-3270(5)	4115(4)	10114(3)	36.8(12)
C38_4	-2493(6)	4131(4)	9596(2)	30.7(12)
C39_4	-351(6)	4218(3)	11218(2)	38.9(10)
C40_4	281(8)	4839(3)	11058(3)	41.4(14)
C41_4	891(7)	3609(3)	11440(3)	41.8(9)
C42_4	-1341(8)	4200(4)	11747(3)	41.7(15)
C43_4	-4857(5)	4047(3)	10143(3)	44.8(12)
C44_4	-5400(7)	3975(5)	9552(3)	55.0(19)
C45_4	-5793(7)	4668(4)	10210(4)	47.5(16)
C46_4	-5021(8)	3439(4)	10700(4)	50.6(17)
O2_5	1239(8)	724(4)	8474(3)	54.4(16)
O3_5	1694(7)	1642(4)	7789(3)	57(2)
O4_5	1025(9)	1568(4)	8742(4)	61.6(19)
C5_5	1532(13)	404(6)	8017(5)	61.2(19)
C6_5	1331(11)	1351(5)	8269(4)	48(2)
C7_5	978(13)	2267(5)	8602(6)	61.2(19)
O2_7	10217(10)	2503(5)	6604(4)	83(2)
O3_7	8806(9)	1997(6)	7320(4)	81(2)
O4_7	9748(13)	1515(6)	6763(6)	108(3)
C5_7	10080(20)	3063(8)	6833(8)	102(4)
C6_7	9528(18)	2041(6)	6941(8)	99(3)
C7_7	10842(16)	1553(10)	6293(7)	98(4)

Table S25. Anisotropic Displacement Parameters (×10[4]) for **Compound 8d**. The anisotropic displacement factor exponent takes the form: $-2\pi^2[h^2a^{*2} \times U_{11} + ... + 2hka^* \times b^* \times U_{12}]$

Atom	U_{11}	U_{22}	U_{33}	U_{23}	U_{13}	U_{12}
Rh1	25.5(4)	18.9(3)	18.4(3)	-9.7(2)	1.5(2)	-2.6(2)
Rh2	26.4(4)	16.4(3)	23.2(3)	-8.2(2)	0.2(2)	-3.2(2)
Rh2B	48(3)	9(2)	4.3(18)	-1.2(15)	9.8(17)	-3.7(18)
Rh1B	54(4)	19(3)	5(2)	-7(2)	1(2)	6(2)
O1_1	38.3(7)	25.0(17)	29.0(10)	-12.3(10)	2.7(6)	-5.0(7)
O2_1	37.9(7)	25.4(11)	28.4(9)	-10.8(8)	2.0(6)	-4.1(7)
O3_1	54(3)	29.4(11)	30(3)	-16.1(14)	2(2)	-6.1(12)
O4_1	44.6(13)	29.0(16)	28.8(14)	-15.4(12)	2.8(9)	-5.4(10)
N1_1	47.0(13)	27.9(14)	25.6(17)	-13.2(13)	2.4(12)	-6.3(11)
C1_1	38.3(7)	26.5(8)	28.8(8)	-12.0(6)	2.4(5)	-5.0(5)
C2_1	46.6(12)	27.5(18)	28.6(9)	-12.1(10)	2.3(7)	-6.8(10)
C3_1	46.1(12)	34.4(16)	27.4(10)	-9.4(11)	1.3(8)	-6.8(10)
C4_1	45.4(13)	34.5(16)	37(2)	-9.9(14)	0.9(12)	-6.9(10)
C5_1	42(3)	35.7(19)	40(4)	-14(2)	5(3)	-6.4(19)
C6_1	49(4)	51(4)	28.4(12)	-13.4(19)	1.7(12)	-9(3)
C7_1	47.8(12)	28.9(10)	26.0(13)	-14.6(9)	2.6(7)	-6.3(7)
C8_1	45.5(16)	27.9(13)	23(2)	-12.4(14)	5.4(15)	-7.9(12)
C9_1	49(2)	29.7(18)	29(2)	-16.2(17)	0.7(19)	-4.3(14)
C10_1	51(3)	31.4(14)	30(3)	-18.3(16)	-1(2)	-1.9(15)
C11_1	41.0(19)	29.2(12)	24.9(13)	-15.0(9)	5.0(14)	-6.0(11)
C12_1	46.1(15)	28.6(16)	29(2)	-14.6(17)	1.9(15)	-6.0(12)
C13_1	46.9(16)	31(2)	30(2)	-17.2(19)	0.5(15)	-4.6(14)
C14_1	47(2)	34(2)	34(3)	-20(2)	-2.9(18)	-2.5(16)
C15_1	45(2)	31(2)	31(2)	-17.9(19)	-0.8(17)	-4.8(16)

Atom	U_{11}	U_{22}	U_{33}	U_{23}	U_{13}	U_{12}
C16_1	41.6(13)	30.9(13)	27.7(19)	-16.4(14)	2.9(14)	-6.9(12)
C17_1	43.9(16)	28.8(14)	25(2)	-13.7(14)	4.2(15)	-7.3(12)
C18_1	44.7(11)	29.9(9)	26.3(10)	-15.1(8)	2.8(7)	-6.4(7)
C19_1	42.8(7)	28.8(7)	27.8(8)	-14.8(6)	2.5(5)	-6.1(5)
C20_1	46.6(17)	36(2)	24(2)	-12.4(17)	2.6(15)	-11.9(16)
C21_1	51(2)	39.2(19)	27(2)	-11.5(16)	-1.0(16)	-11.6(16)
C22_1	44(2)	37(2)	31(3)	-11.7(17)	2.0(17)	-7.7(17)
C23_1	41.9(16)	33.4(14)	28.3(15)	-12.5(12)	3.7(13)	-5.3(11)
C24_1	43.0(8)	28.7(8)	28.0(8)	-14.9(6)	2.6(6)	-6.2(6)
C25_1	57(2)	52(3)	34(2)	-14(2)	-3.5(16)	-19.3(19)
C26_1	62(4)	55(3)	51(4)	-10(3)	-3(3)	-24(3)
C27_1	69(5)	83(6)	40(2)	-28(3)	-1(3)	-29(4)
C28_1	56(2)	58(3)	52(4)	-17(3)	-4(2)	-18(3)
C29_1	48(2)	33.6(14)	26.0(19)	-13.5(12)	5.0(16)	-7.0(13)
C30_1	51(3)	35(3)	28(3)	-7(3)	1(2)	-11(2)
C31_1	52(3)	33(2)	31(3)	-13(2)	10(2)	-7(2)
C32_1	55(4)	37(4)	26.4(19)	-14.5(15)	5.5(18)	-9(3)
C33_1	41.9(8)	31.2(8)	27.0(7)	-15.4(8)	3.2(6)	-6.7(6)
C34_1	43.3(18)	31.5(9)	32(4)	-16.2(13)	2(2)	-7.0(9)
C35_1	43.9(17)	31.8(11)	33(3)	-16.6(18)	2.2(18)	-6.6(10)
C36_1	44.2(17)	29.2(14)	27(3)	-9.9(19)	0(2)	-6.7(11)
C37_1	42.0(9)	29.5(13)	31(3)	-11.3(15)	2.5(11)	-6.6(8)
C38_1	41.8(8)	29.8(12)	18.8(19)	-10.5(12)	2.3(8)	-6.5(7)
C39_1	45(2)	32.7(13)	34(2)	-18.1(17)	-0.3(19)	-6.6(13)
C40_1	53(3)	31(3)	51(3)	-25(3)	11(3)	-10(2)
C41_1	58(4)	50(5)	38(2)	-26(2)	-3(2)	-8(3)
C42_1	49(3)	34.0(19)	41(3)	-18(2)	-1(2)	-4(2)
C43_1	41.7(9)	31.5(14)	32.1(15)	-13.3(12)	3.3(10)	-7.0(8)
C44_1	44(4)	32.6(15)	41(4)	-15.6(18)	6(3)	-8.7(16)
C45_1	49(3)	39(3)	34.6(16)	-10.2(17)	-1.3(17)	-13(2)
C46_1	48(3)	43(4)	41(2)	-22(3)	10(2)	-11(3)
O1_2	38.3(7)	26.4(10)	21.5(16)	-10.4(10)	1.0(7)	-5.0(6)
O2_2	38.1(8)	25.1(17)	27(3)	-12.1(19)	2.4(9)	-5.7(8)
O3_2	42.4(19)	35.2(18)	26.5(8)	-16.3(8)	2.4(7)	-4.7(14)
O4_2	41.5(12)	29.6(12)	29.4(12)	-15.1(9)	1.7(8)	-5.8(8)
N1_2	36.6(16)	24.1(16)	28.8(10)	-15.3(9)	1.7(9)	-0.3(14)
C1_2	38.3(7)	26.5(8)	28.8(8)	-12.0(6)	2.4(5)	-5.0(5)
C2_2	38.8(7)	26.7(8)	25.9(10)	-12.1(7)	2.0(6)	-4.9(5)
C3_2	38.6(8)	27.5(10)	34.3(15)	-13.1(10)	1.5(8)	-5.0(6)
C4_2	38.1(13)	32(2)	35.2(15)	-14.7(15)	1.9(9)	-6.1(12)
C5_2	35(3)	35(3)	33.6(17)	-11.5(18)	-0.2(17)	-4(2)
C6_2	43(3)	27.5(11)	41(3)	-14.1(16)	2(3)	-5.2(10)
C7_2	41(2)	27(2)	26.6(8)	-16.6(9)	2.5(7)	-4.0(19)
C8_2	41.0(11)	28.3(13)	27.1(7)	-17.1(8)	3.2(6)	-5.2(8)
C9_2	42(3)	30(2)	29.3(13)	-15.2(14)	1.5(14)	-7(2)
C10_2	42(3)	30(3)	29.6(13)	-15.3(13)	1.6(13)	-6(2)
C11_2	41(2)	28.3(18)	29.7(13)	-15.6(11)	1.7(12)	-4.4(19)
C12_2	39(2)	28(2)	29.0(10)	-14.3(11)	1.0(10)	-3.7(18)
C13_2	40(2)	29.4(19)	29.6(12)	-13.8(12)	0.4(12)	-4.8(19)
C14_2	42(3)	31(2)	29.7(12)	-13.5(12)	-0.1(13)	-6(2)
C15_2	43(3)	32(2)	29.8(16)	-13.6(15)	-0.3(17)	-7(2)
C16_2	40(2)	30(2)	27.9(10)	-15.8(11)	2.5(11)	-4.9(18)
C17_2	41.9(11)	29.7(12)	27.1(7)	-16.9(8)	3.2(6)	-5.9(7)
C18_2	41(3)	28.1(18)	27.6(10)	-16.7(11)	2.5(11)	-4.3(18)
C19_2	42.8(7)	28.8(7)	27.8(8)	-14.8(6)	2.5(5)	-6.1(5)
C20_2	44.1(12)	32(3)	35(3)	-20(3)	5.4(16)	-8.5(17)
C21_2	44.5(16)	33(2)	35(2)	-21(2)	6.1(16)	-9.6(16)
C22_2	44.4(8)	27.6(16)	30.1(10)	-15.4(11)	3.7(7)	-6.7(8)

Atom	U_{11}	U_{22}	U_{33}	U_{23}	U_{13}	U_{12}
C23_2	45.4(11)	34(2)	32(2)	-20(2)	5.4(13)	-9.2(14)
C24_2	43.0(8)	28.7(8)	28.0(8)	-14.9(6)	2.6(6)	-6.2(6)
C25_2	45.3(17)	37(3)	38(2)	-21.1(19)	7.7(15)	-11.1(16)
C26_2	50(3)	38(3)	49(4)	-22(3)	15(3)	-11(2)
C27_2	51(4)	46(4)	40(2)	-16(2)	7(2)	-14(3)
C28_2	46(3)	50(4)	48(3)	-31(3)	10(3)	-16(3)
C29_2	51(2)	38(2)	34(2)	-22.3(18)	4.4(16)	-6.1(17)
C30_2	52(2)	50(4)	36(4)	-31(3)	7.8(18)	-9(2)
C31_2	61(4)	40(4)	36(2)	-23(2)	0(2)	-8(3)
C32_2	65(4)	38(2)	35(3)	-24(2)	1(3)	-10(2)
C33_2	41.9(8)	31.2(8)	27.0(7)	-15.4(8)	3.2(6)	-6.7(6)
C34_2	44.2(13)	29(4)	27.6(11)	-15.2(16)	4.6(10)	-8.4(18)
C35_2	44.9(9)	40(3)	29.6(12)	-19.1(17)	4.0(9)	-9.0(11)
C36_2	44.4(8)	27.6(16)	30.1(10)	-15.4(11)	3.7(7)	-6.7(8)
C37_2	41.4(11)	38(3)	30.8(12)	-18.3(16)	3.1(9)	-7.4(15)
C38_2	41.7(9)	26(3)	29.0(15)	-14(2)	2.8(9)	-5.8(10)
C39_2	44(2)	48(2)	29.3(14)	-19.6(16)	3.5(13)	-7.8(16)
C40_2	44(3)	49(2)	35(3)	-23(2)	2(2)	-7(2)
C41_2	48.1(19)	49.2(19)	31(2)	-17.8(15)	3.2(13)	-10.1(15)
C42_2	46(3)	57(4)	32(2)	-23(3)	2(2)	-9(3)
C43_2	41.0(12)	39(3)	45(2)	-18(2)	2.3(11)	-7.4(15)
C44_2	33(3)	56(4)	49(3)	-26(3)	3(2)	-14(3)
C45_2	42(4)	41(3)	49(4)	-20(3)	0(3)	-8(2)
C46_2	43(3)	44(3)	54(3)	-11(3)	0(2)	-8(2)
O1_3	38.3(7)	25.0(17)	29.0(10)	-12.3(10)	2.7(6)	-5.0(7)
O2_3	37.9(7)	25.4(11)	28.4(9)	-10.8(8)	2.0(6)	-4.1(7)
O3_3	58(3)	30.6(12)	31(3)	-17.8(15)	11(2)	-10.8(13)
O4_3	44.6(13)	29.0(16)	28.8(14)	-15.4(12)	2.8(9)	-5.4(10)
N1_3	47.2(16)	26.7(14)	24.3(17)	-12.1(13)	1.8(15)	-5.1(12)
C1_3	38.3(7)	26.5(8)	28.8(8)	-12.0(6)	2.4(5)	-5.0(5)
C2_3	45.1(13)	25.3(18)	28.2(9)	-11.2(10)	1.6(7)	-3.5(13)
C3_3	46.1(12)	34.4(16)	27.4(10)	-9.4(11)	1.3(8)	-6.8(10)
C4_3	45.4(13)	34.5(16)	37(2)	-9.9(14)	0.9(12)	-6.9(10)
C5_3	41(2)	36.1(19)	44(4)	-15(2)	0(2)	-7.4(17)
C6_3	55(4)	57(4)	28.6(12)	-15.0(18)	2.7(13)	-13(3)
C7_3	47.8(12)	28.9(10)	26.0(13)	-14.6(9)	2.6(7)	-6.3(7)
C8_3	49(3)	29.6(15)	26(2)	-15.0(16)	3(2)	-7.1(17)
C9_3	53(3)	29.9(19)	30(3)	-17(2)	7(2)	-10(2)
C10_3	58(4)	32.0(16)	32(3)	-19.3(18)	10(3)	-13(2)
C11_3	44(3)	29.2(11)	26.6(15)	-15.6(10)	2.9(18)	-7.1(13)
C12_3	44.6(19)	29.0(17)	30(3)	-15.8(19)	4(2)	-6.0(15)
C13_3	45(2)	30(2)	25(2)	-15.1(19)	2(2)	-6.6(18)
C14_3	34(3)	30(2)	26(2)	-15.3(18)	-0.7(19)	-2.7(19)
C15_3	44(3)	37(3)	31(2)	-21(2)	6(2)	-11(2)
C16_3	43(2)	31.8(14)	29(2)	-17.4(15)	5(2)	-7.8(16)
C17_3	34(2)	25.5(14)	15.5(19)	-8.4(14)	-8.0(16)	0.3(14)
C18_3	44.7(11)	29.9(9)	26.3(10)	-15.1(8)	2.8(7)	-6.4(7)
C19_3	42.8(7)	28.8(7)	27.8(8)	-14.8(6)	2.5(5)	-6.1(5)
C20_3	42.8(12)	31(2)	30(3)	-14.3(16)	3.0(14)	-5.9(11)
C21_3	44.2(19)	33.1(19)	33(2)	-12.5(16)	4.4(16)	-6.1(13)
C22_3	42.2(18)	32(2)	38(3)	-11.4(17)	3.7(16)	-7.7(14)
C23_3	40.6(17)	32.6(14)	27.1(17)	-12.6(12)	-0.8(14)	-7.7(11)
C24_3	43.0(8)	28.7(8)	28.0(8)	-14.9(6)	2.6(6)	-6.2(6)
C25_3	44.4(19)	42(2)	40(3)	-18(2)	6.2(16)	-6.3(14)
C26_3	48(3)	50(3)	58(4)	-7(3)	10(3)	-1(2)
C27_3	44(4)	79(6)	46(3)	-34(3)	11(2)	-16(4)
C28_3	53(3)	53(3)	50(4)	-26(3)	14(3)	-16(3)
C29_3	44(2)	32.5(14)	28.8(19)	-11.8(11)	0.5(16)	-6.6(13)

Atom	U_{11}	U_{22}	U_{33}	U_{23}	U_{13}	U_{12}
C30_3	46(2)	27(3)	39(3)	-13(3)	5(2)	-6.5(16)
C31_3	49(3)	34(2)	34(3)	-13(3)	1(3)	-10(3)
C32_3	52(4)	36(4)	28.8(19)	-12.1(16)	-0.7(17)	-4(3)
C33_3	41.9(8)	31.2(8)	27.0(7)	-15.4(8)	3.2(6)	-6.7(6)
C34_3	44.5(17)	31.5(9)	41(4)	-17.0(14)	7(2)	-6.6(9)
C35_3	43.8(15)	30.3(11)	29(3)	-12.5(18)	2.5(18)	-5.6(10)
C36_3	43.7(15)	30.0(13)	22(3)	-11.2(19)	1.7(18)	-5.6(10)
C37_3	41.6(10)	29.7(12)	24(3)	-11.0(15)	2.5(13)	-6.4(8)
C38_3	41.8(8)	29.8(12)	18.8(19)	-10.5(12)	2.3(8)	-6.5(7)
C39_3	46(2)	31.8(12)	39(2)	-16.3(17)	1.8(19)	-4.7(13)
C40_3	51(2)	34(3)	55(3)	-24(3)	-8(3)	-1.1(19)
C41_3	55(4)	36(4)	39(2)	-18(2)	3(2)	0(3)
C42_3	47(3)	31.2(18)	51(4)	-14(3)	4(3)	-4(2)
C43_3	41.7(9)	31.5(14)	32.1(15)	-13.3(12)	3.3(10)	-7.0(8)
C44_3	40(3)	32.3(15)	45(4)	-15.6(17)	2(3)	-6.2(15)
C45_3	39(3)	40(3)	35.7(17)	-8.2(17)	4.9(17)	-5(3)
C46_3	41(3)	52(4)	45(2)	-29(3)	2(2)	-7(3)
O1_4	38.3(7)	26.4(10)	21.5(16)	-10.4(10)	1.0(7)	-5.0(6)
O2_4	37.9(7)	25.4(11)	28.4(9)	-10.8(8)	2.0(6)	-4.1(7)
O3_4	42.4(19)	35.2(18)	26.5(8)	-16.3(8)	2.4(7)	-4.7(14)
O4_4	41.5(12)	29.6(12)	29.4(12)	-15.1(9)	1.7(8)	-5.8(8)
N1_4	40.2(11)	30.2(17)	27.4(10)	-15.4(10)	2.6(8)	-5.3(11)
C1_4	38.8(7)	26.7(8)	25.9(10)	-12.1(7)	2.0(6)	-4.9(5)
C2_4	38.8(7)	26.7(8)	25.9(10)	-12.1(7)	2.0(6)	-4.9(5)
C3_4	38.6(8)	27.5(10)	34.3(15)	-13.1(10)	1.5(8)	-5.0(6)
C4_4	38.1(13)	32(2)	35.2(15)	-14.7(15)	1.9(9)	-6.1(12)
C5_4	44(3)	38(4)	36.6(18)	-16(2)	-0.8(17)	-8(2)
C6_4	41(3)	27.1(11)	49(3)	-12.4(16)	9(3)	-5.4(10)
C7_4	39.6(13)	26(2)	26.5(8)	-16.7(9)	2.6(7)	-6.6(13)
C8_4	41.0(11)	28.3(13)	27.1(7)	-17.1(8)	3.2(6)	-5.2(8)
C9_4	40.8(15)	28(2)	30.5(13)	-16.2(15)	3.1(12)	-5.3(15)
C10_4	44(3)	30(3)	31.1(12)	-14.8(13)	4.5(12)	-2.0(19)
C11_4	40.2(15)	24.6(19)	31.0(12)	-15.6(11)	3.9(11)	-10.0(13)
C12_4	40.9(13)	26(2)	26.9(10)	-17.1(11)	1.8(8)	-7.0(13)
C13_4	41.4(14)	28(2)	27.2(12)	-18.4(12)	2.0(10)	-7.1(13)
C14_4	43(3)	39(3)	28.1(12)	-15.1(13)	3.3(11)	-3.0(18)
C15_4	45(2)	31(2)	29.5(16)	-14.2(16)	5.6(15)	-5.9(17)
C16_4	42.8(17)	28(2)	28.0(10)	-16.0(11)	4.3(10)	-8.1(14)
C17_4	41.9(11)	29.7(12)	27.1(7)	-16.9(8)	3.2(6)	-5.9(7)
C18_4	42.7(17)	27(2)	27.9(10)	-16.4(11)	4.6(10)	-7.1(14)
C19_4	42.8(7)	28.8(7)	27.8(8)	-14.8(6)	2.5(5)	-6.1(5)
C20_4	42.5(9)	33(3)	32(3)	-20(2)	2.4(9)	-5.0(9)
C21_4	42.6(11)	33(2)	35(2)	-21(2)	3.7(12)	-5.7(12)
C22_4	44.4(8)	27.6(16)	30.1(10)	-15.4(11)	3.7(7)	-6.7(8)
C23_4	44.5(9)	33(2)	33(2)	-20(2)	3.3(8)	-6.4(8)
C24_4	43.0(8)	28.7(8)	28.0(8)	-14.9(6)	2.6(6)	-6.2(6)
C25_4	43.0(12)	38(3)	38(2)	-23.5(19)	2.5(13)	-5.2(13)
C26_4	42(3)	40(3)	61(5)	-31(3)	2(3)	-5(2)
C27_4	49(4)	46(3)	38(2)	-21(2)	2.7(17)	-1(3)
C28_4	39(2)	47(3)	40(3)	-30(3)	0(2)	-6(2)
C29_4	53(2)	37(2)	35(2)	-22.0(18)	2.1(17)	-9.0(18)
C30_4	54(2)	42(4)	45(4)	-25(3)	0(2)	-11(2)
C31_4	60(4)	45(4)	35(2)	-22(2)	4(2)	-8(3)
C32_4	62(4)	37(2)	39(3)	-24(2)	-2(3)	-7(2)
C33_4	41.9(8)	31.2(8)	27.0(7)	-15.4(8)	3.2(6)	-6.7(6)
C34_4	43.3(15)	30(4)	27.0(11)	-15.2(17)	3.1(10)	-7(2)
C35_4	44.8(10)	32(3)	27.8(13)	-15.8(17)	4.4(9)	-8.0(13)
C36_4	44.4(8)	27.6(16)	30.1(10)	-15.4(11)	3.7(7)	-6.7(8)

Atom	U_{11}	U_{22}	U_{33}	U_{23}	U_{13}	U_{12}
C37_4	43.7(13)	42(4)	31.6(13)	-20.1(17)	6.0(10)	-11.6(17)
C38_4	41.3(9)	24(3)	28.3(15)	-13(2)	2.7(10)	-4.8(12)
C39_4	47.2(18)	46(2)	29.2(14)	-19.8(15)	4.6(12)	-11.8(15)
C40_4	54(4)	50(2)	32(3)	-25(2)	7(3)	-16(2)
C41_4	48.1(19)	49.2(19)	31(2)	-17.8(15)	3.2(13)	-10.1(15)
C42_4	49(3)	53(4)	30.1(19)	-22(2)	6(2)	-14(3)
C43_4	44.3(14)	52(3)	49(3)	-28(2)	8.3(13)	-14.8(17)
C44_4	41(4)	85(5)	57(3)	-45(3)	10(2)	-18(4)
C45_4	45(3)	53(3)	53(4)	-28(3)	9(3)	-12(2)
C46_4	37(4)	55(3)	61(3)	-21(3)	11(3)	-12(3)
O2_5	51(4)	54(3)	54(4)	-11(3)	3(3)	-14(3)
O3_5	24(3)	74(5)	46(3)	8(3)	5(3)	-3(3)
O4_5	71(5)	66(4)	52(4)	-25(3)	12(4)	-17(4)
C5_5	58(5)	64(4)	64(5)	-24(4)	6(4)	-15(4)
C6_5	44(5)	49(4)	45(4)	-11(3)	14(4)	-11(4)
C7_5	58(5)	64(4)	64(5)	-24(4)	6(4)	-15(4)
O2_7	54(4)	113(4)	67(4)	-8(3)	0(3)	-26(4)
O3_7	43(3)	116(5)	72(4)	-12(4)	-5(2)	-31(3)
O4_7	97(6)	122(4)	101(6)	-25(4)	31(5)	-44(4)
C5_7	100(11)	121(5)	80(8)	-18(5)	26(7)	-46(6)
C6_7	78(5)	117(4)	93(5)	-18(3)	22(4)	-37(3)
C7_7	79(7)	119(7)	80(6)	-12(6)	11(5)	-27(6)

Compound 10

Submitted by: **Yannick Boni**

Solved by: **John Bacsa**

R_1=6.7%

Crystal Data and Experimental

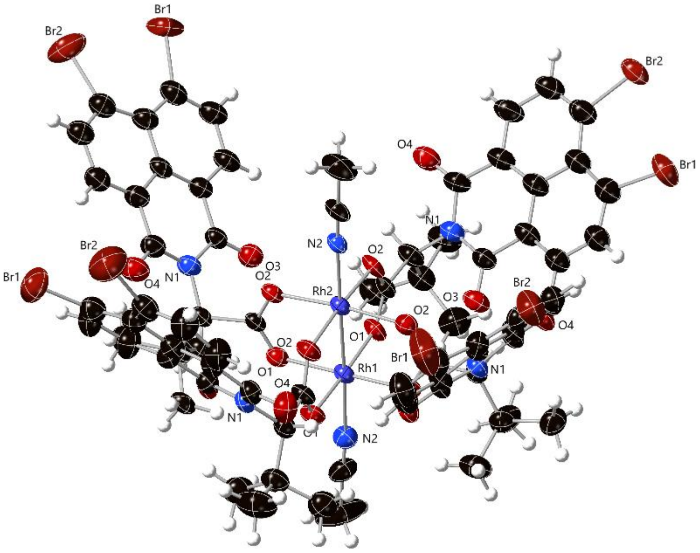

Experimental. Single reddish green needle-shaped crystals of ytb-e36_br2 were chosen from the sample as supplied. A suiTable Scrystal with dimensions 0.42 × 0.27 × 0.15 mm³ was selected and mounted on a loop with paratone on a XtaLAB Synergy-S diffractometer. The crystal was kept at a steady T = 196.7(1) K during data collection. The structure was solved with the ShelXT (Sheldrick, 2015) solution program and by using Olex2 1.5-dev (Dolomanov et al., 2009) as the graphical interface. The model was refined with ShelXL 2019/3 (Sheldrick, 2015) using full matrix least squares minimisation on F^2.

Crystal Data. $C_{388}H_{356}Br_{32}N_{48}O_{64}Rh_8$, M_r = 10095.59, monoclinic, $P2_1$ (No. 4), a = 17.72776(13) Å, b = 20.61886(17) Å, c = 55.6334(4) Å, β = 92.2273(6)°, α = γ = 90°, V = 20320.1(3) Å³, T = 196.7(1) K, Z = 2, Z' = 1, μ(Cu K$_\alpha$) = 6.879, 188532 reflections measured, 69067 unique (R_{int} = 0.0813) which were used in all calculations. The final wR_2 was 0.1787 (all data) and R_1 was 0.0668 (I≥2 σ(I)).

**Compound**	ytb-e36_br2
Formula	$C_{388}H_{356}Br_{32}N_{48}O_{64}Rh_8$
$D_{calc.}$/ g cm^{-3}	1.650
μ/mm^{-1}	6.879
Formula Weight	10095.59
Colour	reddish green
Shape	needle-shaped
Size/mm³	0.42×0.27×0.15
T/K	196.69(10)
Crystal System	monoclinic
Flack Parameter	0.062(6)
Hooft Parameter	-0.006(3)
Space Group	$P2_1$
a/Å	17.72776(13)
b/Å	20.61886(17)
c/Å	55.6334(4)
α/°	90
β/°	92.2273(6)
γ/°	90
V/Å³	20320.1(3)
Z	2
Z'	1
Wavelength/Å	1.54184
Radiation type	Cu K$_\alpha$
Θ_{min}/°	2.647
Θ_{max}/°	73.208
Measured Refl's.	188532
Indep't Refl's	69067
Refl's I≥2 σ(I)	58170
R_{int}	0.0813
Parameters	4427
Restraints	13699
Largest Peak	1.745
Deepest Hole	-1.224
GooF	1.100
wR_2 (all data)	0.1787
wR_2	0.1723
R_1 (all data)	0.0762
R_1	0.0668

Structure Quality Indicators

**Reflections:**	d min (CuKα) 2Θ=146.4° 0.81	I/σ(I) 12.2	Rint m=2.73 8.13%	Full 135.4° 96% to 146.4° 99.4
**Refinement:**	Shift 0.002	Max Peak 1.7	Min Peak -1.2	GooF 1.100

A reddish green needle-shaped-shaped crystal with dimensions 0.42 × 0.27 × 0.15 mm^3 was mounted on a loop with paratone. Data were collected using a XtaLAB Synergy, Dualflex, HyPix diffractometer equipped with an Oxford Cryosystems low-temperature device operating at T = 196.69(10) K.

Data were measured using ω scans with Cu K_α radiation. The diffraction pattern was indexed and the total number of runs and images was based on the strategy calculation from the program CrysAlisPro 1.171.41.108a (Rigaku OD, 2021). The maximum resolution that was achieved was Θ = 73.208° (0.83 Å).

The unit cell was refined using CrysAlisPro 1.171.41.108a (Rigaku OD, 2021) on 82850 reflections, 44% of the observed reflections.

Data reduction, scaling and absorption corrections were performed using CrysAlisPro 1.171.41.108a (Rigaku OD, 2021). The final completeness is 99.40 % out to 73.208° in Θ. A numerical absorption correction based on gaussian integration over a multifaceted crystal model was performed using CrysAlisPro 1.171.41.108a (Rigaku Oxford Diffraction, 2021). An empirical absorption correction using spherical harmonics, implemented in SCALE3 ABSPACK scaling algorithm was also applied. The absorption coefficient μ of this material is 6.879 mm^{-1} at this wavelength (λ = 1.54184Å) and the minimum and maximum transmissions are 0.130 and 0.691.

The structure was solved and the space group $P2_1$ (# 4) determined by the ShelXT (Sheldrick, 2015) structure solution program and refined by full matrix least squares minimisation on $\boldsymbol{F}^2$ using version 2019/3 of ShelXL 2019/3 (Sheldrick, 2015). All non-hydrogen atoms were refined anisotropically. Hydrogen atom positions were calculated geometrically and refined using the riding model.

_refine_special_details: Refined as a 2-component inversion twin.

There is a single formula unit in the asymmetric unit, which is represented by the reported sum formula. In other words: Z is 2 and Z' is 1. The moiety formula is 4(C76 H62 Br8 N6 O16 Rh2), 6(C6 H6), 2(C2 H3 N), 22[C2 H3 N1].

The Flack parameter was refined to 0.1(1). Determination of absolute structure using Bayesian statistics on Bijvoet differences using the Olex2 results in -0.006(3). The chiral atoms in this structure are: C13_1(S), C13_10(S), C13_11(S), C13_12(S), C13_15(S), C13_16(S), C13_17(S), C13_18(S), C13_2(S), C13_27(S), C13_3(S), C13_4(S), C13_5(S), C13_6(S), C13_7(S), C13_8(S), C13_9(S). Note: The Flack parameter is used to determine chirality of the crystal studied, the value should be near 0, a value of 1 means that the stereochemistry is wrong and the model should be inverted. A value of 0.5 means that the crystal consists of a racemic mixture of the two enantiomers.

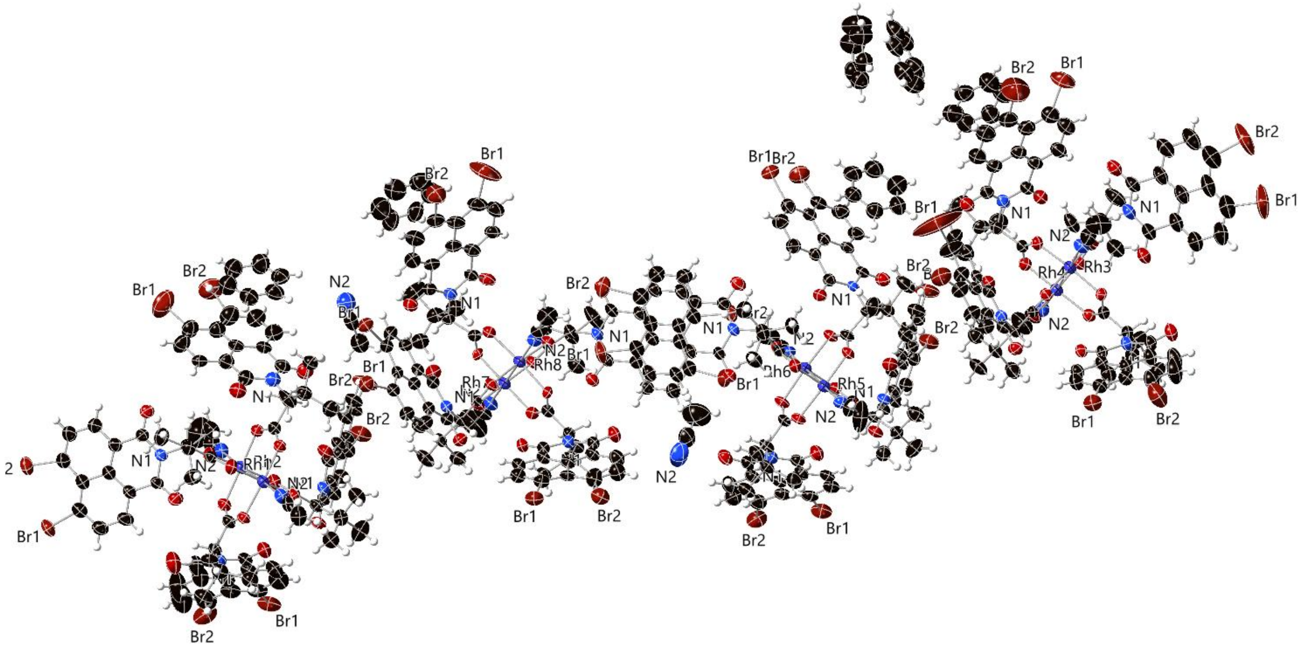

Figure S318. Thermal ellipsoidal representation (50% probability for all atoms, excluding hydrogens) of the asymmetric unit in the crystal with the minor disorder components hidden for clarity. The chiral atoms in this structure have S-configuration.

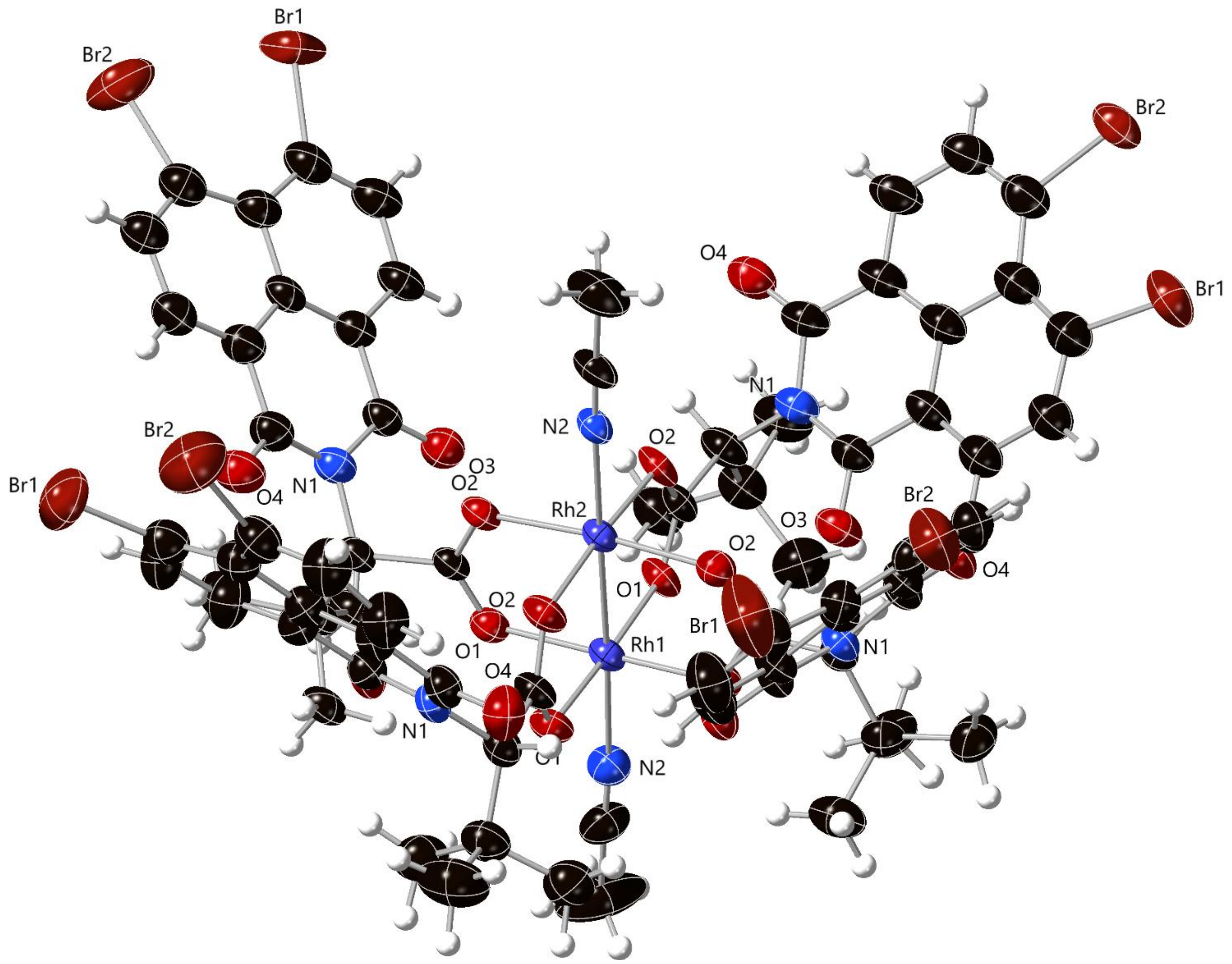

Figure S319. Thermal ellipsoidal representation (50% probability for all atoms, excluding hydrogens) of the molecular structure of the (C76 H62 Br8 N6 O16 Rh2) unit in the crystal with the minor disorder components hidden for clarity. The chiral atoms in this structure have S-configuration. There are 4 of these molecules in the asymmetric unit.

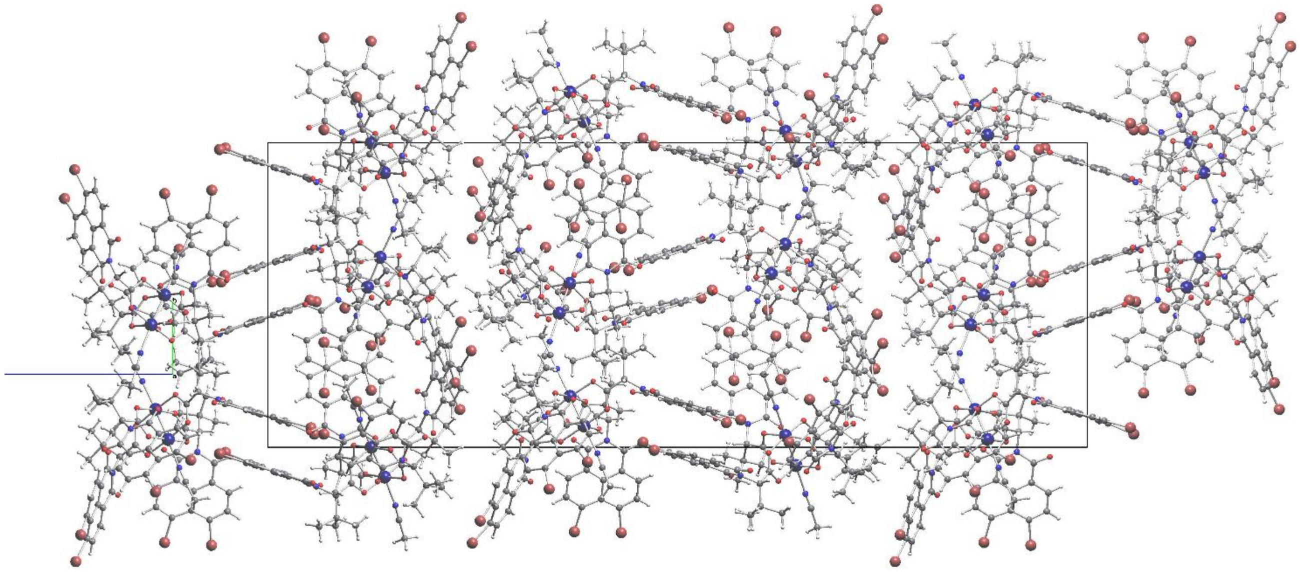

Figure S320. Molecular packing in the crystal viewed along the a-axis

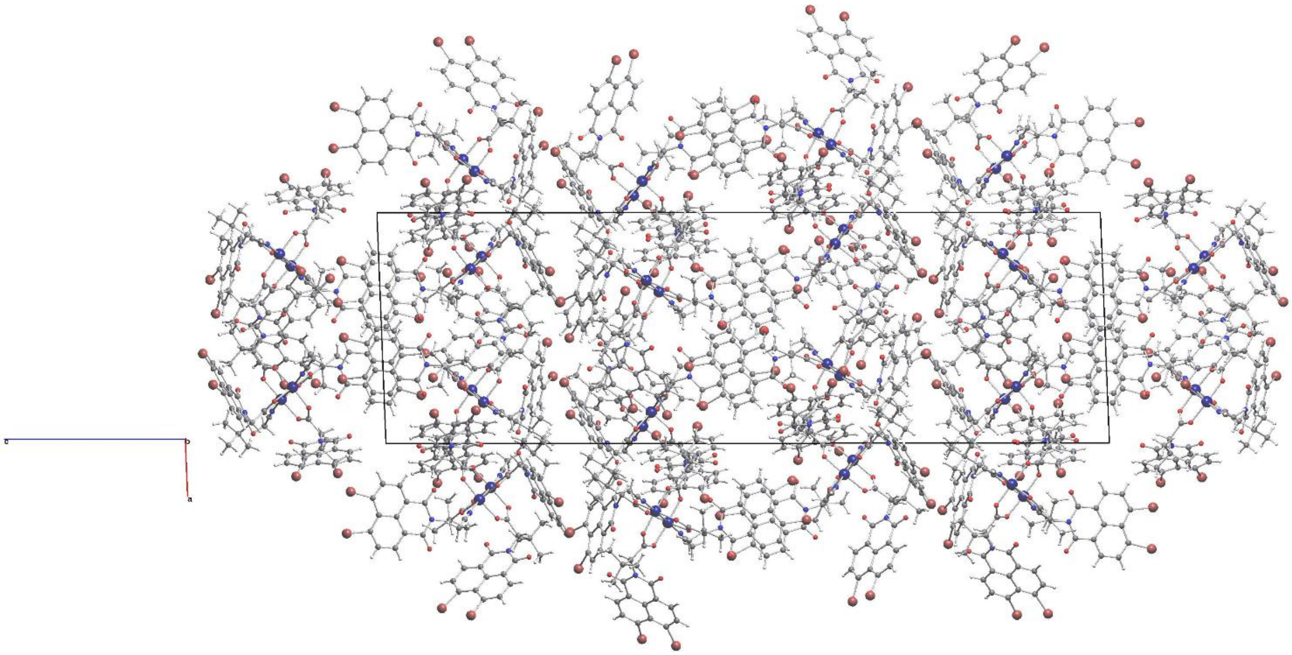

Figure S321. Molecular packing in the crystal viewed along the b-axis

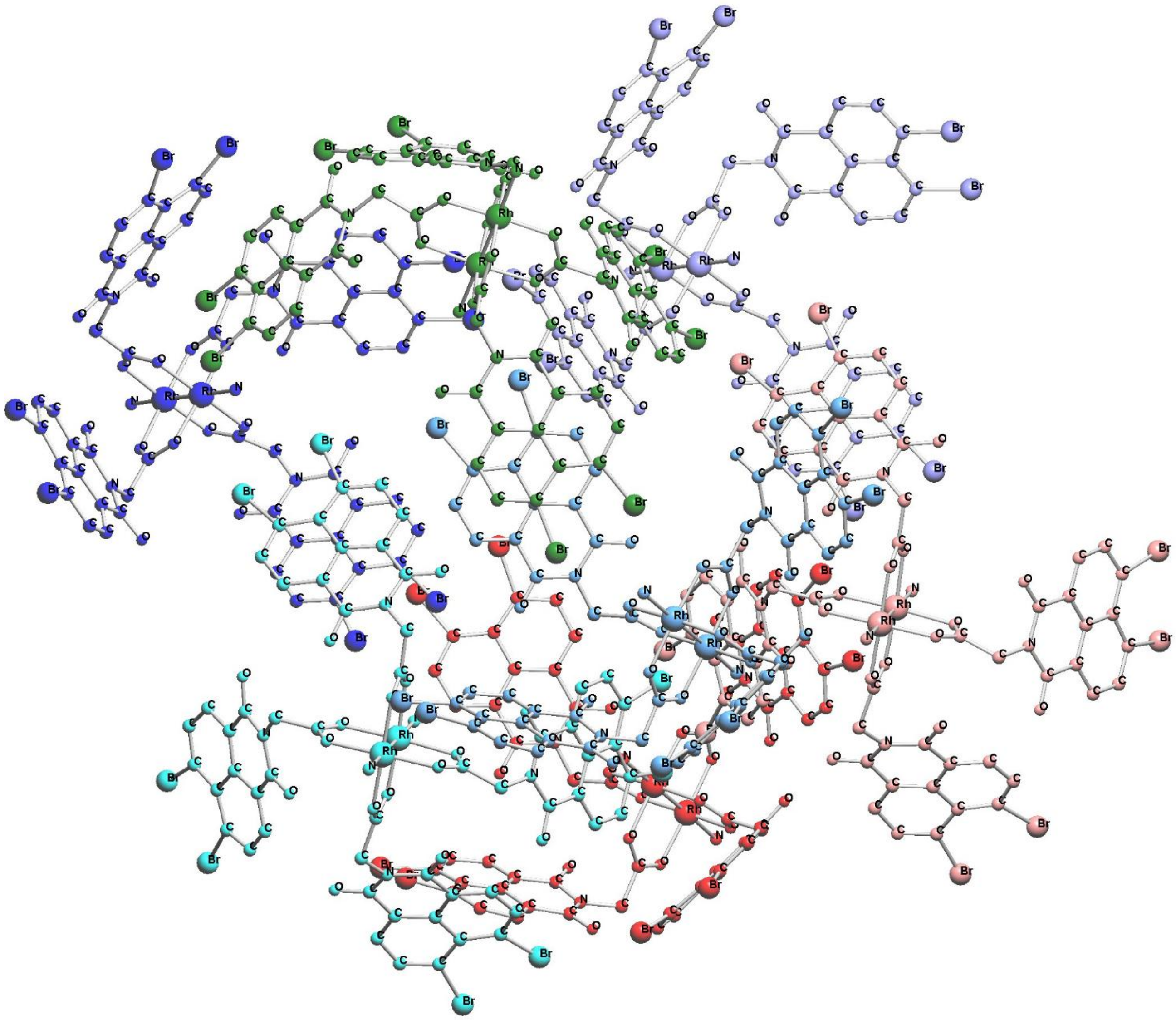

Figure S322. A simplified representation of the relationships of the 4 symmetry independent Rh2 complexes with each Rh2 complex displayed using a different color.

Data Plots: Diffraction Data

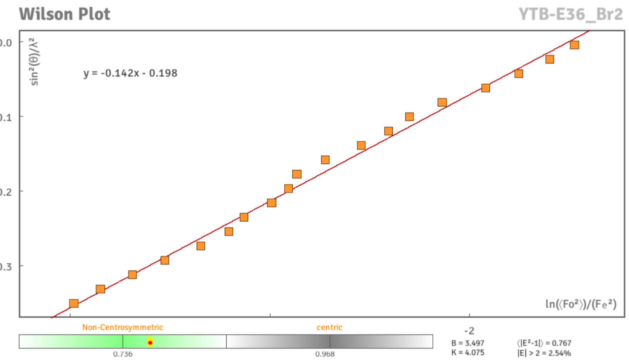

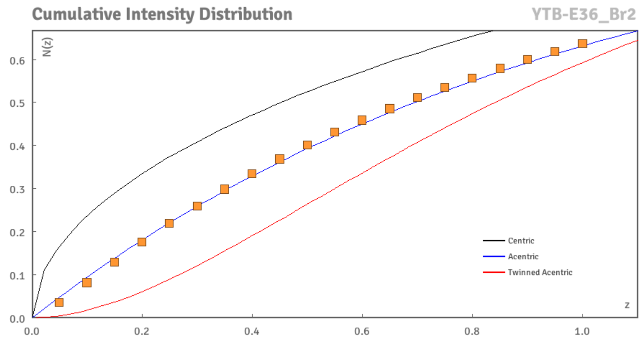

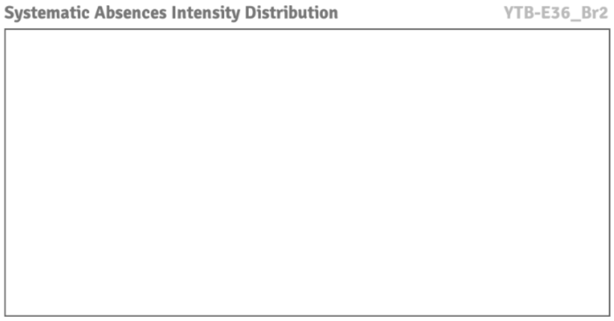

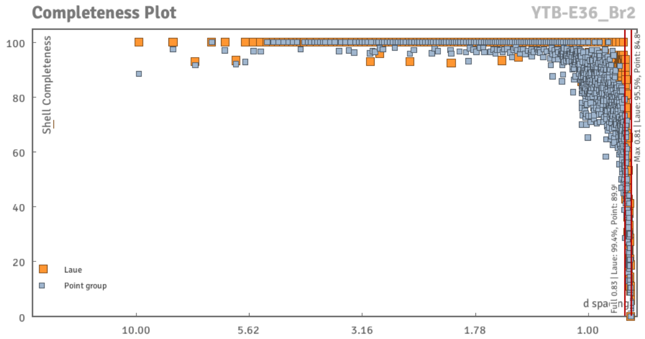

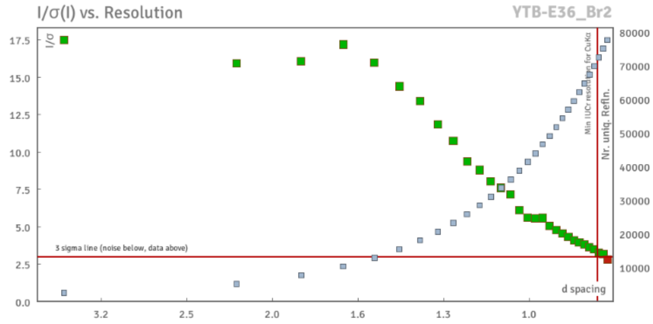

Data Plots: Refinement and Data

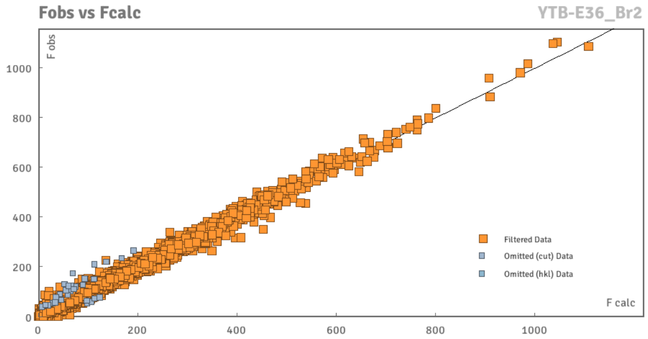

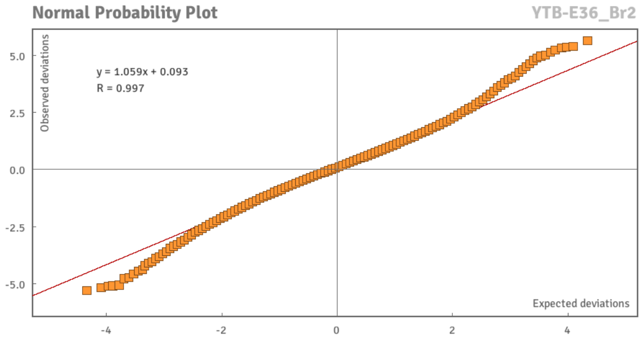

Reflection Statistics

Total reflections (after filtering)	188532	Unique reflections	69067
Completeness	0.848	Mean I/σ	8.85
hkl_{max} collected	(20, 25, 68)	hkl_{min} collected	(-21, -21, -48)
hkl_{max} used	(21, 25, 68)	hkl_{min} used	(-21, -21, 0)
Lim d_{max} collected	100.0	Lim d_{min} collected	0.77
d_{max} used	16.69	d_{min} used	0.81
Friedel pairs	13784	Friedel pairs merged	0
Inconsistent equivalents	97	R_{int}	0.0813
R_{sigma}	0.0823	Intensity transformed	0
Omitted reflections	0	Omitted by user (OMIT hkl)	124
Multiplicity	(33148, 20858, 13181, 7663, 3860, 2175, 1071, 379, 72, 7)	Maximum multiplicity	10
Removed systematic absences	0	Filtered off (Shel/OMIT)	0

Table S26. Fractional Atomic Coordinates (×10⁴) and Equivalent Isotropic Displacement Parameters (Å^2×10^3) for ytb-e36_br2. U_{eq} is defined as 1/3 of the trace of the orthogonalised U_{ij}.

Atom	x	y	z	U_{eq}
Rh1	8093.0(5)	4026.8	1424.3(2)	35.51(19)
Rh02	7057.7(5)	6676.26	3687.2(2)	31.78(18)
Rh2	7558.5(5)	5006.02	1260.1(2)	36.79(19)
Rh3	7695.0(5)	5263.2	8784.3(2)	33.64(18)
Rh4	8211.0(5)	6245.48	8629.2(2)	35.52(19)
Rh5	9256.7(5)	4407.22	6445.1(2)	32.58(18)
Rh6	8645.5(4)	5387.95	6316.5(2)	31.50(17)
Rh06	6569.2(4)	5704.49	3857.6(2)	31.36(17)
Br1_1	6862.1(12)	5167.1(8)	4577.6(2)	68.6(5)
Br2_1	5123.7(14)	4906.4(16)	4713.3(3)	108.1(8)
O1_1	8514(3)	3897(3)	6230.0(12)	38.7(17)
O2_1	7920(4)	4815(2)	6109.3(12)	37.9(17)
O3_1	8334(2)	4075(5)	5650.9(10)	50(2)
O4_1	5926(3)	4239(6)	5886.6(10)	63(3)
N1_1	7122(2)	4076(3)	5766.7(7)	43(2)
C1_1	7675(3)	4184(6)	5598.9(9)	41(2)
C2_1	7426(3)	4411(5)	5355.8(9)	40(2)
C3_1	7958(3)	4514(6)	5189.6(10)	46(3)
C4_1	7739(3)	4731(7)	4960.1(11)	57(3)
C5_1	6999(3)	4837(6)	4895.2(10)	51(3)
C6_1	6417(3)	4741(6)	5058.6(9)	45(2)
C7_1	5622(3)	4789(7)	5020.9(9)	56(3)
C8_1	5125(3)	4727(7)	5198.5(10)	60(3)
C9_1	5375(3)	4558(8)	5430.5(11)	63(4)
C10_1	6131(3)	4453(7)	5479.4(10)	53(3)
C11_1	6654(3)	4536(5)	5297.4(9)	40(2)
C12_1	6373(3)	4260(7)	5727.6(10)	53(3)
C13_1	7344(3)	3810(3)	6003.5(8)	41(2)
C14_1	7996(4)	4203(3)	6118.2(13)	36(2)
C15_1	7390(4)	3061(3)	6014.0(11)	46(2)
C16_1	8071(6)	2777(4)	5888.9(19)	56(3)
C17_1	7419(7)	2847(4)	6277.3(12)	56(4)
C18_1	6662(5)	2779(4)	5894(2)	61(4)
Br1_2	3136.6(9)	5160.9(13)	6362.6(4)	90.3(6)
Br2_2	3397.7(12)	6571.7(13)	6610.4(4)	102.5(7)
O1_2	8517(4)	4336(3)	6713.7(11)	38.5(17)
O2_2	7939(4)	5263(3)	6593.5(11)	40.8(18)
O3_2	6778(3)	4208(3)	6574.7(12)	45.6(19)
O4_2	6772(3)	5779(4)	7143.4(13)	60(3)
N1_2	6764(2)	4943(3)	6877.5(9)	36.7(18)
C1_2	6427(3)	4616(4)	6681.4(14)	42(2)
C2_2	5647(3)	4800(4)	6604.6(15)	42(2)
C3_2	5279(4)	4440(5)	6431(2)	62(4)
C4_2	4533(4)	4588(5)	6364(2)	63(4)
C5_2	4174(3)	5110(4)	6458.0(18)	58(3)
C6_2	4541(3)	5532(3)	6625.4(16)	46(3)
C7_2	4284(4)	6123(4)	6724.9(16)	55(3)
C8_2	4669(5)	6453(5)	6901(2)	69(4)
C9_2	5355(4)	6216(5)	6995.4(18)	58(3)
C10_2	5676(3)	5678(4)	6895.2(15)	44(2)
C11_2	5293(3)	5348(4)	6705.3(15)	41(2)
C12_2	6444(3)	5483(4)	6983.1(14)	42(2)
C13_2	7542(2)	4775(3)	6952.8(9)	44(2)
C14_2	8044(4)	4785(4)	6735.0(13)	42(2)

Atom	x	y	z	U_{eq}
C15_2	7613(4)	4197(3)	7129.8(11)	57(3)
C16_2	7445(8)	3536(3)	7014(2)	76(4)
C17_2	8409(5)	4189(6)	7245.3(17)	68(4)
C18_2	7053(6)	4306(7)	7332.0(17)	97(7)
Br1_3	5630.1(17)	2124.6(15)	7715.9(16)	296(5)
Br2_3	7094.2(11)	1245.2(9)	7656.4(4)	82.1(5)
O1_3	8733(5)	5688(2)	8386.2(12)	46.3(19)
O2_3	8268(4)	4750(3)	8528.8(11)	41.4(18)
O3_3	7562(3)	4840(2)	8048.8(14)	46.7(19)
O4_3	9516(3)	3439(3)	8169.2(15)	55(2)
N1_3	8562(2)	4161(2)	8103.7(12)	41.6(19)
C1_3	7805(3)	4290(2)	8042.6(19)	42(2)
C2_3	7321(3)	3738(2)	7964.0(18)	40(2)
C3_3	6571(3)	3850(3)	7921(2)	50(3)
C4_3	6106(3)	3345(3)	7840(3)	65(4)
C5_3	6389(3)	2740(4)	7801(4)	96(7)
C6_3	7166(3)	2594(3)	7833(2)	56(3)
C7_3	7555(4)	2004(3)	7794(3)	60(3)
C8_3	8293(4)	1911(3)	7858(3)	65(4)
C9_3	8723(4)	2418(3)	7957(2)	58(3)
C10_3	8404(3)	3019(3)	7986(2)	48(3)
C11_3	7633(3)	3115(2)	7926.4(18)	44(2)
C12_3	8871(3)	3541(2)	8100.3(17)	43(2)
C13_3	9045(3)	4695(2)	8191.7(9)	39(2)
C14_3	8640(5)	5083(3)	8382.9(11)	36(2)
C15_3	9431(4)	5080(3)	7991.6(11)	52(3)
C16_3	8901(6)	5543(5)	7851.0(18)	68(4)
C17_3	10088(6)	5468(6)	8105.0(18)	74(5)
C18_3	9760(7)	4592(5)	7812.9(17)	73(5)
Br1_4	7907.5(9)	4834.1(7)	-515.5(2)	56.0(4)
Br2_4	6096.8(11)	4787.6(9)	-590.7(2)	68.5(5)
O1_4	7479(4)	3512(3)	1172.5(11)	44.2(19)
O2_4	6997(4)	4431(2)	1009.1(12)	44.1(18)
O3_4	7972(3)	3748(6)	638.0(11)	72(3)
O4_4	5427(3)	3830(6)	547.5(12)	62(3)
N1_4	6698(2)	3712(4)	590.4(8)	49(3)
C1_4	7419(3)	3813(6)	504.6(10)	46(3)
C2_4	7478(3)	4039(8)	253.2(12)	57(3)
C3_4	8179(3)	4145(9)	169.1(14)	70(4)
C4_4	8253(3)	4356(9)	-66.6(14)	70(4)
C5_4	7638(3)	4500(7)	-212.4(12)	54(3)
C6_4	6893(3)	4412(6)	-135.8(10)	47(3)
C7_4	6192(3)	4499(6)	-264.4(10)	52(3)
C8_4	5512(3)	4425(8)	-164.6(12)	64(4)
C9_4	5467(3)	4207(8)	71.3(12)	61(4)
C10_4	6116(3)	4112(7)	210.1(11)	51(3)
C11_4	6825(3)	4176(6)	106.4(10)	42(2)
C12_4	6041(3)	3884(6)	461.3(10)	42(2)
C13_4	6625(3)	3429(3)	830.3(7)	45(2)
C14_4	7087(5)	3821(3)	1017.4(11)	38(2)
C15_4	6687(4)	2680(3)	839.2(11)	51(3)
C16_4	7496(5)	2425(5)	825(2)	69(4)
C17_4	6352(7)	2437(4)	1071.5(16)	62(4)
C18_4	6210(7)	2396(4)	626.1(17)	66(4)
Br1_5	1220(2)	4513(2)	3900.4(7)	195(2)
Br2_5	1509.6(13)	3626.9(11)	3433.2(4)	94.8(7)
O1_5	6081(3)	6776(3)	3506.7(11)	33.9(16)
O2_5	5567(4)	5855(3)	3645.9(11)	38.6(17)

Atom	x	y	z	U_{eq}
O3_5	4139(4)	6567(4)	3725.0(13)	58(2)
O4_5	4310(4)	5499(4)	3017.4(10)	51(2)
N1_5	4282(3)	6010(3)	3378.2(9)	41(2)
C1_5	3919(5)	6135(4)	3591.6(13)	41(2)
C2_5	3254(5)	5733(5)	3646.2(15)	53(3)
C3_5	2898(6)	5852(5)	3854.3(16)	60(4)
C4_5	2317(8)	5443(7)	3922(2)	86(6)
C5_5	2065(7)	4948(7)	3777(2)	81(5)
C6_5	2408(6)	4794(5)	3558.7(16)	63(4)
C7_5	2212(6)	4313(4)	3384.3(14)	60(3)
C8_5	2558(7)	4251(6)	3172.3(16)	68(4)
C9_5	3134(6)	4673(5)	3112.8(14)	57(3)
C10_5	3374(5)	5142(4)	3273.7(12)	44(3)
C11_5	3010(5)	5219(5)	3492.4(14)	52(3)
C12_5	4018(5)	5559(5)	3209.1(12)	45(3)
C13_5	4938(3)	6399(3)	3317.6(9)	42(2)
C14_5	5563(3)	6358(3)	3514.6(13)	38(2)
C15_5	4758(4)	7076(3)	3209.1(13)	59(3)
C16_5	4720(8)	7618(4)	3397(2)	72(4)
C17_5	5363(6)	7246(5)	3030.7(19)	67(4)
C18_5	3993(6)	7041(6)	3068(3)	108(8)
Br1_6	8378.6(9)	5812.9(7)	5595.6(2)	55.2(4)
Br2_6	6674.6(10)	5904.5(10)	5770.8(2)	72.3(5)
O1_6	6670(4)	7165(3)	3971.3(10)	41.4(19)
O2_6	6232(4)	6250(2)	4140.0(12)	39.4(17)
O3_6	7537(3)	6822(5)	4453.7(9)	51(2)
O4_6	5131(2)	6854(6)	4694.4(11)	63(3)
N1_6	6340(2)	6923(3)	4576.9(8)	41(2)
C1_6	7106(3)	6789(6)	4617.8(9)	42(2)
C2_6	7363(3)	6581(6)	4862.4(9)	41(2)
C3_6	8116(3)	6495(7)	4909.5(12)	53(3)
C4_6	8372(3)	6281(7)	5135.8(12)	59(3)
C5_6	7883(3)	6149(6)	5313.4(10)	46(3)
C6_6	7095(3)	6234(6)	5277.8(9)	44(2)
C7_6	6509(3)	6154(7)	5442.2(10)	55(3)
C8_6	5769(3)	6263(7)	5385.9(11)	58(3)
C9_6	5536(3)	6433(7)	5152.4(10)	54(3)
C10_6	6065(3)	6538(6)	4982.3(10)	46(3)
C11_6	6840(3)	6469(6)	5044.5(9)	44(3)
C12_6	5798(3)	6788(5)	4743.5(9)	37(2)
C13_6	6091(3)	7242(2)	4351.6(7)	36(2)
C14_6	6357(5)	6856(3)	4135.5(9)	34(2)
C15_6	6214(5)	7986(3)	4350.0(11)	49(3)
C16_6	7044(5)	8189(5)	4341(2)	68(4)
C17_6	5775(6)	8274(4)	4133.8(16)	60(4)
C18_6	5885(9)	8271(4)	4580.1(16)	104(8)
Br1_7	6108.8(12)	8175.1(10)	2482.2(5)	96.7(7)
Br2_7	7449.7(11)	9123.8(8)	2356.7(4)	74.2(5)
O1_7	8666(4)	4578(2)	1666.4(11)	42.2(18)
O2_7	8173(4)	5513(3)	1518.9(12)	43.4(18)
O3_7	7686(4)	5467(2)	2019.2(15)	49(2)
O4_7	9573(3)	6835(3)	1828.2(14)	47(2)
N1_7	8664(3)	6128.3(19)	1935.6(12)	39.9(19)
C1_7	7952(4)	6010(2)	2025.1(19)	41(2)
C2_7	7565(4)	6556(2)	2142(2)	44(3)
C3_7	6902(5)	6435(3)	2250(2)	53(3)
C4_7	6515(6)	6943(3)	2355(3)	62(4)
C5_7	6768(5)	7567(2)	2344(2)	55(3)

Atom	x	y	z	U_{eq}
C6_7	7453(4)	7729(2)	2235.9(19)	39(2)
C7_7	7803(5)	8344(2)	2212.5(19)	51(3)
C8_7	8476(5)	8429(3)	2109(2)	54(3)
C9_7	8857(4)	7902(2)	2014(2)	47(3)
C10_7	8535(4)	7296(2)	2015.2(17)	35(2)
C11_7	7846(4)	7199(2)	2127.6(18)	41(2)
C12_7	8966(4)	6747(2)	1915.2(17)	35(2)
C13_7	9090(3)	5582(2)	1840.1(10)	41(2)
C14_7	8583(4)	5185(3)	1666.3(12)	43(2)
C15_7	9574(4)	5208(3)	2032.3(11)	49(3)
C16_7	9107(6)	4821(5)	2208.7(16)	63(4)
C17_7	10105(5)	4746(4)	1906.6(18)	55(3)
C18_7	10064(7)	5702(4)	2176(2)	80(5)
Br1_8	11426.7(11)	8340.6(8)	1259.4(4)	82.2(6)
Br2_8	11292.0(13)	8452.7(10)	683.8(5)	102.4(8)
O1_8	8940(4)	4123(3)	1191.7(11)	38.1(17)
O2_8	8459(3)	5066(3)	1047.9(13)	42.0(18)
O3_8	9940(5)	5310(3)	1311.1(8)	49(2)
O4_8	9686(8)	5498(4)	502.3(9)	90(4)
N1_8	9898(4)	5354(2)	903.7(8)	46(2)
C1_8	10064(6)	5618(3)	1130.8(8)	39(2)
C2_8	10393(7)	6278(3)	1143.4(9)	50(3)
C3_8	10533(10)	6546(4)	1364.5(9)	74(5)
C4_8	10834(11)	7169(4)	1382.4(10)	86(5)
C5_8	10986(7)	7523(4)	1182.7(10)	66(3)
C6_8	10820(8)	7288(3)	947.5(9)	66(3)
C7_8	10904(13)	7595(5)	721.7(10)	102(7)
C8_8	10674(18)	7327(7)	508.8(12)	145(12)
C9_8	10361(17)	6708(7)	500.4(10)	136(12)
C10_8	10240(10)	6380(4)	710.6(10)	75(5)
C11_8	10490(8)	6650(3)	932.3(9)	57(3)
C12_8	9907(10)	5719(4)	693.9(10)	75(5)
C13_8	9600(3)	4690(2)	888.0(10)	43(2)
C14_8	8943(4)	4613(3)	1056.6(15)	42(2)
C15_8	10213(3)	4154(3)	892.6(13)	56(3)
C16_8	10634(6)	4079(6)	1137.0(16)	63(4)
C17_8	9847(6)	3507(3)	822(2)	59(3)
C18_8	10794(5)	4319(5)	702(2)	74(5)
Br1_9	11486.9(10)	1876.2(8)	8740.0(3)	65.6(4)
Br2_9	11713.3(18)	1785.5(11)	9312.0(4)	112.9(10)
O1_9	9114(4)	6114(3)	8865.8(11)	41.5(18)
O2_9	8624(3)	5186(3)	9007.1(11)	33.9(16)
O3_9	10019(4)	4921(3)	8733.8(8)	44.0(19)
O4_9	10108(6)	4692(4)	9544.5(8)	63(3)
N1_9	10115(4)	4849(2)	9140.3(7)	42(2)
C1_9	10176(6)	4592(3)	8909.7(8)	39(2)
C2_9	10480(7)	3926(3)	8887.5(8)	44(2)
C3_9	10543(7)	3672(3)	8663.2(9)	53(3)
C4_9	10809(8)	3041(4)	8636.7(10)	58(3)
C5_9	11087(7)	2693(3)	8829.2(9)	53(3)
C6_9	11047(7)	2929(3)	9067.3(8)	50(3)
C7_9	11250(11)	2621(5)	9288.9(9)	82(6)
C8_9	11176(16)	2902(7)	9506.9(10)	121(10)
C9_9	10849(13)	3513(6)	9525.6(9)	97(7)
C10_9	10626(8)	3846(4)	9320.9(8)	56(3)
C11_9	10727(8)	3566(3)	9093.2(8)	49(3)
C12_9	10259(7)	4489(3)	9348.0(8)	48(3)
C13_9	9827(3)	5513(2)	9164.9(9)	40(2)

Atom	x	y	z	U_{eq}
C14_9	9136(3)	5613(3)	8995.5(14)	37(2)
C15_9	10450(3)	6041(3)	9167.8(12)	46(2)
C16_9	10821(6)	6129(6)	8925.8(16)	64(4)
C17_9	10108(6)	6686(3)	9244(2)	74(4)
C18_9	11067(5)	5854(6)	9358.8(19)	72(4)
Br1_10	7215(2)	5436.3(10)	10567.1(3)	136.0(14)
Br2_10	5411(2)	5646.0(16)	10526.2(4)	160.9(17)
O1_10	7630(4)	6729(3)	8872.2(13)	43.6(19)
O2_10	7125(4)	5818(2)	9022.7(13)	45.6(19)
O3_10	7863(3)	6563(4)	9445.7(10)	51(2)
O4_10	5315(3)	6470(5)	9346.3(10)	61(2)
N1_10	6589(3)	6569(3)	9394.2(8)	48(2)
C1_10	7254(3)	6462(6)	9531.2(10)	48(3)
C2_10	7189(3)	6234(7)	9782.0(11)	60(3)
C3_10	7835(3)	6100(7)	9913.1(12)	69(4)
C4_10	7791(3)	5870(8)	10147.7(14)	93(5)
C5_10	7113(3)	5790(7)	10252.4(12)	90(4)
C6_10	6419(3)	5917(7)	10126.9(10)	80(4)
C7_10	5670(3)	5886(7)	10207.8(10)	94(5)
C8_10	5049(3)	5988(9)	10062.7(12)	96(5)
C9_10	5120(3)	6155(9)	9822.2(12)	89(5)
C10_10	5825(3)	6238(7)	9732.6(10)	63(3)
C11_10	6474(3)	6128(7)	9881.3(10)	67(3)
C12_10	5877(3)	6434(6)	9476.8(10)	53(3)
C13_10	6639(3)	6829(3)	9149.7(8)	48(3)
C14_10	7198(5)	6429(3)	9008.1(13)	39(2)
C15_10	6701(4)	7577(3)	9136.7(11)	57(3)
C16_10	7481(6)	7841(5)	9215(2)	73(4)
C17_10	6508(8)	7795(4)	8878.9(14)	75(5)
C18_10	6109(7)	7875(4)	9301(2)	84(6)
Br1_11	2603.5(17)	4578(2)	9059.9(6)	160.8(15)
Br2_11	2551(3)	3536(3)	8628.4(8)	207(2)
O1_11	7303(3)	6290(3)	8402.1(12)	43.0(18)
O2_11	6821(4)	5358(3)	8543.1(10)	37.7(17)
O3_11	5602(4)	6246(3)	8655.3(11)	52(2)
O4_11	5307(5)	4941(4)	8002.2(11)	64(3)
N1_11	5464(3)	5611(3)	8324.2(8)	46(2)
C1_11	5267(5)	5804(4)	8554.0(12)	48(3)
C2_11	4654(5)	5447(5)	8669.7(12)	55(3)
C3_11	4454(6)	5635(6)	8893.2(14)	66(4)
C4_11	3865(8)	5320(8)	9003.8(18)	109(7)
C5_11	3453(7)	4846(7)	8887.9(18)	101(6)
C6_11	3650(6)	4607(5)	8660.3(15)	83(4)
C7_11	3310(8)	4108(6)	8517.6(16)	89(5)
C8_11	3527(9)	3950(7)	8294.1(17)	92(5)
C9_11	4083(7)	4309(6)	8184.3(14)	72(4)
C10_11	4459(5)	4789(4)	8311.4(11)	54(3)
C11_11	4248(5)	4948(5)	8545.7(12)	55(3)
C12_11	5114(5)	5102(4)	8199.9(12)	49(3)
C13_11	6146(3)	5887(3)	8223.1(9)	48(3)
C14_11	6803(3)	5857(3)	8409.8(14)	38(2)
C15_11	6014(4)	6531(3)	8084.2(13)	62(3)
C16_11	5991(8)	7132(4)	8246(2)	87(5)
C17_11	6639(6)	6614(6)	7904.9(19)	72(4)
C18_11	5256(5)	6481(6)	7939(2)	99(7)
Br1_12	2651(6)	5790(6)	880.6(17)	194(3)
Br2_12	2544(4)	6780(4)	1320.4(15)	152(2)
O1_12	7212(3)	3962(3)	1647.7(12)	43.3(17)

Atom	x	y	z	U_{eq}
O2_12	6717(4)	4880(3)	1486.9(12)	45.8(18)
O3_12	5486(13)	3981(9)	1389(3)	58(3)
O4_12	5373(14)	5421(11)	2001(3)	80(5)
N1_12	5399(4)	4655(4)	1708.2(13)	49(2)
C1_12	5191(11)	4455(8)	1475(2)	51(3)
C2_12	4609(12)	4834(9)	1339(2)	48(4)
C3_12	4427(12)	4659(10)	1108(2)	59(7)
C4_12	3839(17)	4976(15)	982(3)	95(9)
C5_12	3479(11)	5495(10)	1078(2)	71(5)
C6_12	3658(13)	5718(11)	1314(3)	74(4)
C7_12	3325(13)	6224(11)	1447(3)	85(4)
C8_12	3594(15)	6423(11)	1666(3)	85(4)
C9_12	4143(15)	6062(12)	1792(3)	73(6)
C10_12	4489(10)	5554(8)	1679.3(19)	43(5)
C11_12	4258(10)	5379(9)	1443(2)	48(5)
C12_12	5103(10)	5206(8)	1814(2)	52(6)
C13_12	6109(3)	4401(4)	1817.0(12)	47(5)
C14_12	6717(3)	4399(4)	1629.1(13)	44(2)
C15_12	6016(6)	3781(6)	1970(2)	49(4)
C16_12	5984(18)	3153(5)	1823(4)	68(5)
C17_12	6672(10)	3741(13)	2157(4)	64(4)
C18_12	5279(10)	3841(10)	2108(4)	58(8)
C1S_13	2193(11)	5568(8)	8418(3)	99(6)
C2S_13	2294(10)	5389(8)	8183(3)	92(5)
C3S_13	2778(11)	5737(9)	8050(3)	102(6)
C4S_13	3117(11)	6279(9)	8144(3)	96(6)
C5S_13	3037(11)	6454(8)	8368(3)	93(5)
C6S_13	2549(12)	6110(9)	8511(3)	104(7)
C1S_14	4331(9)	5006(8)	7439(3)	87(5)
C2S_14	3750(8)	5075(8)	7269(3)	89(5)
C3S_14	3726(7)	4674(7)	7075(2)	75(4)
C4S_14	4297(7)	4244(7)	7041(2)	71(4)
C5S_14	4866(8)	4184(8)	7196(2)	78(4)
C6S_14	4889(8)	4554(7)	7406(2)	72(4)
Br1_15	4565.3(8)	2584.7(7)	2591.7(3)	52.6(3)
Br2_15	6151.2(9)	1856.7(7)	2495.0(3)	58.0(4)
O1_15	7431(5)	6123(2)	3419.3(12)	43.7(19)
O2_15	6994(4)	5189(3)	3573.2(11)	42.1(19)
O3_15	6272(3)	5236(2)	3093.2(15)	49(2)
O4_15	8271(3)	3880(3)	3204.7(14)	48(2)
N1_15	7293(2)	4580(2)	3145.3(11)	35.2(18)
C1_15	6544(3)	4699(2)	3071.6(17)	37(2)
C2_15	6107(3)	4158(3)	2960.0(16)	35(2)
C3_15	5361(3)	4254(3)	2902(2)	46(3)
C4_15	4942(3)	3758(3)	2791(2)	46(3)
C5_15	5265(3)	3179(3)	2735.1(18)	39(2)
C6_15	6043(3)	3057(3)	2780.1(15)	33(2)
C7_15	6488(3)	2517(3)	2714.9(16)	42(2)
C8_15	7218(4)	2434(4)	2791(2)	46(3)
C9_15	7581(4)	2901(3)	2934.7(19)	44(3)
C10_15	7213(3)	3467(3)	2988.4(16)	35(2)
C11_15	6458(3)	3559(3)	2907.0(17)	37(2)
C12_15	7632(3)	3974(3)	3127.7(16)	38(2)
C13_15	7763(3)	5121(2)	3233.9(9)	39(2)
C14_15	7345(5)	5516(2)	3420.2(11)	33(2)
C15_15	8159(4)	5504(3)	3034.6(11)	45(3)
C16_15	7619(6)	5926(6)	2880.1(18)	73(4)
C17_15	8771(5)	5934(5)	3152.4(18)	59(4)

Atom	x	y	z	U_{eq}
C18_15	8548(7)	5016(5)	2869.1(18)	77(5)
Br1_16	10341.6(9)	2226.8(7)	3740.4(3)	59.4(4)
Br2_16	10728.1(10)	2197.3(8)	4313.0(3)	68.1(4)
O1_16	8040(3)	6502(3)	3874.7(11)	36.3(16)
O2_16	7581(3)	5591(3)	4039.6(11)	29.1(15)
O3_16	8836(4)	5250(3)	3733.4(8)	39.9(17)
O4_16	9152(5)	5120(3)	4543.6(8)	52(2)
N1_16	9053(4)	5224(2)	4137.5(7)	38.1(19)
C1_16	9056(6)	4944(3)	3909.0(8)	38(2)
C2_16	9318(6)	4265(3)	3890.0(8)	39(2)
C3_16	9326(6)	3990(3)	3667.4(9)	40(2)
C4_16	9617(7)	3368(3)	3641.3(10)	48(3)
C5_16	9931(6)	3036(3)	3832.7(9)	50(3)
C6_16	9957(6)	3297(3)	4068.2(8)	42(2)
C7_16	10234(7)	3020(4)	4288.0(9)	55(3)
C8_16	10183(9)	3311(4)	4505.2(10)	68(4)
C9_16	9814(8)	3904(4)	4525.2(9)	64(4)
C10_16	9553(6)	4222(3)	4321.2(8)	47(3)
C11_16	9615(6)	3926(3)	4094.5(8)	38(2)
C12_16	9250(6)	4889(3)	4347.1(8)	41(2)
C13_16	8827(3)	5907(2)	4157.3(9)	40(2)
C14_16	8087(3)	6017(3)	4011.7(14)	33(2)
C15_16	9480(3)	6395(3)	4123.3(11)	43(2)
C16_16	9759(6)	6418(5)	3866.2(14)	55(3)
C17_16	9223(5)	7073(3)	4195.4(19)	54(3)
C18_16	10148(4)	6201(5)	4294.6(19)	57(3)
Br1_17	12604.0(10)	8657.8(8)	6199.8(4)	72.5(5)
Br2_17	12391.2(11)	8788.9(9)	5624.4(4)	84.5(6)
O1_17	9960(4)	4513(3)	6167.9(12)	40.2(18)
O2_17	9407(3)	5454(3)	6052.9(12)	38.4(17)
O3_17	11010(5)	5665(3)	6261.6(9)	55(2)
O4_17	10510(5)	5948(4)	5464.4(9)	63(3)
N1_17	10811(4)	5762(2)	5858.8(8)	45(2)
C1_17	11083(6)	5990(3)	6082.0(9)	43(2)
C2_17	11448(6)	6637(3)	6091.9(9)	48(3)
C3_17	11689(6)	6872(3)	6310.4(9)	45(2)
C4_17	12015(7)	7486(3)	6326.5(10)	54(3)
C5_17	12115(6)	7857(3)	6127.0(9)	51(3)
C6_17	11882(6)	7644(3)	5894.1(9)	49(2)
C7_17	11933(8)	7960(4)	5668.5(10)	67(4)
C8_17	11636(9)	7715(4)	5459.4(11)	70(4)
C9_17	11287(9)	7110(4)	5453.7(10)	68(4)
C10_17	11206(6)	6771(3)	5663.7(9)	49(3)
C11_17	11519(6)	7018(3)	5881.6(8)	46(2)
C12_17	10817(6)	6133(3)	5650.4(9)	49(3)
C13_17	10478(3)	5111(2)	5843.7(9)	47(3)
C14_17	9913(4)	5021(3)	6042.3(13)	34(2)
C15_17	11051(4)	4558(3)	5803.3(12)	51(3)
C16_17	11587(6)	4429(6)	6020.6(17)	68(4)
C17_17	10618(6)	3937(3)	5739(2)	63(4)
C18_17	11526(6)	4738(5)	5586.3(18)	73(5)
Br1_18	7365.9(11)	8418.9(11)	7503.0(5)	102.9(8)
Br2_18	8747.5(10)	9378.1(8)	7433.4(3)	70.9(5)
O1_18	9945(4)	4947(2)	6663.3(12)	40.4(17)
O2_18	9382(3)	5876(3)	6538.3(11)	39.4(17)
O3_18	9104(4)	5764(2)	7055.0(14)	45.4(19)
O4_18	10824(3)	7227(3)	6812.3(14)	44(2)
N1_18	9998(3)	6475.7(19)	6942.5(11)	37.9(19)

Atom	x	y	z	U_{eq}
C1_18	9334(4)	6319(2)	7056.9(17)	35(2)
C2_18	8911(4)	6851(2)	7169.3(19)	40(2)
C3_18	8271(5)	6703(3)	7286(2)	62(4)
C4_18	7841(6)	7198(3)	7383(3)	70(4)
C5_18	8067(4)	7830(2)	7374(2)	55(3)
C6_18	8767(4)	8010(2)	7278.9(19)	43(2)
C7_18	9118(4)	8628(2)	7274.0(18)	45(3)
C8_18	9761(5)	8747(3)	7157.6(19)	46(3)
C9_18	10126(4)	8249(2)	7038.8(18)	39(2)
C10_18	9840(4)	7629(2)	7041.1(18)	38(2)
C11_18	9170(4)	7502(2)	7161.4(17)	37(2)
C12_18	10263(4)	7107(2)	6921.7(17)	36(2)
C13_18	10410(3)	5956(2)	6823.3(9)	35(2)
C14_18	9862(4)	5554(3)	6664.6(13)	37(2)
C15_18	10983(3)	5586(3)	6990.1(11)	44(2)
C16_18	10616(6)	5186(5)	7186.2(17)	69(4)
C17_18	11458(6)	5138(5)	6837.7(17)	64(4)
C18_18	11521(6)	6086(5)	7113(2)	69(4)
C1S_19	2156(10)	4797(8)	1552(2)	84(5)
C2S_19	2219(13)	4978(10)	1791(3)	111(7)
C3S_19	2683(9)	4633(8)	1943(2)	82(4)
C4S_19	3071(9)	4112(8)	1861(2)	83(4)
C5S_19	3024(10)	3934(8)	1635(2)	87(5)
C6S_19	2563(11)	4278(9)	1472(2)	94(6)
C1S_20	-231(9)	2807(8)	7378(5)	133(10)
C2S_20	52(9)	2184(7)	7390(4)	104(7)
C3S_20	805(9)	2096(7)	7367(3)	90(4)
C4S_20	1250(8)	2600(8)	7302(4)	98(5)
C5S_20	974(9)	3180(7)	7261(3)	97(5)
C6S_20	217(9)	3306(6)	7302(4)	105(6)
C1S_21	1904(12)	6536(9)	2993(2)	102(7)
C2S_21	1529(14)	5974(10)	2926(3)	123(8)
C3S_21	1098(13)	5674(9)	3089(3)	110(7)
C4S_21	1067(11)	5907(9)	3317(3)	108(6)
C5S_21	1436(13)	6428(10)	3387(3)	113(7)
C6S_21	1847(12)	6777(9)	3222(3)	105(6)
C1S_22	1261(9)	7972(7)	7796(4)	103(6)
C2S_22	1695(8)	7426(8)	7763(4)	109(6)
C3S_22	1345(9)	6862(7)	7700(4)	105(6)
C4S_22	581(9)	6844(7)	7662(3)	99(5)
C5S_22	155(8)	7348(9)	7702(3)	105(6)
C6S_22	488(9)	7940(7)	7764(3)	95(5)
N2_23	6985(6)	5911(4)	1119.3(18)	45(2)
C19_23	6716(11)	6352(6)	1045(3)	74(5)
C20_23	6339(18)	6956(9)	962(6)	146(12)
N2_24	8606(6)	3090(5)	1555.2(18)	48(2)
C19_24	8857(7)	2616(5)	1601(2)	46(3)
C20_24	9241(11)	2004(6)	1669(3)	74(5)
N2_25	8639(7)	7168(5)	8467.3(19)	52(3)
C19_25	8757(9)	7628(5)	8376(2)	55(3)
C20_25	8886(16)	8274(6)	8270(4)	103(8)
N2_26	7195(6)	4325(4)	8918.7(18)	47(2)
C19_26	6997(9)	3870(6)	8998(3)	62(4)
C20_26	6745(13)	3258(8)	9108(5)	112(8)
Br1_27	2850(4)	5437(4)	684.5(11)	194(3)
Br2_27	2549(3)	6505(3)	1085.3(10)	152(2)
O1_27	7212(3)	3962(3)	1647.7(12)	43.3(17)
O2_27	6717(4)	4880(3)	1486.9(12)	45.8(18)

Atom	x	y	z	U_{eq}
O3_27	5610(8)	3862(7)	1332.3(18)	58(3)
O4_27	5079(10)	5310(9)	1916(2)	80(5)
N1_27	5345(3)	4561(4)	1632.2(11)	49(2)
C1_27	5240(9)	4320(7)	1398.5(17)	51(3)
C2_27	4657(8)	4627(7)	1238.1(15)	50(4)
C3_27	4590(9)	4423(8)	1005.2(16)	60(5)
C4_27	4032(14)	4688(12)	852(2)	95(9)
C5_27	3575(10)	5177(9)	925.5(19)	71(5)
C6_27	3651(11)	5445(9)	1159.2(19)	74(4)
C7_27	3261(12)	5974(9)	1261.0(19)	85(4)
C8_27	3385(15)	6178(12)	1491(2)	85(4)
C9_27	3924(11)	5876(10)	1641.0(19)	73(6)
C10_27	4342(9)	5369(8)	1556.2(16)	56(5)
C11_27	4226(8)	5162(7)	1316.3(15)	54(4)
C12_27	4950(8)	5092(7)	1716.2(16)	54(4)
C13_27	6008(3)	4336(3)	1775.9(11)	51(4)
C14_27	6717(3)	4399(4)	1629.1(13)	44(2)
C15_27	5887(4)	3699(4)	1916.4(16)	50(4)
C16_27	5911(13)	3086(4)	1761(3)	68(5)
C17_27	6491(8)	3649(9)	2119(3)	64(4)
C18_27	5110(6)	3733(8)	2032(3)	64(7)
N2_28	9794(6)	3474(5)	6561.6(18)	44(2)
C19_28	10002(8)	3003(5)	6628(2)	56(3)
C20_28	10325(18)	2370(7)	6703(4)	129(12)
N2_29	8092(5)	6304(4)	6201.2(17)	37(2)
C19_29	7848(6)	6761(5)	6132(2)	49(3)
C20_29	7534(10)	7391(7)	6051(4)	87(6)
N2_30	6089(5)	4799(4)	4024.0(17)	39(2)
C19_30	5853(7)	4335(5)	4083(2)	49(3)
C20_30	5536(11)	3729(6)	4178(3)	80(5)
N2_31	3830(9)	4803(9)	2463(3)	95(5)
C19_31	4341(9)	5106(9)	2474(3)	75(4)
C20_31	5008(9)	5530(9)	2510(3)	83(5)
N2_32	7489(6)	7577(4)	3527.7(18)	45(2)
C19_32	7682(9)	8035(5)	3450(3)	57(4)
C20_32	7920(20)	8656(8)	3343(5)	160(14)
N2_33	10125(14)	2252(16)	5073(3)	182(15)
C19_33	9723(12)	2546(11)	5172(3)	97(7)
C20_33	9158(16)	2950(15)	5290(6)	156(13)

Table S27. Anisotropic Displacement Parameters (×10^4) for ytb-e36_br2. The anisotropic displacement factor exponent takes the form: $-2\pi^2[h^2a^{*2} \times U_{11} + ... + 2hka^* \times b^* \times U_{12}]$

Atom	U_{11}	U_{22}	U_{33}	U_{23}	U_{13}	U_{12}
Rh1	45.5(4)	25.8(5)	35.7(4)	5.2(3)	7.5(3)	-2.2(4)
Rh02	38.4(4)	21.9(4)	35.8(4)	0.2(3)	10.9(3)	-2.5(3)
Rh2	44.3(4)	26.4(5)	40.2(4)	6.9(4)	8.2(3)	-1.9(4)
Rh3	41.9(4)	26.0(5)	33.5(4)	2.4(3)	7.2(3)	3.2(4)
Rh4	42.9(4)	28.2(5)	36.0(4)	3.1(3)	8.1(3)	2.7(4)
Rh5	38.0(4)	23.9(5)	36.3(4)	2.4(3)	7.4(3)	-0.9(3)
Rh6	36.0(4)	22.5(4)	36.4(4)	1.7(3)	6.8(3)	-1.1(3)
Rh06	37.5(4)	21.8(4)	35.6(4)	1.1(3)	10.7(3)	-1.5(3)
Br1_1	137.9(15)	35.5(8)	32.7(6)	3.3(6)	6.7(7)	10.1(9)
Br2_1	107.3(16)	153(2)	61.7(10)	15.6(12)	-25.3(10)	20.4(16)
O1_1	48(4)	32(5)	37(4)	-1(3)	2(3)	-6(3)

Atom	U_{11}	U_{22}	U_{33}	U_{23}	U_{13}	U_{12}
O2_1	48(4)	29(4)	37(4)	8(3)	8(3)	-5(3)
O3_1	48(4)	59(6)	44(4)	5(4)	13(3)	-2(4)
O4_1	53(5)	79(8)	56(5)	18(5)	18(4)	0(5)
N1_1	52(4)	46(6)	31(4)	3(4)	12(3)	-2(4)
C1_1	48(4)	45(7)	31(4)	-9(4)	12(3)	-4(5)
C2_1	58(5)	23(6)	39(4)	-2(4)	12(4)	3(5)
C3_1	73(7)	28(7)	39(4)	0(4)	19(4)	-3(6)
C4_1	80(6)	49(9)	42(5)	6(5)	12(5)	-2(7)
C5_1	80(6)	24(7)	49(6)	-8(5)	13(4)	6(5)
C6_1	67(5)	25(6)	44(5)	-1(5)	-2(4)	-4(5)
C7_1	68(5)	34(8)	63(7)	10(6)	-16(5)	-3(6)
C8_1	65(7)	34(8)	80(6)	13(6)	-9(5)	-1(6)
C9_1	50(5)	65(10)	73(7)	15(7)	-4(5)	-10(6)
C10_1	52(5)	55(9)	51(5)	16(6)	4(4)	-10(6)
C11_1	57(5)	20(6)	44(4)	0(4)	2(4)	-7(5)
C12_1	51(5)	64(10)	45(5)	8(5)	12(4)	-2(5)
C13_1	53(6)	30(5)	39(4)	6(4)	4(4)	-11(4)
C14_1	43(5)	27(4)	37(5)	1(4)	8(4)	-6(3)
C15_1	66(6)	29(5)	43(5)	-10(4)	2(4)	-11(5)
C16_1	79(8)	27(7)	64(8)	5(6)	17(6)	0(6)
C17_1	88(10)	29(7)	52(6)	11(5)	9(6)	-17(7)
C18_1	74(8)	45(9)	64(8)	-8(7)	-6(6)	-19(7)
Br1_2	45.2(8)	138(2)	87.3(11)	0.4(12)	-4.7(8)	0.6(10)
Br2_2	78.3(12)	112.5(19)	116.4(15)	2.9(14)	-0.7(11)	48.4(13)
O1_2	47(4)	35(4)	35(4)	9(3)	16(3)	5(3)
O2_2	46(4)	34(4)	44(4)	2(3)	22(3)	0(3)
O3_2	44(4)	45(5)	49(4)	-13(4)	8(3)	-13(3)
O4_2	52(5)	73(7)	58(5)	-25(5)	9(4)	-6(5)
N1_2	35(4)	41(5)	35(4)	-6(3)	18(3)	-9(3)
C1_2	45(5)	42(6)	40(5)	-4(4)	6(3)	-7(4)
C2_2	44(5)	41(6)	42(5)	-8(4)	7(4)	3(4)
C3_2	57(6)	67(10)	63(7)	-22(6)	3(5)	-9(6)
C4_2	58(6)	58(8)	72(8)	-19(6)	-5(6)	-20(6)
C5_2	62(7)	65(8)	45(6)	-1(5)	-5(5)	-6(6)
C6_2	45(5)	51(6)	42(5)	-2(4)	2(4)	3(5)
C7_2	51(6)	53(7)	62(6)	3(5)	25(5)	11(5)
C8_2	58(6)	74(10)	75(8)	-9(7)	14(6)	15(6)
C9_2	54(6)	62(8)	60(7)	-26(6)	11(5)	7(6)
C10_2	41(4)	46(6)	47(5)	-14(5)	17(4)	-5(4)
C11_2	43(4)	38(6)	44(5)	-8(4)	11(4)	0(4)
C12_2	45(5)	40(6)	43(5)	-9(4)	17(4)	-7(4)
C13_2	35(4)	50(7)	47(5)	-1(4)	15(4)	-7(4)
C14_2	47(6)	33(5)	47(5)	0(4)	18(4)	2(4)
C15_2	59(6)	68(7)	45(6)	10(5)	18(4)	-5(6)
C16_2	87(11)	64(8)	76(9)	17(7)	-16(8)	-7(8)
C17_2	70(7)	93(13)	40(6)	11(7)	7(5)	4(8)
C18_2	88(11)	126(17)	80(10)	36(10)	49(9)	29(12)
Br1_3	84.6(18)	72(2)	719(14)	-88(4)	-129(4)	0.3(15)
Br2_3	83.2(12)	49.3(11)	112.9(14)	-21.2(10)	-5.8(10)	-15.1(9)
O1_3	57(5)	40(4)	42(4)	-3(4)	10(4)	-3(4)
O2_3	46(4)	41(5)	37(4)	-1(3)	4(3)	-1(4)
O3_3	51(5)	43(4)	46(4)	7(4)	4(4)	3(4)
O4_3	47(4)	60(6)	58(5)	-15(4)	9(4)	7(4)
N1_3	43(4)	46(5)	37(4)	-4(4)	9(3)	3(3)
C1_3	43(4)	41(5)	41(5)	6(4)	1(4)	-3(4)
C2_3	44(4)	43(5)	34(5)	2(4)	4(4)	-5(4)
C3_3	43(4)	59(8)	50(6)	-14(6)	9(4)	-4(5)
C4_3	59(7)	58(7)	78(9)	-1(7)	-7(7)	-10(5)

Atom	U_{11}	U_{22}	U_{33}	U_{23}	U_{13}	U_{12}
C5_3	61(6)	61(8)	164(18)	-28(10)	-33(9)	-11(6)
C6_3	64(6)	36(6)	68(8)	5(6)	-9(6)	-6(4)
C7_3	64(6)	37(6)	77(9)	-5(6)	-9(6)	-10(5)
C8_3	63(6)	43(7)	89(10)	-10(7)	-5(6)	1(5)
C9_3	57(7)	46(6)	72(8)	-9(6)	2(6)	1(5)
C10_3	52(5)	44(6)	50(6)	-5(5)	11(4)	-2(4)
C11_3	52(5)	42(5)	39(5)	1(5)	13(4)	-6(4)
C12_3	47(4)	41(5)	42(5)	-5(4)	12(4)	-1(4)
C13_3	42(5)	41(5)	35(5)	-1(4)	11(4)	8(4)
C14_3	42(5)	41(4)	24(4)	-6(4)	0(3)	0(4)
C15_3	61(6)	53(7)	42(5)	-4(4)	19(4)	-4(5)
C16_3	83(9)	68(10)	54(7)	17(7)	6(6)	-9(7)
C17_3	73(9)	91(13)	60(8)	-8(8)	13(6)	-21(8)
C18_3	94(12)	71(10)	56(8)	-18(7)	41(8)	-11(8)
Br1_4	86.5(10)	40.6(8)	42.3(6)	5.4(6)	21.3(6)	5.8(7)
Br2_4	93.5(12)	70.9(12)	41.5(7)	8.5(7)	7.1(7)	23.0(9)
O1_4	61(5)	28(5)	43(4)	8(3)	-4(3)	0(4)
O2_4	55(5)	32(4)	45(4)	15(3)	-1(4)	6(4)
O3_4	48(4)	107(9)	60(5)	35(6)	4(4)	-21(5)
O4_4	46(4)	87(8)	55(5)	25(5)	6(4)	-1(5)
N1_4	44(4)	70(8)	31(4)	6(4)	6(3)	-3(5)
C1_4	47(4)	51(8)	40(5)	1(5)	11(3)	4(5)
C2_4	52(5)	66(10)	53(5)	21(6)	9(4)	6(6)
C3_4	58(6)	84(12)	68(7)	26(8)	17(6)	1(7)
C4_4	58(6)	85(12)	67(7)	27(7)	21(5)	-2(8)
C5_4	68(5)	39(8)	55(6)	5(6)	16(4)	3(6)
C6_4	62(5)	38(7)	42(5)	2(5)	5(4)	7(5)
C7_4	65(5)	49(8)	42(5)	-16(5)	1(4)	6(6)
C8_4	65(6)	81(12)	47(6)	10(6)	-1(5)	12(7)
C9_4	52(6)	81(12)	49(5)	11(6)	4(4)	10(7)
C10_4	50(5)	52(9)	52(5)	11(5)	3(4)	-2(5)
C11_4	53(5)	33(7)	41(5)	2(4)	5(4)	0(5)
C12_4	47(4)	29(7)	50(5)	9(5)	5(3)	-3(5)
C13_4	53(6)	48(5)	35(4)	6(4)	11(4)	-6(5)
C14_4	42(5)	32(4)	40(4)	14(4)	10(4)	-1(4)
C15_4	59(6)	47(6)	47(5)	3(5)	2(5)	-7(5)
C16_4	65(7)	67(11)	75(9)	-13(8)	0(6)	4(7)
C17_4	93(11)	28(7)	66(7)	18(6)	21(7)	6(7)
C18_4	79(9)	51(9)	68(7)	-5(7)	-9(7)	-13(8)
Br1_5	157(3)	207(4)	231(4)	-116(3)	141(3)	-129(3)
Br2_5	99.3(15)	82.6(15)	103.4(14)	-12.4(11)	16.8(11)	-48.1(13)
O1_5	38(3)	28(4)	35(3)	13(3)	-12(3)	1(3)
O2_5	41(4)	34(4)	42(4)	4(3)	11(3)	2(3)
O3_5	55(5)	55(6)	67(5)	-20(4)	30(4)	-12(4)
O4_5	51(5)	50(6)	52(4)	-13(4)	5(3)	-1(4)
N1_5	44(4)	36(5)	43(4)	-7(4)	11(4)	-7(4)
C1_5	37(5)	34(6)	53(5)	1(4)	20(4)	-6(4)
C2_5	38(5)	46(7)	77(7)	-9(5)	14(5)	-16(5)
C3_5	48(6)	60(9)	74(7)	-12(6)	11(5)	-12(6)
C4_5	74(10)	99(12)	89(10)	-20(8)	35(8)	-40(9)
C5_5	58(8)	87(12)	101(9)	-16(8)	37(7)	-34(8)
C6_5	48(7)	51(8)	90(7)	-11(6)	17(6)	-19(6)
C7_5	36(6)	59(8)	86(7)	-3(6)	-2(5)	-26(6)
C8_5	74(8)	37(8)	94(8)	-12(7)	11(7)	-20(7)
C9_5	69(8)	39(7)	64(7)	-5(5)	-1(6)	-18(6)
C10_5	34(5)	39(6)	59(5)	-5(4)	-3(4)	-4(4)
C11_5	42(6)	44(7)	71(6)	-5(5)	6(5)	-10(5)
C12_5	39(5)	41(7)	56(5)	-10(4)	6(4)	-3(4)

Atom	U_{11}	U_{22}	U_{33}	U_{23}	U_{13}	U_{12}
C13_5	38(5)	44(6)	44(5)	7(5)	8(4)	0(4)
C14_5	36(4)	32(5)	46(5)	0(4)	8(4)	-1(4)
C15_5	51(6)	36(6)	90(8)	8(5)	-3(5)	-6(5)
C16_5	71(10)	35(7)	112(10)	5(7)	21(8)	12(7)
C17_5	79(9)	42(9)	81(9)	15(7)	5(7)	-9(7)
C18_5	80(10)	66(13)	172(19)	38(12)	-56(11)	-13(10)
Br1_6	79.2(10)	37.4(8)	48.3(7)	5.4(6)	-7.2(6)	-8.3(7)
Br2_6	87.8(11)	90.4(14)	38.9(7)	9.3(7)	6.0(7)	-27.4(10)
O1_6	50(4)	34(5)	42(4)	3(3)	17(3)	2(4)
O2_6	45(4)	26(4)	48(4)	-2(3)	16(3)	-3(3)
O3_6	52(5)	62(6)	40(4)	4(4)	12(3)	8(4)
O4_6	55(4)	88(8)	48(4)	13(5)	23(4)	10(5)
N1_6	53(4)	36(6)	34(4)	2(4)	12(3)	4(4)
C1_6	48(4)	34(7)	44(4)	-7(4)	11(3)	-5(5)
C2_6	49(4)	27(6)	48(4)	3(4)	13(4)	-8(5)
C3_6	51(5)	52(9)	57(6)	14(6)	1(5)	-17(6)
C4_6	53(6)	73(11)	49(5)	-1(6)	-2(4)	-3(7)
C5_6	60(5)	39(8)	37(5)	-8(5)	-5(4)	-7(5)
C6_6	59(5)	34(7)	40(4)	3(5)	3(4)	-6(5)
C7_6	73(6)	41(8)	54(6)	-5(6)	16(5)	-13(6)
C8_6	70(6)	67(10)	39(5)	2(6)	20(5)	-21(7)
C9_6	63(6)	59(9)	41(5)	6(5)	19(4)	-17(6)
C10_6	52(5)	43(8)	45(5)	10(5)	15(4)	-5(5)
C11_6	54(5)	44(8)	34(4)	-2(4)	5(3)	-11(5)
C12_6	55(4)	17(6)	39(4)	-4(4)	14(3)	-1(4)
C13_6	50(6)	22(5)	38(4)	5(4)	14(4)	3(4)
C14_6	41(5)	29(4)	34(4)	-1(3)	6(4)	-6(4)
C15_6	78(7)	18(5)	53(5)	3(4)	23(5)	19(5)
C16_6	97(8)	42(9)	64(8)	-5(7)	6(7)	-20(7)
C17_6	88(9)	31(8)	63(6)	19(6)	30(6)	33(7)
C18_6	200(20)	41(10)	75(9)	-10(8)	73(12)	15(12)
Br1_7	85.8(13)	64.2(12)	145.2(18)	-29.9(12)	70.9(13)	-5.4(10)
Br2_7	81.4(11)	38.6(9)	104.9(12)	-16.8(9)	32.6(10)	5.5(8)
O1_7	59(5)	36(4)	32(4)	5(3)	8(3)	3(4)
O2_7	55(5)	23(4)	52(4)	1(3)	4(3)	2(3)
O3_7	56(5)	34(4)	59(5)	-3(4)	18(4)	-4(4)
O4_7	47(4)	38(5)	57(5)	4(4)	19(3)	6(4)
N1_7	51(4)	34(4)	35(4)	-2(4)	12(4)	3(3)
C1_7	50(5)	29(4)	45(6)	-4(4)	13(4)	3(4)
C2_7	53(6)	31(5)	49(6)	-7(5)	12(5)	4(4)
C3_7	63(6)	48(7)	50(6)	-5(6)	22(5)	1(5)
C4_7	73(9)	51(6)	65(8)	-3(6)	39(7)	-1(5)
C5_7	47(6)	50(6)	67(8)	-6(6)	19(5)	4(5)
C6_7	38(5)	32(5)	48(6)	-6(5)	8(4)	9(4)
C7_7	68(6)	40(6)	45(6)	7(5)	9(5)	-1(5)
C8_7	65(7)	28(6)	68(8)	-15(6)	12(6)	-15(5)
C9_7	55(7)	32(5)	56(7)	5(5)	10(5)	0(4)
C10_7	50(5)	24(4)	31(5)	1(4)	12(4)	10(4)
C11_7	56(5)	27(4)	41(5)	8(4)	19(5)	15(4)
C12_7	45(4)	24(4)	38(5)	8(4)	14(4)	14(4)
C13_7	49(6)	33(5)	44(5)	0(4)	19(4)	1(4)
C14_7	53(6)	34(4)	41(5)	7(4)	14(4)	2(4)
C15_7	74(7)	21(6)	53(6)	-1(4)	3(4)	1(4)
C16_7	106(11)	30(7)	55(7)	10(6)	30(7)	22(7)
C17_7	67(8)	22(6)	76(8)	1(6)	2(6)	3(5)
C18_7	107(12)	27(7)	101(12)	-7(7)	-42(9)	7(7)
Br1_8	74.1(11)	38.6(10)	136.5(16)	-18.7(10)	39.0(11)	-19.0(8)
Br2_8	88.7(13)	58.4(12)	158(2)	55.3(13)	-18.3(13)	-28.5(11)

Atom	U_{11}	U_{22}	U_{33}	U_{23}	U_{13}	U_{12}
O1_8	46(4)	31(4)	38(4)	7(3)	13(3)	-3(3)
O2_8	47(4)	35(5)	44(4)	9(3)	11(3)	-4(3)
O3_8	69(6)	33(5)	44(4)	1(3)	11(4)	-11(4)
O4_8	129(11)	82(9)	58(5)	12(5)	-1(6)	-56(8)
N1_8	50(5)	38(5)	49(4)	-2(3)	9(4)	-7(4)
C1_8	49(6)	23(5)	46(4)	1(3)	18(4)	6(4)
C2_8	63(7)	24(5)	65(5)	0(4)	28(5)	0(5)
C3_8	105(12)	54(8)	65(6)	-12(6)	44(7)	-24(8)
C4_8	106(13)	52(8)	103(9)	-17(7)	43(9)	-20(8)
C5_8	44(7)	41(8)	116(8)	-5(6)	25(6)	-16(6)
C6_8	65(8)	26(6)	108(7)	14(5)	10(7)	-5(6)
C7_8	112(16)	72(12)	122(9)	38(8)	-9(9)	-54(11)
C8_8	220(30)	96(14)	117(11)	56(10)	-34(14)	-88(17)
C9_8	210(30)	109(14)	83(9)	45(9)	-39(12)	-100(18)
C10_8	102(12)	52(7)	70(6)	20(5)	-24(7)	-31(8)
C11_8	61(8)	40(6)	70(5)	8(5)	0(5)	-15(6)
C12_8	107(12)	55(7)	62(5)	16(5)	-5(6)	-39(8)
C13_8	53(6)	37(5)	39(5)	7(4)	11(4)	-8(4)
C14_8	47(5)	40(6)	40(5)	10(4)	8(4)	-1(4)
C15_8	57(6)	43(6)	70(6)	-3(6)	12(5)	1(5)
C16_8	61(8)	56(10)	73(7)	0(7)	7(6)	11(7)
C17_8	57(8)	39(7)	81(9)	-7(7)	12(7)	8(5)
C18_8	76(10)	63(11)	87(9)	-7(8)	35(8)	2(8)
Br1_9	71.2(10)	38.1(9)	87.7(10)	-8.3(8)	4.7(8)	11.4(7)
Br2_9	176(2)	76.4(15)	88.5(12)	37.3(11)	38.2(14)	74.4(16)
O1_9	45(4)	40(5)	40(4)	7(3)	5(3)	1(4)
O2_9	41(4)	27(4)	33(3)	5(3)	-5(3)	3(3)
O3_9	45(4)	52(5)	35(3)	7(3)	4(3)	0(4)
O4_9	88(7)	62(7)	39(4)	7(4)	10(4)	21(6)
N1_9	52(5)	43(5)	32(3)	2(3)	5(4)	8(4)
C1_9	50(6)	32(5)	36(4)	1(3)	3(4)	-7(4)
C2_9	52(6)	40(5)	42(4)	0(4)	7(4)	4(5)
C3_9	64(8)	45(6)	50(5)	-10(5)	6(5)	-4(6)
C4_9	77(9)	53(7)	44(5)	-15(5)	3(6)	12(6)
C5_9	47(7)	55(8)	57(5)	-6(5)	0(5)	-4(6)
C6_9	63(8)	37(6)	52(4)	10(4)	16(5)	12(6)
C7_9	108(13)	80(11)	58(5)	26(6)	23(7)	51(10)
C8_9	200(30)	105(13)	56(7)	13(8)	-8(11)	87(16)
C9_9	157(18)	98(12)	36(6)	9(7)	18(8)	69(13)
C10_9	80(9)	47(7)	43(4)	4(4)	11(5)	16(6)
C11_9	73(8)	30(6)	46(4)	3(4)	6(5)	7(5)
C12_9	61(7)	45(6)	40(4)	9(4)	12(4)	11(5)
C13_9	39(5)	41(5)	42(5)	7(4)	6(4)	2(4)
C14_9	43(4)	33(5)	34(5)	-2(4)	1(4)	-4(4)
C15_9	56(6)	39(6)	43(5)	-1(5)	-8(4)	-7(4)
C16_9	67(9)	59(10)	68(7)	7(7)	11(6)	-27(8)
C17_9	81(10)	42(7)	95(11)	-26(8)	-23(8)	0(7)
C18_9	59(8)	81(12)	73(8)	18(8)	-19(6)	-11(8)
Br1_10	311(4)	54.0(12)	41.4(8)	10.9(8)	-20.4(14)	-59.8(19)
Br2_10	303(5)	124(2)	62.1(11)	-4.3(13)	82.4(19)	-76(3)
O1_10	52(4)	27(4)	52(4)	-1(3)	15(3)	-3(4)
O2_10	58(5)	33(4)	46(4)	-2(4)	15(4)	2(4)
O3_10	70(5)	34(5)	49(4)	-1(4)	6(4)	4(4)
O4_10	69(5)	56(6)	59(5)	4(5)	23(4)	18(5)
N1_10	71(5)	26(6)	47(4)	7(4)	19(4)	9(5)
C1_10	77(5)	29(7)	41(5)	-2(5)	14(4)	8(5)
C2_10	107(7)	30(7)	44(5)	4(5)	10(5)	1(7)
C3_10	116(9)	44(9)	46(6)	10(6)	-3(6)	-4(8)

Atom	U_{11}	U_{22}	U_{33}	U_{23}	U_{13}	U_{12}
C4_10	163(12)	65(12)	48(6)	10(7)	-15(7)	-44(10)
C5_10	178(11)	46(10)	48(7)	0(7)	1(6)	-38(10)
C6_10	167(10)	35(8)	38(5)	-4(5)	16(5)	-35(8)
C7_10	186(11)	39(9)	62(8)	-22(7)	55(7)	-31(10)
C8_10	161(12)	65(11)	69(7)	-10(8)	75(7)	-22(12)
C9_10	114(9)	99(14)	55(7)	-21(8)	42(7)	-30(10)
C10_10	104(7)	44(8)	44(5)	0(6)	30(4)	0(7)
C11_10	117(7)	46(9)	38(5)	-3(5)	23(5)	4(7)
C12_10	72(5)	45(8)	45(5)	5(5)	26(4)	15(6)
C13_10	55(7)	44(5)	44(5)	10(5)	12(4)	13(5)
C14_10	51(6)	34(4)	31(5)	1(4)	5(4)	0(4)
C15_10	89(8)	42(6)	42(5)	16(5)	33(5)	17(5)
C16_10	106(9)	41(9)	73(9)	5(7)	20(7)	7(7)
C17_10	105(12)	62(11)	60(6)	33(7)	27(7)	47(10)
C18_10	122(13)	50(10)	85(10)	-24(9)	57(10)	1(9)
Br1_11	105.1(19)	238(4)	144(2)	37(3)	69.1(18)	-37(2)
Br2_11	174(4)	245(5)	205(4)	19(4)	42(3)	-144(4)
O1_11	46(4)	40(5)	43(4)	9(4)	-3(3)	6(3)
O2_11	54(4)	26(4)	33(3)	8(3)	0(3)	7(3)
O3_11	58(5)	44(5)	55(5)	4(4)	11(4)	2(4)
O4_11	68(6)	69(7)	56(4)	-19(5)	11(4)	-8(5)
N1_11	50(5)	45(6)	43(4)	2(4)	-1(4)	12(4)
C1_11	50(6)	41(6)	53(5)	5(4)	10(4)	7(4)
C2_11	49(6)	56(7)	60(6)	14(5)	15(5)	8(5)
C3_11	60(7)	76(10)	63(6)	10(6)	22(6)	11(7)
C4_11	92(12)	160(18)	77(10)	18(10)	32(9)	-29(11)
C5_11	69(10)	144(16)	93(9)	28(9)	21(8)	-15(10)
C6_11	48(7)	110(12)	91(8)	32(7)	8(6)	-14(7)
C7_11	70(10)	99(12)	99(9)	39(8)	11(7)	-29(9)
C8_11	86(11)	94(13)	98(9)	27(8)	3(8)	-31(10)
C9_11	70(8)	59(9)	88(9)	8(6)	15(7)	-6(7)
C10_11	45(6)	54(7)	64(6)	14(5)	0(4)	20(5)
C11_11	47(6)	55(7)	64(6)	19(5)	10(5)	8(5)
C12_11	47(5)	52(7)	49(5)	-4(4)	0(4)	13(4)
C13_11	53(5)	48(7)	42(5)	0(5)	3(4)	6(5)
C14_11	43(5)	27(5)	44(5)	10(4)	10(4)	12(3)
C15_11	50(6)	69(7)	68(7)	25(5)	0(5)	-3(6)
C16_11	86(12)	64(9)	114(12)	15(8)	12(10)	32(9)
C17_11	72(8)	82(13)	63(8)	15(8)	11(6)	-8(9)
C18_11	69(8)	105(16)	122(14)	78(12)	-31(9)	-29(10)
Br1_12	177(5)	229(7)	169(5)	-28(4)	-82(4)	103(5)
Br2_12	113(3)	162(5)	180(4)	44(4)	10(3)	81(3)
O1_12	44(2)	40(3)	45(3)	3(2)	6(2)	-3(2)
O2_12	46(3)	43(3)	48(3)	4(2)	7(2)	0(2)
O3_12	60(5)	53(4)	61(5)	-3(4)	9(4)	4(3)
O4_12	94(12)	77(10)	69(9)	-7(7)	9(7)	16(10)
N1_12	49(3)	49(3)	50(3)	0(2)	5(2)	0(2)
C1_12	51(3)	50(4)	51(3)	0(2)	3(2)	2(2)
C2_12	48(5)	49(5)	48(5)	0(3)	2(3)	-2(3)
C3_12	46(13)	90(20)	47(8)	-7(9)	6(8)	-10(12)
C4_12	87(13)	111(16)	86(11)	-30(11)	-33(11)	21(12)
C5_12	65(11)	74(13)	75(7)	7(7)	3(7)	-6(9)
C6_12	73(4)	73(4)	75(4)	1.7(19)	2.4(19)	1.7(19)
C7_12	66(7)	85(8)	104(9)	3(7)	6(7)	21(7)
C8_12	66(7)	85(8)	104(9)	3(7)	6(7)	21(7)
C9_12	69(11)	61(11)	90(11)	-15(8)	6(8)	20(9)
C10_12	56(11)	28(11)	48(9)	17(7)	27(7)	-5(8)
C11_12	53(10)	40(10)	51(9)	12(7)	28(7)	-10(8)

Atom	U_{11}	U_{22}	U_{33}	U_{23}	U_{13}	U_{12}
C12_12	67(12)	46(10)	45(10)	15(8)	27(8)	9(9)
C13_12	49(7)	39(11)	52(10)	6(9)	11(5)	1(8)
C14_12	45(3)	44(3)	44(3)	2(2)	5(2)	0(2)
C15_12	60(8)	38(8)	49(9)	6(6)	5(6)	-6(8)
C16_12	104(13)	47(7)	55(10)	-1(6)	26(9)	-31(8)
C17_12	64(5)	65(6)	63(5)	-2(5)	-3(4)	0(5)
C18_12	59(8)	58(9)	58(9)	1(5)	5(5)	-2(5)
C1S_13	122(15)	74(11)	103(9)	-13(8)	15(9)	14(10)
C2S_13	77(10)	98(13)	102(9)	-20(8)	5(8)	15(9)
C3S_13	92(12)	110(13)	105(12)	-19(9)	8(9)	3(9)
C4S_13	98(13)	106(13)	85(8)	-3(8)	18(8)	-8(10)
C5S_13	102(12)	94(13)	82(8)	2(7)	9(8)	8(10)
C6S_13	133(15)	86(11)	96(11)	-18(8)	35(10)	-4(11)
C1S_14	103(9)	52(9)	106(10)	-9(8)	-1(7)	-10(7)
C2S_14	84(9)	86(11)	98(8)	12(7)	14(7)	-18(8)
C3S_14	61(7)	79(10)	84(8)	23(6)	12(6)	-15(6)
C4S_14	71(5)	73(10)	69(6)	20(6)	15(4)	-15(5)
C5S_14	77(4)	77(5)	79(4)	3(3)	6(2)	-1(3)
C6S_14	96(10)	47(7)	73(5)	19(5)	7(6)	-14(6)
Br1_15	51.1(7)	40.1(8)	66.0(8)	-3.9(6)	-5.1(6)	-8.1(6)
Br2_15	67.1(9)	41.5(9)	64.9(8)	-20.8(7)	-4.5(7)	-0.2(7)
O1_15	54(5)	34(4)	44(4)	-12(3)	17(4)	-19(4)
O2_15	47(4)	41(5)	39(4)	-7(3)	15(3)	-8(4)
O3_15	53(5)	29(4)	65(5)	3(4)	-1(4)	7(4)
O4_15	44(4)	43(5)	56(5)	-12(4)	-2(3)	5(4)
N1_15	43(4)	27(4)	36(4)	-2(3)	6(3)	-3(3)
C1_15	45(4)	26(4)	42(5)	-2(4)	8(4)	-1(3)
C2_15	43(4)	30(5)	34(5)	-5(4)	9(4)	-1(4)
C3_15	42(4)	41(7)	54(6)	-12(5)	7(4)	5(4)
C4_15	43(6)	40(6)	54(7)	-2(5)	-10(5)	9(4)
C5_15	38(4)	33(5)	45(6)	-3(5)	7(4)	-9(4)
C6_15	43(4)	22(5)	33(5)	3(4)	1(4)	-5(3)
C7_15	52(5)	33(6)	42(5)	-1(5)	7(4)	1(4)
C8_15	51(5)	28(6)	60(7)	-12(5)	-2(5)	0(4)
C9_15	54(6)	24(5)	53(6)	-5(4)	-9(5)	3(4)
C10_15	44(4)	26(5)	34(5)	-1(4)	4(4)	0(4)
C11_15	43(4)	29(5)	39(5)	-6(4)	2(4)	-1(4)
C12_15	44(4)	31(5)	39(5)	-6(4)	0(4)	0(4)
C13_15	49(6)	28(5)	42(5)	-8(4)	16(4)	-7(4)
C14_15	36(5)	31(4)	32(4)	-11(3)	2(3)	-9(4)
C15_15	57(6)	49(7)	32(5)	-9(4)	16(4)	-17(5)
C16_15	71(8)	92(13)	59(8)	27(8)	15(6)	-12(7)
C17_15	42(6)	61(9)	77(8)	-13(7)	17(6)	-20(6)
C18_15	108(13)	62(9)	65(8)	-21(7)	52(9)	-17(8)
Br1_16	58.0(8)	33.9(8)	86.9(10)	-8.2(7)	9.5(7)	9.7(7)
Br2_16	76.3(10)	43.5(9)	85.6(10)	20.5(8)	16.0(8)	19.5(8)
O1_16	43(4)	23(4)	43(4)	7(3)	3(3)	-2(3)
O2_16	37(3)	18(4)	32(3)	11(3)	-6(3)	4(3)
O3_16	49(4)	31(5)	40(3)	-8(3)	4(3)	3(4)
O4_16	71(6)	47(5)	38(3)	0(3)	16(4)	4(5)
N1_16	42(5)	38(4)	35(3)	-2(3)	3(3)	-1(4)
C1_16	42(5)	31(5)	41(4)	-5(3)	4(4)	-3(4)
C2_16	43(6)	28(5)	46(4)	0(3)	9(4)	-8(4)
C3_16	46(6)	33(5)	43(4)	0(4)	10(4)	5(5)
C4_16	65(7)	31(6)	49(5)	-17(4)	3(5)	5(5)
C5_16	42(6)	40(7)	67(5)	3(4)	14(5)	3(5)
C6_16	37(5)	21(5)	68(5)	6(4)	10(4)	-7(4)
C7_16	61(8)	36(7)	69(5)	11(5)	11(5)	7(6)

Atom	U_{11}	U_{22}	U_{33}	U_{23}	U_{13}	U_{12}
C8_16	98(11)	45(8)	63(6)	17(5)	11(7)	22(7)
C9_16	91(10)	48(7)	54(6)	15(5)	25(6)	19(7)
C10_16	55(7)	41(6)	44(4)	4(4)	20(4)	0(5)
C11_16	42(5)	25(5)	47(4)	3(4)	11(4)	-1(4)
C12_16	45(6)	43(6)	36(3)	1(3)	9(4)	3(5)
C13_16	42(4)	38(5)	40(5)	-2(4)	11(4)	0(4)
C14_16	43(4)	16(4)	40(5)	-2(3)	6(4)	-4(3)
C15_16	40(5)	45(6)	45(5)	-3(5)	9(4)	-4(4)
C16_16	55(7)	57(9)	54(6)	1(6)	24(5)	-19(7)
C17_16	60(8)	46(6)	57(7)	1(6)	17(6)	-3(6)
C18_16	56(7)	38(8)	75(8)	-3(7)	-10(6)	-4(6)
Br1_17	68.8(10)	42.5(10)	107.8(13)	-0.1(9)	23.4(9)	-15.5(8)
Br2_17	75.2(11)	62.4(12)	115.5(14)	43.5(11)	-2.5(10)	-20.8(9)
O1_17	39(4)	33(4)	50(4)	5(3)	19(3)	-2(3)
O2_17	47(4)	22(4)	48(4)	3(3)	22(3)	-6(3)
O3_17	73(6)	41(5)	53(4)	10(4)	9(4)	-12(5)
O4_17	72(6)	70(7)	47(4)	9(4)	16(4)	-15(5)
N1_17	49(5)	41(5)	44(4)	9(4)	12(3)	2(4)
C1_17	38(5)	44(6)	49(4)	6(4)	11(4)	2(4)
C2_17	49(6)	42(6)	54(5)	4(4)	12(4)	-3(5)
C3_17	41(6)	40(6)	54(5)	-1(5)	17(4)	7(4)
C4_17	47(7)	48(6)	68(6)	-6(5)	10(5)	1(5)
C5_17	29(5)	43(7)	82(6)	-2(5)	13(4)	3(5)
C6_17	44(6)	40(6)	66(5)	17(4)	18(4)	11(4)
C7_17	57(8)	72(10)	72(6)	28(6)	9(5)	-19(7)
C8_17	71(9)	64(8)	73(7)	32(7)	-4(6)	-5(7)
C9_17	88(10)	65(8)	52(6)	26(6)	8(6)	-8(7)
C10_17	57(6)	42(6)	49(4)	14(4)	19(4)	7(5)
C11_17	42(6)	45(6)	53(4)	4(4)	23(4)	8(4)
C12_17	53(6)	44(6)	49(4)	14(4)	13(4)	9(5)
C13_17	59(6)	40(5)	42(5)	0(4)	22(5)	3(4)
C14_17	46(5)	19(4)	38(5)	-8(3)	14(4)	-9(3)
C15_17	58(6)	48(6)	49(5)	-10(5)	23(4)	6(5)
C16_17	69(8)	62(11)	74(7)	-4(7)	8(6)	20(8)
C17_17	84(10)	52(7)	54(7)	-5(6)	20(7)	-7(7)
C18_17	66(9)	85(12)	71(8)	9(8)	41(7)	19(8)
Br1_18	70.8(11)	78.8(14)	163(2)	-50.0(14)	60.3(13)	-13.4(10)
Br2_18	74.0(10)	48.0(10)	92.6(11)	-26.6(9)	29.3(9)	-2.9(8)
O1_18	46(4)	37(4)	38(4)	4(3)	5(3)	-3(3)
O2_18	39(4)	32(5)	48(4)	2(3)	6(3)	7(3)
O3_18	45(4)	28(4)	64(5)	-4(4)	6(4)	-6(3)
O4_18	42(4)	36(5)	55(4)	-8(4)	22(3)	-4(4)
N1_18	35(4)	37(4)	42(5)	1(4)	13(3)	-1(3)
C1_18	48(5)	26(4)	33(5)	-1(4)	16(4)	-2(4)
C2_18	51(6)	32(4)	40(5)	-1(4)	20(4)	5(4)
C3_18	60(7)	64(8)	67(8)	-2(7)	34(6)	-8(6)
C4_18	68(9)	68(7)	78(9)	-18(7)	38(8)	-14(6)
C5_18	50(6)	61(7)	55(7)	-5(6)	10(5)	0(5)
C6_18	43(5)	39(5)	48(6)	-13(5)	0(4)	3(4)
C7_18	53(6)	42(6)	40(6)	-5(5)	-10(4)	-4(5)
C8_18	57(6)	28(6)	53(6)	-15(5)	0(5)	-3(5)
C9_18	41(5)	32(5)	45(6)	-4(4)	7(4)	-4(4)
C10_18	48(5)	31(4)	37(5)	-9(4)	8(4)	-3(4)
C11_18	45(5)	31(4)	35(5)	1(4)	3(4)	3(4)
C12_18	40(5)	35(4)	35(5)	-2(4)	15(4)	-1(4)
C13_18	40(5)	33(5)	31(4)	3(4)	8(3)	-1(4)
C14_18	38(5)	36(4)	36(5)	2(4)	1(3)	-1(4)
C15_18	51(6)	39(6)	43(5)	6(4)	4(4)	12(4)

Atom	U_{11}	U_{22}	U_{33}	U_{23}	U_{13}	U_{12}
C16_18	73(9)	62(10)	73(9)	26(7)	18(7)	10(8)
C17_18	62(8)	72(10)	58(7)	-8(7)	-6(6)	35(7)
C18_18	69(9)	47(8)	88(10)	4(7)	-23(7)	8(6)
C1S_19	93(12)	75(11)	83(8)	-8(7)	-5(7)	0(9)
C2S_19	114(14)	129(16)	88(8)	-25(8)	-7(8)	23(12)
C3S_19	71(9)	89(11)	88(9)	-11(7)	9(7)	-17(7)
C4S_19	77(10)	85(10)	88(7)	-4(7)	20(7)	-18(8)
C5S_19	84(11)	92(13)	87(7)	-4(7)	20(7)	6(9)
C6S_19	109(12)	83(11)	89(9)	-15(8)	0(8)	6(9)
C1S_20	113(13)	65(9)	220(30)	48(12)	29(16)	17(8)
C2S_20	112(9)	63(9)	138(17)	46(11)	29(10)	15(8)
C3S_20	105(8)	89(8)	75(10)	-11(8)	4(8)	4(6)
C4S_20	99(6)	98(6)	98(6)	-3(3)	3(3)	1(3)
C5S_20	115(10)	94(8)	81(11)	-13(7)	6(9)	3(7)
C6S_20	121(10)	60(10)	136(17)	12(11)	25(11)	5(7)
C1S_21	116(15)	113(14)	78(8)	-27(9)	-10(9)	-28(11)
C2S_21	134(18)	121(15)	115(13)	-33(10)	-4(11)	-42(13)
C3S_21	121(16)	86(15)	125(11)	-23(9)	8(10)	2(12)
C4S_21	82(12)	115(15)	126(11)	-30(10)	1(10)	16(10)
C5S_21	123(16)	128(15)	87(11)	-26(10)	-11(10)	-1(11)
C6S_21	108(14)	127(16)	78(8)	-30(8)	-22(8)	-4(12)
C1S_22	118(10)	87(11)	106(13)	20(10)	32(9)	16(8)
C2S_22	113(12)	94(11)	121(16)	0(10)	22(12)	16(8)
C3S_22	125(10)	106(12)	87(11)	-7(11)	33(10)	6(9)
C4S_22	127(10)	111(13)	60(9)	4(9)	24(9)	-5(9)
C5S_22	132(13)	120(11)	66(10)	16(9)	34(10)	5(8)
C6S_22	119(10)	95(11)	74(10)	45(9)	30(9)	18(9)
N2_23	63(6)	21(5)	50(5)	6(4)	1(5)	-4(4)
C19_23	119(12)	33(6)	67(9)	7(6)	-13(8)	18(7)
C20_23	200(30)	48(11)	190(30)	31(13)	-30(20)	45(14)
N2_24	63(6)	37(5)	44(5)	3(4)	11(5)	1(4)
C19_24	67(7)	32(5)	39(6)	-7(4)	-5(5)	-3(5)
C20_24	112(13)	47(7)	63(8)	-1(6)	-6(8)	20(7)
N2_25	63(7)	38(5)	55(6)	3(4)	1(5)	-19(5)
C19_25	71(9)	36(5)	59(7)	0(5)	22(6)	-10(5)
C20_25	180(20)	42(7)	97(13)	11(8)	50(14)	-14(9)
N2_26	67(6)	24(5)	52(5)	10(4)	15(5)	4(4)
C19_26	79(9)	37(5)	70(8)	25(5)	-1(7)	-8(6)
C20_26	105(15)	49(9)	180(20)	63(12)	30(15)	-5(9)
Br1_27	177(5)	229(7)	169(5)	-28(4)	-82(4)	103(5)
Br2_27	113(3)	162(5)	180(4)	44(4)	10(3)	81(3)
O1_27	44(2)	40(3)	45(3)	3(2)	6(2)	-3(2)
O2_27	46(3)	43(3)	48(3)	4(2)	7(2)	0(2)
O3_27	60(5)	53(4)	61(5)	-3(4)	9(4)	4(3)
O4_27	94(12)	77(10)	69(9)	-7(7)	9(7)	16(10)
N1_27	49(3)	49(3)	50(3)	0(2)	5(2)	0(2)
C1_27	51(3)	50(4)	51(3)	0(2)	3(2)	2(2)
C2_27	50(4)	49(4)	52(4)	0(3)	1(2)	0(3)
C3_27	39(9)	83(16)	57(7)	-12(8)	-3(7)	-15(9)
C4_27	87(13)	111(16)	86(11)	-30(11)	-33(11)	21(12)
C5_27	65(11)	74(13)	75(7)	7(7)	3(7)	-6(9)
C6_27	73(4)	73(4)	75(4)	1.7(19)	2.4(19)	1.7(19)
C7_27	66(7)	85(8)	104(9)	3(7)	6(7)	21(7)
C8_27	66(7)	85(8)	104(9)	3(7)	6(7)	21(7)
C9_27	69(11)	61(11)	90(11)	-15(8)	6(8)	20(9)
C10_27	64(11)	39(11)	67(9)	9(8)	24(8)	7(8)
C11_27	61(9)	34(9)	68(8)	11(7)	21(6)	-4(7)
C12_27	57(10)	48(9)	58(8)	11(7)	28(6)	1(7)

Atom	U_{11}	U_{22}	U_{33}	U_{23}	U_{13}	U_{12}
C13_27	47(6)	51(9)	56(9)	2(7)	8(5)	0(7)
C14_27	45(3)	44(3)	44(3)	2(2)	5(2)	0(2)
C15_27	51(4)	50(4)	51(4)	0(3)	4(2)	-1(3)
C16_27	104(13)	47(7)	55(10)	-1(6)	26(9)	-31(8)
C17_27	64(5)	65(6)	63(5)	-2(5)	-3(4)	0(5)
C18_27	58(9)	90(20)	46(12)	9(12)	9(9)	-1(11)
N2_28	43(5)	36(5)	54(5)	4(4)	4(4)	11(4)
C19_28	73(9)	34(5)	59(7)	-4(5)	-21(7)	13(5)
C20_28	240(30)	55(9)	84(12)	-16(8)	-70(16)	70(13)
N2_29	32(4)	24(4)	56(5)	11(4)	8(4)	2(3)
C19_29	36(5)	35(5)	78(8)	25(5)	14(5)	7(4)
C20_29	75(10)	50(8)	139(16)	50(9)	22(11)	24(7)
N2_30	46(5)	17(4)	54(5)	7(4)	6(4)	-9(3)
C19_30	52(7)	23(4)	71(8)	15(5)	2(6)	-14(4)
C20_30	88(11)	34(7)	119(13)	24(8)	2(10)	-36(7)
N2_31	101(9)	91(12)	92(10)	6(9)	3(8)	-1(8)
C19_31	95(9)	82(12)	49(7)	6(8)	11(7)	6(7)
C20_31	97(10)	75(12)	79(10)	-6(9)	11(8)	9(8)
N2_32	59(6)	21(4)	57(6)	7(4)	21(5)	8(4)
C19_32	79(9)	22(5)	73(8)	8(5)	34(7)	5(5)
C20_32	260(40)	52(10)	170(20)	40(13)	90(30)	-31(15)
N2_33	159(19)	290(30)	99(12)	-53(17)	-35(12)	140(20)
C19_33	110(14)	121(17)	60(9)	19(10)	-6(8)	54(12)
C20_33	140(20)	140(20)	190(30)	-20(20)	50(20)	41(19)

Compound 13

Submitted by: **Duc Ly, Davies Group**

Solved by: **Mackenzie Young**

R_1=3.89%

Crystal Data and Experimental

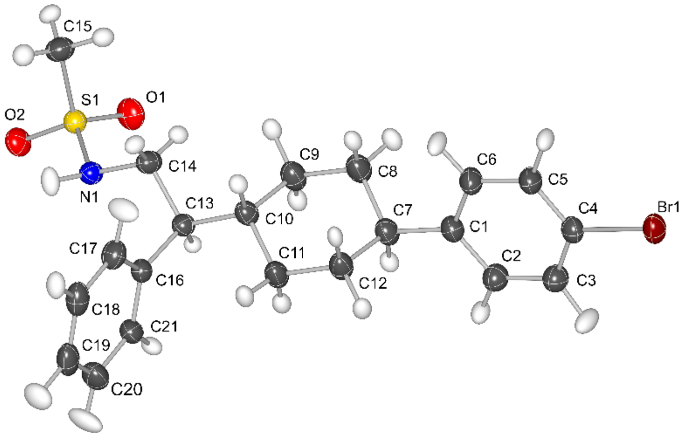

Experimental. Single colorless needle-shaped crystals of COMPOUND 13were recrystallised from a mixture of chloroform and Br-butane by slow evaporation. A suiTable Scrystal with dimensions 0.94 × 0.06 × 0.04 mm³ was selected and mounted on a loop with paratone on a XtaLAB AFC11 (RCD3): quarter-chi single diffractometer. The crystal was kept at a steady *T* = 173.02(19) K during data collection. The structure was solved with the ShelXT (Sheldrick, 2015) solution program using dual methods and by using Olex2 1.5-alpha (Dolomanov et al., 2009) as the graphical interface. The model was refined with olex2.refine 1.5-alpha (Bourhis et al., 2015) using full matrix least squares minimisation on F^2.

Crystal Data. $C_{21}H_{26}BrNO_2S$, M_r = 436.416, monoclinic, $P2_1$ (No. 4), a = 13.5729(4) Å, b = 5.4942(1) Å, c = 14.9041(4) Å, β = 110.576(3)°, $\alpha = \gamma$ = 90°, V = 1040.53(5) Å³, T = 173.02(19) K, Z = 2, Z' = 1, μ(Cu K_α) = 3.740, 16932 reflections measured, 3716 unique (R_{int} = 0.0397) which were used in all calculations. The final wR_2 was 0.1010 (all data) and R_1 was 0.0389 (I≥2 σ(I)).

**Compound**	**13**
Formula	$C_{21}H_{26}BrNO_2S$
$D_{calc.}$/ g cm^{-3}	1.393
μ/mm^{-1}	3.740
Formula Weight	436.416
Color	colorless
Shape	needle-shaped
Size/mm³	0.94×0.06×0.04
T/K	173.02(19)
Crystal System	monoclinic
Flack Parameter	-0.068(9)
Hooft Parameter	-0.068(9)
Space Group	$P2_1$
a/Å	13.5729(4)
b/Å	5.4942(1)
c/Å	14.9041(4)
α/°	90
β/°	110.576(3)
γ/°	90
V/Å³	1040.53(5)
Z	2
Z'	1
Wavelength/Å	1.54184
Radiation type	Cu K_α
Θ_{min}/°	3.17
Θ_{max}/°	70.81
Measured Refl's.	16932
Indep't Refl's	3716
Refl's I≥2 σ(I)	3396
R_{int}	0.0397
Parameters	495
Restraints	406
Largest Peak	0.2385
Deepest Hole	-0.2583
GooF	1.0505
wR_2 (all data)	0.1010
wR_2	0.0987
R_1 (all data)	0.0428
R_1	0.0389

Structure Quality Indicators

Reflections:

d min (CuKα) 2Θ=141.6°	I/σ(I)	Rint m=4.55	Full 135.4° 97% to 141.6°
0.82	31.4	3.97%	98.9

Refinement:

Shift	Max Peak	Min Peak	GooF	Hooft
-0.001	0.2	-0.3	1.050	-.068(9)

A colourless needle-shaped-shaped crystal with dimensions 0.94 × 0.06 × 0.04 mm^3 was mounted on a loop with paratone. Data were collected using a XtaLAB AFC11 (RCD3): quarter-chi single diffractometer operating at T = 173.02(19) K.

Data were measured using ω scans with Cu K_{α} radiation. The diffraction pattern was indexed and the total number of runs and images was based on the strategy calculation from the program CrysAlisPro system (CCD 43.92a 64-bit (release 05-10-2023)). The maximum resolution that was achieved was Θ = 70.81° (0.82 Å).

The unit cell was refined using CrysAlisPro 1.171.43.92a (Rigaku OD, 2023) on 7323 reflections, 43% of the observed reflections.

Data reduction, scaling and absorption corrections were performed using CrysAlisPro 1.171.43.92a (Rigaku OD, 2023). The final completeness is 99.86 % out to 70.81° in Θ. A numerical absorption correction based on gaussian integration over a multifaceted crystal model was performed using CrysAlisPro 1.171.42.74a (Rigaku Oxford Diffraction, 2022). An empirical absorption correction using spherical harmonics, implemented in SCALE3 ABSPACK scaling algorithm was also applied. The absorption coefficient μ of this material is 3.740 mm^{-1} at this wavelength (λ = 1.54184Å) and the minimum and maximum transmissions are 0.338 and 1.000.

The structure was solved and the space group $P2_1$ (# 4) determined by the ShelXT (Sheldrick, 2015) structure solution program using using dual methods and refined by full matrix least squares minimisation on $\boldsymbol{F}^2$ using version of olex2.refine 1.5-alpha (Bourhis et al., 2015). Hydrogen atom positions were located from the electron densities and freely refined using Hirshfeld scattering factors Refinement was by using NoSpherA2, an implementation of non-spherical atom-form-factors (F. Kleemiss, H. Puschmann, O. Dolomanov, S.Grabowsky - https://doi.org/10.1039/D0SC05526C – 2020). NoSpherA2 implementation of HAR makes use of tailor-made aspherical atomic form factors calculated from a Hirshfeld-partitioned electron density (ED) not from spherical-atom form factors. The ED was calculated from a Gaussian basis set single determinant SCF wavefunction from DFT using selected functionals for a fragment of this crystal. This fragment was embedded in an electrostatic crystal field by employing cluster charges. The following options were used: SOFTWARE: ORCA PARTITIONING: NoSpherA2 INT ACCURACY: Normal METHOD: PBE BASIS SET: def2-SVP CHARGE: 0 MULTIPLICITY: 1 SOLVATION: Chloroform DATE: 2024-04-10_16-01-17

There is a single formula unit in the asymmetric unit, which is represented by the reported sum formula. In other words: Z is 2 and Z' is 1.

The Flack parameter was refined to -0.068(9). Determination of absolute structure using Bayesian statistics on Bijvoet differences using the Olex2 results in -0.068(9). The chiral atom was identified in this structure as being C13. It's important to note that the Flack parameter is a crucial parameter for determining the chirality of the crystal under study. Ideally, its value should be close to 0. A value of 1 indicates that the stereochemistry is incorrect and the model should be inverted. A value of 0.5 signifies that the crystal is a racemic mixture of both enantiomers.

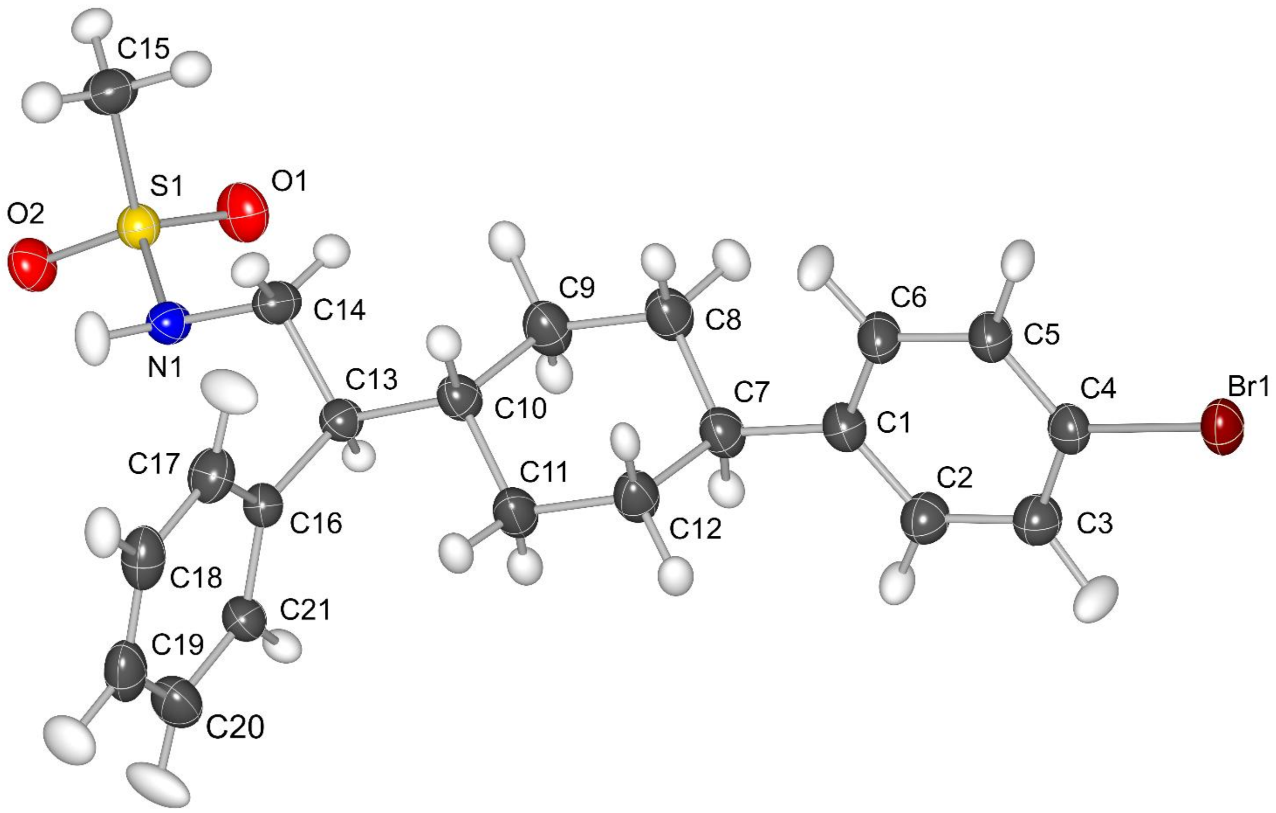

Figure S323. The independent molecule in the crystal structure. The crystal structure is chiral and the chiral atom was identified in this structure as being C13(S).

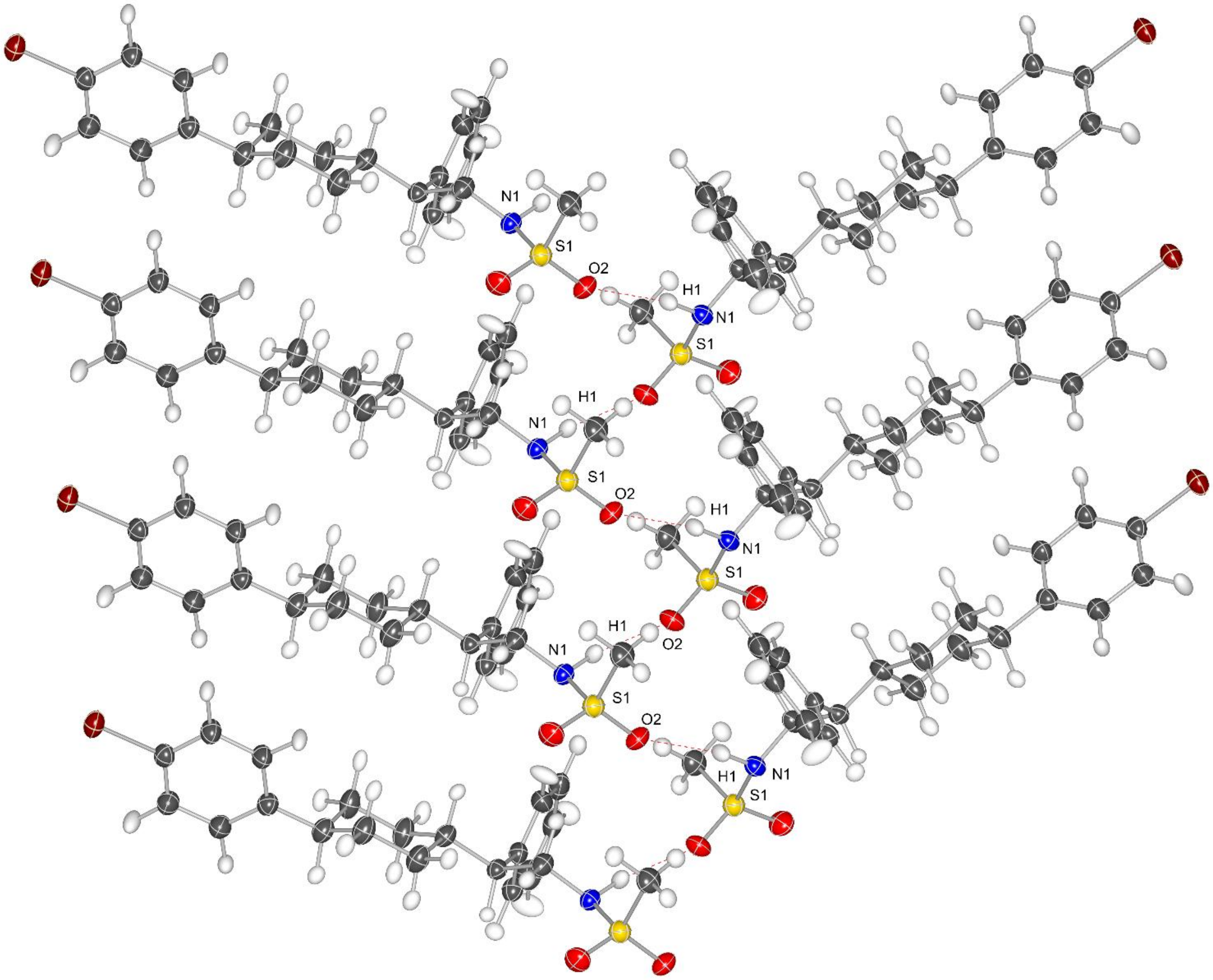

Figure S324. Hydrogen bonding in the crystal structure occurs between pairs of molecules and forms infinite one-dimensional chains along the crystallographic axis, b. It is responsible for the needle-like morphology of the crystals.

Data Plots: Diffraction Data

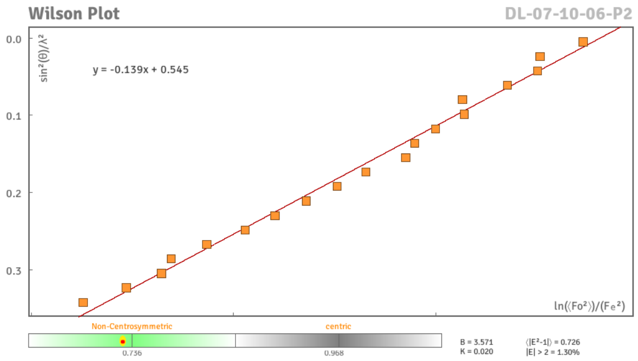

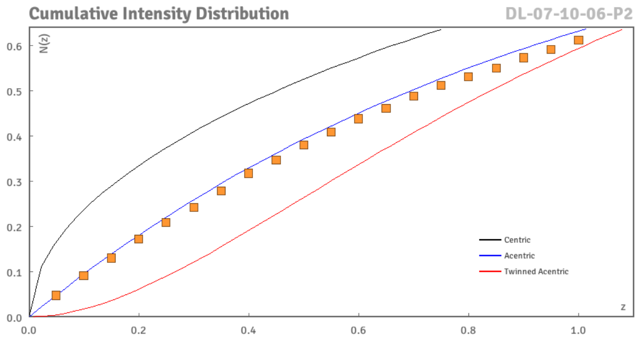

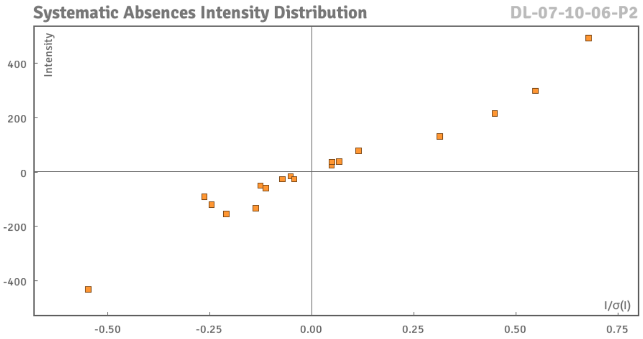

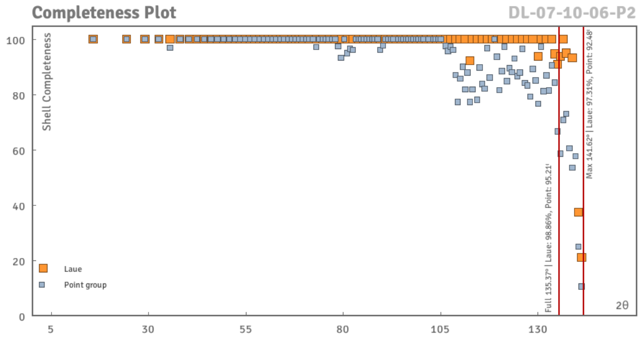

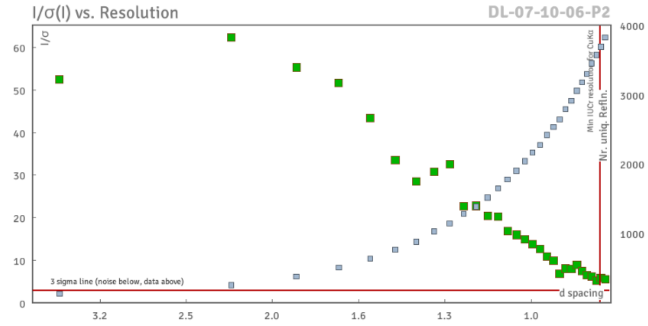

Data Plots: Refinement and Data

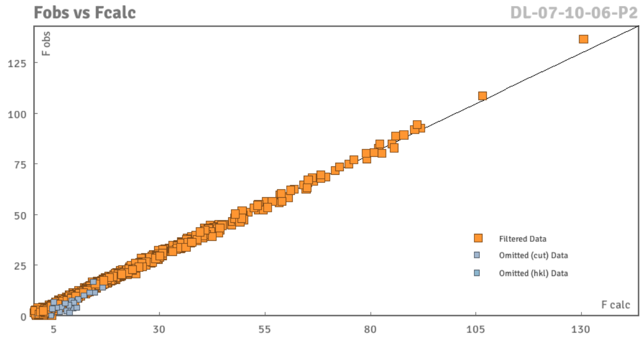

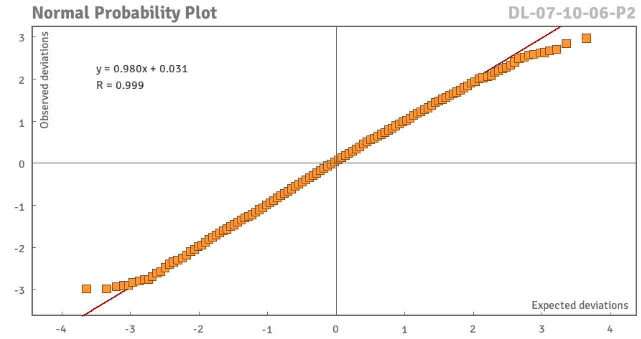

Reflection Statistics

Total reflections (after filtering)	16892	Unique reflections	3716
Completeness	0.925	Mean I/σ	22.43
hkl_{max} collected	(16, 6, 18)	hkl_{min} collected	(-16, -6, -18)
hkl_{max} used	(15, 6, 18)	hkl_{min} used	(-16, -6, 0)
Lim d_{max} collected	100.0	Lim d_{min} collected	0.77
d_{max} used	13.95	d_{min} used	0.82
Friedel pairs	2173	Friedel pairs merged	0
Inconsistent equivalents	3	R_{int}	0.0397
R_{sigma}	0.0318	Intensity transformed	0
Omitted reflections	59	Omitted by user (OMIT hkl)	1
Multiplicity	(1563, 1206, 1094, 705, 426, 272, 163, 115, 61, 26, 12, 7, 2)	Maximum multiplicity	17
Removed systematic absences	19	Filtered off (Shel/OMIT)	0

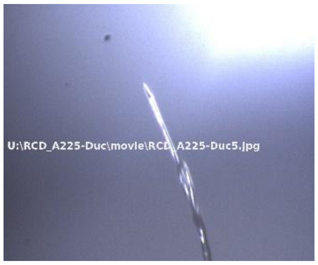

Table S28. Fractional Atomic Coordinates (×10⁴) and Equivalent Isotropic Displacement Parameters (Å²×10³) for **Compound 13**. U_{eq} is defined as 1/3 of the trace of the orthogonalised U_{ij}.

Atom	x	y	z	U_{eq}
Br1	8620.4(7)	11683.3(5)	2534.6(6)	46.9(5)
S1	4589.7(6)	-72.5(15)	8561.4(6)	33.6(2)
O1	4435(2)	-1101(5)	7639.1(19)	50.3(7)
O2	4673(2)	-1698(4)	9334.5(19)	45.2(6)
N1	5668.8(19)	1495(6)	8875(2)	34.5(6)
C1	7825(3)	7310(6)	4886(3)	39.5(8)
C2	8644(3)	6583(8)	4580(3)	44.8(8)
C3	8895(3)	7887(7)	3888(3)	45.0(9)
C4	8322(3)	9967(7)	3504(2)	39.1(8)
C5	7507(3)	10761(7)	3796(3)	44.0(9)
C6	7270(3)	9405(6)	4484(3)	43.0(9)
C7	7617(3)	5888(7)	5666(3)	42.8(8)
C8	6468(3)	5672(8)	5568(3)	50.4(10)
C9	6326(3)	4145(8)	6371(3)	49.1(9)
C10	6985(3)	5045(7)	7376(3)	37.6(7)
C11	8121(3)	5397(8)	7453(3)	49.8(10)
C12	8243(3)	6938(9)	6652(3)	48.3(9)
C13	6900(3)	3319(6)	8166(2)	33.4(7)
C14	5770(3)	3271(7)	8173(3)	39.9(8)
C15	3565(3)	1998(8)	8442(3)	48.0(9)
C16	7682(3)	3942(6)	9137(2)	33.3(7)
C17	7602(3)	6074(6)	9610(3)	44.1(8)
C18	8366(3)	6679(9)	10495(3)	51.4(8)
C19	9209(3)	5111(8)	10915(3)	53.5(9)
C20	9298(3)	2974(8)	10452(3)	53.4(10)
C21	8531(3)	2397(6)	9571(3)	39.3(8)

Table S29. Anisotropic Displacement Parameters (×10⁴) for **Compound 13.** The anisotropic displacement factor exponent takes the form: $-2\pi^2[h^2a^{*2} \times U_{11} + ... +2hka^* \times b^* \times U_{12}]$

Atom	U_{11}	U_{22}	U_{33}	U_{23}	U_{13}	U_{12}
Br1	50.3(7)	56.0(8)	37.2(7)	-9.8(6)	18.8(5)	3.5(6)
S1	34.9(4)	32.8(4)	33.2(5)	-0.9(3)	12.3(3)	0.5(3)
O1	59.3(16)	49.0(16)	39.5(14)	-8.2(13)	13.6(12)	-11.4(13)
O2	50.6(15)	39.9(14)	44.9(15)	-2.0(11)	16.6(12)	11.5(12)
N1	31.5(12)	35.0(14)	36.6(14)	0.0(11)	11.6(10)	3.6(12)
H1	90(30)	50(13)	43(4)	-19(8)	28(5)	-5(3)
C1	43.8(15)	41.0(17)	33.6(16)	-0.3(10)	13.5(10)	3.1(11)
C2	47.2(16)	43.9(18)	46.3(18)	0.7(13)	20.1(11)	3.6(14)
H2	68(16)	54(9)	61(18)	15(6)	39(8)	16(6)
C3	46.0(18)	45.9(18)	45.7(19)	-0.3(12)	19.2(12)	3.8(12)
H3	84(14)	76(19)	110(20)	33(8)	67(9)	45(10)

Atom	U_{11}	U_{22}	U_{33}	U_{23}	U_{13}	U_{12}
C4	43.8(17)	43.8(17)	29.7(16)	-2.8(11)	12.7(11)	-1.9(12)
C5	47.6(18)	49.7(19)	37.5(18)	4.6(12)	18.4(12)	7.7(13)
H5	68(17)	63(11)	60(20)	21(7)	41(9)	25(8)
C6	50.4(19)	45.0(19)	37.8(18)	6.7(12)	20.6(12)	6.8(12)
H6	84(14)	70(15)	80(18)	35(7)	58(8)	39(8)
C7	43.8(16)	47.2(19)	35.4(16)	-4.3(11)	11.3(9)	6.7(11)
H7	49(9)	49(3)	42(10)	-3(2)	13(4)	7(2)
C8	45.9(19)	63(2)	39.1(19)	-8.4(13)	10.8(11)	8.6(15)
H8a	42(9)	62(4)	38(9)	-10(2)	10(4)	9(2)
H8b	54(8)	73(11)	41(4)	-11(4)	9(2)	5(3)
C9	48(2)	58(2)	37.7(17)	-12.3(14)	9.8(11)	3.8(12)
H9a	48(3)	64(11)	41(11)	-13(2)	11(2)	3(4)
H9b	55(9)	58(3)	43(10)	-11(2)	14(4)	4(2)
C10	36.6(15)	39.2(17)	34.6(16)	-3.8(11)	9.4(9)	5.9(11)
H10	43(9)	40(4)	42(10)	-3(2)	14(4)	7(2)
C11	39.2(18)	72(3)	35.6(18)	-8.0(12)	10.3(11)	10.0(16)
H11a	46(8)	81(10)	38(4)	-12(4)	11(2)	6(3)
H11b	44(9)	73(4)	43(10)	-6(2)	14(4)	10(2)
C12	40.0(17)	67(2)	36.6(16)	-12.8(15)	11.9(10)	4.8(13)
H12a	42(3)	75(11)	43(10)	-14(2)	14(2)	4(4)
H12b	43(9)	67(4)	29(10)	-12(2)	11(4)	6(2)
C13	32.5(14)	33.2(16)	34.5(15)	1.2(9)	11.8(9)	3.2(10)
H13	35(9)	34(3)	37(9)	1.6(18)	12(4)	2.7(19)
C14	33.2(15)	41.2(18)	46(2)	2.7(11)	14.5(11)	9.0(14)
H14a	38(7)	44(10)	46(4)	0(3)	13.7(19)	9(2)
H14b	40(10)	42(4)	56(10)	2(2)	22(4)	7(2)
C15	36.9(18)	53(3)	55(2)	3.3(18)	17.0(18)	3(2)
H15a	39(6)	58(16)	59(10)	1(5)	21(3)	-2(5)
H15b	55(16)	55(13)	57(5)	0(6)	22(4)	2(4)
H15c	43(15)	62(11)	59(6)	2(6)	18(4)	8(4)
C16	34.4(14)	36.9(15)	29.8(14)	0.9(9)	12.9(10)	3.1(9)
C17	52.1(18)	41.5(19)	44.3(18)	-2.3(12)	23.9(13)	-5.0(11)
H17	85(17)	66(13)	66(14)	26(7)	0(7)	-25(7)
C18	62.7(18)	56(2)	40.9(16)	-17.6(14)	25.6(12)	-4.5(14)
H18	78(16)	60(10)	46(12)	-14(6)	28(7)	-7(5)
C19	56.6(19)	69(2)	39.0(19)	-17.2(13)	22.5(14)	2.4(13)
H19	85(17)	92(18)	57(12)	2(8)	1(7)	-16(7)
C20	44.6(19)	71(2)	38.8(18)	-4.3(14)	7.1(13)	3.9(13)
H20	90(20)	94(13)	65(15)	28(8)	-26(9)	-19(7)
C21	37.8(15)	44.8(19)	34.9(16)	4.0(11)	12.1(11)	7.6(11)
H21	44(14)	50(9)	41(12)	10(6)	7(7)	3(5)

Compound 13a

Submitted by: **Duc Ly**

Solved by: **John Bacsa**

R_1=2.56%

Crystal Data and Experimental

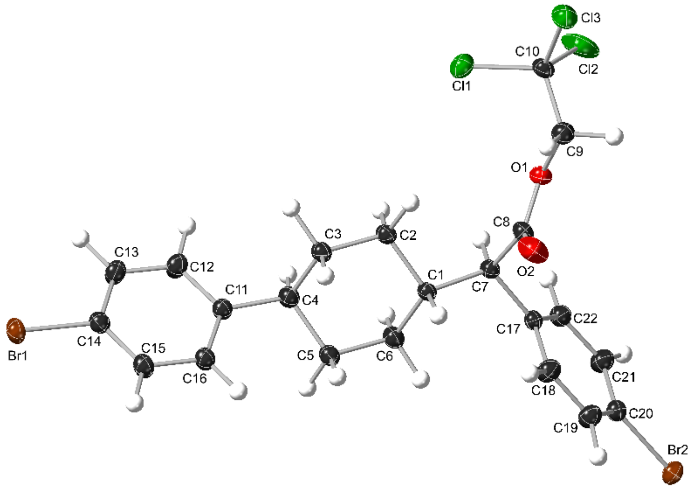

Experimental. Single colorless plate-shaped crystals of **Compound 13a** were chosen from the sample as supplied. A suiTable Scrystal with dimensions $0.31 \times 0.16 \times 0.09$ mm^3 was selected and mounted on a loop with paratone on a XtaLAB Synergy, Dualflex, HyPix diffractometer. The crystal was kept at a steady T = 100.00(10) K during data collection. The structure was solved with the ShelXT (Sheldrick, 2015) solution program using dual methods and by using Olex2 1.5-alpha (Dolomanov et al., 2009) as the graphical interface. The model was refined with olex2.refine 1.5-alpha (Bourhis et al., 2015) using full matrix least squares minimisation on $\boldsymbol{F^2}$.

Crystal Data. $C_{22}H_{21}Br_2Cl_3O_2$, M_r = 583.575, monoclinic, $P2_1$ (No. 4), a = 10.36803(9) Å, b = 5.59520(5) Å, c = 19.93580(16) Å, β = 93.9587(7)°, $\alpha = \gamma$ = 90°, V = 1153.740(17) Å^3, T = 100.00(10) K, Z = 2, Z' = 1, μ(Cu K_α) = 7.787, 14600 reflections measured, 4475 unique (R_{int} = 0.0283) which were used in all calculations. The final wR_2 was 0.0661 (all data) and R_1 was 0.0256 (I≥2 σ(I)).

**Compound**	**13a**
Formula	$C_{22}H_{21}Br_2Cl_3O_2$
$D_{calc.}$/ g cm^{-3}	1.680
μ/mm^{-1}	7.787
Formula Weight	583.575
Color	colorless
Shape	plate-shaped
Size/mm^3	0.31×0.16×0.09
T/K	100.00(10)
Crystal System	monoclinic
Flack Parameter	-0.006(6)
Hooft Parameter	-0.006(6)
Space Group	$P2_1$
a/Å	10.36803(9)
b/Å	5.59520(5)
c/Å	19.93580(16)
α/°	90
β/°	93.9587(7)
γ/°	90
V/Å^3	1153.740(17)
Z	2
Z'	1
Wavelength/Å	1.54184
Radiation type	Cu K_α
Θ_{min}/°	2.22
Θ_{max}/°	79.70
Measured Refl's.	14600
Indep't Refl's	4475
Refl's I≥2 σ(I)	4448
R_{int}	0.0283
Parameters	553
Restraints	388
Largest Peak	0.3081
Deepest Hole	-0.1594
GooF	1.0731
wR_2 (all data)	0.0661
wR_2	0.0659
R_1 (all data)	0.0258
R_1	0.0256

Structure Quality Indicators

**Reflections:**	d min (CuKα) 2Θ=159.4° 0.78	I/σ(I) 41.2	Rint m=3.26 2.83%	Full 135.4° 93% to 159.4° 100	
**Refinement:**	Shift -0.001	Max Peak 0.3	Min Peak -0.2	GooF 1.073	Hooft -.006(6)

A colourless plate-shaped-shaped crystal with dimensions 0.31 × 0.16 × 0.09 mm^3 was mounted on a loop with paratone. Data were collected using a XtaLAB Synergy, Dualflex, HyPix diffractometer operating at T = 100.00(10) K.

Data were measured using ω scans with Cu K_α radiation. The diffraction pattern was indexed and the total number of runs and images was based on the strategy calculation from the program CrysAlisPro 1.171.41.98a (Rigaku OD, 2021). The maximum resolution that was achieved was Θ = 79.70° (0.78 Å).

The unit cell was refined using CrysAlisPro 1.171.41.98a (Rigaku OD, 2021) on 13257 reflections, 91% of the observed reflections.

Data reduction, scaling and absorption corrections were performed using CrysAlisPro 1.171.41.98a (Rigaku OD, 2021). The final completeness is 100.00 % out to 79.70° in Θ. A numerical absorption correction based on gaussian integration over a multifaceted crystal model was performed using CrysAlisPro 1.171.41.108a (Rigaku Oxford Diffraction, 2021). An empirical absorption correction using spherical harmonics, implemented in SCALE3 ABSPACK scaling algorithm was also applied. The absorption coefficient μ of this material is 7.787 mm^{-1} at this wavelength (λ = 1.54184Å) and the minimum and maximum transmissions are 0.257 and 0.799.

The structure was solved and the space group $P2_1$ (# 4) determined by the ShelXT (Sheldrick, 2015) structure solution program using using dual methods and refined by full matrix least squares minimisation on $\boldsymbol{F}^2$ using version of olex2.refine 1.5-alpha (Bourhis et al., 2015). All non-hydrogen atoms were refined anisotropically. Hydrogen atom positions were calculated geometrically and refined using the riding model.

There is a single formula unit in the asymmetric unit, which is represented by the reported sum formula. In other words: Z is 2 and Z' is 1. The moiety formula is C22 H21 Br2 Cl3 O2.

The Flack parameter was refined to -0.01(1). Determination of absolute structure using Bayesian statistics on Bijvoet differences using the Olex2 results in -0.01(1). The chiral atoms in this structure are: C7(R). Note: The Flack parameter is used to determine chirality of the crystal studied, the value should be near 0, a value of 1 means that the stereochemistry is wrong and the model should be inverted. A value of 0.5 means that the crystal consists of a racemic mixture of the two enantiomers.

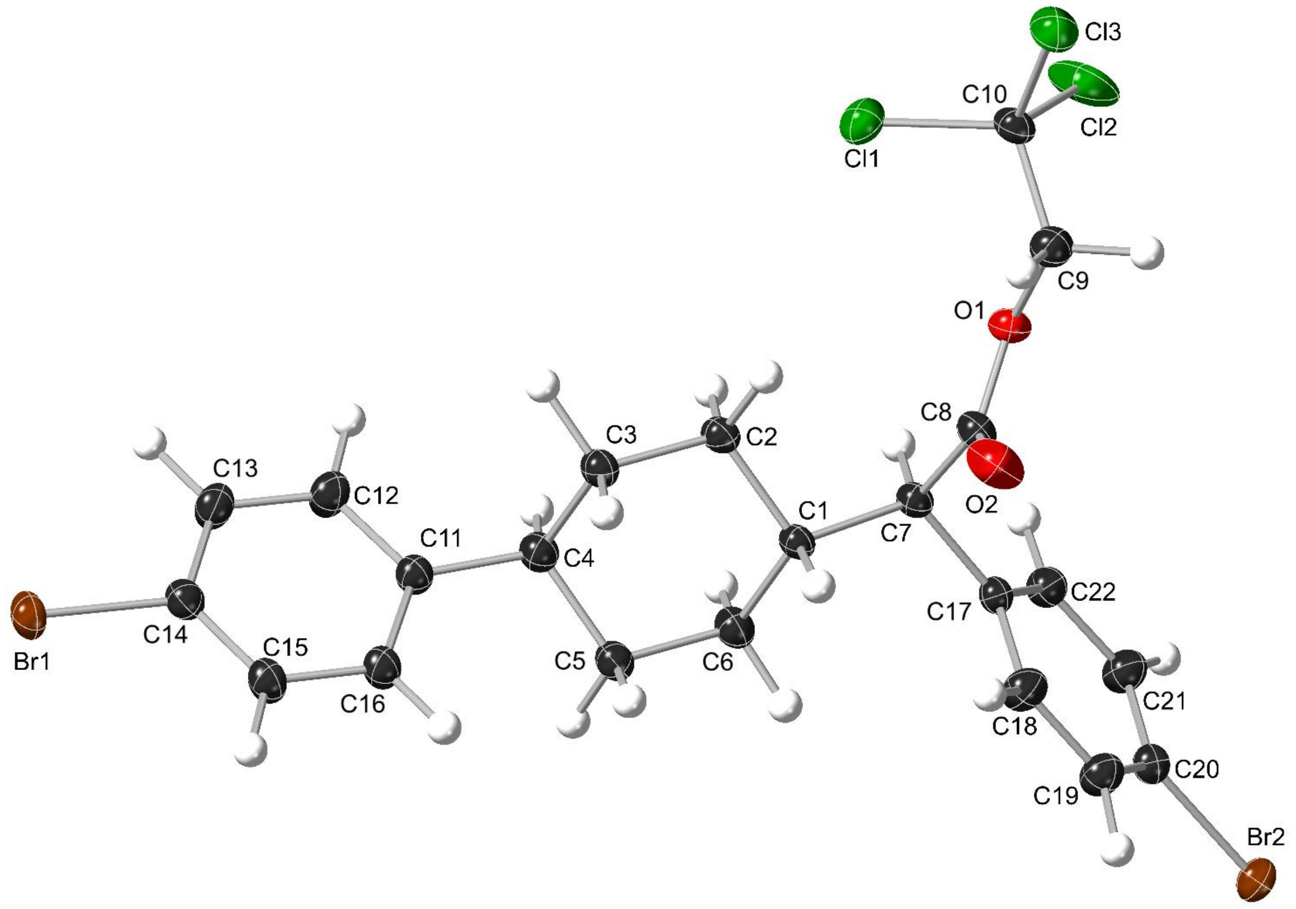

Figure S325. Thermal ellipsoidal representation (50% probability for all atoms, excluding hydrogens) of the molecular structure

_olex2_refine_details: Refinement using NoSpherA2, an implementation ofNOn-SPHERical Atom-form-factors in Olex2.Please cite:F. Kleemiss et al. Chem. Sci. DOI 10.1039/D0SC05526C - 2021NoSpherA2 implementation of HAR makes use oftailor-made aspherical atomic form factors calculatedon-the-fly from a Hirshfeld-partitioned electron density (ED) - not fromspherical-atom form factors.The ED is calculated from a gaussian basis set single determinant SCFwavefunction - either Hartree-Fock or DFT using selected funtionals- for a fragment of the crystal.This fragment can be embedded in an electrostatic crystal field by employing cluster chargesor modelled using implicit solvation models, depending on the software used.The following options were used: SOFTWARE: ORCA PARTITIONING: NoSpherA2 INT ACCURACY: Normal METHOD: PBE BASIS SET: def2-TZVP CHARGE: 0 MULTIPLICITY: 1 SOLVATION: Chloroform DATE: 2022-03-11_11-41-51

Data Plots: Diffraction Data

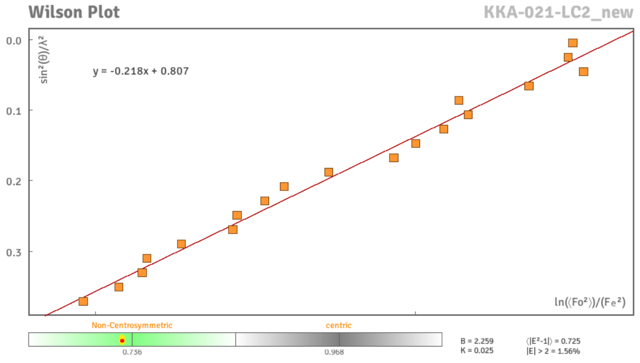

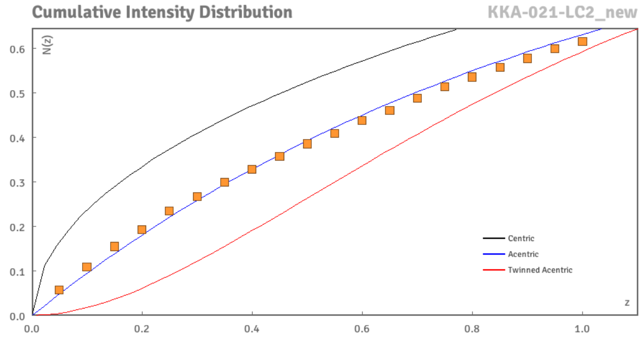

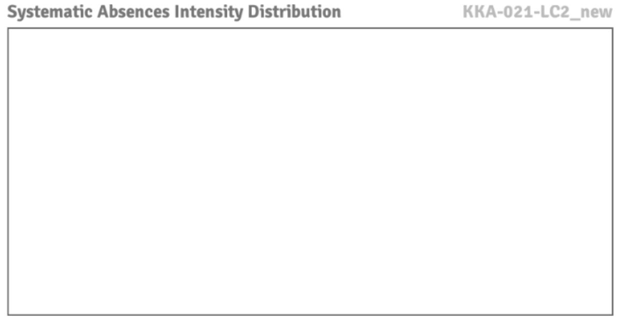

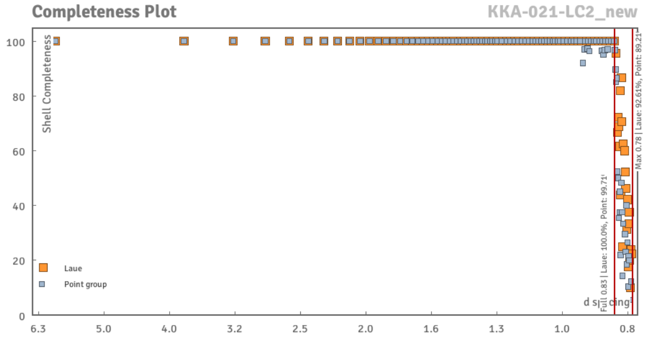

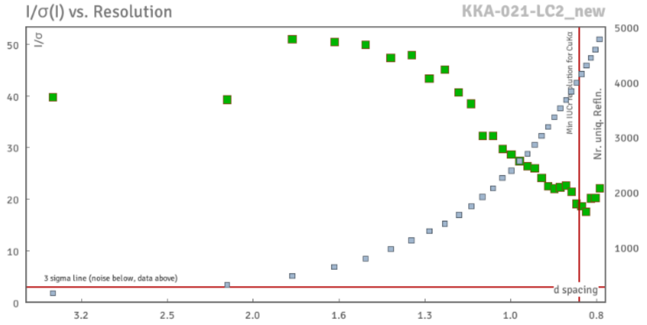

Data Plots: Refinement and Data

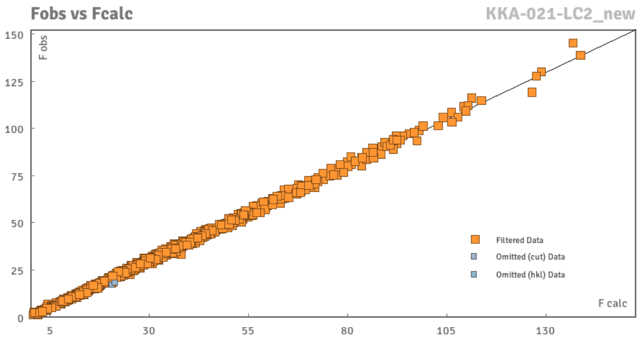

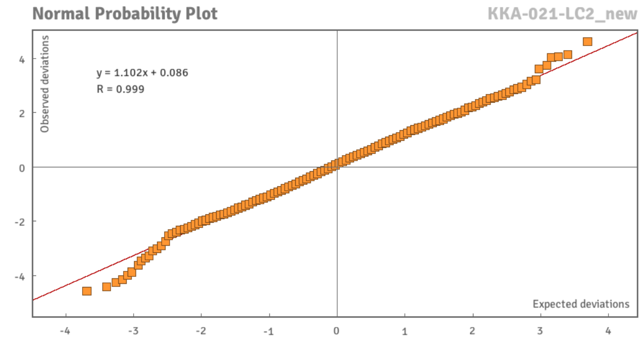

Reflection Statistics

Total reflections (after filtering)	14598	Unique reflections	4475

Completeness	0.892	Mean I/σ	32.99
hkl_{max} collected	(13, 6, 24)	hkl_{min} collected	(-12, -6, -24)
hkl_{max} used	(13, 6, 24)	hkl_{min} used	(-13, -6, 0)
Lim d_{max} collected	100.0	Lim d_{min} collected	0.77
d_{max} used	19.89	d_{min} used	0.78
Friedel pairs	2078	Friedel pairs merged	0
Inconsistent equivalents	6	R_{int}	0.0283
R_{sigma}	0.0243	Intensity transformed	0
Omitted reflections	0	Omitted by user (OMIT hkl)	2
Multiplicity	(2667, 1633, 1016, 516, 300, 158, 105, 29, 12, 2, 0, 1)	Maximum multiplicity	12
Removed systematic absences	0	Filtered off (Shel/OMIT)	0

Table S30. Fractional Atomic Coordinates (×10^4) and Equivalent Isotropic Displacement Parameters (Å^2×10^3) for **Compound 13a**. U_{eq} is defined as 1/3 of the trace of the orthogonalised U_{ij}.

Atom	x	y	z	U_{eq}
Br1	527.5(6)	833.9(3)	6265.1(3)	30.7(3)
Br2	11014.7(6)	9905.3(3)	2075.8(3)	32.3(3)
Cl1	1838.5(15)	3809.3(12)	792.4(8)	29.5(5)
Cl2	3418.9(17)	4066.6(12)	-336.7(8)	50.5(6)
Cl3	1210.3(16)	7161.5(12)	-277.2(8)	36.5(6)
O1	4253.8(15)	6475(3)	992.5(8)	21.8(4)
O2	4244(2)	9580(3)	1698.6(9)	33.5(4)
C1	4791(2)	5929(4)	2732.6(10)	18.0(4)
C2	3482(2)	4654(5)	2629.2(11)	23.8(5)
C3	2823(2)	4359(4)	3286.0(12)	23.8(5)
C4	3707(2)	3068(4)	3818.9(12)	22.5(5)
C5	4967(2)	4487(4)	3938.4(12)	24.1(5)
C6	5649(2)	4708(5)	3281.6(11)	23.5(5)
C7	5421(2)	6016(4)	2049.3(11)	18.2(4)
C8	4582(2)	7587(4)	1578.7(11)	21.1(5)
C9	3297(2)	7651(4)	560.3(13)	27.2(5)
C10	2487(2)	5727(5)	199.5(11)	25.3(5)
C11	2994(2)	2554(4)	4442.7(12)	22.4(5)
C12	2209(3)	539(5)	4447.1(14)	31.4(6)
C13	1483(3)	2(5)	4988.6(13)	30.4(5)
C14	1532(2)	1539(4)	5530.1(12)	23.8(5)
C15	2288(3)	3583(5)	5541.6(13)	30.3(6)
C16	3021(3)	4071(4)	4994.8(13)	28.4(5)
C17	6798(2)	6955(4)	2075.4(11)	18.0(4)
C18	7130(3)	9126(4)	2384.3(13)	27.3(5)
C19	8384(2)	10013(5)	2387.6(12)	27.8(5)
C20	9304(2)	8713(4)	2071.8(12)	24.2(5)
C21	9004(2)	6548(4)	1759.6(13)	27.7(5)
C22	7743(2)	5688(4)	1763.8(12)	22.4(5)

Table S31. Anisotropic Displacement Parameters (×10^4) for **Compound 13a**. The anisotropic displacement factor exponent takes the form: $-2\pi^2[h^2a^{*2} \times U_{11} + ... + 2hka^* \times b^* \times U_{12}]$

Atom	U_{11}	U_{22}	U_{33}	U_{23}	U_{13}	U_{12}
Br1	24.4(5)	46.4(6)	22.1(5)	-2.6(4)	7.4(3)	6.8(4)
Br2	23.6(5)	43.1(6)	30.6(5)	-12.1(4)	4.3(3)	2.7(4)

Atom	*U11*	*U22*	*U33*	*U23*	*U13*	*U12*
Cl1	18.3(9)	32.1(11)	37.8(10)	-0.5(8)	-1.3(7)	7.6(9)
Cl2	31.5(10)	95.1(14)	24.7(10)	14.8(9)	-0.7(8)	-25.3(9)
Cl3	21.8(9)	55.8(13)	30.5(10)	0.4(9)	-9.0(7)	13.3(9)
O1	21.1(8)	23.3(8)	20.5(7)	1.3(6)	-3.2(6)	-1.6(6)
O2	46.3(11)	25.4(10)	28.1(9)	16.0(8)	-3.3(8)	-4.2(7)
C1	16.3(9)	22.8(10)	15.0(9)	1.9(7)	1.8(6)	-1.5(7)
C2	19.2(10)	35.1(13)	17.3(10)	-2.5(8)	1.9(6)	-1.9(8)
C3	20.7(10)	33.2(14)	18.0(10)	-1.7(8)	4.1(7)	-1.9(7)
C4	25.0(11)	22.6(11)	20.2(10)	0.3(7)	4.3(7)	-3.2(7)
C5	22.0(10)	31.3(13)	18.9(10)	-0.3(8)	1.1(7)	2.1(8)
C6	19.2(10)	30.9(12)	20.4(10)	4.0(8)	1.5(6)	2.4(7)
C7	18.2(10)	20.5(11)	16.2(10)	2.2(9)	2.0(7)	-2.2(8)
C8	24.1(11)	21.9(11)	17.3(11)	4.0(8)	1.5(8)	-0.2(8)
C9	24.3(12)	28.2(13)	28.1(13)	-1.0(10)	-5.4(10)	6.5(10)
C10	17.3(10)	41.3(14)	17.0(10)	0.0(10)	-0.8(8)	-0.1(10)
C11	26.0(11)	21.3(10)	20.2(10)	-2.1(8)	3.7(7)	-1.1(6)
C12	38.0(13)	28.8(13)	28.8(11)	-11.7(9)	12.1(8)	-7.9(8)
C13	33.3(12)	29.6(12)	29.5(11)	-10.5(9)	10.2(8)	-3.5(8)
C14	23.2(10)	27.4(11)	20.8(10)	0.9(8)	1.2(7)	1.9(7)
C15	39.6(13)	32.3(13)	20.1(11)	-7.8(9)	8.5(8)	-3.3(8)
C16	38.7(13)	26.8(12)	20.6(10)	-9.1(9)	8.4(8)	-4.8(7)
C17	18.9(10)	19.4(10)	16.2(10)	0.9(7)	3.8(7)	-0.4(7)
C18	26.8(12)	24.4(11)	31.3(12)	-2.8(8)	6.9(8)	-8.8(9)
C19	28.8(11)	24.7(11)	30.3(12)	-4.5(8)	5.2(8)	-5.2(9)
C20	24.6(11)	28.1(11)	20.4(10)	-3.8(8)	4.1(7)	-0.4(8)
C21	22.4(11)	31.9(13)	29.6(13)	-2.6(8)	8.4(8)	-8.0(9)
C22	21.8(10)	23.1(11)	22.7(10)	1.1(8)	5.2(7)	-4.4(9)
H2	35(18)	20(6)	28(15)	-1(5)	10(12)	-5(4)
H3	35(9)	46(11)	62(12)	-1(5)	21(5)	-21(6)
H4	35(9)	46(11)	62(12)	-1(5)	21(5)	-21(6)
H5	22(12)	33(3)	41(12)	-1(2)	3(6)	-4(3)
H6	25(12)	33(4)	8(10)	2(3)	-2(5)	1(3)
H8	29(12)	27(5)	52(15)	-2(3)	11(6)	-15(4)
H9	25(9)	42(12)	29(9)	-4(5)	-3(4)	5(4)
H10	28(16)	49(18)	40(16)	-4(9)	-6(7)	21(8)
H11	35(9)	46(11)	62(12)	-1(5)	21(5)	-21(6)
H12	35(9)	46(11)	62(12)	-1(5)	21(5)	-21(6)
H13	28(14)	26(3)	31(15)	5(3)	-2(7)	-7(3)
H14	120(20)	57(8)	30(11)	-58(6)	44(9)	-27(5)
H15	23(5)	41(11)	37(12)	-1(3)	2(3)	-2(5)
H16	29(9)	44(12)	19(7)	8(5)	1(3)	3(4)
H17	36(17)	35(15)	50(18)	-2(7)	6(8)	-2(7)
H18	48(14)	33(3)	26(12)	8(3)	11(6)	3(3)
H20	26(5)	46(12)	35(14)	-9(4)	4(4)	-5(6)
H21	56(18)	41(10)	64(17)	-27(7)	21(9)	-10(7)
H22	100(30)	50(11)	38(11)	-29(9)	31(9)	-21(5)
H23	25(12)	36(4)	27(12)	-4(3)	8(6)	-4(3)
H24	120(20)	57(8)	30(11)	-58(6)	44(9)	-27(5)

Compound 42

Submitted by: **Duc Ly**

Solved by: **John Bacsa**

R_1=2.51%

Crystal Data and Experimental

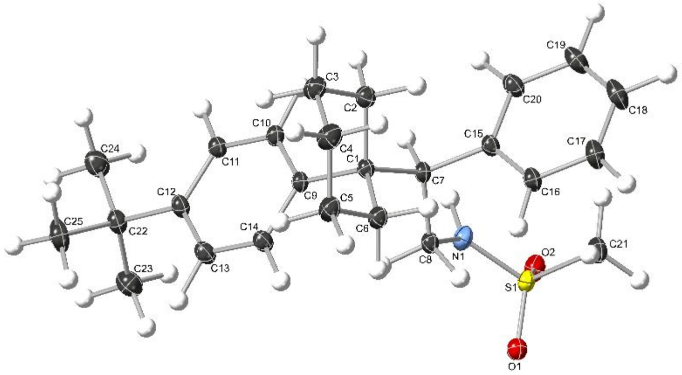

Experimental. Single colorless needle-shaped crystals of **Compound 42** recrystallised from a mixture of heptane and DMC by solvent layering. A suitable crystal with dimensions 0.19 × 0.02 × 0.02 mm³ was selected and mounted on a XtaLAB Synergy, Dualflex, HyPix diffractometer. The crystal was kept at a steady T = 100.00(10) K during data collection. The structure was solved with the ShelXT 2018/2 (Sheldrick, 2018) solution program and by using Olex2 1.5-alpha (Dolomanov et al., 2009) as the graphical interface. The model was refined with olex2.refine 1.5-alpha (Bourhis et al., 2015) using full matrix least squares minimisation on $\boldsymbol{F}^2$.

Crystal Data. $C_{25}H_{35}NO_2S$, M_r = 413.628, monoclinic, $P2_1$ (No. 4), a = 8.4387(2) Å, b = 8.8688(2) Å, c = 15.3317(3) Å, β = 97.229(2)°, α = γ = 90°, V = 1138.32(4) Å³, T = 100.00(10) K, Z = 2, Z' = 1, μ(Cu K$_\alpha$) = 1.410, 12403 reflections measured, 4064 unique (R_{int} = 0.0406) which were used in all calculations. The final wR_2 was 0.0569 (all data) and R_1 was 0.0251 (I≥2 σ(I)).

**Compound**	**42**
Formula	$C_{25}H_{35}NO_2S$
$D_{calc.}$/ g cm^{-3}	1.207
μ/mm^{-1}	1.410
Formula Weight	413.628
Color	colorless
Shape	needle-shaped
Size/mm³	0.19×0.02×0.02
T/K	100.00(10)
Crystal System	monoclinic
Flack Parameter	-0.002(9)
Space Group	$P2_1$
a/Å	8.4387(2)
b/Å	8.8688(2)
c/Å	15.3317(3)
α/°	90
β/°	97.229(2)
γ/°	90
V/Å³	1138.32(4)
Z	2
Z'	1
Wavelength/Å	1.54184
Radiation type	Cu K$_\alpha$
Θ_{min}/°	2.91
Θ_{max}/°	72.23
Measured Refl's.	12403
Indep't Refl's	4064
Refl's I≥2 σ(I)	3809
R_{int}	0.0406
Parameters	493
Restraints	520
Largest Peak	0.1176
Deepest Hole	-0.1521
GooF	1.0601
wR_2 (all data)	0.0569
wR_2	0.0560
R_1 (all data)	0.0281
R_1	0.0251

Structure Quality Indicators

**Reflections:**	d min (CuKα) 2Θ=144.5° 0.81	I/σ(I) 23.3	Rint m=3.03 4.06%	Full 135.4° 94% to 144.5° 98.5
**Refinement:**	Shift 0.001	Max Peak 0.1	Min Peak -0.2	GooF 1.060	Hooft -.002(9)

A colourless needle-shaped-shaped crystal with dimensions 0.19 × 0.02 × 0.02 mm^3 was mounted. Data were collected using a XtaLAB Synergy, Dualflex, HyPix diffractometer operating at T = 100.00(10) K.

Data were measured using ω scans with Cu K_α radiation. The diffraction pattern was indexed and the total number of runs and images was based on the strategy calculation from the program CrysAlisPro system (CCD 44.57a 64-bit (release 20-06-2024)). The maximum resolution that was achieved was Θ = 72.23° (0.83 Å).

The unit cell was refined using CrysAlisPro 1.171.43.121a (Rigaku OD, 2024) on 6812 reflections, 55% of the observed reflections.

Data reduction, scaling and absorption corrections were performed using CrysAlisPro 1.171.43.121a (Rigaku OD, 2024). The final completeness is 98.51 % out to 72.23° in Θ. A numerical absorption correction based on gaussian integration over a multifaceted crystal model was performed using CrysAlisPro 1.171.41.108a (Rigaku Oxford Diffraction, 2021). An empirical absorption correction using spherical harmonics, implemented in SCALE3 ABSPACK scaling algorithm was also applied. The absorption coefficient μ of this material is 1.410 mm^{-1} at this wavelength (λ = 1.54184Å) and the minimum and maximum transmissions are 0.748 and 1.000.

The structure was solved and the space group $P2_1$ (# 4) determined by the ShelXT 2018/2 (Sheldrick, 2018) structure solution program using using dual methods and refined by full matrix least squares minimisation on $\boldsymbol{F^2}$ using version of olex2.refine 1.5-alpha (Bourhis et al., 2015). All atoms, even hydrogen atoms, were refined anisotropically. Hydrogen atom positions were located from the electron densities and freely refined using Hirshfeld scattering factors. Refinement was by using NoSpherA2, an implementation of non-spherical atom-form-factors (F. Kleemiss, H. Puschmann, O. Dolomanov, S.Grabowsky - https://doi.org/10.1039/D0SC05526C – 2020). NoSpherA2 implementation of HAR makes use of tailor-made aspherical atomic form factors calculated from a Hirshfeld-partitioned electron density (ED) not from spherical-atom form factors. The ED was calculated from a Gaussian basis set single determinant SCF wavefunction from DFT using selected functionals for a fragment of this crystal. This fragment was embedded in an electrostatic crystal field by employing cluster charges. SOFTWARE: ORCA 5.0 PARTITIONING: NoSpherA2 INT ACCURACY: Normal METHOD: PBE BASIS SET: def2-SVP CHARGE: 0 MULTIPLICITY: 1 SOLVATION: Chloroform DATE: 2024-12-13_13-50-18

There is a single formula unit in the asymmetric unit, which is represented by the reported sum formula. In other words: Z is 2 and Z' is 1. The moiety formula is C25 H35 N O2 S.

The Flack parameter was refined to -0.002(9). Determination of absolute structure using Bayesian statistics on Bijvoet differences using the Olex2 results in None. The chiral atoms in this structure are: C7(S). Note: The Flack parameter is used to determine chirality of the crystal studied, the value should be near 0, a value of 1 means that the stereochemistry is wrong and the model should be inverted. A value of 0.5 means that the crystal consists of a racemic mixture of the two enantiomers.

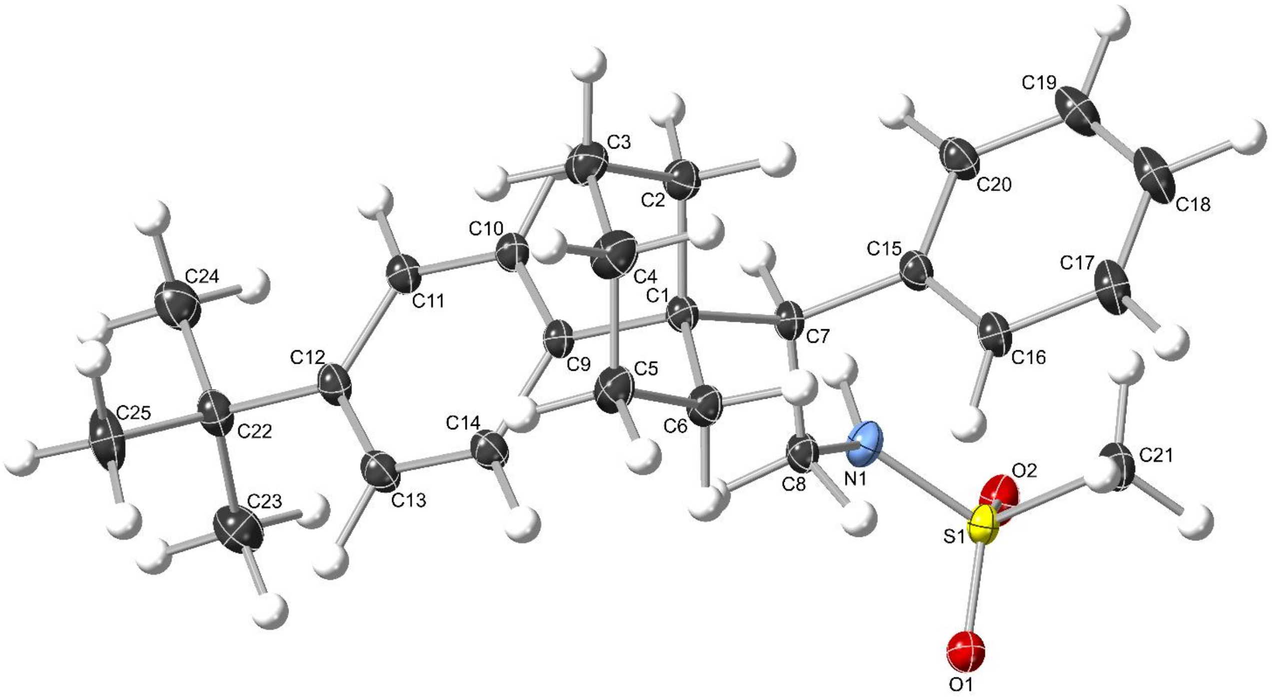

Figure S326. Thermal ellipsoidal representation (50% probability for all atoms, excluding hydrogens) of the asymmetric unit in the crystal.

Data Plots: Diffraction Data

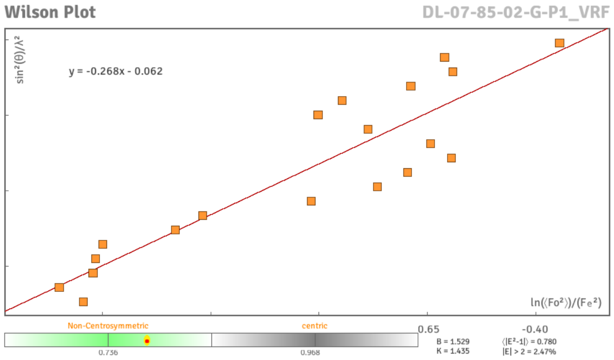

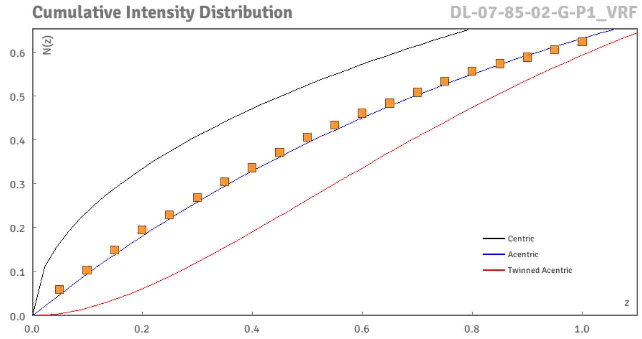

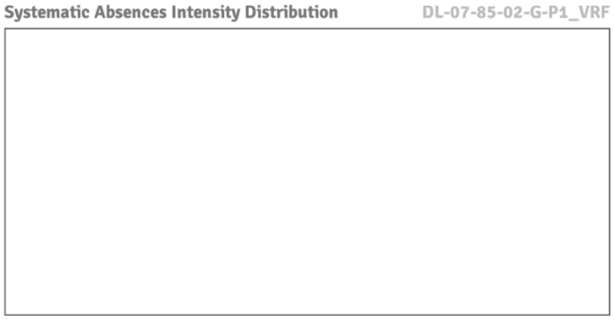

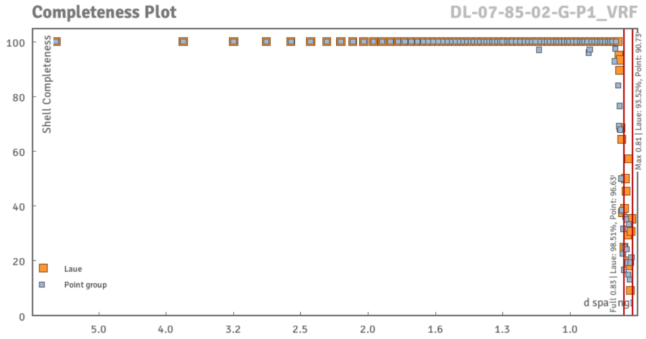

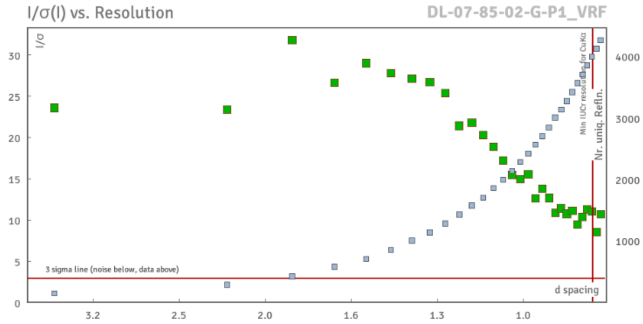

Data Plots: Refinement and Data

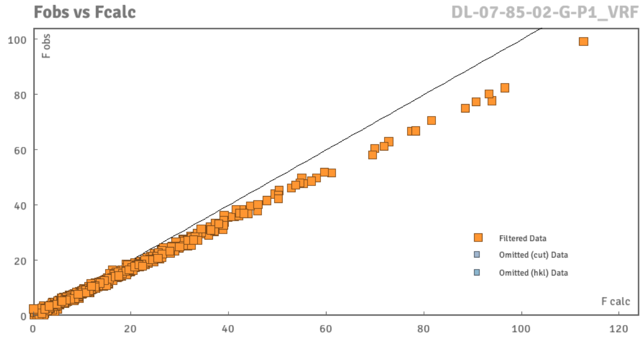

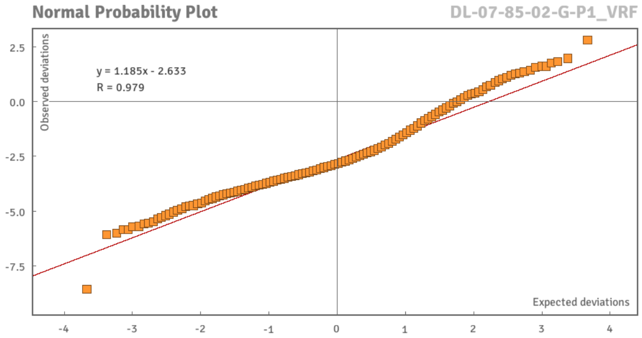

Reflection Statistics

Total reflections (after filtering)	12403	Unique reflections	4094
Completeness	0.914	Mean I/σ	18.38
hkl_{max} collected	(10, 10, 18)	hkl_{min} collected	(-10, -10, -18)
hkl_{max} used	(10, 10, 18)	hkl_{min} used	(-10, -10, 0)
Lim d_{max} collected	100.0	Lim d_{min} collected	0.77
d_{max} used	15.21	d_{min} used	0.81
Friedel pairs	2106	Friedel pairs merged	0
Inconsistent equivalents	8	R_{int}	0.0406
R_{sigma}	0.0429	Intensity transformed	0
Omitted reflections	0	Omitted by user (OMIT hkl)	0
Multiplicity	(2447, 1615, 980, 528, 187, 83, 26, 4, 3)	Maximum multiplicity	10
Removed systematic absences	0	Filtered off (Shel/OMIT)	0

Table S32. Fractional Atomic Coordinates (×10⁴) and Equivalent Isotropic Displacement Parameters (Å²×10³) for **Compound 42.** U_{eq} is defined as 1/3 of the trace of the orthogonalised U_{ij}.

Atom	x	y	z	U_{eq}
C1	5528.3(14)	6705.7(15)	2232.2(8)	16.0(3)
N1	5290.3(15)	6126.9(13)	4690.8(8)	20.4(3)
O1	4102.8(11)	7982.5(11)	5622.1(6)	21.2(2)
S1	5408.7(4)	6930.0(4)	5633.7(2)	16.10(8)
C2	6914.0(17)	6347.4(17)	1691.3(10)	19.7(3)
O2	5557.6(12)	5738.7(11)	6274.2(6)	23.0(2)
C3	6469.5(18)	6622.1(16)	707.3(9)	24.2(3)
C4	5986.1(19)	8263.0(18)	533.3(10)	26.8(3)
C5	4606.2(18)	8680.3(18)	1041.3(9)	23.1(3)

Atom	x	y	z	U_{eq}
C6	5005.4(17)	8343.6(16)	2025.2(9)	18.5(3)
C7	6164.8(15)	6477.7(15)	3234.4(9)	16.5(3)
C8	4901.9(14)	6887.4(19)	3839.2(8)	17.6(3)
C9	4159.7(16)	5559.4(16)	2014.9(9)	16.7(3)
C10	4474.6(17)	4006.5(16)	2018.5(9)	18.2(3)
C11	3264.1(16)	2933.4(17)	1863.4(9)	19.5(3)
C12	1659.1(16)	3349.5(16)	1690.3(9)	19.6(3)
C13	1334.4(17)	4898.8(16)	1693.0(10)	22.9(3)
C14	2544.5(16)	5973.6(16)	1850.3(10)	21.2(3)
C15	7752.9(15)	7222.1(15)	3559.3(9)	17.7(3)
C16	7910.4(16)	8778.2(16)	3686.1(9)	19.7(3)
C17	9377.6(17)	9416.8(18)	4011.8(10)	24.9(3)
C18	10715.2(17)	8503(2)	4226.2(11)	30.6(4)
C19	10569.8(16)	6951(2)	4112.0(10)	29.0(3)
C20	9102.2(17)	6315.0(19)	3782.0(10)	23.4(3)
C21	7182.3(16)	7994.7(18)	5788.9(10)	23.8(3)
C22	286.5(16)	2211.1(16)	1526.3(10)	23.0(3)
C23	-785.5(19)	2355.9(18)	2262.5(11)	28.9(4)
C24	879(2)	578.0(18)	1520.2(13)	33.4(4)
C25	-709(2)	2543(2)	636.1(11)	34.1(4)

Table S33. Anisotropic Displacement Parameters (×10[4]) for **Compound 42.** The anisotropic displacement factor exponent takes the form: $-2\pi^2[h^2a^{*2} \times U_{11} + \ldots + 2hka^* \times b^* \times U_{12}]$

Atom	U_{11}	U_{22}	U_{33}	U_{23}	U_{13}	U_{12}
C1	15.6(6)	18.0(8)	13.5(6)	1.2(5)	-1.7(5)	-0.7(5)
H1	58(13)	24(4)	21(9)	9(3)	-14(5)	-6(2)
N1	28.2(6)	19.7(7)	12.8(6)	-1.3(5)	0.4(5)	-0.3(5)
O1	20.9(5)	22.5(5)	19.7(5)	1.2(4)	0.0(4)	-2.0(4)
S1	18.87(15)	16.46(15)	12.20(15)	-1.53(14)	-1.01(11)	0.33(14)
C2	19.6(7)	23.2(7)	16.1(7)	0.5(6)	1.0(5)	-0.3(6)
O2	32.5(5)	19.3(5)	16.2(5)	-2.7(4)	-1.1(4)	3.9(4)
C3	27.7(7)	29.1(10)	15.9(7)	1.7(6)	2.4(5)	-2.2(6)
C4	35.2(8)	28.5(9)	16.6(8)	3.0(7)	2.9(6)	2.4(7)
C5	29.6(8)	25.1(8)	13.0(7)	2.8(6)	-3.1(6)	1.7(6)
C6	21.5(7)	20.1(8)	12.9(7)	1.7(6)	-1.4(6)	0.9(6)
C7	16.5(6)	18.8(8)	13.1(7)	-0.8(5)	-2.3(5)	-0.9(5)
H7	33(8)	19(3)	34(10)	1.9(17)	4(5)	2.2(18)
C8	18.0(6)	21.4(7)	12.7(6)	-1.7(6)	-0.8(4)	-0.4(6)
C9	15.9(6)	19.3(7)	13.9(7)	-0.1(6)	-2.1(5)	-2.0(5)
C10	17.6(7)	18.8(7)	17.2(7)	0.2(6)	-1.6(5)	-2.5(6)
H10	20(6)	42(9)	59(9)	6(4)	2(4)	-12(7)
C11	17.3(7)	21.3(8)	18.9(7)	0.4(6)	-1.4(5)	-2.3(6)
H11	24(6)	30(6)	49(9)	3(3)	2(5)	-9(4)
C12	16.2(6)	22.6(8)	18.5(7)	-0.2(6)	-3.6(5)	0.0(6)
C13	16.4(7)	22.9(8)	28.0(8)	1.4(6)	-2.8(6)	-0.5(6)
H13	24(6)	30(6)	49(9)	3(3)	2(5)	-9(4)
C14	16.3(7)	19.5(8)	26.6(8)	2.2(6)	-2.3(6)	-0.9(6)
H14	20(6)	42(9)	59(9)	6(4)	2(4)	-12(7)
C15	15.8(6)	21.7(8)	14.6(6)	0.2(5)	-2.1(5)	0.2(5)
C16	18.2(7)	22.3(8)	17.2(7)	-2.8(5)	-3.5(5)	1.8(6)
H16	24(4)	24(5)	59(9)	1(2)	-13(3)	-6(4)
C17	21.7(7)	28.9(9)	22.3(8)	-8.3(6)	-5.0(6)	3.9(6)
H17	29(7)	30(3)	68(11)	-8.8(18)	-14(5)	-0.6(18)
C18	18.1(7)	41.7(10)	29.6(9)	-7.1(6)	-6.2(6)	6.1(7)

Atom	U_{11}	U_{22}	U_{33}	U_{23}	U_{13}	U_{12}
C19	15.1(6)	39.0(8)	31.2(8)	-0.8(7)	-4.1(5)	6.6(8)
H19	29(7)	30(3)	68(11)	-8.8(18)	-14(5)	-0.6(18)
C20	16.4(7)	29.2(8)	23.2(8)	2.6(6)	-2.9(5)	1.1(6)
H20	24(4)	24(5)	59(9)	1(2)	-13(3)	-6(4)
C21	22.4(7)	30.2(8)	17.7(8)	-3.8(6)	-1.8(6)	-0.8(6)
C22	19.1(6)	24.0(9)	24.1(8)	-2.9(6)	-3.8(5)	0.0(6)
C23	23.7(8)	31.1(9)	31.6(9)	-0.8(6)	2.2(6)	6.3(7)
C24	22.9(8)	27.2(9)	48.7(12)	-3.4(7)	-1.6(7)	-4.7(8)
C25	29.7(9)	41.6(10)	27.2(9)	-9.4(7)	-11.5(7)	2.1(7)
H2a	21(6)	26(7)	26(8)	1(3)	-1(4)	2(5)
H3a	34(6)	38(9)	35(8)	-2(4)	12(3)	-11(5)
H4a	67(11)	52(12)	22(5)	5(6)	2(3)	7(3)
H5a	40(9)	23(5)	21(9)	5(3)	1(6)	6(3)
H6a	38(7)	34(8)	25(9)	-11(3)	-7(4)	5(5)
H8a	24(4)	43(8)	33(7)	-7(2)	-6(2)	-2(4)
H21a	42(10)	35(7)	29(7)	-4(5)	9(4)	1(3)
H23a	40(8)	40(11)	83(14)	-9(5)	7(6)	10(7)
H24a	41(10)	27(11)	67(9)	-9(7)	-11(5)	4(5)
H25a	62(13)	52(8)	46(12)	4(5)	-16(8)	2(5)
H2b	28(9)	29(5)	33(9)	6(3)	10(5)	4(3)
H3b	41(7)	44(9)	31(9)	-10(4)	-3(4)	2(5)
H4b	48(7)	41(9)	50(11)	-9(4)	1(4)	2(5)
H5b	41(6)	31(8)	30(9)	-3(3)	-9(4)	3(5)
H6b	30(6)	30(9)	29(8)	6(4)	7(3)	3(5)
H8b	29(7)	24(3)	34(8)	-1.0(17)	3(4)	-2.7(17)
H18	22(4)	59(9)	52(12)	-14(3)	-10(3)	2(5)
H21b	50(10)	50(10)	32(6)	-6(6)	1(4)	-10(3)
H23b	53(11)	87(15)	42(8)	3(8)	-7(5)	15(6)
H24b	39(9)	30(10)	105(17)	-9(5)	-5(6)	-3(7)
H25b	40(9)	101(19)	37(9)	-8(7)	-7(4)	-4(7)
H21c	35(7)	48(9)	60(11)	6(4)	4(4)	-4(5)
H23c	53(12)	45(8)	72(14)	9(4)	27(8)	9(5)
H24c	33(9)	59(13)	62(10)	9(7)	2(5)	-1(6)
H25c	39(9)	51(11)	58(12)	-13(5)	-15(6)	3(7)

Citations for crystallography study

CrysAlisPro (ROD), Rigaku Oxford Diffraction, Poland.

CrysAlisPro Software System, Rigaku Oxford Diffraction, (2024).

L.J. Bourhis and O.V. Dolomanov and R.J. Gildea and J.A.K. Howard and H. Puschmann, The Anatomy of a Comprehensive Constrained, Restrained, Refinement Program for the Modern Computing Environment - Olex2 Disected, *Acta Cryst. A*, (2015), **A71**, 59-71.

O.V. Dolomanov and L.J. Bourhis and R.J. Gildea and J.A.K. Howard and H. Puschmann, Olex2: A complete structure solution, refinement and analysis program, *J. Appl. Cryst.*, (2009), **42**, 339-341.

Sheldrick, G.M., ShelXT-Integrated space-group and crystal-structure determination, *Acta Cryst.*, (2015), **A71**, 3-8.

12. DFT calculations

All calculations were performed using Gaussian 16 suite of programs.[20] Images of 3D structures were rendered using VMD[21], CYLView[22], Mercury[23], and GaussView 6.1[24]. Geometry optimizations were carried out using the density functional B3LYP-D3(BJ)[25-30] in conjunction with Lanl2dz[31, 32] basic set for rhodium and 6-31G(d,p)[33, 34] basic set for other atoms in chloroform ($CHCl_3$) as solvent using the Conductor-like Polarizable Continuum Solvation Model (CPCM)[35, 36]. Ground and transition state geometries were validated by vibrational analysis at the same level employed for optimization, showing zero and one imaginary frequencies respectively. Gibbs free energy and Zero-point energy corrections were calculated at a temperature and pressure corresponding to standard reaction conditions (298.15K, 1 atm). To confirm that all located saddle points correspond to relevant transformations, Intrinsic Reaction Coordinate (IRC) calculations[37, 38] were performed (total of 50 points for each directions) followed by subsequent optimization of the end points with the previously mentioned optimization method. The choice of computational approach was based on prior studies showing that the used [B3LYP-D3(BJ) + PCM($CHCl_3$)]/[6-31G(d,p)+Lanl2dz] approach describes appropriately the geometries of organic and organometallic species.[39-42]

The natural bond orbital (NBO) analysis was used for computing charges on the atoms.[43, 44] The electrophilicity was estimated using global electrophilicity index:[45] $\omega = \frac{\mu^2}{2\eta}$; η(harness) = $\Delta E_{HOMO-LUMO}$; μ(electronegativity) = $\frac{(E_{HOMO}+E_{LUMO})}{2}$. The distortion-interaction analysis was performed following the Houk-Bickelhaupt protocol[46]. The steric plots were generated with by SambVca 2.1 program[47]. IGMH analysis[48] and ETS-NOCV analysis [49] were performed with Multiwfn 3.8(dev)[50] and visualized by VMD program using the default parameters and a grid resolution of 0.15 Bohr with the isovalue of 0.0035. The inputs for IGMH and ETS-NOCV analyses are obtained from the computational output (.fchk) from Gaussian 16. sobEDAw[51] calculation was also performed with Multiwfn 3.8(dev)[50].

12.1. Evaluation of catalyst structure

The optimized structure from DFT calculations showed a good alignment with one obtained from X-ray. This would imply that the solution structure of Rh_2(*S*-TPNTTL)$_4$ has not changed much compared to the solid state. This result also supports for the selection of [B3LYP-D3(BJ)+PCM($CHCl_3$)/ Lan2ldz+6-31G(d,p)] approach. (**Figure S327**)

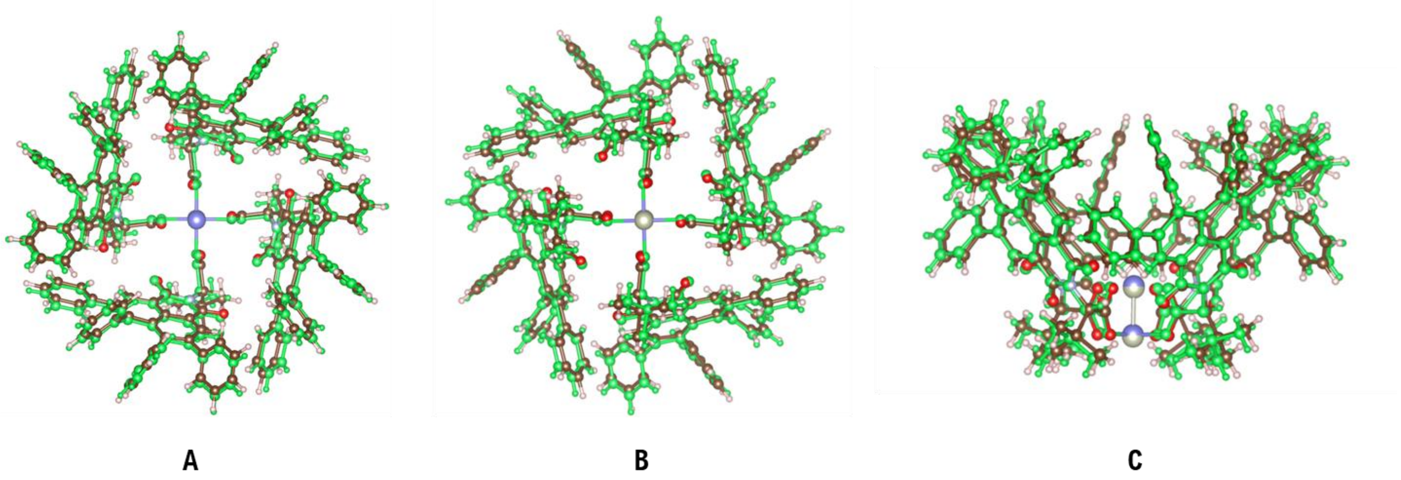

Figure S327. Superimposed X-ray and DFT calculated structures of Rh_2(*S*-TPNTTL)$_4$ (3a). Green: solid-state structure from X-ray; Colorful: structure from DFT calculation. A. Top view; B. Bottom view; C. Side view (VESTA program[52] was used to overlay 2 structure). Root means square deviation (RMSD) between the 2 structures is 0.38 Å (this was calculated by pymol program using align function).

12.2. A comparison of imino carbene and carboxylate carbene complexes of $Rh_2(OAc)_4$

A comparison between metal-imino carbene and metal-carboxylate carbene intermediates was conducted to gain a better understanding about the difference between two metal-carbene intermediates. The structures of model metal-imino carbene **S-1** and metal-carboxylate carbene **S-2** are illustrated in **Figure S328**. The most significant structural difference between **S-1** and **S-2** from DFT-optimized structures is the dihedral angel between the π-plane of electron-withdrawing group (EWG) and rhodium carbene π plane. While the π plane of the carboxylate group of **S-2** stays almost orthogonal to rhodium carbene plane with the dihedral angle of -85.3°,[40, 42] the π plane of the imine group of **S-1** slightly rotates and partially interacts with rhodium carbene π plane via the dihedral angle of 115.6°. The interaction of the π-bond of the imine group could be visualized by the LUMO picture of the complex. (**Figure S328**) As the imine group is a withdrawing group, the interaction between imine group with rhodium carbene bond makes **S-1** become more electrophilic as supported by the LUMO energy (ε_{LUMO}), global electrophilicity index (ω), and the NBO-charges on carbene center. (**Figure S328**)

As the imine group in **S-1** interacts differently with the carbene center, compared to the carboxylate group in **S-2**, we then evaluated the rotational barrier of the imine group around the C-C bond. (**Figure S329**) The rotational barrier around the C-C bond stems from the fact that having the π-plane of the electron-withdrawing group in the same plane with the rhodium carbene π bond would electronically destabilized the electrophilic carbene center.[40] The reported interconversion barrier of **S-2** to **S-3** is 13.0 kcal/mol,[40] but the interconversion barrier of **S-1** to **S-4** is only 8.7 kcal/mol. The lower rotational barrier of imino carbene **S-1** could be attributed to the weaker electron-withdrawing ability of imine group compared to the ester group, hence the destabilization effect is lessened.

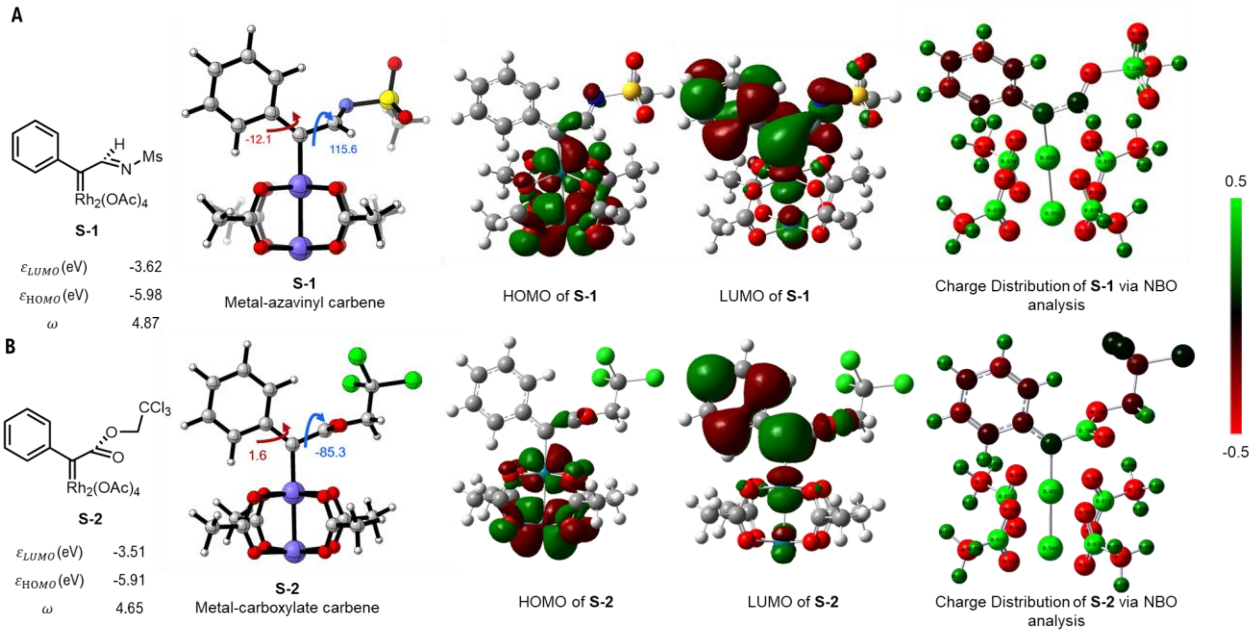

Figure S328. **A comparison of imino carbene (a) and carboxylate carbene (b) complexes of $Rh_2(OAc)_4$**. From left to right: chemical structure with LUMO energy (ε_{LUMO}), HOMO energy (ε_{LUMO}) and electrophilicity index (ω), DFT optimized structure with highlighted important dihedral angle, HOMO, LUMO, and Charge distribution via NBO analysis.

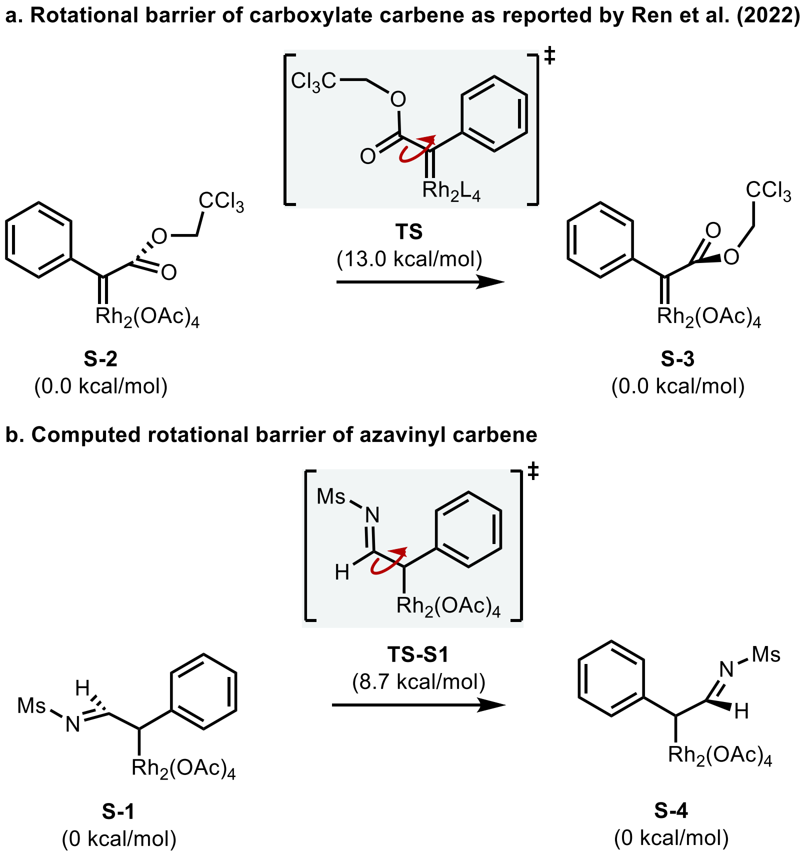

Figure S329. The comparison of the rotation barrier of carboxylate and imino carbene. a) The reported rotational barrier of carboxylate carbene.[40] b) The computed rotational barrier of imine carbene. Reported free energies are in kcal/mol.

12.3.Study on the C-H insertion with [imino-carbene]-$Rh_2(OAc)_4$

Due to the diastereomerism of donor-acceptor carbene, the substrate could approach the electrophilic carbene center from either the nitrogen side (N approach) or the hydrogen side (H approach) of the imine. We first evaluated the model system with $Rh_2(OAc)_4$ with cyclohexane as a substrate. The results revealed that the attack of the cyclohexane from the N side (**TS-S2**) is more energetically favorable than the attack from the H side (**TS-S1**) by 0.5 kcal/mol. (**Figure S330)** Although the energy difference is minimal, the preference of **TS-S2** could be attributed to the extra interaction of nucleophilic nitrogen atom with the hydrogens on the approaching cyclohexane.

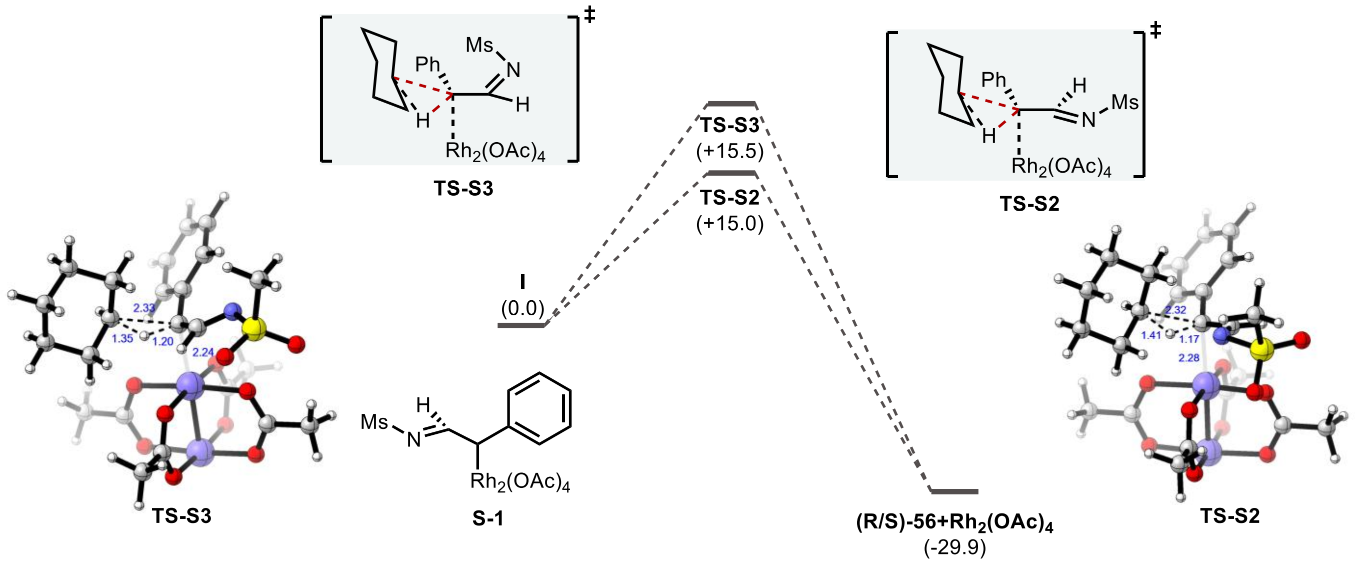

Figure S330. Free energy diagram for the C-H insertion of $Rh_2(OAc)_4$-imino carbene with cyclohexane. TS-S2 and TS-S3 are transition states for the C-H insertion with cyclohexane from N approach and H approach, respectively. Reported energies are in kcal/mol.

12.4. Study on the origin of enantioselectivity

12.4.1. Model study with Rh_2(*S*-NTTL)$_4$

The energy diagram for the C-H insertion of cyclohexane with [imine-carbene]-Rh_2(*S*-NTTL)$_4$ is depicted in **Figure S331**. Two most stable diastereomers **I** and **II** could be formed upon the coordination of imino carbene to dirhodium catalysts. Similar to the model study with $Rh_2(OAc)_4$ in **Figure S330**, the approach of cyclohexane from the N side (**TS1**, **TS2**) is more energetically favorable than the approach from H side (**TS1'**,**TS2'**) for both diastereomers **I** and **II**. Because the isomerization barrier of **II** to **I** is lower than the subsequent C-H functionalization barrier, the studied system is under Curtin-Hammet control. It means that the enantioselectivity does not depend on the ratio of **I** and **II**, but it mainly depends on the energy difference of **TS1** with **TS2** and with **TS1'**.

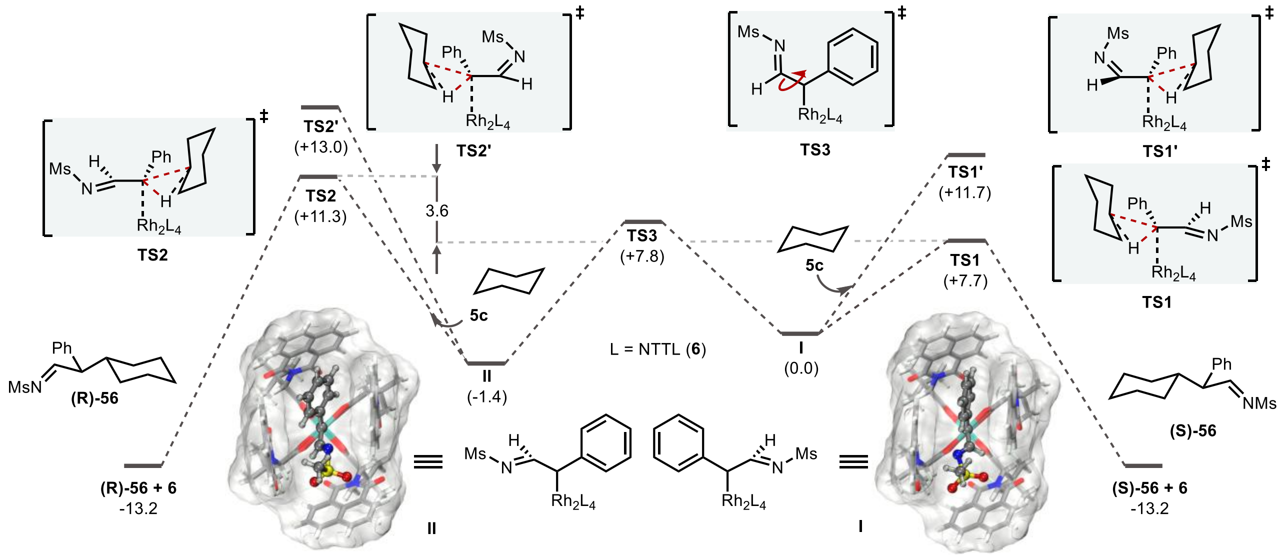

Figure S331. The calculated free energy diagram of the cyclohexane C-H insertion by [imine-carbene]-Rh_2(*S*-NTTL)$_4$ complex. The relative free energies (r.t. **I**) are in kcal/mol.

We further analyzed the studied transition states by comparing of their important geometry parameters, as well as the degree of non-covalent interactions (NCI, green density surface, in Figure S6) between the substrate (cyclohexane), and metal-carbene fragments, rationalized by independent gradient model based on Hirshfeld partition (IGMH) approach[48] (see **Figure S332**). Close examination of structures presented in **Figure S332** shows that the interaction between substate and catalyst pocket generally consists of green surface, representing weak interactions, except the area around the carbene center where there is strong attractive interaction as the C-H insertion happens. As a result, the interactions between substate and catalyst (i.e. ligand) are generally weaker than those between substrate and coordinated carbene. The activated C-H bond distance is longest in **TS1** followed by **TS2**, **TS2'**, and **TS1'**. This implies that the C-H insertion transition state **TS1** is a later transition state (more like product) compared to **TS2**, **TS2'**, and **TS1'**.

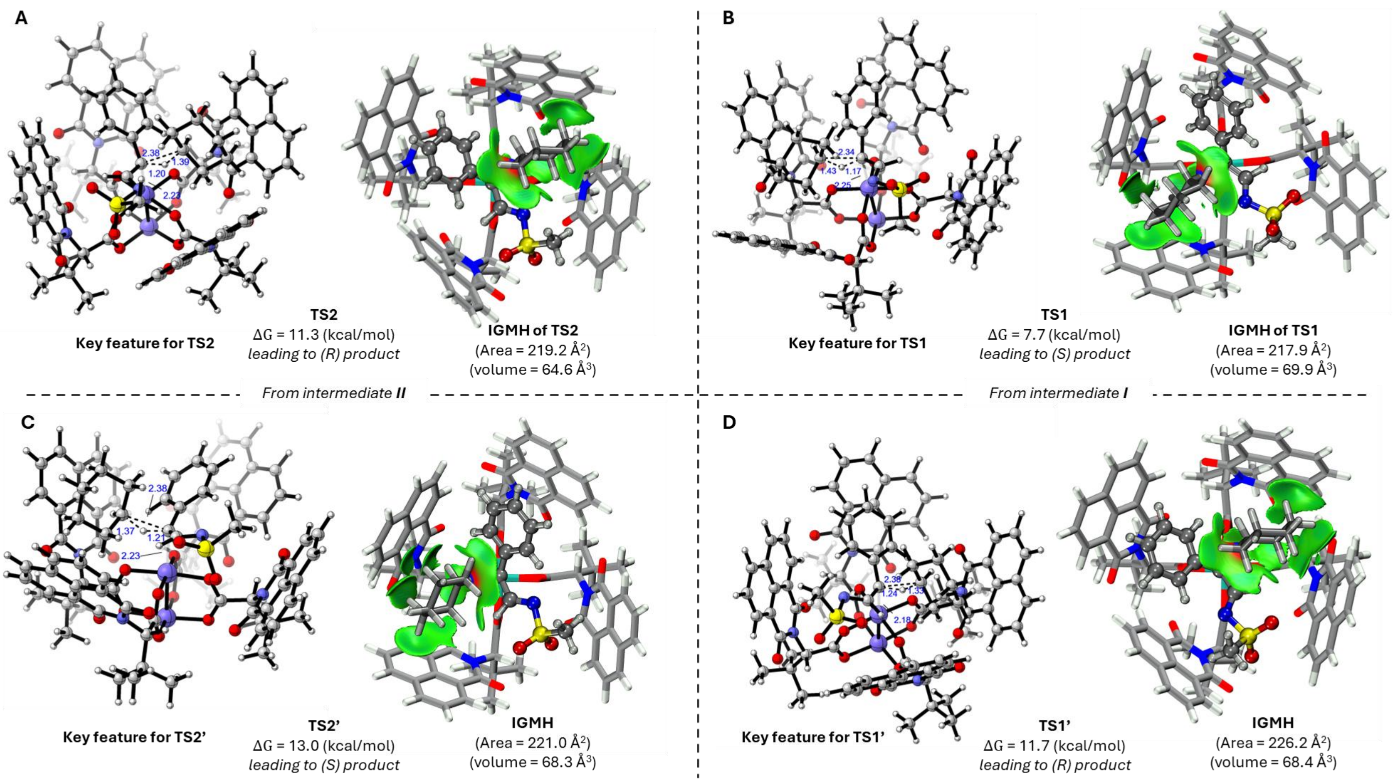

Figure S332. Key geometry features and NCI maps for TS1, TS1', TS2, TS2'. The area and volume of the isosurface are generated by the IGMH method by using isovalue = 0.0035.

In order to gain an further insight into the energy differences of the identified transition states **TS1, TS1', TS2, TS2'**, we performed distortion-interaction analysis by Houk-Bickelhaupt protocol with slightly modification.[46] Particularly, the distortion and interaction energies were studied relative to each other instead of respect to reactants. Each transition state structure was divided into two fragments: cyclohexane **5c** (or **5a**) (substrate) and a metal-carbene fragment. (**Figure S333**)

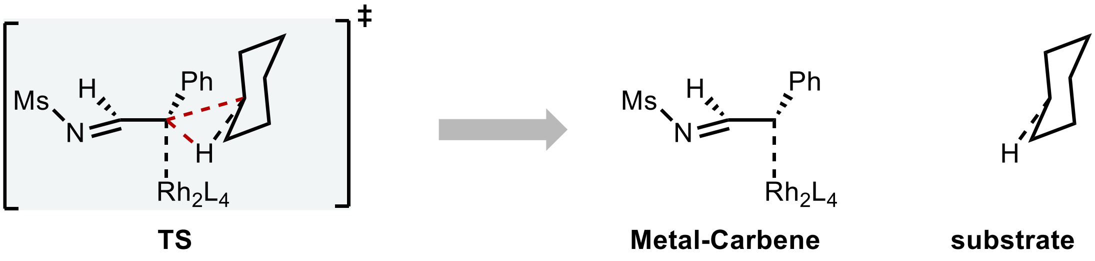

Figure S333. Schematic presentation of fragmentation. This fragmentation is used in distortion-interaction analysis and ETS-NOCV.

Then, the single-point energy calculations of each transition state and their separated fragments were performed at the B3LYP-D3(BJ)/6-31G(d,p) (for C, H, N, O, S) and Lan2ldz (for Rh) level of theory (no solvation model). The calculated relative (r.t. TS1) total (ΔE_{TS}), distortion ($\Delta E_{dis} = \Delta E_{cat} + \Delta E_{sub}$), and interaction ($\Delta E_{int}$) energies of each transition states are shown in **Table S34**. These analyses show that although metal-carbene and substrate fragments are most distorted in **TS1**, the key dominant factor making **TS1** the energetically most stable is the interaction between these fragments. The energy decomposition analyses performed at the sobEDAw method[51] were then conducted to deconvolute the types of interactions that are affecting the difference of interaction energy between **TS1**, **TS1'**, **TS2**, and **TS2'**. In sobEDAw studies, the transition state was divided into the same two fragments: cyclohexane **5c** (substrate)

and a metal-carbene. The sobEDAw method decomposed interaction into four components: ΔE_{int} = ΔE_{els}(electrostatic) + ΔE_{xrep} (exchange-repulsion) + ΔE_{orb} (orbital) + ΔE_{disp} (dispersion). These analysis were performed using Multiwfn 3.8 program at the B3LYP-D3(BJ)/6-31G(d,p)-Lan2ldz(Rh) level of theory (no solvation). The results of sobEDAw analysis are illustrated in **Table S34**. As seen in this table, the total interaction in **TS1** is ΔE_{int} = -57.06 kcal/mol while that in **TS1'** and **TS2** are only -41.58 kcal/mol, and -49.73 kcal/mol, respectively. Further analyses show that the main reason for the calculated difference in interaction energies stemmed from the electrostatic and orbital components. The electrostatic component could be explained by the fact that **TS1** is a slightly later transition state than **TS2** and **TS1'**, as the formed C-H bond lengths (between the carbene carbon and the hydrogen of substrate) are 1.70 Å, 1.20 Å, and 1.24, respectively. To further understand the electrostatic component, we analyzed the NBO charges of atoms mainly involving in the C-H insertion, i.e. C (carbene), Rh (carbene), C (substrate), and H (substrate). The results showed that C (carbene) atoms and H (substrate) atoms are both most negatively charged and most positively charged in **TS1**, respectively. (**Table S36**) On the other hand, Extended Transition State-Natural Orbital for Chemical Valance-method (ETS-NOCV)[49] was applied to identified the major orbital interaction involved in the orbital component. (**Figure S334**) The pair of orbitals dominantly contributing to the ΔE_{orb} component was σ (C–H) orbital of the substrate and π* orbital of the three-center RhRhC superelectrophile[53, 54] in all four transition states (see Figure S8A). The stronger interaction of this orbital pair in **TS1** could be explained as the attack of substrate to the *Si*-face of carbene intermediate **I** is supported by the surrounding ligand environment, leading to a better orbital overlap.

Table S34. Relative (r.t. TS1) distortion-interaction analysis of the calculated C-H insertion transition states. The single point energy of metal-carbene (E_{cat}) and substrate (E_{sub}) fragments at their geometries in corresponding transition states were computed at the B3LYP-D3(BJ)/Lan2ldz+6-31G(d,p) level of theory in gas phase. Here, ΔE_{dis}, ΔE_{cat}, and ΔE_{sub} are differences (relative to that in TS1) in total distortion energy, distortion energy of metal-carbene fragment, and distortion energy of substrate fragment, respectively. The total distortion energy is defined as $\Delta E_{dis} = \Delta E_{sub} + \Delta E_{cat}$. $\Delta E_{int} = \Delta E_{TS} - \Delta E_{dis}$, and is difference in interaction energies. (All presented energies are in kcal/mol).

Structure	E_{TS}	E_{cat}	E_{sub}	ΔE_{TS}	ΔE_{cat}	ΔE_{sub}	ΔE_{dis}	ΔE_{int}
**TS1**	-3521872.540	-3373804.268	-148011.211	0.0	0.0	0.0	0.0	0.0
**TS1'**	-3521868.716	-3373804.436	-148022.702	3.8	-0.2	-11.5	-11.7	**15.5**
**TS2**	-3521867.801	-3373801.343	-148016.731	4.7	2.9	-5.5	-2.6	**7.3**
**TS2'**	-3521865.548	-3373799.256	-148018.472	7.0	5.0	-7.3	-2.3	**9.3**

Table S35. Result of the energy-decomposition analysis via the sobEDAw method for TS1, TS1', TS2, and TS2'. These analyses were performed by Multiwfn 3.8 program and computed at the B3LYP-D3(BJ)/6-31G(d,p)-Lan2ldz(Rh) level of theory in the gas phase. d/e is a ratio between dispersion and electrostatic component. (All presented energies are in kcal/mol).

	**TS1**	**TS1'**	**TS2**	**TS2'**
ΔE_{int} interaction	**-57.06**	-41.58	-49.73	-47.82
ΔE_{els} electrostatic	**-78.48**	-62.87	-69.87	-70.23
ΔE_{xrep} exchange-repulsion	226.78	187.41	207.45	205.01
ΔE_{orb} orbital	**-160.89**	-120.79	-141.79	-136.31
ΔE_{disp} dispersion	-44.47	-45.33	-45.52	-46.30
$\mathrm{d/e} = \Delta E_{disp}/\Delta E_{els}$	0.57	0.72	0.65	0.66

Table S36. Charge distribution of important atoms in TS1, TS1', TS2, and TS2' via NBO analysis. The unit of charge is e. The figure on the left illustrates the annotation of mentioned atoms in this analysis

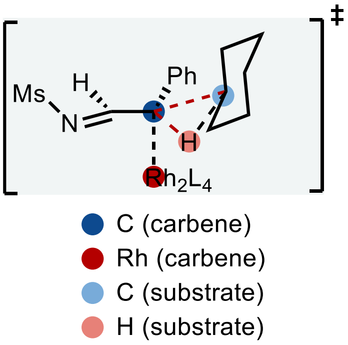

**Atom**	**TS1**	**TS1'**	**TS2**	**TS2'**
**C (carbene)**	-0.281	-0.207	-0.255	-0.232
**Rh (carbene)**	0.530	0.522	0.535	0.522
**C (substrate)**	-0.086	-0.158	-0.102	-0.135
**H (substrate)**	0.288	0.275	0.276	0.276

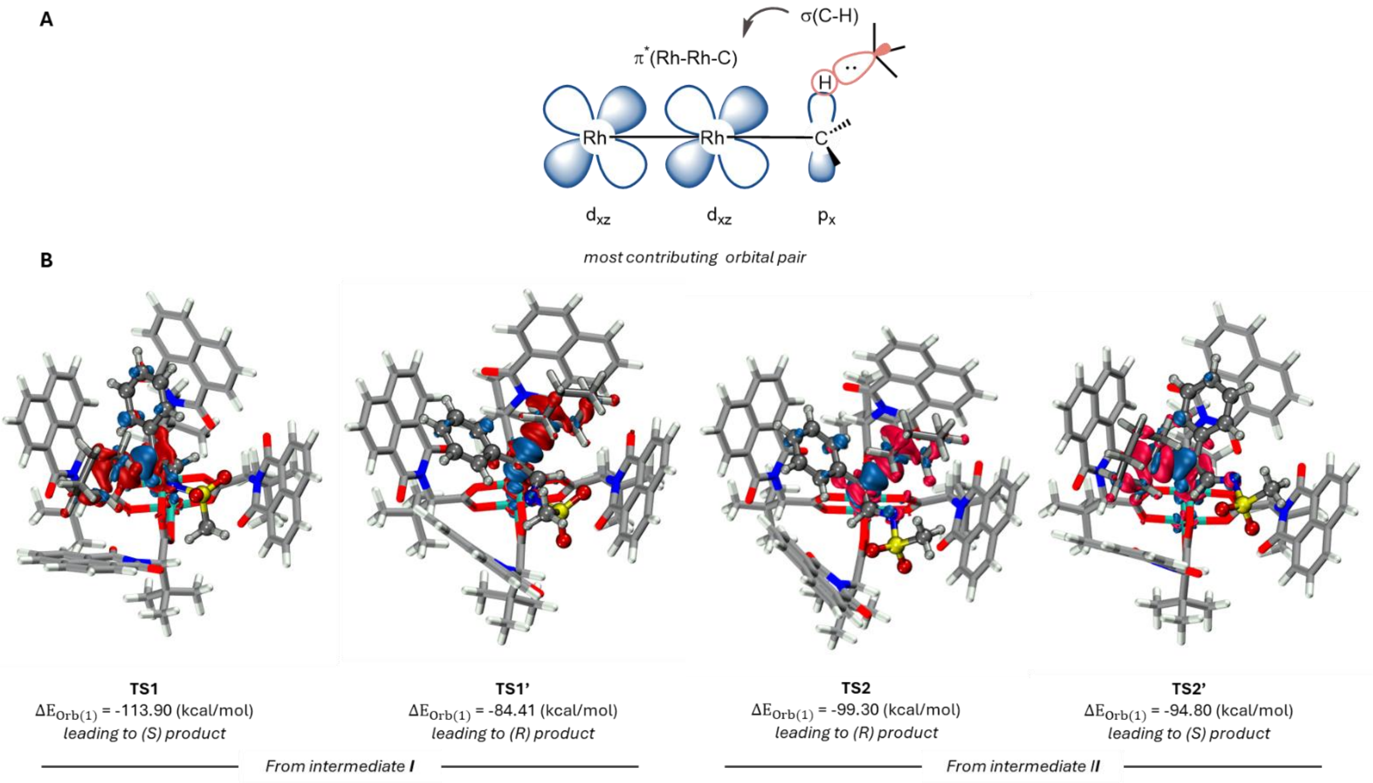

Figure S334. ETS-NOCV analysis. A. Schematic drawing of the most contributing pair of orbitals. **B.** Plot of the deformation density of the main pairwise orbital interactions present in **TS1**, **TS1'**, **TS2**, and **TS2'**. The analysis was performed by the Multiwfn 3.8 program and computed at the B3LYP-D3(BJ)/6-31G(d,p)-Lan2ldz(Rh) level of theory in the gas phase using generated Fock/KS matrix (option -2). (all energies are in kcal/mol). The sign of orbital is color coded of the charge flow is red → blue. (isovalue = 0.0035) $\Delta E_{Orb(1)}$ is the energy contributed by the orbital pair with largest eigenvalue.

12.4.2. Model study with Rh_2(*S*-TPNTTL)$_4$

12.4.2.1. Carbene intermediate

Similar to the model study with Rh_2(*S*-NTTL)$_4$ (**6**), herein, we first studied the geometry of two metal-imino carbene intermediates generated by reaction of Rh_2(*S*-TPNTTL)$_4$ (**7a**) with 1-(methylsulfonyl)-4-phenyl-1H-1,2,3-triazole (**11**). We noticed that the shape of catalyst is significantly distorted from the C_4 symmetry upon the coordination of the carbene to the rhodium center, indicating an induced fit model in which the structure of catalyst changes to maximize weak interaction inside the catalyst pocket. (**Figure S335**) To visualize a non-covalent interaction (NCI) inside the catalyst, we, again, utilized the IGMH method by dividing intermediate **III** and **IV** into catalyst and carbene fragments. We observed the same phenomenon (as in the case with Rh_2(*S*-NTTL)$_4$ catalyst) that diastereomer **III** with the N-Ms group locating on the (*Si*)-face is less thermodynamically favored than diastereomer **IV** with the N-Ms group on (*Re*)-face.

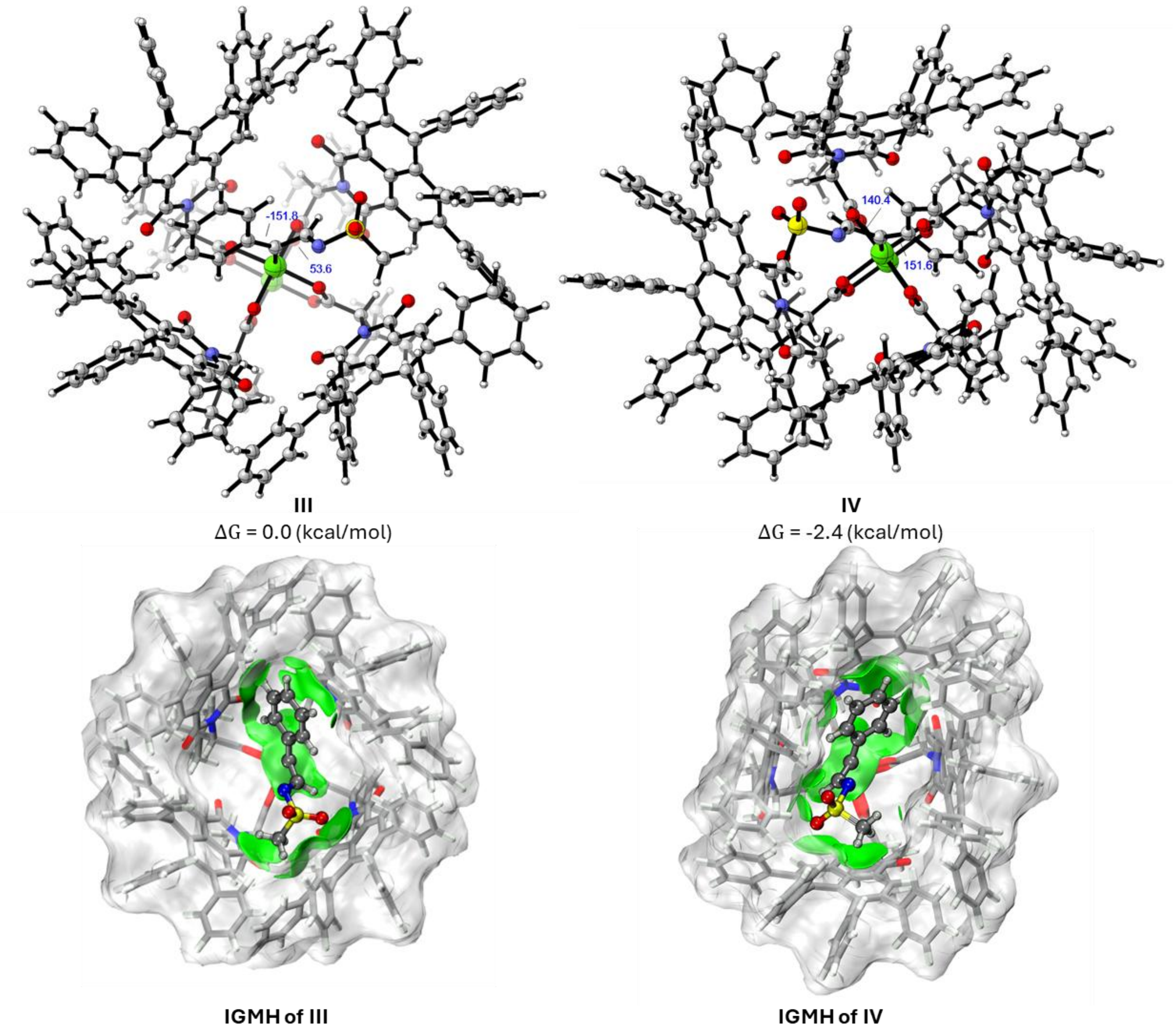

Figure S335. Key geometry features and NCI maps for metal-imine carbene intermediate with Rh_2(*S*-TPNTTL)$_4$. The green surface represents the NCI inside the catalyst pocket. All reported Free energies are in kcal/mol.

12.4.2.2. Analyzing the enantio-determining transition states for the C4-selectivity

As shown in the model studies with $Rh_2(OAc)_4$ and Rh_2(*S*-NTTL)$_4$ catalysts, the approach of substrate to the metal-carbene complex from the N-side is energetically favorable than the approach from the H-side. Therefore, herein, we only conducted analysis of the N-side approach of substrate to the Rh_2(*S*-TPNTTL)$_4$-carbene systems **III** and **IV**. (**Figure S336 A** and **B**) To further support our reason to consider the approach from the N side only, the steric map for **III** and **IV** were studied using SambVca 2.1 program.[47] The steric maps (**Figure S336 C** and **D**) clearly showed that the N face is more accessible than the H face.

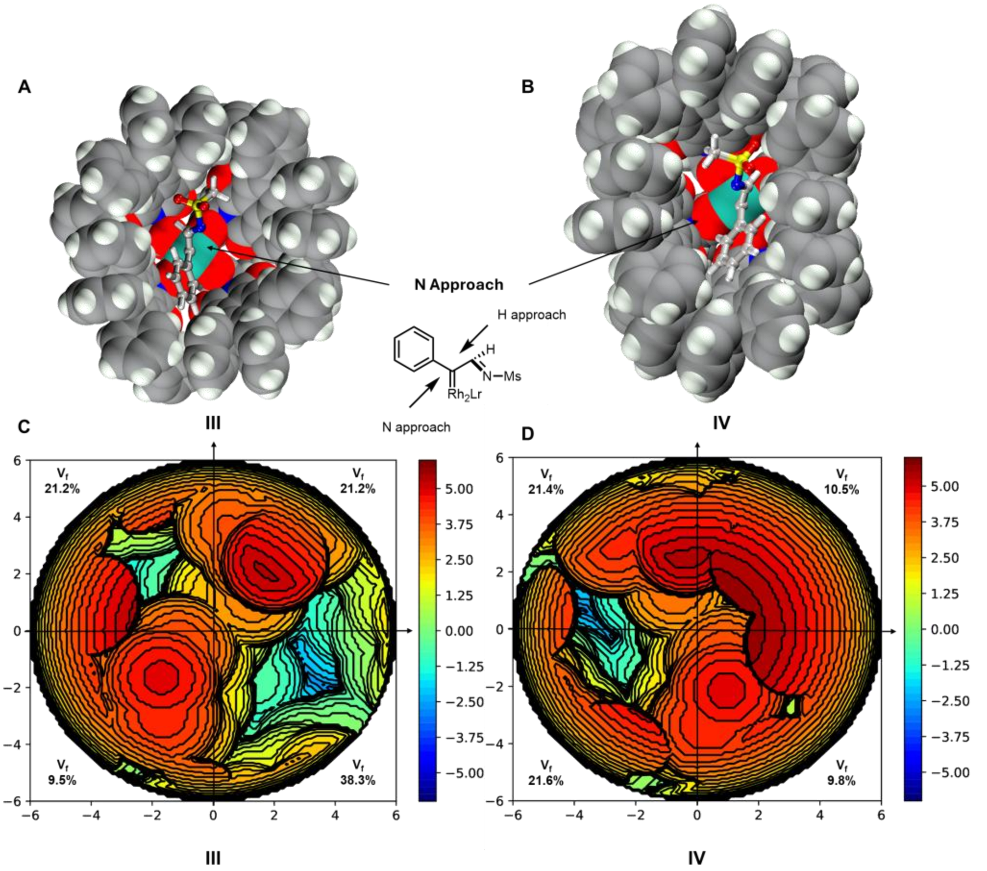

Figure S336. Steric maps of the carbene intermediates III and IV. Here, we used the carbene's carbon as a center of the plot.

The transition states for the C-H functionalization of 4-bromophenyl cyclohexane (**5a**) leading to major and minor enantiomers of the C4 functionalization product with Rh_2(*S*-TPNTTL)$_4$ (**7a**) are shown in **Figure S337**. As the substrate approaches, the catalyst shape changes again to accommodate the substrate inside the pocket and maximize the interaction. The IGMH method in which the transition state was separated into substrate and metal-carbene fragments was used to visualize non-covalent interaction (NCI). Similar to the model study with Rh_2(*S*-NTTL)$_4$, the interaction generally composed by a green surface, indicating for weak interaction. However, substrate **5a** involves stronger interaction via the aryl group of carbene and the extended surface of Rh_2(*S*-TPNTTL)$_4$ (**7a**). Presumably, the reaction of Rh_2(*S*-TPNTTL)$_4$ (**7a**) is also under Curtin-Hammet control. The enantioselectivity of **7a** will be controlled by the energy difference of transition states **TS-C4** and **TS-C4'**. The **TS-C4** leading to the observed major enantiomer is 4.0 kcal/mol more stable than **TS-C4'**. This result is in good agreement with the experiment results (95-99% ee). A closer look at the important bond distances of **TS-C4** and **TS-C4'** revealed that **TS-C4** is a later transition state than **TS-C4'** because the formed C(carbene)-H(substrate) is slightly shorter, 1.17 Å and 1.20 Å, respectively. (**Figure S337**) The distortion-interaction analysis revealed that the 4.0 kcal/mol gap of **TS-C4** and **TS-C4'** stemmed from the interaction components. (**Table S37**) sobEDAw analysis revealed that the main reason for this energy difference is due to orbital interaction. (**Table S38**)

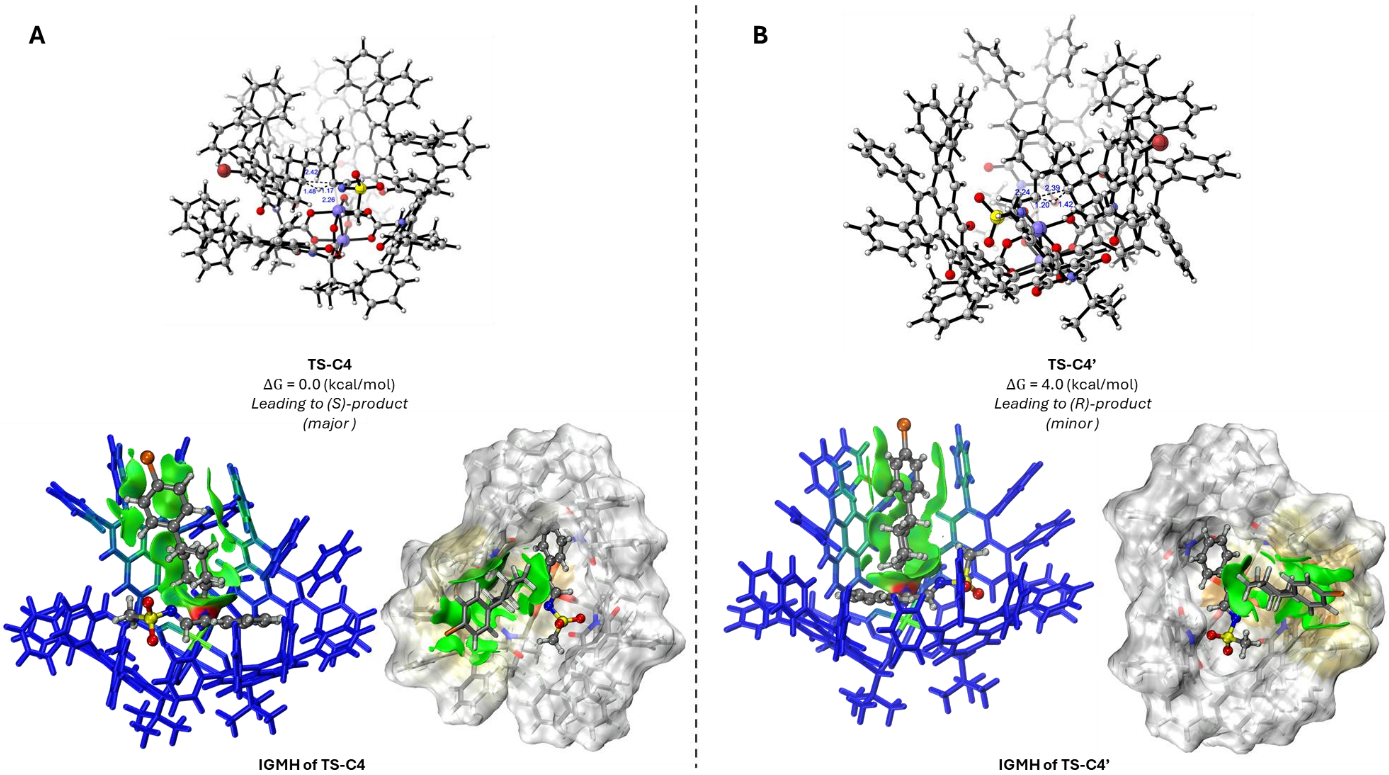

Figure S337. Analysis of C4 selective C-H functionalization transition states. A. The transition state leading to the major C4 enantiomer product B. The transition state leading to the minor C4 enantiomer product. The green surface represents the weak interaction of the substrate with metal-carbene fragment. Atoms in brighter color (green, left) or (yellow, right) contribute to intermolecular interaction. The NCI maps were shown in ball and stick model and a surf model to highlight the key features and the overall shape of the catalyst, respectively. The reported relative energies are free energy (in kcal/mol).

Table S37. Relative (r.t. TS-C4) distortion-interaction analysis of the calculated C-H insertion transition states. The single point energy of metal-carbene (E_{cat}) and substrate (E_{sub}) fragments at their geometries in corresponding transition states were computed at the B3LYP-D3(BJ)/Lan2ldz+6-31G(d,p) level of theory in gas phase. Here, ΔE_{dis}, ΔE_{cat}, and ΔE_{sub} are differences (relative to that in **TS-C4**) in total distortion energy, distortion energy of metal-carbene fragment, and distortion energy of substrate fragment, respectively. The total distortion energy is defined as $\Delta E_{dis} = \Delta E_{sub} + \Delta E_{cat}$. $\Delta E_{int} = \Delta E_{TS} - \Delta E_{dis}$, and is difference in interaction energies. (All presented energies are in kcal/mol).

Structure	E_{TS}	E_{cat}	E_{sub}	ΔE_{TS}	ΔE_{cat}	ΔE_{sub}	ΔE_{dis}	ΔE_{int}
**TS-C4**	-7600238.158	-5693939.767	-1906216.582	0.0	0.0	0.0	0.0	0.0
**TS-C4'**	-7600232.644	-5693939.414	-1906223.740	5.513	0.353	-7.157	-6.8	**12.3**
**TS-C3**	-7600237.784	-1906222.353	-5693942.527	0.374	-2.760	-5.771	-8.5	**8.9**
**TS-C1**	-7600228.956	-5693931.845	-1906229.594	9.202	7.922	-13.01	-5.1	**14.3**

Table S38. Result of the energy-decomposition analysis via the sobEDAw method for TS-C4, TS-C4', TS-C3, and TS-C1. These analyses were performed by Multiwfn 3.8 program and computed at the B3LYP-D3(BJ)/6-31G(d,p)-Lan2ldz(Rh) level of theory in the gas phase. The d/e is a ratio between dispersion and electrostatic component. (All presented energies are in kcal/mol).

Term	TS-C4	TS-C4'	TS-C3	TS-C1
ΔE_{int} interaction	**-81.81**	-69.49	-72.90	-67.52
ΔE_{els} electrostatic	-84.74	-85.35	-86.42	-50.94
ΔE_{xrep} exchange-repulsion	**251.82**	246.65	257.55	163.55
ΔE_{orb} orbital	**-169.98**	-151.38	-164.63	-99.15
ΔE_{disp} dispersion	-78.91	-79.41	-79.40	-80.97
$\mathrm{d/e} = \Delta E_{disp}/\Delta E_{els}$	0.96	0.93	0.92	1.6

12.5. Study on the origin of regio-selectivity with Rh_2(*S*-TPNTTL)$_4$

12.5.1. Analyzing the regio-determining transition states for the C4-selectivity over C3-selectivity

To understand the origin of C4 over C3 functionalization, a comparison between the associated **TS-C4** and **TS-C3** transition states was conducted. Again, the relative distortion-interaction concept was used to deconvolute the energy difference between the **TS-C4** and **TS-C3**. Although **TS-C4** is more distorted than **TS-C3** by 8.5 kcal/mol, the interaction component of **TS-C4** is more prominent than **TS-C3** by 8.9 kcal/mol. (**Table S37)** It is noteworthy that the single point energy (gas phase) of **TS-C4** is only lower than **TS-C3** by 0.37 kcal/mol while the free energy difference (including solvation model) between **TS-C4** and **TS-C3** was 2.2 kcal/mol. This means that the entropic factor significantly contributes to the regioselectivity. This effect can be partially explained by the overall shape of the catalyst. (**Figure S338**) The structure of the catalyst in **TS-C3** is more compact and organized than **TS-C4** in which the two arms of the catalyst in **TS-C3** have to reorganize significantly to strongly interact with the substrate while the reorganization in **TS-C4** is less pronounced. This catalyst re-organization is strongly influenced by the weak interaction inside the catalyst pocket, highlighting the induce-fit mechanism of the current system. Furthermore, to determine which type of interaction is a key factor, we performed the energy-decomposition analyses by the sobEDAw method. (**Table S38**) These analyses demonstrated, again, the dominance of orbital interaction. Additionally, the exchange-repulsion component in **TS-C3** is higher than in **TS-C4**, indicating that there is more repulsive interaction in **TS-C3** than in **TS-C4**. Therefore, the observed C4 functionalization over C3 functionalization is a result of a combination of better orbital interaction and less steric interference (less repulsive interaction).

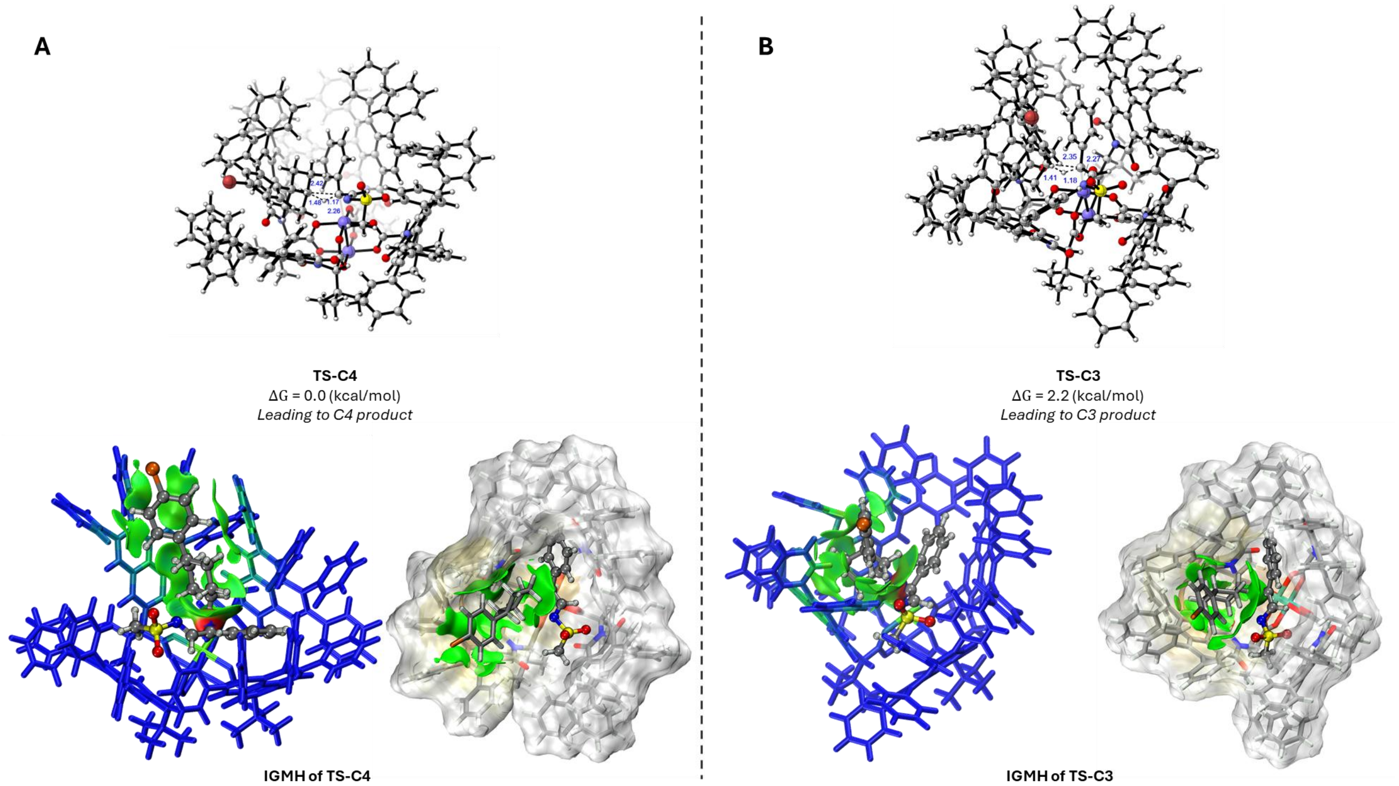

Figure S338. A comparison of transition states TS-C4 and TS-C3. A. The transition state leading to the major C4 regioisomer. **B.** The transition state leading to the minor C3 regioisomer. The green density-surface represents the weak interaction of the substrate with metal-carbene fragments. Atoms in brighter color (green, left) or (yellow, right) contribute to intermolecular interaction. The NCI maps were shown in ball and stick model and a surf model to highlight the key features and the overall shape of the catalyst, respectively. The reported relative energies are free energies in kcal/mol.

12.5.2. Analyses of the C4- over C1-selectivity determining transition states

To understand the origin of the selective C4 functionalization over C1, a comparison of the associated transition states **TS-C4** and **TS-C1** was conducted. Although the DFT calculations predict **TS-C4** to be lower in energy than **TS-C1**, the calculated energy gap between them, 5.8 kcal/mol, seems to be overestimated. Another significant difference is that **TS-C1** is an earlier transition state than **TS-C4** and the C-H insertion has a significant hydride transfer character. The relative distortion-interaction analysis (**Table S37**) showed that the metal-carbene fragment in **TS-C1** is more distorted than in **TS-C4**, but the distortion of substrate in **TS-C1** is less pronounced than **TS-C4**. The total effect is that **TS-C1** is less distorted than **TS-C4**. However, the interaction between the fragments is a dominant factor. The energy-decomposition analyses via the sobEDAw method showed that the interaction is mainly due to electrostatic and orbital interactions. (**Table S38**)

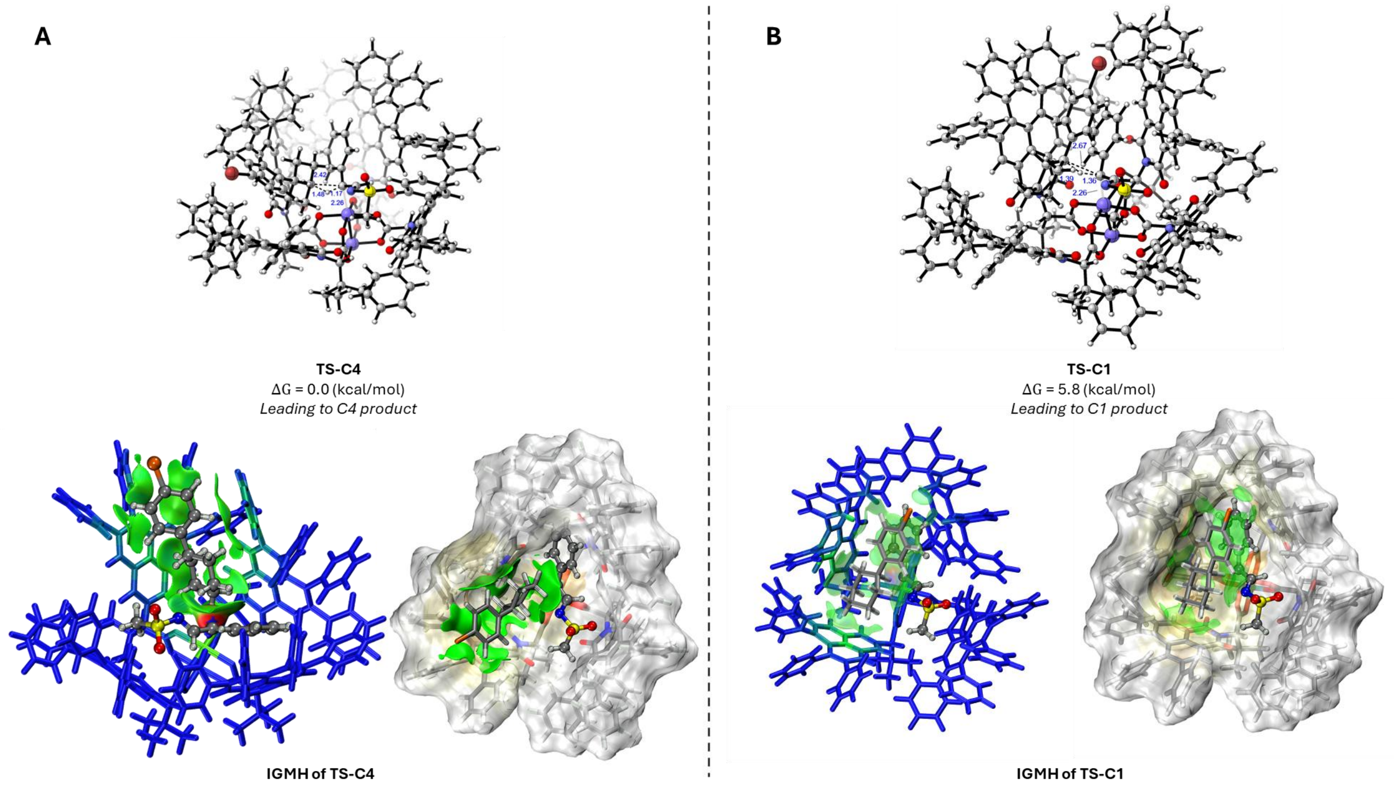

Figure S339. A comparison of the calculated transition states TS-C4 and TS-C1. A. The transition state leading to the major C4 regioisomer. **B.** The transition state leading to the minor C1 regioisomer. The green surface represents the weak interaction of the substrate with metal-carbene fragment. Atoms in brighter color (green, left) or (yellow, right) contribute to intermolecular interaction. The NCI maps were shown in ball and stick model and a surf model to highlight the key features and the overall shape of the catalyst, respectively. The reported relative energies are free energy (kcal/mol).

As the above analysis, the orbital component of interaction energy was a major factor that stablizes the **TS-C4** compared to **TS-C4', TS-C3**, and **TS-C1**. Therefore, ETS-NOCV analysis was conducted to gain a better understanding of this orbital component in these four transition states. (**Figure S340**) Similar to the case of Rh_2(*S*-NTTL)$_4$, the pair of orbitals dominantly contributing to the ΔE_{orb} are the σ (C−H) orbital of the substrate and π*-orbital of the three center RhRhC superelectrophile[53, 54]. This interaction is strongest in **TS-C4** indicating the best orbital overlap. This is the result of the fact that the weak NCI inside the catalyst pocket aligns the substrate in a way that maximizes the orbital overlap leading to the observed enantioselectivity and regioselectivity.

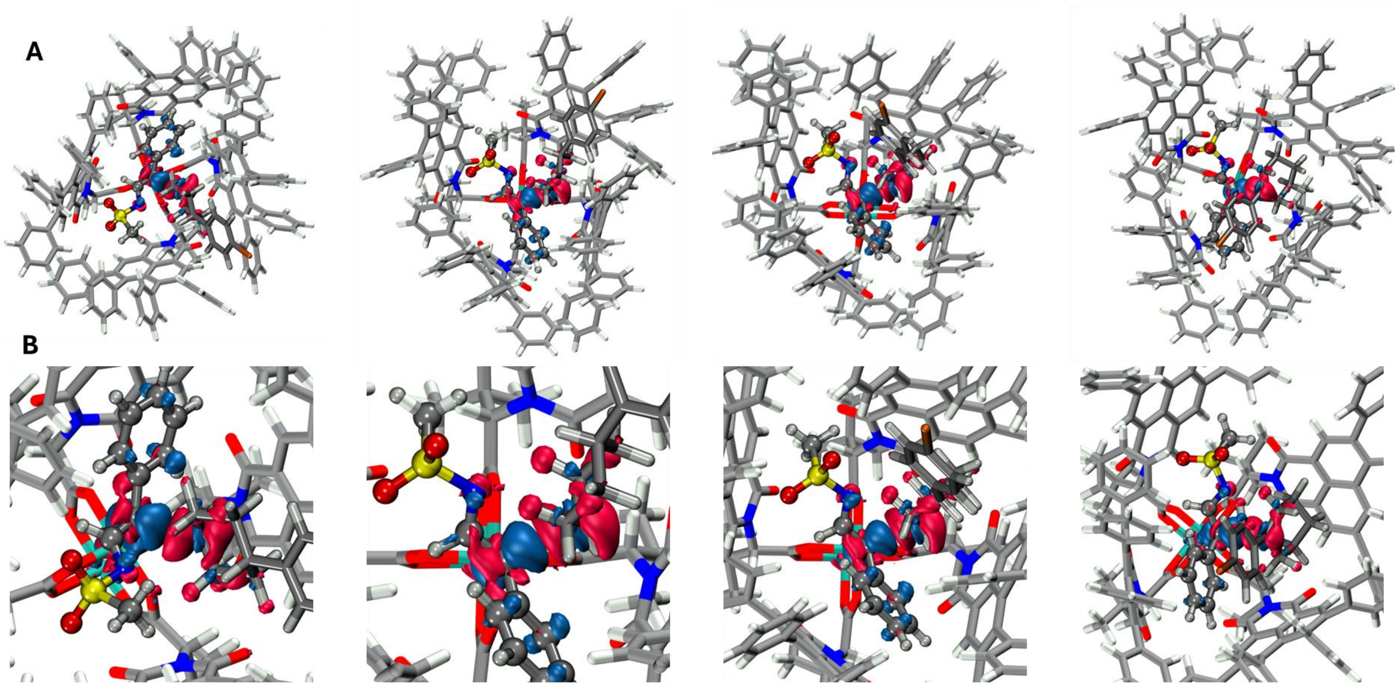

TS-C4’	TS-C4	TS-C3	TS-C1
$\Delta E_{Orb(1)}$ = -100.19 (kcal/mol)	$\Delta E_{Orb(1)}$ = -111.56 (kcal/mol)	$\Delta E_{Orb(1)}$ = -109.49 (kcal/mol)	$\Delta E_{Orb(1)}$ = -60.65 (kcal/mol)
leading to minor C4 product	*leading to major C4 product*	*leading to C3 product*	*leading to C1 product*
From intermediate ***IV***	From intermediate ***III***		

Figure S340. ETS-NOCV analysis of the calculated transition states TS-C4. TS-C4’, TS-C3, and TS-C1. A. Plot of the deformation density of the main pairwise orbital interactions present in these transition states. **B.** Zoom in picture. The analysis was performed by Multiwfn 3.8 program and computed at the B3LYP-D3(BJ)/6-31G(d,p)-Lan2ldz(Rh) level of theory in gas phase using generated Fock/KS matrix (option -2). The sign of orbital is color coded of the charge flow is red →blue. $\Delta E_{Orb(1)}$ is the contributing energy of orbital pair with largest eigenvalue. (isovalue = 0.0035)

12.6. The optimized structures of the catalysts, intermediates, and transition states

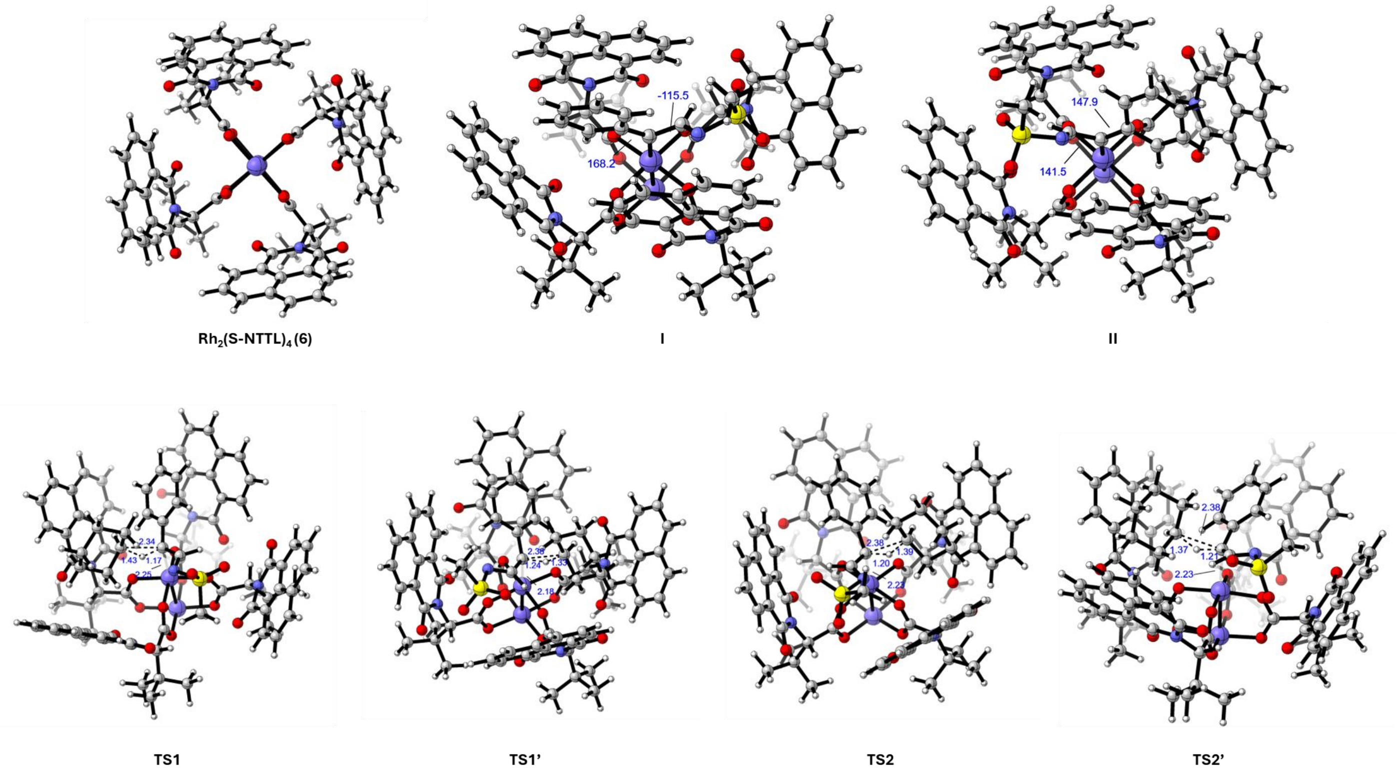

Figure S341. C-H insertion reaction of cyclohexane (5c) with Rh_2(*S*-NTTL)$_4$

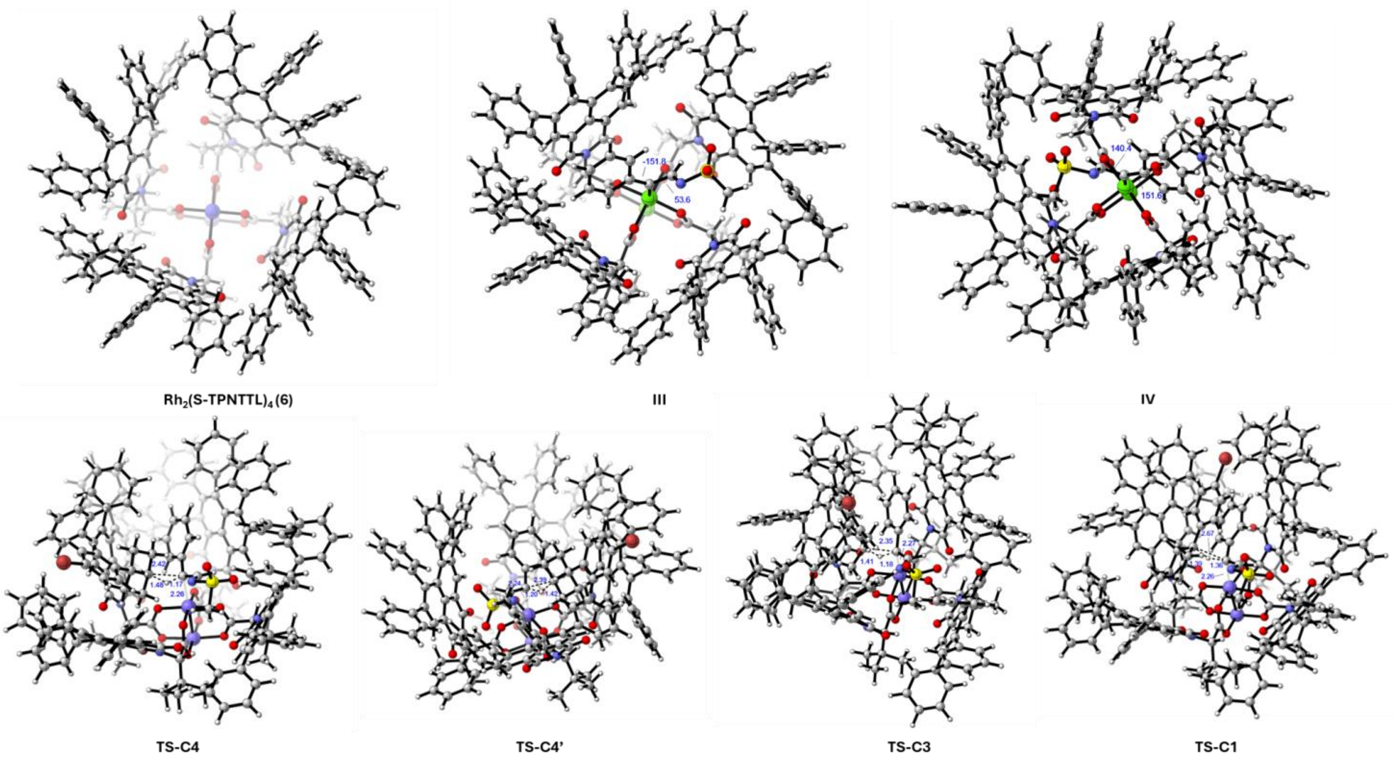

Figure S342. C-H insertion reaction of 4-bromophenyl cyclohexane (5a) with Rh$_2$(*S*-TPNTTL)$_4$

12.7. Energy and Coordination

Table S39. Zero-point correction (ZPE), energies (E), enthalpies (H), and Gibbs free energies (G) (in Hartree) of the structures calculated at the [B3LYP-D3(BJ) +CPCM(CHCl$_3$)] level of theory

Structure	ZPE	E	H	G	Imaginary Frequency
cyclohexane (5c)	0.170539	-235.918926	-235.741743	-235.777149	-
Rh$_2$(OAc)$_4$	0.210452	-1133.217551	-1132.984465	-1133.061137	-
(R/S)-56	0.340270	-1187.676973	-1187.317295	-1187.386417	-
S-1 or S4	0.377250	-2084.928463	-2084.515024	-2084.622814	-
S-2 or S-3	0.359844	-3049.538926	-3049.140539	-3049.256552	-
TS-S1	0.376958	-2084.914447	-2084.501730	-2084.608984	145.85i
TS-S2	0.545958	-2320.844079	-2320.255139	-2320.376135	448.72i
TS-S3	0.546380	-2320.843100	-2320.253909	-2320.375222	326.30i
Rh$_2$(S-NTTL)$_4$ (6)	1.244192	-4424.827076	-4423.499470	-4423.702267	-
I	1.411439	-5376.570226	-5375.062306	-5375.288213	-
II	1.411829	-5376.573203	-5375.064974	-5375.290507	-
TS1	1.581331	-5612.495968	-5610.811328	-5611.053175	561.19i

TS1'	1.581102	-5612.490513	-5610.806342	-5611.046641	292.60i
TS2	1.580681	-5612.491626	-5610.807725	-5611.047358	387.68i
TS2'	1.581200	-5612.489115	-5610.804733	-5611.044706	399.26i
TS3	1.411260	-5376.557701	-5375.050414	-5375.275813	181.65i
5a	0.241818	-3037.806798	-3037.552346	-3037.604527	-
13	0.411366	-3989.564659	-3989.127708	-3989.213698	-
Rh_2(*S*-TPNTTL)$_4$ (7a)	2.536466	-8122.196655	-8119.499731	-8119.868646	-
III	2.704461	-9073.948329	-9071.070326	-9071.46556	-
IV	2.705348	-9073.956718	-9071.078363	-9071.469382	-
TS-C4	2.947194	-12111.785485	-12108.653094	-12109.069516	261.45i
TS-C4'	2.946846	-12111.7799	-12108.64798	-12109.06321	719.31i
TS-C3	2.947618	-12111.78288	-12108.65012	-12109.06597	610.70i
TS-C1'	2.944341	-12111.77102	-12108.64131	-12109.06024	1464.94i

Cartesian coordinates for calculated structure

Cyclohexane (5c)

C	-3.33991100	-1.78775200	-0.00782400
C	-1.80452800	-1.78849800	-0.00842800
C	-1.24531500	-0.35861200	-0.00678400
C	-1.80310900	0.45796600	1.16770600
C	-3.33849200	0.45873300	1.16830600
C	-3.89772800	-0.97114600	1.16663900
H	-0.14961700	-0.37829100	0.02947200
H	-1.44555800	-2.31526500	0.88718000
H	-1.42436500	-2.34511700	-0.87324500
H	-3.69886200	-1.35135500	-0.95078600
H	-3.72126500	-2.81516100	0.02758600
H	-1.44416800	0.02152300	2.11065200
H	-1.42173700	1.48536800	1.13234400
H	-3.71865300	1.01533700	2.03313200
H	-3.69745100	0.98552200	0.27270700
H	-3.62310000	-1.46498700	2.10958800
H	-4.99342500	-0.95143800	1.13032400
H	-1.52000200	0.13522900	-0.94971800

Structure $Rh_2(OAc)_4$

Rh	5.99032900	12.50714700	7.01652000
Rh	8.02169300	12.63337500	5.75874700
O	7.06829600	13.93990400	4.47677500
O	8.58532300	14.21881400	6.95330600
O	8.86623600	11.32066600	7.10621500
O	7.35139200	11.04041000	4.63266100
O	5.14500300	13.81948500	5.66831600
O	6.66072300	14.10036100	8.14334600
O	6.94282400	11.20014500	8.29767300
O	5.42683600	10.92166500	5.82286200
C	5.85503800	14.25406000	4.70419900
C	7.79520000	14.61202600	7.87184200
C	8.15937500	10.89468100	8.07657200
C	6.21161500	10.53636900	4.89656600
C	8.82969000	9.97586900	9.06579100
H	9.29441600	10.58466600	9.84862500
H	8.09481700	9.31840200	9.53156800
H	9.61056100	9.39424900	8.57442200
C	5.21666700	15.24988100	3.76984600
H	5.62127500	15.13620900	2.76318400
H	4.13314900	15.12843200	3.76536700
H	5.45265800	16.25990700	4.12133800
C	8.22784400	15.80540700	8.68481000
H	7.92643100	16.71606900	8.15634400
H	7.74572800	15.79599300	9.66268300
H	9.31352000	15.81644100	8.79065700
C	5.73698500	9.40866600	4.01614300
H	5.21110200	9.83528500	3.15536900
H	6.58642100	8.83235100	3.64751100
H	5.04345100	8.76722300	4.56085800

Structure 56

C	-1.78981100	-2.24001000	0.44009300
C	-0.26213100	-2.22588200	0.31924800
C	0.27516300	-0.81332100	0.04928600
C	-0.19813000	0.19048400	1.11607400
C	-1.73064600	0.16308400	1.23707100
C	-2.25850900	-1.24993200	1.51239200
H	1.36927600	-0.84811200	0.00840500
H	0.17667300	-2.60088000	1.25410300
H	0.06792400	-2.90401000	-0.47573300
H	-2.23232800	-1.96184100	-0.52663900
H	-2.14416100	-3.25125600	0.67036800
H	0.23127000	-0.11477100	2.08000200
H	-2.05430200	0.84933500	2.02504600
H	-2.16177400	0.53624700	0.29674900
H	-1.89847600	-1.58380100	2.49539000
H	-3.35292200	-1.23253200	1.56863600
H	-0.06831500	-0.47309300	-0.93838500
C	0.29088000	1.65030900	0.83603600
H	-0.17759900	1.95522200	-0.10930400
C	1.77252300	1.64812300	0.56923700
H	2.09409700	1.30216800	-0.41830700
N	2.63024500	2.00927500	1.44766300
S	4.27458900	1.84320600	0.97851500
O	4.90053800	0.96481200	1.97716300
O	4.41157000	1.50324100	-0.44982500
C	4.83006900	3.52496000	1.23727300
H	4.60186100	3.80789300	2.26447800
H	5.90675200	3.53381000	1.06172100
H	4.31772900	4.17429800	0.52720900
C	-0.13194600	2.62441900	1.91770400
C	-0.92073300	3.73040400	1.58435300
C	0.23250200	2.43634600	3.25811300
C	-1.33912700	4.63365000	2.56376400
H	-1.21220200	3.88420500	0.54875200
C	-0.18361500	3.33625200	4.23837100
H	0.84984500	1.58903200	3.53458100
C	-0.97090300	4.43847800	3.89509900
H	-1.95264600	5.48533100	2.28553900
H	0.10794200	3.17725900	5.27229300
H	-1.29497700	5.13740000	4.66022800

Structure S-1 or S-4

Rh	6.58332100	13.06654500	6.89762700
Rh	8.49320400	12.61704200	5.42002700
O	7.51068400	13.74806700	3.98842500
O	9.36250200	14.35088700	6.16752500
O	9.31864200	11.53078200	6.97362800
O	7.46240700	10.93458500	4.79434000
O	5.75788300	14.18451100	5.35986600
O	7.60353400	14.74681200	7.54731600
O	7.54342300	11.91894700	8.33339400
O	5.69118500	11.35438600	6.14904700
C	6.38701500	14.29426000	4.26745700
C	8.73403100	15.02908100	7.05256400
C	8.66974000	11.40566400	8.07075500
C	6.30871200	10.67150600	5.28048800
C	9.98353800	12.22145500	4.10743500
C	10.52816700	10.96374700	3.75329200
C	10.21868800	9.80197500	4.51654400
C	11.39496900	10.83629300	2.62802900
C	10.76062300	8.57575400	4.16749400
H	9.57236600	9.90141300	5.37642000
C	11.92038700	9.60322000	2.28418700
H	11.63850900	11.71636200	2.04476900
C	11.60338500	8.47455100	3.05331900
H	10.53186500	7.69309300	4.75485300
H	12.57488200	9.50880600	1.42464500
C	9.32903600	10.55454900	9.12784400
H	10.34458800	10.91550100	9.30876600
H	8.75235200	10.57551900	10.05196400
H	9.40387900	9.52565400	8.76341700
C	5.78430800	15.14517300	3.17751100
H	6.45725600	15.98009100	2.96176300
H	5.68870700	14.55395300	2.26310700
H	4.80952700	15.52479500	3.48191600
C	9.41059400	16.29307700	7.52510000
H	9.33007300	17.05277700	6.74072900
H	8.93157700	16.66602300	8.43007700
H	10.47078500	16.10532700	7.70760200
C	5.62964500	9.43992300	4.73361800
H	5.22494600	9.67113000	3.74297700
H	6.35529600	8.63242000	4.61872700
H	4.81398700	9.13211900	5.38772000
H	12.01848900	7.50893000	2.78128000
C	10.38479400	13.46119200	3.48705600
H	9.59779300	14.04397700	2.99469900
N	11.57589400	13.92769700	3.67443800
S	11.80020000	15.53436400	3.11076200
O	10.55667900	16.09078400	2.54425100
O	13.01212700	15.51809600	2.27925300
C	12.14221700	16.34739000	4.66818200
H	11.27614800	16.20290900	5.31278400
H	13.03262700	15.89537500	5.10419200
H	12.30539000	17.40441500	4.45322400

Structure S-2 or S-3

Rh	6.06221200	12.64062200	7.06243700
Rh	7.99755100	12.62740600	5.54598100
O	6.86101200	13.77212600	4.25393000
O	8.61392000	14.36510700	6.49273400
O	8.96716800	11.47473700	6.96893800
O	7.19230200	10.91360600	4.70670100
O	5.07042400	13.78521200	5.64930800
O	6.83358600	14.37323500	7.89923600
O	7.18499900	11.49022300	8.37317600
O	5.41883100	10.90807200	6.12271800
C	5.67113400	14.10487400	4.57984800
C	7.90813700	14.85852500	7.43720000
C	8.36330900	11.15717800	8.05094400
C	6.10473200	10.42650900	5.17398300
C	9.63692600	12.62158800	4.36303000
C	10.44777400	11.52731800	3.97323600
C	10.12932300	10.20466200	4.39659500
C	11.61449200	11.72656300	3.17526900
C	10.94351100	9.14233000	4.03819100
H	9.24800800	10.05007000	5.00111800
C	12.41518800	10.65677600	2.81914600
H	11.87467200	12.72655500	2.84639400
C	12.08045000	9.36522400	3.25177800
H	10.70078900	8.13727400	4.36561900
H	13.29963100	10.81497100	2.21198900
C	9.15809600	10.32368400	9.02611500
H	9.93184000	10.95038400	9.48060400
H	8.51093300	9.92712300	9.80806700
H	9.65812800	9.51004700	8.49614000
C	4.93866600	14.98477700	3.59637800
H	5.14970900	14.66400200	2.57467700
H	3.86626300	14.96352600	3.79047000
H	5.29991400	16.01239400	3.70775000
C	8.42272700	16.14593700	8.03393800
H	8.38465000	16.93220500	7.27398400
H	7.82121200	16.43952800	8.89371500
H	9.46736500	16.02138200	8.32995800
C	5.61819200	9.15991600	4.51362300
H	5.45540500	9.34526900	3.44836900
H	6.38819600	8.38828200	4.60041400
H	4.69350700	8.81574700	4.97569700
C	10.02324800	13.97829000	3.93456900
O	9.55580600	14.54867700	2.97107800
O	10.91192200	14.51780700	4.80826000
C	11.16329300	15.91414400	4.70593000
H	10.51656100	16.36639900	3.95360800
H	10.98321900	16.35665600	5.68639900
C	12.62475100	16.15411700	4.31611900
Cl	12.88536700	17.93168800	4.27280900
Cl	12.96769700	15.45424300	2.69534900
Cl	13.72733800	15.41034800	5.52405600
H	12.71304700	8.52797100	2.97373000

Structure TS-S1

Rh	6.61539900	13.01412400	6.96051600
Rh	8.46283000	12.59804000	5.38543300
O	7.35194200	13.65601700	3.98984100
O	9.29275400	14.37948000	6.03316500
O	9.41870200	11.60525100	6.92903600
O	7.48366200	10.85583100	4.85702700
O	5.65044400	14.04212500	5.44106600
O	7.59182400	14.77008600	7.48380200
O	7.70758300	11.96173300	8.37530100
O	5.77879300	11.23104600	6.30847200
C	6.21538200	14.14806700	4.31430900
C	8.68041800	15.07583400	6.91725300
C	8.83731800	11.48709100	8.06516700
C	6.37674500	10.55265700	5.42283500
C	9.84758800	12.31809100	3.97053300
C	10.33807100	11.05012000	3.53557400
C	10.24158900	9.94613500	4.43438000
C	10.89140500	10.81256700	2.24123000
C	10.68902500	8.68735200	4.06496000
H	9.84162600	10.11580000	5.42356100
C	11.30573100	9.54343900	1.87535100
H	10.96520300	11.63322700	1.54253300
C	11.21222300	8.48023100	2.78461200
H	10.62521600	7.86238900	4.76657200
H	11.70603400	9.37192100	0.88165200
C	9.58804400	10.69159600	9.10443200
H	10.61845400	11.04839100	9.17084400
H	9.09729000	10.76946300	10.07410100
H	9.61940700	9.64243100	8.79444500
C	5.50353500	14.91443500	3.22680300
H	6.16467500	15.69258300	2.83687900
H	5.27010500	14.23540800	2.40163800
H	4.58511400	15.35823600	3.60972200
C	9.35067700	16.37016300	7.30725400
H	9.58376900	16.94611000	6.40823800
H	8.70924900	16.94890700	7.97114700
H	10.29547000	16.14706600	7.81189700
C	5.73064300	9.27175600	4.95380300
H	5.26499200	9.44816600	3.97879000
H	6.48842700	8.49609600	4.82681800
H	4.96610600	8.94875600	5.66020900
H	11.54761600	7.49010700	2.49123100
C	10.16348100	13.60749300	3.31047800
H	9.81848300	14.49242200	3.84861800
N	10.80157400	13.72897900	2.19997600
S	10.96451500	15.36074400	1.63601100
O	10.55757200	16.33649500	2.66046200
O	10.31331000	15.39033500	0.31981100
C	12.74183900	15.41979500	1.43340100
H	13.20698100	15.32368300	2.41472700
H	13.03955800	14.60658100	0.77217800
H	12.97484700	16.38840900	0.98826300

Structure TS-S2

Rh	6.33877800	12.77904300	6.57819400
Rh	8.29190200	12.53902700	5.14638500
O	7.27053800	13.61744600	3.72401100
O	9.00398000	14.29291000	5.97798900
O	9.18117400	11.47318700	6.68784300
O	7.44625900	10.80450900	4.38570600
O	5.48653200	13.94241100	5.08356300
O	7.20736400	14.49719500	7.34620200
O	7.32499500	11.62203000	7.98614000
O	5.60026200	11.05277200	5.67986100
C	6.13411700	14.12072000	4.00519500
C	8.31912200	14.88875000	6.87347600
C	8.51377600	11.24053000	7.74763700
C	6.28659700	10.45718300	4.79709400
C	10.06561600	12.29748200	3.73994300
C	11.06682500	11.31376500	4.16061300
C	10.66687400	10.01482700	4.53183400
C	12.43544500	11.63711200	4.19848800
C	11.60932900	9.07459700	4.93231300
H	9.61138100	9.76725400	4.51975500
C	13.37360200	10.70035400	4.63061700
H	12.76514500	12.62336200	3.88750300
C	12.96483800	9.41593200	4.99311700
H	11.28744400	8.07646200	5.21324100
H	14.42470500	10.96912900	4.66634800
C	9.22240700	10.46080200	8.82964700
H	9.78600600	11.16115200	9.45524800
H	8.50081900	9.93887600	9.45914000
H	9.92790700	9.75638700	8.38633100
C	5.53633800	15.00968700	2.94378000
H	4.59071900	15.43618200	3.27754300
H	6.25616800	15.79946000	2.71102000
H	5.37845700	14.42651700	2.03165700
C	8.91355000	16.15930200	7.42988600
H	8.12545000	16.82343100	7.78670800
H	9.56157800	15.90482900	8.27539600
H	9.51950500	16.65153600	6.66779700
C	5.68432100	9.24038700	4.13742100
H	5.30149500	9.52875900	3.15270900
H	6.44993700	8.47647800	3.98816700
H	4.86239200	8.84575200	4.73456800
H	13.69690800	8.68287600	5.31739000

C	10.37354900	13.68025700	3.52204800
H	10.95207000	14.15164500	4.32293800
N	9.90595700	14.38537900	2.53651300
S	10.08025000	16.05811000	2.69389400
O	10.98047900	16.43243200	3.80451100
O	8.73037000	16.64954900	2.66757700
C	10.89221500	16.44056200	1.14224400
H	11.87886900	15.97667900	1.14146900
H	10.27788800	16.05309100	0.32975700
H	10.97553000	17.52644900	1.08019800
C	7.94421200	11.27695200	-0.25779400
C	8.10924000	11.90235500	1.15336800
C	9.41601400	11.40064600	1.70484100
C	10.63672100	11.74428500	0.91124200
C	10.45666100	11.12128300	-0.49962300
C	9.15038500	11.59345800	-1.14878200
H	9.24299100	11.90211000	3.01029100
H	8.14322600	12.99100000	1.07853500
H	7.27923000	11.62788200	1.80628600
H	7.82766300	10.19004100	-0.16412200
H	7.01720900	11.66178000	-0.69526600
H	10.71923800	12.83016400	0.81589500
H	11.54109000	11.35792100	1.38816500
H	11.32281400	11.39511200	-1.11040000
H	10.45489000	10.02720700	-0.41778000
H	9.20056400	12.67649700	-1.32073400
H	9.02723600	11.12000400	-2.12888100
H	9.39657200	10.37388100	2.07626700

<u>Structure TS-S3</u>

Rh	6.35040300	12.98628400	6.36244700
Rh	8.39813500	12.63707000	5.08966100
O	7.62716900	13.91013400	3.65086700
O	9.19089800	14.25475100	6.08704600
O	9.03240600	11.40358800	6.62419700
O	7.45654900	11.03551100	4.15696800
O	5.70357200	14.21067800	4.81155400
O	7.27294600	14.61707300	7.24227100
O	7.13220400	11.74091700	7.81871800
O	5.56597200	11.32959600	5.37445500
C	6.46493400	14.41168100	3.81836300
C	8.47197000	14.89453800	6.92411100
C	8.28224300	11.23143500	7.63989700
C	6.26474400	10.72915700	4.50546700
C	10.24766000	12.26780600	3.87192400
C	11.12537400	11.15858800	4.25570000
C	10.55341200	9.89631800	4.51526600
C	12.52084600	11.30678900	4.36024500
C	11.35486100	8.81660100	4.86692100
H	9.47723300	9.78450200	4.44688200
C	13.31594800	10.22521800	4.73774300
H	12.96817800	12.27307400	4.16659800
C	12.73930400	8.97862000	4.98636700
H	10.90137700	7.84936500	5.06051100
H	14.39001200	10.35556000	4.82728700
C	8.83609100	10.35985000	8.74126900
H	9.41453600	10.98749400	9.42749500
H	8.02557100	9.89217000	9.30148200
H	9.50363700	9.60422700	8.32468600
C	5.97877500	15.32810400	2.72233300
H	4.95389100	15.64684800	2.90978400
H	6.63387700	16.20269000	2.67069000
H	6.04231500	14.81152600	1.76089000
C	9.12938000	16.06664300	7.60847900
H	8.38971000	16.84016200	7.82186400
H	9.54727900	15.72384400	8.56141200
H	9.93958100	16.45365500	6.98951400
C	5.66329000	9.52632100	3.81981200
H	5.85093900	9.57955600	2.74493800
H	6.15092900	8.62214200	4.19811100
H	4.59302200	9.46553300	4.01543500
H	13.36311100	8.13717700	5.27198700
C	10.64388800	13.62307600	3.60423900
H	9.97612100	14.19471100	2.95396800
N	11.63484900	14.20673100	4.20829600
S	11.70399600	15.88932800	3.98674700
O	11.75031100	16.51743700	5.31767400
O	10.68972800	16.36462400	3.02304800
C	13.32660600	16.04188700	3.23840300
H	14.05549300	15.57764600	3.90228900
H	13.31066500	15.54758200	2.26689200
H	13.52914700	17.10808500	3.12728600
C	8.30701200	11.73383800	-0.32987900
C	8.42372500	12.24703300	1.13032900
C	9.62489800	11.57397800	1.74053800
C	10.93158800	11.84218700	1.05191300
C	10.80415800	11.32439000	-0.40319800
C	9.60486400	11.96675300	-1.11113900
H	9.45245200	11.93990100	3.03141000
H	8.56101900	13.33236200	1.12358500
H	7.52128300	12.02505800	1.70086600
H	8.06864700	10.66310400	-0.31677700
H	7.46322200	12.24400500	-0.80572100
H	11.13208900	12.91884600	1.02544300
H	11.75958900	11.34856700	1.56659800
H	11.73681000	11.54022000	-0.93385900
H	10.68757500	10.23354300	-0.39010500
H	9.77905500	13.04592000	-1.21249500
H	9.50837900	11.56368000	-2.12496600
H	9.47202000	10.52137300	1.99122700

<u>Structure Rh_2(*S*-NTTL)$_4$ (6)</u>

Rh	16.37417000	5.08897400	4.00215300
Rh	16.57318900	4.39202700	6.28483100
O	17.34103000	6.82239200	4.51479800
O	17.48006100	6.19796000	6.68492700
O	19.50431900	10.09760000	4.32260600
O	15.51269900	8.59443300	5.96604700
O	14.58009000	5.95380300	4.48424000
O	14.72952700	5.24619700	6.62813600
O	11.43827700	8.31329500	4.47741300
O	12.52789000	3.88391700	4.77458800
O	15.41788300	3.31819200	3.61342900
O	15.65481900	2.62633000	5.75447500
O	13.08865300	0.61553600	1.98115500
O	17.36079200	1.12548500	3.52382100
O	18.17760700	4.18482600	3.64106500
O	18.40450600	3.57705500	5.80864100
O	21.15484900	2.39021900	1.81901700
O	20.34257900	5.84276000	4.70233300
N	17.50876000	9.39117900	5.19844600
N	11.92651700	6.08298600	4.66656000
N	15.23093300	0.80222100	2.77085100
N	20.81266100	4.10968100	3.29368300
C	17.61566000	7.02984400	5.73882100
C	18.23268600	8.39999300	6.02486900
H	19.22051900	8.34507000	5.56275400
C	18.27700800	10.16950100	4.31879700
C	17.54725900	11.06678500	3.40083100
C	18.25656600	11.89927800	2.55068800
H	19.33949700	11.89329700	2.59778900
C	17.57730000	12.73359800	1.64076400
H	18.14614100	13.38308800	0.98407800
C	16.19777100	12.72049900	1.57705400
H	15.67498800	13.35592900	0.86832400
C	15.43895700	11.87688100	2.42863600
C	14.02113700	11.81116100	2.38409200
H	13.49104400	12.43462700	1.67007400
C	13.32748400	10.96425000	3.22472600
H	12.24587700	10.90275900	3.17783500
C	14.01627000	10.16151600	4.15621500
H	13.46314700	9.49990800	4.80974100
C	15.39850000	10.20360600	4.22823900
C	16.13212200	11.04848100	3.36098000
C	16.10366800	9.34421700	5.19594600
C	18.47928900	8.81271700	7.50753600
C	19.02771000	10.25257700	7.51944800
H	19.28757700	10.53448500	8.54446800
H	18.28312600	10.96704600	7.15568800
H	19.92174100	10.34699100	6.89757700

C	17.23024800	8.75273300	8.40239700
H	16.78744800	7.75646000	8.40150900
H	16.46843600	9.46317300	8.07657900
H	17.51691600	9.00766100	9.42877500
C	19.56868600	7.87859700	8.07490000
H	19.22221900	6.84560500	8.12407000
H	19.84028900	8.20218900	9.08474200
H	20.47391100	7.90904800	7.45821500
C	14.11897800	5.78456000	5.65720200
C	12.72369100	6.37023000	5.87945500
H	12.88221200	7.44992100	5.83943000
C	11.35976100	7.18196900	4.00265900
C	10.67387800	6.91493000	2.72264900
C	10.04786000	7.95320500	2.05260800
H	10.05259500	8.94079400	2.49936600
C	9.42137900	7.72455800	0.81135700
H	8.93144700	8.54655100	0.30052300
C	9.43672200	6.46609500	0.24273300
H	8.96267000	6.29210600	-0.71873600
C	10.07353400	5.38058600	0.89753700
C	10.13630600	4.07535900	0.34183700
H	9.67557400	3.89646200	-0.62523400
C	10.77886800	3.05374300	1.01150500
H	10.84002100	2.06165200	0.57805700
C	11.37006300	3.28595700	2.26975200
H	11.87326100	2.47872000	2.78553700
C	11.32429500	4.54626000	2.84168100
C	10.68933600	5.61437800	2.16325400
C	11.96332100	4.77587600	4.14990600
C	12.00652600	6.09539700	7.23582900
C	10.60087100	6.72307000	7.17040200
H	10.64718900	7.78684500	6.92291400
H	10.10841900	6.61474900	8.14169700
H	9.97566500	6.22649100	6.42225600
C	11.86629200	4.60617700	7.59253200
H	11.23040400	4.08056100	6.87789900
H	11.40696200	4.52068300	8.58366400
H	12.83618900	4.10863100	7.61331100
C	12.80535500	6.81986600	8.33961900
H	12.91498200	7.88597300	8.11137400
H	13.80092800	6.39075500	8.45843100
H	12.27439000	6.73431500	9.29305000
C	15.33005700	2.45391200	4.54176500
C	14.69832600	1.12873200	4.11218800
H	13.65350600	1.38464000	3.92402900
C	14.29461800	0.61647100	1.74215200
C	14.82328500	0.42531300	0.37672800
C	13.94681300	0.18344600	-0.66832900
H	12.88657100	0.11210900	-0.45451100
C	14.43001800	0.03791600	-1.98391800
H	13.73188600	-0.15519500	-2.79136800
C	15.78110000	0.14847500	-2.24782500
H	16.15224700	0.04666300	-3.26336500
C	16.70719900	0.40138400	-1.20326200
C	18.10168000	0.54706400	-1.42833400
H	18.47952900	0.45891800	-2.44272200
C	18.96206800	0.80602600	-0.38062000
H	20.02440300	0.93370500	-0.55657000
C	18.47127700	0.91313200	0.93627200
H	19.15395700	1.11825300	1.75032600
C	17.11739900	0.77336700	1.19129200
C	16.21353100	0.52875700	0.12943600
C	16.61856900	0.90848800	2.57164000
C	14.67079000	-0.05077200	5.13039300
C	14.06070100	-1.27770200	4.42570500
H	13.07587400	-1.05258000	4.00799600
H	13.95275800	-2.09477100	5.14572700
H	14.70212600	-1.63178900	3.61305000
C	16.04778500	-0.43889400	5.69401400
H	16.71015700	-0.81383100	4.91178800
H	15.91573000	-1.23240400	6.43799100
H	16.53578300	0.41046800	6.17261500
C	13.73283600	0.35562600	6.28641500
H	12.74066500	0.62971500	5.91067100

H	14.13280300	1.20193400	6.84621200
H	13.60974900	-0.48782000	6.97320500
C	18.82656500	3.69737900	4.61976500
C	20.20913200	3.15634700	4.25133600
H	19.99334700	2.27538900	3.64318500
C	21.20730300	3.59692500	2.04822400
C	21.68154200	4.56635800	1.04031800
C	22.13528700	4.11059700	-0.18657800
H	22.15836600	3.04264600	-0.37063500
C	22.55384300	5.02348600	-1.17499400
H	22.91055900	4.65143500	-2.12946600
C	22.50321400	6.38285500	-0.93653300
H	22.81594800	7.08660600	-1.70225500
C	22.03881100	6.88578100	0.30604900
C	21.94556700	8.27421000	0.58897400
H	22.24445800	8.98477200	-0.17606400
C	21.47474800	8.71541800	1.80911500
H	21.38796400	9.77597700	2.01772300
C	21.09415400	7.79188600	2.80344000
H	20.72532800	8.14822600	3.75616000
C	21.17489200	6.43087800	2.56140200
C	21.63467000	5.95554500	1.30970600
C	20.75285100	5.47953800	3.60493900
C	21.14813600	2.66494500	5.39375700
C	22.49193000	2.25024100	4.76419600
H	23.01249400	3.10963400	4.33100900
H	22.35337700	1.50451100	3.97696300
H	23.13905100	1.82254300	5.53618600
C	21.41716400	3.70828700	6.49079300
H	21.94874700	4.57662200	6.09744600
H	22.03888400	3.25218100	7.26927500
H	20.48911400	4.05623300	6.94508400
C	20.50232100	1.41049600	6.01871900
H	19.56002500	1.65083000	6.51255000
H	21.18206000	0.97799000	6.75976400
H	20.30681000	0.64722200	5.25723000

Structure I

Rh	16.40486800	5.08521100	4.03716500
Rh	16.58767300	4.35675500	6.38717100
O	17.35522900	6.82239900	4.58857000
O	17.52923400	6.16507000	6.75300800
O	19.38757600	10.09450600	4.28815700
O	15.60769600	8.53528400	6.31654800
O	14.65572100	5.97765500	4.61018200
O	14.69468200	5.17474200	6.72172600
O	11.62688600	8.47773800	4.68923000
O	12.53311800	4.02200700	4.22386800
O	15.45972900	3.30024200	3.65948800
O	15.62011700	2.61749600	5.81510200
O	13.43454800	0.67792400	1.75105500
O	17.38717100	1.00323700	3.98434600
O	18.16425200	4.10658500	3.70700200
O	18.45689000	3.58602000	5.88899000
O	21.16035900	2.33323400	1.88837400
O	20.25773200	6.00816200	4.44901700
N	17.52211500	9.40016800	5.42282200
N	12.14700800	6.26055800	4.47774500
N	15.38231900	0.63954900	2.95779900
N	20.66754300	4.15651300	3.17886100
C	17.66029700	6.99745300	5.81552800
C	18.32701100	8.34867300	6.08546700
H	19.24654100	8.30705400	5.49873900
C	18.18593800	10.22900900	4.50427800
C	17.37905900	11.25724700	3.81664800
C	17.98963200	12.11380100	2.91561700
H	19.05664400	12.02019800	2.74880100
C	17.23149500	13.08406300	2.22997300
H	17.72377300	13.74885600	1.52839700
C	15.87108000	13.18660900	2.44362400
H	15.28526100	13.92864300	1.90935900
C	15.21203500	12.31974000	3.35300800
C	13.80764800	12.34713900	3.56338100
H	13.21229900	13.07983500	3.02682500
C	13.20394900	11.44469600	4.41611600

H	12.12890900	11.45225400	4.55422400
C	13.97624300	10.48750500	5.10625500
H	13.49706200	9.76502800	5.75333100
C	15.34864000	10.44167300	4.93327100
C	15.98744700	11.34821100	4.05321000
C	16.13258900	9.39427300	5.61594300
C	18.75975500	8.68646200	7.54319700
C	19.37886400	10.09791100	7.54496100
H	19.77089700	10.32221400	8.54175500
H	18.63520600	10.86278900	7.30090700
H	20.19883400	10.17847000	6.82617700
C	17.61604500	8.64842300	8.56992700
H	17.13252700	7.67176700	8.58781800
H	16.85629900	9.40299900	8.35458500
H	18.02426500	8.85766100	9.56493300
C	19.85648000	7.67973600	7.94765600
H	19.46515200	6.66319300	7.99762700
H	20.25584700	7.94741700	8.93113400
H	20.68627600	7.69338800	7.23263600
C	14.12853000	5.73185100	5.74566300
C	12.68361700	6.24565400	5.86208100
H	12.79192100	7.30748000	6.09821000
C	11.83793100	7.51933200	3.94775600
C	11.84301300	7.65685300	2.47539300
C	11.55194700	8.88290900	1.90197500
H	11.24416900	9.69970600	2.54415800
C	11.69205300	9.06922700	0.51256600
H	11.46119200	10.03555700	0.07709500
C	12.15085700	8.04153200	-0.28832400
H	12.28910700	8.19612000	-1.35412500
C	12.45472300	6.77129700	0.26356600
C	12.95272600	5.69029000	-0.51135400
H	13.12206200	5.84316300	-1.57303500
C	13.21711100	4.46528900	0.06889100
H	13.61749600	3.64939400	-0.52331900
C	12.99025800	4.26555500	1.44738800
H	13.19243500	3.30376800	1.90497500
C	12.53172900	5.30885200	2.23539100
C	12.26993500	6.57649400	1.66437600
C	12.38645900	5.11262700	3.68977500
C	11.77291300	5.66769400	6.98683200
C	10.34069800	6.17138400	6.73409700
H	10.31097600	7.26092600	6.64389700
H	9.69391400	5.87350900	7.56538100
H	9.92951900	5.74376100	5.81495000
C	11.75987200	4.13424600	7.08845100
H	11.32155300	3.67809500	6.20150100
H	11.16706700	3.84194300	7.96225400
H	12.76847700	3.73644000	7.20868600
C	12.27548300	6.25699800	8.32184600
H	12.25940100	7.35268800	8.30144700
H	13.29462400	5.93095700	8.53788000
H	11.62642300	5.92791300	9.13937100
C	15.32838100	2.44704500	4.60092600
C	14.65679700	1.15466700	4.13799700
H	13.69646500	1.48657800	3.73806800
C	14.62304100	0.37047100	1.80531700
C	15.31559100	-0.32193700	0.70270400
C	14.59424600	-0.73846100	-0.40203300
H	13.52388300	-0.56798500	-0.42107100
C	15.24722400	-1.36155100	-1.48398500
H	14.66886100	-1.68781100	-2.34198100
C	16.61529000	-1.54839800	-1.45903000
H	17.12084300	-2.01901800	-2.29731000
C	17.38590400	-1.12644300	-0.34527100
C	18.79600000	-1.27323100	-0.28570800
H	19.30889800	-1.73923600	-1.12214700
C	19.50768600	-0.82284600	0.80681100
H	20.58697500	-0.92546300	0.83673600
C	18.84157500	-0.21397600	1.88793600
H	19.39832500	0.15073300	2.74229500
C	17.46565700	-0.06593700	1.86697200
C	16.71637700	-0.51319400	0.75338400
C	16.78331500	0.56483600	3.00949700

C	14.34143900	0.06369200	5.20421900
C	13.64591000	-1.11480900	4.49458200
H	12.75731800	-0.78756300	3.94739600
H	13.33821400	-1.85698400	5.23764800
H	14.31744400	-1.61207400	3.78816100
C	15.58105000	-0.46797800	5.94192600
H	16.26943100	-0.97229500	5.25962600
H	15.26316800	-1.19797900	6.69457800
H	16.12136600	0.33738000	6.43908500
C	13.34857200	0.66763500	6.21753500
H	12.45945000	1.06002500	5.71212900
H	13.80400100	1.47854600	6.78633800
H	13.02170200	-0.10736800	6.91824600
C	18.85344900	3.69243100	4.69789500
C	20.27320100	3.26340400	4.29619500
H	20.13624700	2.29585500	3.80636000
C	20.93031500	3.53772700	1.94538900
C	20.88021500	4.39079300	0.73834900
C	21.13776600	3.83388200	-0.50108800
H	21.43264300	2.79321800	-0.55376600
C	20.98478600	4.60354300	-1.67272600
H	21.20117400	4.15604600	-2.63720300
C	20.53890300	5.90914000	-1.59978100
H	20.39959900	6.49362100	-2.50480700
C	20.25261300	6.50833800	-0.34595700
C	19.75963300	7.83338300	-0.21787200
H	19.58427600	8.41553100	-1.11799300
C	19.50301200	8.37443800	1.02639600
H	19.11718600	9.38435900	1.11785700
C	19.74244000	7.62167300	2.19346100
H	19.55402300	8.04964400	3.16799700
C	20.21029600	6.32194900	2.10088400
C	20.45607600	5.73761200	0.83683200
C	20.39370500	5.52905500	3.32891100
C	21.32337900	3.01731700	5.41883400
C	22.68312300	2.75891800	4.74446900
H	23.04092500	3.65190900	4.22329400
H	22.62005900	1.94555400	4.01679800
H	23.42447400	2.49117300	5.50405500
C	21.47512100	4.16885900	6.42586300
H	21.85610100	5.07099000	5.94669800
H	22.18027600	3.86506500	7.20770500
H	20.52174100	4.41382200	6.89569000
C	20.89693400	1.73779200	6.16879700
H	19.93799100	1.87481500	6.67142800
H	21.64953800	1.48226100	6.92148800
H	20.80849100	0.88828900	5.48194200
C	16.23474300	5.63363400	2.10494600
C	15.89830300	6.90671900	1.56660000
C	16.41666200	4.50590500	1.21571900
C	15.35411900	7.92112800	2.40127000
C	16.06531900	7.18357800	0.17731600
H	15.66527600	3.71684600	1.33429900
N	17.38910800	4.39376400	0.37661500
C	14.95983300	9.13074900	1.85438800
H	15.20487700	7.70984200	3.45025300
C	15.73972000	8.42536200	-0.33755000
H	16.48571000	6.42314700	-0.46585900
S	17.30494600	2.96393200	-0.57799800
C	15.16597500	9.39271700	0.49660200
H	14.47489900	9.87026100	2.47334500
H	15.89778200	8.63652700	-1.38972800
O	15.97625000	2.33166800	-0.44915400
O	18.50695200	2.16122400	-0.33024800
C	17.42364000	3.71305700	-2.20003600
H	14.86929300	10.35310000	0.08717000
H	16.55001700	4.34609900	-2.35600700
H	18.34730700	4.28924700	-2.23970700
H	17.44146800	2.89457200	-2.92135000

Structure II

Rh	16.41300700	4.96749200	4.03473500
Rh	16.57859000	4.28486500	6.40080700
O	17.34863400	6.69539800	4.60747600
O	17.35270800	6.17081400	6.80746500

O	19.47252100	9.92404400	4.36688200
O	15.31077700	8.52505900	5.63824300
O	14.56232500	5.72855500	4.50708900
O	14.68929200	5.08815800	6.67955300
O	11.43061500	7.92424900	4.16524200
O	12.55326000	3.58775100	5.04158700
O	15.51472400	3.16215800	3.71619600
O	15.80073300	2.43546100	5.83634300
O	13.19210800	0.43858400	2.15220100
O	17.59461300	1.18679500	3.12373500
O	18.26389400	4.15195300	3.74097700
O	18.45027900	3.53937600	5.91696000
O	21.25929300	2.67597700	1.88552200
O	20.37036500	5.77926200	5.10434500
N	17.39116100	9.22852800	5.01725800
N	11.87579700	5.74046400	4.69950200
N	15.37979500	0.86225000	2.66966400
N	20.97261000	4.18645000	3.58499900
C	17.51066200	6.96574400	5.84448800
C	18.00717200	8.39676100	6.07897200
H	19.06076100	8.36135500	5.79135000
C	18.27606500	9.83259700	4.10785200
C	17.70809000	10.31689000	2.83247000
C	18.54574400	10.87405300	1.88221200
H	19.60204000	10.96108400	2.10514300
C	18.03004800	11.29990000	0.64133000
H	18.69870900	11.73651600	-0.09278600
C	16.68647400	11.15658000	0.35498100
H	16.29041800	11.48280700	-0.60229400
C	15.79976100	10.58039300	1.30200900
C	14.41371100	10.40097500	1.05131000
H	14.01005400	10.71881400	0.09441100
C	13.59458900	9.82343600	2.00062400
H	12.53979700	9.67282100	1.79911500
C	14.11992500	9.40754300	3.24045100
H	13.47185000	8.94618900	3.97447700
C	15.46638000	9.57270900	3.51871100
C	16.32787100	10.15715000	2.55908200
C	16.00999100	9.08188100	4.79937600
C	17.98910500	8.96989000	7.52641600
C	18.39495900	10.45328800	7.44969600
H	18.48666900	10.86145300	8.46109100
H	17.64381200	11.04263800	6.91539700
H	19.35239600	10.57951500	6.93670000
C	16.63817900	8.85478600	8.25147200
H	16.28630300	7.82256700	8.27415700
H	15.87125400	9.46233200	7.77007200
H	16.75931700	9.20404600	9.28294600
C	19.06469300	8.20930700	8.33049900
H	18.81475500	7.15122800	8.42393100
H	19.14497000	8.63654300	9.33502400
H	20.04719900	8.29093000	7.85193800
C	14.09612400	5.58815500	5.68526700
C	12.69955900	6.19424300	5.84310900
H	12.85702400	7.25720700	5.65148300
C	11.29251300	6.73277100	3.89739900
C	10.50452300	6.28137300	2.73227900
C	9.88645400	7.21908000	1.92207000
H	9.98008300	8.26906300	2.17442500
C	9.15039400	6.80980000	0.79216000
H	8.66950400	7.55578900	0.16863400
C	9.04372000	5.46954600	0.47618000
H	8.48273500	5.15406900	-0.39848000
C	9.67471100	4.48317200	1.27697800
C	9.64694700	3.09946600	0.95783200
H	9.09569400	2.77584700	0.07999400
C	10.32715900	2.18277500	1.73413500
H	10.32399600	1.13067000	1.47331900
C	11.04963900	2.60488100	2.86949300
H	11.60236000	1.88725700	3.46108400
C	11.08472200	3.94402000	3.21601600
C	10.40751200	4.90362800	2.42628500
C	11.88834200	4.37376000	4.37604900
C	11.99969300	6.10450400	7.23029900

C	10.61515600	6.77077300	7.11180500
H	10.69314700	7.79521700	6.73744600
H	10.13825400	6.79879400	8.09638800
H	9.95733900	6.21164200	6.43939800
C	11.81688800	4.67171800	7.75640800
H	11.15959600	4.08641300	7.10964000
H	11.35988800	4.71293700	8.75134200
H	12.77257700	4.15278700	7.83097900
C	12.84461100	6.92258200	8.22903200
H	12.98882100	7.94912300	7.87454300
H	13.82632200	6.47292700	8.38182800
H	12.32969300	6.96929000	9.19388800
C	15.50941300	2.26390300	4.62383700
C	15.04031000	0.89182400	4.11378500
H	13.94939300	0.96316100	4.11135800
C	14.29393400	0.84356100	1.78347400
C	14.51351800	1.39747600	0.43144300
C	13.45722300	1.45280000	-0.46288200
H	12.50965100	1.01024200	-0.17895200
C	13.60672100	2.10855100	-1.70099800
H	12.76879700	2.14910400	-2.38883100
C	14.80266500	2.71638100	-2.02946200
H	14.91089900	3.24004900	-2.97477500
C	15.90857700	2.67310300	-1.14222800
C	17.15132500	3.29865000	-1.42561800
H	17.26084500	3.84307400	-2.35903000
C	18.19920500	3.23332000	-0.52972600
H	19.13010700	3.74776200	-0.73828300
C	18.05388700	2.53222000	0.68465500
H	18.87385000	2.48404200	1.39062600
C	16.85015400	1.92141600	0.99763600
C	15.75856500	1.98695500	0.09954200
C	16.68075900	1.28723500	2.31524000
C	15.36204700	-0.37340400	4.96475600
C	14.98424700	-1.60635000	4.12454200
H	13.95117000	-1.54604100	3.77040400
H	15.09205900	-2.51185300	4.72998100
H	15.63640700	-1.70228500	3.25157000
C	16.82429700	-0.50037000	5.42162000
H	17.49683800	-0.64241900	4.57651200
H	16.91227500	-1.36469800	6.08922800
H	17.14931100	0.38810300	5.96517300
C	14.44890000	-0.32433600	6.20827000
H	13.39216100	-0.27625100	5.92155100
H	14.67683500	0.54283300	6.83105500
H	14.59188200	-1.22828800	6.80856400
C	18.89342100	3.67268500	4.74318200
C	20.27956400	3.14676100	4.37490100
H	20.07373700	2.35611800	3.64991100
C	21.45281000	3.81046600	2.31853000
C	22.20404800	4.82721100	1.56111100
C	22.79082100	4.48384200	0.35495500
H	22.69669000	3.46355300	0.00118600
C	23.48958700	5.44836700	-0.39545900
H	23.94856700	5.16471200	-1.33667600
C	23.58456100	6.74940600	0.05761000
H	24.11605600	7.49630200	-0.52515100
C	22.98887700	7.13718600	1.28430100
C	23.04176500	8.46596200	1.77923100
H	23.56641700	9.21943400	1.19865100
C	22.42948100	8.80118300	2.96899900
H	22.45909800	9.82236500	3.33276000
C	21.74846800	7.82330800	3.71989100
H	21.25681200	8.09062800	4.64597000
C	21.68584000	6.51580200	3.27090500
C	22.29642300	6.15201100	2.04727500
C	20.96295800	5.50714500	4.06359700
C	21.15028100	2.50095500	5.49267600
C	22.48679800	2.06114200	4.86358300
H	23.07274000	2.92100700	4.52509100
H	22.32953700	1.39850800	4.00799800
H	23.08238800	1.52570200	5.60943900
C	21.44558800	3.43289900	6.67909400
H	22.03344900	4.30060700	6.37164600

H	22.02517700	2.88328100	7.42916900
H	20.52490500	3.79081000	7.13990800
C	20.41450000	1.23991400	5.99010900
H	19.47567000	1.49441400	6.48298000
H	21.04704900	0.70295400	6.70427400
H	20.19328600	0.55972800	5.16030100
C	16.21204500	5.77932100	2.20858200
C	15.01280300	5.82178600	1.43983900
C	17.37797400	6.51271000	1.75671500
C	14.09701100	4.73943500	1.49836200
C	14.74171300	6.90417900	0.55690500
H	17.99171500	7.03252900	2.49645700
N	17.71176800	6.42200500	0.50922800
C	13.00570700	4.70864900	0.64589500
H	14.29232700	3.92737500	2.18482500
C	13.60379500	6.89665400	-0.23242400
H	15.41757300	7.74827200	0.52596000
S	19.20893100	7.14172200	0.08757300
C	12.74705000	5.78928100	-0.20406400
H	12.34714000	3.85128000	0.64577400
H	13.38402100	7.74124900	-0.87656600
O	20.05854500	6.07198400	-0.45649300
O	19.72779500	7.99593900	1.16769000
C	18.64584200	8.18015300	-1.25918200
H	11.86450500	5.77332400	-0.83541300
H	18.13402800	7.55184400	-1.98732000
H	17.98435000	8.94562500	-0.85381600
H	19.53672900	8.63475800	-1.69551200

Structure TS1

Rh	16.23325300	5.28347900	3.98518300
Rh	16.79795600	4.61199900	6.26517900
O	16.88338700	7.19419200	4.47082800
O	17.45795800	6.55228000	6.56545900
O	17.96971700	10.95712300	4.20297700
O	15.00249500	8.41949500	6.56826500
O	14.37082200	5.80238700	4.68073700
O	14.88082400	5.19312400	6.80389400
O	11.14606000	7.78694300	4.87607200
O	12.61080400	3.48626200	5.32242700
O	15.53787300	3.35864700	3.71997200
O	16.06991800	2.73349700	5.82917900
O	13.03474000	0.32933800	2.68960100
O	17.46526100	1.36016000	3.16474900
O	18.13504500	4.67000700	3.43145700
O	18.63234600	3.99564800	5.53599500
O	20.59723500	2.86547100	1.07605100
O	20.71721700	5.99030600	4.38432100
N	16.51663700	9.75196900	5.50302700
N	11.77436800	5.60645900	5.19566600
N	15.25865000	0.81649600	2.94724900
N	20.83020500	4.33154100	2.82175700
C	17.27394900	7.41546100	5.66219600
C	17.63439800	8.88260500	5.93958800
H	18.42748400	9.10994600	5.22628800
C	16.82325900	10.79485700	4.61399100
C	15.72054000	11.69159500	4.21669600
C	15.96946800	12.73050900	3.33451700
H	16.97073700	12.84797100	2.93651900
C	14.93651300	13.61668600	2.96986300
H	15.14506100	14.42301400	2.27514200
C	13.66882500	13.46665700	3.49703500
H	12.87416800	14.15322200	3.22098200
C	13.37780500	12.41121300	4.39897900
C	12.08421500	12.19661500	4.94352600
H	11.28511800	12.88334400	4.68116100
C	11.83895400	11.12360500	5.77630300
H	10.84436400	10.95810000	6.17508200
C	12.87225200	10.22244900	6.10303100
H	12.67510900	9.37052900	6.74009600
C	14.14935600	10.41110000	5.60352300
C	14.42366000	11.50700100	4.75089800
C	15.21705300	9.44677300	5.93445600
C	18.20891700	9.24450600	7.34340300
C	18.50539700	10.75687700	7.37241300
H	18.98350000	11.01432300	8.32245500
H	17.58845000	11.34879900	7.29293300
H	19.17342000	11.05309700	6.55925600
C	17.27339100	8.90598200	8.51602100
H	17.00762500	7.84941100	8.51837400
H	16.35194400	9.49163900	8.47781700
H	17.78134700	9.14539400	9.45676900
C	19.54600500	8.49199700	7.50658400
H	19.39361500	7.41356800	7.55546200
H	20.03909300	8.81593800	8.42857500
H	20.22353300	8.70711200	6.67210800
C	14.09510800	5.61325500	5.91282300
C	12.67550800	6.03948200	6.28658100
H	12.70181000	7.12525300	6.18497600
C	11.07948600	6.61570600	4.51046400
C	10.27902100	6.20563600	3.33831800
C	9.56374800	7.15851600	2.63079800
H	9.60043600	8.18994400	2.96195100
C	8.79771900	6.78624600	1.50815500
H	8.24833700	7.54468500	0.96095300
C	8.73664300	5.46465900	1.11159800
H	8.14021400	5.17680000	0.25101700
C	9.44588100	4.46036900	1.81933400
C	9.40373500	3.08734400	1.45919400
H	8.80142500	2.78836200	0.60654100
C	10.11892500	2.14832900	2.17438300
H	10.09279600	1.10287200	1.88797300
C	10.91689700	2.53917500	3.27000200
H	11.50913500	1.80536500	3.80017000
C	10.97581600	3.86897600	3.64942000
C	10.23603200	4.84711400	2.94244000
C	11.84481000	4.27022600	4.77209900
C	12.15257100	5.75716700	7.72608500
C	10.69457900	6.25048800	7.80284100
H	10.61077300	7.30168400	7.51144200
H	10.32620400	6.14801400	8.82822200
H	10.03821900	5.66508000	7.15197200
C	12.20246700	4.27884500	8.14636400
H	11.53336600	3.66284200	7.54281400
H	11.88686700	4.19616400	9.19246800
H	13.21147400	3.87603400	8.05297800
C	12.99803000	6.59336700	8.70908800
H	12.99823400	7.65277200	8.43424100
H	14.03330000	6.25282700	8.72785600
H	12.57968600	6.50367000	9.71683000
C	15.62018000	2.51620900	4.66714300
C	15.01092700	1.14320600	4.36683900
H	13.93432200	1.32574600	4.41026700
C	14.15076000	0.42476400	2.18281700
C	14.38782000	0.12523500	0.75712300
C	13.35463400	-0.37180100	-0.01827500
H	12.40125200	-0.57968800	0.45365300
C	13.53939600	-0.57994900	-1.39985100
H	12.72329500	-0.97529800	-1.99526700
C	14.74421800	-0.26497900	-1.99797400
H	14.87974500	-0.40453700	-3.06641600
C	15.82275600	0.24847300	-1.23281000
C	17.06876300	0.61563400	-1.80531400
H	17.20824800	0.49726400	-2.87590400
C	18.08304100	1.12222800	-1.01833400
H	19.02879100	1.41349200	-1.46274700
C	17.90968800	1.26091500	0.37445100
H	18.71615900	1.64975900	0.98393000
C	16.70570100	0.91209900	0.96260600
C	15.64056900	0.42097500	0.17094500
C	16.54069900	1.04669500	2.42194500
C	15.29813700	-0.01891000	5.37178900
C	14.71089700	-1.32524800	4.80325200
H	13.64329700	-1.22926700	4.59179900
H	14.84834900	-2.12922200	5.53304700
H	15.21646300	-1.62548900	3.88043100
C	16.79257700	-0.23943300	5.65741900
H	17.33225100	-0.53645100	4.75637800
H	16.90057200	-1.03836500	6.39961000

H	17.26023400	0.66392800	6.04773800
C	14.55618600	0.29973000	6.68674400
H	13.48611400	0.45169700	6.50573000
H	14.95436800	1.19661300	7.16140300
H	14.66268200	-0.53986000	7.38139600
C	18.87527800	4.10520700	4.30039800
C	20.10030700	3.40334400	3.70915800
H	19.65743300	2.67049800	3.03281200
C	21.06597900	3.91871800	1.50037000
C	21.93045600	4.78285800	0.67269000
C	22.23541000	4.40540800	-0.62339700
H	21.81617800	3.48403500	-1.01120200
C	23.07959100	5.20650900	-1.41917000
H	23.30750800	4.89835100	-2.43395200
C	23.61695500	6.37263400	-0.91240800
H	24.26954100	6.98867300	-1.52389500
C	23.32509100	6.78956600	0.41235900
C	23.84740400	7.98206000	0.97709700
H	24.50651200	8.60121500	0.37590500
C	23.52665400	8.35243700	2.26824200
H	23.93712100	9.26276600	2.69179300
C	22.65608100	7.55719000	3.04097000
H	22.38099800	7.85042500	4.04781000
C	22.12462600	6.39189400	2.51646100
C	22.46291000	5.97952800	1.20725400
C	21.17804100	5.59373100	3.31635100
C	21.03697700	2.60331700	4.66627100
C	22.16137600	1.97316200	3.81985900
H	22.81323400	2.73436900	3.38054900
H	21.75779200	1.36078400	3.00851500
H	22.78143700	1.33446600	4.45635800
C	21.67345900	3.45274200	5.77853400
H	22.34309000	4.21586200	5.37425400
H	22.26783800	2.80186000	6.42940900
H	20.91170700	3.94870100	6.38035300
C	20.21519200	1.45782300	5.29143300
H	19.45065800	1.84048600	5.96710600
H	20.88102700	0.79753600	5.85689000
H	19.71578800	0.86405600	4.52036100
C	15.06296900	4.84724400	1.02481500
C	16.94589600	6.45589200	1.44181800
C	13.90099500	4.18715500	1.46755300
C	15.54587300	4.56565400	-0.26731800
H	17.70145800	5.73189000	1.12064500
N	17.23115700	7.71811900	1.50012400
C	13.23112000	3.30201300	0.63142500
H	13.54379000	4.36953800	2.47317600
C	14.89085400	3.65056500	-1.08899800
H	16.43055600	5.07303000	-0.63748400
S	18.82457500	8.09094000	1.03949800
C	13.72249600	3.03124100	-0.64897100
H	12.33780600	2.80338100	0.98490800
H	15.28759300	3.43238900	-2.07504000
O	18.73318100	9.20138900	0.07592800
O	19.57104300	6.88034400	0.63076200
C	19.50295100	8.70048600	2.57060200
H	13.20825200	2.32298900	-1.28897700
H	18.88845600	9.52809900	2.92126100
H	19.51774700	7.88344500	3.29205500
H	20.51685700	9.03483000	2.34992200
C	15.70940700	5.86211700	1.87005300
H	12.13593100	10.70181900	0.77544200
C	12.98274700	10.05666200	1.03489500
C	12.76439100	9.43592600	2.41590300
C	13.15486000	8.96684400	-0.02978000
H	13.87505200	10.69577300	1.06041700
C	13.94853600	8.50923700	2.80284800
H	11.83926100	8.85077500	2.42452200
H	12.65787800	10.19849800	3.18644500
C	14.30745400	7.99274200	0.34278500
H	13.37458800	9.39841000	-1.01141300
H	12.21992900	8.40086600	-0.12759000
C	14.02681800	7.47776700	1.71726100
H	14.87654400	9.08628800	2.83464900

H	13.77940300	8.04906300	3.77566300
H	15.25649000	8.53496200	0.33891100
H	14.36297900	7.17617800	-0.38182000
H	13.25531300	6.70744000	1.76361600
H	14.98218700	6.63337700	2.36477600

<u>Structure TS1’</u>

Rh	16.57735600	5.29195900	4.09110800
Rh	16.72357700	4.45536400	6.38468200
O	17.33937600	7.08080500	4.81808800
O	17.45300100	6.30887600	6.94127600
O	19.11105800	10.76576000	5.38691000
O	15.15317400	8.56042900	5.96171000
O	14.65976600	5.91679200	4.54959600
O	14.82114000	5.18196500	6.68816400
O	11.36517900	8.05325400	4.50488500
O	12.71995700	3.70906600	4.86298200
O	15.82340800	3.45809600	3.54599200
O	15.90995400	2.68765200	5.67617600
O	13.54456600	1.10699800	1.61771800
O	17.65782900	1.11387300	3.58245800
O	18.46941200	4.53631900	3.77987700
O	18.60599400	3.73642700	5.89478500
O	21.15490000	2.41762100	1.81936600
O	20.87311900	5.77937200	4.90158700
N	17.15418700	9.60239100	5.62550300
N	11.93919700	5.85260500	4.78784100
N	15.57872300	0.91859500	2.65867400
N	21.07954100	4.08015400	3.39397600
C	17.49347200	7.22693800	6.07241200
C	17.83411000	8.65453200	6.53310900
H	18.89189100	8.78883300	6.28910000
C	17.95029900	10.59822900	5.03310600
C	17.31692300	11.42466200	3.98225300
C	18.04790900	12.42982900	3.37383600
H	19.06739400	12.59983700	3.69908600
C	17.47725000	13.19865700	2.33924500
H	18.06197800	13.98387400	1.87190600
C	16.18903300	12.94542400	1.90829300
H	15.75442200	13.52889400	1.10199100
C	15.41143100	11.92214600	2.50960300
C	14.08272500	11.62285000	2.10460000
H	13.64334500	12.19177700	1.29038000
C	13.36023500	10.63341200	2.73836800
H	12.34603400	10.40768400	2.42718800
C	13.92671000	9.90125100	3.80309800
H	13.34651900	9.13399900	4.29999000
C	15.22143300	10.16083800	4.21439000
C	15.98778400	11.16431800	3.57297800
C	15.80290200	9.38320100	5.32560500
C	17.70813200	8.96791800	8.06235900
C	17.90579500	10.48070500	8.27631400
H	17.88614500	10.69778900	9.34904000
H	17.10521900	11.05911600	7.80534900
H	18.85883500	10.82499500	7.86906100
C	16.36222700	8.56185500	8.68683400
H	16.14933400	7.50558900	8.52586200
H	15.53709400	9.13963400	8.26880000
H	16.40220800	8.75004500	9.76575400
C	18.85457400	8.23128400	8.78618000
H	18.75285300	7.14946900	8.69355800
H	18.84561300	8.49155200	9.84962200
H	19.82888100	8.52520600	8.37918000
C	14.20202500	5.70244700	5.71593900
C	12.78282200	6.22747800	5.94371900
H	12.89327400	7.30969200	5.85848200
C	11.23423600	6.88400600	4.15004100
C	10.31770100	6.49886700	3.05905800
C	9.55125000	7.47019700	2.43831600
H	9.62569700	8.49393100	2.78660000
C	8.70067400	7.12927000	1.36764800
H	8.10289400	7.89998000	0.89302600
C	8.63585400	5.82563900	0.91618300
H	7.99183600	5.56432100	0.08176600
C	9.41508400	4.80746000	1.52424700

C	9.42369500	3.46530400	1.06136300
H	8.79201000	3.19775900	0.21954500
C	10.23278900	2.51890200	1.65758500
H	10.24784900	1.50008000	1.28633800
C	11.05677900	2.86767900	2.74720800
H	11.70710900	2.12945500	3.19738000
C	11.06745900	4.16586100	3.22655600
C	10.25435000	5.15485500	2.62353600
C	11.96986000	4.52532300	4.33735800
C	12.10681600	5.96073900	7.32504000
C	10.68284500	6.55053800	7.29185900
H	10.69273900	7.61118900	7.02671200
H	10.22438500	6.44719600	8.28019800
H	10.04438900	6.02534600	6.57492300
C	12.01245700	4.47266900	7.70278700
H	11.37038200	3.92127300	7.01223800
H	11.57791300	4.38619600	8.70499600
H	12.99513900	4.00122600	7.70475200
C	12.90891400	6.72165700	8.40022200
H	12.99422800	7.78393400	8.14772400
H	13.91472800	6.31672600	8.50784900
H	12.39502300	6.64403800	9.36395500
C	15.64708300	2.57383800	4.44903500
C	14.95133000	1.31534100	3.93680000
H	13.96446400	1.67892700	3.64668700
C	14.74018600	0.83796300	1.53449800
C	15.36178700	0.39912400	0.26817000
C	14.57689200	0.25256300	-0.86368600
H	13.51870200	0.47804800	-0.79638100
C	15.14820400	-0.18726400	-2.07451500
H	14.52083700	-0.29746700	-2.95244000
C	16.49567900	-0.48166400	-2.14471800
H	16.93537000	-0.82401300	-3.07673300
C	17.33143900	-0.33540000	-1.00768400
C	18.72678800	-0.59677000	-1.03766800
H	19.17348600	-0.94632900	-1.96362300
C	19.50742200	-0.39163800	0.08164900
H	20.57536500	-0.57604500	0.04328200
C	18.93089700	0.07706300	1.27956700
H	19.54805000	0.27185500	2.14738400
C	17.56936300	0.31897500	1.34954300
C	16.74878800	0.11847000	0.21315300
C	16.97670300	0.81208700	2.60719000
C	14.70964300	0.13456400	4.92334000
C	14.00274200	-0.99521800	4.14879700
H	13.08112800	-0.64232200	3.67664000
H	13.74590900	-1.80545700	4.83800800
H	14.64616200	-1.41398500	3.36905400
C	15.99207300	-0.42534600	5.56045600
H	16.65068700	-0.87782600	4.81565200
H	15.72154500	-1.20248400	6.28389200
H	16.54881500	0.35636600	6.07778200
C	13.75418400	0.62738900	6.03010700
H	12.84960300	1.07156200	5.60328400
H	14.23104800	1.37887400	6.65941300
H	13.45784800	-0.21890000	6.65837600
C	19.04391400	3.89728900	4.72249000
C	20.32885700	3.18209900	4.29611400
H	19.96741800	2.38860500	3.63765000
C	21.40406600	3.58361500	2.12417700
C	22.03794300	4.52253100	1.17694300
C	22.38575400	4.08405100	-0.09153800
H	22.20363700	3.04869200	-0.35462800
C	22.97110200	4.97089700	-1.01563500
H	23.23267500	4.61514400	-2.00647300
C	23.21868000	6.28363200	-0.66163800
H	23.67441500	6.96661400	-1.37255200
C	22.88541900	6.76382600	0.62954400
C	23.12714700	8.10104400	1.03934500
H	23.59189900	8.78659900	0.33679400
C	22.77064200	8.52785300	2.30053200
H	22.94622700	9.55521700	2.59973400
C	22.15434700	7.64086400	3.20521800
H	21.84958700	7.97646600	4.18895800
C	21.91673100	6.32655700	2.84339400
C	22.27810900	5.86544300	1.55536100
C	21.26180700	5.41515300	3.79688300
C	21.18261800	2.47082200	5.39024300
C	22.44690300	1.89827400	4.72067300
H	23.09297900	2.69338100	4.33626400
H	22.19530200	1.23355900	3.89016000
H	23.02206700	1.32886100	5.45744400
C	21.60921400	3.38251700	6.55224900
H	22.26511600	4.18663400	6.21331300
H	22.15649800	2.78574900	7.29068200
H	20.74343000	3.83170900	7.03958800
C	20.35464500	1.28874800	5.93726000
H	19.47412900	1.63843600	6.47691600
H	20.97075600	0.69541800	6.62080700
H	20.01763200	0.63426400	5.12661500
C	16.37414800	6.02387500	2.04480300
C	15.01875200	6.28828700	1.54373400
C	17.50684300	6.88828300	1.81557600
H	16.74282000	4.92151600	1.61782500
C	14.11928000	5.20993600	1.41387800
C	14.59860500	7.57347400	1.15740400
H	18.45741100	6.36816500	1.67395000
N	17.47217600	8.18193200	1.81763900
C	17.07844800	4.41612900	0.43368500
C	12.87229200	5.39977600	0.83400800
H	14.41202300	4.22740900	1.76668400
C	13.31554100	7.76504700	0.64359000
H	15.27118700	8.41243000	1.27450700
S	18.99618300	8.88914700	1.50517800
C	18.42934000	3.81951400	0.70889600
C	17.01384100	5.33680300	-0.75440400
H	16.25350300	3.70077500	0.47098000
C	12.45933300	6.68098700	0.45321900
H	12.21390100	4.55068800	0.69410900
H	12.99700200	8.76684200	0.37387300
O	19.54255200	9.45049200	2.74991100
O	19.84058900	7.97175100	0.71276400
C	18.45741500	10.22661900	0.44641600
C	18.86126700	3.03847700	-0.55741500
H	19.16366200	4.60984800	0.89090000
H	18.41017000	3.17219100	1.58370900
C	17.44922900	4.53523000	-2.00566300
H	17.71065200	6.17209800	-0.62511800
H	16.00842700	5.74343600	-0.88394700
H	11.47160300	6.82940200	0.02919100
H	17.72724400	10.82534000	0.98721600
H	18.02435500	9.79708800	-0.45674400
H	19.34179700	10.81942800	0.20912200
C	18.84205300	3.92827600	-1.80056600
H	18.18146500	2.19574100	-0.70633400
H	19.85097100	2.62096100	-0.36345300
H	17.42818800	5.20394500	-2.87206600
H	16.72325800	3.73486600	-2.19788300
H	19.13051100	3.34385300	-2.68139000
H	19.58307600	4.73134600	-1.69257100

Structure TS2

Rh	16.58290900	5.38907900	4.00140900
Rh	16.75923200	4.56272800	6.29561300
O	17.34201600	7.18778000	4.70849400
O	17.48707300	6.42519400	6.83216700
O	19.12264300	10.85537200	5.20074800
O	15.16154300	8.67091300	5.84821200
O	14.66931900	5.99651200	4.47016600
O	14.85345200	5.28903100	6.61577800
O	11.38537100	8.05986100	4.31994800
O	12.75055200	3.74757500	4.90788800
O	15.85566600	3.54320400	3.48581600
O	15.95047500	2.77604600	5.61525300
O	13.48456000	1.35941200	1.59473600
O	17.65828400	1.18203900	3.41469200
O	18.48056400	4.63445700	3.66873400
O	18.63162600	3.84499100	5.78656700
O	21.09900500	2.35162900	1.71156600

O	20.96174000	5.79591500	4.71189900
N	17.16496400	9.70187200	5.48215800
N	11.94928100	5.87780800	4.73656800
N	15.54788500	1.07004800	2.55162700
N	21.09720900	4.04631200	3.25426800
C	17.51272500	7.34044200	5.95973300
C	17.85049600	8.77327900	6.40494800
H	18.90692000	8.90854400	6.15518200
C	17.96028400	10.67721700	4.85333900
C	17.33085500	11.45509600	3.76746100
C	18.05370800	12.44812100	3.13175400
H	19.05642400	12.66613700	3.48104200
C	17.50156100	13.14094900	2.03629500
H	18.08029400	13.91860300	1.54900600
C	16.24208400	12.82192300	1.57086800
H	15.82316700	13.34377300	0.71533100
C	15.47087400	11.81129100	2.20041800
C	14.17162900	11.44482500	1.76017400
H	13.75039200	11.95077600	0.89617900
C	13.45307600	10.46567700	2.41691000
H	12.46254300	10.18616800	2.07447600
C	13.99405200	9.81747000	3.54601200
H	13.41932500	9.06041100	4.06414900
C	15.26201500	10.14666500	3.99517800
C	16.02499100	11.13513100	3.32716700
C	15.81914700	9.45613000	5.17358100
C	17.73069100	9.10678200	7.92931800
C	17.91406100	10.62471100	8.11616800
H	17.90671900	10.86036100	9.18515200
H	17.10074400	11.18525700	7.64548700
H	18.85757900	10.97217900	7.68949300
C	16.39466900	8.69463900	8.56959700
H	16.20058200	7.63073400	8.43641500
H	15.55796500	9.24576100	8.13938800
H	16.43551700	8.91013000	9.64337600
C	18.88945300	8.39148100	8.65472600
H	18.80066600	7.30753800	8.57115300
H	18.88155000	8.65978800	9.71617700
H	19.85834900	8.69396600	8.24119200
C	14.22294900	5.79482600	5.64357100
C	12.80173700	6.31693200	5.86386100
H	12.90070500	7.39442300	5.72471600
C	11.24649800	6.87171700	4.03895200
C	10.32208500	6.42369800	2.97841200
C	9.56643000	7.35933500	2.29282800
H	9.66298600	8.40502900	2.56126500
C	8.69457700	6.95364000	1.26270600
H	8.10678600	7.69752800	0.73568300
C	8.59167600	5.61946700	0.92046300
H	7.92609400	5.30690500	0.12132200
C	9.35840600	4.63568700	1.59696800
C	9.32053600	3.25889100	1.25234100
H	8.66382200	2.93818100	0.44910200
C	10.11185200	2.34361900	1.91634500
H	10.08586400	1.29573400	1.63840900
C	10.97210700	2.76236800	2.95163800
H	11.61046400	2.04914800	3.45564400
C	11.03218800	4.09653800	3.31302000
C	10.22795300	5.05183100	2.64761700
C	11.97502400	4.52664600	4.36294300
C	12.14272900	6.11381200	7.26252800
C	10.72280500	6.71237200	7.21899000
H	10.73773600	7.76016600	6.90638700
H	10.27411200	6.65720400	8.21564100
H	10.07301600	6.16080600	6.53268000
C	12.04448000	4.64458300	7.70624500
H	11.38855600	4.06740000	7.05046000
H	11.62435200	4.60479800	8.71749800
H	13.02461700	4.16785400	7.71401600
C	12.96554800	6.91420900	8.29225500
H	13.06739600	7.96087600	7.98690900
H	13.96582100	6.49867600	8.41106900
H	12.45948500	6.89187300	9.26296000
C	15.68018300	2.66118500	4.38932400
C	14.97301300	1.41172400	3.87059600
H	13.97337900	1.77957200	3.63258700
C	14.67472000	1.09031600	1.45145500
C	15.25231200	0.76134400	0.13212900
C	14.43432000	0.73638100	-0.98539100
H	13.38440900	0.97932100	-0.86793200
C	14.96228800	0.39397200	-2.24640100
H	14.31023800	0.38102100	-3.11308400
C	16.29754700	0.06665700	-2.37944700
H	16.70196600	-0.20638500	-3.34955700
C	17.16588300	0.08717800	-1.25766200
C	18.54905500	-0.22254500	-1.34605600
H	18.95961500	-0.51221200	-2.30869600
C	19.36355300	-0.13617000	-0.23554700
H	20.42235800	-0.35588800	-0.31689100
C	18.83547100	0.25978800	1.01030000
H	19.48150500	0.36832600	1.87155400
C	17.48589100	0.54153100	1.13674700
C	16.62991500	0.45619800	0.01213200
C	16.94212100	0.95495800	2.44451100
C	14.77188600	0.19930200	4.82582600
C	14.06097100	-0.91406500	4.03184900
H	13.12832000	-0.55511300	3.58581300
H	13.82164200	-1.74633900	4.70083000
H	14.69392400	-1.30322400	3.22864700
C	16.07355500	-0.36173700	5.42149200
H	16.72214000	-0.78710100	4.65292700
H	15.82647300	-1.15843700	6.13198900
H	16.63209900	0.41372200	5.94691400
C	13.83762500	0.64824600	5.96893200
H	12.91403600	1.08671900	5.57866600
H	14.31890900	1.39344000	6.60259400
H	13.57334500	-0.21761200	6.58468100
C	19.05116200	3.98346800	4.60258700
C	20.29742500	3.20544000	4.16910900
H	19.88967000	2.42300800	3.52449200
C	21.43935600	3.49419400	2.01279700
C	22.22371700	4.34108900	1.09064000
C	22.61966700	3.83332800	-0.13606100
H	22.35284100	2.81393000	-0.38932500
C	23.36417700	4.62794400	-1.03038400
H	23.66134200	4.21782500	-1.98956000
C	23.72215600	5.91670200	-0.68656200
H	24.30113000	6.52764700	-1.37267500
C	23.34556900	6.46546100	0.56612600
C	23.69278500	7.78304800	0.96685100
H	24.27962900	8.39790200	0.29097800
C	23.28304400	8.27995500	2.18682100
H	23.54276800	9.29168100	2.47831800
C	22.51252300	7.48535000	3.06030200
H	22.17095500	7.87634400	4.00992700
C	22.16793300	6.19238400	2.70763100
C	22.57628700	5.66153500	1.46022200
C	21.37117900	5.36964300	3.63866300
C	21.12720100	2.46832600	5.26796200
C	22.36109100	1.82710400	4.60311000
H	23.04394300	2.58358900	4.20441700
H	22.07677700	1.15968200	3.78559900
H	22.91133800	1.24458500	5.34849400
C	21.60448900	3.37790300	6.41223500
H	22.29305000	4.14678900	6.05583100
H	22.13279100	2.76904800	7.15453900
H	20.76438600	3.87181000	6.90089900
C	20.25252600	1.33124200	5.83748100
H	19.39182200	1.72567000	6.37776100
H	20.84786300	0.72180200	6.52531500
H	19.88276800	0.68127300	5.03784700
C	16.30043000	6.12226100	1.91929900
C	14.91860800	6.16571900	1.41092500
C	17.12128100	7.30111600	1.85143500
H	16.91154000	5.17245900	1.52510100
C	14.14803800	4.98647500	1.36472400
C	14.35549600	7.35390600	0.90712700
H	16.63291000	8.22134100	2.18722300

N	18.36204300	7.29340700	1.47704100
C	17.19611300	4.66311000	0.26874700
C	12.88295300	4.98809600	0.79005200
H	14.54892900	4.07694300	1.79292700
C	13.06146000	7.36166100	0.38871000
H	14.92061500	8.27847300	0.91740400
S	19.13842100	8.79839900	1.52148000
C	18.55781500	4.09530800	0.53800900
C	17.09282200	5.59713100	-0.89665900
H	16.37374100	3.94801000	0.33857500
C	12.32889100	6.17760900	0.30721500
H	12.32277900	4.06118100	0.73987000
H	12.63702000	8.29333400	0.02790400
O	18.20797100	9.88001300	1.14465700
O	19.89438600	8.93367100	2.77779000
C	20.29099400	8.53676600	0.17785500
C	18.98798800	3.33414200	-0.74184700
H	19.25701100	4.91041600	0.73690900
H	18.54781200	3.42935300	1.39922800
C	17.54972600	4.82642700	-2.16381700
H	17.76184300	6.44776900	-0.74363600
H	16.07180200	5.96623200	-1.01946700
H	11.32923100	6.18231400	-0.11395400
H	19.72529300	8.43271000	-0.74791200
H	20.86459000	7.63617700	0.38777300
H	20.94469500	9.40828000	0.13919200
C	18.95368200	4.24281400	-1.97067100
H	18.31405500	2.48835300	-0.89974900
H	19.98071100	2.91736600	-0.56227000
H	17.51406600	5.51310500	-3.01564500
H	16.84146500	4.01483400	-2.37368000
H	19.68036800	5.05714800	-1.84955300
H	19.25068900	3.67872900	-2.86199200

Structure TS2’

Rh	16.22548000	5.20592600	4.02876500
Rh	16.75018600	4.49125700	6.30508700
O	16.99578500	7.06288100	4.51100100
O	17.54152200	6.38411100	6.60159100
O	18.48033900	10.68912800	4.20098500
O	15.23926800	8.44904200	6.51288000
O	14.39671700	5.84050500	4.72389500
O	14.87064300	5.19212600	6.84168400
O	11.12883800	7.96766000	5.09240800
O	12.55585000	3.62657600	5.25671200
O	15.42980300	3.31964100	3.74565600
O	15.91862000	2.66012100	5.85477200
O	12.96966900	0.35300700	2.53672500
O	17.37568500	1.29047300	3.30954200
O	18.09760100	4.51417100	3.49600100
O	18.54654600	3.77338300	5.59161500
O	20.51848100	2.91036100	1.10071300
O	20.75451100	5.72813800	4.67569500
N	16.88666700	9.62914000	5.46151000
N	11.79488800	5.77942800	5.22247200
N	15.18667900	0.75034700	2.95913200
N	20.79052900	4.23160200	2.95469500
C	17.42644800	7.25414100	5.69474500
C	17.91844700	8.68388200	5.94545200
H	18.74056400	8.81744100	5.23807300
C	17.30817700	10.61493100	4.54741700
C	16.28086900	11.55861300	4.05860800
C	16.63539600	12.54027700	3.14905300
H	17.65814400	12.57354400	2.79178300
C	15.68036200	13.47695300	2.70440300
H	15.97225000	14.23811700	1.98884500
C	14.38520800	13.43504700	3.18083300
H	13.65225400	14.16287800	2.84568700
C	13.98551700	12.43941800	4.10949600
C	12.66278200	12.34341400	4.61699400
H	11.92551300	13.07403100	4.29800500
C	12.31216200	11.32952700	5.48547500
H	11.29544800	11.25355200	5.85465100
C	13.26518000	10.37139700	5.88667400
H	12.98458200	9.56376300	6.55040400
C	14.56900300	10.44740000	5.42741900
C	14.95155600	11.48053700	4.53844700
C	15.55547100	9.43002100	5.84639600
C	18.48999000	9.02727700	7.35428400
C	18.90962500	10.51028000	7.36303800
H	19.38154900	10.74754400	8.32158600
H	18.04683100	11.17308900	7.24564900
H	19.62048800	10.73264500	6.56312000
C	17.49869900	8.79189700	8.50646300
H	17.14560700	7.76108300	8.52125200
H	16.63020200	9.45030900	8.43006300
H	17.99900800	9.00802800	9.45697300
C	19.75487000	8.17082400	7.56938800
H	19.51189100	7.11024800	7.63697300
H	20.25013000	8.47483000	8.49718600
H	20.46743400	8.30737900	6.74814600
C	14.10767400	5.65568100	5.95350200
C	12.70561800	6.13599000	6.33486300
H	12.77766900	7.22339000	6.27265200
C	11.08077800	6.83041300	4.62871000
C	10.30232700	6.51622000	3.41233200
C	9.54767300	7.50816400	2.80691200
H	9.50857100	8.48851100	3.26746600
C	8.85295100	7.24469600	1.60956000
H	8.27085300	8.03323700	1.14522300
C	8.91523300	5.99368500	1.02705800
H	8.38536800	5.79224100	0.10092300
C	9.66381500	4.94898900	1.62790300
C	9.74583200	3.64391600	1.07521900
H	9.22003900	3.43432900	0.14854900
C	10.47848600	2.65739800	1.70377800
H	10.54144600	1.66141700	1.27928400
C	11.16495500	2.93081100	2.90428000
H	11.75065500	2.15382900	3.37797700
C	11.11255300	4.19561900	3.46589900
C	10.36172300	5.22144900	2.84253000
C	11.86553000	4.47471600	4.70351300
C	12.17243900	5.82083400	7.76413600
C	10.72959000	6.35164300	7.86633100
H	10.67591800	7.41700100	7.62653400
H	10.36007400	6.20792200	8.88637200
H	10.05642100	5.81633200	7.18979700
C	12.17817400	4.32651200	8.12660500
H	11.49448400	3.75649200	7.49465000
H	11.85441600	4.21191500	9.16714400
H	13.17486700	3.89719300	8.02147500
C	13.04362900	6.59904200	8.77249900
H	13.07934200	7.66551700	8.52543000
H	14.06599200	6.22127200	8.78694400
H	12.61845600	6.49869600	9.77633300
C	15.48139000	2.46567800	4.68431100
C	14.85158200	1.10833400	4.35433400
H	13.78034800	1.31866700	4.32552500
C	14.12950600	0.35897300	2.12719400
C	14.47272200	-0.05828500	0.75286000
C	13.48621600	-0.56756700	-0.07390400
H	12.48609900	-0.69635300	0.32382000
C	13.77977900	-0.89551600	-1.41300300
H	12.99904900	-1.30084900	-2.04775800
C	15.04767400	-0.68799100	-1.92072100
H	15.26847900	-0.92139300	-2.95812200
C	16.07844100	-0.15506400	-1.10454300
C	17.37918900	0.13113800	-1.59499400
H	17.60499900	-0.08155700	-2.63583700
C	18.33550600	0.68662600	-0.76962900
H	19.32153500	0.92475900	-1.15376900
C	18.04770100	0.94940300	0.58541600
H	18.80660300	1.38550200	1.22196900
C	16.79136400	0.67249100	1.09655100
C	15.78355800	0.13953800	0.25920400
C	16.50898300	0.92966000	2.52079800
C	15.04328300	-0.05318300	5.38169700
C	14.41633700	-1.33698100	4.80353500
H	13.36436300	-1.19171200	4.54574500

H	14.48219300	-2.13648800	5.54794300
H	14.94456200	-1.67597700	3.90720700
C	16.51433600	-0.34004900	5.72515000
H	17.07278700	-0.67351000	4.84821000
H	16.55726500	-1.13532500	6.47771200
H	17.00900400	0.54520700	6.12340800
C	14.26667300	0.31425000	6.66344000
H	13.21416800	0.52023500	6.43818900
H	14.69170600	1.19222100	7.14959900
H	14.30065800	-0.52500900	7.36572500
C	18.81353600	3.91812100	4.36466200
C	20.05463100	3.24582400	3.77320000
H	19.63028000	2.55600400	3.04374700
C	20.96401300	3.94357400	1.59540100
C	21.72689000	4.92589700	0.79529900
C	21.91651200	4.70305400	-0.55885200
H	21.48243500	3.81662100	-1.00691300
C	22.66396000	5.61447000	-1.33214500
H	22.80034200	5.42986100	-2.39232100
C	23.22492000	6.73227400	-0.74553400
H	23.80456800	7.43181100	-1.34055600
C	23.05072100	6.99161600	0.63810200
C	23.59657200	8.13210500	1.28382700
H	24.18651500	8.83235800	0.69991000
C	23.38096800	8.35034600	2.62918900
H	23.80352800	9.22472600	3.11256600
C	22.59418000	7.45409100	3.38098400
H	22.38663900	7.64029900	4.42848500
C	22.05131300	6.33302300	2.78270000
C	22.28106900	6.07373200	1.41229900
C	21.15737700	5.44947400	3.55312500
C	20.96437100	2.38069000	4.69725400
C	22.09599000	1.79836900	3.82694800
H	22.75828200	2.58322300	3.44908900
H	21.69865900	1.24690000	2.96963800
H	22.70221900	1.11154800	4.42568500
C	21.58843700	3.13774100	5.88087900
H	22.28504200	3.91066900	5.54931400
H	22.14805600	2.42770100	6.50030600
H	20.82079000	3.60967400	6.49432700
C	20.11762900	1.20745700	5.23154500
H	19.34004500	1.55852000	5.91018800
H	20.76406800	0.50946400	5.77382400
H	19.63042800	0.66544100	4.41627100
C	15.37545000	4.75381100	0.97311200
C	16.68588100	6.85214000	1.67618100
C	14.20955900	3.99332600	1.19690600
C	16.12031500	4.52020300	-0.19742900
H	16.45072400	7.81236700	2.13603100
N	17.80485800	6.69597000	1.03739100
C	13.77223000	3.07655900	0.25145800
H	13.64816300	4.13668100	2.11117100
C	15.70270700	3.55809000	-1.11654800
H	17.02415900	5.08860000	-0.37283800
S	18.79045300	8.08427000	1.07267000
C	14.51800500	2.85315400	-0.91026900
H	12.86023500	2.51973000	0.42903800
H	16.30006000	3.37019000	-2.00304300
O	19.76384300	7.95237900	2.16871500
O	17.97686400	9.31623200	1.01018800
C	19.64687500	7.86533000	-0.48222500
H	14.18483400	2.11776000	-1.63452000
H	20.11796200	6.88350000	-0.47729200
H	18.92472200	7.95025100	-1.29399800
H	20.39895400	8.65233800	-0.54453300
C	15.73978100	5.79935600	1.93479800
H	11.67293400	10.16601000	0.73293000
C	12.56552000	9.55448500	0.90599800
C	12.75679400	9.33687900	2.40894400
C	12.44209200	8.22421500	0.15460900
H	13.41944800	10.11810900	0.50748800
C	13.96817500	8.41077000	2.70676900
H	11.85815900	8.90017500	2.84922000
H	12.92803800	10.28505500	2.91938700
C	13.67441300	7.32382200	0.42614800
H	12.36037300	8.38547700	-0.92509900
H	11.53304000	7.69949900	0.47125900
C	13.77883100	7.15335100	1.91096100
H	14.87534600	8.93799600	2.39503700
H	14.03825800	8.18932600	3.77130900
H	14.57110200	7.81794200	0.03555300
H	13.56213100	6.36168300	-0.07696200
H	13.03053100	6.47443000	2.32671800
H	14.74651700	6.31389500	2.38902600

Structure TS3

Rh	16.32620600	5.11136200	4.02712400
Rh	16.55838700	4.38079600	6.38228500
O	17.27888200	6.84031800	4.55642200
O	17.54703800	6.18014600	6.71080100
O	19.27596700	10.00177300	4.03267800
O	15.64143000	8.57398400	6.38739700
O	14.58003600	5.97356400	4.65036500
O	14.68949700	5.22285600	6.77717800
O	11.55354000	8.50413300	4.82912800
O	12.45043400	4.05062900	4.31416100
O	15.39517700	3.31391800	3.66547900
O	15.54658700	2.65651800	5.83259800
O	13.31874700	0.80832300	1.78070100
O	17.28417000	0.95286000	4.01538500
O	18.08265900	4.11506000	3.66961600
O	18.38943300	3.57038100	5.84362400
O	21.11863000	2.15403800	1.90540600
O	20.17754000	5.94265200	4.28673800
N	17.49916200	9.40080900	5.34937400
N	12.07579000	6.28949900	4.59377200
N	15.27010300	0.67817300	2.97739200
N	20.59474400	4.03324300	3.10280500
C	17.64480400	7.00996800	5.76807400
C	18.33599400	8.35736700	5.98479700
H	19.21824200	8.30062000	5.34416300
C	18.09787300	10.16869500	4.33738400
C	17.25072000	11.17486200	3.66479700
C	17.79565600	11.97046500	2.67096600
H	18.84334600	11.84775600	2.42126500
C	16.99670600	12.91680000	1.99821700
H	17.43798500	13.53376700	1.22287800
C	15.66056800	13.05636700	2.31788900
H	15.04287200	13.77999100	1.79434500
C	15.06703500	12.24964300	3.32270000
C	13.68433400	12.30981100	3.64138600
H	13.05729800	13.02502500	3.11746400
C	13.13836900	11.45846300	4.58118000
H	12.07735300	11.48933300	4.80020000
C	13.95059400	10.52042200	5.25157400
H	13.51680400	9.83305500	5.96593700
C	15.30465800	10.44602700	4.97502900
C	15.88393700	11.30171500	4.00743600
C	16.12479500	9.41017200	5.63146400
C	18.85601100	8.71495700	7.40670100
C	19.49130900	10.11746000	7.34039700
H	19.94878400	10.35809600	8.30499900
H	18.74306200	10.88649500	7.12539600
H	20.26553900	10.17105700	6.56967900
C	17.77557800	8.70937600	8.50007600
H	17.27955700	7.74048900	8.55989000
H	17.01528300	9.47237900	8.31972200
H	18.24541300	8.92482000	9.46617500
C	19.96051100	7.69931800	7.76436600
H	19.55770500	6.68995400	7.85539000
H	20.42077900	7.97737600	8.71787900
H	20.74679000	7.68714700	7.00180500
C	14.09037200	5.75582000	5.80877800
C	12.64981000	6.27195100	5.96222000
H	12.76389300	7.33362800	6.19746700
C	11.74270200	7.54806900	4.07880200
C	11.69935800	7.68918100	2.60731200
C	11.37004600	8.91123200	2.04743700
H	11.05829000	9.71762900	2.70090700

C	11.48438400	9.11063500	0.65708400
H	11.22455500	10.07440800	0.23243500
C	11.95597500	8.10011900	-0.15817300
H	12.07524500	8.26591400	-1.22456000
C	12.29597900	6.83316100	0.37954100
C	12.79579000	5.76635600	-0.41244400
H	12.93472700	5.92549700	-1.47750500
C	13.09468100	4.54500200	0.15716900
H	13.46580900	3.73422600	-0.45927100
C	12.90882500	4.33119500	1.53855900
H	13.14226000	3.37237500	1.98586700
C	12.43610300	5.35888800	2.33937600
C	12.13711200	6.62342300	1.78169500
C	12.30420300	5.14714900	3.79231900
C	11.76988100	5.69071800	7.11034700
C	10.32792500	6.18128100	6.88982400
H	10.28690500	7.27009700	6.79585900
H	9.70401900	5.88168300	7.73780100
H	9.89845200	5.74602300	5.98272600
C	11.77296000	4.15761800	7.21493800
H	11.32548300	3.69602800	6.33547900
H	11.19741700	3.86089400	8.09868100
H	12.78734300	3.77004800	7.31999800
C	12.29841100	6.28749100	8.43180300
H	12.27005300	7.38290900	8.41007700
H	13.32596300	5.97273600	8.62335400
H	11.67301000	5.95294400	9.26541500
C	15.25102500	2.47798800	4.62106800
C	14.56282700	1.18867700	4.17199500
H	13.60054900	1.52927500	3.78464500
C	14.50746100	0.49725400	1.81384900
C	15.19861300	-0.09095500	0.64817600
C	14.47411200	-0.38961100	-0.49529200
H	13.40689300	-0.19967400	-0.50089800
C	15.12118600	-0.92284000	-1.62704200
H	14.54248500	-1.15580600	-2.51440700
C	16.48595300	-1.14309700	-1.61123500
H	16.98630000	-1.54575100	-2.48702600
C	17.25799800	-0.84507100	-0.45962800
C	18.66438200	-1.03349700	-0.40509300
H	19.17357200	-1.42742600	-1.27969300
C	19.37441300	-0.71374300	0.73273200
H	20.45038300	-0.84445900	0.76101700
C	18.71308200	-0.19616100	1.86432500
H	19.27216500	0.07137900	2.75215400
C	17.34335700	-0.00610400	1.84724800
C	16.59483500	-0.32090300	0.68886300
C	16.67187600	0.57233000	3.02313900
C	14.24502900	0.09883700	5.23699200
C	13.54094600	-1.07150600	4.52249200
H	12.65611800	-0.73411300	3.97480500
H	13.22455800	-1.81392400	5.26160600
H	14.20988100	-1.57109600	3.81518200
C	15.47800200	-0.44256100	5.97843500
H	16.16158700	-0.95930600	5.30111100
H	15.15020000	-1.16274300	6.73629300
H	16.02699700	0.36020200	6.47048000
C	13.25715200	0.71050200	6.25055200
H	12.37026900	1.10918800	5.74633900
H	13.71952400	1.51895000	6.81734900
H	12.92628700	-0.06127700	6.95287400
C	18.77237800	3.67275400	4.64868400
C	20.17243000	3.19075300	4.24392200
H	20.00553300	2.21107100	3.78795300
C	20.93836300	3.36878100	1.91438900
C	21.05727700	4.19767000	0.69655600
C	21.50800700	3.63119400	-0.48271900
H	21.81761600	2.59239200	-0.47672600
C	21.54093100	4.38912300	-1.66958100
H	21.90628000	3.93345300	-2.58360500
C	21.07087000	5.68622900	-1.68433800
H	21.05920100	6.25629800	-2.60862500
C	20.58533000	6.29494300	-0.49889700
C	20.06174200	7.61467200	-0.46195400
H	20.00596300	8.18003400	-1.38772500
C	19.63111300	8.17371000	0.72559500
H	19.22278600	9.17873800	0.74288000
C	19.73819700	7.45425800	1.93341100
H	19.43580000	7.90394800	2.87045000
C	20.23072200	6.15999600	1.92702400
C	20.62461600	5.54620500	0.71409300
C	20.34224100	5.41571500	3.19252700
C	21.22232400	2.96033900	5.37428800
C	22.58049800	2.65960100	4.71393600
H	22.95277000	3.52717700	4.16094000
H	22.51090900	1.81932100	4.01886800
H	23.31490300	2.41339300	5.48738800
C	21.39237200	4.14610400	6.33816600
H	21.77559100	5.02714100	5.82237700
H	22.10368900	3.86573700	7.12309000
H	20.44646300	4.41600200	6.80900300
C	20.78098500	1.71285700	6.16781300
H	19.82747400	1.87965100	6.67100400
H	21.53495500	1.47050900	6.92359600
H	20.67660500	0.84331200	5.50874600
C	16.20675500	5.67113500	2.09605800
C	15.81403300	6.94700600	1.58878000
C	16.67980300	4.56514400	1.22454100
C	15.16484300	7.86523300	2.46640200
C	16.02055900	7.36644800	0.23600400
H	16.78207100	3.60024100	1.72463900
N	16.99707600	4.70368800	-0.01491400
C	14.71036700	9.08949900	2.00689100
H	14.99253600	7.57722600	3.49159500
C	15.61383100	8.61702600	-0.19117900
H	16.52098700	6.69928300	-0.44760000
S	17.60371600	3.28418000	-0.79911400
C	14.94191600	9.47787700	0.68660500
H	14.15901100	9.73884300	2.66976100
H	15.80153600	8.92281200	-1.21522000
O	18.14975800	2.34400900	0.18874500
O	18.44812900	3.77160700	-1.89243400
C	16.08951200	2.61484600	-1.48921700
H	14.60053400	10.44931900	0.34355800
H	15.44057500	2.29816400	-0.67497800
H	15.62075400	3.38435500	-2.10203100
H	16.37416400	1.75408500	-2.09564600

Structure 5a

C	-3.94442400	-1.45953900	0.04260200
C	-2.41100500	-1.41996400	0.01609300
C	-1.88990900	0.02068200	-0.05822200
C	-2.43879900	0.88330800	1.09757300
C	-3.98116600	0.83559900	1.11695900
C	-4.50010400	-0.60597200	1.18985700
H	-0.79423000	0.03641200	-0.03914000
H	-2.02187700	-1.89686200	0.92651000
H	-2.02778400	-2.00088600	-0.83074700
H	-4.33078900	-1.07498600	-0.91177400
H	-4.29733400	-2.49345000	0.13160300
H	-2.08625500	0.43420400	2.03644600
H	-4.35554500	1.42392500	1.96244700
H	-4.36629400	1.31388400	0.20637800
H	-4.19743100	-1.04924900	2.14881000
H	-5.59610100	-0.61052600	1.17415000
H	-2.19391700	0.46705500	-1.01451800
C	-1.91334800	2.30241000	1.04758300
C	-1.13081800	2.81341700	2.08962800
C	-2.19334400	3.14335700	-0.03962100
C	-0.63728400	4.11883800	2.06052600
H	-0.90013000	2.18150900	2.94283800
C	-1.71125900	4.45023800	-0.09015900
H	-2.79728900	2.77873200	-0.86490500
C	-0.93521300	4.92447400	0.96566800
H	-0.03250500	4.49985900	2.87520700
H	-1.93498300	5.08951400	-0.93630500
Br	-0.26653400	6.71963100	0.90741700

Structure 13

C	-1.76159600	-2.27320400	0.50755300

C	-0.22623900	-2.23852100	0.38766300
C	0.29621800	-0.82739200	0.09233600
C	-0.17786800	0.18912300	1.14508800
C	-1.70867900	0.15044200	1.26997300
C	-2.22381100	-1.25993400	1.57206400
H	1.38996600	-0.85903900	0.04673800
H	0.21603800	-2.59690700	1.32625300
H	0.10044500	-2.93022900	-0.39626700
H	-2.17517400	-1.94846500	-0.45704200
H	0.25661900	-0.09865500	2.11196600
H	-2.03793000	0.84336600	2.04940900
H	-2.14726800	0.50544100	0.32645400
H	-1.85755000	-1.57772500	2.55696300
H	-3.31775500	-1.25667800	1.63071500
H	-0.05306800	-0.50642800	-0.89940100
C	0.29883700	1.64725600	0.84023700
H	-0.17219200	1.93298500	-0.10975300
C	1.78086400	1.65142700	0.57354200
H	2.10526200	1.29275100	-0.40861400
N	2.63528900	2.03182800	1.44672300
S	4.28141100	1.86861100	0.98093900
O	4.91165500	1.01157700	1.99515300
O	4.42032200	1.50434300	-0.44113100
C	4.82590000	3.55813100	1.21020700
H	4.59528700	3.85774300	2.23211700
H	5.90262600	3.57058900	1.03510800
H	4.30997900	4.19149300	0.48838400
C	-0.13217600	2.63513800	1.90608900
C	-0.93033000	3.72867900	1.55454700
C	0.23332900	2.47157300	3.24941800
C	-1.35688100	4.64386900	2.51921000
H	-1.22257700	3.86334500	0.51650800
C	-0.19106900	3.38347700	4.21492800
H	0.85802900	1.63434000	3.53975000
C	-0.98763600	4.47318300	3.85361600
H	-1.97750400	5.48569300	2.22717400
H	0.10132800	3.24368700	5.25136000
H	-1.31809500	5.18147100	4.60728600
C	-2.28048800	-3.66918800	0.77999500
C	-3.12540200	-4.30870100	-0.13428600
C	-1.93088100	-4.35899700	1.95016100
C	-3.61331200	-5.59543000	0.09932100
H	-3.40995900	-3.79445000	-1.04804100
C	-2.40629200	-5.64442300	2.20402000
H	-1.27698200	-3.89197400	2.68033100
C	-3.24587400	-6.25004900	1.27109400
H	-4.26687900	-6.07774900	-0.61818000
H	-2.12867300	-6.16692300	3.11207600
Br	-3.90578400	-8.01682700	1.60920500

Structure Rh_2(*S*-TPNTTL)$_4$ (7a)

Rh	13.01886100	0.00145900	9.81110200
Rh	13.01786900	0.00216100	12.20696500
O	14.86508100	0.87007100	9.87326600
O	14.84987800	0.94501000	12.13621300
O	14.86580400	3.77446800	10.53542900
O	18.41002900	1.48814700	8.80691900
N	16.74061700	2.73482200	9.75556800
C	15.36150200	1.18344800	11.00092000
C	16.74578200	1.81247400	10.90819400
C	17.42527500	2.35896900	12.19682800
C	17.71202200	1.15455700	13.11777800
H	16.78809800	0.68915600	13.46362500
H	18.28140100	1.48708100	13.99160300
H	18.30644500	0.39446600	12.59859700
C	16.59561400	3.40466700	12.95916800
H	16.44531600	4.30912800	12.36707700
H	17.12649400	3.68248100	13.87656700
H	15.61525700	3.01050600	13.23003600
C	18.77322100	2.98439200	11.78807900
H	19.39064800	2.27428000	11.22994800
H	19.32311900	3.28884900	12.68394500
H	18.62992300	3.87226400	11.16513100
C	15.71669400	3.68782500	9.65473200
C	15.72658200	4.56843200	8.46691000
C	16.65882000	4.36226300	7.42320400
C	17.57900000	3.29370300	7.51957400
C	17.62575400	2.42820900	8.71162800
C	18.46708800	3.05027700	6.49518400
H	19.15541200	2.21986500	6.58610400
C	18.55898800	3.92620500	5.39506700
C	17.70870700	5.03369700	5.30158500
C	16.65659700	5.21246500	6.27116100
C	15.60736200	6.19113800	6.15349000
C	14.72544000	6.40857300	7.22276400
C	14.80505100	5.59027700	8.37461400
H	14.07948600	5.71887700	9.16775000
C	19.64675300	3.64354900	4.41281800
C	20.75249200	4.49423800	4.27802700
H	20.79136800	5.41286400	4.85306600
C	21.79511600	4.16567000	3.41296900
H	22.64838800	4.83108000	3.32283200
C	21.74762400	2.98217900	2.67117600
H	22.56268400	2.72517500	2.00158700
C	20.64982400	2.12990700	2.79837500
H	20.60849300	1.20182600	2.23924700
C	19.60722900	2.45860400	3.66581900
H	18.75735400	1.79039300	3.76945400
C	18.04331900	6.06835400	4.28248000
C	18.53740800	7.30465000	4.71903600
H	18.57266900	7.51549900	5.78348600
C	18.99001500	8.25071000	3.80076400
H	19.37584400	9.20236300	4.15342800
C	18.94661300	7.97399700	2.43328500
H	19.29740800	8.70992800	1.71628800
C	18.44907000	6.74583600	1.99105700
H	18.40832200	6.52542800	0.92869800
C	18.00795400	5.79652400	2.91004000
H	17.63377000	4.83804400	2.56698100
C	15.34066800	6.87370500	4.85542600
C	15.44760100	8.26019500	4.70311300
H	15.80624300	8.86039700	5.53231800
C	15.09652000	8.86616100	3.49916800
H	15.19165900	9.94210100	3.39049200
C	14.62280400	8.09431900	2.43548400
H	14.34746900	8.56873100	1.49849200
C	14.50707800	6.71059200	2.58178300
H	14.14048100	6.10300200	1.75993900
C	14.86574800	6.10453200	3.78493500
H	14.77563800	5.02916000	3.90342200
C	13.67577400	7.46944300	7.22952700
C	13.57682800	8.29680100	8.35930000
H	14.27850300	8.16551400	9.17747000
C	12.59996900	9.28855100	8.43970600
H	12.54508300	9.91835600	9.32253000
C	11.70052600	9.47122300	7.38826200
H	10.93878800	10.24274900	7.44759100
C	11.78635200	8.65457500	6.25910000
H	11.08797500	8.77809600	5.43855200
C	12.76344300	7.66299900	6.18108300
H	12.80707400	7.03347500	5.30271500
O	11.17253100	-0.86715800	9.87222900
O	11.18599800	-0.94072600	12.13523700
O	11.17206100	-3.77122800	10.53608200
O	7.62839100	-1.48696400	8.80370400
N	9.29750200	-2.73254200	9.75432900
C	10.67531500	-1.17996900	10.99967900
C	9.29128800	-1.80947500	10.90636000
C	8.61112000	-2.35543100	12.19487800
C	8.32325200	-1.15054400	13.11487700
H	9.24676700	-0.68462900	13.46113500
H	7.75333300	-1.48273400	13.98847800
H	7.72896600	-0.39096800	12.59479200
C	9.44067000	-3.40028900	12.95848800
H	9.59174300	-4.30508200	12.36712400
H	8.90925900	-3.67765900	13.87571100
H	10.42068300	-3.00554400	13.22976300
C	7.26371200	-2.98164500	11.78558300
H	6.64638700	-2.27213500	11.22656800

H	6.71329500	-3.28574400	12.68125200
H	7.40779700	-3.86986000	11.16330400
C	10.32166100	-3.68542300	9.65482100
C	10.31265800	-4.56695600	8.46768100
C	9.38111700	-4.36166900	7.42317000
C	8.46074000	-3.29316700	7.51814700
C	8.41300400	-2.42687200	8.70957700
C	7.57333500	-3.05057400	6.49295000
H	6.88485900	-2.22017700	6.58279600
C	7.48240000	-3.92723600	5.39335800
C	8.33290200	-5.03468900	5.30123600
C	9.38425400	-5.21271500	6.27174400
C	10.43362800	-6.19142100	6.15559700
C	11.31479100	-6.40799000	7.22566600
C	11.23433700	-5.58878600	8.37681400
H	11.95937800	-5.71670300	9.17053500
C	6.39537300	-3.64539400	4.41004300
C	5.28980900	-4.49627600	4.27510000
H	5.25056500	-5.41441400	4.85090000
C	4.24787000	-4.16852900	3.40890900
H	3.39471600	-4.83407100	3.31863300
C	4.29588100	-2.98565600	2.66616200
H	3.48136200	-2.72927800	1.99567500
C	5.39350000	-2.13317100	2.79355600
H	5.43518200	-1.20555000	2.23368600
C	6.43542300	-2.46106600	3.66211900
H	7.28512900	-1.79267300	3.76595800
C	7.99912200	-6.07000200	4.28251100
C	7.50464400	-7.30599400	4.71947900
H	7.46854200	-7.51615600	5.78403800
C	7.05275600	-8.25265000	3.80146600
H	6.66663200	-9.20406600	4.15444600
C	7.09724500	-7.97682700	2.43384300
H	6.74698800	-8.71321200	1.71704700
C	7.59517900	-6.74897000	1.99121100
H	7.63677500	-6.52925200	0.92874100
C	8.03561600	-5.79907900	2.90992300
H	8.41013700	-4.84085100	2.56653200
C	10.70125900	-6.87505400	4.85828400
C	10.59434400	-8.26165200	4.70703400
H	10.23500200	-8.86114300	5.53645000
C	10.94636300	-8.86864200	3.50387800
H	10.85126000	-9.94466700	3.39602100
C	11.42102000	-8.09771900	2.43994900
H	11.69707300	-8.57293400	1.50357500
C	11.53671000	-6.71387500	2.58518800
H	11.90402100	-6.10699700	1.76313500
C	11.17709100	-6.10679100	3.78753900
H	11.26718900	-5.03133000	3.90522100
C	12.36447000	-7.46883800	7.23403300
C	12.46272500	-8.29519800	8.36460300
H	11.76053900	-8.16320100	9.18221900
C	13.43955800	-9.28684800	8.44650200
H	13.49390600	-9.91585600	9.32992600
C	14.33967200	-9.47043300	7.39578700
H	15.10139900	-10.24187900	7.45628400
C	14.25453200	-8.65479700	6.26584400
H	14.95343100	-8.77901300	5.44584700
C	13.27745800	-7.66332200	6.18633700
H	13.23438900	-7.03458000	5.30739000
O	12.15030300	1.84776500	9.87188000
O	12.07493900	1.83405800	12.13483100
O	9.24571500	1.84811100	10.53344800
O	11.53155500	5.39169000	8.80300000
N	10.28501400	3.72269200	9.75259400
C	11.83660300	2.34486000	10.99914500
C	11.20710300	3.72886700	10.90541300
C	10.66002000	4.40889400	12.19352500
C	11.86407700	4.69630700	13.11474800
H	12.32951500	3.77262000	13.46118700
H	11.53116600	5.26603600	13.98819900
H	12.62424100	5.29062000	12.59554900
C	9.61423700	3.57942900	12.95596800
H	8.70991500	3.42881000	12.36375800

H	9.33621600	4.11067600	13.87308900
H	10.00843000	2.59921900	13.22732200
C	10.03448800	5.75652000	11.78389800
H	10.74466700	6.37380500	11.22569300
H	9.72963700	6.30682300	12.67938100
H	9.14684200	5.61274900	11.16073300
C	9.33224500	2.69852300	9.65226700
C	8.45178000	2.70749000	8.46433700
C	8.65796900	3.63904800	7.42002000
C	9.72636900	4.55945600	7.51593000
C	10.59164300	4.60716300	8.70810000
C	9.96984200	5.44685800	6.49094700
H	10.80013900	6.13535900	6.58152600
C	9.09412600	5.53778900	5.39058600
C	7.98679900	4.68724500	5.29749400
C	7.80794800	3.63588600	6.26784200
C	6.82941600	2.58645800	6.15078500
C	6.61191200	1.70527400	7.22065500
C	7.43008700	1.78575000	8.37253100
H	7.30151700	1.06068800	9.16612300
C	9.37681300	6.62486300	4.40757200
C	8.52599300	7.73037900	4.27179800
H	7.60725100	7.76953900	4.84663600
C	8.85457800	8.77239300	3.40601700
H	8.18906900	9.62550400	3.31510000
C	10.03822500	8.72451300	2.66449300
H	10.29523300	9.53909600	1.99432400
C	10.89063800	7.62693900	2.79268700
H	11.81885000	7.58534700	2.23379000
C	10.56191000	6.58494400	3.66084900
H	11.23022500	5.73525000	3.76527700
C	6.95239600	5.02090900	4.27780500
C	5.71597000	5.51532800	4.71361300
H	5.50482800	5.55148300	5.77797700
C	4.77012900	5.96709600	3.79470000
H	3.81836000	6.35317500	4.14677700
C	5.04721500	5.92253300	2.42733300
H	4.31146700	6.27268600	1.70983400
C	6.27551300	5.42465900	1.98586000
H	6.49620700	5.38301000	0.92359400
C	7.22459500	4.98435200	2.90547500
H	8.18317200	4.60988100	2.56299700
C	6.14707800	2.31875800	4.85279800
C	4.76063000	2.42565300	4.70009800
H	4.16029000	2.78508100	5.52886100
C	4.15489300	2.07348100	3.49636200
H	3.07897700	2.16853500	3.38738400
C	4.92692300	1.59869700	2.43328100
H	4.45267500	1.32250300	1.49645900
C	6.31061200	1.48306100	2.57995400
H	6.91835100	1.11568500	1.75856700
C	6.91645400	1.84284300	3.78289200
H	7.99179600	1.75276000	3.90168800
C	5.55106700	0.65558800	7.22806100
C	4.72381000	0.55726900	8.35796800
H	4.85518800	1.25937600	9.17575200
C	3.73202700	-0.41951100	8.43900700
H	3.10232500	-0.47388900	9.32193600
C	3.54921500	-1.31950900	7.38806700
H	2.77766300	-2.08118600	7.44784900
C	4.36577200	-1.23432300	6.25878400
H	4.24215400	-1.93315100	5.43864100
C	5.35737100	-0.25731800	6.18013700
H	5.98683300	-0.21424200	5.30169800
O	13.88735300	-1.84484800	9.87362800
O	13.96070400	-1.82983600	12.13662100
O	16.79135600	-1.84578800	10.53800200
O	14.50644000	-5.39000000	8.80757100
N	15.75223300	-3.72049300	9.75721500
C	14.19991200	-2.34138400	11.00145600
C	14.82895100	-3.72569700	10.90909200
C	15.37442400	-4.40528700	12.19812100
C	14.16923300	-4.69208800	13.11805800
H	13.70345900	-3.76817300	13.46343600

H	14.50104600	-5.26136600	13.99222100
H	13.40964900	-5.28662500	12.59826900
C	16.41951200	-3.57572600	12.96140900
H	17.32429900	-3.42505300	12.36993600
H	16.69680500	-4.10691100	13.87878800
H	16.02501200	-2.59553300	13.23239700
C	16.00017200	-5.75319900	11.78978400
H	15.29056000	-6.37053500	11.23092300
H	16.30378600	-6.30322100	12.68585800
H	16.88862700	-5.60985100	11.16767200
C	16.70537000	-2.69663700	9.65718900
C	17.58689000	-2.70644200	8.47004000
C	17.38153800	-3.63864600	7.42613200
C	16.31296200	-4.55887200	7.52167400
C	15.44652600	-4.60564900	8.71303700
C	16.07035900	-5.44696200	6.49707700
H	15.23990700	-6.13531900	6.58735300
C	16.94708500	-5.53874100	5.39760700
C	18.05457800	-4.68834300	5.30494300
C	18.23260200	-3.63632200	6.27472200
C	19.21130000	-2.58701600	6.15784100
C	19.42791600	-1.70515500	7.22731500
C	18.60874800	-1.78485900	8.37854600
H	18.73670300	-1.05929700	9.17177800
C	16.66522500	-6.62645000	4.41505400
C	17.51603800	-7.73216900	4.28095300
H	18.43415900	-7.77106500	4.85680000
C	17.18824200	-8.77474800	3.41554500
H	17.85373900	-9.62800700	3.32593500
C	16.00539900	-8.72722000	2.67272800
H	15.74898300	-9.54223100	2.00285300
C	15.15297100	-7.62945700	2.79928700
H	14.22536500	-7.58814000	2.23936700
C	15.48091000	-6.58690100	3.66706400
H	14.81257400	-5.73707100	3.77024200
C	19.08990900	-5.02281800	4.28646100
C	20.32581900	-5.51718800	4.72379300
H	20.53592100	-5.55267200	5.78838400
C	21.27244700	-5.96978000	3.80610000
H	22.22379600	-6.35582200	4.15935200
C	20.99668800	-5.92608600	2.43843500
H	21.73305000	-6.27689600	1.72188800
C	19.76892800	-5.42824300	1.99544400
H	19.54926200	-5.38726400	0.93293900
C	18.81904800	-4.98712200	2.91384800
H	17.86088300	-4.61268400	2.57018600
C	19.89474700	-2.32020300	4.86025600
C	21.28132800	-2.42717700	4.70884800
H	21.88097200	-2.78588000	5.53843000
C	21.88809600	-2.07602700	3.50532500
H	22.96410600	-2.17116200	3.39735400
C	21.11695700	-1.60226000	2.44115400
H	21.59198100	-1.32692900	1.50446900
C	19.73313300	-1.48652000	2.58654600
H	19.12609500	-1.11988900	1.76431000
C	19.12628000	-1.84519700	3.78929700
H	18.05083300	-1.75506600	3.90709300
C	20.48875700	-0.65547100	7.23490800
C	21.31516600	-0.55648100	8.36538300
H	21.18317200	-1.25811500	9.18347400
C	22.30683400	0.42038700	8.44660800
H	22.93587400	0.47530800	9.32997500
C	22.49038400	1.31981100	7.39529800
H	23.26185200	2.08155600	7.45526100
C	21.67471000	1.23393800	6.26544300
H	21.79891300	1.93229300	5.44498200
C	20.68322900	0.25681000	6.18659400
H	20.05446900	0.21315200	5.30768500
H	14.00126300	-4.35361300	10.56821700
H	8.66327700	-0.98194400	10.56526800
H	12.03488400	4.35694100	10.56506300
H	17.37373100	0.98451400	10.56800000

Structure III

Rh	12.90491600	0.07623600	9.73464900
Rh	12.98545800	0.01297100	12.19786600
O	14.75267300	0.95830500	9.78586500
O	14.83278500	0.93019600	12.05328400
O	15.01533100	3.85113700	10.64509500
O	18.24294500	1.39334400	8.57108700
N	16.75390200	2.71453500	9.70123300
C	15.30857600	1.18490300	10.91494600
C	16.73293500	1.71058500	10.78332200
C	17.52676800	2.09475400	12.06444700
C	17.73982600	0.80439300	12.88385000
H	16.79468000	0.41482200	13.26387400
H	18.39684700	1.01383200	13.73408100
H	18.21020000	0.02423400	12.27568800
C	16.84470300	3.15621200	12.94195600
H	16.75174200	4.11156100	12.42177600
H	17.44787600	3.31763300	13.84235200
H	15.84712200	2.83493800	13.24426900
C	18.90744700	2.61662400	11.61982500
H	19.42288100	1.89265700	10.98151500
H	19.52968000	2.80318900	12.50051400
H	18.82143500	3.55622900	11.06590600
C	15.82736300	3.76452000	9.72836100
C	15.86995400	4.73487200	8.61115000
C	16.68337800	4.48998600	7.48019900
C	17.48998200	3.33095200	7.44397900
C	17.54191000	2.40013300	8.58366500
C	18.25763100	3.05546100	6.33471000
H	18.86080500	2.15662600	6.32877700
C	18.35431400	3.97692600	5.27127800
C	17.61634900	5.16567700	5.30387300
C	16.66843000	5.38866600	6.36810100
C	15.69222300	6.44620200	6.36792400
C	14.93027200	6.70042400	7.51893000
C	15.05229800	5.84599300	8.64256500
H	14.40818100	5.99901500	9.49962900
C	19.32558600	3.62997300	4.19355400
C	20.45711900	4.41532100	3.93220200
H	20.60242900	5.34156300	4.47671900
C	21.39514500	4.00771000	2.98482100
H	22.27119200	4.62153700	2.79867000
C	21.21678900	2.81094000	2.28530300
H	21.95215400	2.49243800	1.55288900
C	20.09143900	2.02442800	2.53644800
H	19.94987800	1.08640400	2.01063900
C	19.15397600	2.43187000	3.48602300
H	18.28401400	1.81418900	3.68861500
C	17.95890300	6.22895500	4.31898100
C	18.55395800	7.40823900	4.78728000
H	18.66932600	7.55583900	5.85666900
C	19.00440000	8.37770900	3.89262800
H	19.46938400	9.28375800	4.26899000
C	18.85683500	8.18269900	2.51839400
H	19.20508200	8.93729600	1.81983000
C	18.25807100	7.01241600	2.04521500
H	18.13549700	6.85655800	0.97778800
C	17.81961100	6.03860400	2.93919300
H	17.36513500	5.12522800	2.57123800
C	15.35307900	7.15042800	5.10019400
C	15.49718300	8.53290200	4.94514400
H	15.92884300	9.11695400	5.75062700
C	15.09627500	9.15384600	3.76452600
H	15.22479300	10.22557400	3.64985200
C	14.53327900	8.40211600	2.73029000
H	14.22577900	8.88839500	1.80953600
C	14.37286000	7.02337200	2.88276600
H	13.93446400	6.43217300	2.08465400
C	14.78527200	6.40144600	4.05983800
H	14.67314100	5.32834400	4.17903700
C	13.91210300	7.78694200	7.61594900
C	13.91216500	8.61103400	8.75141500
H	14.67615800	8.47140400	9.51038400
C	12.95186700	9.61025100	8.91134300
H	12.97366500	10.23910700	9.79612300
C	11.96988700	9.80051600	7.93817900

H	11.22113800	10.57695400	8.06162700
C	11.95612400	8.98500200	6.80442000
H	11.19430200	9.11446200	6.04237700
C	12.91756800	7.98874000	6.64573300
H	12.88789100	7.36464800	5.76312400
O	11.07695300	-0.85402400	9.89338100
O	11.14473900	-0.92661500	12.15852800
O	11.20545000	-3.78984400	10.64143300
O	7.60963300	-1.64542000	8.84765400
N	9.29087100	-2.83511000	9.84999300
C	10.62440600	-1.18601800	11.04057300
C	9.26495100	-1.87360200	10.96912500
C	8.61669800	-2.40374600	12.28127400
C	8.30002400	-1.18209500	13.16907600
H	9.21183200	-0.67829900	13.49232800
H	7.74887700	-1.50627800	14.05757800
H	7.67678300	-0.45682000	12.63389200
C	9.48570200	-3.39949300	13.06675900
H	9.65443200	-4.31921000	12.50381300
H	8.97325500	-3.66163500	13.99913500
H	10.45709000	-2.96862800	13.31175300
C	7.28609000	-3.08400800	11.90447600
H	6.64148900	-2.41144700	11.33049100
H	6.75319000	-3.37694900	12.81430800
H	7.45188600	-3.98621100	11.30833700
C	10.32298500	-3.78161600	9.78693400
C	10.28532300	-4.74738900	8.66872700
C	9.34187500	-4.60391000	7.62669600
C	8.44436200	-3.51326500	7.65199600
C	8.39393600	-2.58972000	8.79645400
C	7.62550900	-3.27056200	6.57114900
H	6.97905200	-2.40237900	6.58595400
C	7.56623900	-4.17329900	5.49179400
C	8.34759800	-5.33230100	5.49781300
C	9.35222400	-5.50378000	6.51584300
C	10.40881100	-6.47391200	6.43585400
C	11.30438100	-6.63318400	7.50603400
C	11.21908900	-5.76210300	8.61821000
H	11.95801300	-5.83425400	9.40576700
C	6.62794700	-3.83073300	4.38419000
C	5.44396900	-4.54265700	4.15698800
H	5.21607300	-5.40840200	4.76988200
C	4.56508700	-4.14288200	3.15039900
H	3.64654100	-4.69824900	2.98664600
C	4.85977700	-3.02873800	2.35950000
H	4.17094300	-2.71645000	1.58057500
C	6.04118700	-2.31699600	2.57682900
H	6.27345500	-1.44290900	1.97759300
C	6.91936300	-2.71698900	3.58457400
H	7.83767500	-2.16471900	3.76111200
C	8.00869100	-6.41084500	4.52834800
C	7.49637500	-7.61559200	5.02891400
H	7.43566700	-7.75932500	6.10331600
C	7.06308100	-8.61739200	4.16149600
H	6.66426400	-9.54351300	4.56423100
C	7.14424100	-8.42911800	2.78095700
H	6.81086700	-9.20894200	2.10305600
C	7.65785900	-7.23226100	2.27496000
H	7.72950600	-7.08089200	1.20220600
C	8.07863900	-6.22695800	3.14187500
H	8.47203800	-5.29704400	2.74720600
C	10.69807400	-7.17634900	5.15351600
C	10.65834800	-8.56918100	5.03321400
H	10.31864100	-9.16659600	5.87234700
C	11.05924600	-9.18534200	3.84976600
H	11.01772200	-10.26685900	3.76548900
C	11.51848700	-8.41709200	2.77676700
H	11.83477000	-8.90044900	1.85741800
C	11.55771800	-7.02572300	2.88778500
H	11.90336600	-6.41904200	2.05645000
C	11.14155200	-6.40926100	4.06671000
H	11.14108200	-5.32761000	4.14564600
C	12.39411700	-7.65413300	7.54748400
C	12.56523400	-8.38794100	8.73254300
H	11.88009700	-8.22797600	9.55941200
C	13.59203000	-9.32245500	8.86113800
H	13.69992400	-9.87715700	9.78831500
C	14.47227400	-9.54529000	7.80154300
H	15.27293800	-10.27228000	7.89771000
C	14.31317000	-8.82738900	6.61486000
H	14.99213800	-8.98377900	5.78310600
C	13.28737900	-7.89134700	6.48995300
H	13.19191400	-7.34603900	5.56101900
O	12.07220300	1.92840700	9.97274900
O	12.05132300	1.85915800	12.23680700
O	9.23191100	1.94507600	10.54105100
O	11.59115400	5.55677500	9.05736200
N	10.31417200	3.83941100	9.87198300
C	11.79539500	2.39599900	11.12559300
C	11.18417600	3.79250500	11.06375000
C	10.58786800	4.41644200	12.35890600
C	11.75853400	4.68361800	13.32771400
H	12.23022200	3.75298000	13.64601200
H	11.38974100	5.20756600	14.21529900
H	12.52176800	5.31479800	12.85853900
C	9.53347400	3.54230200	13.05672300
H	8.65138300	3.39873300	12.43062100
H	9.21917200	4.03374600	13.98421100
H	9.93683800	2.55925800	13.30412000
C	9.95456700	5.76945500	11.98165900
H	10.67176100	6.41709800	11.46872600
H	9.61550500	6.28102500	12.88776900
H	9.08805300	5.63674100	11.32717300
C	9.39427500	2.79841600	9.67440300
C	8.65174800	2.78185300	8.39769400
C	8.90500300	3.75267300	7.40108900
C	9.87565200	4.75096800	7.63941900
C	10.66666900	4.76681400	8.88245000
C	10.10856800	5.73035200	6.69968500
H	10.85236300	6.48838700	6.90872100
C	9.33415500	5.79995800	5.52503900
C	8.36670200	4.82672000	5.25681100
C	8.18480300	3.72287000	6.16403000
C	7.29248300	2.61900600	5.92494900
C	6.99274600	1.72243600	6.96165200
C	7.68974600	1.81588200	8.18974900
H	7.50697600	1.07759200	8.96129400
C	9.52184900	7.00758800	4.66562900
C	8.55940500	8.02704400	4.66395100
H	7.64998200	7.89901300	5.24107200
C	8.76498100	9.19532800	3.93184500
H	8.01161300	9.97713300	3.94383300
C	9.93850800	9.36300200	3.19237900
H	10.10143600	10.27487800	2.62621100
C	10.90143200	8.35346800	3.18740700
H	11.82088700	8.47498500	2.62641100
C	10.69375500	7.18382500	3.91998500
H	11.44876400	6.40479300	3.90497400
C	7.45157700	5.08655200	4.10908700
C	6.13852800	5.49861900	4.36921500
H	5.78562800	5.52949500	5.39557000
C	5.29489100	5.87338300	3.32507000
H	4.28040900	6.19478200	3.54041900
C	5.75414400	5.83465100	2.00740200
H	5.09804800	6.12431300	1.19234300
C	7.06208700	5.42263300	1.74131500
H	7.42474500	5.38810100	0.71851300
C	7.90824900	5.05843400	2.78704800
H	8.92778000	4.74857700	2.58285200
C	6.79189200	2.31455300	4.55340100
C	5.43944200	2.39078100	4.20488200
H	4.72401300	2.76890400	4.92672700
C	5.01346500	1.98172700	2.94355600
H	3.96218000	2.05220700	2.68226100
C	5.93197500	1.47695300	2.01953500
H	5.59622600	1.15370700	1.03893900
C	7.28286000	1.39169300	2.36169300
H	8.00360700	0.99850700	1.65124300

C	7.70902200	1.81185000	3.62123500
H	8.75561200	1.73345500	3.89758700
C	5.92685500	0.68131500	6.87268000
C	4.99299200	0.62214300	7.92060800
H	5.07357600	1.32578500	8.74350400
C	3.95788100	-0.31094500	7.91052300
H	3.24687800	-0.33427700	8.73082300
C	3.83500000	-1.20415900	6.84550200
H	3.02753000	-1.92993300	6.82931100
C	4.75787800	-1.15958500	5.79947000
H	4.67669000	-1.85372900	4.97161100
C	5.79888400	-0.23106600	5.81463900
H	6.51262800	-0.22490900	5.00161700
O	13.78909900	-1.78894800	9.73273700
O	13.90884400	-1.81197800	11.99908000
O	16.69331900	-1.80698800	10.35623600
O	14.48939700	-5.45186800	8.73004600
N	15.67549300	-3.71283200	9.62470000
C	14.11701700	-2.30143700	10.85560600
C	14.72722500	-3.69395000	10.75460800
C	15.25065900	-4.37795600	12.05364200
C	14.04061300	-4.63139900	12.97707200
H	13.61092200	-3.69662100	13.33741000
H	14.35717000	-5.22579200	13.84026700
H	13.25608100	-5.18754400	12.45249700
C	16.31893700	-3.56465100	12.80266800
H	17.22314600	-3.44091200	12.20298000
H	16.59259000	-4.09394500	13.72220400
H	15.94909900	-2.57364100	13.06736600
C	15.84654100	-5.74641200	11.66656300
H	15.12170600	-6.36321600	11.12794300
H	16.14764400	-6.28060900	12.57295800
H	16.73412500	-5.63559700	11.03633200
C	16.62882900	-2.69023200	9.50614000
C	17.52746900	-2.73639400	8.33212200
C	17.38064000	-3.74030800	7.34614000
C	16.35926700	-4.70408200	7.49204000
C	15.43729900	-4.67410200	8.63793600
C	16.18233600	-5.67949200	6.53586400
H	15.38720000	-6.40162000	6.66911400
C	17.06106500	-5.79029000	5.44210900
C	18.11923300	-4.88497100	5.29518400
C	18.24113900	-3.77024800	6.20187700
C	19.16616300	-2.68072900	6.02543300
C	19.33387500	-1.73071200	7.04407100
C	18.51207600	-1.78091400	8.19542300
H	18.60254000	-1.00433600	8.94365300
C	16.80946400	-6.93568600	4.51968800
C	17.70909300	-8.00276200	4.39585900
H	18.65463600	-7.96804600	4.92584600
C	17.39168700	-9.10367600	3.60124100
H	18.09457500	-9.92724400	3.51970100
C	16.17288500	-9.15324800	2.91926000
H	15.92642600	-10.01412800	2.30539600
C	15.27300600	-8.09223100	3.03249200
H	14.31933500	-8.12206200	2.51682700
C	15.59019300	-6.99196100	3.82939800
H	14.88820800	-6.16819800	3.91719400
C	19.16272800	-5.21314300	4.28382200
C	20.42903600	-5.61346300	4.73004500
H	20.65053700	-5.58515500	5.79252400
C	21.39225600	-6.05347500	3.82352500
H	22.36775400	-6.36626000	4.18322800
C	21.10233500	-6.08975100	2.45855700
H	21.85187000	-6.42985100	1.75058000
C	19.84361400	-5.68576600	2.00681500
H	19.61288200	-5.70741400	0.94613000
C	18.87706700	-5.25889100	2.91452200
H	17.89552300	-4.95720900	2.56517800
C	19.83467600	-2.44496600	4.71426900
C	21.22521200	-2.47713400	4.56497700
H	21.84455500	-2.76405500	5.40792900
C	21.81105500	-2.13924500	3.34730100
H	22.89102000	-2.17346100	3.24215000

C	21.01436200	-1.75362700	2.26649100
H	21.47307500	-1.48476600	1.31993000
C	19.62578100	-1.71869100	2.40792700
H	18.99828200	-1.42538100	1.57169000
C	19.03994500	-2.06396700	3.62498800
H	17.96091900	-2.03232900	3.74052700
C	20.33900000	-0.62860600	6.99422700
C	21.15743200	-0.42786900	8.11689700
H	21.06238700	-1.09315000	8.96975200
C	22.09302200	0.60536000	8.14753700
H	22.71662900	0.73986900	9.02623100
C	22.22792500	1.45911500	7.05155000
H	22.95601000	2.26444800	7.07160400
C	21.41987900	1.27130800	5.92874500
H	21.50709300	1.93320800	5.07434400
C	20.48248000	0.23940200	5.90116200
H	19.85607900	0.11868300	5.02750600
H	13.89359900	-4.30791300	10.40524100
H	8.60044200	-1.08507900	10.60630000
H	12.02792900	4.43051500	10.78505200
H	17.26451500	0.85908800	10.35122700
C	12.55321700	0.23568400	7.75446600
C	12.45803900	1.44837200	7.01476600
C	12.15029400	-0.99068900	7.10121800
C	13.17138700	2.60651900	7.42435400
C	11.65657800	1.51821200	5.83589300
H	11.29642900	-1.54292300	7.51013000
N	12.84636800	-1.39981800	6.09006700
C	13.12604000	3.75584000	6.65286600
H	13.76276900	2.56029900	8.32737700
C	11.58538900	2.68650100	5.09882100
H	11.08925800	0.64949000	5.52505000
S	12.27196100	-2.81822800	5.32088000
C	12.33656100	3.79750000	5.49980800
H	13.67775200	4.63221500	6.95446600
H	10.96037800	2.74282800	4.21458600
O	12.60494000	-2.65175800	3.89940400
O	10.86715200	-3.07265900	5.68522500
C	13.31572500	-4.08147900	6.02321800
H	12.30510300	4.70704100	4.91221200
H	14.35050400	-3.77897500	5.87783300
H	13.08184100	-4.20471400	7.07905000
H	13.10792200	-5.00320800	5.48060100

Structure IV

Rh	13.24064900	-0.09442600	9.83738000
Rh	13.11232000	-0.00529500	12.30063400
O	15.08684200	0.78892900	10.06653100
O	15.02199500	0.78622000	12.33364600
O	15.09056400	3.63866900	11.34340500
O	18.46279500	1.81866600	8.88497900
N	16.89171000	2.80486100	10.22286700
C	15.56285400	1.04030600	11.22413800
C	16.96330400	1.65205600	11.14765200
C	17.75447000	1.90575600	12.46007800
C	17.96954300	0.53794100	13.13927500
H	17.02490800	0.11067100	13.47850500
H	18.62748400	0.65712200	14.00605700
H	18.43591300	-0.17577200	12.45243200
C	17.07327600	2.86878200	13.44506900
H	16.97413600	3.87220300	13.02546400
H	17.68452600	2.94192100	14.35160800
H	16.07930000	2.51473300	13.71892000
C	19.13105200	2.47832600	12.06926600
H	19.65110100	1.82454100	11.36299400
H	19.75255500	2.58471100	12.96381600
H	19.03594900	3.46630200	11.60848200
C	15.88776700	3.75689900	10.41724300
C	15.81646300	4.87809500	9.46290800
C	16.56803600	4.86900400	8.26703700
C	17.48098400	3.81088500	8.05681100
C	17.67131700	2.73954200	9.05829400
C	18.18217100	3.73228600	6.87557600
H	18.86844900	2.90920800	6.72644600
C	18.04072600	4.72362400	5.88522200

C	17.16952000	5.80612500	6.07356700
C	16.35862000	5.87760600	7.26546600
C	15.31436300	6.84863000	7.50336000
C	14.63842600	6.86667200	8.73098700
C	14.91463700	5.88627100	9.70382700
H	14.34247500	5.86800500	10.62198300
C	18.86981100	4.55170400	4.65532400
C	19.88830800	5.45200400	4.31631900
H	20.04933400	6.33107400	4.93096200
C	20.68812700	5.22353600	3.19737000
H	21.47765900	5.92648200	2.94954000
C	20.48181300	4.09395200	2.40015300
H	21.10917000	3.91785000	1.53182800
C	19.46856900	3.19205700	2.72862800
H	19.30656300	2.30150400	2.12970200
C	18.67193900	3.42186800	3.85028700
H	17.89436800	2.71439200	4.11252900
C	17.17324600	6.85733200	5.01736500
C	17.64866700	8.14254100	5.29935600
H	17.94658800	8.38904700	6.31351200
C	17.73924900	9.09958400	4.28913700
H	18.10993800	10.09315900	4.52155700
C	17.35061900	8.78386300	2.98604400
H	17.41709000	9.53130200	2.20146900
C	16.87402200	7.50299400	2.69715900
H	16.56157900	7.24994200	1.68861600
C	16.79519900	6.54657100	3.70467600
H	16.43145000	5.55118100	3.47631200
C	14.80650000	7.77948100	6.45724900
C	14.83336700	9.16541400	6.65336800
H	15.32851100	9.56829800	7.53071900
C	14.21855300	10.02048500	5.74184200
H	14.25057100	11.09359600	5.90419700
C	13.55387000	9.49840100	4.62971600
H	13.06430400	10.16401000	3.92518900
C	13.52360700	8.11769300	4.42438800
H	13.01468000	7.69989000	3.56384700
C	14.15252400	7.26695900	5.33098700
H	14.13444800	6.19622300	5.16189800
C	13.52860700	7.80574600	9.06872300
C	13.60049700	8.53907100	10.26149700
H	14.48990200	8.46160400	10.88021700
C	12.54964700	9.36931800	10.65529600
H	12.62704000	9.93407600	11.57939600
C	11.40496700	9.46883100	9.86442700
H	10.58383800	10.11032900	10.16950600
C	11.31911700	8.73164700	8.68006200
H	10.42615200	8.79232200	8.06508300
C	12.36925700	7.90745800	8.28463000
H	12.28844100	7.32728200	7.37551100
O	11.35247200	-0.89171200	9.82989300
O	11.22942200	-0.82158900	12.09498400
O	11.05615700	-3.69880700	10.70882900
O	7.73148500	-1.35554100	8.64794800
N	9.28644000	-2.60734800	9.77063900
C	10.76865400	-1.08230500	10.95049100
C	9.33039600	-1.56704400	10.81451100
C	8.52932700	-1.90360100	12.10772900
C	8.34435500	-0.59279100	12.90059600
H	9.29727900	-0.21201600	13.26928100
H	7.68635400	-0.77156400	13.75710100
H	7.88403400	0.18174400	12.27726000
C	9.19412600	-2.96429100	12.99976700
H	9.27389800	-3.92617200	12.48895400
H	8.58515200	-3.10825900	13.89918300
H	10.19516900	-2.65541800	13.30217900
C	7.13482400	-2.40862700	11.68785600
H	6.62332200	-1.68703700	11.04483800
H	6.52237100	-2.56990900	12.58030600
H	7.19699000	-3.35972500	11.15057000
C	10.22492000	-3.65078100	9.80712700
C	10.15364600	-4.66274400	8.73306700
C	9.19648900	-4.54722200	7.69847100
C	8.34296900	-3.42085200	7.66449200

C	8.41379200	-2.37549000	8.69969000
C	7.43412400	-3.27060300	6.63927300
H	6.80836400	-2.38835500	6.62227300
C	7.24355800	-4.29106500	5.68825800
C	8.01583100	-5.45720600	5.74021000
C	9.11171600	-5.54342300	6.67502100
C	10.13855800	-6.54952100	6.62603900
C	11.08551100	-6.63330600	7.65484100
C	11.05360600	-5.70735000	8.72178700
H	11.81146100	-5.75831100	9.49446600
C	6.14426000	-4.08771900	4.69944400
C	4.93710200	-4.79155400	4.79573500
H	4.81702400	-5.53869100	5.57328300
C	3.90136700	-4.54013300	3.89694000
H	2.96886000	-5.08956300	3.98311300
C	4.05977100	-3.58171600	2.89208000
H	3.25110700	-3.38475400	2.19499900
C	5.25972600	-2.87593800	2.79138500
H	5.39014300	-2.11982000	2.02488600
C	6.29607400	-3.12697100	3.69164500
H	7.22611200	-2.57215800	3.61411500
C	7.55035800	-6.62826500	4.94581800
C	7.09786200	-7.76046600	5.63707800
H	7.18864600	-7.79245900	6.71837200
C	6.52635400	-8.83010800	4.94957700
H	6.17489300	-9.69754300	5.49985200
C	6.40813900	-8.78453400	3.55967900
H	5.96508100	-9.61713000	3.02190900
C	6.86485100	-7.66289800	2.86316200
H	6.78305500	-7.62291400	1.78127700
C	7.42495600	-6.59001300	3.55159500
H	7.77111400	-5.71765400	3.00894300
C	10.29017900	-7.41380000	5.42349100
C	10.22569100	-8.80960000	5.50875500
H	10.02637800	-9.27399400	6.46858100
C	10.40841800	-9.59636000	4.37416600
H	10.34762600	-10.67735800	4.45370200
C	10.66111000	-8.99829200	3.13722900
H	10.79821400	-9.61170600	2.25216000
C	10.73458900	-7.60777400	3.04542800
H	10.92152200	-7.13199000	2.08759200
C	10.55951900	-6.82279600	4.18283500
H	10.62693800	-5.74324600	4.10748500
C	12.20444900	-7.61640400	7.64697700
C	12.45301300	-8.39856300	8.78313700
H	11.80212800	-8.30660900	9.64776000
C	13.52041500	-9.29711600	8.80803300
H	13.69617800	-9.89853600	9.69487700
C	14.35844300	-9.42199000	7.69851600
H	15.18970800	-10.12034400	7.71890100
C	14.12386000	-8.64130700	6.56366300
H	14.77545200	-8.71388000	5.69835100
C	13.05710300	-7.74578600	6.54098600
H	12.89391800	-7.13349600	5.66550400
O	12.40619400	1.75118000	9.81828900
O	12.24396000	1.89024900	12.07110500
O	9.42732900	2.04661200	10.24158600
O	12.45351500	4.85304400	8.35157400
N	10.77636600	3.66935900	9.36807400
C	12.08001200	2.33770700	10.90678200
C	11.54154900	3.74254900	10.63648800
C	10.90433200	4.57490300	11.78181900
C	12.01438900	4.86176100	12.81496300
H	12.35608700	3.94367800	13.29474300
H	11.62954000	5.53728400	13.58545400
H	12.88026300	5.34099400	12.34748000
C	9.72242400	3.89927000	12.49411500
H	8.86403300	3.77769200	11.83224300
H	9.41555800	4.52094700	13.34262100
H	10.00133300	2.91310300	12.87001000
C	10.44977800	5.91552500	11.17375900
H	11.27218300	6.42598900	10.66393900
H	10.08180300	6.57473600	11.96615000
H	9.64085800	5.77326100	10.45116600

C	9.74214500	2.72287600	9.26814100
C	9.07530700	2.58353900	7.95606800
C	9.63785200	3.17187500	6.79993000
C	10.79861000	3.96575300	6.92427100
C	11.41043800	4.21408300	8.24018100
C	11.39668200	4.49680400	5.80144600
H	12.28983600	5.09609700	5.91845900
C	10.81960200	4.34127800	4.52470200
C	9.62613000	3.62225600	4.38149800
C	9.05824100	2.93773600	5.51398700
C	7.94587300	2.02751100	5.42703600
C	7.32896000	1.55907500	6.59557700
C	7.92448400	1.83210000	7.85225000
H	7.51185400	1.37782900	8.74391800
C	11.54070200	4.97197200	3.38031500
C	10.96236200	5.94612100	2.55409900
H	9.93737400	6.25379600	2.72399900
C	11.69615500	6.51610900	1.51397300
H	11.23504000	7.27111900	0.88469600
C	13.01858100	6.12670000	1.28472700
H	13.58702300	6.57351200	0.47506500
C	13.60531700	5.16284100	2.10614300
H	14.62973100	4.84808000	1.93362900
C	12.87157400	4.59265800	3.14672400
H	13.32321300	3.83267600	3.77670600
C	8.90192200	3.72405500	3.08208800
C	7.76380500	4.53945500	3.02226700
H	7.39940400	5.00904900	3.93089500
C	7.10907800	4.75173700	1.81019900
H	6.23106400	5.38953000	1.77574900
C	7.58048600	4.14169400	0.64677800
H	7.06923400	4.30014900	-0.29779500
C	8.71247700	3.32544300	0.70192400
H	9.08142100	2.84509000	-0.19909300
C	9.37706000	3.12745800	1.91018800
H	10.26231700	2.50234100	1.95179300
C	7.57534000	1.42649400	4.11273500
C	6.37995400	1.70274600	3.44340400
H	5.69038000	2.42837600	3.85973700
C	6.08605600	1.06401000	2.24071200
H	5.16411000	1.29997700	1.71864800
C	6.97255300	0.12680900	1.70453900
H	6.74315500	-0.36318500	0.76325600
C	8.15727300	-0.17254300	2.37971100
H	8.85069600	-0.90184200	1.97188500
C	8.45706000	0.47789000	3.57571100
H	9.38082600	0.25904900	4.10255100
C	6.05131700	0.78844000	6.61455900
C	5.06870500	1.18928300	7.53605800
H	5.25984200	2.04343200	8.17837400
C	3.84906700	0.52041100	7.62688600
H	3.10592500	0.85390000	8.34483300
C	3.58618200	-0.56866600	6.79512200
H	2.63793000	-1.09326100	6.86223900
C	4.55207100	-0.97916400	5.87495400
H	4.36507500	-1.82920400	5.22848200
C	5.77111900	-0.30743800	5.78363100
H	6.50829900	-0.65065400	5.07179700
O	13.99988500	-1.97387900	10.08259100
O	14.01373900	-1.87802600	12.34380600
O	16.88253800	-1.78375900	10.58252200
O	14.66598900	-5.51686300	9.18315000
N	15.85751400	-3.71501900	9.93462700
C	14.31459900	-2.40769400	11.24081500
C	15.05938600	-3.74161400	11.18110700
C	15.76945700	-4.28032200	12.45616000
C	14.66654500	-4.67138700	13.46208200
H	14.09436900	-3.79831800	13.78070700
H	15.11987600	-5.12973600	14.34666500
H	13.97320700	-5.39912700	13.02545200
C	16.73621300	-3.29128700	13.12561500
H	17.58404400	-3.05821900	12.48101700
H	17.11667700	-3.73728200	14.05133600
H	16.23469800	-2.35469800	13.37338200
C	16.53859800	-5.55280900	12.05432900
H	15.88319300	-6.27512100	11.55814800
H	16.96025400	-6.02591500	12.94666200
H	17.36285300	-5.32025300	11.37380800
C	16.67197400	-2.60086600	9.69131700
C	17.23616500	-2.44613900	8.33399700
C	16.85189000	-3.30794600	7.27934500
C	15.98528700	-4.38731500	7.57289500
C	15.45013500	-4.60641700	8.92971300
C	15.62519200	-5.27603200	6.58557400
H	14.98049900	-6.10797000	6.83458900
C	16.10665600	-5.11860700	5.27522300
C	16.91993300	-4.03440300	4.93076200
C	17.33963900	-3.09322900	5.94292900
C	18.26332500	-2.00281400	5.73474300
C	18.70115600	-1.22969000	6.82328000
C	18.14406000	-1.43631800	8.10448600
H	18.42482600	-0.77864400	8.91711900
C	15.78892100	-6.20361100	4.29871200
C	16.76816100	-7.16344000	4.00891800
H	17.75188700	-7.06912400	4.45812800
C	16.48502600	-8.23270700	3.15950200
H	17.25201300	-8.97160800	2.94738900
C	15.21590000	-8.35193700	2.58919400
H	14.98923400	-9.18481100	1.93038000
C	14.23945600	-7.39478400	2.86927000
H	13.25159500	-7.48587200	2.43440500
C	14.51999800	-6.32546700	3.72137800
H	13.76663700	-5.57359600	3.93109800
C	17.25500200	-3.94027500	3.47814600
C	18.50556200	-4.30743900	2.97348700
H	19.29177500	-4.60675200	3.65804800
C	18.74714500	-4.27409500	1.60132300
H	19.72651800	-4.54834600	1.22141400
C	17.73556600	-3.88930700	0.71895600
H	17.92667100	-3.86330600	-0.34967500
C	16.47747600	-3.54487400	1.21611100
H	15.68275600	-3.25411200	0.53535000
C	16.23534800	-3.57597800	2.58822800
H	15.25709400	-3.31922700	2.98245000
C	18.81521600	-1.64814400	4.39465600
C	20.17960100	-1.81209200	4.12791900
H	20.81542800	-2.27037600	4.87813200
C	20.72338700	-1.37664700	2.92161400
H	21.78335100	-1.51075300	2.72890500
C	19.90917300	-0.76496000	1.96706700
H	20.33135200	-0.42340200	1.02696300
C	18.54553800	-0.60259700	2.22246000
H	17.90108700	-0.14290700	1.47963500
C	18.00446100	-1.03888900	3.43025900
H	16.94279100	-0.92855100	3.61923900
C	19.80319100	-0.22563100	6.73759600
C	20.83302500	-0.30629600	7.68861700
H	20.80114000	-1.08870300	8.44058400
C	21.90161200	0.58867500	7.67080500
H	22.68760900	0.50237100	8.41495100
C	21.96153200	1.58476200	6.69578100
H	22.79417400	2.28132600	6.67427900
C	20.94346100	1.67827000	5.74494800
H	20.97371200	2.45162300	4.98551900
C	19.87313000	0.78519900	5.76864500
H	19.09211400	0.87349500	5.02587400
H	14.27783200	-4.47246800	10.95505600
H	8.81940000	-0.71637400	10.35522300
H	12.44135700	4.29124100	10.34766100
H	17.53515100	0.90769200	10.59183900
C	13.68616300	-0.25491200	7.87973300
C	14.36766900	0.73330300	7.10764300
C	13.38105700	-1.53996600	7.28552500
C	14.14631700	2.10688000	7.38754200
C	15.16029200	0.38330600	5.97674400
H	13.46619400	-2.44092700	7.89756300
N	12.88633300	-1.54789100	6.08566400
C	14.58622300	3.08033100	6.50538500

H	13.57955500	2.37964300	8.26507800
C	15.64886600	1.37081300	5.13809700
H	15.37510800	-0.65918500	5.77579800
S	12.39476200	-3.06542700	5.46929700
C	15.33545300	2.71522000	5.38321900
H	14.35291800	4.11730900	6.70568600
H	16.25496700	1.10225600	4.28036800
O	12.46824100	-4.13563400	6.47537200
O	13.12719000	-3.23909200	4.20179700
C	10.68231500	-2.69013800	5.09862100
H	15.69058500	3.48134200	4.70346300
H	10.18026600	-2.40503300	6.02120800
H	10.66417600	-1.87800600	4.37322900
H	10.22647400	-3.59100000	4.68763500

Structure TS-C4

Rh	-0.63821400	-0.03149400	-5.07870400
Rh	-0.78087500	-0.28763700	-2.65167400
C	1.56567800	-1.49800500	-3.86652900
C	2.95841200	-2.11768100	-3.73756600
H	3.52834300	-1.31168500	-3.27303200
C	3.75798600	-2.50656800	-5.01466100
C	4.10661200	-1.20064600	-5.75984400
H	3.20747300	-0.70699500	-6.13076800
H	4.63044200	-0.49679500	-5.10582900
H	4.75751200	-1.42812000	-6.61025600
C	3.02254900	-3.45320600	-5.97633300
H	2.84147100	-4.43005000	-5.52565100
H	2.06145200	-3.03507000	-6.27966800
H	3.63736300	-3.59833000	-6.87177100
C	5.07225800	-3.16744100	-4.55559700
H	5.62322300	-2.53001800	-3.85824500
H	4.88791200	-4.12631700	-4.06185800
H	5.71196800	-3.35536600	-5.42370600
C	3.60053800	-2.87930500	-1.49817900
C	3.33303800	-3.74336900	-0.33289200
C	3.95956300	-3.47069600	0.86472100
H	4.69201100	-2.67393600	0.89145600
C	3.57946000	-4.12640700	2.06009800
C	2.58710800	-5.11744400	2.01109400
C	1.91394500	-4.14276900	-2.78389600
C	1.76712400	-5.07472300	-1.64752000
C	0.92955100	-6.16090700	-1.76373900
H	0.45149500	-6.34712900	-2.71776400
C	0.71417100	-7.04607000	-0.67951700
C	1.29543000	-6.76462800	0.56568600
C	2.40570200	-4.80414900	-0.41730500
C	2.09638600	-5.58138700	0.73774500
C	4.26954300	-3.68499000	3.30899900
C	3.60907900	-3.40198000	4.51700400
H	2.53277200	-3.48529700	4.57971200
C	4.32833200	-2.99392200	5.64041000
H	3.79691200	-2.77861800	6.56206700
C	5.71575400	-2.84548500	5.58154600
C	6.38109800	-3.09143400	4.37929900
H	7.45687800	-2.96394900	4.31102600
C	5.66416400	-3.51015600	3.25952300
H	6.19247000	-3.72566400	2.33644900
C	1.88103600	-5.52054600	3.26034100
C	2.44998400	-6.30270500	4.26869500
H	3.44596200	-6.70907100	4.13409900
C	1.73138100	-6.57169400	5.43174500
H	2.17328800	-7.19492900	6.20291100
C	0.44939800	-6.04442200	5.60903200
C	-0.11823500	-5.25070200	4.61085900
H	-1.11397000	-4.83538700	4.73679800
C	0.59377900	-4.99339500	3.44081800
H	0.15257600	-4.38772900	2.65676800
C	1.17461700	-7.76159800	1.66715200
C	2.28674000	-8.55246600	1.98356400
H	3.22227800	-8.38865200	1.45739000
C	2.19189200	-9.54694500	2.95650200
H	3.05809200	-10.15915100	3.18828700
C	0.98782600	-9.74941400	3.63270300
C	-0.12086500	-8.95631800	3.32745700
H	-1.06004400	-9.10234500	3.85122500
C	-0.03079100	-7.97708700	2.34154100
H	-0.89160900	-7.36405700	2.10476400
C	-0.19372600	-8.20273700	-0.93123100
C	0.09588300	-9.51742200	-0.53064700
H	1.01849900	-9.73275200	-0.00748800
C	-0.79318000	-10.55487900	-0.80798500
H	-0.54922200	-11.56441400	-0.49111300
C	-1.98560300	-10.30480700	-1.49154500
C	-2.27470600	-9.00635100	-1.91025500
H	-3.19099800	-8.78814000	-2.45002000
C	-1.38668700	-7.96740500	-1.63538900
H	-1.64678400	-6.96757100	-1.96414200
N	2.89426600	-3.14965700	-2.67974400
O	1.14762800	-1.10480300	-4.98918700
O	0.97453600	-1.35342100	-2.74339900
O	4.37471700	-1.92999000	-1.43020800
O	1.18619600	-4.19795800	-3.76953600
C	0.70940600	2.04277500	-3.54581800
C	1.50541700	3.34251600	-3.35327400
H	0.82935400	3.99869600	-2.80260000
C	1.94243500	4.12515200	-4.63526500
C	0.68114700	4.53992200	-5.41976600
H	0.17869200	3.67741800	-5.85471900
H	-0.03783000	5.05261200	-4.77504700
H	0.96736000	5.22543200	-6.22403800
C	2.88446000	3.32826700	-5.55411900
H	3.84251900	3.12135300	-5.07028300
H	2.43925100	2.38010100	-5.85403700
H	3.09040300	3.92212300	-6.45137900
C	2.66449400	5.41642500	-4.19952400
H	2.02698700	6.04560100	-3.57403100
H	3.58714500	5.20541000	-3.65045300
H	2.93858500	5.99097000	-5.08962500
C	2.79663000	4.01352500	-1.36128200
C	4.03515200	3.90510700	-0.56585500
C	4.30059700	4.85098500	0.40317000
H	3.58107300	5.64424700	0.56863400
C	5.53155700	4.86195800	1.09623300
C	6.47062400	3.84519800	0.86867100
C	3.45620900	1.99491300	-2.61931700
C	4.66224200	1.88405700	-1.77820600
C	5.48547700	0.78951900	-1.92664300
H	5.23778800	0.03871100	-2.66220100
C	6.56076600	0.56127500	-1.04090000
C	6.88764800	1.51842400	-0.07239400
C	4.95155500	2.85377300	-0.79149000
C	6.13300400	2.74263000	0.00755200
C	5.76683100	6.00328400	2.02617700
C	4.80805200	6.28196500	3.01260100
H	3.92588900	5.65369800	3.09258100
C	4.97915500	7.34820200	3.89616300
H	4.23200100	7.53871200	4.66037300
C	6.10643600	8.16428400	3.79470000
C	7.05498400	7.90979300	2.80115500
H	7.92820800	8.54803100	2.70658500
C	6.88897400	6.83925400	1.92467500
H	7.63185600	6.64922600	1.15930100
C	7.85510100	3.99962600	1.39690300
C	8.89336300	4.20952900	0.47893600
H	8.67512000	4.18663100	-0.58435100
C	10.19184500	4.45543100	0.92335500
H	10.98539500	4.62329600	0.20162600
C	10.46961800	4.48025200	2.29126500
C	9.44058000	4.26008600	3.21007300
H	9.64521200	4.26202100	4.27617200
C	8.14149800	4.03249800	2.76539000
H	7.34955300	3.85959800	3.48046100
C	7.91614800	1.15419100	0.94184600
C	9.24407300	0.90488200	0.57689300
H	9.55496200	1.07366300	-0.44869500
C	10.15827500	0.43972700	1.51933400
H	11.18783100	0.25772000	1.22657400
C	9.74995300	0.20087100	2.83392300

C	8.42635300	0.44525900	3.20305200
H	8.10148000	0.26887900	4.22221500
C	7.51565700	0.92608500	2.26398100
H	6.48698100	1.11110200	2.54770100
C	7.23561500	-0.76356300	-1.14807300
C	7.74437200	-1.18042400	-2.38431200
H	7.70000900	-0.50501100	-3.23367200
C	8.30803900	-2.44908400	-2.53325500
H	8.70023700	-2.75341100	-3.49876100
C	8.36282300	-3.32160800	-1.44629400
C	7.85354800	-2.91508400	-0.20968700
H	7.89322200	-3.58908400	0.64110900
C	7.29413200	-1.64917000	-0.06083200
H	6.89779200	-1.34469500	0.90066800
N	2.63219100	3.11005600	-2.42045500
O	0.42780400	1.68949800	-4.72406600
O	0.33440800	1.46994700	-2.47064500
O	1.94860500	4.87147400	-1.12853300
O	3.16498400	1.15507800	-3.46542600
C	-2.93576700	1.24781800	-3.84848700
C	-4.24998700	2.03493900	-3.77028600
H	-4.94378500	1.34552900	-3.28575800
C	-4.92351000	2.48557500	-5.10211700
C	-5.27644300	1.22185100	-5.90942400
H	-4.37968400	0.70598100	-6.25037300
H	-5.86303500	0.52028000	-5.30734400
H	-5.87335200	1.49923500	-6.78438700
C	-4.05405900	3.42397900	-5.95589400
H	-3.86518500	4.37265400	-5.44703600
H	-3.09398900	2.96740100	-6.19542500
H	-4.58222400	3.64880700	-6.88919200
C	-6.23766300	3.21417900	-4.75672200
H	-6.89449800	2.59086700	-4.14402100
H	-6.05207000	4.14693700	-4.21567300
H	-6.76557200	3.46769700	-5.68131900
C	-5.03914900	3.24123700	-1.76454500
C	-4.90348600	4.39054300	-0.84546000
C	-5.78766000	4.52360200	0.20308200
H	-6.60018800	3.81380500	0.30282000
C	-5.56182900	5.47714300	1.22192000
C	-4.47310700	6.35002300	1.14412800
C	-2.96267400	3.95374700	-2.89854200
C	-2.90753300	5.14412000	-2.03349900
C	-1.88977000	6.05706700	-2.21275900
H	-1.18236900	5.90754600	-3.01911000
C	-1.78990700	7.20456700	-1.39972300
C	-2.67457800	7.37611600	-0.32685100
C	-3.83792000	5.30944900	-0.98498900
C	-3.66320500	6.37032400	-0.04366500
C	-6.48288300	5.46106000	2.39385400
C	-6.03369100	5.13522000	3.68138600
H	-4.98413600	4.91664500	3.84081700
C	-6.92771500	5.08454600	4.74881800
H	-6.56756100	4.82204700	5.73884700
C	-8.28251600	5.36384300	4.54711000
C	-8.73793700	5.68966900	3.26843000
H	-9.78856600	5.90707100	3.10196700
C	-7.84287300	5.73271000	2.19791800
H	-8.19671700	5.98051300	1.20161400
C	-4.07367700	7.10068400	2.36827400
C	-4.86684900	8.10792800	2.92772500
H	-5.77001700	8.42023100	2.41490300
C	-4.50019900	8.70739600	4.13046800
H	-5.11921200	9.49484300	4.54951400
C	-3.34355200	8.29568700	4.79710900
C	-2.55314400	7.28312400	4.25072800
H	-1.65764700	6.95023600	4.76668400
C	-2.91431100	6.69017700	3.04172000
H	-2.31323000	5.88324200	2.63340300
C	-2.64537700	8.66956100	0.41064100
C	-3.72335600	9.55301200	0.27061200
H	-4.59019300	9.24518300	-0.30614600
C	-3.68212300	10.81737000	0.85605300
H	-4.52168500	11.49520900	0.73540500

C	-2.56491500	11.20838100	1.59581300
C	-1.48927300	10.32915800	1.74439000
H	-0.61933400	10.62561600	2.32240700
C	-1.52646900	9.07030000	1.14949900
H	-0.68732500	8.39097100	1.25401300
C	-0.72679400	8.19228500	-1.74254000
C	-1.02792600	9.51872200	-2.08249700
H	-2.05890500	9.85429800	-2.06954500
C	-0.01040800	10.40613300	-2.42894800
H	-0.25801900	11.42999100	-2.69276700
C	1.32128800	9.98249000	-2.43935700
C	1.62741900	8.66211900	-2.10667000
H	2.65828200	8.32029400	-2.10989800
C	0.60984000	7.76988800	-1.76462000
H	0.85756100	6.74801700	-1.49980700
N	-4.09233600	3.13987600	-2.80080500
O	-2.42654500	0.99417000	-4.97793100
O	-2.50879900	0.84850500	-2.71708600
O	-5.93103000	2.40657900	-1.65053200
O	-2.05501700	3.67452200	-3.67752300
C	-2.19335000	-2.29813100	-4.24850100
C	-3.13104000	-3.50173000	-4.39331200
H	-2.47151700	-4.37233400	-4.33235900
C	-3.91472900	-3.66729800	-5.73805300
C	-2.90549300	-4.12135600	-6.81292000
H	-2.13983800	-3.36308200	-6.98224800
H	-2.41168900	-5.05465200	-6.51902800
H	-3.42919800	-4.30346700	-7.75688200
C	-4.62729200	-2.39193200	-6.21302600
H	-5.39574100	-2.07611400	-5.50721600
H	-3.92313500	-1.56894400	-6.33414800
H	-5.10225200	-2.58711200	-7.18091800
C	-4.96032900	-4.78418400	-5.55969000
H	-4.50026100	-5.71483900	-5.21797300
H	-5.73206100	-4.49877800	-4.83863600
H	-5.45337000	-4.97409200	-6.51817200
C	-3.87779000	-4.82273200	-2.48036100
C	-4.51678000	-4.89207800	-1.15339000
C	-4.50746000	-6.09736200	-0.49396700
H	-4.10624500	-6.96672200	-0.99789400
C	-4.96755900	-6.20651200	0.83295800
C	-5.45795600	-5.07624900	1.50315900
C	-4.55704600	-2.45971700	-2.64673200
C	-5.04436000	-2.52341400	-1.25260800
C	-5.49396400	-1.36352600	-0.65937200
H	-5.48850400	-0.43351000	-1.21728900
C	-5.94665600	-1.37745400	0.67318000
C	-5.97849200	-2.56579900	1.41543700
C	-5.03684900	-3.73756200	-0.52470500
C	-5.51954500	-3.80000500	0.82827900
C	-4.80849400	-7.54471700	1.47264200
C	-3.57655000	-8.20661500	1.34697500
H	-2.76568400	-7.73332700	0.80504200
C	-3.38038500	-9.47270600	1.89852700
H	-2.41823400	-9.95927900	1.78242100
C	-4.41998400	-10.10198300	2.58402000
C	-5.65618100	-9.46049300	2.70183000
H	-6.47527600	-9.94926400	3.22078700
C	-5.85090300	-8.19608400	2.14933300
H	-6.81514800	-7.71173500	2.24232700
C	-5.78620700	-5.25845700	2.94948600
C	-7.10610900	-5.32007800	3.40870300
H	-7.92087400	-5.17364000	2.70857700
C	-7.37485300	-5.55597000	4.75483100
H	-8.40371100	-5.58829600	5.09889800
C	-6.32700500	-5.74595800	5.65855400
C	-5.00811700	-5.70597000	5.20579700
H	-4.18626200	-5.86227300	5.89812300
C	-4.74156600	-5.47011700	3.85827900
H	-3.71545000	-5.46058300	3.50709800
C	-6.57204700	-2.44622400	2.78382900
C	-7.95984500	-2.30177000	2.90372200
H	-8.57594600	-2.34945000	2.01154900
C	-8.54760600	-2.09519100	4.15021800

H	-9.62541200	-1.98795500	4.22686000
C	-7.75156100	-2.02345700	5.29410900
C	-6.36583100	-2.14968500	5.18015800
H	-5.73808500	-2.08917200	6.06372700
C	-5.78047000	-2.34972000	3.93208900
H	-4.70322200	-2.43026700	3.84962700
C	-6.43216300	-0.09633100	1.26266900
C	-7.63039100	0.46589800	0.80744500
H	-8.17062500	-0.01665400	-0.00142600
C	-8.14785100	1.61093000	1.41364600
H	-9.09106000	2.02498400	1.06973400
C	-7.46097400	2.21646000	2.46654400
C	-6.23604300	1.69289300	2.88643200
H	-5.68551500	2.18146600	3.68361900
C	-5.72558000	0.54178100	2.29034700
H	-4.78597300	0.12096600	2.63273200
N	-3.93667500	-3.60816100	-3.16147100
O	-1.75143800	-1.75636000	-5.30156200
O	-1.87609900	-1.99419000	-3.05621900
O	-3.29514300	-5.78992100	-2.97233400
O	-4.65299500	-1.44754000	-3.33033900
C	-0.90332000	-0.69205100	-0.43036300
C	-1.42986700	-1.97625700	0.06345600
C	-1.46250000	0.52704400	0.09228600
H	0.26495700	-0.68133900	-0.47155800
C	-1.03994000	-3.18665300	-0.53820700
C	-2.19802500	-2.02515200	1.24333100
H	-2.54816600	0.62465500	-0.02052100
N	-0.75513800	1.47612400	0.61965600
C	1.28826300	-0.86713800	0.57641900
C	-1.39150100	-4.40035500	0.04002400
H	-0.46608000	-3.15959800	-1.45476000
C	-2.53297200	-3.24477000	1.82491100
H	-2.49572500	-1.10271900	1.73120000
S	-1.60443100	2.85837900	1.09672400
C	2.20807500	0.21342400	0.07832000
C	0.81672100	-0.81809100	1.99778300
H	1.47881800	-1.86953900	0.19295700
C	-2.11772400	-4.43511400	1.23137900
H	-1.09428600	-5.32469600	-0.43115200
H	-3.10167100	-3.26753800	2.74656300
O	-1.35034000	3.06107300	2.53561200
O	-3.00952900	2.81592500	0.64612400
C	-0.69058700	4.08453200	0.18158900
C	3.35234300	0.42450200	1.09765200
H	1.64788900	1.14683900	-0.00695700
H	2.59645700	-0.04972300	-0.90433800
C	2.01533900	-0.57586200	2.94508800
H	0.13394500	0.02497700	2.12923600
H	0.29766500	-1.73985600	2.26418600
H	-2.36773500	-5.38814200	1.68227100
H	0.34144000	4.06729800	0.52454900
H	-0.73680000	3.82897700	-0.87415600
H	-1.14568600	5.05630700	0.37070500
C	2.77882100	0.67654100	2.50182300
H	4.01731400	-0.44516700	1.11644700
H	3.94896600	1.27429400	0.77037600
H	1.63235100	-0.47123000	3.96491900
H	2.68013900	-1.44353400	2.93735000
H	2.05166300	1.49506100	2.41177400
H	-4.27294900	-11.08715600	3.01614300
H	-6.53914400	-5.92746500	6.70772300
H	-8.20625800	-1.86484100	6.26718500
H	-7.87003800	3.09386000	2.95064900
H	-3.06265200	8.75975000	5.73763900
H	8.79832100	-4.30956100	-1.55984300
H	10.46053700	-0.17040100	3.56612600
H	11.48131100	4.66553500	2.63898000
H	6.24303800	8.99477300	4.48034400
H	6.27033800	-2.53267000	6.46078200
H	-0.10333000	-6.25478800	6.51948000
H	0.91353400	-10.51810100	4.39572600
H	2.11222900	10.67709300	-2.70576200
H	-2.53154400	12.19189900	2.05447100
H	-8.97722700	5.32501500	5.38046500
H	-2.67769700	-11.11525300	-1.69798500
C	3.84618800	1.13956700	3.46665300
C	4.13793400	2.50729000	3.55503300
C	4.57228100	0.25137900	4.26769700
C	5.12245400	2.98586900	4.41639000
H	3.58632900	3.20984600	2.93721700
C	5.56113500	0.71060200	5.13839200
H	4.37554700	-0.81178800	4.22463700
C	5.83421300	2.07415000	5.19337600
H	5.32950300	4.04750300	4.47776000
H	6.11251800	0.00961600	5.75299100
Br	7.23855900	2.71011200	6.32891700

Structure TS-C4’

Rh	13.90719200	13.11437300	0.68400800
Rh	14.03058700	13.11783900	3.12771700
C	16.13720900	11.62392100	1.80490100
C	17.39531600	10.75023900	1.87473900
H	18.12409700	11.38584100	2.38302700
C	18.07149500	10.28043500	0.55338500
C	18.56220200	11.53606500	-0.19287700
H	17.72596600	12.15231700	-0.52251500
H	19.20914000	12.14862900	0.44429800
H	19.14221500	11.23825700	-1.07230600
C	17.15978400	9.46035500	-0.37433100
H	16.87160800	8.50883400	0.07703500
H	16.25088900	10.01009500	-0.61953400
H	17.70086900	9.24120600	-1.30164400
C	19.30074000	9.42897100	0.92731000
H	19.97682500	9.97240700	1.59411400
H	19.00903400	8.49853200	1.42361500
H	19.85270500	9.16413200	0.02017100
C	17.99385800	9.57994700	3.96148200
C	17.74446300	8.47901300	4.91925500
C	18.55612300	8.37819900	6.02793100
H	19.35562200	9.09634400	6.16682300
C	18.31532800	7.39526000	7.00689300
C	17.25314900	6.49072800	6.87147700
C	16.00703600	8.86949600	2.69241500
C	15.85516500	7.71267500	3.59560700
C	14.86605500	6.79339400	3.33092100
H	14.26040800	6.90661000	2.44088900
C	14.63305300	5.71414800	4.20475700
C	15.34370900	5.60828300	5.40664100
C	16.68527100	7.55962600	4.73113800
C	16.42222500	6.52451600	5.69435300
C	19.21548800	7.37960900	8.19653500
C	18.73323600	7.65215900	9.48407000
H	17.68050100	7.86817600	9.62636300
C	19.59304000	7.63644700	10.58026600
H	19.20503100	7.85012100	11.57151300
C	20.94987800	7.34926400	10.40553800
C	21.44197000	7.09093900	9.12475500
H	22.49538500	6.87264300	8.97877500
C	20.58003800	7.11357300	8.02682300
H	20.96115600	6.90914000	7.03080600
C	17.05855200	5.53702100	8.00346400
C	18.02071900	4.55193700	8.25544200
H	18.86491600	4.45019400	7.58133000
C	17.90255100	3.71229200	9.36135900
H	18.65404100	2.94965400	9.54196500
C	16.82160600	3.85284100	10.23270100
C	15.86180400	4.83810300	9.99163800
H	15.01075400	4.94637800	10.65578100
C	15.98406100	5.67971600	8.88909800
H	15.23038100	6.43637100	8.70213000
C	14.82560700	4.58545800	6.36583800
C	15.47563700	3.37582600	6.62563500
H	16.43087600	3.16949400	6.15551600
C	14.90986000	2.44808900	7.49817000
H	15.43013800	1.51736400	7.70193400
C	13.68484700	2.71452700	8.11455800
C	13.01948700	3.91151600	7.84599700
H	12.07020500	4.13521600	8.32258500

C	13.58492800	4.83351000	6.96863400
H	13.06924700	5.76260300	6.75231300
C	13.61371000	4.70016200	3.80522300
C	13.98616200	3.36280600	3.60651300
H	15.01687700	3.06679900	3.76993000
C	13.04581500	2.41813200	3.19861300
H	13.35046200	1.38699600	3.04636200
C	11.71823200	2.79619700	2.98230100
C	11.34081300	4.12634800	3.17330400
H	10.31119400	4.43073100	3.00906600
C	12.28237000	5.07326900	3.57885200
H	11.99300900	6.10315900	3.74619200
N	17.15008600	9.65823800	2.84321000
O	15.67341800	12.00442900	0.69129100
O	15.70643300	11.94863700	2.95660200
O	18.90053100	10.39189600	4.12334400
O	15.17673700	9.13871800	1.82844100
C	15.52814400	15.23430300	1.79722200
C	16.53154700	16.38635700	1.71703300
H	15.91459700	17.28917700	1.70578900
C	17.42163300	16.48042500	0.42568300
C	16.54230800	17.04788800	-0.70788600
H	15.71444500	16.37623500	-0.93886100
H	16.13058800	18.02565200	-0.43291400
H	17.14527000	17.18099800	-1.61204200
C	18.02646700	15.14106300	-0.02381900
H	18.72565900	14.75098600	0.71528300
H	17.25170900	14.39120900	-0.18377200
H	18.56268600	15.28948100	-0.96781100
C	18.56421000	17.47853500	0.68972700
H	18.18224500	18.44899300	1.01667900
H	19.25463900	17.10631100	1.45219500
H	19.13459000	17.62572400	-0.23276600
C	17.18910300	17.73561300	3.63576900
C	17.73565100	17.81991300	4.99748900
C	17.67009500	19.02318300	5.65737900
H	17.25406400	19.87709400	5.14263900
C	18.18521800	19.17811900	6.96061600
C	18.72531500	18.07040100	7.62739600
C	17.85007800	15.35286500	3.55028700
C	18.30150100	15.45036700	4.95477400
C	18.75226500	14.31478800	5.59635500
H	18.78363300	13.37860900	5.04965700
C	19.07834600	14.33790500	6.97154600
C	18.99817800	15.54221700	7.68962500
C	18.25292700	16.68093700	5.64919800
C	18.68174600	16.76830900	7.01005700
C	18.12709200	20.56460900	7.51023700
C	16.91776900	21.27220100	7.40513600
H	16.04656400	20.78194500	6.98325600
C	16.82539400	22.60081200	7.81882700
H	15.88170100	23.12645100	7.71638200
C	17.94253200	23.24547000	8.35210200
C	19.15126800	22.55323500	8.45850700
H	20.02915400	23.05000100	8.86081500
C	19.24588300	21.22728200	8.03866000
H	20.19300800	20.70707600	8.11405100
C	19.44334700	18.30952200	8.91014000
C	20.83695200	18.17072700	8.94299200
H	21.35396100	17.80475200	8.06121100
C	21.55550700	18.50936000	10.08870500
H	22.63602400	18.40285600	10.09879300
C	20.88627200	18.98301100	11.21848300
C	19.49550800	19.11470700	11.19524500
H	18.96834700	19.47670900	12.07275100
C	18.78010700	18.78751200	10.04592300
H	17.70153800	18.90067700	10.02130300
C	19.06443900	15.48237900	9.17770400
C	20.23074500	15.13666600	9.86697100
H	21.15308700	14.99150700	9.31470600
C	20.20796500	14.97355900	11.25035200
H	21.12086800	14.71186200	11.77670900
C	19.01433900	15.13840100	11.95726800
C	17.84628500	15.48223600	11.27366200
H	16.91545200	15.61386100	11.81759400
C	17.87047100	15.65892800	9.89088900
H	16.96854300	15.92148900	9.34709200
C	19.50025100	13.04429400	7.58400800
C	20.50383900	12.30226100	6.94136100
H	20.94329400	12.68529100	6.02560100
C	20.95981600	11.09791800	7.47727200
H	21.74618900	10.54623000	6.97088900
C	20.41463200	10.60882800	8.66435700
C	19.39628200	11.32392500	9.29700700
H	18.95356900	10.93791800	10.21030100
C	18.93937400	12.52738400	8.76310100
H	18.14819600	13.06905900	9.26616800
N	17.24700200	16.49164200	2.99835500
O	15.06618700	14.81051300	0.69734200
O	15.18310500	14.84571300	2.95683400
O	16.69129500	18.70569700	3.06643600
O	17.96939000	14.32684200	2.89016500
C	11.68331700	14.42447300	2.05701600
C	10.26059000	14.97304600	2.23009200
H	9.73179600	14.15806200	2.72980700
C	9.43039200	15.30999800	0.95461300
C	9.18638000	13.99289800	0.18926300
H	10.11937600	13.57669400	-0.19165000
H	8.71983800	13.24307900	0.83546000
H	8.51730000	14.18107000	-0.65679300
C	10.09922600	16.33556100	0.02522700
H	10.22152600	17.30419700	0.51488600
H	11.08112900	15.99128200	-0.30025800
H	9.46752500	16.48209800	-0.85802500
C	8.06126400	15.86243000	1.39847200
H	7.54254200	15.16776800	2.06462500
H	8.16219700	16.82039800	1.91779000
H	7.43470900	16.02846200	0.51662000
C	9.42479900	15.96625900	4.32686700
C	9.48628300	17.04791300	5.33837600
C	8.57424100	17.03486700	6.37126000
H	7.82472600	16.25148200	6.39459800
C	8.61106200	18.01448000	7.38558200
C	9.58948900	19.02375300	7.36130400
C	11.29503300	17.03073100	3.12576900
C	11.33324400	18.08920000	4.15300900
C	12.25219500	19.09916900	4.00827500
H	12.90477000	19.08997600	3.14462000
C	12.35459400	20.13594400	4.95820600
C	11.53249500	20.13482200	6.09371500
C	10.44683200	18.08435000	5.25523700
C	10.52604000	19.10671400	6.26530800
C	7.54494600	17.94846900	8.43013700
C	7.82126800	17.97168200	9.80617000
H	8.84773200	18.01804000	10.14592500
C	6.78955300	17.93991100	10.74059900
H	7.02639300	17.95656000	11.79975400
C	5.45893700	17.86465300	10.32164700
C	5.17137400	17.80546900	8.95710300
H	4.14230600	17.73982500	8.61719000
C	6.20647900	17.84549500	8.02068800
H	5.97392800	17.81869000	6.96063300
C	9.55668600	19.99844400	8.49152300
C	8.48755500	20.89542900	8.59838300
H	7.73087900	20.91398000	7.82057900
C	8.38843400	21.75125700	9.69337500
H	7.55641600	22.44541300	9.76188700
C	9.35479000	21.71241900	10.69920000
C	10.41870800	20.81245600	10.60425700
H	11.17464100	20.77759500	11.38234400
C	10.51539300	19.95644400	9.50997400
H	11.33734000	19.25239600	9.44153200
C	11.84229700	21.18094900	7.11536400
C	11.02581600	22.29617100	7.32921400
H	10.09767900	22.39159100	6.77722000
C	11.39524300	23.27486700	8.24881500
H	10.74672400	24.12988500	8.41111900
C	12.58988500	23.15569000	8.96286900

C	13.42045600	22.05652100	8.74159700
H	14.35717700	21.95809100	9.28007500
C	13.05321300	21.08489000	7.81256000
H	13.71459200	20.24908700	7.61116600
C	13.38154100	21.18258600	4.68563900
C	13.08634800	22.55432900	4.73201600
H	12.09001800	22.88160500	5.00154900
C	14.06269300	23.50118900	4.42937500
H	13.81590900	24.55780900	4.47392200
C	15.34677000	23.09555200	4.05607300
C	15.64029400	21.73414200	3.97821000
H	16.62150500	21.39650300	3.66101100
C	14.66858000	20.78460900	4.29418400
H	14.91848000	19.73193800	4.24581500
N	10.29842200	16.05497300	3.23854800
O	12.11635200	14.15296600	0.90129000
O	12.27976200	14.20230600	3.16110100
O	8.64105400	15.02542800	4.43375700
O	12.09777000	16.98813000	2.19978800
C	12.52198900	10.92719000	1.93024700
C	11.80097900	9.59317700	2.06483600
H	12.60699600	8.91408500	2.35294300
C	11.12806200	8.95009400	0.81800000
C	12.24663000	8.58102900	-0.17969600
H	12.71613500	9.47314900	-0.59475900
H	13.02635000	7.98224300	0.30195700
H	11.82298100	7.99369200	-1.00070800
C	10.09607300	9.84552100	0.11379400
H	9.22263200	10.03198800	0.74103000
H	10.53071500	10.80872700	-0.15675400
H	9.75667000	9.34676400	-0.80078900
C	10.44558900	7.64643500	1.27474000
H	11.16154800	6.96258000	1.74030900
H	9.64092400	7.83917300	1.99049300
H	10.00987200	7.13763700	0.40939300
C	11.36818100	8.88978500	4.36173800
C	10.58874900	8.99531500	5.60385200
C	10.99009200	8.24875600	6.68413000
H	11.87663100	7.63539000	6.59488100
C	10.25516900	8.25642600	7.88324300
C	9.05833500	8.98380200	7.97459800
C	10.02937900	10.70369800	3.40389300
C	9.17954100	10.69943900	4.61370400
C	8.13393100	11.58933100	4.69530800
H	7.98294300	12.30591800	3.89780500
C	7.27403200	11.59085600	5.81360900
C	7.47617300	10.69313000	6.87377100
C	9.45447600	9.83228300	5.69745000
C	8.63209000	9.82406800	6.87563200
C	10.85333900	7.46623100	8.99885400
C	12.18170400	7.74000800	9.36381000
H	12.71670200	8.54483000	8.86728700
C	12.81363300	6.99893200	10.36233500
H	13.83531500	7.23577500	10.64282300
C	12.13307000	5.95872200	10.99687800
C	10.81681300	5.66833200	10.62875000
H	10.28365000	4.85569400	11.11254700
C	10.17956000	6.41696500	9.63973900
H	9.15695800	6.18875300	9.36309300
C	8.32517000	8.87353500	9.26911100
C	7.15906900	8.10678100	9.37772700
H	6.73989000	7.64437600	8.49102100
C	6.53096000	7.95498400	10.61124800
H	5.61914100	7.37060500	10.68141100
C	7.06781700	8.55694300	11.75205400
C	8.24201700	9.30376500	11.65381600
H	8.67141100	9.76587100	12.53743500
C	8.87071100	9.45368000	10.41911700
H	9.79111000	10.02132300	10.34022900
C	6.38931600	10.63107900	7.89826500
C	5.19384300	9.99832000	7.53423600
H	5.11146100	9.54441300	6.55138600
C	4.12024400	9.94708600	8.42276600
H	3.20120200	9.44826000	8.13008800
C	4.22969300	10.54042400	9.68041800
C	5.41352200	11.18741400	10.04096500
H	5.50111300	11.65634700	11.01374300
C	6.48535700	11.23774400	9.15377600
H	7.40056900	11.73873800	9.43817500
C	6.19029900	12.61323400	5.80338900
C	5.37463000	12.76092600	4.67228500
H	5.48348600	12.07045300	3.84120600
C	4.42354700	13.78011100	4.60824000
H	3.79726300	13.87585900	3.72655400
C	4.28029900	14.67386500	5.67101900
C	5.09035400	14.53726600	6.80120100
H	4.99658000	15.23226200	7.62925500
C	6.03508200	13.51554100	6.86576500
H	6.66456300	13.42548900	7.73930500
N	10.95799800	9.66210800	3.27492500
O	12.79552300	11.37931500	0.78676900
O	12.86600500	11.42991700	3.05021900
O	12.34388000	8.14563100	4.27653500
O	9.93003400	11.55962300	2.53176900
C	14.20899000	12.99033700	5.35450800
C	14.82551600	11.78158400	5.93427800
C	14.65531500	14.29262800	5.78127800
H	13.01354700	12.93922700	5.41108400
C	14.28202100	10.52318600	5.60982700
C	15.81914900	11.84463600	6.93128900
H	15.74031500	14.43441500	5.82220800
N	13.83044100	15.24955800	6.07409100
C	12.13044000	12.71410400	6.49730300
C	14.63441100	9.38688900	6.32732100
H	13.56810500	10.45630700	4.80146600
C	16.20321000	10.69342200	7.61743200
H	16.27311600	12.79304500	7.19867800
S	14.49859500	16.70804400	6.59861100
C	11.00749100	13.62582900	6.10735000
C	12.68674500	12.90095100	7.87495900
H	12.01357600	11.66840400	6.20536600
C	15.58216200	9.47381800	7.34963600
H	14.17724900	8.43862800	6.06977100
H	16.97451300	10.75599600	8.37522200
O	15.72107000	16.48002700	7.39325300
O	14.55721300	17.66228600	5.47922100
C	13.19120000	17.20320700	7.71592300
C	9.88859300	13.47226900	7.16640900
H	11.36376400	14.65805300	6.10353700
H	10.62889100	13.38795300	5.11482100
C	11.54026100	12.77142400	8.90178000
H	13.11180900	13.90330100	7.96503300
H	13.46569600	12.16885600	8.09064700
H	15.85895700	8.59133900	7.91281700
H	13.10928400	16.45665100	8.50540600
H	12.26280700	17.28446400	7.15421800
H	13.47471400	18.16899200	8.13351500
C	10.41076900	13.75870200	8.58693700
H	9.48349600	12.45782200	7.14059800
H	9.07889300	14.14344700	6.87602400
H	11.96344100	12.94940200	9.89480300
H	11.14618500	11.74714100	8.89288800
H	10.84218100	14.77134400	8.57994900
H	3.54474000	15.47083300	5.61909300
H	4.65623700	17.83988200	11.05185600
H	9.28069400	22.37881400	11.55306300
H	12.87185200	23.91664900	9.68401700
H	17.87490500	24.27988600	8.67522500
H	21.44358900	19.24607800	12.11236300
H	21.61831400	7.33250900	11.26071300
H	10.98594200	2.05946100	2.66608400
H	18.99654900	15.00218500	13.03440000
H	20.77467600	9.68157700	9.09027800
H	12.62240300	5.37847800	11.77314700
H	6.57414600	8.44087400	12.71193500
H	3.39678400	10.50338600	10.37608000
H	16.72493900	3.19728700	11.09277800
H	16.10559500	23.83460900	3.81749000

H	13.25279100	1.99164200	8.79997700
C	9.29184500	13.76173400	9.61542700
C	9.31982600	12.98336000	10.77936700
C	8.18578000	14.59893400	9.41563900
C	8.28237200	13.03426700	11.71377600
H	10.15603400	12.32608900	10.98203300
C	7.13194000	14.65154400	10.32271700
H	8.14448600	15.23570100	8.54128900
C	7.19727800	13.86943600	11.47219800
H	8.31920600	12.42407500	12.60797300
H	6.28771000	15.30306300	10.14365700
Br	5.76835600	13.95857000	12.74384400

Structure TS-C3

Rh	13.60467000	12.79222000	0.64398300
Rh	13.60446600	12.62029400	3.08380100
C	15.86213000	11.34002100	1.75339400
C	17.27601100	10.74792800	1.75266700
H	17.88693400	11.56308000	2.15010700
C	17.92401800	10.36205200	0.38747800
C	18.17621900	11.66701500	-0.39719400
H	17.23958100	12.15519700	-0.66809300
H	18.76954100	12.37270200	0.19356600
H	18.72992400	11.44189800	-1.31453300
C	17.08251000	9.39933600	-0.46579000
H	16.94954600	8.43583800	0.02951000
H	16.09558000	9.81385800	-0.67371500
H	17.59603100	9.22638600	-1.41824700
C	19.28930300	9.70600900	0.67177000
H	19.93029100	10.35591400	1.27267400
H	19.17436100	8.75653100	1.20345800
H	19.79609500	9.49824500	-0.27581000
C	18.30755200	9.88669300	3.80524500
C	18.17415500	9.05726500	5.01630200
C	19.06437600	9.22799900	6.05500100
H	19.93212800	9.85764700	5.89956800
C	18.75843400	8.76348300	7.35633600
C	17.58135200	8.03429100	7.57235400
C	16.30040200	8.77413200	2.87156200
C	16.21656900	7.93913300	4.08588300
C	15.25650300	6.95601600	4.17547200
H	14.63693600	6.75904400	3.30910700
C	15.10492600	6.17805100	5.34828500
C	15.85803300	6.50052200	6.49004400
C	17.07576000	8.18534000	5.17829100
C	16.83245100	7.55535700	6.43377200
C	19.68287900	9.22309900	8.43534700
C	19.26084700	10.04003000	9.49281700
H	18.21738000	10.30258100	9.57675800
C	20.16881300	10.51499900	10.43702900
H	19.81713300	11.15535800	11.24036100
C	21.51918300	10.17021900	10.34463500
C	21.95469500	9.36257300	9.29267700
H	23.00278900	9.09216200	9.21049900
C	21.04385200	8.89682000	8.34323600
H	21.38427600	8.26710900	7.52682300
C	16.98382500	7.96938000	8.93259000
C	17.64046200	7.44441500	10.05113000
H	18.60412300	6.96311300	9.92857000
C	17.05946900	7.53784100	11.31441000
H	17.57542700	7.12122700	12.17384100
C	15.82102100	8.16483700	11.47897200
C	15.15534600	8.68495600	10.36712400
H	14.19006700	9.16899300	10.47854800
C	15.73111700	8.58346100	9.10275100
H	15.20777600	8.97768000	8.23746700
C	15.70727700	5.67852000	7.72229300
C	16.76645600	4.87021000	8.15341400
H	17.70554300	4.88916700	7.60896800
C	16.61454700	4.03995900	9.26344500
H	17.43990600	3.41126600	9.58308000
C	15.40629600	4.02023200	9.96244100
C	14.35051800	4.83542500	9.54580200
H	13.40936700	4.83243200	10.08614400
C	14.49876500	5.65166900	8.42749100
H	13.68107200	6.28482000	8.10430700
C	14.11994500	5.05937100	5.28747600
C	14.39301500	3.77905800	5.79879700
H	15.34163900	3.58151800	6.28114100
C	13.45731800	2.75295000	5.68306300
H	13.69045700	1.77138600	6.08457200
C	12.23245600	2.97781500	5.04966000
C	11.95851200	4.23823100	4.52041000
H	11.01998100	4.43544400	4.01210200
C	12.89224500	5.26700900	4.63762900
H	12.64099500	6.23607200	4.22286000
N	17.33703200	9.71408400	2.80647400
O	15.35626000	11.69741400	0.65391400
O	15.35001600	11.50663500	2.91117500
O	19.18350100	10.74045000	3.69477200
O	15.46658400	8.70443800	1.97472800
C	15.12088900	14.85979700	2.00775400
C	15.93951600	16.15156200	2.11117000
H	15.22945400	16.87198200	2.52375100
C	16.48012600	16.80067700	0.79786100
C	15.27046300	17.25893400	-0.04378300
H	14.70192000	16.40982400	-0.42143600
H	14.59183300	17.88418700	0.54527200
H	15.62412800	17.85123500	-0.89389800
C	17.37150100	15.87358800	-0.04561600
H	18.30138100	15.62645400	0.47026600
H	16.85801900	14.94276900	-0.28699300
H	17.63026500	16.38379900	-0.98007700
C	17.28883400	18.05675400	1.17892300
H	16.68057000	18.77031400	1.74049400
H	18.16862800	17.80721100	1.77947200
H	17.63869000	18.55085800	0.26735300
C	16.88052500	16.91325900	4.24006900
C	17.72318600	16.64869200	5.42071800
C	17.49300900	17.37297600	6.56631100
H	16.72998500	18.14136800	6.56186200
C	18.21967900	17.11837700	7.74723500
C	19.30244000	16.22676500	7.72835900
C	17.81774100	14.90685900	3.17445800
C	18.84045000	14.84409400	4.24589000
C	19.84292400	13.90511400	4.16119600
H	19.88133800	13.23933100	3.30869700
C	20.78269400	13.75976500	5.20608800
C	20.68250500	14.52916400	6.37481400
C	18.73539000	15.66253000	5.39656200
C	19.60948800	15.48457000	6.52354500
C	17.70060200	17.78142000	8.97919800
C	16.33304800	17.63352500	9.26282200
H	15.70216200	17.03594500	8.61396200
C	15.76965200	18.22281300	10.39476300
H	14.71276100	18.07910200	10.59752700
C	16.56346200	18.98425000	11.25464000
C	17.92092300	19.15430200	10.96840900
H	18.54350900	19.75367800	11.62563800
C	18.48662100	18.55887000	9.84179200
H	19.54125000	18.69344900	9.63450300
C	20.01881300	16.03984700	9.02735900
C	21.25434200	16.64893700	9.27338800
H	21.74293700	17.20561600	8.48156700
C	21.86099200	16.52883100	10.52165800
H	22.82613400	16.99387600	10.69564800
C	21.23321200	15.81237800	11.54386400
C	19.98991600	15.22289400	11.31249500
H	19.48089500	14.67889800	12.10275500
C	19.38465400	15.34253600	10.06282000
H	18.40819400	14.90629600	9.89781700
C	21.77686100	14.35343100	7.37825700
C	23.01323100	14.95466800	7.11401400
H	23.12751900	15.56144800	6.22105300
C	24.08736500	14.78131200	7.98668500
H	25.03899700	15.25793300	7.77171600
C	23.93621700	13.99670200	9.12982800
C	22.71054300	13.37920000	9.38776700
H	22.58341000	12.76441700	10.27105100

C	21.64052700	13.54819400	8.51411400
H	20.69660200	13.05848300	8.72040600
C	21.82757600	12.71449300	5.01533000
C	22.63185100	12.74688300	3.86808300
H	22.51573900	13.56253200	3.16058500
C	23.58236100	11.75209200	3.63468000
H	24.20175100	11.79793600	2.74417700
C	23.73537200	10.70398600	4.54302400
C	22.92918100	10.65587200	5.68295500
H	23.03283100	9.84205100	6.39297000
C	21.98242900	11.65054300	5.91724700
H	21.35858800	11.59499700	6.80065600
N	16.94596000	16.00170500	3.18503700
O	14.77488100	14.47049700	0.85758100
O	14.78585800	14.33806400	3.12055600
O	16.11361500	17.87237000	4.19750800
O	17.72248600	14.04715400	2.30495000
C	11.42688500	14.18726100	1.95020700
C	10.13807400	15.00607300	2.08680200
H	9.47553600	14.35431600	2.65833600
C	9.36141700	15.39944200	0.79465800
C	8.93503600	14.10150400	0.08253200
H	9.79732200	13.56536400	-0.31261800
H	8.40491500	13.43104100	0.76629800
H	8.26367600	14.34187200	-0.74827700
C	10.15934100	16.28474800	-0.17686800
H	10.39482000	17.25720900	0.26253900
H	11.09357000	15.80708500	-0.47133000
H	9.55527300	16.46399800	-1.07331200
C	8.08616200	16.15630600	1.21686700
H	7.48119400	15.56793000	1.91224000
H	8.32387200	17.11035300	1.69705400
H	7.48158800	16.37284300	0.33063900
C	9.55926600	16.28502600	4.11628500
C	9.82565700	17.42978200	5.01128900
C	9.13397900	17.52691800	6.20012300
H	8.36199900	16.80099000	6.42521400
C	9.53956900	18.45009300	7.18754900
C	10.55141100	19.37700200	6.92722800
C	11.50837300	16.95392800	2.74849500
C	11.65192300	18.17073400	3.56750200
C	12.63901400	19.08043400	3.25161100
H	13.24781300	18.91285000	2.37140700
C	12.81923000	20.25219200	4.01656600
C	12.07452700	20.43997800	5.18851600
C	10.85611600	18.35278700	4.72084400
C	11.14962200	19.42558300	5.62037600
C	8.96772000	18.29524300	8.55636500
C	9.75485900	17.74696300	9.57905800
H	10.77821000	17.45837900	9.36406900
C	9.22465800	17.56711500	10.85538600
H	9.84205800	17.13980900	11.63974300
C	7.90017400	17.92588200	11.12279200
C	7.11017600	18.46564000	10.10632200
H	6.08026500	18.74520000	10.30666000
C	7.64212000	18.64764200	8.82808400
H	7.02907900	19.06666100	8.03578900
C	11.09401400	20.16043600	8.07512700
C	10.31616400	21.10877700	8.74943200
H	9.32033400	21.33504100	8.38400500
C	10.81440800	21.76236800	9.87427200
H	10.20233000	22.50204400	10.38136200
C	12.09558000	21.46914700	10.34746000
C	12.87441400	20.51726400	9.68818100
H	13.87086600	20.27991700	10.04638300
C	12.37518900	19.86632700	8.56113400
H	12.97837400	19.10960200	8.07175000
C	12.18016800	21.74371200	5.89871800
C	11.06614100	22.59227000	5.92950600
H	10.12893100	22.25321000	5.49893300
C	11.15984800	23.86233700	6.49663000
H	10.29095000	24.51327000	6.50790600
C	12.36668000	24.29271800	7.05024500
C	13.47882600	23.44692100	7.03144600

H	14.41846200	23.77303100	7.46670800
C	13.38846900	22.18420700	6.45151100
H	14.25405000	21.53096300	6.42645800
C	13.80007900	21.25203000	3.50537700
C	13.41710000	22.56248100	3.18564400
H	12.39112000	22.87486200	3.34587200
C	14.34743000	23.46288600	2.66969600
H	14.03682100	24.47389600	2.42359000
C	15.67239800	23.06789200	2.46673700
C	16.05938500	21.76391400	2.77984000
H	17.08724400	21.44733800	2.62839700
C	15.12896500	20.85838000	3.29234500
H	15.43452100	19.85017200	3.54962800
N	10.39146600	16.14984600	2.98849300
O	11.85972300	13.88447200	0.80301000
O	11.91728900	13.81834700	3.06759300
O	8.66702100	15.47169800	4.33356300
O	12.33356500	16.64370100	1.89328100
C	12.03571200	10.60589900	1.66189700
C	11.05962400	9.42566900	1.62714800
H	11.69593100	8.54101300	1.72094300
C	10.21839200	9.19794500	0.32844900
C	11.17691100	8.68052700	-0.76412500
H	11.93519800	9.42461700	-1.01110600
H	11.68215500	7.76292400	-0.44170100
H	10.60983500	8.44811400	-1.67124100
C	9.49169900	10.45200200	-0.18115600
H	8.75446100	10.80919800	0.53839100
H	10.19296100	11.26331500	-0.37515700
H	8.97426900	10.20934400	-1.11602100
C	9.17585600	8.09916200	0.60977600
H	9.64561500	7.18408600	0.97938500
H	8.43732400	8.42650900	1.34764000
H	8.64057700	7.86327100	-0.31520600
C	10.35226800	8.25641100	3.65192100
C	9.74349800	8.28662800	4.99409200
C	9.77569000	7.13528400	5.74332000
H	10.19213400	6.23786600	5.30644100
C	9.31374700	7.11641500	7.07340400
C	8.78330500	8.27962200	7.65044700
C	9.71874100	10.61763400	3.34373300
C	9.19782800	10.63819300	4.72700900
C	8.70429600	11.82409600	5.22560400
H	8.70747000	12.71416300	4.60632900
C	8.20413100	11.89293000	6.54051400
C	8.19994200	10.76302800	7.37064700
C	9.20865700	9.47705500	5.53685900
C	8.70753500	9.50302200	6.88458300
C	9.50740400	5.83501900	7.81361300
C	10.76258300	5.20780700	7.75145300
H	11.56373900	5.66464200	7.18183700
C	10.99464700	3.99553400	8.40113500
H	11.97410100	3.53475900	8.33212800
C	9.96806200	3.38418600	9.12146900
C	8.70909700	3.98871300	9.17554900
H	7.90000900	3.51233700	9.72103200
C	8.47901100	5.20024300	8.52657400
H	7.49788900	5.65645900	8.57314600
C	8.40982900	8.18741900	9.09374800
C	7.07467700	8.12443100	9.50678300
H	6.28457300	8.20671700	8.76877700
C	6.75961400	7.96832400	10.85446200
H	5.71927300	7.93391800	11.16194200
C	7.77618400	7.86033600	11.80614000
C	9.11023800	7.90379900	11.40057000
H	9.90754800	7.81301600	12.13202300
C	9.42328200	8.06041200	10.05151400
H	10.46140600	8.07675500	9.73738600
C	7.58331700	10.96297200	8.71829400
C	6.19320900	11.10436900	8.81085000
H	5.58901100	10.99505600	7.91598200
C	5.58901700	11.38986300	10.03343500
H	4.50973700	11.49450100	10.09015800
C	6.37064600	11.54442700	11.17919100

C	7.75839800	11.41819400	11.09169200
H	8.37427400	11.54109800	11.97709800
C	8.36048800	11.13810200	9.86715000
H	9.43939400	11.05652900	9.80172500
C	7.61616500	13.18998700	6.98464800
C	6.48909700	13.68392600	6.31583000
H	6.08479200	13.12814500	5.47512500
C	5.87280800	14.86169500	6.73764000
H	4.99228200	15.22614400	6.21706100
C	6.38309700	15.56238400	7.83056100
C	7.52714400	15.09493700	8.48351300
H	7.93785000	15.64991200	9.31874900
C	8.14390000	13.92033400	8.05790000
H	9.02863400	13.55659200	8.56860500
N	10.29675000	9.42369000	2.89114700
O	12.41500900	11.10620000	0.56497800
O	12.44596400	10.93183600	2.82008700
O	10.90373000	7.24773700	3.21057200
O	9.67427900	11.59946900	2.61106000
C	13.46404900	12.36518100	5.33065300
C	12.86905100	11.14762100	5.92113700
C	13.00571100	13.63740500	5.82930000
H	14.64424600	12.31818600	5.32424600
C	13.24945600	9.87602400	5.45276900
C	12.08835700	11.22707000	7.09140400
H	11.92067200	13.78709300	5.81597200
N	13.80195400	14.56426700	6.25652400
C	15.54960200	12.02693700	6.36329200
C	12.90696700	8.73412700	6.16898600
H	13.83013000	9.79909200	4.54173000
C	11.74566400	10.07878600	7.80084600
H	11.78272100	12.19400500	7.47683500
S	13.05688100	15.93033500	6.91409500
C	16.71376000	12.72878000	5.73718900
C	15.20614400	12.42743100	7.76491600
H	15.50554300	10.95453100	6.16553500
C	12.17690300	8.83134100	7.35556000
H	13.21583100	7.76479000	5.80704700
H	11.16464800	10.16168300	8.71191900
O	13.58072700	16.07495100	8.28935300
O	11.59285000	15.89314500	6.74508300
C	13.79390200	17.18763600	5.89809200
C	17.91707800	12.36546200	6.63381900
H	16.55054000	13.80894200	5.75273300
H	16.86820100	12.40844200	4.70683300
C	16.44437400	12.22613900	8.68428400
H	14.93277900	13.48210500	7.77539300
H	14.36873000	11.84258800	8.15101900
H	11.92976900	7.93667500	7.91508300
H	14.86053200	17.16722600	6.08985300
H	13.59660100	16.95660000	4.85549900
H	13.36937700	18.15186700	6.16860200
C	17.69225500	12.86571700	8.05061700
H	18.04770000	11.28288900	6.64162700
H	18.81911900	12.78468200	6.20035100
H	16.61345600	11.14861800	8.77228100
H	17.56985200	13.95406800	8.03722000
H	18.56021600	12.65074800	8.67971300
H	10.14292300	2.44080100	9.62973200
H	7.52831300	7.74046700	12.85631900
H	5.90297500	11.76581500	12.13368300
H	5.90542900	16.47468700	8.17030000
H	12.48280900	21.97852800	11.22461900
H	24.47385100	9.92874100	4.36338300
H	24.76813200	13.86308700	9.81456800
H	21.70907000	15.72105000	12.51549800
H	16.12999200	19.44200800	12.13852300
H	22.22620800	10.52515400	11.08747300
H	15.37708200	8.24218600	12.46651800
H	15.28833400	3.37729200	10.82915600
H	16.39595900	23.77213100	2.06731100
H	12.44093300	25.27998100	7.49583100
H	7.48689600	17.78371900	12.11660600
H	11.50428900	2.17708500	4.96664800

C	16.23608700	12.79227900	10.07237400
C	16.52229500	12.01981700	11.20425000
C	15.88734200	14.13888200	10.25252300
C	16.50666200	12.57863900	12.48343000
H	16.76262000	10.96726100	11.09544100
C	15.87140600	14.71559900	11.52078700
H	15.63722000	14.76255200	9.40076500
C	16.19586100	13.92852700	12.62374900
H	16.74068900	11.97398300	13.35183500
H	15.63329600	15.76534500	11.64053100
Br	16.22321600	14.72576900	14.36494400

Structure TS-C1

Rh	-0.19344600	-0.00219900	-4.91310200
Rh	-0.20903800	-0.21007800	-2.46380100
C	2.02924300	-1.50812300	-3.82850700
C	3.40485200	-2.18307600	-3.86722200
H	4.07025000	-1.41411800	-3.46678000
C	4.00746500	-2.57622500	-5.25228000
C	4.31718900	-1.27420600	-6.02101100
H	3.40336300	-0.74220300	-6.28585400
H	4.94053300	-0.60108000	-5.42263800
H	4.86196000	-1.51344200	-6.94012600
C	3.09840700	-3.47572700	-6.10581700
H	2.93004400	-4.44354900	-5.63035500
H	2.12860200	-3.00791300	-6.27832100
H	3.57908000	-3.64999900	-7.07500700
C	5.34079300	-3.31019500	-5.00689100
H	6.03291800	-2.70415600	-4.41704700
H	5.18686200	-4.25767400	-4.48159600
H	5.81078300	-3.53598800	-5.96920600
C	4.42430400	-3.11908900	-1.85304200
C	4.25077600	-3.91334100	-0.62067400
C	5.07224700	-3.63563600	0.44570500
H	5.86179100	-2.90458400	0.32463000
C	4.84287500	-4.23242600	1.69933700
C	3.84427800	-5.20294600	1.86137200
C	2.39615500	-4.16757700	-2.77245900
C	2.36636900	-5.07667900	-1.60352800
C	1.44629500	-6.09704400	-1.57715800
H	0.83726800	-6.27322700	-2.45560300
C	1.26021200	-6.88321300	-0.41762900
C	1.97606600	-6.58676500	0.75548900
C	3.20962400	-4.85857800	-0.48888200
C	3.00842900	-5.57566500	0.74141600
C	5.68092000	-3.71789600	2.82036800
C	5.10005200	-3.05505500	3.91103000
H	4.02015700	-2.98854000	3.98408200
C	5.90150200	-2.47398800	4.89318500
H	5.43771000	-1.95991800	5.72950800
C	7.29334400	-2.54373200	4.79835600
C	7.88095000	-3.19766900	3.71369400
H	8.96176900	-3.25129600	3.62868100
C	7.07867600	-3.77908900	2.73208400
H	7.53272300	-4.29313900	1.89090300
C	3.78548500	-5.83958100	3.21206300
C	4.81040000	-6.71636200	3.58859700
H	5.58977500	-6.96014900	2.87339100
C	4.82849600	-7.27757900	4.86478900
H	5.62415600	-7.96291600	5.14074800
C	3.82851000	-6.95682600	5.78376300
C	2.81767800	-6.06351500	5.42240800
H	2.03972500	-5.80064100	6.13234100
C	2.80223600	-5.50458400	4.14745700
H	2.02692900	-4.80187300	3.87624500
C	1.50989900	-7.26382900	2.00472200
C	2.19894600	-8.31807300	2.61117000
H	3.15117500	-8.64003500	2.20486200
C	1.67024900	-8.94846700	3.73600700
H	2.21970400	-9.75967900	4.20284000
C	0.44207700	-8.54114000	4.26262600
C	-0.25721600	-7.49559800	3.65760800
H	-1.21759000	-7.17539700	4.04991700
C	0.27526200	-6.86640300	2.53354100
H	-0.26321100	-6.05647200	2.05776100

C	0.21929000	-7.94933000	-0.49677000
C	0.44669700	-9.25015100	-0.01916700
H	1.39689000	-9.49524300	0.43751800
C	-0.53298300	-10.23426700	-0.13482800
H	-0.33536600	-11.23237400	0.24429100
C	-1.75571900	-9.94542900	-0.74557300
C	-1.98229300	-8.66536100	-1.25031100
H	-2.91542000	-8.42433100	-1.74900800
C	-1.00713200	-7.67651000	-1.12570900
H	-1.22392600	-6.69040000	-1.51983100
N	3.43677600	-3.23898700	-2.83570900
O	1.54608000	-1.09756400	-4.91891200
O	1.53468100	-1.33665400	-2.66492800
O	5.34687600	-2.32129000	-1.98492700
O	1.52489100	-4.18938200	-3.63546500
C	1.20517000	2.10264000	-3.49930000
C	1.92662600	3.44200900	-3.34623400
H	1.22817900	4.05108600	-2.77022400
C	2.27431900	4.25860000	-4.63134700
C	0.96595400	4.60837100	-5.36851300
H	0.49461300	3.71937300	-5.78593000
H	0.24417400	5.08186500	-4.69808200
H	1.18758600	5.30569300	-6.18308300
C	3.22616700	3.52976000	-5.59436700
H	4.20922700	3.36506300	-5.14661000
H	2.82080600	2.56386900	-5.89553600
H	3.36885100	4.14740300	-6.48797100
C	2.93454200	5.58108300	-4.19064300
H	2.28793400	6.15380600	-3.52056900
H	3.88906100	5.41073600	-3.68316700
H	3.13699700	6.19623900	-5.07278100
C	3.17429400	4.11625300	-1.34065200
C	4.42954800	4.07749600	-0.56902300
C	4.63131600	5.00089800	0.43468300
H	3.86881500	5.74860600	0.61913300
C	5.84527100	5.03991400	1.15448400
C	6.81862200	4.05628300	0.93584800
C	3.96794800	2.20043300	-2.68443000
C	5.17647900	2.12965100	-1.84243200
C	6.04648100	1.07132500	-2.00228700
H	5.84070900	0.32996100	-2.76398600
C	7.10744200	0.85533200	-1.09370800
C	7.34176500	1.77869600	-0.06434100
C	5.40184600	3.08309500	-0.82266100
C	6.55414800	2.98060100	0.01636600
C	6.03052500	6.17648300	2.10101200
C	5.08134900	6.38754700	3.11120000
H	4.24987100	5.69603000	3.21245200
C	5.20658100	7.46252100	3.99201000
H	4.46816900	7.60606200	4.77496400
C	6.27859000	8.34727600	3.86697400
C	7.22205200	8.15178300	2.85499200
H	8.05399700	8.84092600	2.74600500
C	7.10041300	7.07536500	1.97871500
H	7.83730200	6.92646900	1.19764700
C	8.15795600	4.21397000	1.56529700
C	9.26811600	4.43593400	0.73967200
H	9.13902600	4.42203400	-0.33817500
C	10.52394800	4.68076100	1.29315900
H	11.37481100	4.85753300	0.64239000
C	10.68530200	4.69504600	2.67943300
C	9.58344400	4.46705700	3.50814800
H	9.70373800	4.47108400	4.58711700
C	8.32652700	4.23683500	2.95492100
H	7.46929500	4.06884800	3.59766400
C	8.25655500	1.38482400	1.04408100
C	9.62550900	1.17395900	0.85139100
H	10.06900900	1.40002000	-0.11226800
C	10.41162900	0.66907200	1.88515200
H	11.47439000	0.51455200	1.72586000
C	9.83493500	0.35206600	3.11753200
C	8.46929800	0.56184300	3.31766500
H	8.01060000	0.31052200	4.26807100
C	7.68677500	1.08197600	2.28877400

H	6.62309400	1.23801100	2.43686300
C	7.87820400	-0.41018700	-1.26320400
C	8.40863100	-0.70680400	-2.52664000
H	8.28716900	0.00808500	-3.33510400
C	9.09575300	-1.89950600	-2.75363200
H	9.50301000	-2.10703900	-3.73847400
C	9.26166100	-2.81743500	-1.71602500
C	8.73197600	-2.53337700	-0.45542300
H	8.85520100	-3.24083100	0.35791500
C	8.04259700	-1.34442200	-0.22904900
H	7.63081800	-1.14495900	0.75295900
N	3.08686000	3.26598600	-2.44996300
O	0.88346900	1.72331300	-4.65798200
O	0.92761900	1.52070700	-2.39785200
O	2.24719800	4.86658800	-1.04516600
O	3.72232600	1.37145800	-3.55494000
C	-2.41257700	1.33269500	-3.64599000
C	-3.73557300	2.10331500	-3.57406300
H	-4.42421900	1.40765900	-3.09137300
C	-4.39538100	2.53789100	-4.92302300
C	-4.73344300	1.27201400	-5.73367300
H	-3.83265100	0.76277200	-6.07210000
H	-5.31648700	0.56583800	-5.13400600
H	-5.32980500	1.54724500	-6.60979800
C	-3.51488800	3.48167000	-5.76011500
H	-3.33959500	4.43028400	-5.24547600
H	-2.54919900	3.03019700	-5.98555400
H	-4.02775100	3.70815400	-6.70139600
C	-5.72134500	3.26177500	-4.61184000
H	-6.38930100	2.64015300	-4.01005700
H	-5.55649900	4.20197400	-4.07719300
H	-6.22757000	3.50337900	-5.55151800
C	-4.62770500	3.39418900	-1.67448200
C	-4.61692100	4.65099300	-0.89335000
C	-5.62752600	4.86300700	0.01068300
H	-6.40112600	4.11320000	0.11505300
C	-5.61749100	5.99151700	0.85766500
C	-4.59328600	6.94436400	0.76644200
C	-2.49071100	4.05134500	-2.69549000
C	-2.51452800	5.30256400	-1.91307600
C	-1.47518900	6.18952200	-2.07273500
H	-0.68359100	5.95535200	-2.77325400
C	-1.44213800	7.40193800	-1.35956100
C	-2.45143900	7.70878600	-0.43338700
C	-3.56503200	5.58893400	-1.01280300
C	-3.53917900	6.78195700	-0.21026300
C	-6.71863100	6.06110200	1.86083600
C	-6.46569000	6.18076000	3.23614100
H	-5.44607500	6.28856900	3.58485500
C	-7.51139100	6.14536500	4.15595800
H	-7.29523100	6.23120200	5.21651800
C	-8.82960000	5.98589300	3.71992100
C	-9.09264200	5.86289000	2.35489000
H	-10.11272900	5.73916400	2.00420900
C	-8.04444500	5.89779200	1.43462900
H	-8.25302700	5.79902200	0.37365200
C	-4.67256300	8.08822900	1.72023700
C	-5.74878000	8.98085300	1.63928200
H	-6.47521600	8.86340400	0.84163600
C	-5.89098800	10.00705200	2.57019000
H	-6.72703400	10.69537300	2.49121900
C	-4.96131700	10.14706600	3.60187100
C	-3.89187300	9.25416600	3.69601300
H	-3.16534900	9.35627800	4.49611700
C	-3.75137200	8.22824900	2.76396100
H	-2.92398200	7.53082000	2.84114300
C	-2.30259000	9.01288800	0.27568500
C	-3.17941000	10.07617200	0.03766900
H	-4.02853900	9.92973100	-0.62133900
C	-2.97004300	11.31251200	0.64583900
H	-3.66319700	12.12692800	0.45940000
C	-1.87542400	11.50342800	1.49104900
C	-0.98904900	10.45030200	1.72406500
H	-0.13350400	10.59067600	2.37776300

C	-1.20001000	9.21575100	1.11606700
H	-0.50717300	8.39834400	1.28772900
C	-0.29074700	8.30666500	-1.64718500
C	-0.47132300	9.61925100	-2.10641700
H	-1.47485200	10.01164500	-2.22566400
C	0.62903800	10.42159100	-2.40235600
H	0.47334600	11.43546700	-2.75879500
C	1.92641000	9.92564900	-2.24597400
C	2.11409200	8.61677200	-1.79994100
H	3.11603700	8.21570800	-1.67827900
C	1.01311300	7.81020000	-1.50752900
H	1.17242600	6.79433200	-1.16445900
N	-3.61326100	3.23106400	-2.62985300
O	-1.93677900	1.06514500	-4.78496100
O	-1.94737800	0.94963400	-2.52433800
O	-5.49554400	2.53954600	-1.53323600
O	-1.53099500	3.74407100	-3.39862600
C	-1.75403400	-2.21924700	-3.98084200
C	-2.71287900	-3.40993800	-4.09760200
H	-2.07294300	-4.28765300	-3.96925900
C	-3.45304600	-3.62547300	-5.46097200
C	-2.41246500	-4.11143400	-6.49117800
H	-1.65123800	-3.35281700	-6.67527500
H	-1.91621600	-5.02575400	-6.14615100
H	-2.91204800	-4.34029600	-7.43811500
C	-4.16101100	-2.37111300	-5.99599900
H	-4.95012600	-2.03833900	-5.32080300
H	-3.46056700	-1.54718400	-6.12738600
H	-4.61204600	-2.60275600	-6.96736500
C	-4.50015200	-4.74200100	-5.28303900
H	-4.04796200	-5.66365000	-4.90895000
H	-5.29275200	-4.44354700	-4.59037000
H	-4.96703000	-4.95421100	-6.24984500
C	-3.60599700	-4.64945200	-2.19420700
C	-4.36376500	-4.67603500	-0.93090400
C	-4.39089000	-5.85661200	-0.22781900
H	-3.94091100	-6.73715600	-0.66563800
C	-4.94297700	-5.92364500	1.06429300
C	-5.48223000	-4.77400900	1.66105700
C	-4.20206600	-2.27311700	-2.46226500
C	-4.82085200	-2.29798600	-1.11858300
C	-5.24086400	-1.10653600	-0.56535100
H	-5.14939700	-0.18412200	-1.12842200
C	-5.72921500	-1.07050200	0.75480800
C	-5.85235600	-2.24104000	1.51506000
C	-4.91282300	-3.49860200	-0.37356500
C	-5.46738000	-3.51337000	0.95356100
C	-4.82446200	-7.23675800	1.76345900
C	-3.56423400	-7.85132100	1.83228000
H	-2.70201100	-7.37024300	1.38353800
C	-3.39936000	-9.07859800	2.47432300
H	-2.41135400	-9.52480200	2.52234900
C	-4.49976500	-9.71883800	3.04501800
C	-5.76323200	-9.12583100	2.96399100
H	-6.62606100	-9.62351400	3.39633000
C	-5.92527300	-7.89569500	2.33000100
H	-6.90625900	-7.43728100	2.28078100
C	-5.96703000	-4.93043200	3.06288100
C	-7.32555700	-4.85556300	3.38518100
H	-8.04509700	-4.61216400	2.61075200
C	-7.75250000	-5.08284300	4.69232500
H	-8.80890300	-5.01263100	4.93203600
C	-6.82699300	-5.39556500	5.68995400
C	-5.47036500	-5.48027100	5.37310200
H	-4.74236400	-5.72163200	6.14179100
C	-5.04583700	-5.25459000	4.06694200
H	-3.99148600	-5.32298000	3.82294600
C	-6.34522500	-2.04165700	2.91294500
C	-7.68172200	-1.68656400	3.13192900
H	-8.36325400	-1.64152900	2.28891100
C	-8.13597400	-1.38913000	4.41493900
H	-9.17603300	-1.11762600	4.56858400
C	-7.25604300	-1.43824700	5.49755900
C	-5.91974900	-1.78146300	5.28562700

H	-5.22321000	-1.82482500	6.11724600
C	-5.46692500	-2.07130500	4.00082200
H	-4.42399200	-2.32435400	3.85196400
C	-6.06200300	0.25967600	1.34788100
C	-7.25290300	0.91193400	1.00918700
H	-7.91902300	0.45965100	0.28070700
C	-7.59915200	2.11278100	1.62875800
H	-8.53427500	2.60427500	1.37785600
C	-6.74649900	2.68571200	2.57427300
C	-5.53162400	2.06757100	2.87553100
H	-4.84503700	2.52465200	3.58142300
C	-5.19418400	0.85848300	2.26964400
H	-4.26324800	0.36431100	2.52840600
N	-3.57614700	-3.44797100	-2.90181700
O	-1.33050200	-1.70646500	-5.05628600
O	-1.39985100	-1.89974400	-2.80383800
O	-2.99895700	-5.63771100	-2.60730500
O	-4.19159400	-1.26688000	-3.16191000
H	-4.37730900	-10.67419000	3.54623400
H	-7.16228200	-5.57075100	6.70764600
H	-7.60832300	-1.20848400	6.49856500
H	-7.02140200	3.61304500	3.06083700
H	-5.06885000	10.94617000	4.32901200
H	9.79826900	-3.74564400	-1.88703000
H	10.44667400	-0.05521200	3.91670900
H	11.66311200	4.88135400	3.11282100
H	6.37787000	9.18457100	4.55091800
H	7.91571800	-2.08951800	5.56303100
H	3.83939700	-7.39463000	6.77715500
H	0.03370400	-9.03650500	5.13802100
H	2.78176600	10.55447200	-2.47393600
H	-1.71438200	12.46747000	1.96386600
H	-9.64289900	5.95562100	4.43838900
H	-2.51894200	-10.71230300	-0.83250100
C	-0.48134100	-0.44299400	-0.22885200
C	-1.04680200	-1.73388400	0.14698800
C	-1.18843300	0.75084600	0.17566300
H	0.64642000	-0.34972200	0.52238800
C	-0.46435400	-2.92171600	-0.34615700
C	-2.08334400	-1.83714000	1.09977000
H	-2.22114200	0.84626100	-0.17619900
N	-0.61717600	1.69881300	0.84536200
C	1.39666700	-0.57143300	1.66668200
C	-0.89755200	-4.15841500	0.11237100
H	0.31369300	-2.85028800	-1.09549800
C	-2.48855700	-3.07529000	1.57489100
H	-2.54630800	-0.93784700	1.48684100
S	-1.56932900	3.01895100	1.26572000
C	1.86048200	0.78302300	2.20020700
C	2.47605400	-1.40173600	0.98633300
C	0.51074500	-1.28518300	2.61864100
C	-1.89725000	-4.23699400	1.08220600
H	-0.44467500	-5.05946900	-0.27014700
H	-3.24799700	-3.14136500	2.33910600
O	-1.59631400	3.06397400	2.74111800
O	-2.86041900	3.01668700	0.55460400
C	-0.53557700	4.35105700	0.66651500
C	2.83741800	1.53554700	1.30621600
H	2.37493100	0.53466000	3.14186400
H	1.00882200	1.40513800	2.45999100
C	3.42700700	-0.57125500	0.12483200
H	3.05605700	-1.88678600	1.78412000
H	2.01952900	-2.20917100	0.40659000
C	-0.44417700	-0.57756000	3.38160700
C	0.61144800	-2.67358400	2.81441400
H	-2.21804100	-5.20022500	1.45779800
H	0.41623600	4.30480300	1.19385000
H	-0.37731600	4.21992600	-0.40168100
H	-1.05432600	5.28895400	0.86933900
C	3.98198600	0.60433600	0.92086000
H	2.31829400	1.88426700	0.40502700
H	3.19891600	2.42411100	1.83499200
H	4.24208600	-1.19819400	-0.22814800
H	2.90119800	-0.21001600	-0.76618600

C	-1.25081100	-1.22348500	4.31196300
H	-0.58651300	0.48535500	3.23084800
C	-0.19266300	-3.33277200	3.73755000
H	1.31573700	-3.25688100	2.23586500
H	4.47491600	0.23040500	1.83023300
H	4.74202000	1.12141800	0.34828900
C	-1.11652900	-2.60000800	4.47924800
H	-1.98769900	-0.66800100	4.87959300
H	-0.11907900	-4.40559800	3.86489200
Br	-2.25762900	-3.51030600	5.70685400

13. References

(1) Davies, H. M. L.; Bruzinski, P. R.; Lake, D. H.; Kong, N.; Fall, M. J. Asymmetric Cyclopropanations by Rhodium(II) N-(Arylsulfonyl)prolinate Catalyzed Decomposition of Vinyldiazomethanes in the Presence of Alkenes. Practical Enantioselective Synthesis of the Four Stereoisomers of 2-Phenylcyclopropan-1-amino Acid. *J. Am. Chem. Soc.* **1996**, *118*, 6897-6907.
(2) Fu, J.; Ren, Z.; Bacsa, J.; Musaev, D. G.; Davies, H. M. L. Desymmetrization of cyclohexanes by site- and stereoselective C–H functionalization. *Nature* **2018**, *564*, 395-399.
(3) Müller, P.; Allenbach, Y.; Robert, E. Rhodium(II)-catalyzed olefin cyclopropanation with the phenyliodonium ylide derived from Meldrum's acid. *Tetrahedron: Asymmetry* **2003**, *14*, 779-785.
(4) Reddy, R. P.; Davies, H. M. L. Dirhodium Tetracarboxylates Derived from Adamantylglycine as Chiral Catalysts for Enantioselective C−H Aminations. *Org. Lett.* **2006**, *8*, 5013-5016.
(5) Liu, W.; Ren, Z.; Bosse, A. T.; Liao, K.; Goldstein, E. L.; Bacsa, J.; Musaev, D. G.; Stoltz, B. M.; Davies, H. M. L. Catalyst-Controlled Selective Functionalization of Unactivated C–H Bonds in the Presence of Electronically Activated C–H Bonds. *J. Am. Chem. Soc.* **2018**, *140*, 12247-12255.
(6) Chuprakov, S.; Kwok, S. W.; Zhang, L.; Lercher, L.; Fokin, V. V. Rhodium-Catalyzed Enantioselective Cyclopropanation of Olefins with N-Sulfonyl 1,2,3-Triazoles. *J. Am. Chem. Soc.* **2009**, *131*, 18034-18035.
(7) Chuprakov, S.; Malik, J. A.; Zibinsky, M.; Fokin, V. V. Catalytic Asymmetric C–H Insertions of Rhodium(II) Azavinyl Carbenes. *J. Am. Chem. Soc.* **2011**, *133*, 10352-10355.
(8) Miura, T.; Nakamuro, T.; Liang, C.-J.; Murakami, M. Synthesis of trans-Cycloalkenes via Enantioselective Cyclopropanation and Skeletal Rearrangement. *J. Am. Chem. Soc.* **2014**, *136*, 15905-15908.
(9) Kubiak, R. W., II; Davies, H. M. L. Rhodium-Catalyzed Intermolecular C–H Functionalization as a Key Step in the Synthesis of Complex Stereodefined β-Arylpyrrolidines. *Org. Lett.* **2018**, *20*, 3771-3775.
(10) Guan, C.; Ji, J.; Li, Z.; Wei, Q.; Wu, X.; Liu, S. Facile synthesis of N2-substituted-1,2,3-triazole from aryl ethynylene and azide via a one-pot two-step strategy. *Tetrahedron* **2022**, *108*, 132670.
(11) Pospech, J.; Ferraccioli, R.; Neumann, H.; Beller, M. Rhodium(II)-Catalyzed Annulation of Azavinyl Carbenes Through Ring-Expansion of 1,3,5-Trioxane: Rapid Access to Nine-Membered 1,3,5,7-Trioxazonines. *Chem. – Asian J.* **2015**, *10*, 2624-2630.
(12) Martin-Montero, R.; Yatham, V. R.; Yin, H.; Davies, J.; Martin, R. Ni-catalyzed Reductive Deaminative Arylation at sp3 Carbon Centers. *Org. Lett.* **2019**, *21*, 2947-2951.
(13) Bongini, A.; Barbarella, G.; Favaretto, L.; Sotgiu, G.; Zambianchi, M.; Casarini, D. Conformational profile, energy barriers and optical properties of quinquethiophene-S,S-dioxides. *Tetrahedron* **2002**, *58*, 10151-10158.
(14) Pauli, L.; Tannert, R.; Scheil, R.; Pfaltz, A. Asymmetric Hydrogenation of Furans and Benzofurans with Iridium–Pyridine–Phosphinite Catalysts. *Chem. – Eur. J.* **2015**, *21*, 1482-1487.
(15) Wesenberg, L. J.; Sivo, A.; Vilé, G.; Noël, T. Ni-Catalyzed Electro-Reductive Cross-Electrophile Couplings of Alkyl Amine-Derived Radical Precursors with Aryl Iodides. *J. Org. Chem.* **2023**, *89*, 16121-16125.
(16) Kreisel, T.; Mendel, M.; Queen, A. E.; Deckers, K.; Hupperich, D.; Riegger, J.; Fricke, C.; Schoenebeck, F. Modular Generation of (Iodinated) Polyarenes Using Triethylgermane as Orthogonal Masking Group. *Angew. Chem., Int. Ed.* **2022**, *61*, e202201475.
(17) Clive, D. L. J.; Pham, M. P. Conversion of Weinreb Amides into Benzene Rings Incorporating the Amide Carbonyl Carbon. *J. Org. Chem.* **2009**, *74*, 1685-1690.
(18) Nakamura, M.; Matsuo, K.; Ito, S.; Nakamura, E. Iron-Catalyzed Cross-Coupling of Primary and Secondary Alkyl Halides with Aryl Grignard Reagents. *J. Am. Chem. Soc.* **2004**, *126*, 3686-3687.
(19) Uersfeld, D.; Stappert, S.; Li, C.; Müllen, K. Practical Syntheses of Terrylene Chromophores from Naphthalene and Perylene Building Blocks. *Adv. Synth. Catal.* **2017**, *359*, 4184-4189.
(20) Frisch, M. J.; Trucks, G. W.; Schlegel, H. B.; Scuseria, G. E.; Robb, M. A.; Cheeseman, J. R.; Scalmani, G.; Barone, V.; Petersson, G. A.; Nakatsuji, H.; et al. Gaussian 16 Rev. C.01. **2016**.
(21) Humphrey, W.; Dalke, A.; Schulten, K. VMD: Visual molecular dynamics. *J. Mol. Graph.* **1996**, *14*, 33-38.
(22) Legault, C. Y. CYLview20. **2020**.
(23) Macrae, C. F.; Sovago, I.; Cottrell, S. J.; Galek, P. T.; McCabe, P.; Pidcock, E.; Platings, M.; Shields, G. P.; Stevens, J. S.; Towler, M. Mercury 4.0: From visualization to analysis, design and prediction. *Applied Crystallography* **2020**, *53*, 226-235.
(24) Roy Dennington, T. A. K., John M. Millam. GaussView, Version 6.1. **2019**.
(25) Lee, C.; Yang, W.; Parr, R. G. Development of the Colle-Salvetti correlation-energy formula into a functional of the electron density. *Phys. Rev. B* **1988**, *37*, 785-789.
(26) Becke, A. D. A new mixing of Hartree–Fock and local density-functional theories. *J. Chem. Phys.* **1993**, *98*, 1372-1377.
(27) Becke, A. D. Density-functional thermochemistry. III. The role of exact exchange. *J. Chem. Phys.* **1993**, *98*, 5648-5652.
(28) Grimme, S.; Hansen, A.; Brandenburg, J. G.; Bannwarth, C. Dispersion-Corrected Mean-Field Electronic Structure Methods. *Chem. Rev.* **2016**, *116*, 5105-5154.

(29) Grimme, S.; Antony, J.; Ehrlich, S.; Krieg, H. A consistent and accurate ab initio parametrization of density functional dispersion correction (DFT-D) for the 94 elements H-Pu. *J. Chem. Phys.* **2010**, *132*, 154104.
(30) Johnson, E. R.; Becke, A. D. A post-Hartree-Fock model of intermolecular interactions: Inclusion of higher-order corrections. *J. Chem. Phys.* **2006**, *124*, 174104.
(31) Hay, P. J.; Wadt, W. R. Ab initio effective core potentials for molecular calculations. Potentials for K to Au including the outermost core orbitals. *J. Chem. Phys.* **1985**, *82*, 299-310.
(32) Roy, L. E.; Hay, P. J.; Martin, R. L. Revised Basis Sets for the LANL Effective Core Potentials. *J. Chem. Theory Comput.* **2008**, *4*, 1029-1031.
(33) Hariharan, P. C.; Pople, J. A. The influence of polarization functions on molecular orbital hydrogenation energies. *Theoretica chimica acta* **1973**, *28*, 213-222.
(34) Hehre, W. J.; Ditchfield, R.; Pople, J. A. Self—Consistent Molecular Orbital Methods. XII. Further Extensions of Gaussian—Type Basis Sets for Use in Molecular Orbital Studies of Organic Molecules. *J. Chem. Phys.* **1972**, *56*, 2257-2261.
(35) Cossi, M.; Rega, N.; Scalmani, G.; Barone, V. Energies, structures, and electronic properties of molecules in solution with the C-PCM solvation model. *J. Comput. Chem.* **2003**, *24*, 669-681.
(36) Barone, V.; Cossi, M. Quantum Calculation of Molecular Energies and Energy Gradients in Solution by a Conductor Solvent Model. *J. Phys. Chem. A* **1998**, *102*, 1995-2001.
(37) Fukui, K. The path of chemical reactions - the IRC approach. *Acc. Chem. Res.* **1981**, *14*, 363-368.
(38) Fukui, K. Formulation of the reaction coordinate. *J. Phys. Chem.* **1970**, *74*, 4161-4163.
(39) Musaev, D. G.; Figg, T. M.; Kaledin, A. L. Versatile reactivity of Pd-catalysts: mechanistic features of the mono-N-protected amino acid ligand and cesium-halide base in Pd-catalyzed C–H bond functionalization. *Chem. Soc. Rev.* **2014**, *43*, 5009-5031.
(40) Ren, Z.; Musaev, D. G.; Davies, H. M. L. Key Selectivity Controlling Elements in Rhodium-Catalyzed C–H Functionalization with Donor/Acceptor Carbenes. *ACS Catal.* **2022**, *12*, 13446-13456.
(41) Hansen, J.; Autschbach, J.; Davies, H. M. L. Computational Study on the Selectivity of Donor/Acceptor-Substituted Rhodium Carbenoids. *J. Org. Chem.* **2009**, *74*, 6555-6563.
(42) Nakamura, E.; Yoshikai, N.; Yamanaka, M. Mechanism of C−H Bond Activation/C−C Bond Formation Reaction between Diazo Compound and Alkane Catalyzed by Dirhodium Tetracarboxylate. *J. Am. Chem. Soc.* **2002**, *124*, 7181-7192.
(43) Weigend, F.; Ahlrichs, R. Balanced basis sets of split valence, triple zeta valence and quadruple zeta valence quality for H to Rn: Design and assessment of accuracy. *Phys. Chem. Chem. Phys.* **2005**, *7*, 3297-3305, 10.1039/B508541A.
(44) Weigend, F. Accurate Coulomb-fitting basis sets for H to Rn. *Phys. Chem. Chem. Phys.* **2006**, *8*, 1057-1065, 10.1039/B515623H.
(45) Parr, R. G.; Szentpály, L. v.; Liu, S. Electrophilicity Index. *J. Am. Chem. Soc.* **1999**, *121*, 1922-1924.
(46) Bickelhaupt, F. M.; Houk, K. N. Analyzing Reaction Rates with the Distortion/Interaction-Activation Strain Model. *Angew. Chem., Int. Ed.* **2017**, *56*, 10070-10086.
(47) Falivene, L.; Cao, Z.; Petta, A.; Serra, L.; Poater, A.; Oliva, R.; Scarano, V.; Cavallo, L. Towards the online computer-aided design of catalytic pockets. *Nat. Chem.* **2019**, *11*, 872-879.
(48) Lu, T.; Chen, Q. Independent gradient model based on Hirshfeld partition: A new method for visual study of interactions in chemical systems. *J. Comput. Chem.* **2022**, *43*, 539-555.
(49) Mitoraj, M. P.; Michalak, A.; Ziegler, T. A Combined Charge and Energy Decomposition Scheme for Bond Analysis. *J. Chem. Theory Comput.* **2009**, *5*, 962-975.
(50) Lu, T.; Chen, F. Multiwfn: A multifunctional wavefunction analyzer. *J. Comput. Chem.* **2012**, *33*, 580-592.
(51) Lu, T.; Chen, Q. Simple, Efficient, and Universal Energy Decomposition Analysis Method Based on Dispersion-Corrected Density Functional Theory. *J. Phys. Chem. A* **2023**, *127*, 7023-7035.
(52) Momma, K.; Izumi, F. VESTA 3 for three-dimensional visualization of crystal, volumetric and morphology data. *J. Appl. Crystallogr.* **2011**, *44*, 1272-1276.
(53) Brunard, E.; Boquet, V.; Saget, T.; Sosa Carrizo, E. D.; Sircoglou, M.; Dauban, P. Catalyst-Controlled Intermolecular Homobenzylic C(sp3)–H Amination for the Synthesis of β-Arylethylamines. *J. Am. Chem. Soc.* **2024**, *146*, 5843-5854.
(54) Berry, J. F. The role of three-center/four-electron bonds in superelectrophilic dirhodium carbene and nitrene catalytic intermediates. *Dalton Trans.* **2012**, *41*, 700-713, 10.1039/C1DT11434D.
